# Single-Cell and Transcriptome-Based Immune Cell-Related Prognostic Model in Clear Cell Renal Cell Carcinoma

**DOI:** 10.1155/2023/5355269

**Published:** 2023-03-07

**Authors:** Guanlin Wu, Weiming Guo, Shuai Zhu, Gang Fan

**Affiliations:** ^1^School of Clinical Medicine, Shanghai University of Medicine & Health Sciences, Shanghai 201318, China; ^2^The 2nd Affiliated Hospital of South China University, Hengyang 421001, China; ^3^Department of Urology, The Affiliated Cancer Hospital of Xiangya School of Medicine of Central South University, Hunan Cancer Hospital, Changsha 410013, Hunan, China; ^4^Department of Urology, Huazhong University of Science and Technology Union Shenzhen Hospital, The 6th Affiliated Hospital of Shenzhen University Health Science Center, Shenzhen 518060, China

## Abstract

Traditional studies mostly focus on the role of single gene in regulating clear cell renal cell carcinoma (ccRCC), while it ignores the impact of tumour heterogeneity on disease progression. The purpose of this study is to construct a prognostic risk model for ccRCC by analysing the differential marker genes related to immune cells in the single-cell database to provide help in clinical diagnosis and targeted therapy. Single-cell data and ligand-receptor relationship pair data were downloaded from related publications, and ccRCC phenotype and expression profile data were downloaded from TCGA and CPTAC. Based on the DEGs of each cluster acquired from single-cell data, immune cell marker genes, and ligand-receptor gene data, we constructed a multilayer network. Then, the genes in the network and the genes in TCGA were used to construct the WGCNA network, which screened out prognosis-associated genes for subsequent analysis. Finally, a prognostic risk scoring model was obtained, and CPTAC data showed that the effectiveness of this model was good. A nomogram based on the predictive model for predicting the overall survival was established, and internal validation was performed well. Our findings suggest that the predictive model built and based on the immune cell scRNA-seq will enable us to judge the prognosis of patients with ccRCC and provide more accurate directions for basic relevant research and clinical practice.

## 1. Introduction

RCC is a typical type of malignant tumour of the urinary system. According to the most recent report on cancer statistics, the number of newly diagnosed cases has climbed to 65,000 annually in the United States, resulting in around 15,000 fatalities annually, making it the sixth most prevalent tumour [1]. Clear cell renal cell carcinoma (ccRCC) accounts for around 80% of renal cancer pathological types, and its survival results were poorer than other subtypes of kidney tumours (such as papillary renal cell carcinoma and chromophobe renal cell carcinoma) [2]. Nearly 20% of ccRCC cases progress to an advanced stage at the beginning of diagnosis, and the five-year overall survival (OS) rate of metastatic cases dropped to about 10% [3]. With the development of immunotherapy, radiotherapy, and surgical intervention, combined strategies have greatly promoted carcinoma control. However, the actual clinical efficacy still needs to be improved, and 30% of patients with local ccRCC inevitably experience cancer-related progression and recurrence [4]. Recently, although targeted therapy has been shown to prolong the survival time of patients with metastases, the median survival time is still less than 3 years [5]. In addition, drug resistance and economic burden are unavoidable major problems in clinical practice [6]. Therefore, exploring the molecular mechanism of ccRCC pathogenesis and new therapeutic targets is still a challenging issue.

A crucial aspect of carcinoma is its comprehensive heterogeneity, which can cause individuals to react differently to the same treatment. Despite many efforts to clarify tumour heterogeneity, so far, the understanding of it is still mainly limited to the level of tumour cells [7]. Previously, it has also been proved that stromal cells and tumour-infiltrating immune cells exhibit heterogeneity [8]. Similarly, the tumour microenvironment (TME) is gradually regarded as a potential solution for drug treatment targets [9]. In addition to anti-PD-1/PD-L1 or anti-CTLA-4 treatment strategies, tumour-associated macrophages (TAMs) [10] and cancer-associated fibroblasts (CAFs) [11] have also been previously reported as potential strategies for cancer treatment research. ROS is also an important factor in cancer treatment, it causes structural proteins to oxidise, which disables the proteolytic process. These reactions change how an enzyme works or how proteins are created. The latter could have a wide range of functional impacts downstream, including inhibition of binding and enzymatic activity, an increase or decrease in cellular uptake, inactivation of DNA repair enzymes, and a reduction in the fidelity of damaged DNA polymerases during DNA replication [12]. The successful implementation of these treatment plans requires a deeper insight of intratumoural heterogeneity.

It is obviously impossible to analyse intratumoural heterogeneity at the cellular level since traditional bulk RNA sequencing is predicated on the idea that every gene is expressed equally in each cell. However, through the application of single-cell RNA sequencing (scRNA-seq), it is possible to conduct single-cell transcriptome analysis. The latest progress in scRNA-seq has facilitated the transcriptional classification of many malignant tumour cell types, including breast cancer, lung cancer, and pancreatic ductal adenocarcinoma [13, 14]. Moreover, scRNA-seq is expected to have clinical utility in refractory cancer cases and is a noninvasive method for monitoring circulating cancer cells, analysing intratumoural heterogeneity, and sensitively estimating recurrent tumours [15].

We conducted a series of bioinformatics analyses using data from other publications about scRNA-seq in order to investigate the intratumour heterogeneity in ccRCC. We combined these analyses with ligand-receptor network analysis, immune cell multilayer network analysis, and weighted gene co-expression network analysis (WGCNA) to identify hub genes for creating an immune cell-related prognostic model. It would have several potential targets for ccRCC therapy. Moreover, we also investigated the prognostic value of immune cell clusters by correlating the scRNA-seq data with the data from The Cancer Genome Atlas (TCGA) and Clinical Proteomic Tumor Analysis Consortium (CPTAC) datasets. Our work will help elucidate the biology of ccRCC and promote the improvement of clinical diagnosis and treatment strategies for patients with ccRCC.

## 2. Methods

### 2.1. Raw Data Acquisition

ccRCC single-cell transcriptome data was downloaded from a database published by Young et al. [16]. The datasets of RNA sequencing profiles and the related patient clinical traits of ccRCC were downloaded from TCGA (https://portal.gdc.cancer.gov/) and CPTAC (https://cptac-data-portal.georgetown.edu/study-summary/S050), separately. Ligand and receptor data for building the multilayer network were acquired from [17].

### 2.2. Data Processing

For single-cell data, “limma,” “Seurat,” “dplyr,” and “magrittr” R packages were used for analysis. Data filtering criteria included the following: (1) the number of genes in the data sample was controlled between 200 and 5,000; (2) the number of gene sequences was mainly distributed between 0 and 20,000; and (3) the percentage of mitochondria was controlled below 5%. Then, the log was taken for standardisation, and the first 2,000 genes with the larger coefficient of variation between cells were selected for analysis. Next, principal component analysis (PCA) dimensionality reduction was performed, the data were standardised before dimensionality reduction, and finally, significant dimensions were selected for subsequent analysis. Since the form of data downloaded from TCGA is log2-(data + 1), log processing is not necessary and the standardisation was done directly. Before standardisation, the data must be processed using log2-(data + 1) after being retrieved from the CPTAC database. The “limma” R package was used to carry out the standardisation.

### 2.3. Cell Type Recognition and Clustering Analysis

The recognition of different cell types was based on the “limma,” “Seurat,” “dplyr,” and “magrittr” R packages. We used the 20 principal components (PCs) selected in the previous step to perform TSNE clustering. Subsequently, the cell type was annotated through the “singleR” R package. In order to facilitate the display of subsequent results, we have annotated both subpopulations and single cells.

### 2.4. Identification of Differentially Expressed Genes in Each Cluster

We used several R packages, including “limma,” “Seurat,” “dplyr,” and “magrittr” to analyse the genes contained in each cluster. The FindAllMarkers algorithm was used to screen and calculate the differentially expressed genes (DEGs) in each cluster. The screening standard is |logFC| > 0.5, and the *P* value after correction is <0.05.

### 2.5. Immune Cell Function Status Analysis

We used “GSVA” and “GSEABase” R packages to conduct functional status analysis on samples annotated by single cell, and we referred to the marker genes of immune cell functional status provided by the CancerSEA (https://biocc.hrbmu.edu.cn/CancerSEA/home.jsp) database to clarify the functional status of DEGs in immune cells.

### 2.6. Immune Cell Marker Gene Expression Analysis

The marker genes of immune cells in kidney cancer tissues were obtained from the CellMarker (https://bio-bigdata.hrbmu.edu.cn/CellMarker/) database. In addition, marker genes associated with macrophages and monocytes were acquired from [18]. The expression levels of these marker genes were analysed and displayed through a heat map.

### 2.7. Construction of Ligand-Receptor and Immune Cell Multilayer Networks

The construction of the ligand-receptor network was carried out using the “igraph” R package. To make sure that the ligand genes and associated receptor genes were all included in the gene set taken in union, we first took the intersection of the genes in the ligand-receptor table provided in the literature [17] and the differential genes in all immune cell clusters and the marker genes of all included immune cells. Then, we obtained the data for transcription factors and their target genes from the TRRUST (https://www.grnpedia.org/trrust/) database and combined it with the data for ligand-receptor network genes, which is the intersection of the transcription factors' target genes and network genes.

### 2.8. Weighted Gene Co-Expression Network Analysis

Through the WGCNA algorithm [19], the genes in the immune cell multifactor network were used to construct a co-expression network to find the key modules related to OS and OS time. An appropriate soft threshold value was determined by an R software package (https://www.r-project.org/) to implement according to the WGCNA algorithm. The gradient method was used to test different power values (ranging from 1 to 20) in both the scale independence degree and the module's average connectivity. The most suitable power value could be fixed when the independence degree was above 0.9, as well as when the average connectivity degree was relatively higher [20, 21]. The WGCNA algorithm was also implemented in the construction of scale-free gene co-expression networks. Meanwhile, the corresponding gene sequencing information in each module was extracted. To assess modular feature associations, correlations between module eigengenes (MEs) and clinical features were applied. MEs are the most important components in the PCA of each gene module. The determination of relevant modules needs to be based on the calculation of the correlation strength between MEs and clinical features. The correlation was assessed by gene significance (GS = lgP), where the *P* value was derived from the linear regression analysis of gene expression and clinical information. The key module takes the highest correlation coefficient among all modules, which was selected out for the next step [22].

### 2.9. Key Module Functional Enrichment Analysis

The sequencing information of genes in the key modules from WGCNA was utilized by using the “clusterProfiler” R package to perform gene ontology (GO) and Kyoto Encyclopedia of Genes and Genomes (KEGG) pathway analyses. Among them, GO is to annotate biological processes (BPs), molecular functions (MFs), and cellular components (CCs). The criterion for screening in GO term is *P* value <0.05. The screening criteria for the KEGG pathway are minGSSize = 5, maxGSSize = 500, and qvalueCutoff = 0.05.

### 2.10. Selection of Candidate Prognostic Related Genes

Univariate Cox regression analysis was performed through the “survival” R package to screen prognostic characteristic genes from the previous OS-related WGCNA key modules. When the *P* value is less than 0.05, that is, when the differential expression of these genes has a significant impact on the patient's OS, these genes can be regarded as potential prognostic related genes. Data in this step were from ccRCC cancer samples in TCGA.

### 2.11. Construction, Evaluation, and Validation of Disease Prognosis Risk Model

For the candidate prognostic related genes, combined with their expression in TCGA, univariate Cox regression analysis was used to obtain genes with more significant risk. Then, LASSO dimensionality reduction with 1,000 iterations was performed to screen out redundant genes to obtain more precise prognostic related genes with high hazard ratio (HR) to construct a risk prognosis model. The following formula was used to calculate the risk score for each patient by using a linear combination of specific features that were weighted by their respective coefficients from LASSO:

(1) $$\begin{array}{c} risk\;score=\overset{n}{\underset{i=1}{\sum }}\exp_{i}*{ß}_{i}\text{,} \end{array}$$

where *n* is the number of prognostic genes, exp_*i*_ is the expression value of the *i*-th gene, and *ß*_*i*_ is the regression coefficient of the *i*-th gene in the LASSO algorithm. According to the risk score of each patient given by the model, the median was taken as the cutoff value, and the samples were divided into high and low risk groups. The time-dependent receiver operating characteristic (ROC) curve was used to evaluate the predictive ability of the model's 1-, 3-, and 5-year survival periods. The survival curves of the high and low risk groups were also analysed. The CPTAC dataset was taken as the external validation database to verify the prognostic risk model.

### 2.12. Construction and Assessment of a Predictive Nomogram

Nomograms are widely used to predict the prognosis of cancer patients, mainly because they can simplify statistical prediction models into a single numerical estimate of OS probability tailored to individual patient conditions. In this study, the prognostic model was used to construct a nomogram to assess the probability of OS in patients with ccRCC at one, three, and five years. Subsequently, discrimination and calibration were carried out. The discrimination of the nomogram was calculated by the bootstrap method using the consistency index (C-index), with 1,000 resamples. The value of the C-index is between 0.5 and 1.0, where 1.0 means that the results of the model can be correctly distinguished and 0.5 means random chance. The calibration curve of the nomogram is evaluated graphically by plotting the relationship between the predicted probability of the nomogram and the observed rate. Overlapping with the reference line indicates that the model is exactly the same. In addition, we also compared the predictive accuracy between nomogram built only with risk score and nomogram combined with all factors using ROC analysis.

## 3. Results

### 3.1. Pretreated Data

The single-cell data of the downloaded ccRCC were preprocessed as described in the Methods section, and we obtained 30,092 cells in total. In addition, we found that the correlation between the sequencing depth and the detected genes was 0.95, indicating that the deeper the sequencing depth was, the more the genes were detected. Subsequently, we selected 2,000 genes with large variances for PCA analysis. The differences of all 20 PCs were extremely significant, indicating that the theoretical value and the actual value are quite different which can be used for subsequent analysis.

There were 607 samples in the KIRC expression profile data of TGCA, of which 72 were paracancerous samples, 535 were cancer samples, and one sample had incomplete clinical information which was then removed. Finally, the data of 534 ccRCC tumour tissue samples were used for subsequent relative analysis. Among the data downloaded from the CPTAC database, there were 110 cancer samples and 75 paracancerous samples. Only 103 cancer samples contained clinical information. As a result, the data of these 103 samples were eventually used for analysis.

### 3.2. ccRCC Heterogeneity

**Figure 1:**
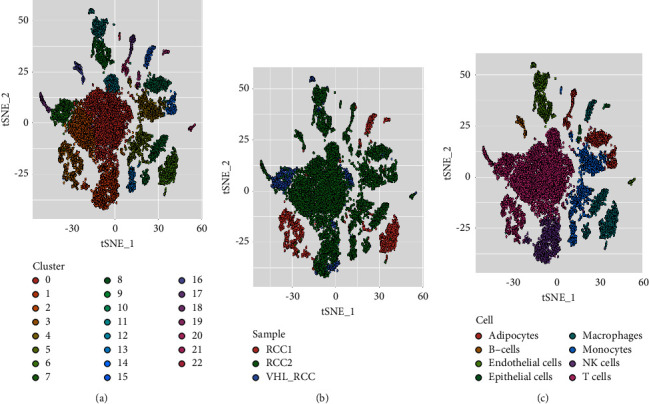
Cell clustering. (a) Clustering of single-cell subpopulations. (b) The distribution of samples in clusters. (c) Annotation for all cell types.

For cluster analysis of single-cell data, we obtained a total of 23 subgroup clusters. After annotating by cell type, we found that immune cells were mainly concentrated in subgroups 0, 1, 2, 3, 4, 5, 6, 7, 9, 12, 13, 15, 16, 18, and 22 (Figure 1, Supplementary Table 1). Specifically, CD8+ T cells were distributed in clusters 0, 2, 3, 7, 12, and 18. NK cells were only annotated in cluster 1. Monocytes assembled in clusters 4, 5, 13, and 22. Clusters 6, 9, and 15 were annotated to macrophages. B cells annotated only cluster 16.

### 3.3. Differentially Expressed Genes and Functional Enrichment in Different Immune Cell Subgroups

**Figure 2:**
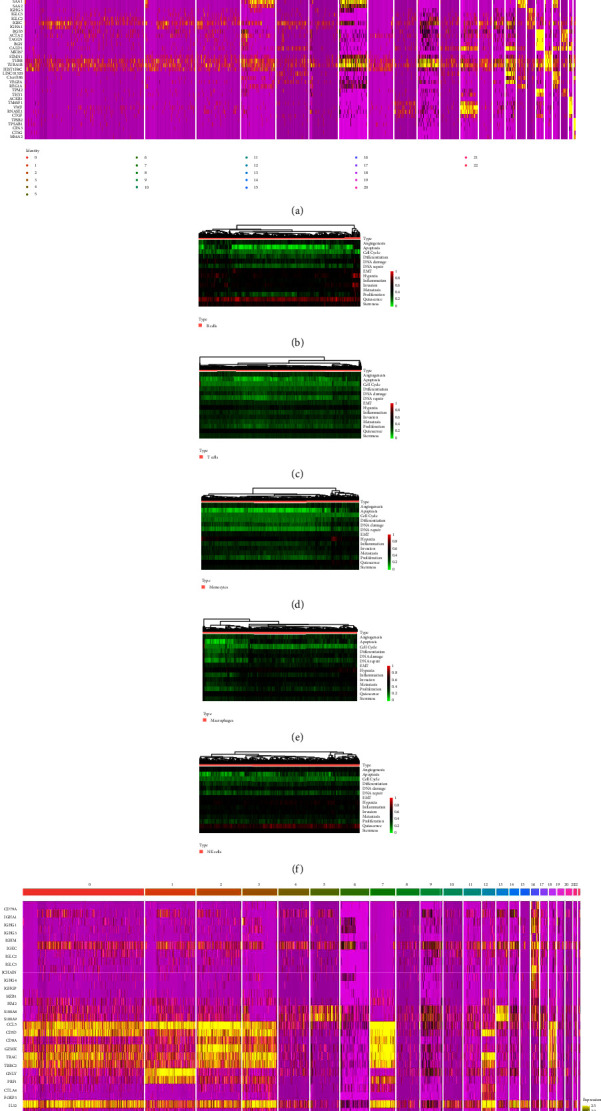
(a) Heat map of the top five differential genes in each cluster. (b) B cell functional status analysis. (c) T cell functional status analysis. (d) Monocyte functional status analysis. (e) Macrophage functional status analysis. (f) NK cell functional status analysis. (g) Heat map of immune cell marker genes.

We performed differential expression analysis on the genes in 23 clusters obtained in the above step and displayed the first five genes in each cluster (Figure 2(a), Supplementary Table 2). According to the results of gene differential expression, we analysed the functional status of the annotated immune cell clusters. In each immune cell type, the enrichment degree of hypoxia and quiescence was relatively high. Besides the enrichment levels of EMT, invasion and stemness in B cells were also relatively high (Figures 2(b)–2(f)).

### 3.4. Identification of Immune Cell Marker Gene Expression

A total of 42 immune cell marker genes related to ccRCC were downloaded from the CellMarker database [18] and subjected to differential expression analysis. The results are shown in the heat map (Figure 2(g)).

### 3.5. Ligand-Receptor Network

**Figure 3:**
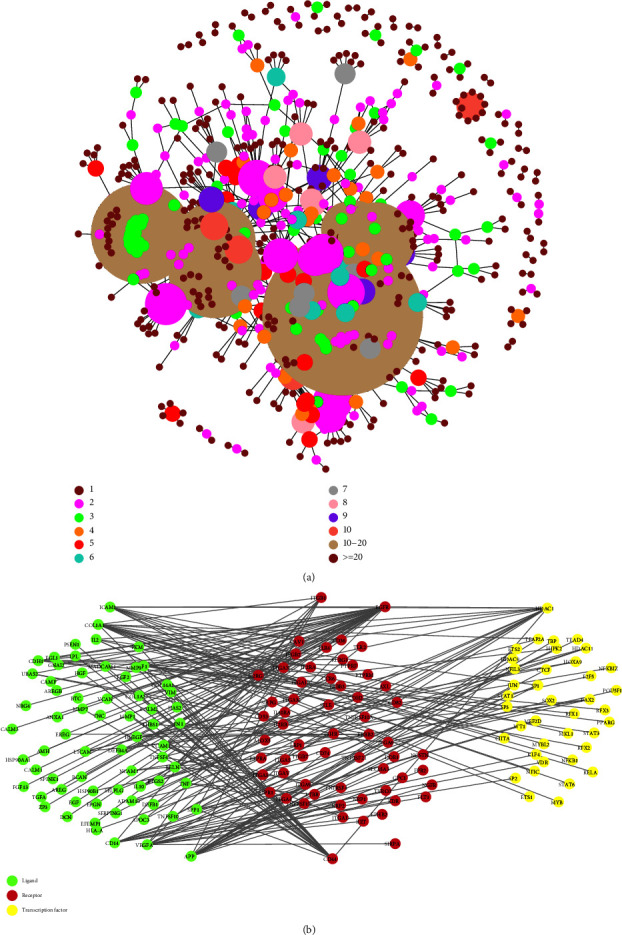
(a) Ligand-receptor interaction network diagram (different colours and sizes represent different numbers of node connections). (b) Multilayer network diagram (green is the ligand gene, red is the receptor gene, and yellow is the transcription factor).

In order to construct the ligand-receptor network, we first took the union of the differential genes of all immune cell clusters and the marker genes of all these immune cells. Afterwards, we intersected them with the ligand-receptor relationship pairs downloaded from [17]. Finally, a total of 981 pairs of ligand-receptor relationships were obtained (Figure 3(a), Supplementary Table 3).

### 3.6. Immune Cell Multifactor Network Based on Ligand-Receptor Network Combined with Transcription Factors

Intersecting genes in 981 ligand-receptor relationship pairs with transcription factor target genes, we obtained 7,987 immune cell multifactor network relationship pairs (Supplementary Table 4). Then, 966 genes were obtained by intersecting the 973 genes contained in the network and the genes in TCGA dataset about ccRCC (Supplementary Table 5). Because there are many relationship pairs, Figure 3(b) only shows a network diagram of partial genes.

### 3.7. Co-Expression Network

**Figure 4:**
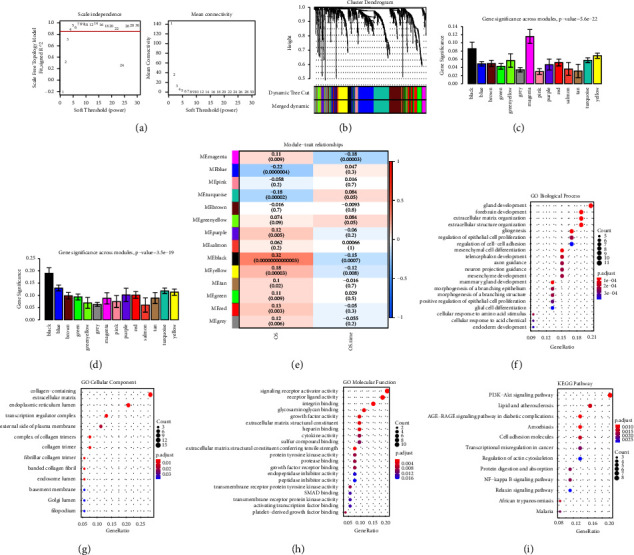
WGCNA and functional analysis in black and magenta modules. (a) Schematic diagram of threshold screening and determination. (b) Clustering dendrogram of all genes from last step. (c) Correlations of OS time with mean gene significance and errors in all modules. (d) Correlations of OS with mean gene significance and errors in all modules. (e) Heat map of the correlations between MEs and OS traits. (f) BP term in GO. (g) CC term in GO. (h) MF term in GO. (i) KEGG pathway analysis.

The construction of co-expression modules included 966 genes from the immune cell multifactor network. The appropriate scale-free power value was screened out as 4 (Figure 4(a)). All constructed modules are painted with different colours, and the cluster trees of genes are also shown in Figure 4(b). The black and magenta modules were chosen as the key modules, since they had the highest correlations with OS and OS time of ccRCC (Figures 4(c) and 4(d)). The correlations between MEs and clinic traits are shown in Figure 4(e). There were 53 genes in these two modules (Supplementary Table 6). For a deeper understanding about the biofunctions of these modules, genes in these modules were carried out to conduct GO and KEGG pathway analyses by using the “clusterProfiler” R package. According to the *P* value of each term, the top terms in the GO and KEGG pathways were extracted out and visualized (Figures 4(f)–4(i)).

### 3.8. Prognostic Risk Scoring Model

**Figure 5:**
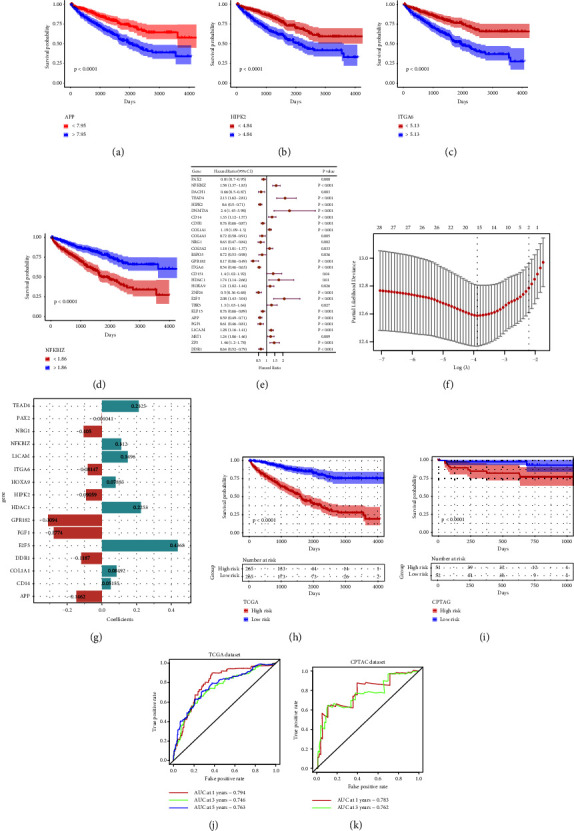
Construction and assessment of disease prognosis risk model. (a–d) Survival analysis of four genes among the candidate genes used to construct the risk prediction model. (e) Forest plot for univariate regression analysis of 28 genes. (f) Selection of appropriate *λ* value through LASSO regression analysis. (g) Scoring chart of risk model constructed by 16 genes. (h, j) Evaluation of predictive effectiveness of risk prognostic model. (i, k) Use of the data in the CPTAC database as an external dataset for verification.

Using the “survival” *R* package to perform univariate Cox regression analysis on the 53 genes contained in the key modules of WGCNA, 28 genes with *P* value <0.05 were obtained. Figures 5(a)–5(d) show the survival analysis results of four genes among them. Then, the 28 genes with significant prognostic differences were subjected to LASSO regression analysis. We adopted the Cox proportional hazard model (family = “Cox”) to calculate the HR and *P* values of these genes (Figure 5(e)) and then randomly simulated 1,000 times (maxit = 1000) to find the most suitable *λ* value in LASSO regression (Figure 5(f)). Finally, “lambda.min” was used to screen out 16 genes for constructing a risk scoring model from these 28 genes (Figure 5(g)).

(2) $$\begin{array}{c} Risk\;score=PAX2*\left(-0.00104\right)+NFKBIZ*0.11197+TEAD4*0.212538+HIPK2*\left(-0.09059\right)+CD14*0.051547+COL1A1*0.084915+NRG1*\left(-0.10501\right)+GPR182*\left(-0.30944\right)+ITGA6*\left(-0.08147\right)+HDAC1*0.225345+HOXA9*0.078825+E2F5*0.436804+APP*\left(-0.14625\right)+FGF1*\left(-0.27736\right)+L1CAM*0.149607+DDR1*\left(-0.11874\right)\text{.} \end{array}$$

### 3.9. Prognostic Model Prediction Effectiveness Evaluation and External Dataset Verification

In the evaluation of the predictive efficacy of the prognosis model, Kaplan–Meier (KM) survival analysis was performed on the high and low risk groups, and the difference was significant (Figure 5(h)). Moreover, in its ROC curve, the one-year AUC value was 0.794, the three-year AUC value was 0.746, and the five-year AUC value was 0.763 (Figure 5(j)). In the external CPTAC dataset, KM survival analysis was performed on the high and low risk groups, and the difference was also significant (Figure 5(i)). In addition, the one-year AUC value in its ROC curve was 0.783, and the three-year AUC value was 0.762 (Figure 5(k)). Because the external data do not have five-year survival data, only one-year and three-year ROC analysis was performed.

### 3.10. Predictive Nomogram

**Figure 6:**
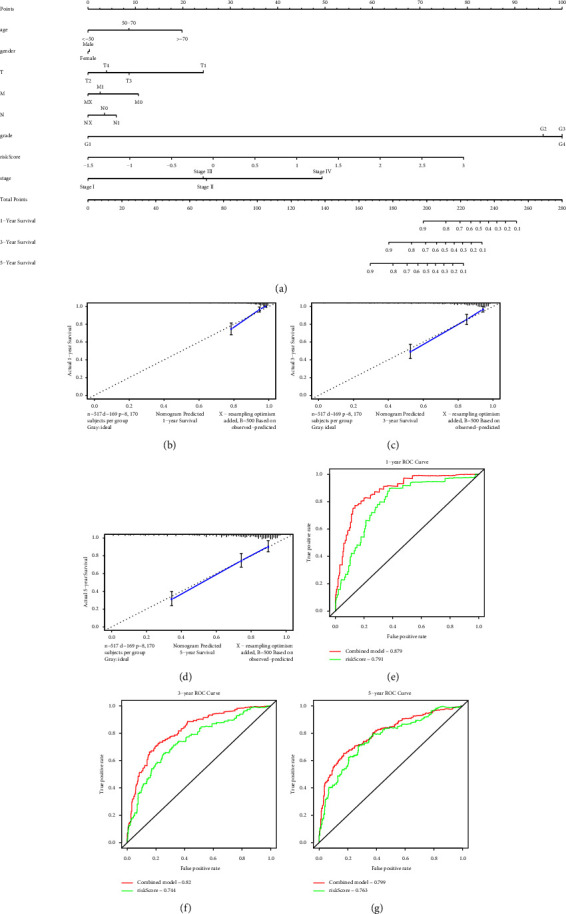
(a) Nomogram predicting 1-, 3-, and 5- year OS for patients with ccRCC. (b–d) The calibration curve for predicting 1-, 3-, and 5-year OS for patients with ccRCC. (e–g) Time-dependent ROC curve analysis evaluates the accuracy of the nomograms.

For the purpose of building a clinically applicable method to estimate the survival possibility of patients with ccRCC, we developed a nomogram to predict the probability of 1-, 3-, and 5-year OS based on the data in TCGA. The predictors of the nomogram included age, gender, T, N, M, grade, risk score, and stage (Figure 6(a)). The C-index for the model for evaluation of OS was 0.799. The visual calibration chart was used to evaluate the performance of the nomogram. When the angle of the line is 45°, it represents the best prediction result. Thus, our calibration chart indicated that the nomogram has a good predictive ability (Figures 6(b)–6(d)). The AUC values of the nomograms combined with all factors were greater than the nomograms built only with risk score in spite of the fact that their values were all more than 0.7. This indicated that the predictive precision of the nomogram combined with all factors was better (Figures 6(e)–6(g)).

## 4. Discussion

The emergence of next-generation sequencing (NGS) has provided a feasible and cost-effective way to determine the transcriptional landscape of human cancers, including both bulk and single-cell resolution with unprecedented base-pair precision [23–25]. It has been established that cancer is attributed to dysregulated evolution [26, 27] in acquiring inheritable genetic/epigenetic traits [28–30]. However, the presence of tumour heterogeneity poses substantial challenges in the diagnosis and treatment of tumours [31–34]. Tumour heterogeneity exerts a vital role in various aspects (e.g., intertumour, intratumour, and intermetastasis heterogeneity, interdisease and interpatient heterogeneity, epigenetic and metabolic heterogeneity, TME heterogeneity, and tumour-intrinsic genetic heterogeneity) [35, 36, 36–38]. A landmark paper has demonstrated that ccRCC is a heterogeneous disease with marked genetic intermetastases and intratumour heterogeneity (G-IMH and G-ITH) [39]. Further studies have elucidated whether somatic mutation landscape and genetic heterogeneity influence the clinical outcomes of ccRCC tumour management [40]. Because of this, we adopted a series of bioinformatics methods to use the ccRCC single-cell data in published articles and the ccRCC-related data in public databases to study whether immune cell-related genes can construct a predictive prognostic model for patients with ccRCC, which may be helpful for further understanding of the intratumour heterogeneity of ccRCC, and provide corresponding support for related basic research and clinical applications in the future.

Since there are many genes used to annotate a certain cell, it is usually difficult to determine which of these genes are critical. As a result, we built some networks, hoping to better find key genes related to our target clinical traits to construct a risk prediction model. Researchers have traditionally been concerned with a few or linear pathways between different cells. Identifying the signalling network of communication within different cell types is invaluable in the diagnosis and treatment of ccRCC tumours. Furthermore, a complete network of cell-cell signalling includes not only intercellular signalling pathways but also intracellular signalling transduction and gene expression [41]. Thus, a complete network of molecular signalling mechanisms is required to show the interaction between the TME and related cell types. A study has proved a potential scRNA-seqtranscriptome-based multilayer network approach, which can be considered as a useful technique to identify the interplay between the TME and tumour cells, further predicting the prognostic and predictive signatures of cancer patients [17]. In addition to the multilayer network, we also applied the WGCNA algorithm to explore the hub genes in key modules associated with the OS and OS time. The WGCNA algorithm explores the relationship between co-expression modules and clinical traits, which provides an opportunity to identify the hub genes in a module but not a downstream gene; thus, it possesses the distinct advantage of making the results more reliable and higher in biological significance [42]. In our study, we found all the genes related to the immune cells in the ccRCC samples through multilayer networks, then divided these genes into multiple modules using the WGCNA method, and used the genes in the modules with the strongest correlation with OS and OS time as the candidate genes for risk scoring model construction. Through the survival difference analysis of the genes in the key modules of the WGCNA algorithm, the genes with significant prognostic significance were found and the genes used to construct the risk prediction model were confirmed after LASSO dimensionality reduction processing. Subsequently, we verified the feasibility and effectiveness of the model for assessing the prognosis of patients with ccRCC through nomogram, which also showed that the immune cells in ccRCC do have an impact on the prognosis of patients.

Some genes in the prognostic risk scoring model have been proven to exert various effects on the regulation of certain tumours or diseases. Previous study has found that an imbalance of APP may be involved with the negative correlation between cancer and Alzheimer's disease [43]. As a vital target in the TLR signalling pathway, CD14 exerts a dual effect on oncogenesis, which can initiate several tumour-related signalling pathways or alter the immune microenvironment in the tumour [44]. COL1A1 was considered to be associated with the pathogenesis of COL1A1-PDGFB fusion uterine sarcoma [45]. It was reported that DDR1 is involved in the development of cancer and fibrotic diseases [46]. Regulating E2F5 is of great significance in maintaining genome stability and the cell cycle [47]. Study has shown that if certain signal mutations cause the destruction of FGF1, it is likely to give rise to cancer [48]. The dysregulation of HDAC1, a chromatin modifier, may cause harmful effects on cell fate and function, which could lead to cancer [49]. HIPK2, a multitalented protein, utilizes its kinase activity to regulate several pathways to limit the proliferation and differentiation of tumour cells and induce positive responses to therapies [50]. Since they are susceptible to ROS, proteins are typically the target of increased free radical production. ROS lead to the oxidation of structural proteins, which shuts down the proteolytic mechanism. These reactions alter the way proteins are built or how an enzyme functions. The latter could have many different downstream functional effects, such as inhibition of enzymatic and binding activities, an increase or decrease in cellular absorption, inactivation of DNA repair enzymes, and a decrease in the fidelity of damaged DNA polymerases in DNA replication [12]. HOXA9, a homeodomain-containing transcription factor, exerts a vital role in the proliferation of hematopoietic stem cells and is commonly negatively affected in acute leukaemias [51]. Recent study has shown that ITGA6 can be a useful biomarker for early detection of colorectal cancer cells in a noninvasive assay and as a prognostic factor [52]. L1CAM has been found in various types of human cancers, which indicates a bad prognosis [53]. NFKBIZ is a psoriasis susceptibility gene that encodes the functions of TRAF6 signalling players, especially in terms of positive and regulatory immune controls by interactions between immune cells and epithelial cells [54]. Oncogenic gene fusion involving NRG1 contributes to the activation of ErbB-mediated pathways, representing a potential target for tumour management [55]. PAX2 has been found in epithelial tumours of the kidney and the female genital tract [56]. TEAD4 binds with YAP, TAZ, VGLL, and other transcription factors to modulate various tumour-related processes, including tumour cell proliferation, cell survival, tissue regeneration, and stem cell maintenance, in cancer via its transcriptional output [57]. The above-reported functions and mechanisms of these genes could help elucidate their correlations with ccRCC and provide reliable evidence for further determination of diagnostic and therapeutic methods.

Although our study only used published data and information in public databases and did not directly use clinical samples for experimental testing and analysis, it is still sufficient to show that the data obtained through single-cell sequencing is able to provide an effective support to predict the prognosis of patients with ccRCC. Additionally, our research can also provide ideas for clinical diagnosis and treatment. For example, the genes in the risk prediction model we have established are more likely to become marker genes for clinical screening of ccRCC or therapeutic targets for metastatic ccRCC. Furthermore, our methods and results would enhance theoretical assistance for other researchers to explore other cancers related to tumour heterogeneity in the future.

## 5. Conclusion

Cancer has been proven to be caused by dysregulated evolution [27] that results in the acquisition of heritable genetic or epigenetic characteristics. However, the occurrence of tumour heterogeneity creates significant difficulties for both tumour identification and treatment. ccRCC is a heterogeneous disease with marked genetic intermetastases and intratumour heterogeneity (G-IMH and G-ITH). The purpose of this study is to determine whether immune cell-related genes can be used to build a predictive prognostic model for patients with ccRCC.

In our study, we used multilayer networks to identify all the immune cell-related genes in the ccRCC samples. We then used the WGCNA method to separate these genes into various modules, and we used the genes in the modules with the strongest correlation with OS and OS time as the candidate genes for risk scoring model construction. Following all steps as detailed in result and discussion section, we then used a nomogram to validate the viability and efficacy of the model for determining a patient's prognosis for ccRCC, which also demonstrated that the immune cells in ccRCC do affect the prognosis of patients.

In a nutshell, our results indicate that the immune cell scRNA-seq predictive model will help us to assess the prognosis of patients with ccRCC and provide more precise guidelines for basic related research and clinical management. As a result, it may help to further our understanding of the intratumour heterogeneity of ccRCC and support future basic research and clinical applications.

## Data Availability

The data and materials can be obtained by contacting the corresponding author.

## Abbreviations

RCC: Renal cell carcinoma
ccRCC: Clear cell renal cell carcinoma
OS: Overall survival
TME: Tumour microenvironment
TAMs: Tumour-associated macrophages
CAFs: Cancer-associated fibroblasts
scRNA-seq: Single-cell RNA sequencing
WGCNA: Weighted gene co-expression network analysis
TCGA: The Cancer Genome Atlas
CPTAC: Consortium for Clinical Proteome Cancer Analysis
PCA: Principal component analysis
PC: Principal component
DEGs: Differentially expressed genes
MEs: Module eigengenes
GO: Gene ontology
KEGG: Kyoto Encyclopedia of Genes and Genomes
BP: Biological process
MF: Molecular function
CC: Cellular component
HR: Hazard ratio
ROC: Receiver operating characteristic
C-index: Concordance index
KM: Kaplan–Meier
NGS: Next-generation sequencing.

## Supplementary Materials

Supplementary Materials Supplementary Table 1: Notes on cell clustering. Supplementary Table 2: Differential genes in each cell cluster. Supplementary Table 3: Ligand-receptor relationship pair. Supplementary Table 4: Immune cell multifactor network relationship pair. Supplementary Table 5: Intersection genes in immune cell multifactor network relationship pair and TCGA. Supplementary Table 6: Genes in black and magenta models of WGCNA.

## References

[1] Siegel R. L., Miller K. D., Jemal A. (2018). Cancer statistics. *CA: A Cancer Journal for Clinicians*.

[2] Hakimi A. A., Voss M. H., Kuo F. (2019). Transcriptomic profiling of the tumor microenvironment reveals distinct subgroups of clear cell renal cell cancer: data from a randomized phase III trial. *Cancer Discovery*.

[3] Mitchell T. J., Turajlic S., Rowan A. (2018). Timing the landmark events in the evolution of clear cell renal cell cancer: TRACERx renal. *Cell*.

[4] Miao D., Margolis C. A., Gao W. (2018). Genomic correlates of response to immune checkpoint therapies in clear cell renal cell carcinoma. *Science*.

[5] Greef B., Eisen T. (2016). Medical treatment of renal cancer: new horizons. *British Journal of Cancer*.

[6] Shih Y. C. T., Chien C. R., Xu Y., Pan I. W., Smith G. L., Buchholz T. A. (2011). Economic burden of renal cell carcinoma in the US: Part II--an updated analysis. *PharmacoEconomics*.

[7] Patel A. P., Tirosh I., Trombetta J. J. (2014). Single-cell RNA-seq highlights intratumoral heterogeneity in primary glioblastoma. *Science*.

[8] Papalexi E., Satija R. (2018). Single-cell RNA sequencing to explore immune cell heterogeneity. *Nature Reviews Immunology*.

[9] Albini A., Sporn M. B. (2007). The tumour microenvironment as a target for chemoprevention. *Nature Reviews Cancer*.

[10] Mantovani A., Marchesi F., Malesci A., Laghi L., Allavena P. (2017). Tumour-associated macrophages as treatment targets in oncology. *Nature Reviews Clinical Oncology*.

[11] Hanley C. J., Mellone M., Ford K. (2018). Targeting the myofibroblastic cancer-associated fibroblast phenotype through inhibition of NOX4. *Journal of the National Cancer Institute: Journal of the National Cancer Institute*.

[12] Palozza P., Calviello G., Serini S. (2001 May 1). *β*-Carotene at high concentrations induces apoptosis by enhancing oxy-radical production in human adenocarcinoma cells. *Free Radical Biology and Medicine*.

[13] Peng J., Sun B. F., Chen C. Y. (2019). Single-cell RNA-seq highlights intra-tumoral heterogeneity and malignant progression in pancreatic ductal adenocarcinoma. *Cell Research*.

[14] Chung W., Eum H. H., Lee H. O. (2017). Single-cell RNA-seq enables comprehensive tumour and immune cell profiling in primary breast cancer. *Nature Communications*.

[15] Navin N. E. (2015). The first five years of single-cell cancer genomics and beyond. *Genome Research*.

[16] Young M. D., Mitchell T. J., Vieira Braga F. A. (2018). Single-cell transcriptomes from human kidneys reveal the cellular identity of renal tumors. *Science*.

[17] Zhang J., Guan M., Wang Q., Zhang J., Zhou T., Sun X. (2020). Single-cell transcriptome-based multilayer network biomarker for predicting prognosis and therapeutic response of gliomas. *Briefings in Bioinformatics*.

[18] Vishwakarma A., Nicholas B., Michael S. C. (2019). Mapping the immune landscape of clear cell renal cell carcinoma by single-cell RNA-seq. https://www.biorxiv.org/content/10.1101/824482v1.

[19] Langfelder P., Horvath S. (2008). WGCNA: an R package for weighted correlation network analysis. *BMC Bioinformatics*.

[20] Chen L., Yuan L., Wang Y. (2017). Co-expression network analysis identified FCER1G in association with progression and prognosis in human clear cell renal cell carcinoma. *International Journal of Biological Sciences*.

[21] Wu G., Xia P., Yan S., Chen D., Xie L., Fan G. (2021). Identification of unique long non-coding RNAs as putative biomarkers for chromophobe renal cell carcinoma. *Personalized Medicine*.

[22] Xia M. D., Yu R. R., Chen D. M. (2021). Identification of hub biomarkers and immune-related pathways participating in the progression of antineutrophil cytoplasmic antibody-associated glomerulonephritis. *Frontiers in Immunology*.

[23] Stratton M. R. (2011). Exploring the genomes of cancer cells: progress and promise. *Science*.

[24] Navin N., Kendall J., Troge J. (2011). Tumour evolution inferred by single-cell sequencing. *Nature*.

[25] Tirosh I., Izar B., Prakadan S. M. (2016). Dissecting the multicellular ecosystem of metastatic melanoma by single-cellRNA-seq. *Science*.

[26] Nowell P. C. (1976). The clonal evolution of tumor cell populations. *Science*.

[27] Yates L. R., Campbell P. J. (2012). Evolution of the cancer genome. *Nature Reviews Genetics*.

[28] Vogelstein B., Kinzler K. W. (2015). The path to cancer --Three strikes and you’re out. *New England Journal of Medicine*.

[29] Gerlinger M., McGranahan N., Dewhurst S. M., Burrell R. A., Tomlinson I., Swanton C. (2014). Cancer: evolution within a lifetime. *Annual Review of Genetics*.

[30] Heppner G. H. (1984). Tumor heterogeneity. *Cancer Research*.

[31] Marusyk A., Almendro V., Polyak K. (2012). Intra-tumour heterogeneity: a looking glass for cancer?. *Nature Reviews Cancer*.

[32] Burrell R. A., McGranahan N., Bartek J., Swanton C. (2013). The causes and consequences of genetic heterogeneity in cancer evolution. *Nature*.

[33] Alizadeh A. A., Aranda V., Bardelli A. (2015). Toward understanding and exploiting tumor heterogeneity. *Nature Medicine*.

[34] Longo D. L. (2012). Tumor heterogeneity and personalized medicine. *New England Journal of Medicine*.

[35] Jamal-Hanjani M., Quezada S. A., Larkin J., Swanton C. (2015). Translational implications of tumor heterogeneity. *Clinical Cancer Research*.

[36] Voss M. H., Hsieh J. J. (2016). Therapeutic guide for mTOuRing through the braided kidney cancer genomic river. *Clinical Cancer Research*.

[37] Bedard P. L., Hansen A. R., Ratain M. J., Siu L. L. (2013). Tumour heterogeneity in the clinic. *Nature*.

[38] Andor N., Graham T. A., Jansen M. (2016). Pan-cancer analysis of the extent and consequences of intratumor heterogeneity. *Nature Medicine*.

[39] Gerlinger M., Rowan A. J., Horswell S. (2012). Intratumor heterogeneity and branched evolution revealed by multiregion sequencing. *New England Journal of Medicine*.

[40] Hsieh J. J., Manley B. J., Khan N., Gao J., Carlo M. I., Cheng E. H. (2017). Overcome tumor heterogeneity-imposed therapeutic barriers through convergent genomic biomarker discovery: a braided cancer river model of kidney cancer. *Seminars in Cell & Developmental Biology*.

[41] Sun X., Su J., Bao J. (2012). Cytokine combination therapy prediction for bone remodeling in tissue engineering based on the intracellular signaling pathway. *Biomaterials*.

[42] Chou W. C., Cheng A. L., Brotto M., Chuang C. Y. (2014). Visual gene-network analysis reveals the cancer gene co-expression in human endometrial cancer. *BMC Genomics*.

[43] Galvao F., Grokoski K. C., da Silva B. B., Lamers M. L., Siqueira I. R. (2019). The amyloid precursor protein (APP) processing as a biological link between Alzheimer’s disease and cancer. *Ageing Research Reviews*.

[44] Wu Z., Zhang Z., Lei Z., Lei P. (2019). CD14: biology and role in the pathogenesis of disease. *Cytokine & Growth Factor Reviews*.

[45] Croce S., Hostein I., McCluggage W. G. (2021). *NTRK* and other recently described kinase fusion positive uterine sarcomas: a review of a group of rare neoplasms. *Genes Chromosomes & Cancer*.

[46] Yeh Y. C., Lin H. H., Tang M. J. (2019). Dichotomy of the function of DDR1 in cells and disease progression. *Biochimica et Biophysica Acta (BBA) - Molecular Cell Research*.

[47] Dominguez-Brauer C., Brauer P. M., Chen Y. J., Pimkina J., Raychaudhuri P. (2010). Tumor suppression by ARF: gatekeeper and caretaker. *Cell Cycle*.

[48] Jiang X., Skibba M., Zhang C., Tan Y., Xin Y., Qu Y. (2013). The roles of fibroblast growth factors in the testicular development and tumor. *Journal of Diabetes Research*.

[49] Laugesen A., Helin K. (2014). Chromatin repressive complexes in stem cells, development, and cancer. *Cell Stem Cell*.

[50] D’Orazi G., Rinaldo C., Soddu S. (2012). Updates on HIPK2: a resourceful oncosuppressor for clearing cancer. *Journal of Experimental & Clinical Cancer Research*.

[51] Collins C. T., Hess J. L. (2016). Role of HOXA9 in leukemia: dysregulation, cofactors and essential targets. *Oncogene*.

[52] Beaulieu J. F. (2019). Integrin *α*6*β*4 in colorectal cancer: expression, regulation, functional alterations and use as a biomarker. *Cancers*.

[53] Altevogt P., Doberstein K., Fogel M. (2016). L1CAM in human cancer. *International Journal of Cancer*.

[54] Dainichi T., Matsumoto R., Mostafa A., Kabashima K. (2019). Immune control by TRAF6-mediated pathways of epithelial cells in the EIME (epithelial immune microenvironment). *Frontiers in Immunology*.

[55] Laskin J., Liu S., Tolba K. (2020). NRG1 fusion-driven tumors: biology, detection, and the therapeutic role of afatinib and other ErbB-targeting agents. *Annals of Oncology*.

[56] Ordonez N. G. (2012). Value of PAX2 immunostaining in tumor diagnosis: a review and update. *Advances in Anatomic Pathology*.

[57] Chen M., Huang B., Zhu L., Chen K., Liu M., Zhong C. (2020). Structural and Functional Overview of TEAD4 in Cancer Biology. *OncoTargets and Therapy*.

[58] Wu G., Miao L., Weifeng Y. (2022). Integrated analysis of single-cell and transcriptome based RNA-seq multilayer network and WGCNA for construction and validation of an immune cell-related prognostic model in clear cell renal cell carcinoma. https://www.biorxiv.org/content/10.1101/2021.10.15.464475v1.

