# Supplementary material for: Single-Cell and Transcriptome-Based Immune Cell-Related Prognostic Model in Clear Cell Renal Cell Carcinoma
Source: J Oncol. 2023 Mar 7;2023:5355269. doi: 10.1155/2023/5355269 (PMC10014191; doi:10.1155/2023/5355269)
Supplement: Supplementary Materials — Supplementary Table 1: Notes on cell clustering. Supplementary Table 2: Differential genes in each cell cluster. Supplementary Table 3: Ligand-receptor relationship pair. Supplementary Table 4: Immune cell multifactor network relationship pair. Supplementary Table 5: Intersection genes in immune cell multifactor network relationship pair and TCGA. Supplementary Table 6: Genes in black and magenta models of WGCNA. [file 5355269.f1.zip › Supplementary Table 1. Notes on cell clustering.pdf]

0	CD8+ T-cells
1	NK cells
2	CD8+ T-cells
3	CD8+ T-cells
4	Monocytes
5	Monocytes
6	Macrophages
7	CD8+ T-cells
8	Endothelial cells
9	Macrophages
10	Adipocytes
11	Endothelial cells
12	CD8+ T-cells
13	Monocytes
14	Adipocytes
15	Macrophages
16	B-cells
17	Adipocytes
18	CD8+ T-cells
19	Epithelial cells
20	Adipocytes
21	Endothelial cells
22	Monocytes
