# Supplementary material for: Single-Cell and Transcriptome-Based Immune Cell-Related Prognostic Model in Clear Cell Renal Cell Carcinoma
Source: J Oncol. 2023 Mar 7;2023:5355269. doi: 10.1155/2023/5355269 (PMC10014191; doi:10.1155/2023/5355269)
Supplement: Supplementary Materials — Supplementary Table 1: Notes on cell clustering. Supplementary Table 2: Differential genes in each cell cluster. Supplementary Table 3: Ligand-receptor relationship pair. Supplementary Table 4: Immune cell multifactor network relationship pair. Supplementary Table 5: Intersection genes in immune cell multifactor network relationship pair and TCGA. Supplementary Table 6: Genes in black and magenta models of WGCNA. [file 5355269.f1.zip › Supplementary Table 2. Differential genes in each cell cluster.pdf]

	p_val	avg_log2FC	pct.1	pct.2	p_val_adj	cluster
IL7R	0	2.498747386	0.707	0.184	0	0
ZFP36L2	0	1.639237107	0.919	0.633	0	0
GPR171	0	1.476813325	0.393	0.083	0	0
CD69	0	1.45374069	0.825	0.441	0	0
TNFAIP3	0	1.337307943	0.684	0.377	0	0
CD40LG	0	1.25010645	0.257	0.024	0	0
BTG1	0	1.187860856	0.972	0.804	0	0
CD52	0	1.186332075	0.805	0.473	0	0
CXCR4	0	1.185357238	0.936	0.727	0	0
TSC22D3	0	1.069593535	0.916	0.741	0	0
RPS29	0	0.806734413	0.97	0.907	0	0
RPS27	0	0.654865721	0.985	0.972	0	0
RPL28	0	0.580367218	0.968	0.922	0	0
RPLP2	0	0.563589726	0.968	0.937	0	0
JUNB	6.99E-290	1.080647433	0.87	0.68	1.61E-285	0
SARAF	3.18E-286	0.909846794	0.826	0.634	7.31E-282	0
KLF6	1.11E-258	1.01702788	0.814	0.6	2.56E-254	0
RPS3	3.06E-258	0.504751999	0.962	0.906	7.03E-254	0
JUN	2.47E-256	1.06240465	0.848	0.645	5.67E-252	0
CD2	3.94E-253	0.869416926	0.724	0.378	9.05E-249	0
AC016831.7	6.20E-249	1.269719505	0.374	0.13	1.43E-244	0
FTL	2.74E-243	-1.729311751	0.917	0.943	6.30E-239	0
CD74	1.99E-234	-2.011099254	0.741	0.809	4.58E-230	0
CD3D	1.24E-230	0.715680058	0.749	0.393	2.85E-226	0
STK4	4.25E-228	1.053060726	0.654	0.418	9.78E-224	0
RORA	3.55E-227	1.090030826	0.411	0.161	8.16E-223	0
TXNIP	7.72E-222	0.924205351	0.877	0.73	1.77E-217	0
CD44	6.71E-220	0.932583829	0.673	0.437	1.54E-215	0
HLA-DRB1	2.66E-214	-2.17716892	0.408	0.645	6.12E-210	0
CD63	2.12E-213	-1.429592734	0.305	0.583	4.88E-209	0
CST3	3.73E-210	-2.250465459	0.17	0.449	8.57E-206	0
CD3E	1.28E-209	0.821543033	0.629	0.329	2.95E-205	0
RPL14	7.23E-209	0.526996971	0.933	0.85	1.66E-204	0
ANXA1	2.32E-208	1.10455708	0.646	0.419	5.34E-204	0
IL32	3.65E-206	0.695611553	0.797	0.513	8.39E-202	0
PIK3IP1	1.72E-200	1.003359431	0.541	0.304	3.94E-196	0
RHOB	1.15E-199	-1.592006696	0.127	0.414	2.64E-195	0
LEPROTL1	5.49E-199	1.04887008	0.553	0.341	1.26E-194	0
LTB	4.81E-198	0.974451254	0.502	0.242	1.11E-193	0
HLA-DRA	5.02E-196	-2.412741148	0.544	0.711	1.15E-191	0
TYROBP	2.73E-195	-2.1458991	0.186	0.447	6.28E-191	0
RP11-138A9.2	7.83E-192	1.211646869	0.359	0.142	1.80E-187	0
STK17A	1.92E-190	0.949716514	0.547	0.313	4.40E-186	0
CYTIP	1.08E-187	0.849830431	0.649	0.424	2.47E-183	0
IFITM3	4.01E-187	-1.983243301	0.166	0.441	9.22E-183	0
TRAC	1.22E-184	0.647581344	0.672	0.361	2.81E-180	0
RP11-347P5.1	6.12E-181	0.975126582	0.564	0.32	1.41E-176	0
RP11-138A9.1	1.78E-177	1.062154212	0.35	0.144	4.10E-173	0
ODF2L	8.47E-171	1.055341006	0.423	0.211	1.95E-166	0
FCGR3A	7.00E-170	-1.585384164	0.053	0.287	1.61E-165	0
FCER1G	1.00E-166	-1.790182178	0.118	0.358	2.30E-162	0
HLA-DMA	1.16E-164	-1.310329922	0.098	0.343	2.66E-160	0
SERPINA1	3.38E-164	-1.700517266	0.09	0.334	7.77E-160	0
HSPB1	9.09E-164	-1.590313777	0.237	0.493	2.09E-159	0
RPSA	8.39E-163	0.528744602	0.894	0.777	1.93E-158	0
GSTP1	2.89E-157	-1.127427056	0.289	0.548	6.65E-153	0
SPOCK2	7.01E-156	0.865324317	0.398	0.186	1.61E-151	0

GPX1	7.09E-154	-1.754464431	0.25	0.482	1.63E-149	0
DDX5	1.89E-153	0.519557846	0.919	0.779	4.34E-149	0
FCGRT	6.01E-153	-1.12781939	0.065	0.293	1.38E-148	0
ZFP36	1.39E-150	0.829310749	0.78	0.629	3.19E-146	0
RGS1	5.05E-150	0.707902447	0.863	0.648	1.16E-145	0
PPP2R5C	1.29E-149	0.887740298	0.547	0.344	2.95E-145	0
GSN	4.34E-146	-1.610495118	0.058	0.274	9.98E-142	0
TRAT1	7.15E-145	0.797572403	0.264	0.093	1.64E-140	0
PTMS	5.01E-144	-0.975472827	0.067	0.294	1.15E-139	0
PDK4	2.86E-143	-1.570404894	0.044	0.251	6.58E-139	0
CD9	9.35E-143	-1.324876968	0.058	0.272	2.15E-138	0
AC092580.4	2.75E-142	0.960806646	0.328	0.139	6.32E-138	0
GRN	1.12E-140	-1.250439832	0.085	0.304	2.56E-136	0
HLA-DQA1	1.30E-140	-1.742592934	0.169	0.395	2.99E-136	0
CD3G	4.85E-140	0.684477867	0.472	0.24	1.11E-135	0
NFKBIA	1.18E-139	0.878385425	0.679	0.522	2.72E-135	0
IDS	4.02E-139	0.980019011	0.509	0.333	9.23E-135	0
NPC2	4.70E-137	-1.541576043	0.208	0.43	1.08E-132	0
EVL	5.38E-136	0.702119343	0.643	0.452	1.24E-131	0
APLP2	3.81E-135	-0.942663783	0.084	0.306	8.75E-131	0
PTGER4	1.96E-133	0.891003675	0.363	0.181	4.50E-129	0
S100A4	3.24E-133	0.557395764	0.83	0.661	7.45E-129	0
PTPRC	4.88E-133	0.617731208	0.786	0.566	1.12E-128	0
AIF1	9.61E-132	-1.662125065	0.101	0.306	2.21E-127	0
SRSF7	4.96E-129	0.705057435	0.68	0.504	1.14E-124	0
NNMT	5.75E-125	-2.139264673	0.132	0.349	1.32E-120	0
HLA-DPA1	2.56E-124	-1.909608527	0.444	0.608	5.87E-120	0
HLA-DMB	3.51E-123	-1.055784926	0.061	0.255	8.07E-119	0
CD96	1.18E-122	0.780593443	0.394	0.206	2.72E-118	0
KLRB1	1.31E-122	1.182059618	0.355	0.17	3.02E-118	0
PSAP	7.18E-122	-1.304473252	0.316	0.513	1.65E-117	0
PDCD4	1.69E-118	0.863618039	0.433	0.265	3.89E-114	0
NDUFA4L2	2.80E-117	-2.233840883	0.132	0.335	6.43E-113	0
HLA-DQB1	7.56E-117	-1.49473879	0.235	0.439	1.74E-112	0
SPP1	1.03E-115	-2.50625772	0.073	0.259	2.36E-111	0
ADIRF	1.32E-115	-2.06112359	0.09	0.286	3.04E-111	0
HLA-DPB1	2.24E-115	-1.801767973	0.534	0.661	5.16E-111	0
AIM1	2.29E-114	0.972943556	0.308	0.151	5.27E-110	0
CEBPD	2.37E-112	-1.058604	0.126	0.324	5.44E-108	0
FTH1	3.29E-111	-1.244435836	0.956	0.964	7.55E-107	0
ASAH1	5.78E-111	-0.88083955	0.089	0.285	1.33E-106	0
TRBC2	5.81E-110	0.57490145	0.542	0.328	1.34E-105	0
LAPTM4A	6.70E-110	-0.930337301	0.182	0.402	1.54E-105	0
RAC1	2.26E-109	-1.014922808	0.297	0.493	5.19E-105	0
LYZ	1.19E-108	-2.40223536	0.209	0.393	2.74E-104	0
ACAP1	1.18E-106	0.706980333	0.476	0.291	2.71E-102	0
FOSB	2.66E-105	0.67552563	0.562	0.385	6.11E-101	0
YWHAH	4.96E-103	-1.144875462	0.134	0.327	1.14E-98	0
CRYAB	1.94E-102	-1.96627258	0.114	0.302	4.45E-98	0
CYB5A	3.60E-102	-1.445123938	0.089	0.271	8.28E-98	0
GAPDH	1.41E-101	-0.933802234	0.836	0.875	3.23E-97	0
TUBA4A	2.47E-100	0.686623232	0.407	0.233	5.68E-96	0
TPI1	1.57E-98	-0.975921867	0.409	0.61	3.60E-94	0
FKBP1A	5.46E-98	-1.070863345	0.247	0.462	1.26E-93	0
IGFBP7	1.01E-93	-2.82009584	0.233	0.422	2.33E-89	0
BST2	1.10E-93	-0.780670749	0.197	0.409	2.52E-89	0
FKBP11	1.67E-93	0.654439154	0.273	0.129	3.85E-89	0
SOD2	1.80E-93	-1.384677146	0.148	0.336	4.13E-89	0

PKM	1.99E-93	-0.906007269	0.273	0.481	4.57E-89	0
ETS1	1.63E-92	0.616510367	0.475	0.298	3.75E-88	0
AAK1	4.30E-92	0.759699127	0.479	0.338	9.89E-88	0
APOC1	2.98E-91	-1.92851962	0.147	0.324	6.85E-87	0
GPR183	4.86E-91	0.678492799	0.352	0.197	1.12E-86	0
EML4	2.00E-90	0.801408838	0.312	0.173	4.60E-86	0
ATOX1	5.24E-90	-0.759762676	0.088	0.265	1.21E-85	0
YWHAE	2.88E-89	-0.745512629	0.157	0.358	6.62E-85	0
LCK	4.05E-88	0.581571045	0.422	0.248	9.32E-84	0
SMCHD1	8.69E-85	0.780021513	0.43	0.297	2.00E-80	0
TRBC1	1.06E-84	0.550265954	0.49	0.312	2.45E-80	0
FXYD5	2.87E-84	0.567328327	0.579	0.452	6.59E-80	0
CCNH	3.47E-84	0.810527951	0.389	0.253	7.98E-80	0
AKR1B1	1.52E-83	-0.71824902	0.104	0.276	3.50E-79	0
HSPA1A	2.18E-83	-1.237644624	0.105	0.272	5.01E-79	0
ISG20	8.15E-83	0.582298691	0.479	0.326	1.87E-78	0
HSBP1	1.60E-81	-0.639757238	0.103	0.279	3.67E-77	0
ANXA5	6.66E-81	-0.742744324	0.204	0.395	1.53E-76	0
TYMP	5.36E-80	-0.857143108	0.162	0.335	1.23E-75	0
NDUFC1	3.56E-79	-0.944634387	0.134	0.311	8.18E-75	0
C1QA	5.08E-79	-1.77686517	0.129	0.286	1.17E-74	0
CLEC2D	6.40E-79	0.653189526	0.426	0.271	1.47E-74	0
PPP1R2	7.32E-79	0.75030613	0.439	0.314	1.68E-74	0
PRDX1	3.43E-78	-0.877702723	0.259	0.447	7.89E-74	0
CTSB	1.03E-77	-0.97505354	0.174	0.34	2.37E-73	0
TMEM173	3.43E-77	0.769523432	0.323	0.198	7.88E-73	0
AP2S1	1.60E-76	-0.595457173	0.164	0.354	3.67E-72	0
LST1	2.60E-76	-1.115956061	0.111	0.261	5.98E-72	0
HSP90B1	5.16E-76	-0.779220261	0.262	0.449	1.19E-71	0
TNFAIP8	1.44E-75	0.698460695	0.315	0.187	3.30E-71	0
STK17B	6.09E-74	0.670519191	0.502	0.37	1.40E-69	0
FAM129A	3.16E-73	0.713909893	0.301	0.177	7.27E-69	0
PLIN2	4.54E-73	-1.247766369	0.204	0.375	1.04E-68	0
NAMPT	5.59E-73	-0.84382974	0.11	0.269	1.28E-68	0
CD59	1.23E-72	-1.392094262	0.115	0.268	2.83E-68	0
PARP8	2.45E-72	0.692740353	0.305	0.175	5.64E-68	0
C1QB	7.60E-72	-1.854670502	0.122	0.269	1.75E-67	0
ALDOA	8.10E-72	-0.887700701	0.524	0.67	1.86E-67	0
NKG7	3.61E-71	-1.450070314	0.321	0.459	8.29E-67	0
ATP6V0B	3.61E-69	-0.615203788	0.164	0.335	8.30E-65	0
CLTA	1.06E-68	-0.552014151	0.155	0.331	2.44E-64	0
EMB	1.51E-68	0.693524971	0.366	0.241	3.46E-64	0
GNG5	2.94E-67	-0.576113454	0.21	0.394	6.75E-63	0
SAMSN1	4.55E-67	0.582865597	0.422	0.293	1.05E-62	0
CMC1	2.88E-64	-0.985193393	0.167	0.32	6.63E-60	0
HLA-DRB5	5.17E-64	-1.496081002	0.268	0.404	1.19E-59	0
CNOT6L	9.52E-64	0.678027045	0.321	0.201	2.19E-59	0
FKBP2	5.25E-63	-0.613471392	0.119	0.272	1.21E-58	0
AP1S2	2.56E-62	-0.638298868	0.113	0.258	5.88E-58	0
RNF19A	3.03E-62	0.656213306	0.373	0.248	6.97E-58	0
CTSD	3.39E-62	-0.816266846	0.31	0.475	7.80E-58	0
SLIRP	5.50E-62	-0.563504792	0.148	0.312	1.26E-57	0
CTSS	3.79E-61	-1.040835977	0.258	0.394	8.71E-57	0
LYAR	5.57E-61	0.631867014	0.288	0.172	1.28E-56	0
MT2A	1.77E-60	-1.867192166	0.572	0.685	4.07E-56	0
BTG2	1.79E-60	0.586683328	0.449	0.33	4.12E-56	0
RGS2	2.08E-60	-0.9593072	0.221	0.369	4.79E-56	0
TOB1	1.71E-59	0.782416709	0.265	0.156	3.92E-55	0

RHOC	3.37E-59	-0.618322212	0.193	0.362	7.75E-55	0
APOE	3.88E-59	-2.199859659	0.328	0.47	8.92E-55	0
ELF1	4.33E-59	0.58039693	0.514	0.419	9.95E-55	0
CD48	1.03E-58	0.53148082	0.463	0.349	2.36E-54	0
SNX3	2.74E-58	-0.6171641	0.25	0.424	6.29E-54	0
SAT1	4.12E-58	-1.036046769	0.571	0.649	9.47E-54	0
DSTN	5.55E-58	-1.026722886	0.194	0.341	1.27E-53	0
SLC2A3	6.66E-58	0.531623309	0.358	0.237	1.53E-53	0
RTN4	7.79E-58	-0.553424425	0.222	0.388	1.79E-53	0
IGFBP3	2.51E-57	-1.956087344	0.142	0.274	5.76E-53	0
PLP2	2.82E-57	0.542802836	0.382	0.269	6.48E-53	0
PDE4B	9.42E-57	0.640908474	0.256	0.153	2.17E-52	0
CANX	1.09E-56	-0.502488036	0.174	0.337	2.51E-52	0
TIMP1	3.58E-56	-1.20167512	0.204	0.347	8.23E-52	0
GLRX	4.21E-55	-0.504646487	0.175	0.334	9.67E-51	0
GSTO1	4.26E-55	-0.561976394	0.197	0.36	9.79E-51	0
FYN	4.96E-55	0.519335879	0.36	0.238	1.14E-50	0
PRDX6	6.49E-55	-0.588533112	0.198	0.363	1.49E-50	0
BNIP3L	9.33E-55	-0.560844636	0.122	0.264	2.15E-50	0
SYNE2	1.11E-54	0.507347534	0.43	0.303	2.54E-50	0
LINC-PINT	1.11E-54	0.652604567	0.287	0.182	2.54E-50	0
FOXP1	1.13E-54	0.711952389	0.338	0.234	2.59E-50	0
CELF2	2.74E-54	0.539106159	0.514	0.4	6.29E-50	0
RNASET2	5.60E-54	-0.936328048	0.26	0.391	1.29E-49	0
HMGN3	4.61E-53	-0.503160244	0.183	0.342	1.06E-48	0
PPT1	1.60E-51	-0.559458358	0.138	0.272	3.67E-47	0
SOCS1	2.09E-51	0.566161679	0.262	0.157	4.81E-47	0
ARHGAP15	3.44E-51	0.637647898	0.314	0.215	7.91E-47	0
ID2	8.69E-51	0.573517094	0.627	0.545	2.00E-46	0
GPX4	9.48E-51	-0.696191129	0.346	0.486	2.18E-46	0
PBXIP1	5.29E-50	0.540143048	0.273	0.169	1.22E-45	0
CAPZA2	5.95E-50	-0.531716627	0.189	0.348	1.37E-45	0
HSPD1	1.87E-49	-0.532420526	0.179	0.327	4.30E-45	0
PRDM1	2.20E-49	0.703563882	0.372	0.268	5.05E-45	0
TUBB	5.18E-49	-0.689559919	0.231	0.383	1.19E-44	0
KIAA1551	1.28E-48	0.609084989	0.416	0.312	2.95E-44	0
GLUL	1.75E-48	-0.848159413	0.206	0.336	4.01E-44	0
CALR	6.42E-48	-0.566625998	0.317	0.481	1.47E-43	0
VDAC1	9.55E-48	-0.543538592	0.166	0.31	2.19E-43	0
NEAT1	2.87E-47	-0.760245598	0.778	0.746	6.59E-43	0
ZC3HAV1	8.40E-47	0.663080461	0.284	0.187	1.93E-42	0
DDX24	4.35E-46	0.504250834	0.515	0.439	9.99E-42	0
DBI	4.40E-46	-0.554886077	0.302	0.459	1.01E-41	0
RPS27L	7.09E-46	-0.674384547	0.357	0.507	1.63E-41	0
POMP	8.89E-46	-0.604170977	0.326	0.485	2.04E-41	0
RHOH	1.18E-44	0.542284705	0.304	0.204	2.72E-40	0
LAMTOR5	1.23E-44	-0.564352092	0.227	0.381	2.83E-40	0
YPEL5	2.21E-44	0.537336293	0.412	0.324	5.08E-40	0
PIK3R1	1.70E-43	0.635351792	0.377	0.288	3.91E-39	0
ZFAND5	3.53E-41	-0.503416627	0.203	0.338	8.12E-37	0
ATP6V1F	5.21E-40	-0.515681147	0.237	0.383	1.20E-35	0
TUBA1B	1.40E-39	-0.771436268	0.318	0.443	3.23E-35	0
VAMP5	2.71E-39	-0.599690396	0.205	0.338	6.22E-35	0
MGST3	1.79E-38	-0.608427245	0.205	0.336	4.10E-34	0
ENO1	1.86E-38	-0.863187596	0.503	0.62	4.28E-34	0
TAGAP	2.34E-38	0.514308029	0.257	0.167	5.38E-34	0
SEPW1	3.83E-38	-0.638921159	0.25	0.392	8.81E-34	0
LGALS1	1.20E-37	-0.846682328	0.499	0.594	2.75E-33	0

ARL4C		1.97E-36	0.507163371	0.285	0.193	4.53E-32	0
ANKRD12		2.06E-36	0.517467143	0.438	0.374	4.73E-32	0
ANXA2		2.46E-36	-0.714941599	0.3	0.421	5.65E-32	0
ITM2B		1.79E-32	-0.563287853	0.778	0.78	4.12E-28	0
NDUFB4		3.23E-32	-0.536692891	0.305	0.442	7.42E-28	0
SDCBP		8.43E-32	-0.528542238	0.279	0.4	1.94E-27	0
HNRNPUL1		1.81E-31	0.588356297	0.286	0.217	4.16E-27	0
IKZF1		2.37E-31	0.516080772	0.303	0.226	5.45E-27	0
CSTB		2.73E-31	-0.602507557	0.3	0.427	6.27E-27	0
HSP90AB1		1.93E-30	-0.51396422	0.534	0.622	4.43E-26	0
MT1X		1.76E-29	-2.022392702	0.208	0.305	4.05E-25	0
	6-Sep	1.31E-28	0.516129817	0.331	0.262	3.01E-24	0
PCSK7		3.78E-27	0.516895808	0.373	0.305	8.68E-23	0
UQCRQ		2.87E-26	-0.537488738	0.363	0.49	6.60E-22	0
LDHA		2.55E-23	-0.98000315	0.508	0.6	5.86E-19	0
IL10RA		3.37E-23	0.528354653	0.302	0.246	7.74E-19	0
VIM		5.60E-23	-0.674969443	0.789	0.766	1.29E-18	0
LGALS3		6.96E-22	-0.508618078	0.176	0.266	1.60E-17	0
MIF		1.40E-20	-0.946560888	0.433	0.527	3.21E-16	0
S100A9		1.49E-19	-2.031981127	0.194	0.268	3.43E-15	0
PEBP1		5.64E-17	-0.527180574	0.316	0.412	1.30E-12	0
TXN		4.83E-15	-0.55903883	0.315	0.411	1.11E-10	0
NR3C1		8.99E-14	0.517413601	0.259	0.223	2.07E-09	0
RBPJ		1.49E-13	0.536460449	0.34	0.316	3.41E-09	0
S100A11		2.43E-12	-0.657558487	0.576	0.595	5.58E-08	0
GNLY.1		0	4.504520706	0.9	0.184	0	1
GZMB		0	3.414580159	0.751	0.079	0	1
KLRD1		0	3.348997425	0.926	0.084	0	1
FGFBP2		0	2.777833612	0.549	0.017	0	1
PRF1		0	2.479451674	0.731	0.162	0	1
NKG7.1		0	2.443413048	0.961	0.371	0	1
KLRF1		0	2.37608845	0.491	0.015	0	1
CCL4.1		0	2.167350763	0.906	0.391	0	1
CLIC3		0	2.115485262	0.489	0.058	0	1
GZMH		0	2.070645914	0.566	0.134	0	1
SPON2		0	1.986892715	0.474	0.097	0	1
PLAC8		0	1.967695752	0.536	0.094	0	1
CD7		0	1.955882978	0.752	0.252	0	1
KLRB1.1		0	1.934496134	0.637	0.167	0	1
TRDC		0	1.875536367	0.366	0.023	0	1
HOPX		0	1.826245902	0.564	0.107	0	1
CST7		0	1.360937648	0.834	0.342	0	1
S1PR5		0	1.224235308	0.254	0.007	0	1
GZMM		1.37E-300	1.35894042	0.467	0.109	3.14E-296	1
CD247		8.76E-299	1.537574079	0.613	0.201	2.01E-294	1
MATK		1.62E-296	1.254609316	0.35	0.059	3.73E-292	1
CTSW		2.78E-276	1.400323733	0.584	0.184	6.40E-272	1
FCGR3A.1		3.80E-249	1.321790208	0.597	0.195	8.74E-245	1
PTGDR		1.26E-232	0.998597538	0.253	0.037	2.90E-228	1
SYTL3.1		6.97E-228	1.289442303	0.614	0.241	1.60E-223	1
CCL5		2.08E-215	1.082883146	0.899	0.526	4.77E-211	1
CMC1.1		2.14E-213	1.517669537	0.605	0.251	4.91E-209	1
CCL3		2.69E-210	2.713236307	0.495	0.17	6.18E-206	1
BIN2.1		1.33E-208	1.251997505	0.552	0.217	3.06E-204	1
GZMA		5.63E-195	0.933490161	0.807	0.378	1.29E-190	1
VIM.1		1.93E-183	-1.758220622	0.579	0.791	4.43E-179	1
XCL2		8.49E-183	1.225757699	0.333	0.081	1.95E-178	1
TXK		1.91E-177	1.210608685	0.334	0.087	4.38E-173	1

SAMD3	4.24E-177	1.08850678	0.364	0.102	9.73E-173	1
KLRG1	4.65E-171	1.194744644	0.327	0.087	1.07E-166	1
DUSP2.1	1.28E-168	1.111995916	0.755	0.397	2.93E-164	1
GPR65	2.34E-166	1.207433618	0.464	0.182	5.37E-162	1
HCST.1	2.62E-156	0.938771228	0.805	0.517	6.02E-152	1
ZEB2.1	4.19E-156	1.159905913	0.496	0.213	9.62E-152	1
PYHIN1	4.99E-154	1.070137992	0.47	0.177	1.15E-149	1
LITAF	2.30E-149	1.006967937	0.631	0.339	5.28E-145	1
MT-CO1	3.77E-142	0.526886926	0.999	0.918	8.67E-138	1
IL2RB	1.67E-141	0.962418081	0.274	0.072	3.85E-137	1
AREG	9.89E-140	2.003368852	0.446	0.184	2.27E-135	1
CD69.1	7.57E-135	1.109261071	0.812	0.499	1.74E-130	1
MT-CO2	1.09E-133	0.561144742	0.985	0.863	2.50E-129	1
ID2.1	4.09E-132	0.913219567	0.805	0.539	9.40E-128	1
TYROBP.1	7.41E-132	0.504782109	0.735	0.351	1.70E-127	1
AKNA	7.51E-131	1.050971594	0.498	0.236	1.73E-126	1
ADGRE5.1	2.81E-125	0.984302974	0.547	0.273	6.45E-121	1
FTH1.1	2.39E-124	-1.477240063	0.969	0.962	5.49E-120	1
HLA-C	3.06E-122	0.505298301	0.985	0.872	7.04E-118	1
FYN.1	4.65E-122	0.879748293	0.512	0.24	1.07E-117	1
IRF1.1	6.87E-122	1.011113537	0.552	0.285	1.58E-117	1
MYL12A	6.97E-121	0.73047169	0.877	0.67	1.60E-116	1
CD74.1	6.59E-118	-2.164522936	0.724	0.801	1.51E-113	1
MAPK1	6.91E-116	1.050734396	0.355	0.139	1.59E-111	1
KLF2	3.37E-115	1.15038372	0.426	0.186	7.74E-111	1
TRGC2	1.55E-114	0.948802351	0.271	0.079	3.55E-110	1
AC092580.4.1	1.49E-113	0.968628754	0.406	0.159	3.43E-109	1
CHST12	9.24E-111	0.856521048	0.426	0.186	2.12E-106	1
MYO1F	1.26E-108	0.943501196	0.48	0.249	2.90E-104	1
HLA-E	1.53E-106	0.542472823	0.951	0.772	3.52E-102	1
IER2	1.18E-105	1.013125692	0.621	0.378	2.72E-101	1
IFITM1	4.41E-105	0.954921797	0.384	0.16	1.01E-100	1
MBP	9.28E-104	1.014335465	0.352	0.147	2.13E-99	1
GAPDH.1	4.00E-103	-1.249366923	0.831	0.87	9.18E-99	1
ANXA1.1	9.60E-102	1.005393537	0.697	0.447	2.21E-97	1
ZAP70	4.49E-101	0.762844029	0.269	0.088	1.03E-96	1
HLA-DRB1.1	1.18E-99	-2.335845252	0.393	0.611	2.71E-95	1
PPP2R5C.1	4.35E-99	0.823267278	0.609	0.367	9.99E-95	1
CCND3.1	1.02E-97	0.788321848	0.572	0.337	2.36E-93	1
SRGN	1.19E-91	0.501767157	0.966	0.773	2.73E-87	1
ARL4C.1	4.14E-91	0.909043081	0.407	0.194	9.50E-87	1
HLA-DRA.1	8.16E-91	-2.541503258	0.524	0.689	1.88E-86	1
CD53.1	3.84E-90	0.707449984	0.683	0.448	8.82E-86	1
RUNX3.1	8.61E-90	0.773042526	0.411	0.188	1.98E-85	1
PTPN4	1.64E-89	0.809074009	0.306	0.119	3.77E-85	1
RAC2.1	4.91E-89	0.770696635	0.583	0.35	1.13E-84	1
FKBP11.1	7.49E-86	0.833780303	0.339	0.143	1.72E-81	1
STK4.1	1.67E-84	0.621524989	0.683	0.45	3.85E-80	1
RNF125	1.32E-83	0.763933751	0.283	0.108	3.04E-79	1
CLEC2B	6.46E-81	0.749285869	0.563	0.359	1.49E-76	1
COTL1	7.04E-81	-1.255388009	0.199	0.459	1.62E-76	1
SUN2	3.28E-80	0.784143915	0.372	0.18	7.55E-76	1
PIP4K2A	1.37E-79	0.747095285	0.406	0.201	3.15E-75	1
HLA-DQB1.1	5.89E-79	-1.833129915	0.17	0.416	1.35E-74	1
RBM39	2.44E-77	0.679586747	0.703	0.529	5.61E-73	1
LYAR.1	5.54E-76	0.89611571	0.369	0.181	1.27E-71	1
ISG20.1	2.21E-71	0.646615972	0.552	0.341	5.09E-67	1
HSPB1.1	1.18E-70	-1.525745617	0.215	0.458	2.71E-66	1

GNG2	1.83E-70	0.742528202	0.295	0.129	4.20E-66	1
RORA.1	4.36E-70	0.642067983	0.4	0.199	1.00E-65	1
TLE4	1.22E-69	0.746515123	0.352	0.172	2.80E-65	1
TSC22D3.1	1.52E-69	0.532712648	0.911	0.767	3.49E-65	1
S100A11.1	2.22E-69	-1.373102169	0.435	0.607	5.10E-65	1
EVL.1	5.70E-69	0.662650617	0.671	0.477	1.31E-64	1
CCL4L2	1.02E-68	1.18132208	0.361	0.174	2.35E-64	1
SYNE1	1.09E-67	0.787855247	0.318	0.15	2.50E-63	1
ANXA5.1	1.68E-66	-1.04026112	0.133	0.375	3.85E-62	1
CYBA	2.14E-66	0.506997138	0.861	0.669	4.91E-62	1
CD27	3.67E-65	-1.391511413	0.078	0.287	8.43E-61	1
RAP1B	5.06E-65	0.758292747	0.476	0.308	1.16E-60	1
TAGAP.1	2.73E-64	0.717576432	0.346	0.171	6.29E-60	1
GPX1.1	7.00E-64	-1.812209021	0.242	0.449	1.61E-59	1
FTL.1	1.05E-63	-1.506990068	0.957	0.935	2.41E-59	1
HLA-DMA.1	4.57E-63	-1.296487115	0.095	0.308	1.05E-58	1
CST3.1	5.25E-63	-2.128669951	0.204	0.405	1.21E-58	1
STAT4	2.05E-62	0.607774718	0.258	0.106	4.70E-58	1
PRDX1.1	6.38E-62	-1.148102276	0.19	0.426	1.47E-57	1
PIK3R1.1	1.26E-61	0.844841755	0.465	0.292	2.90E-57	1
SERPINA1.1	2.00E-59	-1.610540271	0.092	0.298	4.60E-55	1
HLA-DRB5.1	6.81E-59	-1.884866442	0.186	0.393	1.56E-54	1
TBC1D10C.1	3.02E-58	0.653050634	0.394	0.215	6.94E-54	1
RARRES3.1	1.06E-57	0.557376936	0.583	0.392	2.43E-53	1
GNPTAB	2.75E-57	0.753348928	0.292	0.146	6.32E-53	1
CTD-3252C9.4.1	2.77E-56	0.749170323	0.352	0.186	6.36E-52	1
CH17-189H20.1.1	1.01E-55	0.681115912	0.384	0.219	2.32E-51	1
CYTIP.1	3.12E-55	0.515031293	0.652	0.457	7.17E-51	1
EFHD2	3.43E-55	0.792122377	0.273	0.135	7.88E-51	1
AES	1.05E-54	0.715098794	0.395	0.236	2.42E-50	1
LGALS3.1	1.20E-54	-1.001123821	0.068	0.264	2.76E-50	1
PLEK	1.35E-54	0.731563133	0.281	0.138	3.10E-50	1
VAMP2.1	7.90E-54	0.550336935	0.61	0.45	1.82E-49	1
ARHGAP9	1.97E-53	0.704428433	0.32	0.169	4.52E-49	1
HLA-DPA1.1	1.42E-52	-1.95702024	0.442	0.584	3.27E-48	1
CTSB.1	1.59E-52	-1.130106859	0.121	0.321	3.65E-48	1
ATM	1.15E-51	0.767751744	0.383	0.235	2.65E-47	1
TYMP.1	2.97E-51	-1.036548358	0.117	0.314	6.81E-47	1
SELPLG	1.37E-50	0.690849224	0.277	0.139	3.15E-46	1
BTG2.1	3.72E-50	0.69026246	0.508	0.341	8.56E-46	1
RNASET2.1	4.84E-50	-1.206422207	0.187	0.379	1.11E-45	1
APMAP	6.21E-50	0.706422298	0.343	0.199	1.43E-45	1
LTB.1	8.08E-50	-1.250017647	0.132	0.319	1.86E-45	1
FCGRT.1	1.00E-49	-1.048942666	0.077	0.259	2.30E-45	1
HLA-DQA1.1	1.96E-48	-1.704317734	0.182	0.361	4.51E-44	1
ETS1.1	1.00E-47	0.512753181	0.506	0.321	2.30E-43	1
NPC2.1	1.41E-47	-1.463639658	0.218	0.396	3.23E-43	1
RP5-1171I10.5	1.44E-47	0.67716138	0.259	0.127	3.31E-43	1
LCP1.1	3.26E-47	0.558485692	0.546	0.389	7.50E-43	1
EIF3G	4.06E-47	0.609669548	0.535	0.408	9.34E-43	1
AIF1.1	8.13E-47	-1.542138773	0.104	0.276	1.87E-42	1
GRN.1	1.11E-46	-1.092707982	0.093	0.271	2.54E-42	1
ITGB2	1.18E-46	0.644009973	0.559	0.4	2.70E-42	1
ABI3	2.77E-46	0.66818437	0.294	0.163	6.36E-42	1
S100A6	3.47E-46	-0.934861279	0.71	0.772	7.98E-42	1
NR4A2	6.35E-46	0.688601388	0.415	0.262	1.46E-41	1
CDC42SE1.1	1.52E-45	0.642546087	0.375	0.225	3.48E-41	1
LINC00869	2.16E-45	0.625434985	0.269	0.138	4.97E-41	1

IFITM3.1	3.39E-45	-1.71170248	0.215	0.396	7.79E-41	1
PPT1.1	3.51E-45	-0.793945389	0.082	0.258	8.07E-41	1
CORO1B.1	5.94E-45	-0.690665562	0.076	0.257	1.37E-40	1
LYZ.1	3.43E-44	-2.429199927	0.203	0.367	7.87E-40	1
PTMS.1	4.35E-44	-0.878057279	0.084	0.258	1.00E-39	1
ZFP36.1	1.29E-43	0.578392857	0.793	0.65	2.96E-39	1
PKM.1	1.58E-43	-1.022872041	0.26	0.452	3.62E-39	1
PTGER4.1	1.99E-43	0.658457843	0.355	0.208	4.58E-39	1
ADIRF.1	5.91E-43	-1.972746532	0.091	0.257	1.36E-38	1
NNMT.1	1.77E-42	-2.028455662	0.14	0.316	4.07E-38	1
C1orf56	2.78E-42	0.724650488	0.316	0.173	6.39E-38	1
PTPN7.1	4.96E-42	0.584373638	0.356	0.207	1.14E-37	1
ARPC5L	1.79E-41	0.578298427	0.358	0.219	4.11E-37	1
TIMP1.1	1.99E-41	-1.348324837	0.153	0.331	4.58E-37	1
AOAH	3.73E-41	0.664941355	0.253	0.134	8.57E-37	1
ARHGEF1.1	5.26E-41	0.534131022	0.39	0.249	1.21E-36	1
WIPF1.1	7.58E-41	0.547281868	0.468	0.33	1.74E-36	1
SOD2.1	1.19E-40	-1.372091277	0.135	0.31	2.73E-36	1
IQGAP2	3.59E-40	0.623454605	0.289	0.163	8.24E-36	1
NDUFA4L2.1	1.01E-39	-2.150575946	0.141	0.304	2.32E-35	1
YWHAH.1	2.53E-39	-1.082648181	0.132	0.299	5.80E-35	1
RGS10	3.52E-39	-0.737060568	0.139	0.312	8.08E-35	1
ENO1.1	1.03E-38	-1.212167921	0.466	0.607	2.38E-34	1
NDUFC1.1	2.26E-38	-1.018494718	0.116	0.287	5.19E-34	1
GPATCH8	2.84E-38	0.686099442	0.343	0.216	6.53E-34	1
LIMD2	3.06E-38	0.554131287	0.436	0.297	7.04E-34	1
CRYAB.1	8.75E-38	-1.823524835	0.114	0.274	2.01E-33	1
C9orf142.1	1.49E-37	0.59416792	0.376	0.249	3.43E-33	1
DOK2	3.01E-37	0.566968413	0.254	0.136	6.92E-33	1
IKZF1.1	6.36E-37	0.553004896	0.364	0.231	1.46E-32	1
ITM2B.1	8.02E-37	-0.756870122	0.796	0.777	1.84E-32	1
RSRP1.1	9.25E-37	0.518534268	0.523	0.401	2.13E-32	1
PRKCH	9.44E-37	0.514024185	0.376	0.24	2.17E-32	1
FKBP1A.1	1.01E-36	-1.037758241	0.251	0.43	2.32E-32	1
PARP8.1	2.35E-36	0.527270606	0.325	0.192	5.39E-32	1
LIMS1.1	7.51E-36	-0.587204429	0.102	0.264	1.73E-31	1
GPX4.1	8.53E-36	-0.896779973	0.309	0.469	1.96E-31	1
NFKBIA.1	8.75E-35	0.664114411	0.663	0.547	2.01E-30	1
SOCS1.1	1.81E-34	0.542584686	0.293	0.169	4.15E-30	1
TMEM2	3.63E-34	0.543888674	0.289	0.167	8.34E-30	1
RAC1.1	5.61E-34	-0.928157893	0.32	0.462	1.29E-29	1
FLNA	1.10E-33	0.539764713	0.287	0.173	2.53E-29	1
RBPJ.1	2.88E-33	-0.716402101	0.168	0.337	6.61E-29	1
RGS1.1	2.42E-32	-0.886923549	0.671	0.699	5.55E-28	1
JAK1	3.43E-32	0.599027128	0.485	0.386	7.88E-28	1
LGALS1.1	3.44E-31	-1.035963737	0.458	0.584	7.90E-27	1
S100A10	1.79E-30	-1.003992906	0.53	0.618	4.12E-26	1
CNOT6L.1	2.26E-30	0.51523949	0.337	0.217	5.19E-26	1
SPN	7.87E-30	0.530152695	0.255	0.148	1.81E-25	1
APOC1.1	1.20E-29	-1.819351867	0.16	0.297	2.75E-25	1
CD47	2.18E-29	0.552703884	0.336	0.233	5.02E-25	1
ANXA2.1	6.43E-29	-0.899930924	0.256	0.408	1.48E-24	1
CD84	7.01E-29	-0.508386603	0.11	0.253	1.61E-24	1
PLIN2.1	1.75E-28	-1.213961287	0.2	0.35	4.02E-24	1
HLA-DPB1.1	2.00E-28	-1.665707065	0.587	0.637	4.60E-24	1
IGFBP3.1	5.70E-28	-2.024564236	0.127	0.256	1.31E-23	1
ALDOA.1	2.16E-27	-0.905461174	0.555	0.646	4.97E-23	1
IDI1	2.97E-27	0.504615399	0.291	0.187	6.83E-23	1

DUSP4.1	5.53E-27	-0.826933119	0.124	0.253	1.27E-22	1
GZMK.1	7.47E-27	-1.411937483	0.263	0.376	1.72E-22	1
TXN.1	1.11E-26	-0.899561503	0.251	0.404	2.55E-22	1
MT1X.1	3.00E-26	-2.14961381	0.163	0.296	6.90E-22	1
YWHAE.1	4.15E-26	-0.665638759	0.173	0.327	9.53E-22	1
MGST3.1	6.63E-26	-0.721328291	0.174	0.32	1.52E-21	1
SAT1.1	7.02E-26	-1.091790048	0.587	0.636	1.61E-21	1
REL	1.09E-25	0.55481	0.418	0.312	2.51E-21	1
RPS27L.1	2.93E-25	-0.843674274	0.345	0.486	6.72E-21	1
MT2A.1	3.40E-25	-1.793611813	0.576	0.668	7.82E-21	1
C1QA.1	6.30E-25	-1.643348255	0.141	0.262	1.45E-20	1
GSTO1.1	1.96E-24	-0.587632295	0.185	0.337	4.50E-20	1
HSBP1.1	2.07E-24	-0.565214248	0.117	0.252	4.77E-20	1
RHOB.1	6.74E-24	-0.984791398	0.231	0.361	1.55E-19	1
PSAP.1	1.59E-23	-1.032454709	0.379	0.478	3.66E-19	1
CD55	3.21E-23	0.510695009	0.263	0.172	7.37E-19	1
DYNLL1.1	1.85E-22	-0.639639006	0.272	0.424	4.24E-18	1
LAPTM4A.1	2.26E-22	-0.709764386	0.226	0.365	5.20E-18	1
SLFN5	2.79E-22	0.511504622	0.292	0.196	6.40E-18	1
APOE.1	3.20E-22	-2.202681355	0.341	0.448	7.35E-18	1
BIRC3.1	3.79E-22	-0.761260788	0.161	0.284	8.71E-18	1
PEBP1.1	7.64E-22	-0.765069545	0.26	0.404	1.76E-17	1
FMNL1	2.14E-21	0.509300433	0.262	0.177	4.92E-17	1
NAP1L1	3.42E-21	-0.561674787	0.467	0.575	7.86E-17	1
HERPUD1.1	4.28E-21	-0.790981532	0.399	0.502	9.85E-17	1
CLTA.1	1.10E-20	-0.518474785	0.168	0.304	2.52E-16	1
RPLP0	2.24E-20	-0.502956909	0.871	0.84	5.15E-16	1
SOD1.1	2.74E-20	-0.631245661	0.355	0.495	6.29E-16	1
APLP2.1	7.36E-20	-0.600766083	0.147	0.267	1.69E-15	1
HINT1	1.04E-19	-0.585255388	0.538	0.635	2.39E-15	1
MIF.1	1.92E-19	-1.123779991	0.399	0.517	4.42E-15	1
VMP1	2.88E-19	-0.550919362	0.2	0.333	6.61E-15	1
SNX3.1	2.01E-16	-0.551330256	0.268	0.396	4.61E-12	1
CSTB.1	5.78E-16	-0.646898471	0.284	0.41	1.33E-11	1
LDHB.1	2.86E-15	-0.520928383	0.36	0.478	6.57E-11	1
LDHA.1	7.88E-15	-1.083775795	0.511	0.586	1.81E-10	1
S100A9.1	7.94E-15	-2.202742054	0.168	0.26	1.82E-10	1
TSPO.1	1.32E-13	-0.504604036	0.331	0.43	3.03E-09	1
VAMP5.1	2.94E-11	-0.54703549	0.221	0.317	6.76E-07	1
TPI1.1	3.85E-11	-0.773908754	0.506	0.571	8.86E-07	1
TRAC.1	6.31E-10	-0.640398036	0.389	0.437	1.45E-05	1
NDUFB4.1	3.01E-09	-0.512742192	0.321	0.42	6.91E-05	1
TUBA1B.1	3.81E-09	-0.690453188	0.349	0.421	8.74E-05	1
TUBB.1	4.78E-08	-0.553257657	0.274	0.356	0.001097496	1
ZFAS1.1	1.30E-07	-0.537741056	0.414	0.491	0.002992414	1
SEPW1.1	2.11E-07	-0.598322906	0.286	0.367	0.00485905	1
PGK1.1	3.75E-07	-0.509862168	0.376	0.458	0.008607529	1
CTSS.1	9.87E-07	-0.807034747	0.328	0.366	0.022682553	1
GLUL.1	1.98E-06	-0.621623764	0.259	0.311	0.045407077	1
GZMK.2	0	2.333501979	0.903	0.316	0	2
CCL5.1	0	1.734788642	0.967	0.525	0	2
CD8A	2.40E-280	1.533642036	0.63	0.193	5.51E-276	2
CCL4.2	2.87E-274	1.505940932	0.846	0.403	6.59E-270	2
CD27.1	5.40E-265	1.527700935	0.662	0.231	1.24E-260	2
GZMA.1	1.58E-255	1.376683621	0.835	0.381	3.63E-251	2
CD8B	2.08E-240	1.443967711	0.527	0.152	4.78E-236	2
RGS1.2	3.93E-240	1.283729333	0.956	0.673	9.02E-236	2
CST7.1	2.36E-224	1.334719915	0.774	0.354	5.43E-220	2

NKG7.2	1.81E-211	1.039741725	0.844	0.389	4.15E-207	2
DUSP4.2	1.43E-201	1.372261871	0.579	0.21	3.29E-197	2
CCL4L2.1	4.15E-198	1.799205287	0.498	0.164	9.54E-194	2
CD3D.2	7.30E-194	1.121365372	0.839	0.441	1.68E-189	2
DUSP2.2	1.35E-180	1.156250451	0.771	0.4	3.09E-176	2
ITM2A.2	7.91E-174	1.450452584	0.615	0.283	1.82E-169	2
CRTAM	2.37E-172	1.427408893	0.315	0.075	5.45E-168	2
BTG1.2	1.26E-164	0.803136393	0.98	0.83	2.90E-160	2
HLA-DQA2	3.22E-142	0.889723525	0.432	0.144	7.39E-138	2
CXCR4.2	1.10E-140	0.815892586	0.956	0.759	2.53E-136	2
CD2.1	5.92E-136	0.873874712	0.79	0.426	1.36E-131	2
HLA-A	6.60E-130	0.611755844	0.977	0.886	1.52E-125	2
CD3E.1	4.86E-129	0.97626453	0.68	0.372	1.12E-124	2
TRAC.2	6.51E-115	0.766063791	0.74	0.404	1.50E-110	2
TRBC2.1	8.62E-113	1.062427601	0.638	0.353	1.98E-108	2
VIM.2	6.26E-112	-1.443120658	0.613	0.785	1.44E-107	2
RPS26.1	8.45E-106	0.555521796	0.966	0.861	1.94E-101	2
FTH1.2	1.98E-104	-1.480533081	0.952	0.963	4.55E-100	2
APOBEC3G.1	1.19E-101	1.058743691	0.499	0.251	2.73E-97	2
RP11-347P5.1.2	2.63E-99	0.967156901	0.608	0.354	6.05E-95	2
TOX	5.08E-98	0.886880821	0.279	0.09	1.17E-93	2
CMC1.2	1.35E-96	1.467521904	0.502	0.265	3.10E-92	2
LYST.1	5.15E-95	1.111218906	0.437	0.208	1.18E-90	2
TRBC1.1	5.85E-92	1.041277146	0.591	0.331	1.34E-87	2
CD69.2	1.54E-91	0.666606513	0.775	0.506	3.55E-87	2
CTSW.1	2.56E-88	0.870353729	0.441	0.202	5.88E-84	2
LCK.2	3.96E-86	0.895988903	0.497	0.268	9.11E-82	2
TPT1.1	1.75E-81	-0.604456559	0.934	0.923	4.02E-77	2
LAG3	3.86E-81	0.922344341	0.256	0.087	8.88E-77	2
TNFRSF9	2.96E-75	0.859322566	0.252	0.088	6.80E-71	2
SRSF7.1	1.69E-74	0.817492696	0.71	0.53	3.89E-70	2
HCST.2	2.91E-72	0.710230451	0.723	0.528	6.69E-68	2
EVL.2	3.20E-70	0.780525234	0.661	0.48	7.35E-66	2
CD3G.1	7.82E-69	0.78087035	0.497	0.275	1.80E-64	2
CORO1A.2	9.12E-69	0.663811529	0.736	0.549	2.10E-64	2
PTPRC.2	6.03E-68	0.590423911	0.811	0.599	1.39E-63	2
STK4.2	8.16E-65	0.785300461	0.628	0.458	1.87E-60	2
TRAT1.1	2.43E-64	0.757144463	0.284	0.118	5.59E-60	2
SIT1	1.51E-63	0.805995953	0.275	0.116	3.46E-59	2
TIMP1.2	2.35E-63	-1.556264715	0.091	0.335	5.39E-59	2
ACAP1.2	1.64E-62	0.81569346	0.507	0.317	3.78E-58	2
IFITM2.1	3.58E-61	-1.292738636	0.234	0.497	8.24E-57	2
TMEM123.1	3.91E-56	-0.820710321	0.065	0.29	8.97E-52	2
CCDC167	8.87E-56	0.846008139	0.32	0.162	2.04E-51	2
CEBPD.2	1.11E-55	-1.264758642	0.083	0.297	2.56E-51	2
IFNGR1	2.52E-53	-0.978719338	0.066	0.277	5.79E-49	2
SUB1	6.68E-53	0.567694959	0.683	0.564	1.53E-48	2
CCL3.1	1.09E-52	0.868012622	0.357	0.187	2.50E-48	2
IFITM3.2	2.07E-52	-1.71860529	0.174	0.397	4.77E-48	2
ITM2C	8.42E-52	0.726046571	0.301	0.144	1.93E-47	2
RARRES3.2	1.32E-51	0.704141037	0.557	0.397	3.04E-47	2
IL2RG.2	6.96E-50	0.699706195	0.532	0.358	1.60E-45	2
KIAA1551.1	3.37E-49	0.741423805	0.479	0.322	7.75E-45	2
CD84.1	4.17E-49	0.888508542	0.375	0.227	9.59E-45	2
RHOB.2	5.35E-49	-1.380158151	0.155	0.366	1.23E-44	2
CLEC2D.1	5.36E-49	0.763376755	0.459	0.292	1.23E-44	2
SOD2.2	7.08E-49	-1.457840299	0.102	0.311	1.63E-44	2
SERPINA1.2	1.03E-48	-1.548493469	0.095	0.295	2.37E-44	2

DSTN.1	2.77E-48	-1.19965549	0.112	0.325	6.36E-44	2
TERF2IP.1	9.08E-47	0.76648508	0.485	0.354	2.09E-42	2
XIST	2.14E-46	0.583361746	0.583	0.41	4.91E-42	2
CST3.2	1.05E-44	-1.898843618	0.22	0.401	2.41E-40	2
TYROBP.2	5.74E-44	-1.826624325	0.232	0.402	1.32E-39	2
PYHIN1.1	7.58E-44	0.697612209	0.347	0.192	1.74E-39	2
FCER1G.2	4.45E-43	-1.503799815	0.138	0.318	1.02E-38	2
TMSB10.1	4.48E-43	-0.514556278	0.969	0.96	1.03E-38	2
APLP2.2	1.96E-42	-0.795875717	0.081	0.271	4.51E-38	2
NNMT.2	3.71E-41	-2.052252806	0.127	0.315	8.52E-37	2
GRN.2	6.78E-41	-1.0677958	0.091	0.269	1.56E-36	2
RNASET2.2	7.96E-41	-1.05358737	0.18	0.377	1.83E-36	2
GZMH.1	1.80E-40	0.695208552	0.31	0.163	4.14E-36	2
PSMB9	2.06E-40	0.580332156	0.585	0.475	4.74E-36	2
GLUL.2	1.05E-39	-1.095597367	0.135	0.322	2.41E-35	2
S100A11.2	2.55E-39	-1.132643811	0.452	0.603	5.86E-35	2
TUBA4A.2	8.40E-39	0.594042999	0.409	0.26	1.93E-34	2
ISG20.2	6.51E-37	0.576314052	0.485	0.35	1.50E-32	2
PLIN2.2	8.57E-37	-1.301467676	0.164	0.352	1.97E-32	2
CYTIP.2	3.27E-36	0.517852043	0.6	0.464	7.51E-32	2
LINC00152.1	3.95E-36	0.616799513	0.437	0.306	9.07E-32	2
CTSB.2	1.76E-35	-0.929522861	0.14	0.317	4.04E-31	2
WNK1	2.46E-35	0.789303122	0.356	0.239	5.66E-31	2
NDUFA4L2.2	1.30E-34	-2.095451289	0.138	0.302	2.99E-30	2
PPP2R5C.2	9.85E-34	0.600449814	0.508	0.379	2.26E-29	2
STK17A.2	2.16E-33	0.577455923	0.489	0.355	4.97E-29	2
HLA-F.2	2.48E-33	0.595713422	0.535	0.429	5.70E-29	2
C10orf54.1	3.28E-33	-0.641206102	0.121	0.296	7.55E-29	2
LDHA.2	3.40E-33	-1.277658082	0.413	0.594	7.82E-29	2
RAC2.2	3.87E-33	0.573409892	0.488	0.362	8.90E-29	2
ANXA2.2	5.17E-33	-0.909444523	0.217	0.409	1.19E-28	2
DDIT4	2.16E-32	-0.987884164	0.343	0.534	4.97E-28	2
FCGRT.2	2.59E-32	-0.76334814	0.095	0.255	5.96E-28	2
S100A10.1	3.10E-32	-0.989433156	0.469	0.623	7.13E-28	2
CD63.2	2.49E-31	-0.945363789	0.379	0.533	5.72E-27	2
RPL17	4.46E-31	-0.737767036	0.465	0.605	1.02E-26	2
PNISR.1	5.25E-31	0.562095679	0.586	0.503	1.21E-26	2
MSI2	1.82E-30	0.667794125	0.26	0.151	4.19E-26	2
CRYAB.2	2.80E-30	-1.720365413	0.118	0.272	6.43E-26	2
ASAH1.1	3.86E-30	-0.656658025	0.097	0.254	8.87E-26	2
APOBEC3C	5.54E-30	0.805618277	0.301	0.189	1.27E-25	2
HSPB1.2	1.69E-29	-1.153698334	0.284	0.448	3.89E-25	2
PMAIP1.1	4.38E-29	0.691953786	0.313	0.193	1.01E-24	2
BTN3A2.2	6.98E-29	0.66462631	0.364	0.254	1.60E-24	2
MT-ND4L	1.40E-28	-0.534064802	0.19	0.376	3.21E-24	2
GPX1.2	1.45E-28	-1.451932602	0.301	0.441	3.32E-24	2
FTL.2	1.62E-28	-1.307326105	0.943	0.936	3.72E-24	2
ANXA6	2.16E-28	0.546380735	0.355	0.24	4.97E-24	2
PTPN7.2	6.20E-28	0.622207496	0.327	0.212	1.43E-23	2
ZBTB38	6.37E-28	0.641989169	0.337	0.228	1.46E-23	2
ADIRF.2	1.37E-27	-1.768097442	0.112	0.253	3.15E-23	2
HAVCR2	1.37E-27	0.915300165	0.291	0.192	3.16E-23	2
YWHAE.2	1.51E-27	-0.726279802	0.155	0.327	3.46E-23	2
TMEM2.1	1.53E-27	0.669047484	0.279	0.169	3.53E-23	2
YWHAH.2	2.11E-27	-0.872169594	0.142	0.296	4.85E-23	2
LAPTM4A.2	2.22E-27	-0.712121277	0.19	0.367	5.10E-23	2
AC090498.1	3.24E-26	-0.804954939	0.369	0.513	7.44E-22	2
CFLAR.1	4.37E-26	0.561144657	0.497	0.415	1.00E-21	2

IL7R.1	7.58E-26	-1.23976131	0.182	0.314	1.74E-21	2
AP3S1.1	8.34E-26	0.615755897	0.359	0.264	1.92E-21	2
RTN4.2	8.55E-26	-0.600923289	0.189	0.365	1.97E-21	2
ITM2B.2	1.29E-25	-0.655732423	0.753	0.782	2.97E-21	2
PPP1R2.1	3.24E-25	0.641792146	0.424	0.335	7.46E-21	2
GALM	8.26E-25	0.635270646	0.255	0.157	1.90E-20	2
TYMP.2	8.71E-25	-0.735158364	0.159	0.308	2.00E-20	2
RAC1.2	1.81E-24	-0.745890662	0.313	0.461	4.16E-20	2
RHOH.1	7.96E-24	0.584109469	0.321	0.219	1.83E-19	2
TBC1D10C.2	9.49E-24	0.575001663	0.326	0.224	2.18E-19	2
ZFAND5.2	1.30E-23	-0.591762363	0.165	0.32	2.98E-19	2
NPC2.2	6.19E-23	-1.103216022	0.256	0.39	1.42E-18	2
LAT	2.53E-22	0.566044003	0.255	0.159	5.80E-18	2
RUNX3.2	1.17E-21	0.519912515	0.303	0.201	2.68E-17	2
TAGLN2.1	1.22E-21	-0.538884737	0.205	0.37	2.81E-17	2
PPT1.2	2.26E-21	-0.512609628	0.121	0.252	5.20E-17	2
ANXA1.2	4.89E-21	-0.719250765	0.333	0.484	1.12E-16	2
UCP2.1	1.86E-20	0.586941932	0.416	0.34	4.27E-16	2
CCND3.2	2.26E-20	0.549457946	0.43	0.353	5.20E-16	2
TSPYL2.2	5.81E-20	0.61184301	0.325	0.231	1.34E-15	2
PSAP.2	8.94E-20	-0.934850291	0.359	0.478	2.05E-15	2
TRAF3IP3.2	1.59E-19	0.521140204	0.337	0.248	3.66E-15	2
MIF.2	2.23E-19	-1.074895481	0.365	0.518	5.12E-15	2
MGST3.2	4.67E-19	-0.557332285	0.174	0.319	1.07E-14	2
DOCK8.1	6.46E-19	0.629705883	0.357	0.285	1.49E-14	2
LYZ.2	1.48E-18	-2.070342964	0.26	0.359	3.41E-14	2
GPATCH8.1	2.12E-18	0.544721503	0.303	0.222	4.87E-14	2
SNX3.2	3.82E-18	-0.541789574	0.24	0.397	8.77E-14	2
CEBPB.1	5.81E-18	-0.617739819	0.202	0.34	1.34E-13	2
CHST12.1	6.96E-18	0.539433815	0.286	0.202	1.60E-13	2
WIPF1.2	1.14E-17	0.524384353	0.406	0.337	2.61E-13	2
GPSM3.1	1.75E-17	0.503423124	0.444	0.385	4.03E-13	2
CBLB.1	1.90E-17	0.574152742	0.262	0.182	4.37E-13	2
PCSK7.2	2.04E-17	0.535991308	0.39	0.314	4.70E-13	2
PIP4K2A.1	4.30E-17	0.606221838	0.291	0.214	9.88E-13	2
PRKCH.1	5.66E-17	0.537864135	0.322	0.247	1.30E-12	2
IGFBP3.2	5.88E-17	-1.811449979	0.147	0.253	1.35E-12	2
LIMD2.1	6.24E-17	0.517634395	0.38	0.304	1.43E-12	2
LGALS3.2	9.63E-17	-0.586916216	0.137	0.256	2.21E-12	2
NDUFC1.2	2.01E-16	-0.640887823	0.155	0.281	4.62E-12	2
AIF1.2	7.34E-15	-1.109996205	0.177	0.267	1.69E-10	2
HLA-DRA.2	7.53E-15	-0.979860709	0.816	0.66	1.73E-10	2
IGFBP7.2	1.47E-14	-2.396083598	0.279	0.388	3.37E-10	2
GNLY.2	5.51E-14	-2.273847838	0.17	0.26	1.27E-09	2
ENO1.2	7.86E-14	-0.877963563	0.485	0.603	1.81E-09	2
TUBA1B.2	2.67E-13	-0.561045899	0.297	0.425	6.14E-09	2
NFKBIA.2	6.84E-13	-0.541043926	0.437	0.569	1.57E-08	2
SEPT6.1	3.93E-12	0.540990098	0.327	0.273	9.03E-08	2
C1QA.2	3.10E-10	-1.341463998	0.18	0.256	7.13E-06	2
S100A6.1	5.83E-10	-0.646409936	0.764	0.766	1.34E-05	2
NEAT1.1	7.16E-10	-0.586416387	0.777	0.751	1.65E-05	2
TPI1.2	4.46E-09	-0.675414955	0.475	0.573	0.000102474	2
FOS.2	7.42E-08	-0.593387625	0.687	0.681	0.001704336	2
TXN.2	8.71E-08	-0.54170774	0.289	0.398	0.002000924	2
SAT1.2	3.59E-07	-0.779629902	0.604	0.634	0.008245408	2
APOC1.2	8.80E-07	-1.35676428	0.227	0.289	0.02021482	2
MT2A.2	1.53E-06	-1.594819752	0.618	0.663	0.035146415	2
HSPA1B	0	3.010641075	0.57	0.115	0	3

RPS4Y1	0	2.207207157	0.78	0.075	0	3
HSPA1A.1	5.04E-285	2.731748998	0.669	0.204	1.16E-280	3
HSPA6	5.11E-283	2.792778633	0.406	0.068	1.17E-278	3
DNAJB4	4.53E-247	1.537786305	0.385	0.068	1.04E-242	3
AC090498.1.1	3.80E-189	1.186071824	0.888	0.474	8.74E-185	3
RPL17.1	1.32E-176	1.068135973	0.917	0.571	3.02E-172	3
PSMD5-AS1	2.68E-170	0.863701513	0.362	0.083	6.15E-166	3
TRBC2.2	1.42E-146	1.041154126	0.779	0.349	3.26E-142	3
CTSW.2	5.33E-143	1.122089474	0.558	0.199	1.23E-138	3
TRAC.3	1.83E-141	0.913795334	0.844	0.403	4.21E-137	3
CD27.2	2.03E-141	0.916988511	0.643	0.241	4.65E-137	3
XIST.1	1.30E-140	-2.154046027	0.004	0.453	2.99E-136	3
GOLGA8A	3.85E-140	0.688626252	0.307	0.068	8.86E-136	3
CD3E.2	1.77E-139	0.83548767	0.819	0.368	4.06E-135	3
LINC00649	1.79E-130	0.641072169	0.294	0.065	4.10E-126	3
CD3G.2	5.07E-128	0.756755172	0.681	0.266	1.16E-123	3
HSPH1	3.86E-126	1.464690942	0.408	0.135	8.87E-122	3
NABP1	1.40E-125	0.912002054	0.471	0.168	3.21E-121	3
CD2.2	8.96E-123	0.772809465	0.859	0.429	2.06E-118	3
PTPRC.3	2.42E-121	0.806189265	0.924	0.595	5.57E-117	3
SLFN5.1	2.60E-121	0.818927886	0.511	0.184	5.97E-117	3
OXNAD1	5.56E-120	0.672074334	0.405	0.119	1.28E-115	3
ITM2A.3	3.56E-117	0.853441102	0.668	0.285	8.19E-113	3
CRIP1.1	3.00E-116	0.796247252	0.64	0.269	6.90E-112	3
TBC1D4	2.90E-109	0.769334932	0.287	0.074	6.67E-105	3
SIRPG	1.13E-108	0.623816533	0.292	0.074	2.60E-104	3
GOLGA8B	6.67E-102	0.556301686	0.303	0.082	1.53E-97	3
HNRNPA1L2	4.64E-101	0.722761992	0.398	0.143	1.07E-96	3
SPOCK2.1	1.55E-98	0.640765674	0.537	0.213	3.55E-94	3
KIAA1551.2	2.00E-97	0.717769103	0.649	0.314	4.60E-93	3
TTN	5.47E-96	0.687623906	0.279	0.074	1.26E-91	3
PYHIN1.2	2.58E-95	0.677877168	0.495	0.185	5.94E-91	3
RPS4X.1	3.87E-95	-0.68451463	0.97	0.936	8.88E-91	3
HLA-DRB5.3	7.62E-92	-2.661939953	0.064	0.395	1.75E-87	3
CACYBP	1.08E-90	0.938047246	0.445	0.19	2.48E-86	3
CD3D.3	1.11E-90	0.685262145	0.82	0.451	2.56E-86	3
EOMES	5.61E-90	0.690900996	0.271	0.074	1.29E-85	3
TRAF3IP3.3	1.29E-88	0.599261873	0.548	0.235	2.95E-84	3
TNFRSF9.1	1.38E-87	0.675733808	0.299	0.088	3.18E-83	3
GABPB1-AS1	3.70E-86	0.75899293	0.487	0.211	8.51E-82	3
DENND2D	5.38E-86	0.627660655	0.373	0.129	1.24E-81	3
RPS29.1	7.96E-86	0.505124713	0.964	0.918	1.83E-81	3
HSPA8	9.29E-84	0.978037565	0.817	0.536	2.13E-79	3
PILRB	6.41E-83	0.596125945	0.285	0.089	1.47E-78	3
HSPE1.1	7.03E-83	1.239314492	0.621	0.367	1.61E-78	3
FTH1.3	7.53E-83	-1.447237672	0.988	0.961	1.73E-78	3
LTB.2	3.90E-81	0.845249631	0.594	0.281	8.97E-77	3
ITGA4.1	1.94E-80	0.713183758	0.478	0.208	4.46E-76	3
MSI2.1	2.12E-80	0.644866335	0.38	0.144	4.88E-76	3
IFITM1.1	2.71E-80	0.682462269	0.421	0.165	6.23E-76	3
HSPD1.3	8.24E-80	1.152729394	0.53	0.277	1.89E-75	3
CD82	1.34E-79	0.627249634	0.303	0.099	3.08E-75	3
HSP90AA1.1	5.26E-79	1.764461987	0.854	0.64	1.21E-74	3
MT-ND5.1	3.80E-77	0.655586364	0.891	0.591	8.74E-73	3
DNAJB1.2	1.03E-76	1.922216672	0.616	0.406	2.37E-72	3
DUSP1.1	6.32E-76	-0.948674773	0.641	0.804	1.45E-71	3
SKAP1.2	1.35E-74	0.54845498	0.43	0.168	3.10E-70	3
TXK.1	1.96E-74	0.630197754	0.298	0.098	4.51E-70	3

LCP1.3	3.57E-74	0.61702763	0.696	0.384	8.20E-70	3
IKZF1.3	9.20E-73	0.537192024	0.5	0.226	2.11E-68	3
CCND2	6.47E-71	0.603573151	0.371	0.145	1.49E-66	3
CCL3L3	1.19E-70	0.621871407	0.263	0.084	2.73E-66	3
IL32.3	4.20E-70	0.700876734	0.849	0.559	9.65E-66	3
SH2D1A	4.59E-70	0.500649257	0.356	0.129	1.05E-65	3
RBL2	9.33E-70	0.660829156	0.385	0.161	2.14E-65	3
EMB.2	1.13E-69	0.561349129	0.525	0.251	2.60E-65	3
IL2RG.3	1.92E-69	0.514996562	0.677	0.351	4.40E-65	3
RNF213.2	2.85E-69	0.621855312	0.657	0.368	6.54E-65	3
CD8A.1	3.65E-68	0.788913855	0.468	0.213	8.39E-64	3
MDFIC	7.27E-68	0.50117849	0.285	0.099	1.67E-63	3
MT-ND4L.1	1.86E-66	0.634072634	0.622	0.342	4.27E-62	3
AAK1.3	7.11E-66	0.538481243	0.637	0.352	1.63E-61	3
ARAP2	5.82E-65	0.511355516	0.348	0.134	1.34E-60	3
LAPTM5.1	1.36E-64	0.51368318	0.875	0.546	3.13E-60	3
PBXIP1.1	4.64E-64	0.558367084	0.41	0.178	1.07E-59	3
PIK3IP1.3	5.92E-64	0.508867791	0.633	0.339	1.36E-59	3
CD8B.1	1.71E-63	0.672323874	0.4	0.169	3.92E-59	3
ITM2C.1	1.15E-61	0.554812623	0.356	0.143	2.63E-57	3
TRBC1.2	2.19E-61	0.844562365	0.613	0.335	5.04E-57	3
BCL2	2.48E-61	0.694115415	0.361	0.158	5.69E-57	3
ZFP36.2	5.22E-61	-1.284971169	0.535	0.672	1.20E-56	3
CYLD	1.69E-60	0.565658087	0.375	0.164	3.88E-56	3
LYST.2	2.38E-60	0.623111984	0.446	0.212	5.47E-56	3
RAC2.3	1.48E-59	0.503708124	0.647	0.353	3.40E-55	3
RP11-796E2.4	1.84E-59	0.559345199	0.339	0.139	4.23E-55	3
UCP2.2	7.11E-59	0.515453978	0.603	0.329	1.63E-54	3
NR4A1	1.12E-56	0.743297169	0.27	0.105	2.58E-52	3
CHORDC1	3.98E-56	0.518160039	0.266	0.1	9.14E-52	3
PHTF2	5.58E-56	0.54491944	0.268	0.1	1.28E-51	3
TRIM22	5.10E-55	0.551381266	0.456	0.234	1.17E-50	3
GZMK.3	6.28E-55	0.658966454	0.604	0.349	1.44E-50	3
JUNB.2	1.44E-49	-0.778875079	0.55	0.735	3.31E-45	3
TSC22D3.2	1.05E-48	-1.067481107	0.815	0.778	2.40E-44	3
UGP2.2	1.29E-48	0.57446959	0.532	0.312	2.97E-44	3
NKG7.3	1.11E-44	0.562065745	0.657	0.411	2.55E-40	3
CMC1.3	2.37E-41	0.787070434	0.469	0.272	5.45E-37	3
TMSB10.2	8.74E-37	-0.512954894	0.98	0.959	2.01E-32	3
DNAJA1	2.46E-35	1.091005224	0.479	0.321	5.65E-31	3
FTL.3	8.80E-35	-1.303838142	0.982	0.934	2.02E-30	3
FOS.3	1.89E-30	-0.808708516	0.58	0.689	4.35E-26	3
CCL4L2.2	1.24E-28	0.53191586	0.337	0.182	2.84E-24	3
SPP1.1	3.08E-28	-0.774614308	0.378	0.205	7.08E-24	3
KLRB1.2	1.07E-26	0.662113926	0.353	0.202	2.47E-22	3
CEBPB.2	4.85E-24	-1.066546607	0.195	0.338	1.11E-19	3
MT1E	1.33E-21	-0.795877267	0.303	0.164	3.06E-17	3
IFITM3.3	3.53E-20	-1.771890497	0.288	0.385	8.11E-16	3
HBA2.2	3.93E-20	3.95903653	0.372	0.242	9.04E-16	3
IGFBP3.3	8.69E-20	-2.154885735	0.132	0.252	2.00E-15	3
S100A9.3	1.42E-18	-2.346941953	0.139	0.259	3.25E-14	3
GNLY.3	2.71E-18	-1.728717584	0.138	0.261	6.23E-14	3
GSTP1.2	4.20E-16	-0.953404697	0.452	0.492	9.65E-12	3
IGFBP7.3	6.90E-16	-2.617407331	0.286	0.385	1.59E-11	3
RP11-347P5.1.3	1.41E-15	-0.962680494	0.303	0.38	3.24E-11	3
ANXA1.3	5.01E-15	-0.626697349	0.378	0.478	1.15E-10	3
APOE.2	6.24E-15	-1.554956496	0.674	0.421	1.43E-10	3
YBX1.2	2.61E-14	-0.653717624	0.75	0.665	6.00E-10	3

HLA-DRB1.3	1.04E-13	-0.601139029	0.841	0.573	2.40E-09	3
RAC1.3	2.07E-13	-0.934136773	0.431	0.449	4.75E-09	3
MT2A.3	3.95E-13	-1.897145775	0.697	0.657	9.07E-09	3
ITM2B.3	3.40E-12	-0.633065293	0.898	0.771	7.82E-08	3
IGKC	1.03E-11	-2.655202647	0.213	0.302	2.38E-07	3
RGCC.2	1.20E-11	-1.150093441	0.234	0.312	2.77E-07	3
CD63.3	1.42E-11	-0.922220884	0.535	0.519	3.26E-07	3
VIM.3	1.83E-11	-0.735741328	0.872	0.764	4.21E-07	3
HBB	3.63E-11	3.753898616	0.492	0.385	8.34E-07	3
KLF6.2	4.04E-11	-0.621316678	0.654	0.649	9.28E-07	3
HLA-DPB1.2	5.93E-11	-0.782273455	0.865	0.616	1.36E-06	3
TNFAIP3.3	6.30E-11	-0.646214811	0.384	0.452	1.45E-06	3
NNMT.3	6.89E-11	-1.329863125	0.442	0.289	1.58E-06	3
CEBPD.3	3.04E-10	-0.914274911	0.223	0.283	6.98E-06	3
POMP.2	6.12E-10	-0.755272408	0.407	0.451	1.41E-05	3
CRYAB.3	1.03E-09	-1.129506844	0.378	0.251	2.37E-05	3
DSTN.2	2.27E-09	-1.063751676	0.246	0.312	5.21E-05	3
LAPTM4A.3	1.01E-08	-0.799954143	0.306	0.355	0.000232303	3
RGS1.3	1.29E-08	-0.724495487	0.812	0.689	0.000295466	3
HLA-DPA1.3	3.11E-07	-0.908182857	0.803	0.554	0.00715078	3
NR4A2.1	6.53E-07	-0.515687151	0.215	0.281	0.015015818	3
MT1X.2	7.48E-07	-2.113196141	0.239	0.286	0.017194521	3
MT1X.3	0	3.821195713	0.797	0.25	0	4
MT2A.4	0	3.291209383	0.97	0.639	0	4
NNMT.4	0	3.078621801	0.814	0.266	0	4
ANGPTL4	3.71E-296	2.955186376	0.574	0.141	8.52E-292	4
B2M.3	2.07E-288	-1.426731961	0.922	0.992	4.77E-284	4
NDUFA4L2.4	2.80E-283	2.311173651	0.747	0.259	6.44E-279	4
TMSB4X.2	5.28E-271	-1.598393928	0.872	0.983	1.21E-266	4
HILPDA	7.77E-195	2.743074306	0.43	0.108	1.79E-190	4
CD24	9.64E-191	2.183059798	0.521	0.156	2.21E-186	4
HLA-B.3	3.40E-180	-1.225553014	0.616	0.933	7.81E-176	4
GAPDH.3	9.13E-171	1.244087126	0.903	0.864	2.10E-166	4
HLA-E.2	3.31E-167	-1.497921368	0.243	0.824	7.60E-163	4
HLA-C.2	1.84E-164	-1.266320592	0.499	0.907	4.23E-160	4
RPLP0.2	6.43E-163	1.403130941	0.854	0.842	1.48E-158	4
RPL36.2	3.33E-161	1.10072077	0.907	0.913	7.66E-157	4
TMSB10.3	4.37E-157	0.916647488	0.979	0.959	1.00E-152	4
FTH1.4	3.15E-154	0.819712199	0.98	0.961	7.23E-150	4
EGLN3	6.35E-149	2.130802094	0.282	0.057	1.46E-144	4
ARHGDIB.1	2.88E-147	-1.221347975	0.131	0.707	6.62E-143	4
PTPRC.4	7.40E-145	-1.52428557	0.104	0.649	1.70E-140	4
BTG1.3	5.76E-138	-1.468526497	0.463	0.867	1.32E-133	4
CORO1A.3	2.57E-136	-1.507490119	0.081	0.596	5.91E-132	4
ZFP36L2.3	3.02E-131	-1.641425383	0.232	0.728	6.94E-127	4
HLA-A.2	2.22E-130	-1.09318445	0.592	0.913	5.11E-126	4
RPS8.2	3.95E-130	0.981026093	0.92	0.918	9.08E-126	4
MYL12A.1	1.04E-127	-1.113311938	0.174	0.723	2.40E-123	4
ARPC2.1	5.26E-122	-1.054709094	0.138	0.67	1.21E-117	4
LAPTM5.2	7.80E-121	-1.371831396	0.106	0.597	1.79E-116	4
GMFG.1	1.02E-119	-1.109142898	0.092	0.585	2.35E-115	4
RPL41.1	6.06E-119	0.634231555	0.988	0.975	1.39E-114	4
ENO1.3	1.06E-118	1.717677109	0.717	0.585	2.44E-114	4
RPL10.1	1.31E-117	0.644411735	0.986	0.974	3.01E-113	4
TMA7.2	5.14E-117	-1.01651069	0.189	0.728	1.18E-112	4
CD53.3	1.66E-113	-1.230948284	0.05	0.498	3.81E-109	4
HCST.4	7.55E-113	-1.170730638	0.097	0.573	1.73E-108	4
SRGN.2	1.12E-112	-1.216287526	0.344	0.82	2.57E-108	4

FYB.3	3.28E-112	-1.415302359	0.045	0.486	7.54E-108	4
HLA-DPB1.3	6.00E-111	-1.790972366	0.175	0.661	1.38E-106	4
CELF2.3	6.39E-110	-1.375517261	0.029	0.451	1.47E-105	4
RPS5	2.20E-109	1.197791301	0.802	0.844	5.05E-105	4
CFL1	6.39E-108	-0.999649395	0.264	0.777	1.47E-103	4
EVL.4	1.92E-107	-1.167002139	0.074	0.522	4.41E-103	4
CYBA.1	7.44E-107	-0.999134587	0.209	0.717	1.71E-102	4
CRYAB.4	9.10E-107	1.648213735	0.519	0.243	2.09E-102	4
RGS1.4	1.38E-106	-1.572778751	0.298	0.722	3.17E-102	4
S100A4.3	7.79E-106	-1.292681152	0.259	0.727	1.79E-101	4
CXCR4.3	9.16E-105	-1.356477061	0.388	0.8	2.11E-100	4
CD44.3	5.70E-104	-1.133703564	0.075	0.518	1.31E-99	4
SRSF7.2	1.84E-103	-1.093664616	0.111	0.572	4.22E-99	4
CD74.3	2.30E-103	-1.76441899	0.372	0.821	5.28E-99	4
CD37.3	5.60E-103	-1.099719989	0.06	0.489	1.29E-98	4
STK4.3	8.05E-102	-1.208591286	0.069	0.498	1.85E-97	4
RPL13.1	5.19E-101	0.60193365	0.956	0.965	1.19E-96	4
LDHA.4	5.88E-101	1.546799608	0.696	0.571	1.35E-96	4
RBM39.1	2.55E-100	-0.806262599	0.112	0.573	5.85E-96	4
GPSM3.2	1.24E-99	-1.235874255	0.023	0.414	2.84E-95	4
LSP1.3	5.83E-99	-1.14878282	0.049	0.459	1.34E-94	4
HLA-DPA1.4	8.84E-97	-1.768987114	0.156	0.597	2.03E-92	4
RARRES2	1.33E-95	2.048938326	0.31	0.099	3.06E-91	4
PNISR.3	1.87E-94	-0.74002865	0.098	0.537	4.31E-90	4
PRPF38B.4	2.66E-94	-0.975417389	0.056	0.459	6.12E-90	4
HLA-F.4	5.04E-94	-0.944766419	0.058	0.462	1.16E-89	4
DDX5.3	2.68E-92	-0.796701037	0.368	0.839	6.15E-88	4
ITGB2.2	4.29E-92	-1.186607531	0.049	0.438	9.85E-88	4
MGST1	7.06E-92	1.886677486	0.308	0.099	1.62E-87	4
CD2.3	1.30E-91	-1.445576593	0.081	0.481	2.99E-87	4
ARGLU1.2	2.00E-91	-0.881837771	0.058	0.457	4.59E-87	4
RHOA.1	8.77E-91	-0.866102093	0.106	0.534	2.01E-86	4
CCL5.3	9.66E-91	-1.871100701	0.206	0.584	2.22E-86	4
AKAP13.4	1.31E-90	-0.776862166	0.06	0.456	3.01E-86	4
ELF1.3	1.83E-90	-0.862776224	0.064	0.464	4.20E-86	4
DDX24.2	1.89E-90	-0.802729091	0.074	0.481	4.35E-86	4
CALM1.1	4.06E-90	-0.714435539	0.16	0.624	9.32E-86	4
CYTIP.3	6.74E-90	-1.064522917	0.091	0.5	1.55E-85	4
JAK1.1	1.49E-89	-0.885902163	0.042	0.418	3.43E-85	4
SH3BGRL3.3	5.73E-89	-0.772915771	0.228	0.711	1.32E-84	4
LCP1.4	5.75E-89	-1.030212904	0.047	0.427	1.32E-84	4
ARL6IP5.2	2.22E-88	-0.632729327	0.076	0.482	5.10E-84	4
SLA.4	5.44E-88	-1.038077806	0.045	0.422	1.25E-83	4
VAMP2.3	1.07E-87	-0.712502595	0.082	0.49	2.46E-83	4
VIM.4	1.13E-87	1.281035684	0.797	0.769	2.60E-83	4
CD52.3	1.19E-87	-1.258325558	0.161	0.573	2.73E-83	4
RPL18A.1	1.26E-87	0.726940146	0.906	0.931	2.91E-83	4
RPS19.1	4.74E-87	0.624344507	0.957	0.951	1.09E-82	4
TNFAIP3.4	5.87E-87	-1.128618043	0.079	0.471	1.35E-82	4
KMT2E.3	3.99E-86	-0.748160067	0.05	0.427	9.17E-82	4
ATP1B1	5.22E-86	2.248849533	0.332	0.125	1.20E-81	4
RNF213.3	8.41E-85	-0.923236333	0.043	0.409	1.93E-80	4
YWHAZ.3	1.99E-84	-0.673238233	0.106	0.519	4.57E-80	4
CFLAR.3	2.46E-84	-0.829160002	0.064	0.445	5.65E-80	4
MALAT1.3	2.94E-84	-0.760119386	0.97	0.983	6.76E-80	4
CD69.4	9.11E-84	-1.53048171	0.158	0.552	2.09E-79	4
GIMAP4.1	9.29E-84	-1.230987356	0.018	0.359	2.13E-79	4
TRAC.4	1.39E-83	-1.530238599	0.082	0.454	3.19E-79	4

YWHAB	1.70E-83	-0.624784747	0.101	0.512	3.91E-79	4
IL2RG.4	1.79E-83	-1.20982194	0.038	0.394	4.11E-79	4
N4BP2L2.3	5.94E-83	-0.579619195	0.128	0.555	1.36E-78	4
GZMA.3	6.27E-83	-1.647646739	0.078	0.441	1.44E-78	4
FNBP1.3	1.03E-82	-0.95292507	0.035	0.389	2.38E-78	4
CD3E.3	1.13E-82	-1.191942847	0.055	0.419	2.59E-78	4
HSP90AA1.2	1.18E-82	-1.040063342	0.228	0.681	2.71E-78	4
AAK1.4	2.93E-82	-0.907489367	0.038	0.392	6.73E-78	4
CD48.4	3.51E-82	-0.964270469	0.041	0.396	8.06E-78	4
ATP5E.2	4.53E-82	-0.789879329	0.409	0.842	1.04E-77	4
SOD2.3	6.69E-82	2.095715881	0.493	0.281	1.54E-77	4
RAC2.4	7.39E-82	-1.045897724	0.039	0.394	1.70E-77	4
HMGB2.1	2.53E-81	-0.834245111	0.103	0.5	5.81E-77	4
HSPA8.1	6.04E-81	-0.687725854	0.148	0.58	1.39E-76	4
FXYD5.2	6.66E-81	-0.683996707	0.101	0.505	1.53E-76	4
WIPF1.4	1.64E-80	-1.058174717	0.026	0.363	3.77E-76	4
DNAJB1.3	1.70E-80	-1.334799378	0.074	0.442	3.91E-76	4
TPM3	2.56E-80	-0.675279075	0.088	0.476	5.89E-76	4
KIAA1551.3	2.94E-80	-1.114367453	0.023	0.355	6.76E-76	4
ARPC1B	5.12E-80	-0.627904214	0.129	0.55	1.18E-75	4
PSMB9.1	1.23E-79	-0.651360273	0.106	0.508	2.82E-75	4
DDX17.1	3.37E-79	-0.744323044	0.058	0.421	7.74E-75	4
CAP1	4.86E-79	-0.711178543	0.066	0.434	1.12E-74	4
RSRP1.3	1.18E-78	-0.721947393	0.068	0.435	2.71E-74	4
RPL3.1	1.21E-78	0.680061974	0.926	0.932	2.78E-74	4
STK17A.4	1.35E-78	-0.998288288	0.042	0.387	3.11E-74	4
RPL7.2	2.20E-78	0.753030343	0.9	0.921	5.05E-74	4
DUSP2.3	3.90E-78	-1.426847583	0.089	0.453	8.96E-74	4
ATP5L	6.62E-78	-0.578401764	0.226	0.703	1.52E-73	4
SON.3	1.05E-77	-0.513760635	0.158	0.596	2.40E-73	4
NKTR.3	1.88E-77	-1.004441577	0.032	0.367	4.31E-73	4
RPL21.1	3.70E-77	0.586002368	0.947	0.952	8.50E-73	4
SP100.3	7.44E-77	-0.732323336	0.062	0.42	1.71E-72	4
GNAS.1	9.70E-77	-0.954464397	0.032	0.365	2.23E-72	4
RBM25.2	1.68E-76	-0.882666847	0.042	0.38	3.86E-72	4
HLA-DRB1.4	1.94E-76	-1.659648659	0.218	0.614	4.46E-72	4
SRSF2.3	7.39E-76	-0.61522357	0.076	0.445	1.70E-71	4
MT1E.1	8.39E-76	2.367834729	0.366	0.161	1.93E-71	4
UCP2.3	9.55E-76	-1.009503454	0.035	0.366	2.19E-71	4
RPS2.2	1.05E-75	0.60152367	0.949	0.944	2.41E-71	4
ITGB1	1.82E-75	-0.888427965	0.107	0.487	4.19E-71	4
PDK4.1	8.33E-75	1.998742151	0.401	0.191	1.91E-70	4
CD3D.4	1.08E-74	-1.119860214	0.126	0.497	2.49E-70	4
CAPZB.1	2.75E-74	-0.599574823	0.101	0.484	6.32E-70	4
SEPT7.1	5.16E-74	-0.562516889	0.134	0.537	1.19E-69	4
ARPC3.1	8.43E-74	-0.594106476	0.191	0.631	1.94E-69	4
LRRFIP1	1.06E-73	-0.626076119	0.074	0.434	2.44E-69	4
PFN1.1	1.80E-73	-0.686187839	0.323	0.788	4.13E-69	4
HLA-DRA.4	2.92E-73	-1.727436919	0.289	0.697	6.71E-69	4
TERF2IP.2	4.07E-73	-0.772740753	0.049	0.385	9.35E-69	4
PSME1.1	5.90E-73	-0.535710623	0.179	0.607	1.36E-68	4
PIK3IP1.4	7.30E-73	-0.945113691	0.047	0.378	1.68E-68	4
RPL24	8.18E-73	0.949964086	0.777	0.857	1.88E-68	4
HERPUD1.2	1.69E-72	-0.938860034	0.134	0.515	3.89E-68	4
NKG7.4	3.06E-72	-1.837779203	0.112	0.447	7.04E-68	4
LITAF.1	1.34E-71	-0.876977157	0.054	0.387	3.09E-67	4
PRRC2C.2	5.02E-71	-0.555819368	0.092	0.455	1.15E-66	4
IDS.2	7.60E-71	-0.821957038	0.06	0.393	1.75E-66	4

TRBC2.3	2.00E-70	-1.219069297	0.067	0.397	4.59E-66	4
MBNL1.2	2.59E-70	-0.711744631	0.048	0.375	5.95E-66	4
ISG20.3	2.86E-70	-0.861519201	0.053	0.381	6.57E-66	4
CTSC.2	4.28E-70	-0.843233808	0.044	0.366	9.83E-66	4
SMAP2	2.69E-69	-0.911198227	0.018	0.316	6.17E-65	4
SH3KBP1.2	3.31E-69	-0.875695559	0.024	0.326	7.60E-65	4
SRSF11.1	3.81E-69	-0.507262417	0.062	0.396	8.75E-65	4
ID2.3	3.89E-69	-0.808317337	0.187	0.588	8.95E-65	4
C9orf16	1.15E-68	-0.700291219	0.048	0.369	2.65E-64	4
GIMAP7.3	2.98E-68	-1.237091896	0.013	0.302	6.84E-64	4
ARPC5.1	3.36E-68	-0.594127861	0.097	0.457	7.72E-64	4
CST7.3	6.79E-68	-1.267008944	0.081	0.409	1.56E-63	4
MSN.1	7.12E-68	-0.68038567	0.06	0.386	1.64E-63	4
MT-ND5.2	8.00E-68	-0.580693678	0.209	0.636	1.84E-63	4
ATRX.2	2.21E-67	-0.565259366	0.072	0.407	5.08E-63	4
ACTR2	6.40E-67	-0.668660348	0.061	0.387	1.47E-62	4
PCSK7.4	6.63E-67	-0.850622675	0.035	0.338	1.52E-62	4
CCND3.3	8.00E-67	-0.638633246	0.056	0.379	1.84E-62	4
ACAP1.4	9.91E-67	-0.890411899	0.043	0.351	2.28E-62	4
SMCHD1.1	1.10E-66	-0.857044841	0.039	0.346	2.54E-62	4
BCLAF1.1	1.44E-66	-0.726602875	0.044	0.354	3.30E-62	4
LIMD2.3	2.41E-66	-0.812326097	0.03	0.328	5.54E-62	4
CAV1	3.34E-66	1.557584532	0.315	0.126	7.67E-62	4
DOCK8.3	3.50E-66	-0.851937912	0.02	0.309	8.04E-62	4
RPS6	4.32E-66	0.618142485	0.936	0.944	9.92E-62	4
REL.1	5.48E-66	-0.880149788	0.037	0.34	1.26E-61	4
EVI2B	1.00E-65	-0.793450285	0.045	0.354	2.30E-61	4
ROCK1	3.51E-65	-0.667842796	0.043	0.349	8.07E-61	4
SRRM2	4.31E-65	-0.532900551	0.049	0.36	9.90E-61	4
ETS1.4	4.38E-65	-0.890782789	0.049	0.357	1.01E-60	4
RP11-347P5.1.4	7.93E-65	-0.991428921	0.073	0.395	1.82E-60	4
RPS18.1	9.40E-65	0.514767692	0.962	0.959	2.16E-60	4
BTG2.2	2.65E-64	-0.820514335	0.061	0.376	6.09E-60	4
RGS2.3	2.93E-64	-1.319028101	0.051	0.353	6.72E-60	4
NCL.2	7.30E-64	-0.509063628	0.104	0.452	1.68E-59	4
LCK.4	1.07E-63	-1.055740298	0.023	0.304	2.46E-59	4
PIK3R1.3	1.69E-63	-0.827526008	0.033	0.326	3.88E-59	4
CKLF.1	2.07E-63	-0.621311011	0.064	0.382	4.77E-59	4
HCLS1	2.59E-63	-0.71391686	0.042	0.34	5.96E-59	4
IGFBP3.4	6.51E-63	1.493033785	0.439	0.232	1.50E-58	4
EMB.3	6.68E-63	-0.944632648	0.013	0.286	1.53E-58	4
UBC.2	8.40E-63	1.022447345	0.771	0.844	1.93E-58	4
GCC2.3	2.02E-62	-0.647133793	0.066	0.378	4.65E-58	4
FKBP5.1	2.06E-62	-0.722338459	0.087	0.415	4.74E-58	4
SEPT6.3	3.75E-62	-0.935302715	0.02	0.294	8.62E-58	4
PPP1R2.2	9.05E-62	-0.641465222	0.056	0.361	2.08E-57	4
CRIP1.2	1.46E-61	-1.115010772	0.03	0.31	3.36E-57	4
ARID4B.3	1.68E-61	-0.544159014	0.055	0.358	3.86E-57	4
RBPJ.3	2.37E-61	-0.859105342	0.047	0.339	5.45E-57	4
GLIPR1.1	1.14E-60	-0.781126666	0.033	0.316	2.62E-56	4
UBB.2	2.59E-60	-0.568353547	0.303	0.746	5.94E-56	4
CARD16.1	2.68E-60	-0.767463779	0.044	0.335	6.16E-56	4
MYO1F.1	3.11E-60	-0.805552751	0.019	0.288	7.16E-56	4
APOBEC3G.3	4.67E-60	-1.141543656	0.02	0.287	1.07E-55	4
GNAI2.2	5.85E-60	-0.787400759	0.013	0.276	1.34E-55	4
TRBC1.3	6.32E-60	-1.323198074	0.073	0.371	1.45E-55	4
STK17B.1	9.16E-60	-0.580820618	0.093	0.42	2.11E-55	4
RPS28.1	1.00E-59	0.510847997	0.928	0.948	2.31E-55	4

CD27.3	1.17E-59	-1.473383858	0.02	0.283	2.68E-55	4
IQGAP1.2	1.38E-59	-0.559815141	0.058	0.356	3.16E-55	4
CCL4.4	1.95E-59	-1.733755068	0.143	0.459	4.48E-55	4
NR4A2.2	2.55E-59	-1.034842437	0.024	0.293	5.87E-55	4
YPEL5.4	2.99E-59	-0.632119228	0.062	0.362	6.86E-55	4
ANKRD44.2	3.51E-59	-0.864839651	0.014	0.274	8.07E-55	4
GABPB1.3	3.84E-59	-0.746834868	0.026	0.297	8.83E-55	4
ARHGEF1.4	5.17E-59	-0.885660979	0.017	0.278	1.19E-54	4
HLA-DQB1.3	7.21E-59	-1.165048988	0.101	0.411	1.66E-54	4
TMEM50A	9.25E-59	-0.542455397	0.058	0.355	2.13E-54	4
COTL1.1	1.07E-58	-0.739191119	0.121	0.454	2.47E-54	4
CD3G.3	5.20E-58	-0.969820501	0.036	0.31	1.20E-53	4
CD84.2	7.59E-58	-0.955644662	0.006	0.254	1.74E-53	4
RPS4X.2	1.30E-57	0.56081237	0.919	0.939	2.98E-53	4
ITM2A.4	1.49E-57	-1.108267295	0.049	0.327	3.43E-53	4
TRAF3IP3.4	1.51E-57	-0.949007283	0.016	0.271	3.47E-53	4
RCSD1.2	1.76E-57	-0.796501896	0.012	0.264	4.04E-53	4
MYCBP2	2.19E-57	-0.782829047	0.018	0.273	5.04E-53	4
ABRACL.2	2.25E-57	-0.712641566	0.032	0.302	5.16E-53	4
LTB.3	3.34E-57	-1.150090461	0.043	0.318	7.68E-53	4
EPC1.3	9.14E-57	-0.67207911	0.025	0.286	2.10E-52	4
PPP2R5C.3	1.60E-56	-0.600779299	0.094	0.409	3.67E-52	4
RPL34.2	1.06E-55	0.546106248	0.946	0.953	2.44E-51	4
RPL26	1.32E-55	0.613287985	0.896	0.938	3.03E-51	4
H2AFY.2	1.78E-55	-0.748450372	0.035	0.299	4.10E-51	4
SYNE2.3	2.46E-55	-0.67230391	0.064	0.349	5.66E-51	4
AKNA.2	3.01E-55	-0.836257146	0.023	0.276	6.91E-51	4
CD7.2	5.40E-55	-1.049607077	0.048	0.316	1.24E-50	4
FYN.2	9.37E-55	-0.91588005	0.026	0.281	2.15E-50	4
EIF3A	1.35E-54	-0.516877027	0.044	0.314	3.11E-50	4
RGCC.3	1.71E-54	-1.371309259	0.055	0.323	3.93E-50	4
C10orf10	2.74E-54	1.645475494	0.265	0.106	6.29E-50	4
PNN.2	2.81E-54	-0.52990433	0.049	0.32	6.46E-50	4
ICAM3.4	2.94E-54	-0.739423367	0.031	0.288	6.75E-50	4
CCNH.2	3.45E-54	-0.655310608	0.037	0.3	7.92E-50	4
IKZF1.4	5.03E-54	-0.778375135	0.016	0.258	1.16E-49	4
SAMSN1.2	6.32E-54	-0.673117585	0.06	0.339	1.45E-49	4
WNK1.1	6.48E-54	-0.763206582	0.019	0.264	1.49E-49	4
PAK2.1	7.01E-54	-0.607661888	0.03	0.284	1.61E-49	4
ACTB	1.04E-53	-0.618119034	0.733	0.941	2.39E-49	4
BIN2.3	1.14E-53	-0.737767276	0.019	0.264	2.63E-49	4
RGS10.1	1.23E-53	-0.709147757	0.044	0.311	2.83E-49	4
EVI2A.1	1.52E-53	-0.58282939	0.036	0.295	3.50E-49	4
KIF5B	2.06E-53	-0.501475895	0.038	0.3	4.74E-49	4
MACF1.2	2.35E-53	-0.660471419	0.024	0.272	5.41E-49	4
MT-CYB.2	3.40E-53	-0.587603407	0.495	0.824	7.80E-49	4
ALOX5AP.1	3.76E-53	-0.890358626	0.067	0.345	8.64E-49	4
YWHAH.3	4.00E-53	-0.94326735	0.039	0.299	9.20E-49	4
AES.1	7.63E-53	-0.765227077	0.021	0.266	1.75E-48	4
RHOG	8.60E-53	-0.624723107	0.029	0.279	1.98E-48	4
MYL6.2	1.07E-52	-0.587781711	0.396	0.822	2.46E-48	4
TRIM22.1	1.31E-52	-0.668107316	0.02	0.263	3.01E-48	4
HLA-DQA1.2	1.40E-52	-1.282619332	0.084	0.36	3.23E-48	4
RNF19A.3	1.45E-52	-0.638629242	0.036	0.292	3.34E-48	4
CLEC2D.3	3.56E-52	-0.806107047	0.055	0.322	8.17E-48	4
PRKCH.3	3.94E-52	-0.757445696	0.024	0.268	9.05E-48	4
2-Sep	5.88E-52	-0.616706621	0.029	0.277	1.35E-47	4
ARL6IP1.1	1.40E-51	-0.571250746	0.063	0.337	3.21E-47	4

JMJD1C	1.88E-51	-0.578918047	0.027	0.273	4.33E-47	4
C10orf54.2	3.29E-51	-0.673318322	0.042	0.297	7.55E-47	4
IL10RA.2	5.65E-51	-0.583414798	0.029	0.273	1.30E-46	4
RPS29.2	8.80E-51	-0.563931882	0.802	0.929	2.02E-46	4
TGOLN2	1.29E-50	-0.579915224	0.031	0.276	2.97E-46	4
ARHGAP15.1	1.59E-50	-0.666719742	0.018	0.252	3.65E-46	4
ADGRE5.3	2.26E-50	-0.615777084	0.054	0.315	5.20E-46	4
IFITM2.2	2.42E-50	-0.618134887	0.16	0.496	5.56E-46	4
TUBA4A.3	3.01E-50	-0.723639941	0.038	0.287	6.92E-46	4
BTN3A2.3	3.85E-50	-0.534480345	0.032	0.278	8.84E-46	4
C9orf142.2	8.99E-50	-0.537961801	0.032	0.275	2.07E-45	4
ATP1B3.2	1.14E-49	-0.601843277	0.037	0.283	2.63E-45	4
ATM.2	1.22E-49	-0.568893627	0.026	0.263	2.81E-45	4
RPL36A.2	2.48E-49	0.898437413	0.73	0.806	5.69E-45	4
BNIP3	6.91E-49	1.494317718	0.282	0.124	1.59E-44	4
CMC1.4	1.71E-48	-1.163747972	0.053	0.3	3.94E-44	4
OSTF1.1	2.40E-48	-0.565953565	0.025	0.257	5.51E-44	4
SPP1.2	2.56E-48	1.108074479	0.379	0.206	5.88E-44	4
GZMK.4	4.19E-48	-1.468292568	0.121	0.381	9.62E-44	4
CTSS.3	5.56E-48	-0.770936154	0.099	0.38	1.28E-43	4
BUB3.3	1.00E-47	-0.53516081	0.042	0.286	2.31E-43	4
FKBP1A.3	1.24E-47	-0.580871449	0.128	0.431	2.85E-43	4
PRDM1.2	2.91E-47	-0.613077204	0.055	0.307	6.70E-43	4
ODF2L.1	2.95E-47	-0.726021447	0.037	0.273	6.78E-43	4
CREM.2	3.42E-47	-0.783896206	0.05	0.295	7.86E-43	4
SYTL3.3	7.51E-47	-0.633801011	0.048	0.292	1.73E-42	4
PCM1	8.24E-47	-0.524158351	0.033	0.267	1.89E-42	4
USP15	9.10E-47	-0.528603434	0.039	0.277	2.09E-42	4
TXNIP.3	9.22E-47	-0.739091161	0.467	0.782	2.12E-42	4
ANXA6.2	1.07E-46	-0.61003373	0.032	0.264	2.45E-42	4
ITSN2.1	3.73E-46	-0.565212294	0.027	0.253	8.58E-42	4
CD247.3	3.81E-46	-0.667280452	0.029	0.254	8.76E-42	4
PPP1R18.3	4.41E-46	-0.549691626	0.029	0.253	1.01E-41	4
MIF.4	1.42E-45	1.362828512	0.564	0.502	3.27E-41	4
RPL31.1	2.42E-45	0.576437013	0.863	0.91	5.56E-41	4
HLA-DMA.3	4.45E-45	-0.818864571	0.06	0.302	1.02E-40	4
LIMS1.2	9.22E-45	-0.547875518	0.036	0.262	2.12E-40	4
AC090498.1.2	5.24E-44	-0.533723549	0.199	0.52	1.20E-39	4
DUSP4.3	5.00E-43	-0.972636764	0.036	0.254	1.15E-38	4
CD96.4	8.00E-43	-0.566246621	0.039	0.262	1.84E-38	4
CLU.1	3.42E-42	1.599165368	0.34	0.189	7.86E-38	4
TPI1.3	1.21E-41	1.324482511	0.58	0.564	2.79E-37	4
IL7R.2	6.21E-41	-1.347657419	0.089	0.317	1.43E-36	4
RPL18	1.40E-40	0.670367511	0.772	0.876	3.21E-36	4
C1QA.4	8.16E-39	-1.450889206	0.055	0.263	1.88E-34	4
RPL35A.1	2.45E-38	0.509862381	0.87	0.92	5.63E-34	4
RPL38	6.02E-38	0.64426067	0.79	0.872	1.38E-33	4
TYROBP.4	3.81E-37	-1.008783066	0.162	0.402	8.75E-33	4
SAT1.3	5.57E-37	-0.705344944	0.329	0.651	1.28E-32	4
EGR1	1.39E-36	1.859268653	0.264	0.134	3.19E-32	4
RPL5.1	3.88E-36	0.687044735	0.754	0.859	8.92E-32	4
HLA-DRB5.4	3.99E-36	-0.892441678	0.141	0.388	9.18E-32	4
RPL8	5.03E-35	0.58328741	0.791	0.888	1.16E-30	4
NUPR1	4.78E-34	1.545303771	0.252	0.127	1.10E-29	4
RPS16.1	6.41E-34	0.549106999	0.811	0.896	1.47E-29	4
RPS13	2.46E-33	0.570182293	0.8	0.883	5.66E-29	4
APOE.3	6.99E-33	-1.269027329	0.195	0.453	1.61E-28	4
RPL6	2.22E-32	0.572302367	0.791	0.888	5.10E-28	4

PLIN2.3	1.85E-31	1.478484703	0.428	0.33	4.25E-27	4
RPS17	7.21E-31	0.522345117	0.819	0.903	1.66E-26	4
RPL9	1.49E-30	0.535585614	0.817	0.907	3.42E-26	4
ZFAS1.3	6.51E-30	1.538105543	0.498	0.483	1.50E-25	4
DDIT4.2	2.21E-29	1.44369339	0.527	0.517	5.09E-25	4
GPX1.4	4.94E-29	-0.686241137	0.208	0.443	1.13E-24	4
CYB5A.1	9.77E-28	1.203818468	0.34	0.222	2.25E-23	4
SERPINA1.3	1.08E-27	1.657479455	0.374	0.272	2.47E-23	4
FCER1G.4	1.82E-27	-0.729325144	0.119	0.315	4.18E-23	4
AIF1.4	5.34E-25	-0.683471897	0.097	0.27	1.23E-20	4
DUSP1.2	1.26E-23	0.554245454	0.758	0.795	2.89E-19	4
S100A10.2	2.69E-23	1.007325205	0.574	0.612	6.18E-19	4
CST3.3	2.58E-22	-0.670095619	0.203	0.397	5.92E-18	4
RPL22.2	2.35E-20	0.640991266	0.678	0.819	5.40E-16	4
NACA.1	1.10E-18	0.614359594	0.662	0.812	2.53E-14	4
ALDOA.3	1.24E-15	0.876709815	0.549	0.643	2.85E-11	4
GNLY.4	7.11E-15	-1.229411256	0.132	0.26	1.63E-10	4
PGAM1.2	2.91E-14	0.5933075	0.181	0.372	6.69E-10	4
RNASET2.4	3.10E-14	1.318589219	0.384	0.359	7.12E-10	4
IFITM3.4	4.89E-14	0.755998457	0.427	0.375	1.12E-09	4
CCT8.1	3.16E-13	0.511175232	0.125	0.27	7.26E-09	4
SSBP1	4.42E-13	0.561241172	0.144	0.302	1.02E-08	4
MZT2A.1	1.15E-11	0.515942065	0.137	0.279	2.65E-07	4
RSL1D1	1.18E-11	0.537338299	0.148	0.298	2.72E-07	4
MZT2B	2.23E-11	0.52642727	0.193	0.372	5.12E-07	4
TMED2.1	2.62E-11	0.59472549	0.177	0.345	6.02E-07	4
TOMM20	3.23E-11	0.563741561	0.162	0.319	7.42E-07	4
ZFP36L1.2	6.03E-11	0.559914485	0.198	0.377	1.39E-06	4
SNRPE.1	1.81E-10	0.627064763	0.165	0.315	4.15E-06	4
PRDX6.1	4.46E-10	0.509951064	0.179	0.335	1.02E-05	4
IGKC.1	4.71E-10	-1.323601244	0.326	0.294	1.08E-05	4
PCNP	6.17E-10	0.533025683	0.159	0.3	1.42E-05	4
EIF2S3	6.22E-10	0.554009563	0.141	0.273	1.43E-05	4
RHOB.4	8.73E-10	0.992835769	0.372	0.347	2.01E-05	4
HSPD1.4	1.31E-09	0.532171761	0.162	0.302	3.01E-05	4
FBL	6.63E-09	0.548817723	0.142	0.265	0.000152336	4
HSPB1.4	4.14E-08	0.85347768	0.426	0.435	0.000950303	4
RSL24D1	8.52E-08	0.648509544	0.209	0.368	0.001957394	4
NPM1.1	2.13E-07	0.756131834	0.536	0.709	0.004905963	4
VMP1.2	6.14E-07	0.816097237	0.19	0.328	0.014117673	4
FTL.4	4.43E-280	2.716738001	0.976	0.935	1.02E-275	5
FTH1.5	8.37E-273	2.078214916	0.988	0.961	1.92E-268	5
LYZ.4	6.04E-227	2.740277147	0.749	0.328	1.39E-222	5
S100A9.4	1.67E-213	4.123186064	0.627	0.23	3.84E-209	5
TYROBP.5	1.41E-174	2.077138113	0.729	0.368	3.23E-170	5
CXCL8	4.19E-164	4.858318773	0.349	0.075	9.63E-160	5
AIF1.5	1.03E-152	2.427169612	0.577	0.241	2.37E-148	5
S100A8	8.19E-143	4.415350094	0.508	0.194	1.88E-138	5
HLA-DRA.5	4.54E-129	1.677443686	0.825	0.664	1.04E-124	5
FCER1G.5	1.12E-123	2.175078499	0.58	0.287	2.57E-119	5
IL32.5	7.29E-111	-1.795783065	0.147	0.602	1.68E-106	5
CD2.4	2.59E-103	-1.977482638	0.053	0.48	5.95E-99	5
RPS27.2	1.71E-100	-0.767274044	0.89	0.98	3.93E-96	5
CCL5.4	8.05E-99	-2.21738475	0.178	0.584	1.85E-94	5
SRSF7.3	8.96E-97	-1.227099487	0.131	0.568	2.06E-92	5
ZFP36L2.4	1.48E-95	-1.399232363	0.285	0.722	3.40E-91	5
SON.4	4.24E-94	-0.82031629	0.146	0.594	9.74E-90	5
CD69.5	1.52E-91	-1.649524046	0.133	0.551	3.50E-87	5

S100A11.4	5.03E-90	1.983003232	0.683	0.585	1.16E-85	5
CD3D.5	3.33E-87	-1.513635758	0.098	0.496	7.66E-83	5
PNISR.4	1.07E-86	-0.881262544	0.121	0.532	2.46E-82	5
EVL.5	3.84E-86	-1.020239562	0.11	0.517	8.82E-82	5
TRAC.5	1.85E-85	-1.706354651	0.07	0.453	4.25E-81	5
CD14	2.45E-84	2.347029205	0.351	0.13	5.62E-80	5
STK4.4	9.98E-84	-0.967586196	0.098	0.493	2.29E-79	5
CD3E.4	2.79E-83	-1.484161741	0.052	0.417	6.41E-79	5
MALAT1.4	1.08E-82	-0.817148463	0.958	0.984	2.48E-78	5
JUN.3	2.46E-79	-1.255163059	0.296	0.714	5.66E-75	5
FCN1	1.37E-78	1.94124442	0.257	0.073	3.15E-74	5
DDX24.3	1.62E-77	-0.864529836	0.102	0.477	3.72E-73	5
RBM39.2	4.08E-76	-0.567401783	0.16	0.568	9.37E-72	5
N4BP2L2.4	2.08E-74	-0.72103351	0.154	0.551	4.78E-70	5
TSC22D3.3	2.26E-74	-0.887369811	0.391	0.803	5.19E-70	5
VAMP2.4	3.52E-74	-0.684142068	0.107	0.485	8.09E-70	5
RPL23A.3	7.01E-74	-0.658554614	0.675	0.945	1.61E-69	5
GZMA.4	2.06E-73	-1.56896364	0.086	0.438	4.73E-69	5
CIRBP	1.11E-72	-0.614023169	0.187	0.6	2.55E-68	5
XIST.3	5.99E-72	-0.902115925	0.074	0.444	1.38E-67	5
DUSP2.4	1.23E-71	-1.504629449	0.099	0.45	2.83E-67	5
RP11-1143G9.4	2.29E-71	2.659677814	0.308	0.112	5.27E-67	5
ITGB1.1	8.47E-71	-1.069735564	0.127	0.483	1.95E-66	5
FUS.4	1.88E-70	-0.727908598	0.123	0.489	4.32E-66	5
RNF213.4	2.06E-70	-1.142067448	0.07	0.405	4.72E-66	5
CST7.4	2.14E-70	-1.482301853	0.068	0.407	4.91E-66	5
TRBC2.4	2.76E-70	-1.596442493	0.066	0.395	6.33E-66	5
ANAPC16.1	5.19E-70	-0.542377331	0.171	0.56	1.19E-65	5
SYNE2.4	2.19E-69	-1.294447095	0.031	0.349	5.02E-65	5
ETS1.5	2.50E-69	-1.180972678	0.034	0.356	5.74E-65	5
PTPRC.5	4.06E-69	-0.751024457	0.232	0.638	9.33E-65	5
ARGLU1.3	5.99E-69	-0.635622902	0.099	0.452	1.38E-64	5
AAK1.5	9.95E-69	-0.915060322	0.06	0.388	2.29E-64	5
PPDPF.2	1.74E-68	-0.607885582	0.263	0.682	4.00E-64	5
RARRES3.4	3.83E-67	-0.899300221	0.088	0.429	8.81E-63	5
MT-ND2.1	5.00E-67	-0.721895243	0.537	0.885	1.15E-62	5
RSRP1.4	2.09E-66	-0.730551628	0.092	0.431	4.79E-62	5
C12orf57.2	5.36E-66	-1.117515832	0.066	0.389	1.23E-61	5
ACAP1.5	1.16E-65	-1.199878408	0.041	0.35	2.67E-61	5
HLA-F.5	1.55E-65	-0.634079607	0.113	0.457	3.56E-61	5
POLR2J3.3	2.48E-65	-0.852278082	0.088	0.412	5.69E-61	5
KLF6.4	5.76E-65	-0.811266024	0.271	0.671	1.32E-60	5
KMT2E.4	9.60E-65	-0.807602556	0.092	0.422	2.21E-60	5
RPL13A.3	1.30E-62	-0.519774216	0.808	0.972	2.98E-58	5
PRRC2C.3	1.62E-62	-0.555305653	0.115	0.452	3.73E-58	5
RPS29.3	1.70E-62	-0.680025308	0.761	0.93	3.90E-58	5
STK17A.5	1.84E-61	-0.863063042	0.069	0.383	4.23E-57	5
ATRX.3	2.58E-61	-0.7217422	0.086	0.404	5.93E-57	5
CD99.3	3.33E-61	-0.591539025	0.145	0.489	7.66E-57	5
ACTB.1	5.56E-61	0.840135833	0.926	0.929	1.28E-56	5
PIK3IP1.5	7.18E-61	-0.876819359	0.065	0.375	1.65E-56	5
LY6E.3	7.87E-61	-0.970892666	0.076	0.382	1.81E-56	5
ID2.4	2.10E-60	-0.800311764	0.216	0.584	4.82E-56	5
SAT1.4	4.59E-60	1.337414722	0.694	0.628	1.05E-55	5
NKG7.5	6.66E-60	-1.807293967	0.137	0.444	1.53E-55	5
JAK1.2	8.49E-60	-0.624825249	0.093	0.413	1.95E-55	5
CXCR4.4	1.77E-59	-0.850161467	0.447	0.794	4.06E-55	5
CD74.4	2.09E-59	1.054500345	0.801	0.794	4.79E-55	5

TNFAIP3.5	6.22E-59	-0.875937403	0.135	0.465	1.43E-54	5
CFLAR.4	1.07E-58	-0.523537012	0.114	0.44	2.46E-54	5
GCC2.4	1.71E-58	-0.70450806	0.074	0.376	3.94E-54	5
RPS26.3	5.38E-58	-0.63343257	0.511	0.891	1.24E-53	5
PPP2R5C.4	7.68E-58	-0.67537839	0.092	0.407	1.77E-53	5
CUTA.1	1.01E-57	-0.550228197	0.115	0.433	2.33E-53	5
NKTR.4	1.81E-57	-0.822059342	0.066	0.362	4.16E-53	5
TERF2IP.3	2.62E-57	-0.636367611	0.077	0.381	6.03E-53	5
AKAP13.5	4.19E-57	-0.506035274	0.123	0.449	9.62E-53	5
CD3G.4	5.91E-57	-1.101233843	0.029	0.308	1.36E-52	5
LCK.5	6.34E-57	-1.090111838	0.028	0.302	1.46E-52	5
LEPROTL1.2	1.23E-56	-0.682125929	0.098	0.406	2.83E-52	5
FNBP1.4	1.68E-56	-0.553159444	0.082	0.384	3.85E-52	5
TRBC1.4	2.03E-56	-1.460214669	0.077	0.369	4.66E-52	5
PRPF38B.5	2.11E-56	-0.571375828	0.131	0.453	4.84E-52	5
MT-CYB.3	2.21E-56	-0.64979407	0.439	0.825	5.08E-52	5
C1QC	3.05E-56	2.12313894	0.334	0.144	7.00E-52	5
IDS.3	3.06E-56	-0.844209929	0.09	0.389	7.03E-52	5
BTG1.4	1.41E-55	-0.817952613	0.56	0.858	3.25E-51	5
ITM2A.5	2.60E-54	-1.210220327	0.05	0.325	5.98E-50	5
AKAP9.2	2.87E-54	-0.728666611	0.072	0.357	6.60E-50	5
IL2RG.5	8.47E-54	-0.709793829	0.092	0.388	1.95E-49	5
CLEC2D.4	1.69E-53	-0.969067709	0.046	0.321	3.89E-49	5
ANKRD12.2	1.79E-53	-0.540327402	0.102	0.405	4.11E-49	5
PEBP1.4	6.41E-53	-0.679155936	0.105	0.406	1.47E-48	5
SPP1.3	1.11E-52	3.444981361	0.382	0.207	2.55E-48	5
ARID4B.4	1.31E-52	-0.644215823	0.073	0.356	3.00E-48	5
GZMK.5	6.38E-52	-1.72954962	0.103	0.38	1.47E-47	5
CD7.3	1.37E-51	-1.28780923	0.053	0.314	3.14E-47	5
GLUL.3	5.10E-51	2.069087556	0.456	0.298	1.17E-46	5
GIMAP7.4	7.21E-51	-1.074155141	0.04	0.298	1.66E-46	5
RP11-347P5.1.5	1.33E-50	-0.631960298	0.097	0.391	3.06E-46	5
HLA-DRB5.5	1.95E-50	1.937857424	0.517	0.365	4.49E-46	5
KIAA1551.4	2.83E-50	-0.668303293	0.074	0.35	6.51E-46	5
CCND3.4	4.56E-50	-0.556603081	0.092	0.375	1.05E-45	5
HLA-DRB1.5	1.27E-49	1.36590595	0.66	0.587	2.92E-45	5
HLA-A.3	1.66E-49	-0.587402697	0.66	0.907	3.82E-45	5
DDX5.4	2.98E-49	-0.535597022	0.455	0.831	6.84E-45	5
OCIAD2.3	5.47E-49	-0.840568982	0.027	0.274	1.26E-44	5
RPS3.2	8.84E-49	-0.515950248	0.69	0.932	2.03E-44	5
LST1.1	1.19E-48	1.950491671	0.394	0.217	2.74E-44	5
PCSK7.5	1.81E-48	-0.754433768	0.072	0.334	4.16E-44	5
FYN.3	2.54E-48	-0.968035204	0.034	0.279	5.83E-44	5
RPL3.2	6.02E-48	-0.528270764	0.686	0.946	1.38E-43	5
MPHOSPH8.1	1.41E-47	-0.568825563	0.06	0.316	3.25E-43	5
RNF19A.4	1.62E-47	-0.982977608	0.045	0.29	3.72E-43	5
GPX1.5	1.72E-47	1.482875493	0.556	0.422	3.96E-43	5
SYTL3.4	1.92E-47	-0.9074498	0.044	0.29	4.42E-43	5
CCL4.5	2.78E-47	-1.517366569	0.167	0.455	6.39E-43	5
CSTA	3.68E-47	1.903421448	0.281	0.123	8.45E-43	5
SF1.3	9.82E-47	-0.542550867	0.068	0.324	2.26E-42	5
TAF7.2	2.92E-46	-0.545876493	0.074	0.334	6.72E-42	5
EZR	4.31E-46	-0.649217268	0.08	0.338	9.91E-42	5
PPP1R2.3	4.88E-46	-0.590765376	0.092	0.357	1.12E-41	5
BTN3A2.4	8.68E-46	-0.610463295	0.034	0.276	2.00E-41	5
MT-ATP6.1	1.36E-45	-0.50821135	0.541	0.877	3.12E-41	5
CD27.4	1.93E-45	-1.281615311	0.045	0.28	4.44E-41	5
PIK3R1.4	2.25E-45	-0.748024195	0.07	0.322	5.16E-41	5

ODF2L.2	3.44E-45	-0.86847674	0.036	0.272	7.90E-41	5
MBNL1.3	1.34E-44	-0.526174129	0.106	0.37	3.08E-40	5
TNRC6B.2	1.71E-44	-0.550772224	0.077	0.331	3.93E-40	5
CST3.4	2.67E-44	1.339732473	0.537	0.377	6.14E-40	5
EPC1.4	2.83E-44	-0.603333988	0.046	0.283	6.51E-40	5
RPL31.2	1.03E-43	-0.505019488	0.614	0.924	2.36E-39	5
FOSB.1	1.07E-43	-0.657930498	0.158	0.44	2.47E-39	5
SEPT6.4	1.42E-43	-0.632948728	0.052	0.29	3.27E-39	5
CD96.5	1.50E-43	-0.832478755	0.033	0.261	3.45E-39	5
CCNH.3	1.60E-43	-0.637859355	0.057	0.297	3.67E-39	5
APOBEC3G.4	1.65E-43	-0.788009546	0.048	0.284	3.79E-39	5
AKNA.3	2.06E-43	-0.855465035	0.044	0.274	4.73E-39	5
CD247.4	2.89E-43	-0.916871194	0.028	0.253	6.64E-39	5
CMC1.5	4.05E-43	-1.293343694	0.06	0.298	9.31E-39	5
YWHAQ	2.21E-42	-0.514704201	0.103	0.363	5.09E-38	5
BUB3.4	3.59E-42	-0.606488724	0.053	0.284	8.24E-38	5
LIMD2.4	6.13E-42	-0.559942658	0.08	0.324	1.41E-37	5
NPC2.3	9.08E-42	1.752350851	0.492	0.373	2.09E-37	5
SRGN.3	5.72E-41	1.286163383	0.748	0.794	1.31E-36	5
C9orf142.3	5.81E-41	-0.612894374	0.049	0.273	1.34E-36	5
PDCD4.2	9.53E-41	-0.569612338	0.078	0.317	2.19E-36	5
MYCBP2.1	1.05E-40	-0.609071957	0.049	0.27	2.41E-36	5
PRKCH.4	1.33E-40	-0.641300773	0.046	0.265	3.05E-36	5
C1QB.2	3.46E-40	2.059411326	0.39	0.226	7.96E-36	5
CRIP1.3	6.57E-40	-0.945204887	0.076	0.306	1.51E-35	5
ATM.3	1.00E-39	-0.681739924	0.045	0.261	2.30E-35	5
RHOC.3	1.14E-39	-0.512268738	0.094	0.336	2.62E-35	5
MNDA	1.42E-39	2.476312206	0.279	0.133	3.27E-35	5
DOCK8.4	3.36E-39	-0.555274739	0.074	0.304	7.73E-35	5
GABPB1.4	5.22E-39	-0.504613357	0.069	0.292	1.20E-34	5
AES.2	8.45E-39	-0.622616053	0.049	0.263	1.94E-34	5
IGFBP7.5	1.21E-38	-2.238191153	0.145	0.392	2.79E-34	5
SEPT9.3	5.81E-38	-0.623414523	0.05	0.261	1.33E-33	5
DUSP4.4	7.10E-38	-1.045894548	0.044	0.252	1.63E-33	5
PRDM1.3	8.08E-38	-0.518605336	0.076	0.304	1.86E-33	5
DNAJB1.4	1.19E-37	-0.563319991	0.156	0.435	2.73E-33	5
IL7R.3	1.82E-37	-1.3844543	0.093	0.315	4.18E-33	5
C1QA.5	2.53E-37	1.975542337	0.399	0.242	5.82E-33	5
BBX.1	2.99E-37	-0.609386498	0.046	0.255	6.88E-33	5
TRAF3IP3.5	3.06E-37	-0.577128986	0.056	0.267	7.02E-33	5
RSF1.1	5.89E-37	-0.572922718	0.057	0.268	1.35E-32	5
IKZF1.5	6.81E-37	-0.501755258	0.046	0.255	1.57E-32	5
MACF1.3	9.90E-37	-0.56734686	0.058	0.269	2.28E-32	5
WHSC1L1.2	2.27E-36	-0.567843486	0.048	0.251	5.22E-32	5
TSTD1.1	4.19E-36	-0.55232705	0.049	0.257	9.63E-32	5
EMB.4	6.27E-36	-0.521685063	0.068	0.281	1.44E-31	5
BIN2.4	6.49E-36	-0.504468042	0.053	0.261	1.49E-31	5
WNK1.2	6.96E-36	-0.621826022	0.056	0.26	1.60E-31	5
DSTN.3	1.09E-35	-0.866992516	0.095	0.319	2.50E-31	5
REL.2	1.58E-35	-0.551164805	0.106	0.334	3.64E-31	5
BIRC3.3	1.63E-35	-0.823418441	0.07	0.283	3.75E-31	5
ARID5B.2	6.64E-35	-0.626526221	0.06	0.266	1.53E-30	5
S100A6.2	8.23E-35	1.091441311	0.683	0.771	1.89E-30	5
CCDC85B.1	1.45E-34	-0.636330961	0.062	0.264	3.32E-30	5
ANXA6.3	2.00E-34	-0.581417551	0.06	0.261	4.59E-30	5
HLA-DPA1.5	4.78E-34	1.347674031	0.611	0.568	1.10E-29	5
LTB.4	1.06E-32	-0.641056889	0.095	0.313	2.44E-28	5
CD68	6.01E-32	1.797951075	0.322	0.189	1.38E-27	5

HLA-DPB1.4	2.68E-31	1.105090961	0.638	0.632	6.15E-27	5
CTSS.4	2.23E-30	1.56310611	0.458	0.357	5.11E-26	5
RGCC.4	9.94E-29	-0.828241726	0.114	0.318	2.29E-24	5
NAMPT.1	3.02E-28	2.29771325	0.345	0.227	6.94E-24	5
IGFBP3.5	7.48E-28	-1.636105353	0.076	0.254	1.72E-23	5
NDUFA4L2.5	2.55E-25	-1.411092425	0.121	0.298	5.86E-21	5
MS4A6A.1	3.53E-25	1.472142213	0.31	0.187	8.12E-21	5
RGS1.5	4.68E-24	-0.54616047	0.473	0.709	1.08E-19	5
C1orf162	6.05E-23	1.589388716	0.316	0.207	1.39E-18	5
HLA-DQA1.3	2.30E-21	1.383175426	0.436	0.338	5.29E-17	5
GNLY.5	2.62E-20	-1.692119997	0.11	0.261	6.02E-16	5
CRYAB.5	2.87E-20	-1.08194921	0.114	0.267	6.60E-16	5
APOC1.3	1.72E-19	2.103980829	0.373	0.278	3.94E-15	5
H3F3A.4	6.54E-19	1.236893935	0.613	0.746	1.50E-14	5
IFITM3.5	1.09E-17	-0.525424702	0.21	0.388	2.52E-13	5
APOE.4	2.41E-16	1.97622848	0.471	0.436	5.54E-12	5
OAZ1.3	5.91E-16	0.926010412	0.605	0.743	1.36E-11	5
HLA-DQB1.4	6.96E-16	1.356826048	0.448	0.389	1.60E-11	5
PSAP.5	1.03E-15	1.345065844	0.476	0.468	2.36E-11	5
NNMT.5	1.78E-14	-0.774018856	0.167	0.307	4.09E-10	5
TIMP1.5	9.60E-14	1.636464301	0.362	0.311	2.21E-09	5
CTSB.4	2.18E-12	1.569274015	0.341	0.3	5.00E-08	5
SDCBP.4	2.29E-12	1.63288974	0.394	0.371	5.27E-08	5
GMFG.2	1.48E-11	0.595884404	0.324	0.569	3.40E-07	5
MAP1LC3B.1	1.74E-10	0.58801859	0.178	0.323	4.00E-06	5
LAMTOR4	4.06E-10	0.604013983	0.244	0.428	9.33E-06	5
ATP6AP2.1	5.36E-10	0.576725039	0.175	0.314	1.23E-05	5
HCLS1.1	1.12E-09	0.581341461	0.187	0.33	2.56E-05	5
RGS10.2	1.55E-09	0.51205978	0.172	0.302	3.56E-05	5
CEBPB.3	1.57E-09	1.612706469	0.355	0.327	3.61E-05	5
LGALS1.2	4.10E-09	1.049275972	0.503	0.576	9.42E-05	5
C4orf3.1	6.79E-09	0.625602294	0.269	0.463	0.000155988	5
LITAF.2	7.71E-09	0.608108983	0.22	0.376	0.000177165	5
RAP1A.1	8.78E-09	0.533005995	0.211	0.358	0.000201754	5
CYBA.2	1.03E-08	0.850299224	0.561	0.694	0.00023629	5
ATP6V0E1.3	1.63E-08	0.553399143	0.313	0.53	0.000374334	5
CALR.1	2.77E-08	0.738263563	0.271	0.453	0.000636679	5
RGS2.4	4.59E-08	1.694941905	0.358	0.334	0.001054086	5
HSD17B11	6.06E-08	0.513199716	0.149	0.258	0.001392703	5
FCGR3A.2	7.79E-08	0.999015267	0.285	0.231	0.00179095	5
CARD16.2	8.48E-08	0.707162322	0.194	0.324	0.001948496	5
MT2A.5	1.64E-07	0.661031548	0.582	0.664	0.003761777	5
PGK1.4	6.82E-07	0.643461053	0.281	0.46	0.015663697	5
SERPINA1.4	6.94E-07	0.789212735	0.313	0.276	0.015951023	5
NOP10.1	8.90E-07	0.657235727	0.215	0.344	0.020445411	5
CTSC.3	1.27E-06	0.747292644	0.219	0.354	0.029228021	5
TYMP.4	1.91E-06	1.356623547	0.312	0.295	0.043781797	5
RAB5C.3	2.00E-06	0.516429668	0.17	0.274	0.045947091	5
APOE.5	0	3.299321439	0.995	0.406	0	6
C3	0	2.968853184	0.992	0.093	0	6
HLA-DRB1.6	0	2.944818115	1	0.567	0	6
CST3.5	0	2.891460258	1	0.351	0	6
C1QB.3	0	2.85182091	0.971	0.193	0	6
C1QC.1	0	2.838326778	0.951	0.109	0	6
APOC1.4	0	2.823523212	0.977	0.244	0	6
HLA-DPB1.5	0	2.777529596	1	0.611	0	6
C1QA.6	0	2.76331102	0.983	0.208	0	6
CD74.5	0	2.759481561	1	0.782	0	6

HLA-DRA.6	0	2.733667729	1	0.654	0	6
HLA-DPA1.6	0	2.70351187	1	0.546	0	6
GPX1.6	0	2.596188213	1	0.396	0	6
MS4A6A.2	0	2.533869989	1	0.148	0	6
HLA-DQA1.4	0	2.341688428	0.997	0.306	0	6
FCGR3A.3	0	2.31417835	0.996	0.19	0	6
TREM2	0	2.234456691	0.931	0.043	0	6
NPC2.4	0	2.233224304	0.999	0.344	0	6
KCTD12	0	2.173440024	0.979	0.088	0	6
HLA-DMB.1	0	2.171521588	0.993	0.166	0	6
SGK1	0	2.147359314	0.963	0.14	0	6
MS4A7	0	2.145862011	0.988	0.104	0	6
SLC1A3	0	2.143317543	0.944	0.057	0	6
TYROBP.6	0	2.127580436	1	0.353	0	6
HLA-DMA.5	0	2.095716324	0.997	0.247	0	6
FCGR2A	0	2.023658105	0.983	0.092	0	6
C1orf162.1	0	2.006796592	0.995	0.169	0	6
PLXDC2	0	2.000616257	0.96	0.078	0	6
MAFB	0	1.991998708	0.914	0.088	0	6
HLA-DQB1.5	0	1.951033258	0.997	0.358	0	6
GPR34	0	1.926938372	0.91	0.05	0	6
CSF1R	0	1.924492347	0.939	0.063	0	6
MEF2C	0	1.909545854	0.968	0.104	0	6
GRN.5	0	1.904875493	0.992	0.212	0	6
LILRB4	0	1.889154472	0.938	0.054	0	6
OGFRL1	0	1.867150602	0.936	0.08	0	6
CSF2RA	0	1.85896314	0.924	0.06	0	6
CXCL16	0	1.849512336	0.955	0.094	0	6
ALDH2	0	1.849202834	0.963	0.109	0	6
PSAP.6	0	1.841416254	1	0.438	0	6
CD302	0	1.841313092	0.952	0.079	0	6
AIF1.6	0	1.832972121	1	0.217	0	6
FGL2	0	1.83030473	0.951	0.105	0	6
HERPUD1.3	0	1.829897419	0.993	0.464	0	6
MARCKS	0	1.814916163	0.939	0.107	0	6
CTSB.5	0	1.791831852	0.995	0.263	0	6
FCGRT.3	0	1.790324581	0.993	0.199	0	6
SLC11A1	0	1.789795948	0.902	0.087	0	6
MEF2A	0	1.784220642	0.951	0.117	0	6
USP53	0	1.773952233	0.855	0.064	0	6
SAT1.5	0	1.767921534	1	0.61	0	6
FN1	0	1.764016302	0.812	0.085	0	6
CEBPD.4	0	1.761697258	0.999	0.238	0	6
CD14.1	0	1.743617936	0.884	0.1	0	6
VSIG4	0	1.726813342	0.828	0.06	0	6
CD163	0	1.714431022	0.894	0.082	0	6
IL18	0	1.710545014	0.908	0.061	0	6
PLD4	0	1.690534291	0.84	0.034	0	6
FCER1G.6	0	1.683286233	0.996	0.264	0	6
MSR1	0	1.673881693	0.936	0.068	0	6
LY86	0	1.648460326	0.91	0.068	0	6
PPT1.4	0	1.647348692	0.979	0.2	0	6
CPVL	0	1.635121073	0.899	0.081	0	6
ALOX5AP.3	0	1.620391497	0.993	0.291	0	6
FAM26F	0	1.616356839	0.908	0.091	0	6
FCGR1A	0	1.615789975	0.839	0.042	0	6
LIPA	0	1.597851223	0.928	0.142	0	6
RPS4Y1.1	0	1.596369542	0.941	0.075	0	6

MNDA.1	0	1.588572694	0.964	0.094	0	6
CAPG.1	0	1.580823425	0.969	0.188	0	6
LST1.2	0	1.532454752	0.996	0.183	0	6
LPAR6	0	1.524256006	0.916	0.108	0	6
SPI1	0	1.519971933	0.952	0.097	0	6
RGS10.3	0	1.505761503	0.98	0.256	0	6
IL13RA1	0	1.504980321	0.87	0.071	0	6
MFSD1	0	1.501096003	0.928	0.102	0	6
EPB41L2	0	1.498941765	0.778	0.057	0	6
RNASET2.6	0	1.496458911	0.999	0.325	0	6
TLR2	0	1.493744382	0.887	0.061	0	6
CD68.1	0	1.462733309	0.965	0.153	0	6
RNF130	0	1.462000149	0.955	0.121	0	6
RNASE6	0	1.445570449	0.93	0.105	0	6
LYZ.5	0	1.437748946	0.995	0.314	0	6
TBXAS1	0	1.433217626	0.911	0.082	0	6
LPCAT2	0	1.430159161	0.822	0.044	0	6
CSF3R	0	1.426512814	0.851	0.05	0	6
SORL1	0	1.413701043	0.926	0.142	0	6
FKBP5.2	0	1.398942397	0.996	0.361	0	6
CTSS.5	0	1.381088193	0.999	0.327	0	6
ADAP2	0	1.380267629	0.807	0.044	0	6
SLC8A1	0	1.373931947	0.823	0.043	0	6
CTSZ	0	1.372972521	0.896	0.116	0	6
TYMP.5	0	1.342227507	0.968	0.257	0	6
KCNMA1	0	1.336282396	0.742	0.035	0	6
SRGAP1	0	1.333487861	0.836	0.069	0	6
CLEC7A	0	1.32748083	0.907	0.091	0	6
SLCO2B1	0	1.315455885	0.794	0.037	0	6
ARHGAP24	0	1.292680557	0.815	0.04	0	6
CYBB	0	1.292661582	0.854	0.083	0	6
CTSH	0	1.29225379	0.926	0.134	0	6
OLR1	0	1.287421646	0.709	0.046	0	6
YWHAH.4	0	1.28433949	0.967	0.244	0	6
IFNGR1.3	0	1.259839819	0.971	0.219	0	6
CPM	0	1.256295523	0.789	0.1	0	6
DOCK4	0	1.253484766	0.765	0.033	0	6
IFI30	0	1.251533907	0.773	0.074	0	6
GLUL.4	0	1.223112689	0.988	0.267	0	6
RAB31	0	1.222953576	0.874	0.088	0	6
IGSF6	0	1.220530413	0.766	0.06	0	6
SERPINF1	0	1.219624084	0.757	0.051	0	6
OLFML3	0	1.20835113	0.614	0.015	0	6
CD9.1	0	1.204103989	0.947	0.182	0	6
CLEC4E	0	1.202079587	0.777	0.072	0	6
ARL5A.1	0	1.201621326	0.918	0.195	0	6
FCGR1B	0	1.189189487	0.677	0.02	0	6
1-Mar	0	1.177208833	0.795	0.061	0	6
CD86	0	1.176408507	0.805	0.055	0	6
PABPC4	0	1.172941034	0.899	0.162	0	6
C10orf54.4	0	1.172144197	0.956	0.243	0	6
HCLS1.2	0	1.167394581	0.977	0.285	0	6
GAPT	0	1.163240935	0.715	0.038	0	6
CNPY3	0	1.161149451	0.936	0.177	0	6
PKIB	0	1.152839904	0.714	0.039	0	6
ALOX5	0	1.142397026	0.809	0.06	0	6
ADAM28	0	1.14235901	0.771	0.061	0	6
TM6SF1	0	1.13931393	0.757	0.039	0	6

LAIR1	0	1.132746329	0.852	0.08	0	6
SRGAP2	0	1.129717402	0.785	0.063	0	6
FCGR2B	0	1.126198313	0.73	0.049	0	6
ANKRD22	0	1.107976637	0.726	0.041	0	6
DAB2	0	1.095369078	0.879	0.121	0	6
RP11-552D4.1	0	1.094075946	0.57	0.006	0	6
NCOA4	0	1.091474316	0.84	0.14	0	6
TNFSF13B	0	1.088359064	0.793	0.094	0	6
PLBD1	0	1.076466842	0.773	0.056	0	6
NUDT3	0	1.075684205	0.843	0.107	0	6
CLEC5A	0	1.070987659	0.586	0.025	0	6
GNB4	0	1.069773766	0.717	0.06	0	6
CADM1	0	1.065764434	0.681	0.044	0	6
MIS18BP1	0	1.064044634	0.874	0.172	0	6
ACSL1	0	1.05781412	0.781	0.079	0	6
C3AR1	0	1.057202697	0.797	0.076	0	6
ASAH1.3	0	1.052301251	0.918	0.202	0	6
LTC4S	0	1.044954482	0.693	0.047	0	6
GABARAP	0	1.044168545	0.888	0.144	0	6
RASSF4	0	1.043645125	0.886	0.123	0	6
ALCAM	0	1.040001362	0.673	0.047	0	6
TGFBI.1	0	1.033901879	0.951	0.14	0	6
AKR1B1.1	0	1.030899851	0.94	0.197	0	6
RGS18	0	1.030033508	0.746	0.058	0	6
PLTP	0	1.027153354	0.564	0.041	0	6
RB1	0	1.017236991	0.794	0.102	0	6
FCGBP	0	1.013146042	0.351	0.011	0	6
SESN1	0	1.011270797	0.785	0.105	0	6
PRKAG2	0	1.009492182	0.82	0.114	0	6
GNAQ	0	1.008024383	0.777	0.1	0	6
APBB1IP.1	0	1.004607439	0.872	0.193	0	6
ELMO1	0	1.001593917	0.83	0.112	0	6
PILRA	0	0.999902051	0.734	0.058	0	6
ADORA3	0	0.999686	0.626	0.024	0	6
HEXA	0	0.997699663	0.883	0.157	0	6
ARHGAP18	0	0.997232819	0.876	0.159	0	6
TNPO1	0	0.99474023	0.747	0.095	0	6
PICALM	0	0.987848468	0.864	0.151	0	6
PAK1	0	0.986723367	0.739	0.064	0	6
SAMHD1	0	0.983679939	0.87	0.17	0	6
DSE	0	0.979206951	0.684	0.048	0	6
HTRA1	0	0.975857895	0.731	0.074	0	6
LIMS1.4	0	0.972735154	0.9	0.212	0	6
UBL3	0	0.969561796	0.799	0.13	0	6
HLA-DOA	0	0.969252914	0.695	0.046	0	6
CFD	0	0.966506182	0.737	0.106	0	6
NUDT16	0	0.961565296	0.836	0.12	0	6
FRMD4A	0	0.961062126	0.657	0.042	0	6
TANC2	0	0.959119657	0.64	0.038	0	6
SLC7A7	0	0.956261756	0.739	0.065	0	6
LINC01094	0	0.954046018	0.576	0.021	0	6
SCPEP1	0	0.94323676	0.775	0.101	0	6
ATP6V0B.3	0	0.941088583	0.953	0.258	0	6
DPYSL2	0	0.939171589	0.786	0.103	0	6
ATP6V1B2	0	0.936614886	0.765	0.106	0	6
ZFHX3	0	0.936054096	0.684	0.053	0	6
CCDC88A	0	0.93487238	0.701	0.072	0	6
MAN2A1	0	0.933944094	0.709	0.065	0	6

AZI2	0	0.929649005	0.656	0.101	0	6
FPR1	0	0.928771786	0.734	0.061	0	6
PYCARD.3	0	0.921448689	0.911	0.209	0	6
LHFPL2	0	0.919908269	0.616	0.023	0	6
BHLHE41	0	0.918380368	0.634	0.048	0	6
LYN	0	0.915307518	0.763	0.085	0	6
DST	0	0.914363823	0.641	0.05	0	6
KCNMB1	0	0.905273801	0.61	0.031	0	6
SRGAP2C	0	0.90092938	0.758	0.101	0	6
SIGLEC10	0	0.89711418	0.622	0.044	0	6
FILIP1L	0	0.895701007	0.637	0.047	0	6
SRGAP2B	0	0.887670279	0.705	0.085	0	6
MAP3K8	0	0.886076089	0.795	0.115	0	6
AXL	0	0.88118086	0.625	0.052	0	6
SDCCAG8	0	0.875690347	0.742	0.097	0	6
VMO1	0	0.875365445	0.555	0.028	0	6
LAP3	0	0.875162065	0.749	0.123	0	6
RHBDF2	0	0.868373929	0.731	0.082	0	6
A2M	0	0.867689502	0.922	0.155	0	6
APLP2.4	0	0.863653459	0.934	0.217	0	6
SKAP2	0	0.856559784	0.82	0.141	0	6
CSTA.1	0	0.85612834	0.812	0.093	0	6
ZNF812P	0	0.855808705	0.477	0.014	0	6
METTL7A	0	0.853543161	0.707	0.08	0	6
FEZ2	0	0.850522069	0.791	0.103	0	6
LGALS9	0	0.845986148	0.717	0.086	0	6
SWAP70	0	0.836434895	0.685	0.074	0	6
TMEM176B	0	0.835016971	0.783	0.119	0	6
P2RY13	0	0.833295242	0.56	0.032	0	6
IRAK3	0	0.831817065	0.705	0.06	0	6
GAA	0	0.831154427	0.67	0.063	0	6
SLC43A2	0	0.828030187	0.637	0.054	0	6
SNX10	0	0.820213241	0.851	0.161	0	6
CLN8	0	0.81760275	0.677	0.078	0	6
BMP2K	0	0.815659889	0.581	0.03	0	6
TFEC	0	0.81131864	0.586	0.03	0	6
LY96	0	0.811252203	0.727	0.087	0	6
SYK	0	0.809495689	0.638	0.044	0	6
BTK	0	0.806033765	0.614	0.035	0	6
FMNL2	0	0.80551859	0.552	0.03	0	6
FCHO2	0	0.805217528	0.577	0.042	0	6
TMEM14C	0	0.79863477	0.884	0.19	0	6
LAT2	0	0.796867817	0.676	0.063	0	6
ST14	0	0.793924407	0.602	0.033	0	6
RNF13	0	0.793892641	0.847	0.17	0	6
FGD4	0	0.792675383	0.644	0.053	0	6
DPYD	0	0.790225229	0.689	0.079	0	6
DNASE2	0	0.785610261	0.767	0.124	0	6
TOP1	0	0.785138863	0.815	0.177	0	6
BASP1	0	0.774997685	0.601	0.04	0	6
SAP30	0	0.774969847	0.678	0.079	0	6
CEP170	0	0.774326763	0.646	0.057	0	6
SOAT1	0	0.7736995	0.662	0.074	0	6
SCIN	0	0.773429329	0.434	0.011	0	6
FAM105A	0	0.772249358	0.658	0.082	0	6
PTGS1	0	0.772181518	0.576	0.023	0	6
DRAM2	0	0.77145971	0.806	0.138	0	6
RIN3	0	0.769502642	0.713	0.089	0	6

CHPT1	0	0.76770384	0.717	0.106	0	6
HEXB	0	0.763909835	0.791	0.15	0	6
SFMBT2	0	0.763470564	0.625	0.065	0	6
SYNGR2	0	0.75973782	0.773	0.133	0	6
MS4A4A	0	0.755773472	0.578	0.062	0	6
CD300A	0	0.754896697	0.686	0.075	0	6
STX6	0	0.752249357	0.626	0.058	0	6
STX7	0	0.744682815	0.705	0.1	0	6
AP1S2.1	0	0.739893666	0.867	0.189	0	6
GRINA	0	0.737526921	0.79	0.124	0	6
THEMIS2	0	0.734775381	0.715	0.085	0	6
AP1B1	0	0.731279953	0.609	0.061	0	6
FRMD4B	0	0.727923772	0.737	0.107	0	6
CAMK1D	0	0.727547805	0.626	0.066	0	6
TKT.1	0	0.724667714	0.854	0.193	0	6
PADI2	0	0.72048682	0.46	0.011	0	6
CRTAP	0	0.715512647	0.718	0.11	0	6
LRP1	0	0.714049629	0.548	0.041	0	6
ARRB2	0	0.712387903	0.816	0.161	0	6
PEA15	0	0.707377022	0.656	0.08	0	6
RAB32	0	0.706875739	0.621	0.063	0	6
HCK	0	0.70352417	0.624	0.053	0	6
CTTNBP2NL	0	0.703505765	0.624	0.064	0	6
PAPOLG	0	0.700421341	0.565	0.042	0	6
LRRK2	0	0.699537386	0.544	0.044	0	6
DAGLB	0	0.697427666	0.537	0.032	0	6
SCIMP	0	0.697230359	0.545	0.034	0	6
NUP214	0	0.695408738	0.652	0.083	0	6
GSN.1	0	0.694394171	0.96	0.183	0	6
SPINT2	0	0.684295115	0.783	0.114	0	6
SKIL	0	0.680123425	0.789	0.153	0	6
HIF1A	0	0.676503773	0.747	0.151	0	6
PARVG	0	0.676157146	0.749	0.133	0	6
TLR5	0	0.674628114	0.46	0.019	0	6
CD81	0	0.674405677	0.741	0.129	0	6
C5AR1	0	0.673499669	0.516	0.06	0	6
DENND3	0	0.666517477	0.577	0.069	0	6
CREG1	0	0.665635139	0.68	0.101	0	6
SLC40A1	0	0.663089836	0.488	0.055	0	6
APPL1	0	0.662614865	0.689	0.112	0	6
CD4	0	0.660425429	0.699	0.106	0	6
ABHD12	0	0.659454719	0.61	0.074	0	6
CYFIP1	0	0.65412042	0.525	0.049	0	6
SSH2	0	0.65385985	0.733	0.126	0	6
CTNND1	0	0.652639376	0.585	0.059	0	6
SHTN1	0	0.651443796	0.511	0.036	0	6
LRRC25	0	0.65035642	0.552	0.041	0	6
SERPINA1.5	0	0.649170653	0.976	0.239	0	6
CHN2	0	0.648731206	0.496	0.038	0	6
PSTPIP2	0	0.647613381	0.521	0.052	0	6
CNDP2	0	0.646067712	0.85	0.177	0	6
PLSCR1	0	0.643717769	0.765	0.127	0	6
ALOX15B	0	0.641614231	0.4	0.022	0	6
QKI	0	0.640828387	0.628	0.088	0	6
ATP6AP1	0	0.633487595	0.737	0.134	0	6
UNC93B1	0	0.632812726	0.535	0.05	0	6
TPP1	0	0.631714364	0.766	0.151	0	6
CAT	0	0.630483597	0.751	0.133	0	6

RHOBTB3	0	0.630058441	0.489	0.033	0	6
HAVCR2.2	0	0.625095981	0.86	0.163	0	6
NAIP	0	0.62349551	0.507	0.041	0	6
INTS10	0	0.623414466	0.632	0.103	0	6
SPTLC2	0	0.623180485	0.613	0.08	0	6
GPR155	0	0.622910305	0.553	0.074	0	6
COMT	0	0.617852503	0.767	0.153	0	6
NAGK	0	0.616076074	0.69	0.109	0	6
TGFBR1	0	0.610678873	0.459	0.032	0	6
PTEN	0	0.610433654	0.688	0.12	0	6
FGR	0	0.610340121	0.645	0.085	0	6
CLEC12A	0	0.607418002	0.516	0.061	0	6
CD33	0	0.606421097	0.505	0.034	0	6
KLHL5	0	0.606009184	0.557	0.062	0	6
ETS2	0	0.605446949	0.658	0.088	0	6
IFNGR2	0	0.605313275	0.644	0.093	0	6
PLEKHO1	0	0.602851749	0.601	0.084	0	6
BEX4	0	0.602576383	0.641	0.091	0	6
ZYX	0	0.600146204	0.681	0.126	0	6
DAPK1	0	0.598089056	0.5	0.042	0	6
PRKAG2-AS1	0	0.595392226	0.501	0.044	0	6
SCAMP2	0	0.591865038	0.761	0.153	0	6
HSPA6.1	0	0.586736296	0.505	0.066	0	6
ACSL4	0	0.581686252	0.523	0.057	0	6
RP11-108M9.4	0	0.581136699	0.529	0.045	0	6
SVBP	0	0.579142204	0.6	0.086	0	6
IER3	0	0.578168454	0.544	0.084	0	6
C1orf54	0	0.578137175	0.547	0.061	0	6
RBM47	0	0.577142517	0.528	0.054	0	6
ITPR2	0	0.574130602	0.566	0.077	0	6
ENTPD1	0	0.57311187	0.709	0.122	0	6
SLC31A2	0	0.572650453	0.585	0.079	0	6
RTN1	0	0.570811918	0.404	0.015	0	6
ST3GAL6	0	0.569827854	0.463	0.021	0	6
STK38L	0	0.569589003	0.5	0.049	0	6
EPB41L3	0	0.569561265	0.48	0.038	0	6
BCAT1	0	0.568835112	0.445	0.038	0	6
HMOX1	0	0.568002109	0.63	0.085	0	6
CTSL	0	0.564334418	0.701	0.127	0	6
ITPRIPL2	0	0.563047682	0.447	0.031	0	6
C20orf27	0	0.562392286	0.572	0.074	0	6
RNASE2	0	0.562318342	0.342	0.011	0	6
FAM96A	0	0.557303709	0.731	0.135	0	6
TAX1BP3	0	0.552482725	0.658	0.114	0	6
LILRB1	0	0.551659812	0.479	0.038	0	6
AP001055.6	0	0.548429606	0.463	0.04	0	6
SNCA	0	0.546323933	0.431	0.023	0	6
POU2F2	0	0.543260459	0.499	0.064	0	6
KLF7	0	0.541839841	0.537	0.066	0	6
NLRP3	0	0.541756827	0.461	0.053	0	6
PLXNC1	0	0.541411042	0.469	0.032	0	6
NDRG2	0	0.541268742	0.509	0.052	0	6
MMP2	0	0.541121613	0.395	0.014	0	6
PTAFR	0	0.540630686	0.445	0.027	0	6
RNF144B	0	0.540168784	0.495	0.058	0	6
ADPGK	0	0.536747176	0.713	0.13	0	6
FAM110B	0	0.536502004	0.383	0.01	0	6
MITF	0	0.535220135	0.452	0.031	0	6

KIAA1033	0	0.534770215	0.621	0.104	0	6
PALD1	0	0.533994228	0.398	0.016	0	6
TNFSF12	0	0.533104928	0.52	0.055	0	6
MERTK	0	0.533071435	0.358	0.022	0	6
ZSWIM7	0	0.53262996	0.628	0.098	0	6
BRI3	0	0.531458839	0.703	0.131	0	6
FGD2	0	0.530847343	0.479	0.039	0	6
IRS2	0	0.523517547	0.556	0.081	0	6
JAK2	0	0.523393954	0.43	0.038	0	6
VAMP3	0	0.520747858	0.653	0.102	0	6
ZNF385A	0	0.51901672	0.439	0.031	0	6
EMILIN2	0	0.518178282	0.423	0.031	0	6
MANBA	0	0.518094051	0.487	0.049	0	6
PRCP	0	0.516700992	0.629	0.094	0	6
C10orf11	0	0.51475048	0.505	0.045	0	6
HNMT	0	0.514245419	0.628	0.087	0	6
ITGAM	0	0.513231601	0.415	0.031	0	6
SPECC1	0	0.511558922	0.426	0.032	0	6
PKP2	0	0.511546044	0.396	0.033	0	6
GALNT2	0	0.51081624	0.489	0.056	0	6
LPL	0	0.510211639	0.279	0.021	0	6
NAGA	0	0.507629412	0.505	0.052	0	6
UBE2E2	0	0.507428888	0.493	0.047	0	6
TIMP2	0	0.50587962	0.501	0.057	0	6
LACTB	0	0.505836899	0.524	0.071	0	6
TCF4	0	0.501187634	0.681	0.087	0	6
GPRIN3.1	3.92E-307	0.542935478	0.613	0.103	9.01E-303	6
POLD4	5.94E-307	0.506694604	0.585	0.095	1.37E-302	6
ZNF106	3.15E-306	0.567822532	0.669	0.124	7.24E-302	6
CD84.3	2.96E-304	0.721894806	0.887	0.203	6.80E-300	6
ZEB2.3	8.70E-304	0.720897121	0.879	0.204	2.00E-299	6
BAZ2B	8.96E-303	0.563580477	0.689	0.13	2.06E-298	6
PTPN18	7.91E-301	0.537609578	0.612	0.107	1.82E-296	6
ERCC1	1.43E-300	0.507135687	0.636	0.115	3.28E-296	6
CMTM6	1.10E-296	0.630463594	0.84	0.19	2.54E-292	6
FOXO3	1.89E-293	0.50322621	0.637	0.116	4.34E-289	6
LAPTM5.4	2.38E-292	1.290551343	1	0.543	5.46E-288	6
GLIPR1.3	3.37E-292	0.897708069	0.955	0.262	7.75E-288	6
SMAP2.2	1.05E-290	0.939396236	0.948	0.261	2.42E-286	6
NCF1	1.33E-288	0.731692195	0.725	0.15	3.06E-284	6
AOAH.1	7.92E-288	0.503843183	0.644	0.117	1.82E-283	6
AZIN1	1.34E-287	0.514929917	0.613	0.111	3.08E-283	6
RNF149.3	2.44E-287	0.733413831	0.904	0.233	5.61E-283	6
GNAI2.3	1.12E-286	0.792843499	0.886	0.224	2.57E-282	6
RAC1.4	6.92E-284	1.091790279	0.997	0.417	1.59E-279	6
PDK4.2	1.77E-283	0.5369224	0.773	0.171	4.07E-279	6
MAP3K2	4.14E-282	0.501127452	0.654	0.125	9.50E-278	6
ZBTB16	6.63E-280	0.594737651	0.814	0.187	1.52E-275	6
MAT2A	2.93E-278	0.668310793	0.656	0.133	6.73E-274	6
DCK	2.01E-276	0.535135451	0.795	0.174	4.63E-272	6
PGLS	2.04E-276	0.577300823	0.85	0.204	4.69E-272	6
RHOG.2	1.70E-270	0.735045187	0.882	0.229	3.91E-266	6
TSPO.3	2.60E-270	1.114881124	0.989	0.388	5.97E-266	6
REL.3	4.52E-270	0.9860819	0.945	0.286	1.04E-265	6
NCKAP1L.1	4.16E-267	0.506968404	0.695	0.142	9.56E-263	6
GRB2.1	1.96E-262	0.636388047	0.827	0.204	4.51E-258	6
H2AFY.4	1.41E-260	0.730205878	0.908	0.248	3.23E-256	6
TMEM219.3	4.22E-258	0.559065242	0.898	0.235	9.70E-254	6

SPP1.4	5.15E-257	1.012622669	0.759	0.185	1.18E-252	6
COTL1.2	3.79E-254	1.138907719	0.992	0.402	8.70E-250	6
FTL.5	1.03E-251	0.992097157	1	0.933	2.37E-247	6
S100A11.5	4.57E-247	1.150755851	0.999	0.567	1.05E-242	6
FAM49B.2	1.84E-241	0.810613026	0.949	0.305	4.23E-237	6
HN1.1	2.75E-241	0.630689458	0.805	0.207	6.33E-237	6
HSD17B11.1	1.22E-240	0.604159796	0.826	0.219	2.79E-236	6
TAOK3.1	1.67E-240	0.627711451	0.866	0.231	3.83E-236	6
OSBPL8	5.68E-240	0.522876143	0.765	0.183	1.30E-235	6
SEC11A.1	6.22E-239	0.77866582	0.941	0.305	1.43E-234	6
MTPN.1	5.59E-235	0.503347381	0.778	0.192	1.28E-230	6
ITM2B.5	5.94E-233	1.045226553	1	0.767	1.36E-228	6
ATP1B3.4	1.36E-228	0.686370111	0.846	0.236	3.13E-224	6
DHRS7.1	2.97E-220	0.579807872	0.858	0.242	6.82E-216	6
MT-ND4L.2	2.06E-217	0.800226175	0.955	0.327	4.73E-213	6
SH3BGRL.3	5.18E-214	0.773671826	0.973	0.357	1.19E-209	6
PRMT2.4	2.40E-212	0.590348674	0.878	0.256	5.51E-208	6
SDCBP.5	5.27E-211	0.722072928	0.951	0.34	1.21E-206	6
WASF2.1	2.65E-210	0.66652938	0.922	0.296	6.08E-206	6
AP2S1.5	8.40E-210	0.523902762	0.908	0.276	1.93E-205	6
RCSD1.4	6.31E-205	0.541441354	0.805	0.218	1.45E-200	6
CANX.4	3.93E-203	0.510480116	0.88	0.267	9.03E-199	6
CTSD.2	1.64E-200	0.947632602	0.969	0.407	3.77E-196	6
ATP6AP2.2	4.13E-200	0.532783183	0.895	0.273	9.50E-196	6
ITGB2.4	3.60E-197	0.871404516	0.972	0.383	8.28E-193	6
RHOA.3	1.97E-192	0.856530957	0.996	0.48	4.52E-188	6
GPNMB	3.01E-189	0.531419615	0.403	0.073	6.91E-185	6
MT-ND1.2	2.45E-187	0.892137726	0.999	0.775	5.63E-183	6
CYBA.3	5.28E-185	0.905417865	1	0.669	1.21E-180	6
GPX4.4	1.57E-184	0.651317057	0.988	0.423	3.61E-180	6
ANXA5.4	2.56E-184	0.514226191	0.943	0.318	5.89E-180	6
MT-ND3.4	4.37E-184	0.850575244	0.999	0.821	1.00E-179	6
ROCK1.1	3.09E-182	0.629677795	0.898	0.299	7.11E-178	6
CLU.2	7.95E-181	-0.62825208	0.694	0.17	1.83E-176	6
RGS2.5	5.52E-172	0.734164696	0.875	0.305	1.27E-167	6
MT-ND5.4	1.08E-170	0.971759277	0.997	0.588	2.49E-166	6
CELF2.5	1.31E-164	0.715508011	0.98	0.394	3.02E-160	6
MTDH.2	2.62E-162	0.621121837	0.938	0.356	6.02E-158	6
ACTR2.2	1.49E-159	0.576130329	0.922	0.336	3.43E-155	6
EVI2B.2	6.78E-159	0.532527996	0.887	0.304	1.56E-154	6
TUBA1B.4	1.20E-156	0.832010614	0.903	0.386	2.76E-152	6
PDZK1IP1	8.04E-153	-0.56074417	0.469	0.103	1.85E-148	6
SLC25A5.2	6.87E-147	0.528485655	0.93	0.355	1.58E-142	6
VAMP8.3	3.12E-145	0.606386949	0.979	0.431	7.17E-141	6
HSPA1A.2	1.01E-143	0.539054079	0.68	0.209	2.32E-139	6
SLA.5	4.21E-143	0.503687177	0.961	0.367	9.68E-139	6
TPT1.4	8.81E-143	0.621511039	1	0.919	2.03E-138	6
FYB.4	2.47E-142	0.696954195	0.975	0.431	5.67E-138	6
RPS27.3	1.02E-138	-0.86492891	1	0.974	2.35E-134	6
ARPC1B.1	1.51E-138	0.666201359	0.991	0.498	3.46E-134	6
DDX17.3	3.95E-138	0.527823447	0.93	0.369	9.07E-134	6
NEAT1.4	6.15E-137	0.706924274	0.997	0.739	1.41E-132	6
SERP1	6.26E-137	0.531648169	0.971	0.406	1.44E-132	6
CD63.5	2.75E-134	0.535950097	0.995	0.493	6.32E-130	6
RPS4X.4	1.68E-133	-0.86700222	1	0.935	3.86E-129	6
RGS1.6	2.19E-133	0.809389216	0.997	0.68	5.04E-129	6
AC090498.1.3	3.98E-129	0.544610276	0.991	0.473	9.16E-125	6
FXYD5.3	4.36E-126	0.584194261	0.971	0.453	1.00E-121	6

XIST.4	3.05E-115	-2.197328449	0.005	0.448	7.00E-111	6
ARPC3.3	2.81E-111	0.510590521	0.995	0.583	6.46E-107	6
MT-ND4.2	7.84E-105	0.563831964	0.999	0.867	1.80E-100	6
IL32.6	1.67E-103	-2.976091022	0.323	0.592	3.83E-99	6
CD2.5	3.41E-102	-2.558228706	0.094	0.477	7.84E-98	6
RPL17.4	2.36E-101	0.505155294	0.997	0.57	5.43E-97	6
CCL5.5	3.61E-100	-3.651707308	0.291	0.577	8.30E-96	6
HLA-A.4	2.22E-99	-0.943452795	1	0.888	5.10E-95	6
B2M.4	3.15E-99	-0.560931106	1	0.987	7.23E-95	6
CD3D.6	8.96E-98	-2.540326902	0.138	0.494	2.06E-93	6
EEF1B2	1.80E-95	0.548769826	0.997	0.643	4.14E-91	6
MALAT1.5	2.10E-95	-0.868203152	1	0.981	4.82E-91	6
MT-CO3.3	2.28E-94	0.525404735	0.997	0.873	5.23E-90	6
CD52.4	1.51E-92	-2.607507373	0.289	0.564	3.47E-88	6
TXNIP.5	1.26E-87	0.672188169	1	0.75	2.90E-83	6
GZMA.5	1.12E-85	-2.623602987	0.096	0.437	2.56E-81	6
APP	1.90E-85	-0.547567564	0.504	0.16	4.37E-81	6
TRAC.6	1.16E-82	-2.489358203	0.126	0.449	2.66E-78	6
MT1E.2	5.02E-82	-1.011488965	0.493	0.155	1.15E-77	6
DUSP2.5	3.90E-81	-2.503667197	0.125	0.449	8.96E-77	6
RPS26.4	6.79E-80	-0.780307568	1	0.863	1.56E-75	6
CD3E.5	1.69E-79	-1.91114787	0.089	0.415	3.89E-75	6
RPL23A.4	1.54E-78	-0.585216513	0.999	0.926	3.55E-74	6
SOD2.5	1.46E-76	-0.542690035	0.726	0.269	3.34E-72	6
RPS15A.3	6.89E-76	-0.544106393	1	0.93	1.58E-71	6
RPL21.2	2.14E-73	-0.545380455	1	0.949	4.93E-69	6
HLA-C.3	1.33E-69	-0.681885969	0.999	0.876	3.05E-65	6
PLIN2.4	1.27E-66	-0.678017559	0.798	0.31	2.92E-62	6
NNMT.6	2.09E-66	-1.518030408	0.734	0.274	4.80E-62	6
CST7.5	1.57E-65	-2.255016176	0.128	0.404	3.61E-61	6
CD69.6	1.08E-61	-2.729419095	0.382	0.537	2.47E-57	6
PPDPF.3	2.67E-61	-1.686432987	0.723	0.656	6.14E-57	6
CD3G.5	3.99E-60	-1.590688256	0.033	0.308	9.18E-56	6
NDUFC1.5	2.19E-57	-0.533023116	0.638	0.25	5.03E-53	6
ETS1.6	1.06E-55	-1.548565234	0.098	0.352	2.43E-51	6
CRYAB.6	1.88E-55	-1.440322228	0.621	0.239	4.32E-51	6
GZMK.6	2.96E-54	-2.896259585	0.137	0.378	6.79E-50	6
NKG7.6	5.00E-53	-3.003982851	0.233	0.438	1.15E-48	6
TRBC2.5	1.25E-52	-1.998600358	0.16	0.389	2.88E-48	6
LCK.6	1.53E-52	-1.353955086	0.052	0.301	3.52E-48	6
TRBC1.5	2.11E-52	-2.332182241	0.129	0.366	4.85E-48	6
INSR	1.68E-51	-0.972743681	0.352	0.125	3.87E-47	6
RPL35A.3	6.70E-51	-0.507892183	1	0.912	1.54E-46	6
RPS25.3	3.20E-50	-0.516829158	0.999	0.913	7.34E-46	6
ITM2A.6	7.67E-50	-1.736218864	0.086	0.323	1.76E-45	6
CCL4.6	2.49E-48	-2.857478759	0.266	0.45	5.73E-44	6
H3F3B.3	3.24E-47	-0.784199852	0.999	0.825	7.44E-43	6
RPL36.4	6.13E-46	-0.59971591	1	0.907	1.41E-41	6
SYNE2.5	7.30E-46	-1.496794667	0.128	0.344	1.68E-41	6
IFITM3.6	7.42E-46	-0.987227126	0.835	0.352	1.70E-41	6
IL7R.4	6.34E-45	-2.451675097	0.096	0.315	1.46E-40	6
TUBA4A.4	5.84E-39	-1.372125769	0.084	0.283	1.34E-34	6
FYN.4	9.12E-39	-1.23778732	0.077	0.276	2.10E-34	6
CD96.6	1.72E-38	-1.199770551	0.061	0.259	3.95E-34	6
FOSB.2	3.75E-38	-1.560074229	0.281	0.433	8.62E-34	6
CD247.5	8.56E-38	-1.225613286	0.056	0.251	1.97E-33	6
DUSP4.5	1.46E-37	-1.593190197	0.057	0.251	3.36E-33	6
GNLY.6	4.18E-35	-3.074805233	0.076	0.263	9.61E-31	6

CD27.5	4.44E-35	-1.608266939	0.093	0.277	1.02E-30	6
KLF6.5	1.40E-33	-1.669483942	0.818	0.639	3.22E-29	6
BTG1.5	6.44E-31	-0.86953365	0.996	0.834	1.48E-26	6
CYB5A.2	7.04E-31	-1.105092232	0.487	0.215	1.62E-26	6
CRIP1.4	3.05E-30	-1.439274095	0.141	0.302	7.02E-26	6
LTB.5	3.70E-30	-1.774849453	0.154	0.31	8.49E-26	6
TPM1	2.16E-29	-1.027703764	0.414	0.182	4.95E-25	6
DDX5.5	7.58E-29	-0.599299334	0.996	0.801	1.74E-24	6
IL2RG.6	2.88E-28	-1.393010214	0.263	0.379	6.62E-24	6
SEPW1.6	6.80E-28	-0.624465607	0.721	0.339	1.56E-23	6
ATP1B1.1	9.24E-28	-0.631775376	0.301	0.128	2.12E-23	6
ADIRF.4	3.94E-27	-1.692249885	0.499	0.227	9.04E-23	6
HNRNPH1	3.83E-24	-0.608392943	0.625	0.303	8.80E-20	6
ANXA6.4	5.92E-23	-1.048949478	0.124	0.257	1.36E-18	6
HLA-DRB5.6	6.22E-23	-2.274943728	0.297	0.378	1.43E-18	6
ACAP1.6	1.61E-22	-1.237692118	0.233	0.339	3.71E-18	6
CDC42SE1.3	2.51E-22	-0.576164706	0.468	0.227	5.76E-18	6
S100A13	4.25E-22	-0.58992197	0.278	0.125	9.77E-18	6
JUNB.5	1.72E-21	-1.347409187	0.938	0.711	3.96E-17	6
HBA2.4	1.00E-20	-2.730873151	0.475	0.238	2.30E-16	6
DSTN.4	4.93E-20	-0.898529545	0.593	0.291	1.13E-15	6
RP11-347P5.1.6	1.55E-19	-1.538308634	0.319	0.379	3.55E-15	6
CD24.1	4.62E-19	-1.620418812	0.35	0.168	1.06E-14	6
TNFAIP3.6	7.38E-19	-1.655071503	0.452	0.447	1.70E-14	6
TIMP1.6	9.65E-19	-0.990854991	0.593	0.298	2.22E-14	6
C12orf57.3	4.51E-18	-1.315312195	0.319	0.375	1.04E-13	6
ANGPTL4.1	5.74E-18	-1.423408248	0.326	0.158	1.32E-13	6
PGK1.5	7.77E-18	-0.513967052	0.843	0.428	1.79E-13	6
EEF1D.3	2.90E-17	-0.545830964	0.987	0.758	6.67E-13	6
JUND.3	6.91E-17	-1.134429304	0.226	0.314	1.59E-12	6
TAGLN2.4	9.47E-17	-0.527765916	0.662	0.339	2.18E-12	6
SMCHD1.2	1.47E-16	-0.503892816	0.609	0.311	3.39E-12	6
TSC22D3.4	7.33E-16	-0.90429995	0.992	0.769	1.68E-11	6
HPCAL1	6.29E-15	-0.565324336	0.318	0.163	1.44E-10	6
CDC42SE2.2	2.80E-14	-0.524708799	0.573	0.301	6.44E-10	6
ADGRE5.5	2.84E-14	-1.121313139	0.23	0.303	6.54E-10	6
ITGB1.2	1.69E-13	-0.703470167	0.854	0.442	3.89E-09	6
DNAJA1.3	2.12E-13	-0.547151737	0.585	0.317	4.87E-09	6
UBC.3	4.09E-13	-0.578440186	0.996	0.831	9.39E-09	6
BIRC3.4	6.27E-13	-1.361832607	0.205	0.276	1.44E-08	6
CREM.3	7.84E-13	-0.640570439	0.491	0.268	1.80E-08	6
SUMO1	1.35E-12	-0.51125244	0.693	0.366	3.10E-08	6
GYPC.4	2.23E-12	-0.978364053	0.246	0.31	5.14E-08	6
MYL12A.3	4.21E-12	-0.819006457	0.964	0.674	9.66E-08	6
RPS27L.4	5.71E-12	-0.616412274	0.835	0.452	1.31E-07	6
BTG2.3	7.56E-12	-0.537487442	0.612	0.343	1.74E-07	6
APOBEC3C.1	1.10E-11	-0.553543657	0.348	0.19	2.54E-07	6
UBB.3	1.19E-11	-0.683227441	0.979	0.705	2.74E-07	6
RARRES3.5	1.92E-11	-1.227074632	0.435	0.409	4.41E-07	6
ANXA1.6	3.73E-11	-1.018108534	0.87	0.449	8.57E-07	6
MT2A.6	9.75E-11	-1.942842618	0.866	0.647	2.24E-06	6
LYST.3	1.10E-09	-0.641693291	0.384	0.218	2.53E-05	6
PIK3IP1.6	1.37E-09	-0.567014839	0.62	0.343	3.16E-05	6
CLEC2D.5	1.76E-09	-1.188156673	0.281	0.308	4.04E-05	6
RGCC.5	1.85E-09	-1.597951261	0.27	0.309	4.24E-05	6
LINC00152.4	1.91E-09	-1.185293465	0.295	0.318	4.38E-05	6
SEPT9.4	2.71E-09	-0.857448193	0.199	0.253	6.23E-05	6
SOD1.3	6.55E-09	-0.519300185	0.838	0.461	0.00015057	6

CALM3.1	1.04E-08	-0.559725281	0.527	0.3	0.000238041	6
PDCD4.3	1.49E-08	-0.629072935	0.513	0.292	0.000342207	6
KMT2E.5	4.21E-08	-0.526387485	0.69	0.388	0.000968045	6
YWHAQ.1	4.67E-08	-0.60789853	0.593	0.335	0.001074331	6
CYTIP.4	7.49E-08	-1.237170586	0.602	0.468	0.0017202	6
HBB.2	8.20E-08	-3.024473159	0.669	0.376	0.001884086	6
OCIAD2.4	1.03E-07	-1.029664031	0.229	0.263	0.002376236	6
BUB3.5	1.09E-07	-0.50938702	0.447	0.261	0.002509726	6
IER2.2	2.11E-07	-1.24096661	0.441	0.399	0.004847179	6
SEC61G.3	2.28E-07	-0.688806852	0.774	0.419	0.005249215	6
CD99.4	3.01E-07	-0.500790298	0.802	0.451	0.006928332	6
CCNL1.5	3.08E-07	-0.558406279	0.681	0.383	0.007067803	6
ICAM3.5	3.30E-07	-0.827906881	0.243	0.274	0.007590396	6
RBM8A	3.69E-07	-0.559282769	0.62	0.355	0.008472083	6
CYCS.3	5.82E-07	-0.553361873	0.664	0.381	0.013367824	6
PRDX2.3	6.60E-07	-0.689620081	0.431	0.255	0.015165714	6
RNF19A.5	1.22E-06	-0.989207159	0.257	0.278	0.02805475	6
ODF2L.3	1.54E-06	-0.980067639	0.235	0.261	0.035433966	6
LYST.4	0	2.05609404	0.821	0.198	0	7
CD8A.2	0	2.052112401	0.862	0.198	0	7
CD27.6	0	2.041209663	0.918	0.235	0	7
TTN.1	0	1.950423803	0.589	0.063	0	7
CD8B.2	0	1.928555356	0.798	0.153	0	7
TNFRSF9.2	0	1.922476371	0.63	0.076	0	7
CST7.6	0	1.913296938	0.954	0.361	0	7
RP11-160E2.6	0	1.85088707	0.599	0.112	0	7
VCAM1	0	1.808758921	0.564	0.073	0	7
LAG3.1	0	1.766396153	0.613	0.076	0	7
CD200R1	0	1.389120559	0.396	0.037	0	7
ADTRP	0	1.241837114	0.347	0.016	0	7
NKG7.7	8.82E-299	1.94170777	0.98	0.4	2.03E-294	7
GZMK.7	2.05E-296	1.850336582	0.953	0.336	4.71E-292	7
APOBEC3G.5	4.65E-286	1.843465471	0.813	0.245	1.07E-281	7
PTCH2	1.17E-284	1.305730766	0.285	0.023	2.68E-280	7
TRAC.7	3.05E-276	1.917344006	0.956	0.406	7.01E-272	7
CCDC141	1.41E-266	1.186982487	0.373	0.044	3.24E-262	7
DUSP4.6	1.55E-262	1.758278741	0.771	0.215	3.57E-258	7
CCL5.6	1.51E-250	1.759991501	0.989	0.541	3.46E-246	7
GZMA.6	9.94E-250	1.718071185	0.944	0.393	2.28E-245	7
CD2.6	7.59E-248	1.697531573	0.95	0.433	1.74E-243	7
RNU12	6.95E-242	1.406501652	0.387	0.055	1.60E-237	7
DUSP2.6	1.80E-239	1.801073884	0.933	0.406	4.14E-235	7
CD3D.7	1.07E-235	1.536557236	0.959	0.451	2.45E-231	7
CCDC64	4.32E-227	0.98416072	0.316	0.037	9.93E-223	7
CLECL1	9.05E-225	1.16180945	0.363	0.051	2.08E-220	7
PRR5L	1.97E-208	0.897763057	0.296	0.035	4.54E-204	7
FTH1.7	1.97E-203	-2.267864704	0.938	0.964	4.54E-199	7
CCND2.2	4.32E-202	1.382597996	0.56	0.139	9.93E-198	7
TNIP3	4.78E-201	0.956469083	0.272	0.03	1.10E-196	7
FASLG	1.18E-194	0.88291713	0.263	0.029	2.71E-190	7
RNF19A.6	1.29E-192	1.411192915	0.734	0.254	2.98E-188	7
ZNF331.3	4.13E-191	1.592917909	0.668	0.209	9.48E-187	7
MCTP2	8.33E-188	1.024197357	0.379	0.064	1.91E-183	7
CMC1.6	4.22E-187	1.643515478	0.739	0.262	9.70E-183	7
NR4A2.4	1.80E-186	1.7350618	0.713	0.255	4.13E-182	7
CD3G.6	2.15E-186	1.245178896	0.788	0.269	4.94E-182	7
HAVCR2.3	2.05E-185	1.483399851	0.605	0.18	4.72E-181	7
TOX2	8.29E-184	0.895655919	0.272	0.034	1.91E-179	7

LIMD2.6	8.28E-182	1.330959491	0.757	0.289	1.90E-177	7
SIT1.2	4.96E-180	1.066325524	0.498	0.111	1.14E-175	7
ITGA4.3	8.00E-175	1.417520514	0.634	0.205	1.84E-170	7
TOX.2	1.95E-174	1.051105321	0.437	0.09	4.49E-170	7
MIR155HG	6.94E-168	1.069842421	0.331	0.055	1.59E-163	7
TRBC2.6	1.25E-167	1.386394784	0.827	0.355	2.87E-163	7
SNAP47	1.98E-167	1.095623032	0.388	0.078	4.55E-163	7
TIGIT.1	6.89E-163	1.051978997	0.49	0.117	1.58E-158	7
SLF1.1	2.82E-162	1.10746233	0.48	0.117	6.49E-158	7
SLFN12L	1.27E-160	0.894293712	0.36	0.066	2.91E-156	7
IFNG	2.31E-160	1.258711564	0.407	0.084	5.31E-156	7
RUNX3.3	3.07E-158	1.352691166	0.604	0.19	7.06E-154	7
FAM3C.1	6.50E-158	1.299479298	0.493	0.137	1.49E-153	7
RASGRP1.1	7.10E-157	1.004968251	0.464	0.111	1.63E-152	7
AC069363.1	3.02E-153	0.880897103	0.267	0.039	6.94E-149	7
HLA-A.5	1.02E-150	0.875234343	0.998	0.888	2.35E-146	7
ATXN1.1	6.82E-150	1.112917731	0.514	0.148	1.57E-145	7
BHLHE40	2.49E-149	1.176941416	0.525	0.155	5.73E-145	7
LBH.3	1.98E-148	1.190304329	0.577	0.181	4.54E-144	7
DTHD1	2.35E-147	1.004111072	0.354	0.069	5.41E-143	7
CRIP1.5	1.04E-146	1.258350856	0.71	0.273	2.39E-142	7
CH17-373J23.1	2.53E-146	1.283260952	0.426	0.103	5.82E-142	7
ARAP2.1	1.10E-144	1.080466472	0.486	0.132	2.52E-140	7
IL2RG.7	4.39E-142	1.146049604	0.8	0.351	1.01E-137	7
NPIPB5.1	5.45E-140	1.056653105	0.419	0.106	1.25E-135	7
RP11-386I14.4	3.52E-139	1.729513168	0.385	0.093	8.08E-135	7
PTPN7.4	2.10E-137	1.114066205	0.604	0.203	4.83E-133	7
TRAF5	1.21E-136	1.020828651	0.373	0.085	2.77E-132	7
TSC22D3.5	8.60E-132	-2.068954987	0.484	0.795	1.98E-127	7
AC090498.1.4	1.56E-131	1.002458263	0.883	0.482	3.58E-127	7
RPL39.2	1.94E-130	-0.915231818	0.976	0.943	4.47E-126	7
TMSB10.5	1.06E-124	-1.111349987	0.965	0.96	2.43E-120	7
PTPRC.7	1.28E-124	0.969071009	0.951	0.6	2.95E-120	7
SPN.2	1.68E-124	1.056787577	0.472	0.143	3.87E-120	7
SIRPG.1	2.14E-122	0.805798755	0.343	0.075	4.91E-118	7
WDR74	6.22E-122	1.069233355	0.432	0.124	1.43E-117	7
DGKH	1.52E-121	0.953125607	0.329	0.073	3.50E-117	7
EOMES.1	2.19E-121	0.819048904	0.34	0.074	5.03E-117	7
FTL.6	6.76E-121	-2.111912788	0.904	0.939	1.55E-116	7
PDCD1	1.26E-120	0.780895898	0.273	0.05	2.89E-116	7
LCK.7	3.10E-120	0.997994882	0.681	0.268	7.12E-116	7
SRSF7.5	8.54E-119	1.070631246	0.874	0.528	1.96E-114	7
CREM.4	1.21E-118	1.319671285	0.631	0.263	2.78E-114	7
FUT8	3.59E-117	0.889207931	0.3	0.064	8.25E-113	7
TXNIP.6	1.50E-112	-2.05038047	0.566	0.773	3.45E-108	7
SH2D2A	4.36E-112	0.808053594	0.369	0.092	1.00E-107	7
FNBP1.6	9.67E-112	1.003867162	0.725	0.35	2.22E-107	7
NAB1	1.05E-110	0.744410728	0.261	0.051	2.41E-106	7
VPS37B	6.40E-109	0.814913597	0.399	0.109	1.47E-104	7
TMEM107	1.39E-108	1.042015413	0.387	0.111	3.20E-104	7
PYHIN1.3	1.58E-108	0.928335238	0.548	0.188	3.64E-104	7
MALAT1.6	1.44E-106	0.552037923	1	0.982	3.31E-102	7
CD3E.6	6.19E-106	0.846529536	0.812	0.377	1.42E-101	7
CDK6	2.23E-104	0.959292124	0.338	0.09	5.14E-100	7
RPL3.4	3.93E-103	-0.818922913	0.982	0.93	9.04E-99	7
TRAF1	1.93E-102	0.80722233	0.288	0.065	4.44E-98	7
SLC38A1.4	4.44E-102	0.941309005	0.555	0.21	1.02E-97	7
HLA-F.6	5.21E-102	0.963941146	0.771	0.422	1.20E-97	7

SUB1.2	3.47E-101	0.892430055	0.857	0.56	7.96E-97	7
GTPBP8	5.85E-101	0.815403267	0.285	0.065	1.34E-96	7
MSI2.3	8.95E-101	0.949946052	0.445	0.146	2.06E-96	7
XIST.5	4.21E-100	0.851046907	0.798	0.406	9.67E-96	7
PRF1.1	7.28E-100	0.615710188	0.571	0.199	1.67E-95	7
LTB.6	8.40E-100	1.023799591	0.66	0.284	1.93E-95	7
IL32.7	1.37E-99	0.886964305	0.91	0.561	3.16E-95	7
FAM118A	2.94E-98	0.86672024	0.316	0.081	6.75E-94	7
CCL4L2.3	2.46E-97	1.356257595	0.504	0.177	5.66E-93	7
HNRNPLL.1	7.78E-97	0.832964027	0.36	0.103	1.79E-92	7
ACAP1.7	1.14E-96	0.889891709	0.692	0.315	2.62E-92	7
RAB27A.1	7.88E-95	0.83283084	0.445	0.148	1.81E-90	7
TPT1.5	1.01E-94	-0.861987182	0.971	0.921	2.32E-90	7
RPS18.4	1.49E-94	-0.72618935	0.991	0.957	3.43E-90	7
CRTAM.1	4.46E-94	1.061261682	0.323	0.084	1.03E-89	7
HLA-B.5	7.51E-94	0.564886445	0.997	0.91	1.73E-89	7
APMAP.2	9.37E-93	0.890779075	0.507	0.198	2.15E-88	7
CHST12.3	5.61E-92	0.872501773	0.513	0.194	1.29E-87	7
VIM.5	1.00E-91	-1.798723913	0.63	0.778	2.31E-87	7
RPL13.4	1.31E-89	-0.662073389	0.988	0.964	3.00E-85	7
APOE.6	1.36E-89	-3.481241038	0.047	0.457	3.14E-85	7
FOS.5	1.50E-89	-1.942113379	0.373	0.697	3.45E-85	7
APOBEC3C.2	2.33E-89	0.951950687	0.489	0.184	5.37E-85	7
UBE2F	3.06E-89	1.121660072	0.361	0.116	7.03E-85	7
RNF213.5	3.58E-89	0.942790264	0.706	0.372	8.24E-85	7
CCL4.7	6.91E-89	0.833154349	0.829	0.421	1.59E-84	7
SLA2	9.48E-89	0.632715747	0.256	0.057	2.18E-84	7
WIPF1.7	1.05E-88	0.911740682	0.662	0.327	2.41E-84	7
CLEC2D.6	1.59E-87	0.8649964	0.648	0.289	3.66E-83	7
BCL11B	2.01E-87	0.82783921	0.402	0.126	4.62E-83	7
ARHGAP30.2	2.13E-87	0.914337379	0.514	0.21	4.90E-83	7
PRKCH.6	3.51E-87	0.84748133	0.568	0.238	8.06E-83	7
PLEK.1	4.38E-87	0.89669665	0.408	0.139	1.01E-82	7
KIAA1551.5	3.92E-86	0.910802834	0.659	0.32	9.01E-82	7
S100A10.3	2.46E-85	-1.936466656	0.276	0.627	5.65E-81	7
IKZF3.3	1.22E-84	0.858791972	0.458	0.163	2.80E-80	7
TBC1D10C.4	2.23E-83	0.856486218	0.534	0.217	5.13E-79	7
TMSB4X.3	5.05E-83	0.540333707	0.998	0.976	1.16E-78	7
ANXA1.7	6.18E-83	-2.182870238	0.1	0.49	1.42E-78	7
RPL7.4	9.33E-83	-0.795229723	0.954	0.918	2.14E-78	7
RPL17.5	1.17E-82	0.728169899	0.894	0.578	2.68E-78	7
CD82.1	1.75E-82	0.811596338	0.337	0.102	4.02E-78	7
HCST.5	2.27E-82	0.798792432	0.873	0.528	5.21E-78	7
PAG1	2.68E-82	0.892024002	0.464	0.184	6.16E-78	7
DENND2D.1	3.31E-82	0.81031825	0.399	0.133	7.60E-78	7
DZIP3	5.19E-82	0.741467401	0.284	0.075	1.19E-77	7
ITGB2.5	8.22E-82	0.820332531	0.754	0.398	1.89E-77	7
FKBP5.3	9.97E-82	-1.734318831	0.029	0.414	2.29E-77	7
LAT.2	1.24E-80	0.789470356	0.437	0.154	2.85E-76	7
TXK.2	6.86E-80	0.673621942	0.343	0.099	1.58E-75	7
RPS8.3	9.36E-80	-0.809772832	0.973	0.915	2.15E-75	7
ITM2A.7	1.87E-79	0.807700319	0.645	0.294	4.31E-75	7
NCL.3	3.08E-79	0.821864173	0.731	0.416	7.09E-75	7
RPL10.4	5.12E-79	-0.597430622	0.992	0.974	1.18E-74	7
DUSP1.4	5.36E-79	-1.475904286	0.663	0.8	1.23E-74	7
ETS1.7	4.70E-78	0.763670597	0.68	0.321	1.08E-73	7
ITGAL	5.61E-78	0.739518741	0.316	0.092	1.29E-73	7
AKNA.5	6.04E-78	0.855963529	0.557	0.246	1.39E-73	7

RPL8.1	7.18E-78	-0.840862491	0.933	0.88	1.65E-73	7
TMEM2.2	8.70E-78	0.806012745	0.449	0.165	2.00E-73	7
CORO1A.5	2.61E-77	0.762648777	0.857	0.55	6.00E-73	7
SMG1.1	2.92E-77	0.87680121	0.476	0.198	6.70E-73	7
RGS1.7	6.59E-77	0.72956139	0.956	0.684	1.51E-72	7
ASXL2	1.99E-76	0.788725776	0.281	0.078	4.57E-72	7
TYROBP.7	7.61E-76	-2.814712322	0.042	0.405	1.75E-71	7
LINC00152.5	1.97E-75	0.70082925	0.631	0.301	4.52E-71	7
LINC01116.1	3.22E-73	0.788119311	0.381	0.137	7.39E-69	7
PTPN22	4.67E-73	0.784491493	0.363	0.123	1.07E-68	7
EEF1A1.4	6.89E-73	-0.559340416	0.997	0.966	1.58E-68	7
CD247.6	7.23E-73	0.657102192	0.546	0.226	1.66E-68	7
SYTL2	2.66E-72	0.654319432	0.303	0.089	6.11E-68	7
ARID4B.6	7.91E-72	0.793461048	0.628	0.326	1.82E-67	7
CCL3.2	2.43E-71	0.774958478	0.463	0.188	5.58E-67	7
CXCR3	2.54E-71	0.635605259	0.307	0.09	5.85E-67	7
ZBTB1	4.38E-71	0.694186683	0.288	0.085	1.01E-66	7
SYNE2.6	9.33E-71	0.798169788	0.646	0.317	2.14E-66	7
MBNL1.4	3.92E-70	0.839252152	0.63	0.342	9.01E-66	7
ICOS	9.91E-70	0.718298816	0.287	0.083	2.28E-65	7
SYNRG.1	2.15E-69	0.817949746	0.423	0.168	4.95E-65	7
SON.6	3.72E-69	0.650162663	0.854	0.556	8.55E-65	7
LINC00649.1	4.72E-69	0.636229795	0.261	0.071	1.08E-64	7
RPS2.3	5.03E-69	-0.590425298	0.992	0.942	1.16E-64	7
ANKRD36C.1	9.24E-69	0.810128586	0.42	0.166	2.12E-64	7
RAB8B	9.95E-69	0.757731481	0.341	0.12	2.29E-64	7
CST3.6	1.51E-68	-2.766784151	0.065	0.401	3.47E-64	7
CTSW.3	1.66E-68	0.702710838	0.511	0.208	3.82E-64	7
STK17B.3	1.19E-67	0.734441233	0.701	0.386	2.75E-63	7
EVL.6	1.26E-67	0.700321391	0.785	0.481	2.89E-63	7
RPL35.2	2.22E-66	-0.710522671	0.964	0.894	5.10E-62	7
ATRX.4	4.65E-66	0.786233214	0.662	0.373	1.07E-61	7
PRPF4B	7.99E-65	0.744870785	0.49	0.225	1.84E-60	7
ANKRD36.1	8.95E-65	0.717906784	0.343	0.122	2.06E-60	7
GPBP1.5	1.18E-64	0.727381323	0.616	0.332	2.72E-60	7
RPS14.4	1.27E-64	-0.55996802	0.985	0.945	2.91E-60	7
TRBC1.6	1.32E-64	1.327176781	0.622	0.34	3.03E-60	7
LYZ.6	3.32E-64	-3.242944637	0.044	0.366	7.62E-60	7
PARK7.4	3.44E-64	0.645294343	0.75	0.479	7.90E-60	7
TRAF3IP3.7	5.32E-64	0.738505928	0.527	0.242	1.22E-59	7
GGA2.1	9.33E-64	0.732907898	0.317	0.111	2.14E-59	7
SPTAN1	9.40E-64	0.697711476	0.316	0.106	2.16E-59	7
RPL34.5	2.08E-63	-0.584485601	0.989	0.951	4.79E-59	7
OTULIN	6.28E-63	0.687947331	0.267	0.082	1.44E-58	7
RPL36.5	1.51E-62	-0.742865965	0.962	0.91	3.47E-58	7
GOLGA4	1.93E-62	0.831650038	0.473	0.22	4.43E-58	7
PRRC2C.4	6.34E-62	0.713368617	0.701	0.42	1.46E-57	7
PRPF38B.7	1.02E-61	0.736219968	0.704	0.422	2.34E-57	7
DOCK8.5	1.21E-61	0.756469337	0.548	0.279	2.78E-57	7
NIN.1	3.79E-61	0.797679621	0.344	0.131	8.70E-57	7
SH3KBP1.5	1.93E-60	0.705669162	0.569	0.295	4.44E-56	7
ANXA6.5	2.94E-60	0.715614117	0.508	0.237	6.76E-56	7
PTPN6	3.76E-60	0.807386526	0.384	0.16	8.63E-56	7
RPL18A.3	7.44E-60	-0.593889241	0.98	0.927	1.71E-55	7
GLUL.5	1.90E-59	-1.787673944	0.017	0.321	4.37E-55	7
TIMP1.7	2.98E-59	-2.007220429	0.024	0.328	6.85E-55	7
DDX24.5	4.56E-59	0.619271034	0.73	0.443	1.05E-54	7
RAC2.7	1.11E-58	0.635872833	0.666	0.358	2.54E-54	7

ARHGAP9.2	1.25E-58	0.690728742	0.414	0.172	2.87E-54	7
NFAT5.1	1.49E-58	0.689157535	0.316	0.114	3.42E-54	7
IRF1.5	1.79E-58	0.780524893	0.581	0.297	4.12E-54	7
BCLAF1.4	7.82E-58	0.648057793	0.599	0.322	1.80E-53	7
RPS3A.1	1.33E-57	-0.556339708	0.976	0.927	3.06E-53	7
NBEAL1.1	1.40E-57	0.608906325	0.842	0.604	3.23E-53	7
HNRNPA1L2.1	1.55E-57	0.725829645	0.366	0.15	3.57E-53	7
PTMS.2	2.38E-57	0.627362514	0.484	0.23	5.47E-53	7
TRAT1.2	4.62E-57	0.5989976	0.343	0.122	1.06E-52	7
SEPT1.3	4.77E-57	0.628742757	0.46	0.201	1.10E-52	7
SCAPER	7.03E-57	0.708596322	0.308	0.111	1.62E-52	7
HBB.3	3.14E-56	-4.10390998	0.093	0.406	7.22E-52	7
RHOH.3	4.65E-56	0.631233305	0.476	0.215	1.07E-51	7
SRSF5.4	5.39E-56	0.540984545	0.877	0.61	1.24E-51	7
RPL11.1	9.15E-56	-0.525854397	0.983	0.936	2.10E-51	7
SLA.6	9.55E-56	0.727568229	0.675	0.386	2.19E-51	7
CBLB.3	1.12E-55	0.681821589	0.417	0.177	2.57E-51	7
P2RY10	3.65E-55	0.541889396	0.272	0.086	8.38E-51	7
PNISR.5	6.22E-55	0.660773494	0.765	0.498	1.43E-50	7
NNMT.7	8.64E-55	-2.618003739	0.024	0.313	1.99E-50	7
RPL32.1	9.46E-55	-0.534759961	0.98	0.924	2.17E-50	7
HMGN1.1	1.32E-54	0.576967392	0.771	0.491	3.04E-50	7
LCP1.6	1.63E-54	0.632909207	0.687	0.39	3.74E-50	7
SH2D1A.1	2.28E-54	0.608720001	0.357	0.133	5.25E-50	7
IL7R.5	2.80E-54	-2.389347021	0.032	0.317	6.43E-50	7
RP5-1171I10.5.2	4.84E-54	0.687288976	0.338	0.13	1.11E-49	7
FYB.5	7.81E-54	0.586764712	0.757	0.445	1.79E-49	7
ARGLU1.4	1.15E-53	0.686163674	0.681	0.421	2.65E-49	7
CEBPD.5	1.28E-53	-1.692698247	0.014	0.292	2.95E-49	7
IL2RB.1	2.40E-53	0.531051303	0.263	0.083	5.53E-49	7
RGS2.6	3.53E-53	0.817638835	0.593	0.322	8.10E-49	7
MT-ND6.1	4.00E-53	0.73573999	0.413	0.19	9.19E-49	7
SYTL3.6	8.71E-53	0.631246399	0.543	0.264	2.00E-48	7
ILF3	1.74E-52	0.705294954	0.425	0.198	3.99E-48	7
IGFBP7.7	2.49E-52	-3.421564273	0.105	0.392	5.73E-48	7
RPS15.4	3.04E-52	-0.518549845	0.98	0.929	6.98E-48	7
NKTR.6	6.43E-52	0.709864646	0.598	0.334	1.48E-47	7
DRAP1.2	8.22E-52	0.626885016	0.613	0.358	1.89E-47	7
CYLD.1	1.29E-51	0.713439401	0.384	0.168	2.96E-47	7
FCER1G.7	1.66E-51	-2.125841525	0.041	0.316	3.81E-47	7
SURF4	2.41E-51	0.698232497	0.358	0.152	5.54E-47	7
KIF2A	2.53E-51	0.671553826	0.303	0.114	5.82E-47	7
SAMD9L	3.18E-51	0.671557135	0.334	0.134	7.31E-47	7
PSMB9.2	3.69E-51	0.528545128	0.747	0.471	8.48E-47	7
SHFM1.3	3.98E-51	0.53158648	0.653	0.395	9.15E-47	7
APOC1.5	6.06E-51	-2.509883156	0.024	0.296	1.39E-46	7
RPS6.2	9.10E-51	-0.545102139	0.983	0.941	2.09E-46	7
RFTN1	1.95E-50	0.644810096	0.307	0.116	4.48E-46	7
SMC4.1	2.05E-50	0.707906711	0.346	0.142	4.72E-46	7
IRF2	2.91E-50	0.680727813	0.392	0.179	6.68E-46	7
AKAP9.3	5.43E-50	0.69868741	0.566	0.331	1.25E-45	7
TUBA4A.5	1.04E-49	0.621981897	0.527	0.26	2.40E-45	7
GPX1.7	1.05E-49	-2.168819826	0.185	0.441	2.41E-45	7
RPL37A	1.31E-49	-0.514935535	0.974	0.903	3.01E-45	7
MT-ND4L.3	2.31E-49	0.652470459	0.612	0.348	5.31E-45	7
NDUFA4L2.7	2.57E-49	-2.746586664	0.033	0.301	5.91E-45	7
TPR.2	2.66E-49	0.662655978	0.537	0.297	6.10E-45	7
SAMD3.2	3.55E-49	0.646591656	0.316	0.118	8.16E-45	7

C12orf57.4	4.79E-49	0.561413282	0.63	0.359	1.10E-44	7
DGKZ	9.69E-49	0.637640276	0.355	0.149	2.23E-44	7
RPL14.3	1.01E-48	-0.640087275	0.951	0.865	2.31E-44	7
KMT2A	1.71E-48	0.694648791	0.36	0.157	3.94E-44	7
GOLGA8B.1	2.06E-48	0.581766922	0.258	0.088	4.74E-44	7
RARRES3.6	3.34E-48	0.567723824	0.672	0.398	7.68E-44	7
IGKC.4	1.18E-47	-4.253183715	0.039	0.309	2.70E-43	7
RCAN3.2	1.39E-47	0.638180009	0.352	0.146	3.20E-43	7
GCC2.5	1.55E-47	0.647703197	0.593	0.348	3.56E-43	7
CRYAB.7	1.64E-47	-2.342043063	0.015	0.271	3.76E-43	7
RASSF5.1	1.84E-47	0.583132272	0.355	0.149	4.24E-43	7
DDX17.4	3.42E-47	0.632006372	0.637	0.387	7.87E-43	7
WNK1.3	4.98E-47	0.621682447	0.47	0.238	1.14E-42	7
CLDND1.2	4.99E-47	0.780525402	0.404	0.191	1.15E-42	7
ITM2B.6	7.58E-47	-1.103193607	0.778	0.779	1.74E-42	7
NIPBL.2	1.28E-46	0.705039257	0.414	0.204	2.94E-42	7
TANK.1	1.56E-46	0.638613449	0.442	0.22	3.59E-42	7
TERF2IP.5	1.62E-46	0.523705986	0.605	0.353	3.73E-42	7
BOD1L1	4.85E-46	0.618587613	0.335	0.143	1.12E-41	7
TMEM123.4	5.39E-46	-1.09267937	0.027	0.283	1.24E-41	7
SRRM2.2	5.85E-46	0.641830561	0.566	0.33	1.34E-41	7
PSMB10.2	7.87E-46	0.645664408	0.451	0.236	1.81E-41	7
PLIN2.5	9.57E-46	-1.819447307	0.083	0.348	2.20E-41	7
C1QA.7	3.01E-45	-2.289763003	0.017	0.262	6.92E-41	7
ITM2C.2	4.46E-45	0.662008074	0.352	0.147	1.02E-40	7
ZCCHC11	4.84E-45	0.717905879	0.378	0.177	1.11E-40	7
VPS13C.2	5.00E-45	0.65657341	0.414	0.205	1.15E-40	7
ASH1L	1.03E-44	0.659311504	0.437	0.222	2.36E-40	7
IFITM3.7	2.11E-44	-2.151955228	0.135	0.39	4.84E-40	7
ITSN2.3	3.67E-44	0.704037962	0.442	0.229	8.42E-40	7
TAF7.4	4.16E-44	0.581305432	0.548	0.309	9.55E-40	7
ZBTB38.2	4.90E-44	0.587844763	0.454	0.227	1.13E-39	7
CCDC12.2	4.91E-44	0.621194774	0.437	0.219	1.13E-39	7
PSTPIP1.1	2.59E-43	0.592990834	0.305	0.123	5.95E-39	7
M6PR.2	4.25E-43	0.654876013	0.439	0.231	9.76E-39	7
IL16	4.52E-43	0.650113395	0.314	0.132	1.04E-38	7
BPTF.1	4.96E-43	0.655945296	0.414	0.208	1.14E-38	7
PTPRA	5.68E-43	0.659570011	0.355	0.163	1.30E-38	7
NSD1	7.47E-43	0.59962649	0.313	0.133	1.72E-38	7
CDK12	1.34E-42	0.58990649	0.314	0.135	3.09E-38	7
SERPINA1.6	1.77E-42	-1.846127279	0.047	0.29	4.07E-38	7
HNRNPD.2	2.43E-42	0.620835087	0.443	0.237	5.59E-38	7
SRSF9	3.31E-42	0.590243024	0.446	0.24	7.61E-38	7
JUND.4	7.43E-42	0.504941052	0.54	0.298	1.71E-37	7
ADIRF.5	2.50E-41	-2.341353572	0.021	0.252	5.74E-37	7
CCDC186	3.14E-41	0.578574076	0.323	0.142	7.22E-37	7
IFI16.3	6.21E-41	0.501468187	0.716	0.469	1.43E-36	7
HNRNPA3.1	6.47E-41	0.504271531	0.693	0.452	1.49E-36	7
HERC2	1.58E-40	0.655255314	0.29	0.123	3.63E-36	7
KMT2E.6	1.89E-40	0.543385728	0.624	0.393	4.34E-36	7
CHD3	2.23E-40	0.519744544	0.27	0.106	5.12E-36	7
PCSK7.6	4.82E-40	0.521133738	0.549	0.309	1.11E-35	7
TSPYL2.4	1.16E-39	0.813118316	0.443	0.229	2.66E-35	7
RBM25.4	1.38E-39	0.526783471	0.578	0.349	3.16E-35	7
RBM23	1.41E-39	0.568400055	0.357	0.169	3.23E-35	7
NELFCD.2	1.49E-39	0.586881331	0.308	0.134	3.43E-35	7
MT-ND5.5	1.85E-39	0.524203925	0.86	0.598	4.25E-35	7
SRSF2.6	4.73E-39	0.529396235	0.642	0.412	1.09E-34	7

SFPQ.2	4.86E-39	0.606989516	0.514	0.301	1.12E-34	7
MLLT6	8.55E-39	0.578860147	0.255	0.098	1.96E-34	7
BTG2.4	1.02E-38	0.56641648	0.58	0.346	2.34E-34	7
ASCC3	1.03E-38	0.572145299	0.255	0.101	2.36E-34	7
DDX3X.7	1.05E-38	0.506575923	0.639	0.403	2.42E-34	7
AC092580.4.2	1.36E-38	0.533729525	0.384	0.172	3.12E-34	7
ICAM3.6	1.37E-38	0.50749235	0.493	0.261	3.14E-34	7
PCM1.2	1.44E-38	0.6038741	0.445	0.243	3.31E-34	7
GNLY.7	1.46E-38	-3.069477055	0.039	0.263	3.37E-34	7
SLFN5.2	1.72E-38	0.543603438	0.402	0.196	3.95E-34	7
RPL29	2.23E-38	-0.561128755	0.936	0.851	5.13E-34	7
NFKBIA.5	2.42E-38	-1.290484173	0.338	0.569	5.56E-34	7
BTN3A2.5	2.72E-38	0.532137194	0.475	0.253	6.24E-34	7
CCNL1.6	3.29E-38	0.543973331	0.608	0.388	7.57E-34	7
BLVRA	1.24E-37	0.508397362	0.303	0.135	2.85E-33	7
KLF6.6	1.61E-37	-1.257597871	0.504	0.656	3.70E-33	7
TNFRSF1B.1	2.23E-37	0.603493689	0.42	0.224	5.13E-33	7
NOP58	2.86E-37	0.640435428	0.329	0.154	6.57E-33	7
USP34	4.69E-37	0.566568196	0.349	0.168	1.08E-32	7
TERF1	1.57E-36	0.569127012	0.332	0.157	3.61E-32	7
DDX39B	2.62E-36	0.597610629	0.282	0.123	6.02E-32	7
IGFBP3.6	2.95E-36	-2.474485347	0.039	0.254	6.78E-32	7
ARL4C.3	3.52E-36	0.66637322	0.402	0.205	8.08E-32	7
CD84.4	3.96E-36	0.571037556	0.434	0.23	9.09E-32	7
IFNGR1.4	4.01E-36	-1.114183837	0.05	0.269	9.21E-32	7
ANKRD44.4	4.64E-36	0.628070358	0.448	0.249	1.07E-31	7
PAFAH1B1	6.52E-36	0.555754301	0.347	0.172	1.50E-31	7
PPP4R3A	8.04E-36	0.564099307	0.284	0.124	1.85E-31	7
PSMA3-AS1.5	8.73E-36	0.533202708	0.472	0.265	2.01E-31	7
HNRNPM	4.92E-35	0.517899596	0.511	0.309	1.13E-30	7
SAMD9.1	5.48E-35	0.588665633	0.326	0.154	1.26E-30	7
DSTN.5	9.28E-35	-1.430104811	0.094	0.318	2.13E-30	7
SNRPN	1.18E-34	0.541648806	0.408	0.219	2.72E-30	7
TLN1.2	1.31E-34	0.544011653	0.451	0.256	3.01E-30	7
RASA2.1	1.38E-34	0.508324325	0.287	0.125	3.16E-30	7
S100A9.6	3.62E-34	-2.736642713	0.052	0.261	8.31E-30	7
MACF1.5	5.26E-34	0.563228886	0.445	0.248	1.21E-29	7
KDM5A.1	7.07E-34	0.518633787	0.366	0.188	1.62E-29	7
RBL2.1	7.25E-34	0.561199224	0.341	0.168	1.67E-29	7
BAX.1	9.14E-34	0.538336549	0.498	0.299	2.10E-29	7
G3BP2.1	1.18E-33	0.609330616	0.382	0.2	2.72E-29	7
DNAJC1.1	1.91E-33	0.51371085	0.39	0.208	4.38E-29	7
RPLP0.4	1.91E-33	-0.805988548	0.904	0.84	4.38E-29	7
RSRP1.5	5.29E-33	0.5084122	0.608	0.403	1.22E-28	7
XRN1.1	5.83E-33	0.50595063	0.293	0.136	1.34E-28	7
GNB2L1	6.61E-33	-0.606284784	0.903	0.832	1.52E-28	7
ARPC5L.4	8.49E-33	0.563394654	0.408	0.223	1.95E-28	7
SMC5	1.42E-32	0.574032097	0.276	0.126	3.26E-28	7
LBR	1.43E-32	0.519557845	0.31	0.148	3.30E-28	7
CNTRL.1	1.76E-32	0.586571991	0.316	0.154	4.05E-28	7
TAP1.4	2.87E-32	0.506307642	0.428	0.238	6.59E-28	7
NPC2.5	9.00E-32	-1.715568999	0.199	0.388	2.07E-27	7
CENPC	1.49E-31	0.500609566	0.3	0.141	3.42E-27	7
ZC3H7A.1	1.77E-31	0.552936041	0.308	0.151	4.07E-27	7
PDE7A	3.49E-31	0.525502769	0.261	0.115	8.03E-27	7
RPS5.2	3.69E-31	-0.667682759	0.906	0.838	8.48E-27	7
GTF2I.1	7.14E-31	0.510144114	0.367	0.199	1.64E-26	7
RHOB.5	8.99E-31	-1.52866052	0.156	0.358	2.07E-26	7

RPS11.1	2.09E-30	-0.523158186	0.939	0.851	4.81E-26	7
PARVG.1	2.51E-30	0.523404436	0.314	0.159	5.77E-26	7
EPC1.5	2.59E-30	0.50603079	0.449	0.261	5.96E-26	7
PARP14.1	2.73E-30	0.570483005	0.364	0.197	6.27E-26	7
PJA2	3.61E-30	0.516808616	0.311	0.158	8.29E-26	7
MYCBP2.2	4.12E-30	0.559414659	0.425	0.25	9.46E-26	7
RPL5.2	5.31E-30	-0.565540463	0.939	0.848	1.22E-25	7
BDP1	6.19E-30	0.558076181	0.344	0.181	1.42E-25	7
HBA2.5	1.03E-29	-3.616962876	0.064	0.26	2.38E-25	7
DOCK2.1	1.47E-29	0.524559116	0.266	0.124	3.37E-25	7
PSAP.7	4.45E-29	-1.48996603	0.323	0.475	1.02E-24	7
ZNF655	5.12E-29	0.501484306	0.282	0.137	1.18E-24	7
ZNF292	5.77E-29	0.53475686	0.338	0.178	1.33E-24	7
WAPL	1.25E-28	0.509061678	0.319	0.166	2.87E-24	7
APOL6	1.53E-28	0.534122664	0.299	0.148	3.51E-24	7
STAT3.4	2.59E-28	0.606291086	0.483	0.31	5.95E-24	7
SRPRA	2.91E-28	0.524356339	0.278	0.135	6.68E-24	7
GRN.6	3.09E-28	-1.242227342	0.077	0.262	7.11E-24	7
CTSB.6	4.68E-27	-1.207206808	0.126	0.311	1.07E-22	7
TMPO.1	9.23E-27	0.500084606	0.287	0.145	2.12E-22	7
ANKRD11.1	1.18E-26	0.512802377	0.387	0.223	2.72E-22	7
JUN.4	1.74E-26	-1.201979573	0.631	0.695	4.00E-22	7
SOD2.6	1.93E-26	-1.582016952	0.115	0.302	4.44E-22	7
WHSC1L1.4	2.60E-26	0.509627723	0.396	0.232	5.98E-22	7
CEBPB.5	2.73E-26	-1.130222359	0.144	0.338	6.28E-22	7
SENP6	3.47E-26	0.517186068	0.282	0.144	7.96E-22	7
GOLGB1	5.81E-26	0.563635154	0.367	0.215	1.34E-21	7
DDIT4.4	7.12E-26	-1.191219812	0.341	0.526	1.64E-21	7
SETX	7.72E-26	0.512740553	0.272	0.138	1.77E-21	7
HSPB1.6	1.15E-25	-1.542956782	0.278	0.442	2.65E-21	7
S100A11.6	1.73E-25	-1.309575702	0.534	0.593	3.98E-21	7
GPRIN3.2	2.52E-25	0.540104509	0.252	0.124	5.80E-21	7
LDHA.6	1.17E-24	-1.475255018	0.457	0.585	2.69E-20	7
REST	3.08E-24	0.512321399	0.259	0.131	7.07E-20	7
LGALS3.3	9.27E-24	-0.968327497	0.082	0.254	2.13E-19	7
RPL22.3	2.82E-23	-0.582158671	0.886	0.807	6.48E-19	7
FOSB.3	1.20E-22	-0.920441671	0.255	0.434	2.75E-18	7
PPP1R10.1	1.84E-22	0.510470406	0.308	0.175	4.23E-18	7
GAPDH.5	1.59E-20	-0.848861466	0.891	0.865	3.66E-16	7
GLIPR1.4	6.91E-20	-0.73045617	0.144	0.307	1.59E-15	7
YBX1.3	1.98E-19	-0.789835556	0.666	0.67	4.54E-15	7
SAT1.6	2.30E-19	-1.250252998	0.592	0.633	5.29E-15	7
C10orf54.5	2.94E-19	-0.791427296	0.135	0.289	6.77E-15	7
PRDX2.4	5.47E-18	-0.808183112	0.12	0.272	1.26E-13	7
EEF2.1	6.21E-17	-0.665035734	0.677	0.686	1.43E-12	7
AIF1.7	1.23E-16	-1.521488241	0.141	0.265	2.83E-12	7
TOMM7.3	5.05E-16	-0.555331364	0.809	0.752	1.16E-11	7
ISG20.5	1.05E-15	-0.739540938	0.226	0.368	2.42E-11	7
ANXA2.3	1.13E-15	-1.055320854	0.281	0.399	2.60E-11	7
PIK3IP1.7	1.17E-15	-0.804689003	0.232	0.364	2.68E-11	7
RGCC.6	1.98E-15	-1.263734231	0.179	0.313	4.56E-11	7
S100A6.3	2.75E-15	-0.874808225	0.819	0.764	6.33E-11	7
HLA-DRA.7	1.21E-14	-1.839368726	0.64	0.674	2.79E-10	7
TAGLN2.5	2.19E-14	-0.745686748	0.222	0.363	5.03E-10	7
ZFAND5.5	2.87E-14	-0.771855312	0.181	0.314	6.61E-10	7
APLP2.5	8.93E-14	-0.763122938	0.137	0.261	2.05E-09	7
RNASET2.7	4.30E-13	-1.033750984	0.264	0.366	9.87E-09	7
EIF3E.3	2.91E-12	-0.664790684	0.473	0.548	6.68E-08	7

RAC1.5	4.21E-12	-0.893073095	0.384	0.451	9.67E-08	7
CITED2.6	4.60E-12	-0.754972903	0.155	0.273	1.06E-07	7
HLA-DRB5.7	5.09E-12	-0.510462454	0.561	0.364	1.17E-07	7
GLTSCR2.3	5.76E-12	-0.657602242	0.534	0.59	1.32E-07	7
RTN4.5	1.26E-11	-0.695954363	0.241	0.356	2.91E-07	7
PPP1R15A.4	1.94E-11	-0.652007392	0.287	0.409	4.46E-07	7
TPI1.5	1.36E-10	-0.957411904	0.527	0.566	3.13E-06	7
FOXP1.2	2.01E-10	-0.554839423	0.152	0.263	4.62E-06	7
TUBB.5	2.80E-10	-0.751744832	0.241	0.353	6.43E-06	7
HSP90AA1.4	4.78E-10	-0.944761577	0.678	0.653	1.10E-05	7
TUBA1B.5	5.20E-10	-0.896393128	0.332	0.418	1.19E-05	7
CYTIP.5	8.48E-10	-0.750517657	0.416	0.478	1.95E-05	7
LAPTM4A.6	9.56E-10	-0.760437966	0.253	0.357	2.20E-05	7
YWHAH.5	1.57E-09	-0.947436784	0.196	0.287	3.61E-05	7
PPP1CB.4	3.31E-09	-0.522558217	0.187	0.302	7.61E-05	7
CD63.6	2.73E-08	-0.867273517	0.514	0.52	0.000627428	7
GDI2.6	3.69E-08	-0.54872814	0.217	0.317	0.000849132	7
ZFP36L2.6	2.94E-07	-0.737118716	0.774	0.695	0.00676614	7
GPX4.5	1.76E-06	-0.660697117	0.414	0.456	0.040524165	7
ESM1	0	4.036877744	0.693	0.099	0	8
SPARCL1	0	3.443117763	0.718	0.139	0	8
IGFBP7.8	4.62E-274	3.043237871	0.871	0.357	1.06E-269	8
ENPP2	1.68E-257	3.548280277	0.543	0.107	3.86E-253	8
SPARC	5.09E-247	3.104212383	0.638	0.162	1.17E-242	8
GNG11	9.10E-214	3.318953156	0.529	0.127	2.09E-209	8
PLVAP	1.06E-191	2.937208489	0.421	0.081	2.44E-187	8
SLC9A3R2	1.17E-191	3.101070108	0.344	0.053	2.69E-187	8
RAMP2	2.00E-179	2.832184474	0.337	0.054	4.59E-175	8
CD59.2	5.49E-172	3.109774928	0.608	0.216	1.26E-167	8
IFI27	1.98E-148	2.970184009	0.482	0.14	4.55E-144	8
SDPR	3.40E-147	2.785642917	0.344	0.068	7.82E-143	8
RBP7	3.10E-143	3.269496331	0.341	0.069	7.12E-139	8
IFITM3.8	5.78E-139	2.679718531	0.693	0.364	1.33E-134	8
IGFBP3.7	1.07E-136	2.995807378	0.583	0.229	2.46E-132	8
PTPRC.8	1.37E-127	-2.018734489	0.068	0.641	3.14E-123	8
CLEC14A	1.39E-125	2.439074948	0.27	0.048	3.20E-121	8
PLPP1	4.86E-123	2.683011207	0.356	0.086	1.12E-118	8
CXCR4.6	5.78E-117	-1.713337719	0.244	0.799	1.33E-112	8
ZFP36L2.7	2.14E-116	-1.837575178	0.157	0.723	4.92E-112	8
ADGRL4	6.66E-112	2.303995807	0.255	0.047	1.53E-107	8
RGS1.8	3.21E-111	-1.866557915	0.179	0.72	7.38E-107	8
PRSS23	1.26E-109	2.692577075	0.371	0.103	2.89E-105	8
FLT1	2.20E-106	2.189679117	0.287	0.061	5.05E-102	8
MT-CO1.3	2.74E-106	-1.085452758	0.629	0.939	6.29E-102	8
RNASE1	5.32E-105	2.612786464	0.326	0.082	1.22E-100	8
TIMP3	9.93E-99	2.366769911	0.324	0.084	2.28E-94	8
CORO1A.6	1.48E-96	-1.404867103	0.083	0.586	3.40E-92	8
LAPTM5.6	2.51E-96	-1.350751614	0.083	0.589	5.78E-92	8
HCST.6	3.41E-96	-1.451720515	0.072	0.565	7.83E-92	8
CCL5.7	3.10E-95	-2.152418524	0.1	0.582	7.12E-91	8
TSC22D1	7.49E-94	2.83900456	0.442	0.172	1.72E-89	8
MGP	2.91E-92	2.379158202	0.409	0.139	6.68E-88	8
CYBA.4	2.19E-91	-1.036448121	0.175	0.71	5.04E-87	8
EVL.7	1.47E-90	-1.452914169	0.052	0.515	3.38E-86	8
CD44.4	7.98E-90	-1.498502879	0.052	0.511	1.83E-85	8
CD52.5	3.16E-89	-1.68403242	0.097	0.569	7.26E-85	8
BTG1.7	3.24E-88	-1.271816442	0.401	0.862	7.45E-84	8
FYB.6	5.07E-88	-1.665355927	0.035	0.479	1.17E-83	8

PABPC1.3	6.69E-88	-0.895000095	0.162	0.685	1.54E-83	8
CD69.8	1.12E-84	-1.953584937	0.093	0.548	2.57E-80	8
SRSF7.6	1.12E-84	-1.306422092	0.095	0.565	2.58E-80	8
CD37.5	4.80E-84	-1.34971087	0.042	0.482	1.10E-79	8
STK4.5	3.97E-82	-1.478155026	0.057	0.491	9.12E-78	8
RPS29.5	8.49E-80	-0.906574037	0.634	0.934	1.95E-75	8
RPL17.6	1.03E-79	-0.988550796	0.134	0.614	2.37E-75	8
SPRY1	1.02E-78	2.507906943	0.322	0.102	2.35E-74	8
S100A4.6	2.21E-77	-1.180171433	0.235	0.72	5.08E-73	8
LSP1.7	2.26E-77	-1.447682186	0.042	0.452	5.19E-73	8
CD53.4	3.90E-77	-1.09388551	0.062	0.489	8.96E-73	8
CD2.7	6.56E-77	-1.82941191	0.063	0.474	1.51E-72	8
CELF2.6	5.03E-76	-1.382456684	0.038	0.443	1.16E-71	8
SRSF5.5	5.27E-76	-0.730511295	0.155	0.643	1.21E-71	8
RPS27.4	1.82E-74	-0.719405933	0.885	0.979	4.19E-70	8
ITGB2.6	4.26E-74	-1.451527454	0.037	0.432	9.79E-70	8
PNISR.6	1.52E-73	-0.858436682	0.088	0.529	3.49E-69	8
CYTIP.6	7.92E-73	-1.100938421	0.075	0.493	1.82E-68	8
KLF6.7	8.15E-73	-1.031500673	0.197	0.669	1.87E-68	8
SLA.7	8.67E-73	-1.460081105	0.028	0.416	1.99E-68	8
TNFAIP3.8	4.93E-72	-1.415992805	0.06	0.464	1.13E-67	8
RGCC.7	4.90E-71	2.674230875	0.519	0.298	1.13E-66	8
CRIP2	1.19E-70	2.406272617	0.295	0.094	2.74E-66	8
SARAF.3	5.69E-70	-0.57885673	0.2	0.699	1.31E-65	8
TRAC.8	3.34E-69	-1.720574945	0.06	0.449	7.68E-65	8
OAZ1.5	1.03E-68	-0.674515811	0.25	0.757	2.37E-64	8
RARRES3.7	1.19E-68	-1.265327351	0.043	0.427	2.74E-64	8
LCP1.7	2.94E-68	-1.146966952	0.042	0.42	6.75E-64	8
HMGB2.4	2.95E-68	-0.770433853	0.082	0.494	6.79E-64	8
GSN.2	4.30E-68	2.451995363	0.446	0.215	9.89E-64	8
STK17B.4	7.18E-68	-1.164160581	0.038	0.417	1.65E-63	8
CD3D.8	1.10E-67	-1.312218109	0.09	0.492	2.53E-63	8
GZMA.7	1.47E-67	-1.73074771	0.055	0.435	3.38E-63	8
MT-ND5.6	2.82E-66	-0.646417135	0.165	0.63	6.48E-62	8
RPSA.3	3.17E-66	-0.809152262	0.322	0.826	7.28E-62	8
NKG7.8	9.11E-66	-2.156374026	0.068	0.443	2.09E-61	8
JUN.5	1.08E-65	-1.076499629	0.252	0.711	2.48E-61	8
HES1	1.10E-65	2.581980869	0.26	0.077	2.53E-61	8
CST7.7	1.20E-65	-1.800454322	0.04	0.405	2.77E-61	8
GPSM3.5	5.22E-65	-0.911563099	0.038	0.406	1.20E-60	8
DUSP2.7	5.27E-65	-1.54606327	0.065	0.448	1.21E-60	8
PNRC1.3	5.73E-65	-0.614168001	0.18	0.655	1.32E-60	8
NBEAL1.2	8.23E-65	-0.560226241	0.174	0.635	1.89E-60	8
CD3E.7	2.62E-64	-1.371481241	0.047	0.413	6.02E-60	8
AC090498.1.5	4.17E-64	-0.923196667	0.109	0.518	9.58E-60	8
ELF1.6	1.34E-63	-0.70961208	0.068	0.457	3.07E-59	8
CD48.7	3.14E-63	-1.075021017	0.035	0.39	7.22E-59	8
MCL1.4	3.87E-63	-0.549074287	0.114	0.532	8.89E-59	8
IDS.5	2.10E-62	-1.191210127	0.033	0.388	4.84E-58	8
DNAJB1.6	2.35E-62	-1.304052673	0.065	0.436	5.40E-58	8
RPL41.5	1.31E-61	-0.612645606	0.838	0.982	3.02E-57	8
ID3	1.33E-61	2.323456051	0.339	0.131	3.06E-57	8
PRRC2C.5	1.65E-61	-0.770977769	0.068	0.45	3.79E-57	8
CCL4.8	1.94E-61	-2.220421869	0.088	0.456	4.45E-57	8
EPAS1	2.50E-61	2.163815638	0.304	0.107	5.74E-57	8
IL2RG.8	2.61E-61	-1.275779408	0.037	0.387	6.01E-57	8
EEF1B2.2	2.83E-61	-0.591629858	0.209	0.682	6.51E-57	8
RP11-347P5.1.7	7.84E-61	-1.513089219	0.042	0.39	1.80E-56	8

MT-CO3.4	2.11E-60	-0.756524338	0.508	0.896	4.85E-56	8
TRBC2.7	3.02E-60	-1.492720788	0.043	0.392	6.95E-56	8
RNF213.6	4.35E-60	-1.083706993	0.047	0.402	9.99E-56	8
RAC2.8	4.46E-60	-1.094160545	0.04	0.387	1.03E-55	8
CAV1.1	6.38E-60	2.335328308	0.332	0.129	1.47E-55	8
MT-ND2.4	1.41E-59	-0.68734593	0.442	0.885	3.24E-55	8
HSP90AA1.5	2.81E-59	-0.732682947	0.215	0.673	6.47E-55	8
LITAF.5	3.97E-59	-1.076429955	0.037	0.382	9.12E-55	8
PPDPF.4	1.97E-58	-0.544162642	0.21	0.679	4.54E-54	8
UCP2.6	2.24E-58	-1.130092778	0.028	0.361	5.14E-54	8
ZFAS1.6	2.51E-58	-0.723685672	0.109	0.501	5.76E-54	8
MT-ND3.6	2.54E-58	-0.726572434	0.402	0.849	5.85E-54	8
ACAP1.8	1.15E-57	-1.387815573	0.023	0.347	2.64E-53	8
YWHAZ.6	1.76E-57	-0.554713548	0.114	0.511	4.04E-53	8
AKAP13.7	2.68E-57	-0.645440707	0.078	0.448	6.15E-53	8
FKBP1A.6	3.88E-57	2.481684523	0.551	0.407	8.91E-53	8
EVI2B.3	6.51E-57	-1.15362812	0.025	0.349	1.50E-52	8
AAK1.8	1.92E-56	-1.032455189	0.047	0.385	4.42E-52	8
FNBP1.7	4.23E-56	-0.847878915	0.047	0.382	9.72E-52	8
WIPF1.8	7.88E-56	-1.095458382	0.032	0.357	1.81E-51	8
RPS21.4	8.95E-56	-0.710712883	0.409	0.882	2.06E-51	8
EMP3.3	1.97E-55	-0.77102223	0.057	0.405	4.52E-51	8
FAM49B.3	4.02E-55	-1.013613314	0.032	0.354	9.25E-51	8
SNHG8.3	4.50E-55	-0.735912108	0.083	0.45	1.03E-50	8
RSRP1.6	5.47E-55	-0.816749003	0.073	0.428	1.26E-50	8
GZMK.8	5.76E-55	-2.031257246	0.052	0.379	1.32E-50	8
CIB1	1.61E-54	-0.683190333	0.06	0.402	3.69E-50	8
ZFP36.5	2.00E-54	-0.776990881	0.25	0.682	4.59E-50	8
ALOX5AP.5	5.73E-54	-1.530411553	0.03	0.342	1.32E-49	8
PPP1R15A.5	1.70E-53	-0.924457375	0.072	0.418	3.90E-49	8
RNASET2.8	2.02E-53	-1.219732804	0.048	0.375	4.64E-49	8
CCNL1.7	2.95E-53	-0.661456376	0.068	0.414	6.78E-49	8
POLR2J3.5	2.98E-53	-0.715825972	0.065	0.409	6.85E-49	8
STK17A.7	3.14E-53	-1.010990977	0.053	0.38	7.21E-49	8
NFKBIA.6	3.24E-53	-0.61899441	0.165	0.575	7.45E-49	8
INSR.1	6.66E-53	2.405123685	0.319	0.129	1.53E-48	8
CNN3	7.84E-53	2.270478837	0.277	0.1	1.80E-48	8
FOS.6	8.10E-53	-0.958943419	0.285	0.699	1.86E-48	8
RGS2.7	8.79E-53	-1.346251167	0.032	0.349	2.02E-48	8
MT-ND1.3	1.82E-52	-0.641162348	0.342	0.807	4.19E-48	8
PIK3IP1.8	2.03E-52	-0.955355574	0.047	0.372	4.67E-48	8
PPP2R5C.6	2.95E-52	-0.990882615	0.07	0.404	6.77E-48	8
COTL1.3	5.98E-52	-0.844346107	0.097	0.449	1.37E-47	8
BTG2.5	9.97E-52	-0.943151941	0.048	0.371	2.29E-47	8
RBM25.5	1.07E-51	-0.745440138	0.05	0.373	2.46E-47	8
HNRNPA1.2	1.65E-51	-0.513871191	0.309	0.798	3.78E-47	8
LIMD2.7	1.74E-51	-1.136933786	0.023	0.323	4.00E-47	8
ADGRE5.7	5.87E-51	-1.229223798	0.018	0.312	1.35E-46	8
RPS2.4	9.43E-51	-0.587368376	0.661	0.957	2.17E-46	8
IL32.8	1.88E-50	-0.868108498	0.207	0.594	4.33E-46	8
CTSS.6	4.44E-50	-1.150600398	0.057	0.376	1.02E-45	8
FOSB.4	5.95E-50	-0.788793289	0.097	0.44	1.37E-45	8
ARF6.4	6.23E-50	-0.707396173	0.065	0.393	1.43E-45	8
REL.4	6.77E-50	-0.976367412	0.033	0.335	1.56E-45	8
JUNB.7	1.86E-49	-0.802550504	0.332	0.741	4.28E-45	8
KIAA1551.6	2.05E-49	-0.969241159	0.042	0.349	4.70E-45	8
SAMSN1.3	2.17E-49	-1.034215421	0.035	0.335	4.99E-45	8
RPS15A.4	2.71E-49	-0.585476089	0.681	0.945	6.23E-45	8

PIK3R1.5	3.37E-49	-1.113614017	0.027	0.321	7.74E-45	8
DEK.2	1.46E-48	-0.621161878	0.06	0.378	3.36E-44	8
RPL36A.4	1.99E-48	-0.532859863	0.344	0.822	4.56E-44	8
TRBC1.7	4.82E-48	-1.467047087	0.06	0.366	1.11E-43	8
MT-ND4L.4	1.33E-47	-0.616016582	0.058	0.374	3.05E-43	8
STAT3.5	4.36E-47	-0.825087654	0.035	0.331	1.00E-42	8
TAF7.5	1.31E-46	-0.718196761	0.04	0.333	3.00E-42	8
SH3KBP1.6	1.48E-46	-0.682764665	0.033	0.32	3.40E-42	8
RBMX.2	1.56E-46	-0.5319371	0.073	0.393	3.58E-42	8
CD7.5	2.15E-46	-1.245500434	0.028	0.312	4.94E-42	8
CLEC2D.7	2.78E-46	-1.153491691	0.035	0.318	6.39E-42	8
RGS10.4	8.27E-46	-1.053027765	0.028	0.307	1.90E-41	8
TAF1D.2	1.04E-45	-0.859651078	0.042	0.331	2.39E-41	8
RPL28.5	1.42E-45	-0.54083958	0.684	0.944	3.26E-41	8
MT-CYB.5	1.55E-45	-0.593775415	0.387	0.823	3.56E-41	8
NR4A2.5	2.28E-45	-1.279736545	0.02	0.288	5.23E-41	8
ROCK1.3	3.00E-45	-0.601001958	0.048	0.344	6.90E-41	8
RBPJ.4	5.01E-45	-0.71896785	0.043	0.334	1.15E-40	8
GLIPR1.5	5.71E-45	-0.827959023	0.032	0.311	1.31E-40	8
CTSC.6	8.55E-45	-0.648509132	0.06	0.359	1.96E-40	8
CRIP1.6	8.73E-45	-1.22566402	0.03	0.305	2.01E-40	8
SYTL3.7	9.54E-45	-1.177776828	0.02	0.288	2.19E-40	8
HCLS1.3	1.07E-44	-0.697925643	0.047	0.335	2.46E-40	8
SMAP2.4	1.22E-44	-0.875867305	0.032	0.31	2.80E-40	8
DOCK8.6	1.28E-44	-0.929626072	0.03	0.303	2.94E-40	8
HSPE1.4	1.41E-44	-0.534265025	0.083	0.397	3.25E-40	8
EVI2A.4	1.59E-44	-0.813085896	0.02	0.292	3.65E-40	8
CD3G.7	3.13E-44	-1.221880315	0.033	0.305	7.19E-40	8
LTB.7	5.57E-44	-1.454369021	0.038	0.313	1.28E-39	8
SRRM2.3	6.72E-44	-0.550382605	0.055	0.354	1.54E-39	8
APOBEC3G.6	1.04E-43	-1.189892201	0.02	0.283	2.38E-39	8
BCLAF1.5	1.40E-43	-0.502998904	0.055	0.348	3.23E-39	8
ARID4B.7	1.51E-43	-0.663639263	0.06	0.353	3.48E-39	8
MT-ND4.3	9.48E-43	-0.559453692	0.503	0.891	2.18E-38	8
VMP1.5	1.15E-42	-0.784922246	0.05	0.332	2.65E-38	8
TRAF3IP3.8	1.71E-42	-1.01413794	0.013	0.267	3.94E-38	8
GLUL.6	2.74E-42	-0.899129217	0.042	0.318	6.30E-38	8
BIRC3.6	7.74E-42	-1.262379268	0.025	0.283	1.78E-37	8
LCK.8	1.40E-41	-0.852163776	0.035	0.299	3.21E-37	8
ANXA1.8	1.75E-41	-0.724905082	0.15	0.486	4.02E-37	8
H3F3B.4	2.05E-41	-0.544676761	0.397	0.854	4.72E-37	8
EIF3A.4	4.03E-41	-0.59102107	0.04	0.31	9.26E-37	8
EZR.2	8.89E-41	-0.540539235	0.055	0.337	2.04E-36	8
TYROBP.8	9.68E-41	-1.309411632	0.11	0.4	2.22E-36	8
MYO1F.4	1.35E-40	-0.796632766	0.027	0.282	3.10E-36	8
GLRX.3	1.54E-40	-0.625108641	0.042	0.309	3.55E-36	8
ZEB2.4	1.57E-40	-1.027561889	0.008	0.251	3.60E-36	8
HLA-DQB1.6	2.22E-40	-1.019908407	0.105	0.405	5.10E-36	8
MT2A.8	2.49E-40	-1.142094677	0.289	0.676	5.72E-36	8
RPL10A.5	2.58E-40	-0.511072755	0.447	0.884	5.93E-36	8
PCSK7.7	4.99E-40	-0.796836496	0.058	0.332	1.15E-35	8
SERPINA1.7	5.11E-40	-1.375181013	0.032	0.289	1.17E-35	8
USP15.2	8.64E-40	-0.785684083	0.023	0.273	1.99E-35	8
TYMP.6	2.05E-39	-0.684224191	0.043	0.307	4.71E-35	8
SEPT6.5	2.46E-39	-0.667697059	0.033	0.288	5.65E-35	8
RPL23A.5	2.77E-39	-0.533886383	0.688	0.941	6.36E-35	8
EMB.6	6.04E-39	-0.707432467	0.03	0.28	1.39E-34	8
IKZF1.8	8.50E-39	-0.927699938	0.017	0.254	1.95E-34	8

CD27.7	9.17E-39	-1.32498442	0.032	0.278	2.11E-34	8
SMCHD1.3	1.32E-38	-0.548330322	0.065	0.339	3.04E-34	8
IL7R.6	1.47E-38	-1.500313841	0.055	0.314	3.38E-34	8
PLP2.4	1.71E-38	-0.56826414	0.045	0.306	3.93E-34	8
DHRS7.3	2.52E-38	-0.531728476	0.033	0.286	5.80E-34	8
HLA-DQA1.5	2.60E-38	-1.279555339	0.083	0.355	5.97E-34	8
CD96.8	2.81E-38	-0.946579165	0.02	0.259	6.47E-34	8
RNF149.5	2.90E-38	-0.624527035	0.032	0.28	6.66E-34	8
ATM.4	1.15E-37	-0.856308292	0.022	0.259	2.63E-33	8
HLA-DPB1.7	1.63E-37	-1.067543629	0.289	0.648	3.75E-33	8
CD247.7	2.45E-37	-0.979247548	0.018	0.251	5.63E-33	8
CEBPB.6	7.84E-37	-0.615279031	0.07	0.34	1.80E-32	8
ANKRD44.5	8.66E-37	-0.78433694	0.03	0.268	1.99E-32	8
AKNA.6	1.39E-36	-0.782566791	0.033	0.271	3.19E-32	8
TXNIP.7	1.92E-36	-0.602509477	0.411	0.779	4.41E-32	8
IL10RA.5	2.93E-36	-0.745906596	0.032	0.269	6.74E-32	8
BIN2.6	4.09E-36	-0.769700685	0.027	0.259	9.40E-32	8
BBX.3	6.00E-36	-0.729061494	0.023	0.254	1.38E-31	8
HSPD1.5	6.96E-36	-0.579447082	0.053	0.304	1.60E-31	8
MYCBP2.3	7.15E-36	-0.62579211	0.032	0.268	1.64E-31	8
PRDM1.5	8.62E-36	-0.631533607	0.052	0.302	1.98E-31	8
TRIM22.4	1.05E-35	-0.583152586	0.027	0.258	2.41E-31	8
RCSD1.6	1.23E-35	-0.577788993	0.027	0.259	2.83E-31	8
CCNH.6	1.52E-35	-0.572867036	0.048	0.295	3.49E-31	8
CMC1.7	1.65E-35	-1.258113436	0.052	0.295	3.79E-31	8
FCER1G.8	2.46E-35	-1.139438504	0.063	0.314	5.66E-31	8
MGEA5.6	2.78E-35	-0.604696771	0.037	0.276	6.38E-31	8
CHCHD10.6	3.01E-35	-0.50196627	0.025	0.256	6.91E-31	8
RNF19A.7	3.69E-35	-0.708953768	0.047	0.287	8.49E-31	8
ARID5B.3	8.21E-35	-0.856672418	0.033	0.265	1.89E-30	8
PSMA3-AS1.6	8.49E-35	-0.633703166	0.043	0.285	1.95E-30	8
HLA-DPA1.8	1.68E-34	-0.976163216	0.25	0.585	3.86E-30	8
RPS8.4	1.94E-34	-0.511385946	0.586	0.933	4.46E-30	8
TUBA4A.6	2.71E-34	-0.831985316	0.047	0.283	6.23E-30	8
LDHA.7	4.06E-34	-0.510100111	0.239	0.594	9.33E-30	8
PTMA.2	2.85E-33	0.75082223	0.825	0.921	6.56E-29	8
PYCARD.4	4.79E-33	-0.532374615	0.033	0.256	1.10E-28	8
ANXA6.6	5.27E-33	-0.512881788	0.035	0.259	1.21E-28	8
GBP2.2	9.06E-33	-0.52942971	0.043	0.272	2.08E-28	8
TSTD1.3	1.81E-32	-0.625980796	0.035	0.256	4.15E-28	8
OCIAD2.5	1.79E-31	-0.647698272	0.047	0.27	4.10E-27	8
FOXP1.3	2.73E-31	-0.585321581	0.043	0.267	6.28E-27	8
MT1X.4	4.08E-31	-1.829635403	0.063	0.293	9.37E-27	8
LYZ.7	2.06E-30	-1.573269109	0.117	0.362	4.73E-26	8
CITED2.7	3.11E-30	-0.73893603	0.057	0.277	7.14E-26	8
YWHAH.6	2.29E-29	2.305196211	0.397	0.278	5.26E-25	8
PECAM1.1	5.85E-29	2.141009557	0.331	0.195	1.34E-24	8
APP.1	3.91E-27	2.174387713	0.304	0.173	8.98E-23	8
AIF1.8	4.05E-27	-0.993050425	0.062	0.268	9.31E-23	8
NNMT.8	8.49E-27	-1.101818729	0.088	0.309	1.95E-22	8
SEPW1.8	1.85E-26	2.188150463	0.437	0.356	4.26E-22	8
CRYAB.8	2.11E-26	-1.171438834	0.063	0.268	4.84E-22	8
VIM.6	8.14E-25	0.902921101	0.701	0.774	1.87E-20	8
NDUFA4L2.8	9.18E-25	-1.336631418	0.093	0.297	2.11E-20	8
HBB.4	1.69E-24	-1.306286441	0.149	0.402	3.87E-20	8
SOD2.7	1.29E-22	-0.519360874	0.097	0.302	2.96E-18	8
PSMA1.1	3.53E-21	0.503413508	0.122	0.349	8.11E-17	8
GNLY.8	8.69E-21	-1.625685742	0.083	0.26	2.00E-16	8

COX7A2.1	7.88E-20	0.532203025	0.25	0.575	1.81E-15	8
C9orf16.1	1.05E-19	0.555451003	0.134	0.359	2.42E-15	8
TPM3.2	3.65E-19	0.514519299	0.195	0.465	8.39E-15	8
EIF2S2	9.98E-19	0.523042878	0.115	0.319	2.29E-14	8
HBA2.6	1.13E-18	-1.670572839	0.083	0.258	2.59E-14	8
APOC1.6	1.25E-18	-0.760102482	0.109	0.291	2.86E-14	8
ATP6V1F.6	1.93E-18	0.512288407	0.14	0.359	4.44E-14	8
SNRPB2.1	5.10E-18	0.794987582	0.135	0.349	1.17E-13	8
S100A9.7	1.65E-17	-1.431368419	0.09	0.258	3.79E-13	8
AP2M1	3.26E-17	0.503513574	0.129	0.332	7.48E-13	8
NDUFB10.4	7.10E-17	0.553522695	0.142	0.353	1.63E-12	8
C1QA.8	7.44E-17	-0.673773944	0.095	0.257	1.71E-12	8
PSMB3.3	8.26E-17	0.517792709	0.147	0.358	1.90E-12	8
ERH.1	1.10E-16	0.531839941	0.154	0.37	2.53E-12	8
ATP5I.1	1.85E-16	0.576546925	0.245	0.532	4.26E-12	8
SUMO1.1	5.36E-16	0.525413384	0.167	0.394	1.23E-11	8
SLC25A3.2	7.20E-16	0.605092133	0.184	0.422	1.65E-11	8
HLA-DRB1.8	8.15E-16	-0.693566728	0.349	0.601	1.87E-11	8
TMEM59.1	9.44E-16	0.657036972	0.222	0.488	2.17E-11	8
WASF2.2	9.74E-16	0.615041989	0.137	0.339	2.24E-11	8
BRK1.1	1.16E-15	0.595013649	0.199	0.449	2.66E-11	8
ITM2B.7	1.99E-15	1.002005448	0.636	0.786	4.58E-11	8
PSMB1.1	3.83E-15	0.673082228	0.195	0.434	8.81E-11	8
OSTC.1	4.45E-15	0.527088545	0.102	0.272	1.02E-10	8
MORF4L1	7.01E-15	0.633410767	0.257	0.548	1.61E-10	8
PSMD8.2	7.40E-15	0.554384925	0.132	0.318	1.70E-10	8
NDUFA11.2	1.05E-14	0.651342813	0.179	0.404	2.40E-10	8
CD9.2	2.13E-14	1.946590086	0.299	0.22	4.89E-10	8
PSMB7.2	2.23E-14	0.513335676	0.114	0.284	5.12E-10	8
RAB7A.3	2.25E-14	0.632947808	0.137	0.323	5.17E-10	8
IGKC.5	4.72E-14	-2.507020314	0.13	0.303	1.08E-09	8
LSM3.1	5.21E-14	0.602980655	0.102	0.264	1.20E-09	8
SNRPG.2	6.31E-14	0.656142156	0.164	0.366	1.45E-09	8
CSTB.4	7.07E-14	0.566045898	0.187	0.407	1.62E-09	8
TMEM219.5	7.76E-14	0.531890814	0.112	0.278	1.78E-09	8
NDUFB4.5	8.03E-14	0.573698711	0.195	0.42	1.85E-09	8
SRP14.2	9.23E-14	1.332083084	0.566	0.722	2.12E-09	8
PSMD4	1.10E-13	0.573801709	0.1	0.26	2.54E-09	8
PSMA4.1	1.34E-13	0.65670133	0.145	0.332	3.09E-09	8
NFE2L2.5	1.58E-13	0.573755165	0.109	0.27	3.63E-09	8
COX6A1.2	1.60E-13	0.773257234	0.254	0.53	3.68E-09	8
UQCRQ.6	1.98E-13	0.559016525	0.229	0.472	4.56E-09	8
GYPC.5	2.11E-13	0.581233466	0.137	0.314	4.84E-09	8
ATP1B3.5	2.26E-13	0.619499359	0.112	0.275	5.20E-09	8
DBI.1	3.51E-13	0.676890417	0.204	0.433	8.07E-09	8
A2M.1	4.25E-13	1.659450391	0.272	0.193	9.76E-09	8
NDUFB7.1	5.57E-13	0.665819891	0.154	0.343	1.28E-08	8
IFITM2.5	5.89E-13	1.5181708	0.457	0.476	1.35E-08	8
YWHAQ.3	1.10E-12	0.551550882	0.165	0.357	2.52E-08	8
PDIA6.2	1.26E-12	0.630249844	0.139	0.313	2.89E-08	8
CLTA.4	1.27E-12	0.703084774	0.129	0.298	2.92E-08	8
ROMO1.3	1.57E-12	0.586298868	0.142	0.319	3.60E-08	8
BSG.2	1.60E-12	0.560518619	0.154	0.336	3.68E-08	8
MRFAP1	1.83E-12	0.643912055	0.144	0.317	4.20E-08	8
ARL6IP1.2	2.70E-12	0.762954689	0.149	0.328	6.20E-08	8
PSMB6	2.82E-12	0.695735546	0.122	0.284	6.49E-08	8
SEPT2.2	3.26E-12	0.61206541	0.114	0.268	7.49E-08	8
SHFM1.4	3.30E-12	0.72960991	0.197	0.416	7.59E-08	8

PSMC5.1	3.35E-12	0.58351425	0.11	0.264	7.70E-08	8
FIS1.1	3.58E-12	0.711767917	0.142	0.316	8.22E-08	8
SEP15.1	5.70E-12	0.803442945	0.149	0.327	1.31E-07	8
MRPL51.3	6.21E-12	0.606200703	0.117	0.272	1.43E-07	8
PPP1CB.5	8.07E-12	0.526564025	0.137	0.303	1.85E-07	8
RHOB.6	8.64E-12	1.532829235	0.387	0.347	1.99E-07	8
NDUFC2.2	1.06E-11	0.746272327	0.137	0.302	2.43E-07	8
COX6C.2	1.06E-11	0.74229139	0.287	0.58	2.43E-07	8
METTL9.2	1.07E-11	0.560872449	0.129	0.288	2.46E-07	8
DYNLRB1.3	1.22E-11	0.75851613	0.152	0.329	2.80E-07	8
ATP5J.4	1.44E-11	0.746494038	0.197	0.403	3.32E-07	8
GNB2.1	1.59E-11	0.749557418	0.115	0.27	3.66E-07	8
AP2S1.6	2.11E-11	0.653872949	0.147	0.317	4.84E-07	8
APOE.7	2.23E-11	-0.848136319	0.254	0.446	5.13E-07	8
CD99.5	3.22E-11	0.736987706	0.239	0.48	7.41E-07	8
PRDX2.5	3.31E-11	0.522181088	0.122	0.271	7.62E-07	8
PRDX5.2	3.35E-11	0.821827012	0.172	0.359	7.71E-07	8
GNG5.4	8.90E-11	0.797456786	0.175	0.36	2.05E-06	8
GABARAPL2.2	1.09E-10	0.824832089	0.174	0.356	2.50E-06	8
TMEM50A.1	1.28E-10	0.715098927	0.165	0.345	2.94E-06	8
ANXA5.5	1.33E-10	0.673210672	0.175	0.36	3.06E-06	8
TMEM230	1.45E-10	0.763153538	0.162	0.335	3.32E-06	8
RAB2A.2	3.06E-10	0.614441813	0.13	0.276	7.04E-06	8
CALR.2	5.35E-10	0.636722388	0.237	0.453	1.23E-05	8
CHMP2A.4	6.92E-10	0.808475652	0.159	0.323	1.59E-05	8
IFNGR1.5	1.03E-09	0.529318281	0.125	0.265	2.36E-05	8
RHOA.4	1.20E-09	0.716594469	0.27	0.519	2.75E-05	8
GSTO1.2	1.97E-09	0.870159239	0.164	0.33	4.52E-05	8
PPIB.2	2.27E-09	0.754062608	0.259	0.497	5.22E-05	8
SH3GLB1.2	3.55E-09	0.744091314	0.134	0.274	8.16E-05	8
EID1.2	4.46E-09	0.898205496	0.224	0.437	0.000102515	8
DAD1.1	8.58E-09	0.844282838	0.19	0.367	0.00019722	8
GNAS.3	1.11E-08	0.827056117	0.182	0.352	0.000254589	8
NDUFC1.6	1.78E-08	0.624194605	0.142	0.277	0.000410006	8
LAMP1.2	2.55E-08	0.917875234	0.16	0.311	0.000586263	8
CD63.7	2.93E-08	1.252300507	0.466	0.522	0.000674088	8
KDELR2.3	6.84E-08	0.772906754	0.137	0.266	0.001573032	8
RAB5C.4	7.39E-08	0.884064026	0.139	0.274	0.001697724	8
SKP1.1	9.36E-08	0.713768269	0.331	0.615	0.002151295	8
VAMP5.4	1.21E-07	1.978297849	0.324	0.307	0.002790898	8
COX17.3	1.56E-07	0.927056069	0.172	0.325	0.003574066	8
PSMA7.3	1.70E-07	0.920281448	0.287	0.534	0.003902777	8
MRPL33.1	2.20E-07	0.860903619	0.159	0.298	0.005064037	8
POLR2L.2	3.63E-07	0.804103623	0.244	0.446	0.008341645	8
HLA-C.5	3.73E-07	0.51382419	0.78	0.887	0.00856594	8
TCEB2.2	4.01E-07	0.841744501	0.316	0.582	0.009224235	8
RABAC1.2	4.93E-07	0.932849267	0.2	0.364	0.011334315	8
HSP90B1.2	6.90E-07	0.830849881	0.234	0.414	0.015852434	8
NDUFA4.3	7.45E-07	0.886868763	0.302	0.55	0.017130604	8
SLIRP.5	8.15E-07	0.902205206	0.152	0.28	0.01873346	8
GUK1.1	1.48E-06	0.867675776	0.289	0.516	0.033919493	8
YWHAE.6	1.65E-06	0.996718335	0.177	0.318	0.037851533	8
BCAP31.1	1.89E-06	0.97928669	0.157	0.285	0.043333952	8
ANXA2.4	2.04E-06	0.726573785	0.232	0.4	0.046857032	8
HLA-DRB5.8	0	3.179752372	0.974	0.347	0	9
HLA-DRA.9	0	2.978219886	1	0.659	0	9
HLA-DPA1.9	0	2.814642076	1	0.552	0	9
HLA-DQA1.6	0	2.788872073	0.998	0.315	0	9

HLA-DPB1.8	0	2.750163409	1	0.616	0	9
HLA-DQB1.7	0	2.704578751	0.998	0.366	0	9
C1QB.4	0	2.649218072	0.842	0.209	0	9
HLA-DQA2.1	0	2.558996848	0.91	0.136	0	9
C1QC.2	0	2.432305039	0.75	0.129	0	9
LYZ.8	0	2.243230498	0.986	0.324	0	9
MS4A6A.3	0	2.145181836	0.936	0.162	0	9
GPX1.8	0	2.140842384	1	0.404	0	9
CST3.8	0	2.109766811	0.998	0.359	0	9
SGK1.1	0	2.097863837	0.903	0.153	0	9
HLA-DMA.7	0	1.971624779	0.986	0.257	0	9
GRN.8	0	1.923384344	0.965	0.223	0	9
NPC2.7	0	1.915414002	0.991	0.353	0	9
HLA-DMB.2	0	1.883279168	0.972	0.178	0	9
CTSH.1	0	1.860867225	0.931	0.144	0	9
MS4A7.1	0	1.848489544	0.893	0.12	0	9
MAFB.1	0	1.832306037	0.771	0.105	0	9
CPVL.1	0	1.792711505	0.811	0.096	0	9
FAM26F.1	0	1.75923433	0.827	0.105	0	9
RNASE6.1	0	1.74877906	0.873	0.118	0	9
KLF4	0	1.737208366	0.811	0.09	0	9
GPNMB.1	0	1.736301761	0.539	0.072	0	9
AIF1.9	0	1.678471788	0.971	0.229	0	9
CD68.2	0	1.677349323	0.88	0.167	0	9
ALDH2.1	0	1.554565306	0.894	0.123	0	9
GPR34.1	0	1.537522642	0.614	0.074	0	9
MSR1.1	0	1.486081612	0.737	0.088	0	9
C1orf162.2	0	1.457556851	0.919	0.183	0	9
PHACTR1.1	0	1.432347165	0.641	0.059	0	9
KCTD12.1	0	1.418534302	0.861	0.105	0	9
FCGR2A.1	0	1.403542866	0.823	0.111	0	9
CSF2RA.1	0	1.388021356	0.778	0.078	0	9
LST1.3	0	1.370869832	0.96	0.195	0	9
PKIB.1	0	1.352672144	0.601	0.053	0	9
MS4A4A.1	0	1.336747281	0.579	0.069	0	9
PPT1.5	0	1.335150608	0.894	0.213	0	9
CXCL16.1	0	1.300994345	0.828	0.111	0	9
C3AR1.1	0	1.30096392	0.672	0.091	0	9
FGL2.1	0	1.28987511	0.809	0.122	0	9
IFI30.1	0	1.274995981	0.679	0.087	0	9
CPM.1	0	1.273049446	0.702	0.113	0	9
IGSF6.1	0	1.259029693	0.652	0.074	0	9
SPI1.1	0	1.258946035	0.879	0.111	0	9
OLR1.1	0	1.2405654	0.594	0.06	0	9
VSIG4.1	0	1.232143352	0.607	0.079	0	9
CYBB.1	0	1.21733793	0.761	0.097	0	9
LY86.1	0	1.216219336	0.776	0.085	0	9
MNDA.2	0	1.19974518	0.83	0.111	0	9
SLC1A3.1	0	1.196394228	0.702	0.079	0	9
NCOA4.1	0	1.189023745	0.806	0.151	0	9
PLXDC2.1	0	1.180566695	0.754	0.098	0	9
IL18.1	0	1.167850586	0.745	0.079	0	9
RNF130.1	0	1.148587446	0.818	0.138	0	9
CTSZ.1	0	1.140096192	0.756	0.132	0	9
CSF1R.1	0	1.133953492	0.711	0.085	0	9
ANKRD22.1	0	1.118360154	0.591	0.055	0	9
CD163.1	0	1.098449652	0.707	0.101	0	9
CLEC4E.1	0	1.08003712	0.655	0.087	0	9

CSTA.2	0	1.075608276	0.742	0.105	0	9
FCGR1A.1	0	1.073618644	0.553	0.065	0	9
TNFSF13B.1	0	1.072474123	0.726	0.106	0	9
RP11-1143G9.4.1	0	1.066826631	0.7	0.098	0	9
SERPINF1.1	0	1.06553312	0.61	0.067	0	9
CD302.1	0	1.054266009	0.756	0.099	0	9
SLC7A7.1	0	1.047428311	0.664	0.077	0	9
CLEC10A	0	1.043056538	0.364	0.009	0	9
MARCH1.1	0	1.039976788	0.685	0.075	0	9
PLAUR.1	0	1.025921473	0.646	0.1	0	9
LY96.1	0	1.013146754	0.731	0.095	0	9
CLEC7A.1	0	1.012442483	0.782	0.107	0	9
RAB31.1	0	0.999084247	0.74	0.104	0	9
FCGR2B.1	0	0.993228589	0.522	0.067	0	9
OGFRL1.1	0	0.984235785	0.66	0.103	0	9
CD83	0	0.979565496	0.548	0.055	0	9
AXL.1	0	0.96924061	0.53	0.064	0	9
CD86.1	0	0.964248107	0.664	0.071	0	9
IRF8.1	0	0.9570397	0.515	0.063	0	9
SLC31A2.1	0	0.951127005	0.636	0.083	0	9
NLRP3.1	0	0.932307262	0.485	0.057	0	9
MFSD1.1	0	0.91401687	0.712	0.122	0	9
TBXAS1.1	0	0.910822611	0.667	0.103	0	9
LILRB4.1	0	0.898813722	0.612	0.079	0	9
CLEC12A.1	0	0.896995424	0.607	0.063	0	9
HLA-DQB2	0	0.895835453	0.397	0.023	0	9
CECR1.1	0	0.889294332	0.653	0.09	0	9
IL13RA1.1	0	0.889103158	0.655	0.091	0	9
LGALS2	0	0.87807869	0.575	0.08	0	9
RGS18.1	0	0.876425358	0.622	0.072	0	9
FPR3	0	0.873521896	0.463	0.02	0	9
NCF2.1	0	0.868515116	0.579	0.066	0	9
BASP1.1	0	0.851531405	0.482	0.053	0	9
TLR2.1	0	0.849011219	0.631	0.083	0	9
SRGAP1.1	0	0.834122202	0.619	0.089	0	9
PILRA.1	0	0.830931291	0.57	0.074	0	9
RAB32.1	0	0.804475891	0.553	0.073	0	9
CCDC88A.1	0	0.795535433	0.591	0.085	0	9
TM6SF1.1	0	0.78775736	0.515	0.059	0	9
LYN.1	0	0.77666336	0.648	0.099	0	9
SDS	0	0.772989546	0.258	0.014	0	9
HCK.1	0	0.765245446	0.556	0.063	0	9
PLBD1.1	0	0.75263117	0.56	0.074	0	9
ADAP2.1	0	0.71188269	0.522	0.066	0	9
LILRB3.1	0	0.674115644	0.539	0.058	0	9
ST14.1	0	0.672229883	0.477	0.046	0	9
GCLC.1	0	0.662656174	0.452	0.046	0	9
SLAMF8.1	0	0.658774742	0.414	0.03	0	9
HLA-DOA.1	0	0.623553019	0.511	0.062	0	9
KYNU.1	0	0.56699132	0.432	0.045	0	9
FCER1G.9	2.77E-306	1.610573574	0.981	0.274	6.38E-302	9
SAP30.1	1.15E-305	0.833145536	0.596	0.09	2.65E-301	9
GPR183.1	2.04E-303	1.674260658	0.87	0.205	4.68E-299	9
C1QA.9	2.84E-303	2.542018606	0.849	0.224	6.52E-299	9
ATP6V1B2.1	9.15E-302	0.851780746	0.683	0.118	2.10E-297	9
PAK1.1	1.48E-301	0.72510517	0.565	0.08	3.40E-297	9
IFNGR2.1	3.81E-297	0.716669025	0.626	0.101	8.75E-293	9
HLA-DRB1.9	2.34E-296	2.36269474	1	0.573	5.38E-292	9

GNAQ.1	6.91E-296	0.84069171	0.666	0.113	1.59E-291	9
CD74.7	7.69E-296	2.384380821	1	0.785	1.77E-291	9
TYROBP.9	1.24E-293	1.933782682	0.988	0.362	2.85E-289	9
MARCKS.1	7.73E-293	1.146240263	0.698	0.128	1.78E-288	9
LGALS9.1	1.13E-291	0.774440795	0.617	0.098	2.60E-287	9
CEBPD.6	1.58E-291	1.54336365	0.924	0.251	3.63E-287	9
C1orf54.1	3.52E-291	0.843489712	0.51	0.069	8.08E-287	9
SNX10.1	4.38E-291	1.036790578	0.809	0.172	1.01E-286	9
GAPT.1	5.61E-290	0.888922054	0.466	0.058	1.29E-285	9
CTSB.8	6.02E-290	1.661167916	0.946	0.274	1.38E-285	9
CTSS.7	3.12E-289	1.668607583	0.979	0.336	7.17E-285	9
MPEG1	9.62E-289	0.574876831	0.385	0.038	2.21E-284	9
AP1S2.2	7.50E-288	1.085774776	0.861	0.198	1.72E-283	9
NUDT16.1	6.43E-284	0.786904625	0.721	0.135	1.48E-279	9
IFNGR1.6	3.56E-282	1.173199727	0.917	0.231	8.17E-278	9
LACTB.1	7.21E-281	0.6647325	0.53	0.076	1.66E-276	9
HEXB.1	5.87E-279	0.911081454	0.769	0.16	1.35E-274	9
CHPT1.1	2.04E-277	0.780570158	0.66	0.117	4.70E-273	9
THEMIS2.1	3.15E-276	0.648998929	0.605	0.098	7.24E-272	9
SRGAP2B.1	4.90E-276	0.741189642	0.6	0.097	1.13E-271	9
PLD4.1	1.26E-274	0.748062274	0.473	0.061	2.89E-270	9
CD14.2	1.28E-274	1.180662047	0.66	0.12	2.93E-270	9
CD300A.1	3.13E-274	0.718871578	0.57	0.088	7.20E-270	9
TREM2.1	1.32E-273	0.973656929	0.516	0.072	3.02E-269	9
P2RY13.1	6.76E-273	0.786206708	0.402	0.045	1.55E-268	9
CAPG.2	1.63E-272	1.257977235	0.858	0.203	3.74E-268	9
DSE.1	1.69E-271	0.624100774	0.484	0.065	3.89E-267	9
RGS10.5	3.49E-271	1.293442737	0.946	0.267	8.01E-267	9
DAB2.1	6.08E-271	1.099731728	0.695	0.139	1.40E-266	9
FCGRT.4	2.22E-270	1.069842667	0.884	0.214	5.11E-266	9
GNB4.1	6.17E-270	0.693685863	0.525	0.077	1.42E-265	9
MEF2C.1	2.50E-268	0.737383749	0.705	0.127	5.75E-264	9
CFD.1	3.29E-267	1.328191102	0.636	0.119	7.57E-263	9
FBP1.1	6.22E-267	0.731925934	0.484	0.067	1.43E-262	9
RBM47.1	7.19E-267	0.582163778	0.468	0.063	1.65E-262	9
LIMS1.6	2.34E-262	1.116038394	0.873	0.222	5.37E-258	9
FGR.1	3.13E-259	0.626131663	0.581	0.095	7.20E-255	9
BTK.1	4.76E-257	0.549610875	0.418	0.051	1.09E-252	9
SIGLEC10.1	1.16E-254	0.614706732	0.447	0.059	2.67E-250	9
SHTN1.1	5.72E-254	0.556739471	0.395	0.047	1.31E-249	9
ACSL1.1	1.23E-253	0.690429677	0.574	0.097	2.84E-249	9
TYMP.7	1.52E-252	1.314051027	0.912	0.269	3.49E-248	9
GRINA.1	2.32E-252	0.765470149	0.683	0.137	5.34E-248	9
C5AR1.1	2.84E-252	0.844912433	0.471	0.067	6.53E-248	9
SLCO2B1.1	4.27E-252	0.702217234	0.452	0.062	9.81E-248	9
TCOF1	3.57E-248	1.073408979	0.594	0.107	8.22E-244	9
KCNMA1.1	4.02E-247	0.775492171	0.433	0.058	9.23E-243	9
LRRC25.1	4.23E-247	0.518954436	0.418	0.053	9.72E-243	9
FAM105A.1	3.04E-246	0.662261243	0.563	0.094	6.98E-242	9
RNF13.1	8.21E-246	0.87578913	0.778	0.182	1.89E-241	9
CLN8.1	1.55E-245	0.723508602	0.548	0.091	3.56E-241	9
LAIR1.1	7.29E-245	0.711503477	0.582	0.101	1.68E-240	9
ALCAM.1	2.21E-243	0.62791443	0.458	0.065	5.09E-239	9
NAMPT.2	3.60E-243	0.825909307	0.834	0.207	8.28E-239	9
C15orf48	1.27E-242	1.048892289	0.354	0.038	2.93E-238	9
TNFAIP2.1	1.54E-242	0.544714615	0.402	0.05	3.54E-238	9
H2AFY.5	5.97E-241	1.097561222	0.896	0.257	1.37E-236	9
SLC8A1.1	1.69E-240	0.582547031	0.471	0.068	3.89E-236	9

ASAH1.4	2.12E-240	1.256505193	0.818	0.216	4.87E-236	9
LPCAT2.1	4.12E-240	0.605191022	0.473	0.069	9.48E-236	9
UBE2E2.1	1.26E-238	0.507087508	0.419	0.056	2.90E-234	9
PSAP.8	2.51E-238	1.865841675	0.983	0.446	5.77E-234	9
CREG1.1	1.10E-236	0.690121902	0.596	0.112	2.52E-232	9
GLUL.7	1.35E-234	1.204046609	0.922	0.28	3.11E-230	9
ADORA3.1	1.50E-234	0.579791095	0.364	0.043	3.44E-230	9
BRI3.1	1.70E-233	0.756420651	0.671	0.14	3.90E-229	9
YWHAH.7	2.73E-233	1.199092284	0.88	0.257	6.27E-229	9
PSTPIP2.1	1.13E-230	0.635315602	0.437	0.062	2.59E-226	9
ITGAX.1	1.59E-230	0.56735874	0.395	0.051	3.65E-226	9
NAGA.1	4.38E-228	0.502841128	0.432	0.061	1.01E-223	9
UNC93B1.1	5.14E-228	0.512893792	0.43	0.061	1.18E-223	9
SCPEP1.1	5.92E-228	0.749070309	0.603	0.117	1.36E-223	9
PYCARD.5	1.29E-227	0.939712506	0.851	0.221	2.97E-223	9
PABPC4.1	1.24E-226	0.798236144	0.764	0.177	2.84E-222	9
TPP1.1	9.42E-225	0.718845226	0.723	0.161	2.16E-220	9
DAPK1.1	1.09E-223	0.510519565	0.395	0.053	2.50E-219	9
MGAT1.1	1.31E-223	0.718862899	0.759	0.177	3.01E-219	9
CD9.3	6.29E-221	1.022184537	0.789	0.199	1.45E-216	9
FNIP2	1.64E-220	0.550729999	0.426	0.061	3.77E-216	9
DRAM2.1	1.70E-219	0.730892142	0.686	0.152	3.92E-215	9
SLC11A1.1	2.14E-218	0.803322784	0.588	0.112	4.91E-214	9
LGMN	2.72E-217	0.927623439	0.438	0.068	6.25E-213	9
RGS2.8	5.16E-216	1.308635571	0.915	0.31	1.18E-211	9
FMNL2.1	1.50E-215	0.538169192	0.357	0.045	3.45E-211	9
ALOX5.1	8.35E-215	0.573752812	0.494	0.083	1.92E-210	9
HBEGF.1	9.54E-215	0.651518212	0.352	0.043	2.19E-210	9
APOC1.7	1.07E-213	2.045125573	0.847	0.259	2.45E-209	9
SRGAP2.1	1.33E-212	0.557990399	0.501	0.085	3.06E-208	9
LAT2.1	1.63E-207	0.546942079	0.477	0.08	3.73E-203	9
ABHD12.1	4.91E-202	0.531974902	0.489	0.086	1.13E-197	9
FCGR3A.4	2.01E-201	1.210572875	0.789	0.21	4.61E-197	9
SAT1.7	2.51E-201	1.505737223	0.995	0.616	5.76E-197	9
NR4A3	7.02E-201	0.523235914	0.289	0.031	1.61E-196	9
PRCP.1	9.35E-198	0.541097973	0.539	0.105	2.15E-193	9
ATP6V0B.5	3.53E-197	0.831243779	0.88	0.27	8.11E-193	9
ATP6AP1.1	8.86E-197	0.57537285	0.652	0.145	2.04E-192	9
ALOX15B.1	1.75E-195	0.503997566	0.286	0.031	4.02E-191	9
C3.1	6.22E-194	0.564202115	0.596	0.121	1.43E-189	9
STX7.1	8.86E-193	0.519207033	0.562	0.114	2.04E-188	9
TKT.2	4.60E-189	0.675395602	0.775	0.205	1.06E-184	9
ATP1B3.6	7.05E-189	0.936138372	0.825	0.245	1.62E-184	9
MRPL3.1	2.57E-186	0.53384367	0.607	0.133	5.91E-182	9
RAC1.6	7.49E-186	1.023451415	0.984	0.425	1.72E-181	9
ZFAND5.6	1.91E-185	1.120664201	0.86	0.284	4.39E-181	9
CD4.1	1.88E-184	0.574939726	0.57	0.119	4.32E-180	9
LGALS3.4	9.68E-183	1.112425097	0.769	0.223	2.23E-178	9
PICALM.1	2.02E-181	0.604468462	0.692	0.168	4.64E-177	9
FTH1.8	9.85E-181	1.196987638	1	0.961	2.26E-176	9
ATG3.1	1.04E-179	0.52894676	0.627	0.146	2.39E-175	9
SKAP2.1	5.27E-179	0.579540236	0.657	0.157	1.21E-174	9
LIPA.1	1.23E-178	1.188207452	0.64	0.165	2.82E-174	9
CNPY3.1	1.36E-178	0.608164703	0.757	0.194	3.14E-174	9
SAMHD1.1	6.61E-178	0.691639914	0.728	0.185	1.52E-173	9
HMGA1.1	2.09E-177	0.633480597	0.475	0.091	4.81E-173	9
MAP3K8.1	1.52E-175	0.695765526	0.586	0.132	3.49E-171	9
FTL.7	1.55E-173	1.712149825	1	0.934	3.55E-169	9

HMOX1.1	1.64E-173	0.687190126	0.49	0.098	3.78E-169	9
C10orf54.6	1.73E-173	0.788077345	0.842	0.257	3.97E-169	9
SRGAP2C.1	1.44E-172	0.580365649	0.551	0.119	3.31E-168	9
IER3.1	3.01E-171	0.638692242	0.47	0.093	6.91E-167	9
ATF3	3.05E-171	0.694220831	0.579	0.132	7.02E-167	9
VMO1.1	4.41E-170	0.510084473	0.321	0.045	1.01E-165	9
MEF2A.1	8.35E-170	0.580291207	0.619	0.142	1.92E-165	9
HEXA.1	1.21E-169	0.576988989	0.688	0.175	2.79E-165	9
SYNGR2.1	3.05E-169	0.524221569	0.622	0.148	7.00E-165	9
LAP3.1	3.26E-166	0.621535267	0.586	0.138	7.50E-162	9
AOAH.2	6.33E-166	0.586355709	0.567	0.128	1.46E-161	9
AKR1B1.2	3.02E-164	0.647671637	0.752	0.215	6.93E-160	9
ANXA5.6	1.32E-163	0.830549722	0.906	0.328	3.02E-159	9
SDCBP.7	2.43E-162	0.954486705	0.899	0.35	5.59E-158	9
AP2S1.7	2.10E-161	0.621981338	0.88	0.286	4.84E-157	9
ARRB2.1	1.93E-160	0.502574525	0.686	0.176	4.44E-156	9
HCLS1.4	2.00E-160	0.824245461	0.884	0.298	4.61E-156	9
SLC40A1.1	4.30E-160	0.826808176	0.374	0.066	9.89E-156	9
APOE.8	1.26E-159	2.395774849	0.905	0.418	2.91E-155	9
COTL1.4	7.99E-159	1.11677683	0.951	0.412	1.84E-154	9
ARL5A.2	4.12E-158	0.558767926	0.759	0.211	9.48E-154	9
HERPUD1.6	1.56E-157	1.340858572	0.958	0.472	3.58E-153	9
COMT.1	1.93E-157	0.506548844	0.645	0.166	4.43E-153	9
SOAT1.1	7.19E-157	0.509502	0.452	0.091	1.65E-152	9
CMTM6.1	3.56E-155	0.556394854	0.73	0.204	8.19E-151	9
LTA4H.1	4.20E-155	0.586595087	0.574	0.14	9.66E-151	9
ATP6V1F.7	1.98E-153	0.762663859	0.894	0.326	4.56E-149	9
SLC43A2.1	2.66E-153	0.518317294	0.392	0.072	6.10E-149	9
SLC25A5.3	1.18E-152	0.872880979	0.915	0.363	2.71E-148	9
FABP5.1	3.37E-151	0.799446041	0.671	0.187	7.75E-147	9
PLEK.2	2.02E-148	0.571224862	0.563	0.134	4.64E-144	9
ATP6AP2.4	1.63E-146	0.661168873	0.844	0.284	3.75E-142	9
FAM49B.4	2.11E-146	0.705943799	0.899	0.316	4.85E-142	9
LPAR6.1	2.33E-144	0.597979451	0.553	0.134	5.37E-140	9
PLD3.1	1.91E-143	0.71489112	0.529	0.132	4.40E-139	9
RHOG.3	3.10E-142	0.609409145	0.78	0.242	7.14E-138	9
PPA1.1	4.82E-142	0.78472137	0.686	0.21	1.11E-137	9
MIS18BP1.2	6.36E-142	0.595996163	0.679	0.189	1.46E-137	9
RNH1.3	9.61E-142	0.533638772	0.787	0.249	2.21E-137	9
AZI2.1	1.15E-140	0.551308516	0.494	0.115	2.63E-136	9
PTPRE	4.40E-140	0.616847573	0.565	0.144	1.01E-135	9
RTN4.7	9.75E-140	0.62140954	0.901	0.326	2.24E-135	9
GLIPR1.6	1.33E-138	0.599167008	0.853	0.275	3.05E-134	9
JAML.1	4.28E-137	0.520311198	0.636	0.174	9.83E-133	9
REL.5	4.12E-132	0.779218578	0.856	0.299	9.47E-128	9
VAMP8.4	4.57E-132	0.835718187	0.941	0.44	1.05E-127	9
CTSL.1	1.11E-131	0.761660527	0.536	0.142	2.55E-127	9
CEBPB.7	1.25E-131	0.657466345	0.844	0.306	2.88E-127	9
GDI2.8	1.35E-131	0.572596053	0.827	0.29	3.11E-127	9
SNX3.7	2.66E-128	0.677715218	0.915	0.361	6.12E-124	9
RHOB.7	9.04E-128	0.683074065	0.873	0.326	2.08E-123	9
TUBA1B.7	2.84E-127	0.742024084	0.92	0.392	6.54E-123	9
SEC11A.3	5.78E-125	0.578726995	0.873	0.316	1.33E-120	9
MT-CO1.4	7.54E-125	0.682529823	0.991	0.923	1.73E-120	9
YBX1.5	9.66E-124	0.785696774	0.997	0.656	2.22E-119	9
GSTO1.3	5.03E-122	0.523459939	0.83	0.301	1.16E-117	9
GPX4.7	3.30E-121	0.677856042	0.941	0.433	7.58E-117	9
FKBP5.5	1.22E-120	0.724934514	0.927	0.373	2.81E-116	9

ACP5	1.34E-120	0.561492219	0.402	0.089	3.09E-116	9
HSPA1A.3	1.76E-119	0.705312639	0.657	0.216	4.04E-115	9
S100A11.8	1.63E-118	0.921633017	0.979	0.574	3.76E-114	9
SMAP2.5	1.49E-117	0.582370749	0.801	0.276	3.42E-113	9
ANXA2.5	2.09E-117	0.76330166	0.889	0.372	4.80E-113	9
AREG.1	3.47E-115	1.584445643	0.589	0.193	7.98E-111	9
SH3BGRL.4	1.41E-110	0.576562506	0.908	0.368	3.23E-106	9
CYBA.5	2.82E-109	0.811548416	0.991	0.674	6.49E-105	9
CTSC.7	2.22E-104	0.667701397	0.818	0.326	5.10E-100	9
LAPTM5.7	5.27E-100	0.739748393	0.99	0.549	1.21E-95	9
ITGB2.7	1.44E-96	0.678277986	0.901	0.394	3.32E-92	9
MALAT1.8	1.68E-96	-1.0503903	0.998	0.982	3.86E-92	9
ARPC3.4	4.42E-96	0.63484575	0.979	0.589	1.02E-91	9
CSTB.5	6.11E-94	0.654129905	0.865	0.378	1.40E-89	9
ARPC5.4	6.74E-88	0.504413448	0.899	0.415	1.55E-83	9
MT-CO3.5	1.28E-86	0.574597402	0.988	0.875	2.94E-82	9
NEAT1.5	1.59E-85	0.644128122	0.976	0.743	3.66E-81	9
ARPC1B.2	8.42E-85	0.585758873	0.96	0.506	1.93E-80	9
CD63.8	8.30E-83	0.627466945	0.962	0.501	1.91E-78	9
RHOA.5	7.73E-79	0.545844964	0.943	0.489	1.78E-74	9
OAZ1.6	1.18E-78	0.569267319	0.99	0.725	2.71E-74	9
TSPO.5	1.90E-77	0.548351871	0.858	0.401	4.37E-73	9
FOS.7	6.42E-76	0.697149037	0.971	0.669	1.48E-71	9
RPS27.5	2.11E-75	-0.701954875	1	0.974	4.85E-71	9
MT-ND4.4	3.97E-75	0.531120521	0.988	0.869	9.11E-71	9
ITM2B.8	1.02E-74	0.663208365	0.997	0.77	2.34E-70	9
CTSD.4	1.28E-72	1.130104798	0.832	0.42	2.95E-68	9
MT-ND1.4	7.96E-69	0.564447521	0.986	0.778	1.83E-64	9
LGALS1.3	2.28E-67	0.529793811	0.938	0.557	5.24E-63	9
RPS26.6	2.69E-61	-0.789064785	0.988	0.865	6.19E-57	9
IL32.9	1.19E-58	-2.643970214	0.426	0.584	2.73E-54	9
PABPC1.4	3.41E-57	0.523855626	0.979	0.649	7.83E-53	9
CD2.8	2.01E-54	-2.337075903	0.211	0.467	4.63E-50	9
INSR.2	3.27E-52	-0.709414862	0.378	0.126	7.51E-48	9
SEPP1	6.84E-50	1.304680846	0.34	0.122	1.57E-45	9
S100A9.8	3.98E-49	-1.022046181	0.575	0.237	9.14E-45	9
IGLC2	5.43E-49	-2.671695112	0.277	0.081	1.25E-44	9
ETS1.9	7.45E-49	-1.554222035	0.068	0.35	1.71E-44	9
RGS5	2.75E-48	-1.045827339	0.354	0.116	6.32E-44	9
CD3E.8	6.11E-48	-1.823035872	0.153	0.408	1.40E-43	9
TRAC.9	8.83E-46	-2.327998858	0.217	0.441	2.03E-41	9
RPL23A.6	4.24E-44	-0.500551288	0.998	0.927	9.74E-40	9
CCL5.8	5.49E-44	-3.248565491	0.478	0.565	1.26E-39	9
CD3G.8	7.93E-43	-1.555689611	0.047	0.304	1.82E-38	9
CD3D.9	8.86E-43	-2.204829075	0.308	0.482	2.04E-38	9
RPS29.6	1.54E-42	-0.593680976	0.998	0.918	3.54E-38	9
SYNE2.8	2.65E-42	-1.480047071	0.088	0.343	6.09E-38	9
RPL31.6	2.89E-39	-0.557358201	0.995	0.903	6.64E-35	9
GZMA.8	3.74E-39	-2.353174477	0.231	0.427	8.60E-35	9
S100A8.2	1.40E-38	-1.312095842	0.48	0.199	3.22E-34	9
APP.2	2.83E-38	-0.554163545	0.426	0.168	6.50E-34	9
HLA-A.6	1.80E-36	-0.626243127	0.998	0.889	4.13E-32	9
IFITM3.9	1.76E-34	-0.726677335	0.776	0.361	4.06E-30	9
ITM2A.9	5.58E-34	-1.66427008	0.106	0.319	1.28E-29	9
LCK.9	8.33E-32	-1.287086478	0.092	0.296	1.91E-27	9
TRBC2.8	4.55E-31	-1.873180667	0.21	0.384	1.05E-26	9
BTG1.8	1.46E-30	-0.953489993	0.981	0.836	3.35E-26	9
CD59.3	5.24E-30	-0.923957397	0.504	0.221	1.20E-25	9

CST7.8	5.85E-28	-1.92008804	0.243	0.395	1.34E-23	9
ENPP2.1	1.78E-27	-1.734757682	0.303	0.118	4.09E-23	9
CD7.6	1.09E-26	-1.564949481	0.13	0.307	2.52E-22	9
CD27.8	2.10E-26	-1.569003697	0.095	0.275	4.82E-22	9
CD69.9	9.69E-25	-2.245613002	0.506	0.53	2.23E-20	9
IGKC.6	1.48E-24	-3.241159783	0.596	0.283	3.40E-20	9
TRBC1.8	1.19E-22	-2.129225338	0.229	0.358	2.74E-18	9
CLEC2D.8	1.43E-21	-1.293053964	0.165	0.312	3.29E-17	9
ATP1B1.2	7.99E-21	-0.50510341	0.293	0.131	1.84E-16	9
ACAP1.9	1.44E-20	-1.232216957	0.208	0.338	3.32E-16	9
CD96.9	2.82E-20	-1.116719604	0.109	0.255	6.47E-16	9
GZMK.9	6.65E-19	-2.629458997	0.274	0.369	1.53E-14	9
LTB.8	2.41E-18	-1.439410569	0.18	0.307	5.54E-14	9
TUBA4A.7	5.37E-18	-1.225194195	0.147	0.278	1.23E-13	9
NDUFC1.7	1.30E-17	-0.61119348	0.511	0.26	2.98E-13	9
CD52.6	6.91E-17	-1.635028012	0.607	0.546	1.59E-12	9
PPDPF.5	1.82E-16	-1.088637943	0.816	0.653	4.18E-12	9
DUSP2.8	2.11E-16	-1.819286054	0.386	0.433	4.85E-12	9
TSC22D3.6	4.27E-14	-0.899572375	0.945	0.774	9.81E-10	9
IGFBP7.9	4.67E-13	-2.525080741	0.714	0.364	1.07E-08	9
FYN.5	5.03E-13	-1.072098471	0.175	0.27	1.16E-08	9
OCIAD2.6	9.05E-13	-1.077466858	0.168	0.265	2.08E-08	9
RNF19A.8	1.25E-12	-1.098620803	0.189	0.28	2.86E-08	9
IL7R.7	1.80E-12	-2.032293564	0.225	0.307	4.14E-08	9
EVL.8	2.35E-12	-1.212337623	0.544	0.493	5.39E-08	9
MGP.1	9.10E-12	-1.514923348	0.284	0.144	2.09E-07	9
HBA2.7	1.23E-11	-3.10284795	0.445	0.242	2.82E-07	9
NKG7.9	1.32E-11	-2.511656168	0.437	0.427	3.03E-07	9
RGCC.8	1.46E-11	-0.512762966	0.508	0.299	3.35E-07	9
STK17A.8	3.95E-11	-1.104380713	0.321	0.368	9.07E-07	9
SYTL3.8	4.46E-11	-1.103107485	0.201	0.28	1.03E-06	9
HNRNPH1.2	4.71E-11	-0.501553337	0.544	0.31	1.08E-06	9
CRIP1.7	1.46E-10	-0.986750983	0.213	0.297	3.35E-06	9
HBB.5	1.52E-10	-3.26422534	0.69	0.379	3.50E-06	9
ODF2L.4	3.04E-10	-0.959580035	0.18	0.263	6.98E-06	9
GPX3	3.10E-10	-0.588753435	0.289	0.16	7.12E-06	9
BIRC3.7	2.36E-09	-1.126768776	0.199	0.275	5.42E-05	9
SEPW1.9	4.80E-09	-0.703253409	0.619	0.348	0.000110199	9
DNAJB1.7	1.17E-08	-0.506287512	0.678	0.409	0.000269449	9
DSTN.7	1.38E-08	-0.879574924	0.525	0.298	0.000316494	9
SEC61G.5	3.56E-08	-0.51002983	0.74	0.425	0.000819048	9
ADIRF.6	4.20E-08	-1.751410617	0.411	0.234	0.00096493	9
JUND.6	5.45E-08	-0.502601346	0.518	0.3	0.001253258	9
SPARC.1	5.59E-08	-1.645188059	0.312	0.177	0.001284899	9
CCL4.9	9.55E-08	-2.301507239	0.482	0.438	0.002195045	9
IVNS1ABP.1	1.11E-07	-0.556726069	0.338	0.199	0.002543127	9
GNLY.9	1.34E-07	-2.441106525	0.432	0.245	0.003070282	9
IGFBP3.8	2.76E-07	-1.92328705	0.411	0.237	0.006331768	9
KIAA1551.7	3.56E-07	-1.011668959	0.317	0.336	0.008190398	9
IL2RG.9	5.45E-07	-1.053721955	0.383	0.372	0.012531846	9
ROMO1.4	8.03E-07	-0.524804597	0.506	0.303	0.01846251	9
RARRES3.8	2.10E-06	-0.977350867	0.43	0.41	0.04817984	9
NDUFA4L2.9	1.34E-254	2.985929806	0.829	0.269	3.09E-250	10
BBOX1	2.35E-252	2.727853546	0.301	0.025	5.40E-248	10
CD24.2	5.44E-231	2.804527171	0.669	0.16	1.25E-226	10
MALAT1.9	1.74E-228	-3.169818309	0.855	0.987	4.01E-224	10
TMSB4X.4	1.07E-201	-2.103811389	0.811	0.983	2.46E-197	10
CRYAB.9	7.30E-185	2.852420521	0.707	0.243	1.68E-180	10

KRT18	3.83E-180	2.738028737	0.464	0.092	8.79E-176	10
FXYD2	6.14E-180	2.696772184	0.522	0.119	1.41E-175	10
GAPDH.6	6.39E-163	1.838012567	0.926	0.864	1.47E-158	10
CMBL	5.37E-162	2.448268383	0.331	0.05	1.23E-157	10
B2M.7	6.01E-161	-1.385114066	0.908	0.991	1.38E-156	10
BHMT	2.33E-157	2.330573453	0.267	0.032	5.35E-153	10
CYB5A.3	6.37E-154	2.819936948	0.622	0.215	1.46E-149	10
SRGN.6	1.28E-144	-2.298930279	0.157	0.815	2.95E-140	10
BTG1.9	1.24E-142	-2.278607171	0.303	0.862	2.84E-138	10
MT-CO1.5	5.78E-138	-1.536186987	0.612	0.937	1.33E-133	10
LDHA.9	2.41E-136	2.294730335	0.817	0.57	5.54E-132	10
NAT8	2.24E-134	2.835831413	0.263	0.037	5.15E-130	10
CXCL14	4.42E-133	2.505065566	0.319	0.056	1.02E-128	10
ADIRF.7	3.55E-130	2.22293341	0.624	0.227	8.16E-126	10
MT-ND3.8	1.74E-128	-1.819934449	0.279	0.85	3.99E-124	10
NEAT1.6	2.46E-128	-2.100720763	0.161	0.775	5.65E-124	10
BNIP3.1	5.86E-120	2.410687531	0.448	0.122	1.35E-115	10
CXCR4.7	6.87E-118	-2.176372568	0.235	0.795	1.58E-113	10
FTH1.9	1.17E-117	0.90147522	0.976	0.962	2.70E-113	10
HLA-E.8	4.08E-117	-1.666376734	0.213	0.811	9.38E-113	10
ZFP36L2.9	5.02E-117	-2.369836586	0.131	0.719	1.15E-112	10
HLA-B.7	8.78E-117	-1.346380964	0.562	0.927	2.02E-112	10
PTPRC.9	1.83E-116	-2.280208281	0.042	0.638	4.21E-112	10
KRT8	2.32E-114	2.477873279	0.376	0.088	5.32E-110	10
DDX5.7	2.83E-112	-1.599211443	0.259	0.831	6.51E-108	10
ANXA4.2	4.83E-112	2.726141668	0.466	0.146	1.11E-107	10
MT-CO2.5	2.11E-110	-1.420589577	0.454	0.89	4.86E-106	10
HLA-C.6	1.60E-109	-1.38742519	0.414	0.9	3.67E-105	10
MT-ND2.5	2.22E-108	-1.423162076	0.406	0.883	5.11E-104	10
MT-ATP6.5	3.62E-108	-1.406405749	0.394	0.876	8.31E-104	10
RGS1.10	7.77E-104	-2.335960419	0.185	0.716	1.79E-99	10
S100A4.8	9.82E-104	-2.06557217	0.173	0.719	2.26E-99	10
ALDOA.4	1.44E-102	2.070128478	0.769	0.632	3.31E-98	10
MT-ND4.5	2.48E-102	-1.271505124	0.438	0.89	5.70E-98	10
FTL.8	2.76E-101	0.808849628	0.958	0.936	6.35E-97	10
MT-ND1.5	1.24E-99	-1.52406474	0.275	0.806	2.85E-95	10
MT-CO3.6	1.02E-97	-1.229159183	0.468	0.895	2.35E-93	10
MT-CYB.6	5.23E-95	-1.343093422	0.327	0.822	1.20E-90	10
TXNIP.8	1.36E-94	-1.635864318	0.267	0.782	3.13E-90	10
ARHGDIB.3	2.90E-94	-1.383137453	0.135	0.692	6.65E-90	10
ENO1.8	9.95E-89	2.091427182	0.745	0.588	2.29E-84	10
PDZK1IP1.1	1.56E-88	2.229037537	0.386	0.113	3.59E-84	10
LAPTM5.8	1.19E-85	-1.592910605	0.076	0.585	2.73E-81	10
EVL.9	2.31E-85	-1.832826983	0.026	0.513	5.32E-81	10
CORO1A.7	1.17E-84	-1.452537278	0.08	0.583	2.70E-80	10
LGALS2.1	2.81E-84	2.258028548	0.335	0.091	6.47E-80	10
ARPC2.5	1.83E-83	-1.286740379	0.137	0.656	4.21E-79	10
SRSF7.7	2.70E-83	-1.540498237	0.064	0.562	6.21E-79	10
N4BP2L2.7	2.61E-81	-1.33528524	0.06	0.547	6.01E-77	10
CD44.6	8.48E-81	-1.692364981	0.034	0.508	1.95E-76	10
HCST.8	1.20E-80	-1.538078764	0.072	0.562	2.76E-76	10
PNISR.7	5.82E-79	-1.471351812	0.052	0.527	1.34E-74	10
FYB.8	1.13E-77	-1.80989335	0.024	0.476	2.61E-73	10
NNMT.10	1.59E-77	1.832540521	0.59	0.288	3.66E-73	10
CD52.7	1.98E-77	-1.929699745	0.1	0.565	4.54E-73	10
MT-ND5.7	2.86E-77	-1.287932033	0.133	0.628	6.56E-73	10
CD53.5	5.64E-77	-1.612062106	0.034	0.487	1.29E-72	10
SH3BGRL3.6	2.29E-76	-1.14636774	0.193	0.7	5.25E-72	10

STK4.6	7.37E-76	-1.566217392	0.032	0.488	1.69E-71	10
CCL5.9	5.17E-74	-2.352593658	0.133	0.578	1.19E-69	10
CD37.6	3.34E-73	-1.539259225	0.038	0.479	7.67E-69	10
JUNB.9	6.35E-72	-1.361734012	0.265	0.74	1.46E-67	10
CD69.10	1.02E-71	-1.941256432	0.092	0.545	2.34E-67	10
CD2.9	5.69E-70	-2.07065763	0.046	0.472	1.31E-65	10
JUN.6	6.39E-70	-1.454496604	0.245	0.708	1.47E-65	10
GMFG.4	1.55E-69	-1.069104053	0.106	0.573	3.57E-65	10
FXYD5.4	1.61E-68	-1.147773821	0.056	0.497	3.71E-64	10
PRPF38B.9	1.89E-68	-1.476407562	0.028	0.45	4.35E-64	10
SON.7	1.90E-68	-0.869378026	0.114	0.586	4.38E-64	10
MCL1.5	1.92E-68	-1.251735322	0.082	0.53	4.41E-64	10
MIF.8	1.99E-68	1.902847376	0.669	0.499	4.57E-64	10
TNFAIP3.10	1.80E-67	-1.710538938	0.042	0.462	4.13E-63	10
MGST1.1	4.66E-67	2.120869385	0.333	0.104	1.07E-62	10
ACTB.5	1.02E-66	-1.031222673	0.675	0.938	2.34E-62	10
CYTIP.8	2.06E-66	-1.316174536	0.06	0.491	4.72E-62	10
KLF6.8	4.19E-66	-1.273884814	0.199	0.666	9.63E-62	10
AKAP13.8	6.29E-66	-1.330202538	0.032	0.447	1.45E-61	10
RBM39.7	6.66E-66	-1.042497675	0.11	0.562	1.53E-61	10
ANXA1.9	7.83E-66	-1.876306738	0.064	0.486	1.80E-61	10
ARGLU1.6	1.23E-65	-1.364657617	0.032	0.448	2.83E-61	10
LCP1.8	4.27E-65	-1.593467275	0.022	0.418	9.81E-61	10
GATM	1.23E-64	2.174314058	0.253	0.066	2.82E-60	10
POLR2J3.6	1.24E-64	-1.561826458	0.014	0.408	2.85E-60	10
HLA-F.8	1.33E-64	-1.166592683	0.038	0.453	3.05E-60	10
TMEM176A.1	3.03E-64	2.048782912	0.378	0.138	6.97E-60	10
CELF2.7	3.16E-64	-1.287017465	0.032	0.44	7.27E-60	10
TPI1.7	3.51E-64	1.907167028	0.675	0.56	8.06E-60	10
ZFP36.6	7.37E-64	-1.238556937	0.221	0.68	1.69E-59	10
CD3D.10	8.45E-64	-1.611257187	0.068	0.49	1.94E-59	10
CCND1	1.22E-63	2.032321194	0.291	0.085	2.81E-59	10
XIST.6	2.57E-62	-1.629497017	0.038	0.438	5.91E-58	10
NUPR1.2	7.53E-62	2.021719752	0.355	0.126	1.73E-57	10
ELF1.8	1.68E-61	-1.225817671	0.05	0.455	3.86E-57	10
EEF1A1.6	2.48E-61	0.703462384	0.978	0.968	5.70E-57	10
LSP1.9	9.36E-60	-1.236485029	0.054	0.448	2.15E-55	10
TMEM176B.1	1.40E-59	2.114540221	0.378	0.146	3.22E-55	10
STK17B.6	1.46E-59	-1.341230775	0.03	0.414	3.36E-55	10
CFL1.2	1.47E-59	-0.870645888	0.299	0.763	3.37E-55	10
ITGB2.8	2.92E-59	-1.31189237	0.04	0.429	6.71E-55	10
LRRFIP1.3	4.04E-59	-1.195156516	0.038	0.426	9.28E-55	10
PTMA.3	2.19E-58	-0.777379653	0.627	0.928	5.02E-54	10
SLA.8	2.22E-58	-1.375057888	0.034	0.413	5.11E-54	10
ARPC1B.3	2.42E-58	-0.911728446	0.114	0.54	5.56E-54	10
RSRP1.7	3.76E-58	-1.148944556	0.042	0.426	8.65E-54	10
IL2RG.10	1.10E-57	-1.562427558	0.018	0.386	2.54E-53	10
CD3E.9	1.82E-57	-1.495737411	0.034	0.411	4.18E-53	10
TRAC.10	2.85E-57	-1.6792087	0.062	0.446	6.55E-53	10
ALDH1A1	3.28E-57	2.267027103	0.275	0.084	7.54E-53	10
GPSM3.6	4.45E-57	-1.143240321	0.028	0.403	1.02E-52	10
GZMA.9	7.41E-57	-1.804300328	0.054	0.433	1.70E-52	10
DUSP2.9	8.86E-57	-1.899791506	0.066	0.445	2.04E-52	10
DDX24.7	1.09E-56	-0.878459805	0.072	0.471	2.51E-52	10
CCNL1.8	1.33E-56	-1.093992547	0.036	0.412	3.05E-52	10
MYL12A.4	1.94E-56	-0.845177211	0.253	0.706	4.45E-52	10
PRRC2C.7	3.32E-56	-1.086250141	0.06	0.447	7.63E-52	10
IFI16.4	3.33E-56	-0.830070011	0.092	0.495	7.66E-52	10

CYBA.6	6.72E-56	-0.835343703	0.259	0.703	1.55E-51	10
SRSF5.6	1.02E-55	-0.500927541	0.185	0.638	2.34E-51	10
RP11-347P5.1.9	5.15E-55	-1.663980189	0.028	0.388	1.18E-50	10
RPL7.5	1.61E-54	0.829142414	0.863	0.922	3.69E-50	10
ITGB1.5	1.62E-54	-1.173643193	0.086	0.478	3.72E-50	10
RAC2.10	3.12E-54	-1.383126676	0.028	0.385	7.18E-50	10
FOSB.5	1.34E-53	-1.265178945	0.064	0.439	3.09E-49	10
DDX3X.10	2.60E-53	-1.211605441	0.06	0.428	5.97E-49	10
VAMP2.7	2.70E-53	-0.872480838	0.088	0.479	6.21E-49	10
WIPF1.10	4.61E-53	-1.249931168	0.012	0.355	1.06E-48	10
TRBC2.9	7.24E-53	-1.626795173	0.034	0.39	1.66E-48	10
FNBP1.9	7.75E-53	-1.1732081	0.028	0.38	1.78E-48	10
RNF213.7	7.82E-53	-1.188351015	0.04	0.4	1.80E-48	10
PFN1.4	1.05E-52	-0.757984627	0.341	0.776	2.40E-48	10
TPT1.7	1.35E-52	0.779263891	0.9	0.925	3.09E-48	10
RPS28.5	1.37E-52	0.616016601	0.93	0.948	3.15E-48	10
MBNL1.6	1.40E-52	-1.264628154	0.022	0.368	3.22E-48	10
AAK1.9	1.46E-52	-1.206561971	0.032	0.383	3.34E-48	10
CCL4.10	1.99E-52	-2.419394456	0.09	0.453	4.58E-48	10
PLIN2.7	3.42E-52	2.041366094	0.536	0.329	7.87E-48	10
TMA7.6	3.80E-52	-0.629796643	0.255	0.712	8.73E-48	10
CD48.8	4.86E-52	-1.117090637	0.034	0.388	1.12E-47	10
RHOA.6	1.31E-51	-0.928739808	0.131	0.522	3.01E-47	10
ANKRD12.6	1.37E-51	-0.855458177	0.042	0.401	3.15E-47	10
NKTR.7	3.31E-51	-1.231765798	0.018	0.359	7.61E-47	10
CLEC2B.5	6.25E-51	-0.85285877	0.038	0.391	1.44E-46	10
PPP2R5C.8	6.35E-51	-1.338336655	0.05	0.403	1.46E-46	10
HMGB2.6	1.14E-50	-0.982380303	0.108	0.49	2.63E-46	10
SEPT7.6	1.36E-50	-0.578995119	0.124	0.527	3.12E-46	10
FKBP5.6	1.40E-50	-1.156456888	0.052	0.408	3.22E-46	10
BTG2.6	1.67E-50	-1.350866925	0.026	0.369	3.83E-46	10
RPS6.3	1.99E-50	0.708332305	0.92	0.944	4.58E-46	10
H3F3B.6	2.09E-50	-0.810215092	0.438	0.849	4.81E-46	10
PEBP1.8	2.24E-50	2.337966306	0.546	0.384	5.14E-46	10
KMT2E.9	2.74E-50	-0.956351394	0.06	0.417	6.31E-46	10
CST7.9	6.28E-50	-1.674698006	0.058	0.401	1.44E-45	10
ATRX.6	9.84E-50	-1.016819193	0.046	0.399	2.26E-45	10
RPL3.5	1.18E-49	0.636452095	0.898	0.933	2.70E-45	10
IDS.7	1.25E-49	-1.226897361	0.044	0.385	2.88E-45	10
HLA-DPA1.10	1.32E-49	-1.703601724	0.201	0.584	3.04E-45	10
YWHAZ.8	1.46E-49	-0.667977608	0.118	0.508	3.35E-45	10
CD99.6	2.29E-49	-0.807275414	0.102	0.484	5.25E-45	10
SRSF2.9	2.36E-49	-0.885404921	0.074	0.436	5.42E-45	10
FUS.9	2.69E-49	-0.857009229	0.102	0.483	6.19E-45	10
DNAJB1.8	3.62E-49	-1.336494287	0.076	0.433	8.31E-45	10
YWHAB.3	3.86E-49	-0.790520118	0.116	0.501	8.88E-45	10
HNRNPA2B1.4	8.85E-49	-0.510704164	0.211	0.643	2.03E-44	10
ARL6IP5.7	9.47E-49	-0.555238429	0.092	0.471	2.18E-44	10
ARID4B.9	1.00E-48	-1.159674275	0.022	0.352	2.31E-44	10
RPLP0.6	1.03E-48	1.041591447	0.771	0.845	2.38E-44	10
WSB1.3	1.58E-48	-1.012485094	0.034	0.372	3.63E-44	10
PPP1R15A.6	1.70E-48	-1.099018526	0.064	0.415	3.90E-44	10
DDX17.6	2.84E-48	-0.876242826	0.06	0.411	6.52E-44	10
HLA-A.7	3.41E-48	-0.6538517	0.572	0.906	7.83E-44	10
KIAA1551.8	6.79E-48	-1.232344558	0.022	0.347	1.56E-43	10
GIMAP4.6	4.33E-47	-1.060912617	0.026	0.35	9.95E-43	10
EVI2B.5	5.09E-47	-1.039437929	0.024	0.347	1.17E-42	10
SMCHD1.4	9.46E-47	-1.148592067	0.02	0.339	2.17E-42	10

REL.6	1.32E-46	-1.24150731	0.018	0.333	3.03E-42	10
TRA2B.4	1.83E-46	-0.779672474	0.046	0.381	4.20E-42	10
SH3KBP1.7	4.37E-46	-1.097140429	0.01	0.319	1.00E-41	10
NKG7.10	6.75E-46	-1.970615428	0.106	0.439	1.55E-41	10
STK17A.9	7.47E-46	-1.122964154	0.046	0.378	1.72E-41	10
ACTR2.5	7.93E-46	-0.733328908	0.048	0.379	1.82E-41	10
PIK3IP1.9	8.03E-46	-1.060073686	0.042	0.37	1.85E-41	10
FOS.8	1.08E-45	-0.956857047	0.307	0.695	2.49E-41	10
TPM3.3	1.27E-45	-0.728922739	0.102	0.466	2.91E-41	10
RBM25.6	1.57E-45	-0.752580177	0.04	0.371	3.61E-41	10
ACAP1.10	1.83E-45	-1.22562972	0.028	0.344	4.20E-41	10
PCSK7.9	2.21E-45	-1.169369279	0.02	0.331	5.07E-41	10
ISG20.7	2.71E-45	-0.999459866	0.046	0.373	6.24E-41	10
SAMSN1.4	2.84E-45	-1.329017492	0.022	0.333	6.52E-41	10
ROCK1.5	3.21E-45	-0.912161306	0.026	0.342	7.37E-41	10
LIMD2.9	4.17E-45	-1.183319623	0.016	0.321	9.59E-41	10
ETS1.10	5.01E-45	-1.101400022	0.03	0.35	1.15E-40	10
HCLS1.5	6.80E-45	-1.171941261	0.022	0.333	1.56E-40	10
CTSS.8	6.91E-45	-1.32207211	0.048	0.374	1.59E-40	10
SYNE2.9	7.44E-45	-1.240406146	0.03	0.343	1.71E-40	10
CKLF.5	7.71E-45	-0.934432016	0.048	0.375	1.77E-40	10
MT-ND4L.6	8.75E-45	-0.89784378	0.048	0.372	2.01E-40	10
SP100.6	1.03E-44	-0.679990855	0.072	0.411	2.36E-40	10
UCP2.7	1.35E-44	-0.867841483	0.038	0.358	3.11E-40	10
COTL1.5	1.85E-44	-1.034503865	0.1	0.446	4.25E-40	10
CFLAR.7	1.90E-44	-0.794468475	0.088	0.435	4.37E-40	10
HLA-DPB1.9	3.48E-44	-1.494284644	0.277	0.645	8.01E-40	10
FAM49B.5	3.95E-44	-0.983091656	0.04	0.351	9.09E-40	10
IQGAP1.4	5.09E-44	-0.955254071	0.034	0.349	1.17E-39	10
ARF6.5	5.17E-44	-0.896446888	0.062	0.391	1.19E-39	10
SARAF.4	8.57E-44	-0.644053666	0.281	0.693	1.97E-39	10
ALOX5AP.7	1.23E-43	-1.335105645	0.03	0.34	2.82E-39	10
ZFP36L1.6	1.33E-43	-0.961331985	0.056	0.378	3.06E-39	10
PPP1R2.7	1.65E-43	-1.046002644	0.04	0.354	3.80E-39	10
GPBP1.8	1.92E-43	-0.922987494	0.042	0.357	4.41E-39	10
AKAP9.6	2.27E-43	-0.749212058	0.038	0.353	5.22E-39	10
GZMK.10	2.81E-43	-2.011222611	0.064	0.376	6.46E-39	10
ITM2A.10	2.92E-43	-1.42340478	0.02	0.321	6.70E-39	10
RGS2.9	6.68E-43	-1.484282907	0.038	0.346	1.53E-38	10
CCND3.5	6.80E-43	-0.742565206	0.05	0.371	1.56E-38	10
CAPZB.4	7.75E-43	-0.616601544	0.12	0.473	1.78E-38	10
SCAF11.5	1.59E-42	-0.693761162	0.054	0.372	3.64E-38	10
IRF1.7	2.68E-42	-1.201126187	0.024	0.321	6.16E-38	10
FAM133B.4	3.33E-42	-1.071057284	0.02	0.313	7.65E-38	10
GLIPR1.7	3.48E-42	-0.952868112	0.016	0.309	8.01E-38	10
PRDM1.6	9.31E-42	-1.418662753	0.014	0.302	2.14E-37	10
CD7.7	1.18E-41	-1.503973239	0.02	0.31	2.70E-37	10
BCLAF1.7	1.33E-41	-0.837611411	0.042	0.346	3.06E-37	10
TERF2IP.6	1.90E-41	-0.824659885	0.062	0.376	4.37E-37	10
TNRC6B.6	2.07E-41	-1.09459695	0.032	0.328	4.75E-37	10
DOCK8.8	3.94E-41	-1.023221204	0.016	0.302	9.06E-37	10
GIMAP7.9	4.88E-41	-1.313699127	0.012	0.295	1.12E-36	10
JAK1.6	7.59E-41	-0.546122882	0.08	0.407	1.74E-36	10
STAT3.7	8.61E-41	-0.825940982	0.032	0.329	1.98E-36	10
LCK.10	1.70E-40	-1.159325402	0.014	0.297	3.91E-36	10
CLEC2D.9	1.96E-40	-1.23981743	0.03	0.317	4.50E-36	10
GCC2.7	2.34E-40	-0.739234068	0.06	0.371	5.38E-36	10
RPS4X.6	2.60E-40	0.678978578	0.9	0.94	5.99E-36	10

CD3G.9	2.73E-40	-1.173840783	0.02	0.303	6.28E-36	10
CLK1.8	3.33E-40	-0.76606452	0.06	0.368	7.65E-36	10
MSN.4	5.03E-40	-0.693729705	0.064	0.377	1.16E-35	10
ARPC5.5	2.13E-39	-0.608666126	0.11	0.447	4.90E-35	10
PABPC1.5	2.59E-39	-0.529116606	0.275	0.677	5.96E-35	10
COX7C.2	3.91E-39	1.269082084	0.689	0.756	9.00E-35	10
CREM.6	4.14E-39	-1.250849086	0.016	0.29	9.51E-35	10
RARRES2.1	5.27E-39	1.594790133	0.279	0.106	1.21E-34	10
RGS10.6	6.69E-39	-0.949180546	0.026	0.305	1.54E-34	10
SEPT6.7	6.96E-39	-0.89929444	0.014	0.287	1.60E-34	10
EMB.7	7.30E-39	-1.104067164	0.01	0.279	1.68E-34	10
LGALS1.4	7.61E-39	-0.839801292	0.223	0.585	1.75E-34	10
C9orf16.2	1.01E-38	-0.664350117	0.06	0.36	2.32E-34	10
PIK3R1.7	1.63E-38	-0.880862593	0.034	0.318	3.74E-34	10
SMAP2.6	2.50E-38	-0.822744362	0.026	0.308	5.74E-34	10
CEBPB.8	2.97E-38	-1.129448363	0.046	0.339	6.83E-34	10
RAP1B.4	3.49E-38	-0.885233295	0.046	0.334	8.01E-34	10
MYO1F.5	3.55E-38	-0.921014239	0.012	0.281	8.17E-34	10
RPS8.5	3.83E-38	0.6703701	0.859	0.92	8.80E-34	10
PDCD4.4	3.84E-38	-0.97361729	0.034	0.314	8.83E-34	10
DAB2.2	3.99E-38	2.134367233	0.333	0.156	9.18E-34	10
HLA-DRB1.10	4.55E-38	-1.485716135	0.263	0.603	1.05E-33	10
LTB.9	5.11E-38	-1.317434036	0.032	0.311	1.18E-33	10
ADGRE5.8	6.57E-38	-1.096037114	0.032	0.309	1.51E-33	10
TRBC1.9	8.38E-38	-1.487140353	0.072	0.364	1.93E-33	10
EVI2A.6	9.78E-38	-0.986468662	0.02	0.29	2.25E-33	10
GNAS.4	1.23E-37	-0.741068554	0.06	0.355	2.83E-33	10
SYTL3.9	1.30E-37	-1.203291005	0.018	0.287	2.99E-33	10
RBPJ.5	1.34E-37	-1.068806798	0.048	0.331	3.08E-33	10
VMP1.6	1.35E-37	-0.793783299	0.042	0.33	3.09E-33	10
MYH9.6	1.47E-37	-0.723455288	0.038	0.321	3.38E-33	10
TSC22D3.7	1.70E-37	-0.768252753	0.412	0.794	3.90E-33	10
HSPB1.8	2.22E-37	1.482124958	0.574	0.429	5.11E-33	10
SF3B1.3	2.79E-37	-0.729427168	0.046	0.336	6.41E-33	10
TXN.7	3.53E-37	2.430725141	0.512	0.384	8.11E-33	10
IL7R.8	3.85E-37	-1.872706217	0.04	0.313	8.84E-33	10
CTSC.8	1.11E-36	-0.628229577	0.062	0.357	2.54E-32	10
SF1.6	1.65E-36	-0.629442773	0.04	0.321	3.79E-32	10
RPL31.7	3.18E-36	0.720609835	0.837	0.909	7.30E-32	10
MYCBP2.4	3.62E-36	-0.904375497	0.012	0.267	8.32E-32	10
FOXP1.5	4.16E-36	-1.074135175	0.012	0.267	9.55E-32	10
SRRM2.4	4.69E-36	-0.555095896	0.064	0.351	1.08E-31	10
LITAF.7	7.54E-36	-0.650403024	0.08	0.378	1.73E-31	10
CARD16.5	1.01E-35	-0.855253929	0.052	0.327	2.33E-31	10
ATM.6	1.43E-35	-0.906360652	0.008	0.258	3.28E-31	10
RPL36.8	1.89E-35	0.727730162	0.841	0.915	4.35E-31	10
NR4A2.7	1.93E-35	-1.191621782	0.026	0.286	4.42E-31	10
ANKRD44.7	3.13E-35	-0.917742252	0.014	0.267	7.19E-31	10
HLA-DQB1.8	3.22E-35	-1.147125088	0.108	0.403	7.40E-31	10
PRKCH.7	4.49E-35	-0.975477042	0.012	0.262	1.03E-30	10
PPP1R12A.5	4.61E-35	-0.666556774	0.026	0.288	1.06E-30	10
IFITM2.7	6.30E-35	-0.634895149	0.157	0.487	1.45E-30	10
APOBEC3G.8	6.46E-35	-0.996969143	0.022	0.281	1.48E-30	10
MACF1.6	8.56E-35	-0.940039027	0.016	0.266	1.97E-30	10
DEK.3	1.08E-34	-0.544056992	0.082	0.375	2.47E-30	10
RPS15.6	1.16E-34	0.597911125	0.898	0.933	2.66E-30	10
IER2.5	2.28E-34	-0.582195747	0.102	0.412	5.25E-30	10
ODF2L.5	4.06E-34	-1.078772323	0.02	0.268	9.33E-30	10

AKNA.7	4.97E-34	-0.892909612	0.02	0.27	1.14E-29	10
CD27.9	5.79E-34	-1.206768916	0.024	0.276	1.33E-29	10
IL10RA.7	5.83E-34	-0.92517284	0.02	0.267	1.34E-29	10
CD74.8	8.13E-34	-1.406354133	0.504	0.805	1.87E-29	10
NACA.3	8.79E-34	0.948248709	0.717	0.807	2.02E-29	10
ACTR3.3	9.21E-34	-0.551505298	0.098	0.392	2.12E-29	10
RGCC.9	9.78E-34	-1.223761161	0.05	0.317	2.25E-29	10
TAPBP.6	1.14E-33	-0.51397596	0.058	0.335	2.62E-29	10
IKZF1.9	1.58E-33	-0.953796527	0.012	0.252	3.63E-29	10
EPC1.8	1.91E-33	-0.785354054	0.028	0.279	4.39E-29	10
SFPQ.4	2.43E-33	-0.533122515	0.05	0.321	5.59E-29	10
CMC1.8	2.75E-33	-1.401020964	0.038	0.294	6.31E-29	10
BIN2.7	3.37E-33	-1.005885522	0.016	0.258	7.75E-29	10
RNF19A.9	4.20E-33	-1.044844661	0.034	0.286	9.66E-29	10
RCSD1.7	4.43E-33	-0.816603651	0.016	0.258	1.02E-28	10
PDK4.4	4.44E-33	1.849618426	0.371	0.197	1.02E-28	10
MGEA5.7	4.56E-33	-0.628062326	0.024	0.274	1.05E-28	10
NFKBIA.8	4.82E-33	-0.643199371	0.227	0.57	1.11E-28	10
GABPB1.6	4.98E-33	-0.776688572	0.036	0.289	1.14E-28	10
PTP4A2.5	7.14E-33	-0.848901746	0.03	0.28	1.64E-28	10
GSTP1.5	9.18E-33	1.669868827	0.56	0.486	2.11E-28	10
TLN1.3	1.36E-32	-0.582996729	0.026	0.274	3.13E-28	10
LINC00152.6	1.55E-32	-0.830156971	0.062	0.326	3.57E-28	10
ABRACL.3	2.13E-32	-0.646630409	0.042	0.295	4.90E-28	10
S100A6.5	3.16E-32	-0.674919468	0.404	0.78	7.26E-28	10
YPEL5.8	3.81E-32	-0.566260157	0.078	0.354	8.75E-28	10
HSP90AA1.6	4.74E-32	-0.615533841	0.297	0.667	1.09E-27	10
SLC2A3.5	5.82E-32	-1.083675313	0.03	0.273	1.34E-27	10
CD96.10	5.96E-32	-0.884146414	0.02	0.257	1.37E-27	10
PSMA3-AS1.8	7.25E-32	-0.727297593	0.036	0.284	1.67E-27	10
TAOK3.5	8.02E-32	-0.617408416	0.03	0.274	1.84E-27	10
HLA-DQA1.7	9.41E-32	-1.557966319	0.092	0.353	2.16E-27	10
PYCARD.6	1.45E-31	-0.721524035	0.018	0.255	3.33E-27	10
ICAM3.9	1.57E-31	-0.800316843	0.036	0.281	3.61E-27	10
PNN.6	2.02E-31	-0.60238472	0.054	0.313	4.65E-27	10
ARHGEF1.9	2.28E-31	-0.663070127	0.028	0.271	5.24E-27	10
HP1BP3.4	2.45E-31	-0.626833817	0.044	0.294	5.63E-27	10
CCNH.7	2.68E-31	-0.79862648	0.044	0.293	6.15E-27	10
IFNGR1.7	3.40E-31	-0.900796743	0.028	0.267	7.82E-27	10
WNK1.6	3.42E-31	-0.662927443	0.022	0.257	7.86E-27	10
ORMDL1.1	7.94E-31	-0.773541543	0.026	0.262	1.82E-26	10
CDC42SE2.5	9.34E-31	-0.717638034	0.066	0.324	2.15E-26	10
C10orf54.7	9.38E-31	-0.607329664	0.042	0.29	2.16E-26	10
JMJD1C.3	1.24E-30	-0.74922575	0.03	0.266	2.86E-26	10
USP15.3	3.09E-30	-0.598236956	0.034	0.271	7.09E-26	10
TRAF3IP3.10	4.89E-30	-0.649872339	0.03	0.264	1.12E-25	10
RNPS1.4	6.12E-30	-0.710414309	0.034	0.27	1.41E-25	10
TRIM22.5	6.54E-30	-0.615926893	0.026	0.256	1.50E-25	10
RPL35.4	7.74E-30	0.669066444	0.801	0.901	1.78E-25	10
TYROBP.10	8.13E-30	-1.30268026	0.135	0.397	1.87E-25	10
VIM.8	9.87E-30	0.989696461	0.739	0.772	2.27E-25	10
PSMB10.3	2.83E-29	-0.693289944	0.026	0.254	6.51E-25	10
BTN3A2.7	5.16E-29	-0.773988122	0.04	0.271	1.18E-24	10
RPL11.2	7.43E-29	0.515130206	0.88	0.94	1.71E-24	10
S100A10.4	7.80E-29	1.230151717	0.637	0.609	1.79E-24	10
PKM.5	1.41E-28	1.744859722	0.518	0.43	3.24E-24	10
TUBA1A.2	2.41E-28	-0.540663663	0.088	0.347	5.55E-24	10
FCER1G.10	3.26E-28	-1.191105355	0.07	0.312	7.48E-24	10

RPS5.4	1.39E-27	0.70951683	0.753	0.845	3.20E-23	10
BRD2.5	1.81E-27	-0.644777529	0.034	0.255	4.16E-23	10
DDX6.7	3.10E-27	-0.690547309	0.036	0.254	7.13E-23	10
CASP4.3	3.71E-27	-0.524200822	0.062	0.298	8.52E-23	10
C9orf142.7	3.89E-27	-0.605083191	0.044	0.269	8.94E-23	10
HNRNPH1.3	6.07E-27	-0.590607311	0.08	0.329	1.39E-22	10
SEPT9.7	7.12E-27	-0.552965503	0.038	0.258	1.64E-22	10
NDUFC1.8	9.21E-27	1.890690342	0.402	0.266	2.12E-22	10
RPL37.4	9.63E-27	0.61321549	0.839	0.911	2.21E-22	10
RPL8.2	9.76E-27	0.654198093	0.781	0.886	2.24E-22	10
RHOG.4	2.22E-25	-0.578774497	0.054	0.272	5.10E-21	10
PLP2.5	5.34E-25	-0.502479906	0.072	0.303	1.23E-20	10
GYPC.7	1.07E-24	-0.552837878	0.084	0.315	2.45E-20	10
RPL26.3	1.07E-24	0.52974089	0.865	0.938	2.46E-20	10
FYN.6	1.56E-24	-0.535924356	0.056	0.273	3.58E-20	10
AES.4	1.71E-24	-0.55046903	0.048	0.259	3.94E-20	10
PSAP.9	5.05E-24	-0.545083163	0.203	0.478	1.16E-19	10
BIRC3.8	1.05E-23	-0.884563849	0.066	0.28	2.42E-19	10
TPM1.1	6.38E-23	1.339801408	0.335	0.189	1.47E-18	10
RPL19.4	7.04E-23	0.50271715	0.867	0.927	1.62E-18	10
HLA-DMA.8	2.79E-22	-0.505101396	0.082	0.295	6.42E-18	10
GPX4.8	8.31E-21	1.622552823	0.496	0.452	1.91E-16	10
RHOB.8	3.83E-19	1.284674195	0.454	0.345	8.81E-15	10
RPS25.6	4.10E-19	0.517777729	0.835	0.921	9.43E-15	10
SAT1.8	3.36E-18	-0.627810985	0.365	0.641	7.72E-14	10
AIF1.10	1.12E-17	-0.769903177	0.084	0.266	2.58E-13	10
HLA-DRB5.9	1.84E-17	-0.874080815	0.175	0.381	4.23E-13	10
GNLY.10	2.16E-16	-1.845069039	0.092	0.259	4.96E-12	10
ESD.1	2.27E-16	1.735802844	0.329	0.232	5.21E-12	10
SERPINA1.9	3.50E-16	1.734981331	0.376	0.275	8.04E-12	10
LYZ.9	8.26E-16	-1.419180488	0.175	0.358	1.90E-11	10
NPM1.4	1.09E-15	1.159502178	0.598	0.702	2.51E-11	10
HLA-DRA.10	2.02E-15	-1.380769458	0.45	0.681	4.64E-11	10
RPS7.6	2.42E-15	0.512171241	0.779	0.889	5.55E-11	10
C1QA.10	3.03E-15	-0.999819361	0.092	0.256	6.96E-11	10
RPL22.4	3.41E-15	0.729138397	0.667	0.816	7.83E-11	10
HBB.6	5.45E-15	-0.928680645	0.183	0.399	1.25E-10	10
OCIAD2.7	6.68E-15	1.510116339	0.347	0.258	1.53E-10	10
ACAA2	5.64E-14	1.668554806	0.255	0.16	1.30E-09	10
RPL5.3	5.86E-14	0.577141368	0.729	0.857	1.35E-09	10
RPL24.5	2.34E-13	0.629098265	0.681	0.859	5.38E-09	10
RPL38.4	5.87E-13	0.52543795	0.763	0.871	1.35E-08	10
GPX3.1	1.51E-12	1.656350287	0.255	0.162	3.47E-08	10
CST3.9	5.57E-12	-0.76930429	0.229	0.391	1.28E-07	10
UQCRB.1	9.38E-12	1.011804302	0.608	0.749	2.16E-07	10
HBA2.8	1.29E-11	-0.998170003	0.108	0.256	2.96E-07	10
RPS11.4	2.10E-11	0.50342613	0.699	0.861	4.82E-07	10
PPDPF.6	2.76E-11	1.162642108	0.558	0.663	6.34E-07	10
RPL23.3	3.00E-11	0.625472613	0.655	0.823	6.90E-07	10
UBC.4	5.20E-11	0.764222204	0.697	0.845	1.20E-06	10
TMEM59.2	2.19E-10	0.505144124	0.251	0.485	5.04E-06	10
LAMTOR5.6	1.34E-09	1.620760214	0.378	0.344	3.09E-05	10
RPL36A.5	3.73E-09	0.691856765	0.649	0.807	8.57E-05	10
GNB2L1.1	6.55E-09	0.644950131	0.683	0.841	0.000150427	10
TMBIM6.2	7.18E-09	0.60258382	0.271	0.503	0.00016507	10
UQCRQ.7	9.49E-09	1.481856313	0.442	0.462	0.000218178	10
PRDX1.5	1.22E-08	1.235920493	0.418	0.403	0.000279541	10
PFDN5.2	1.25E-08	0.713875062	0.616	0.79	0.000287066	10

USMG5.4	2.26E-08	1.376585843	0.474	0.53	0.000518314	10
DSTN.8	3.15E-08	1.139799257	0.357	0.306	0.000724284	10
PRDX6.4	4.20E-08	1.458237429	0.353	0.324	0.00096554	10
SPP1.5	5.93E-08	0.565541949	0.289	0.214	0.001362071	10
MPC2.6	8.94E-08	1.442443474	0.309	0.263	0.002055409	10
OCIAD1.2	1.06E-07	0.518036952	0.139	0.274	0.002443429	10
IGKC.7	1.63E-07	-2.342822028	0.159	0.301	0.003756354	10
CUTA.4	2.18E-07	0.549951793	0.235	0.423	0.00499926	10
CNDP2.1	3.29E-07	1.532127063	0.263	0.211	0.007562491	10
COX17.4	3.48E-07	0.510807044	0.173	0.323	0.008001007	10
S100A9.9	3.71E-07	-1.21451615	0.143	0.255	0.008521403	10
PSMA4.2	3.83E-07	0.651092882	0.179	0.33	0.008801003	10
ATOX1.2	4.52E-07	1.506758984	0.273	0.222	0.010390305	10
ST13.6	5.11E-07	1.42890823	0.376	0.372	0.01174312	10
PGAM1.5	5.90E-07	1.497503751	0.371	0.36	0.013549481	10
CHCHD10.8	8.27E-07	1.414504421	0.287	0.245	0.019006718	10
SEC62.8	1.04E-06	0.620691655	0.247	0.432	0.023832278	10
APOC1.8	1.05E-06	-0.635208773	0.173	0.288	0.024201672	10
C19orf53.1	1.21E-06	0.52595579	0.225	0.394	0.027785988	10
ZNHIT1.2	1.22E-06	0.521458279	0.155	0.287	0.028098227	10
SLC25A5.4	1.23E-06	0.511074836	0.221	0.392	0.028215925	10
NDUFB9.1	1.28E-06	0.601010389	0.187	0.338	0.029383559	10
COX7A2L	1.31E-06	0.551764275	0.185	0.336	0.030134574	10
PSMB6.1	1.76E-06	0.596564632	0.155	0.282	0.040442935	10
ENPP2.2	0	4.185240006	0.889	0.1	0	11
SPARCL1.2	0	4.113805923	0.987	0.136	0	11
FLT1.1	0	4.055228066	0.942	0.042	0	11
ESM1.1	0	3.83673901	0.96	0.097	0	11
INSR.3	0	3.745767832	0.956	0.109	0	11
PLVAP.1	0	3.55681695	0.965	0.067	0	11
TIMP3.1	0	3.554019479	0.933	0.066	0	11
SPARC.2	0	3.408221015	0.989	0.156	0	11
A2M.2	0	3.335794759	0.945	0.171	0	11
IGFBP3.10	0	3.242626405	0.889	0.223	0	11
VWF	0	3.211145213	0.911	0.061	0	11
GSN.4	0	3.17353076	0.971	0.2	0	11
EPAS1.1	0	3.17069619	0.936	0.088	0	11
PLPP1.1	0	3.156091258	0.931	0.07	0	11
STC1	0	3.117279786	0.796	0.046	0	11
NOTCH4	0	3.108152969	0.92	0.033	0	11
IGFBP7.10	0	3.091457973	0.998	0.358	0	11
PRSS23.1	0	3.054908088	0.909	0.088	0	11
ADGRL4.1	0	3.013016306	0.925	0.027	0	11
CLEC14A.1	0	3.010740472	0.894	0.029	0	11
PECAM1.3	0	2.978452959	0.96	0.175	0	11
EDNRB	0	2.97383817	0.845	0.037	0	11
HSPG2	0	2.97169207	0.84	0.037	0	11
COL4A1	0	2.926259443	0.765	0.046	0	11
APP.3	0	2.873451087	0.945	0.153	0	11
SPRY1.1	0	2.777939994	0.86	0.087	0	11
RBP7.1	0	2.765710352	0.758	0.058	0	11
SDPR.1	0	2.76309349	0.829	0.055	0	11
PLPP3	0	2.755658513	0.747	0.039	0	11
CD59.4	0	2.717384684	0.971	0.208	0	11
BTNL9	0	2.679735574	0.756	0.02	0	11
MGP.2	0	2.669689047	0.878	0.126	0	11
RNASE1.1	0	2.64809429	0.867	0.067	0	11
INHBB	0	2.630217319	0.723	0.012	0	11

SLC9A3R2.1	0	2.619608574	0.814	0.041	0	11
AQP1	0	2.611881123	0.667	0.04	0	11
RAMP2.1	0	2.604209922	0.863	0.04	0	11
TCF4.1	0	2.601686351	0.847	0.095	0	11
GNG11.1	0	2.598633494	0.92	0.118	0	11
ENG.1	0	2.551978587	0.816	0.084	0	11
EFNB2	0	2.5459659	0.716	0.013	0	11
ANGPT2	0	2.519264017	0.701	0.051	0	11
CLDN5	0	2.49594077	0.534	0.026	0	11
TSC22D1.2	0	2.489428729	0.911	0.159	0	11
IFI27.1	0	2.472192603	0.925	0.129	0	11
ADGRF5	0	2.463596974	0.741	0.021	0	11
ITGA6	0	2.445334951	0.747	0.049	0	11
ARHGAP29	0	2.431245423	0.843	0.044	0	11
SPTBN1	0	2.403075075	0.834	0.079	0	11
COL4A2	0	2.367834151	0.696	0.042	0	11
PODXL	0	2.343564217	0.667	0.015	0	11
HES1.1	0	2.339954469	0.65	0.066	0	11
BCAM	0	2.325001022	0.772	0.043	0	11
PTPRB	0	2.31273466	0.683	0.016	0	11
FAM84A	0	2.311229053	0.659	0.017	0	11
EGFL7	0	2.254318843	0.765	0.03	0	11
ESAM	0	2.215030846	0.778	0.04	0	11
NFIB	0	2.21248297	0.78	0.049	0	11
GJA1	0	2.201174841	0.667	0.013	0	11
HYAL2	0	2.198712927	0.736	0.049	0	11
LIFR	0	2.195877139	0.67	0.023	0	11
PDGFD	0	2.178606632	0.743	0.029	0	11
RAMP3	0	2.158192976	0.678	0.03	0	11
F8	0	2.158061102	0.588	0.026	0	11
CALCRL	0	2.141969207	0.705	0.025	0	11
SEC14L1.1	0	2.133803669	0.827	0.167	0	11
TM4SF1	0	2.122311128	0.647	0.042	0	11
CDH13	0	2.063756624	0.627	0.017	0	11
APOLD1	0	2.063033426	0.729	0.081	0	11
KCNE3	0	2.033589027	0.568	0.037	0	11
CD93.1	0	2.022469325	0.652	0.066	0	11
CRIP2.1	0	2.001357977	0.765	0.08	0	11
EMCN	0	1.975776051	0.594	0.019	0	11
GRB10	0	1.974883253	0.634	0.017	0	11
EMP1	0	1.966192112	0.599	0.025	0	11
TMEM204	0	1.963396801	0.754	0.058	0	11
LDB2	0	1.95084653	0.701	0.029	0	11
FSTL1	0	1.936095892	0.645	0.031	0	11
PCAT19	0	1.924464678	0.656	0.024	0	11
CD34	0	1.921926485	0.652	0.021	0	11
ITGA1	0	1.914611566	0.723	0.097	0	11
EFNA1	0	1.912330721	0.718	0.047	0	11
NRP1	0	1.884070823	0.665	0.052	0	11
CDH5	0	1.8767691	0.605	0.012	0	11
FCN3	0	1.855251159	0.353	0.005	0	11
TMEM88	0	1.851872828	0.61	0.022	0	11
UACA	0	1.851359664	0.619	0.052	0	11
KDR	0	1.838464593	0.517	0.006	0	11
VWA1	0	1.832473263	0.543	0.014	0	11
CA2	0	1.829398312	0.554	0.038	0	11
MMRN2	0	1.805860711	0.61	0.012	0	11
F2RL3	0	1.804801651	0.508	0.008	0	11

ID3.1	0	1.800681487	0.769	0.119	0	11
WWTR1	0	1.78421892	0.623	0.038	0	11
DOCK9	0	1.782513727	0.625	0.027	0	11
S1PR1	0	1.771395119	0.616	0.029	0	11
CAV1.2	0	1.761834776	0.812	0.115	0	11
PTRF	0	1.749890897	0.701	0.061	0	11
OSBPL1A	0	1.748819854	0.581	0.058	0	11
FILIP1	0	1.747761534	0.561	0.021	0	11
MTUS1	0	1.730223461	0.583	0.042	0	11
CX3CL1	0	1.726768504	0.521	0.013	0	11
TSPAN13	0	1.720378722	0.583	0.016	0	11
SMTN	0	1.709319845	0.568	0.028	0	11
IGFBP4	0	1.705045592	0.745	0.097	0	11
SORBS2	0	1.698903502	0.574	0.029	0	11
DLL4	0	1.691909557	0.554	0.007	0	11
HTRA1.1	0	1.691867577	0.636	0.092	0	11
CRIM1	0	1.680263525	0.599	0.063	0	11
COL8A1	0	1.665330301	0.554	0.019	0	11
MAGI1	0	1.661434616	0.532	0.011	0	11
NTN4	0	1.65512044	0.492	0.016	0	11
LAMC1	0	1.644210819	0.572	0.023	0	11
JAG1	0	1.62643909	0.53	0.032	0	11
COL15A1	0	1.620934748	0.375	0.006	0	11
TMEM47	0	1.619060401	0.583	0.027	0	11
TSPAN7	0	1.618658667	0.51	0.017	0	11
SLCO2A1	0	1.59155818	0.424	0.013	0	11
IDO1	0	1.583001508	0.388	0.017	0	11
RAPGEF5	0	1.580243597	0.528	0.018	0	11
NPDC1	0	1.570323929	0.603	0.044	0	11
TMEM150C	0	1.567784321	0.494	0.009	0	11
CNN3.1	0	1.543996253	0.721	0.087	0	11
PINK1	0	1.535047281	0.572	0.042	0	11
UNC5B	0	1.523008306	0.481	0.013	0	11
TJP1	0	1.515745037	0.53	0.022	0	11
MYO6	0	1.510559734	0.568	0.04	0	11
CYYR1	0	1.501195961	0.517	0.013	0	11
S100A16	0	1.499804049	0.643	0.063	0	11
PALMD	0	1.480022102	0.512	0.015	0	11
TM4SF18	0	1.471440827	0.466	0.022	0	11
TMTC1	0	1.466257922	0.443	0.011	0	11
LAMA4	0	1.4544792	0.506	0.019	0	11
PLXND1	0	1.452442539	0.525	0.048	0	11
PPFIBP1	0	1.448388675	0.545	0.038	0	11
PTPRK	0	1.445227026	0.488	0.021	0	11
FAM167B	0	1.442264422	0.448	0.021	0	11
PLK2	0	1.437213905	0.499	0.045	0	11
NOSTRIN	0	1.43268869	0.47	0.021	0	11
COX7A1	0	1.431529715	0.61	0.05	0	11
TIE1	0	1.406740809	0.477	0.011	0	11
CXorf36	0	1.401565718	0.506	0.016	0	11
ANKRD26	0	1.397919337	0.517	0.054	0	11
THSD7A	0	1.394301912	0.399	0.01	0	11
FZD4	0	1.377246881	0.446	0.011	0	11
SULF1	0	1.360044842	0.344	0.012	0	11
NID1	0	1.353599635	0.455	0.017	0	11
GAS6	0	1.350380636	0.525	0.06	0	11
HEY1	0	1.342818015	0.437	0.009	0	11
TNS2	0	1.339742426	0.497	0.027	0	11

DOCK6	0	1.303635154	0.459	0.014	0	11
LIMCH1	0	1.294145652	0.421	0.018	0	11
DKK3	0	1.280763295	0.494	0.036	0	11
ECSCR	0	1.280249884	0.683	0.024	0	11
MATN2	0	1.267604963	0.37	0.01	0	11
ARL15	0	1.258676832	0.461	0.032	0	11
RPS6KA2	0	1.256100964	0.452	0.021	0	11
PTPRG	0	1.254660995	0.406	0.021	0	11
EMP2	0	1.245145315	0.466	0.027	0	11
ROBO4	0	1.235567277	0.415	0.009	0	11
FAM107A	0	1.233701825	0.397	0.011	0	11
KCNN3	0	1.231383747	0.399	0.007	0	11
HES4	0	1.223958637	0.446	0.033	0	11
FGFR1	0	1.222994247	0.424	0.027	0	11
GALNT15	0	1.213987135	0.392	0.012	0	11
SASH1	0	1.211816973	0.452	0.024	0	11
ANGPTL2	0	1.209679894	0.373	0.01	0	11
LNX1	0	1.19394033	0.368	0.009	0	11
ACVRL1	0	1.177184683	0.435	0.014	0	11
LAMB2	0	1.172657278	0.428	0.031	0	11
ECE1	0	1.168037974	0.455	0.033	0	11
MECOM	0	1.164242485	0.379	0.006	0	11
MYCT1	0	1.161890405	0.424	0.007	0	11
GALNT18	0	1.161845299	0.419	0.012	0	11
CAV2	0	1.159125863	0.548	0.061	0	11
ERG	0	1.157891366	0.386	0.008	0	11
TMEM255B	0	1.153702278	0.43	0.021	0	11
LRRC8A	0	1.152530063	0.401	0.024	0	11
PCDH17	0	1.139187759	0.333	0.006	0	11
SOX18	0	1.134152431	0.381	0.005	0	11
JAG2	0	1.132997549	0.364	0.007	0	11
PTPN14	0	1.130535346	0.384	0.024	0	11
DYSF	0	1.10325283	0.37	0.009	0	11
ITIH5	0	1.090065783	0.339	0.019	0	11
NCKAP1	0	1.084250294	0.41	0.026	0	11
TMEM233	0	1.083355005	0.346	0.006	0	11
GPIHBP1	0	1.063556394	0.288	0.005	0	11
LAMA5	0	1.055549538	0.355	0.019	0	11
MPZL2	0	1.052369517	0.379	0.025	0	11
PREX2	0	1.04908244	0.357	0.013	0	11
RBMS3	0	1.046543628	0.364	0.018	0	11
NUAK1	0	1.028348376	0.346	0.013	0	11
SCARF1	0	1.024002814	0.359	0.016	0	11
PXDN	0	1.012652492	0.306	0.007	0	11
LAMB1	0	0.999609723	0.344	0.009	0	11
IL3RA	0	0.998421802	0.361	0.012	0	11
DPYSL3	0	0.991775083	0.313	0.005	0	11
BTBD3	0	0.989005945	0.361	0.018	0	11
RASIP1	0	0.983762194	0.364	0.009	0	11
PDZD2	0	0.982731338	0.306	0.013	0	11
HOXD9	0	0.966032073	0.324	0.009	0	11
CMTM8	0	0.952493864	0.35	0.022	0	11
MPDZ	0	0.950410038	0.31	0.009	0	11
PLXNA2	0	0.94927171	0.33	0.009	0	11
ACE	0	0.945956343	0.253	0.006	0	11
EVA1B	0	0.936111741	0.361	0.022	0	11
FOLH1	0	0.92493401	0.259	0.003	0	11
IPO11	0	0.921601862	0.377	0.028	0	11

HIF3A	0	0.915760052	0.271	0.006	0	11
LRRC32	0	0.91055168	0.275	0.012	0	11
PLS3	0	0.908775564	0.361	0.025	0	11
RAPGEF4	0	0.895536909	0.282	0.005	0	11
PLEKHG1	0	0.890434867	0.299	0.011	0	11
JAM3	0	0.886629521	0.302	0.007	0	11
GAS2L1	0	0.884980483	0.33	0.02	0	11
TANC1	0	0.884739145	0.322	0.013	0	11
PIK3R3	0	0.884214565	0.45	0.023	0	11
ABCG2	0	0.88319989	0.288	0.01	0	11
ITGB5	0	0.88076734	0.31	0.016	0	11
MYO10	0	0.87760649	0.313	0.01	0	11
AFAP1L1	0	0.868245568	0.279	0.006	0	11
MCF2L	0	0.865127277	0.31	0.016	0	11
SHE	0	0.854671623	0.282	0.004	0	11
HECW2	0	0.84985895	0.284	0.004	0	11
LIMS2	0	0.848844296	0.33	0.014	0	11
EML1	0	0.837153526	0.275	0.013	0	11
SEMA3F	0	0.82460547	0.255	0.007	0	11
FGD5	0	0.823441946	0.288	0.006	0	11
RAI14	0	0.808622539	0.286	0.014	0	11
BOC	0	0.806937321	0.271	0.004	0	11
RAPGEF3	0	0.798790567	0.273	0.008	0	11
HOXD8	0	0.775961502	0.302	0.016	0	11
PLD1	0	0.772663258	0.286	0.014	0	11
FLT4	0	0.763559054	0.257	0.005	0	11
GPR4	0	0.725729356	0.264	0.006	0	11
SPRY4	0	0.648228722	0.262	0.006	0	11
COL18A1	1.80E-307	1.261928206	0.514	0.056	4.13E-303	11
CDC42BPA	1.21E-306	0.976346375	0.388	0.031	2.79E-302	11
ZNF704	1.55E-306	0.789026226	0.277	0.014	3.56E-302	11
ATOH8	8.77E-305	0.826337625	0.253	0.011	2.02E-300	11
GRAMD3	2.37E-304	1.25367714	0.439	0.041	5.45E-300	11
JUP	6.15E-301	1.068624255	0.373	0.029	1.41E-296	11
SERPINE1	1.47E-297	1.469088687	0.426	0.039	3.37E-293	11
LAYN	1.54E-296	0.943978989	0.346	0.024	3.54E-292	11
ACKR3	6.84E-294	0.930085303	0.271	0.014	1.57E-289	11
FNBP1L	4.73E-293	0.990245056	0.359	0.027	1.09E-288	11
CALD1	2.76E-292	1.01386139	0.769	0.129	6.35E-288	11
MYO1B	1.60E-291	0.935803239	0.333	0.022	3.67E-287	11
FKBP9	2.12E-290	0.81461115	0.317	0.021	4.88E-286	11
MCAM	2.34E-290	1.424166611	0.428	0.04	5.37E-286	11
TNFRSF4	2.96E-288	1.250297124	0.508	0.057	6.79E-284	11
PLLP	2.33E-286	0.877916407	0.302	0.018	5.36E-282	11
PVRL2	2.87E-285	1.172627897	0.452	0.048	6.59E-281	11
EHD4	1.72E-284	1.301481833	0.514	0.063	3.96E-280	11
RASGRP3	9.18E-280	1.05887016	0.377	0.032	2.11E-275	11
MGLL	2.03E-276	1.182788201	0.492	0.058	4.67E-272	11
TACC1.1	7.93E-275	2.008220855	0.831	0.209	1.82E-270	11
ITPRIP	8.54E-271	1.179823463	0.463	0.052	1.96E-266	11
NOTCH1	1.03E-270	0.930584119	0.317	0.022	2.37E-266	11
SYNPO	2.47E-269	0.848881309	0.288	0.018	5.67E-265	11
MGST2.2	1.36E-268	1.617973082	0.725	0.147	3.12E-264	11
MEF2C.2	2.06E-265	1.613993926	0.71	0.132	4.73E-261	11
HEG1	2.02E-264	1.083213527	0.355	0.029	4.64E-260	11
RPGR	5.60E-264	1.415870679	0.455	0.052	1.29E-259	11
RAB13.2	1.11E-260	1.350172747	0.667	0.119	2.55E-256	11
TSPAN12	1.16E-260	0.865489985	0.361	0.031	2.67E-256	11

GNAI1	2.41E-259	0.760909501	0.271	0.016	5.55E-255	11
SHROOM4	2.31E-253	0.636062797	0.255	0.014	5.30E-249	11
SERPINI1	2.06E-252	1.266844208	0.328	0.026	4.74E-248	11
FERMT2	1.26E-251	0.76984903	0.328	0.026	2.90E-247	11
PTPRM	1.61E-249	0.929622078	0.366	0.034	3.70E-245	11
PPIC	9.97E-249	0.7064271	0.306	0.023	2.29E-244	11
ITGA5	1.10E-248	1.151514877	0.463	0.057	2.53E-244	11
MLLT4	9.10E-248	0.88606494	0.306	0.023	2.09E-243	11
EPB41L4A	3.00E-246	0.778054035	0.288	0.02	6.89E-242	11
A4GALT	5.89E-245	0.737688146	0.29	0.021	1.35E-240	11
KIAA1147	2.89E-244	1.026374714	0.341	0.03	6.65E-240	11
IFITM3.10	1.05E-243	2.088020136	0.962	0.359	2.42E-239	11
TSPAN15	3.46E-243	0.70064811	0.257	0.016	7.95E-239	11
TTC28	1.19E-240	0.772506291	0.273	0.018	2.73E-236	11
ARHGEF10	7.20E-240	0.801878201	0.271	0.018	1.66E-235	11
FRY	6.96E-238	0.77556622	0.264	0.017	1.60E-233	11
RAPGEF2	7.47E-237	0.94209255	0.328	0.029	1.72E-232	11
SIPA1L2	3.97E-236	0.875160361	0.335	0.03	9.11E-232	11
TGM2	1.54E-235	1.155228085	0.426	0.05	3.53E-231	11
MAP3K6	6.80E-234	0.816597505	0.304	0.024	1.56E-229	11
CYR61	7.78E-234	1.607588021	0.361	0.035	1.79E-229	11
FAM101B	1.02E-232	0.846716989	0.284	0.021	2.35E-228	11
BAIAP2	1.76E-232	0.72046329	0.262	0.017	4.05E-228	11
APLP2.9	9.51E-230	1.793634911	0.831	0.236	2.19E-225	11
CDC42EP1	5.48E-228	0.67636838	0.253	0.017	1.26E-223	11
LIMA1	3.51E-226	1.219707001	0.497	0.072	8.06E-222	11
SNCG	3.42E-225	0.77292289	0.328	0.03	7.87E-221	11
FAM198B	3.80E-225	0.880874405	0.313	0.028	8.73E-221	11
PTP4A3	3.26E-223	0.723501818	0.29	0.023	7.50E-219	11
CD9.4	5.98E-223	1.748194756	0.8	0.204	1.37E-218	11
ITGB1.6	1.38E-221	1.964882992	0.96	0.448	3.18E-217	11
ARHGEF12	2.81E-221	1.173981071	0.466	0.065	6.47E-217	11
PON2	2.81E-220	1.266161318	0.517	0.082	6.46E-216	11
NGFRAP1	4.61E-219	1.111722762	0.572	0.097	1.06E-214	11
TIMP2.2	1.15E-214	1.159469576	0.461	0.068	2.63E-210	11
SEPW1.10	1.59E-214	1.924784773	0.898	0.341	3.65E-210	11
SCARB1	7.88E-214	1.00403136	0.335	0.034	1.81E-209	11
RDX.1	8.75E-214	1.387254532	0.683	0.155	2.01E-209	11
OAZ2.1	4.88E-213	1.57584576	0.698	0.166	1.12E-208	11
TGFBR2.1	2.93E-211	1.73505919	0.725	0.184	6.74E-207	11
IL4R	5.67E-211	1.141735506	0.486	0.074	1.30E-206	11
THBS1	1.55E-210	0.918979915	0.426	0.054	3.56E-206	11
LMO2	5.08E-208	0.945994236	0.317	0.031	1.17E-203	11
YES1	1.45E-207	0.834270074	0.35	0.037	3.33E-203	11
IVNS1ABP.2	4.72E-206	1.829176537	0.723	0.187	1.09E-201	11
CSGALNACT1	2.63E-205	0.99121255	0.368	0.043	6.05E-201	11
ACACB	2.30E-203	0.732752461	0.257	0.02	5.28E-199	11
TMCC3.1	4.74E-203	1.071154881	0.368	0.045	1.09E-198	11
TNFAIP8L1	7.54E-203	0.936687999	0.381	0.046	1.73E-198	11
PKIG	4.12E-201	0.802641679	0.404	0.052	9.46E-197	11
NETO2	8.37E-199	0.737125484	0.282	0.025	1.92E-194	11
YWHAH.9	4.29E-198	1.641057741	0.838	0.265	9.87E-194	11
F2R.1	7.22E-198	1.203539574	0.603	0.114	1.66E-193	11
FNDC3B	5.93E-197	1.201306597	0.506	0.088	1.36E-192	11
LMCD1	6.40E-197	0.877813295	0.317	0.032	1.47E-192	11
REEP3	1.13E-195	1.10015999	0.483	0.079	2.59E-191	11
DGKH.1	1.07E-194	1.09991528	0.47	0.072	2.47E-190	11
RBMS2	4.92E-194	0.794019194	0.297	0.029	1.13E-189	11

STOM	8.31E-194	1.55210625	0.705	0.185	1.91E-189	11
ARHGEF7	1.01E-193	1.020466287	0.419	0.059	2.32E-189	11
GFOD2	1.48E-192	0.685561351	0.295	0.028	3.41E-188	11
CTGF	1.95E-192	1.331885928	0.337	0.037	4.48E-188	11
PROS1	1.42E-191	0.872219672	0.348	0.041	3.26E-187	11
FSCN1	1.60E-191	0.689021897	0.264	0.023	3.67E-187	11
ICAM2	1.71E-191	1.162964402	0.525	0.094	3.92E-187	11
PDLIM1	5.36E-191	1.02187122	0.57	0.105	1.23E-186	11
CTTN	7.00E-191	0.682687674	0.308	0.031	1.61E-186	11
ADIPOR2	1.73E-189	1.003423786	0.412	0.059	3.98E-185	11
RGCC.10	5.44E-189	2.117798481	0.843	0.289	1.25E-184	11
RASAL2.1	1.09E-188	0.952999676	0.37	0.048	2.51E-184	11
VAMP5.5	2.36E-188	1.597364206	0.834	0.29	5.42E-184	11
NFIA	5.44E-188	1.028049433	0.452	0.072	1.25E-183	11
CDC42BPB	8.96E-188	0.625585544	0.264	0.023	2.06E-183	11
CDC42EP3	7.58E-187	1.214732868	0.57	0.113	1.74E-182	11
MLEC	4.32E-186	1.451237651	0.634	0.152	9.92E-182	11
TNFAIP1	9.02E-186	0.641906246	0.293	0.029	2.07E-181	11
BCAP29	4.94E-185	1.257052523	0.563	0.115	1.14E-180	11
RPL41.8	3.41E-183	-1.403663626	0.987	0.976	7.84E-179	11
MYO1C	3.48E-183	0.839054513	0.368	0.048	8.01E-179	11
S100A13.1	4.70E-183	1.029042465	0.588	0.118	1.08E-178	11
SERPINB6	9.73E-183	1.259128968	0.65	0.159	2.24E-178	11
CLIC4	6.92E-182	1.002462781	0.47	0.08	1.59E-177	11
TINAGL1	5.73E-181	0.890273045	0.357	0.044	1.32E-176	11
TJP2	7.00E-181	0.686458276	0.255	0.022	1.61E-176	11
DEPTOR	9.77E-181	0.849790463	0.322	0.037	2.25E-176	11
TP53I11	4.62E-177	0.792443191	0.251	0.022	1.06E-172	11
KIAA0355	8.75E-177	1.096400133	0.472	0.083	2.01E-172	11
RPS27.7	6.80E-176	-1.56614623	0.982	0.975	1.56E-171	11
ID1	7.73E-175	1.295746619	0.404	0.059	1.78E-170	11
HDAC7	1.26E-174	0.962641068	0.439	0.07	2.90E-170	11
WBP5	1.95E-173	0.856053695	0.446	0.073	4.49E-169	11
SWAP70.2	5.49E-173	1.126726207	0.492	0.094	1.26E-168	11
ZEB1	1.98E-172	0.973377685	0.461	0.077	4.54E-168	11
MARCKSL1	5.09E-172	1.070456907	0.45	0.075	1.17E-167	11
GOLIM4.1	2.77E-171	1.116513213	0.47	0.086	6.36E-167	11
PGM2L1	1.05E-170	1.038351224	0.361	0.049	2.41E-166	11
LHFP	5.27E-170	0.613292733	0.337	0.041	1.21E-165	11
CYB5R3.2	8.40E-170	1.147086168	0.665	0.171	1.93E-165	11
SERPINH1	1.67E-167	0.901094217	0.424	0.069	3.83E-163	11
TMEM184B	1.71E-166	0.654851164	0.282	0.03	3.92E-162	11
SH2D3C	6.62E-166	0.688806862	0.299	0.034	1.52E-161	11
SEC62.9	1.17E-164	1.475003483	0.905	0.409	2.69E-160	11
KLHL5.1	1.75E-164	1.034698881	0.437	0.078	4.02E-160	11
LXN	4.90E-164	0.778216029	0.299	0.035	1.13E-159	11
ELK3	7.61E-164	1.013432324	0.466	0.085	1.75E-159	11
LEPROT.1	2.57E-163	1.316041026	0.656	0.182	5.91E-159	11
EPHX1	1.40E-160	0.742324581	0.29	0.034	3.21E-156	11
PRKCDBP	7.08E-160	0.869533529	0.43	0.072	1.63E-155	11
PHACTR2	2.23E-159	0.989484561	0.494	0.096	5.13E-155	11
RPS2.6	3.17E-159	-1.488364941	0.927	0.945	7.29E-155	11
SH3D19	7.57E-159	0.666579719	0.268	0.028	1.74E-154	11
RPL10.7	8.51E-159	-1.268600019	0.978	0.975	1.96E-154	11
FAM213A	9.34E-158	1.036843176	0.499	0.1	2.15E-153	11
HEBP1	1.01E-157	0.958673619	0.463	0.09	2.32E-153	11
IL6ST.1	5.06E-157	1.375697327	0.63	0.164	1.16E-152	11
AKT3	2.13E-156	0.832770666	0.357	0.052	4.89E-152	11

NEAT1.7	3.91E-156	1.608411357	0.967	0.746	8.98E-152	11
SH3BP5	4.66E-156	1.215368502	0.541	0.123	1.07E-151	11
BMPR2	1.00E-155	1.052064525	0.39	0.064	2.30E-151	11
RPL13.6	2.53E-153	-1.269292769	0.969	0.965	5.83E-149	11
GIMAP8	1.57E-152	0.711934213	0.304	0.039	3.61E-148	11
SLC44A2	6.01E-152	1.039976197	0.446	0.082	1.38E-147	11
PTK2	8.20E-152	0.626453581	0.288	0.035	1.88E-147	11
CALU	1.70E-151	0.933041022	0.463	0.091	3.91E-147	11
ADD1	6.48E-151	1.287567624	0.55	0.131	1.49E-146	11
RPS29.8	5.61E-150	-1.714513416	0.938	0.921	1.29E-145	11
RPL13A.8	1.41E-149	-1.188974256	0.984	0.962	3.24E-145	11
RPL28.7	1.47E-149	-1.394128779	0.938	0.932	3.38E-145	11
RPS15A.6	4.37E-149	-1.3547066	0.945	0.933	1.00E-144	11
ABLIM1	5.35E-149	0.990392457	0.379	0.06	1.23E-144	11
PTTG1IP.1	6.21E-149	1.114222879	0.614	0.162	1.43E-144	11
CD81.2	7.19E-148	1.212622298	0.576	0.148	1.65E-143	11
THEM6	3.11E-146	0.651353797	0.264	0.03	7.14E-142	11
RPL23A.8	1.87E-144	-1.372827784	0.942	0.93	4.29E-140	11
CCPG1	2.60E-144	1.168399997	0.583	0.15	5.98E-140	11
CPNE2	8.41E-144	0.635651444	0.293	0.037	1.93E-139	11
CDA	1.16E-143	0.707048765	0.259	0.03	2.67E-139	11
NCOA7	1.39E-143	1.105566106	0.528	0.12	3.19E-139	11
MED13L	3.38E-143	1.009628623	0.448	0.09	7.77E-139	11
DHRS3	3.63E-143	1.096426924	0.483	0.104	8.35E-139	11
FKBP1A.7	7.44E-142	1.424956358	0.847	0.398	1.71E-137	11
CCDC85B.4	4.64E-141	1.386129166	0.705	0.238	1.07E-136	11
B3GNT5	3.38E-139	0.694061596	0.271	0.034	7.76E-135	11
RPS18.7	1.55E-138	-1.242339235	0.965	0.959	3.57E-134	11
TMCO3	3.52E-138	0.751579879	0.339	0.053	8.09E-134	11
HIPK2	4.26E-138	1.081153242	0.475	0.104	9.78E-134	11
KLF9	2.87E-137	1.207862297	0.565	0.148	6.60E-133	11
RFK	1.50E-136	1.102964155	0.368	0.064	3.46E-132	11
CTTNBP2NL.2	3.49E-136	0.940058025	0.419	0.084	8.02E-132	11
RPLP2.6	1.12E-135	-1.186019859	0.958	0.944	2.58E-131	11
RPL39.4	2.42E-135	-1.312432956	0.936	0.945	5.55E-131	11
XAF1	7.83E-135	1.071245407	0.477	0.105	1.80E-130	11
CD46	3.15E-134	1.216477388	0.641	0.198	7.24E-130	11
GBP4	3.35E-134	1.329132144	0.532	0.133	7.70E-130	11
FN1.1	3.53E-134	1.907969395	0.49	0.112	8.12E-130	11
DSTN.9	5.66E-133	1.064177418	0.792	0.291	1.30E-128	11
EHD2	2.26E-132	0.643758429	0.295	0.041	5.20E-128	11
RHOB.9	2.53E-132	1.29398615	0.847	0.332	5.81E-128	11
ASAP1	2.95E-132	0.864126035	0.435	0.089	6.77E-128	11
C10orf10.1	4.47E-132	1.878021133	0.466	0.104	1.03E-127	11
RPL21.5	7.98E-132	-1.172314581	0.956	0.952	1.83E-127	11
RPS19.6	4.36E-131	-1.247600315	0.967	0.951	1.00E-126	11
RPL26.4	8.36E-131	-1.226270109	0.949	0.935	1.92E-126	11
PIK3C2A	4.35E-130	0.922689672	0.408	0.081	9.99E-126	11
RPS12.3	9.36E-130	-1.238140129	0.953	0.938	2.15E-125	11
RHOC.8	1.25E-127	1.155127198	0.769	0.308	2.88E-123	11
EEF1A1.7	8.50E-127	-1.066586774	0.973	0.968	1.95E-122	11
ADM	9.75E-127	1.303131658	0.472	0.106	2.24E-122	11
CTSL.2	3.25E-126	0.939272795	0.552	0.145	7.47E-122	11
RPS3.7	3.37E-126	-1.256091878	0.936	0.918	7.75E-122	11
RCN2.1	3.99E-126	0.940348072	0.548	0.141	9.17E-122	11
CAPZA2.6	1.65E-125	1.144410671	0.763	0.296	3.80E-121	11
LUZP1	3.67E-125	0.949770247	0.468	0.107	8.42E-121	11
NEDD9.1	3.69E-125	1.043128619	0.574	0.156	8.47E-121	11

MAP4.2	5.90E-125	1.077695081	0.588	0.165	1.36E-120	11
CD151.1	1.06E-124	0.939733473	0.63	0.186	2.44E-120	11
RPL27A.6	1.93E-124	-1.162164268	0.942	0.93	4.43E-120	11
EXOC6	9.05E-124	0.935993827	0.344	0.06	2.08E-119	11
YBX3	2.43E-123	1.028789156	0.596	0.171	5.59E-119	11
RPL18A.6	4.80E-123	-1.218953829	0.936	0.93	1.10E-118	11
MTRNR2L8.1	9.68E-123	1.61871325	0.641	0.211	2.22E-118	11
RPS27A.7	2.17E-122	-1.08815293	0.953	0.939	4.99E-118	11
TSPAN4	3.90E-122	0.856690613	0.421	0.091	8.96E-118	11
PAM	1.87E-121	0.764627311	0.368	0.068	4.29E-117	11
RPLP1.2	1.94E-121	-1.021331199	0.978	0.966	4.46E-117	11
PTPN12	6.20E-121	0.903407489	0.439	0.099	1.42E-116	11
RPL31.8	1.67E-120	-1.349353403	0.894	0.907	3.83E-116	11
RABGAP1	3.93E-120	0.841031287	0.335	0.058	9.03E-116	11
GNAS.5	1.29E-118	1.336135887	0.765	0.331	2.97E-114	11
RIN2	2.01E-118	0.68091187	0.266	0.038	4.62E-114	11
SLC27A3	1.22E-117	0.752364386	0.271	0.039	2.81E-113	11
ITM2B.9	3.27E-117	1.128849082	0.991	0.772	7.51E-113	11
HSP90B1.3	5.44E-117	1.210088961	0.838	0.392	1.25E-112	11
HLA-E.9	9.44E-117	1.015220303	0.984	0.783	2.17E-112	11
DUSP6	1.58E-116	0.89852765	0.459	0.106	3.63E-112	11
ARHGAP5	1.90E-116	0.80672757	0.401	0.084	4.37E-112	11
RPS14.7	2.10E-116	-1.064037827	0.956	0.946	4.83E-112	11
ATXN3	2.36E-116	0.904442598	0.419	0.093	5.42E-112	11
RPL19.5	2.62E-116	-1.113416599	0.933	0.924	6.01E-112	11
RPL37.5	2.63E-116	-1.279608777	0.896	0.909	6.05E-112	11
CTNNA1	4.50E-116	0.860030615	0.481	0.123	1.03E-111	11
WARS	9.93E-116	0.921021391	0.386	0.08	2.28E-111	11
RPL30.4	1.85E-115	-1.24167947	0.905	0.897	4.25E-111	11
10-Sep	4.88E-115	0.59906103	0.264	0.039	1.12E-110	11
CALCOCO2	2.64E-114	1.024795781	0.57	0.17	6.07E-110	11
LMNA	6.33E-114	1.128742967	0.57	0.162	1.46E-109	11
AAMDC	2.29E-113	0.732434089	0.388	0.081	5.27E-109	11
GFOD1	4.33E-113	0.814666203	0.386	0.077	9.95E-109	11
PPM1F	8.43E-113	0.633140217	0.251	0.035	1.94E-108	11
SERINC3	5.46E-112	0.975901737	0.574	0.173	1.26E-107	11
CTNND1.1	8.89E-111	0.859766781	0.368	0.078	2.04E-106	11
ROCK2	3.97E-110	0.705520346	0.299	0.05	9.13E-106	11
CTNNB1.1	1.01E-108	1.165268	0.599	0.191	2.32E-104	11
TNS1	1.01E-108	0.583469899	0.268	0.041	2.33E-104	11
PTPRC.10	1.82E-107	-2.677045618	0.093	0.634	4.18E-103	11
ARHGAP18.2	6.14E-107	1.050003192	0.588	0.185	1.41E-102	11
PLOD1	6.87E-107	0.626517968	0.268	0.043	1.58E-102	11
FAM43A	8.93E-107	0.572551811	0.257	0.038	2.05E-102	11
NASP	5.25E-106	1.083848007	0.619	0.206	1.21E-101	11
RPL12.5	3.27E-104	-1.17388506	0.927	0.911	7.51E-100	11
GINM1	9.29E-104	0.832073149	0.463	0.122	2.13E-99	11
IFITM2.8	2.23E-102	0.932294359	0.863	0.463	5.13E-98	11
SNRK	2.29E-102	0.870635123	0.43	0.103	5.25E-98	11
RPS24.5	3.53E-102	-1.066159981	0.947	0.912	8.11E-98	11
MAPK3	3.66E-102	0.606542142	0.286	0.05	8.41E-98	11
RPS3A.3	5.36E-102	-1.038654661	0.947	0.929	1.23E-97	11
ARRDC2	6.91E-102	0.980106056	0.377	0.086	1.59E-97	11
TCEAL4.1	7.34E-102	0.731400929	0.512	0.147	1.69E-97	11
RPL10A.7	1.21E-101	-1.359261597	0.823	0.866	2.77E-97	11
XIST.7	3.77E-101	0.999811084	0.876	0.409	8.66E-97	11
RPS21.6	1.19E-100	-1.349968086	0.836	0.863	2.73E-96	11
RPL11.3	2.16E-100	-0.952101452	0.969	0.937	4.96E-96	11

RPS25.7	3.05E-100	-1.091769714	0.929	0.917	7.01E-96	11
RPS15.7	3.15E-100	-0.970444537	0.949	0.931	7.24E-96	11
RPL6.6	1.65E-99	-1.247800938	0.858	0.883	3.78E-95	11
RGL2	5.62E-99	0.591499374	0.304	0.056	1.29E-94	11
ADGRG1	5.90E-99	0.634256792	0.368	0.077	1.36E-94	11
VGLL4	2.16E-98	0.61196797	0.339	0.069	4.96E-94	11
PEA15.2	2.45E-98	0.791028526	0.406	0.101	5.63E-94	11
INPP1	4.41E-97	0.585169621	0.286	0.051	1.01E-92	11
ITPRIPL2.1	6.33E-97	0.652864436	0.266	0.046	1.45E-92	11
KTN1.4	2.09E-96	0.996982293	0.787	0.369	4.80E-92	11
FRMD8	2.88E-96	0.648143164	0.295	0.054	6.61E-92	11
RPL34.7	3.11E-96	-0.938598998	0.987	0.952	7.14E-92	11
RGS3	4.43E-96	0.581319805	0.253	0.04	1.02E-91	11
WDR60	5.52E-96	0.754156473	0.401	0.096	1.27E-91	11
CYSTM1.1	1.78E-95	0.681306831	0.475	0.129	4.08E-91	11
RPL32.2	1.93E-95	-0.982863851	0.942	0.926	4.44E-91	11
SLK	2.36E-95	0.804634602	0.348	0.076	5.42E-91	11
FYN.7	3.43E-95	1.023693193	0.69	0.252	7.88E-91	11
RPS16.5	3.85E-95	-1.11928849	0.9	0.891	8.86E-91	11
CCDC50.2	1.21E-94	0.784753428	0.428	0.113	2.78E-90	11
RPS8.6	5.99E-94	-1.135929345	0.931	0.917	1.38E-89	11
COPS8	1.16E-93	0.830162207	0.417	0.106	2.67E-89	11
NPTN.1	1.69E-93	0.78237397	0.304	0.061	3.88E-89	11
HMBOX1	3.26E-92	0.842082903	0.428	0.111	7.49E-88	11
RPL35A.7	4.16E-92	-1.015331203	0.918	0.917	9.57E-88	11
SRP14.3	4.62E-92	1.042541524	0.927	0.709	1.06E-87	11
SLC1A5.1	6.09E-92	0.846716327	0.361	0.085	1.40E-87	11
RPS23.4	1.20E-91	-0.954294685	0.945	0.918	2.75E-87	11
CDC37.3	1.55E-91	0.940010233	0.701	0.295	3.57E-87	11
TRIOBP	2.08E-91	0.584020459	0.297	0.058	4.77E-87	11
CD40	3.29E-91	0.537626962	0.295	0.058	7.55E-87	11
CRK	3.29E-91	0.734219489	0.337	0.075	7.56E-87	11
MAP4K4	5.18E-91	0.753328298	0.322	0.067	1.19E-86	11
RPSA.5	7.89E-91	-1.583562241	0.698	0.807	1.81E-86	11
ACTN4.1	1.43E-90	0.909597301	0.616	0.222	3.29E-86	11
SYPL1	1.55E-90	0.83669922	0.543	0.177	3.56E-86	11
RPS6.4	2.87E-90	-0.942203262	0.967	0.942	6.59E-86	11
GIMAP7.10	3.35E-90	1.090628383	0.687	0.271	7.69E-86	11
RGS1.11	9.63E-90	-3.096171955	0.377	0.707	2.21E-85	11
MXD4	2.07E-89	0.768422859	0.419	0.109	4.76E-85	11
MPZL1.1	3.39E-89	0.633288725	0.282	0.055	7.80E-85	11
RP11-553L6.5	3.79E-89	0.593100817	0.259	0.046	8.70E-85	11
CXCR4.8	3.67E-88	-2.38869003	0.599	0.781	8.43E-84	11
NAA38.6	4.91E-88	0.941231503	0.659	0.274	1.13E-83	11
NUCB2.1	5.86E-88	0.692023026	0.528	0.16	1.35E-83	11
FAU.3	5.87E-88	-1.01578862	0.905	0.888	1.35E-83	11
ABL2.2	6.28E-88	0.694053776	0.306	0.064	1.44E-83	11
GIMAP6	8.06E-88	0.644147727	0.386	0.093	1.85E-83	11
BST2.6	9.91E-88	1.066485832	0.741	0.348	2.28E-83	11
EDEM3	1.05E-87	0.714018177	0.271	0.051	2.41E-83	11
CD63.9	1.23E-87	0.868997746	0.907	0.507	2.82E-83	11
LRCH1	1.73E-86	0.581973448	0.257	0.047	3.97E-82	11
EPS8.1	2.43E-86	0.730904109	0.317	0.07	5.59E-82	11
RPS17.4	4.09E-86	-1.015718902	0.914	0.897	9.40E-82	11
RPS20.4	1.71E-85	-1.020448773	0.92	0.896	3.94E-81	11
VIM.9	1.87E-85	0.867155946	0.98	0.764	4.29E-81	11
RPL35.5	2.48E-85	-1.044338768	0.914	0.897	5.71E-81	11
RCN1	2.93E-85	0.698859037	0.35	0.084	6.73E-81	11

TBC1D15	7.90E-85	0.785966492	0.39	0.102	1.82E-80	11
PDLIM5	1.19E-84	0.658396343	0.33	0.075	2.74E-80	11
RPS4X.7	2.90E-84	-0.966808837	0.933	0.938	6.66E-80	11
FEZ2.1	9.35E-84	0.756762704	0.441	0.13	2.15E-79	11
RAC1.7	9.52E-84	0.818403163	0.843	0.435	2.19E-79	11
TRIM56.2	2.86E-83	0.848089327	0.534	0.179	6.58E-79	11
RRBP1.1	2.91E-83	0.937626507	0.459	0.145	6.68E-79	11
POMP.6	2.05E-82	0.935017076	0.812	0.436	4.70E-78	11
RPL14.5	2.94E-82	-1.129692053	0.878	0.868	6.76E-78	11
NDRG2.1	3.19E-82	0.684896507	0.308	0.069	7.33E-78	11
DYNC1LI2	3.74E-82	0.703895588	0.406	0.11	8.59E-78	11
CORO1A.8	3.77E-82	-2.205015891	0.126	0.579	8.66E-78	11
FAM214A	5.02E-82	0.75361758	0.339	0.079	1.15E-77	11
RPS7.7	5.38E-82	-1.029900652	0.898	0.884	1.24E-77	11
ICA1	1.60E-81	0.536523591	0.255	0.047	3.67E-77	11
RPL3.6	3.41E-80	-0.906428482	0.962	0.931	7.83E-76	11
TAX1BP3.1	7.95E-80	0.680105111	0.441	0.133	1.83E-75	11
DHRS7B	1.06E-79	0.594732352	0.262	0.052	2.43E-75	11
SLC44A1	3.86E-79	0.644104255	0.308	0.071	8.87E-75	11
RSU1	4.52E-79	0.745877882	0.441	0.133	1.04E-74	11
GRN.10	4.67E-79	0.604668314	0.667	0.24	1.07E-74	11
IFI6	5.42E-79	0.788425766	0.55	0.195	1.25E-74	11
ZBTB16.1	5.72E-78	0.911223305	0.563	0.209	1.31E-73	11
RPL38.5	9.04E-78	-1.112783155	0.865	0.868	2.08E-73	11
PRDX1.6	1.15E-77	0.67870043	0.798	0.39	2.64E-73	11
LAMP1.3	1.25E-77	0.844834722	0.681	0.292	2.87E-73	11
FNIP2.1	1.30E-77	0.696312636	0.302	0.069	3.00E-73	11
RPS28.6	1.73E-77	-0.763128674	0.991	0.946	3.97E-73	11
LAPTM4A.8	3.04E-77	0.657143294	0.758	0.338	6.99E-73	11
QKI.2	1.10E-76	0.798287632	0.381	0.108	2.52E-72	11
ZFP36L2.10	2.70E-76	-2.492592429	0.448	0.707	6.20E-72	11
TMEM245	3.67E-76	0.606836687	0.302	0.068	8.44E-72	11
RPL15.1	2.02E-75	-0.843789144	0.94	0.906	4.65E-71	11
ZFYVE21	5.79E-75	0.568942789	0.299	0.069	1.33E-70	11
RPS26.7	7.05E-75	-1.073575066	0.887	0.87	1.62E-70	11
RBMS1.2	9.39E-75	0.843183993	0.543	0.194	2.16E-70	11
BTG1.10	2.09E-74	-1.734141029	0.891	0.841	4.81E-70	11
MTRNR2L12	3.40E-74	0.961046092	0.499	0.17	7.80E-70	11
GNAI2.7	1.48E-73	0.955442893	0.594	0.249	3.40E-69	11
ASPH.1	2.18E-73	0.65455937	0.35	0.094	5.01E-69	11
MYL12B.2	2.43E-73	0.855440256	0.9	0.626	5.58E-69	11
HCST.9	4.08E-73	-2.108306915	0.133	0.558	9.37E-69	11
RPS13.4	1.22E-72	-0.985335975	0.905	0.877	2.79E-68	11
ABCG1.1	2.29E-72	0.690460316	0.379	0.107	5.27E-68	11
TNFSF10.1	2.30E-72	0.958094695	0.463	0.157	5.29E-68	11
RPL7.6	2.97E-72	-0.928150648	0.945	0.919	6.83E-68	11
RNF115.3	5.27E-72	0.837740515	0.554	0.209	1.21E-67	11
GPX3.2	1.21E-71	0.945962849	0.466	0.155	2.79E-67	11
PXN	1.92E-71	0.568686589	0.302	0.071	4.40E-67	11
LRMP	7.99E-71	0.721788477	0.353	0.095	1.84E-66	11
RPL36.9	1.07E-70	-0.984640735	0.931	0.912	2.46E-66	11
MALAT1.10	2.11E-70	0.738963	0.998	0.982	4.85E-66	11
BCAP31.3	4.45E-70	0.758902155	0.623	0.268	1.02E-65	11
PLEKHA1	4.54E-70	0.522606835	0.395	0.112	1.04E-65	11
HSPB1.9	5.35E-70	0.615425637	0.829	0.421	1.23E-65	11
JMJD1C.4	2.50E-69	0.949477439	0.588	0.247	5.74E-65	11
RALB	2.99E-69	0.585479569	0.341	0.092	6.87E-65	11
CD44.7	3.75E-69	-1.934956634	0.073	0.505	8.61E-65	11

RPL9.4	4.32E-69	-0.853813845	0.942	0.9	9.92E-65	11
SH3GLB1.3	1.01E-68	0.772939394	0.603	0.257	2.32E-64	11
DPYSL2.2	4.32E-68	0.769362908	0.41	0.131	9.92E-64	11
S100A4.9	4.56E-68	-2.169469805	0.479	0.707	1.05E-63	11
RPLP0.7	4.66E-68	-1.335917736	0.825	0.843	1.07E-63	11
TMOD3	4.72E-68	0.705887824	0.468	0.161	1.09E-63	11
ARL2	4.98E-68	0.641334791	0.43	0.14	1.14E-63	11
TMSB4X.5	6.21E-68	-0.789661562	1	0.976	1.43E-63	11
CLCN3	7.18E-68	0.6007284	0.251	0.054	1.65E-63	11
PARP14.2	7.56E-68	0.903611837	0.517	0.194	1.74E-63	11
RPS10.5	1.12E-67	-1.249051354	0.727	0.788	2.57E-63	11
CLSTN1	1.23E-67	0.616329375	0.335	0.09	2.82E-63	11
CEP68	1.93E-67	0.516149922	0.255	0.056	4.43E-63	11
SECISBP2L	3.28E-67	0.66455374	0.306	0.078	7.54E-63	11
PARVB	3.99E-67	0.616645148	0.29	0.072	9.16E-63	11
TUG1	9.93E-67	0.628925132	0.308	0.078	2.28E-62	11
RPL18.5	4.76E-66	-0.953118494	0.885	0.87	1.09E-61	11
PSMB5	1.34E-65	0.536303427	0.435	0.144	3.08E-61	11
UBE2J1	1.56E-65	0.67307979	0.377	0.112	3.58E-61	11
VIMP.1	3.15E-65	0.683557361	0.55	0.218	7.23E-61	11
RTN4.8	4.25E-65	0.748518054	0.703	0.338	9.77E-61	11
FCGRT.5	6.12E-65	0.555471806	0.605	0.229	1.41E-60	11
USP34.2	6.96E-65	0.794649958	0.47	0.167	1.60E-60	11
LPP	1.51E-64	0.718616514	0.457	0.156	3.48E-60	11
ZFYVE16	2.64E-64	0.562454776	0.279	0.068	6.08E-60	11
FYB.9	2.90E-64	-1.918946159	0.069	0.473	6.66E-60	11
SDF4	6.08E-64	0.717432654	0.497	0.186	1.40E-59	11
RPL36A.6	2.31E-63	-1.309232387	0.725	0.804	5.31E-59	11
MIR4435-2HG	2.38E-63	0.761019283	0.43	0.142	5.46E-59	11
CAMTA1	2.38E-63	0.576103341	0.497	0.185	5.46E-59	11
DNAJC3	4.32E-63	0.664689995	0.439	0.152	9.94E-59	11
RAB11A.3	1.85E-62	0.611206213	0.545	0.218	4.25E-58	11
RPS9.4	3.26E-62	-0.88468122	0.891	0.878	7.49E-58	11
ZBTB20.1	3.93E-62	0.663919386	0.437	0.144	9.04E-58	11
ERLEC1	4.42E-62	0.683833287	0.448	0.158	1.02E-57	11
TMEM30A	1.63E-61	0.749646498	0.406	0.136	3.75E-57	11
AP1S2.3	3.40E-61	0.565530178	0.557	0.214	7.82E-57	11
DOCK4.1	3.75E-61	0.615285151	0.266	0.066	8.61E-57	11
CD2.10	3.78E-61	-2.391332575	0.082	0.469	8.69E-57	11
CYTIP.9	3.90E-61	-1.889018708	0.095	0.488	8.97E-57	11
QSOX1	7.30E-61	0.638791284	0.299	0.081	1.68E-56	11
IFNGR1.8	7.54E-61	0.68990598	0.603	0.248	1.73E-56	11
ELMO1.1	1.28E-60	0.73040457	0.417	0.142	2.95E-56	11
ATOX1.3	2.49E-60	0.539446596	0.532	0.214	5.73E-56	11
GABARAPL2.3	2.95E-60	0.719568581	0.676	0.337	6.77E-56	11
NFIC	3.62E-60	0.621073475	0.359	0.112	8.32E-56	11
NDUFA12.5	4.17E-60	0.689727559	0.594	0.263	9.57E-56	11
RPL27.2	6.73E-60	-0.920563314	0.856	0.852	1.55E-55	11
CD69.11	6.86E-60	-2.774090533	0.186	0.54	1.58E-55	11
RAB6A	1.22E-59	0.619126424	0.435	0.151	2.80E-55	11
CD37.7	1.62E-59	-1.659992047	0.086	0.476	3.72E-55	11
EID1.4	1.90E-59	0.753009076	0.732	0.418	4.38E-55	11
EIF4G3	2.48E-59	0.562160214	0.262	0.064	5.70E-55	11
C12orf57.6	2.85E-59	0.760129805	0.71	0.361	6.54E-55	11
UBA52.2	2.91E-59	-0.846773876	0.878	0.866	6.68E-55	11
CELF2.8	3.54E-59	-1.705808837	0.051	0.438	8.14E-55	11
PTMS.3	5.75E-59	0.572387349	0.563	0.231	1.32E-54	11
PML	1.49E-58	0.508549995	0.262	0.065	3.42E-54	11

PICALM.2	2.70E-58	0.740077982	0.472	0.18	6.20E-54	11
RPS5.5	3.88E-58	-1.081536545	0.86	0.841	8.92E-54	11
TMEM50B	1.32E-57	0.538063629	0.384	0.122	3.04E-53	11
APOL3	1.43E-57	0.592064519	0.335	0.099	3.28E-53	11
ADAM9.1	2.65E-57	0.543315462	0.257	0.066	6.10E-53	11
LINC00657	4.07E-57	0.585995876	0.43	0.152	9.36E-53	11
ARGLU1.7	4.70E-57	0.83674393	0.765	0.422	1.08E-52	11
SCARB2.2	7.09E-57	0.572223238	0.319	0.096	1.63E-52	11
ABI2	7.93E-57	0.54119286	0.304	0.085	1.82E-52	11
RPL37A.2	8.90E-57	-0.723889146	0.936	0.905	2.04E-52	11
CLTC.1	1.09E-56	0.560892649	0.357	0.113	2.50E-52	11
CD52.8	3.72E-56	-2.352724258	0.246	0.559	8.55E-52	11
RPL7A.4	4.32E-56	-0.885306265	0.874	0.847	9.94E-52	11
TSPAN14	4.71E-56	0.650669116	0.492	0.193	1.08E-51	11
ADAM10	4.86E-56	0.712336136	0.406	0.141	1.12E-51	11
RPL29.1	4.90E-56	-0.879517055	0.891	0.854	1.13E-51	11
RABAC1.5	5.51E-56	0.690347106	0.683	0.346	1.27E-51	11
C5orf24	7.42E-56	0.611106602	0.348	0.11	1.70E-51	11
FDPS.1	1.36E-55	0.646621005	0.472	0.183	3.13E-51	11
LSP1.10	1.42E-55	-1.69814857	0.08	0.446	3.26E-51	11
RPL5.4	1.46E-55	-0.956372963	0.867	0.852	3.37E-51	11
AHNAK.4	1.55E-55	0.743821787	0.605	0.284	3.56E-51	11
SEPT2.4	1.79E-55	0.688238538	0.572	0.251	4.11E-51	11
MAGED2	2.35E-55	0.592110289	0.41	0.143	5.41E-51	11
GIMAP4.7	2.83E-55	0.733772859	0.674	0.327	6.50E-51	11
RPL8.3	4.40E-55	-0.821284646	0.911	0.881	1.01E-50	11
MAGT1	5.33E-55	0.621414964	0.317	0.095	1.22E-50	11
UXS1	7.03E-55	0.523974153	0.302	0.085	1.62E-50	11
TRAC.11	1.87E-54	-2.331158736	0.082	0.444	4.29E-50	11
ZFHX3.2	2.62E-54	0.613565898	0.284	0.081	6.02E-50	11
CALR.3	3.28E-54	0.621627854	0.778	0.432	7.54E-50	11
UTRN.2	3.79E-54	0.832713323	0.514	0.221	8.71E-50	11
AES.5	4.25E-54	0.727668662	0.557	0.241	9.76E-50	11
MACF1.7	5.22E-54	0.768230048	0.563	0.247	1.20E-49	11
NCOA3	6.42E-54	0.647771539	0.344	0.11	1.48E-49	11
SLC2A3.6	1.17E-53	0.96032486	0.565	0.254	2.68E-49	11
LAPTM5.9	1.27E-53	-1.719955621	0.295	0.576	2.91E-49	11
CCL5.10	1.40E-53	-3.131235179	0.284	0.571	3.21E-49	11
TM9SF2.1	1.55E-53	0.624681739	0.401	0.144	3.56E-49	11
KLF3.1	2.37E-53	0.566143241	0.404	0.141	5.45E-49	11
CHMP3	2.66E-53	0.556200826	0.446	0.171	6.10E-49	11
GALNT1	2.88E-53	0.659511142	0.286	0.082	6.63E-49	11
RPL4.3	6.16E-53	-1.143780889	0.736	0.77	1.42E-48	11
HNRNPA1.3	9.61E-53	-1.128750603	0.743	0.779	2.21E-48	11
MIDN	1.13E-52	0.514932716	0.368	0.12	2.59E-48	11
MBNL2	4.50E-52	0.558325136	0.293	0.086	1.03E-47	11
IFNAR1.1	4.78E-52	0.568839626	0.397	0.143	1.10E-47	11
CD3D.11	4.96E-52	-2.202839455	0.157	0.485	1.14E-47	11
TPT1.8	6.16E-52	-0.784964475	0.949	0.923	1.42E-47	11
GNB2L1.2	9.49E-52	-0.947786419	0.825	0.836	2.18E-47	11
RPL24.6	1.15E-51	-0.957231435	0.863	0.852	2.64E-47	11
TMEM50A.3	1.27E-51	0.763070344	0.634	0.327	2.93E-47	11
CPD	2.09E-51	0.547751532	0.255	0.067	4.81E-47	11
SLA.9	2.61E-51	-1.633073013	0.058	0.411	6.00E-47	11
HACD3	5.45E-51	0.596904974	0.262	0.072	1.25E-46	11
VPS37A	6.76E-51	0.529407387	0.266	0.073	1.55E-46	11
BSG.5	7.65E-51	0.592686902	0.645	0.318	1.76E-46	11
ACTB.6	8.46E-51	-0.908505393	0.965	0.928	1.94E-46	11

SLC39A10.1	1.63E-50	0.537206656	0.306	0.093	3.75E-46	11
SERINC1	1.67E-50	0.662529876	0.497	0.209	3.83E-46	11
PKN2.1	2.38E-50	0.536551777	0.39	0.138	5.47E-46	11
HLA-B.8	2.90E-50	0.512083475	0.998	0.911	6.66E-46	11
LCP1.9	6.76E-50	-1.567149761	0.071	0.415	1.55E-45	11
PPIB.3	8.58E-50	0.668522828	0.805	0.477	1.97E-45	11
GZMA.10	1.41E-49	-2.446572978	0.098	0.43	3.24E-45	11
RPS11.5	3.15E-49	-0.83718436	0.889	0.854	7.24E-45	11
ADD3.1	3.80E-49	0.607953108	0.424	0.159	8.72E-45	11
TMEM59.3	3.97E-49	0.600472719	0.796	0.466	9.13E-45	11
DYNC1I2.4	5.10E-49	0.562166608	0.528	0.234	1.17E-44	11
SLIRP.6	5.54E-49	0.590236697	0.563	0.265	1.27E-44	11
TMEM165.4	7.56E-49	0.624934109	0.523	0.229	1.74E-44	11
BAZ2B.1	8.38E-49	0.661058252	0.404	0.152	1.93E-44	11
ITGB2.9	8.48E-49	-1.576789945	0.084	0.426	1.95E-44	11
STK4.7	1.61E-48	-1.659006165	0.153	0.483	3.70E-44	11
RFC1.2	1.78E-48	0.684232047	0.446	0.179	4.08E-44	11
MRPL33.2	1.85E-48	0.605477716	0.592	0.282	4.25E-44	11
RILPL2	2.31E-48	0.597484244	0.47	0.194	5.31E-44	11
RAPGEF1	2.51E-48	0.532052933	0.257	0.072	5.78E-44	11
FMNL3.1	3.56E-48	0.530369933	0.255	0.071	8.18E-44	11
CD48.9	4.03E-48	-1.448904993	0.044	0.386	9.26E-44	11
COX17.5	4.33E-48	0.679747017	0.603	0.309	9.96E-44	11
CD3E.10	6.15E-48	-1.730563059	0.073	0.408	1.41E-43	11
IL2RG.11	9.93E-48	-1.615098151	0.047	0.383	2.28E-43	11
MT-ATP6.6	1.53E-47	0.563962015	0.98	0.855	3.51E-43	11
CAPN2.1	2.62E-47	0.54960552	0.477	0.193	6.03E-43	11
MT-CYB.7	3.56E-47	0.578202856	0.978	0.799	8.19E-43	11
C1orf54.2	9.10E-47	0.563831624	0.271	0.081	2.09E-42	11
KLF7.1	9.56E-47	0.538180261	0.277	0.085	2.20E-42	11
UVRAG	1.13E-46	0.565631011	0.29	0.09	2.61E-42	11
GUK1.3	1.84E-46	0.581723918	0.785	0.497	4.24E-42	11
ENTPD1.1	5.92E-46	0.59913759	0.388	0.146	1.36E-41	11
DYNLL1.5	1.80E-45	0.559620125	0.707	0.399	4.14E-41	11
EIF1.2	4.22E-45	-0.702986092	0.92	0.896	9.70E-41	11
NKTR.8	5.56E-45	0.696289805	0.654	0.336	1.28E-40	11
PRMT1	6.87E-45	0.502645629	0.41	0.16	1.58E-40	11
BNIP2	9.25E-45	0.60858414	0.43	0.175	2.12E-40	11
NCK1	1.73E-44	0.563615065	0.417	0.165	3.99E-40	11
CYBA.7	2.69E-44	-1.377738712	0.583	0.69	6.19E-40	11
RP11-347P5.1.10	3.93E-44	-1.79280684	0.067	0.386	9.02E-40	11
RPL23.4	4.08E-44	-0.906319345	0.823	0.817	9.38E-40	11
AFF1	5.57E-44	0.584359495	0.302	0.1	1.28E-39	11
COX5B.4	5.60E-44	0.52727149	0.812	0.537	1.29E-39	11
GNAQ.2	8.38E-44	0.624423753	0.348	0.129	1.93E-39	11
TAGLN2.8	1.27E-43	0.529356715	0.645	0.347	2.91E-39	11
EFCAB14	1.92E-43	0.594460182	0.359	0.134	4.42E-39	11
DENND3.1	2.16E-43	0.511802746	0.282	0.09	4.96E-39	11
DAD1.4	3.13E-43	0.531975516	0.652	0.35	7.18E-39	11
GNB1	3.50E-43	0.573488789	0.443	0.186	8.05E-39	11
PDIA6.4	3.98E-43	0.592818434	0.588	0.296	9.14E-39	11
ACAP1.11	4.30E-43	-1.49406702	0.031	0.343	9.88E-39	11
ITGAV.1	5.53E-43	0.515764283	0.251	0.076	1.27E-38	11
IFITM1.2	5.94E-43	0.583924191	0.439	0.173	1.37E-38	11
PCMTD1.1	6.16E-43	0.657543232	0.408	0.168	1.42E-38	11
TM9SF3	6.37E-43	0.608978736	0.333	0.12	1.46E-38	11
RAC2.11	9.65E-43	-1.452972101	0.069	0.383	2.22E-38	11
LMAN1	1.67E-42	0.524794757	0.439	0.182	3.84E-38	11

DUSP2.10	1.95E-42	-2.238933616	0.146	0.441	4.49E-38	11
ARF1.1	3.24E-42	0.534288786	0.696	0.399	7.44E-38	11
KCNQ1OT1	3.67E-42	0.648984116	0.282	0.091	8.44E-38	11
CST7.10	8.99E-42	-2.080086261	0.1	0.399	2.07E-37	11
OAZ1.7	1.12E-41	-1.11649857	0.687	0.737	2.58E-37	11
PRKAR1A.1	1.79E-41	0.530169513	0.532	0.252	4.12E-37	11
RAB2A.4	2.50E-41	0.51680966	0.539	0.261	5.75E-37	11
EVI2B.6	3.14E-41	-1.36782926	0.04	0.345	7.22E-37	11
DDIT4.7	4.25E-41	0.62153055	0.807	0.508	9.76E-37	11
CAPNS1	8.30E-41	0.555625546	0.388	0.156	1.91E-36	11
PDIA3.3	2.36E-40	0.636012168	0.707	0.417	5.43E-36	11
CPM.2	3.98E-40	0.593682271	0.35	0.13	9.15E-36	11
ATP1B3.8	5.11E-40	0.525358076	0.545	0.259	1.18E-35	11
TRBC2.10	5.37E-40	-1.887651792	0.093	0.386	1.23E-35	11
GIMAP1	5.70E-40	0.537890252	0.377	0.145	1.31E-35	11
AFF4	7.53E-40	0.513327194	0.392	0.159	1.73E-35	11
EVL.10	8.75E-40	-1.48647898	0.231	0.504	2.01E-35	11
WASF2.5	9.68E-40	0.589599989	0.61	0.321	2.23E-35	11
GNB2.2	1.83E-39	0.530068424	0.521	0.255	4.20E-35	11
TMEM109.1	2.04E-39	0.533434233	0.386	0.157	4.70E-35	11
FRYL	2.09E-38	0.509657933	0.341	0.128	4.81E-34	11
STK17B.7	6.41E-38	-1.445874104	0.122	0.41	1.47E-33	11
NFE2L2.8	9.60E-38	0.565300988	0.523	0.254	2.21E-33	11
GTF2I.2	1.17E-37	0.548797469	0.439	0.199	2.70E-33	11
RAB5C.6	1.29E-37	0.520554219	0.521	0.26	2.97E-33	11
CALM1.5	1.81E-37	0.693566394	0.827	0.588	4.15E-33	11
CD99.7	1.83E-37	0.578106205	0.745	0.461	4.21E-33	11
SPAG9	2.03E-37	0.564586979	0.348	0.139	4.66E-33	11
UCP2.8	2.22E-37	-1.290922713	0.067	0.356	5.10E-33	11
CD3G.10	3.57E-37	-1.530372722	0.024	0.302	8.20E-33	11
PABPC1.6	1.74E-36	-1.191796516	0.581	0.665	3.99E-32	11
OS9.2	2.53E-36	0.501893508	0.463	0.217	5.82E-32	11
RARRES3.9	9.71E-36	-1.389122481	0.144	0.419	2.23E-31	11
LITAF.8	1.02E-35	-1.350342653	0.1	0.376	2.34E-31	11
RPL17.8	1.30E-35	-1.450277811	0.428	0.599	3.00E-31	11
ANKRD11.2	1.95E-35	0.516936261	0.479	0.222	4.48E-31	11
NPM1.5	2.15E-35	-1.143910348	0.614	0.701	4.93E-31	11
ISG15.1	3.93E-35	0.532234821	0.443	0.203	9.02E-31	11
SYNE2.10	5.61E-35	0.559974256	0.634	0.322	1.29E-30	11
CD53.6	1.22E-34	-1.303558853	0.231	0.479	2.81E-30	11
CANX.6	1.28E-34	0.510229966	0.557	0.291	2.95E-30	11
RSF1.4	1.50E-34	0.506567814	0.508	0.248	3.44E-30	11
EVI2A.7	4.68E-34	-1.168104822	0.027	0.288	1.08E-29	11
TNFAIP3.11	6.49E-34	-1.655433959	0.206	0.455	1.49E-29	11
TRBC1.10	8.16E-34	-2.126226746	0.102	0.362	1.87E-29	11
SAMSN1.5	1.31E-33	-1.389521238	0.064	0.331	3.01E-29	11
FTH1.10	1.98E-33	-1.393073479	0.971	0.962	4.56E-29	11
GPSM3.7	2.16E-33	-1.219803498	0.137	0.399	4.96E-29	11
TRAM1.2	2.49E-33	0.516796624	0.539	0.282	5.72E-29	11
ETS2.2	2.74E-33	0.525829136	0.29	0.113	6.30E-29	11
NKG7.11	3.52E-33	-2.717729175	0.2	0.435	8.10E-29	11
CCL4.11	3.76E-33	-2.847419089	0.208	0.448	8.65E-29	11
EMB.8	1.13E-32	-1.13285552	0.024	0.277	2.59E-28	11
LIMD2.10	1.17E-32	-1.207013625	0.06	0.319	2.69E-28	11
CD7.8	1.58E-32	-1.560879231	0.051	0.308	3.63E-28	11
STK17A.10	2.13E-32	-1.336729021	0.12	0.375	4.90E-28	11
SYTL3.10	2.16E-32	-1.311929281	0.031	0.285	4.97E-28	11
NR4A1.2	2.52E-32	0.618532938	0.288	0.11	5.80E-28	11

LTB.10	3.08E-32	-1.791995707	0.058	0.309	7.09E-28	11
ALOX5AP.8	4.38E-32	-1.638541064	0.084	0.337	1.01E-27	11
RGS10.7	2.46E-31	-1.201309396	0.053	0.303	5.65E-27	11
MT2A.11	2.93E-30	-2.238772762	0.492	0.665	6.72E-26	11
FABP5.2	3.89E-30	1.35880573	0.397	0.2	8.95E-26	11
TRAF3IP3.11	4.62E-30	-1.09387776	0.027	0.263	1.06E-25	11
EEF1B2.4	7.51E-30	-1.050929982	0.588	0.664	1.73E-25	11
GLIPR1.8	8.22E-30	-1.125247984	0.062	0.307	1.89E-25	11
COTL1.6	8.57E-30	-1.532947382	0.228	0.441	1.97E-25	11
IL32.10	2.69E-29	-1.648673347	0.43	0.583	6.18E-25	11
DOCK8.9	4.65E-29	-1.051752232	0.062	0.299	1.07E-24	11
BIRC3.9	5.22E-29	-1.500067224	0.044	0.279	1.20E-24	11
BIN2.8	1.03E-28	-1.080541073	0.027	0.257	2.36E-24	11
IKZF1.10	1.09E-28	-1.009412355	0.022	0.251	2.51E-24	11
MSN.5	1.41E-28	0.614745035	0.599	0.359	3.25E-24	11
NACA.4	1.87E-28	-0.760335632	0.838	0.802	4.30E-24	11
EEF1D.7	2.25E-28	-0.807756827	0.778	0.77	5.16E-24	11
LCK.11	2.78E-28	-1.149285242	0.06	0.295	6.40E-24	11
PPDPF.7	2.80E-28	-1.186220582	0.541	0.663	6.44E-24	11
GZMK.11	6.58E-28	-2.506440429	0.153	0.372	1.51E-23	11
BTG2.7	6.78E-28	-1.370164659	0.131	0.365	1.56E-23	11
RGS2.10	1.27E-27	-1.810912412	0.118	0.342	2.92E-23	11
RPL22.5	2.02E-27	-0.741215399	0.856	0.809	4.65E-23	11
MYO1F.6	2.10E-27	-0.97197129	0.049	0.279	4.84E-23	11
IL10RA.8	2.25E-27	-1.013385874	0.04	0.266	5.18E-23	11
ADGRE5.9	6.31E-27	-1.228639738	0.08	0.307	1.45E-22	11
PNRC1.5	3.85E-26	-1.097956728	0.55	0.637	8.85E-22	11
IL7R.9	5.02E-26	-2.22722197	0.095	0.31	1.15E-21	11
PIK3IP1.10	5.78E-26	-1.220258183	0.144	0.365	1.33E-21	11
FAM49B.6	7.30E-26	-1.090478938	0.124	0.347	1.68E-21	11
APOBEC3G.9	1.50E-25	-1.192292924	0.06	0.278	3.45E-21	11
CD96.11	1.60E-25	-1.120730314	0.044	0.256	3.69E-21	11
RPL36AL.4	1.68E-25	-0.912167812	0.676	0.713	3.85E-21	11
EMP3.5	2.08E-25	-1.230748981	0.18	0.397	4.78E-21	11
SARAF.5	2.71E-25	-1.009357733	0.636	0.679	6.23E-21	11
DNAJB1.9	8.79E-25	-1.608663425	0.231	0.426	2.02E-20	11
TSTD1.4	8.96E-25	-0.973972184	0.042	0.253	2.06E-20	11
HMGB2.7	1.07E-24	-1.22178796	0.279	0.483	2.45E-20	11
SH3BGRL3.7	7.56E-24	-1.042575598	0.661	0.682	1.74E-19	11
MYH9.7	7.59E-24	0.507574479	0.525	0.304	1.74E-19	11
SEPT6.8	1.27E-23	-0.943226319	0.075	0.284	2.92E-19	11
CLEC2D.10	1.87E-23	-1.252592292	0.109	0.313	4.30E-19	11
PIK3R1.8	1.88E-23	-1.163771167	0.109	0.315	4.31E-19	11
H3F3B.7	2.93E-23	-0.701046587	0.902	0.832	6.73E-19	11
CD27.10	6.56E-23	-1.489062445	0.078	0.274	1.51E-18	11
FTL.9	1.08E-22	-1.478719975	0.949	0.937	2.48E-18	11
PFDN5.3	1.08E-22	-0.709042446	0.834	0.782	2.49E-18	11
LY6E.7	3.16E-22	-1.100300141	0.169	0.372	7.26E-18	11
NBEAL1.4	3.69E-22	-1.01199641	0.532	0.618	8.49E-18	11
CRIP1.9	4.67E-22	-1.303911127	0.102	0.3	1.07E-17	11
AKNA.8	1.34E-21	-0.967779139	0.073	0.267	3.08E-17	11
ZFAS1.8	5.65E-21	-1.232042837	0.326	0.489	1.30E-16	11
HLA-DQA1.8	7.49E-21	-1.960897968	0.173	0.349	1.72E-16	11
ATP5G2.3	1.13E-19	-0.896434284	0.63	0.656	2.60E-15	11
AC090498.1.6	1.30E-19	-1.210675986	0.364	0.505	2.99E-15	11
RNASET2.10	2.06E-19	-1.411611648	0.2	0.366	4.72E-15	11
LDHB.7	3.05E-19	-1.042957261	0.308	0.472	7.00E-15	11
TOMM7.5	3.65E-19	-0.727544762	0.789	0.753	8.38E-15	11

COX7C.3	1.04E-18	-0.747749226	0.772	0.753	2.38E-14	11
PPP2R5C.9	1.57E-18	-1.134156844	0.226	0.396	3.60E-14	11
RCSD1.8	4.84E-18	-0.781543523	0.082	0.255	1.11E-13	11
ICAM3.10	6.56E-18	-0.919038075	0.109	0.278	1.51E-13	11
SRSF7.8	1.14E-17	-1.129524995	0.439	0.548	2.61E-13	11
SLC25A6.4	1.35E-17	-0.860561542	0.641	0.656	3.11E-13	11
TAF7.6	1.54E-17	-0.883445896	0.151	0.326	3.53E-13	11
SH3KBP1.8	5.96E-17	-0.783400166	0.14	0.314	1.37E-12	11
SNHG8.4	3.07E-16	-1.013730273	0.302	0.439	7.06E-12	11
SMAP2.7	3.38E-16	-0.878247284	0.142	0.303	7.77E-12	11
BTF3.2	3.92E-16	-0.670134051	0.729	0.726	9.01E-12	11
TUBA4A.9	4.90E-16	-1.072736655	0.122	0.278	1.13E-11	11
ANXA1.10	6.70E-16	-1.354927359	0.355	0.475	1.54E-11	11
MT1X.6	1.07E-15	-2.393817935	0.129	0.288	2.45E-11	11
RGS5.1	2.04E-15	-0.645277295	0.255	0.122	4.70E-11	11
WIPF1.11	2.23E-15	-0.871381383	0.191	0.348	5.13E-11	11
TYROBP.11	2.70E-15	-2.074191762	0.282	0.391	6.19E-11	11
GLUL.8	2.84E-15	-1.333883114	0.166	0.311	6.54E-11	11
GAPDH.7	3.78E-15	-0.987265827	0.916	0.864	8.68E-11	11
HMGN2.3	4.28E-15	-0.980406372	0.401	0.503	9.85E-11	11
NAP1L1.3	8.64E-15	-0.893452719	0.512	0.567	1.99E-10	11
NR4A2.8	8.69E-15	-1.013161199	0.131	0.281	2.00E-10	11
AAK1.10	1.81E-14	-0.860470194	0.228	0.375	4.17E-10	11
FCER1G.11	2.01E-14	-1.749557485	0.177	0.307	4.61E-10	11
SERPINA1.10	9.21E-14	-1.572872277	0.146	0.283	2.12E-09	11
CITED2.9	1.11E-13	-1.089250834	0.131	0.272	2.55E-09	11
DUSP1.7	1.15E-13	-0.829407113	0.887	0.79	2.64E-09	11
JUN.7	1.32E-13	-1.026259014	0.676	0.692	3.02E-09	11
HCLS1.6	2.16E-13	-0.814072257	0.191	0.327	4.96E-09	11
ATP5L.2	2.83E-13	-0.677712224	0.69	0.674	6.51E-09	11
LEPROTL1.5	3.78E-13	-0.95710508	0.266	0.393	8.68E-09	11
ANKRD44.8	3.88E-13	-0.736630645	0.122	0.263	8.93E-09	11
PRPF38B.10	3.90E-13	-0.882126472	0.326	0.439	8.97E-09	11
TXNIP.9	9.02E-13	-0.88479936	0.851	0.76	2.07E-08	11
CCNH.8	1.15E-12	-0.865206549	0.153	0.289	2.64E-08	11
ANXA6.8	1.21E-12	-0.821874865	0.122	0.254	2.77E-08	11
SRGN.7	1.83E-12	-0.649528826	0.858	0.789	4.21E-08	11
CST3.10	6.51E-12	-0.854031682	0.676	0.376	1.50E-07	11
EEF2.4	8.49E-12	-0.694899838	0.738	0.683	1.95E-07	11
EZR.3	1.13E-11	-0.847546577	0.204	0.328	2.59E-07	11
COMMD6.4	1.59E-11	-0.709037907	0.585	0.606	3.66E-07	11
ENO1.9	1.79E-11	-1.243674877	0.572	0.594	4.10E-07	11
FNBP1.10	2.05E-11	-0.783402905	0.251	0.371	4.72E-07	11
IDS.8	2.36E-11	-0.899939112	0.259	0.377	5.43E-07	11
HLA-DQB1.9	4.13E-11	-1.579452069	0.313	0.395	9.48E-07	11
CMC1.9	6.93E-11	-1.525656743	0.173	0.289	1.59E-06	11
EIF3H.1	8.12E-11	-0.737640509	0.361	0.454	1.87E-06	11
PLP2.6	1.22E-10	-0.809160858	0.182	0.299	2.80E-06	11
HLA-DPB1.10	1.64E-10	-1.718026087	0.672	0.631	3.76E-06	11
FOS.9	1.69E-10	-0.980555572	0.712	0.681	3.89E-06	11
JUNB.10	1.87E-10	-1.000542712	0.783	0.721	4.30E-06	11
NNMT.11	4.40E-10	-2.075165666	0.193	0.303	1.01E-05	11
HLA-DRB1.11	4.48E-10	-0.65264384	0.851	0.582	1.03E-05	11
PRR13.1	5.80E-10	-0.780086511	0.293	0.395	1.33E-05	11
MCL1.6	6.07E-10	-0.799952523	0.452	0.517	1.39E-05	11
ZNF90.4	7.94E-10	-0.803973439	0.248	0.357	1.82E-05	11
MZT2A.7	1.50E-09	-0.710207676	0.16	0.275	3.44E-05	11
GLRX.4	2.33E-09	-0.874783058	0.197	0.301	5.36E-05	11

GLTSCR2.5	2.39E-09	-0.668965502	0.548	0.589	5.50E-05	11
C9orf142.8	3.23E-09	-0.629525635	0.153	0.264	7.43E-05	11
AIF1.11	3.85E-09	-1.479435445	0.166	0.262	8.84E-05	11
EIF3E.4	5.54E-09	-0.746884451	0.528	0.545	0.000127395	11
REL.7	5.66E-09	-0.846182267	0.233	0.325	0.000130136	11
YPEL5.9	9.20E-09	-0.813599849	0.251	0.347	0.000211353	11
NPC2.8	1.27E-08	-0.518641841	0.61	0.371	0.000291591	11
RPL22L1.2	2.39E-08	-0.609082161	0.157	0.264	0.000549839	11
ADIRF.8	4.68E-08	-0.994996166	0.379	0.237	0.00107551	11
EIF3M.4	7.05E-08	-0.645216883	0.213	0.309	0.001619763	11
EIF3L.4	1.12E-07	-0.666125684	0.297	0.377	0.002584565	11
EIF4A2.6	1.18E-07	-0.670999778	0.565	0.58	0.002712731	11
LYZ.10	1.27E-07	-2.405024247	0.299	0.353	0.002913409	11
VAMP8.5	2.21E-07	-0.669980747	0.417	0.462	0.00508205	11
ARID5B.5	2.25E-07	-0.801607037	0.166	0.258	0.005181932	11
SNRPD2.4	2.46E-07	-0.679375093	0.525	0.558	0.005645875	11
ZFP36.7	2.54E-07	-0.717821961	0.694	0.662	0.005831663	11
ARF6.6	2.62E-07	-0.720678449	0.302	0.382	0.006012449	11
OCIAD2.8	4.22E-07	-0.761692784	0.177	0.264	0.009698135	11
CYCS.5	4.23E-07	-0.775677388	0.335	0.398	0.009729947	11
CIB1.1	4.51E-07	-0.634963317	0.304	0.39	0.010363001	11
HIGD2A.1	6.06E-07	-0.641738171	0.324	0.392	0.013921828	11
TYMP.8	1.30E-06	-0.890254428	0.226	0.298	0.029864072	11
RTKN2	0	1.922615944	0.447	0.025	0	12
FOXP3	0	1.415440225	0.389	0.013	0	12
AC002331.1	2.01E-293	1.328993035	0.265	0.01	4.63E-289	12
TIGIT.2	8.75E-254	2.125496457	0.718	0.12	2.01E-249	12
TBC1D4.1	4.06E-249	1.792921766	0.576	0.076	9.33E-245	12
TNFRSF18	1.29E-238	2.005002163	0.49	0.055	2.97E-234	12
IL2RA	5.53E-228	1.330059727	0.305	0.021	1.27E-223	12
BATF.1	6.24E-221	2.062059527	0.709	0.133	1.43E-216	12
CTLA4	1.87E-187	1.454397681	0.447	0.057	4.29E-183	12
LAIR2	4.85E-186	1.132287481	0.288	0.023	1.11E-181	12
AC133644.2	2.36E-180	1.598190212	0.444	0.059	5.43E-176	12
PMAIP1.4	2.80E-179	2.080686365	0.761	0.188	6.43E-175	12
IL32.11	1.62E-155	2.051158177	0.974	0.568	3.72E-151	12
LTB.11	1.76E-147	2.0092372	0.856	0.287	4.04E-143	12
ICA1.1	6.74E-139	1.443521068	0.346	0.047	1.55E-134	12
SLAMF1	1.35E-132	1.150139868	0.314	0.039	3.09E-128	12
TNFRSF4.1	1.14E-128	1.743406696	0.395	0.064	2.62E-124	12
HPGD	7.19E-128	1.269729972	0.346	0.048	1.65E-123	12
CARD16.6	1.97E-125	1.769278643	0.784	0.305	4.54E-121	12
ICOS.1	6.45E-118	1.403345218	0.438	0.083	1.48E-113	12
TRAC.12	6.96E-114	1.491577709	0.939	0.419	1.60E-109	12
BTG3.1	1.06E-106	1.551291691	0.582	0.164	2.44E-102	12
CD27.11	1.90E-105	1.199457845	0.781	0.254	4.36E-101	12
S100A4.10	7.88E-96	1.31108734	0.98	0.692	1.81E-91	12
CORO1B.4	1.54E-90	1.514542419	0.625	0.23	3.53E-86	12
RP11-347P5.1.11	3.47E-83	1.238934986	0.813	0.364	7.98E-79	12
SELL	1.23E-82	1.086126971	0.38	0.085	2.82E-78	12
UGP2.8	3.72E-81	1.394700504	0.706	0.316	8.55E-77	12
ARID5B.6	1.40E-80	1.155297927	0.666	0.244	3.22E-76	12
STAM	1.16E-79	0.888625005	0.262	0.044	2.67E-75	12
SAMSN1.6	2.36E-74	1.125842697	0.723	0.312	5.42E-70	12
CD79B	5.56E-73	0.845716913	0.277	0.052	1.28E-68	12
BIRC3.10	2.56E-71	1.190455921	0.66	0.262	5.88E-67	12
ENTPD1.2	1.71E-65	1.214602304	0.45	0.146	3.93E-61	12
CD2.11	1.01E-62	0.938059472	0.89	0.446	2.33E-58	12

TNFRSF9.3	4.76E-61	0.954412651	0.369	0.095	1.09E-56	12
GBP2.5	3.48E-60	1.14546286	0.591	0.254	8.01E-56	12
RGS1.12	2.44E-59	0.93121007	0.983	0.689	5.61E-55	12
UCP2.9	1.18E-58	1.147163911	0.68	0.338	2.70E-54	12
PHTF2.1	4.22E-58	0.97400432	0.366	0.104	9.69E-54	12
CLEC2D.11	2.70E-56	1.22984907	0.651	0.298	6.20E-52	12
CTSC.9	1.97E-55	1.068166322	0.68	0.338	4.54E-51	12
SPOCK2.3	6.51E-55	0.931320663	0.571	0.226	1.50E-50	12
RP11-138A9.1.1	2.13E-54	0.953051892	0.504	0.183	4.90E-50	12
TSC22D3.8	1.94E-52	0.840797274	0.988	0.775	4.45E-48	12
P2RY10.1	5.28E-52	0.839084322	0.326	0.089	1.21E-47	12
CD3D.12	3.76E-51	0.801316944	0.885	0.464	8.64E-47	12
NCK2	1.91E-50	0.765477166	0.28	0.07	4.38E-46	12
IL2RG.12	1.08E-49	0.985926603	0.712	0.364	2.49E-45	12
AC016831.7.1	3.08E-49	0.934276146	0.478	0.178	7.09E-45	12
PBXIP1.2	2.29E-48	0.985537517	0.476	0.186	5.26E-44	12
ISG20.9	2.67E-48	0.924653838	0.686	0.353	6.13E-44	12
DNPH1	4.08E-48	0.966685443	0.386	0.132	9.38E-44	12
TRBC2.11	4.06E-46	1.047073445	0.72	0.368	9.34E-42	12
PRDM1.7	3.67E-45	0.969660572	0.608	0.284	8.44E-41	12
PIK3IP1.11	5.30E-45	0.909407487	0.671	0.35	1.22E-40	12
PHLDA1	5.38E-45	0.886771469	0.403	0.141	1.24E-40	12
SKAP1.4	1.31E-43	0.890870231	0.458	0.178	3.02E-39	12
CYTIP.10	1.02E-42	0.774322364	0.784	0.467	2.34E-38	12
DUSP4.7	4.26E-42	0.842967289	0.553	0.233	9.79E-38	12
FYB.10	1.08E-41	0.977811488	0.749	0.452	2.48E-37	12
RPS26.8	1.65E-41	0.559436938	0.988	0.867	3.80E-37	12
PIM2	6.78E-41	0.775855452	0.3	0.09	1.56E-36	12
ARPC1B.4	3.03E-40	0.715797918	0.833	0.517	6.96E-36	12
BTG1.11	4.36E-40	0.571486323	1	0.838	1.00E-35	12
LINC00152.7	8.30E-40	0.785135138	0.614	0.309	1.91E-35	12
HLA-A.8	3.03E-39	0.538641937	0.991	0.891	6.97E-35	12
LCK.12	3.94E-39	0.730510827	0.602	0.279	9.05E-35	12
SIRPG.2	8.42E-39	0.737851484	0.28	0.083	1.94E-34	12
ARHGDIB.5	8.82E-39	0.716165936	0.919	0.666	2.03E-34	12
RP11-138A9.2.1	2.03E-38	0.876557685	0.455	0.185	4.68E-34	12
ACTB.7	3.59E-38	0.586295296	1	0.927	8.26E-34	12
ANXA1.11	1.91E-37	-1.8684194	0.127	0.48	4.39E-33	12
CCL5.11	8.35E-37	-2.741520623	0.294	0.569	1.92E-32	12
KLF6.10	1.83E-36	0.706222049	0.919	0.642	4.22E-32	12
CCNG2	4.57E-36	0.744725454	0.265	0.083	1.05E-31	12
GATA3	8.13E-36	0.806655054	0.288	0.092	1.87E-31	12
TNFAIP3.12	2.47E-35	0.700368568	0.752	0.439	5.67E-31	12
GZMA.11	5.09E-35	-2.085957015	0.104	0.427	1.17E-30	12
DUSP10	8.27E-35	0.895385241	0.288	0.097	1.90E-30	12
STAT3.8	3.57E-34	0.853300891	0.562	0.312	8.21E-30	12
MIR4435-2HG.1	1.49E-33	0.696573885	0.369	0.146	3.41E-29	12
USP15.5	1.82E-33	0.905793253	0.499	0.257	4.19E-29	12
IPCEF1.1	2.31E-32	0.717856622	0.291	0.099	5.30E-28	12
GADD45A	3.36E-32	0.688094605	0.291	0.101	7.73E-28	12
CD3E.11	1.68E-31	0.608813189	0.723	0.389	3.87E-27	12
ACP5.1	2.46E-31	0.677096676	0.28	0.098	5.66E-27	12
RORA.2	1.89E-30	0.684516868	0.47	0.212	4.34E-26	12
GCHFR	3.32E-30	0.737060216	0.363	0.151	7.62E-26	12
CD7.9	2.04E-29	0.576790273	0.579	0.293	4.68E-25	12
ICAM3.11	2.13E-29	0.676163024	0.519	0.266	4.90E-25	12
ARL6IP5.8	2.68E-29	0.68638535	0.695	0.452	6.15E-25	12
RAC2.12	6.63E-29	0.694072748	0.634	0.366	1.52E-24	12

FAM129A.2	2.34E-28	0.792501548	0.421	0.2	5.37E-24	12
NKG7.12	2.43E-28	-2.483068296	0.159	0.434	5.57E-24	12
GBP5.1	3.40E-28	0.727073614	0.277	0.1	7.81E-24	12
CCL4.12	5.18E-28	-2.823262997	0.179	0.446	1.19E-23	12
STK17B.8	2.26E-27	0.674134251	0.646	0.394	5.19E-23	12
ITM2A.11	4.55E-27	0.607706104	0.585	0.303	1.05E-22	12
PTPRC.11	5.38E-27	0.527890175	0.893	0.609	1.24E-22	12
GMFG.5	1.27E-26	0.542551803	0.801	0.55	2.92E-22	12
CCNI.1	1.48E-26	0.580847514	0.824	0.587	3.40E-22	12
CALM3.5	1.60E-26	0.601327203	0.539	0.307	3.69E-22	12
PHACTR2.1	1.83E-26	0.606576709	0.277	0.104	4.21E-22	12
GLRX.5	2.55E-26	0.586461647	0.516	0.292	5.86E-22	12
TRBC1.11	9.54E-26	1.016873689	0.585	0.347	2.19E-21	12
SRSF7.9	1.69E-25	0.586583065	0.764	0.539	3.89E-21	12
FCMR	3.83E-25	0.564386109	0.256	0.092	8.81E-21	12
CYCS.6	5.25E-25	0.639532331	0.617	0.391	1.21E-20	12
C9orf16.3	5.43E-25	0.670965547	0.556	0.344	1.25E-20	12
CD44.8	7.14E-25	0.565348567	0.746	0.485	1.64E-20	12
LAT.3	1.32E-24	0.598634471	0.366	0.162	3.04E-20	12
RASGRP1.2	1.71E-24	0.612935238	0.305	0.123	3.94E-20	12
ZBTB38.3	5.89E-24	0.632687322	0.447	0.232	1.35E-19	12
WHSC1L1.6	1.28E-23	0.680572015	0.441	0.235	2.95E-19	12
GZMK.12	1.32E-23	-2.190954147	0.124	0.372	3.04E-19	12
TNFRSF1B.2	1.58E-23	0.702791136	0.429	0.228	3.62E-19	12
IQGAP1.5	1.63E-23	0.722500238	0.542	0.333	3.75E-19	12
PELI1	1.78E-23	0.711119442	0.268	0.11	4.10E-19	12
ANAPC16.3	2.99E-23	0.523166501	0.735	0.534	6.87E-19	12
UBE2D2.2	3.20E-23	0.588694541	0.591	0.38	7.36E-19	12
CD53.7	5.35E-23	0.5696824	0.692	0.465	1.23E-18	12
HSPA8.6	1.43E-22	0.609857977	0.761	0.549	3.28E-18	12
ALOX5AP.9	4.46E-22	-1.482093768	0.095	0.335	1.03E-17	12
CREB3L2	1.40E-21	0.635912428	0.259	0.108	3.22E-17	12
RHOH.4	1.62E-21	0.678970471	0.418	0.222	3.72E-17	12
CASP1.1	2.69E-21	0.658889896	0.38	0.2	6.18E-17	12
TYROBP.12	4.98E-21	-2.156934937	0.173	0.393	1.14E-16	12
CLPP.2	5.73E-21	0.577976488	0.372	0.186	1.32E-16	12
HTATIP2	1.28E-20	0.609733967	0.268	0.118	2.95E-16	12
BUB3.8	1.31E-20	0.590136131	0.467	0.266	3.01E-16	12
EVI2A.8	4.57E-20	0.679711688	0.467	0.275	1.05E-15	12
CD63.10	7.85E-20	-1.363945929	0.337	0.524	1.80E-15	12
DDX24.8	9.78E-20	0.521167901	0.657	0.452	2.25E-15	12
SEPT6.9	1.44E-19	0.627386636	0.461	0.273	3.31E-15	12
GLUL.9	2.23E-19	-1.420709421	0.092	0.312	5.14E-15	12
EPSTI1.1	2.98E-19	0.623704318	0.277	0.127	6.85E-15	12
SLA.10	3.66E-19	0.551726932	0.605	0.394	8.42E-15	12
CHST11	8.75E-19	0.577825823	0.349	0.18	2.01E-14	12
CLIP1	1.16E-18	0.673389493	0.3	0.147	2.66E-14	12
RSBN1.1	1.29E-18	0.59710082	0.334	0.167	2.97E-14	12
RAP1A.5	2.07E-18	0.550465466	0.522	0.346	4.77E-14	12
CEBPD.7	2.49E-18	-1.357546513	0.078	0.284	5.71E-14	12
GALM.3	2.78E-18	0.581394838	0.326	0.161	6.40E-14	12
RHBDD2	2.04E-17	0.60343734	0.251	0.113	4.68E-13	12
IL10RA.9	5.12E-17	0.560447811	0.429	0.254	1.18E-12	12
AKIRIN2.1	5.39E-17	0.576403808	0.346	0.186	1.24E-12	12
GSTP1.6	7.68E-17	-1.175242026	0.294	0.494	1.76E-12	12
NABP1.1	1.26E-16	0.578957972	0.34	0.184	2.89E-12	12
GIMAP4.8	5.08E-16	-0.877183235	0.133	0.344	1.17E-11	12
RAB9A	2.34E-15	0.570923658	0.254	0.121	5.38E-11	12

GIMAP7.11	5.64E-15	-0.948617089	0.098	0.289	1.30E-10	12
ZNF292.2	5.70E-15	0.572100524	0.329	0.182	1.31E-10	12
PPM1G.1	1.28E-14	0.504858087	0.372	0.222	2.94E-10	12
RNF145.2	2.26E-14	0.566124578	0.375	0.227	5.20E-10	12
CST3.11	2.36E-14	-2.053292661	0.231	0.389	5.43E-10	12
CDV3	3.38E-14	0.50108445	0.294	0.157	7.78E-10	12
FTH1.11	6.97E-14	-1.242249404	0.983	0.962	1.60E-09	12
FCER1G.12	7.65E-14	-1.649508279	0.141	0.307	1.76E-09	12
NR3C1.3	1.25E-13	0.578738354	0.372	0.228	2.88E-09	12
DEF6.1	2.32E-13	0.503014597	0.297	0.163	5.34E-09	12
SERPINA1.11	7.07E-13	-1.589468011	0.121	0.282	1.63E-08	12
CLEC2B.6	1.04E-12	-0.765314533	0.19	0.383	2.39E-08	12
IFNGR1.9	2.98E-12	-0.924556376	0.101	0.263	6.84E-08	12
WNK1.7	5.58E-12	0.523037744	0.383	0.246	1.28E-07	12
IFITM3.11	8.68E-12	-1.644245303	0.219	0.382	2.00E-07	12
BCL2.2	1.15E-11	0.505211044	0.294	0.168	2.65E-07	12
ZC3H7A.2	3.72E-11	0.555400985	0.271	0.156	8.56E-07	12
RGS2.11	7.10E-11	-1.274901757	0.187	0.339	1.63E-06	12
DSTN.10	7.35E-11	-1.123307599	0.15	0.311	1.69E-06	12
RHOB.10	7.73E-11	-1.252858636	0.199	0.352	1.78E-06	12
CRYAB.11	1.07E-10	-1.848904496	0.112	0.263	2.47E-06	12
RNASET2.11	5.15E-10	-1.079559903	0.225	0.364	1.18E-05	12
CEBPB.9	1.30E-09	-1.007693251	0.19	0.332	2.98E-05	12
CTSB.10	3.29E-09	-0.974994748	0.17	0.306	7.56E-05	12
HSD17B11.5	3.59E-09	-0.581539498	0.11	0.256	8.26E-05	12
GRN.11	4.31E-09	-1.037010208	0.13	0.257	9.91E-05	12
FTL.10	6.61E-09	-1.314559218	0.98	0.936	0.00015187	12
CMC1.10	8.98E-09	-1.406042303	0.161	0.288	0.000206375	12
CANX.7	1.76E-08	-0.646267408	0.159	0.303	0.000404967	12
SOD2.9	2.77E-08	-1.210749749	0.161	0.297	0.000635428	12
LITAF.9	4.58E-08	-0.648157091	0.228	0.371	0.001053351	12
AIF1.12	6.08E-08	-1.417577467	0.153	0.262	0.001397945	12
ANXA2.6	8.13E-08	-0.947694884	0.274	0.396	0.001868089	12
LYZ.11	9.89E-08	-2.298613325	0.259	0.353	0.002271846	12
NNMT.12	3.37E-07	-1.930187514	0.187	0.302	0.007743329	12
NDUFA4L2.11	7.44E-07	-2.017554902	0.184	0.291	0.017104376	12
CD99.8	1.02E-06	-0.584861506	0.34	0.473	0.023351891	12
ANXA5.7	1.32E-06	-0.586689779	0.228	0.355	0.030380295	12
SDCBP.9	1.43E-06	-0.657093017	0.259	0.375	0.032953911	12
FCN1.1	0	3.498257796	0.974	0.063	0	13
LST1.4	0	2.927502523	0.997	0.209	0	13
CSTA.3	0	2.449493083	0.913	0.114	0	13
IL1B	0	2.423356907	0.547	0.032	0	13
VCAN	0	2.407132094	0.595	0.048	0	13
S100A12	0	2.293388615	0.489	0.026	0	13
LINC01272	0	2.239033047	0.803	0.044	0	13
MS4A7.2	0	2.180765321	0.896	0.135	0	13
CFP	0	1.846737711	0.744	0.022	0	13
BCL2A1	0	1.822457164	0.767	0.072	0	13
SPI1.2	0	1.718500325	0.867	0.126	0	13
SLC11A1.2	0	1.712078043	0.848	0.115	0	13
LILRB2.2	0	1.70265363	0.657	0.046	0	13
C5AR1.2	0	1.660663381	0.693	0.07	0	13
CLEC12A.2	0	1.636672998	0.715	0.071	0	13
CLEC7A.2	0	1.578710304	0.845	0.119	0	13
NCF2.2	0	1.562423039	0.78	0.071	0	13
LILRA5	0	1.417416358	0.563	0.015	0	13
MPEG1.1	0	1.35086368	0.576	0.04	0	13

APOBEC3A	0	1.253837357	0.44	0.013	0	13
CTB-61M7.2	0	1.089422911	0.359	0.007	0	13
FPR2	0	1.065011761	0.421	0.01	0	13
C19orf38	0	1.053179539	0.447	0.026	0	13
CD300E	0	1.041334547	0.379	0.01	0	13
CYP1B1	0	0.986677983	0.294	0.008	0	13
KCNE1	0	0.874286046	0.35	0.015	0	13
LILRA1	0	0.86517866	0.382	0.012	0	13
CFD.2	2.56E-304	1.945107936	0.841	0.124	5.89E-300	13
EREG	4.89E-301	1.73581645	0.366	0.02	1.12E-296	13
GPBAR1	1.42E-297	0.522190091	0.252	0.008	3.27E-293	13
FPR1.2	4.56E-287	1.544480444	0.683	0.084	1.05E-282	13
PILRA.2	8.44E-286	1.378139615	0.673	0.082	1.94E-281	13
HCK.2	2.31E-281	1.290739161	0.625	0.072	5.30E-277	13
AQP9	6.07E-281	0.732223128	0.291	0.012	1.39E-276	13
FGR.2	5.43E-280	1.476135554	0.735	0.101	1.25E-275	13
KLF4.1	5.58E-279	1.62404246	0.764	0.106	1.28E-274	13
PLAUR.2	1.74E-278	2.026750718	0.754	0.108	3.99E-274	13
CLEC4E.2	4.28E-276	1.990879078	0.715	0.096	9.83E-272	13
RGS18.2	1.84E-260	1.281709162	0.65	0.082	4.22E-256	13
AIF1.13	1.60E-258	2.466580245	0.99	0.243	3.68E-254	13
CPPED1	9.06E-258	1.127576302	0.563	0.061	2.08E-253	13
NAMPT.3	4.47E-257	2.19593772	0.955	0.217	1.03E-252	13
IFI30.2	1.01E-254	1.583943201	0.702	0.098	2.33E-250	13
CYBB.2	1.18E-251	1.402575753	0.751	0.11	2.72E-247	13
SLC7A7.2	6.33E-248	1.389393056	0.657	0.089	1.45E-243	13
IRAK3.2	7.51E-248	1.255861978	0.634	0.083	1.73E-243	13
AP1S2.4	1.86E-241	1.898598541	0.935	0.209	4.26E-237	13
FCER1G.13	1.04E-237	2.460973259	1	0.287	2.38E-233	13
MAFB.2	3.50E-234	1.539726215	0.761	0.118	8.04E-230	13
S100A9.11	8.37E-234	3.181208843	0.951	0.235	1.92E-229	13
SLC31A2.2	1.08E-232	1.418236274	0.65	0.094	2.47E-228	13
CTSS.10	3.17E-230	2.82150287	0.99	0.349	7.29E-226	13
LILRB3.2	3.30E-229	1.106513151	0.557	0.067	7.59E-225	13
VNN1	5.02E-227	1.119773834	0.356	0.026	1.15E-222	13
ADGRE2.2	4.43E-226	0.981973503	0.476	0.049	1.02E-221	13
LYN.2	2.21E-224	1.288315982	0.696	0.109	5.09E-220	13
CLEC4A.2	2.93E-223	0.995670015	0.492	0.054	6.72E-219	13
TKT.3	2.15E-222	1.814645703	0.896	0.213	4.94E-218	13
C1orf162.3	1.94E-220	1.68570979	0.929	0.197	4.45E-216	13
CEACAM4	6.77E-220	0.622434893	0.288	0.017	1.56E-215	13
MARCH1.2	2.52E-219	1.216747138	0.625	0.089	5.80E-215	13
SULT1A1	2.53E-218	0.82110759	0.424	0.039	5.82E-214	13
STX11.1	5.25E-218	1.111547512	0.553	0.068	1.21E-213	13
MTSS1	2.81E-217	1.143204546	0.54	0.065	6.45E-213	13
GCA.2	5.38E-216	1.248783574	0.696	0.114	1.24E-211	13
ABHD5	3.94E-215	1.008831992	0.447	0.044	9.06E-211	13
TREM1	9.04E-215	1.040512361	0.414	0.038	2.08E-210	13
RP6-159A1.4	5.07E-214	0.673862498	0.285	0.017	1.17E-209	13
ARL4A.1	9.65E-213	1.739986296	0.806	0.162	2.22E-208	13
FAM26F.2	9.39E-209	1.617844322	0.718	0.121	2.16E-204	13
LRRC25.2	1.08E-207	1.076413718	0.492	0.059	2.47E-203	13
MNDA.3	1.31E-198	1.536031866	0.728	0.128	3.02E-194	13
KYNU.2	4.62E-195	0.991258486	0.453	0.052	1.06E-190	13
LYZ.12	1.98E-194	3.133548088	0.994	0.337	4.56E-190	13
DUSP6.1	3.16E-194	1.430520482	0.647	0.106	7.26E-190	13
RNF144B.2	4.89E-193	1.254763864	0.524	0.072	1.12E-188	13
RP11-1143G9.4.2	8.27E-193	1.958964106	0.657	0.111	1.90E-188	13

S100A8.3	6.06E-191	2.796781491	0.832	0.197	1.39E-186	13
TYROBP.13	6.22E-190	2.137773948	1	0.374	1.43E-185	13
STXBP2.1	1.54E-188	1.542116406	0.796	0.175	3.53E-184	13
CD55.1	3.02E-183	1.352353856	0.79	0.167	6.95E-179	13
SERPINA1.12	3.98E-183	1.331431485	0.961	0.263	9.14E-179	13
TCF7L2	2.21E-182	0.910312089	0.346	0.031	5.07E-178	13
TNFSF13B.2	4.05E-178	1.365914399	0.657	0.119	9.31E-174	13
ASAH1.6	5.45E-176	1.496180365	0.874	0.226	1.25E-171	13
MXD1	7.13E-175	1.07124521	0.534	0.077	1.64E-170	13
CD68.3	1.14E-172	1.227501657	0.832	0.182	2.63E-168	13
IRS2.2	2.27E-171	1.210671059	0.579	0.096	5.21E-167	13
FAM49A.1	8.71E-169	0.906794132	0.434	0.054	2.00E-164	13
ASGR1	1.77E-168	0.751061635	0.304	0.026	4.07E-164	13
CD302.2	1.92E-168	1.12461056	0.65	0.114	4.41E-164	13
ZEB2.6	1.31E-167	1.479179004	0.867	0.226	3.00E-163	13
LGALS2.2	2.38E-165	1.270911765	0.563	0.09	5.48E-161	13
MTMR11	5.08E-165	0.587946774	0.272	0.021	1.17E-160	13
CD36	2.25E-163	0.677999923	0.401	0.045	5.16E-159	13
POU2F2.2	1.10E-160	1.190428398	0.505	0.078	2.53E-156	13
COTL1.7	1.96E-160	1.890011656	0.99	0.422	4.50E-156	13
RAB31.3	5.57E-159	1.1498402	0.628	0.119	1.28E-154	13
LILRA2.2	9.60E-158	0.7865011	0.366	0.041	2.21E-153	13
FGL2.2	1.80E-157	1.210566873	0.696	0.138	4.14E-153	13
LRRK2.2	1.66E-156	0.818449325	0.45	0.063	3.82E-152	13
ARHGEF40.1	2.22E-155	1.021676299	0.337	0.036	5.09E-151	13
NUDT16.2	7.74E-155	1.269493113	0.686	0.147	1.78E-150	13
PHACTR1.2	2.83E-154	1.126476641	0.489	0.074	6.49E-150	13
THBS1.1	2.30E-152	2.004786657	0.427	0.058	5.28E-148	13
C9orf72.2	2.11E-151	0.824613847	0.434	0.06	4.85E-147	13
NAAA.2	2.64E-151	1.1829942	0.579	0.106	6.07E-147	13
SAT1.10	1.16E-149	2.184771169	1	0.623	2.66E-145	13
RGS2.12	1.59E-149	1.609597637	0.964	0.321	3.66E-145	13
ETS2.3	3.02E-149	1.152481463	0.576	0.108	6.94E-145	13
SCIMP.2	2.94E-142	0.792820411	0.398	0.054	6.76E-138	13
LINC00936.1	9.46E-142	0.976554179	0.515	0.085	2.17E-137	13
CEBPB.10	2.11E-141	1.460266883	0.929	0.315	4.85E-137	13
C10orf54.8	1.35E-140	1.395188578	0.874	0.268	3.11E-136	13
FTH1.12	7.61E-139	1.614811075	1	0.961	1.75E-134	13
PYCARD.7	4.81E-138	1.204334463	0.832	0.234	1.11E-133	13
MTPN.3	1.25E-137	1.18984889	0.783	0.211	2.88E-133	13
LGALS9.2	7.01E-137	0.878008276	0.573	0.11	1.61E-132	13
LY96.2	7.28E-137	0.957474975	0.573	0.111	1.67E-132	13
CD163.2	1.28E-136	1.208167582	0.589	0.115	2.93E-132	13
TYMP.9	1.68E-136	1.368425821	0.893	0.282	3.85E-132	13
ZFAND5.7	2.66E-134	1.724508424	0.867	0.295	6.12E-130	13
FCGR2A.2	3.35E-133	0.822715801	0.65	0.129	7.70E-129	13
BRI3.3	7.84E-132	1.022524601	0.66	0.151	1.80E-127	13
CNIH4	3.84E-131	1.041888724	0.608	0.13	8.83E-127	13
CEBPD.8	4.59E-129	1.326809624	0.883	0.265	1.06E-124	13
RNF130.3	5.77E-129	0.885721809	0.686	0.154	1.32E-124	13
FTL.11	2.18E-128	1.327766701	1	0.936	5.01E-124	13
PSAP.11	2.37E-128	1.450346925	0.984	0.456	5.45E-124	13
FAM110A	3.70E-127	0.800942771	0.32	0.038	8.49E-123	13
H3F3A.7	5.47E-127	1.304181676	0.997	0.733	1.26E-122	13
PRAM1.1	1.17E-126	0.583696126	0.294	0.033	2.69E-122	13
LTA4H.2	1.47E-126	1.019694767	0.638	0.147	3.37E-122	13
CST3.12	5.94E-126	1.296065825	0.99	0.372	1.36E-121	13
MS4A4A.2	8.13E-126	0.76528191	0.476	0.081	1.87E-121	13

PTPRE.1	3.17E-125	0.978429125	0.657	0.15	7.29E-121	13
HBEGF.2	1.28E-124	0.969168955	0.359	0.049	2.95E-120	13
UPP1	1.86E-124	1.03718492	0.654	0.153	4.28E-120	13
GLUL.10	2.78E-124	1.412151354	0.893	0.293	6.40E-120	13
PGD.2	4.73E-124	0.75897795	0.427	0.069	1.09E-119	13
IGSF6.2	1.12E-123	0.883536086	0.495	0.089	2.58E-119	13
AGTRAP	7.53E-123	0.867210509	0.55	0.111	1.73E-118	13
TIMP1.11	8.96E-123	1.507361624	0.893	0.301	2.06E-118	13
NFKBIZ	1.01E-121	1.282473175	0.751	0.204	2.33E-117	13
CD83.1	1.05E-120	0.830537118	0.424	0.067	2.42E-116	13
MT-CO1.6	5.31E-120	0.927222198	0.997	0.924	1.22E-115	13
RIPK2.1	2.04E-118	0.675772215	0.453	0.078	4.70E-114	13
RILPL2.1	2.69E-117	1.000440195	0.722	0.191	6.19E-113	13
NUP214.2	7.99E-117	0.893563856	0.518	0.105	1.84E-112	13
KCTD12.2	2.86E-116	0.86997519	0.599	0.125	6.58E-112	13
SNX10.2	9.13E-115	0.884250581	0.722	0.186	2.10E-110	13
GRINA.2	1.14E-114	1.005587162	0.615	0.15	2.61E-110	13
CSF3R.2	1.20E-114	0.800445851	0.466	0.085	2.75E-110	13
FAM45A	3.63E-114	0.765291122	0.495	0.094	8.34E-110	13
ITGAX.2	3.47E-113	0.696296637	0.375	0.058	7.97E-109	13
ACSL1.2	1.21E-111	0.933589181	0.515	0.107	2.78E-107	13
WARS.1	2.17E-111	0.887278615	0.447	0.082	4.99E-107	13
S100A6.6	2.72E-111	1.322641831	0.994	0.761	6.24E-107	13
CSGALNACT2	3.67E-111	0.801148343	0.472	0.09	8.44E-107	13
CMTM6.2	1.09E-110	1.002761924	0.744	0.214	2.51E-106	13
NEAT1.8	1.55E-110	1.285435397	0.99	0.748	3.56E-106	13
OAZ1.8	1.23E-109	1.138386749	1	0.73	2.83E-105	13
ZNF281	4.11E-109	0.803719769	0.466	0.088	9.44E-105	13
TLR2.2	1.08E-107	0.834956753	0.485	0.097	2.49E-103	13
NLRP3.2	4.13E-107	0.814251945	0.398	0.068	9.49E-103	13
FGD2.2	1.16E-106	0.669654453	0.356	0.056	2.67E-102	13
MAPKAPK3	3.49E-105	0.717605891	0.424	0.077	8.02E-101	13
CPVL.2	6.03E-105	0.846851582	0.54	0.116	1.39E-100	13
CD300A.2	8.54E-105	0.791939532	0.485	0.1	1.96E-100	13
ATP6V1B2.2	1.01E-104	0.829365172	0.566	0.132	2.33E-100	13
SERPINB1.4	3.96E-104	1.109217565	0.796	0.257	9.10E-100	13
S100A4.11	1.57E-103	1.40869101	0.997	0.693	3.62E-99	13
ATG3.2	2.31E-103	0.791898922	0.615	0.156	5.31E-99	13
RNF149.9	5.77E-103	1.053924844	0.79	0.258	1.33E-98	13
RNASE6.2	1.00E-102	1.10745798	0.586	0.139	2.30E-98	13
SMAP2.9	1.54E-102	1.175264266	0.828	0.286	3.53E-98	13
TRIB1.1	2.56E-102	0.615495603	0.32	0.046	5.88E-98	13
PRELID1.4	3.10E-102	1.005261208	0.883	0.344	7.13E-98	13
TNFAIP2.2	4.60E-102	0.736383522	0.356	0.058	1.06E-97	13
RASGEF1B.1	8.91E-102	0.656604732	0.44	0.081	2.05E-97	13
NPC2.10	9.22E-102	1.033900201	0.942	0.366	2.12E-97	13
MEGF9	9.83E-102	0.617390258	0.333	0.051	2.26E-97	13
CASP1.2	5.95E-100	0.996752565	0.673	0.193	1.37E-95	13
HLA-DRB5.11	6.48E-100	0.926370114	0.935	0.361	1.49E-95	13
THEMIS2.2	2.93E-98	0.689915856	0.502	0.11	6.74E-94	13
IL1R2	2.31E-97	1.00934704	0.272	0.035	5.30E-93	13
LILRB1.2	8.09E-97	0.749948291	0.34	0.056	1.86E-92	13
PTEN.2	1.35E-96	0.7788255	0.573	0.141	3.09E-92	13
CALM2.3	1.88E-96	1.305497424	0.971	0.623	4.33E-92	13
DDX21.2	5.31E-96	0.892851018	0.751	0.231	1.22E-91	13
CARD16.7	8.23E-96	1.007167746	0.841	0.305	1.89E-91	13
CD14.3	1.34E-94	1.310225019	0.547	0.133	3.08E-90	13
EVI2B.7	2.37E-94	1.081263073	0.871	0.323	5.44E-90	13

TLR4.1	1.55E-93	0.549635167	0.265	0.036	3.57E-89	13
CD86.2	2.79E-93	0.745213493	0.43	0.088	6.41E-89	13
HLA-DQA2.3	3.11E-93	0.634835067	0.638	0.157	7.16E-89	13
P2RY13.2	3.44E-93	0.64108293	0.33	0.054	7.91E-89	13
PLBD1.2	1.49E-92	0.868061831	0.421	0.087	3.43E-88	13
S100A11.11	4.72E-92	1.072797902	0.984	0.582	1.09E-87	13
SLC43A2.2	1.25E-91	0.709613076	0.401	0.078	2.88E-87	13
SAMSN1.7	1.80E-91	1.296475652	0.832	0.311	4.15E-87	13
THBD	1.94E-91	0.741287629	0.262	0.035	4.46E-87	13
TBXAS1.2	2.82E-91	0.74884505	0.511	0.118	6.48E-87	13
UBE2R2.2	5.44E-91	0.737799964	0.479	0.109	1.25E-86	13
CTSH.2	7.68E-91	0.769364375	0.631	0.166	1.76E-86	13
FAM65B.2	2.36E-89	0.811567428	0.576	0.142	5.43E-85	13
H2AFY.6	2.67E-89	0.935661286	0.796	0.272	6.13E-85	13
UBE2D1.1	3.19E-89	0.636360913	0.466	0.104	7.33E-85	13
SOD2.10	3.28E-89	0.739709226	0.809	0.282	7.53E-85	13
JAML.2	4.91E-89	0.761222165	0.654	0.182	1.13E-84	13
SRGN.9	9.68E-89	1.249815769	0.997	0.786	2.22E-84	13
BLVRB.1	1.04E-88	0.607807667	0.576	0.151	2.40E-84	13
BCAT1.2	5.85E-88	0.624431397	0.32	0.054	1.34E-83	13
BID.1	9.24E-88	0.758047526	0.476	0.11	2.12E-83	13
MT-CO2.7	9.83E-87	0.798905631	0.997	0.872	2.26E-82	13
SMCO4.2	5.25E-86	0.650793911	0.379	0.075	1.21E-81	13
TNFRSF1B.3	4.23E-85	0.822493809	0.706	0.222	9.72E-81	13
CXCL16.2	7.86E-85	0.681976183	0.54	0.131	1.81E-80	13
SERP1.4	1.34E-84	1.040389106	0.903	0.426	3.09E-80	13
ARRB2.2	1.53E-84	0.776816898	0.644	0.187	3.51E-80	13
RNF13.2	1.84E-84	0.798320984	0.66	0.197	4.23E-80	13
CXCL8.2	2.24E-84	0.932155831	0.401	0.083	5.15E-80	13
SCPEP1.2	4.26E-84	0.726365928	0.515	0.128	9.78E-80	13
RHOG.6	1.38E-83	0.887018827	0.741	0.253	3.16E-79	13
MS4A6A.4	3.09E-83	0.580208762	0.68	0.183	7.11E-79	13
CHPT1.2	7.25E-83	0.820916371	0.511	0.131	1.67E-78	13
CXorf21.2	2.50E-82	0.565560015	0.275	0.043	5.74E-78	13
PTPN6.1	4.80E-82	0.837820468	0.576	0.161	1.10E-77	13
TALDO1.4	3.27E-81	0.704978728	0.751	0.252	7.51E-77	13
GAPT.2	1.89E-80	0.721441967	0.353	0.069	4.35E-76	13
SIGLEC10.2	7.63E-80	0.748416767	0.35	0.069	1.75E-75	13
SELL.1	8.49E-80	0.652219141	0.408	0.086	1.95E-75	13
TMEM167A	1.35E-79	0.830421812	0.579	0.17	3.11E-75	13
VAPA.4	1.37E-79	0.907151706	0.861	0.353	3.16E-75	13
PRKCB	1.85E-78	0.622969552	0.424	0.092	4.25E-74	13
AKIRIN2.2	4.11E-78	0.766016391	0.615	0.181	9.46E-74	13
HLA-DRA.13	3.58E-77	0.672758118	0.987	0.666	8.23E-73	13
AZI2.2	4.52E-77	0.684625463	0.489	0.123	1.04E-72	13
ARPC3.6	9.25E-77	0.936787523	0.968	0.596	2.13E-72	13
RHOB.11	1.07E-76	0.95629596	0.854	0.337	2.46E-72	13
VNN2	8.55E-76	0.562944401	0.262	0.041	1.96E-71	13
VMP1.7	4.38E-75	0.843487879	0.799	0.309	1.01E-70	13
PKP2.1	5.41E-75	0.636167585	0.278	0.048	1.24E-70	13
SNX2.2	6.55E-75	0.585063066	0.57	0.165	1.51E-70	13
IER3.2	1.17E-74	0.858153592	0.424	0.102	2.70E-70	13
PELI1.1	1.55E-74	0.678895665	0.44	0.106	3.55E-70	13
EMP3.6	2.38E-74	0.984593264	0.854	0.379	5.47E-70	13
SLC25A37.2	2.49E-74	0.764644811	0.524	0.146	5.71E-70	13
IFNGR2.2	2.56E-74	0.662507193	0.453	0.115	5.88E-70	13
TSPO.7	5.11E-73	0.929782939	0.893	0.41	1.17E-68	13
CD48.11	9.06E-73	0.967690378	0.851	0.364	2.08E-68	13

GRN.12	1.19E-72	0.576840128	0.751	0.243	2.73E-68	13
GDI2.10	1.22E-72	0.729361545	0.783	0.302	2.81E-68	13
LGALS3.5	2.64E-72	0.596572052	0.709	0.235	6.07E-68	13
BTK.2	3.88E-72	0.534940958	0.314	0.061	8.93E-68	13
DAPK1.2	4.03E-72	0.592576517	0.314	0.061	9.27E-68	13
APLP2.11	8.59E-72	0.672832154	0.718	0.245	1.98E-67	13
VAMP5.6	1.24E-71	0.829506745	0.757	0.297	2.84E-67	13
ALOX5.2	1.94E-71	0.567597375	0.401	0.094	4.47E-67	13
NINJ1	3.32E-71	0.565028435	0.45	0.112	7.63E-67	13
TES	8.96E-71	0.763056787	0.531	0.151	2.06E-66	13
SKAP2.2	5.01E-70	0.619463226	0.566	0.169	1.15E-65	13
PGLS.2	7.51E-70	0.673811551	0.676	0.229	1.73E-65	13
CECR1.2	9.77E-70	0.683694522	0.427	0.106	2.25E-65	13
CUX1.1	1.82E-69	0.66697471	0.395	0.094	4.19E-65	13
ANXA5.8	3.03E-69	0.722892336	0.828	0.341	6.96E-65	13
FOS.11	7.01E-69	1.06865849	1	0.674	1.61E-64	13
RPS9.5	1.41E-68	0.670133165	0.99	0.876	3.25E-64	13
CYBA.8	1.89E-68	0.90330598	0.99	0.68	4.34E-64	13
IRF8.2	2.21E-68	0.563871675	0.35	0.076	5.08E-64	13
HSD17B11.6	3.15E-68	0.67763407	0.696	0.242	7.24E-64	13
C3AR1.2	4.66E-68	0.601314015	0.434	0.108	1.07E-63	13
LACTB.2	3.04E-67	0.536456868	0.379	0.089	6.99E-63	13
NBPF14.1	3.10E-67	0.614540095	0.311	0.063	7.12E-63	13
GSTP1.7	7.97E-67	0.769794592	0.945	0.479	1.83E-62	13
GPX1.11	1.45E-66	0.778344977	0.916	0.418	3.32E-62	13
MAP3K8.2	1.85E-66	0.880120186	0.502	0.143	4.24E-62	13
SLC36A4	3.37E-66	0.538739286	0.275	0.05	7.75E-62	13
AREG.2	4.26E-66	1.432352226	0.602	0.2	9.80E-62	13
DRAM2.2	5.10E-66	0.601277487	0.55	0.165	1.17E-61	13
ARPC5.6	8.79E-66	0.797605507	0.89	0.425	2.02E-61	13
MGAT1.3	5.83E-65	0.628199179	0.592	0.192	1.34E-60	13
ATP6V0D1.2	9.36E-65	0.597573573	0.55	0.168	2.15E-60	13
IFNGR1.10	2.78E-64	0.552951377	0.731	0.248	6.39E-60	13
VPS35.2	6.99E-64	0.570155248	0.56	0.174	1.61E-59	13
SPG21.2	9.59E-64	0.636941456	0.524	0.158	2.20E-59	13
PLEK.3	2.45E-63	0.639744795	0.505	0.144	5.62E-59	13
KLF2.3	2.49E-63	0.784019788	0.612	0.2	5.71E-59	13
FCGR3A.5	1.09E-62	1.344232776	0.615	0.225	2.50E-58	13
PABPC1.7	1.23E-62	0.854389027	0.981	0.655	2.83E-58	13
VASP.1	7.84E-62	0.655319354	0.628	0.217	1.80E-57	13
GNAQ.3	2.86E-61	0.629090379	0.456	0.129	6.57E-57	13
MBD2	1.12E-60	0.517438221	0.33	0.074	2.56E-56	13
HSBP1.4	1.43E-60	0.509055916	0.647	0.23	3.28E-56	13
FBP1.2	1.50E-60	0.528977673	0.34	0.079	3.44E-56	13
SAP30.2	1.52E-60	0.653160019	0.398	0.105	3.49E-56	13
GNAI2.8	1.72E-60	0.695211662	0.689	0.25	3.96E-56	13
RAB10.1	4.54E-60	0.535167475	0.537	0.169	1.04E-55	13
STK17B.9	9.91E-60	0.759237679	0.877	0.39	2.28E-55	13
TCIRG1	4.23E-59	0.531445097	0.447	0.124	9.71E-55	13
MT-CO3.7	4.32E-59	0.644909755	0.994	0.877	9.92E-55	13
ATP6V0B.7	4.74E-59	0.697009269	0.735	0.286	1.09E-54	13
LY86.2	1.38E-58	0.576240704	0.411	0.107	3.16E-54	13
CCDC88A.2	1.77E-58	0.683270969	0.379	0.099	4.06E-54	13
SNX3.9	1.92E-58	0.517171062	0.858	0.373	4.41E-54	13
GLIPR1.9	6.26E-58	0.685382163	0.748	0.289	1.44E-53	13
HMOX1.2	1.05E-57	0.509856034	0.401	0.108	2.41E-53	13
NUMB.2	1.42E-57	0.567512886	0.44	0.127	3.27E-53	13
WDR26	1.69E-57	0.529444724	0.356	0.087	3.87E-53	13

RTN4.10	2.14E-57	0.558313994	0.816	0.34	4.93E-53	13
MFSD1.2	2.44E-57	0.552125489	0.476	0.139	5.60E-53	13
ARPC1B.5	2.45E-57	0.772306065	0.932	0.515	5.62E-53	13
METTL7A.2	3.95E-57	0.54511678	0.395	0.108	9.09E-53	13
ZNF706.3	7.55E-57	0.64666873	0.686	0.267	1.74E-52	13
SNX18	1.27E-56	0.51661429	0.288	0.061	2.92E-52	13
BACH1.1	1.87E-56	0.570050509	0.343	0.085	4.29E-52	13
SDCBP.10	2.27E-56	0.646006338	0.832	0.362	5.22E-52	13
SRGAP2B.2	2.70E-56	0.502370303	0.408	0.111	6.21E-52	13
CNPY3.2	8.50E-56	0.57802626	0.608	0.209	1.95E-51	13
ATP1B3.10	9.67E-56	0.764540702	0.663	0.26	2.22E-51	13
DICER1.2	4.29E-55	0.631373742	0.392	0.108	9.85E-51	13
CAPNS1.1	1.06E-54	0.549939083	0.492	0.156	2.44E-50	13
ACTB.8	1.24E-54	0.674987579	1	0.927	2.86E-50	13
CTSB.11	1.27E-54	0.548921707	0.767	0.292	2.91E-50	13
CHP1	2.03E-54	0.521056622	0.375	0.1	4.66E-50	13
LIMS1.9	2.06E-54	0.609019249	0.654	0.24	4.73E-50	13
HN1.3	3.46E-54	0.747196533	0.621	0.231	7.96E-50	13
FKBP5.8	4.12E-54	0.691428759	0.864	0.385	9.46E-50	13
SELPLG.1	4.52E-54	0.537283947	0.482	0.145	1.04E-49	13
MPHOSPH6	6.49E-54	0.54693784	0.298	0.068	1.49E-49	13
PAG1.1	6.72E-54	0.785494413	0.544	0.189	1.54E-49	13
C7orf73.2	7.81E-54	0.504515639	0.644	0.238	1.80E-49	13
ACTR2.6	1.18E-53	0.645373409	0.819	0.357	2.72E-49	13
PABPC4.2	3.17E-53	0.602980987	0.563	0.193	7.29E-49	13
ZBTB16.2	2.00E-52	0.595018653	0.592	0.212	4.60E-48	13
HACD4.2	2.35E-52	0.530706054	0.401	0.113	5.39E-48	13
RHOA.8	4.38E-52	0.668914913	0.922	0.499	1.01E-47	13
SLC25A6.5	4.52E-52	0.713367626	0.971	0.648	1.04E-47	13
XRN2	9.13E-52	0.506740417	0.605	0.219	2.10E-47	13
DUSP1.8	1.57E-51	0.763209302	0.997	0.789	3.61E-47	13
C4orf48.2	2.60E-51	0.528396167	0.553	0.19	5.97E-47	13
C14orf2.3	5.05E-51	0.644350558	0.929	0.506	1.16E-46	13
RGS19.2	6.06E-51	0.522969788	0.453	0.141	1.39E-46	13
KIAA1033.3	7.55E-51	0.502525364	0.421	0.126	1.74E-46	13
IFITM2.9	7.60E-51	0.782199837	0.858	0.467	1.75E-46	13
SH3BGRL.7	1.14E-50	0.652044386	0.838	0.38	2.63E-46	13
PCBP1.2	1.75E-50	0.684232226	0.841	0.41	4.01E-46	13
FGD4.2	2.86E-50	0.553374477	0.314	0.08	6.58E-46	13
ARPC2.7	3.58E-50	0.716470712	0.974	0.63	8.23E-46	13
PLAC8.1	4.12E-50	0.558675728	0.44	0.129	9.46E-46	13
GSTO1.6	4.13E-50	0.543679786	0.735	0.314	9.50E-46	13
ZCCHC6.1	4.60E-50	0.559685576	0.398	0.116	1.06E-45	13
SH3BGRL3.9	5.63E-50	0.744173076	0.977	0.675	1.29E-45	13
GMFG.6	7.86E-50	0.710532821	0.935	0.547	1.81E-45	13
FNIP2.2	1.68E-49	0.503197812	0.294	0.072	3.85E-45	13
LGALS1.5	5.53E-49	0.59352794	0.951	0.564	1.27E-44	13
GNG5.7	6.52E-49	0.518190489	0.777	0.342	1.50E-44	13
RAP1A.6	1.29E-48	0.588044192	0.767	0.341	2.96E-44	13
OAS1.1	1.34E-48	0.506340534	0.282	0.067	3.07E-44	13
WAC-AS1	1.38E-48	0.524343764	0.362	0.101	3.17E-44	13
AHR.1	2.68E-48	0.553920823	0.307	0.077	6.15E-44	13
SOCS3.2	4.81E-48	0.544329898	0.476	0.154	1.11E-43	13
LAMTOR4.2	4.98E-48	0.557284558	0.864	0.408	1.14E-43	13
LRRFIP1.4	5.66E-48	0.690378646	0.822	0.403	1.30E-43	13
HCLS1.7	5.68E-48	0.569562194	0.761	0.312	1.31E-43	13
YBX1.6	1.70E-47	0.661228684	0.955	0.664	3.91E-43	13
AP2S1.10	2.25E-47	0.521668989	0.718	0.301	5.16E-43	13

NR4A1.3	2.93E-47	0.516694542	0.382	0.109	6.73E-43	13
ANXA2.7	1.00E-46	0.533036962	0.796	0.384	2.30E-42	13
PFDN5.4	2.48E-46	0.553191102	0.994	0.779	5.71E-42	13
EIF1.3	3.53E-46	0.544173242	0.997	0.894	8.11E-42	13
MYO1G.4	4.06E-46	0.516518804	0.489	0.161	9.33E-42	13
BASP1.2	1.69E-45	0.520428186	0.272	0.066	3.87E-41	13
NAP1L1.4	2.19E-45	0.936748515	0.9	0.557	5.02E-41	13
MCL1.7	7.72E-45	0.681391553	0.906	0.506	1.77E-40	13
GIMAP2	1.01E-44	0.548460232	0.479	0.164	2.32E-40	13
ABRACL.5	1.03E-44	0.508044033	0.68	0.277	2.36E-40	13
ABI3.4	2.08E-44	0.584541739	0.479	0.169	4.78E-40	13
BLOC1S1.5	6.44E-44	0.558128698	0.744	0.337	1.48E-39	13
SLC8A1.2	7.60E-44	0.517039845	0.301	0.08	1.75E-39	13
MT-ND4.7	2.13E-43	0.532427167	0.997	0.871	4.90E-39	13
IL32.12	2.15E-42	-2.735836882	0.291	0.584	4.94E-38	13
RGS1.13	2.74E-42	-2.573400048	0.566	0.7	6.30E-38	13
HLA-A.9	4.56E-42	-0.94289362	0.987	0.892	1.05E-37	13
MT-ATP6.7	1.11E-41	0.530622283	0.997	0.856	2.56E-37	13
CHMP1B.3	2.12E-41	0.547101143	0.599	0.239	4.88E-37	13
SLC25A5.5	1.52E-40	0.574574294	0.786	0.377	3.50E-36	13
CD37.9	2.13E-40	0.607381642	0.864	0.454	4.89E-36	13
NACA.5	1.31E-38	0.517807738	0.99	0.799	3.01E-34	13
VAMP8.7	2.26E-38	0.560193333	0.845	0.452	5.19E-34	13
EVI2A.9	2.86E-37	0.556580073	0.628	0.272	6.58E-33	13
CD3D.13	5.55E-37	-2.312343266	0.155	0.482	1.27E-32	13
ITGB2.10	3.28E-36	0.566848689	0.835	0.405	7.54E-32	13
CTSC.10	4.34E-36	0.599783858	0.699	0.339	9.97E-32	13
RPL31.9	8.22E-36	-0.72846868	0.994	0.905	1.89E-31	13
SLC35E3.2	1.32E-35	0.512713159	0.333	0.108	3.03E-31	13
CD52.10	5.09E-35	0.671306784	0.919	0.54	1.17E-30	13
LAPTM5.10	7.08E-35	0.545551444	0.958	0.558	1.63E-30	13
LCP1.10	9.08E-35	0.503531007	0.799	0.395	2.09E-30	13
CCNI.2	2.29E-34	0.531105419	0.939	0.585	5.26E-30	13
CD2.12	3.41E-34	-2.268132778	0.155	0.464	7.83E-30	13
FYB.11	1.59E-33	0.511088332	0.883	0.45	3.65E-29	13
CYTIP.11	2.32E-33	0.519592657	0.861	0.467	5.34E-29	13
MT-ND1.7	4.09E-33	0.525464572	0.974	0.783	9.41E-29	13
RPL23A.9	9.99E-33	-0.580909993	0.994	0.929	2.30E-28	13
CD3E.12	3.27E-32	-1.782435472	0.087	0.405	7.51E-28	13
GZMA.12	3.48E-31	-2.41912553	0.126	0.426	7.99E-27	13
CCL5.12	2.55E-30	-3.197453265	0.375	0.566	5.86E-26	13
TRAC.13	5.30E-30	-2.226375869	0.152	0.438	1.22E-25	13
CST7.11	9.84E-28	-2.101290552	0.113	0.395	2.26E-23	13
TRBC2.12	1.61E-26	-1.94702921	0.11	0.383	3.70E-22	13
RPS29.9	2.68E-26	-0.63618606	1	0.919	6.16E-22	13
RPS3.8	2.72E-25	-0.519709598	0.994	0.917	6.25E-21	13
SYNE2.12	5.52E-25	-1.458518811	0.068	0.338	1.27E-20	13
RPL3.7	1.72E-24	-0.542757953	0.997	0.931	3.94E-20	13
CD69.12	1.75E-24	-2.359015956	0.35	0.533	4.03E-20	13
CD3G.12	3.34E-24	-1.534840177	0.039	0.299	7.68E-20	13
ETS1.11	7.52E-23	-1.407614484	0.094	0.344	1.73E-18	13
IGHA1	5.64E-22	-1.738481026	0.288	0.101	1.30E-17	13
LCK.13	1.49E-21	-1.254154742	0.052	0.293	3.42E-17	13
ITM2A.12	4.21E-21	-1.634480269	0.081	0.316	9.67E-17	13
TRBC1.12	6.14E-21	-2.138830332	0.129	0.358	1.41E-16	13
MALAT1.12	8.39E-21	-0.585427441	1	0.982	1.93E-16	13
CLEC2D.12	3.52E-20	-1.379402628	0.081	0.311	8.10E-16	13
HSPA1A.4	1.80E-19	0.605474968	0.45	0.229	4.14E-15	13

CD7.10	1.64E-18	-1.555329893	0.091	0.305	3.78E-14	13
RPS26.9	1.96E-18	-0.572516706	0.984	0.868	4.51E-14	13
ACAP1.13	3.22E-18	-1.283882615	0.129	0.338	7.40E-14	13
STK17A.11	1.01E-17	-1.311069635	0.168	0.371	2.31E-13	13
GZMK.13	1.02E-17	-2.603932492	0.178	0.37	2.35E-13	13
OCIAD2.9	4.58E-16	-1.17146835	0.071	0.265	1.05E-11	13
PIK3IP1.12	9.54E-16	-1.227452067	0.175	0.362	2.19E-11	13
RPSA.7	1.23E-15	-0.649811695	0.916	0.801	2.82E-11	13
CD96.13	2.59E-15	-1.116401996	0.068	0.253	5.95E-11	13
CD27.12	6.97E-15	-1.500821126	0.091	0.271	1.60E-10	13
RPLP0.8	1.42E-14	-0.762275387	0.951	0.84	3.27E-10	13
DUSP2.12	1.81E-14	-1.78378663	0.294	0.434	4.17E-10	13
PRDM1.8	3.29E-14	-1.224479429	0.113	0.296	7.57E-10	13
CCL4.13	1.83E-13	-2.456586977	0.317	0.443	4.20E-09	13
IL7R.11	5.09E-13	-2.050430197	0.146	0.307	1.17E-08	13
HMGB1.7	3.44E-12	-0.814369609	0.88	0.731	7.91E-08	13
IGKC.9	9.19E-10	-3.07772941	0.518	0.291	2.11E-05	13
PRKCH.8	1.41E-09	-0.855080944	0.123	0.256	3.24E-05	13
C12orf57.7	5.22E-09	-1.084059511	0.272	0.374	0.000119896	13
PIK3R1.10	9.32E-09	-0.983582764	0.191	0.311	0.000214293	13
EVL.12	1.06E-08	-1.034957391	0.45	0.496	0.000243198	13
GYPC.8	1.21E-08	-0.935457619	0.191	0.309	0.000279024	13
SYTL3.11	1.46E-08	-1.048925492	0.165	0.28	0.00033458	13
AAK1.12	2.10E-08	-0.891741358	0.272	0.373	0.000483279	13
CD99.9	2.24E-08	-0.859065048	0.395	0.472	0.000513777	13
C1QA.12	3.12E-08	-0.589402051	0.434	0.246	0.000715958	13
ODF2L.7	4.05E-08	-0.958733592	0.149	0.262	0.000930673	13
KIAA1551.9	5.14E-08	-0.950445365	0.233	0.338	0.001181076	13
RARRES3.10	7.52E-08	-1.020028327	0.343	0.412	0.001727053	13
IL2RG.13	8.07E-08	-1.007520428	0.298	0.374	0.001854171	13
JUN.8	2.59E-07	-0.954652804	0.757	0.69	0.005945033	13
SRSF7.10	3.23E-07	-1.122984237	0.579	0.544	0.007418276	13
HBB.7	3.33E-07	-2.45547009	0.621	0.386	0.0076511	13
LDHB.8	3.47E-07	-0.920412788	0.424	0.467	0.007963108	13
APOBEC3G.10	4.93E-07	-0.948748989	0.172	0.274	0.011318511	13
NKG7.13	5.45E-07	-2.280258653	0.405	0.428	0.012535979	13
RNF19A.10	1.29E-06	-0.904110666	0.184	0.279	0.029586166	13
CMC1.11	1.63E-06	-1.632830813	0.197	0.287	0.037444488	13
RGCC.11	1.71E-06	-1.459794974	0.223	0.309	0.039186651	13
EMB.9	2.16E-06	-0.729031364	0.175	0.271	0.049639028	13
HILPDA.1	0	2.86343331	0.884	0.113	0	14
EGLN3.1	0	2.143252343	0.903	0.054	0	14
BNIP3.2	0	2.103455248	0.911	0.119	0	14
LINC01320	0	2.053848368	0.873	0.046	0	14
CCND1.1	0	2.044843944	0.784	0.08	0	14
RARRES2.2	0	2.016037805	0.931	0.096	0	14
VEGFA	0	1.967768878	0.865	0.076	0	14
ERRFI1	0	1.902761882	0.73	0.053	0	14
ADM.1	0	1.901728311	0.861	0.104	0	14
CA9	0	1.878994603	0.83	0.033	0	14
NUPR1.3	0	1.847081996	0.919	0.119	0	14
NOL3	0	1.810829749	0.861	0.061	0	14
MGST1.2	0	1.695323495	0.915	0.097	0	14
CP	0	1.675982293	0.529	0.036	0	14
PLOD2	0	1.673394428	0.772	0.032	0	14
LOX	0	1.634367845	0.653	0.032	0	14
CA12	0	1.470927144	0.683	0.027	0	14
CMBL.1	0	1.431566491	0.741	0.047	0	14

KRT8.1	0	1.357908048	0.819	0.085	0	14
EDN1	0	1.307252075	0.417	0.02	0	14
TFPI.1	0	1.292309906	0.699	0.061	0	14
IRX3	0	1.276632413	0.571	0.021	0	14
NPTX2	0	1.247512669	0.517	0.021	0	14
TCEA3	0	1.244912671	0.745	0.044	0	14
AK4	0	1.194856913	0.687	0.025	0	14
ADSSL1	0	1.107216223	0.517	0.024	0	14
P3H2	0	1.073118894	0.637	0.04	0	14
MAOB	0	1.061706281	0.622	0.026	0	14
BHMT.1	0	1.057620525	0.625	0.03	0	14
FSTL3	0	1.057136149	0.471	0.027	0	14
EMX2	0	1.045206103	0.544	0.023	0	14
UGT2B7	0	1.007711538	0.571	0.032	0	14
ANG	0	0.934703068	0.56	0.038	0	14
CDK18	0	0.909010029	0.533	0.026	0	14
ENPP3	0	0.904513111	0.514	0.017	0	14
FBXO17	0	0.90363076	0.521	0.021	0	14
RP11-14N7.2	0	0.878175488	0.579	0.037	0	14
BICC1	0	0.876767547	0.448	0.017	0	14
RP1-60O19.1	0	0.816842725	0.533	0.023	0	14
KISS1R	0	0.815993385	0.409	0.016	0	14
ZNF395	0	0.791902254	0.514	0.023	0	14
RP11-11N9.4	0	0.787058631	0.494	0.031	0	14
PCOLCE2	0	0.778442191	0.344	0.007	0	14
FABP6	0	0.771348422	0.544	0.033	0	14
P4HA2	0	0.770155696	0.456	0.022	0	14
CDCA2	0	0.730704211	0.429	0.021	0	14
EDIL3	0	0.711994	0.432	0.018	0	14
MAPT	0	0.707657452	0.351	0.006	0	14
SPINK13	0	0.696883203	0.309	0.009	0	14
GALNT14	0	0.693681833	0.432	0.017	0	14
CDH2	0	0.6930213	0.448	0.01	0	14
STC2	0	0.691850616	0.32	0.008	0	14
BACE2	0	0.649101251	0.386	0.018	0	14
ARSE	0	0.634255192	0.417	0.013	0	14
BHMT2	0	0.624589678	0.432	0.019	0	14
SLC22A2	0	0.623916444	0.398	0.018	0	14
FMO2	0	0.61321035	0.263	0.004	0	14
SPAG4	0	0.545613606	0.413	0.021	0	14
RASSF6	0	0.545317822	0.29	0.006	0	14
RP11-798K3.2	0	0.532876038	0.34	0.012	0	14
CLEC18A	0	0.514631417	0.34	0.012	0	14
SLC2A1	6.43E-304	0.960122601	0.525	0.036	1.48E-299	14
SOX4	6.61E-302	2.136740356	0.784	0.09	1.52E-297	14
ANGPTL4.2	1.16E-300	2.913743924	0.961	0.152	2.67E-296	14
CXCL14.1	1.41E-300	1.22386362	0.637	0.054	3.24E-296	14
CD24.3	9.58E-299	2.810169848	0.992	0.162	2.20E-294	14
SLC3A1	1.45E-298	0.93801204	0.49	0.031	3.33E-294	14
FAM13A	1.10E-296	1.0766083	0.653	0.06	2.52E-292	14
ASS1	9.48E-295	0.639987746	0.386	0.019	2.18E-290	14
PROS1.1	1.02E-294	0.766193634	0.552	0.041	2.33E-290	14
CKB	5.24E-294	0.960841747	0.533	0.039	1.20E-289	14
NR1H4	1.41E-290	0.561297946	0.344	0.015	3.23E-286	14
RBPMS.1	3.85E-290	1.047778491	0.629	0.055	8.85E-286	14
HSF4	7.04E-287	0.53067326	0.359	0.017	1.62E-282	14
MT3	1.85E-284	1.80612603	0.51	0.037	4.26E-280	14
C14orf105	4.85E-284	0.520440631	0.305	0.012	1.11E-279	14

TMEM91	3.69E-274	1.058527014	0.614	0.056	8.48E-270	14
CRNDE	4.26E-274	0.610358369	0.402	0.023	9.79E-270	14
KRT18.1	7.32E-269	1.29039571	0.784	0.093	1.68E-264	14
ENPEP	4.76E-261	0.702104945	0.44	0.029	1.09E-256	14
SLC39A14	1.03E-258	0.719060306	0.444	0.03	2.37E-254	14
BHLHE41.2	3.25E-258	1.137931375	0.649	0.069	7.47E-254	14
SEZ6L2	4.17E-257	0.521101191	0.263	0.009	9.59E-253	14
SEPP1.2	1.04E-256	1.194869549	0.861	0.117	2.38E-252	14
ALDOC	4.72E-256	0.875396578	0.552	0.047	1.08E-251	14
PFKP	5.66E-256	1.279433065	0.788	0.1	1.30E-251	14
DPCD	1.02E-255	0.715095036	0.483	0.036	2.34E-251	14
GATM.1	5.79E-254	1.041890952	0.618	0.062	1.33E-249	14
TMEM176A.2	1.45E-253	1.619849577	0.873	0.133	3.33E-249	14
SSPN.1	2.58E-251	0.765510582	0.486	0.038	5.94E-247	14
SLC37A4	1.49E-247	0.845746953	0.537	0.047	3.43E-243	14
FOLR1	7.08E-246	0.610731486	0.34	0.018	1.63E-241	14
AEBP1	1.48E-245	0.738679948	0.386	0.024	3.40E-241	14
MGLL.1	1.35E-240	0.8521163	0.61	0.062	3.11E-236	14
PHLDA3	2.41E-240	0.65769178	0.382	0.024	5.54E-236	14
P4HA1	3.88E-239	0.988560684	0.707	0.086	8.91E-235	14
SDC2	6.42E-238	0.596959983	0.486	0.039	1.48E-233	14
FHL1	1.32E-236	0.95792889	0.517	0.045	3.02E-232	14
WFDC2	3.89E-235	0.84584096	0.498	0.042	8.93E-231	14
ALDH1A1.1	1.35E-233	1.130443607	0.672	0.08	3.10E-229	14
NMB	1.03E-232	1.823048403	0.506	0.045	2.38E-228	14
EGFR	3.68E-231	0.597348362	0.375	0.024	8.45E-227	14
C1S	3.90E-231	0.884453336	0.479	0.04	8.97E-227	14
ATP1B1.3	7.96E-231	1.410363326	0.826	0.124	1.83E-226	14
EFEMP2	8.60E-231	0.578077902	0.417	0.029	1.98E-226	14
TMEM45A	4.83E-230	0.581944855	0.332	0.018	1.11E-225	14
DIXDC1	1.11E-228	0.545469463	0.363	0.022	2.54E-224	14
CAV1.3	1.89E-228	1.593481981	0.834	0.124	4.34E-224	14
C1orf186	4.94E-228	0.893705667	0.537	0.05	1.13E-223	14
C19orf33	7.55E-228	0.719610447	0.537	0.05	1.73E-223	14
NDRG1.1	9.86E-227	1.620678817	0.819	0.13	2.27E-222	14
SERINC2	1.45E-226	0.67857914	0.425	0.032	3.33E-222	14
INSIG2	9.13E-226	0.917666459	0.653	0.077	2.10E-221	14
PDLIM1.1	2.40E-225	1.084360954	0.788	0.108	5.52E-221	14
CAV2.1	1.32E-224	0.804350651	0.622	0.067	3.04E-220	14
WDR34	7.16E-223	0.675509712	0.421	0.031	1.65E-218	14
C1R	6.34E-222	1.032512273	0.506	0.046	1.46E-217	14
PMP22	6.35E-222	0.942965106	0.541	0.053	1.46E-217	14
HSD3B7	1.70E-221	0.646001072	0.39	0.027	3.90E-217	14
TMEM176B.2	4.30E-221	1.473244563	0.857	0.141	9.89E-217	14
CALD1.1	3.06E-218	0.897549226	0.876	0.135	7.03E-214	14
GPRC5C	1.30E-216	0.58879534	0.378	0.026	2.99E-212	14
S100A16.1	9.77E-216	0.75259085	0.629	0.071	2.25E-211	14
MYL6B	1.87E-214	0.885929899	0.653	0.08	4.29E-210	14
RRAD	1.45E-212	1.032131214	0.32	0.019	3.33E-208	14
MIR4458HG	2.36E-212	0.681506906	0.606	0.068	5.43E-208	14
SNHG19	1.58E-208	0.7996202	0.517	0.051	3.63E-204	14
PDZK1IP1.2	3.98E-207	0.897798247	0.757	0.111	9.14E-203	14
AKR1C3	2.01E-202	0.595254314	0.61	0.073	4.61E-198	14
PRDX4	3.38E-202	0.959067107	0.734	0.11	7.77E-198	14
ENO2	3.32E-201	0.732351196	0.444	0.039	7.64E-197	14
CLU.3	2.04E-200	1.668099164	0.911	0.185	4.69E-196	14
NDUFA4L2.13	4.64E-199	2.609597026	0.992	0.275	1.07E-194	14
CCL28	1.19E-198	0.606532297	0.39	0.03	2.75E-194	14

KCTD3	1.32E-198	0.569920778	0.355	0.025	3.04E-194	14
TMEM37	2.75E-197	0.871015181	0.456	0.042	6.31E-193	14
NT5C3B	8.95E-193	0.692461773	0.517	0.055	2.06E-188	14
PGRMC1	3.33E-192	1.139171957	0.707	0.108	7.64E-188	14
CFAP36	3.43E-192	0.815064013	0.649	0.085	7.88E-188	14
S100A13.2	5.30E-192	0.940114246	0.772	0.121	1.22E-187	14
FAM213A.1	1.01E-190	1.016021433	0.695	0.102	2.31E-186	14
NNMT.14	2.42E-190	2.525163096	1	0.286	5.56E-186	14
CRYAB.13	1.82E-189	2.230678711	0.985	0.245	4.19E-185	14
YBX3.2	4.67E-189	1.569510938	0.853	0.172	1.07E-184	14
EIF4EBP1	7.43E-188	1.483435681	0.819	0.153	1.71E-183	14
PDK4.6	1.05E-187	2.180049016	0.892	0.19	2.42E-183	14
C10orf10.2	1.18E-187	1.563916774	0.695	0.104	2.71E-183	14
FARP1	4.74E-187	0.665520317	0.44	0.042	1.09E-182	14
CYB5A.4	1.08E-186	1.420054225	0.954	0.215	2.48E-182	14
NEK6	1.33E-186	0.663235519	0.44	0.042	3.06E-182	14
TRIP6	3.56E-186	0.647673961	0.467	0.048	8.17E-182	14
PDZK1	7.26E-186	0.587682642	0.336	0.024	1.67E-181	14
EPB41L4A-AS1.2	9.57E-186	1.429190954	0.799	0.147	2.20E-181	14
SMPDL3A	2.36E-183	0.602806719	0.417	0.038	5.42E-179	14
PRKCDBP.1	2.84E-182	0.725649519	0.591	0.074	6.52E-178	14
CNN3.2	1.73E-179	0.584851306	0.691	0.097	3.99E-175	14
SPINT2.2	5.50E-178	1.074275757	0.784	0.138	1.26E-173	14
FAIM	9.25E-178	0.546323964	0.386	0.033	2.13E-173	14
SLC16A3.2	8.51E-175	1.188501603	0.822	0.159	1.96E-170	14
TMEM27	2.42E-174	0.561821324	0.351	0.028	5.57E-170	14
HSBP1L1	2.52E-174	0.593822328	0.463	0.049	5.79E-170	14
CRYZ	9.79E-174	0.571225071	0.502	0.057	2.25E-169	14
HNMT.3	1.26E-172	0.904465112	0.672	0.105	2.90E-168	14
PLEKHA1.1	3.19E-170	0.992381697	0.695	0.11	7.33E-166	14
IMPA2	6.68E-170	0.630544346	0.405	0.039	1.53E-165	14
DOC2A	8.39E-169	0.559191837	0.266	0.016	1.93E-164	14
HPCAL1.3	8.33E-168	1.278511952	0.811	0.16	1.91E-163	14
PMM1	2.82E-167	0.703480717	0.548	0.07	6.47E-163	14
CD151.2	2.71E-166	1.180725928	0.876	0.188	6.22E-162	14
RP11-798M19.6	3.59E-166	0.815126138	0.552	0.073	8.25E-162	14
ADIRF.9	6.18E-165	1.204314573	0.954	0.228	1.42E-160	14
MAP3K13	1.36E-162	0.860476157	0.649	0.103	3.13E-158	14
GPX3.3	6.47E-162	1.381657122	0.78	0.154	1.49E-157	14
PTGR1	1.38E-161	0.668735579	0.444	0.049	3.17E-157	14
TGM2.1	1.44E-161	0.756069226	0.471	0.054	3.32E-157	14
DARS.2	3.74E-160	1.385964038	0.849	0.196	8.59E-156	14
FAM134B	1.79E-159	0.575815439	0.39	0.037	4.11E-155	14
RPF2	6.65E-158	0.757421789	0.529	0.07	1.53E-153	14
GADD45A.1	4.88E-155	0.868799666	0.625	0.096	1.12E-150	14
BLVRB.2	3.53E-154	0.871392634	0.772	0.149	8.11E-150	14
RAB13.3	1.57E-153	0.698741039	0.714	0.126	3.62E-149	14
MT1F	3.59E-151	1.233634074	0.68	0.119	8.24E-147	14
SHMT1	5.94E-151	0.649435827	0.425	0.048	1.37E-146	14
MXI1	1.32E-150	0.707102214	0.49	0.063	3.03E-146	14
UGDH	7.97E-149	0.510211524	0.39	0.04	1.83E-144	14
FAHD1	2.88E-148	0.540709754	0.421	0.047	6.61E-144	14
MT1X.9	2.82E-147	3.551826191	0.869	0.272	6.48E-143	14
TSPAN12.1	3.83E-145	0.57188743	0.363	0.036	8.81E-141	14
GATM-AS1	3.15E-143	0.649517036	0.259	0.018	7.24E-139	14
SCCPDH	6.39E-143	0.592661455	0.506	0.07	1.47E-138	14
RASSF4.2	6.40E-142	1.096191871	0.753	0.153	1.47E-137	14
BNIP3L.2	1.24E-141	1.307699619	0.853	0.22	2.85E-137	14

KIF1BP	2.42E-139	0.548666663	0.378	0.041	5.55E-135	14
CCNB1IP1	3.82E-139	0.668675693	0.525	0.076	8.78E-135	14
DEFB1.1	1.13E-137	0.778876374	0.533	0.081	2.59E-133	14
SMIM3	1.54E-135	0.923557997	0.544	0.086	3.53E-131	14
TMSB4X.6	1.97E-135	-3.165445068	0.988	0.976	4.52E-131	14
SLC25A37.3	9.85E-135	1.038343778	0.71	0.144	2.26E-130	14
CES4A	2.42E-134	0.544326976	0.324	0.031	5.55E-130	14
VKORC1.1	4.80E-133	1.020643399	0.788	0.184	1.10E-128	14
SPP1.6	2.95E-132	0.880074317	0.857	0.204	6.78E-128	14
POLD2	9.29E-132	0.65275554	0.475	0.067	2.13E-127	14
ANXA4.3	1.26E-130	1.170341258	0.703	0.148	2.89E-126	14
PLIN2.10	1.46E-130	1.825582405	0.946	0.324	3.36E-126	14
TPM1.2	8.65E-130	0.802131662	0.807	0.183	1.99E-125	14
C5orf46	3.25E-129	0.929163992	0.355	0.039	7.47E-125	14
B2M.10	7.61E-129	-2.255765185	1	0.988	1.75E-124	14
SPATS2L	5.49E-128	0.83256172	0.625	0.115	1.26E-123	14
PPP1R16A	2.44E-126	0.547567113	0.336	0.035	5.62E-122	14
EGR1.2	9.87E-126	1.30362676	0.656	0.132	2.27E-121	14
SERPING1	1.35E-125	0.725373009	0.579	0.1	3.11E-121	14
KHK	8.01E-125	0.626465212	0.386	0.047	1.84E-120	14
ENO1.11	1.34E-124	2.068714447	0.992	0.586	3.07E-120	14
STC1.1	1.69E-124	0.543177656	0.459	0.063	3.88E-120	14
LDHA.11	2.79E-123	1.746875485	0.992	0.571	6.40E-119	14
BOD1	5.64E-123	0.554871771	0.432	0.059	1.30E-118	14
RCN1.1	1.88E-122	0.688651468	0.521	0.085	4.33E-118	14
PIGP	4.88E-122	0.649988627	0.61	0.113	1.12E-117	14
BEX2	7.35E-122	0.516515753	0.382	0.046	1.69E-117	14
ESD.2	5.00E-121	0.959296121	0.834	0.224	1.15E-116	14
AK2	7.23E-121	0.701883574	0.672	0.136	1.66E-116	14
PLA2G16	9.14E-121	0.94535216	0.753	0.174	2.10E-116	14
IGFBP4.1	3.03E-120	0.63345872	0.61	0.108	6.97E-116	14
C16orf13.2	9.74E-120	0.976623394	0.857	0.227	2.24E-115	14
GCSH	1.14E-119	0.52583499	0.498	0.078	2.63E-115	14
RBP5	4.97E-119	0.530006951	0.417	0.056	1.14E-114	14
MIF.11	7.76E-119	1.582798948	0.977	0.497	1.78E-114	14
FXYD2.1	1.44E-118	0.972499633	0.637	0.124	3.31E-114	14
ABCA1	2.75E-118	0.531383674	0.39	0.051	6.33E-114	14
POLR2H	1.65E-117	0.561808425	0.544	0.093	3.79E-113	14
ALDH3A2	1.77E-117	0.562611831	0.409	0.056	4.08E-113	14
SLC39A1.1	1.51E-116	0.669951056	0.629	0.126	3.46E-112	14
LGALS3.6	5.80E-116	1.229165036	0.842	0.234	1.33E-111	14
RHOB.12	7.02E-116	1.75955145	0.942	0.337	1.61E-111	14
TSC22D1.3	9.89E-116	0.837892229	0.761	0.173	2.27E-111	14
TMEM205	1.12E-114	0.698260326	0.676	0.144	2.57E-110	14
IGFBP3.11	1.44E-114	1.041861751	0.849	0.233	3.31E-110	14
BCKDK	1.64E-114	0.64037554	0.544	0.098	3.78E-110	14
TMCC1	8.68E-114	0.589608492	0.394	0.053	1.99E-109	14
PGRMC2	6.24E-113	0.524668811	0.529	0.09	1.43E-108	14
ERO1A	3.33E-112	0.611051747	0.475	0.076	7.65E-108	14
UBR4	1.13E-110	0.639490274	0.533	0.094	2.59E-106	14
CYR61.1	1.95E-110	0.534702863	0.34	0.04	4.47E-106	14
MRPS18B	7.32E-108	0.705904489	0.641	0.135	1.68E-103	14
ARPC1A.1	1.33E-107	0.654595027	0.618	0.126	3.06E-103	14
RAB34.1	1.76E-107	0.600319965	0.471	0.079	4.04E-103	14
PTTG1IP.2	3.02E-107	0.779899692	0.71	0.167	6.95E-103	14
DAB2.3	1.09E-106	0.930197663	0.68	0.152	2.50E-102	14
RNASET2.13	2.67E-106	1.502968212	0.946	0.35	6.15E-102	14
FAM162A.2	6.14E-106	0.679682096	0.734	0.175	1.41E-101	14

LINC00998	6.61E-106	0.676088366	0.741	0.179	1.52E-101	14
SOCS3.3	9.55E-106	1.236856732	0.656	0.151	2.19E-101	14
MORF4L2.2	2.98E-104	0.615652029	0.726	0.17	6.85E-100	14
CISD1	5.01E-104	0.530102769	0.548	0.104	1.15E-99	14
LRRC41	3.36E-103	0.509374524	0.452	0.073	7.73E-99	14
HCFC1R1.1	3.37E-103	0.641693137	0.587	0.119	7.74E-99	14
CTHRC1	5.17E-103	0.510741136	0.286	0.031	1.19E-98	14
TNIP1.2	7.50E-103	0.738811022	0.668	0.151	1.72E-98	14
WRB	2.21E-102	0.521776287	0.421	0.065	5.07E-98	14
PGK1.9	2.91E-102	1.326958645	0.961	0.441	6.68E-98	14
SOD2.11	8.09E-102	1.695581927	0.846	0.283	1.86E-97	14
ZFAS1.10	3.86E-101	1.508472019	0.954	0.475	8.86E-97	14
CDKN1A.1	2.35E-99	0.635355116	0.56	0.112	5.40E-95	14
ACTN1	3.11E-99	0.588633624	0.444	0.075	7.15E-95	14
PCBD1	3.74E-99	0.584904963	0.571	0.117	8.60E-95	14
ERGIC1.1	8.89E-99	0.656450009	0.583	0.123	2.04E-94	14
VIM.11	1.91E-98	1.509375901	1	0.767	4.39E-94	14
ALDOA.5	3.81E-97	1.312963646	0.996	0.63	8.76E-93	14
GAPDH.8	8.78E-97	1.26673523	1	0.863	2.02E-92	14
CERS2	9.30E-97	0.594748732	0.517	0.098	2.14E-92	14
CRYL1	1.25E-95	0.582705787	0.483	0.09	2.88E-91	14
POLR2I.2	1.53E-95	0.695694128	0.764	0.203	3.52E-91	14
C5orf15	1.90E-95	0.563736785	0.49	0.092	4.37E-91	14
RNF187	5.33E-95	0.569025521	0.541	0.106	1.23E-90	14
TPI1.8	8.83E-95	1.398129868	0.992	0.556	2.03E-90	14
WDR60.1	1.22E-94	0.62688911	0.514	0.098	2.81E-90	14
NFIA.1	1.31E-94	0.585697535	0.444	0.077	3.02E-90	14
HLA-B.11	1.22E-93	-1.954447254	0.942	0.914	2.79E-89	14
RPLP0.9	9.17E-93	1.241009754	0.996	0.84	2.11E-88	14
NDUFC1.9	9.73E-93	0.782997365	0.83	0.26	2.24E-88	14
VDAC1.5	1.04E-92	0.923085435	0.83	0.267	2.39E-88	14
PKM.7	1.15E-92	1.32419728	0.95	0.424	2.63E-88	14
SERPINE2	1.38E-92	0.534090223	0.297	0.037	3.17E-88	14
LAPTM4A.9	9.16E-92	0.932105019	0.938	0.341	2.10E-87	14
SNHG8.5	5.93E-91	1.271041922	0.927	0.425	1.36E-86	14
RBM47.3	6.14E-91	0.501866586	0.421	0.074	1.41E-86	14
ARL1	4.61E-90	0.538427096	0.506	0.099	1.06E-85	14
NFIC.1	3.59E-89	0.587889355	0.533	0.112	8.25E-85	14
KDSR.1	7.89E-89	0.547623362	0.49	0.097	1.81E-84	14
CYB5R3.3	9.67E-89	0.654020325	0.683	0.177	2.22E-84	14
RPS5.7	2.64E-88	1.131162939	1	0.838	6.06E-84	14
AIG1	4.61E-88	0.534751667	0.448	0.084	1.06E-83	14
TIPIN	1.22E-87	0.684395749	0.595	0.137	2.81E-83	14
FLOT1.1	6.94E-87	0.536595209	0.672	0.17	1.59E-82	14
MT2A.14	1.04E-86	2.839795017	0.95	0.654	2.38E-82	14
PEBP1.10	1.68E-86	1.118665934	0.923	0.38	3.87E-82	14
ODF3B.1	6.10E-86	0.558757959	0.479	0.097	1.40E-81	14
SNX10.3	9.73E-86	0.684730589	0.71	0.188	2.24E-81	14
MT1E.3	1.23E-85	2.595226154	0.598	0.165	2.82E-81	14
IFITM3.12	2.35E-85	0.765349589	0.954	0.367	5.40E-81	14
NDUFS4	4.18E-85	0.683815349	0.718	0.197	9.61E-81	14
SYPL1.1	7.51E-85	0.573823535	0.691	0.18	1.73E-80	14
APEX1.2	1.30E-84	0.674879205	0.718	0.196	2.98E-80	14
NDUFB4.9	1.40E-84	0.919121522	0.934	0.401	3.21E-80	14
MRPS33	1.48E-84	0.508661281	0.595	0.138	3.41E-80	14
EBPL	4.94E-84	0.504100279	0.459	0.089	1.14E-79	14
MGST3.10	3.54E-83	0.786275085	0.873	0.296	8.14E-79	14
SELM.2	1.41E-82	0.616913437	0.68	0.175	3.24E-78	14

CNDP2.2	1.46E-82	0.739926988	0.722	0.203	3.35E-78	14
HES1.2	9.09E-82	0.581452382	0.429	0.078	2.09E-77	14
SEC61G.7	2.64E-81	1.045027232	0.931	0.428	6.07E-77	14
RPL36.11	3.56E-81	1.018400805	1	0.911	8.17E-77	14
DDIT4.8	1.49E-80	1.734244116	0.95	0.509	3.43E-76	14
MRPL23	2.33E-80	0.587919074	0.68	0.184	5.35E-76	14
PDCD5.2	4.01E-79	0.586019038	0.656	0.176	9.22E-75	14
NDUFA5.2	1.12E-78	0.622747447	0.776	0.23	2.56E-74	14
RPS8.7	4.91E-78	0.987842273	0.996	0.916	1.13E-73	14
PCNP.5	3.13E-77	0.777299336	0.838	0.281	7.19E-73	14
ST13.8	5.13E-77	0.947729561	0.884	0.362	1.18E-72	14
HSPB1.12	8.77E-77	1.046977163	0.965	0.424	2.02E-72	14
FUNDC2.2	5.41E-76	0.619830244	0.656	0.18	1.24E-71	14
SNRPE.3	5.72E-76	0.775394446	0.846	0.296	1.31E-71	14
SRGN.10	6.75E-76	-3.079070177	0.541	0.796	1.55E-71	14
TCEAL4.2	9.78E-76	0.506533347	0.595	0.151	2.25E-71	14
STOML2	3.90E-75	0.50836705	0.595	0.15	8.96E-71	14
OCIAD2.10	4.09E-75	0.79683691	0.803	0.251	9.40E-71	14
MYC	4.99E-75	0.646001657	0.359	0.064	1.15E-70	14
CEBPD.9	1.01E-74	0.909192236	0.846	0.268	2.32E-70	14
GPI	1.27E-74	0.583980402	0.579	0.145	2.91E-70	14
YWHAE.8	4.97E-74	0.726082152	0.842	0.302	1.14E-69	14
MMP24-AS1.1	6.43E-74	0.545076154	0.622	0.164	1.48E-69	14
HLA-C.10	1.33E-73	-1.763859813	0.958	0.881	3.06E-69	14
ZBTB20.2	1.42E-73	0.59849894	0.587	0.145	3.27E-69	14
VDAC2.4	1.58E-73	0.74988577	0.83	0.288	3.62E-69	14
TNFSF10.3	6.34E-73	0.62679841	0.602	0.159	1.46E-68	14
RPL7.7	1.41E-72	0.815505826	0.992	0.918	3.23E-68	14
TOMM20.3	4.38E-72	0.819418148	0.834	0.3	1.01E-67	14
S100A10.7	1.51E-71	1.043308924	0.988	0.603	3.46E-67	14
PHPT1.6	1.73E-71	0.69689509	0.795	0.266	3.97E-67	14
TMEM123.6	1.78E-71	0.639331132	0.795	0.261	4.08E-67	14
EIF4B.4	2.23E-70	0.900568977	0.853	0.348	5.13E-66	14
RPS27L.7	2.48E-70	1.040760361	0.938	0.464	5.71E-66	14
PERP	8.84E-70	0.612361566	0.359	0.066	2.03E-65	14
PLSCR1.4	5.61E-69	0.581047235	0.575	0.154	1.29E-64	14
PSMC1	2.12E-67	0.510139317	0.664	0.193	4.87E-63	14
CFDP1.1	6.14E-67	0.524589599	0.637	0.184	1.41E-62	14
NENF	7.96E-67	0.517074748	0.645	0.188	1.83E-62	14
ACTB.9	8.34E-67	-1.535903015	0.992	0.928	1.92E-62	14
RPL24.7	1.48E-66	0.87035287	0.996	0.85	3.41E-62	14
ACAA2.1	5.38E-66	0.511147192	0.575	0.155	1.24E-61	14
KCNQ1OT1.1	5.61E-65	0.539853481	0.421	0.091	1.29E-60	14
DUSP23	2.62E-64	0.600445595	0.591	0.166	6.03E-60	14
CAMLG	4.09E-64	0.507261808	0.687	0.207	9.39E-60	14
UBC.5	1.64E-63	1.135347361	1	0.837	3.77E-59	14
FTH1.13	2.55E-63	0.747458151	1	0.962	5.86E-59	14
PRDX6.6	2.99E-63	0.64804824	0.83	0.316	6.88E-59	14
RPL9.5	3.32E-63	0.820031884	0.996	0.9	7.62E-59	14
TUBB.9	3.38E-63	0.691554754	0.849	0.339	7.78E-59	14
SERPINE1.1	3.96E-63	0.627699936	0.282	0.047	9.10E-59	14
AQP3	5.51E-63	0.671661801	0.34	0.065	1.27E-58	14
RAD23A.3	6.56E-63	0.618439014	0.764	0.261	1.51E-58	14
ZFAND5.8	1.02E-62	0.843781124	0.788	0.298	2.34E-58	14
ATF3.1	1.99E-62	0.922976624	0.529	0.143	4.56E-58	14
TMEM14C.3	2.15E-62	0.575273134	0.691	0.218	4.95E-58	14
SLC17A3	4.86E-62	0.568405593	0.266	0.043	1.12E-57	14
NPM1.7	6.01E-62	0.997631572	0.961	0.693	1.38E-57	14

TBCA.6	9.08E-62	0.749400912	0.88	0.394	2.09E-57	14
GSTP1.8	1.18E-61	0.824353995	0.961	0.48	2.71E-57	14
FOXO3.4	1.91E-61	0.597899925	0.517	0.137	4.40E-57	14
NELFE	2.22E-61	0.505918208	0.544	0.148	5.09E-57	14
RSL1D1.5	2.32E-61	0.644167459	0.776	0.28	5.34E-57	14
CHCHD10.9	2.48E-61	0.546535718	0.737	0.237	5.71E-57	14
PGF	9.54E-61	1.414082469	0.286	0.049	2.19E-56	14
ATF4.2	4.66E-59	0.605724452	0.83	0.313	1.07E-54	14
RSL24D1.3	1.21E-58	0.750265844	0.834	0.349	2.78E-54	14
RPL18.6	1.68E-58	0.798784591	0.996	0.868	3.87E-54	14
RPS19BP1.2	1.92E-58	0.500372405	0.726	0.243	4.42E-54	14
RPL18A.7	1.01E-57	0.738587635	1	0.928	2.32E-53	14
RPL5.5	1.34E-57	0.785330972	0.992	0.85	3.08E-53	14
RPL3.8	4.49E-57	0.661645561	0.996	0.931	1.03E-52	14
IGHA1.1	5.06E-57	-1.497488511	0.432	0.098	1.16E-52	14
BTF3.3	5.57E-57	0.788946784	0.981	0.721	1.28E-52	14
FBL.4	1.24E-56	0.595721409	0.714	0.249	2.85E-52	14
HSP90AB1.6	2.15E-56	0.816767473	0.981	0.595	4.95E-52	14
ZNF90.5	3.17E-56	0.727569856	0.826	0.344	7.29E-52	14
MZT2A.9	4.06E-56	0.504092154	0.764	0.262	9.32E-52	14
PTPRC.12	2.91E-55	-2.585422959	0.174	0.625	6.68E-51	14
RPS4X.8	5.51E-55	0.641912867	0.992	0.937	1.27E-50	14
MRFAP1.4	1.60E-54	0.511086876	0.807	0.301	3.68E-50	14
SERPINA1.13	1.67E-54	0.994115502	0.761	0.269	3.83E-50	14
MZT2B.4	6.40E-54	0.615973826	0.838	0.353	1.47E-49	14
GLRX.7	1.72E-53	0.747046801	0.757	0.289	3.96E-49	14
EIF3E.6	2.74E-53	0.855555379	0.946	0.537	6.31E-49	14
HLA-A.10	4.28E-53	-1.287839893	0.973	0.892	9.85E-49	14
RPL8.4	6.14E-53	0.6543431	0.996	0.88	1.41E-48	14
NACA.6	6.92E-53	0.700378783	0.988	0.8	1.59E-48	14
RPL41.10	1.53E-52	0.608164503	1	0.976	3.51E-48	14
LSM5	4.85E-52	0.508172289	0.641	0.213	1.11E-47	14
RPL11.4	7.49E-52	0.594058475	1	0.937	1.72E-47	14
PGAM1.6	1.72E-51	0.643055305	0.83	0.352	3.96E-47	14
RPL10.8	2.14E-51	0.58615603	1	0.974	4.93E-47	14
ARHGDIB.7	3.61E-51	-2.22012491	0.382	0.678	8.29E-47	14
RPL22.6	1.50E-50	0.70512346	0.988	0.807	3.45E-46	14
RPS6.6	2.13E-50	0.690321121	0.988	0.942	4.89E-46	14
P4HB.5	5.98E-50	0.650184727	0.803	0.337	1.37E-45	14
DUSP1.9	1.45E-49	1.033017592	0.992	0.789	3.33E-45	14
TIPARP.1	1.74E-49	0.682623504	0.452	0.126	4.01E-45	14
EEF1A1.8	6.50E-49	0.666196037	1	0.967	1.49E-44	14
NDUFS5.4	3.68E-48	0.655350037	0.934	0.525	8.45E-44	14
BTG1.13	5.00E-48	-1.860290111	0.919	0.841	1.15E-43	14
CCT4	5.21E-48	0.508316522	0.66	0.229	1.20E-43	14
MALAT1.13	7.56E-48	-1.153963872	1	0.982	1.74E-43	14
EEF2.5	1.25E-47	0.747029672	0.965	0.68	2.86E-43	14
ANXA2.8	7.38E-47	0.666379075	0.865	0.384	1.70E-42	14
RTN4.11	1.22E-46	0.623848511	0.819	0.341	2.81E-42	14
TXN.9	4.74E-46	0.527182925	0.849	0.38	1.09E-41	14
RPS2.7	5.63E-46	0.58022039	0.996	0.943	1.29E-41	14
CEBPB.11	1.20E-45	0.854754611	0.745	0.321	2.76E-41	14
RPL36A.8	2.56E-45	0.783243186	0.969	0.798	5.89E-41	14
TAF1D.6	4.76E-45	0.580904898	0.768	0.31	1.09E-40	14
S100A4.12	6.92E-45	-2.718901474	0.556	0.702	1.59E-40	14
RPS13.6	2.73E-44	0.620957817	1	0.876	6.28E-40	14
ATP5E.8	2.94E-44	-1.331940758	0.888	0.814	6.76E-40	14
CXCR4.10	4.07E-44	-2.432129722	0.822	0.774	9.36E-40	14

HLA-E.12	7.11E-44	-1.557216432	0.842	0.789	1.63E-39	14
TPT1.9	2.77E-43	0.599490816	0.996	0.922	6.36E-39	14
RPS28.7	6.00E-43	0.512495827	1	0.946	1.38E-38	14
CD63.11	2.31E-42	0.547246921	0.961	0.511	5.32E-38	14
ZFP36L2.11	3.00E-42	-2.638468166	0.548	0.701	6.90E-38	14
RPS18.8	5.62E-42	0.546050092	1	0.958	1.29E-37	14
TMSB10.8	6.59E-42	0.784988357	0.996	0.96	1.51E-37	14
RGS1.14	3.51E-41	-3.208853595	0.622	0.698	8.07E-37	14
GLTSCR2.6	1.06E-39	0.663284477	0.931	0.581	2.43E-35	14
RPL35A.8	1.36E-39	0.516211738	0.988	0.916	3.12E-35	14
RPL13.7	3.04E-39	0.510101728	1	0.964	6.99E-35	14
RPL38.6	2.91E-38	0.597667183	0.985	0.865	6.68E-34	14
RPL34.8	4.22E-38	0.519502711	0.996	0.952	9.70E-34	14
CORO1A.10	4.31E-38	-2.13136751	0.259	0.57	9.90E-34	14
RPL37.6	1.10E-37	0.544706951	0.988	0.907	2.52E-33	14
HCST.10	1.97E-37	-2.177437179	0.22	0.55	4.53E-33	14
HSD17B7	6.34E-37	0.525399394	0.297	0.076	1.46E-32	14
IER2.6	8.89E-37	0.818480778	0.792	0.394	2.04E-32	14
LAPTM5.11	1.51E-36	-2.087599616	0.274	0.573	3.47E-32	14
UQCRH.3	3.30E-36	0.510364524	0.865	0.456	7.59E-32	14
EZR.5	7.14E-36	0.695955924	0.722	0.317	1.64E-31	14
CFL1.3	1.85E-35	-1.458453207	0.803	0.746	4.24E-31	14
CITED2.11	2.12E-35	0.713234914	0.637	0.261	4.88E-31	14
GMFG.7	2.32E-35	-1.86576471	0.263	0.561	5.33E-31	14
RPS21.8	1.57E-34	0.559004346	0.992	0.86	3.60E-30	14
FYB.12	8.79E-34	-1.921992905	0.112	0.466	2.02E-29	14
CD74.10	1.57E-33	-2.454436832	0.861	0.793	3.62E-29	14
COX7C.4	3.42E-33	0.507004565	0.977	0.749	7.87E-29	14
SLC25A6.6	4.10E-33	0.547549639	0.965	0.649	9.42E-29	14
RPL31.10	7.91E-33	0.508918381	0.981	0.906	1.82E-28	14
SH3BGRL3.10	1.49E-32	-1.737996342	0.61	0.683	3.43E-28	14
RPS25.9	1.83E-32	0.501437819	0.996	0.916	4.20E-28	14
ARPC2.8	1.95E-32	-1.754437371	0.517	0.64	4.49E-28	14
RPL6.7	6.04E-32	0.51353154	1	0.88	1.39E-27	14
DDX5.9	7.79E-32	-1.079933257	0.857	0.81	1.79E-27	14
RPL35.7	1.56E-31	0.543898751	0.981	0.896	3.58E-27	14
CELF2.10	2.12E-31	-1.690889951	0.085	0.432	4.86E-27	14
PFN1.8	6.26E-31	-1.336683035	0.846	0.759	1.44E-26	14
CCNI.3	8.36E-31	0.558781186	0.927	0.587	1.92E-26	14
CD37.10	9.42E-30	-1.690092181	0.154	0.469	2.16E-25	14
CD53.8	7.15E-29	-1.703639421	0.181	0.476	1.64E-24	14
IGKC.10	1.29E-28	-1.344193187	0.691	0.288	2.97E-24	14
HLA-DPB1.11	1.67E-28	-2.814429897	0.525	0.634	3.85E-24	14
PNRC1.7	3.05E-28	0.524800037	0.95	0.628	7.01E-24	14
CD2.13	3.09E-28	-2.366105949	0.17	0.462	7.09E-24	14
EVL.13	3.16E-28	-1.770463969	0.216	0.5	7.27E-24	14
GADD45B.2	3.25E-28	0.525219464	0.556	0.228	7.47E-24	14
HNRNPA1.5	4.96E-28	0.536022443	0.973	0.774	1.14E-23	14
RP11-347P5.1.13	6.84E-28	-1.900460825	0.054	0.381	1.57E-23	14
CD44.9	1.11E-27	-1.747811971	0.212	0.496	2.56E-23	14
CYBA.9	1.21E-27	-1.595843067	0.699	0.687	2.79E-23	14
TMA7.7	7.88E-27	-1.407203513	0.718	0.695	1.81E-22	14
MYL12A.5	1.16E-26	-1.546778431	0.714	0.689	2.67E-22	14
SLA.11	1.47E-26	-1.617100405	0.097	0.405	3.37E-22	14
STK4.10	1.57E-26	-1.749574437	0.197	0.477	3.62E-22	14
IGFBP7.12	3.49E-26	-1.375563508	0.811	0.371	8.03E-22	14
ZFP36L1.9	4.77E-26	0.5420758	0.71	0.36	1.10E-21	14
CYTIP.12	1.17E-25	-1.832529637	0.22	0.48	2.68E-21	14

TGFBI.3	1.22E-25	1.088786832	0.452	0.178	2.80E-21	14
TRAC.14	2.45E-25	-2.313382598	0.158	0.437	5.62E-21	14
CD3E.13	6.56E-25	-1.81696504	0.116	0.403	1.51E-20	14
CD69.13	2.75E-24	-2.69964613	0.336	0.532	6.32E-20	14
CD48.12	3.18E-24	-1.432526438	0.089	0.38	7.31E-20	14
CCL5.13	8.96E-24	-3.223776157	0.417	0.564	2.06E-19	14
CD3D.14	3.60E-23	-2.275218103	0.263	0.479	8.27E-19	14
LSP1.13	6.65E-23	-1.583302413	0.193	0.439	1.53E-18	14
GPSM3.9	1.07E-21	-1.398077345	0.139	0.395	2.46E-17	14
IL2RG.14	2.06E-21	-1.515583094	0.112	0.377	4.74E-17	14
CD52.11	2.81E-21	-2.344577914	0.436	0.551	6.46E-17	14
HLA-DPA1.12	3.23E-21	-2.732209775	0.475	0.572	7.42E-17	14
LCP1.11	3.69E-21	-1.456487299	0.158	0.409	8.47E-17	14
RAC2.13	4.23E-21	-1.418501981	0.112	0.377	9.73E-17	14
ITGB2.11	5.26E-21	-1.505826785	0.178	0.419	1.21E-16	14
FXYD5.7	5.71E-21	-1.478776304	0.286	0.485	1.31E-16	14
ARPC1B.6	8.72E-21	-1.567665368	0.386	0.527	2.00E-16	14
STK17B.10	3.00E-20	-1.527799342	0.162	0.405	6.89E-16	14
GZMA.13	1.65E-19	-2.367972415	0.212	0.423	3.78E-15	14
FOS.12	2.40E-19	0.608355477	0.927	0.677	5.52E-15	14
WIPF1.12	2.76E-19	-1.310918173	0.104	0.348	6.35E-15	14
ETS1.12	1.41E-18	-1.470310961	0.108	0.343	3.23E-14	14
SH3KBP1.10	1.71E-18	-1.089435839	0.066	0.313	3.94E-14	14
EVI2B.8	2.34E-18	-1.270760807	0.1	0.34	5.38E-14	14
CD3G.13	4.38E-18	-1.48181476	0.062	0.298	1.01E-13	14
EVI2A.10	5.90E-18	-1.188399131	0.05	0.284	1.35E-13	14
PTMA.5	7.58E-18	-0.50760807	0.992	0.916	1.74E-13	14
HBA2.10	1.08E-17	-2.126569883	0.541	0.245	2.49E-13	14
GIMAP7.12	1.16E-17	-1.343877726	0.054	0.289	2.66E-13	14
GIMAP4.9	2.10E-17	-1.260271376	0.108	0.343	4.82E-13	14
LIMD2.13	2.59E-17	-1.222126609	0.085	0.315	5.96E-13	14
PIK3IP1.13	2.60E-17	-1.402813696	0.139	0.362	5.98E-13	14
HLA-F.9	4.10E-17	-1.370689084	0.251	0.442	9.42E-13	14
DUSP2.13	4.92E-17	-2.244592756	0.259	0.434	1.13E-12	14
CST7.12	6.30E-17	-2.058058762	0.197	0.393	1.45E-12	14
KIAA1551.10	9.60E-17	-1.37132284	0.124	0.339	2.21E-12	14
SAMSN1.8	5.24E-16	-1.397089692	0.112	0.326	1.20E-11	14
MYO1F.9	5.26E-16	-1.039756489	0.058	0.276	1.21E-11	14
SMCHD1.5	6.05E-16	-1.26899638	0.12	0.331	1.39E-11	14
LCK.14	6.38E-16	-1.249955945	0.077	0.291	1.47E-11	14
FNBP1.12	6.91E-16	-1.251474228	0.17	0.371	1.59E-11	14
HCLS1.8	8.70E-16	-1.209989469	0.12	0.326	2.00E-11	14
EPAS1.2	1.27E-15	-0.571725782	0.297	0.112	2.91E-11	14
RCSD1.9	1.36E-15	-0.954999809	0.042	0.253	3.13E-11	14
UCP2.10	1.54E-15	-1.28804965	0.151	0.35	3.54E-11	14
SEPT6.10	1.63E-15	-1.075038632	0.069	0.281	3.74E-11	14
AAK1.13	1.71E-15	-1.261540922	0.178	0.374	3.92E-11	14
BIN2.9	3.08E-15	-1.090226328	0.046	0.253	7.08E-11	14
ITM2A.13	4.31E-15	-1.604483971	0.112	0.314	9.89E-11	14
HLA-DRA.14	4.62E-15	-2.707623694	0.788	0.671	1.06E-10	14
AKAP13.9	5.64E-15	-1.202524456	0.266	0.435	1.30E-10	14
RPS29.10	7.36E-15	-0.523230353	0.985	0.92	1.69E-10	14
RGS10.8	1.06E-14	-1.225162676	0.1	0.299	2.43E-10	14
SPARC.3	1.54E-14	-1.177778824	0.417	0.178	3.53E-10	14
STK17A.12	1.64E-14	-1.420426394	0.189	0.37	3.77E-10	14
TNFAIP3.13	1.74E-14	-1.734244099	0.309	0.45	3.99E-10	14
PRDM1.9	2.44E-14	-1.381315005	0.097	0.295	5.60E-10	14
TRBC2.13	2.55E-14	-1.800576646	0.208	0.38	5.86E-10	14

APOBEC3G.11	3.83E-14	-1.281495235	0.077	0.275	8.79E-10	14
REL.9	5.20E-14	-1.288895826	0.139	0.325	1.19E-09	14
ISG20.10	7.20E-14	-1.343954942	0.185	0.364	1.66E-09	14
APOE.9	9.47E-14	-1.129535712	0.811	0.431	2.18E-09	14
SERF2.4	1.56E-13	-0.627078781	0.95	0.815	3.58E-09	14
EMB.10	1.65E-13	-1.036783853	0.081	0.273	3.80E-09	14
SYTL3.12	2.37E-13	-1.249258162	0.093	0.28	5.44E-09	14
IL10RA.10	4.48E-13	-0.96158822	0.073	0.262	1.03E-08	14
PPP2R5C.10	4.87E-13	-1.415632531	0.236	0.393	1.12E-08	14
HBB.8	9.05E-13	-2.211308494	0.699	0.386	2.08E-08	14
ELF1.11	1.13E-12	-1.2264294	0.313	0.443	2.60E-08	14
ACAP1.14	1.74E-12	-1.273111742	0.17	0.336	3.99E-08	14
GLIPR1.10	2.36E-12	-1.090146701	0.127	0.302	5.43E-08	14
ACTA2	2.37E-12	-1.156997679	0.309	0.132	5.46E-08	14
CD27.13	2.41E-12	-1.544811662	0.097	0.271	5.53E-08	14
ABRACL.6	2.49E-12	-0.955024809	0.108	0.29	5.73E-08	14
FYN.9	2.63E-12	-1.138298112	0.093	0.269	6.04E-08	14
TRAF3IP3.13	2.84E-12	-0.994478425	0.081	0.259	6.52E-08	14
SMAP2.10	6.73E-12	-1.031039757	0.131	0.301	1.55E-07	14
CTSS.11	1.02E-11	-1.62563621	0.22	0.365	2.34E-07	14
LTB.13	1.15E-11	-1.697646185	0.143	0.304	2.63E-07	14
DOCK8.10	1.97E-11	-0.965582093	0.127	0.294	4.54E-07	14
HLA-DRB1.12	2.35E-11	-2.433399377	0.699	0.588	5.41E-07	14
CD96.14	2.54E-11	-1.12070874	0.089	0.252	5.84E-07	14
ANKRD44.9	3.15E-11	-0.881884655	0.093	0.261	7.25E-07	14
ALOX5AP.10	3.65E-11	-1.520152091	0.181	0.331	8.40E-07	14
PRKCH.9	3.65E-11	-0.983019305	0.093	0.256	8.40E-07	14
NR4A2.10	4.45E-11	-1.294561162	0.12	0.279	1.02E-06	14
S100A9.12	5.52E-11	-1.945232047	0.517	0.246	1.27E-06	14
CRIP1.10	9.72E-11	-1.252361884	0.135	0.296	2.23E-06	14
GZMK.14	1.38E-10	-2.593529229	0.247	0.368	3.17E-06	14
TRBC1.13	1.60E-10	-2.015514028	0.228	0.356	3.67E-06	14
PIK3R1.11	3.38E-10	-1.137455905	0.162	0.311	7.76E-06	14
RGS2.13	3.66E-10	-1.735102324	0.205	0.338	8.42E-06	14
AKNA.9	5.12E-10	-0.966791755	0.112	0.264	1.18E-05	14
RGCC.12	1.06E-09	-1.772703508	0.17	0.31	2.44E-05	14
PCSK7.12	1.36E-09	-1.141366863	0.189	0.323	3.13E-05	14
IL7R.12	1.59E-09	-2.148476382	0.174	0.306	3.64E-05	14
CD7.11	1.75E-09	-1.463314394	0.17	0.303	4.02E-05	14
S100A8.4	2.45E-09	-2.06926973	0.429	0.207	5.63E-05	14
MYL6.5	2.57E-09	-0.624611175	0.946	0.794	5.89E-05	14
NKG7.14	3.07E-09	-2.618073857	0.367	0.428	7.04E-05	14
CCNH.9	4.07E-09	-1.04923918	0.151	0.287	9.35E-05	14
GRN.13	6.45E-09	-0.570779155	0.51	0.249	0.000148192	14
HLA-DQA1.10	7.47E-09	-1.891631141	0.228	0.346	0.000171568	14
CARD16.8	8.77E-09	-1.165369299	0.197	0.32	0.000201517	14
CREM.8	9.98E-09	-1.25635735	0.154	0.283	0.000229302	14
PRPF38B.11	2.50E-08	-1.096950516	0.378	0.436	0.000575135	14
TRIM22.6	2.62E-08	-0.79082953	0.12	0.251	0.000602159	14
C9orf16.4	2.71E-08	-0.913921529	0.243	0.352	0.000622871	14
NKTR.9	6.79E-08	-1.033025112	0.243	0.348	0.001560918	14
FAM49B.8	8.31E-08	-1.044560086	0.251	0.341	0.001908796	14
COTL1.8	8.62E-08	-1.462327167	0.425	0.434	0.001981703	14
OAZ1.9	9.20E-08	-0.640183122	0.927	0.732	0.002114637	14
CCL4.14	1.16E-07	-2.783473208	0.421	0.44	0.002656699	14
CLEC2D.13	1.83E-07	-1.224615705	0.208	0.308	0.004211554	14
ARHGEF1.10	2.74E-07	-0.873256342	0.151	0.264	0.006289946	14
RHOA.9	3.12E-07	-1.035261186	0.56	0.507	0.007178911	14

ICAM3.12	3.31E-07	-0.930456827	0.166	0.274	0.007611839	14
FOXP1.8	5.32E-07	-0.935950763	0.147	0.26	0.012227289	14
RSRP1.8	7.60E-07	-0.952058909	0.359	0.414	0.017464342	14
RNF213.9	1.58E-06	-0.997621847	0.328	0.388	0.03630687	14
GNAS.6	1.93E-06	-0.960394882	0.266	0.346	0.04430199	14
PSAP.12	1.99E-06	-0.511136143	0.838	0.461	0.045691512	14
DEFB1.2	0	4.541399046	0.808	0.076	0	15
S100A1	0	3.869418961	0.776	0.029	0	15
KRT19	0	3.845960186	0.763	0.053	0	15
MT1G.2	0	3.829161785	0.624	0.05	0	15
C19orf33.1	0	3.319988293	0.641	0.048	0	15
GSTA2	0	3.018463002	0.531	0.027	0	15
MACROD2	0	2.440107478	0.371	0.014	0	15
RP11-284H18.1	0	1.969157114	0.327	0.008	0	15
PPP1R14D	0	1.90195669	0.331	0.01	0	15
FABP6.1	3.49E-305	2.586478167	0.51	0.034	8.03E-301	15
RP11-1C1.6	4.41E-295	2.125691612	0.371	0.017	1.01E-290	15
SAA1.2	1.54E-291	6.52376672	0.824	0.118	3.53E-287	15
EGFR-AS1	1.43E-279	1.904029028	0.302	0.011	3.29E-275	15
PDZK1IP1.3	2.19E-279	3.683311046	0.8	0.111	5.04E-275	15
LYG1	2.53E-275	1.818508859	0.314	0.013	5.82E-271	15
WFDC2.1	1.18E-260	2.962087604	0.514	0.042	2.72E-256	15
C5orf46.1	2.83E-259	2.804997372	0.486	0.037	6.51E-255	15
FXYD2.2	3.21E-259	3.57165864	0.808	0.122	7.37E-255	15
GSTA1	9.73E-254	1.994181653	0.347	0.018	2.24E-249	15
REG1A	1.92E-245	3.735600624	0.535	0.05	4.41E-241	15
TSPAN1	2.90E-236	2.377055296	0.433	0.032	6.65E-232	15
SAA2	3.64E-232	5.522237648	0.641	0.082	8.37E-228	15
SLPI	6.95E-219	2.843679963	0.408	0.031	1.60E-214	15
CRYAB.14	3.83E-208	3.650213692	0.939	0.247	8.80E-204	15
MT1E.4	4.97E-204	3.455476341	0.816	0.162	1.14E-199	15
UGT2A3	2.51E-195	1.76250973	0.273	0.014	5.77E-191	15
ADIRF.10	1.12E-194	3.387563593	0.906	0.229	2.58E-190	15
CD24.4	5.34E-190	2.869557289	0.82	0.166	1.23E-185	15
MAOA	4.82E-189	1.839227491	0.306	0.019	1.11E-184	15
KRT18.2	3.36E-178	2.525602937	0.633	0.096	7.73E-174	15
RBP5.1	2.19E-174	2.420310861	0.478	0.055	5.03E-170	15
S100A13.3	2.44E-171	2.547122196	0.682	0.123	5.60E-167	15
MGST1.3	1.84E-166	2.439225957	0.633	0.103	4.23E-162	15
CYB5A.5	4.39E-163	3.263527071	0.82	0.219	1.01E-158	15
PRAP1	3.57E-161	1.729984179	0.273	0.018	8.21E-157	15
NNMT.15	3.01E-154	2.797710675	0.918	0.288	6.92E-150	15
TPM1.3	3.30E-151	3.092525339	0.759	0.184	7.59E-147	15
TMEM176A.3	4.77E-151	2.641885888	0.673	0.137	1.10E-146	15
NDUFC1.10	1.80E-149	3.163308611	0.833	0.261	4.13E-145	15
BNIP3.3	5.64E-148	2.313752291	0.657	0.124	1.30E-143	15
CLU.4	1.70E-144	3.150906516	0.751	0.188	3.91E-140	15
IFI27.2	3.51E-137	2.358217972	0.686	0.145	8.07E-133	15
MT1F.1	1.23E-135	2.554555907	0.608	0.121	2.83E-131	15
MALAT1.14	4.23E-131	-4.233263233	0.878	0.984	9.73E-127	15
NDUFA4L2.14	2.21E-128	2.280866335	0.873	0.278	5.07E-124	15
MIF.12	5.40E-125	3.304303674	0.906	0.498	1.24E-120	15
SNHG25.1	1.96E-122	3.477950415	0.567	0.118	4.49E-118	15
CXCL14.2	5.85E-120	1.833371056	0.42	0.059	1.35E-115	15
GAPDH.9	7.90E-118	2.465090949	0.971	0.864	1.82E-113	15
TMSB4X.7	3.78E-116	-2.638463818	0.82	0.979	8.70E-112	15
TCEA3.1	1.38E-111	1.848706621	0.38	0.051	3.17E-107	15
HCFC1R1.2	8.81E-111	2.217528005	0.555	0.12	2.03E-106	15

KRT8.2	1.39E-110	2.010602094	0.502	0.091	3.20E-106	15
LDHA.12	2.25E-110	2.850667074	0.927	0.572	5.17E-106	15
CCDC146	1.07E-105	1.954989535	0.314	0.038	2.45E-101	15
SERPINA1.14	5.63E-105	2.930694602	0.759	0.27	1.29E-100	15
B2M.11	2.70E-104	-1.801502963	0.947	0.989	6.21E-100	15
FTH1.14	3.01E-101	1.936009894	0.996	0.962	6.93E-97	15
TMEM27.1	1.47E-99	1.485526848	0.273	0.029	3.37E-95	15
UGT2B7.1	1.66E-99	1.518476025	0.306	0.037	3.81E-95	15
TMSB10.9	3.03E-99	1.634773933	0.992	0.96	6.95E-95	15
MT-CO1.7	3.73E-97	-2.293862858	0.576	0.932	8.57E-93	15
HP	8.89E-95	3.300789041	0.253	0.027	2.04E-90	15
MIR4458HG.1	3.99E-93	2.1865239	0.408	0.072	9.18E-89	15
SRGN.11	6.00E-91	-3.379986846	0.127	0.803	1.38E-86	15
SLC17A3.1	1.92E-89	1.738966533	0.31	0.042	4.42E-85	15
SMIM24	3.30E-86	1.344353351	0.253	0.029	7.59E-82	15
C11orf54.1	5.64E-84	1.839709342	0.392	0.072	1.30E-79	15
TGFBI.4	1.62E-83	2.790376913	0.592	0.176	3.73E-79	15
MT-CO2.8	8.07E-80	-2.059652829	0.424	0.883	1.86E-75	15
S100A10.8	1.16E-79	2.14296136	0.869	0.605	2.66E-75	15
BTG1.14	2.62E-79	-2.74335455	0.4	0.85	6.03E-75	15
HLA-B.12	3.99E-79	-1.713091847	0.645	0.919	9.18E-75	15
CTHRC1.1	7.67E-79	1.557292458	0.253	0.032	1.76E-74	15
LGALS2.3	3.25E-78	2.109912217	0.433	0.094	7.46E-74	15
NUPR1.4	1.77E-77	1.926917354	0.51	0.127	4.08E-73	15
CD70	6.52E-77	1.756524212	0.335	0.057	1.50E-72	15
ANXA4.4	6.89E-77	2.093974312	0.539	0.151	1.58E-72	15
HLA-C.11	1.14E-76	-1.893698555	0.531	0.889	2.61E-72	15
MT-ND2.8	1.14E-75	-2.027220986	0.42	0.874	2.63E-71	15
DDX5.10	1.80E-75	-2.239243783	0.249	0.821	4.13E-71	15
CXCR4.11	2.41E-74	-2.989058026	0.208	0.785	5.54E-70	15
MT-ND3.10	2.90E-74	-2.105852956	0.306	0.839	6.67E-70	15
RPS27L.8	1.68E-73	2.069276351	0.804	0.467	3.87E-69	15
SOD2.12	4.59E-72	2.206988877	0.702	0.286	1.05E-67	15
JUNB.12	1.30E-71	-3.187725	0.122	0.734	2.99E-67	15
HLA-E.13	4.93E-71	-2.231825258	0.241	0.799	1.13E-66	15
DSTN.12	1.61E-70	2.000741297	0.698	0.3	3.70E-66	15
ZFP36L2.12	2.74E-70	-3.238635591	0.098	0.709	6.29E-66	15
MT-ATP6.9	1.23E-69	-1.856534865	0.49	0.866	2.82E-65	15
SERPING1.1	1.72E-68	1.936711678	0.429	0.103	3.96E-64	15
UQCRQ.8	5.04E-68	2.266548826	0.767	0.456	1.16E-63	15
RARRES2.3	1.90E-67	1.582431856	0.445	0.106	4.37E-63	15
FTL.12	2.00E-67	0.925250542	0.971	0.936	4.60E-63	15
C1orf186.1	5.18E-66	1.490632255	0.31	0.055	1.19E-61	15
NAT8.1	1.31E-64	1.255960447	0.265	0.041	3.01E-60	15
GNB2L1.4	1.63E-64	1.276750695	0.906	0.834	3.74E-60	15
MT-CO3.8	8.05E-64	-1.602675721	0.555	0.886	1.85E-59	15
RPL21.7	1.25E-62	-1.153875264	0.808	0.954	2.87E-58	15
ALDOA.6	1.79E-62	1.702938234	0.845	0.633	4.12E-58	15
S100A11.12	3.23E-62	1.614816448	0.853	0.586	7.43E-58	15
RPS24.8	5.38E-62	1.033385199	0.963	0.912	1.24E-57	15
ARHGDIB.8	3.48E-61	-2.186206549	0.11	0.682	8.00E-57	15
TMEM176B.3	7.88E-61	1.822747514	0.498	0.149	1.81E-56	15
NEAT1.9	1.82E-60	-2.359292422	0.273	0.761	4.19E-56	15
RPL37.7	1.98E-60	1.115066642	0.955	0.908	4.55E-56	15
RPS29.11	1.21E-59	-1.343972231	0.784	0.924	2.79E-55	15
PTPRC.13	1.30E-58	-2.485355759	0.057	0.626	2.99E-54	15
DUSP23.1	1.42E-58	1.963556561	0.506	0.168	3.27E-54	15
KLF6.11	1.12E-57	-2.514377365	0.098	0.659	2.57E-53	15

FOS.13	4.09E-57	-2.78195622	0.171	0.691	9.39E-53	15
HRSP12.1	6.59E-57	1.599308511	0.29	0.056	1.51E-52	15
GSTP1.9	1.17E-56	1.756150224	0.767	0.484	2.69E-52	15
TXNIP.11	1.57E-56	-2.238075604	0.306	0.772	3.61E-52	15
MT-ND1.9	1.81E-56	-1.727498012	0.327	0.795	4.17E-52	15
ZFP36.9	9.71E-56	-2.513933691	0.139	0.673	2.23E-51	15
MT-ND4.9	4.31E-55	-1.390076225	0.624	0.878	9.90E-51	15
HSPB1.13	1.80E-54	1.683510923	0.755	0.429	4.15E-50	15
ATP5I.2	2.31E-54	1.98728549	0.751	0.516	5.31E-50	15
DYNLRB1.5	3.06E-54	1.971877025	0.641	0.316	7.02E-50	15
S100A6.7	9.66E-54	1.644590996	0.882	0.764	2.22E-49	15
HINT1.7	8.30E-53	1.514119745	0.808	0.623	1.91E-48	15
OCIAD2.11	2.97E-52	1.857724731	0.592	0.255	6.83E-48	15
MT-CYB.11	3.11E-52	-1.505252183	0.388	0.812	7.14E-48	15
JUN.9	4.46E-52	-2.423427983	0.212	0.7	1.03E-47	15
HLA-A.11	2.49E-51	-1.35007225	0.665	0.898	5.73E-47	15
PLIN2.11	4.65E-51	1.833435937	0.665	0.33	1.07E-46	15
S100A4.13	9.75E-51	-2.328030128	0.249	0.707	2.24E-46	15
IFITM3.13	9.90E-51	1.430402916	0.735	0.372	2.28E-46	15
RPL10.9	1.15E-50	1.075107937	0.976	0.975	2.65E-46	15
TXN.10	1.85E-50	1.652659478	0.694	0.384	4.25E-46	15
CORO1A.11	4.07E-50	-2.145397563	0.049	0.574	9.36E-46	15
RGS1.15	9.81E-50	-2.606245621	0.261	0.704	2.25E-45	15
COX7C.5	3.11E-49	1.631857696	0.824	0.752	7.15E-45	15
CD52.12	2.31E-48	-2.639067323	0.049	0.558	5.30E-44	15
SON.10	2.52E-48	-1.802525612	0.053	0.579	5.80E-44	15
HCST.11	1.75E-47	-2.133904188	0.045	0.553	4.01E-43	15
GMFG.8	2.38E-47	-1.930634037	0.053	0.565	5.48E-43	15
NDUFB4.10	2.41E-47	1.783216471	0.682	0.406	5.54E-43	15
GAMT	1.69E-46	1.242485431	0.261	0.054	3.88E-42	15
CISD1.1	4.17E-46	1.695549765	0.371	0.108	9.59E-42	15
CCL5.14	4.73E-46	-3.184722033	0.086	0.57	1.09E-41	15
ACTB.10	4.79E-46	-1.26389881	0.771	0.932	1.10E-41	15
HMGB1.9	6.76E-46	-1.521828465	0.282	0.743	1.55E-41	15
PPIA.5	7.05E-46	1.389876741	0.8	0.645	1.62E-41	15
WBP5.2	1.21E-45	1.458356105	0.322	0.08	2.79E-41	15
CD69.14	2.81E-45	-2.919737016	0.049	0.537	6.46E-41	15
USMG5.5	6.26E-45	1.610514073	0.743	0.525	1.44E-40	15
SRSF7.12	1.11E-44	-2.006891376	0.069	0.553	2.54E-40	15
PTMA.6	1.38E-44	-1.028016922	0.739	0.92	3.17E-40	15
N4BP2L2.9	4.69E-43	-1.695798011	0.057	0.538	1.08E-38	15
PPDPF.9	2.30E-42	1.390616359	0.796	0.657	5.28E-38	15
RPL31.11	4.06E-42	0.844298245	0.939	0.906	9.34E-38	15
TPI1.9	4.41E-42	1.504965792	0.751	0.561	1.01E-37	15
RNASET2.14	4.54E-42	1.716113106	0.657	0.356	1.04E-37	15
SRSF5.7	5.53E-42	-1.539301391	0.151	0.631	1.27E-37	15
AC090498.1.8	1.97E-41	1.643892192	0.735	0.496	4.53E-37	15
RPL41.11	2.21E-41	0.622075819	0.996	0.976	5.08E-37	15
RPSA.8	2.41E-41	-1.318829544	0.465	0.81	5.54E-37	15
PCBD1.1	5.58E-41	1.436334863	0.38	0.121	1.28E-36	15
MYL6B.1	6.43E-41	1.237197766	0.318	0.086	1.48E-36	15
H3F3B.10	1.07E-39	-1.236124016	0.531	0.84	2.46E-35	15
CD3D.15	1.54E-39	-2.341043303	0.037	0.482	3.55E-35	15
PNISR.8	2.09E-39	-1.623327728	0.065	0.518	4.79E-35	15
ZFAS1.11	2.47E-39	1.515864244	0.714	0.48	5.67E-35	15
PKIG.2	4.93E-39	1.185034089	0.257	0.06	1.13E-34	15
GPX3.4	6.05E-39	1.80168712	0.433	0.161	1.39E-34	15
ROMO1.6	1.09E-38	1.835975482	0.571	0.307	2.50E-34	15

CYTIP.13	1.43E-38	-1.91196379	0.041	0.483	3.29E-34	15
STK4.11	1.49E-38	-1.767688609	0.029	0.48	3.42E-34	15
RPS20.7	2.64E-38	0.816476731	0.955	0.896	6.07E-34	15
XIST.9	4.78E-38	-2.176404779	0	0.432	1.10E-33	15
ATP5H.6	5.45E-38	1.634742218	0.608	0.344	1.25E-33	15
H2AFJ.1	6.99E-38	1.667705899	0.482	0.205	1.61E-33	15
TNFAIP3.14	7.86E-38	-2.153732452	0.02	0.455	1.81E-33	15
SARAF.7	1.23E-37	-1.463369867	0.253	0.686	2.82E-33	15
FYB.13	4.44E-37	-1.762195548	0.037	0.467	1.02E-32	15
CD44.10	4.71E-37	-1.780424912	0.069	0.499	1.08E-32	15
SPP1.7	2.05E-36	0.726499998	0.522	0.211	4.70E-32	15
PRDX1.9	2.78E-36	1.566666088	0.641	0.399	6.38E-32	15
MCL1.8	3.18E-36	-1.637432255	0.086	0.522	7.30E-32	15
HSP90AA1.8	6.98E-36	-1.586842569	0.229	0.661	1.60E-31	15
NIT2	7.00E-36	1.559599384	0.331	0.103	1.61E-31	15
EVL.14	9.28E-36	-1.587686542	0.065	0.503	2.13E-31	15
ATP5J.8	1.30E-35	1.661919112	0.624	0.39	2.98E-31	15
NFKBIA.13	1.52E-35	-1.910704814	0.139	0.565	3.49E-31	15
CYSTM1.3	2.02E-35	1.499988237	0.388	0.136	4.65E-31	15
LAPTM5.12	3.74E-35	-1.418090294	0.143	0.575	8.60E-31	15
ELF1.12	4.45E-35	-1.683710351	0.037	0.448	1.02E-30	15
ARPC2.9	4.67E-35	-1.424972347	0.224	0.645	1.07E-30	15
NACA.7	4.78E-35	0.993724539	0.857	0.802	1.10E-30	15
DUSP1.10	5.15E-35	-1.414311222	0.502	0.798	1.18E-30	15
PRDX2.8	6.05E-35	1.536557885	0.522	0.26	1.39E-30	15
CD2.14	8.64E-35	-2.15433069	0.053	0.464	1.98E-30	15
ENO1.12	8.86E-35	1.226299829	0.776	0.59	2.04E-30	15
COX5B.5	9.66E-35	1.411082013	0.71	0.543	2.22E-30	15
DDX24.11	2.47E-34	-1.322648917	0.045	0.464	5.67E-30	15
CD53.9	2.60E-34	-1.540071569	0.057	0.478	5.98E-30	15
GZMA.14	4.74E-34	-2.526904856	0.024	0.426	1.09E-29	15
TRAC.15	5.20E-34	-2.320755203	0.037	0.439	1.19E-29	15
DUSP2.14	8.16E-34	-2.309521924	0.033	0.438	1.87E-29	15
RPS3.9	8.70E-34	-0.778283333	0.853	0.92	2.00E-29	15
TSC22D3.9	1.67E-33	-1.33768639	0.457	0.786	3.83E-29	15
RPLP0.10	2.20E-33	0.883804464	0.869	0.842	5.06E-29	15
ARPC1B.7	2.91E-33	-1.484363442	0.11	0.532	6.69E-29	15
TMEM256.4	5.50E-33	1.589282651	0.502	0.249	1.26E-28	15
MPC2.9	6.26E-33	1.598072314	0.51	0.26	1.44E-28	15
UQCR10.2	7.64E-33	1.757364654	0.629	0.433	1.76E-28	15
SOD1.8	9.17E-33	1.589601311	0.665	0.478	2.11E-28	15
ANXA2.9	1.12E-32	1.350413694	0.641	0.389	2.56E-28	15
NKG7.15	1.36E-32	-2.802695313	0.041	0.434	3.12E-28	15
TIMP1.13	1.47E-32	1.189497546	0.592	0.309	3.39E-28	15
RPL36.12	1.49E-32	0.719931217	0.939	0.912	3.42E-28	15
NPM1.8	1.71E-32	1.109725858	0.792	0.697	3.92E-28	15
PPP2R5C.11	1.72E-32	-1.822756788	0.012	0.397	3.94E-28	15
PABPC1.8	1.93E-32	-1.201345429	0.265	0.67	4.45E-28	15
NDUFS5.5	2.77E-32	1.337822029	0.694	0.529	6.37E-28	15
CD3E.14	5.32E-32	-1.857507796	0.02	0.404	1.22E-27	15
SLA.12	5.35E-32	-1.692913375	0.02	0.406	1.23E-27	15
POLR2I.3	5.80E-32	1.593009434	0.461	0.209	1.33E-27	15
RPL38.7	7.19E-32	0.7689835	0.922	0.867	1.65E-27	15
LSP1.14	7.99E-32	-1.59602686	0.049	0.441	1.84E-27	15
RP11-347P5.1.14	1.08E-31	-1.999088175	0.004	0.382	2.49E-27	15
DDX3X.11	1.49E-31	-1.609883352	0.037	0.421	3.42E-27	15
CD37.11	1.64E-31	-1.317720362	0.065	0.47	3.78E-27	15
STK17B.11	1.87E-31	-1.617340872	0.024	0.407	4.30E-27	15

LRRFIP1.6	2.05E-31	-1.476924038	0.033	0.419	4.72E-27	15
RBM39.8	2.30E-31	-1.196304469	0.131	0.553	5.29E-27	15
AKAP13.10	3.01E-31	-1.370826483	0.045	0.439	6.91E-27	15
PHPT1.7	4.34E-31	1.550557782	0.518	0.272	9.98E-27	15
RPL17.10	4.83E-31	1.298975252	0.739	0.591	1.11E-26	15
VAMP2.10	6.21E-31	-1.280330357	0.073	0.472	1.43E-26	15
ITGB1.8	7.44E-31	-1.465682677	0.078	0.471	1.71E-26	15
RPS12.4	2.01E-30	-0.74334036	0.816	0.94	4.62E-26	15
HLA-DRB5.13	3.47E-30	-2.755847326	0.012	0.38	7.97E-26	15
CCL4.15	4.60E-30	-2.965678608	0.073	0.446	1.06E-25	15
CD48.13	4.62E-30	-1.539602967	0.016	0.381	1.06E-25	15
RPL22.7	5.14E-30	0.912538348	0.853	0.81	1.18E-25	15
CLIC4.2	6.05E-30	1.455674569	0.286	0.089	1.39E-25	15
CCNL1.9	6.17E-30	-1.22046069	0.029	0.405	1.42E-25	15
POLR2J3.8	7.49E-30	-1.495042188	0.029	0.401	1.72E-25	15
CELF2.11	7.55E-30	-1.194316269	0.049	0.433	1.74E-25	15
FUS.10	9.06E-30	-1.367740507	0.086	0.476	2.08E-25	15
COX6A1.4	1.19E-29	1.416556371	0.665	0.516	2.74E-25	15
IFI16.5	1.20E-29	-1.232672109	0.094	0.488	2.76E-25	15
ITGB2.12	1.44E-29	-1.484770332	0.045	0.422	3.31E-25	15
CFLAR.10	1.58E-29	-1.350742527	0.049	0.429	3.63E-25	15
RAC2.14	1.60E-29	-1.598246345	0.016	0.379	3.68E-25	15
PRPF38B.12	2.26E-29	-1.337163997	0.061	0.442	5.20E-25	15
FOSB.6	3.28E-29	-1.628507132	0.057	0.432	7.53E-25	15
IL2RG.15	3.72E-29	-1.645140247	0.02	0.379	8.56E-25	15
DYNLL1.7	3.80E-29	1.282832834	0.62	0.405	8.74E-25	15
FNBP1.13	6.43E-29	-1.448206183	0.016	0.374	1.48E-24	15
ARGLU1.9	6.70E-29	-1.144869287	0.061	0.44	1.54E-24	15
JAK1.9	1.31E-28	-1.374119789	0.037	0.402	3.00E-24	15
AAK1.14	1.73E-28	-1.44058492	0.02	0.377	3.97E-24	15
NAA38.7	3.00E-28	1.535701599	0.514	0.282	6.89E-24	15
ARL6IP5.10	4.10E-28	-1.231212446	0.086	0.464	9.43E-24	15
ID2.10	4.14E-28	-1.57584934	0.204	0.57	9.52E-24	15
RNF213.10	4.38E-28	-1.246447637	0.033	0.394	1.01E-23	15
IER2.7	6.63E-28	-1.598635549	0.045	0.407	1.52E-23	15
SEC61G.8	1.07E-27	1.387794897	0.62	0.434	2.47E-23	15
HIST1H1C.1	1.29E-27	1.738498269	0.339	0.128	2.95E-23	15
ANKRD12.8	1.41E-27	-1.322135726	0.037	0.395	3.24E-23	15
GPSM3.10	1.62E-27	-1.289950383	0.037	0.396	3.73E-23	15
COX6C.4	2.01E-27	1.371339486	0.694	0.565	4.62E-23	15
LCP1.12	2.41E-27	-1.193911779	0.049	0.41	5.53E-23	15
IDS.10	2.75E-27	-1.498186156	0.037	0.379	6.32E-23	15
RPS3A.5	2.92E-27	0.621159678	0.951	0.929	6.71E-23	15
TRBC2.14	3.60E-27	-1.732088859	0.033	0.383	8.28E-23	15
COTL1.9	3.71E-27	-1.621273945	0.086	0.44	8.52E-23	15
PRRC2C.8	5.41E-27	-1.158413781	0.078	0.44	1.24E-22	15
PIK3IP1.14	6.13E-27	-1.510958208	0.02	0.364	1.41E-22	15
CST7.13	6.88E-27	-1.843345868	0.049	0.395	1.58E-22	15
NDUFA2.3	6.98E-27	1.450557223	0.535	0.32	1.60E-22	15
WIPF1.13	1.09E-26	-1.37898129	0.012	0.349	2.52E-22	15
KMT2E.12	1.91E-26	-1.187262604	0.057	0.41	4.38E-22	15
RPL15.3	2.43E-26	-0.636076856	0.8	0.909	5.59E-22	15
TXNDC17	2.51E-26	1.494835629	0.396	0.179	5.76E-22	15
PDCD5.3	2.76E-26	1.56672124	0.4	0.181	6.35E-22	15
UBR4.1	3.35E-26	1.235165157	0.29	0.099	7.69E-22	15
MT-ND5.9	3.72E-26	-1.041236932	0.241	0.617	8.55E-22	15
ATP5C1.3	4.14E-26	1.465306604	0.522	0.316	9.51E-22	15
MYL6.6	6.41E-26	0.807831467	0.845	0.796	1.47E-21	15

RPS8.8	6.56E-26	0.55725977	0.918	0.918	1.51E-21	15
RSRP1.9	8.14E-26	-1.170300331	0.065	0.419	1.87E-21	15
COX7B.3	9.28E-26	1.360102466	0.641	0.467	2.13E-21	15
GZMK.15	1.06E-25	-2.649171038	0.041	0.371	2.44E-21	15
NKTR.10	1.32E-25	-1.151869738	0.02	0.352	3.02E-21	15
EVI2B.9	1.55E-25	-1.421997407	0.016	0.341	3.56E-21	15
ATRX.9	1.68E-25	-1.007091053	0.045	0.393	3.85E-21	15
HSBP1.6	2.38E-25	1.516245416	0.453	0.235	5.47E-21	15
ARF6.8	4.15E-25	-1.305718878	0.049	0.385	9.53E-21	15
LINC00493.3	4.39E-25	1.48974992	0.482	0.271	1.01E-20	15
STK17A.13	4.54E-25	-1.387796889	0.037	0.372	1.04E-20	15
NGFRAP1.2	5.42E-25	1.138400665	0.306	0.109	1.25E-20	15
ETS1.13	6.87E-25	-1.535501462	0.024	0.344	1.58E-20	15
MBNL1.8	7.55E-25	-1.052243671	0.033	0.361	1.73E-20	15
PRKCDBP.2	1.39E-24	0.96292471	0.257	0.081	3.20E-20	15
DDX17.8	1.59E-24	-1.052582647	0.061	0.405	3.66E-20	15
C12orf75.3	2.45E-24	1.253173157	0.367	0.153	5.63E-20	15
ROCK1.8	2.49E-24	-1.18966831	0.02	0.337	5.73E-20	15
NDUFA7	2.54E-24	1.251517783	0.322	0.128	5.85E-20	15
HLA-F.10	2.58E-24	-1.070287026	0.098	0.444	5.93E-20	15
WSB1.6	2.84E-24	-1.03224869	0.037	0.366	6.54E-20	15
PNRC1.8	3.08E-24	-0.868031689	0.29	0.64	7.07E-20	15
KIAA1551.11	3.32E-24	-1.298478101	0.024	0.341	7.63E-20	15
TCF25.4	3.34E-24	-0.95553052	0.073	0.42	7.67E-20	15
UQCRB.5	4.16E-24	0.989127733	0.771	0.744	9.56E-20	15
ARID4B.11	4.17E-24	-0.996098645	0.024	0.346	9.59E-20	15
HNRNPA2B1.6	4.30E-24	-0.923475679	0.269	0.634	9.89E-20	15
RNF181.6	5.60E-24	1.455740626	0.478	0.266	1.29E-19	15
HERPUD1.10	5.70E-24	-1.283390183	0.151	0.498	1.31E-19	15
AKAP9.7	6.81E-24	-1.14946179	0.029	0.347	1.56E-19	15
UQCRH.4	8.83E-24	-1.1727303	0.114	0.47	2.03E-19	15
PPP1R15A.8	9.94E-24	-1.217938958	0.073	0.409	2.29E-19	15
SCAF11.6	1.09E-23	-0.942721224	0.045	0.366	2.51E-19	15
MINOS1.4	1.16E-23	1.449330141	0.559	0.382	2.67E-19	15
RBM25.7	1.24E-23	-1.151996166	0.045	0.365	2.85E-19	15
STAT3.10	1.28E-23	-1.268065455	0.016	0.323	2.93E-19	15
IRF1.10	3.05E-23	-1.524922833	0.016	0.316	7.00E-19	15
HEBP2	3.55E-23	1.356575315	0.31	0.121	8.15E-19	15
HNRNPA0.3	3.67E-23	-1.04577255	0.078	0.409	8.43E-19	15
ITM2A.14	4.76E-23	-1.59322538	0.012	0.316	1.09E-18	15
CLK1.10	6.00E-23	-1.130192983	0.045	0.362	1.38E-18	15
HMGB2.10	6.68E-23	-1.199196208	0.135	0.482	1.54E-18	15
SMCHD1.6	7.21E-23	-1.188114902	0.024	0.333	1.66E-18	15
PCSK7.13	8.38E-23	-1.24462975	0.02	0.326	1.93E-18	15
SAMSN1.9	9.95E-23	-1.372121486	0.024	0.328	2.29E-18	15
ADI1	1.62E-22	1.382070685	0.351	0.158	3.72E-18	15
CETN2.1	1.96E-22	1.258067264	0.261	0.092	4.49E-18	15
MYH9.9	2.71E-22	-1.176028998	0.02	0.316	6.23E-18	15
CD3G.14	3.03E-22	-1.524289313	0.008	0.298	6.95E-18	15
CCND3.6	3.39E-22	-1.053742883	0.049	0.365	7.79E-18	15
CTSS.12	3.46E-22	-1.477158846	0.057	0.368	7.95E-18	15
HNRNPA3.3	3.59E-22	-1.072070031	0.135	0.469	8.25E-18	15
TRBC1.14	3.66E-22	-1.854248359	0.053	0.358	8.40E-18	15
FAM162A.3	3.72E-22	1.349159063	0.384	0.182	8.56E-18	15
SRSF2.10	4.04E-22	-0.937705495	0.098	0.429	9.28E-18	15
JUND.8	4.56E-22	-1.286472359	0.02	0.315	1.05E-17	15
PPP1R2.8	6.57E-22	-1.218975173	0.045	0.348	1.51E-17	15
LTB.14	6.72E-22	-1.892762758	0.02	0.306	1.54E-17	15

WASF2.6	7.76E-22	-1.164637545	0.037	0.335	1.78E-17	15
GIMAP4.10	9.32E-22	-1.117635262	0.041	0.344	2.14E-17	15
ACAP1.15	9.81E-22	-1.311933341	0.041	0.338	2.25E-17	15
MZT2A.10	9.89E-22	1.299733102	0.469	0.267	2.27E-17	15
TRA2B.5	1.07E-21	-1.037390361	0.065	0.374	2.45E-17	15
BTG2.9	1.14E-21	-1.346737404	0.057	0.363	2.61E-17	15
SYNE2.14	1.55E-21	-1.106084386	0.033	0.338	3.55E-17	15
TNRC6B.7	1.71E-21	-1.27385766	0.033	0.322	3.92E-17	15
LCK.15	1.88E-21	-1.353586972	0.012	0.292	4.32E-17	15
RSRC2.8	1.99E-21	-1.13387658	0.073	0.383	4.56E-17	15
ADGRE5.12	2.05E-21	-1.28850157	0.016	0.304	4.70E-17	15
CYBA.10	2.34E-21	-0.98079532	0.396	0.692	5.37E-17	15
SEPT7.7	2.58E-21	-0.944129578	0.18	0.519	5.92E-17	15
SP100.10	2.73E-21	-0.765285716	0.086	0.404	6.28E-17	15
SRRM2.5	2.77E-21	-1.013802831	0.049	0.346	6.37E-17	15
NCOR1.9	3.05E-21	-1.028643641	0.049	0.353	7.02E-17	15
ACTR2.7	3.46E-21	-0.865326208	0.061	0.373	7.95E-17	15
RGCC.13	3.62E-21	-1.99166262	0.029	0.312	8.33E-17	15
RPS27.9	3.70E-21	-0.555660131	0.955	0.976	8.51E-17	15
PSMA3-AS1.9	3.91E-21	-1.18478753	0.004	0.28	8.98E-17	15
NDUFA1.3	4.41E-21	1.299681785	0.612	0.482	1.01E-16	15
DNPH1.1	5.51E-21	1.145266258	0.318	0.135	1.27E-16	15
GCC2.9	5.91E-21	-0.775895885	0.061	0.365	1.36E-16	15
NCL.5	6.22E-21	-0.840801332	0.118	0.437	1.43E-16	15
IL7R.13	8.31E-21	-2.308628798	0.029	0.308	1.91E-16	15
LAMTOR5.8	1.39E-20	1.321874441	0.522	0.342	3.20E-16	15
TUBA1A.4	1.52E-20	-1.235030505	0.049	0.343	3.49E-16	15
REL.10	1.59E-20	-1.154411718	0.041	0.327	3.65E-16	15
BCLAF1.8	1.60E-20	-1.086804764	0.049	0.341	3.67E-16	15
NDUFB10.6	2.41E-20	1.417188247	0.518	0.341	5.53E-16	15
CLEC2B.8	2.64E-20	-1.179474506	0.09	0.383	6.06E-16	15
KIF5B.3	4.36E-20	-0.995477658	0.016	0.289	1.00E-15	15
RPL36A.9	4.52E-20	0.746383043	0.829	0.801	1.04E-15	15
RPL8.5	4.67E-20	0.548567737	0.882	0.882	1.07E-15	15
LIMD2.14	4.74E-20	-1.075580132	0.033	0.316	1.09E-15	15
CLEC2D.14	4.79E-20	-1.3176082	0.033	0.311	1.10E-15	15
MTDH.5	4.90E-20	-0.765527618	0.082	0.393	1.13E-15	15
CD7.12	5.50E-20	-1.462931758	0.029	0.305	1.26E-15	15
MSN.8	5.90E-20	-0.979045905	0.078	0.371	1.36E-15	15
XRCC5.4	6.80E-20	-0.759565633	0.041	0.329	1.56E-15	15
SLC25A6.7	7.39E-20	-0.977172842	0.359	0.66	1.70E-15	15
CD99.10	1.05E-19	-1.024697625	0.171	0.475	2.42E-15	15
ATP5G3.3	1.10E-19	1.371538245	0.543	0.405	2.53E-15	15
CFAP36.1	1.26E-19	1.042602023	0.253	0.093	2.89E-15	15
SEPT6.11	1.31E-19	-1.044048284	0.016	0.282	3.00E-15	15
POLR2L.6	1.35E-19	1.258069912	0.571	0.434	3.10E-15	15
EVI2A.11	1.36E-19	-1.071930523	0.016	0.285	3.13E-15	15
SH3KBP1.11	1.40E-19	-1.023068746	0.041	0.313	3.23E-15	15
FOXP1.9	1.63E-19	-1.1977289	0.004	0.262	3.74E-15	15
TERF2IP.8	1.64E-19	-0.997882318	0.078	0.37	3.76E-15	15
YWHAZ.10	1.66E-19	-0.970553624	0.188	0.499	3.82E-15	15
LUC7L3.8	1.67E-19	-1.039971561	0.041	0.316	3.83E-15	15
PRDM1.10	1.87E-19	-1.388121913	0.029	0.296	4.29E-15	15
VDAC1.6	1.87E-19	1.349698256	0.465	0.274	4.29E-15	15
MYO1F.10	2.10E-19	-1.019761515	0.012	0.276	4.83E-15	15
PDCD4.6	2.35E-19	-0.982064335	0.033	0.309	5.39E-15	15
CFL1.4	2.58E-19	-0.760922115	0.465	0.752	5.93E-15	15
FKBP5.9	2.76E-19	-1.074368895	0.106	0.401	6.34E-15	15

ATPIF1.3	3.03E-19	1.23720619	0.567	0.418	6.97E-15	15
RBPJ.9	3.16E-19	-1.065258041	0.049	0.326	7.26E-15	15
SYTL3.13	3.25E-19	-1.317412999	0.02	0.282	7.47E-15	15
FAM133B.5	3.82E-19	-1.060711205	0.037	0.307	8.77E-15	15
RPL7.8	4.18E-19	0.538046803	0.918	0.92	9.60E-15	15
PEBP1.11	4.22E-19	1.111170046	0.559	0.387	9.70E-15	15
FKBP2.2	4.50E-19	1.300082621	0.42	0.234	1.03E-14	15
SF1.8	4.91E-19	-0.818013572	0.041	0.315	1.13E-14	15
FYN.10	5.29E-19	-1.224439111	0.012	0.27	1.22E-14	15
CRIP1.11	5.30E-19	-1.283532774	0.029	0.298	1.22E-14	15
MPHOSPH8.6	5.71E-19	-0.983242845	0.037	0.307	1.31E-14	15
TRAF3IP3.14	7.10E-19	-1.124285415	0.008	0.26	1.63E-14	15
SERF2.5	7.77E-19	0.726729657	0.812	0.818	1.79E-14	15
USP15.8	8.15E-19	-0.980295776	0.012	0.267	1.87E-14	15
ZFP36L1.10	8.33E-19	-0.938354435	0.086	0.371	1.92E-14	15
GLIPR1.11	8.39E-19	-1.024118577	0.037	0.304	1.93E-14	15
PNN.7	9.24E-19	-1.002075795	0.041	0.309	2.12E-14	15
SYTL2.2	9.55E-19	1.114167439	0.257	0.097	2.19E-14	15
CDC42SE2.8	1.00E-18	-1.087567982	0.049	0.32	2.30E-14	15
MRPL51.5	1.07E-18	1.19127015	0.449	0.262	2.45E-14	15
EMB.11	1.28E-18	-0.98968796	0.016	0.274	2.95E-14	15
KTN1.8	1.40E-18	-0.843533657	0.094	0.388	3.23E-14	15
IQGAP1.7	1.66E-18	-0.818428732	0.065	0.343	3.82E-14	15
NR4A2.11	1.86E-18	-1.294300378	0.024	0.281	4.27E-14	15
IL10RA.11	1.97E-18	-1.034340799	0.012	0.263	4.53E-14	15
GLTSCR2.7	2.04E-18	-0.779827829	0.269	0.593	4.69E-14	15
BNIP3L.3	2.04E-18	1.180706392	0.42	0.228	4.70E-14	15
TBCA.7	2.09E-18	1.324637095	0.547	0.4	4.81E-14	15
HCLS1.9	2.44E-18	-0.76383885	0.053	0.327	5.61E-14	15
SLC2A3.7	2.49E-18	-1.297827184	0.016	0.269	5.73E-14	15
ANKRD44.10	2.66E-18	-0.948268388	0.012	0.263	6.12E-14	15
SRSF11.5	2.75E-18	-0.80465842	0.098	0.381	6.31E-14	15
THRAP3.3	2.78E-18	-0.792951983	0.033	0.296	6.40E-14	15
ARHGEF1.11	2.94E-18	-0.929659444	0.016	0.267	6.76E-14	15
NDUFA4.5	3.02E-18	1.064518113	0.637	0.538	6.93E-14	15
RPL24.8	3.14E-18	0.63604578	0.837	0.853	7.21E-14	15
FXYD5.8	3.42E-18	-0.805128027	0.18	0.486	7.86E-14	15
PPP1R12A.6	3.57E-18	-0.894395234	0.024	0.284	8.20E-14	15
RPL9.6	3.62E-18	0.517291369	0.914	0.901	8.33E-14	15
BIN2.10	4.24E-18	-1.105032918	0.008	0.254	9.73E-14	15
TAOK3.8	5.19E-18	-0.900330859	0.02	0.27	1.19E-13	15
GNLY.13	5.43E-18	-3.106321454	0.012	0.257	1.25E-13	15
DOCK8.11	5.80E-18	-0.93134355	0.037	0.296	1.33E-13	15
HNRNPU.4	5.90E-18	-0.818446365	0.098	0.379	1.36E-13	15
MT-ND4L.8	6.06E-18	-0.914176766	0.086	0.365	1.39E-13	15
RCSD1.10	6.39E-18	-0.91791668	0.008	0.254	1.47E-13	15
ODF2L.9	6.62E-18	-1.18838675	0.016	0.264	1.52E-13	15
FAM49B.9	7.29E-18	-0.990606987	0.073	0.345	1.68E-13	15
EPC1.9	7.42E-18	-1.005304527	0.024	0.274	1.70E-13	15
BOLA3	7.89E-18	1.128691863	0.273	0.112	1.81E-13	15
PRKCH.10	9.03E-18	-1.008189782	0.012	0.258	2.08E-13	15
SH3BGRL3.11	9.55E-18	-0.852689583	0.388	0.687	2.19E-13	15
ATP5O.7	1.06E-17	1.124986904	0.551	0.408	2.42E-13	15
CD96.15	1.10E-17	-1.198456005	0.012	0.253	2.52E-13	15
PDLIM1.2	1.21E-17	0.791497786	0.29	0.117	2.78E-13	15
RPA3.1	1.60E-17	1.144634833	0.359	0.183	3.68E-13	15
GIMAP7.13	1.65E-17	-0.930493834	0.033	0.289	3.79E-13	15
AKNA.10	2.01E-17	-1.001677981	0.02	0.265	4.61E-13	15

PIK3R1.12	2.02E-17	-1.019476445	0.049	0.313	4.64E-13	15
CNN3.3	2.11E-17	0.517576324	0.273	0.105	4.85E-13	15
RPS21.9	2.43E-17	0.62354139	0.841	0.862	5.58E-13	15
MYCBP2.7	2.70E-17	-0.930227686	0.02	0.262	6.20E-13	15
EMP3.7	2.78E-17	-0.911777373	0.114	0.395	6.39E-13	15
ATM.8	4.38E-17	-0.990935842	0.016	0.253	1.01E-12	15
BTN3A2.8	5.00E-17	-0.80484271	0.024	0.267	1.15E-12	15
EIF4A2.8	5.21E-17	-0.584467449	0.265	0.585	1.20E-12	15
UCP2.11	9.44E-17	-0.871099615	0.082	0.351	2.17E-12	15
NHP2.1	1.00E-16	1.260551678	0.38	0.21	2.30E-12	15
CREM.9	1.02E-16	-1.230272403	0.041	0.284	2.36E-12	15
SRRM1.4	1.08E-16	-0.598357592	0.131	0.424	2.49E-12	15
ANGPTL4.3	1.12E-16	0.833694446	0.355	0.164	2.58E-12	15
LEPROTL1.7	1.16E-16	-0.84934444	0.114	0.394	2.67E-12	15
ISG20.11	1.24E-16	-1.092016254	0.106	0.366	2.85E-12	15
CHMP4A.2	1.34E-16	-0.780218481	0.065	0.323	3.09E-12	15
GLRX.8	1.57E-16	1.36807185	0.457	0.295	3.60E-12	15
PSMB9.6	1.74E-16	-0.726887088	0.188	0.489	4.00E-12	15
PPIG.4	1.75E-16	-0.642023017	0.061	0.325	4.02E-12	15
EEF2.6	1.84E-16	-0.594276194	0.388	0.69	4.22E-12	15
SEPT9.8	2.17E-16	-0.935095045	0.02	0.254	5.00E-12	15
BRD2.6	2.29E-16	-0.92410138	0.02	0.251	5.26E-12	15
COX6B1.1	2.35E-16	0.98573352	0.653	0.572	5.40E-12	15
YPEL5.10	2.62E-16	-1.01466063	0.09	0.349	6.02E-12	15
CAP1.5	3.11E-16	-0.960869341	0.159	0.416	7.15E-12	15
TLN1.4	3.63E-16	-0.837093907	0.033	0.269	8.34E-12	15
RPL23.7	3.84E-16	0.590319307	0.816	0.817	8.83E-12	15
DBI.4	4.94E-16	1.298372694	0.551	0.421	1.14E-11	15
ARPC4.6	4.99E-16	-0.801154686	0.086	0.341	1.15E-11	15
MACF1.9	5.32E-16	-0.729333523	0.029	0.261	1.22E-11	15
RPS11.8	5.40E-16	0.567375392	0.882	0.855	1.24E-11	15
DDX6.8	5.72E-16	-0.904310377	0.02	0.251	1.31E-11	15
JMJD1C.6	6.34E-16	-0.823000227	0.029	0.262	1.46E-11	15
PFKP.1	6.72E-16	1.064453264	0.261	0.11	1.54E-11	15
SF3B1.4	7.15E-16	-0.693635013	0.073	0.33	1.64E-11	15
DDX46.4	9.27E-16	-0.762592092	0.065	0.319	2.13E-11	15
CARHSP1.3	9.45E-16	1.04776621	0.376	0.203	2.17E-11	15
NDUFB6	9.61E-16	1.343506842	0.355	0.192	2.21E-11	15
MGST3.11	1.01E-15	1.07589037	0.465	0.304	2.32E-11	15
CCNH.10	1.07E-15	-0.88732624	0.049	0.288	2.45E-11	15
RNF19A.12	1.12E-15	-0.932337854	0.045	0.281	2.57E-11	15
CD27.14	1.36E-15	-1.205943821	0.037	0.272	3.11E-11	15
APOBEC3G.12	1.45E-15	-1.013425275	0.045	0.275	3.33E-11	15
ATOX1.4	1.46E-15	1.035017473	0.392	0.221	3.36E-11	15
TAPBP.7	1.49E-15	-0.616546625	0.073	0.329	3.43E-11	15
RGS10.9	1.60E-15	-0.947511011	0.061	0.299	3.67E-11	15
SMAP2.11	1.90E-15	-0.785453503	0.057	0.302	4.36E-11	15
MMP24-AS1.2	2.00E-15	1.180440016	0.331	0.169	4.60E-11	15
HNRNPM.1	2.08E-15	-0.765572718	0.069	0.323	4.79E-11	15
TGOLN2.4	2.21E-15	-0.66815284	0.037	0.265	5.07E-11	15
RNPS1.5	3.32E-15	-0.830070534	0.037	0.266	7.64E-11	15
EIF5.2	3.76E-15	-0.697548839	0.078	0.329	8.63E-11	15
MGEA5.9	3.88E-15	-0.762448958	0.041	0.269	8.91E-11	15
DHX36.6	4.62E-15	-0.748696144	0.041	0.269	1.06E-10	15
WNK1.8	4.89E-15	-0.843096761	0.033	0.253	1.12E-10	15
TPR.6	4.90E-15	-0.699792267	0.065	0.312	1.13E-10	15
BLVRB.3	5.50E-15	0.984547781	0.318	0.157	1.26E-10	15
UBXN1.3	5.95E-15	-0.614957234	0.127	0.396	1.37E-10	15

UBB.6	6.58E-15	0.716238553	0.751	0.719	1.51E-10	15
DNAJB6.3	7.30E-15	-0.806949668	0.094	0.344	1.68E-10	15
BUB3.10	7.78E-15	-0.766869234	0.049	0.275	1.79E-10	15
PAPOLA.2	7.82E-15	-0.754000453	0.078	0.317	1.80E-10	15
VIM.12	9.25E-15	0.703293314	0.853	0.77	2.12E-10	15
DNAJA1.5	9.26E-15	-0.665764414	0.086	0.336	2.13E-10	15
RGS2.14	1.04E-14	-1.286271875	0.094	0.339	2.38E-10	15
CFDP1.2	1.04E-14	1.221916172	0.347	0.189	2.39E-10	15
RHOG.8	1.17E-14	-0.664525297	0.041	0.268	2.70E-10	15
SLTM.5	1.20E-14	-0.880286647	0.057	0.284	2.75E-10	15
TAF7.7	1.27E-14	-0.752849369	0.082	0.325	2.91E-10	15
MYL12A.6	1.37E-14	-0.751543874	0.433	0.694	3.15E-10	15
WDR1.2	1.40E-14	-0.670830494	0.045	0.271	3.22E-10	15
GNAI2.10	1.47E-14	-0.748635023	0.041	0.264	3.38E-10	15
ACTR3.5	1.59E-14	-0.592639677	0.131	0.386	3.65E-10	15
HLA-DPB1.12	1.93E-14	-1.708983854	0.429	0.636	4.44E-10	15
MRPL40.1	2.39E-14	0.988423273	0.278	0.131	5.49E-10	15
ZFAND5.9	2.64E-14	-0.980858663	0.078	0.311	6.08E-10	15
CKLF.8	2.97E-14	-0.776838899	0.114	0.368	6.82E-10	15
CTSC.12	3.50E-14	-0.71234382	0.102	0.351	8.03E-10	15
HNRNPH1.5	3.63E-14	-0.711303263	0.086	0.324	8.33E-10	15
ICAM3.13	3.81E-14	-0.736640602	0.053	0.276	8.75E-10	15
RTF1.2	4.06E-14	-0.681142174	0.057	0.281	9.33E-10	15
CCDC85B.5	4.09E-14	-0.686699984	0.037	0.257	9.40E-10	15
ATP5G1.2	4.12E-14	1.241605391	0.392	0.241	9.46E-10	15
ALOX5AP.11	5.19E-14	-1.287130278	0.11	0.333	1.19E-09	15
NDUFB9.2	5.40E-14	1.077193702	0.473	0.33	1.24E-09	15
PRDX6.7	5.95E-14	1.158729937	0.465	0.323	1.37E-09	15
DPY30.2	5.99E-14	1.172672397	0.367	0.212	1.38E-09	15
PRKAR1A.2	6.54E-14	-0.70294801	0.045	0.265	1.50E-09	15
RAB13.4	6.74E-14	0.846546818	0.286	0.134	1.55E-09	15
GABPB1.8	7.41E-14	-0.645109434	0.061	0.284	1.70E-09	15
RABAC1.6	8.58E-14	-0.698623072	0.122	0.361	1.97E-09	15
OST4.4	9.22E-14	0.912328474	0.673	0.66	2.12E-09	15
UQCR11.3	1.03E-13	0.882099962	0.649	0.609	2.36E-09	15
ANXA6.11	1.03E-13	-0.904763684	0.045	0.253	2.37E-09	15
NFE2L2.10	1.03E-13	-0.826755057	0.049	0.267	2.37E-09	15
LAMP1.4	1.20E-13	-0.604427531	0.078	0.308	2.77E-09	15
HSPA8.8	1.69E-13	-0.748968764	0.29	0.559	3.89E-09	15
PCM1.6	1.72E-13	-0.737177124	0.045	0.256	3.96E-09	15
FKBP8.1	1.98E-13	-0.597151333	0.094	0.336	4.55E-09	15
SELM.3	2.09E-13	1.202217099	0.335	0.181	4.81E-09	15
RNF149.11	2.15E-13	-0.903484125	0.061	0.273	4.93E-09	15
FUNDC2.3	2.18E-13	0.960716612	0.335	0.187	5.00E-09	15
HNRNPR.3	2.38E-13	-0.656283992	0.061	0.28	5.46E-09	15
YWHAB.7	2.48E-13	-0.803183394	0.245	0.491	5.69E-09	15
DNAJB1.11	2.54E-13	-0.746848262	0.171	0.424	5.83E-09	15
RAP1A.7	2.85E-13	-0.623095942	0.114	0.354	6.56E-09	15
MGST2.3	3.51E-13	1.013282969	0.31	0.163	8.07E-09	15
SF3B2.6	4.22E-13	-0.72386776	0.094	0.32	9.70E-09	15
SFPQ.6	4.38E-13	-0.607673806	0.086	0.315	1.01E-08	15
CD164.4	4.50E-13	-0.698926112	0.106	0.335	1.03E-08	15
SELT.3	4.87E-13	-0.73410383	0.086	0.314	1.12E-08	15
ATP6AP2.7	5.66E-13	-0.700187509	0.086	0.311	1.30E-08	15
S100A9.13	7.33E-13	-2.542729287	0.053	0.255	1.68E-08	15
TRIM22.7	7.35E-13	-0.587394856	0.045	0.252	1.69E-08	15
VMP1.9	7.57E-13	-0.746603852	0.098	0.324	1.74E-08	15
RSF1.5	8.07E-13	-0.651377285	0.053	0.26	1.86E-08	15

GNG5.9	8.46E-13	1.09699782	0.482	0.349	1.94E-08	15
ATP1B3.11	9.47E-13	-0.662130485	0.061	0.272	2.18E-08	15
ARPC5.7	1.17E-12	-0.555521694	0.188	0.44	2.69E-08	15
RPL36AL.5	1.46E-12	-0.618219676	0.465	0.716	3.37E-08	15
NDUFB7.3	1.57E-12	1.008721831	0.465	0.333	3.61E-08	15
TCEB2.5	1.64E-12	0.901203775	0.612	0.57	3.77E-08	15
CALM1.7	1.85E-12	-0.658290352	0.331	0.601	4.24E-08	15
CASP4.6	1.97E-12	-0.53555729	0.078	0.294	4.54E-08	15
POLR3GL.6	2.00E-12	-0.703499809	0.061	0.266	4.59E-08	15
NDUFV2.5	2.24E-12	1.127236471	0.433	0.301	5.16E-08	15
MT2A.15	2.34E-12	0.561576273	0.735	0.658	5.37E-08	15
TMEM14C.4	2.44E-12	1.167609084	0.363	0.224	5.61E-08	15
LITAF.10	2.77E-12	-0.537925456	0.139	0.371	6.37E-08	15
PSME1.4	3.64E-12	-0.565514932	0.327	0.585	8.37E-08	15
ATP1B1.4	4.11E-12	0.930100312	0.278	0.135	9.44E-08	15
FCER1G.15	4.93E-12	-1.52977294	0.11	0.306	1.13E-07	15
LIMS1.10	5.15E-12	-0.797348886	0.057	0.252	1.18E-07	15
PPP1CA.2	5.29E-12	-0.762024349	0.147	0.368	1.22E-07	15
TUBA4A.12	5.43E-12	-0.8593493	0.073	0.276	1.25E-07	15
ATP6V1G1.6	5.56E-12	-0.559844974	0.212	0.466	1.28E-07	15
GABARAP.3	8.93E-12	1.131279424	0.318	0.182	2.05E-07	15
PRDX5.5	9.70E-12	0.891215888	0.473	0.349	2.23E-07	15
CMC1.13	1.02E-11	-1.299869144	0.09	0.288	2.33E-07	15
DDT.4	1.04E-11	1.064260479	0.412	0.282	2.40E-07	15
SEPT2.5	1.05E-11	-0.617826285	0.065	0.265	2.41E-07	15
ARID5B.8	1.23E-11	-0.731479329	0.061	0.258	2.82E-07	15
PLA2G16.1	1.89E-11	0.92016369	0.322	0.183	4.35E-07	15
HILPDA.2	1.98E-11	0.518982323	0.265	0.125	4.56E-07	15
IFNGR1.11	2.11E-11	-0.641154121	0.069	0.262	4.86E-07	15
PPA1.2	2.94E-11	1.099493255	0.363	0.227	6.75E-07	15
FAM96B.2	2.97E-11	1.129255747	0.416	0.29	6.82E-07	15
C10orf54.10	3.55E-11	-0.725125333	0.086	0.285	8.16E-07	15
SAT1.11	3.57E-11	-1.216418826	0.429	0.635	8.21E-07	15
LAPTM4A.10	4.57E-11	1.114262253	0.478	0.35	1.05E-06	15
EIF4A1.3	5.00E-11	-0.568070248	0.135	0.359	1.15E-06	15
SDCBP.12	6.03E-11	-0.696514127	0.155	0.376	1.39E-06	15
SSU72.2	6.25E-11	-0.598872489	0.11	0.315	1.44E-06	15
RHOA.10	7.76E-11	-0.578359902	0.278	0.512	1.78E-06	15
DRAP1.4	7.79E-11	-0.55784029	0.155	0.374	1.79E-06	15
YBX3.3	8.94E-11	0.821536357	0.322	0.182	2.06E-06	15
MRPL22	8.97E-11	1.18632521	0.265	0.141	2.06E-06	15
SNRNP70.3	9.74E-11	-0.534790713	0.065	0.252	2.24E-06	15
HLA-DPA1.13	1.32E-10	-1.511081585	0.384	0.574	3.03E-06	15
BZW1.4	1.33E-10	-0.634706765	0.078	0.267	3.05E-06	15
HSPE1.7	1.49E-10	0.923739304	0.498	0.381	3.42E-06	15
C16orf13.3	1.61E-10	1.083325313	0.363	0.237	3.71E-06	15
NDUFS4.1	1.96E-10	1.014755524	0.335	0.205	4.52E-06	15
HLA-DQA1.11	1.99E-10	-1.385021039	0.155	0.347	4.57E-06	15
GGNBP2.1	2.04E-10	-0.563280618	0.078	0.263	4.70E-06	15
ST13.9	2.77E-10	0.873951513	0.482	0.37	6.37E-06	15
LINC00152.8	2.89E-10	-0.71242171	0.122	0.32	6.64E-06	15
GTF2H5	2.92E-10	1.058058917	0.306	0.18	6.70E-06	15
TYROBP.15	3.04E-10	-1.516697405	0.212	0.391	6.98E-06	15
ZNHIT1.3	3.21E-10	0.971286395	0.404	0.281	7.39E-06	15
MRPS21.2	3.22E-10	1.237139126	0.437	0.322	7.41E-06	15
MRPS6.2	3.23E-10	1.028225617	0.298	0.169	7.43E-06	15
ATP5G2.6	3.52E-10	0.732716788	0.657	0.655	8.08E-06	15
CAPZB.6	5.35E-10	-0.516839646	0.233	0.465	1.23E-05	15

CD63.12	8.01E-10	0.547578088	0.616	0.518	1.84E-05	15
COX8A.4	8.80E-10	0.970734064	0.543	0.498	2.02E-05	15
AIF1.15	8.98E-10	-1.376449882	0.094	0.262	2.06E-05	15
PKM.8	1.05E-09	0.875889414	0.535	0.432	2.41E-05	15
H2AFV.2	1.35E-09	-0.521577798	0.106	0.291	3.11E-05	15
EIF1AX.3	1.36E-09	-0.553217704	0.122	0.316	3.12E-05	15
CSTB.8	1.44E-09	0.906512988	0.49	0.396	3.32E-05	15
VKORC1.2	1.45E-09	1.083962922	0.314	0.193	3.32E-05	15
COA4.1	1.64E-09	0.947194709	0.253	0.137	3.78E-05	15
C9orf78.6	1.77E-09	-0.580853407	0.09	0.268	4.07E-05	15
GYPC.10	1.88E-09	-0.549745508	0.122	0.31	4.32E-05	15
GPX4.10	2.22E-09	0.868773501	0.535	0.452	5.10E-05	15
ANXA1.13	2.69E-09	-1.038008904	0.286	0.475	6.18E-05	15
CHMP2A.6	2.77E-09	0.882117288	0.42	0.314	6.38E-05	15
CDC37.5	3.36E-09	-0.516580206	0.122	0.312	7.71E-05	15
HMGN3.3	3.52E-09	1.073217121	0.408	0.304	8.09E-05	15
ZCRB1.1	5.21E-09	0.985704709	0.298	0.184	0.000119813	15
C19orf70.1	5.74E-09	1.030264337	0.367	0.255	0.000131927	15
TAGLN2.10	5.96E-09	0.798892296	0.461	0.355	0.00013705	15
PRR13.3	5.98E-09	0.985578349	0.473	0.39	0.00013732	15
LYZ.13	6.77E-09	-1.979697583	0.188	0.354	0.000155649	15
IGKC.11	6.80E-09	-3.461806317	0.118	0.299	0.000156376	15
TIMM13.1	6.96E-09	0.891860139	0.314	0.2	0.000159851	15
CD151.3	8.10E-09	0.951197125	0.318	0.199	0.000186214	15
VAMP5.8	8.95E-09	-0.610308635	0.127	0.311	0.000205578	15
LINC00998.1	9.86E-09	0.872312203	0.302	0.187	0.00022659	15
RAP1B.6	1.01E-08	-0.510719148	0.143	0.327	0.00023228	15
SNRPB2.3	1.09E-08	1.029405982	0.429	0.339	0.000251298	15
NDUFA11.4	1.12E-08	0.878224707	0.473	0.393	0.000258081	15
NDUFB2.3	1.32E-08	0.850610048	0.486	0.408	0.000303124	15
MEA1	1.47E-08	0.865566826	0.273	0.162	0.00033723	15
BIRC3.13	1.75E-08	1.256766179	0.388	0.27	0.000402618	15
SPINT2.3	1.98E-08	1.106205077	0.253	0.148	0.000454611	15
CEBPB.12	2.03E-08	-0.639587519	0.151	0.332	0.00046655	15
BAX.4	2.13E-08	-0.53695768	0.131	0.311	0.00048898	15
HBB.9	2.20E-08	-2.952087328	0.192	0.395	0.00050588	15
ATP5E.9	2.31E-08	0.513369358	0.776	0.816	0.000530044	15
COX5A.4	2.46E-08	1.201357535	0.412	0.319	0.000565812	15
RNF5.1	2.75E-08	0.834296303	0.257	0.149	0.000631991	15
YWHAE.9	3.02E-08	0.907129936	0.412	0.311	0.000695085	15
C3.3	3.14E-08	0.825233164	0.257	0.139	0.000721551	15
MRPS36.1	4.16E-08	0.908910619	0.294	0.184	0.000956808	15
TCEAL4.3	4.24E-08	0.903785191	0.265	0.157	0.000975087	15
PPIB.4	7.02E-08	0.828438931	0.535	0.486	0.001612785	15
SHFM1.8	7.95E-08	0.870705837	0.486	0.405	0.001827758	15
TRAPPC2L.3	7.98E-08	0.929316898	0.286	0.179	0.00183342	15
CNDP2.3	9.15E-08	1.089637541	0.314	0.211	0.002101959	15
GLUL.11	1.02E-07	-0.658727702	0.147	0.309	0.00234989	15
TIMM8B	1.24E-07	0.889452524	0.31	0.205	0.002843053	15
NDUFC2.5	1.56E-07	0.959697504	0.384	0.294	0.00359312	15
COA3.1	1.60E-07	0.815269184	0.257	0.156	0.003667568	15
EDF1.4	2.13E-07	0.830620845	0.555	0.545	0.004884678	15
CEBPD.10	2.17E-07	-0.717994008	0.127	0.282	0.004991668	15
CWC15	2.45E-07	0.9509618	0.318	0.217	0.005640203	15
NDUFB5.6	3.14E-07	0.98116039	0.343	0.247	0.007212442	15
TRMT112.5	3.85E-07	0.813776671	0.478	0.42	0.008843083	15
ECH1.5	5.28E-07	0.999043804	0.404	0.314	0.012125338	15
MORF4L2.3	5.74E-07	0.923285952	0.278	0.178	0.013195693	15

POLR2J.2	6.69E-07	1.003720301	0.347	0.252	0.015379695	15
ATP6V1F.9	7.32E-07	0.738513252	0.429	0.348	0.016823035	15
TMEM258.4	7.38E-07	0.929636191	0.518	0.497	0.016963714	15
EIF4EBP1.1	7.89E-07	0.687625269	0.261	0.164	0.018136768	15
MZT2B.5	8.69E-07	0.884928216	0.437	0.36	0.019977353	15
ATP5L.6	1.08E-06	0.634888152	0.665	0.675	0.024792319	15
CAMTA1.1	1.18E-06	0.878319556	0.286	0.193	0.027163298	15
CD68.4	1.31E-06	0.576233694	0.306	0.194	0.030078897	15
PGAM1.7	1.74E-06	0.747023625	0.429	0.359	0.03995159	15
GUK1.4	1.76E-06	0.795676298	0.535	0.506	0.040474914	15
SRP14.4	1.79E-06	0.5344239	0.673	0.716	0.041105188	15
EIF5A.5	1.86E-06	0.572472388	0.327	0.228	0.042722474	15
TMEM230.2	2.16E-06	1.082413662	0.392	0.327	0.049599718	15
JCHAIN	0	7.010665981	0.739	0.033	0	16
IGHGP	0	5.068958315	0.365	0.008	0	16
CD79A	0	2.292154138	0.374	0.009	0	16
IGHG4	7.51E-280	6.642453192	0.517	0.035	1.72E-275	16
IGHG3	1.42E-250	7.594461463	0.569	0.049	3.26E-246	16
IGHG1	2.09E-212	6.273842686	0.573	0.061	4.81E-208	16
MZB1	3.81E-194	2.587007138	0.341	0.021	8.75E-190	16
IGHM	3.15E-161	4.881207505	0.251	0.013	7.23E-157	16
IGLC3	1.27E-130	8.071569639	0.412	0.049	2.92E-126	16
IGLC2.1	1.61E-96	8.332413104	0.464	0.084	3.71E-92	16
IGKC.12	3.04E-65	8.300476953	0.682	0.29	6.98E-61	16
IGHA1.2	4.15E-43	7.027222881	0.37	0.101	9.53E-39	16
B2M.12	2.35E-42	-0.836145866	0.995	0.988	5.40E-38	16
HLA-A.12	1.32E-38	-1.206849925	0.692	0.897	3.04E-34	16
GAPDH.10	3.86E-33	-1.356189861	0.507	0.872	8.87E-29	16
HLA-B.13	3.01E-30	-0.864524202	0.758	0.916	6.92E-26	16
IL32.13	4.94E-30	-2.080321327	0.19	0.584	1.14E-25	16
TMSB4X.8	9.49E-30	-0.802861703	0.995	0.976	2.18E-25	16
VIM.13	5.75E-29	-1.336515075	0.441	0.776	1.32E-24	16
HCST.12	7.76E-29	-1.388630686	0.128	0.551	1.78E-24	16
ID2.11	4.51E-28	-1.464300287	0.156	0.57	1.04E-23	16
FYB.14	3.58E-26	-1.372602762	0.076	0.466	8.22E-22	16
TMSB10.10	8.14E-26	-0.819444486	0.9	0.961	1.87E-21	16
CD2.15	8.57E-25	-1.682002927	0.085	0.463	1.97E-20	16
MYL6.7	4.59E-24	-0.934928033	0.493	0.801	1.05E-19	16
ARPC2.10	9.60E-24	-1.184696527	0.313	0.643	2.21E-19	16
ACTG1.8	1.67E-23	-1.017005385	0.412	0.75	3.83E-19	16
CD3E.15	7.00E-23	-1.468060903	0.057	0.403	1.61E-18	16
CD3D.16	9.12E-23	-1.593949846	0.133	0.48	2.10E-18	16
ITM2B.11	1.10E-22	-0.924219947	0.483	0.784	2.53E-18	16
PTPRC.14	1.12E-22	-1.179784247	0.275	0.622	2.58E-18	16
UQCR11.4	1.90E-22	-1.035573289	0.261	0.615	4.37E-18	16
TNFAIP3.15	2.07E-22	-1.560719546	0.114	0.452	4.75E-18	16
ACTB.11	2.69E-22	-0.799576113	0.844	0.93	6.19E-18	16
ITGB1.9	7.55E-22	-1.431626938	0.133	0.469	1.73E-17	16
CCL5.15	8.57E-22	-1.92774585	0.256	0.566	1.97E-17	16
S100A10.9	1.96E-21	-1.201497134	0.265	0.615	4.51E-17	16
GIMAP4.11	1.97E-21	-1.314635544	0.019	0.343	4.52E-17	16
HLA-C.12	3.40E-21	-0.698835822	0.782	0.884	7.81E-17	16
ATP5E.10	4.39E-21	-0.870127455	0.555	0.82	1.01E-16	16
COX6C.5	8.58E-21	-1.02432887	0.232	0.572	1.97E-16	16
AAK1.15	3.73E-20	-0.961434698	0.057	0.375	8.57E-16	16
SRP14.5	4.57E-20	-0.926064518	0.417	0.72	1.05E-15	16
GZMA.15	5.19E-20	-1.593430883	0.104	0.424	1.19E-15	16
SSR4.2	5.73E-20	1.900409007	0.649	0.531	1.32E-15	16

ITGB2.13	9.33E-20	-1.021949925	0.095	0.42	2.14E-15	16
CST7.14	1.80E-19	-1.681891103	0.085	0.394	4.13E-15	16
MYL12A.7	1.83E-19	-0.911849805	0.374	0.695	4.20E-15	16
SKP1.3	2.37E-19	-1.000819328	0.28	0.608	5.44E-15	16
SUMO2.4	3.11E-19	-0.881460425	0.299	0.612	7.14E-15	16
MYL12B.4	4.07E-19	-0.786626325	0.299	0.64	9.35E-15	16
RARRES3.11	1.13E-18	-1.264044341	0.109	0.415	2.59E-14	16
ENO1.13	4.32E-18	-1.242148588	0.294	0.598	9.92E-14	16
PSMA7.6	6.24E-18	-0.933554006	0.213	0.528	1.43E-13	16
GUK1.5	1.15E-17	-0.678875274	0.185	0.512	2.64E-13	16
HSP90AA1.9	1.19E-17	-1.114925857	0.37	0.658	2.73E-13	16
NDUFS5.6	1.25E-17	-0.761521648	0.204	0.537	2.86E-13	16
CD99.11	1.66E-17	-0.804859078	0.166	0.475	3.82E-13	16
ARL6IP5.11	3.10E-17	-0.93877803	0.166	0.462	7.13E-13	16
CCL4.16	4.05E-17	-2.257186226	0.161	0.444	9.30E-13	16
HSPA8.9	4.36E-17	-0.847236092	0.261	0.559	1.00E-12	16
HMGB1.10	4.72E-17	-0.785868123	0.431	0.739	1.09E-12	16
YWHAB.8	5.41E-17	-0.643618095	0.175	0.492	1.24E-12	16
HLA-E.14	5.44E-17	-0.782734388	0.493	0.794	1.25E-12	16
SON.11	5.65E-17	-0.809251225	0.265	0.574	1.30E-12	16
HMGN2.4	6.20E-17	-1.030636762	0.199	0.505	1.43E-12	16
COX5B.6	6.92E-17	-0.859759799	0.246	0.551	1.59E-12	16
TMA7.9	9.29E-17	-0.772965376	0.408	0.7	2.14E-12	16
RPS24.9	1.35E-16	-0.586536013	0.777	0.915	3.10E-12	16
GZMK.16	1.99E-16	-2.053948966	0.1	0.369	4.58E-12	16
TPM3.6	2.30E-16	-1.071134884	0.171	0.458	5.29E-12	16
PIK3R1.13	2.32E-16	-1.126953415	0.043	0.312	5.33E-12	16
GIMAP7.14	2.39E-16	-1.062763372	0.024	0.288	5.50E-12	16
ITM2A.15	3.38E-16	-1.521586706	0.052	0.314	7.77E-12	16
CTSD.9	4.35E-16	-1.034058035	0.161	0.441	9.99E-12	16
TRBC2.15	4.45E-16	-1.563605869	0.118	0.381	1.02E-11	16
CD3G.15	4.79E-16	-1.106637008	0.033	0.297	1.10E-11	16
PRKCH.11	7.02E-16	-1.066163223	0.009	0.257	1.61E-11	16
RGS1.16	7.13E-16	-1.196056836	0.46	0.7	1.64E-11	16
DUSP2.15	8.65E-16	-1.572416946	0.166	0.435	1.99E-11	16
PIM2.1	9.06E-16	1.163996499	0.251	0.093	2.08E-11	16
TRAC.16	1.31E-15	-1.104596623	0.142	0.436	3.01E-11	16
USMG5.6	1.43E-15	-0.737514825	0.232	0.533	3.28E-11	16
PRMT2.9	1.52E-15	-0.980403265	0.038	0.293	3.50E-11	16
COMMD6.7	1.68E-15	-0.77000849	0.308	0.61	3.85E-11	16
LINC00152.9	2.02E-15	-1.102026425	0.062	0.321	4.64E-11	16
RNF213.11	2.03E-15	-1.052170656	0.123	0.391	4.67E-11	16
S100A11.13	2.45E-15	-0.865744884	0.294	0.595	5.64E-11	16
CD7.13	3.10E-15	-1.24628965	0.047	0.304	7.12E-11	16
CALM1.8	3.35E-15	-0.796363541	0.303	0.6	7.69E-11	16
S100A4.14	3.40E-15	-0.82070478	0.436	0.703	7.81E-11	16
HNRNPA1.6	3.62E-15	-0.735330225	0.512	0.781	8.31E-11	16
SLA.13	3.84E-15	-0.771666271	0.128	0.403	8.82E-11	16
ATP5I.3	3.94E-15	-0.893842656	0.242	0.524	9.06E-11	16
LCK.16	4.09E-15	-1.033653992	0.043	0.291	9.40E-11	16
TPI1.10	4.77E-15	-0.944476425	0.28	0.569	1.10E-10	16
VAMP8.9	5.70E-15	-0.656968201	0.18	0.465	1.31E-10	16
CFL1.5	6.30E-15	-0.605906783	0.512	0.75	1.45E-10	16
ANXA1.14	7.80E-15	-0.761868532	0.166	0.476	1.79E-10	16
FYN.11	1.05E-14	-1.201717695	0.033	0.269	2.41E-10	16
RAC1.9	1.07E-14	-0.968900835	0.185	0.452	2.46E-10	16
ARPC1B.8	1.10E-14	-0.783881108	0.242	0.529	2.53E-10	16
RHOA.11	1.13E-14	-0.889201291	0.256	0.512	2.60E-10	16

APOE.10	1.28E-14	-2.583647675	0.194	0.442	2.95E-10	16
NUCKS1.5	1.51E-14	-0.896470216	0.133	0.402	3.46E-10	16
IFITM3.14	2.00E-14	-1.536251733	0.128	0.382	4.60E-10	16
LDHA.13	2.25E-14	-1.152148132	0.303	0.583	5.18E-10	16
SHFM1.9	2.26E-14	-0.947760952	0.142	0.411	5.19E-10	16
JAK1.10	2.44E-14	-1.018907062	0.137	0.399	5.61E-10	16
PFN1.10	3.18E-14	-0.592258255	0.517	0.764	7.31E-10	16
SH3BGRL3.12	3.21E-14	-0.748644369	0.436	0.686	7.37E-10	16
ATP5J2.4	3.31E-14	-0.831275517	0.175	0.436	7.61E-10	16
SYNE2.15	3.35E-14	-0.940728777	0.085	0.336	7.70E-10	16
SERF2.6	3.40E-14	-0.661032465	0.616	0.821	7.81E-10	16
CLEC2B.9	3.49E-14	-0.62518188	0.104	0.382	8.01E-10	16
PSME1.5	3.52E-14	-0.624818936	0.308	0.585	8.09E-10	16
MSN.9	3.64E-14	-0.812772425	0.114	0.37	8.36E-10	16
GSTK1.4	3.78E-14	-0.692934072	0.137	0.407	8.68E-10	16
CD96.16	4.16E-14	-0.999517618	0.024	0.252	9.57E-10	16
MYO1F.11	4.43E-14	-0.781152934	0.038	0.275	1.02E-09	16
HMGB2.11	4.77E-14	-0.756508494	0.204	0.48	1.10E-09	16
UBL5.5	4.98E-14	-0.799627567	0.303	0.566	1.14E-09	16
LCP1.13	5.23E-14	-0.753163097	0.147	0.408	1.20E-09	16
IFI16.6	5.50E-14	-0.682263862	0.194	0.485	1.26E-09	16
SOD1.9	5.77E-14	-0.779578211	0.213	0.486	1.32E-09	16
PSMB8.8	6.89E-14	-0.832810825	0.128	0.385	1.58E-09	16
BTG1.15	7.00E-14	-0.686604479	0.635	0.846	1.61E-09	16
TRBC1.15	7.59E-14	-1.23771345	0.1	0.357	1.74E-09	16
SARAF.8	7.65E-14	-0.612140432	0.422	0.682	1.76E-09	16
FKBP5.10	8.19E-14	-0.857974418	0.147	0.399	1.88E-09	16
SEPW1.12	8.71E-14	-0.962501904	0.114	0.363	2.00E-09	16
DNAJB1.12	1.01E-13	-1.055879279	0.156	0.424	2.32E-09	16
ATP5G2.7	1.06E-13	-0.61363879	0.398	0.659	2.43E-09	16
HNRNPC.5	1.13E-13	-0.786206737	0.213	0.48	2.60E-09	16
DYNLT1.4	1.21E-13	-0.816981168	0.085	0.335	2.79E-09	16
CAPZB.7	1.55E-13	-0.537046558	0.204	0.465	3.55E-09	16
CTSC.13	1.85E-13	-0.785032794	0.104	0.35	4.26E-09	16
TPT1.11	1.96E-13	-0.515649743	0.834	0.925	4.49E-09	16
TSPO.8	2.05E-13	-0.620405595	0.166	0.424	4.70E-09	16
EVL.15	2.27E-13	-0.701717554	0.227	0.499	5.21E-09	16
CLIC1.5	3.36E-13	-0.576966312	0.36	0.636	7.73E-09	16
UBB.7	3.61E-13	-0.621935291	0.469	0.723	8.31E-09	16
RHOC.10	3.64E-13	-0.903329009	0.085	0.327	8.37E-09	16
LDHB.9	3.69E-13	-0.840265452	0.213	0.47	8.47E-09	16
SUB1.5	3.72E-13	-0.603366605	0.294	0.578	8.56E-09	16
NDUFA3.6	4.49E-13	-0.679363851	0.142	0.395	1.03E-08	16
IGFBP7.13	4.64E-13	-2.64763262	0.142	0.382	1.07E-08	16
MBNL1.9	4.78E-13	-0.823037103	0.114	0.359	1.10E-08	16
DYNLRB1.6	5.26E-13	-1.002170864	0.095	0.325	1.21E-08	16
UQCRB.6	5.41E-13	-0.624855117	0.474	0.748	1.24E-08	16
GLRX.9	5.94E-13	-0.863917106	0.071	0.301	1.36E-08	16
GNAS.7	6.49E-13	-0.843681912	0.109	0.348	1.49E-08	16
NEDD8.3	6.58E-13	-0.634115404	0.175	0.435	1.51E-08	16
COX7C.6	7.27E-13	-0.646303592	0.521	0.757	1.67E-08	16
IL7R.14	7.27E-13	-1.607376744	0.076	0.307	1.67E-08	16
ALDOA.7	7.56E-13	-0.887007205	0.389	0.641	1.74E-08	16
NKG7.16	8.40E-13	-1.61310822	0.194	0.431	1.93E-08	16
GBP2.7	9.18E-13	-0.772241357	0.043	0.266	2.11E-08	16
COX8A.5	9.54E-13	-0.617589114	0.246	0.503	2.19E-08	16
COX6A1.5	9.61E-13	-0.646671498	0.246	0.523	2.21E-08	16
PSMB9.7	1.04E-12	-0.688914952	0.227	0.488	2.39E-08	16

RBPJ.10	1.08E-12	-0.926747676	0.09	0.325	2.48E-08	16
PA2G4.1	1.11E-12	-0.860963052	0.137	0.382	2.55E-08	16
AKAP13.11	1.11E-12	-0.796390043	0.19	0.436	2.56E-08	16
HSPB1.14	1.13E-12	-1.247579823	0.199	0.438	2.61E-08	16
NDUFB8.2	1.33E-12	-0.8507217	0.147	0.381	3.06E-08	16
HLA-F.11	1.34E-12	-0.664691458	0.19	0.442	3.08E-08	16
SYTL3.14	1.42E-12	-0.805274984	0.052	0.28	3.26E-08	16
RTN4.12	1.54E-12	-0.677937427	0.109	0.354	3.53E-08	16
CD44.11	1.54E-12	-0.864745473	0.246	0.495	3.54E-08	16
PPIA.6	1.89E-12	-0.559102833	0.403	0.651	4.35E-08	16
DDIT4.10	2.19E-12	-1.055804734	0.256	0.522	5.02E-08	16
BSG.7	2.36E-12	-0.873360741	0.104	0.332	5.42E-08	16
WIPF1.14	2.42E-12	-0.732871901	0.114	0.347	5.56E-08	16
JUN.10	2.63E-12	-0.987006371	0.441	0.695	6.04E-08	16
LEPROTL1.8	2.68E-12	-0.601141288	0.142	0.393	6.16E-08	16
SEPT7.8	2.69E-12	-0.624600368	0.242	0.517	6.18E-08	16
MCL1.9	2.77E-12	-0.563643197	0.256	0.518	6.36E-08	16
NDUFC2.6	2.89E-12	-0.809123022	0.076	0.299	6.63E-08	16
MIF.13	2.90E-12	-1.120680226	0.251	0.509	6.67E-08	16
PGK1.10	2.97E-12	-0.546634873	0.199	0.454	6.82E-08	16
ARHGDIB.9	3.04E-12	-0.538114342	0.427	0.676	6.98E-08	16
HSPE1.8	3.15E-12	-0.770922833	0.137	0.387	7.25E-08	16
PSMB1.4	3.18E-12	-0.538775029	0.171	0.428	7.31E-08	16
UBE2L3.5	3.21E-12	-0.90537734	0.09	0.319	7.37E-08	16
CAP1.6	3.77E-12	-0.65046843	0.171	0.415	8.66E-08	16
XRCC5.5	3.82E-12	-0.795648681	0.1	0.328	8.77E-08	16
GSTO1.7	3.97E-12	-0.88582241	0.1	0.326	9.12E-08	16
HNRNPK.3	3.97E-12	-0.573403666	0.275	0.54	9.13E-08	16
SRSF7.13	4.04E-12	-0.77687753	0.284	0.549	9.29E-08	16
TMEM50A.5	4.15E-12	-0.672912953	0.114	0.34	9.54E-08	16
PTMA.7	4.16E-12	-0.503414624	0.744	0.92	9.56E-08	16
RBX1.4	4.42E-12	-0.710727305	0.142	0.379	1.01E-07	16
CBX3.2	5.11E-12	-0.738882326	0.114	0.348	1.17E-07	16
COX7A2.3	5.72E-12	-0.590622156	0.299	0.565	1.31E-07	16
PRPF38B.13	5.93E-12	-0.869021196	0.209	0.439	1.36E-07	16
HNRNPA2B1.7	6.16E-12	-0.638278745	0.36	0.631	1.42E-07	16
AP2M1.3	6.76E-12	-0.662996625	0.104	0.327	1.55E-07	16
FUS.11	6.85E-12	-0.557430996	0.213	0.473	1.57E-07	16
PAIP2.3	7.35E-12	-0.543876258	0.147	0.387	1.69E-07	16
SERBP1.2	7.61E-12	-0.69215844	0.171	0.407	1.75E-07	16
YWHAQ.6	7.65E-12	-0.731350119	0.123	0.352	1.76E-07	16
SCP2.5	8.05E-12	-0.642253889	0.1	0.329	1.85E-07	16
FXYD5.9	8.31E-12	-0.675309898	0.246	0.484	1.91E-07	16
COX7B.4	8.43E-12	-0.526843029	0.223	0.474	1.94E-07	16
EID1.5	8.58E-12	-0.571663273	0.171	0.432	1.97E-07	16
RGCC.14	8.59E-12	-1.087754977	0.085	0.311	1.97E-07	16
SNRPD2.6	8.62E-12	-0.565555836	0.313	0.561	1.98E-07	16
HNRNPA3.4	8.81E-12	-0.542189024	0.209	0.467	2.02E-07	16
PPP1CA.3	9.01E-12	-0.678662119	0.137	0.368	2.07E-07	16
S100A8.5	1.35E-11	0.75310011	0.37	0.208	3.10E-07	16
C12orf57.9	1.52E-11	-0.771045762	0.147	0.375	3.48E-07	16
ERH.3	1.57E-11	-0.60725473	0.133	0.364	3.61E-07	16
PKM.9	1.58E-11	-0.72582327	0.194	0.437	3.63E-07	16
WNK1.9	1.60E-11	-0.815573726	0.047	0.252	3.67E-07	16
HSP90AB1.7	1.68E-11	-0.748841809	0.374	0.605	3.86E-07	16
TXN.11	1.84E-11	-0.948319127	0.166	0.392	4.23E-07	16
KIF5B.4	1.87E-11	-0.578830136	0.071	0.287	4.29E-07	16
H2AFZ.3	2.06E-11	-0.741995139	0.166	0.393	4.73E-07	16

VAMP5.9	2.16E-11	-0.629244802	0.09	0.311	4.96E-07	16
OST4.5	2.28E-11	-0.527384314	0.393	0.664	5.24E-07	16
PIK3IP1.15	2.68E-11	-0.82688938	0.137	0.361	6.16E-07	16
PTP4A2.8	2.81E-11	-0.798678764	0.066	0.274	6.46E-07	16
ODF2L.10	2.88E-11	-0.985766718	0.062	0.262	6.62E-07	16
CARD16.10	3.67E-11	-0.541893156	0.1	0.321	8.42E-07	16
HP1BP3.6	4.13E-11	-0.794189649	0.081	0.288	9.48E-07	16
ARF1.2	4.62E-11	-0.661003691	0.18	0.412	1.06E-06	16
SH3KBP1.12	4.69E-11	-0.639016941	0.095	0.311	1.08E-06	16
CCNH.11	5.34E-11	-0.782438363	0.081	0.287	1.23E-06	16
RAB7A.5	5.63E-11	-0.610502758	0.1	0.318	1.29E-06	16
BLOC1S1.7	6.29E-11	-0.558823445	0.128	0.35	1.45E-06	16
COX6B1.2	6.30E-11	-0.502962857	0.327	0.577	1.45E-06	16
GPX4.11	6.62E-11	-0.876391026	0.237	0.457	1.52E-06	16
CKLF.9	6.90E-11	-0.520281504	0.137	0.367	1.59E-06	16
MORF4L1.2	6.98E-11	-0.674738554	0.308	0.539	1.61E-06	16
VMP1.10	7.44E-11	-0.633188664	0.104	0.323	1.71E-06	16
PLIN2.12	7.61E-11	-1.045962928	0.123	0.339	1.75E-06	16
MPC2.10	7.91E-11	-0.889072745	0.066	0.267	1.82E-06	16
CSNK1A1.3	8.27E-11	-0.621903785	0.09	0.307	1.90E-06	16
BAX.5	9.31E-11	-0.577624043	0.095	0.311	2.14E-06	16
S100A9.14	9.32E-11	1.322290433	0.408	0.249	2.14E-06	16
UGP2.10	9.94E-11	-0.624904477	0.109	0.329	2.28E-06	16
MACF1.10	1.00E-10	-0.613273069	0.057	0.26	2.31E-06	16
CEBPD.11	1.19E-10	-0.88514782	0.081	0.282	2.73E-06	16
HNRNPF.1	1.20E-10	-0.518230327	0.137	0.358	2.76E-06	16
UQCR10.3	1.23E-10	-0.6665039	0.218	0.44	2.82E-06	16
PPP1R12A.7	1.37E-10	-0.627410189	0.076	0.282	3.14E-06	16
TLN1.5	1.40E-10	-0.591639191	0.066	0.268	3.22E-06	16
CALM2.5	1.40E-10	-0.546556953	0.403	0.634	3.23E-06	16
GPX1.12	1.66E-10	-1.105535409	0.213	0.432	3.82E-06	16
HNRNPU.5	1.81E-10	-0.741708119	0.161	0.377	4.15E-06	16
RBM25.8	2.07E-10	-0.568306484	0.142	0.363	4.77E-06	16
ETS1.14	2.08E-10	-0.71491209	0.128	0.342	4.78E-06	16
ALOX5AP.12	2.17E-10	-0.988767972	0.128	0.332	4.98E-06	16
RAN.4	2.19E-10	-0.719655424	0.213	0.429	5.03E-06	16
PSME2.6	2.29E-10	-0.586134602	0.194	0.416	5.26E-06	16
RGS10.10	2.35E-10	-0.588554138	0.095	0.298	5.40E-06	16
CELF2.12	2.39E-10	-0.639133841	0.204	0.429	5.48E-06	16
PEBP1.12	2.42E-10	-0.7373709	0.171	0.393	5.57E-06	16
EMB.12	2.77E-10	-0.735614065	0.076	0.272	6.37E-06	16
MYH9.10	2.79E-10	-0.742992126	0.109	0.314	6.42E-06	16
APOBEC3G.13	3.08E-10	-0.9903988	0.085	0.274	7.09E-06	16
NDUFA4L2.15	3.17E-10	-2.115207211	0.1	0.291	7.29E-06	16
SNRPB2.4	3.21E-10	-0.589684546	0.133	0.343	7.38E-06	16
NFKBIA.14	3.61E-10	-0.913218426	0.336	0.561	8.30E-06	16
YWHAH.13	3.65E-10	-1.115694774	0.095	0.286	8.38E-06	16
GABARAPL2.4	3.71E-10	-0.510597405	0.133	0.351	8.53E-06	16
LIMS1.11	3.72E-10	-0.591730998	0.062	0.252	8.56E-06	16
MINOS1.5	4.08E-10	-0.616852107	0.171	0.389	9.38E-06	16
SDCBP.13	4.29E-10	-0.563079601	0.156	0.376	9.85E-06	16
SNRPG.4	4.60E-10	-0.619119593	0.147	0.361	1.06E-05	16
ROCK1.9	4.74E-10	-0.645797452	0.128	0.334	1.09E-05	16
RANBP1.2	4.92E-10	-0.559534154	0.076	0.272	1.13E-05	16
GCC2.10	5.04E-10	-0.744947065	0.147	0.363	1.16E-05	16
IDS.11	5.10E-10	-0.677967748	0.161	0.376	1.17E-05	16
PCBP1.4	5.38E-10	-0.606662471	0.204	0.422	1.24E-05	16
NDUFC1.11	6.44E-10	-1.177936605	0.085	0.274	1.48E-05	16

CSTB.9	6.59E-10	-0.619827618	0.18	0.401	1.51E-05	16
BIN2.11	6.68E-10	-0.579095334	0.062	0.252	1.53E-05	16
NDUFA13.4	6.78E-10	-0.502666681	0.227	0.454	1.56E-05	16
DHRS7.7	6.90E-10	-0.77114685	0.095	0.278	1.59E-05	16
SEC11A.7	6.95E-10	-0.629070549	0.142	0.342	1.60E-05	16
DDX24.12	7.24E-10	-0.523658371	0.237	0.46	1.66E-05	16
MGST3.12	7.71E-10	-0.813165863	0.114	0.309	1.77E-05	16
FKBP1A.10	8.31E-10	-0.675424802	0.194	0.416	1.91E-05	16
HCLS1.10	8.75E-10	-0.668697603	0.133	0.325	2.01E-05	16
CALM3.6	9.02E-10	-0.586443775	0.109	0.316	2.07E-05	16
LGALS1.7	9.34E-10	-0.949029409	0.365	0.575	2.15E-05	16
C9orf78.7	9.40E-10	-0.537104516	0.076	0.268	2.16E-05	16
DNAJC8.2	9.71E-10	-0.714758723	0.104	0.299	2.23E-05	16
KTN1.9	1.01E-09	-0.539179785	0.171	0.386	2.31E-05	16
RPS27L.9	1.06E-09	-0.599539827	0.242	0.476	2.44E-05	16
ACTR2.8	1.13E-09	-0.728491372	0.166	0.37	2.61E-05	16
PPP2R5C.12	1.15E-09	-0.659351927	0.175	0.393	2.64E-05	16
CIB1.2	1.22E-09	-0.527917189	0.175	0.391	2.80E-05	16
HMGN1.4	1.22E-09	-0.509944587	0.289	0.507	2.81E-05	16
DYNLL1.8	1.37E-09	-0.561222358	0.204	0.412	3.14E-05	16
DNAJA1.6	1.39E-09	-0.709807735	0.133	0.335	3.20E-05	16
SAP18.3	1.40E-09	-0.505076079	0.237	0.465	3.21E-05	16
SSBP1.3	1.46E-09	-0.703095658	0.1	0.296	3.36E-05	16
SH3GLB1.6	1.51E-09	-0.589425325	0.085	0.271	3.46E-05	16
EIF3H.3	1.59E-09	-0.521060185	0.232	0.454	3.66E-05	16
CST3.15	1.85E-09	-1.407274941	0.199	0.388	4.24E-05	16
VPS28.1	1.94E-09	-0.627446232	0.147	0.349	4.46E-05	16
AHNAK.6	1.99E-09	-0.563574715	0.104	0.297	4.57E-05	16
SRI.3	2.13E-09	-0.764699914	0.133	0.323	4.89E-05	16
CHURC1.7	2.16E-09	-0.635465651	0.085	0.275	4.96E-05	16
GNAI2.11	2.25E-09	-0.626213158	0.081	0.263	5.18E-05	16
NDUFB9.3	2.27E-09	-0.603642986	0.137	0.335	5.21E-05	16
BUB3.11	2.27E-09	-0.829927084	0.095	0.274	5.21E-05	16
RNF149.12	2.27E-09	-0.542349398	0.085	0.272	5.21E-05	16
C14orf166.4	2.57E-09	-0.56714752	0.133	0.335	5.91E-05	16
LSP1.15	2.65E-09	-0.608252488	0.232	0.437	6.10E-05	16
DDX3X.12	2.67E-09	-0.671225914	0.209	0.418	6.12E-05	16
PSMA4.4	2.74E-09	-0.725681288	0.128	0.327	6.30E-05	16
ROMO1.7	2.90E-09	-0.740754388	0.123	0.314	6.65E-05	16
IQGAP1.8	2.98E-09	-0.608528485	0.142	0.341	6.84E-05	16
NCL.6	3.03E-09	-0.518794982	0.223	0.434	6.97E-05	16
RWDD1.1	3.57E-09	-0.613925622	0.1	0.285	8.21E-05	16
TBCA.8	3.69E-09	-0.703294188	0.204	0.406	8.49E-05	16
ATP5J.9	3.89E-09	-0.504882023	0.185	0.398	8.95E-05	16
TCF25.5	3.97E-09	-0.500898728	0.199	0.417	9.12E-05	16
SRSF11.6	4.01E-09	-0.602811131	0.171	0.379	9.22E-05	16
ANXA5.10	4.10E-09	-0.779726401	0.166	0.355	9.42E-05	16
NKTR.11	4.13E-09	-0.58488827	0.147	0.35	9.50E-05	16
TUBA1B.10	4.28E-09	-0.665361687	0.227	0.417	9.83E-05	16
TMEM219.8	4.85E-09	-0.675162275	0.095	0.273	0.000111525	16
RBM8A.3	4.92E-09	-0.711939715	0.171	0.372	0.000113122	16
STAT3.11	4.95E-09	-0.572421969	0.128	0.321	0.000113805	16
SRP9.2	4.97E-09	-0.630111621	0.128	0.315	0.000114277	16
LY6E.8	5.66E-09	-0.666115997	0.175	0.368	0.000130133	16
DDX46.5	5.81E-09	-0.526120699	0.123	0.318	0.000133621	16
APOC1.13	5.85E-09	-1.937670704	0.114	0.286	0.000134333	16
PRRC2C.9	6.14E-09	-0.519402596	0.218	0.437	0.00014107	16
ANP32B.5	6.33E-09	-0.575188404	0.147	0.344	0.000145542	16

IFI27L2.4	6.64E-09	-0.517016723	0.118	0.309	0.000152635	16
POMP.8	7.05E-09	-0.579087952	0.237	0.452	0.000162098	16
ATP5F1.4	7.18E-09	-0.561667737	0.123	0.309	0.000164915	16
NDUFB4.11	7.24E-09	-0.598054587	0.213	0.414	0.000166361	16
CD63.13	9.60E-09	-0.528902189	0.294	0.523	0.000220643	16
FIS1.3	9.84E-09	-0.5058962	0.128	0.312	0.000226184	16
PRDX1.10	1.05E-08	-0.579536579	0.204	0.407	0.00024095	16
ATP6V0E1.9	1.07E-08	-0.50722838	0.313	0.521	0.000245219	16
PGAM1.8	1.12E-08	-0.565331341	0.166	0.364	0.000256303	16
COX14.2	1.16E-08	-0.688377462	0.1	0.277	0.000266414	16
TGOLN2.5	1.16E-08	-0.546496088	0.085	0.264	0.000266763	16
DNAJB6.4	1.21E-08	-0.509041817	0.147	0.343	0.000277946	16
SFPQ.7	1.24E-08	-0.566696184	0.128	0.314	0.000286093	16
MT1X.10	1.39E-08	-1.896349237	0.109	0.286	0.000318397	16
LAPTM4A.11	1.44E-08	-0.687940202	0.161	0.355	0.000331889	16
CMC1.14	1.46E-08	-1.220173239	0.114	0.287	0.000335239	16
RNF7.3	1.49E-08	-0.564116035	0.1	0.273	0.000343175	16
WSB1.7	1.88E-08	-0.529299147	0.175	0.363	0.000431404	16
HMGN3.4	1.96E-08	-0.55768789	0.128	0.308	0.000451173	16
ARID4B.12	2.06E-08	-0.549724577	0.161	0.343	0.00047406	16
HNRNPM.2	2.20E-08	-0.609734312	0.133	0.322	0.000504912	16
SF3B2.7	2.22E-08	-0.665906041	0.137	0.318	0.00050963	16
DDX17.9	2.54E-08	-0.678792645	0.204	0.402	0.000583502	16
PRELID1.5	2.72E-08	-0.53706293	0.166	0.359	0.000625637	16
CDC37.6	2.79E-08	-0.652345236	0.133	0.311	0.000641354	16
DSTN.13	2.93E-08	-0.773853499	0.128	0.31	0.00067422	16
RNF181.7	2.95E-08	-0.810073331	0.104	0.273	0.000678839	16
APLP2.13	3.05E-08	-0.740065989	0.09	0.258	0.000701288	16
YWHAE.10	3.24E-08	-0.71910668	0.142	0.315	0.00074365	16
ACAP1.16	3.27E-08	-0.726989383	0.161	0.336	0.000751842	16
GYPC.11	3.29E-08	-0.618764065	0.133	0.309	0.000754967	16
CCT8.5	3.58E-08	-0.58302343	0.095	0.264	0.000823011	16
NDUFB5.7	3.62E-08	-0.696301796	0.09	0.251	0.000830799	16
PCSK7.14	4.46E-08	-0.588848773	0.142	0.323	0.001024951	16
UBC.7	4.51E-08	-0.508018512	0.664	0.843	0.00103537	16
ACTR3.6	5.50E-08	-0.604476412	0.194	0.385	0.001263422	16
ENY2.5	6.54E-08	-0.556740637	0.128	0.307	0.00150404	16
SSB.1	7.09E-08	-0.562013062	0.104	0.27	0.0016298	16
ZFP36.10	7.12E-08	-0.736035217	0.479	0.666	0.001635386	16
ZFP36L2.13	7.57E-08	-0.718621389	0.536	0.701	0.001739794	16
C1QA.13	8.19E-08	-1.847837794	0.104	0.252	0.001882585	16
ATP1B3.12	8.50E-08	-0.55118964	0.1	0.271	0.001953402	16
AC090498.1.9	9.43E-08	-0.540822388	0.299	0.504	0.002166315	16
PHPT1.8	9.82E-08	-0.591493251	0.114	0.278	0.002257523	16
EIF3A.8	9.93E-08	-0.543561442	0.128	0.301	0.002282165	16
MT-ND4L.9	1.05E-07	-0.57699503	0.185	0.363	0.002418914	16
ARHGDIA.2	1.17E-07	-0.515946278	0.118	0.288	0.00268286	16
PPP1R2.9	1.29E-07	-0.630690423	0.171	0.345	0.002953213	16
TAOK3.9	1.63E-07	-0.603445566	0.104	0.268	0.003747418	16
KHDRBS1.1	1.71E-07	-0.619865585	0.128	0.296	0.003920667	16
ATP5B.3	1.74E-07	-0.50912274	0.19	0.372	0.003988913	16
RHOB.13	1.75E-07	-0.705877295	0.185	0.351	0.004018527	16
NFE2L2.11	1.89E-07	-0.532061328	0.104	0.266	0.004337231	16
SRRM2.6	2.01E-07	-0.578322926	0.171	0.344	0.004614217	16
STK4.12	2.16E-07	-0.654045662	0.289	0.475	0.004959635	16
RNF19A.13	2.17E-07	-0.619185407	0.118	0.279	0.004996233	16
RHOG.9	2.21E-07	-0.570014205	0.104	0.266	0.005068893	16
POLR1D.2	2.23E-07	-0.506127788	0.123	0.294	0.005114694	16

RNH1.7	2.30E-07	-0.515905309	0.114	0.273	0.005276333	16
SF3B6.6	2.58E-07	-0.708244955	0.142	0.308	0.005931208	16
NCOR1.10	2.92E-07	-0.525948725	0.175	0.35	0.006709539	16
TAGLN2.11	3.09E-07	-0.542224299	0.185	0.359	0.007095909	16
MZT2B.6	3.35E-07	-0.570020777	0.19	0.364	0.007704948	16
HERPUD1.11	3.71E-07	0.830366875	0.573	0.491	0.008532052	16
LAMTOR5.9	4.20E-07	-0.600862615	0.18	0.348	0.009649032	16
PDCD4.7	4.33E-07	-0.619399308	0.147	0.306	0.009947643	16
C9orf142.11	5.36E-07	-0.501545884	0.109	0.263	0.012315117	16
PPIG.5	5.39E-07	-0.518613407	0.156	0.323	0.012386747	16
TERF2IP.9	6.22E-07	-0.575201341	0.199	0.367	0.014284377	16
HSPD1.8	6.99E-07	-0.618619146	0.133	0.296	0.016064152	16
NNMT.16	7.26E-07	-1.304813142	0.142	0.301	0.016689846	16
ST13.10	7.76E-07	-0.518683937	0.194	0.375	0.017838875	16
TMEM256.5	7.90E-07	-0.543428948	0.109	0.256	0.018152997	16
CRIP1.12	8.00E-07	-0.722878473	0.137	0.296	0.018383006	16
UBXN1.4	9.97E-07	-0.53249558	0.223	0.394	0.022902235	16
CRYAB.15	1.14E-06	-1.516778238	0.118	0.261	0.026153055	16
XIST.10	1.15E-06	-0.613741783	0.265	0.426	0.026442289	16
NSA2.4	1.29E-06	-0.533750077	0.142	0.299	0.029628114	16
PNN.8	1.51E-06	-0.505066112	0.152	0.306	0.034809568	16
EIF1B.1	1.65E-06	-0.507379881	0.109	0.258	0.037859288	16
SQSTM1.4	1.80E-06	-0.529729011	0.161	0.316	0.041257548	16
SERPINA1.15	2.10E-06	-0.880013162	0.133	0.281	0.048288754	16
RGS5.2	0	5.206489268	1	0.114	0	17
ACTA2.1	0	4.897818295	0.979	0.123	0	17
TAGLN	0	4.539159406	0.927	0.057	0	17
BGN	0	4.092446201	0.984	0.052	0	17
CALD1.2	0	4.073561124	1	0.137	0	17
MYL9	0	3.971307016	0.969	0.064	0	17
TPM2	0	3.692570098	0.984	0.042	0	17
CPE	0	3.603918585	0.948	0.028	0	17
GJA4	0	3.546065835	0.964	0.023	0	17
FRZB	0	3.449433493	0.938	0.014	0	17
PLAC9	0	3.189027422	0.922	0.02	0	17
PDGFRB	0	3.176409679	0.917	0.011	0	17
MAP1B	0	3.144352988	0.927	0.023	0	17
CD36.1	0	3.143260366	0.881	0.041	0	17
THY1	0	3.073046674	0.855	0.035	0	17
RASD1	0	2.896172168	0.751	0.027	0	17
PLN	0	2.855506822	0.85	0.009	0	17
COL6A2	0	2.819245561	0.902	0.023	0	17
TINAGL1.2	0	2.675560286	0.896	0.042	0	17
LHFP.1	0	2.654349398	0.902	0.039	0	17
MYLK	0	2.599116818	0.85	0.029	0	17
SOD3	0	2.59347892	0.803	0.021	0	17
COL1A2	0	2.520667401	0.741	0.014	0	17
COL18A1.1	0	2.432551365	0.798	0.061	0	17
MFGE8	0	2.416483583	0.834	0.046	0	17
MYH11	0	2.411021464	0.71	0.015	0	17
PGF.1	0	2.406614579	0.86	0.042	0	17
TPPP3	0	2.353712119	0.777	0.017	0	17
ID1.1	0	2.34366488	0.777	0.06	0	17
MCAM.1	0	2.342860869	0.741	0.043	0	17
FABP4	0	2.340438159	0.404	0.007	0	17
CYGB	0	2.30870794	0.741	0.007	0	17
COX4I2	0	2.300493674	0.736	0.011	0	17
COL4A2.1	0	2.277991987	0.798	0.052	0	17

PPP1R14A		0	2.210010217	0.601	0.015	0	17
EPS8.2		0	2.193731463	0.808	0.068	0	17
KCNE4		0	2.164617157	0.746	0.006	0	17
PALLD.1		0	2.071616991	0.746	0.049	0	17
NOTCH3		0	2.04204238	0.731	0.008	0	17
CSRP2		0	1.99602543	0.679	0.018	0	17
KCNJ8		0	1.930303378	0.658	0.003	0	17
TGFB1I1		0	1.916564302	0.746	0.019	0	17
HIGD1B		0	1.9029943	0.596	0.006	0	17
GJC1		0	1.837592018	0.705	0.007	0	17
COL6A1		0	1.819529059	0.622	0.016	0	17
FILIP1.1		0	1.807401364	0.699	0.03	0	17
CRISPLD2		0	1.805886444	0.596	0.01	0	17
KANK2		0	1.786747998	0.674	0.024	0	17
DKK3.1		0	1.755568982	0.736	0.042	0	17
NR2F2		0	1.755372047	0.689	0.02	0	17
CLMN		0	1.753022818	0.674	0.019	0	17
EBF1		0	1.717768506	0.668	0.008	0	17
S1PR3		0	1.715062587	0.658	0.012	0	17
KLHL23		0	1.705946942	0.658	0.007	0	17
PLS3.1		0	1.687231171	0.663	0.027	0	17
MYO1B.1		0	1.687201037	0.632	0.024	0	17
COL3A1		0	1.666414381	0.549	0.013	0	17
VCL		0	1.661811399	0.674	0.043	0	17
C11orf96		0	1.652707899	0.637	0.011	0	17
FXYD6		0	1.637873168	0.653	0.017	0	17
COX7A1.1		0	1.63504864	0.756	0.059	0	17
NES		0	1.626205647	0.611	0.011	0	17
	4-Sep	0	1.60846715	0.575	0.013	0	17
PLXDC1		0	1.599985572	0.606	0.025	0	17
CCDC102B		0	1.593177592	0.549	0.013	0	17
NEXN		0	1.56750035	0.57	0.007	0	17
CARMN		0	1.556797181	0.513	0.006	0	17
JAG1.1		0	1.556222102	0.622	0.04	0	17
TMEM47.1		0	1.533919091	0.627	0.036	0	17
TBX2		0	1.519058746	0.544	0.006	0	17
ID4		0	1.515312047	0.508	0.003	0	17
AC013461.1		0	1.510588443	0.632	0.038	0	17
GUCY1A3		0	1.507507464	0.591	0.019	0	17
KCNAB1		0	1.457747793	0.575	0.004	0	17
CDH6		0	1.431261446	0.611	0.026	0	17
ADAMTS1		0	1.429955372	0.477	0.022	0	17
SSTR2		0	1.427217766	0.482	0.013	0	17
VASN		0	1.397218858	0.503	0.01	0	17
AKAP12.1		0	1.377505081	0.653	0.037	0	17
SLC38A11		0	1.365664257	0.492	0.002	0	17
PARM1		0	1.347443027	0.544	0.006	0	17
CNN1		0	1.341827947	0.301	0.003	0	17
EFHD1		0	1.340054843	0.528	0.005	0	17
OLFML2A		0	1.335882875	0.534	0.01	0	17
PTK2.1		0	1.313301183	0.585	0.035	0	17
DLC1		0	1.289268892	0.513	0.016	0	17
SLIT3		0	1.286876427	0.508	0.005	0	17
PTN		0	1.285047087	0.409	0.003	0	17
CHST2		0	1.27195337	0.575	0.028	0	17
FRMD3		0	1.269986456	0.513	0.019	0	17
RCAN2		0	1.244792803	0.513	0.021	0	17
GPRC5C.1		0	1.244103142	0.539	0.025	0	17

ADGRF5.1	0	1.237855786	0.617	0.036	0	17
CSPG4	0	1.222404785	0.466	0.007	0	17
GUCY1B3	0	1.220959488	0.466	0.011	0	17
AGTR1	0	1.211387819	0.347	0.001	0	17
HOXC9	0	1.199340515	0.497	0.01	0	17
FHL5	0	1.166387109	0.378	0.002	0	17
FOXS1	0	1.162295644	0.451	0.002	0	17
RERG.1	0	1.146782658	0.503	0.021	0	17
DAAM2	0	1.144068261	0.456	0.007	0	17
HEYL	0	1.078787855	0.44	0.005	0	17
MSRB3	0	1.067456727	0.44	0.014	0	17
FOXC2	0	1.051083272	0.43	0.003	0	17
LGI4	0	1.050558943	0.409	0.005	0	17
NFASC	0	1.046725429	0.415	0.004	0	17
COL5A2	0	1.031097787	0.399	0.005	0	17
NGFR	0	1.013221444	0.332	0.003	0	17
ARHGEF17	0	0.996812876	0.42	0.011	0	17
MOCS1	0	0.976446346	0.394	0.007	0	17
ACAN	0	0.963929882	0.347	0.002	0	17
PRRX1	0	0.963658329	0.347	0.001	0	17
LMOD1	0	0.956054332	0.368	0.002	0	17
RGL3	0	0.95167115	0.373	0.011	0	17
RBP1	0	0.948045782	0.399	0.013	0	17
C1QTNF1	0	0.946380185	0.378	0.006	0	17
MAP2	0	0.938656823	0.358	0.006	0	17
SELENBP1	0	0.934526142	0.337	0.007	0	17
PTGIR	0	0.930743409	0.378	0.009	0	17
ABCC9	0	0.918117038	0.378	0.001	0	17
NGF	0	0.917679484	0.358	0.001	0	17
SYDE1	0	0.915362752	0.409	0.009	0	17
NDN	0	0.912285478	0.368	0.012	0	17
LRRC17	0	0.903607335	0.29	0.002	0	17
SMOC2	0	0.864449076	0.285	0.002	0	17
SLC7A2	0	0.856534059	0.326	0.002	0	17
NR2F2-AS1	0	0.854766674	0.373	0.011	0	17
RASL12	0	0.845223508	0.332	0.001	0	17
PDGFA	0	0.842768	0.347	0.011	0	17
GUCY1A2	0	0.83939728	0.358	0.001	0	17
LURAP1L	0	0.837106685	0.332	0.009	0	17
PRKG1	0	0.819124559	0.332	0.002	0	17
TCF21	0	0.816452669	0.259	0.001	0	17
MRVI1	0	0.80816351	0.368	0.005	0	17
C9orf47	0	0.806833054	0.285	0.001	0	17
PDE5A	0	0.804290826	0.311	0.002	0	17
TBX2-AS1	0	0.801009188	0.347	0.002	0	17
LZTS1	0	0.795024743	0.352	0.004	0	17
ITGA7	0	0.77720912	0.295	0.003	0	17
FBLN1	0	0.773763491	0.285	0.001	0	17
SGIP1	0	0.762322625	0.254	0.005	0	17
CCDC3	0	0.760020223	0.306	0.004	0	17
GPR176	0	0.757197139	0.306	0.003	0	17
EDNRA	0	0.746767413	0.254	0.002	0	17
DDR2	0	0.736069529	0.295	0.004	0	17
TBX3	0	0.731256154	0.259	0.001	0	17
ECM1	0	0.722761358	0.29	0.004	0	17
ANO1	0	0.715251902	0.28	0.001	0	17
CD248	0	0.700431626	0.264	0.004	0	17
HTR1F	0	0.700358969	0.311	0.002	0	17

SEMA5A	0	0.699227597	0.321	0.003	0	17
HEY2	0	0.689709627	0.316	0.005	0	17
TUSC3	0	0.685331291	0.311	0.007	0	17
ANGPT1	0	0.665576994	0.269	0.003	0	17
ADGRA2	0	0.658365804	0.285	0.005	0	17
EBF2	0	0.649023623	0.28	0.001	0	17
ECM2	0	0.646073797	0.259	0.002	0	17
PRR16	0	0.631969132	0.259	0.002	0	17
NEURL1B	0	0.624543335	0.285	0.004	0	17
PDE1A	0	0.620511563	0.254	0.002	0	17
FAM162B	0	0.616597545	0.264	0.001	0	17
RNF180	0	0.613220856	0.275	0.004	0	17
ISYNA1	1.57E-307	1.225273137	0.482	0.023	3.62E-303	17
EPB41L1	3.22E-307	0.70388595	0.29	0.007	7.40E-303	17
ID3.2	2.94E-304	3.537491712	0.984	0.128	6.76E-300	17
CDC42EP4	1.06E-301	0.921464491	0.415	0.017	2.44E-297	17
COL4A1.1	5.38E-300	2.250922237	0.736	0.059	1.24E-295	17
SNAI2	3.94E-297	0.666005482	0.254	0.005	9.05E-293	17
CAV2.2	5.63E-294	1.722595208	0.767	0.067	1.29E-289	17
CPM.3	3.99E-289	2.94235123	0.948	0.125	9.16E-285	17
MGP.3	6.13E-288	3.703695021	0.995	0.138	1.41E-283	17
SCGN	6.88E-286	1.117670734	0.301	0.009	1.58E-281	17
CNN3.4	3.22E-283	2.01948805	0.896	0.097	7.39E-279	17
ACTN1.1	9.46E-283	1.909659844	0.762	0.073	2.17E-278	17
LGALS3BP.2	2.29E-282	2.285315464	0.834	0.092	5.25E-278	17
FILIP1L.2	2.78E-282	1.954920183	0.746	0.07	6.40E-278	17
PCOLCE	9.50E-267	0.87682072	0.337	0.012	2.18E-262	17
CSRP1	2.77E-265	2.31253275	0.824	0.094	6.37E-261	17
FERMT2.1	8.01E-264	1.106851099	0.497	0.03	1.84E-259	17
ENAH	6.97E-261	0.830247694	0.358	0.015	1.60E-256	17
PARVA	3.99E-256	0.823023152	0.399	0.019	9.18E-252	17
HES4.1	2.71E-252	1.151618694	0.56	0.039	6.23E-248	17
EPAS1.3	8.85E-250	1.752416053	0.891	0.104	2.03E-245	17
UACA.1	1.51E-249	1.713194146	0.679	0.062	3.47E-245	17
PTPRG.1	3.27E-247	1.075370306	0.466	0.027	7.51E-243	17
PHLDA1.1	7.36E-247	2.488728425	0.943	0.136	1.69E-242	17
RBMS3.1	5.34E-245	0.916339035	0.435	0.024	1.23E-240	17
MAP3K7CL.1	1.82E-244	1.547276099	0.539	0.038	4.17E-240	17
ANTXR1	2.83E-244	0.650682275	0.269	0.008	6.50E-240	17
PLEKHA4	1.16E-241	0.629515532	0.29	0.01	2.66E-237	17
ARHGAP42	8.96E-240	0.600697732	0.29	0.01	2.06E-235	17
TIMP3.2	3.07E-239	1.505688568	0.798	0.084	7.06E-235	17
APBB2	1.23E-236	0.849247875	0.347	0.015	2.82E-232	17
TNS1.2	9.85E-236	1.161519844	0.549	0.042	2.26E-231	17
SLC12A2	7.38E-235	1.010434176	0.435	0.025	1.70E-230	17
PDLIM3	1.05E-234	0.836247534	0.337	0.014	2.41E-230	17
EHD2.2	1.15E-234	1.150025235	0.554	0.042	2.64E-230	17
DCBLD2	1.43E-232	0.663965865	0.28	0.01	3.29E-228	17
TMEM98	2.02E-231	0.681994421	0.337	0.015	4.65E-227	17
SPARCL1.3	5.43E-230	2.173011	1	0.152	1.25E-225	17
ADAMTS4	3.50E-225	0.880012895	0.321	0.014	8.05E-221	17
IGFBP5	1.17E-224	1.445563294	0.409	0.023	2.69E-220	17
LAMB2.1	3.03E-223	1.088052267	0.508	0.037	6.96E-219	17
FAT1	4.19E-221	0.627891595	0.301	0.012	9.62E-217	17
ENPEP.1	1.84E-220	1.025525752	0.461	0.031	4.22E-216	17
MEIS2	4.69E-219	0.761109178	0.311	0.013	1.08E-214	17
CDC42BPA.1	5.28E-218	1.107774996	0.492	0.036	1.21E-213	17
PMEPA1	2.91E-217	1.254106412	0.492	0.036	6.68E-213	17

EDIL3.1	1.63E-216	0.795004192	0.383	0.021	3.74E-212	17
TPM1.4	4.40E-214	2.789054674	0.984	0.183	1.01E-209	17
MT1M	3.38E-213	0.85397468	0.368	0.019	7.78E-209	17
PTPRK.1	9.06E-213	0.913868862	0.451	0.03	2.08E-208	17
PPP1R12B	5.67E-212	1.132867406	0.497	0.037	1.30E-207	17
SPARC.4	1.68E-210	2.424917046	0.99	0.171	3.86E-206	17
SMTN.1	1.52E-208	0.940787241	0.508	0.039	3.50E-204	17
OLFML2B	1.08E-207	1.145568504	0.42	0.027	2.48E-203	17
ITM2C.3	9.48E-203	2.266359183	0.902	0.146	2.18E-198	17
STEAP4	2.55E-202	0.93483955	0.259	0.01	5.86E-198	17
DMD	7.61E-197	0.588285594	0.275	0.011	1.75E-192	17
PTRF.2	3.22E-195	1.388763877	0.668	0.073	7.39E-191	17
PLEKHH3	1.42E-193	0.644041463	0.269	0.011	3.26E-189	17
PPIC.1	1.20E-192	0.881628267	0.404	0.027	2.76E-188	17
COBLL1	5.25E-190	0.68339794	0.311	0.016	1.21E-185	17
NR2F1.1	4.44E-189	0.682822384	0.264	0.011	1.02E-184	17
P2RY14	2.96E-187	0.752688437	0.269	0.012	6.80E-183	17
CAV1.4	6.14E-187	1.784902846	0.86	0.127	1.41E-182	17
SERPING1.2	1.15E-186	1.768655418	0.736	0.1	2.65E-182	17
LIMA1.1	5.00E-186	1.341582884	0.663	0.078	1.15E-181	17
PLAT	1.69E-185	0.817003023	0.342	0.019	3.88E-181	17
IGFBP4.2	1.04E-182	1.80101815	0.772	0.108	2.40E-178	17
TMEM38B	3.95E-182	1.142034472	0.461	0.038	9.08E-178	17
TNS2.1	1.90E-180	0.99266435	0.456	0.037	4.38E-176	17
ADIRF.11	1.56E-176	3.228127014	0.969	0.231	3.59E-172	17
SEPP1.3	1.84E-174	1.300170416	0.813	0.121	4.23E-170	17
SDC2.1	5.59E-174	1.240687609	0.472	0.042	1.28E-169	17
COL1A1	2.37E-172	1.1299391	0.306	0.017	5.44E-168	17
NRP1.1	1.70E-171	1.038753774	0.591	0.065	3.90E-167	17
TMEM204.1	2.48E-171	1.123883872	0.632	0.072	5.69E-167	17
FHL1.1	2.83E-169	1.014023531	0.503	0.048	6.51E-165	17
CLIC4.3	6.27E-168	1.201021477	0.658	0.085	1.44E-163	17
PAWR	5.76E-163	0.67384215	0.316	0.019	1.32E-158	17
NDRG2.2	5.99E-163	1.217127537	0.585	0.069	1.38E-158	17
ESAM.1	5.68E-162	0.805343977	0.554	0.057	1.31E-157	17
CTGF.1	2.10E-160	1.797763281	0.456	0.041	4.83E-156	17
CLEC11A	1.47E-158	0.86940791	0.409	0.034	3.37E-154	17
BCAM.1	5.71E-158	0.889571147	0.554	0.06	1.31E-153	17
ITGA1.1	1.49E-157	1.54176945	0.741	0.109	3.41E-153	17
GSN.5	8.71E-157	1.928456601	0.959	0.215	2.00E-152	17
INPP4B	1.01E-156	1.185827203	0.642	0.081	2.32E-152	17
IGFBP7.14	3.22E-154	3.809416179	1	0.37	7.40E-150	17
PDLIM7	5.93E-150	1.079352936	0.487	0.052	1.36E-145	17
DSTN.14	2.36E-149	2.480525522	0.99	0.298	5.42E-145	17
TPM4.1	3.08E-147	2.070175465	0.87	0.204	7.07E-143	17
FAM213A.2	7.48E-147	1.317117537	0.689	0.105	1.72E-142	17
CRIP2.3	5.82E-146	0.954811035	0.679	0.094	1.34E-141	17
NGFRAP1.3	1.79E-145	1.11914705	0.699	0.104	4.12E-141	17
NID1.1	5.17E-145	0.786418937	0.352	0.027	1.19E-140	17
PDLIM1.3	4.91E-144	1.295255154	0.72	0.112	1.13E-139	17
APOLD1.1	3.13E-143	1.352756425	0.653	0.094	7.19E-139	17
FHL2.1	3.88E-142	0.71060358	0.332	0.024	8.91E-138	17
LAMC1.1	1.78E-141	0.779893216	0.404	0.036	4.08E-137	17
RND3	7.36E-141	0.844474332	0.311	0.022	1.69E-136	17
FSTL1.1	1.36E-140	0.817857849	0.456	0.046	3.13E-136	17
RBFOX2	6.26E-140	0.696262042	0.295	0.02	1.44E-135	17
MDK	2.12E-139	0.712886311	0.306	0.021	4.88E-135	17
FKBP10.1	1.12E-138	0.616562736	0.342	0.027	2.58E-134	17

ROCK2.1	3.18E-138	0.955246271	0.477	0.053	7.30E-134	17
SPECC1.1	1.24E-137	1.046767647	0.446	0.048	2.85E-133	17
SMIM3.1	4.08E-137	1.13385749	0.611	0.088	9.37E-133	17
TLE1	4.77E-137	0.926740643	0.466	0.05	1.10E-132	17
ST5	5.11E-137	0.58262497	0.259	0.015	1.18E-132	17
CTTN.1	5.66E-135	0.694809928	0.389	0.035	1.30E-130	17
LPP.1	9.45E-135	1.542947146	0.788	0.157	2.17E-130	17
LPL.1	1.46E-133	0.962205653	0.352	0.03	3.35E-129	17
EDNRB.1	2.47E-133	0.804278618	0.503	0.057	5.68E-129	17
WBP5.3	4.35E-132	0.902004017	0.575	0.078	1.00E-127	17
C16orf45	1.10E-131	0.542616793	0.264	0.016	2.52E-127	17
HSPB2.1	7.40E-131	0.581777191	0.285	0.019	1.70E-126	17
SOCS2	2.42E-130	0.736504573	0.337	0.027	5.56E-126	17
ZNF503	4.22E-128	0.702061005	0.306	0.023	9.71E-124	17
PRKCDBP.3	8.10E-128	1.037289872	0.56	0.077	1.86E-123	17
PDLIM5.1	1.63E-127	1.290208783	0.544	0.077	3.75E-123	17
PACSIN2	2.31E-127	1.12629601	0.549	0.077	5.30E-123	17
CFL2.1	3.28E-124	0.919477576	0.435	0.048	7.54E-120	17
NTN4.1	6.46E-124	0.707890415	0.332	0.027	1.48E-119	17
NBL1	3.48E-123	0.777500611	0.383	0.037	7.99E-119	17
NDUFA4L2.16	6.00E-123	1.84000297	0.979	0.279	1.38E-118	17
FBLIM1	6.64E-122	0.996581688	0.451	0.052	1.52E-117	17
CD151.4	3.68E-121	1.348564762	0.839	0.192	8.46E-117	17
RASAL2.2	3.33E-120	0.917225722	0.446	0.053	7.65E-116	17
SEPT11.1	1.10E-119	1.293726381	0.663	0.118	2.52E-115	17
MAP1LC3A.1	1.31E-119	0.932308664	0.513	0.067	3.00E-115	17
TSC22D1.4	3.05E-119	1.393715421	0.824	0.174	7.02E-115	17
EMCN.1	4.51E-119	0.748577649	0.358	0.033	1.04E-114	17
FBXO32	1.92E-118	1.234633548	0.513	0.069	4.41E-114	17
SASH1.1	5.57E-118	0.742634156	0.352	0.033	1.28E-113	17
PTMS.5	1.09E-116	1.497412029	0.886	0.233	2.52E-112	17
GNAI1.1	2.64E-116	0.518613699	0.28	0.021	6.07E-112	17
RBPMS.2	1.34E-115	0.734833556	0.477	0.06	3.08E-111	17
CYSTM1.4	1.40E-115	0.956418089	0.715	0.133	3.21E-111	17
TWSG1	3.46E-115	0.674282545	0.326	0.03	7.96E-111	17
CHN1	2.58E-114	0.508577601	0.269	0.02	5.93E-110	17
FN1.2	2.24E-112	1.308737812	0.658	0.116	5.14E-108	17
TRIP6.1	1.21E-111	0.79944154	0.42	0.05	2.79E-107	17
CAMK2N1.1	8.68E-111	0.521511251	0.259	0.019	2.00E-106	17
PRSS23.2	1.08E-110	0.865525093	0.648	0.107	2.48E-106	17
EFEMP2.1	3.11E-110	0.684075265	0.337	0.032	7.16E-106	17
GAS6.1	4.16E-109	0.879505836	0.492	0.07	9.57E-105	17
KLHDC8B	6.09E-109	0.64068261	0.28	0.023	1.40E-104	17
CHST7	4.23E-108	0.573157914	0.29	0.024	9.72E-104	17
GNG12.1	4.84E-108	0.586824627	0.29	0.025	1.11E-103	17
NUDT4	6.05E-108	1.3075467	0.637	0.12	1.39E-103	17
JADE1	1.44E-107	1.064462318	0.523	0.079	3.30E-103	17
AK1	4.46E-107	0.704910549	0.383	0.042	1.02E-102	17
B3GNT2	5.74E-107	0.97292268	0.549	0.085	1.32E-102	17
MEF2C.3	1.92E-106	1.245963463	0.715	0.143	4.41E-102	17
MAGED2.1	4.47E-106	1.276115959	0.694	0.144	1.03E-101	17
UBA2	7.82E-106	1.238023565	0.71	0.149	1.80E-101	17
H1F0.1	1.43E-105	0.809576943	0.43	0.056	3.28E-101	17
PKIG.3	4.23E-105	0.721048272	0.446	0.058	9.73E-101	17
ILK	6.81E-105	1.257543303	0.705	0.153	1.57E-100	17
RAPGEF5.1	8.67E-105	0.52573693	0.321	0.03	1.99E-100	17
TIMP1.15	2.60E-104	2.121081719	0.927	0.306	5.97E-100	17
GSTM3.1	3.15E-104	0.684821791	0.378	0.043	7.23E-100	17

TRIB2	6.16E-104	0.781786129	0.368	0.04	1.42E-99	17
TCF4.2	2.42E-101	1.034538451	0.611	0.112	5.56E-97	17
LGALS1.8	1.46E-100	1.92650989	1	0.566	3.35E-96	17
SERPINH1.1	4.39E-100	1.109220113	0.487	0.074	1.01E-95	17
SLCO3A1	1.82E-99	0.848451726	0.373	0.044	4.18E-95	17
IFITM3.15	3.14E-99	1.732398418	0.979	0.37	7.22E-95	17
FARP1.1	5.74E-99	0.687689892	0.373	0.045	1.32E-94	17
OAZ2.2	9.35E-99	1.200726281	0.741	0.175	2.15E-94	17
C4orf32	1.17E-98	0.671322778	0.326	0.034	2.70E-94	17
ARHGAP10	3.73E-97	0.793750594	0.342	0.038	8.58E-93	17
GOLIM4.2	1.92E-96	0.950313147	0.534	0.092	4.41E-92	17
C1orf54.3	2.22E-96	0.965928934	0.503	0.081	5.10E-92	17
TJP1.1	6.24E-96	0.555365618	0.326	0.034	1.44E-91	17
ARHGAP29.1	2.98E-93	0.592261942	0.456	0.064	6.84E-89	17
COL6A3	7.97E-93	0.689935182	0.306	0.031	1.83E-88	17
PPFIBP1.2	8.75E-93	0.552342324	0.389	0.049	2.01E-88	17
A2M.3	1.52E-92	1.150510313	0.767	0.188	3.50E-88	17
CYB5R3.4	7.80E-92	0.991042859	0.746	0.179	1.79E-87	17
HSPB1.15	2.82E-91	1.839160513	0.979	0.427	6.49E-87	17
UBE2E2.2	3.61E-91	0.772351291	0.435	0.066	8.30E-87	17
ZFHX3.3	4.88E-91	0.958301205	0.487	0.081	1.12E-86	17
SPTBN1.1	5.09E-91	0.735901689	0.56	0.097	1.17E-86	17
SGCB.1	4.62E-90	0.603359303	0.337	0.039	1.06E-85	17
CD59.6	2.34E-89	0.841798665	0.845	0.224	5.37E-85	17
SPRY2	1.12E-88	0.618317137	0.311	0.034	2.58E-84	17
RGS16	1.67E-88	0.936667404	0.311	0.034	3.84E-84	17
EPHX1.1	2.71E-88	0.630621483	0.326	0.038	6.24E-84	17
TSPAN3.1	3.16E-88	0.811581251	0.534	0.097	7.26E-84	17
PTEN.3	1.05E-87	1.050728175	0.648	0.144	2.41E-83	17
FAM127A	1.49E-87	0.833892297	0.477	0.08	3.42E-83	17
AXL.2	3.76E-87	1.066106158	0.466	0.078	8.63E-83	17
SORBS2.1	1.23E-86	0.65945143	0.347	0.043	2.83E-82	17
LMNA.2	3.89E-86	1.02760976	0.72	0.167	8.93E-82	17
APP.5	2.66E-85	0.814342743	0.72	0.171	6.11E-81	17
ENG.2	2.27E-84	0.788627007	0.544	0.101	5.22E-80	17
GPX3.5	1.02E-83	1.145334645	0.668	0.158	2.35E-79	17
IFI27.3	2.25E-83	0.919709222	0.674	0.147	5.17E-79	17
PPARG	2.56E-83	0.589591424	0.254	0.025	5.87E-79	17
ARMCX2	2.94E-83	0.593993447	0.264	0.027	6.75E-79	17
DST.2	3.56E-83	0.911402384	0.451	0.077	8.18E-79	17
GRK5	1.63E-82	0.574875551	0.321	0.038	3.76E-78	17
ZBTB16.4	4.43E-82	1.249428403	0.767	0.213	1.02E-77	17
FAM46A.1	4.50E-82	0.989268199	0.472	0.081	1.03E-77	17
MYO1C.1	6.82E-81	0.630675347	0.378	0.054	1.57E-76	17
FLNA.3	8.02E-80	1.08825788	0.699	0.177	1.84E-75	17
SERPINI1.1	1.52E-79	0.681931806	0.29	0.032	3.50E-75	17
ITGB1.10	1.10E-78	1.556946421	0.948	0.457	2.53E-74	17
ARHGAP17	8.72E-78	0.601491135	0.306	0.037	2.00E-73	17
SELM.4	2.51E-77	1.163623173	0.689	0.177	5.77E-73	17
MYL6.8	1.87E-75	1.349001624	0.99	0.794	4.30E-71	17
TCF7L2.1	5.01E-75	0.565608579	0.29	0.035	1.15E-70	17
MARCKSL1.1	1.66E-74	0.793031371	0.461	0.082	3.81E-70	17
ARHGEF7.1	3.90E-74	0.667151303	0.409	0.066	8.97E-70	17
WBP4	4.59E-73	0.912466289	0.487	0.096	1.05E-68	17
CDKN1A.2	5.05E-73	0.817884667	0.539	0.114	1.16E-68	17
HIP1	1.61E-72	0.662499657	0.425	0.071	3.69E-68	17
TUBB6	2.20E-72	0.543015977	0.269	0.031	5.06E-68	17
LEPROT.3	6.30E-72	0.92709923	0.699	0.191	1.45E-67	17

CALU.1	1.74E-71	0.724721787	0.492	0.097	4.00E-67	17
MGST3.13	2.48E-71	1.162589979	0.85	0.299	5.69E-67	17
PTPN9	2.71E-71	0.601502203	0.342	0.049	6.24E-67	17
ARHGAP1	1.09E-70	0.604074232	0.358	0.054	2.51E-66	17
SORT1.1	6.26E-70	0.635773155	0.337	0.05	1.44E-65	17
SOX4.1	1.80E-69	0.651705082	0.492	0.097	4.13E-65	17
EPB41L2.3	4.52E-69	0.778119353	0.466	0.091	1.04E-64	17
FSTL3.1	7.32E-69	0.562682305	0.269	0.032	1.68E-64	17
H2AFJ.2	7.96E-69	0.941408139	0.715	0.203	1.83E-64	17
CSNK1E	8.22E-69	0.593887717	0.378	0.061	1.89E-64	17
CST3.16	8.97E-69	0.960351922	0.984	0.377	2.06E-64	17
TRAK1	1.14E-68	0.582457417	0.275	0.034	2.62E-64	17
TOB1.2	2.15E-68	0.926110951	0.679	0.174	4.93E-64	17
KLF9.1	2.17E-68	0.850884675	0.622	0.155	4.99E-64	17
ASAP1.1	1.22E-67	0.791056052	0.472	0.095	2.80E-63	17
PHACTR2.2	1.45E-67	0.772906075	0.503	0.103	3.33E-63	17
CTBP2	1.92E-67	0.641410447	0.389	0.067	4.41E-63	17
TCEAL4.4	2.04E-67	0.840364965	0.606	0.153	4.68E-63	17
HES1.3	4.82E-67	0.766020946	0.44	0.08	1.11E-62	17
TACC1.3	7.99E-67	0.977708405	0.741	0.222	1.84E-62	17
CPQ	1.16E-66	0.747420237	0.461	0.092	2.67E-62	17
MTHFD2.2	2.37E-66	1.035971531	0.513	0.116	5.45E-62	17
INAFM1	8.02E-66	0.553597696	0.264	0.033	1.84E-61	17
IFIT3	1.20E-65	0.717115447	0.321	0.047	2.76E-61	17
UTRN.4	1.62E-65	1.218259269	0.715	0.224	3.71E-61	17
NFIA.2	1.88E-65	0.69539366	0.425	0.079	4.31E-61	17
PELO	3.47E-65	0.517058517	0.321	0.047	7.97E-61	17
MTUS1.1	1.88E-64	0.505734402	0.352	0.056	4.33E-60	17
RPS29.12	3.34E-64	-1.679956859	0.974	0.92	7.68E-60	17
ADAP2.2	4.05E-64	0.777153326	0.42	0.08	9.30E-60	17
SEPT7.9	5.59E-64	1.387093331	0.933	0.507	1.29E-59	17
MAGEF1	2.00E-63	0.52007443	0.316	0.047	4.59E-59	17
CD63.14	8.64E-63	1.144351175	0.974	0.513	1.99E-58	17
KIAA0040	2.81E-62	0.578638976	0.337	0.053	6.45E-58	17
C5orf24.1	3.76E-62	0.758885828	0.503	0.113	8.64E-58	17
PRKAR1A.4	4.04E-62	0.908285491	0.777	0.254	9.29E-58	17
EGR1.3	1.16E-61	1.036390312	0.56	0.136	2.66E-57	17
AAMDC.1	2.05E-61	0.553503376	0.435	0.086	4.71E-57	17
GRAMD3.1	6.24E-61	0.562231035	0.321	0.05	1.43E-56	17
TFPI.2	1.12E-60	0.620268276	0.383	0.068	2.57E-56	17
RHOB.14	1.62E-60	1.005070536	0.907	0.341	3.73E-56	17
RSU1.1	4.80E-60	0.760164231	0.554	0.137	1.10E-55	17
LAPTM4A.12	6.86E-60	0.971943555	0.87	0.345	1.58E-55	17
CRIM1.1	1.52E-59	0.77974042	0.404	0.076	3.50E-55	17
FHL3.1	4.33E-59	0.535139794	0.347	0.06	9.95E-55	17
TNS3	5.59E-59	0.590230556	0.269	0.038	1.29E-54	17
EID1.6	5.45E-58	1.006070684	0.902	0.421	1.25E-53	17
SPATS2L.1	5.69E-57	0.590195415	0.508	0.119	1.31E-52	17
ITGAV.2	7.94E-57	0.617061045	0.389	0.077	1.82E-52	17
SCPEP1.3	1.03E-56	0.740004301	0.528	0.131	2.37E-52	17
STOM.1	3.77E-55	0.847459686	0.642	0.196	8.67E-51	17
DYNLT3	6.05E-55	0.680219618	0.513	0.126	1.39E-50	17
CBX6	8.16E-55	0.551685143	0.404	0.081	1.87E-50	17
OSBPL1A.1	4.12E-54	0.544175095	0.368	0.071	9.48E-50	17
NENF.2	4.34E-54	0.708289256	0.642	0.191	9.98E-50	17
RPL28.11	4.91E-54	-1.123056835	0.99	0.932	1.13E-49	17
HTRA1.3	5.00E-54	0.582702584	0.461	0.105	1.15E-49	17
ACTN4.3	1.16E-53	1.048387731	0.689	0.228	2.67E-49	17

SH3BGRL.10	1.34E-53	0.999075444	0.87	0.383	3.07E-49	17
MIR4435-2HG.3	1.52E-53	0.740016407	0.56	0.146	3.49E-49	17
SSPN.2	2.24E-53	0.594054757	0.275	0.043	5.15E-49	17
BCL6	4.00E-53	0.578529074	0.306	0.052	9.19E-49	17
RCN2.2	8.11E-53	0.713719254	0.56	0.149	1.86E-48	17
IFI6.1	1.15E-52	0.96573578	0.658	0.2	2.64E-48	17
CEBPD.12	1.17E-52	1.047861073	0.788	0.272	2.68E-48	17
ZMAT3	1.52E-52	0.661254867	0.358	0.068	3.50E-48	17
PPP1R12A.8	6.45E-52	0.970114354	0.756	0.272	1.48E-47	17
CYBRD1.1	9.58E-52	0.518190736	0.259	0.04	2.20E-47	17
FABP5.3	2.38E-51	0.896764232	0.648	0.201	5.46E-47	17
HCFC1R1.3	3.31E-51	0.577382891	0.492	0.122	7.61E-47	17
CD9.5	1.12E-50	0.934165105	0.679	0.217	2.58E-46	17
ARMCX3.2	4.42E-50	0.800195194	0.528	0.146	1.02E-45	17
ZBTB20.3	6.80E-50	0.67021986	0.549	0.148	1.56E-45	17
ZHX1	1.15E-49	0.529026994	0.301	0.053	2.64E-45	17
SEPW1.13	1.32E-49	0.80911492	0.829	0.353	3.03E-45	17
ADI1.1	4.10E-49	0.604798009	0.554	0.156	9.43E-45	17
DYNC1I2.6	5.60E-49	0.815383731	0.679	0.238	1.29E-44	17
HIGD1A	1.80E-48	0.609912683	0.446	0.108	4.13E-44	17
SLC39A6.1	2.03E-48	0.587928919	0.337	0.067	4.67E-44	17
RPS27.10	3.20E-48	-1.041115202	1	0.975	7.36E-44	17
TAX1BP3.2	3.20E-48	0.608326268	0.508	0.138	7.36E-44	17
ADD3.3	1.62E-47	0.804536862	0.56	0.162	3.72E-43	17
CDC5L	3.25E-47	0.736197485	0.497	0.134	7.46E-43	17
RPS15A.8	4.49E-47	-0.966607871	1	0.933	1.03E-42	17
SBDS.6	6.52E-47	0.756642492	0.736	0.265	1.50E-42	17
TIMP2.4	2.21E-46	0.650583838	0.358	0.077	5.07E-42	17
AIG1.1	3.11E-46	0.511168457	0.383	0.087	7.15E-42	17
ANXA6.13	7.21E-46	0.886389668	0.705	0.243	1.66E-41	17
DYNLL1.9	7.27E-46	0.861429329	0.876	0.403	1.67E-41	17
BCAP29.1	1.12E-45	0.605134265	0.477	0.125	2.57E-41	17
RPS28.8	1.35E-45	-0.860798041	0.995	0.946	3.10E-41	17
PEBP1.13	1.40E-45	0.713914758	0.87	0.383	3.22E-41	17
MT2A.16	2.08E-45	0.705662443	0.969	0.655	4.77E-41	17
RPS2.8	2.50E-45	-0.945201269	0.99	0.944	5.74E-41	17
RPL39.8	3.28E-45	-0.971890143	0.995	0.944	7.54E-41	17
CALM2.6	4.56E-45	0.974452524	0.984	0.626	1.05E-40	17
BSG.8	5.13E-45	0.752602429	0.788	0.322	1.18E-40	17
RPLP2.8	5.15E-45	-0.887666585	1	0.943	1.18E-40	17
F2R.2	6.92E-45	0.592950951	0.492	0.125	1.59E-40	17
RAC1.10	8.36E-45	0.787929799	0.933	0.441	1.92E-40	17
CXCR4.12	9.33E-45	-2.771115273	0.57	0.778	2.14E-40	17
EI24.2	1.43E-44	0.526411079	0.425	0.104	3.28E-40	17
OLFML3.1	2.47E-44	0.554471812	0.259	0.044	5.68E-40	17
RAB2A.6	4.40E-44	0.672609517	0.72	0.263	1.01E-39	17
RRAGA.1	4.70E-44	0.567205748	0.482	0.132	1.08E-39	17
RPL41.12	1.57E-43	-0.781815854	1	0.976	3.60E-39	17
RABGAP1.1	1.90E-43	0.550024313	0.316	0.064	4.36E-39	17
HSD17B12.2	7.32E-43	0.641246567	0.42	0.108	1.68E-38	17
CRTC3	8.29E-43	0.576451733	0.332	0.07	1.91E-38	17
SMARCB1	3.89E-42	0.625414571	0.456	0.125	8.93E-38	17
MAP4.3	6.94E-42	0.571191807	0.565	0.173	1.59E-37	17
ARHGEF12.1	8.06E-42	0.505692362	0.342	0.074	1.85E-37	17
ADD1.1	2.00E-41	0.543364844	0.497	0.14	4.60E-37	17
ARFGAP3.1	2.01E-41	0.599532411	0.43	0.113	4.63E-37	17
EEA1.1	6.94E-41	0.589653719	0.383	0.095	1.60E-36	17
ABI2.1	1.62E-40	0.586505674	0.368	0.088	3.73E-36	17

RNASEH2C.1	2.08E-40	0.66444758	0.549	0.174	4.78E-36	17
CBR1	2.45E-40	0.533540625	0.378	0.093	5.64E-36	17
TLN1.6	2.77E-40	0.760502548	0.689	0.259	6.36E-36	17
MT-CYB.13	3.31E-40	0.811878827	0.979	0.802	7.61E-36	17
NUCKS1.6	8.47E-40	0.885492497	0.824	0.392	1.95E-35	17
PTPRC.15	1.80E-39	-2.486537834	0.207	0.622	4.15E-35	17
DCTN2	3.02E-39	0.570066665	0.518	0.16	6.94E-35	17
ZNF428.1	3.61E-39	0.529670618	0.544	0.17	8.30E-35	17
RPS12.5	4.35E-39	-0.874579011	0.99	0.938	1.00E-34	17
AP2M1.4	6.48E-39	0.666593613	0.767	0.317	1.49E-34	17
SKP1.4	1.36E-38	0.788994488	0.938	0.598	3.14E-34	17
CHD9.2	1.80E-38	0.695906838	0.58	0.198	4.13E-34	17
FNIP2.3	1.84E-38	0.56734666	0.321	0.073	4.24E-34	17
ACTR10	5.00E-38	0.506927885	0.466	0.134	1.15E-33	17
AHCYL1.1	5.58E-38	0.55309454	0.342	0.083	1.28E-33	17
SYPL1.2	7.11E-38	0.64181949	0.549	0.184	1.63E-33	17
DDAH2.2	7.95E-38	0.649980787	0.43	0.123	1.83E-33	17
VIMP.3	1.22E-37	0.658081107	0.622	0.223	2.80E-33	17
FDX1	2.29E-37	0.650160426	0.503	0.156	5.26E-33	17
KCNMB1.2	3.24E-37	0.587258459	0.28	0.059	7.46E-33	17
GBP2.8	9.37E-37	0.924148202	0.663	0.257	2.15E-32	17
RTN4.13	1.16E-36	0.714198923	0.788	0.344	2.67E-32	17
RPL10.10	1.96E-36	-0.735488429	1	0.974	4.50E-32	17
CHCHD10.10	2.31E-35	0.629559799	0.632	0.241	5.31E-31	17
RPS25.11	2.47E-35	-0.881519591	0.995	0.917	5.67E-31	17
TMEM14C.5	2.93E-35	0.556948996	0.611	0.221	6.72E-31	17
RPS3.11	3.85E-35	-0.83105147	0.984	0.918	8.84E-31	17
ATP5B.4	5.81E-35	0.680836875	0.788	0.363	1.34E-30	17
RPL26.7	9.10E-35	-0.776724656	0.99	0.935	2.09E-30	17
SYNE2.16	1.60E-34	0.654824962	0.798	0.326	3.67E-30	17
OSBPL9.1	2.17E-34	0.557729472	0.326	0.081	5.00E-30	17
BST2.10	2.32E-34	0.719723932	0.788	0.355	5.34E-30	17
ACADVL	2.91E-34	0.661278034	0.58	0.211	6.69E-30	17
KTN1.10	4.80E-34	0.618071146	0.829	0.377	1.10E-29	17
RHOC.11	5.50E-34	0.573594887	0.736	0.317	1.26E-29	17
VASP.2	7.14E-34	0.593986364	0.606	0.221	1.64E-29	17
PTPN12.1	7.40E-34	0.536889318	0.383	0.107	1.70E-29	17
ZFP36L2.14	1.58E-33	-2.559215908	0.487	0.701	3.63E-29	17
TMSB10.11	2.78E-33	-1.004343634	0.995	0.96	6.39E-29	17
NDUFS2.1	5.88E-33	0.553365622	0.534	0.186	1.35E-28	17
TMEM59.5	6.12E-33	0.658599986	0.87	0.471	1.41E-28	17
SLC25A3.5	8.74E-33	0.677995119	0.834	0.405	2.01E-28	17
RGS1.17	9.71E-33	-3.005255704	0.513	0.699	2.23E-28	17
ITFG1.1	1.06E-32	0.549464691	0.389	0.111	2.44E-28	17
ARF4.1	2.23E-32	0.506039264	0.544	0.194	5.11E-28	17
POLR2F	2.93E-32	0.544178136	0.575	0.206	6.74E-28	17
CLTA.10	3.50E-32	0.550634262	0.684	0.285	8.05E-28	17
ZFAND5.10	3.84E-32	0.574320181	0.72	0.302	8.83E-28	17
RPLP1.4	4.79E-32	-0.686362969	1	0.966	1.10E-27	17
MTRNR2L8.3	6.11E-32	0.596737658	0.591	0.22	1.41E-27	17
TBC1D1.1	1.00E-31	0.555934367	0.389	0.113	2.30E-27	17
SEPT2.6	1.74E-31	0.746285245	0.622	0.257	3.99E-27	17
RPS14.9	9.16E-31	-0.699609501	1	0.946	2.10E-26	17
CORO1A.13	1.02E-30	-2.067335855	0.202	0.57	2.35E-26	17
ROCK1.10	1.70E-30	0.64980974	0.736	0.325	3.91E-26	17
VMP1.11	1.74E-30	0.663291266	0.72	0.314	4.00E-26	17
RPS21.10	1.76E-30	-0.991914539	0.938	0.861	4.04E-26	17
RPL23A.11	1.99E-30	-0.745237455	0.99	0.929	4.57E-26	17

COX17.9	7.61E-30	0.598075119	0.71	0.313	1.75E-25	17
MRFAP1.7	1.53E-29	0.51191522	0.699	0.304	3.52E-25	17
NSRP1.1	1.67E-29	0.504519903	0.497	0.174	3.85E-25	17
ATP6AP2.9	1.69E-29	0.568789351	0.694	0.301	3.88E-25	17
TIPARP.2	1.90E-29	0.569182445	0.409	0.128	4.38E-25	17
HSP90AA1.10	1.99E-29	0.605536438	0.984	0.649	4.57E-25	17
RBMS1.3	2.15E-29	0.519344566	0.554	0.201	4.93E-25	17
TMEM165.5	4.76E-29	0.560436833	0.585	0.234	1.09E-24	17
SUMO2.5	5.32E-29	0.602810308	0.959	0.602	1.22E-24	17
CCND1.2	6.13E-29	0.514698784	0.326	0.089	1.41E-24	17
HCST.13	1.35E-28	-2.079659264	0.197	0.549	3.11E-24	17
RPL27A.7	1.97E-28	-0.658270132	1	0.929	4.53E-24	17
RPS24.10	2.78E-28	-0.726504996	0.995	0.912	6.40E-24	17
ISG15.3	2.95E-28	1.098240222	0.544	0.206	6.77E-24	17
RPS6.7	9.23E-28	-0.705671093	0.99	0.943	2.12E-23	17
EIF4A1.5	1.42E-27	0.555408975	0.725	0.35	3.26E-23	17
TMEM30A.1	1.91E-27	0.508893689	0.42	0.141	4.40E-23	17
HSP90B1.5	2.01E-27	0.529544757	0.798	0.4	4.61E-23	17
RPL37.9	2.23E-27	-0.747431674	1	0.907	5.12E-23	17
SRGN.13	2.87E-27	-1.574968678	0.819	0.791	6.59E-23	17
ERH.4	3.20E-27	0.535002521	0.741	0.355	7.36E-23	17
DENND3.3	5.24E-27	0.523662146	0.321	0.093	1.21E-22	17
RPS18.10	7.27E-27	-0.675975383	1	0.958	1.67E-22	17
PAK1.3	7.66E-27	0.555921323	0.326	0.097	1.76E-22	17
RPL11.6	9.35E-27	-0.643040791	1	0.937	2.15E-22	17
RPL30.6	1.06E-26	-0.70769779	0.984	0.896	2.45E-22	17
S100A6.9	2.14E-26	-1.595107325	0.58	0.769	4.91E-22	17
LAPTM5.13	2.89E-26	-1.927984409	0.28	0.571	6.64E-22	17
RPL13.8	2.00E-25	-0.594604532	1	0.964	4.59E-21	17
MT-ATP6.11	2.13E-25	0.590799811	0.984	0.857	4.90E-21	17
VIM.14	2.95E-25	0.567181836	0.99	0.768	6.78E-21	17
MYH9.11	3.90E-25	0.60863595	0.663	0.306	8.96E-21	17
RPL36.13	4.54E-25	-0.812614308	0.995	0.911	1.04E-20	17
RPS19.9	6.26E-25	-0.682420047	1	0.951	1.44E-20	17
RPL34.10	1.06E-24	-0.644158759	0.995	0.952	2.43E-20	17
FKBP5.11	1.76E-24	0.589613152	0.803	0.39	4.04E-20	17
RPL18A.8	2.07E-24	-0.669837057	0.995	0.929	4.75E-20	17
GADD45B.3	2.25E-24	0.63715014	0.554	0.229	5.18E-20	17
LUC7L3.10	3.03E-24	0.516735751	0.668	0.306	6.96E-20	17
CD2.16	6.38E-24	-2.322809399	0.124	0.462	1.47E-19	17
CD164.5	2.12E-23	0.56043916	0.689	0.326	4.88E-19	17
CD37.12	2.83E-23	-1.686054735	0.145	0.467	6.51E-19	17
IL32.14	3.33E-23	-2.448174415	0.332	0.581	7.65E-19	17
RPL19.8	5.72E-23	-0.586737484	0.995	0.924	1.32E-18	17
UBA52.5	7.19E-23	-0.726071773	0.943	0.866	1.65E-18	17
LINC00152.10	9.07E-23	0.543314961	0.663	0.312	2.08E-18	17
ELOVL5.1	1.25E-22	0.520008416	0.466	0.182	2.87E-18	17
RPL38.9	1.35E-22	-0.811471045	0.948	0.866	3.09E-18	17
RPS20.8	1.54E-22	-0.682647938	0.99	0.895	3.53E-18	17
FYB.15	2.41E-22	-1.756851875	0.15	0.464	5.54E-18	17
RPL31.13	3.09E-22	-0.714299839	0.995	0.906	7.10E-18	17
ACTB.12	3.46E-22	0.502574176	1	0.928	7.94E-18	17
BTG1.16	4.46E-22	-1.351661357	0.927	0.841	1.02E-17	17
RPL13A.11	4.79E-22	-0.516919179	1	0.962	1.10E-17	17
S100A10.10	1.11E-21	-1.82752156	0.409	0.613	2.55E-17	17
RPS26.12	4.34E-21	-0.780441608	0.969	0.869	9.97E-17	17
CD69.16	5.92E-21	-2.582527879	0.264	0.532	1.36E-16	17
CYTIP.15	7.92E-21	-1.783681361	0.187	0.479	1.82E-16	17

TXNIP.12	1.01E-20	-1.63866069	0.803	0.763	2.33E-16	17
MAPRE1	2.75E-20	0.508225188	0.446	0.18	6.32E-16	17
SLA.14	6.57E-20	-1.558286551	0.093	0.403	1.51E-15	17
CD3E.16	1.35E-19	-1.785739905	0.104	0.402	3.10E-15	17
RPS17.7	1.76E-19	-0.630316928	0.979	0.897	4.05E-15	17
CD53.11	2.20E-19	-1.488048461	0.192	0.475	5.06E-15	17
RPS27A.11	2.97E-19	-0.521425738	0.995	0.939	6.83E-15	17
STK4.13	3.80E-19	-1.625857388	0.192	0.476	8.72E-15	17
RPSA.9	5.92E-19	-0.915308116	0.933	0.802	1.36E-14	17
CELF2.13	1.50E-18	-1.544569344	0.161	0.43	3.45E-14	17
CD74.12	1.73E-18	-2.208292313	0.85	0.793	3.98E-14	17
DUSP2.16	3.22E-18	-2.324075998	0.166	0.435	7.41E-14	17
TRAC.17	4.61E-18	-2.198427064	0.171	0.436	1.06E-13	17
JUN.11	4.82E-18	0.889543266	0.902	0.689	1.11E-13	17
RPL21.8	1.10E-17	-0.516704547	0.995	0.951	2.52E-13	17
CCL5.16	1.15E-17	-2.967741365	0.394	0.564	2.65E-13	17
CD52.13	1.84E-17	-2.167300966	0.378	0.551	4.22E-13	17
CD3D.17	2.31E-17	-2.057265269	0.249	0.478	5.31E-13	17
SH3BGRL3.13	2.43E-17	-1.380850164	0.648	0.682	5.57E-13	17
FOSB.8	3.76E-17	0.57567883	0.731	0.421	8.65E-13	17
GZMA.16	4.02E-17	-2.27796634	0.166	0.423	9.25E-13	17
RPS15.9	4.14E-17	-0.502051905	1	0.931	9.51E-13	17
RAC2.15	9.38E-17	-1.393509178	0.098	0.376	2.16E-12	17
EVI2B.10	9.54E-17	-1.338549188	0.062	0.339	2.19E-12	17
FAU.6	3.04E-16	-0.539413277	0.995	0.887	6.98E-12	17
S100A4.15	3.04E-16	-1.328089612	0.606	0.701	6.98E-12	17
LSP1.16	4.54E-16	-1.398298539	0.192	0.438	1.04E-11	17
CD48.15	4.87E-16	-1.313438432	0.114	0.379	1.12E-11	17
LCP1.14	4.93E-16	-1.415596886	0.155	0.408	1.13E-11	17
IL2RG.17	6.43E-16	-1.5006807	0.119	0.376	1.48E-11	17
RPL36A.10	8.20E-16	-0.937354789	0.865	0.8	1.88E-11	17
SAMSN1.10	8.73E-16	-1.469596706	0.062	0.326	2.01E-11	17
CST7.15	1.02E-15	-2.042214471	0.145	0.392	2.35E-11	17
RPL18.8	1.38E-15	-0.624166689	0.959	0.869	3.18E-11	17
SYTL3.15	4.56E-15	-1.319619675	0.026	0.281	1.05E-10	17
ACAP1.17	1.19E-14	-1.354213647	0.088	0.336	2.74E-10	17
VAMP8.10	1.21E-14	-1.381724076	0.259	0.464	2.77E-10	17
ITGB2.14	3.99E-14	-1.349620117	0.197	0.418	9.18E-10	17
RPS16.7	5.85E-14	-0.529325836	0.984	0.89	1.34E-09	17
CD3G.16	9.05E-14	-1.467310417	0.062	0.296	2.08E-09	17
EVI2A.12	9.05E-14	-1.148842412	0.047	0.283	2.08E-09	17
RP11-347P5.1.16	1.01E-13	-1.580146759	0.145	0.379	2.33E-09	17
KLF6.13	1.30E-13	-1.511032681	0.56	0.65	2.99E-09	17
TNFAIP3.16	1.61E-13	-1.688450793	0.249	0.45	3.69E-09	17
UCP2.12	2.82E-13	-1.226889886	0.119	0.35	6.48E-09	17
HLA-DRB1.14	3.68E-13	-2.465133175	0.544	0.591	8.45E-09	17
GIMAP4.12	6.69E-13	-1.245184609	0.114	0.342	1.54E-08	17
CLEC2B.10	1.94E-12	-1.206281713	0.155	0.381	4.46E-08	17
RPS10.7	2.25E-12	-0.697770836	0.907	0.785	5.16E-08	17
TRBC2.16	2.33E-12	-1.792122485	0.181	0.38	5.35E-08	17
ID2.12	3.49E-12	-1.587578782	0.461	0.566	8.02E-08	17
FOS.15	3.92E-12	0.530990133	0.902	0.678	9.00E-08	17
BIRC3.14	4.73E-12	-1.5355808	0.062	0.275	1.09E-07	17
FAM49B.11	6.67E-12	-1.161908393	0.135	0.343	1.53E-07	17
ICAM3.15	6.84E-12	-1.066316843	0.062	0.275	1.57E-07	17
FXYD5.10	7.43E-12	-1.240198773	0.337	0.483	1.71E-07	17
STK17A.15	8.12E-12	-1.37285454	0.171	0.369	1.87E-07	17
ALOX5AP.13	1.35E-11	-1.581294045	0.13	0.331	3.10E-07	17

PPP2R5C.13	1.74E-11	-1.295429703	0.187	0.393	4.00E-07	17
STK17B.13	2.10E-11	-1.312676028	0.212	0.403	4.83E-07	17
CCL4.17	2.24E-11	-2.840795378	0.28	0.442	5.16E-07	17
RGS10.11	2.27E-11	-1.196023637	0.098	0.298	5.21E-07	17
ITM2A.16	2.44E-11	-1.555760775	0.114	0.313	5.60E-07	17
HMGB2.12	3.82E-11	-1.38609283	0.337	0.478	8.78E-07	17
CYBA.11	4.01E-11	-1.069469536	0.746	0.686	9.21E-07	17
GZMK.17	4.70E-11	-2.567214408	0.187	0.368	1.08E-06	17
GIMAP7.15	4.81E-11	-1.27623135	0.088	0.287	1.11E-06	17
LTB.15	5.29E-11	-1.720368145	0.109	0.304	1.22E-06	17
EMB.13	6.78E-11	-1.031813581	0.073	0.272	1.56E-06	17
HLA-DPA1.14	8.31E-11	-2.367782112	0.539	0.571	1.91E-06	17
CLEC2D.16	1.19E-10	-1.27145242	0.114	0.309	2.74E-06	17
BIN2.12	1.28E-10	-1.017224813	0.057	0.252	2.94E-06	17
GPSM3.12	2.18E-10	-1.073534643	0.212	0.393	5.01E-06	17
TRAF3IP3.16	2.27E-10	-0.943190017	0.062	0.258	5.21E-06	17
TRBC1.16	2.82E-10	-2.072254179	0.187	0.355	6.47E-06	17
CD7.14	3.15E-10	-1.514249226	0.119	0.303	7.24E-06	17
ATP5E.11	3.94E-10	-0.597383823	0.948	0.814	9.06E-06	17
PIK3IP1.16	4.62E-10	-1.225165046	0.181	0.36	1.06E-05	17
NDUFA4.7	4.77E-10	-1.051105764	0.404	0.541	1.10E-05	17
WIPF1.15	1.11E-09	-1.093744104	0.171	0.346	2.55E-05	17
TUBA4A.14	2.02E-09	-1.20936644	0.098	0.275	4.65E-05	17
PRDM1.11	2.41E-09	-1.28626831	0.119	0.294	5.54E-05	17
LCK.17	4.37E-09	-1.125083639	0.124	0.29	0.000100378	17
EVL.16	4.65E-09	-1.193135836	0.389	0.497	0.000106949	17
HLA-DPB1.13	5.67E-09	-2.167375451	0.705	0.631	0.000130314	17
COTL1.11	6.20E-09	-1.410585046	0.326	0.436	0.000142504	17
IL10RA.13	6.54E-09	-0.919289685	0.088	0.261	0.00015031	17
CD27.15	1.21E-08	-1.478979516	0.109	0.27	0.00027823	17
CD96.17	1.23E-08	-1.057244395	0.088	0.251	0.000283111	17
NKG7.17	1.44E-08	-2.465997409	0.316	0.429	0.000331841	17
IL7R.15	2.03E-08	-2.036666184	0.15	0.305	0.00046631	17
DUSP1.12	2.26E-08	-0.912516035	0.876	0.792	0.000519858	17
LITAF.11	3.09E-08	-1.125784204	0.228	0.369	0.000710345	17
PRKCH.12	3.70E-08	-0.876380088	0.093	0.256	0.000850122	17
OCIAD2.13	4.09E-08	-1.060618655	0.104	0.263	0.000939159	17
CDC42SE2.9	5.65E-08	-0.990451337	0.161	0.317	0.001299137	17
CTSS.13	5.70E-08	-1.527689288	0.233	0.365	0.001309866	17
RPS5.8	6.29E-08	-0.575863758	0.933	0.84	0.00144451	17
TPM3.7	6.34E-08	-0.998575275	0.342	0.455	0.001456688	17
APOBEC3G.14	6.35E-08	-1.147090895	0.119	0.274	0.001460128	17
DOCK8.13	6.55E-08	-0.894624936	0.14	0.293	0.001505577	17
FTL.14	8.99E-08	-1.387277306	0.99	0.936	0.00206567	17
SARAF.9	1.07E-07	-0.912320615	0.819	0.676	0.002460903	17
RPL24.10	1.69E-07	-0.513021923	0.984	0.851	0.003880476	17
RGS2.15	3.18E-07	-1.593317395	0.207	0.337	0.007300958	17
REL.11	3.25E-07	-1.078060527	0.192	0.324	0.007468355	17
OAZ1.11	3.30E-07	-0.691382968	0.85	0.734	0.007579826	17
RPLP0.12	3.47E-07	-0.670120943	0.938	0.842	0.007979997	17
MYO1F.12	3.66E-07	-0.848860937	0.13	0.273	0.008406631	17
DDIT4.11	3.81E-07	-1.38001491	0.435	0.519	0.008758634	17
RNASET2.15	5.03E-07	-1.415919544	0.254	0.362	0.011568669	17
SAT1.12	6.53E-07	-1.477544548	0.694	0.63	0.015011196	17
HLA-DRB5.14	8.91E-07	-2.062360392	0.269	0.375	0.020467686	17
HLA-E.15	9.56E-07	-0.576886103	0.953	0.787	0.021966781	17
HLA-DMA.12	1.10E-06	-1.342336942	0.166	0.289	0.025375372	17
ISG20.12	1.46E-06	-1.082252047	0.249	0.363	0.033620701	17

HLA-DQB1.13	1.94E-06	-1.887895758	0.321	0.393	0.044533947	17
MKI67	0	2.907919202	0.721	0.011	0	18
TOP2A	0	2.758690053	0.65	0.012	0	18
KIAA0101	0	2.684551259	0.732	0.031	0	18
NUSAP1	0	2.674184871	0.727	0.032	0	18
CENPF	0	2.582304479	0.683	0.036	0	18
UBE2C	0	2.48300096	0.574	0.007	0	18
ASPM	0	2.345713834	0.579	0.008	0	18
TYMS	0	2.194775661	0.694	0.025	0	18
BIRC5	0	2.081189145	0.541	0.009	0	18
RRM2	0	1.913526304	0.53	0.005	0	18
TPX2	0	1.798270984	0.541	0.01	0	18
CDK1	0	1.745753492	0.552	0.009	0	18
CCNA2	0	1.707820849	0.514	0.009	0	18
ZWINT	0	1.668354294	0.601	0.017	0	18
CENPM	0	1.58980198	0.574	0.028	0	18
CDKN3	0	1.496955314	0.47	0.01	0	18
MAD2L1	0	1.490881527	0.601	0.029	0	18
CENPU	0	1.453511516	0.568	0.014	0	18
PRC1	0	1.441510155	0.497	0.016	0	18
AURKB	0	1.423879014	0.475	0.006	0	18
CLSPN	0	1.366523259	0.552	0.014	0	18
ASF1B	0	1.346250084	0.552	0.01	0	18
TK1	0	1.343030846	0.443	0.012	0	18
NUF2	0	1.309348953	0.404	0.006	0	18
CDCA8	0	1.27394813	0.361	0.004	0	18
UBE2T	0	1.175421653	0.563	0.019	0	18
CDC20	0	1.154220972	0.301	0.003	0	18
GTSE1	0	1.142011673	0.415	0.005	0	18
FAM111B	0	1.132849513	0.372	0.005	0	18
TCF19	0	1.132443417	0.47	0.015	0	18
KIF15	0	1.118920376	0.383	0.003	0	18
CASC5	0	1.114675186	0.404	0.008	0	18
SPC25	0	1.112559238	0.393	0.002	0	18
CCNB2	0	1.100260372	0.333	0.004	0	18
HIST1H1B	0	1.073746439	0.29	0.003	0	18
CDT1	0	1.073358616	0.432	0.009	0	18
BRCA1	0	1.064876052	0.448	0.015	0	18
KIF23	0	1.063366015	0.344	0.002	0	18
CENPW	0	1.055758705	0.47	0.017	0	18
FEN1	0	1.053397645	0.481	0.017	0	18
RAD51AP1	0	1.047044819	0.437	0.01	0	18
KIFC1	0	1.032863008	0.421	0.006	0	18
DLGAP5	0	1.01723002	0.35	0.002	0	18
KIF11	0	1.009705231	0.339	0.008	0	18
CENPA	0	0.999753851	0.333	0.003	0	18
CKAP2L	0	0.985111087	0.404	0.006	0	18
SPC24	0	0.982679528	0.328	0.003	0	18
KIF2C	0	0.975423489	0.29	0.002	0	18
HJURP	0	0.947011754	0.328	0.001	0	18
CENPN	0	0.927878083	0.437	0.012	0	18
CDCA3	0	0.925085421	0.317	0.003	0	18
CEP55	0	0.918990608	0.426	0.011	0	18
SGOL1	0	0.908457583	0.339	0.004	0	18
ESCO2	0	0.880495701	0.35	0.007	0	18
HMMR	0	0.855140635	0.322	0.004	0	18
CDCA5	0	0.847205167	0.344	0.002	0	18
NCAPG	0	0.831850158	0.317	0.005	0	18

FANCI	0	0.814370732	0.399	0.012	0	18
UHRF1	0	0.799722289	0.344	0.006	0	18
KIF14	0	0.765882674	0.284	0.004	0	18
RAD51	0	0.765218425	0.268	0.003	0	18
TROAP	0	0.76203194	0.295	0.003	0	18
PBK	0	0.713710934	0.279	0.001	0	18
MELK	0	0.625112514	0.284	0.003	0	18
DEPDC1B	0	0.618206172	0.273	0.003	0	18
MCM10	0	0.60535578	0.268	0.002	0	18
MND1	0	0.6046414	0.251	0.004	0	18
CDC45	0	0.543171993	0.251	0.002	0	18
SPAG5	0	0.503798856	0.268	0.004	0	18
SGOL2	8.71E-291	1.05552345	0.421	0.017	2.00E-286	18
KIAA1524	2.70E-287	0.706158892	0.377	0.014	6.21E-283	18
CENPK	4.10E-278	1.056061195	0.475	0.024	9.42E-274	18
NCAPG2	4.58E-276	0.928985923	0.344	0.012	1.05E-271	18
BUB1	1.76E-275	0.577868626	0.284	0.007	4.04E-271	18
ATAD5	8.50E-270	1.204600596	0.53	0.031	1.95E-265	18
SHCBP1	4.71E-267	0.52471934	0.284	0.008	1.08E-262	18
MXD3	3.33E-263	0.699010641	0.328	0.011	7.64E-259	18
GGH	5.79E-262	1.125788888	0.481	0.027	1.33E-257	18
CENPE	9.91E-256	1.587709221	0.492	0.029	2.28E-251	18
STMN1.1	1.64E-246	3.784544768	0.891	0.128	3.77E-242	18
ARHGAP11A	4.40E-244	0.980057117	0.388	0.018	1.01E-239	18
FBXO5	5.75E-242	0.957706751	0.35	0.014	1.32E-237	18
NCAPH	3.27E-241	0.507433582	0.257	0.007	7.51E-237	18
ECT2	5.82E-229	0.726316633	0.284	0.01	1.34E-224	18
SMC2	1.40E-227	1.71488047	0.71	0.074	3.21E-223	18
RACGAP1	1.87E-224	0.746931548	0.339	0.015	4.29E-220	18
HELLS	7.47E-215	1.208646803	0.492	0.034	1.72E-210	18
MCM4	2.80E-213	0.69060459	0.344	0.016	6.44E-209	18
CKS1B.1	1.77E-210	2.08203086	0.721	0.083	4.07E-206	18
SAC3D1	2.73E-202	0.906566897	0.372	0.02	6.28E-198	18
EZH2	2.70E-201	1.379783943	0.53	0.043	6.20E-197	18
BRCA2	6.49E-201	0.9708219	0.426	0.027	1.49E-196	18
CHEK1	1.01E-200	0.579261784	0.306	0.013	2.33E-196	18
MCM2	1.16E-198	0.6146778	0.284	0.012	2.66E-194	18
ARHGAP11B	2.23E-191	1.099581793	0.443	0.031	5.13E-187	18
GINS2	1.16E-190	0.611014444	0.257	0.01	2.66E-186	18
RMI2	2.36E-190	0.573549478	0.257	0.01	5.43E-186	18
CENPH	2.39E-189	0.932142068	0.421	0.028	5.50E-185	18
ATAD2	9.07E-189	1.400127147	0.585	0.056	2.09E-184	18
CDCA7	3.20E-187	0.630643942	0.262	0.01	7.37E-183	18
AURKA	6.68E-181	1.074335154	0.257	0.01	1.54E-176	18
CCDC34	1.24E-180	1.014087381	0.41	0.028	2.84E-176	18
HMGB3	2.45E-178	0.932400698	0.372	0.024	5.63E-174	18
PCNA	6.73E-174	2.131749508	0.694	0.093	1.55E-169	18
C21orf58	7.59E-173	0.583974347	0.251	0.01	1.74E-168	18
MCM7	5.47E-172	1.355644156	0.508	0.047	1.26E-167	18
PTTG1.1	1.20E-169	2.076555909	0.683	0.088	2.75E-165	18
LMNB1	1.20E-166	1.316595455	0.536	0.053	2.77E-162	18
CDCA4	8.08E-165	1.015867477	0.426	0.033	1.86E-160	18
BARD1	1.08E-163	0.785535594	0.404	0.03	2.49E-159	18
NDC80	2.98E-163	0.809198005	0.366	0.024	6.85E-159	18
DNAJC9	7.68E-162	1.187608135	0.628	0.075	1.76E-157	18
DHFR	4.56E-158	0.881481687	0.393	0.03	1.05E-153	18
DTYMK	4.11E-156	1.199701556	0.525	0.055	9.45E-152	18
FANCD2	8.04E-150	0.69466772	0.279	0.015	1.85E-145	18

NCAPD2	1.27E-143	0.641210338	0.328	0.022	2.93E-139	18
CKS2	5.45E-136	2.032467865	0.585	0.081	1.25E-131	18
WDR76	6.62E-136	0.675224717	0.339	0.025	1.52E-131	18
WHSC1	2.41E-133	0.83442617	0.437	0.043	5.53E-129	18
PHF19	3.47E-131	0.741200022	0.399	0.037	7.97E-127	18
TACC3	2.35E-130	1.280192999	0.519	0.063	5.41E-126	18
H2AFX	7.47E-130	1.451346508	0.519	0.064	1.72E-125	18
MCM6	1.69E-129	0.666659599	0.339	0.027	3.89E-125	18
RRM1	6.10E-129	0.980079063	0.443	0.047	1.40E-124	18
CHAF1A	2.72E-126	0.65479222	0.317	0.024	6.25E-122	18
SNRNP25	6.27E-126	0.950357687	0.492	0.058	1.44E-121	18
MCM3	8.59E-124	1.091327842	0.503	0.062	1.97E-119	18
SMC4.2	1.09E-123	1.949418419	0.732	0.144	2.50E-119	18
KIF20B	5.65E-123	1.392326915	0.563	0.077	1.30E-118	18
RFC3	2.21E-118	0.585096463	0.29	0.021	5.08E-114	18
GMNN	1.02E-115	0.702273837	0.421	0.046	2.35E-111	18
RFC4	2.00E-115	0.617354296	0.328	0.028	4.60E-111	18
TUBB.12	2.07E-115	3.0815982	0.934	0.34	4.77E-111	18
HIRIP3	5.98E-115	0.847999259	0.443	0.051	1.37E-110	18
RP11-545E17.3	1.07E-113	0.624059855	0.306	0.024	2.46E-109	18
BCL2L12	1.53E-112	0.690870939	0.361	0.035	3.52E-108	18
TMEM106C.1	1.06E-110	1.178013471	0.514	0.071	2.43E-106	18
YEATS4	3.51E-109	1.082133373	0.552	0.083	8.07E-105	18
TUBA1B.11	2.74E-107	3.00733331	0.945	0.407	6.29E-103	18
RNASEH2A	6.60E-106	0.731936133	0.399	0.045	1.52E-101	18
HMGB2.13	1.10E-105	2.827967255	0.956	0.47	2.52E-101	18
NCAPD3	8.26E-105	0.674790293	0.35	0.035	1.90E-100	18
LIG1	7.70E-102	0.72838624	0.355	0.037	1.77E-97	18
SKA2	1.42E-101	1.047107903	0.568	0.093	3.25E-97	18
USP1	8.81E-100	1.131230934	0.607	0.11	2.02E-95	18
KIF22	1.66E-99	0.931663865	0.492	0.072	3.81E-95	18
TTF2	8.61E-96	0.73824243	0.388	0.046	1.98E-91	18
VRK1	2.51E-95	0.705407138	0.437	0.058	5.76E-91	18
TMPO.2	4.47E-92	1.444350718	0.661	0.145	1.03E-87	18
CEP152	1.47E-89	0.610842921	0.301	0.03	3.38E-85	18
H2AFZ.5	1.86E-88	2.360385029	0.891	0.383	4.28E-84	18
H2AFV.4	3.42E-88	1.81195539	0.831	0.281	7.86E-84	18
UBE2S	5.41E-88	0.954485885	0.443	0.064	1.24E-83	18
POLD3	1.16E-87	0.59189043	0.339	0.039	2.66E-83	18
FANCA	1.08E-86	0.500849146	0.29	0.029	2.49E-82	18
CDC25B	1.16E-86	0.703633896	0.339	0.039	2.67E-82	18
PXMP2	2.27E-85	0.573528242	0.306	0.033	5.23E-81	18
HIST1H1D	2.37E-85	1.433970403	0.443	0.068	5.45E-81	18
HIST1H4C.2	7.16E-85	3.805682202	0.727	0.214	1.65E-80	18
ANP32E.1	3.29E-84	1.450403589	0.667	0.159	7.55E-80	18
DNMT1	3.54E-84	1.285414289	0.645	0.142	8.13E-80	18
PRIM1	1.70E-83	0.623954211	0.333	0.039	3.92E-79	18
TEX30	4.83E-83	0.67392	0.322	0.037	1.11E-78	18
HMGN2.5	2.54E-81	2.362268905	0.913	0.495	5.84E-77	18
NUDT1	1.25E-80	1.025965223	0.563	0.111	2.86E-76	18
KPNA2	1.36E-80	1.536798849	0.475	0.084	3.12E-76	18
SAE1.1	1.43E-79	0.831703644	0.481	0.082	3.30E-75	18
RPA3.2	1.86E-79	1.16238783	0.71	0.179	4.28E-75	18
HIST2H2AC	1.57E-78	0.925945483	0.328	0.041	3.60E-74	18
CDKN2C.1	4.02E-78	0.920143861	0.437	0.07	9.23E-74	18
TUBB4B.1	6.18E-76	1.552376365	0.601	0.137	1.42E-71	18
RPL39L	5.39E-74	0.753426447	0.344	0.046	1.24E-69	18
SMC1A.1	1.27E-73	1.084569184	0.639	0.155	2.92E-69	18

CKAP2	1.35E-73	1.020555986	0.443	0.074	3.09E-69	18
ACYP1.1	7.69E-72	0.719710709	0.399	0.061	1.77E-67	18
DDX39A	9.55E-72	1.096699423	0.574	0.128	2.19E-67	18
MCM5.1	6.61E-71	0.886402366	0.448	0.081	1.52E-66	18
CARHSP1.4	2.42E-69	1.130886991	0.732	0.199	5.55E-65	18
CBX5	3.49E-69	0.800906246	0.563	0.121	8.03E-65	18
HIST1H1E	1.90E-68	0.97855167	0.311	0.042	4.36E-64	18
MAD2L2	3.60E-68	0.88906346	0.443	0.079	8.28E-64	18
C12orf75.4	2.04E-67	1.085587784	0.634	0.151	4.69E-63	18
RAD51C	1.22E-66	0.707345785	0.388	0.063	2.81E-62	18
WDR34.1	1.33E-66	0.568391192	0.284	0.035	3.07E-62	18
CALM3.7	4.65E-66	1.370981489	0.831	0.306	1.07E-61	18
RBBP7.1	7.23E-66	1.13981766	0.634	0.163	1.66E-61	18
ORC6	7.25E-66	0.533779541	0.399	0.064	1.67E-61	18
PTMA.8	4.68E-65	1.084077322	0.995	0.916	1.08E-60	18
CRIP1.14	1.19E-64	1.813767264	0.798	0.287	2.73E-60	18
HMGB1.11	1.28E-64	1.757769263	0.918	0.732	2.93E-60	18
MZT1	8.90E-64	1.004591747	0.486	0.1	2.05E-59	18
RANBP1.3	1.28E-62	1.325413871	0.76	0.263	2.94E-58	18
TOPBP1	2.82E-62	0.521780562	0.328	0.049	6.48E-58	18
DEK.7	1.51E-61	1.723298367	0.798	0.359	3.46E-57	18
DUT.7	9.26E-61	1.770258788	0.71	0.257	2.13E-56	18
CKAP5	1.36E-60	0.510846381	0.284	0.039	3.13E-56	18
SLBP	2.30E-60	0.886080208	0.557	0.135	5.28E-56	18
SUZ12	7.78E-60	0.771979125	0.459	0.094	1.79E-55	18
FABP5.4	1.57E-59	1.281927743	0.667	0.201	3.60E-55	18
RAD21.2	1.02E-58	1.463315463	0.699	0.231	2.34E-54	18
SMC3.1	4.71E-58	1.026981335	0.672	0.205	1.08E-53	18
MIR155HG.1	2.54E-57	0.736030204	0.372	0.064	5.83E-53	18
PSIP1.3	1.47E-56	1.018438673	0.678	0.198	3.38E-52	18
HN1.4	2.85E-56	1.413216797	0.699	0.233	6.56E-52	18
FAM111A.2	3.51E-56	1.003167689	0.557	0.144	8.08E-52	18
NUCKS1.7	6.00E-56	1.39397436	0.852	0.392	1.38E-51	18
CCDC167.2	6.25E-56	0.946228049	0.628	0.169	1.44E-51	18
EBP	2.18E-55	0.826971677	0.454	0.096	5.02E-51	18
NUP37	6.73E-55	0.501427992	0.322	0.052	1.55E-50	18
ANP32B.6	6.88E-55	1.585704719	0.781	0.335	1.58E-50	18
CBX1.1	9.01E-55	0.648013588	0.459	0.1	2.07E-50	18
RAN.5	1.22E-54	1.351427295	0.858	0.42	2.81E-50	18
HLTF	1.46E-53	0.572155017	0.328	0.055	3.36E-49	18
IDH2.6	2.25E-53	1.143074817	0.727	0.254	5.18E-49	18
LSM2	3.33E-53	0.79994198	0.612	0.171	7.65E-49	18
SIVA1	5.47E-53	1.016833805	0.683	0.229	1.26E-48	18
CDKN2D	1.17E-52	1.035044652	0.311	0.051	2.68E-48	18
SUPT16H	2.65E-52	0.709512878	0.536	0.135	6.09E-48	18
APOBEC3H	4.02E-52	0.657263452	0.333	0.056	9.24E-48	18
HNRNPAB.1	6.05E-52	0.943565585	0.557	0.154	1.39E-47	18
LBR.1	9.63E-52	0.85873705	0.563	0.15	2.21E-47	18
ZDHHC12	1.54E-51	0.842413922	0.475	0.113	3.54E-47	18
HMGXB4	2.02E-50	0.53373056	0.459	0.103	4.63E-46	18
TFDP1	5.97E-50	0.597524204	0.383	0.078	1.37E-45	18
HDAC1.1	8.17E-50	0.636780004	0.519	0.129	1.88E-45	18
LAG3.2	1.24E-49	0.842539027	0.443	0.096	2.85E-45	18
HPRT1	1.32E-49	0.578635013	0.481	0.116	3.04E-45	18
DCTN3.2	1.47E-49	0.875487548	0.661	0.205	3.37E-45	18
HMGN1.6	1.74E-49	1.232098077	0.88	0.499	4.01E-45	18
C19orf48	2.90E-49	0.510246258	0.311	0.054	6.67E-45	18
SH2D1A.2	3.04E-49	0.88571488	0.552	0.139	6.99E-45	18

SNRPD1.1	1.31E-48	0.823109515	0.694	0.231	3.00E-44	18
TNFRSF9.4	3.16E-48	1.001956058	0.437	0.097	7.27E-44	18
MPHOSPH9	1.95E-47	0.644303131	0.366	0.073	4.49E-43	18
HSPB11.1	3.41E-47	0.748710618	0.601	0.178	7.84E-43	18
CEP78	3.64E-47	0.688686678	0.333	0.061	8.36E-43	18
ACTL6A.1	8.87E-47	0.610552699	0.322	0.06	2.04E-42	18
SNRPF.3	1.07E-46	0.999828034	0.776	0.31	2.46E-42	18
LSM4.1	4.95E-46	1.024709486	0.612	0.203	1.14E-41	18
BUB3.12	1.17E-45	0.940138005	0.738	0.265	2.69E-41	18
HDGF	1.18E-45	0.579354325	0.366	0.077	2.72E-41	18
NASP.1	2.44E-45	0.915205631	0.634	0.214	5.61E-41	18
CDK5RAP2	3.66E-45	0.501616947	0.333	0.064	8.41E-41	18
UQCC2	4.45E-45	0.747429418	0.475	0.124	1.02E-40	18
TOX.3	6.08E-45	0.595732658	0.448	0.102	1.40E-40	18
CTCF	1.34E-44	0.573931282	0.432	0.102	3.08E-40	18
SNRPB.3	1.51E-44	1.114725946	0.781	0.344	3.46E-40	18
ACTB.13	1.63E-44	1.034338679	0.967	0.928	3.75E-40	18
STRA13	3.33E-44	0.889950717	0.481	0.126	7.66E-40	18
NUDT21	3.61E-44	0.632881637	0.497	0.134	8.30E-40	18
HNRNPD.3	3.72E-44	1.034470797	0.672	0.241	8.55E-40	18
DUSP4.8	8.93E-44	1.038492409	0.694	0.235	2.05E-39	18
COX8A.6	9.22E-44	1.114070309	0.88	0.494	2.12E-39	18
CDK2AP2.1	1.23E-43	0.752029818	0.541	0.154	2.84E-39	18
MIS18BP1.4	2.14E-43	0.763524892	0.639	0.204	4.93E-39	18
CFL1.6	2.99E-43	0.963402401	0.929	0.744	6.87E-39	18
LSM5.2	3.77E-43	0.801274815	0.639	0.215	8.67E-39	18
PMVK.1	5.06E-43	0.777825849	0.492	0.13	1.16E-38	18
SRSF10.5	6.11E-43	0.77901357	0.689	0.241	1.40E-38	18
BAZ1B.2	4.39E-42	0.671201064	0.546	0.161	1.01E-37	18
HAT1	5.17E-42	0.500513786	0.443	0.111	1.19E-37	18
HIST1H1C.2	5.85E-42	1.088323323	0.464	0.127	1.34E-37	18
SSRP1.1	6.35E-42	0.671362446	0.47	0.126	1.46E-37	18
CEP57	1.00E-41	0.608036053	0.481	0.128	2.31E-37	18
ARPC5L.6	2.00E-41	0.922810837	0.661	0.226	4.59E-37	18
ANXA6.14	1.25E-40	0.796296463	0.694	0.244	2.87E-36	18
ANKRD36C.2	1.81E-40	0.782512723	0.563	0.172	4.15E-36	18
TUBA1C.1	1.86E-40	0.892681092	0.421	0.109	4.28E-36	18
SIT1.4	5.05E-40	0.517480234	0.486	0.125	1.16E-35	18
H2AFY.9	7.17E-40	1.020483599	0.721	0.278	1.65E-35	18
SRRT	8.14E-40	0.654386226	0.355	0.08	1.87E-35	18
PPIA.7	1.03E-39	1.013435992	0.918	0.644	2.38E-35	18
PFN1.11	1.52E-39	1.071850943	0.913	0.758	3.49E-35	18
ACTG1.10	2.58E-39	1.145907607	0.923	0.743	5.93E-35	18
CDKN2A	3.90E-39	0.512797926	0.317	0.065	8.96E-35	18
HNRNPR.6	4.36E-39	0.843697013	0.716	0.27	1.00E-34	18
PPM1G.2	4.73E-39	0.773297656	0.634	0.221	1.09E-34	18
SLC25A5.8	5.31E-39	0.990538832	0.781	0.38	1.22E-34	18
KIF2A.1	7.13E-39	0.631256231	0.448	0.119	1.64E-34	18
FAF1	7.77E-39	0.685838818	0.306	0.062	1.79E-34	18
ARL6IP1.5	1.15E-38	1.697180039	0.705	0.316	2.64E-34	18
ARL6IP6	3.83E-38	0.550799556	0.404	0.102	8.79E-34	18
BTG3.2	4.12E-38	0.719634979	0.557	0.169	9.46E-34	18
CD8B.3	9.50E-38	0.804398082	0.574	0.179	2.18E-33	18
PPP1CA.4	9.52E-38	0.877970063	0.781	0.359	2.19E-33	18
CD8A.3	1.19E-37	0.917907337	0.65	0.224	2.74E-33	18
NONO.1	1.71E-37	0.632487019	0.65	0.236	3.92E-33	18
RAB27A.2	1.84E-37	0.632901716	0.53	0.158	4.23E-33	18
PMF1.3	1.99E-37	0.580195854	0.536	0.163	4.57E-33	18

NFATC2IP	4.55E-37	0.519789323	0.47	0.131	1.05E-32	18
PSMB2	5.24E-37	0.82622106	0.656	0.238	1.20E-32	18
XPO1.2	6.92E-37	0.511743558	0.492	0.144	1.59E-32	18
HNRNPF.2	7.16E-37	0.984716952	0.754	0.35	1.65E-32	18
SRP9.4	8.43E-37	0.826795639	0.754	0.306	1.94E-32	18
BANF1.2	8.48E-37	0.803771473	0.634	0.235	1.95E-32	18
HNRNPA2B1.8	8.82E-37	1.011719773	0.874	0.624	2.03E-32	18
GZMA.17	1.35E-36	1.326231573	0.803	0.414	3.10E-32	18
ASH2L	2.20E-36	0.551444035	0.284	0.058	5.06E-32	18
HNRNPA3.6	4.70E-36	1.01248322	0.82	0.459	1.08E-31	18
VCAM1.2	5.40E-36	0.640523851	0.377	0.093	1.24E-31	18
DDB2.2	7.41E-36	0.612275168	0.475	0.137	1.70E-31	18
VBP1	1.23E-35	0.617933584	0.481	0.142	2.82E-31	18
CMC2	5.43E-35	0.525880268	0.481	0.141	1.25E-30	18
CBX3.3	6.62E-35	0.880561524	0.738	0.339	1.52E-30	18
SUB1.6	7.25E-35	1.086520788	0.869	0.57	1.67E-30	18
PSMC3.3	8.68E-35	0.65437469	0.574	0.198	2.00E-30	18
CNTRL.3	1.13E-34	0.784138845	0.497	0.158	2.60E-30	18
TAP1.7	1.19E-34	0.700372077	0.656	0.241	2.73E-30	18
COX5A.6	2.76E-34	0.741546701	0.732	0.316	6.34E-30	18
MZT2B.7	4.76E-34	0.843204029	0.776	0.356	1.09E-29	18
RALY	4.76E-34	0.651741448	0.628	0.236	1.09E-29	18
UBE2A.1	6.43E-34	0.573840603	0.503	0.163	1.48E-29	18
PDIA6.5	6.52E-34	0.723566095	0.727	0.3	1.50E-29	18
RUNX3.4	8.98E-34	0.684000475	0.612	0.204	2.06E-29	18
DCXR	9.94E-34	0.600701086	0.503	0.156	2.28E-29	18
PIN1.2	1.16E-33	0.573202071	0.601	0.209	2.66E-29	18
CD82.2	2.39E-33	0.550441636	0.404	0.109	5.49E-29	18
GAPDH.12	7.62E-33	0.722519935	0.956	0.865	1.75E-28	18
PARP1.2	9.38E-33	0.780691312	0.601	0.219	2.16E-28	18
MAPRE1.1	1.31E-32	0.558837142	0.536	0.179	3.01E-28	18
SFPQ.8	1.90E-32	0.930557197	0.71	0.306	4.37E-28	18
MAGOH	2.33E-32	0.711821841	0.568	0.199	5.36E-28	18
SNRPD3	3.42E-32	0.603315789	0.634	0.234	7.86E-28	18
APOBEC3C.3	3.83E-32	0.702300496	0.568	0.194	8.81E-28	18
ILF2	7.23E-32	0.654594458	0.525	0.181	1.66E-27	18
FTL.15	1.00E-31	-1.644100491	0.929	0.937	2.31E-27	18
CORO1A.14	1.04E-31	1.13782345	0.847	0.561	2.40E-27	18
PSMB9.9	1.19E-31	0.871928115	0.831	0.479	2.72E-27	18
RPL34.11	1.55E-31	-0.767190566	0.978	0.953	3.55E-27	18
SRSF2.12	2.09E-31	0.910318083	0.798	0.418	4.80E-27	18
HSD17B10	5.43E-31	0.560199946	0.53	0.178	1.25E-26	18
RAC2.16	1.16E-30	0.907349186	0.76	0.367	2.66E-26	18
EIF4A3.1	1.46E-30	0.512218134	0.497	0.165	3.35E-26	18
BLVRA.2	1.90E-30	0.636241959	0.448	0.139	4.37E-26	18
RPL13.9	4.26E-30	-0.724451212	0.973	0.965	9.78E-26	18
KPNB1	6.75E-30	0.523024157	0.601	0.224	1.55E-25	18
HNRNPM.4	7.12E-30	0.795908999	0.689	0.314	1.64E-25	18
SNRPG.5	7.49E-30	0.741321721	0.721	0.353	1.72E-25	18
RPS14.10	8.11E-30	-0.721857326	0.967	0.946	1.86E-25	18
NUCB2.2	8.58E-30	0.710231773	0.497	0.167	1.97E-25	18
RHEB.3	9.83E-30	0.706382002	0.585	0.228	2.26E-25	18
PMAIP1.6	1.14E-29	1.00560045	0.546	0.198	2.62E-25	18
LSM3.3	1.27E-29	0.621702482	0.634	0.252	2.92E-25	18
APOBEC3G.15	1.57E-29	0.835652682	0.645	0.266	3.61E-25	18
SPN.3	1.70E-29	0.592746572	0.475	0.154	3.91E-25	18
NDUFC2.7	2.30E-29	0.677004891	0.694	0.29	5.27E-25	18
SRSF3.3	2.77E-29	0.799701876	0.781	0.417	6.36E-25	18

STAG1	2.88E-29	0.663279897	0.383	0.109	6.62E-25	18
BLOC1S1.8	6.45E-29	0.74632216	0.716	0.342	1.48E-24	18
CD27.16	6.54E-29	0.830192191	0.645	0.262	1.50E-24	18
HP1BP3.8	7.04E-29	0.776715206	0.656	0.28	1.62E-24	18
NOP56	8.16E-29	0.563046832	0.459	0.148	1.87E-24	18
PSMB8.9	1.06E-28	0.927322466	0.77	0.376	2.43E-24	18
PGAM1.9	1.07E-28	0.649561393	0.749	0.356	2.45E-24	18
CXCR3.1	1.08E-28	0.596161227	0.361	0.097	2.48E-24	18
WNK1.10	1.13E-28	0.685058434	0.617	0.244	2.60E-24	18
SDF2L1.1	1.35E-28	0.619224374	0.47	0.157	3.10E-24	18
TPR.8	1.50E-28	0.678966009	0.694	0.303	3.44E-24	18
YWHAQ.8	2.01E-28	0.794398985	0.732	0.344	4.62E-24	18
HNRNPA0.6	2.24E-28	0.928785098	0.803	0.398	5.16E-24	18
PTPN11	3.49E-28	0.585267919	0.284	0.069	8.01E-24	18
TPM3.8	3.59E-28	0.763906564	0.836	0.448	8.24E-24	18
LAT.4	3.93E-28	0.50242162	0.503	0.163	9.03E-24	18
NDUFB3.3	6.34E-28	0.56707801	0.623	0.249	1.46E-23	18
PLP2.9	6.74E-28	0.726592954	0.678	0.29	1.55E-23	18
PSMB6.4	7.86E-28	0.653764262	0.65	0.272	1.81E-23	18
C9orf142.12	1.26E-27	0.686538826	0.634	0.256	2.89E-23	18
TIMM10	1.34E-27	0.518353966	0.388	0.118	3.08E-23	18
CRTAM.2	1.43E-27	0.722731669	0.339	0.092	3.28E-23	18
WDR1.5	1.44E-27	0.65720564	0.634	0.262	3.31E-23	18
PSMA4.5	1.56E-27	0.566601612	0.727	0.319	3.59E-23	18
TUBA4A.15	2.09E-27	0.720540376	0.667	0.267	4.80E-23	18
GTF3C6.2	2.49E-27	0.559323888	0.508	0.181	5.73E-23	18
LINC00152.11	4.33E-27	0.594689512	0.71	0.312	9.96E-23	18
IFI16.7	4.40E-27	0.870014107	0.82	0.476	1.01E-22	18
SUMO3.1	4.76E-27	0.551031701	0.59	0.225	1.09E-22	18
PSMA5.2	4.83E-27	0.791363442	0.546	0.211	1.11E-22	18
CD3G.17	6.95E-27	0.626192526	0.71	0.288	1.60E-22	18
UBALD2.1	8.21E-27	0.616031242	0.355	0.103	1.89E-22	18
LYST.5	9.53E-27	0.5608178	0.585	0.222	2.19E-22	18
ATP5G3.5	1.01E-26	0.653682663	0.798	0.402	2.32E-22	18
PSME2.7	1.13E-26	0.842513418	0.765	0.408	2.59E-22	18
FAM195B	1.48E-26	0.522993018	0.497	0.175	3.40E-22	18
CYB5B.1	2.60E-26	0.564429129	0.443	0.145	5.96E-22	18
PRPF4B.2	2.63E-26	0.612985846	0.59	0.232	6.03E-22	18
NDUFS6.4	4.68E-26	0.648663862	0.667	0.294	1.08E-21	18
NDUFA2.5	5.66E-26	0.751866305	0.716	0.319	1.30E-21	18
TALDO1.5	6.32E-26	0.525958883	0.628	0.258	1.45E-21	18
CHCHD2.4	1.02E-25	0.757382443	0.874	0.606	2.35E-21	18
TRAC.18	1.05E-25	0.987891897	0.787	0.427	2.41E-21	18
PARK7.10	1.33E-25	0.721566336	0.825	0.488	3.06E-21	18
ITM2A.17	2.93E-25	0.86196627	0.678	0.306	6.73E-21	18
HNRNPA1L2.2	3.14E-25	0.598933225	0.448	0.156	7.22E-21	18
RBX1.6	3.20E-25	0.763650024	0.749	0.37	7.35E-21	18
XRCC5.6	3.53E-25	0.590583975	0.694	0.319	8.11E-21	18
PKM.10	3.88E-25	0.717577481	0.787	0.429	8.92E-21	18
NDUFB11.3	4.94E-25	0.543882242	0.749	0.363	1.14E-20	18
PSMG2.3	4.96E-25	0.537746416	0.563	0.22	1.14E-20	18
NKG7.18	6.58E-25	0.784016056	0.792	0.422	1.51E-20	18
RPL41.13	9.03E-25	-0.616439521	0.984	0.976	2.08E-20	18
COX17.10	1.00E-24	0.500759907	0.699	0.313	2.30E-20	18
IFI27L2.6	1.11E-24	0.549989137	0.661	0.301	2.54E-20	18
LCP1.15	1.16E-24	0.743840974	0.76	0.399	2.67E-20	18
RP11-160E2.6.1	1.26E-24	0.71301342	0.399	0.131	2.90E-20	18
GALM.4	1.35E-24	0.513178072	0.464	0.161	3.09E-20	18

PSMA3.4	1.80E-24	0.527810205	0.546	0.214	4.13E-20	18
ATPIF1.5	2.15E-24	0.588239807	0.798	0.415	4.94E-20	18
RPL10.11	4.16E-24	-0.582369194	0.984	0.975	9.55E-20	18
CALM2.7	4.67E-24	0.910028271	0.891	0.628	1.07E-19	18
SET.5	4.78E-24	0.572612813	0.738	0.368	1.10E-19	18
ERH.5	5.15E-24	0.688356636	0.705	0.356	1.18E-19	18
NABP2	5.85E-24	0.545929311	0.268	0.069	1.34E-19	18
RHNO1	7.00E-24	0.56436441	0.273	0.072	1.61E-19	18
CD3D.18	7.54E-24	0.892752583	0.809	0.47	1.73E-19	18
SUMO2.6	1.11E-23	0.679859104	0.852	0.604	2.56E-19	18
XRCC6.3	1.16E-23	0.675309871	0.661	0.301	2.66E-19	18
PTPN7.5	1.23E-23	0.503454221	0.574	0.217	2.82E-19	18
HMGN3.5	1.36E-23	0.601746477	0.661	0.301	3.13E-19	18
HNRNPA1.7	1.38E-23	0.691388553	0.929	0.775	3.16E-19	18
RPS27.11	1.52E-23	-0.66178435	0.978	0.975	3.49E-19	18
ANXA5.12	1.84E-23	0.651610657	0.721	0.347	4.23E-19	18
UBB.9	1.94E-23	0.847394617	0.907	0.717	4.47E-19	18
PSMD8.6	2.77E-23	0.512950243	0.672	0.305	6.37E-19	18
NAA38.9	3.98E-23	0.675354957	0.623	0.281	9.14E-19	18
RNPS1.7	4.04E-23	0.5365117	0.607	0.257	9.28E-19	18
TSTD1.7	4.32E-23	0.530117195	0.596	0.242	9.93E-19	18
ATP5L.7	2.19E-22	0.670767231	0.885	0.672	5.02E-18	18
RP11-386I14.4.2	2.59E-22	0.613169641	0.333	0.104	5.96E-18	18
RPL3.9	4.84E-22	-0.678133351	0.934	0.932	1.11E-17	18
ABRACL.9	5.24E-22	0.57541198	0.634	0.282	1.20E-17	18
TMSB4X.9	6.93E-22	0.542830913	0.984	0.977	1.59E-17	18
ARPC2.11	7.32E-22	0.725236673	0.874	0.635	1.68E-17	18
DBI.6	7.83E-22	0.592169706	0.754	0.418	1.80E-17	18
ANAPC11.4	1.28E-21	0.538638715	0.612	0.288	2.93E-17	18
PSMB10.5	1.53E-21	0.524702434	0.568	0.242	3.52E-17	18
THRAP3.4	1.76E-21	0.506830968	0.617	0.287	4.04E-17	18
PTMS.6	2.08E-21	0.71449835	0.552	0.238	4.79E-17	18
ZNF706.4	3.75E-21	0.631441647	0.59	0.272	8.61E-17	18
EEF1A1.11	4.12E-21	-0.546199048	0.962	0.968	9.47E-17	18
RPL26.8	4.76E-21	-0.59602763	0.973	0.935	1.09E-16	18
SVIP.1	5.67E-21	0.559667928	0.443	0.164	1.30E-16	18
MPDU1	6.08E-21	0.525571384	0.273	0.078	1.40E-16	18
SNRPC.2	6.65E-21	0.520599753	0.486	0.191	1.53E-16	18
COX6C.6	8.26E-21	0.607109375	0.836	0.564	1.90E-16	18
NCL.7	8.94E-21	0.662029171	0.77	0.427	2.06E-16	18
DNAJC8.4	1.32E-20	0.563705409	0.628	0.292	3.03E-16	18
PPP4C.4	1.42E-20	0.52769299	0.574	0.247	3.27E-16	18
ATP5J2.5	1.43E-20	0.555953293	0.792	0.427	3.29E-16	18
RBM8A.4	1.78E-20	0.538864318	0.732	0.364	4.08E-16	18
GNG5.11	2.19E-20	0.537654129	0.699	0.347	5.02E-16	18
ARPC5.9	2.19E-20	0.660971617	0.76	0.431	5.03E-16	18
TPT1.13	3.00E-20	-0.714537943	0.94	0.923	6.91E-16	18
PNRC2.1	3.43E-20	0.526267359	0.546	0.231	7.88E-16	18
IL2RG.18	3.96E-20	0.60380874	0.749	0.367	9.10E-16	18
LSM7.2	5.45E-20	0.511499767	0.612	0.282	1.25E-15	18
VDAC1.8	5.82E-20	0.526178343	0.596	0.273	1.34E-15	18
EIF2S2.2	6.13E-20	0.538060037	0.65	0.306	1.41E-15	18
POLR2G.1	2.01E-19	0.528955888	0.557	0.24	4.63E-15	18
RBMX.5	2.12E-19	0.542015169	0.716	0.375	4.88E-15	18
PA2G4.2	2.36E-19	0.645263354	0.683	0.375	5.42E-15	18
NAP1L1.6	4.30E-19	0.628673606	0.852	0.561	9.89E-15	18
RPL13A.12	1.22E-18	-0.502033612	0.989	0.962	2.80E-14	18
RPL39.9	2.25E-18	-0.539517081	0.973	0.944	5.18E-14	18

HLA-A.13	2.40E-18	0.560633024	0.962	0.893	5.51E-14	18
C11orf31.6	2.52E-18	0.723121193	0.656	0.323	5.78E-14	18
BZW1.6	3.57E-18	0.552151279	0.579	0.259	8.19E-14	18
RPL35A.11	4.52E-18	-0.553850805	0.94	0.917	1.04E-13	18
TXNIP.13	4.72E-18	-1.293741298	0.694	0.764	1.08E-13	18
SPCS2.4	5.57E-18	0.573721917	0.727	0.378	1.28E-13	18
RHOA.13	7.14E-18	0.526391034	0.82	0.504	1.64E-13	18
YBX1.9	1.16E-17	0.60369607	0.885	0.667	2.67E-13	18
GZMK.18	1.56E-17	0.734245933	0.678	0.361	3.59E-13	18
CTSW.4	2.04E-17	0.541643529	0.508	0.218	4.69E-13	18
HNRNPC.6	2.26E-17	0.622421853	0.776	0.472	5.20E-13	18
CKLF.11	2.93E-17	0.541706118	0.689	0.359	6.73E-13	18
CD2.17	4.23E-17	0.616046042	0.792	0.452	9.72E-13	18
ATP5E.12	4.30E-17	0.501139053	0.929	0.814	9.88E-13	18
HNRNPU.6	4.31E-17	0.517668826	0.672	0.37	9.90E-13	18
RPS18.11	4.31E-17	-0.512747111	0.978	0.959	9.90E-13	18
HINT1.9	5.44E-17	0.610684473	0.88	0.622	1.25E-12	18
SF3B2.8	7.26E-17	0.573331205	0.634	0.311	1.67E-12	18
RPL9.8	7.62E-17	-0.58513983	0.934	0.901	1.75E-12	18
LIMD2.16	1.06E-16	0.541625558	0.634	0.306	2.44E-12	18
DUSP1.13	1.57E-16	-1.143852855	0.721	0.794	3.61E-12	18
COTL1.12	1.74E-16	0.721508378	0.727	0.43	4.00E-12	18
CAPZB.8	3.17E-16	0.609746492	0.776	0.456	7.28E-12	18
HLA-F.12	3.17E-16	0.525616919	0.754	0.434	7.28E-12	18
RPL30.7	4.24E-16	-0.556875481	0.929	0.897	9.73E-12	18
CD99.12	4.40E-16	0.52129139	0.781	0.466	1.01E-11	18
HSPA8.10	5.32E-16	0.617879118	0.852	0.55	1.22E-11	18
SRSF7.14	5.44E-16	0.535237421	0.842	0.541	1.25E-11	18
FAM3C.4	5.60E-16	0.568922167	0.372	0.151	1.29E-11	18
TRBC2.17	7.26E-16	0.540370282	0.716	0.372	1.67E-11	18
LY6E.9	7.88E-16	0.508151608	0.705	0.361	1.81E-11	18
CALM1.9	1.64E-15	0.50236945	0.842	0.593	3.76E-11	18
RPS25.12	1.66E-15	-0.512135167	0.945	0.917	3.83E-11	18
MALAT1.16	2.67E-15	-0.677125548	0.978	0.983	6.14E-11	18
SEPT7.10	7.08E-15	0.560791817	0.792	0.509	1.63E-10	18
CLTA.11	8.89E-15	0.689184321	0.579	0.287	2.04E-10	18
PCBP2	4.52E-14	0.515441127	0.765	0.456	1.04E-09	18
FTH1.16	7.28E-14	-1.153566968	0.984	0.962	1.67E-09	18
LSP1.17	7.49E-14	0.604683556	0.749	0.43	1.72E-09	18
ARPC1B.10	9.37E-14	0.614449286	0.787	0.521	2.15E-09	18
ITM2B.12	1.71E-13	-0.913601773	0.825	0.779	3.92E-09	18
TRBC1.17	3.30E-13	0.690756841	0.623	0.35	7.59E-09	18
NR4A2.14	4.45E-13	0.635687943	0.536	0.273	1.02E-08	18
RPS13.9	4.81E-13	-0.540933987	0.907	0.878	1.11E-08	18
TSC22D3.11	5.95E-11	-0.859938756	0.699	0.782	1.37E-06	18
PNRC1.10	1.42E-09	-1.019052505	0.634	0.634	3.26E-05	18
PFDN5.8	6.49E-09	-0.612684297	0.847	0.783	0.00014907	18
JUNB.14	1.37E-08	-1.063085096	0.721	0.723	0.000313831	18
DDX5.13	5.07E-08	-0.567573376	0.863	0.81	0.001166162	18
FOS.16	3.50E-07	-0.926364251	0.639	0.682	0.008043207	18
TOMM7.9	4.62E-07	-0.584322976	0.831	0.754	0.010611318	18
RBPJ.11	4.67E-07	0.505116272	0.503	0.319	0.010720941	18
NEAT1.11	6.17E-07	-1.030681527	0.852	0.752	0.014173709	18
BTG1.17	9.48E-07	-0.67583641	0.918	0.841	0.021779392	18
LINC01320.1	0	3.581810718	0.885	0.05	0	19
C1orf186.2	0	3.200422804	0.742	0.05	0	19
ACSM2B	0	2.829962486	0.566	0.021	0	19
ACSM2A	0	2.71605844	0.566	0.016	0	19

PNCK	0	2.08630313	0.599	0.024	0	19
EMX2.1	0	2.079445388	0.593	0.026	0	19
DOC2A.1	0	1.882856418	0.467	0.015	0	19
KCNJ16.1	0	1.769067174	0.527	0.016	0	19
PRUNE2	0	1.708194613	0.462	0.014	0	19
MACROD2.1	0	1.687857583	0.484	0.014	0	19
UGT2A3.1	0	1.609726855	0.44	0.013	0	19
PPP2R3A.1	0	1.607556381	0.522	0.021	0	19
PAX8	0	1.581795144	0.527	0.018	0	19
ELF3	0	1.578549953	0.418	0.013	0	19
PAX2	0	1.559133984	0.5	0.01	0	19
CDHR5	0	1.535288906	0.429	0.013	0	19
ERBB3	0	1.502114671	0.401	0.011	0	19
SLC16A12	0	1.429064623	0.385	0.008	0	19
KIF12	0	1.268803307	0.39	0.011	0	19
DNAH11.1	0	1.261986102	0.363	0.011	0	19
RP11-807H17.1	0	1.192792221	0.368	0.008	0	19
CFB	0	1.17454073	0.407	0.01	0	19
ITGB8	0	1.16597783	0.385	0.012	0	19
AOC1	0	1.13079173	0.346	0.01	0	19
PKHD1	0	1.119422941	0.269	0.003	0	19
AHNAK2	0	1.078436934	0.352	0.009	0	19
SLC39A5	0	1.030689091	0.363	0.009	0	19
PRSS8	0	0.918605178	0.396	0.006	0	19
PROM1	0	0.853200653	0.33	0.004	0	19
HOGA1	0	0.828913668	0.319	0.006	0	19
DCDC2	0	0.75261485	0.286	0.006	0	19
KCNJ15	0	0.735600486	0.33	0.009	0	19
LINC01559	0	0.720824607	0.275	0.003	0	19
MUC1	0	0.711244092	0.302	0.007	0	19
FAM150B	0	0.688327507	0.258	0.004	0	19
NEFL	0	0.642837848	0.297	0.006	0	19
SLC34A2	0	0.57703769	0.258	0.002	0	19
SLC44A4	1.11E-302	0.929640191	0.346	0.011	2.56E-298	19
RAB42	2.25E-298	1.139355423	0.423	0.017	5.17E-294	19
FAM65C	4.18E-298	0.964351164	0.324	0.009	9.62E-294	19
PTPRM.1	8.51E-298	2.452646234	0.599	0.037	1.96E-293	19
HHLA2	4.63E-289	0.859766556	0.313	0.009	1.06E-284	19
HSF4.1	1.00E-287	1.44764141	0.423	0.018	2.30E-283	19
CLDN3	2.92E-284	0.891699135	0.374	0.014	6.71E-280	19
POU5F1	3.36E-282	1.101384057	0.302	0.008	7.71E-278	19
MST1	2.21E-280	1.221623821	0.33	0.01	5.09E-276	19
SLC28A1.1	5.09E-278	1.077228267	0.412	0.017	1.17E-273	19
ARHGEF28	5.25E-277	1.163294737	0.341	0.011	1.21E-272	19
UGT2B7.2	5.96E-275	1.949670769	0.56	0.035	1.37E-270	19
ADAMTS9-AS1	3.28E-266	0.881385932	0.264	0.007	7.54E-262	19
ABCC3.1	5.39E-266	2.113313485	0.577	0.039	1.24E-261	19
TACSTD2	5.10E-259	0.755144718	0.352	0.013	1.17E-254	19
ZNF608.1	1.58E-257	1.075517529	0.308	0.01	3.64E-253	19
CLDN4	1.09E-255	0.745307034	0.341	0.013	2.51E-251	19
SLC39A14.1	1.21E-253	1.785870539	0.511	0.031	2.79E-249	19
RHOBTB1	2.07E-253	1.14026655	0.374	0.016	4.75E-249	19
TNFRSF11B	2.64E-253	0.777631917	0.319	0.011	6.06E-249	19
CDK18.1	3.13E-253	1.702749556	0.495	0.029	7.19E-249	19
PLCB4	3.75E-252	1.795762998	0.368	0.015	8.61E-248	19
SLC6A8	1.14E-251	0.851810014	0.33	0.012	2.62E-247	19
ABHD11-AS1	7.57E-251	0.640377685	0.253	0.006	1.74E-246	19
SLC17A3.2	8.82E-249	2.462108812	0.566	0.04	2.03E-244	19

EGFR.1	1.22E-246	1.351755072	0.451	0.025	2.80E-242	19
HPN	1.38E-246	0.836189187	0.396	0.018	3.17E-242	19
GATM-AS1.1	5.26E-246	2.345617776	0.39	0.018	1.21E-241	19
CCDC64B	6.71E-244	0.8078451	0.346	0.014	1.54E-239	19
ERRFI1.1	7.75E-243	2.46141923	0.67	0.058	1.78E-238	19
PRKAA2.1	1.56E-242	1.138482705	0.341	0.013	3.59E-238	19
LINC01358	4.85E-239	1.042680515	0.33	0.013	1.11E-234	19
AR	3.08E-238	0.895653109	0.302	0.01	7.07E-234	19
VEGFA.1	8.04E-237	3.153774004	0.764	0.082	1.85E-232	19
CLRN3	4.04E-235	0.623535249	0.313	0.011	9.28E-231	19
BICC1.1	3.21E-231	1.14111633	0.401	0.02	7.38E-227	19
SEMA5B	1.11E-229	0.902290724	0.291	0.01	2.56E-225	19
GAL3ST1	1.79E-227	1.093474406	0.396	0.02	4.11E-223	19
AMN	9.31E-227	0.671927822	0.319	0.012	2.14E-222	19
CAPN12	1.98E-223	1.704692858	0.505	0.035	4.55E-219	19
SLC44A3	3.29E-223	0.545822745	0.275	0.009	7.56E-219	19
FBXO17.1	4.12E-223	1.228473782	0.434	0.025	9.47E-219	19
PPP1R14D.1	1.38E-222	0.682422317	0.308	0.012	3.17E-218	19
TMEM98.1	1.08E-221	0.585353478	0.341	0.015	2.48E-217	19
ASPA	1.84E-212	0.884827524	0.286	0.011	4.24E-208	19
ALPK2	1.23E-211	0.939167592	0.269	0.009	2.84E-207	19
PDZK1.1	1.78E-211	0.969353584	0.418	0.024	4.09E-207	19
C1S.1	6.85E-211	1.793308312	0.527	0.042	1.57E-206	19
PARD6B.1	5.41E-210	1.201595157	0.352	0.017	1.24E-205	19
FAT1.1	4.69E-209	0.69799712	0.302	0.012	1.08E-204	19
HSD3B7.1	3.86E-208	1.379564012	0.44	0.028	8.87E-204	19
SLC3A1.1	1.61E-204	1.52826854	0.478	0.034	3.71E-200	19
MET.1	2.00E-202	0.937992164	0.385	0.021	4.60E-198	19
CDH2.1	6.58E-202	0.74222119	0.319	0.014	1.51E-197	19
RP11-1C1.6.1	4.02E-201	0.97358122	0.363	0.019	9.23E-197	19
MAOA.1	8.02E-201	0.890392988	0.368	0.02	1.84E-196	19
DMKN	2.72E-200	1.065758712	0.363	0.019	6.25E-196	19
EMX2OS	2.70E-197	0.761017416	0.319	0.015	6.19E-193	19
EGFR-AS1.1	1.75E-195	0.837608159	0.297	0.013	4.01E-191	19
EPCAM	7.29E-195	0.559508251	0.302	0.013	1.68E-190	19
TSPAN1.1	1.80E-194	1.933708425	0.456	0.033	4.13E-190	19
ACY3	6.52E-193	0.79149773	0.319	0.015	1.50E-188	19
STARD4-AS1	9.42E-190	1.328150743	0.28	0.012	2.16E-185	19
SLC16A4	2.28E-188	1.113622849	0.429	0.029	5.24E-184	19
RERG.2	4.07E-188	0.858908936	0.379	0.023	9.36E-184	19
MVK	5.64E-188	0.732834739	0.357	0.02	1.30E-183	19
SLPI.1	1.60E-187	2.492941978	0.44	0.032	3.69E-183	19
EVA1A.1	1.61E-187	0.641630265	0.286	0.012	3.71E-183	19
ADAMTS9-AS2	2.97E-186	0.808281508	0.264	0.01	6.81E-182	19
ANPEP	3.30E-186	1.973275535	0.511	0.045	7.58E-182	19
TMEM139	1.47E-184	0.767016692	0.297	0.014	3.39E-180	19
SLC4A4	9.62E-184	0.726670961	0.297	0.014	2.21E-179	19
KRT19.1	3.01E-183	3.093774456	0.571	0.059	6.92E-179	19
CLEC18C.1	6.80E-183	0.654710439	0.269	0.011	1.56E-178	19
COBLL1.1	1.08E-180	0.780751195	0.313	0.016	2.47E-176	19
DIXDC1.1	2.21E-178	0.902078819	0.379	0.024	5.07E-174	19
SLC22A2.1	2.24E-176	0.78030469	0.352	0.021	5.14E-172	19
RP11-1C1.4	2.42E-176	0.610596642	0.258	0.011	5.57E-172	19
MTMR11.1	4.72E-174	1.310345521	0.357	0.022	1.08E-169	19
DDR1	2.14E-171	0.526440954	0.275	0.013	4.92E-167	19
CDH6.1	8.98E-170	1.085797355	0.407	0.029	2.06E-165	19
CP.1	6.36E-169	1.816688329	0.467	0.04	1.46E-164	19
HIF1A-AS2	8.14E-168	1.3212961	0.258	0.011	1.87E-163	19

CA9.1	4.18E-167	1.473535469	0.484	0.042	9.60E-163	19
SLC47A1	1.22E-166	1.091075746	0.275	0.013	2.80E-162	19
CFI	1.96E-164	0.857257794	0.407	0.03	4.50E-160	19
ACMSD	2.88E-164	0.522487955	0.28	0.014	6.62E-160	19
CUBN	1.04E-163	1.266021092	0.319	0.018	2.40E-159	19
ZNF320	1.18E-163	1.031192056	0.291	0.015	2.71E-159	19
EPS8L2	2.97E-161	1.279300609	0.505	0.048	6.82E-157	19
DLG5	3.54E-161	0.719561884	0.264	0.012	8.13E-157	19
CLDN7	4.07E-161	1.214202041	0.396	0.03	9.36E-157	19
NR1H4.1	4.26E-161	0.524309918	0.308	0.017	9.80E-157	19
RP11-14N7.2.1	8.98E-161	1.507391214	0.467	0.041	2.06E-156	19
S100A1.1	9.26E-159	1.799171847	0.44	0.037	2.13E-154	19
FN3K	1.89E-158	0.785456595	0.297	0.016	4.34E-154	19
CA12.1	4.17E-158	1.223942199	0.423	0.034	9.58E-154	19
ACSM5	1.93E-157	0.910069005	0.324	0.02	4.43E-153	19
SERPINF2.2	7.25E-157	1.421027735	0.484	0.048	1.67E-152	19
ENPEP.2	2.39E-153	1.14061764	0.401	0.032	5.50E-149	19
KBTBD11	2.64E-153	0.679398348	0.286	0.016	6.07E-149	19
MYEOV.1	6.30E-153	0.55055851	0.302	0.017	1.45E-148	19
SERINC2.1	2.58E-152	0.956477687	0.412	0.034	5.94E-148	19
HILPDA.3	8.02E-152	2.502556833	0.753	0.119	1.84E-147	19
HSPB8.1	4.01E-149	0.82603695	0.368	0.027	9.21E-145	19
HOOK2	2.16E-148	1.697550591	0.533	0.059	4.97E-144	19
REG1A.1	1.53E-146	3.252501138	0.489	0.053	3.52E-142	19
CNKSR3.1	6.92E-146	1.258711894	0.418	0.036	1.59E-141	19
GNG12.2	8.95E-143	0.662548558	0.341	0.024	2.06E-138	19
SLC6A13	3.74E-142	0.721152003	0.297	0.018	8.59E-138	19
CTHRC1.2	1.28E-141	0.916114471	0.385	0.031	2.95E-137	19
LINC00887.1	6.62E-141	1.173960889	0.291	0.018	1.52E-136	19
MIOX	1.10E-140	0.982165384	0.286	0.017	2.53E-136	19
MIR210HG.1	5.16E-140	0.640610319	0.297	0.019	1.19E-135	19
LRRC41.1	2.36E-138	2.109417211	0.566	0.074	5.43E-134	19
CFH	2.59E-138	0.576037683	0.324	0.022	5.96E-134	19
TMEM45A.1	3.31E-138	0.772100044	0.308	0.02	7.60E-134	19
PPP1R3C	4.01E-138	0.816357307	0.275	0.016	9.23E-134	19
WFDC2.2	1.03E-137	2.005192335	0.445	0.045	2.36E-133	19
PAWR.1	1.06E-137	0.81941442	0.302	0.02	2.44E-133	19
HRCT1	1.80E-137	0.610153318	0.302	0.02	4.15E-133	19
PLOD2.1	3.07E-137	1.295901053	0.434	0.041	7.07E-133	19
AEBP1.1	3.70E-137	0.794366582	0.346	0.026	8.49E-133	19
C1R.2	5.63E-137	1.540094866	0.467	0.049	1.29E-132	19
RAB3IP	3.84E-136	1.238747799	0.39	0.034	8.82E-132	19
ZNF395.1	8.11E-136	1.101217413	0.357	0.028	1.86E-131	19
ANGPTL4.5	1.29E-134	2.786969699	0.808	0.159	2.97E-130	19
LMO7	1.54E-134	1.067103713	0.385	0.033	3.53E-130	19
PLEKHA5.1	6.81E-133	1.543416712	0.429	0.042	1.56E-128	19
SLC37A4.1	9.79E-132	1.172711502	0.467	0.05	2.25E-127	19
ITGA3	1.04E-130	0.922118829	0.319	0.023	2.39E-126	19
KISS1R.1	8.65E-130	0.658339081	0.297	0.02	1.99E-125	19
GSTA2.1	4.55E-129	0.941266384	0.368	0.031	1.05E-124	19
APOL1	3.56E-125	1.994830167	0.516	0.07	8.18E-121	19
CDCA2.1	1.49E-124	1.014145791	0.324	0.025	3.42E-120	19
C19orf33.2	4.22E-124	1.883599287	0.462	0.053	9.70E-120	19
C11orf54.2	1.04E-123	1.597152611	0.533	0.072	2.39E-119	19
FAM134B.1	1.26E-123	1.196035692	0.401	0.039	2.89E-119	19
ZNF248	1.68E-122	1.231230492	0.341	0.029	3.87E-118	19
SAA2.1	1.40E-121	3.152976182	0.566	0.085	3.21E-117	19
INADL	2.01E-120	1.501931985	0.429	0.046	4.61E-116	19

RDH5	2.78E-120	0.999392189	0.313	0.024	6.39E-116	19
MT1H	3.50E-120	1.383125899	0.258	0.017	8.05E-116	19
CES4A.1	6.50E-120	1.497940912	0.357	0.032	1.49E-115	19
TFPI.3	1.34E-119	1.476355638	0.516	0.067	3.09E-115	19
FAM13A.1	3.18E-119	1.590185412	0.5	0.065	7.30E-115	19
CES2.1	4.97E-119	1.42628391	0.467	0.058	1.14E-114	19
FABP3	1.25E-117	0.501936831	0.269	0.018	2.87E-113	19
PPP1R13L.1	2.44E-117	0.942851258	0.302	0.023	5.60E-113	19
BHMT2.1	7.36E-116	0.981585722	0.297	0.023	1.69E-111	19
PDK1.1	3.22E-115	1.578434012	0.412	0.044	7.39E-111	19
GSTA1.1	4.41E-115	0.630645512	0.28	0.02	1.01E-110	19
ENPP3.1	2.83E-112	1.050887149	0.291	0.023	6.50E-108	19
SAA1.3	1.15E-111	3.513948681	0.637	0.124	2.65E-107	19
AK4.1	1.28E-111	0.758270961	0.357	0.033	2.94E-107	19
PTER	6.26E-111	1.409023529	0.418	0.049	1.44E-106	19
TNFAIP6	6.85E-111	0.632775985	0.275	0.02	1.57E-106	19
GALE	2.07E-110	0.578151689	0.335	0.03	4.75E-106	19
PPP1R16A.1	2.17E-109	0.601142156	0.368	0.037	4.98E-105	19
GGT1	3.68E-109	0.780122829	0.374	0.038	8.45E-105	19
KRT8.3	1.95E-108	1.677287867	0.571	0.092	4.49E-104	19
HAUS7	1.48E-105	0.860621946	0.379	0.041	3.40E-101	19
SDC4.1	2.29E-105	0.737739533	0.341	0.033	5.26E-101	19
CCL28.1	2.18E-104	0.92240945	0.341	0.033	5.00E-100	19
TMEM91.1	1.09E-103	1.178867557	0.462	0.061	2.50E-99	19
CYP3A5	5.71E-103	0.780082635	0.269	0.021	1.31E-98	19
NBL1.1	1.26E-102	0.738061469	0.363	0.038	2.89E-98	19
TCEA3.2	1.98E-102	0.885734084	0.429	0.052	4.56E-98	19
MMP7	4.62E-102	0.631490568	0.253	0.019	1.06E-97	19
KCTD3.1	1.00E-101	0.94117472	0.308	0.028	2.31E-97	19
ENOSF1	2.00E-101	1.481458616	0.478	0.067	4.59E-97	19
FARP1.2	9.19E-101	0.962391427	0.39	0.045	2.11E-96	19
C5orf46.2	1.07E-99	1.185730397	0.368	0.041	2.46E-95	19
TMEM27.2	2.17E-99	0.518378789	0.319	0.03	4.98E-95	19
TGM2.2	2.13E-97	1.190423595	0.434	0.057	4.89E-93	19
RASSF4.3	2.21E-97	2.129048962	0.692	0.157	5.07E-93	19
DYNC2LI1.1	3.24E-97	0.678611598	0.308	0.029	7.44E-93	19
RP11-83A24.2	1.27E-96	1.397735352	0.462	0.064	2.92E-92	19
MT1G.3	3.84E-96	2.238709241	0.418	0.056	8.83E-92	19
RBPMS.3	1.98E-95	0.884532557	0.451	0.061	4.56E-91	19
ENO2.1	2.85E-95	0.855265158	0.374	0.042	6.55E-91	19
TLN2	7.68E-95	0.664288357	0.258	0.021	1.76E-90	19
SMIM24.1	1.40E-94	0.519978038	0.308	0.029	3.23E-90	19
SPINT1.1	2.55E-92	0.615838288	0.341	0.038	5.86E-88	19
TMSB4X.10	2.59E-92	-2.986469976	0.874	0.978	5.95E-88	19
TNFRSF12A.1	4.41E-91	0.811046814	0.313	0.032	1.01E-86	19
MACC1.1	1.41E-90	0.960832874	0.313	0.032	3.23E-86	19
MGST1.4	2.17E-89	1.421011935	0.571	0.106	4.99E-85	19
RRAD.1	2.56E-89	1.049006012	0.253	0.021	5.89E-85	19
PPFIBP1.3	2.87E-89	1.027010188	0.39	0.049	6.59E-85	19
P3H2.1	1.41E-88	1.34886503	0.374	0.047	3.23E-84	19
GATM.2	3.71E-88	1.158679723	0.451	0.067	8.53E-84	19
B2M.13	3.52E-87	-2.15460018	0.923	0.989	8.08E-83	19
RGS12.1	5.56E-87	1.294437982	0.385	0.05	1.28E-82	19
PTPRF	6.90E-87	0.635742149	0.269	0.025	1.59E-82	19
CALD1.3	1.02E-86	1.432640142	0.681	0.142	2.35E-82	19
TPM1.5	1.67E-86	2.010650098	0.714	0.188	3.83E-82	19
CXCL14.3	1.73E-86	1.22544116	0.423	0.06	3.99E-82	19
ZMYND8	2.00E-86	2.036577746	0.654	0.149	4.59E-82	19

FNBP1L.1	2.84E-86	0.622816117	0.319	0.034	6.53E-82	19
MXRA7.1	3.48E-86	0.615183221	0.33	0.036	8.00E-82	19
AQP3.1	2.52E-85	1.177468051	0.44	0.065	5.78E-81	19
WDR60.2	3.28E-85	1.734468092	0.544	0.1	7.54E-81	19
RBP5.2	3.45E-85	0.912243982	0.412	0.058	7.93E-81	19
RND3.1	5.23E-84	0.724743687	0.253	0.023	1.20E-79	19
FHL1.2	1.40E-83	0.82982134	0.379	0.05	3.22E-79	19
TMEM37.1	5.95E-83	0.724393692	0.363	0.046	1.37E-78	19
TSPAN12.2	9.90E-83	0.539919263	0.33	0.038	2.28E-78	19
RDH13	1.11E-82	0.665094081	0.269	0.026	2.55E-78	19
ASS1.1	1.44E-82	0.51829032	0.253	0.023	3.31E-78	19
DPCD.1	2.06E-82	0.503796274	0.341	0.041	4.74E-78	19
DEFB1.3	2.29E-82	2.289812647	0.473	0.084	5.27E-78	19
BHLHE41.3	4.30E-82	1.171967022	0.456	0.075	9.89E-78	19
EGLN3.2	5.13E-82	0.883291694	0.445	0.065	1.18E-77	19
OXR1	5.38E-82	1.664920084	0.516	0.094	1.24E-77	19
SORBS2.2	2.21E-81	1.243550578	0.346	0.043	5.08E-77	19
FABP6.2	2.81E-81	0.702133912	0.33	0.039	6.46E-77	19
CD70.1	4.13E-81	0.93694313	0.401	0.057	9.48E-77	19
NFIB.3	4.58E-81	1.085368158	0.445	0.067	1.05E-76	19
PROS1.2	7.42E-81	0.75815892	0.363	0.047	1.71E-76	19
EFNA1.2	5.76E-80	0.971307799	0.423	0.064	1.32E-75	19
KHK.1	1.17E-79	0.696885663	0.368	0.049	2.70E-75	19
PPP1R3B	1.26E-79	1.091273544	0.346	0.044	2.89E-75	19
CCDC146.1	1.41E-78	0.816623039	0.324	0.039	3.23E-74	19
WDR34.2	2.02E-78	0.600515478	0.308	0.035	4.65E-74	19
PRRG4.1	9.34E-78	0.740178991	0.385	0.055	2.15E-73	19
ANK3	1.59E-76	0.855972555	0.302	0.035	3.65E-72	19
NEAT1.12	5.01E-76	2.719648453	0.989	0.75	1.15E-71	19
RPS12.7	7.20E-76	-1.897151956	0.709	0.941	1.65E-71	19
RPL21.10	7.57E-76	-1.839092486	0.775	0.954	1.74E-71	19
NAT8.2	8.45E-76	0.796740034	0.33	0.041	1.94E-71	19
ZBTB20.4	1.40E-75	1.560901354	0.637	0.147	3.22E-71	19
CMBL.2	1.44E-75	0.505651719	0.39	0.055	3.31E-71	19
RP1-60O19.1.1	1.86E-75	0.764243553	0.275	0.029	4.27E-71	19
TRIP10.1	1.16E-74	0.694627496	0.264	0.028	2.66E-70	19
FGGY	1.24E-74	0.654099775	0.313	0.039	2.85E-70	19
LINC00969.1	2.17E-74	2.270503525	0.665	0.168	4.99E-70	19
CLU.5	2.26E-74	3.016998216	0.654	0.192	5.19E-70	19
CRYZ.1	7.00E-74	0.890171128	0.396	0.061	1.61E-69	19
PDZK1IP1.4	7.84E-74	2.041433698	0.538	0.117	1.80E-69	19
NOL3.2	1.13E-73	0.847094287	0.44	0.071	2.60E-69	19
PTGR1.1	1.76E-73	0.752859674	0.363	0.052	4.04E-69	19
TJP2.1	2.90E-73	0.642570701	0.258	0.027	6.67E-69	19
PTMA.9	2.72E-72	-2.038456984	0.588	0.922	6.25E-68	19
RPS29.14	3.05E-72	-2.207426845	0.588	0.926	7.01E-68	19
TSPAN4.3	1.05E-71	1.268433433	0.489	0.096	2.41E-67	19
MYO6.1	1.38E-71	0.895147251	0.368	0.053	3.17E-67	19
LACTB2	1.80E-71	0.858929392	0.396	0.063	4.15E-67	19
CKB.1	2.83E-71	1.000391177	0.33	0.044	6.50E-67	19
FAIM.1	9.15E-71	0.698841683	0.297	0.036	2.10E-66	19
EPHX2	1.47E-70	0.544673699	0.286	0.033	3.39E-66	19
LOX.1	5.27E-70	0.753189403	0.313	0.04	1.21E-65	19
YBX3.4	5.52E-70	1.848924836	0.659	0.178	1.27E-65	19
SMPDL3A.1	5.64E-70	0.588475637	0.319	0.041	1.30E-65	19
MUM1.2	5.93E-70	1.234717347	0.478	0.09	1.36E-65	19
TCN2	1.64E-69	0.865730299	0.352	0.052	3.77E-65	19
PILRB.1	2.10E-69	1.375654692	0.495	0.097	4.82E-65	19

MGRN1	2.93E-69	1.4703595	0.495	0.098	6.73E-65	19
ECHDC2	5.98E-69	1.28194957	0.5	0.097	1.37E-64	19
SYTL2.3	9.18E-68	1.308275915	0.484	0.094	2.11E-63	19
GAMT.2	2.34E-67	0.776956182	0.357	0.054	5.37E-63	19
MGLL.2	4.32E-67	0.720550644	0.407	0.067	9.93E-63	19
RPS27.12	7.28E-67	-1.645166354	0.83	0.977	1.67E-62	19
PLEKHA1.2	1.59E-66	1.567291783	0.538	0.116	3.66E-62	19
NDRG1.2	3.63E-66	1.467789701	0.566	0.137	8.33E-62	19
NNMT.18	3.86E-66	1.978118861	0.786	0.293	8.88E-62	19
RPL32.6	9.29E-66	-1.645302128	0.67	0.93	2.13E-61	19
ZNF83	9.24E-65	1.483780304	0.484	0.098	2.12E-60	19
RPS4X.12	3.01E-64	-1.59528382	0.643	0.942	6.92E-60	19
PLXNB2.1	3.77E-64	0.527100176	0.335	0.05	8.66E-60	19
FSTL3.2	4.71E-64	0.84563331	0.269	0.033	1.08E-59	19
SERPING1.3	1.22E-63	1.376630478	0.478	0.104	2.80E-59	19
CRLS1	3.22E-63	0.959873448	0.412	0.075	7.39E-59	19
CAV2.3	5.69E-63	0.646256138	0.418	0.073	1.31E-58	19
AKAP12.2	9.88E-63	0.601729272	0.308	0.043	2.27E-58	19
FXYD2.4	1.23E-62	1.732861022	0.533	0.128	2.82E-58	19
SOD2.14	2.66E-62	2.338276817	0.736	0.288	6.11E-58	19
KRT18.3	5.23E-62	1.46651599	0.473	0.101	1.20E-57	19
C11orf1.1	7.07E-62	0.534877268	0.352	0.055	1.62E-57	19
CNDP2.4	1.27E-61	1.810004432	0.632	0.207	2.92E-57	19
ATP1B1.5	2.15E-61	1.483743597	0.533	0.132	4.94E-57	19
MXI1.1	1.55E-60	0.827695765	0.385	0.066	3.56E-56	19
PLA2G12A	2.82E-60	0.790487341	0.374	0.064	6.48E-56	19
FAM195A	1.03E-59	0.523386045	0.346	0.056	2.37E-55	19
CREB5	3.02E-59	0.820076901	0.258	0.033	6.93E-55	19
SHMT1.1	4.36E-59	0.539004751	0.33	0.051	1.00E-54	19
C11orf49	1.34E-58	0.514423958	0.28	0.038	3.08E-54	19
HSBP1L1.1	6.43E-58	0.617613801	0.335	0.053	1.48E-53	19
FAHD1.1	6.71E-58	0.674445638	0.324	0.051	1.54E-53	19
RPL27A.9	2.55E-57	-1.455610774	0.659	0.934	5.87E-53	19
TMEM176A.4	3.74E-57	1.899846618	0.522	0.141	8.60E-53	19
CARS2	1.06E-56	0.633795879	0.297	0.044	2.44E-52	19
NIT2.1	5.26E-56	0.898190199	0.467	0.103	1.21E-51	19
SSPN.3	6.67E-56	0.832295083	0.291	0.043	1.53E-51	19
SH3BP2	9.03E-56	1.088485594	0.401	0.077	2.08E-51	19
ZNF611	1.08E-55	1.137566863	0.286	0.042	2.49E-51	19
WDR13.2	1.10E-55	0.770668403	0.44	0.092	2.54E-51	19
UACA.2	1.15E-55	0.603718863	0.374	0.067	2.64E-51	19
RPS3.13	1.25E-55	-1.477871971	0.637	0.923	2.88E-51	19
MT1E.7	5.33E-55	2.069625255	0.571	0.168	1.22E-50	19
INTS3	5.91E-55	0.828099451	0.297	0.045	1.36E-50	19
RARRES2.4	6.77E-55	1.008778982	0.478	0.107	1.56E-50	19
SHMT2.2	7.19E-55	0.817227126	0.39	0.074	1.65E-50	19
RPL15.4	8.22E-55	-1.427600345	0.643	0.91	1.89E-50	19
PLAGL1.1	3.81E-54	0.986798197	0.341	0.06	8.75E-50	19
LTBR.2	3.97E-54	0.965411563	0.379	0.072	9.13E-50	19
EVA1C	5.12E-54	0.531660334	0.258	0.034	1.18E-49	19
RPL13A.13	6.30E-54	-1.2353847	0.852	0.964	1.45E-49	19
SNHG25.2	8.67E-54	1.727051109	0.478	0.122	1.99E-49	19
ARHGAP29.2	1.64E-53	0.772847253	0.368	0.066	3.77E-49	19
CRYAB.16	1.84E-53	1.979535798	0.676	0.254	4.24E-49	19
RP11-553L6.5.3	2.16E-53	0.949223432	0.308	0.049	4.97E-49	19
ERGIC1.2	3.04E-53	1.231812188	0.505	0.127	6.99E-49	19
IGFBP4.3	3.09E-53	1.086121799	0.478	0.113	7.09E-49	19
PFKP.2	3.99E-53	1.209260992	0.456	0.108	9.16E-49	19

RPS15A.10	5.38E-53	-1.347064198	0.687	0.937	1.24E-48	19
RPL34.12	7.74E-53	-1.40060081	0.676	0.957	1.78E-48	19
SRGN.15	9.74E-53	-2.400831143	0.335	0.797	2.24E-48	19
DST.3	1.35E-52	1.014553434	0.39	0.078	3.11E-48	19
BDH2.1	1.49E-52	0.670204837	0.363	0.066	3.43E-48	19
HLA-C.14	1.86E-52	-1.650369735	0.648	0.886	4.28E-48	19
RPLP2.9	1.94E-52	-1.284297536	0.736	0.947	4.46E-48	19
AIG1.2	2.19E-52	0.839340054	0.412	0.087	5.03E-48	19
RPL28.12	3.82E-51	-1.339408523	0.709	0.935	8.78E-47	19
DHTKD1	6.53E-51	0.67942139	0.286	0.044	1.50E-46	19
ADM.2	8.74E-50	1.044287033	0.478	0.113	2.01E-45	19
CD24.5	1.84E-49	1.449163402	0.577	0.173	4.23E-45	19
ACTB.14	3.17E-49	-1.630998917	0.786	0.931	7.29E-45	19
ALDH1A1.2	3.30E-49	0.534343924	0.407	0.086	7.59E-45	19
RPS14.11	4.66E-49	-1.300426313	0.698	0.95	1.07E-44	19
TRIP6.2	5.87E-49	0.550402589	0.308	0.052	1.35E-44	19
KIAA1191	7.61E-49	0.579832698	0.357	0.068	1.75E-44	19
SH3YL1	7.80E-49	0.661638607	0.346	0.064	1.79E-44	19
NET1.1	1.16E-48	0.645534968	0.28	0.045	2.66E-44	19
BNIP3.4	3.14E-48	1.169322865	0.495	0.129	7.21E-44	19
RP11-798M19.6.1	3.41E-48	0.734508506	0.385	0.078	7.84E-44	19
ANXA4.6	7.47E-48	1.308633171	0.516	0.153	1.72E-43	19
FAM127B.2	1.22E-47	0.502699935	0.346	0.065	2.81E-43	19
RPL39.10	1.32E-47	-1.345480688	0.654	0.949	3.03E-43	19
SLC2A1.1	2.01E-47	0.648645889	0.269	0.042	4.61E-43	19
HLA-B.15	2.01E-47	-1.338476471	0.758	0.916	4.62E-43	19
EPS8.3	2.17E-47	0.664478497	0.368	0.075	4.98E-43	19
ATP6V0A1	3.43E-47	1.079438412	0.28	0.047	7.87E-43	19
CLIC4.4	4.27E-47	0.747181761	0.396	0.089	9.82E-43	19
HMGCL.1	5.94E-47	0.794035867	0.396	0.084	1.37E-42	19
NFE2L1.3	2.18E-46	0.630713705	0.379	0.079	5.02E-42	19
FRMD4A.2	2.60E-46	0.998197999	0.352	0.071	5.97E-42	19
MIR4458HG.2	2.90E-46	0.560521608	0.363	0.075	6.67E-42	19
CCND1.3	3.11E-46	0.836358442	0.401	0.089	7.15E-42	19
BTG1.18	3.32E-46	-2.222059947	0.621	0.845	7.62E-42	19
FNDC3B.2	3.42E-46	1.185314245	0.418	0.097	7.86E-42	19
RPS27A.13	5.52E-46	-1.161712042	0.758	0.942	1.27E-41	19
RPL12.9	1.15E-45	-1.389275836	0.599	0.915	2.65E-41	19
JADE1.1	1.92E-45	0.888813089	0.379	0.081	4.41E-41	19
S100A13.4	3.25E-45	1.067220087	0.473	0.129	7.46E-41	19
FAU.8	4.06E-45	-1.358024777	0.582	0.893	9.33E-41	19
RASSF7	8.20E-45	0.52141947	0.341	0.066	1.88E-40	19
TMEM176B.4	1.21E-44	1.506194823	0.495	0.15	2.79E-40	19
LARP1B	1.47E-44	0.717045998	0.286	0.049	3.37E-40	19
POMGNT1	3.78E-44	0.889053767	0.253	0.041	8.69E-40	19
RPL14.9	4.08E-44	-1.43093886	0.571	0.873	9.38E-40	19
WBP5.4	8.14E-44	0.565352598	0.374	0.081	1.87E-39	19
RPL23A.13	8.87E-44	-1.217415731	0.692	0.933	2.04E-39	19
MYO9A	9.53E-44	0.671659691	0.291	0.052	2.19E-39	19
PHYKPL.1	9.56E-44	1.152979554	0.571	0.174	2.20E-39	19
RP11-1000B6.3.1	1.28E-43	0.944988491	0.275	0.048	2.94E-39	19
RAB34.3	2.65E-43	0.557038859	0.368	0.082	6.10E-39	19
ABHD11.1	3.11E-43	0.508044235	0.286	0.05	7.14E-39	19
LGALS3BP.3	4.39E-43	0.790436972	0.407	0.098	1.01E-38	19
VPS13A	8.28E-43	1.062292652	0.385	0.087	1.90E-38	19
MTR	3.83E-42	0.919818383	0.396	0.091	8.79E-38	19
CXCR4.13	6.82E-42	-2.467989801	0.478	0.779	1.57E-37	19
BCKDK.1	1.22E-40	0.819126942	0.412	0.103	2.80E-36	19

ANKRD37.1	1.79E-40	0.64453112	0.335	0.069	4.12E-36	19
SMAGP	1.88E-40	0.543866412	0.253	0.042	4.31E-36	19
C5orf45	1.96E-40	0.601178199	0.286	0.053	4.50E-36	19
RBM41	2.35E-40	0.915613589	0.258	0.045	5.40E-36	19
FGD4.3	2.72E-40	1.018389114	0.357	0.081	6.24E-36	19
RPS25.13	3.19E-40	-1.227698213	0.654	0.921	7.32E-36	19
RPL37A.4	6.99E-40	-1.207581206	0.61	0.91	1.61E-35	19
NPEPPS	7.43E-40	0.926273152	0.418	0.107	1.71E-35	19
SLC16A3.3	1.17E-39	1.072702636	0.516	0.167	2.68E-35	19
EIF3J-AS1	1.20E-39	0.890304023	0.308	0.061	2.76E-35	19
UBA52.6	1.86E-39	-1.283074812	0.582	0.87	4.28E-35	19
RNASET2.16	4.98E-39	1.750362802	0.687	0.356	1.14E-34	19
NQO2.2	5.49E-39	0.648072654	0.346	0.078	1.26E-34	19
RPL18A.10	6.82E-39	-1.18268032	0.676	0.933	1.57E-34	19
SPINT2.4	7.38E-39	1.345302123	0.456	0.146	1.70E-34	19
SLC25A37.4	8.62E-39	1.418115934	0.5	0.15	1.98E-34	19
SNHG12	8.94E-39	0.845542158	0.368	0.085	2.05E-34	19
KCNQ1OT1.2	9.93E-39	1.601520635	0.379	0.093	2.28E-34	19
GADD45A.2	2.84E-38	0.697889215	0.401	0.101	6.52E-34	19
TPT1.14	3.46E-38	-1.194813109	0.725	0.926	7.96E-34	19
ZFP36L2.15	3.83E-38	-2.840302444	0.379	0.703	8.79E-34	19
RBM47.4	3.94E-38	0.708694063	0.341	0.076	9.05E-34	19
RPL26.9	4.37E-38	-1.114774607	0.676	0.939	1.00E-33	19
RPSA.11	5.84E-38	-1.555195283	0.522	0.808	1.34E-33	19
STC1.2	1.04E-37	0.99298704	0.324	0.067	2.40E-33	19
GPX3.6	1.08E-37	1.389491287	0.495	0.161	2.49E-33	19
ARHGDIB.12	1.10E-37	-1.974111139	0.253	0.678	2.52E-33	19
BIRC3.15	1.42E-37	2.536089763	0.621	0.267	3.27E-33	19
CYB5R3.5	1.48E-37	1.090113536	0.527	0.182	3.39E-33	19
HCFC1R1.4	2.30E-37	0.795048615	0.434	0.124	5.28E-33	19
CD2AP	2.61E-37	0.758440389	0.346	0.079	6.01E-33	19
ISOC2	7.53E-37	0.61846745	0.374	0.093	1.73E-32	19
LIMA1.2	7.97E-37	0.725631988	0.357	0.082	1.83E-32	19
VCAM1.3	1.38E-36	0.784569199	0.379	0.093	3.17E-32	19
RPS26.13	1.85E-36	-1.260718296	0.593	0.874	4.24E-32	19
RPS16.9	2.40E-36	-1.176949952	0.621	0.895	5.51E-32	19
PTPRC.16	2.56E-36	-2.205413493	0.17	0.622	5.88E-32	19
IRF7.1	2.75E-36	0.592776438	0.357	0.084	6.32E-32	19
RPL30.8	5.05E-36	-1.132114294	0.621	0.901	1.16E-31	19
HPS4.1	5.08E-36	1.293346918	0.357	0.088	1.17E-31	19
NUPR1.5	5.70E-36	0.769626241	0.451	0.13	1.31E-31	19
TMA7.11	7.51E-36	-1.867516476	0.374	0.7	1.73E-31	19
QTRT1.1	8.43E-36	0.766177457	0.352	0.085	1.94E-31	19
RPS15.11	8.73E-36	-1.044638637	0.703	0.935	2.01E-31	19
ASPH.4	9.00E-36	0.86847202	0.385	0.098	2.07E-31	19
MAP3K13.2	9.69E-36	0.834124433	0.407	0.109	2.23E-31	19
P4HA1.1	4.47E-35	0.51419396	0.374	0.094	1.03E-30	19
C3.4	4.49E-35	0.701065482	0.467	0.137	1.03E-30	19
BSDC1.1	5.16E-35	1.08496602	0.44	0.126	1.19E-30	19
PIAS2.1	1.18E-34	0.790676554	0.33	0.077	2.70E-30	19
DAB2.4	1.58E-34	0.89584216	0.505	0.157	3.63E-30	19
UBR4.2	2.09E-34	0.557859367	0.379	0.099	4.80E-30	19
RPLP1.6	4.62E-34	-0.99161939	0.802	0.968	1.06E-29	19
TM7SF3.1	5.87E-34	0.590777547	0.368	0.092	1.35E-29	19
NFIA.3	5.92E-34	0.728726079	0.335	0.081	1.36E-29	19
RUFY3.2	6.37E-34	0.871752106	0.264	0.054	1.46E-29	19
RPL31.15	1.06E-33	-1.161352332	0.626	0.911	2.43E-29	19
RPL19.10	1.30E-33	-0.990555101	0.698	0.928	2.99E-29	19

ARHGEF12.2	1.34E-33	0.501318731	0.324	0.075	3.08E-29	19
GSS	1.57E-33	0.522416433	0.291	0.063	3.60E-29	19
IFI27.4	2.34E-33	0.704972435	0.478	0.15	5.37E-29	19
POLR2J3.10	4.37E-33	1.65138409	0.764	0.39	1.01E-28	19
LRRK2.3	5.02E-33	0.756387569	0.297	0.068	1.15E-28	19
CMB9-22P13.1.1	5.29E-33	0.678060056	0.302	0.069	1.21E-28	19
S100A4.16	5.75E-33	-2.229043974	0.412	0.703	1.32E-28	19
GMFG.10	6.36E-33	-1.852227308	0.115	0.562	1.46E-28	19
CD52.14	8.21E-33	-2.80498812	0.121	0.554	1.89E-28	19
AKAP8	8.55E-33	0.854450345	0.308	0.07	1.96E-28	19
KDSR.2	9.77E-33	0.721717845	0.374	0.1	2.24E-28	19
RPS19.10	2.13E-32	-0.988185677	0.78	0.954	4.89E-28	19
MT1F.2	3.84E-32	1.08832345	0.418	0.126	8.82E-28	19
ATP11A.1	5.83E-32	1.045200891	0.258	0.055	1.34E-27	19
SEC31A.2	5.96E-32	0.895965242	0.445	0.133	1.37E-27	19
HCST.14	6.24E-32	-2.224246748	0.115	0.55	1.43E-27	19
RPS6.9	7.16E-32	-1.007532841	0.692	0.947	1.65E-27	19
SPP1.8	7.58E-32	0.668035274	0.555	0.212	1.74E-27	19
RPL29.5	8.36E-32	-1.136100201	0.56	0.859	1.92E-27	19
CFL1.7	9.95E-32	-1.556487963	0.495	0.75	2.29E-27	19
RPS18.12	1.28E-31	-0.99777051	0.736	0.962	2.93E-27	19
TGFBI.5	1.44E-31	1.643019422	0.484	0.179	3.32E-27	19
ACADM.1	1.76E-31	0.517416781	0.401	0.113	4.04E-27	19
ATP5E.13	2.26E-31	-1.208963224	0.5	0.82	5.20E-27	19
WDR73	2.94E-31	0.549218976	0.291	0.066	6.75E-27	19
ASL	3.04E-31	0.638476489	0.297	0.068	6.98E-27	19
RPS23.8	3.32E-31	-0.995308663	0.648	0.922	7.64E-27	19
VMP1.12	3.41E-31	1.715017669	0.698	0.315	7.84E-27	19
MMP24-AS1.3	3.79E-31	0.982689328	0.484	0.168	8.70E-27	19
POLR2J2	5.54E-31	0.556737557	0.286	0.065	1.27E-26	19
CLK4	5.56E-31	0.844850313	0.363	0.097	1.28E-26	19
RPL35A.12	6.59E-31	-1.020881048	0.698	0.92	1.51E-26	19
RPS28.10	7.26E-31	-0.937614304	0.731	0.95	1.67E-26	19
RPL10A.11	8.62E-31	-1.12638561	0.582	0.869	1.98E-26	19
IFI44L.1	1.13E-30	1.287342468	0.297	0.072	2.60E-26	19
ZNF37A	1.29E-30	0.735864657	0.297	0.069	2.96E-26	19
PTTG1IP.3	1.32E-30	1.035559982	0.473	0.173	3.04E-26	19
TMEM63A	1.37E-30	0.622749735	0.258	0.055	3.16E-26	19
ZSCAN18.1	1.62E-30	1.04839125	0.28	0.064	3.72E-26	19
RPS3A.8	1.64E-30	-0.980469849	0.659	0.933	3.78E-26	19
SLC50A1	1.68E-30	0.892995415	0.368	0.098	3.86E-26	19
CD151.5	4.09E-30	1.149795912	0.495	0.197	9.40E-26	19
CD69.17	4.17E-30	-2.419867655	0.082	0.535	9.58E-26	19
RPL13.10	5.50E-30	-0.915362105	0.83	0.966	1.26E-25	19
CPEB4.1	5.71E-30	0.988976095	0.346	0.092	1.31E-25	19
CERS2.1	6.69E-30	0.52183787	0.368	0.102	1.54E-25	19
RNPC3.1	7.50E-30	1.043641146	0.308	0.078	1.72E-25	19
RPL11.8	7.51E-30	-0.918932251	0.78	0.94	1.72E-25	19
ALDH3A2.1	9.14E-30	0.56745261	0.269	0.059	2.10E-25	19
MVP	1.43E-29	0.858015102	0.445	0.145	3.30E-25	19
AKR1A1.3	2.34E-29	0.88996564	0.516	0.189	5.39E-25	19
UFSP2.1	3.65E-29	0.631895999	0.324	0.082	8.39E-25	19
ALOX5.3	5.28E-29	0.627187106	0.357	0.097	1.21E-24	19
ESF1.1	5.79E-29	0.7385177	0.445	0.141	1.33E-24	19
CBWD5	7.48E-29	1.088749736	0.341	0.092	1.72E-24	19
METTL15.1	9.41E-29	1.909159058	0.363	0.105	2.16E-24	19
RPL27.5	1.24E-28	-1.087301424	0.538	0.856	2.85E-24	19
ZNF33B.1	1.25E-28	0.546901112	0.264	0.06	2.88E-24	19

RPL36AL.6	1.51E-28	-1.559500308	0.456	0.715	3.47E-24	19
AHCYL1.2	1.55E-28	0.761039215	0.324	0.083	3.56E-24	19
MYO1C.2	2.38E-28	0.504282706	0.253	0.056	5.48E-24	19
RPL18.10	2.60E-28	-1.01575006	0.637	0.873	5.98E-24	19
CORO1A.15	2.61E-28	-1.862249737	0.187	0.57	6.01E-24	19
YPEL2.2	2.62E-28	0.77700547	0.363	0.102	6.02E-24	19
LGALS2.4	2.69E-28	0.727869967	0.346	0.097	6.19E-24	19
MBNL2.2	3.49E-28	0.509840442	0.335	0.089	8.02E-24	19
BNIP3L.4	3.75E-28	0.937500897	0.544	0.227	8.63E-24	19
GOLGA8A.1	5.21E-28	1.030239259	0.308	0.08	1.20E-23	19
LPP.2	6.05E-28	0.870501259	0.478	0.162	1.39E-23	19
TFEC.2	6.29E-28	0.636439884	0.253	0.057	1.45E-23	19
DUSP23.2	6.98E-28	1.016318713	0.456	0.17	1.60E-23	19
ADIRF.12	8.46E-28	1.372473131	0.538	0.237	1.94E-23	19
RGS1.18	8.90E-28	-2.397425698	0.478	0.7	2.05E-23	19
MDM2.1	1.07E-27	1.318807866	0.396	0.121	2.47E-23	19
CYB5A.6	5.90E-27	1.007569542	0.522	0.225	1.36E-22	19
EEF1A1.12	6.65E-27	-0.819471416	0.841	0.97	1.53E-22	19
SH3BGRL3.15	8.14E-27	-1.690194549	0.445	0.685	1.87E-22	19
NFIC.3	1.27E-26	0.62025966	0.385	0.116	2.91E-22	19
SMYD4	1.53E-26	0.740796091	0.319	0.087	3.51E-22	19
CYTIP.16	2.76E-26	-1.654686225	0.071	0.481	6.34E-22	19
ACADVL.2	2.92E-26	0.98083333	0.522	0.212	6.71E-22	19
BACH1.2	3.40E-26	0.570780711	0.319	0.087	7.81E-22	19
CD3D.19	4.79E-26	-2.062635684	0.077	0.48	1.10E-21	19
RPS2.10	6.36E-26	-0.820332843	0.791	0.946	1.46E-21	19
MAST4	6.64E-26	0.640392042	0.253	0.059	1.53E-21	19
NDUFA4L2.18	9.79E-26	1.334225177	0.588	0.285	2.25E-21	19
CD2.18	1.25E-25	-2.204860935	0.066	0.462	2.88E-21	19
RPL4.5	1.53E-25	-1.192796719	0.505	0.773	3.52E-21	19
PLIN2.13	1.66E-25	1.352219785	0.61	0.332	3.80E-21	19
ARPC2.12	1.89E-25	-1.699229641	0.401	0.641	4.35E-21	19
H3F3B.12	2.76E-25	-0.9063344	0.632	0.837	6.34E-21	19
EEF1D.12	3.14E-25	-1.195227206	0.505	0.774	7.22E-21	19
CDKN1A.3	5.86E-25	1.015301902	0.374	0.117	1.35E-20	19
PFN1.12	6.74E-25	-1.242858686	0.527	0.763	1.55E-20	19
TOR1AIP2	1.42E-24	0.750104112	0.363	0.11	3.27E-20	19
MALAT1.17	1.74E-24	1.391951845	0.989	0.982	3.99E-20	19
MAVS	1.80E-24	0.745549284	0.269	0.069	4.14E-20	19
TMEM205.1	1.92E-24	0.737985016	0.418	0.15	4.41E-20	19
PDK4.7	2.35E-24	1.587544381	0.505	0.199	5.40E-20	19
RERE.2	3.75E-24	0.70328153	0.385	0.123	8.63E-20	19
HMGB1.12	3.99E-24	-1.317152403	0.555	0.737	9.18E-20	19
ZNF397	5.17E-24	0.912956429	0.286	0.078	1.19E-19	19
RPS10.8	6.56E-24	-1.102576973	0.484	0.79	1.51E-19	19
PDLIM5.2	6.94E-24	0.619627287	0.297	0.08	1.60E-19	19
IGFBP3.13	7.71E-24	0.508117072	0.555	0.24	1.77E-19	19
RPS13.10	8.00E-24	-0.901706578	0.621	0.881	1.84E-19	19
UQCRH.7	1.39E-23	1.870860971	0.753	0.46	3.19E-19	19
RPS7.12	1.42E-23	-0.868532925	0.643	0.888	3.25E-19	19
RPS24.12	1.76E-23	-0.884775984	0.648	0.917	4.05E-19	19
DUSP2.17	1.98E-23	-2.455086702	0.071	0.436	4.55E-19	19
RPS9.8	2.24E-23	-0.917712142	0.654	0.881	5.16E-19	19
LINC00657.2	2.27E-23	0.743074757	0.434	0.157	5.22E-19	19
UGCG	2.31E-23	0.629742879	0.291	0.08	5.30E-19	19
CD53.12	2.74E-23	-1.370252124	0.104	0.476	6.30E-19	19
RPL3.10	2.90E-23	-0.848799717	0.692	0.935	6.67E-19	19
HPCAL1.4	2.97E-23	0.510059945	0.462	0.168	6.82E-19	19

C6orf89	3.59E-23	0.55420523	0.313	0.09	8.26E-19	19
AC159540.1	5.81E-23	0.620492145	0.286	0.08	1.33E-18	19
CHD9.3	8.55E-23	0.931719385	0.511	0.2	1.97E-18	19
TNFSF10.4	9.18E-23	0.874367601	0.434	0.164	2.11E-18	19
RNF114.1	9.61E-23	0.985009398	0.44	0.159	2.21E-18	19
RPL7.10	9.92E-23	-0.89525491	0.692	0.923	2.28E-18	19
LEPROT.4	1.10E-22	0.766544013	0.484	0.194	2.53E-18	19
AFF1.1	1.21E-22	0.797987193	0.335	0.104	2.77E-18	19
RRBP1.3	1.34E-22	0.737760142	0.412	0.151	3.08E-18	19
FOXJ3.1	1.39E-22	0.619536129	0.258	0.067	3.20E-18	19
ANKLE2	1.49E-22	1.03399895	0.346	0.112	3.42E-18	19
TBL1XR1.1	1.68E-22	1.149197344	0.456	0.174	3.86E-18	19
CD37.13	2.08E-22	-1.605293477	0.115	0.468	4.79E-18	19
HIPK2.2	2.21E-22	0.888991939	0.357	0.113	5.09E-18	19
CD3E.18	3.53E-22	-1.819016882	0.044	0.402	8.12E-18	19
SERPINA1.18	4.32E-22	1.157427352	0.566	0.275	9.94E-18	19
ACBD3	5.16E-22	0.625594657	0.269	0.072	1.19E-17	19
CCL5.17	5.86E-22	-2.583290031	0.269	0.566	1.35E-17	19
HLA-A.14	7.72E-22	-0.901315859	0.72	0.896	1.77E-17	19
CYBA.12	8.70E-22	-1.297915378	0.478	0.69	2.00E-17	19
POLR2H.1	1.37E-21	0.614415512	0.324	0.099	3.15E-17	19
RAB11FIP1	1.42E-21	0.529347775	0.379	0.125	3.26E-17	19
ABLIM1.1	1.72E-21	0.53206422	0.258	0.068	3.95E-17	19
RPL35.10	1.87E-21	-0.897494632	0.626	0.901	4.30E-17	19
PDE4D	1.96E-21	0.664803994	0.286	0.083	4.51E-17	19
PRKAG2.3	2.64E-21	1.120805839	0.412	0.148	6.07E-17	19
HSPB1.16	2.69E-21	1.115823182	0.67	0.431	6.18E-17	19
RPL6.10	2.85E-21	-0.909385888	0.593	0.886	6.55E-17	19
TRAC.19	3.07E-21	-1.99190101	0.088	0.437	7.06E-17	19
FYB.16	3.41E-21	-1.431407764	0.115	0.464	7.84E-17	19
FAM63B	3.61E-21	0.599076223	0.335	0.106	8.29E-17	19
ZBTB16.5	3.89E-21	1.154407504	0.511	0.217	8.94E-17	19
GOLGA2.2	4.02E-21	0.666277421	0.297	0.086	9.24E-17	19
RPL41.14	4.25E-21	-0.753406608	0.824	0.978	9.76E-17	19
CD44.13	4.57E-21	-1.701873708	0.181	0.495	1.05E-16	19
HNRNPA1.8	4.94E-21	-1.050769951	0.544	0.78	1.14E-16	19
GOLGB1.4	5.02E-21	0.984635399	0.527	0.218	1.15E-16	19
TTPAL	9.11E-21	0.883400506	0.269	0.079	2.09E-16	19
DDAH2.3	1.47E-20	0.619343009	0.363	0.125	3.37E-16	19
SERF2.7	2.25E-20	-0.983698041	0.538	0.821	5.18E-16	19
MYL12A.9	2.31E-20	-1.21687008	0.473	0.693	5.31E-16	19
ANKRD36.3	2.80E-20	0.688955492	0.374	0.129	6.44E-16	19
CD48.16	2.95E-20	-1.269549507	0.038	0.379	6.78E-16	19
CST7.16	3.23E-20	-2.220172516	0.066	0.393	7.42E-16	19
SFXN1.1	4.39E-20	0.679701961	0.324	0.103	1.01E-15	19
HLA-E.16	4.85E-20	-0.706873343	0.637	0.792	1.12E-15	19
TNFAIP3.17	5.16E-20	-1.689892935	0.121	0.451	1.19E-15	19
SEPT11.2	7.55E-20	0.745187576	0.363	0.123	1.73E-15	19
GDI1	1.31E-19	0.628850274	0.28	0.086	3.00E-15	19
RPS17.9	2.53E-19	-0.792369794	0.665	0.901	5.82E-15	19
PKM.11	2.94E-19	1.094906913	0.626	0.431	6.77E-15	19
MLEC.1	2.99E-19	0.503777284	0.423	0.164	6.88E-15	19
HMGN3.6	3.02E-19	1.013194626	0.588	0.302	6.94E-15	19
POLR2C	3.02E-19	0.548602912	0.396	0.141	6.94E-15	19
CCNL2	3.12E-19	0.833940657	0.297	0.098	7.17E-15	19
PDXDC1	3.37E-19	0.691902347	0.302	0.096	7.75E-15	19
RP11-347P5.1.18	4.25E-19	-1.705976575	0.055	0.38	9.77E-15	19
PPP2R5C.15	4.58E-19	-1.69178872	0.082	0.394	1.05E-14	19

RPL23.10	4.70E-19	-0.918070967	0.56	0.821	1.08E-14	19
CCL4.19	7.19E-19	-3.035587368	0.148	0.444	1.65E-14	19
KIAA0907	1.13E-18	0.916024869	0.302	0.099	2.60E-14	19
SREK1.3	1.32E-18	0.810976164	0.445	0.179	3.03E-14	19
SLC25A36	1.69E-18	0.791500344	0.335	0.117	3.88E-14	19
SELM.5	1.77E-18	0.646762966	0.418	0.181	4.07E-14	19
CFDP1.3	1.86E-18	0.850235919	0.462	0.188	4.29E-14	19
GZMA.18	1.91E-18	-2.244530084	0.126	0.423	4.39E-14	19
SAT2	2.52E-18	0.781856133	0.423	0.165	5.79E-14	19
LMF2	2.64E-18	0.583154588	0.269	0.081	6.07E-14	19
ELF1.14	3.63E-18	-1.550755766	0.165	0.444	8.34E-14	19
GLS	3.90E-18	0.62810498	0.357	0.129	8.95E-14	19
HLA-DRB5.15	5.19E-18	-2.060752453	0.066	0.377	1.19E-13	19
CELF2.14	5.81E-18	-1.171507138	0.115	0.43	1.34E-13	19
CFAP97.1	5.83E-18	0.608044167	0.385	0.144	1.34E-13	19
SOX4.2	6.90E-18	1.248325723	0.302	0.1	1.58E-13	19
RPS11.11	7.29E-18	-0.829040401	0.571	0.859	1.68E-13	19
CBR4.1	7.53E-18	0.647213788	0.258	0.08	1.73E-13	19
KLHDC2.2	7.65E-18	0.502639465	0.412	0.158	1.76E-13	19
GPSM3.13	9.77E-18	-1.341260759	0.099	0.394	2.25E-13	19
PFDN5.9	1.04E-17	-0.913508689	0.544	0.787	2.39E-13	19
RORA.4	1.04E-17	0.940654909	0.505	0.214	2.39E-13	19
IL2RG.19	1.17E-17	-1.477995849	0.071	0.376	2.70E-13	19
STK17B.14	1.25E-17	-1.475596841	0.104	0.404	2.87E-13	19
LCP1.16	1.55E-17	-1.142325252	0.093	0.408	3.57E-13	19
JUNB.15	1.61E-17	-1.266767518	0.522	0.726	3.69E-13	19
MKLN1.1	1.73E-17	0.679410851	0.28	0.091	3.98E-13	19
COX4I1.2	2.00E-17	-1.084990938	0.478	0.699	4.59E-13	19
PSME1.7	2.97E-17	-1.242605993	0.374	0.584	6.84E-13	19
METTL7A.3	2.98E-17	0.620420306	0.319	0.111	6.85E-13	19
RPL38.11	3.45E-17	-0.827896089	0.533	0.872	7.94E-13	19
SLA.15	4.17E-17	-1.161648778	0.099	0.403	9.59E-13	19
EVL.17	4.61E-17	-1.202927487	0.225	0.499	1.06E-12	19
GTF2I.3	4.68E-17	0.821064963	0.462	0.203	1.07E-12	19
WDR11	5.95E-17	0.913871014	0.291	0.1	1.37E-12	19
GATAD1.1	6.27E-17	0.593104026	0.275	0.09	1.44E-12	19
TRIM56.3	6.30E-17	0.782518148	0.445	0.187	1.45E-12	19
YBX1.10	8.20E-17	-1.198449992	0.467	0.673	1.88E-12	19
RPL5.8	8.51E-17	-0.836328353	0.571	0.856	1.96E-12	19
GZMK.19	1.13E-16	-2.789584033	0.088	0.369	2.60E-12	19
RNMT.1	1.17E-16	1.004154167	0.511	0.239	2.68E-12	19
CBWD1	1.30E-16	0.523034901	0.313	0.108	2.99E-12	19
RAC2.17	1.96E-16	-1.1530651	0.082	0.376	4.50E-12	19
BTF3.5	2.69E-16	-0.960535389	0.489	0.729	6.18E-12	19
TIPARP.3	2.98E-16	0.945717606	0.335	0.13	6.85E-12	19
RPL9.9	3.84E-16	-0.718898356	0.659	0.905	8.82E-12	19
USP8.1	3.91E-16	0.961137951	0.412	0.171	8.99E-12	19
SUMO2.7	4.75E-16	-1.161400661	0.407	0.61	1.09E-11	19
STK4.14	5.46E-16	-1.437216357	0.22	0.475	1.25E-11	19
CALM1.10	7.03E-16	-1.290019732	0.401	0.598	1.61E-11	19
LTB.16	7.24E-16	-1.70934333	0.027	0.305	1.66E-11	19
LMAN1.2	9.12E-16	0.580157475	0.429	0.188	2.10E-11	19
LAPTM5.14	1.04E-15	-1.223301083	0.368	0.57	2.38E-11	19
CARHSP1.5	1.33E-15	0.611788418	0.456	0.203	3.05E-11	19
H3F3A.10	1.55E-15	-1.018023421	0.516	0.742	3.56E-11	19
GIMAP4.13	1.61E-15	-1.370356797	0.071	0.342	3.71E-11	19
OAZ1.13	1.71E-15	-0.871149975	0.533	0.738	3.94E-11	19
EVI2B.11	1.74E-15	-1.100754164	0.055	0.339	4.00E-11	19

UQCRB.7	1.79E-15	-0.919703962	0.505	0.747	4.12E-11	19
TRBC2.18	2.36E-15	-1.725000015	0.11	0.38	5.43E-11	19
DDB2.3	3.21E-15	0.749344827	0.352	0.139	7.38E-11	19
TUBGCP2.1	3.35E-15	0.661504306	0.319	0.12	7.69E-11	19
PABPC1.9	3.64E-15	-0.870946687	0.516	0.664	8.36E-11	19
CD3G.18	5.21E-15	-1.276790536	0.027	0.297	1.20E-10	19
EEF1B2.6	6.00E-15	-0.965356988	0.473	0.664	1.38E-10	19
PARP14.3	6.50E-15	0.758554258	0.44	0.202	1.49E-10	19
CDK5RAP3.1	6.52E-15	0.654789149	0.379	0.154	1.50E-10	19
LSP1.18	6.82E-15	-1.341160451	0.187	0.438	1.57E-10	19
GOLGA8B.2	9.46E-15	0.703088221	0.269	0.094	2.17E-10	19
RPL24.12	1.11E-14	-0.780083177	0.566	0.856	2.54E-10	19
IARS	1.24E-14	0.597048807	0.264	0.091	2.84E-10	19
GIMAP7.16	1.34E-14	-1.421732993	0.033	0.288	3.07E-10	19
GOSR1	1.38E-14	0.660883825	0.28	0.098	3.17E-10	19
PSMD5-AS1.2	1.62E-14	0.749598265	0.275	0.099	3.73E-10	19
TRBC1.18	1.67E-14	-1.944271657	0.093	0.357	3.83E-10	19
PIK3IP1.17	2.22E-14	-1.348353044	0.099	0.361	5.11E-10	19
SAMSN1.11	2.35E-14	-1.25089875	0.06	0.326	5.40E-10	19
SPAG9.2	3.29E-14	0.781182723	0.346	0.143	7.57E-10	19
ITM2A.18	3.48E-14	-1.64999457	0.06	0.314	7.99E-10	19
RPL37.11	3.71E-14	-0.629799058	0.654	0.912	8.53E-10	19
LCK.19	4.36E-14	-1.101523565	0.033	0.291	1.00E-09	19
STK17A.16	5.27E-14	-1.275362871	0.121	0.37	1.21E-09	19
RPS5.9	5.87E-14	-0.818653523	0.544	0.845	1.35E-09	19
NKG7.19	6.30E-14	-2.4209643	0.198	0.43	1.45E-09	19
NBEAL1.6	6.76E-14	-1.0279776	0.44	0.617	1.55E-09	19
RGCC.16	7.02E-14	-1.860281303	0.055	0.31	1.61E-09	19
PRDM1.12	7.73E-14	-1.3843873	0.044	0.295	1.78E-09	19
CTSA.4	9.00E-14	0.531418923	0.412	0.195	2.07E-09	19
MT-CO2.11	1.03E-13	-0.618181229	0.78	0.876	2.37E-09	19
6-Mar	1.11E-13	0.832190028	0.341	0.139	2.55E-09	19
CD7.15	1.13E-13	-1.409745263	0.049	0.303	2.60E-09	19
MGST3.14	1.19E-13	0.629933483	0.5	0.304	2.74E-09	19
RPS8.11	1.51E-13	-0.695671125	0.714	0.92	3.48E-09	19
LDHA.14	1.54E-13	1.085534044	0.665	0.578	3.53E-09	19
OST4.6	1.59E-13	-0.904153799	0.434	0.663	3.66E-09	19
EVI2A.13	1.72E-13	-1.119977176	0.038	0.283	3.96E-09	19
CHD2.2	1.80E-13	0.790946313	0.407	0.18	4.13E-09	19
CTSD.10	2.42E-13	0.96364103	0.577	0.435	5.57E-09	19
LONP2	2.47E-13	0.871111826	0.313	0.127	5.69E-09	19
DDX17.10	2.66E-13	0.899311513	0.665	0.395	6.10E-09	19
ARPC1B.11	2.97E-13	-1.269933584	0.379	0.527	6.84E-09	19
CTSS.14	3.20E-13	-1.64362061	0.132	0.366	7.35E-09	19
ANKRD36B	3.24E-13	0.653204628	0.253	0.092	7.46E-09	19
LIMD2.17	3.25E-13	-1.17887087	0.071	0.314	7.46E-09	19
ZNF451	3.55E-13	0.519757149	0.286	0.108	8.16E-09	19
ARPC3.10	4.36E-13	-0.961636107	0.445	0.607	1.00E-08	19
TTC3	4.45E-13	0.581588641	0.473	0.232	1.02E-08	19
NDUFC1.13	4.96E-13	0.77733278	0.451	0.268	1.14E-08	19
TOMM7.10	5.33E-13	-0.787056804	0.484	0.758	1.22E-08	19
SNHG9.1	7.44E-13	1.357075386	0.319	0.132	1.71E-08	19
HSP90AA1.12	7.88E-13	-1.089070433	0.505	0.656	1.81E-08	19
RCSD1.11	1.04E-12	-0.936079424	0.022	0.252	2.38E-08	19
SUB1.7	1.14E-12	-1.151998873	0.429	0.576	2.61E-08	19
AHNAK.7	1.55E-12	0.736635476	0.516	0.291	3.55E-08	19
VEZT	1.63E-12	0.609215783	0.264	0.098	3.74E-08	19
TPI1.13	2.23E-12	0.904670991	0.659	0.563	5.13E-08	19

WSB1.8	2.62E-12	1.193686673	0.593	0.357	6.02E-08	19
EMB.15	2.63E-12	-0.796833102	0.038	0.272	6.05E-08	19
CLEC2B.11	2.89E-12	-1.335193971	0.17	0.381	6.65E-08	19
BIN2.13	3.12E-12	-1.055862471	0.027	0.252	7.18E-08	19
SH3KBP1.14	3.41E-12	-1.057402068	0.082	0.311	7.83E-08	19
CD27.17	3.97E-12	-1.619949293	0.049	0.27	9.12E-08	19
SRP14.7	4.56E-12	-0.87811202	0.456	0.719	1.05E-07	19
RPS20.9	4.76E-12	-0.603553885	0.648	0.9	1.09E-07	19
SARAF.11	4.85E-12	-0.714732926	0.516	0.68	1.11E-07	19
DARS.3	4.95E-12	0.649003365	0.423	0.205	1.14E-07	19
GNG11.3	5.75E-12	-0.504548542	0.352	0.141	1.32E-07	19
SLC35E3.3	5.87E-12	0.810196612	0.275	0.111	1.35E-07	19
BORCS7.1	6.23E-12	0.541726244	0.308	0.125	1.43E-07	19
FNBP1.17	6.55E-12	-1.081645878	0.148	0.37	1.51E-07	19
COMMD6.8	6.66E-12	-0.987344562	0.423	0.608	1.53E-07	19
IER3.3	7.02E-12	0.577018097	0.269	0.107	1.61E-07	19
FYN.12	7.50E-12	-1.054622592	0.049	0.269	1.72E-07	19
PNRC1.11	7.89E-12	-0.908013582	0.5	0.636	1.81E-07	19
MYO1F.13	8.23E-12	-0.966503847	0.049	0.274	1.89E-07	19
PHPT1.10	8.97E-12	0.61775413	0.467	0.273	2.06E-07	19
MYL12B.5	9.20E-12	-0.958465644	0.451	0.637	2.11E-07	19
PRKCH.14	1.13E-11	-0.772113809	0.033	0.256	2.59E-07	19
ZKSCAN1.2	1.43E-11	0.914777867	0.302	0.127	3.29E-07	19
PRDX2.11	1.45E-11	0.532706594	0.445	0.262	3.33E-07	19
NME3.1	1.45E-11	0.534418496	0.39	0.185	3.33E-07	19
WIPF1.17	1.84E-11	-1.105166687	0.132	0.346	4.22E-07	19
RPL36A.12	1.97E-11	-0.628625455	0.56	0.804	4.54E-07	19
FXYD5.11	2.02E-11	-0.932327589	0.313	0.483	4.63E-07	19
IL7R.16	2.28E-11	-1.982626916	0.088	0.306	5.25E-07	19
NR4A2.15	2.42E-11	-1.16507378	0.06	0.279	5.57E-07	19
FNIP1.1	2.49E-11	0.515277294	0.33	0.143	5.71E-07	19
SRGAP1.3	2.55E-11	0.996945939	0.264	0.108	5.86E-07	19
RPL36.15	2.59E-11	-0.65642097	0.687	0.915	5.95E-07	19
EXOC7	2.74E-11	0.838503942	0.313	0.136	6.30E-07	19
LUC7L3.11	2.82E-11	0.936533408	0.555	0.308	6.47E-07	19
ISG20.13	3.13E-11	-1.141646145	0.154	0.364	7.19E-07	19
ATP5L.8	3.33E-11	-0.819510296	0.505	0.677	7.66E-07	19
RPS27L.12	3.96E-11	0.551541482	0.615	0.471	9.10E-07	19
COTL1.13	4.51E-11	-1.418473668	0.264	0.436	1.04E-06	19
ITGB2.16	4.59E-11	-1.242968135	0.236	0.417	1.06E-06	19
CANX.11	5.55E-11	0.656753731	0.467	0.297	1.28E-06	19
EZR.7	5.61E-11	0.742695953	0.495	0.322	1.29E-06	19
GLIPR1.13	6.01E-11	-1.00488842	0.088	0.302	1.38E-06	19
LAPTM4A.13	6.25E-11	0.762000016	0.5	0.35	1.44E-06	19
SQSTM1.5	7.98E-11	0.617914072	0.522	0.311	1.83E-06	19
LGALS1.10	8.45E-11	-1.165894566	0.429	0.574	1.94E-06	19
CCDC14.1	9.21E-11	0.517357959	0.258	0.103	2.12E-06	19
TRAF3IP3.17	9.25E-11	-1.0432731	0.055	0.258	2.13E-06	19
PEBP1.14	1.17E-10	0.564490138	0.544	0.388	2.69E-06	19
SLC38A2.4	1.19E-10	0.670981781	0.423	0.205	2.75E-06	19
NACA.9	1.32E-10	-0.655183143	0.56	0.807	3.03E-06	19
AKNA.12	1.35E-10	-0.911840483	0.06	0.264	3.11E-06	19
KIAA1551.14	1.53E-10	-1.252246909	0.143	0.338	3.51E-06	19
IFI16.8	1.58E-10	-1.206392513	0.335	0.483	3.64E-06	19
HERC4	1.66E-10	0.556457282	0.269	0.112	3.82E-06	19
FUNDC2.4	1.81E-10	0.505073719	0.374	0.187	4.15E-06	19
AFF4.2	1.81E-10	0.7026644	0.352	0.164	4.15E-06	19
TXNIP.14	1.94E-10	-0.867291222	0.703	0.764	4.46E-06	19

OCIAD2.14	1.98E-10	0.521830414	0.451	0.258	4.56E-06	19
HM13.1	2.14E-10	0.502321389	0.385	0.188	4.91E-06	19
CD96.18	3.58E-10	-0.850350337	0.049	0.251	8.23E-06	19
ID2.13	4.18E-10	-1.258916296	0.434	0.566	9.60E-06	19
PHF14.2	4.25E-10	0.594426055	0.39	0.189	9.77E-06	19
EIF3E.7	4.53E-10	-0.970726585	0.407	0.547	1.04E-05	19
ETS1.16	4.56E-10	-1.0224729	0.137	0.341	1.05E-05	19
SLC25A6.9	4.67E-10	-0.737964319	0.511	0.657	1.07E-05	19
SPG7.1	4.71E-10	0.545473987	0.297	0.128	1.08E-05	19
UBB.10	4.93E-10	-0.792644333	0.478	0.723	1.13E-05	19
RPL8.7	7.63E-10	-0.540413661	0.67	0.885	1.75E-05	19
IL10RA.14	9.17E-10	-1.050456359	0.077	0.261	2.11E-05	19
ALDOA.8	1.01E-09	0.866830543	0.654	0.637	2.32E-05	19
SEPT6.15	1.01E-09	-0.91723756	0.082	0.28	2.32E-05	19
RPL22.9	1.06E-09	-0.54375709	0.544	0.814	2.43E-05	19
ANKRD44.11	1.29E-09	-0.75816633	0.071	0.261	2.97E-05	19
ARF6.11	1.36E-09	-1.13999304	0.209	0.382	3.13E-05	19
FOS.17	1.50E-09	-0.543807496	0.538	0.683	3.44E-05	19
ACTG1.11	1.55E-09	-0.648700059	0.566	0.748	3.55E-05	19
ATF6B	1.75E-09	0.66139488	0.418	0.214	4.01E-05	19
SMAP2.13	1.87E-09	-1.070155625	0.121	0.3	4.30E-05	19
ACAP1.18	1.97E-09	-0.77972221	0.132	0.336	4.54E-05	19
GABPB1-AS1.3	1.98E-09	0.695703907	0.423	0.226	4.54E-05	19
CSNK1A1.5	2.21E-09	0.658255925	0.511	0.301	5.08E-05	19
ANKRD36C.3	2.83E-09	0.658067311	0.352	0.175	6.50E-05	19
TNRC6A	3.10E-09	0.625759394	0.269	0.115	7.12E-05	19
SYNE1.4	3.33E-09	0.556357737	0.341	0.163	7.66E-05	19
DOCK8.14	3.63E-09	-0.68984806	0.104	0.294	8.34E-05	19
DDX24.13	3.67E-09	-0.796138243	0.291	0.459	8.42E-05	19
SRSF1	4.13E-09	0.59504495	0.341	0.164	9.48E-05	19
C14orf2.6	5.42E-09	-0.965293267	0.39	0.517	0.000124611	19
COX7A2.6	6.00E-09	-0.873441317	0.423	0.563	0.000137915	19
SEPT7.11	6.54E-09	-0.955669689	0.396	0.514	0.000150283	19
CAPZB.9	6.84E-09	-1.050270498	0.341	0.462	0.000157121	19
FOXP1.10	6.96E-09	-1.027826534	0.082	0.26	0.000159841	19
FAM49B.12	7.29E-09	-1.023208593	0.17	0.342	0.000167421	19
INSR.7	8.56E-09	-0.501333582	0.302	0.135	0.000196703	19
EIF3K.4	9.68E-09	-0.882842779	0.423	0.551	0.000222548	19
GNLY.15	9.77E-09	-2.482256058	0.071	0.255	0.000224521	19
UBL5.7	1.12E-08	-0.873457797	0.401	0.564	0.000258255	19
VIMP.4	1.20E-08	0.52678664	0.429	0.226	0.000275077	19
MT-ND3.12	1.29E-08	-0.500317837	0.692	0.832	0.000296305	19
RTN4.14	1.31E-08	0.50933534	0.516	0.348	0.000300189	19
BRK1.7	1.35E-08	-1.112601245	0.33	0.44	0.000309172	19
CARD16.11	1.43E-08	-0.976936152	0.148	0.319	0.000327505	19
SYTL3.16	1.43E-08	-1.196883215	0.11	0.279	0.000328979	19
ATP1A1.8	1.83E-08	0.566875597	0.456	0.26	0.000419458	19
CRIP1.15	1.83E-08	-1.140626434	0.115	0.296	0.000420242	19
CKLF.12	1.92E-08	-1.09753642	0.214	0.365	0.000440625	19
HLA-DPB1.15	2.00E-08	-1.796911067	0.604	0.633	0.000458882	19
MYL6.10	2.34E-08	-0.569613578	0.599	0.799	0.000538466	19
P4HB.8	2.34E-08	0.677498606	0.478	0.343	0.000538495	19
GTF3A.6	2.35E-08	-1.082570081	0.242	0.382	0.000539933	19
UQCR11.5	2.36E-08	-0.829710772	0.44	0.612	0.00054155	19
AKAP9.9	2.58E-08	0.631421352	0.56	0.339	0.000592167	19
TTC19.2	2.63E-08	0.781368276	0.379	0.195	0.000604146	19
JUND.9	2.76E-08	-0.698617209	0.137	0.312	0.000634321	19
EMP3.9	2.83E-08	-1.036526148	0.236	0.392	0.000649764	19

DDIT4.12	2.90E-08	0.564015741	0.67	0.516	0.000666803	19
RGS2.16	3.21E-08	-1.236090063	0.17	0.337	0.000738413	19
RHOA.14	4.15E-08	-0.992645633	0.423	0.509	0.000952625	19
DSTN.15	4.28E-08	0.50057574	0.445	0.306	0.000984387	19
HLA-F.13	4.32E-08	-1.067914864	0.313	0.44	0.000991856	19
CD99.13	4.78E-08	-1.020733114	0.363	0.471	0.001097359	19
SRSF10.6	5.14E-08	0.715833855	0.44	0.244	0.0011809	19
ARHGEF1.14	5.46E-08	-0.950045946	0.104	0.264	0.001254052	19
CLEC2D.18	5.66E-08	-0.935360279	0.137	0.309	0.001301551	19
SMCHD1.8	6.35E-08	-0.727186888	0.154	0.33	0.001458725	19
ADGRE5.16	6.54E-08	-0.955099749	0.143	0.301	0.001502447	19
SNRNP70.4	1.01E-07	0.526630689	0.44	0.246	0.00231869	19
LINC00152.12	1.10E-07	-1.220244666	0.17	0.319	0.002525204	19
HSPA8.11	1.14E-07	-0.873266151	0.462	0.556	0.002628623	19
FKBP5.12	1.21E-07	0.603701149	0.61	0.393	0.002777297	19
S100A9.16	1.32E-07	-2.158498959	0.082	0.253	0.003027098	19
PRPF38B.14	1.42E-07	-0.870910628	0.313	0.437	0.003261326	19
EIF3F.3	1.64E-07	-0.882206894	0.374	0.468	0.003773789	19
UPF2.1	1.92E-07	0.606357689	0.396	0.217	0.004402426	19
ARPC5.10	2.03E-07	-0.940619417	0.335	0.437	0.004661435	19
COX6B1.4	2.14E-07	-0.803993696	0.407	0.575	0.004907371	19
TES.1	2.19E-07	0.508023615	0.319	0.158	0.005027617	19
ITGB1.11	2.38E-07	-1.330459756	0.374	0.465	0.005468647	19
MYEOV2.6	2.38E-07	-0.943159057	0.214	0.349	0.005470657	19
GNAI2.13	2.84E-07	-0.903792666	0.121	0.262	0.006534622	19
RGS10.12	3.12E-07	-1.031783341	0.154	0.297	0.007159808	19
KLF6.15	3.39E-07	0.757255672	0.813	0.647	0.007785104	19
HMGB2.14	4.06E-07	-0.997561457	0.396	0.477	0.009319386	19
SMARCC2	4.70E-07	0.515861597	0.264	0.128	0.010799708	19
NEDD9.3	5.59E-07	0.62545583	0.324	0.167	0.012838057	19
HCLS1.12	5.94E-07	-0.645376582	0.17	0.324	0.013650157	19
MSN.10	6.62E-07	-0.953397727	0.253	0.368	0.015215429	19
DNAJB6.5	6.75E-07	-0.989070379	0.214	0.341	0.015508783	19
CDC42.4	6.76E-07	-0.947462138	0.396	0.492	0.01553784	19
CLIC1.7	7.11E-07	-0.659032351	0.451	0.634	0.016336309	19
ATP1B3.13	7.76E-07	-1.042286418	0.137	0.27	0.0178376	19
UBXN1.5	9.42E-07	-0.712276404	0.286	0.393	0.021656628	19
HLA-DQA1.13	1.26E-06	-1.71829199	0.225	0.345	0.028986198	19
ARL6IP5.12	1.38E-06	-0.811902483	0.396	0.458	0.031646796	19
SH3BGRL.11	1.43E-06	-1.113032481	0.308	0.391	0.032754676	19
NAP1L1.7	1.52E-06	-0.703264212	0.473	0.566	0.034861029	19
N4BP2L2.11	1.60E-06	0.861677537	0.72	0.527	0.036811912	19
IFITM2.11	1.61E-06	-1.142035356	0.379	0.477	0.036891143	19
PSME2.8	1.66E-06	-1.042316789	0.324	0.414	0.038077987	19
LEPROTL1.10	1.71E-06	-1.093319348	0.286	0.391	0.039301644	19
CCNI.7	1.77E-06	-0.647998475	0.473	0.595	0.040694381	19
PSMB9.10	2.00E-06	-0.549314453	0.401	0.485	0.045932739	19
TAGLN.1	1.42E-191	4.223010109	0.607	0.063	3.26E-187	20
TPM2.1	2.16E-168	3.832672646	0.509	0.049	4.97E-164	20
RGS5.3	1.08E-154	4.16082084	0.723	0.119	2.47E-150	20
ACTA2.2	9.11E-152	4.715776277	0.734	0.128	2.09E-147	20
THY1.1	5.05E-147	3.78523424	0.439	0.041	1.16E-142	20
MGP.4	4.43E-130	3.525070657	0.734	0.143	1.02E-125	20
CALD1.4	1.16E-122	3.660676237	0.705	0.142	2.68E-118	20
PLAC9.1	3.33E-112	3.069335586	0.324	0.028	7.66E-108	20
MYL9.1	3.24E-111	3.601791039	0.497	0.071	7.44E-107	20
FRZB.1	9.87E-108	2.738936301	0.289	0.023	2.27E-103	20
IGFBP7.16	2.26E-101	3.267056995	0.925	0.372	5.19E-97	20

BGN.1	1.10E-76	3.133334738	0.393	0.061	2.54E-72	20
SPARC.5	1.93E-66	2.713029138	0.624	0.177	4.43E-62	20
LGALS1.11	3.34E-58	2.731484248	0.838	0.569	7.68E-54	20
CPE.1	2.73E-52	2.619906072	0.26	0.038	6.27E-48	20
ADIRF.13	4.85E-49	3.168238781	0.613	0.237	1.12E-44	20
CD36.2	6.72E-44	2.577593097	0.277	0.05	1.55E-39	20
RGS1.19	4.38E-37	-2.171573191	0.145	0.704	1.01E-32	20
PTPRC.17	4.41E-37	-1.782144959	0.064	0.623	1.01E-32	20
NDUFA4L2.19	7.41E-37	2.174892354	0.624	0.284	1.70E-32	20
CXCR4.14	6.63E-36	-1.67408141	0.208	0.782	1.52E-31	20
TIMP1.17	7.11E-35	3.020503666	0.595	0.311	1.63E-30	20
SARAF.12	6.30E-34	-1.065701662	0.11	0.685	1.45E-29	20
BTG1.19	1.94E-33	-1.476553957	0.318	0.849	4.45E-29	20
MT2A.19	3.06E-33	1.584510286	0.815	0.657	7.03E-29	20
CD74.14	7.59E-32	-1.979295752	0.26	0.801	1.74E-27	20
LAPTM5.15	2.81E-31	-1.675177321	0.069	0.573	6.45E-27	20
NEAT1.13	5.41E-31	-1.531476299	0.214	0.76	1.24E-26	20
TXNIP.15	5.70E-31	-1.436915114	0.225	0.77	1.31E-26	20
ZFP36L2.16	8.89E-31	-1.562794075	0.168	0.705	2.04E-26	20
CORO1A.16	3.87E-30	-1.303036755	0.075	0.571	8.90E-26	20
HLA-E.17	1.40E-29	-1.071063689	0.214	0.797	3.22E-25	20
RPS29.15	2.42E-29	-1.019755952	0.607	0.925	5.55E-25	20
HCST.15	5.94E-29	-1.471500599	0.069	0.55	1.37E-24	20
FYB.17	7.22E-28	-1.939473299	0.023	0.465	1.66E-23	20
SRGN.16	8.20E-28	-1.335858932	0.266	0.798	1.88E-23	20
PNISR.9	1.61E-27	-1.417997805	0.052	0.516	3.70E-23	20
SH3BGRL3.16	1.76E-27	-0.966613705	0.162	0.688	4.06E-23	20
KLF6.16	2.58E-27	-1.110060954	0.139	0.655	5.94E-23	20
CCL5.18	3.66E-27	-2.111255127	0.104	0.567	8.42E-23	20
SAT1.14	5.42E-27	-1.685203526	0.15	0.637	1.25E-22	20
EVL.18	6.15E-27	-1.521059455	0.052	0.501	1.41E-22	20
SRSF7.16	6.36E-27	-1.280616056	0.081	0.55	1.46E-22	20
ID3.3	7.47E-27	2.92715178	0.382	0.137	1.72E-22	20
STK4.15	1.79E-26	-1.607376798	0.04	0.477	4.10E-22	20
ID2.14	1.88E-26	-1.325423893	0.092	0.57	4.31E-22	20
LSP1.19	2.44E-26	-1.681937294	0.012	0.44	5.62E-22	20
HMGN1.8	6.45E-26	-1.028497116	0.052	0.51	1.48E-21	20
MT-ND1.11	7.12E-26	-1.050827575	0.26	0.793	1.64E-21	20
ZFP36.12	2.16E-25	-1.17692922	0.168	0.67	4.95E-21	20
CD44.14	4.37E-25	-1.194499397	0.052	0.497	1.00E-20	20
NDUFA4.10	5.54E-25	-1.151934253	0.092	0.545	1.27E-20	20
CD53.13	8.71E-25	-1.312440266	0.052	0.476	2.00E-20	20
TMA7.12	1.25E-24	-0.557401155	0.179	0.702	2.87E-20	20
S100A10.11	3.14E-24	-0.919923529	0.133	0.616	7.21E-20	20
CD37.14	5.68E-24	-1.284854293	0.052	0.468	1.31E-19	20
DSTN.16	7.71E-24	2.467682318	0.532	0.305	1.77E-19	20
CELF2.15	8.97E-24	-1.3921993	0.029	0.431	2.06E-19	20
CD52.15	3.19E-23	-1.317344175	0.11	0.554	7.33E-19	20
TPM3.10	3.80E-23	-1.096936222	0.046	0.458	8.73E-19	20
S100A6.10	6.21E-23	-0.828230996	0.249	0.773	1.43E-18	20
HLA-DPA1.16	7.50E-23	-1.774767348	0.145	0.576	1.72E-18	20
TRAC.20	1.82E-22	-1.964464868	0.046	0.437	4.19E-18	20
HLA-DPB1.16	2.93E-22	-1.49719861	0.191	0.638	6.73E-18	20
CD2.19	8.85E-22	-1.591283039	0.064	0.462	2.03E-17	20
ITGB2.17	9.24E-22	-1.469161747	0.04	0.42	2.12E-17	20
MT-ND2.11	9.81E-22	-0.868457773	0.445	0.871	2.25E-17	20
AC090498.1.11	1.14E-21	-1.109013666	0.087	0.506	2.61E-17	20
S100A4.17	1.30E-21	-0.849933089	0.22	0.705	2.98E-17	20

HLA-DRB1.15	1.94E-21	-1.604084804	0.162	0.596	4.47E-17	20
VAMP8.11	2.19E-21	-0.783040917	0.064	0.466	5.03E-17	20
GMFG.11	2.21E-21	-0.688316818	0.121	0.561	5.07E-17	20
HLA-F.14	2.52E-21	-1.025432427	0.052	0.443	5.79E-17	20
GZMA.19	3.56E-21	-1.885325443	0.046	0.424	8.18E-17	20
FXYD5.12	4.12E-21	-0.653680124	0.081	0.486	9.47E-17	20
MALAT1.18	4.39E-21	-0.796982344	0.971	0.983	1.01E-16	20
RPL17.13	5.29E-21	-0.895642883	0.15	0.599	1.22E-16	20
RPS15A.11	5.82E-21	-0.736214683	0.618	0.938	1.34E-16	20
CYTIP.17	6.08E-21	-1.052893467	0.081	0.48	1.40E-16	20
MT-ND5.11	7.70E-21	-0.567201235	0.156	0.616	1.77E-16	20
PABPC1.10	9.66E-21	-0.562714717	0.185	0.669	2.22E-16	20
HMGB2.15	1.16E-20	-0.910785918	0.081	0.481	2.67E-16	20
TSPO.10	1.37E-20	-0.927600709	0.046	0.425	3.15E-16	20
TNFAIP3.18	1.47E-20	-1.242061365	0.064	0.452	3.39E-16	20
DAZAP2.8	1.98E-20	-0.943697744	0.035	0.404	4.55E-16	20
SNHG8.8	2.19E-20	-0.865734593	0.052	0.439	5.03E-16	20
CD69.18	2.50E-20	-1.526771527	0.127	0.534	5.74E-16	20
PRRC2C.10	3.42E-20	-0.889722863	0.058	0.438	7.86E-16	20
MCL1.10	3.74E-20	-0.639975873	0.104	0.52	8.60E-16	20
CCNL1.11	3.86E-20	-1.012770389	0.035	0.403	8.87E-16	20
CD3E.19	3.89E-20	-1.438561966	0.04	0.402	8.95E-16	20
ARGLU1.11	4.84E-20	-0.935246284	0.058	0.438	1.11E-15	20
LCP1.17	4.90E-20	-1.339057583	0.046	0.409	1.13E-15	20
HLA-DRA.17	5.19E-20	-1.735404314	0.243	0.678	1.19E-15	20
HLA-DQB1.14	7.66E-20	-1.57477282	0.035	0.397	1.76E-15	20
FAM49B.13	9.35E-20	-1.437932961	0.006	0.344	2.15E-15	20
SPCS1.3	1.69E-19	-0.786980047	0.04	0.405	3.89E-15	20
RSRP1.11	2.14E-19	-0.722951505	0.046	0.417	4.91E-15	20
DDX3X.13	2.70E-19	-0.810638069	0.052	0.419	6.20E-15	20
COTL1.14	2.76E-19	-1.094922144	0.069	0.439	6.35E-15	20
TPM1.6	5.10E-19	2.303028308	0.405	0.192	1.17E-14	20
FOSB.9	5.10E-19	-1.271326095	0.064	0.43	1.17E-14	20
CD3D.20	5.25E-19	-1.068010692	0.092	0.479	1.21E-14	20
KMT2E.13	5.85E-19	-0.838382698	0.046	0.409	1.34E-14	20
MYL6.11	6.04E-19	1.40569081	0.717	0.798	1.39E-14	20
EIF3F.4	6.29E-19	-0.506804951	0.081	0.471	1.45E-14	20
IL2RG.20	6.82E-19	-1.328564197	0.035	0.377	1.57E-14	20
PRPF38B.15	9.46E-19	-0.71959453	0.069	0.44	2.17E-14	20
IFI16.9	9.93E-19	-0.650671905	0.092	0.486	2.28E-14	20
DUSP2.18	1.01E-18	-1.365303271	0.075	0.436	2.32E-14	20
CD48.17	1.02E-18	-1.102572849	0.035	0.379	2.34E-14	20
RAC2.18	1.02E-18	-1.291922536	0.035	0.377	2.35E-14	20
TRBC2.19	1.11E-18	-1.555351406	0.04	0.381	2.56E-14	20
MT-ND3.13	1.25E-18	-0.723614828	0.399	0.836	2.88E-14	20
CLEC2B.12	1.32E-18	-1.020580632	0.035	0.382	3.03E-14	20
ELF1.15	1.42E-18	-0.68057832	0.069	0.445	3.26E-14	20
RNF213.14	1.48E-18	-0.807763126	0.04	0.392	3.40E-14	20
IFITM3.17	1.52E-18	1.997593076	0.555	0.376	3.49E-14	20
HMGN2.6	2.13E-18	-0.567108524	0.11	0.505	4.88E-14	20
CCL4.20	2.39E-18	-1.826989477	0.081	0.444	5.49E-14	20
IER2.9	3.10E-18	-1.058286726	0.052	0.405	7.11E-14	20
POLR2J3.11	3.55E-18	-0.828993458	0.046	0.399	8.15E-14	20
AAK1.17	4.16E-18	-0.91442794	0.035	0.375	9.57E-14	20
KIAA1551.15	5.43E-18	-1.319320238	0.017	0.339	1.25E-13	20
DNAJB1.13	7.22E-18	-0.98427714	0.064	0.424	1.66E-13	20
FUS.14	7.52E-18	-0.53727438	0.092	0.474	1.73E-13	20
MT-ND4L.10	7.62E-18	-1.063387153	0.035	0.365	1.75E-13	20

LDHA.15	9.84E-18	-0.715467077	0.162	0.584	2.26E-13	20
PRR13.6	9.90E-18	-0.555163656	0.046	0.396	2.28E-13	20
RPSA.12	1.28E-17	-0.632774761	0.329	0.81	2.95E-13	20
HERPUD1.13	1.43E-17	-0.886812809	0.116	0.497	3.28E-13	20
ARL6IP5.13	1.45E-17	-0.521619439	0.092	0.462	3.34E-13	20
LITAF.13	1.53E-17	-1.01411143	0.04	0.371	3.53E-13	20
UCP2.15	1.55E-17	-1.184031698	0.029	0.35	3.57E-13	20
ATRX.11	1.75E-17	-0.881463094	0.052	0.391	4.02E-13	20
SRRM2.7	1.85E-17	-1.005367196	0.023	0.345	4.24E-13	20
ANKRD12.10	1.88E-17	-0.821357249	0.052	0.393	4.33E-13	20
AKAP13.12	2.06E-17	-0.633060226	0.075	0.436	4.74E-13	20
SERP1.6	2.10E-17	-0.602905929	0.081	0.441	4.82E-13	20
ACAP1.19	2.93E-17	-1.293278201	0.023	0.337	6.74E-13	20
ARF6.12	3.10E-17	-0.669362926	0.046	0.384	7.14E-13	20
WIPF1.18	4.06E-17	-1.034223511	0.029	0.347	9.33E-13	20
LEPROTL1.11	4.74E-17	-0.711231576	0.052	0.393	1.09E-12	20
REL.13	5.23E-17	-1.220734723	0.017	0.326	1.20E-12	20
DDX17.11	7.51E-17	-0.799348227	0.064	0.403	1.73E-12	20
DOCK8.15	8.67E-17	-1.184917013	0	0.295	1.99E-12	20
IDS.12	8.83E-17	-0.755265539	0.046	0.377	2.03E-12	20
ISG20.14	9.62E-17	-1.045303265	0.046	0.365	2.21E-12	20
GPSM3.14	1.37E-16	-0.627600726	0.058	0.394	3.15E-12	20
ALOX5AP.15	1.82E-16	-1.365678211	0.023	0.332	4.18E-12	20
PIK3IP1.18	1.84E-16	-1.11559093	0.046	0.362	4.22E-12	20
TRBC1.19	2.68E-16	-1.700230425	0.046	0.357	6.15E-12	20
SLA.16	3.13E-16	-0.762069655	0.069	0.403	7.19E-12	20
MBNL1.10	4.45E-16	-0.752352976	0.04	0.359	1.02E-11	20
CTSS.15	4.75E-16	-1.375077307	0.052	0.367	1.09E-11	20
GCC2.11	5.73E-16	-0.729968094	0.046	0.363	1.32E-11	20
RNASET2.17	5.89E-16	-1.059304357	0.046	0.365	1.35E-11	20
XIST.13	6.70E-16	-0.782144142	0.087	0.428	1.54E-11	20
BTG2.10	6.88E-16	-0.844875924	0.046	0.361	1.58E-11	20
LIMD2.18	7.04E-16	-1.03906965	0.017	0.314	1.62E-11	20
RP11-347P5.1.19	8.67E-16	-1.059991536	0.064	0.379	1.99E-11	20
RSRC2.10	8.83E-16	-0.602071171	0.058	0.382	2.03E-11	20
TCF25.6	9.59E-16	-0.501231504	0.081	0.418	2.20E-11	20
CST7.17	1.03E-15	-1.206621581	0.069	0.393	2.37E-11	20
YPEL5.12	1.18E-15	-0.857958379	0.04	0.348	2.71E-11	20
STK17A.17	1.36E-15	-0.685127601	0.052	0.37	3.12E-11	20
FNBP1.18	2.16E-15	-0.709079289	0.058	0.371	4.96E-11	20
TRA2B.7	2.34E-15	-0.608960779	0.058	0.373	5.39E-11	20
SPARCL1.4	2.51E-15	0.83610168	0.376	0.161	5.77E-11	20
AKAP9.10	2.66E-15	-0.809472521	0.04	0.345	6.11E-11	20
TSC22D3.12	2.93E-15	-0.677965397	0.335	0.786	6.73E-11	20
CCND3.8	3.73E-15	-0.777808908	0.058	0.363	8.58E-11	20
EVI2B.12	4.53E-15	-0.781970756	0.04	0.339	1.04E-10	20
ZNF90.8	6.79E-15	-0.640288151	0.052	0.357	1.56E-10	20
RGS2.17	8.85E-15	-1.410526064	0.046	0.339	2.03E-10	20
GPBP1.12	9.10E-15	-0.538648416	0.046	0.349	2.09E-10	20
ARL6IP1.6	1.12E-14	-0.605062149	0.035	0.324	2.56E-10	20
EVI2A.14	1.13E-14	-0.983668317	0.012	0.283	2.60E-10	20
SMAP2.14	1.20E-14	-1.018056327	0.023	0.301	2.75E-10	20
RPS21.12	1.28E-14	-0.670081648	0.422	0.868	2.94E-10	20
CLEC2D.19	1.49E-14	-1.087927323	0.029	0.31	3.42E-10	20
ITM2A.19	1.86E-14	-1.304442522	0.035	0.314	4.28E-10	20
CDC42SE2.11	1.92E-14	-0.722155563	0.035	0.319	4.42E-10	20
NR4A2.16	2.09E-14	-1.284344232	0.012	0.28	4.81E-10	20
EIF4B.5	2.44E-14	-0.634177682	0.058	0.361	5.61E-10	20

IRF1.14	2.45E-14	-1.029448001	0.035	0.314	5.62E-10	20
LCK.20	3.48E-14	-1.141978596	0.023	0.291	7.99E-10	20
SMCHD1.9	4.34E-14	-0.786084063	0.046	0.331	9.98E-10	20
RPL28.13	8.01E-14	-0.504349096	0.653	0.936	1.84E-09	20
SERPINA1.19	8.07E-14	-1.576806905	0.017	0.282	1.86E-09	20
TAF1D.9	8.32E-14	-0.553342716	0.04	0.322	1.91E-09	20
GZMK.20	8.88E-14	-1.657489005	0.081	0.369	2.04E-09	20
PRKCH.15	1.04E-13	-1.09732975	0.006	0.256	2.39E-09	20
HSPB1.17	1.16E-13	1.845537508	0.543	0.433	2.66E-09	20
MAP1LC3B.4	1.38E-13	-0.631727019	0.04	0.319	3.16E-09	20
NKG7.20	1.70E-13	-1.431865723	0.127	0.431	3.90E-09	20
ANXA1.16	1.86E-13	-0.745560797	0.139	0.475	4.26E-09	20
HSD17B11.7	1.87E-13	-0.8225247	0.006	0.255	4.30E-09	20
SERPINB1.7	2.08E-13	-1.009301636	0.017	0.272	4.77E-09	20
SYTL3.17	2.29E-13	-1.14898301	0.023	0.28	5.26E-09	20
DUSP1.15	2.74E-13	-0.604278983	0.364	0.799	6.30E-09	20
PPP1CA.5	2.83E-13	-0.504016605	0.075	0.368	6.50E-09	20
CNN3.5	2.87E-13	2.07949485	0.26	0.106	6.59E-09	20
LTB.17	3.00E-13	-1.355422067	0.04	0.305	6.90E-09	20
ETS1.17	3.27E-13	-0.5894133	0.058	0.342	7.52E-09	20
PLP2.10	3.63E-13	-0.723729881	0.035	0.298	8.35E-09	20
CCNH.14	4.25E-13	-0.822242075	0.029	0.287	9.76E-09	20
DHRS7.8	4.69E-13	-0.591278997	0.023	0.278	1.08E-08	20
ADGRE5.17	4.71E-13	-0.946362556	0.04	0.302	1.08E-08	20
UFC1.3	4.84E-13	-0.552733705	0.052	0.327	1.11E-08	20
HLA-DMA.13	6.29E-13	-0.996011006	0.029	0.29	1.45E-08	20
GLRX.10	6.35E-13	-0.554881778	0.035	0.301	1.46E-08	20
PPP1R15A.9	8.83E-13	-0.592034004	0.104	0.407	2.03E-08	20
TUBA4A.17	9.11E-13	-0.869615414	0.023	0.276	2.09E-08	20
SAMSN1.12	9.16E-13	-0.578736084	0.052	0.326	2.10E-08	20
GNAI2.14	9.21E-13	-0.630517208	0.017	0.263	2.12E-08	20
GIMAP4.14	9.57E-13	-0.506406332	0.064	0.342	2.20E-08	20
MYO1F.14	9.59E-13	-0.550402151	0.023	0.275	2.20E-08	20
SEPT6.16	9.59E-13	-0.843339924	0.029	0.281	2.20E-08	20
PTP4A2.10	9.66E-13	-0.71195705	0.023	0.274	2.22E-08	20
CD3G.19	9.89E-13	-0.798569697	0.035	0.296	2.27E-08	20
PNN.10	1.14E-12	-0.585190922	0.04	0.307	2.62E-08	20
PCSK7.16	1.20E-12	-0.522842113	0.052	0.324	2.77E-08	20
RGS10.13	1.25E-12	-0.665754007	0.04	0.298	2.88E-08	20
SMDT1.5	1.30E-12	-0.521989329	0.035	0.294	3.00E-08	20
APOBEC3G.17	1.35E-12	-0.748797748	0.023	0.275	3.09E-08	20
EMB.16	1.44E-12	-0.705846742	0.023	0.272	3.30E-08	20
PIK3R1.14	1.45E-12	-0.708926459	0.046	0.312	3.33E-08	20
PSMA3-AS1.10	1.47E-12	-0.903071013	0.029	0.278	3.37E-08	20
IQGAP1.10	1.69E-12	-0.630295461	0.064	0.341	3.88E-08	20
BIRC3.16	2.23E-12	-1.259769947	0.029	0.275	5.12E-08	20
TYROBP.19	2.41E-12	-1.09821039	0.11	0.391	5.54E-08	20
ATM.10	2.43E-12	-0.998951317	0.017	0.252	5.57E-08	20
CD27.18	2.47E-12	-1.260163214	0.029	0.27	5.68E-08	20
AKNA.13	2.49E-12	-0.822200628	0.023	0.264	5.71E-08	20
ENSA.6	2.49E-12	-0.559078315	0.058	0.327	5.73E-08	20
CREM.13	2.68E-12	-0.946914025	0.035	0.283	6.16E-08	20
OCIAD2.15	3.53E-12	-0.972057259	0.023	0.264	8.12E-08	20
IL32.16	4.06E-12	-0.678932697	0.225	0.582	9.33E-08	20
RNF19A.16	4.73E-12	-0.791604999	0.035	0.28	1.09E-07	20
PSAP.16	4.92E-12	-0.57631047	0.15	0.472	1.13E-07	20
HLA-DRB5.16	5.75E-12	-1.183967022	0.098	0.377	1.32E-07	20
GIMAP7.17	6.00E-12	-0.576311216	0.04	0.288	1.38E-07	20

MGEA5.12	6.57E-12	-0.690126871	0.029	0.268	1.51E-07	20
TMCO1.5	6.58E-12	-0.591783211	0.04	0.287	1.51E-07	20
MT-ND4.12	7.95E-12	-0.548447684	0.491	0.879	1.83E-07	20
ZNF207.9	9.31E-12	-0.52707027	0.035	0.271	2.14E-07	20
DHX36.8	1.07E-11	-0.578530782	0.029	0.268	2.46E-07	20
ORMDL1.4	1.36E-11	-0.549687782	0.023	0.256	3.12E-07	20
HLA-DQA1.14	1.38E-11	-1.302073026	0.092	0.347	3.16E-07	20
TYMP.11	1.40E-11	-0.641365786	0.046	0.299	3.21E-07	20
GPX1.14	1.48E-11	-0.733511561	0.133	0.433	3.41E-07	20
BTN3A2.11	1.50E-11	-0.518045579	0.029	0.266	3.45E-07	20
HLA-A.15	1.57E-11	-0.563982763	0.555	0.898	3.62E-07	20
FYN.13	1.62E-11	-0.807378335	0.035	0.269	3.73E-07	20
PRDM1.13	1.78E-11	-0.723715155	0.046	0.295	4.09E-07	20
DPP7.3	1.85E-11	-0.529622888	0.035	0.272	4.24E-07	20
TRAF3IP3.18	2.33E-11	-0.691671951	0.029	0.259	5.35E-07	20
IDH2.7	2.47E-11	-0.502121214	0.029	0.263	5.67E-07	20
PLIN2.14	2.77E-11	-0.747199741	0.075	0.339	6.36E-07	20
EPC1.11	2.93E-11	-0.657551516	0.04	0.273	6.72E-07	20
CD96.19	3.05E-11	-0.976040759	0.029	0.252	7.01E-07	20
IL7R.17	5.24E-11	-1.451399445	0.069	0.306	1.20E-06	20
GLUL.13	5.46E-11	-0.555867375	0.064	0.309	1.25E-06	20
CTSB.15	5.56E-11	-0.630879376	0.058	0.305	1.28E-06	20
WNK1.11	6.11E-11	-0.612593066	0.029	0.252	1.40E-06	20
IL10RA.15	6.43E-11	-0.646706888	0.035	0.261	1.48E-06	20
RAB2A.7	6.63E-11	-0.523615118	0.04	0.272	1.52E-06	20
C9orf142.14	7.00E-11	-0.571760134	0.035	0.264	1.61E-06	20
PHLDA1.2	8.06E-11	2.153877174	0.289	0.146	1.85E-06	20
FCER1G.17	8.09E-11	-1.060546345	0.064	0.306	1.86E-06	20
CD7.16	8.15E-11	-0.760300454	0.064	0.303	1.87E-06	20
CMC1.16	9.21E-11	-1.040070043	0.052	0.288	2.12E-06	20
C10orf54.12	1.15E-10	-0.511830012	0.052	0.284	2.65E-06	20
BIN2.14	1.54E-10	-0.661224464	0.035	0.252	3.53E-06	20
ODF2L.11	4.45E-10	-0.737161723	0.046	0.262	1.02E-05	20
RPL30.9	5.99E-10	-0.527245654	0.532	0.902	1.38E-05	20
RGCC.17	1.84E-09	-0.750326493	0.081	0.31	4.23E-05	20
NPC2.14	3.45E-09	-0.533533428	0.133	0.382	7.92E-05	20
ACTB.15	1.24E-08	0.628059969	0.827	0.93	0.000284576	20
CUTA.7	1.27E-08	0.551755016	0.145	0.419	0.000291581	20
COX8A.7	1.42E-08	0.511485629	0.191	0.503	0.000327075	20
SOD2.15	1.55E-08	-0.597719405	0.081	0.296	0.000357114	20
TRMT112.7	3.37E-08	0.601372096	0.15	0.425	0.000773563	20
LYZ.16	5.61E-08	-1.300262415	0.139	0.354	0.001289079	20
VPS28.3	8.72E-08	0.531717725	0.116	0.349	0.00200324	20
APOC1.14	1.26E-07	-1.07392094	0.092	0.286	0.002899522	20
SEC61B.4	1.29E-07	0.579244254	0.168	0.431	0.002969481	20
RHOA.15	1.37E-07	0.520590746	0.214	0.512	0.003154613	20
SEC11A.8	1.77E-07	0.545082891	0.116	0.342	0.004074846	20
REEP5.4	1.79E-07	0.664359396	0.092	0.305	0.004106021	20
ATP5J2.7	1.97E-07	0.671030526	0.162	0.435	0.004520454	20
APOE.12	2.02E-07	-1.109287206	0.197	0.441	0.004632221	20
NUCKS1.8	3.06E-07	0.629331313	0.15	0.401	0.007022419	20
AIF1.17	4.04E-07	-0.647336746	0.081	0.261	0.009276214	20
COX5A.7	9.11E-07	0.517378913	0.116	0.324	0.020930758	20
CAPZA1.5	9.20E-07	0.524423474	0.098	0.3	0.021134296	20
NEDD8.7	1.07E-06	0.542019991	0.179	0.434	0.024699709	20
NOP10.8	1.13E-06	0.564429302	0.127	0.34	0.025968654	20
FKBP8.3	1.15E-06	0.514238239	0.121	0.335	0.02638283	20
CST3.19	1.16E-06	0.902446268	0.491	0.384	0.026678108	20

NDUFA13.7	1.38E-06	0.657072776	0.191	0.454	0.031712634	20
KTN1.11	1.44E-06	0.602057568	0.15	0.386	0.033085959	20
C14orf166.8	1.82E-06	0.639750882	0.121	0.335	0.041897076	20
COX6C.7	1.92E-06	0.570343527	0.254	0.571	0.044056334	20
ATP5G3.6	1.96E-06	0.538290579	0.168	0.41	0.045074833	20
ACKR1	0	3.714431629	0.91	0.009	0	21
COL8A1.1	0	2.795828017	0.854	0.031	0	21
RAMP3.1	0	2.760583319	0.944	0.045	0	21
CPE.2	0	2.723408518	0.876	0.035	0	21
MPZL2.1	0	2.605797947	0.888	0.031	0	21
SLCO2A1.1	0	2.494282448	0.809	0.021	0	21
FBLN2	0	2.419278091	0.629	0.003	0	21
POSTN	0	2.319432713	0.326	0.003	0	21
PTPRB.1	0	2.272412694	0.843	0.032	0	21
PALMD.1	0	2.233723167	0.854	0.026	0	21
NTS	0	2.18271241	0.472	0.003	0	21
COL3A1.1	0	2.108844902	0.674	0.017	0	21
TFF3	0	1.976063934	0.719	0.007	0	21
CCL14	0	1.844051764	0.607	0.007	0	21
ECSCR.1	0	1.768647178	0.933	0.04	0	21
IL33	0	1.723736286	0.64	0.004	0	21
HSPA2	0	1.670151612	0.584	0.017	0	21
NR2F2.1	0	1.656725547	0.708	0.024	0	21
MMRN1	0	1.601066105	0.416	0.006	0	21
MMRN2.1	0	1.588604814	0.73	0.027	0	21
PPIC.2	0	1.580373123	0.764	0.027	0	21
MALL	0	1.569678476	0.64	0.014	0	21
MEIS2.1	0	1.562794827	0.539	0.014	0	21
ADAMTS18	0	1.233178334	0.371	0	0	21
TPD52L1	0	1.207469099	0.506	0.007	0	21
RSPO3	0	1.196475408	0.427	0.001	0	21
ELN	0	1.193773627	0.427	0.002	0	21
SELP	0	1.193360519	0.427	0.002	0	21
NRN1	0	1.165442897	0.573	0.011	0	21
SRPX	0	1.146135781	0.449	0.002	0	21
CPXM2	0	1.079087894	0.461	0.003	0	21
TLL1	0	1.060349474	0.438	0.003	0	21
TEK	0	1.053015307	0.461	0.008	0	21
VEGFC	0	0.981533807	0.461	0.005	0	21
RASSF9	0	0.969971583	0.393	0.002	0	21
GPM6A	0	0.949789215	0.416	0.004	0	21
PTGIS	0	0.848546555	0.315	0.002	0	21
DOC2B	0	0.808439147	0.416	0.003	0	21
RAB3C	0	0.806810672	0.337	0.001	0	21
MMP28	0	0.780007336	0.382	0.004	0	21
LHX6	0	0.616984486	0.258	0.001	0	21
THSD7A.1	2.52E-307	1.402737705	0.607	0.018	5.80E-303	21
ITGB4	3.86E-306	0.731630581	0.382	0.007	8.87E-302	21
CYYR1.1	4.39E-297	1.569402471	0.685	0.025	1.01E-292	21
PCAT19.1	3.90E-290	1.932215475	0.843	0.039	8.96E-286	21
TM4SF1.1	4.93E-289	3.038850923	0.966	0.056	1.13E-284	21
EPHB4	1.20E-287	0.869071933	0.427	0.009	2.75E-283	21
TSPAN7.1	5.77E-284	1.646243794	0.719	0.029	1.33E-279	21
CNTNAP3B	1.54E-279	1.309703187	0.472	0.012	3.54E-275	21
BMP4	7.91E-278	0.77394566	0.258	0.003	1.82E-273	21
PCDH17.1	3.27E-276	1.424723461	0.494	0.013	7.51E-272	21
ERG.1	2.89E-275	1.049725112	0.551	0.017	6.65E-271	21
ZNF521	2.12E-272	0.740392481	0.36	0.007	4.86E-268	21

CYP1B1.1	3.74E-272	1.437296347	0.461	0.012	8.59E-268	21
FAM167B.1	4.25E-266	1.859314017	0.708	0.03	9.77E-262	21
ADGRG6	7.93E-266	1.571245719	0.528	0.017	1.82E-261	21
TMTC1.1	6.07E-263	1.593111673	0.596	0.021	1.40E-258	21
CXorf36.1	1.41E-253	1.594261574	0.663	0.027	3.24E-249	21
FAM171A1	5.57E-250	0.51923403	0.27	0.004	1.28E-245	21
CLEC14A.2	3.34E-248	2.096830897	0.899	0.052	7.68E-244	21
HSPG2.1	5.54E-247	2.339077036	0.921	0.058	1.27E-242	21
PGM5	7.30E-243	0.987102168	0.416	0.011	1.68E-238	21
SNCG.1	1.27E-242	1.658736711	0.719	0.035	2.92E-238	21
CALCRL.1	9.19E-241	2.002333919	0.787	0.042	2.11E-236	21
NUAK1.1	7.85E-240	1.362915868	0.551	0.02	1.81E-235	21
RAMP2.2	6.04E-239	2.636766379	0.933	0.061	1.39E-234	21
FAM107A.1	1.23E-237	1.310944328	0.551	0.02	2.82E-233	21
MTUS1.2	5.32E-237	2.25582534	0.865	0.055	1.22E-232	21
SYT15	7.25E-237	0.705342278	0.348	0.007	1.67E-232	21
HEG1.1	3.87E-232	2.024384505	0.708	0.036	8.88E-228	21
PLSCR4	2.28E-228	0.968776004	0.461	0.014	5.24E-224	21
WWTR1.3	1.17E-226	2.296887406	0.831	0.052	2.68E-222	21
PIR	1.06E-223	0.96683776	0.371	0.009	2.43E-219	21
PROCR	5.53E-223	1.61430575	0.697	0.036	1.27E-218	21
FGD5.1	1.33E-220	0.759724631	0.427	0.013	3.06E-216	21
VWF.1	6.89E-219	4.094415146	0.978	0.083	1.58E-214	21
LIMS2.1	4.44E-218	1.119725984	0.539	0.021	1.02E-213	21
PLA1A	1.26E-216	0.901312675	0.36	0.009	2.90E-212	21
CDC42EP5	6.07E-216	0.619054619	0.337	0.008	1.39E-211	21
JAM2	2.10E-213	1.197073041	0.517	0.02	4.83E-209	21
SEMA6A	4.95E-213	1.027368845	0.427	0.013	1.14E-208	21
ADGRL4.2	2.87E-212	1.777578113	0.831	0.051	6.60E-208	21
LDB2.1	1.47E-211	1.696235612	0.775	0.046	3.38E-207	21
PALM	2.55E-210	0.788336862	0.326	0.007	5.85E-206	21
PLAT.1	4.70E-207	1.683150799	0.517	0.021	1.08E-202	21
NPDC1.1	3.46E-205	2.091117764	0.831	0.057	7.94E-201	21
RNASE1.2	4.83E-205	3.053899288	1	0.087	1.11E-200	21
SDPR.2	5.68E-203	2.658064298	0.944	0.075	1.31E-198	21
HYAL1	1.17E-201	0.743175125	0.371	0.01	2.69E-197	21
EGFL7.1	8.95E-200	1.86162427	0.775	0.049	2.06E-195	21
HYAL2.1	1.89E-199	2.423949108	0.876	0.066	4.35E-195	21
LRP5	1.09E-197	0.626360629	0.303	0.007	2.51E-193	21
NOSTRIN.1	3.52E-195	1.635761327	0.618	0.032	8.09E-191	21
PLCB4.1	6.06E-195	0.924021254	0.461	0.017	1.39E-190	21
EMCN.2	1.63E-194	1.407502037	0.64	0.034	3.74E-190	21
HSPA12B	6.26E-192	0.625428818	0.315	0.008	1.44E-187	21
EMP1.1	7.67E-192	1.98668136	0.685	0.04	1.76E-187	21
TIE1.1	2.72E-191	1.093654591	0.528	0.023	6.26E-187	21
RHOJ	5.96E-191	0.833236711	0.371	0.011	1.37E-186	21
CTGF.2	1.62E-190	3.828641996	0.697	0.043	3.72E-186	21
FBN1	3.70E-189	0.834001175	0.393	0.013	8.51E-185	21
PTRF.3	5.89E-185	2.061822621	0.91	0.076	1.35E-180	21
MEDAG	1.37E-184	0.686727582	0.27	0.006	3.15E-180	21
LRRC1	6.07E-184	0.780781738	0.371	0.012	1.40E-179	21
LIFR.1	1.19E-182	1.337357436	0.674	0.04	2.73E-178	21
ZNF385D	1.87E-181	0.832546161	0.393	0.013	4.29E-177	21
DNASE1L3	1.98E-179	1.298799327	0.326	0.009	4.55E-175	21
EFEMP1	5.01E-179	2.776660745	0.449	0.018	1.15E-174	21
SEPN1	1.84E-177	1.081885318	0.551	0.028	4.24E-173	21
ROBO4.1	1.35E-176	1.066913873	0.461	0.019	3.11E-172	21
SH3BGRL2	5.23E-175	0.820302445	0.337	0.01	1.20E-170	21

MID1	5.50E-174	0.877616005	0.36	0.012	1.26E-169	21
PODXL.1	9.81E-173	1.188618067	0.596	0.032	2.25E-168	21
LMO2.1	2.88E-172	1.202015014	0.618	0.037	6.62E-168	21
LMCD1.1	8.81E-171	1.32240496	0.629	0.037	2.02E-166	21
LIMCH1.1	4.66E-168	1.028327046	0.539	0.028	1.07E-163	21
FRY.1	1.53E-166	0.876216198	0.483	0.022	3.52E-162	21
PLPP3.1	1.58E-166	1.870405245	0.764	0.057	3.62E-162	21
FLNB	1.83E-165	1.356640538	0.629	0.04	4.21E-161	21
PCGF2	6.29E-164	0.920711495	0.449	0.02	1.45E-159	21
CADPS2	9.29E-163	0.630051492	0.292	0.008	2.13E-158	21
DOCK9.1	1.83E-160	1.320069578	0.652	0.042	4.21E-156	21
FGFR1.1	8.05E-160	1.407850921	0.596	0.036	1.85E-155	21
CDH5.1	9.10E-160	1.242942206	0.528	0.028	2.09E-155	21
PBX1	1.42E-159	0.758859432	0.393	0.015	3.27E-155	21
EMP2.2	3.17E-159	1.242939248	0.607	0.038	7.28E-155	21
MANSC1	1.14E-158	0.760266931	0.461	0.021	2.62E-154	21
ADCY4	1.04E-157	0.892297351	0.393	0.015	2.38E-153	21
IL1R1	1.38E-156	0.919640362	0.404	0.017	3.16E-152	21
MIR99AHG	7.40E-156	0.541292545	0.258	0.006	1.70E-151	21
TSHZ2	1.80E-155	1.301800969	0.551	0.031	4.14E-151	21
PTPRG.2	7.99E-155	1.121335783	0.539	0.03	1.84E-150	21
RP11-382A20.3	3.18E-153	1.072311385	0.551	0.032	7.30E-149	21
CD109	4.42E-153	0.913432807	0.427	0.019	1.02E-148	21
AQP1.1	3.75E-152	1.940788848	0.719	0.056	8.61E-148	21
TGM2.3	3.82E-152	1.509487326	0.73	0.058	8.78E-148	21
CD34.1	7.54E-152	1.096784991	0.607	0.038	1.73E-147	21
RAI14.1	9.43E-151	0.860128315	0.438	0.02	2.17E-146	21
FAM198B.1	1.25E-149	1.357496262	0.551	0.034	2.87E-145	21
ACVRL1.1	2.70E-149	0.905723233	0.483	0.025	6.20E-145	21
LAMA4.1	4.20E-149	1.253111812	0.539	0.031	9.66E-145	21
ADAM15	5.82E-148	1.312714624	0.573	0.037	1.34E-143	21
MARCKSL1.2	8.68E-148	1.909660626	0.843	0.082	1.99E-143	21
FAM84B	9.45E-145	0.95303211	0.393	0.017	2.17E-140	21
EPB41L4A.1	1.10E-144	0.934961568	0.483	0.026	2.53E-140	21
ENG.3	4.02E-143	2.316042535	0.91	0.102	9.25E-139	21
TFPI.4	4.33E-143	1.912734278	0.764	0.068	9.95E-139	21
NFIB.4	4.38E-143	1.570442382	0.775	0.068	1.01E-138	21
TCN2.1	2.75E-142	1.516031123	0.663	0.052	6.32E-138	21
CRIP2.4	7.27E-142	1.788221185	0.91	0.097	1.67E-137	21
MAP2.1	2.21E-141	0.698391421	0.292	0.009	5.08E-137	21
MYCT1.1	1.68E-140	0.924064112	0.404	0.018	3.86E-136	21
RUNX1T1	5.25E-140	0.746956638	0.315	0.011	1.21E-135	21
CTNNAL1	8.60E-138	1.274849759	0.528	0.033	1.98E-133	21
CFH.1	9.13E-138	0.955908383	0.449	0.024	2.10E-133	21
MYO5C	1.16E-137	0.628759955	0.281	0.009	2.67E-133	21
TGFBR3	1.67E-137	1.166100997	0.539	0.034	3.83E-133	21
ITGA6.1	3.18E-137	1.644083189	0.753	0.068	7.30E-133	21
SASH1.2	5.40E-136	0.954926156	0.539	0.034	1.24E-131	21
FN1.3	1.95E-135	2.820432885	0.933	0.119	4.49E-131	21
ACKR3.1	4.13E-135	1.406837144	0.404	0.02	9.50E-131	21
MECOM.1	2.43E-134	0.647862721	0.371	0.016	5.58E-130	21
SLC9A3R2.2	2.19E-133	1.081177014	0.73	0.061	5.04E-129	21
HRCT1.1	4.78E-133	0.862163393	0.416	0.021	1.10E-128	21
SHE.1	8.01E-133	0.589363004	0.303	0.011	1.84E-128	21
ADAMTS1.1	8.71E-133	1.668302771	0.461	0.026	2.00E-128	21
HAPLN3	3.31E-132	1.011996536	0.472	0.027	7.60E-128	21
FZD4.1	1.77E-129	0.898717454	0.427	0.022	4.06E-125	21
IGFBP4.4	2.76E-129	2.323024098	0.91	0.113	6.34E-125	21

GAS6.2	7.36E-129	1.44747285	0.742	0.071	1.69E-124	21
SEMA3F.1	1.24E-128	0.777361191	0.326	0.013	2.85E-124	21
CRIM1.3	3.39E-128	2.003115161	0.764	0.076	7.79E-124	21
C8orf4	1.98E-127	1.160606178	0.382	0.018	4.54E-123	21
TMEM255B.1	3.55E-127	0.859168276	0.494	0.031	8.16E-123	21
PLAC9.2	1.48E-126	0.986000716	0.483	0.029	3.40E-122	21
RFK.1	7.67E-126	1.611318444	0.719	0.069	1.76E-121	21
DIXDC1.2	1.90E-125	0.780512502	0.449	0.026	4.36E-121	21
AHNAK2.1	3.48E-125	0.560198072	0.303	0.012	8.01E-121	21
SOCS2.1	1.12E-124	1.001858238	0.472	0.029	2.58E-120	21
EPAS1.4	1.38E-122	1.865730787	0.91	0.11	3.17E-118	21
IFI27.5	5.64E-122	2.705313483	1	0.149	1.30E-117	21
S100A16.3	3.35E-120	1.341889041	0.764	0.077	7.70E-116	21
ESAM.2	4.59E-120	1.09828808	0.685	0.06	1.06E-115	21
TINAGL1.3	5.11E-120	1.246625351	0.618	0.051	1.17E-115	21
S1PR1.1	9.75E-120	1.068508478	0.584	0.045	2.24E-115	21
PDLIM4	1.52E-118	0.50677541	0.258	0.009	3.50E-114	21
SESN3.1	7.73E-118	1.839078727	0.64	0.06	1.78E-113	21
CFI.1	2.05E-117	1.023773173	0.483	0.032	4.71E-113	21
NPR1	2.24E-115	0.534238679	0.281	0.011	5.15E-111	21
PTPN14.1	1.27E-114	0.837987545	0.483	0.033	2.92E-110	21
SPTBN1.2	5.21E-114	1.776058151	0.82	0.098	1.20E-109	21
TCF4.3	1.51E-113	1.919569684	0.865	0.114	3.48E-109	21
RAPGEF5.2	6.40E-113	0.96577902	0.472	0.031	1.47E-108	21
NID1.2	8.30E-111	0.696993297	0.449	0.029	1.91E-106	21
ST6GALNAC3	1.84E-110	0.638826181	0.303	0.013	4.23E-106	21
TJP1.2	4.25E-110	0.946938891	0.494	0.035	9.77E-106	21
DLC1.1	1.27E-109	1.061047912	0.371	0.02	2.92E-105	21
ATP8B1	3.01E-109	0.713011704	0.281	0.012	6.93E-105	21
FXYD6.1	2.20E-108	1.007122037	0.393	0.023	5.05E-104	21
LDLRAD3	3.14E-107	0.58805554	0.315	0.015	7.22E-103	21
ASS1.2	3.87E-106	0.815556192	0.393	0.023	8.89E-102	21
ITGA5.1	7.32E-106	1.067099078	0.663	0.066	1.68E-101	21
EDN1.1	7.34E-106	1.119632022	0.404	0.025	1.69E-101	21
BMPR2.2	1.16E-104	1.571481533	0.663	0.071	2.67E-100	21
NFIA.4	2.01E-104	1.480924907	0.708	0.08	4.61E-100	21
SPARCL1.5	2.24E-103	2.045515264	0.989	0.158	5.16E-99	21
APP.7	4.33E-103	2.623263335	0.966	0.173	9.96E-99	21
MPDZ.1	6.20E-103	0.605920307	0.326	0.016	1.42E-98	21
PREX2.1	1.90E-102	0.73774059	0.371	0.021	4.37E-98	21
PRSS23.3	2.56E-102	1.399369062	0.854	0.11	5.88E-98	21
AKAP12.3	9.13E-102	1.610738827	0.517	0.043	2.10E-97	21
HOXB7	9.14E-102	0.811418163	0.382	0.023	2.10E-97	21
CNN3.6	1.70E-99	1.09052168	0.831	0.103	3.91E-95	21
TEAD1	2.00E-99	0.723306586	0.315	0.016	4.59E-95	21
CDC42BPA.2	2.32E-99	0.764283266	0.494	0.039	5.34E-95	21
ECE1.2	4.59E-99	1.025439438	0.517	0.043	1.06E-94	21
CLDN5.1	5.27E-99	1.380990307	0.494	0.039	1.21E-94	21
TANC1.1	6.65E-98	0.684997457	0.36	0.021	1.53E-93	21
TIMP3.3	1.51E-97	1.556405198	0.753	0.09	3.47E-93	21
CEP112	5.78E-97	0.637441061	0.303	0.015	1.33E-92	21
GOLM1.1	7.51E-97	0.737491581	0.393	0.026	1.73E-92	21
APBB2.1	8.35E-96	0.697554349	0.326	0.018	1.92E-91	21
SULF2	4.99E-95	0.948347614	0.539	0.049	1.15E-90	21
FZD6	6.09E-95	0.564138894	0.315	0.017	1.40E-90	21
PDLIM1.5	2.54E-94	1.697294642	0.82	0.116	5.83E-90	21
RPS6KA2.1	6.37E-94	0.824926653	0.438	0.033	1.46E-89	21
PTPRM.2	6.88E-94	0.918091244	0.494	0.042	1.58E-89	21

CLEC1A	1.05E-93	0.621332391	0.292	0.015	2.42E-89	21
LHFP.2	6.34E-93	1.067030053	0.528	0.048	1.46E-88	21
GNG11.4	6.81E-93	1.617064095	0.899	0.139	1.57E-88	21
PECAM1.5	1.92E-92	2.372267803	0.966	0.196	4.41E-88	21
EPB41L3.2	5.80E-91	1.206216165	0.562	0.059	1.33E-86	21
TUBB6.1	8.18E-91	0.707307094	0.427	0.032	1.88E-86	21
TSPAN9	9.99E-91	0.601604337	0.337	0.02	2.30E-86	21
ACTN1.3	1.94E-89	1.322108006	0.652	0.078	4.45E-85	21
CLU.6	2.70E-89	2.782170743	0.955	0.193	6.20E-85	21
NCKAP1.2	6.57E-89	0.785650313	0.449	0.036	1.51E-84	21
FSTL1.2	2.34E-88	0.707033036	0.528	0.048	5.39E-84	21
HTRA1.4	2.89E-87	1.54241447	0.742	0.105	6.65E-83	21
SPRY1.2	4.27E-86	1.793042822	0.764	0.107	9.81E-82	21
ABLIM1.2	1.06E-85	1.184104049	0.607	0.067	2.43E-81	21
LRRC32.2	1.50E-85	0.570330621	0.315	0.018	3.44E-81	21
BCAM.2	5.35E-85	0.805888116	0.596	0.063	1.23E-80	21
AFAP1	1.80E-84	0.524079119	0.337	0.021	4.14E-80	21
CD59.9	2.74E-84	2.27127222	1	0.228	6.29E-80	21
WBP5.5	3.18E-84	1.017904424	0.663	0.081	7.32E-80	21
RPGR.1	7.66E-84	1.154476264	0.573	0.061	1.76E-79	21
CDA.2	1.03E-83	0.709414355	0.427	0.034	2.37E-79	21
TNS2.2	2.08E-83	0.702617788	0.461	0.04	4.77E-79	21
EHD2.3	5.19E-83	0.783285556	0.494	0.047	1.19E-78	21
CAV1.5	7.75E-83	1.449452246	0.865	0.133	1.78E-78	21
COX7A1.2	1.05E-82	0.935421435	0.596	0.065	2.41E-78	21
ITGA10	3.00E-81	0.5934708	0.281	0.016	6.90E-77	21
PXDC1	5.68E-81	0.596432459	0.315	0.02	1.31E-76	21
CALD1.5	6.42E-81	0.985644546	0.899	0.144	1.47E-76	21
CD93.3	8.88E-81	1.157723587	0.64	0.082	2.04E-76	21
ABCG2.1	1.22E-80	0.656822435	0.292	0.017	2.82E-76	21
SH3BP5.1	3.63E-80	1.448652849	0.809	0.132	8.34E-76	21
CRNDE.1	6.33E-80	0.533418258	0.371	0.027	1.46E-75	21
ADIRF.14	1.17E-79	2.607512763	0.989	0.237	2.69E-75	21
PLEKHG1.1	5.37E-79	0.712935755	0.303	0.019	1.23E-74	21
MYOF.3	5.60E-79	0.805229275	0.494	0.05	1.29E-74	21
IPO11.1	7.03E-79	0.768399833	0.427	0.037	1.62E-74	21
MARCKS.3	8.93E-79	1.662464615	0.843	0.147	2.05E-74	21
FAM213A.3	1.15E-78	1.368737666	0.73	0.109	2.65E-74	21
RASA4	2.81E-78	0.934230732	0.449	0.042	6.45E-74	21
ARHGAP29.3	3.45E-78	1.075913347	0.584	0.066	7.93E-74	21
ELK3.1	5.41E-78	1.405230458	0.674	0.093	1.24E-73	21
PLXNA2.1	9.23E-78	0.562845816	0.292	0.018	2.12E-73	21
YBX3.5	2.38E-77	2.007487367	0.888	0.18	5.46E-73	21
FERMT2.2	1.13E-76	0.692724224	0.404	0.034	2.59E-72	21
PRCP.2	1.24E-76	1.3550858	0.742	0.119	2.84E-72	21
SULF1.1	2.25E-76	0.894615572	0.315	0.021	5.17E-72	21
CHSY1	2.66E-76	0.741498266	0.438	0.04	6.11E-72	21
MSRB3.1	3.53E-76	0.54006788	0.292	0.018	8.10E-72	21
COL4A1.2	1.12E-75	0.927386105	0.573	0.066	2.57E-71	21
BACE2.1	1.06E-74	0.598910208	0.326	0.023	2.44E-70	21
WWP1	1.88E-74	1.029945243	0.562	0.066	4.32E-70	21
LAMB2.2	3.53E-74	0.924442847	0.438	0.041	8.12E-70	21
RBMS2.1	7.78E-74	0.765422087	0.404	0.035	1.79E-69	21
FKBP9.1	8.99E-74	0.599528436	0.36	0.028	2.07E-69	21
CNRIP1	1.42E-73	0.55098978	0.315	0.022	3.25E-69	21
SNTB2.1	3.19E-73	1.105739947	0.596	0.074	7.33E-69	21
CAV2.4	1.13E-72	0.939584842	0.596	0.074	2.61E-68	21
ST6GAL1.1	1.31E-72	1.234861506	0.596	0.079	3.02E-68	21

HS3ST1	1.92E-72	1.129683968	0.281	0.018	4.41E-68	21
NREP.1	3.01E-72	0.650621021	0.348	0.027	6.92E-68	21
LIMA1.3	3.17E-72	1.122088216	0.618	0.082	7.28E-68	21
TMEM150C.1	5.33E-72	0.5938456	0.326	0.023	1.22E-67	21
CTTN.3	7.15E-72	0.6598108	0.416	0.038	1.64E-67	21
KIAA0355.1	1.99E-71	1.092359515	0.652	0.092	4.57E-67	21
ARL4A.2	3.20E-71	1.858206248	0.854	0.172	7.34E-67	21
S100A13.5	4.06E-71	1.222303938	0.775	0.129	9.32E-67	21
NGFRAP1.5	4.87E-71	1.092196589	0.719	0.108	1.12E-66	21
PHACTR2.3	2.51E-70	1.277359436	0.685	0.105	5.78E-66	21
RBP1.1	2.79E-70	0.545937316	0.27	0.017	6.42E-66	21
PMP22.2	3.69E-70	0.950578702	0.517	0.059	8.48E-66	21
MRAS	4.25E-70	0.57941907	0.27	0.017	9.77E-66	21
MET.2	4.40E-70	0.658993424	0.326	0.024	1.01E-65	21
PKP4	7.39E-70	0.548023294	0.326	0.024	1.70E-65	21
C16orf45.1	1.20E-69	0.617748734	0.281	0.018	2.75E-65	21
LAMC1.2	3.08E-69	0.691013649	0.416	0.039	7.07E-65	21
MDK.1	5.51E-69	0.704329436	0.315	0.023	1.27E-64	21
INPP1.1	1.63E-68	0.870433773	0.494	0.056	3.75E-64	21
RAPH1.1	1.73E-68	0.680622886	0.382	0.034	3.98E-64	21
MKL2	5.45E-68	0.717658312	0.348	0.028	1.25E-63	21
PDLIM3.1	5.99E-68	0.581262168	0.27	0.017	1.38E-63	21
CYR61.3	1.47E-67	2.299334832	0.427	0.044	3.38E-63	21
PLPP1.2	3.26E-67	0.731948817	0.663	0.094	7.49E-63	21
PLS3.2	5.67E-67	0.510813254	0.382	0.034	1.30E-62	21
PVRL2.1	1.08E-66	0.806096906	0.494	0.058	2.47E-62	21
THBD.1	9.48E-66	0.975821151	0.393	0.038	2.18E-61	21
SPARC.6	1.21E-65	0.757532814	0.91	0.178	2.79E-61	21
TACC1.4	1.95E-65	1.607117276	0.921	0.225	4.49E-61	21
PDLIM5.3	2.08E-65	1.164198539	0.573	0.08	4.78E-61	21
KLF9.2	3.00E-65	1.454873938	0.809	0.157	6.90E-61	21
CCDC50.5	1.25E-64	1.195282575	0.697	0.119	2.86E-60	21
CDC42BPB.1	3.68E-64	0.794418174	0.337	0.029	8.46E-60	21
AGRN	4.82E-64	0.608324325	0.326	0.027	1.11E-59	21
FKBP1A.13	5.45E-64	2.797366332	0.989	0.409	1.25E-59	21
C10orf128	8.56E-64	0.646757645	0.382	0.036	1.97E-59	21
PIK3C2A.1	1.48E-63	1.107742924	0.596	0.088	3.40E-59	21
HOXD8.1	4.61E-63	0.526846581	0.303	0.023	1.06E-58	21
ALDH1A1.3	4.99E-63	0.979840308	0.607	0.087	1.15E-58	21
IL6ST.2	2.26E-62	1.628701926	0.82	0.174	5.19E-58	21
ID1.2	2.62E-62	1.05945141	0.528	0.067	6.03E-58	21
ZFYVE21.1	4.75E-62	1.000247069	0.539	0.074	1.09E-57	21
STAB1.1	9.30E-62	0.733550039	0.404	0.043	2.14E-57	21
PLXND1.2	1.04E-61	0.768629201	0.494	0.061	2.38E-57	21
ID3.4	3.41E-61	1.140640261	0.753	0.136	7.83E-57	21
SHC1	4.91E-61	0.69421141	0.449	0.051	1.13E-56	21
IFITM3.18	6.73E-60	2.301384114	1	0.374	1.55E-55	21
AK1.1	8.67E-60	0.762755828	0.416	0.045	1.99E-55	21
HHEX.1	1.08E-59	0.760587445	0.393	0.042	2.48E-55	21
C4orf32.1	1.55E-59	0.506503211	0.371	0.036	3.56E-55	21
NFE2L1.4	1.14E-58	0.901734009	0.551	0.08	2.61E-54	21
GFOD2.1	1.82E-58	0.66145544	0.36	0.035	4.19E-54	21
SYNPO.1	1.98E-58	0.528440594	0.303	0.025	4.54E-54	21
TRIOBP.1	4.57E-58	0.93645508	0.483	0.063	1.05E-53	21
PLVAP.2	4.94E-58	0.656425944	0.618	0.092	1.14E-53	21
GJA1.1	5.78E-58	0.554802319	0.348	0.032	1.33E-53	21
TNFAIP1.1	3.87E-57	0.579231252	0.36	0.035	8.90E-53	21
PLK2.1	6.65E-57	0.870360728	0.461	0.057	1.53E-52	21

PTMS.7	6.92E-57	1.537094442	0.899	0.238	1.59E-52	21
HES1.4	1.00E-56	1.738190619	0.551	0.082	2.30E-52	21
COL4A2.2	1.17E-56	0.585076325	0.483	0.06	2.69E-52	21
IGFBP7.17	1.73E-56	2.179561173	1	0.375	3.98E-52	21
GALNT15.1	3.98E-56	0.532913008	0.281	0.022	9.14E-52	21
NEDD9.4	8.71E-56	1.149155494	0.798	0.165	2.00E-51	21
ASAP1.2	1.83E-55	0.919422949	0.596	0.097	4.21E-51	21
TNS1.3	5.28E-55	0.777776025	0.404	0.046	1.21E-50	21
TGFBR2.2	7.76E-55	1.678318461	0.809	0.198	1.78E-50	21
DUSP23.3	1.31E-54	1.203283154	0.775	0.17	3.00E-50	21
CLIC2.1	1.33E-54	0.551421939	0.348	0.035	3.05E-50	21
SSBP3	1.52E-54	0.560224212	0.315	0.029	3.49E-50	21
ZBTB8A	2.09E-54	0.539521851	0.281	0.023	4.80E-50	21
BGN.2	5.91E-54	1.063987315	0.472	0.063	1.36E-49	21
MYO1C.3	2.73E-53	0.75448535	0.438	0.056	6.28E-49	21
RFX3	5.40E-53	0.52260672	0.315	0.03	1.24E-48	21
LMNA.4	6.03E-53	1.64043295	0.787	0.171	1.39E-48	21
RALGAPA2	1.38E-52	0.743525949	0.382	0.043	3.17E-48	21
PTK2.2	1.80E-52	0.609510148	0.371	0.041	4.13E-48	21
RAB13.7	3.06E-52	1.041612153	0.685	0.133	7.04E-48	21
MGP.5	3.56E-52	2.001811571	0.73	0.146	8.18E-48	21
PTTG1IP.4	1.58E-51	1.098692835	0.775	0.173	3.62E-47	21
ITPR2.1	8.07E-51	0.960904821	0.584	0.1	1.85E-46	21
TM4SF18.1	9.25E-51	0.592750256	0.337	0.035	2.13E-46	21
BTBD3.1	1.90E-50	0.558669799	0.292	0.027	4.37E-46	21
DAAM1.1	1.92E-50	0.781890078	0.427	0.055	4.42E-46	21
TCEAL4.6	8.20E-48	0.865750187	0.719	0.155	1.89E-43	21
EPHX1.2	8.22E-48	0.705213632	0.348	0.04	1.89E-43	21
EXOC6.1	9.88E-48	0.903582234	0.461	0.067	2.27E-43	21
ITPRIP.1	1.01E-47	0.745151401	0.449	0.063	2.32E-43	21
PIEZO1	3.97E-47	0.648548697	0.393	0.05	9.11E-43	21
SERPINB6.3	5.18E-47	1.022372412	0.742	0.171	1.19E-42	21
GSE1	6.29E-47	0.65675502	0.371	0.045	1.45E-42	21
CTNND1.3	6.65E-47	0.666233107	0.517	0.085	1.53E-42	21
STOM.2	9.62E-47	1.210239467	0.787	0.198	2.21E-42	21
ASRGL1	1.79E-46	0.702232418	0.303	0.032	4.12E-42	21
RP11-553L6.5.4	2.02E-46	0.676854265	0.393	0.05	4.64E-42	21
NRP2.1	8.68E-46	0.566116453	0.315	0.034	1.99E-41	21
TSPAN3.3	1.41E-45	0.759596265	0.562	0.1	3.23E-41	21
TPM4.3	2.41E-45	1.406893023	0.787	0.21	5.54E-41	21
TSPAN4.4	3.41E-45	0.816893611	0.551	0.098	7.84E-41	21
MCAM.2	4.10E-45	0.730393699	0.393	0.05	9.42E-41	21
ACACB.1	1.84E-44	0.523645254	0.27	0.026	4.22E-40	21
ANXA2.14	4.32E-44	1.49709925	0.978	0.389	9.92E-40	21
MEF2C.4	5.07E-44	0.967272981	0.697	0.147	1.16E-39	21
S100A6.11	1.02E-43	1.940550419	1	0.765	2.35E-39	21
DKK3.2	1.19E-43	0.698386621	0.382	0.049	2.74E-39	21
PPM1F.1	1.72E-43	0.534756322	0.337	0.04	3.95E-39	21
SWAP70.3	2.76E-43	0.989637549	0.551	0.104	6.34E-39	21
DSTN.17	3.31E-43	1.164627425	0.933	0.303	7.60E-39	21
MLLT4.1	3.32E-43	0.509402701	0.292	0.031	7.64E-39	21
ARHGAP31	3.90E-43	0.501962666	0.258	0.025	8.96E-39	21
C1orf21	4.03E-43	0.707438762	0.416	0.059	9.26E-39	21
EVA1C.1	1.34E-42	0.532916239	0.315	0.036	3.07E-38	21
JAG1.2	1.83E-42	0.572129692	0.36	0.046	4.20E-38	21
CD151.6	1.83E-42	0.95847256	0.775	0.197	4.20E-38	21
TMEM47.3	2.04E-42	0.580179035	0.348	0.043	4.69E-38	21
IRF2BP2.4	3.71E-42	0.996291984	0.674	0.155	8.53E-38	21

GNAQ.4	4.22E-42	0.910588925	0.629	0.133	9.69E-38	21
SPTAN1.2	8.13E-42	0.95122828	0.584	0.113	1.87E-37	21
MGST2.4	8.19E-42	0.859470712	0.697	0.162	1.88E-37	21
SUN1.1	9.28E-42	0.787904697	0.393	0.056	2.13E-37	21
SSFA2	1.59E-41	0.718712824	0.438	0.07	3.66E-37	21
DOCK4.2	2.82E-41	0.80325613	0.438	0.07	6.48E-37	21
NDRG1.3	2.87E-41	0.921054176	0.652	0.14	6.59E-37	21
LRRC16A	5.61E-41	0.507804158	0.292	0.033	1.29E-36	21
LEPROT.5	9.75E-41	1.009652752	0.753	0.194	2.24E-36	21
PRKCDBP.5	1.23E-40	0.535762611	0.483	0.081	2.82E-36	21
SLC40A1.2	1.29E-40	0.631928749	0.461	0.076	2.97E-36	21
HSD17B12.3	2.03E-40	0.886101042	0.551	0.11	4.66E-36	21
CCDC85B.8	3.13E-40	1.221455257	0.82	0.25	7.18E-36	21
TMCC3.2	4.43E-40	0.77365285	0.371	0.053	1.02E-35	21
PINK1.1	7.80E-40	0.544363211	0.393	0.057	1.79E-35	21
TMEM30A.2	8.43E-40	0.880026527	0.64	0.142	1.94E-35	21
SLC44A2.1	1.08E-39	0.921505392	0.506	0.091	2.48E-35	21
ARHGEF12.3	1.48E-39	0.596782196	0.461	0.075	3.40E-35	21
MAPK3.1	2.54E-39	0.519751043	0.382	0.055	5.84E-35	21
GNG12.3	2.82E-39	0.5531686	0.258	0.027	6.48E-35	21
ASPH.5	4.04E-39	0.8260548	0.517	0.099	9.29E-35	21
AHR.2	4.05E-39	0.813799374	0.461	0.08	9.32E-35	21
SEC14L1.3	5.57E-39	1.161619358	0.708	0.185	1.28E-34	21
ATP11C	5.70E-39	0.604869907	0.303	0.037	1.31E-34	21
GALNT1.1	6.70E-39	0.719742964	0.483	0.086	1.54E-34	21
PARVB.1	7.77E-39	0.646470123	0.449	0.076	1.79E-34	21
SLC38A2.5	7.98E-39	1.155980053	0.764	0.205	1.83E-34	21
RCN1.4	8.55E-39	0.67274568	0.494	0.09	1.96E-34	21
HDAC7.1	1.23E-38	0.636803107	0.472	0.079	2.82E-34	21
DUSP6.2	3.09E-38	1.051692367	0.562	0.115	7.10E-34	21
TIMP1.18	3.12E-38	1.108055577	0.944	0.31	7.17E-34	21
ARL2.3	4.31E-38	0.896254382	0.629	0.146	9.91E-34	21
PSMB5.3	1.10E-37	0.686889993	0.652	0.151	2.53E-33	21
ARPC1A.4	1.27E-37	0.702421877	0.607	0.132	2.93E-33	21
CSGALNACT1.1	2.35E-37	0.529584005	0.36	0.051	5.39E-33	21
NOC3L	2.60E-37	0.706534504	0.382	0.058	5.97E-33	21
EBPL.1	2.73E-37	0.708436909	0.494	0.093	6.27E-33	21
MTRNR2L1	4.47E-37	0.590343821	0.27	0.031	1.03E-32	21
EHD4.1	4.52E-37	0.84595497	0.438	0.075	1.04E-32	21
WARS.2	5.77E-37	1.072626613	0.472	0.088	1.33E-32	21
PLEC.1	8.33E-37	0.734158522	0.449	0.08	1.91E-32	21
ABL2.3	1.02E-36	0.677564628	0.416	0.07	2.34E-32	21
FNIP2.4	1.41E-36	0.734999172	0.427	0.074	3.24E-32	21
ZMAT3.1	2.67E-36	0.960299343	0.416	0.07	6.13E-32	21
CLIC4.5	3.54E-36	0.509759074	0.483	0.09	8.14E-32	21
PXN.1	1.11E-35	0.677476206	0.438	0.076	2.55E-31	21
COL18A1.2	1.24E-35	0.57091923	0.416	0.069	2.85E-31	21
NCOA7.1	1.57E-35	0.886273827	0.584	0.13	3.61E-31	21
VGLL4.1	1.71E-35	0.548422662	0.438	0.075	3.93E-31	21
LTC4S.2	2.38E-35	0.804919198	0.438	0.079	5.47E-31	21
IFITM2.12	2.94E-35	1.244592829	0.966	0.472	6.76E-31	21
CD320.2	3.65E-35	0.725960281	0.506	0.096	8.38E-31	21
FCHO2.2	4.21E-35	0.623077143	0.404	0.068	9.68E-31	21
PERP.2	4.72E-35	0.720570681	0.416	0.069	1.09E-30	21
RAB11A.5	5.01E-35	1.141852808	0.753	0.225	1.15E-30	21
CHMP3.2	1.03E-34	0.84193019	0.685	0.177	2.36E-30	21
ZEB1.2	2.29E-34	0.681408882	0.472	0.087	5.27E-30	21
UGCG.1	3.96E-34	0.744179341	0.438	0.081	9.11E-30	21

C1orf54.4	4.10E-34	0.720234506	0.449	0.085	9.41E-30	21
NAA10.1	4.58E-34	0.826288149	0.708	0.188	1.05E-29	21
NFIC.4	4.61E-34	0.706957413	0.539	0.117	1.06E-29	21
FAM43A.1	5.34E-34	0.61025338	0.315	0.043	1.23E-29	21
GIMAP6.1	6.63E-34	0.674469187	0.506	0.1	1.52E-29	21
TRIB2.1	8.03E-34	0.509801658	0.315	0.043	1.85E-29	21
MKNK2	1.24E-33	0.693515544	0.438	0.081	2.84E-29	21
GSN.7	2.44E-33	0.674218671	0.787	0.221	5.61E-29	21
ATL3	3.12E-33	0.589577241	0.449	0.085	7.17E-29	21
SLK.1	6.59E-33	0.682722829	0.438	0.082	1.52E-28	21
GNAI2.15	8.58E-33	1.133746355	0.787	0.257	1.97E-28	21
AKT3.1	9.38E-33	0.546397019	0.371	0.06	2.15E-28	21
DPYSL2.4	1.55E-32	0.931812273	0.562	0.137	3.56E-28	21
ATP11A.2	4.82E-32	0.582256252	0.348	0.056	1.11E-27	21
FOXO1	9.35E-32	0.574513598	0.348	0.055	2.15E-27	21
RASAL2.3	9.66E-32	0.5976906	0.348	0.057	2.22E-27	21
UBE2H	1.18E-31	0.713606108	0.438	0.084	2.71E-27	21
NFAT5.3	1.44E-31	0.752986571	0.539	0.12	3.31E-27	21
TPST2	1.52E-31	0.795775833	0.472	0.097	3.50E-27	21
TNFSF10.5	1.94E-31	0.856547383	0.64	0.164	4.47E-27	21
KLF2.4	1.98E-31	1.312863097	0.708	0.206	4.55E-27	21
SERPINE1.2	2.37E-31	0.759091632	0.326	0.05	5.44E-27	21
VAT1.1	2.41E-31	0.605158198	0.337	0.054	5.54E-27	21
ADD1.3	2.46E-31	0.675897446	0.596	0.142	5.64E-27	21
HEBP1.1	3.21E-31	0.684263965	0.472	0.1	7.38E-27	21
GUK1.6	4.01E-31	1.236459555	0.944	0.504	9.21E-27	21
S100A10.12	4.62E-31	1.267456864	1	0.608	1.06E-26	21
SEPW1.15	5.08E-31	0.992053672	0.899	0.356	1.17E-26	21
TCEAL3.2	1.82E-30	0.548813516	0.393	0.072	4.17E-26	21
TIMP2.5	2.44E-30	0.774854204	0.404	0.079	5.61E-26	21
DNAJC10.1	2.78E-30	0.546557068	0.472	0.097	6.40E-26	21
HSPB1.18	5.26E-30	1.046448087	0.955	0.431	1.21E-25	21
PON2.2	5.92E-30	0.714920525	0.449	0.094	1.36E-25	21
IFI44L.2	6.18E-30	0.744883351	0.393	0.073	1.42E-25	21
METTL7A.4	1.24E-29	0.723167021	0.494	0.111	2.85E-25	21
CTTNBP2NL.3	2.45E-29	0.734076318	0.438	0.092	5.63E-25	21
DYNC1LI2.3	3.75E-29	0.63209532	0.517	0.117	8.62E-25	21
RDX.4	4.45E-29	0.672170713	0.629	0.169	1.02E-24	21
MAN1A1.1	5.30E-29	0.785541264	0.337	0.059	1.22E-24	21
SELM.6	6.97E-29	0.773573777	0.652	0.181	1.60E-24	21
DST.4	7.24E-29	0.581027941	0.404	0.08	1.66E-24	21
VIM.16	1.26E-28	1.051929133	1	0.77	2.89E-24	21
FILIP1.2	1.34E-28	0.506999768	0.27	0.037	3.08E-24	21
AFF1.2	2.04E-28	0.672254325	0.472	0.105	4.70E-24	21
MYL12B.6	2.09E-28	1.044639965	0.978	0.632	4.80E-24	21
ICAM2.1	2.37E-28	0.595661762	0.483	0.106	5.44E-24	21
BST2.12	2.72E-28	1.183930853	0.854	0.358	6.25E-24	21
GIMAP7.18	3.98E-28	1.070031196	0.831	0.281	9.14E-24	21
AHNAK.9	7.64E-28	0.937211654	0.809	0.291	1.76E-23	21
IKBIP.2	9.57E-28	0.516292459	0.483	0.11	2.20E-23	21
KLF7.2	1.11E-26	0.614358693	0.416	0.089	2.56E-22	21
FEZ2.2	1.77E-26	0.630422268	0.539	0.137	4.06E-22	21
YWHAE.13	2.66E-26	0.734663429	0.843	0.309	6.10E-22	21
ENTPD1.4	2.70E-26	0.776452968	0.562	0.151	6.20E-22	21
MT-CYB.15	3.29E-26	0.923672851	0.978	0.803	7.55E-22	21
PEA15.3	3.39E-26	0.66678071	0.461	0.109	7.79E-22	21
NAA38.10	3.49E-26	0.718211833	0.809	0.283	8.03E-22	21
SRP14.8	4.54E-26	0.980495359	1	0.714	1.04E-21	21

CTNNA1.4	4.85E-26	0.615768829	0.517	0.132	1.11E-21	21
C4orf48.3	5.40E-26	0.836665411	0.64	0.195	1.24E-21	21
GINM1.1	7.45E-26	0.578383947	0.517	0.13	1.71E-21	21
TNFRSF1A.4	1.27E-25	0.623739124	0.539	0.146	2.92E-21	21
FRMD4B.1	1.79E-25	0.774396127	0.528	0.139	4.11E-21	21
CTNNB1.2	1.80E-25	0.85539436	0.652	0.201	4.13E-21	21
CDC37.7	2.14E-25	0.773905936	0.82	0.305	4.92E-21	21
RRBP1.4	2.29E-25	0.75707421	0.551	0.152	5.26E-21	21
VAMP3.4	2.29E-25	0.705857569	0.506	0.129	5.27E-21	21
KLF4.3	2.30E-25	0.799579899	0.483	0.118	5.28E-21	21
FNDC3B.3	2.36E-25	0.558996816	0.438	0.099	5.43E-21	21
SNRK.1	2.42E-25	0.730777532	0.472	0.111	5.56E-21	21
TMEM205.2	2.55E-25	0.512552295	0.573	0.151	5.86E-21	21
CALU.2	3.08E-25	0.615623469	0.438	0.101	7.07E-21	21
QKI.4	3.16E-25	0.555662376	0.472	0.115	7.26E-21	21
NTAN1	3.43E-25	0.524571974	0.382	0.079	7.88E-21	21
RHOC.13	3.73E-25	0.841447467	0.854	0.32	8.58E-21	21
BTBD7	1.54E-24	0.627546013	0.36	0.074	3.54E-20	21
NKTR.14	1.90E-24	0.954390069	0.854	0.343	4.38E-20	21
CSRP1.1	2.88E-24	0.556688057	0.438	0.102	6.61E-20	21
FOXP1.11	3.62E-24	0.762528513	0.742	0.254	8.31E-20	21
MBNL2.3	3.93E-24	0.595284359	0.404	0.09	9.02E-20	21
KTN1.12	4.25E-24	0.888597674	0.876	0.38	9.77E-20	21
RPS27L.13	4.66E-24	0.882671973	0.933	0.47	1.07E-19	21
NUCB2.3	1.43E-23	0.633602298	0.596	0.169	3.28E-19	21
LINC00657.3	1.77E-23	0.59947858	0.562	0.158	4.08E-19	21
RRM2B.1	1.81E-23	0.529747024	0.27	0.046	4.15E-19	21
GLS.1	1.84E-23	0.6592387	0.494	0.129	4.23E-19	21
ZKSCAN1.3	2.45E-23	0.506784102	0.494	0.127	5.64E-19	21
FAM3C.5	2.63E-23	0.572771041	0.551	0.151	6.04E-19	21
RPS27.14	2.82E-23	-1.063705971	1	0.975	6.49E-19	21
MACF1.12	3.28E-23	0.962874571	0.708	0.254	7.53E-19	21
CHD9.4	8.10E-23	0.647098112	0.64	0.201	1.86E-18	21
ETS2.4	9.45E-23	0.640666289	0.449	0.116	2.17E-18	21
MED13L.2	1.63E-22	0.585891184	0.416	0.1	3.74E-18	21
CXCR4.15	1.82E-22	-2.914770789	0.551	0.776	4.19E-18	21
MYH9.15	2.27E-22	0.760719073	0.787	0.308	5.21E-18	21
VCAM1.4	2.67E-22	1.550194679	0.404	0.095	6.14E-18	21
NDUFA8.1	3.30E-22	0.627267029	0.506	0.143	7.59E-18	21
CAPNS1.2	4.18E-22	0.683848885	0.539	0.161	9.61E-18	21
FLI1.1	5.24E-22	0.610612558	0.494	0.132	1.20E-17	21
HERC2.2	7.06E-22	0.658156781	0.483	0.129	1.62E-17	21
VAMP5.13	7.23E-22	0.776313015	0.775	0.305	1.66E-17	21
CARHSP1.6	7.51E-22	0.695025432	0.64	0.203	1.73E-17	21
ZBTB20.5	9.84E-22	0.582681884	0.539	0.151	2.26E-17	21
MGST3.15	1.13E-21	0.588039787	0.809	0.303	2.59E-17	21
SERINC3.2	1.15E-21	0.610645613	0.596	0.183	2.64E-17	21
CYBA.14	1.41E-21	-2.474113858	0.303	0.689	3.23E-17	21
XAF1.1	1.44E-21	0.600823026	0.449	0.115	3.32E-17	21
CD63.16	1.92E-21	0.76652742	0.978	0.517	4.40E-17	21
CD55.2	2.52E-21	0.77042509	0.573	0.178	5.79E-17	21
APLP2.17	2.56E-21	0.586908999	0.719	0.253	5.89E-17	21
TMOD3.1	2.66E-21	0.710733426	0.551	0.169	6.10E-17	21
TANC2.2	2.86E-21	0.503557553	0.326	0.068	6.58E-17	21
PLSCR1.6	3.17E-21	0.56655324	0.539	0.159	7.27E-17	21
EID1.7	3.79E-21	0.839643383	0.888	0.425	8.70E-17	21
DYNC1I2.8	6.30E-21	0.633602922	0.674	0.241	1.45E-16	21
MTRNR2L8.4	7.32E-21	1.152688324	0.618	0.223	1.68E-16	21

CLTA.13	7.80E-21	0.628930991	0.764	0.288	1.79E-16	21
ITGB1.12	9.09E-21	0.830143181	0.921	0.461	2.09E-16	21
SERINC1.1	1.20E-20	0.679004475	0.64	0.215	2.76E-16	21
PRDX2.13	1.21E-20	0.515040085	0.742	0.262	2.77E-16	21
HIF1A.5	2.50E-20	0.770615768	0.562	0.18	5.75E-16	21
PIM3.2	4.18E-20	0.55548039	0.438	0.115	9.61E-16	21
UTRN.5	4.33E-20	0.712097839	0.652	0.228	9.96E-16	21
VPS13A.1	4.73E-20	0.500100994	0.382	0.089	1.09E-15	21
CRTAP.3	5.59E-20	0.572702624	0.483	0.14	1.28E-15	21
PTPRC.18	5.74E-20	-2.531643407	0.157	0.619	1.32E-15	21
FILIP1L.3	7.35E-20	0.660552792	0.337	0.077	1.69E-15	21
GIMAP4.15	1.12E-19	0.67265261	0.831	0.335	2.57E-15	21
LAPTM4A.14	1.13E-19	0.580304558	0.843	0.349	2.59E-15	21
RAC1.12	1.14E-19	0.704575607	0.921	0.445	2.61E-15	21
GBP4.2	1.30E-19	0.935785388	0.483	0.144	2.99E-15	21
ATOX1.5	1.32E-19	0.525102264	0.64	0.222	3.02E-15	21
IFITM1.4	2.28E-19	0.689573821	0.573	0.179	5.24E-15	21
POLR2L.8	3.11E-19	0.778493634	0.876	0.434	7.15E-15	21
CDC42EP3.1	3.44E-19	0.508766429	0.461	0.125	7.91E-15	21
HSP90B1.8	3.65E-19	0.708191974	0.865	0.403	8.38E-15	21
CCPG1.2	3.75E-19	0.509429014	0.528	0.161	8.61E-15	21
C5orf24.2	5.12E-19	0.522910071	0.427	0.116	1.18E-14	21
APOL1.1	5.16E-19	0.544478112	0.326	0.074	1.19E-14	21
LPP.3	5.21E-19	0.583705155	0.528	0.164	1.20E-14	21
ENY2.7	7.23E-19	0.54126693	0.764	0.301	1.66E-14	21
TNRC6A.1	7.36E-19	0.532547936	0.427	0.115	1.69E-14	21
LGALS9.3	7.77E-19	0.546655821	0.427	0.118	1.79E-14	21
SH3GLB1.7	8.40E-19	0.586414642	0.697	0.265	1.93E-14	21
NDUFC2.9	8.43E-19	0.547579757	0.764	0.292	1.94E-14	21
MT-ND6.4	9.19E-19	0.643476085	0.596	0.198	2.11E-14	21
RTN4.15	9.37E-19	0.588225516	0.843	0.347	2.15E-14	21
MCFD2.4	1.02E-18	0.53864643	0.371	0.094	2.35E-14	21
LIMS1.13	1.08E-18	0.703038602	0.674	0.246	2.47E-14	21
NUCB1.3	1.19E-18	0.536933732	0.652	0.236	2.74E-14	21
SNHG7.1	1.41E-18	0.643062155	0.551	0.182	3.25E-14	21
ITM2B.13	1.57E-18	0.906601178	0.989	0.778	3.60E-14	21
BRI3.4	1.61E-18	0.649391021	0.506	0.16	3.70E-14	21
PCMTD1.2	2.02E-18	0.594239407	0.539	0.174	4.63E-14	21
ATP1A1.10	2.82E-18	0.608940301	0.685	0.26	6.49E-14	21
TAGLN2.13	2.89E-18	0.691761468	0.787	0.354	6.63E-14	21
MT-ND1.12	4.60E-18	0.76372566	0.978	0.786	1.06E-13	21
CD81.4	5.14E-18	0.614670523	0.506	0.16	1.18E-13	21
RNF115.5	6.11E-18	0.525910877	0.629	0.218	1.40E-13	21
IL13RA1.3	8.26E-18	0.554685776	0.404	0.112	1.90E-13	21
RGS1.20	8.52E-18	-3.343216295	0.483	0.698	1.96E-13	21
LAP3.3	1.06E-17	0.668212016	0.483	0.154	2.44E-13	21
SOCS3.4	1.15E-17	0.697012398	0.506	0.159	2.65E-13	21
TRIM56.4	1.29E-17	0.565823484	0.562	0.188	2.97E-13	21
CNIH1.2	1.80E-17	0.510968555	0.528	0.174	4.14E-13	21
SCARB2.4	2.51E-17	0.55295793	0.371	0.101	5.76E-13	21
PRDX1.12	4.11E-17	0.573832403	0.865	0.401	9.44E-13	21
SEC62.13	5.99E-17	0.548013536	0.876	0.422	1.38E-12	21
BNIP2.2	6.45E-17	0.50236967	0.539	0.181	1.48E-12	21
SPAG9.3	1.27E-16	0.501355632	0.461	0.143	2.93E-12	21
DYNLL1.11	1.37E-16	0.655770104	0.831	0.406	3.15E-12	21
ACLY.1	1.42E-16	0.521627747	0.315	0.077	3.26E-12	21
HCST.16	2.59E-16	-2.217063685	0.124	0.547	5.96E-12	21
MYL12A.10	2.82E-16	0.808518837	0.966	0.688	6.48E-12	21

TM9SF3.1	2.86E-16	0.523311586	0.416	0.125	6.57E-12	21
JMJD1C.9	4.34E-16	0.952350384	0.64	0.256	9.98E-12	21
FIS1.6	4.68E-16	0.662397574	0.697	0.306	1.08E-11	21
ZFP36L2.17	9.31E-16	-2.614179944	0.517	0.7	2.14E-11	21
CORO1A.17	9.38E-16	-2.17471504	0.191	0.567	2.15E-11	21
XIST.14	1.18E-15	0.740262712	0.831	0.421	2.71E-11	21
TPRKB.1	1.32E-15	0.642568415	0.449	0.148	3.04E-11	21
GNG5.12	1.68E-15	0.59300843	0.775	0.349	3.87E-11	21
ARL6IP1.7	2.38E-15	0.664817495	0.708	0.318	5.48E-11	21
MT-ND4.13	2.64E-15	0.591244331	0.966	0.873	6.07E-11	21
WSB1.10	4.89E-15	0.629984821	0.753	0.357	1.12E-10	21
RBMS1.4	5.01E-15	0.507257495	0.562	0.203	1.15E-10	21
ENPP2.3	6.36E-15	-0.754476657	0.427	0.124	1.46E-10	21
BAZ2B.2	7.10E-15	0.507771575	0.472	0.158	1.63E-10	21
S100A4.18	1.31E-14	-2.420401	0.573	0.7	3.01E-10	21
LAPTM5.16	1.32E-14	-2.159949477	0.247	0.569	3.03E-10	21
POLE4.1	1.41E-14	0.503834416	0.517	0.184	3.25E-10	21
ARRDC3.3	1.53E-14	0.626250691	0.528	0.196	3.51E-10	21
STC1.3	1.75E-14	0.739579102	0.281	0.069	4.03E-10	21
RPL28.14	2.56E-14	-0.745412126	0.989	0.932	5.89E-10	21
BTG1.20	3.40E-14	-1.603788104	0.944	0.842	7.81E-10	21
ACTB.16	5.28E-14	-1.060737122	1	0.928	1.21E-09	21
CCL5.19	8.27E-14	-3.526194007	0.236	0.564	1.90E-09	21
CD69.19	1.29E-13	-2.863865376	0.169	0.531	2.95E-09	21
NEAT1.14	1.31E-13	0.590326583	0.966	0.752	3.02E-09	21
SERBP1.5	1.32E-13	0.558282579	0.798	0.401	3.04E-09	21
PARP14.4	2.07E-13	0.782795936	0.528	0.203	4.77E-09	21
NUCKS1.9	2.28E-13	0.519029301	0.82	0.395	5.24E-09	21
LRRFIP1.11	4.81E-13	0.554020862	0.831	0.41	1.11E-08	21
RPS29.16	4.90E-13	-0.823532962	1	0.921	1.13E-08	21
MT-CO3.12	7.62E-13	0.530182214	0.978	0.879	1.75E-08	21
CD37.15	8.66E-13	-1.741115161	0.101	0.465	1.99E-08	21
TRAC.21	1.68E-12	-2.452287243	0.067	0.434	3.86E-08	21
CD44.15	1.79E-12	-1.794647909	0.135	0.493	4.11E-08	21
MTRNR2L12.1	7.94E-12	0.587478324	0.472	0.179	1.82E-07	21
FYB.18	1.48E-11	-1.80567057	0.135	0.462	3.39E-07	21
STK17B.16	2.02E-11	-1.704420517	0.056	0.403	4.63E-07	21
HLA-E.18	2.12E-11	0.545782082	1	0.788	4.87E-07	21
SLA.17	2.40E-11	-1.646023168	0.056	0.401	5.51E-07	21
MT-ND4L.11	2.70E-11	0.555251414	0.73	0.358	6.19E-07	21
GOLGB1.5	3.87E-11	0.533315876	0.517	0.22	8.89E-07	21
CYTIP.18	4.69E-11	-1.788446422	0.157	0.477	1.08E-06	21
CD52.16	8.17E-11	-2.402293728	0.315	0.55	1.88E-06	21
LSP1.20	1.40E-10	-1.605407574	0.124	0.436	3.22E-06	21
CD2.20	1.54E-10	-2.276569551	0.169	0.459	3.55E-06	21
RPL41.16	2.44E-10	-0.503059025	1	0.976	5.62E-06	21
HP1BP3.10	2.99E-10	0.508882129	0.618	0.283	6.88E-06	21
SYNGR2.3	3.59E-10	0.547626225	0.427	0.166	8.25E-06	21
CST7.18	3.83E-10	-2.182113639	0.079	0.391	8.81E-06	21
ITGB2.18	4.79E-10	-1.559612217	0.112	0.417	1.10E-05	21
MT-ND5.12	7.83E-10	0.572429906	0.933	0.608	1.80E-05	21
GMFG.12	9.06E-10	-1.495360591	0.326	0.557	2.08E-05	21
DUSP2.19	1.38E-09	-2.276249268	0.146	0.433	3.17E-05	21
CD3E.20	1.78E-09	-1.744221911	0.112	0.399	4.09E-05	21
GZMA.20	2.94E-09	-2.327280052	0.146	0.421	6.76E-05	21
CD53.14	2.97E-09	-1.448736084	0.202	0.472	6.83E-05	21
SAMSN1.13	4.05E-09	-1.535579263	0.034	0.324	9.30E-05	21
RP11-347P5.1.20	4.46E-09	-1.678194093	0.079	0.377	0.000102453	21

LCP1.18	6.52E-09	-1.413344458	0.124	0.406	0.000149884	21
RAC2.19	9.17E-09	-1.424301728	0.09	0.374	0.000210778	21
RPLP2.11	1.11E-08	-0.502636713	1	0.944	0.000255269	21
GLIPR1.14	1.27E-08	-1.262623262	0.022	0.301	0.000290739	21
H3F3B.13	1.65E-08	-0.883590039	0.955	0.833	0.000378619	21
CD3D.21	1.72E-08	-2.02557695	0.258	0.476	0.000394957	21
RPL27A.10	2.13E-08	-0.52253152	0.966	0.93	0.000489751	21
RPL18A.11	4.03E-08	-0.57030228	1	0.929	0.00092702	21
TXNIP.16	4.53E-08	-1.409050902	0.82	0.763	0.001041308	21
CD48.18	4.78E-08	-1.274263342	0.112	0.377	0.001098322	21
EVI2B.13	4.90E-08	-1.347040146	0.079	0.337	0.001124925	21
RGS10.14	5.16E-08	-1.248338536	0.034	0.297	0.001185121	21
STK4.16	5.71E-08	-1.473860097	0.236	0.473	0.001312562	21
UCP2.16	6.49E-08	-1.316996857	0.09	0.348	0.001491064	21
TRBC2.20	6.88E-08	-1.919723727	0.135	0.378	0.001580795	21
CD3G.20	6.93E-08	-1.430587109	0.034	0.295	0.00159249	21
ARHGDIB.13	9.07E-08	-1.177642587	0.697	0.672	0.002084318	21
IL2RG.21	9.54E-08	-1.540120977	0.135	0.374	0.002192954	21
IL32.17	1.14E-07	-1.902121377	0.438	0.579	0.002626138	21
EVL.19	1.41E-07	-1.484763064	0.303	0.496	0.00324469	21
CELF2.16	2.39E-07	-1.292669346	0.202	0.427	0.005500833	21
SYTL3.18	2.67E-07	-1.282213961	0.034	0.279	0.006145639	21
PRDM1.14	3.87E-07	-1.439208104	0.056	0.293	0.00889068	21
EVI2A.15	4.25E-07	-1.133292128	0.045	0.281	0.009768896	21
LTB.18	4.44E-07	-1.778347477	0.067	0.303	0.01019993	21
ADGRE5.18	4.70E-07	-1.342428906	0.067	0.301	0.010808039	21
RCSD1.13	6.19E-07	-0.964484886	0.022	0.251	0.014222078	21
PIK3R1.15	6.69E-07	-1.287382966	0.079	0.31	0.015368696	21
ACAP1.20	6.95E-07	-1.280463288	0.101	0.334	0.015980345	21
CLEC2D.20	7.47E-07	-1.387802551	0.079	0.308	0.017170766	21
IL10RA.16	8.31E-07	-1.057384978	0.034	0.26	0.019090308	21
TRAF3IP3.19	1.00E-06	-1.079215902	0.034	0.257	0.023049711	21
LCK.21	1.07E-06	-1.253949041	0.067	0.289	0.024647026	21
RNASET2.18	1.16E-06	-1.702256132	0.157	0.362	0.026616853	21
TRBC1.20	1.32E-06	-2.159469626	0.146	0.354	0.030421647	21
BIN2.15	2.17E-06	-1.038316682	0.034	0.251	0.049926296	21
TPSB2	0	8.251276593	1	0.017	0	22
TPSAB1	0	7.937249363	1	0.016	0	22
CPA3	0	4.809157659	0.943	0.005	0	22
CTSG	0	4.287322549	0.415	0.001	0	22
MS4A2	0	3.928966951	0.887	0.003	0	22
HPGDS	0	3.720808044	0.811	0.014	0	22
RGS13	0	3.377325601	0.83	0.01	0	22
VWA5A	0	3.342386805	0.811	0.016	0	22
SLC18A2	0	3.174600699	0.717	0.001	0	22
GATA2	0	3.168052674	0.792	0.012	0	22
IL1RL1	0	3.125006408	0.83	0.003	0	22
KIT	0	2.917228095	0.774	0.003	0	22
HDC	0	2.429023174	0.679	0.001	0	22
RP11-354E11.2	0	1.81396027	0.491	0.001	0	22
MLPH	0	1.502503925	0.377	0.002	0	22
TPSD1	0	1.305977908	0.264	0	0	22
GCSAML	0	1.256758915	0.283	0	0	22
STXBP6	0	1.223559437	0.321	0.002	0	22
AC004791.2	0	0.905269279	0.264	0	0	22
KRT1	8.01E-260	1.66352173	0.302	0.003	1.84E-255	22
NTM	1.57E-246	1.851726608	0.358	0.005	3.60E-242	22
CALB2	2.64E-205	1.378543634	0.321	0.005	6.06E-201	22

P2RX1	4.51E-180	1.386768444	0.321	0.005	1.04E-175	22
C1orf186.3	3.91E-124	2.654359943	0.811	0.057	8.98E-120	22
FLJ21408	1.39E-113	1.259326757	0.321	0.009	3.20E-109	22
MAOB.2	8.76E-109	1.985869411	0.604	0.035	2.01E-104	22
LTC4S.3	2.35E-101	2.928453541	0.83	0.078	5.41E-97	22
HPGD.1	2.92E-94	3.46977765	0.679	0.053	6.72E-90	22
RAB27B	8.26E-83	1.365315555	0.283	0.01	1.90E-78	22
SMYD3	4.13E-63	1.647113959	0.509	0.043	9.50E-59	22
HS3ST1.1	2.51E-51	1.258378219	0.302	0.019	5.76E-47	22
FCER1A	6.48E-46	0.96651783	0.302	0.02	1.49E-41	22
SDPR.3	1.32E-40	1.387280896	0.585	0.078	3.04E-36	22
CAPG.3	2.72E-37	1.993473168	0.868	0.228	6.24E-33	22
BTK.3	4.01E-36	1.491618882	0.472	0.065	9.22E-32	22
AREG.4	6.08E-36	2.572281967	0.849	0.207	1.40E-31	22
ACOT7.1	4.21E-34	1.147923859	0.377	0.042	9.67E-30	22
CD9.6	8.02E-34	1.941419234	0.83	0.221	1.84E-29	22
CSF1	3.81E-33	1.888616868	0.34	0.036	8.77E-29	22
LAPTM4A.15	1.65E-32	2.355380276	0.906	0.35	3.80E-28	22
LMO4.2	3.09E-32	2.234834643	0.585	0.113	7.11E-28	22
CLU.7	6.54E-29	1.84659849	0.755	0.196	1.50E-24	22
EGR1.4	2.00E-28	2.275350135	0.642	0.14	4.59E-24	22
ANKRD28	2.31E-28	1.617982046	0.566	0.109	5.32E-24	22
LMNA.5	3.27E-26	2.020303866	0.679	0.173	7.52E-22	22
STX3	7.15E-26	1.006734213	0.321	0.04	1.64E-21	22
RENBP.2	8.63E-25	1.560799143	0.396	0.065	1.98E-20	22
FER	9.31E-25	0.80196021	0.283	0.033	2.14E-20	22
CD63.17	1.69E-24	1.758479878	0.962	0.518	3.87E-20	22
ALOX5AP.17	4.35E-24	1.731306865	0.849	0.327	1.00E-19	22
MAML1.2	1.43E-22	1.077872399	0.358	0.056	3.28E-18	22
CD69.20	3.04E-22	2.279333022	0.943	0.527	6.97E-18	22
ANXA1.17	3.79E-21	2.043800715	0.887	0.47	8.70E-17	22
JUN.14	6.52E-21	1.609972264	0.962	0.691	1.50E-16	22
ACSL4.2	6.57E-21	1.160202474	0.415	0.081	1.51E-16	22
ALOX5.4	1.29E-20	1.336389169	0.453	0.099	2.96E-16	22
FOXP1.12	2.44E-20	1.655450521	0.755	0.256	5.61E-16	22
PPP1R15A.10	5.23E-20	1.806430228	0.868	0.401	1.20E-15	22
PTGS1.2	7.15E-20	1.247269299	0.321	0.052	1.64E-15	22
FOS.19	1.33E-19	1.811652205	0.943	0.681	3.05E-15	22
MITF.2	1.33E-19	0.92414815	0.321	0.053	3.05E-15	22
CTNNBL1	2.13E-19	1.225342054	0.509	0.125	4.90E-15	22
SLC26A2	3.06E-19	1.350044747	0.302	0.047	7.04E-15	22
PLAUR.3	3.34E-19	1.228317802	0.509	0.121	7.69E-15	22
RNF130.5	8.37E-19	1.315916589	0.585	0.164	1.92E-14	22
GLUL.14	1.04E-18	1.370177028	0.792	0.304	2.38E-14	22
LEO1	1.81E-18	0.858055567	0.302	0.048	4.16E-14	22
H3F3B.14	3.87E-18	1.30082168	0.962	0.834	8.89E-14	22
SAMSN1.14	1.29E-16	1.772666253	0.736	0.321	2.97E-12	22
FTH1.18	1.31E-16	0.877947081	1	0.962	3.02E-12	22
BEX4.2	2.57E-16	1.466249342	0.453	0.119	5.92E-12	22
CD82.3	3.55E-16	1.113120004	0.453	0.111	8.16E-12	22
CXCR4.16	4.53E-16	-2.987768029	0.34	0.777	1.04E-11	22
FOSB.10	5.33E-16	1.43510806	0.83	0.424	1.22E-11	22
IER2.10	6.86E-16	1.950839014	0.792	0.399	1.58E-11	22
TESPA1	1.38E-15	1.058906014	0.321	0.06	3.17E-11	22
ARHGAP18.3	1.64E-15	1.235733824	0.585	0.196	3.77E-11	22
RGS2.19	8.79E-15	2.235535786	0.717	0.334	2.02E-10	22
SRGN.18	1.41E-14	1.133621965	1	0.79	3.23E-10	22
RAB32.2	2.36E-14	1.039661419	0.377	0.092	5.42E-10	22

NR4A1.6	3.36E-14	1.073065718	0.434	0.114	7.73E-10	22
NFKBIA.17	3.70E-14	1.561579351	0.868	0.557	8.50E-10	22
FCER1G.19	6.24E-14	0.902376264	0.792	0.301	1.43E-09	22
TMSB10.14	7.75E-14	-1.265393342	0.943	0.96	1.78E-09	22
ASAH1.8	1.29E-13	1.233140848	0.642	0.239	2.97E-09	22
GALC.1	1.49E-13	0.990146626	0.321	0.07	3.43E-09	22
RHOBTB3.1	1.61E-13	0.964888673	0.283	0.056	3.70E-09	22
MAST4.2	1.77E-13	0.805548811	0.302	0.061	4.06E-09	22
MSRA	1.77E-13	0.875425698	0.34	0.078	4.08E-09	22
FAM46A.2	2.03E-13	1.14689723	0.358	0.086	4.67E-09	22
CD33.3	3.05E-13	1.015253444	0.283	0.058	7.02E-09	22
MT-ND4L.12	3.67E-13	1.257016778	0.736	0.359	8.44E-09	22
RPL28.15	4.97E-13	-0.962944963	0.962	0.932	1.14E-08	22
TXNIP.17	5.20E-13	-2.39506609	0.434	0.765	1.20E-08	22
RPL37A.6	1.08E-12	0.703542873	0.962	0.906	2.48E-08	22
RGS1.21	1.51E-12	1.344834902	0.962	0.696	3.46E-08	22
ADRB2	1.94E-12	1.095157419	0.302	0.067	4.45E-08	22
PTMA.10	1.98E-12	0.721636281	0.962	0.917	4.56E-08	22
SEPT2.10	2.43E-12	1.193756388	0.604	0.26	5.58E-08	22
RPL34.13	2.49E-12	0.659559388	0.981	0.953	5.72E-08	22
SLC44A1.3	2.93E-12	1.015288784	0.321	0.077	6.73E-08	22
CRBN.1	3.07E-12	1.008147561	0.491	0.166	7.06E-08	22
ZSWIM4	3.22E-12	1.144215175	0.34	0.083	7.39E-08	22
STMN1.2	3.64E-12	0.830419473	0.453	0.137	8.36E-08	22
C4orf48.4	4.66E-12	1.187662956	0.528	0.197	1.07E-07	22
CPM.4	5.90E-12	1.136486435	0.453	0.136	1.35E-07	22
HLA-B.17	1.36E-11	-1.047034036	0.906	0.914	3.12E-07	22
GRAP2	1.92E-11	0.961177595	0.321	0.076	4.41E-07	22
MT-ND3.15	2.90E-11	0.776598593	0.981	0.83	6.67E-07	22
CKLF.14	3.04E-11	1.185916295	0.698	0.362	6.99E-07	22
MT-ATP6.15	3.21E-11	0.706507442	0.981	0.859	7.37E-07	22
TMEM154.1	3.37E-11	0.698284792	0.321	0.079	7.74E-07	22
RPL7.11	3.61E-11	0.581294845	1	0.92	8.30E-07	22
PLIN2.15	4.57E-11	0.929118593	0.717	0.334	1.05E-06	22
TSC22D1.6	4.93E-11	0.998388194	0.528	0.182	1.13E-06	22
CTD-3252C9.4.3	5.58E-11	1.015908059	0.547	0.201	1.28E-06	22
ITM2C.5	6.54E-11	0.768569236	0.491	0.155	1.50E-06	22
SEMA4A	1.24E-10	1.016961794	0.264	0.06	2.85E-06	22
NFKBIZ.2	1.76E-10	1.422849919	0.528	0.215	4.06E-06	22
IL32.18	4.10E-10	-2.477360254	0.151	0.579	9.42E-06	22
CCL5.20	4.92E-10	-3.138095431	0.132	0.563	1.13E-05	22
NDUFA4.11	5.07E-10	1.01165716	0.83	0.538	1.17E-05	22
HLA-DPB1.17	6.05E-10	-2.988964422	0.264	0.634	1.39E-05	22
FYB.19	6.58E-10	-2.043688571	0.019	0.461	1.51E-05	22
PEBP1.16	8.44E-10	1.043042365	0.717	0.389	1.94E-05	22
HLA-A.17	8.80E-10	-1.103989019	0.887	0.894	2.02E-05	22
CORO1A.18	1.05E-09	-1.969636424	0.17	0.566	2.41E-05	22
KCNQ1OT1.3	1.08E-09	0.718604331	0.34	0.096	2.49E-05	22
SOCS1.3	1.83E-09	1.390761592	0.472	0.18	4.20E-05	22
MT-ND4.14	2.19E-09	0.676796657	0.943	0.874	5.03E-05	22
PFN1.14	2.34E-09	-1.409438154	0.547	0.761	5.39E-05	22
MT-CO2.13	2.81E-09	0.642985284	0.943	0.874	6.45E-05	22
HLA-C.16	3.16E-09	-0.973769091	0.906	0.883	7.26E-05	22
TMSB4X.12	3.73E-09	-0.722108453	1	0.977	8.57E-05	22
JUNB.18	4.62E-09	0.956060725	0.981	0.722	0.000106062	22
CD74.15	4.89E-09	-2.539369832	0.623	0.795	0.000112373	22
GPR65.3	5.09E-09	1.303581824	0.509	0.208	0.000116868	22
LYL1.3	5.61E-09	1.05427964	0.264	0.07	0.000128874	22

FXYD5.13	6.31E-09	0.99984847	0.774	0.48	0.000145116	22
HLA-DPA1.17	8.11E-09	-2.999300215	0.226	0.572	0.000186321	22
ATP6V1F.11	1.22E-08	1.015599869	0.66	0.349	0.000280978	22
CD3E.21	1.33E-08	-1.998534949	0	0.399	0.00030537	22
PRNP.1	1.82E-08	0.798582446	0.377	0.132	0.00041913	22
SELK.9	2.33E-08	1.065049848	0.623	0.333	0.000536421	22
HINT1.10	2.38E-08	-1.523944637	0.283	0.627	0.000545919	22
S100A6.12	2.70E-08	0.618243886	0.906	0.766	0.000619427	22
LAT2.3	2.90E-08	1.03518479	0.302	0.095	0.000667057	22
CYTIP.19	3.04E-08	-1.459123085	0.075	0.477	0.000699126	22
LPCAT2.2	3.14E-08	1.010759445	0.283	0.085	0.000720752	22
CD2.21	3.88E-08	-2.325002197	0.094	0.458	0.000891391	22
TRAC.22	4.97E-08	-2.321508434	0.057	0.433	0.001143266	22
DUSP10.1	5.44E-08	0.820794666	0.321	0.101	0.001250187	22
BHLHE40.4	5.52E-08	0.938302496	0.434	0.171	0.001268206	22
GPX4.13	6.02E-08	0.768245357	0.774	0.453	0.001383568	22
FOSL2.1	6.39E-08	0.784052181	0.358	0.12	0.001468961	22
AHI1	6.85E-08	0.566194423	0.283	0.082	0.00157308	22
ACTB.17	8.41E-08	-0.971870536	0.981	0.929	0.001933461	22
PLGRKT	1.07E-07	1.029749364	0.302	0.097	0.002469746	22
SPINT2.5	1.30E-07	0.602790353	0.396	0.149	0.002998459	22
SNX29.1	1.40E-07	0.730465272	0.264	0.078	0.003208263	22
SDCBP.16	1.44E-07	0.700450761	0.698	0.371	0.003307687	22
S100A10.13	1.46E-07	-1.889237739	0.321	0.611	0.00334795	22
EVL.20	1.95E-07	-1.547656633	0.132	0.497	0.004470839	22
RPS6KA5.1	2.43E-07	0.893352803	0.283	0.089	0.005588099	22
EMP3.11	2.51E-07	0.856791587	0.679	0.389	0.00577396	22
TSPAN4.5	2.52E-07	0.950260783	0.302	0.101	0.005796887	22
ANXA2.15	2.86E-07	-1.816794693	0.038	0.394	0.006578722	22
STK17A.19	3.03E-07	-1.619094742	0.019	0.368	0.006965955	22
BST2.13	3.32E-07	0.796477608	0.642	0.36	0.007639354	22
CFLAR.13	4.50E-07	-1.478452487	0.075	0.424	0.01034507	22
TRBC2.21	5.00E-07	-1.99511206	0.038	0.378	0.011482283	22
BTG2.11	5.15E-07	1.256099584	0.604	0.356	0.011836418	22
GZMA.21	5.21E-07	-2.003533877	0.075	0.42	0.011982561	22
TSEN54	5.90E-07	0.735610714	0.302	0.098	0.013556069	22
DUSP1.16	5.91E-07	0.646746107	0.943	0.793	0.013580799	22
GADD45B.5	6.01E-07	0.8565421	0.509	0.233	0.013814814	22
ACTG1.12	6.58E-07	-1.194172488	0.642	0.746	0.015125268	22
CTSD.12	6.92E-07	0.760092029	0.679	0.436	0.015907836	22
RPL36AL.7	7.04E-07	0.712806784	0.887	0.711	0.016187591	22
HCST.17	7.60E-07	-1.56079087	0.226	0.546	0.01746903	22
MAPRE1.2	7.68E-07	0.919891817	0.415	0.183	0.017653535	22
ACER3.3	8.06E-07	0.727548241	0.264	0.086	0.018512387	22
PRDX6.10	1.02E-06	0.849433721	0.585	0.324	0.023498779	22
ETS1.18	1.10E-06	-1.59150905	0.019	0.34	0.025186521	22
GIMAP4.16	1.21E-06	-1.46999851	0.019	0.34	0.02772635	22
SYAP1.6	1.21E-06	1.012876734	0.509	0.248	0.027824423	22
IL18.2	1.62E-06	0.657699941	0.302	0.106	0.037120438	22
CD3D.22	1.65E-06	-1.623404799	0.17	0.476	0.037871478	22
SYNE2.18	1.75E-06	-1.552179744	0.019	0.333	0.040261268	22
TRBC1.21	1.85E-06	-2.240037842	0.038	0.354	0.042611102	22
ISG20.16	1.95E-06	-1.376904797	0.038	0.362	0.044724579	22

gene
IL7R
ZFP36L2
GPR171
CD69
TNFAIP3
CD40LG
BTG1
CD52
CXCR4
TSC22D3
RPS29
RPS27
RPL28
RPLP2
JUNB
SARAF
KLF6
RPS3
JUN
CD2
AC016831.7
FTL
CD74
CD3D
STK4
RORA
TXNIP
CD44
HLA-DRB1
CD63
CST3
CD3E
RPL14
ANXA1
IL32
PIK3IP1
RHOB
LEPROTL1
LTB
HLA-DRA
TYROBP
RP11-138A9.2
STK17A
CYTIP
IFITM3
TRAC
RP11-347P5.1
RP11-138A9.1
ODF2L
FCGR3A
FCER1G
HLA-DMA
SERPINA1
HSPB1
RPSA
GSTP1
SPOCK2

GPX1
DDX5
FCGRT
ZFP36
RGS1
PPP2R5C
GSN
TRAT1
PTMS
PDK4
CD9
AC092580.4
GRN
HLA-DQA1
CD3G
NFKBIA
IDS
NPC2
EVL
APLP2
PTGER4
S100A4
PTPRC
AIF1
SRSF7
NNMT
HLA-DPA1
HLA-DMB
CD96
KLRB1
PSAP
PDCD4
NDUFA4L2
HLA-DQB1
SPP1
ADIRF
HLA-DPB1
AIM1
CEBPD
FTH1
ASAH1
TRBC2
LAPTM4A
RAC1
LYZ
ACAP1
FOSB
YWHAH
CRYAB
CYB5A
GAPDH
TUBA4A
TPI1
FKBP1A
IGFBP7
BST2
FKBP11
SOD2

PKM
ETS1
AAK1
APOC1
GPR183
EML4
ATOX1
YWHAE
LCK
SMCHD1
TRBC1
FXYD5
CCNH
AKR1B1
HSPA1A
ISG20
HSBP1
ANXA5
TYMP
NDUFC1
C1QA
CLEC2D
PPP1R2
PRDX1
CTSB
TMEM173
AP2S1
LST1
HSP90B1
TNFAIP8
STK17B
FAM129A
PLIN2
NAMPT
CD59
PARP8
C1QB
ALDOA
NKG7
ATP6V0B
CLTA
EMB
GNG5
SAMSN1
CMC1
HLA-DRB5
CNOT6L
FKBP2
AP1S2
RNF19A
CTSD
SLIRP
CTSS
LYAR
MT2A
BTG2
RGS2
TOB1

RHOC
APOE
ELF1
CD48
SNX3
SAT1
DSTN
SLC2A3
RTN4
IGFBP3
PLP2
PDE4B
CANX
TIMP1
GLRX
GSTO1
FYN
PRDX6
BNIP3L
SYNE2
LINC-PINT
FOXP1
CELF2
RNASET2
HMGN3
PPT1
SOCS1
ARHGAP15
ID2
GPX4
PBXIP1
CAPZA2
HSPD1
PRDM1
TUBB
KIAA1551
GLUL
CALR
VDAC1
NEAT1
ZC3HAV1
DDX24
DBI
RPS27L
POMP
RHOH
LAMTOR5
YPEL5
PIK3R1
ZFAND5
ATP6V1F
TUBA1B
VAMP5
MGST3
ENO1
TAGAP
SEPW1
LGALS1

ARL4C
ANKRD12
ANXA2
ITM2B
NDUFB4
SDCBP
HNRNPUL1
IKZF1
CSTB
HSP90AB1
MT1X
6-Sep
PCSK7
UQCRQ
LDHA
IL10RA
VIM
LGALS3
MIF
S100A9
PEBP1
TXN
NR3C1
RBPJ
S100A11
GNLY
GZMB
KLRD1
FGFBP2
PRF1
NKG7
KLRF1
CCL4
CLIC3
GZMH
SPON2
PLAC8
CD7
KLRB1
TRDC
HOPX
CST7
S1PR5
GZMM
CD247
MATK
CTSW
FCGR3A
PTGDR
SYTL3
CCL5
CMC1
CCL3
BIN2
GZMA
VIM
XCL2
TXK

SAMD3
KLRG1
DUSP2
GPR65
HCST
ZEB2
PYHIN1
LITAF
MT-CO1
IL2RB
AREG
CD69
MT-CO2
ID2
TYROBP
AKNA
ADGRE5
FTH1
HLA-C
FYN
IRF1
MYL12A
CD74
MAPK1
KLF2
TRGC2
AC092580.4
CHST12
MYO1F
HLA-E
IER2
IFITM1
MBP
GAPDH
ANXA1
ZAP70
HLA-DRB1
PPP2R5C
CCND3
SRGN
ARL4C
HLA-DRA
CD53
RUNX3
PTPN4
RAC2
FKBP11
STK4
RNF125
CLEC2B
COTL1
SUN2
PIP4K2A
HLA-DQB1
RBM39
LYAR
ISG20
HSPB1

GNG2
RORA
TLE4
TSC22D3
S100A11
EVL
CCL4L2
SYNE1
ANXA5
CYBA
CD27
RAP1B
TAGAP
GPX1
FTL
HLA-DMA
CST3
STAT4
PRDX1
PIK3R1
SERPINA1
HLA-DRB5
TBC1D10C
RARRES3
GNPTAB
CTD-3252C9.4
CH17-189H20.1
CYTIP
EFHD2
AES
LGALS3
PLEK
VAMP2
ARHGAP9
HLA-DPA1
CTSB
ATM
TYMP
SELPLG
BTG2
RNASET2
APMAP
LTB
FCGRT
HLA-DQA1
ETS1
NPC2
RP5-1171I10.5
LCP1
EIF3G
AIF1
GRN
ITGB2
ABI3
S100A6
NR4A2
CDC42SE1
LINC00869

IFITM3
PPT1
CORO1B
LYZ
PTMS
ZFP36
PKM
PTGER4
ADIRF
NNMT
C1orf56
PTPN7
ARPC5L
TIMP1
AOAH
ARHGEF1
WIPF1
SOD2
IQGAP2
NDUFA4L2
YWHAH
RGS10
ENO1
NDUFC1
GPATCH8
LIMD2
CRYAB
C9orf142
DOK2
IKZF1
ITM2B
RSRP1
PRKCH
FKBP1A
PARP8
LIMS1
GPX4
NFKBIA
SOCS1
TMEM2
RAC1
FLNA
RBPJ
RGS1
JAK1
LGALS1
S100A10
CNOT6L
SPN
APOC1
CD47
ANXA2
CD84
PLIN2
HLA-DPB1
IGFBP3
ALDOA
IDI1

DUSP4
GZMK
TXN
MT1X
YWHAE
MGST3
SAT1
REL
RPS27L
MT2A
C1QA
GSTO1
HSBP1
RHOB
PSAP
CD55
DYNLL1
LAPTM4A
SLFN5
APOE
BIRC3
PEBP1
FMNL1
NAP1L1
HERPUD1
CLTA
RPLP0
SOD1
APLP2
HINT1
MIF
VMP1
SNX3
CSTB
LDHB
LDHA
S100A9
TSPO
VAMP5
TPI1
TRAC
NDUFB4
TUBA1B
TUBB
ZFAS1
SEPW1
PGK1
CTSS
GLUL
GZMK
CCL5
CD8A
CCL4
CD27
GZMA
CD8B
RGS1
CST7

NKG7
DUSP4
CCL4L2
CD3D
DUSP2
ITM2A
CRTAM
BTG1
HLA-DQA2
CXCR4
CD2
HLA-A
CD3E
TRAC
TRBC2
VIM
RPS26
FTH1
APOBEC3G
RP11-347P5.1
TOX
CMC1
LYST
TRBC1
CD69
CTSW
LCK
TPT1
LAG3
TNFRSF9
SRSF7
HCST
EVL
CD3G
CORO1A
PTPRC
STK4
TRAT1
SIT1
TIMP1
ACAP1
IFITM2
TMEM123
CCDC167
CEBPD
IFNGR1
SUB1
CCL3
IFITM3
ITM2C
RARRES3
IL2RG
KIAA1551
CD84
RHOB
CLEC2D
SOD2
SERPINA1

DSTN
TERF2IP
XIST
CST3
TYROBP
PYHIN1
FCER1G
TMSB10
APLP2
NNMT
GRN
RNASET2
GZMH
PSMB9
GLUL
S100A11
TUBA4A
ISG20
PLIN2
CYTIP
LINC00152
CTSB
WNK1
NDUFA4L2
PPP2R5C
STK17A
HLA-F
C10orf54
LDHA
RAC2
ANXA2
DDIT4
FCGRT
S100A10
CD63
RPL17
PNISR
MSI2
CRYAB
ASAH1
APOBEC3C
HSPB1
PMAIP1
BTN3A2
MT-ND4L
GPX1
FTL
ANXA6
PTPN7
ZBTB38
ADIRF
HAVCR2
YWHAE
TMEM2
YWHAH
LAPTM4A
AC090498.1
CFLAR

IL7R
AP3S1
RTN4
ITM2B
PPP1R2
GALM
TYMP
RAC1
RHOH
TBC1D10C
ZFAND5
NPC2
LAT
RUNX3
TAGLN2
PPT1
ANXA1
UCP2
CCND3
TSPYL2
PSAP
TRAF3IP3
MIF
MGST3
DOCK8
LYZ
GPATCH8
SNX3
CEBPB
CHST12
WIPF1
GPSM3
CBLB
PCSK7
PIP4K2A
PRKCH
IGFBP3
LIMD2
LGALS3
NDUFC1
AIF1
HLA-DRA
IGFBP7
GNLY
ENO1
TUBA1B
NFKBIA
6-Sep
C1QA
S100A6
NEAT1
TPI1
FOS
TXN
SAT1
APOC1
MT2A
HSPA1B

RPS4Y1
HSPA1A
HSPA6
DNAJB4
AC090498.1
RPL17
PSMD5-AS1
TRBC2
CTSW
TRAC
CD27
XIST
GOLGA8A
CD3E
LINC00649
CD3G
HSPH1
NABP1
CD2
PTPRC
SLFN5
OXNAD1
ITM2A
CRIP1
TBC1D4
SIRPG
GOLGA8B
HNRNPA1L2
SPOCK2
KIAA1551
TTN
PYHIN1
RPS4X
HLA-DRB5
CACYBP
CD3D
EOMES
TRAF3IP3
TNFRSF9
GABPB1-AS1
DENND2D
RPS29
HSPA8
PILRB
HSPE1
FTH1
LTB
ITGA4
MSI2
IFITM1
HSPD1
CD82
HSP90AA1
MT-ND5
DNAJB1
DUSP1
SKAP1
TXK

LCP1
IKZF1
CCND2
CCL3L3
IL32
SH2D1A
RBL2
EMB
IL2RG
RNF213
CD8A
MDFIC
MT-ND4L
AAK1
ARAP2
LAPTM5
PBXIP1
PIK3IP1
CD8B
ITM2C
TRBC1
BCL2
ZFP36
CYLD
LYST
RAC2
RP11-796E2.4
UCP2
NR4A1
CHORDC1
PHTF2
TRIM22
GZMK
JUNB
TSC22D3
UGP2
NKG7
CMC1
TMSB10
DNAJA1
FTL
FOS
CCL4L2
SPP1
KLRB1
CEBPB
MT1E
IFITM3
HBA2
IGFBP3
S100A9
GNLY
GSTP1
IGFBP7
RP11-347P5.1
ANXA1
APOE
YBX1

HLA-DRB1
RAC1
MT2A
ITM2B
IGKC
RGCC
CD63
VIM
HBB
KLF6
HLA-DPB1
TNFAIP3
NNMT
CEBPD
POMP
CRYAB
DSTN
LAPTM4A
RGS1
HLA-DPA1
NR4A2
MT1X
MT1X
MT2A
NNMT
ANGPTL4
B2M
NDUFA4L2
TMSB4X
HILPDA
CD24
HLA-B
GAPDH
HLA-E
HLA-C
RPLP0
RPL36
TMSB10
FTH1
EGLN3
ARHGDIB
PTPRC
BTG1
CORO1A
ZFP36L2
HLA-A
RPS8
MYL12A
ARPC2
LAPTM5
GMFG
RPL41
ENO1
RPL10
TMA7
CD53
HCST
SRGN

FYB
HLA-DPB1
CELF2
RPS5
CFL1
EVL
CYBA
CRYAB
RGS1
S100A4
CXCR4
CD44
SRSF7
CD74
CD37
STK4
RPL13
LDHA
RBM39
GPSM3
LSP1
HLA-DPA1
RARRES2
PNISR
PRPF38B
HLA-F
DDX5
ITGB2
MGST1
CD2
ARGLU1
RHOA
CCL5
AKAP13
ELF1
DDX24
CALM1
CYTIP
JAK1
SH3BGRL3
LCP1
ARL6IP5
SLA
VAMP2
VIM
CD52
RPL18A
RPS19
TNFAIP3
KMT2E
ATP1B1
RNF213
YWHAZ
CFLAR
MALAT1
CD69
GIMAP4
TRAC

YWHAB
IL2RG
N4BP2L2
GZMA
FNBP1
CD3E
HSP90AA1
AAK1
CD48
ATP5E
SOD2
RAC2
HMGB2
HSPA8
FXYD5
WIPF1
DNAJB1
TPM3
KIAA1551
ARPC1B
PSMB9
DDX17
CAP1
RSRP1
RPL3
STK17A
RPL7
DUSP2
ATP5L
SON
NKTR
RPL21
SP100
GNAS
RBM25
HLA-DRB1
SRSF2
MT1E
UCP2
RPS2
ITGB1
PDK4
CD3D
CAPZB
7-Sep
ARPC3
LRRFIP1
PFN1
HLA-DRA
TERF2IP
PSME1
PIK3IP1
RPL24
HERPUD1
NKG7
LITAF
PRRC2C
IDS

TRBC2
MBNL1
ISG20
CTSC
SMAP2
SH3KBP1
SRSF11
ID2
C9orf16
GIMAP7
ARPC5
CST7
MSN
MT-ND5
ATRX
ACTR2
PCSK7
CCND3
ACAP1
SMCHD1
BCLAF1
LIMD2
CAV1
DOCK8
RPS6
REL
EVI2B
ROCK1
SRRM2
ETS1
RP11-347P5.1
RPS18
BTG2
RGS2
NCL
LCK
PIK3R1
CKLF
HCLS1
IGFBP3
EMB
UBC
GCC2
FKBP5
6-Sep
PPP1R2
CRIP1
ARID4B
RBPJ
GLIPR1
UBB
CARD16
MYO1F
APOBEC3G
GNAI2
TRBC1
STK17B
RPS28

CD27
IQGAP1
CCL4
NR4A2
YPEL5
ANKRD44
GABPB1
ARHGEF1
HLA-DQB1
TMEM50A
COTL1
CD3G
CD84
RPS4X
ITM2A
TRAF3IP3
RCSD1
MYCBP2
ABRACL
LTB
EPC1
PPP2R5C
RPL34
RPL26
H2AFY
SYNE2
AKNA
CD7
FYN
EIF3A
RGCC
C10orf10
PNN
ICAM3
CCNH
IKZF1
SAMSN1
WNK1
PAK2
ACTB
BIN2
RGS10
EVI2A
KIF5B
MACF1
MT-CYB
ALOX5AP
YWHAH
AES
RHOG
MYL6
TRIM22
HLA-DQA1
RNF19A
CLEC2D
PRKCH
2-Sep
ARL6IP1

JMJD1C
C10orf54
IL10RA
RPS29
TGOLN2
ARHGAP15
ADGRE5
IFITM2
TUBA4A
BTN3A2
C9orf142
ATP1B3
ATM
RPL36A
BNIP3
CMC1
OSTF1
SPP1
GZMK
CTSS
BUB3
FKBP1A
PRDM1
ODF2L
CREM
SYTL3
PCM1
USP15
TXNIP
ANXA6
ITSN2
CD247
PPP1R18
MIF
RPL31
HLA-DMA
LIMS1
AC090498.1
DUSP4
CD96
CLU
TPI1
IL7R
RPL18
C1QA
RPL35A
RPL38
TYROBP
SAT1
EGR1
RPL5
HLA-DRB5
RPL8
NUPR1
RPS16
RPS13
APOE
RPL6

PLIN2
RPS17
RPL9
ZFAS1
DDIT4
GPX1
CYB5A
SERPINA1
FCER1G
AIF1
DUSP1
S100A10
CST3
RPL22
NACA
ALDOA
GNLY
PGAM1
RNASET2
IFITM3
CCT8
SSBP1
MZT2A
RSL1D1
MZT2B
TMED2
TOMM20
ZFP36L1
SNRPE
PRDX6
IGKC
PCNP
EIF2S3
RHOB
HSPD1
FBL
HSPB1
RSL24D1
NPM1
VMP1
FTL
FTH1
LYZ
S100A9
TYROBP
CXCL8
AIF1
S100A8
HLA-DRA
FCER1G
IL32
CD2
RPS27
CCL5
SRSF7
ZFP36L2
SON
CD69

S100A11
CD3D
PNISR
EVL
TRAC
CD14
STK4
CD3E
MALAT1
JUN
FCN1
DDX24
RBM39
N4BP2L2
TSC22D3
VAMP2
RPL23A
GZMA
CIRBP
XIST
DUSP2
RP11-1143G9.4
ITGB1
FUS
RNF213
CST7
TRBC2
ANAPC16
SYNE2
ETS1
PTPRC
ARGLU1
AAK1
PPDPF
RARRES3
MT-ND2
RSRP1
C12orf57
ACAP1
HLA-F
POLR2J3
KLF6
KMT2E
RPL13A
PRRC2C
RPS29
STK17A
ATRX
CD99
ACTB
PIK3IP1
LY6E
ID2
SAT1
NKG7
JAK1
CXCR4
CD74

TNFAIP3
CFLAR
GCC2
RPS26
PPP2R5C
CUTA
NKTR
TERF2IP
AKAP13
CD3G
LCK
LEPROTL1
FNBP1
TRBC1
PRPF38B
MT-CYB
C1QC
IDS
BTG1
ITM2A
AKAP9
IL2RG
CLEC2D
ANKRD12
PEBP1
SPP1
ARID4B
GZMK
CD7
GLUL
GIMAP7
RP11-347P5.1
HLA-DRB5
KIAA1551
CCND3
HLA-DRB1
HLA-A
DDX5
OCIAD2
RPS3
LST1
PCSK7
FYN
RPL3
MPHOSPH8
RNF19A
GPX1
SYTL3
CCL4
CSTA
SF1
TAF7
EZR
PPP1R2
BTN3A2
MT-ATP6
CD27
PIK3R1

ODF2L	
MBNL1	
TNRC6B	
CST3	
EPC1	
RPL31	
FOSB	
	6-Sep
CD96	
CCNH	
APOBEC3G	
AKNA	
CD247	
CMC1	
YWHAQ	
BUB3	
LIMD2	
NPC2	
SRGN	
C9orf142	
PDCD4	
MYCBP2	
PRKCH	
C1QB	
CRIP1	
ATM	
RHOC	
MNDA	
DOCK8	
GABPB1	
AES	
IGFBP7	
	9-Sep
DUSP4	
PRDM1	
DNAJB1	
IL7R	
C1QA	
BBX	
TRAF3IP3	
RSF1	
IKZF1	
MACF1	
WHSC1L1	
TSTD1	
EMB	
BIN2	
WNK1	
DSTN	
REL	
BIRC3	
ARID5B	
S100A6	
CCDC85B	
ANXA6	
HLA-DPA1	
LTB	
CD68	

HLA-DPB1
CTSS
RGCC
NAMPT
IGFBP3
NDUFA4L2
MS4A6A
RGS1
C1orf162
HLA-DQA1
GNLY
CRYAB
APOC1
H3F3A
IFITM3
APOE
OAZ1
HLA-DQB1
PSAP
NNMT
TIMP1
CTSB
SDCBP
GMFG
MAP1LC3B
LAMTOR4
ATP6AP2
HCLS1
RGS10
CEBPB
LGALS1
C4orf3
LITAF
RAP1A
CYBA
ATP6V0E1
CALR
RGS2
HSD17B11
FCGR3A
CARD16
MT2A
PGK1
SERPINA1
NOP10
CTSC
TYMP
RAB5C
APOE
C3
HLA-DRB1
CST3
C1QB
C1QC
APOC1
HLA-DPB1
C1QA
CD74

HLA-DRA
HLA-DPA1
GPX1
MS4A6A
HLA-DQA1
FCGR3A
TREM2
NPC2
KCTD12
HLA-DMB
SGK1
MS4A7
SLC1A3
TYROBP
HLA-DMA
FCGR2A
C1orf162
PLXDC2
MAFB
HLA-DQB1
GPR34
CSF1R
MEF2C
GRN
LILRB4
OGFRL1
CSF2RA
CXCL16
ALDH2
PSAP
CD302
AIF1
FGL2
HERPUD1
MARCKS
CTSB
FCGRT
SLC11A1
MEF2A
USP53
SAT1
FN1
CEBPD
CD14
VSIG4
CD163
IL18
PLD4
FCER1G
MSR1
LY86
PPT1
CPVL
ALOX5AP
FAM26F
FCGR1A
LIPA
RPS4Y1

MNDA
CAPG
LST1
LPAR6
SPI1
RGS10
IL13RA1
MFSD1
EPB41L2
RNASET2
TLR2
CD68
RNF130
RNASE6
LYZ
TBXAS1
LPCAT2
CSF3R
SORL1
FKBP5
CTSS
ADAP2
SLC8A1
CTSZ
TYMP
KCNMA1
SRGAP1
CLEC7A
SLCO2B1
ARHGAP24
CYBB
CTSH
OLR1
YWHAH
IFNGR1
CPM
DOCK4
IFI30
GLUL
RAB31
IGSF6
SERPINF1
OLFML3
CD9
CLEC4E
ARL5A
FCGR1B
1-Mar
CD86
PABPC4
C10orf54
HCLS1
GAPT
CNPY3
PKIB
ALOX5
ADAM28
TM6SF1

LAIR1
SRGAP2
FCGR2B
ANKRD22
DAB2
RP11-552D4.1
NCOA4
TNFSF13B
PLBD1
NUDT3
CLEC5A
GNB4
CADM1
MIS18BP1
ACSL1
C3AR1
ASAH1
LTC4S
GABARAP
RASSF4
ALCAM
TGFBI
AKR1B1
RGS18
PLTP
RB1
FCGBP
SESN1
PRKAG2
GNAQ
APBB1IP
ELMO1
PILRA
ADORA3
HEXA
ARHGAP18
TNPO1
PICALM
PAK1
SAMHD1
DSE
HTRA1
LIMS1
UBL3
HLA-DOA
CFD
NUDT16
FRMD4A
TANC2
SLC7A7
LINC01094
SCPEP1
ATP6V0B
DPYSL2
ATP6V1B2
ZFHX3
CCDC88A
MAN2A1

AZI2
FPR1
PYCARD
LHFPL2
BHLHE41
LYN
DST
KCNMB1
SRGAP2C
SIGLEC10
FILIP1L
SRGAP2B
MAP3K8
AXL
SDCCAG8
VMO1
LAP3
RHBDF2
A2M
APLP2
SKAP2
CSTA
ZNF812P
METTL7A
FEZ2
LGALS9
SWAP70
TMEM176B
P2RY13
IRAK3
GAA
SLC43A2
SNX10
CLN8
BMP2K
TFEC
LY96
SYK
BTK
FMNL2
FCHO2
TMEM14C
LAT2
ST14
RNF13
FGD4
DPYD
DNASE2
TOP1
BASP1
SAP30
CEP170
SOAT1
SCIN
FAM105A
PTGS1
DRAM2
RIN3

CHPT1
HEXB
SFMBT2
SYNGR2
MS4A4A
CD300A
STX6
STX7
AP1S2
GRINA
THEMIS2
AP1B1
FRMD4B
CAMK1D
TKT
PADI2
CRTAP
LRP1
ARRB2
PEA15
RAB32
HCK
CTTNBP2NL
PAPOLG
LRRK2
DAGLB
SCIMP
NUP214
GSN
SPINT2
SKIL
HIF1A
PARVG
TLR5
CD81
C5AR1
DENND3
CREG1
SLC40A1
APPL1
CD4
ABHD12
CYFIP1
SSH2
CTNND1
SHTN1
LRRC25
SERPINA1
CHN2
PSTPIP2
CNDP2
PLSCR1
ALOX15B
QKI
ATP6AP1
UNC93B1
TPP1
CAT

RHOBTB3
HAVCR2
NAIP
INTS10
SPTLC2
GPR155
COMT
NAGK
TGFBR1
PTEN
FGR
CLEC12A
CD33
KLHL5
ETS2
IFNGR2
PLEKHO1
BEX4
ZYX
DAPK1
PRKAG2-AS1
SCAMP2
HSPA6
ACSL4
RP11-108M9.4
SVBP
IER3
C1orf54
RBM47
ITPR2
ENTPD1
SLC31A2
RTN1
ST3GAL6
STK38L
EPB41L3
BCAT1
HMOX1
CTSL
ITPRIPL2
C20orf27
RNASE2
FAM96A
TAX1BP3
LILRB1
AP001055.6
SNCA
POU2F2
KLF7
NLRP3
PLXNC1
NDRG2
MMP2
PTAFR
RNF144B
ADPGK
FAM110B
MITF

KIAA1033
PALD1
TNFSF12
MERTK
ZSWIM7
BRI3
FGD2
IRS2
JAK2
VAMP3
ZNF385A
EMILIN2
MANBA
PRCP
C10orf11
HNMT
ITGAM
SPECC1
PKP2
GALNT2
LPL
NAGA
UBE2E2
TIMP2
LACTB
TCF4
GPRIN3
POLD4
ZNF106
CD84
ZEB2
BAZ2B
PTPN18
ERCC1
CMTM6
FOXO3
LAPTM5
GLIPR1
SMAP2
NCF1
AOAH
AZIN1
RNF149
GNAI2
RAC1
PDK4
MAP3K2
ZBTB16
MAT2A
DCK
PGLS
RHOG
TSPO
REL
NCKAP1L
GRB2
H2AFY
TMEM219

SPP1
COTL1
FTL
S100A11
FAM49B
HN1
HSD17B11
TAOK3
OSBPL8
SEC11A
MTPN
ITM2B
ATP1B3
DHRS7
MT-ND4L
SH3BGRL
PRMT2
SDCBP
WASF2
AP2S1
RCSD1
CANX
CTSD
ATP6AP2
ITGB2
RHOA
GPNMB
MT-ND1
CYBA
GPX4
ANXA5
MT-ND3
ROCK1
CLU
RGS2
MT-ND5
CELF2
MTDH
ACTR2
EVI2B
TUBA1B
PDZK1IP1
SLC25A5
VAMP8
HSPA1A
SLA
TPT1
FYB
RPS27
ARPC1B
DDX17
NEAT1
SERP1
CD63
RPS4X
RGS1
AC090498.1
FXYD5

XIST
ARPC3
MT-ND4
IL32
CD2
RPL17
CCL5
HLA-A
B2M
CD3D
EEF1B2
MALAT1
MT-CO3
CD52
TXNIP
GZMA
APP
TRAC
MT1E
DUSP2
RPS26
CD3E
RPL23A
SOD2
RPS15A
RPL21
HLA-C
PLIN2
NNMT
CST7
CD69
PPDPF
CD3G
NDUFC1
ETS1
CRYAB
GZMK
NKG7
TRBC2
LCK
TRBC1
INSR
RPL35A
RPS25
ITM2A
CCL4
H3F3B
RPL36
SYNE2
IFITM3
IL7R
TUBA4A
FYN
CD96
FOSB
CD247
DUSP4
GNLY

CD27
KLF6
BTG1
CYB5A
CRIP1
LTB
TPM1
DDX5
IL2RG
SEPW1
ATP1B1
ADIRF
HNRNPH1
ANXA6
HLA-DRB5
ACAP1
CDC42SE1
S100A13
JUNB
HBA2
DSTN
RP11-347P5.1
CD24
TNFAIP3
TIMP1
C12orf57
ANGPTL4
PGK1
EEF1D
JUND
TAGLN2
SMCHD1
TSC22D3
HPCAL1
CDC42SE2
ADGRE5
ITGB1
DNAJA1
UBC
BIRC3
CREM
SUMO1
GYPC
MYL12A
RPS27L
BTG2
APOBEC3C
UBB
RARRES3
ANXA1
MT2A
LYST
PIK3IP1
CLEC2D
RGCC
LINC00152
9-Sep
SOD1

CALM3
PDCD4
KMT2E
YWHAQ
CYTIP
HBB
OCIAD2
BUB3
IER2
SEC61G
CD99
CCNL1
ICAM3
RBM8A
CYCS
PRDX2
RNF19A
ODF2L
LYST
CD8A
CD27
TTN
CD8B
TNFRSF9
CST7
RP11-160E2.6
VCAM1
LAG3
CD200R1
ADTRP
NKG7
GZMK
APOBEC3G
PTCH2
TRAC
CCDC141
DUSP4
CCL5
GZMA
CD2
RNU12
DUSP2
CD3D
CCDC64
CLECL1
PRR5L
FTH1
CCND2
TNIP3
FASLG
RNF19A
ZNF331
MCTP2
CMC1
NR4A2
CD3G
HAVCR2
TOX2

LIMD2
SIT1
ITGA4
TOX
MIR155HG
TRBC2
SNAP47
TIGIT
SLF1
SLFN12L
IFNG
RUNX3
FAM3C
RASGRP1
AC069363.1
HLA-A
ATXN1
BHLHE40
LBH
DTHD1
CRIP1
CH17-373J23.1
ARAP2
IL2RG
NPIPB5
RP11-386I14.4
PTPN7
TRAF5
TSC22D3
AC090498.1
RPL39
TMSB10
PTPRC
SPN
SIRPG
WDR74
DGKH
EOMES
FTL
PDCD1
LCK
SRSF7
CREM
FUT8
TXNIP
SH2D2A
FNBP1
NAB1
VPS37B
TMEM107
PYHIN1
MALAT1
CD3E
CDK6
RPL3
TRAF1
SLC38A1
HLA-F

SUB1
GTPBP8
MSI2
XIST
PRF1
LTB
IL32
FAM118A
CCL4L2
HNRNPLL
ACAP1
RAB27A
TPT1
RPS18
CRTAM
HLA-B
APMAP
CHST12
VIM
RPL13
APOE
FOS
APOBEC3C
UBE2F
RNF213
CCL4
SLA2
WIPF1
CLEC2D
BCL11B
ARHGAP30
PRKCH
PLEK
KIAA1551
S100A10
IKZF3
TBC1D10C
TMSB4X
ANXA1
RPL7
RPL17
CD82
HCST
PAG1
DENND2D
DZIP3
ITGB2
FKBP5
LAT
TXK
RPS8
ITM2A
NCL
RPL10
DUSP1
ETS1
ITGAL
AKNA

RPL8
TMEM2
CORO1A
SMG1
RGS1
ASXL2
TYROBP
LINC00152
LINC01116
PTPN22
EEF1A1
CD247
SYTL2
ARID4B
CCL3
CXCR3
ZBTB1
SYNE2
MBNL1
ICOS
SYNRG
SON
LINC00649
RPS2
ANKRD36C
RAB8B
CST3
CTSW
STK17B
EVL
RPL35
ATRX
PRPF4B
ANKRD36
GPBP1
RPS14
TRBC1
LYZ
PARK7
TRAF3IP3
GGA2
SPTAN1
RPL34
OTULIN
RPL36
GOLGA4
PRRC2C
PRPF38B
DOCK8
NIN
SH3KBP1
ANXA6
PTPN6
RPL18A
GLUL
TIMP1
DDX24
RAC2

ARHGAP9
NFAT5
IRF1
BCLAF1
RPS3A
NBEAL1
HNRNPA1L2
PTMS
TRAT1
1-Sep
SCAPER
HBB
RHOH
SRSF5
RPL11
SLA
CBLB
P2RY10
PNISR
NNMT
RPL32
HMGN1
LCP1
SH2D1A
IL7R
RP5-1171I10.5
FYB
ARGLU1
CEBPD
IL2RB
RGS2
MT-ND6
SYTL3
ILF3
IGFBP7
RPS15
NKTR
DRAP1
CYLD
FCER1G
SURF4
KIF2A
SAMD9L
PSMB9
SHFM1
APOC1
RPS6
RFTN1
SMC4
IRF2
AKAP9
TUBA4A
GPX1
RPL37A
MT-ND4L
NDUFA4L2
TPR
SAMD3

C12orf57
DGKZ
RPL14
KMT2A
GOLGA8B
RARRES3
IGKC
RCAN3
GCC2
CRYAB
RASSF5
DDX17
WNK1
CLDND1
ITM2B
NIPBL
TANK
TERF2IP
BOD1L1
TMEM123
SRRM2
PSMB10
PLIN2
C1QA
ITM2C
ZCCHC11
VPS13C
ASH1L
IFITM3
ITSN2
TAF7
ZBTB38
CCDC12
PSTPIP1
M6PR
IL16
BPTF
PTPRA
NSD1
CDK12
SERPINA1
HNRNPD
SRSF9
JUND
ADIRF
CCDC186
IFI16
HNRNPA3
HERC2
KMT2E
CHD3
PCSK7
TSPYL2
RBM25
RBM23
NELFCD
MT-ND5
SRSF2

SFPQ
MLLT6
BTG2
ASCC3
DDX3X
AC092580.4
ICAM3
PCM1
GNLY
SLFN5
RPL29
NFKBIA
BTN3A2
CCNL1
BLVRA
KLF6
TNFRSF1B
NOP58
USP34
TERF1
DDX39B
IGFBP3
ARL4C
CD84
IFNGR1
ANKRD44
PAFAH1B1
PPP4R3A
PSMA3-AS1
HNRNPM
SAMD9
DSTN
SNRPN
TLN1
RASA2
S100A9
MACF1
KDM5A
RBL2
BAX
G3BP2
DNAJC1
RPLP0
RSRP1
XRN1
GNB2L1
ARPC5L
SMC5
LBR
CNTRL
TAP1
NPC2
CENPC
ZC3H7A
PDE7A
RPS5
GTF2I
RHOB

RPS11
PARVG
EPC1
PARP14
PJA2
MYCBP2
RPL5
BDP1
HBA2
DOCK2
PSAP
ZNF655
ZNF292
WAPL
APOL6
STAT3
SRPRA
GRN
CTSB
TMPO
ANKRD11
JUN
SOD2
WHSC1L1
CEBPB
SENP6
GOLGB1
DDIT4
SETX
HSPB1
S100A11
GPRIN3
LDHA
REST
LGALS3
RPL22
FOSB
PPP1R10
GAPDH
GLIPR1
YBX1
SAT1
C10orf54
PRDX2
EEF2
AIF1
TOMM7
ISG20
ANXA2
PIK3IP1
RGCC
S100A6
HLA-DRA
TAGLN2
ZFAND5
APLP2
RNASET2
EIF3E

RAC1
CITED2
HLA-DRB5
GLTSCR2
RTN4
PPP1R15A
TPI1
FOXP1
TUBB
HSP90AA1
TUBA1B
CYTIP
LAPTM4A
YWHAH
PPP1CB
CD63
GDI2
ZFP36L2
GPX4
ESM1
SPARCL1
IGFBP7
ENPP2
SPARC
GNG11
PLVAP
SLC9A3R2
RAMP2
CD59
IFI27
SDPR
RBP7
IFITM3
IGFBP3
PTPRC
CLEC14A
PLPP1
CXCR4
ZFP36L2
ADGRL4
RGS1
PRSS23
FLT1
MT-CO1
RNASE1
TIMP3
CORO1A
LAPTM5
HCST
CCL5
TSC22D1
MGP
CYBA
EVL
CD44
CD52
BTG1
FYB

PABPC1
CD69
SRSF7
CD37
STK4
RPS29
RPL17
SPRY1
S100A4
LSP1
CD53
CD2
CELF2
SRSF5
RPS27
ITGB2
PNISR
CYTIP
KLF6
SLA
TNFAIP3
RGCC
CRIP2
SARAF
TRAC
OAZ1
RARRES3
LCP1
HMGB2
GSN
STK17B
CD3D
GZMA
MT-ND5
RPSA
NKG7
JUN
HES1
CST7
GPSM3
DUSP2
PNRC1
NBEAL1
CD3E
AC090498.1
ELF1
CD48
MCL1
IDS
DNAJB1
RPL41
ID3
PRRC2C
CCL4
EPAS1
IL2RG
EEF1B2
RP11-347P5.1

MT-CO3
TRBC2
RNF213
RAC2
CAV1
MT-ND2
HSP90AA1
LITAF
PPDPF
UCP2
ZFAS1
MT-ND3
ACAP1
YWHAZ
AKAP13
FKBP1A
EVI2B
AAK1
FNBP1
WIPF1
RPS21
EMP3
FAM49B
SNHG8
RSRP1
GZMK
CIB1
ZFP36
ALOX5AP
PPP1R15A
RNASET2
CCNL1
POLR2J3
STK17A
NFKBIA
INSR
CNN3
FOS
RGS2
MT-ND1
PIK3IP1
PPP2R5C
COTL1
BTG2
RBM25
HNRNPA1
LIMD2
ADGRE5
RPS2
IL32
CTSS
FOSB
ARF6
REL
JUNB
KIAA1551
SAMSN1
RPS15A

PIK3R1
DEK
RPL36A
TRBC1
MT-ND4L
STAT3
TAF7
SH3KBP1
RBMX
CD7
CLEC2D
RGS10
TAF1D
RPL28
MT-CYB
NR4A2
ROCK1
RBPJ
GLIPR1
CTSC
CRIP1
SYTL3
HCLS1
SMAP2
DOCK8
HSPE1
EVI2A
CD3G
LTB
SRRM2
APOBEC3G
BCLAF1
ARID4B
MT-ND4
VMP1
TRAF3IP3
GLUL
BIRC3
LCK
ANXA1
H3F3B
EIF3A
EZR
TYROBP
MYO1F
GLRX
ZEB2
HLA-DQB1
MT2A
RPL10A
PCSK7
SERPINA1
USP15
TYMP
6-Sep
RPL23A
EMB
IKZF1

CD27
SMCHD1
IL7R
PLP2
DHRS7
HLA-DQA1
CD96
RNF149
ATM
HLA-DPB1
CD247
CEBPB
ANKRD44
AKNA
TXNIP
IL10RA
BIN2
BBX
HSPD1
MYCBP2
PRDM1
TRIM22
RCSD1
CCNH
CMC1
FCER1G
MGEA5
CHCHD10
RNF19A
ARID5B
PSMA3-AS1
HLA-DPA1
RPS8
TUBA4A
LDHA
PTMA
PYCARD
ANXA6
GBP2
TSTD1
OCIAD2
FOXP1
MT1X
LYZ
CITED2
YWHAH
PECAM1
APP
AIF1
NNMT
SEPW1
CRYAB
VIM
NDUFA4L2
HBB
SOD2
PSMA1
GNLY

COX7A2
C9orf16
TPM3
EIF2S2
HBA2
APOC1
ATP6V1F
SNRPB2
S100A9
AP2M1
NDUFB10
C1QA
PSMB3
ERH
ATP5I
SUMO1
SLC25A3
HLA-DRB1
TMEM59
WASF2
BRK1
ITM2B
PSMB1
OSTC
MORF4L1
PSMD8
NDUFA11
CD9
PSMB7
RAB7A
IGKC
LSM3
SNRPG
CSTB
TMEM219
NDUFB4
SRP14
PSMD4
PSMA4
NFE2L2
COX6A1
UQCRQ
GYPC
ATP1B3
DBI
A2M
NDUFB7
IFITM2
YWHAQ
PDIA6
CLTA
ROMO1
BSG
MRFAP1
ARL6IP1
PSMB6
2-Sep
SHFM1

PSMC5
FIS1
15-Sep
MRPL51
PPP1CB
RHOB
NDUFC2
COX6C
METTL9
DYNLRB1
ATP5J
GNB2
AP2S1
APOE
CD99
PRDX2
PRDX5
GNG5
GABARAPL2
TMEM50A
ANXA5
TMEM230
RAB2A
CALR
CHMP2A
IFNGR1
RHOA
GSTO1
PPIB
SH3GLB1
EID1
DAD1
GNAS
NDUFC1
LAMP1
CD63
KDELR2
RAB5C
SKP1
VAMP5
COX17
PSMA7
MRPL33
POLR2L
HLA-C
TCEB2
RABAC1
HSP90B1
NDUFA4
SLIRP
GUK1
YWHAE
BCAP31
ANXA2
HLA-DRB5
HLA-DRA
HLA-DPA1
HLA-DQA1

HLA-DPB1
HLA-DQB1
C1QB
HLA-DQA2
C1QC
LYZ
MS4A6A
GPX1
CST3
SGK1
HLA-DMA
GRN
NPC2
HLA-DMB
CTSH
MS4A7
MAFB
CPVL
FAM26F
RNASE6
KLF4
GPNMB
AIF1
CD68
ALDH2
GPR34
MSR1
C1orf162
PHACTR1
KCTD12
FCGR2A
CSF2RA
LST1
PKIB
MS4A4A
PPT1
CXCL16
C3AR1
FGL2
IFI30
CPM
IGSF6
SPI1
OLR1
VSIG4
CYBB
LY86
MNDA
SLC1A3
NCOA4
PLXDC2
IL18
RNF130
CTSZ
CSF1R
ANKRD22
CD163
CLEC4E

CSTA
FCGR1A
TNFSF13B
RP11-1143G9.4
SERPINF1
CD302
SLC7A7
CLEC10A
1-Mar
PLAUR
LY96
CLEC7A
RAB31
FCGR2B
OGFRL1
CD83
AXL
CD86
IRF8
SLC31A2
NLRP3
MFSD1
TBXAS1
LILRB4
CLEC12A
HLA-DQB2
CECR1
IL13RA1
LGALS2
RGS18
FPR3
NCF2
BASP1
TLR2
SRGAP1
PILRA
RAB32
CCDC88A
TM6SF1
LYN
SDS
HCK
PLBD1
ADAP2
LILRB3
ST14
GCLC
SLAMF8
HLA-DOA
KYNU
FCER1G
SAP30
GPR183
C1QA
ATP6V1B2
PAK1
IFNGR2
HLA-DRB1

GNAQ
CD74
TYROBP
MARCKS
LGALS9
CEBPD
C1orf54
SNX10
GAPT
CTSB
CTSS
MPEG1
AP1S2
NUDT16
IFNGR1
LACTB
HEXB
CHPT1
THEMIS2
SRGAP2B
PLD4
CD14
CD300A
TREM2
P2RY13
CAPG
DSE
RGS10
DAB2
FCGRT
GNB4
MEF2C
CFD
FBP1
RBM47
LIMS1
FGR
BTK
SIGLEC10
SHTN1
ACSL1
TYMP
GRINA
C5AR1
SLCO2B1
TCOF1
KCNMA1
LRRC25
FAM105A
RNF13
CLN8
LAIR1
ALCAM
NAMPT
C15orf48
TNFAIP2
H2AFY
SLC8A1

ASAH1
LPCAT2
UBE2E2
PSAP
CREG1
GLUL
ADORA3
BRI3
YWHAH
PSTPIP2
ITGAX
NAGA
UNC93B1
SCPEP1
PYCARD
PABPC4
TPP1
DAPK1
MGAT1
CD9
FNIP2
DRAM2
SLC11A1
LGMN
RGS2
FMNL2
ALOX5
HBEGF
APOC1
SRGAP2
LAT2
ABHD12
FCGR3A
SAT1
NR4A3
PRCP
ATP6V0B
ATP6AP1
ALOX15B
C3
STX7
TKT
ATP1B3
MRPL3
RAC1
ZFAND5
CD4
LGALS3
PICALM
FTH1
ATG3
SKAP2
LIPA
CNPY3
SAMHD1
HMGA1
MAP3K8
FTL

HMOX1
C10orf54
SRGAP2C
IER3
ATF3
VMO1
MEF2A
HEXA
SYNGR2
LAP3
AOAH
AKR1B1
ANXA5
SDCBP
AP2S1
ARRB2
HCLS1
SLC40A1
APOE
COTL1
ARL5A
HERPUD1
COMT
SOAT1
CMTM6
LTA4H
ATP6V1F
SLC43A2
SLC25A5
FABP5
PLEK
ATP6AP2
FAM49B
LPAR6
PLD3
RHOG
PPA1
MIS18BP1
RNH1
AZI2
PTPRE
RTN4
GLIPR1
JAML
REL
VAMP8
CTSL
CEBPB
GDI2
SNX3
RHOB
TUBA1B
SEC11A
MT-CO1
YBX1
GSTO1
GPX4
FKBP5

ACP5
HSPA1A
S100A11
SMAP2
ANXA2
AREG
SH3BGRL
CYBA
CTSC
LAPTM5
ITGB2
MALAT1
ARPC3
CSTB
ARPC5
MT-CO3
NEAT1
ARPC1B
CD63
RHOA
OAZ1
TSPO
FOS
RPS27
MT-ND4
ITM2B
CTSD
MT-ND1
LGALS1
RPS26
IL32
PABPC1
CD2
INSR
SEPP1
S100A9
IGLC2
ETS1
RGS5
CD3E
TRAC
RPL23A
CCL5
CD3G
CD3D
RPS29
SYNE2
RPL31
GZMA
S100A8
APP
HLA-A
IFITM3
ITM2A
LCK
TRBC2
BTG1
CD59

CST7
ENPP2
CD7
CD27
CD69
IGKC
TRBC1
CLEC2D
ATP1B1
ACAP1
CD96
GZMK
LTB
TUBA4A
NDUFC1
CD52
PPDPF
DUSP2
TSC22D3
IGFBP7
FYN
OCIAD2
RNF19A
IL7R
EVL
MGP
HBA2
NKG7
RGCC
STK17A
SYTL3
HNRNPH1
CRIP1
HBB
ODF2L
GPX3
BIRC3
SEPW1
DNAJB1
DSTN
SEC61G
ADIRF
JUND
SPARC
CCL4
IVNS1ABP
GNLY
IGFBP3
KIAA1551
IL2RG
ROMO1
RARRES3
NDUFA4L2
BBOX1
CD24
MALAT1
TMSB4X
CRYAB

KRT18
FXYD2
GAPDH
CMBL
B2M
BHMT
CYB5A
SRGN
BTG1
MT-CO1
LDHA
NAT8
CXCL14
ADIRF
MT-ND3
NEAT1
BNIP3
CXCR4
FTH1
HLA-E
ZFP36L2
HLA-B
PTPRC
KRT8
DDX5
ANXA4
MT-CO2
HLA-C
MT-ND2
MT-ATP6
RGS1
S100A4
ALDOA
MT-ND4
FTL
MT-ND1
MT-CO3
MT-CYB
TXNIP
ARHGDIB
ENO1
PDZK1IP1
LAPTM5
EVL
CORO1A
LGALS2
ARPC2
SRSF7
N4BP2L2
CD44
HCST
PNISR
FYB
NNMT
CD52
MT-ND5
CD53
SH3BGRL3

STK4
CCL5
CD37
JUNB
CD69
CD2
JUN
GMFG
FXYD5
PRPF38B
SON
MCL1
MIF
TNFAIP3
MGST1
ACTB
CYTIP
KLF6
AKAP13
RBM39
ANXA1
ARGLU1
LCP1
GATM
POLR2J3
HLA-F
TMEM176A
CELF2
TPI1
ZFP36
CD3D
CCND1
XIST
NUPR1
ELF1
EEF1A1
LSP1
TMEM176B
STK17B
CFL1
ITGB2
LRRFIP1
PTMA
SLA
ARPC1B
RSRP1
IL2RG
CD3E
TRAC
ALDH1A1
GPSM3
GZMA
DUSP2
DDX24
CCNL1
MYL12A
PRRC2C
IFI16

CYBA
SRSF5
RP11-347P5.1
RPL7
ITGB1
RAC2
FOSB
DDX3X
VAMP2
WIPF1
TRBC2
FNBP1
RNF213
PFN1
TPT1
RPS28
MBNL1
AAK1
CCL4
PLIN2
TMA7
CD48
RHOA
ANKRD12
NKTR
CLEC2B
PPP2R5C
HMGB2
7-Sep
FKBP5
BTG2
RPS6
H3F3B
PEBP1
KMT2E
CST7
ATRX
RPL3
IDS
HLA-DPA1
YWHAZ
CD99
SRSF2
FUS
DNAJB1
YWHAB
HNRNPA2B1
ARL6IP5
ARID4B
RPLP0
WSB1
PPP1R15A
DDX17
HLA-A
KIAA1551
GIMAP4
EVI2B
SMCHD1

REL
TRA2B
SH3KBP1
NKG7
STK17A
ACTR2
PIK3IP1
FOS
TPM3
RBM25
ACAP1
PCSK7
ISG20
SAMSN1
ROCK1
LIMD2
ETS1
HCLS1
CTSS
SYNE2
CKLF
MT-ND4L
SP100
UCP2
COTL1
CFLAR
HLA-DPB1
FAM49B
IQGAP1
ARF6
SARAF
ALOX5AP
ZFP36L1
PPP1R2
GPBP1
AKAP9
GZMK
ITM2A
RGS2
CCND3
CAPZB
SCAF11
IRF1
FAM133B
GLIPR1
PRDM1
CD7
BCLAF1
TERF2IP
TNRC6B
DOCK8
GIMAP7
JAK1
STAT3
LCK
CLEC2D
GCC2
RPS4X

CD3G
CLK1
MSN
ARPC5
PABPC1
COX7C
CREM
RARRES2
RGS10
6-Sep
EMB
LGALS1
C9orf16
PIK3R1
SMAP2
CEBPB
RAP1B
MYO1F
RPS8
PDCD4
DAB2
HLA-DRB1
LTB
ADGRE5
TRBC1
EVI2A
GNAS
SYTL3
RBPJ
VMP1
MYH9
TSC22D3
HSPB1
SF3B1
TXN
IL7R
CTSC
SF1
RPL31
MYCBP2
FOXP1
SRRM2
LITAF
CARD16
ATM
RPL36
NR4A2
ANKRD44
HLA-DQB1
PRKCH
PPP1R12A
IFITM2
APOBEC3G
MACF1
DEK
RPS15
IER2
ODF2L

AKNA
CD27
IL10RA
CD74
NACA
ACTR3
RGCC
TAPBP
IKZF1
EPC1
SFPQ
CMC1
BIN2
RNF19A
RCSD1
PDK4
MGEA5
NFKBIA
GABPB1
PTP4A2
GSTP1
TLN1
LINC00152
ABRACL
S100A6
YPEL5
HSP90AA1
SLC2A3
CD96
PSMA3-AS1
TAOK3
HLA-DQA1
PYCARD
ICAM3
PNN
ARHGEF1
HP1BP3
CCNH
IFNGR1
WNK1
ORMDL1
CDC42SE2
C10orf54
JMJD1C
USP15
TRAF3IP3
RNPS1
TRIM22
RPL35
TYROBP
VIM
PSMB10
BTN3A2
RPL11
S100A10
PKM
TUBA1A
FCER1G

RPS5
BRD2
DDX6
CASP4
C9orf142
HNRNPH1
9-Sep
NDUFC1
RPL37
RPL8
RHOG
PLP2
GYPC
RPL26
FYN
AES
PSAP
BIRC3
TPM1
RPL19
HLA-DMA
GPX4
RHOB
RPS25
SAT1
AIF1
HLA-DRB5
GNLY
ESD
SERPINA1
LYZ
NPM1
HLA-DRA
RPS7
C1QA
RPL22
HBB
OCIAD2
ACAA2
RPL5
RPL24
RPL38
GPX3
CST3
UQCRB
HBA2
RPS11
PPDPF
RPL23
UBC
TMEM59
LAMTOR5
RPL36A
GNB2L1
TMBIM6
UQCRQ
PRDX1
PFDN5

USMG5
DSTN
PRDX6
SPP1
MPC2
OCIAD1
IGKC
CUTA
CNDP2
COX17
S100A9
PSMA4
ATOX1
ST13
PGAM1
CHCHD10
SEC62
APOC1
C19orf53
ZNHIT1
SLC25A5
NDUFB9
COX7A2L
PSMB6
ENPP2
SPARCL1
FLT1
ESM1
INSR
PLVAP
TIMP3
SPARC
A2M
IGFBP3
VWF
GSN
EPAS1
PLPP1
STC1
NOTCH4
IGFBP7
PRSS23
ADGRL4
CLEC14A
PECAM1
EDNRB
HSPG2
COL4A1
APP
SPRY1
RBP7
SDPR
PLPP3
CD59
BTNL9
MGP
RNASE1
INHBB

SLC9A3R2
AQP1
RAMP2
TCF4
GNG11
ENG
EFNB2
ANGPT2
CLDN5
TSC22D1
IFI27
ADGRF5
ITGA6
ARHGAP29
SPTBN1
COL4A2
PODXL
HES1
BCAM
PTPRB
FAM84A
EGFL7
ESAM
NFIB
GJA1
HYAL2
LIFR
PDGFD
RAMP3
F8
CALCRL
SEC14L1
TM4SF1
CDH13
APOLD1
KCNE3
CD93
CRIP2
EMCN
GRB10
EMP1
TMEM204
LDB2
FSTL1
PCAT19
CD34
ITGA1
EFNA1
NRP1
CDH5
FCN3
TMEM88
UACA
KDR
VWA1
CA2
MMRN2
F2RL3

ID3
WWTR1
DOCK9
S1PR1
CAV1
PTRF
OSBPL1A
FILIP1
MTUS1
CX3CL1
TSPAN13
SMTN
IGFBP4
SORBS2
DLL4
HTRA1
CRIM1
COL8A1
MAGI1
NTN4
LAMC1
JAG1
COL15A1
TMEM47
TSPAN7
SLCO2A1
IDO1
RAPGEF5
NPDC1
TMEM150C
CNN3
PINK1
UNC5B
TJP1
MYO6
CYYR1
S100A16
PALMD
TM4SF18
TMTC1
LAMA4
PLXND1
PPFIBP1
PTPRK
FAM167B
PLK2
NOSTRIN
COX7A1
TIE1
CXorf36
ANKRD26
THSD7A
FZD4
SULF1
NID1
GAS6
HEY1
TNS2

DOCK6
LIMCH1
DKK3
ECSCR
MATN2
ARL15
RPS6KA2
PTPRG
EMP2
ROBO4
FAM107A
KCNN3
HES4
FGFR1
GALNT15
SASH1
ANGPTL2
LNX1
ACVRL1
LAMB2
ECE1
MECOM
MYCT1
GALNT18
CAV2
ERG
TMEM255B
LRRC8A
PCDH17
SOX18
JAG2
PTPN14
DYSF
ITIH5
NCKAP1
TMEM233
GPIHBP1
LAMA5
MPZL2
PREX2
RBMS3
NUAK1
SCARF1
PXDN
LAMB1
IL3RA
DPYSL3
BTBD3
RASIP1
PDZD2
HOXD9
CMTM8
MPDZ
PLXNA2
ACE
EVA1B
FOLH1
IPO11

HIF3A
LRRC32
PLS3
RAPGEF4
PLEKHG1
JAM3
GAS2L1
TANC1
PIK3R3
ABCG2
ITGB5
MYO10
AFAP1L1
MCF2L
SHE
HECW2
LIMS2
EML1
SEMA3F
FGD5
RAI14
BOC
RAPGEF3
HOXD8
PLD1
FLT4
GPR4
SPRY4
COL18A1
CDC42BPA
ZNF704
ATOH8
GRAMD3
JUP
SERPINE1
LAYN
ACKR3
FNBP1L
CALD1
MYO1B
FKBP9
MCAM
TNFRSF4
PLLP
PVRL2
EHD4
RASGRP3
MGLL
TACC1
ITPRIP
NOTCH1
SYNPO
MGST2
MEF2C
HEG1
RPGR
RAB13
TSPAN12

GNAI1
SHROOM4
SERPINI1
FERMT2
PTPRM
PPIC
ITGA5
MLLT4
EPB41L4A
A4GALT
KIAA1147
IFITM3
TSPAN15
TTC28
ARHGEF10
FRY
RAPGEF2
SIPA1L2
TGM2
MAP3K6
CYR61
FAM101B
BAIAP2
APLP2
CDC42EP1
LIMA1
SNCG
FAM198B
PTP4A3
CD9
ITGB1
ARHGEF12
PON2
NGFRAP1
TIMP2
SEPW1
SCARB1
RDX
OAZ2
TGFBR2
IL4R
THBS1
LMO2
YES1
IVNS1ABP
CSGALNACT1
ACACB
TMCC3
TNFAIP8L1
PKIG
NETO2
YWHAH
F2R
FNDC3B
LMCD1
REEP3
DGKH
RBMS2

STOM
ARHGEF7
GFOD2
CTGF
PROS1
FSCN1
ICAM2
PDLIM1
CTTN
ADIPOR2
RGCC
RASAL2
VAMP5
NFIA
CDC42BPB
CDC42EP3
MLEC
TNFAIP1
BCAP29
RPL41
MYO1C
S100A13
SERPINB6
CLIC4
TINAGL1
TJP2
DEPTOR
TP53I11
KIAA0355
RPS27
ID1
HDAC7
WBP5
SWAP70
ZEB1
MARCKSL1
GOLIM4
PGM2L1
LHFP
CYB5R3
SERPINH1
TMEM184B
SH2D3C
SEC62
KLHL5
LXN
ELK3
LEPROT
EPHX1
PRKCDBP
PHACTR2
RPS2
SH3D19
RPL10
FAM213A
HEBP1
IL6ST
AKT3

NEAT1
SH3BP5
BMPR2
RPL13
GIMAP8
SLC44A2
PTK2
CALU
ADD1
RPS29
RPL13A
RPL28
RPS15A
ABLIM1
PTTG1IP
CD81
THEM6
RPL23A
CCPG1
CPNE2
CDA
NCOA7
MED13L
DHRS3
FKBP1A
CCDC85B
B3GNT5
RPS18
TMCO3
HIPK2
KLF9
RFK
CTTNBP2NL
RPLP2
RPL39
XAF1
CD46
GBP4
FN1
DSTN
EHD2
RHOB
ASAP1
C10orf10
RPL21
RPS19
RPL26
PIK3C2A
RPS12
RHOC
EEF1A1
ADM
CTSL
RPS3
RCN2
CAPZA2
LUZP1
NEDD9

MAP4
CD151
RPL27A
EXOC6
YBX3
RPL18A
MTRNR2L8
RPS27A
TSPAN4
PAM
RPLP1
PTPN12
RPL31
RABGAP1
GNAS
RIN2
SLC27A3
ITM2B
HSP90B1
HLA-E
DUSP6
ARHGAP5
RPS14
ATXN3
RPL19
RPL37
CTNNA1
WARS
RPL30
10-Sep
CALCOCO2
LMNA
AAMDC
GFOD1
PPM1F
SERINC3
CTNND1
ROCK2
CTNNB1
TNS1
PTPRC
ARHGAP18
PLOD1
FAM43A
NASP
RPL12
GINM1
IFITM2
SNRK
RPS24
MAPK3
RPS3A
ARRDC2
TCEAL4
RPL10A
XIST
RPS21
RPL11

RPS25
RPS15
RPL6
RGL2
ADGRG1
VGLL4
PEA15
INPP1
ITPRIPL2
KTN1
FRMD8
RPL34
RGS3
WDR60
CYSTM1
RPL32
SLK
FYN
RPS16
CCDC50
RPS8
COPS8
NPTN
HMBOX1
RPL35A
SRP14
SLC1A5
RPS23
CDC37
TRIOBP
CD40
CRK
MAP4K4
RPSA
ACTN4
SYPL1
RPS6
GIMAP7
RGS1
MXD4
MPZL1
RP11-553L6.5
CXCR4
NAA38
NUCB2
FAU
ABL2
GIMAP6
BST2
EDEM3
CD63
LRCH1
EPS8
RPS17
RPS20
VIM
RPL35
RCN1

TBC1D15
PDLIM5
RPS4X
FEZ2
RAC1
TRIM56
RRBP1
POMP
RPL14
NDRG2
DYNC1LI2
CORO1A
FAM214A
RPS7
ICA1
RPL3
TAX1BP3
DHRS7B
SLC44A1
RSU1
GRN
IFI6
ZBTB16
RPL38
PRDX1
LAMP1
FNIP2
RPS28
LAPTM4A
QKI
ZFP36L2
TMEM245
RPL15
ZFYVE21
RPS26
RBMS1
BTG1
MTRNR2L12
GNAI2
ASPH
MYL12B
HCST
RPS13
ABCG1
TNFSF10
RPL7
RNF115
GPX3
PXN
LRMP
RPL36
MALAT1
BCAP31
PLEKHA1
HSPB1
JMJD1C
RALB
CD44

RPL9
SH3GLB1
DPYSL2
S100A4
RPLP0
TMOD3
ARL2
TMSB4X
CLCN3
PARP14
RPS10
CLSTN1
CEP68
SECISBP2L
PARVB
TUG1
RPL18
PSMB5
UBE2J1
VIMP
RTN4
FCGRT
USP34
LPP
ZFYVE16
FYB
SDF4
RPL36A
MIR4435-2HG
CAMTA1
DNAJC3
RAB11A
RPS9
ZBTB20
ERLEC1
TMEM30A
AP1S2
DOCK4
CD2
CYTIP
QSOX1
IFNGR1
ELMO1
ATOX1
GABARAPL2
NFIC
NDUFA12
RPL27
CD69
RAB6A
CD37
EID1
EIF4G3
C12orf57
UBA52
CELF2
PTMS
PML

PICALM
RPS5
TMEM50B
APOL3
ADAM9
LINC00657
ARGLU1
SCARB2
ABI2
RPL37A
CLTC
CD52
RPL7A
TSPAN14
ADAM10
RPL29
RABAC1
C5orf24
FDPS
LSP1
RPL5
AHNAK
2-Sep
MAGED2
GIMAP4
RPL8
MAGT1
UXS1
TRAC
ZFHX3
CALR
UTRN
AES
MACF1
NCOA3
SLC2A3
LAPTM5
CCL5
TM9SF2
KLF3
CHMP3
GALNT1
RPL4
HNRNPA1
MIDN
MBNL2
IFNAR1
CD3D
TPT1
GNB2L1
RPL24
TMEM50A
CPD
SLA
HACD3
VPS37A
BSG
ACTB

SLC39A10
SERINC1
PKN2
HLA-B
LCP1
PPIB
GZMA
RPS11
ADD3
TMEM59
DYNC1I2
SLIRP
TMEM165
BAZ2B
ITGB2
STK4
RFC1
MRPL33
RILPL2
RAPGEF1
FMNL3
CD48
COX17
CD3E
IL2RG
MT-ATP6
CAPN2
MT-CYB
C1orf54
KLF7
UVRAG
GUK1
ENTPD1
DYNLL1
EIF1
NKTR
PRMT1
BNIP2
NCK1
CYBA
RP11-347P5.1
RPL23
AFF1
COX5B
GNAQ
TAGLN2
EFCAB14
DENND3
DAD1
GNB1
PDIA6
ACAP1
ITGAV
IFITM1
PCMTD1
TM9SF3
RAC2
LMAN1

DUSP2
ARF1
KCNQ1OT1
CST7
OAZ1
PRKAR1A
RAB2A
EVI2B
DDIT4
CAPNS1
PDIA3
CPM
ATP1B3
TRBC2
GIMAP1
AFF4
EVL
WASF2
GNB2
TMEM109
FRYL
STK17B
NFE2L2
GTF2I
RAB5C
CALM1
CD99
SPAG9
UCP2
CD3G
PABPC1
OS9
RARRES3
LITAF
RPL17
ANKRD11
NPM1
ISG15
SYNE2
CD53
CANX
RSF1
EVI2A
TNFAIP3
TRBC1
SAMSN1
FTH1
GPSM3
TRAM1
ETS2
NKG7
CCL4
EMB
LIMD2
CD7
STK17A
SYTL3
NR4A1

LTB
ALOX5AP
RGS10
MT2A
FABP5
TRAF3IP3
EEF1B2
GLIPR1
COTL1
IL32
DOCK8
BIRC3
BIN2
IKZF1
MSN
NACA
EEF1D
LCK
PPDPF
GZMK
BTG2
RGS2
RPL22
MYO1F
IL10RA
ADGRE5
PNRC1
IL7R
PIK3IP1
FAM49B
APOBEC3G
CD96
RPL36AL
EMP3
SARAF
DNAJB1
TSTD1
HMGB2
SH3BGRL3
MYH9
6-Sep
CLEC2D
PIK3R1
H3F3B
CD27
FTL
PFDN5
LY6E
NBEAL1
CRIP1
AKNA
ZFAS1
HLA-DQA1
ATP5G2
AC090498.1
RNASET2
LDHB
TOMM7

COX7C
PPP2R5C
RCSD1
ICAM3
SRSF7
SLC25A6
TAF7
SH3KBP1
SNHG8
SMAP2
BTF3
TUBA4A
ANXA1
MT1X
RGS5
WIPF1
TYROBP
GLUL
GAPDH
HMGN2
NAP1L1
NR4A2
AAK1
FCER1G
SERPINA1
CITED2
DUSP1
JUN
HCLS1
ATP5L
LEPROTL1
ANKRD44
PRPF38B
TXNIP
CCNH
ANXA6
SRGN
CST3
EEF2
EZR
COMMD6
ENO1
FNBP1
IDS
HLA-DQB1
CMC1
EIF3H
PLP2
HLA-DPB1
FOS
JUNB
NNMT
HLA-DRB1
PRR13
MCL1
ZNF90
MZT2A
GLRX

GLTSCR2
C9orf142
AIF1
EIF3E
REL
YPEL5
NPC2
RPL22L1
ADIRF
EIF3M
EIF3L
EIF4A2
LYZ
VAMP8
ARID5B
SNRPD2
ZFP36
ARF6
OCIAD2
CYCS
CIB1
HIGD2A
TYMP
RTKN2
FOXP3
AC002331.1
TIGIT
TBC1D4
TNFRSF18
IL2RA
BATF
CTLA4
LAIR2
AC133644.2
PMAIP1
IL32
LTB
ICA1
SLAMF1
TNFRSF4
HPGD
CARD16
ICOS
TRAC
BTG3
CD27
S100A4
CORO1B
RP11-347P5.1
SELL
UGP2
ARID5B
STAM
SAMSN1
CD79B
BIRC3
ENTPD1
CD2

TNFRSF9
GBP2
RGS1
UCP2
PHTF2
CLEC2D
CTSC
SPOCK2
RP11-138A9.1
TSC22D3
P2RY10
CD3D
NCK2
IL2RG
AC016831.7
PBXIP1
ISG20
DNPH1
TRBC2
PRDM1
PIK3IP1
PHLDA1
SKAP1
CYTIP
DUSP4
FYB
RPS26
PIM2
ARPC1B
BTG1
LINC00152
HLA-A
LCK
SIRPG
ARHGDIB
RP11-138A9.2
ACTB
ANXA1
CCL5
KLF6
CCNG2
GATA3
TNFAIP3
GZMA
DUSP10
STAT3
MIR4435-2HG
USP15
IPCEF1
GADD45A
CD3E
ACP5
RORA
GCHFR
CD7
ICAM3
ARL6IP5
RAC2

FAM129A
NKG7
GBP5
CCL4
STK17B
ITM2A
PTPRC
GMFG
CCNI
CALM3
PHACTR2
GLRX
TRBC1
SRSF7
FCMR
CYCS
C9orf16
CD44
LAT
RASGRP1
ZBTB38
WHSC1L1
GZMK
TNFRSF1B
IQGAP1
PELI1
ANAPC16
UBE2D2
CD53
HSPA8
ALOX5AP
CREB3L2
RHOH
CASP1
TYROBP
CLPP
HTATIP2
BUB3
EVI2A
CD63
DDX24
6-Sep
GLUL
EPSTI1
SLA
CHST11
CLIP1
RSBN1
RAP1A
CEBPD
GALM
RHBDD2
IL10RA
AKIRIN2
GSTP1
NABP1
GIMAP4
RAB9A

GIMAP7
ZNF292
PPM1G
RNF145
CST3
CDV3
FTH1
FCER1G
NR3C1
DEF6
SERPINA1
CLEC2B
IFNGR1
WNK1
IFITM3
BCL2
ZC3H7A
RGS2
DSTN
RHOB
CRYAB
RNASET2
CEBPB
CTSB
HSD17B11
GRN
FTL
CMC1
CANX
SOD2
LITAF
AIF1
ANXA2
LYZ
NNMT
NDUFA4L2
CD99
ANXA5
SDCBP
FCN1
LST1
CSTA
IL1B
VCAN
S100A12
LINC01272
MS4A7
CFP
BCL2A1
SPI1
SLC11A1
LILRB2
C5AR1
CLEC12A
CLEC7A
NCF2
LILRA5
MPEG1

APOBEC3A
CTB-61M7.2
FPR2
C19orf38
CD300E
CYP1B1
KCNE1
LILRA1
CFD
EREG
GPBAR1
FPR1
PILRA
HCK
AQP9
FGR
KLF4
PLAUR
CLEC4E
RGS18
AIF1
CPPED1
NAMPT
IFI30
CYBB
SLC7A7
IRAK3
AP1S2
FCER1G
MAFB
S100A9
SLC31A2
CTSS
LILRB3
VNN1
ADGRE2
LYN
CLEC4A
TKT
C1orf162
CEACAM4
1-Mar
SULT1A1
STX11
MTSS1
GCA
ABHD5
TREM1
RP6-159A1.4
ARL4A
FAM26F
LRRC25
MNDA
KYNU
LYZ
DUSP6
RNF144B
RP11-1143G9.4

S100A8
TYROBP
STXBP2
CD55
SERPINA1
TCF7L2
TNFSF13B
ASAH1
MXD1
CD68
IRS2
FAM49A
ASGR1
CD302
ZEB2
LGALS2
MTMR11
CD36
POU2F2
COTL1
RAB31
LILRA2
FGL2
LRRK2
ARHGEF40
NUDT16
PHACTR1
THBS1
C9orf72
NAAA
SAT1
RGS2
ETS2
SCIMP
LINC00936
CEBPB
C10orf54
FTH1
PYCARD
MTPN
LGALS9
LY96
CD163
TYMP
ZFAND5
FCGR2A
BRI3
CNIH4
CEBPD
RNF130
FTL
PSAP
FAM110A
H3F3A
PRAM1
LTA4H
CST3
MS4A4A

PTPRE
HBEGF
UPP1
GLUL
PGD
IGSF6
AGTRAP
TIMP1
NFKBIZ
CD83
MT-CO1
RIPK2
RILPL2
NUP214
KCTD12
SNX10
GRINA
CSF3R
FAM45A
ITGAX
ACSL1
WARS
S100A6
CSGALNACT2
CMTM6
NEAT1
OAZ1
ZNF281
TLR2
NLRP3
FGD2
MAPKAPK3
CPVL
CD300A
ATP6V1B2
SERPINB1
S100A4
ATG3
RNF149
RNASE6
SMAP2
TRIB1
PRELID1
TNFAIP2
RASGEF1B
NPC2
MEGF9
CASP1
HLA-DRB5
THEMIS2
IL1R2
LILRB1
PTEN
CALM2
DDX21
CARD16
CD14
EVI2B

TLR4
CD86
HLA-DQA2
P2RY13
PLBD1
S100A11
SLC43A2
SAMSN1
THBD
TBXAS1
UBE2R2
CTSH
FAM65B
H2AFY
UBE2D1
SOD2
JAML
SRGN
BLVRB
BCAT1
BID
MT-CO2
SMCO4
TNFRSF1B
CXCL16
SERP1
ARRB2
RNF13
CXCL8
SCPEP1
RHOG
MS4A6A
CHPT1
CXorf21
PTPN6
TALDO1
GAPT
SIGLEC10
SELL
TMEM167A
VAPA
PRKCB
AKIRIN2
HLA-DRA
AZI2
ARPC3
RHOB
VNN2
VMP1
PKP2
SNX2
IER3
PELI1
EMP3
SLC25A37
IFNGR2
TSPO
CD48

GRN
GDI2
LGALS3
BTK
DAPK1
APLP2
VAMP5
ALOX5
NINJ1
TES
SKAP2
PGLS
CECR1
CUX1
ANXA5
FOS
RPS9
CYBA
IRF8
HSD17B11
C3AR1
LACTB
NBPF14
GSTP1
GPX1
MAP3K8
SLC36A4
AREG
DRAM2
ARPC5
MGAT1
ATP6V0D1
IFNGR1
VPS35
SPG21
PLEK
KLF2
FCGR3A
PABPC1
VASP
GNAQ
MBD2
HSBP1
FBP1
SAP30
GNAI2
RAB10
STK17B
TCIRG1
MT-CO3
ATP6V0B
LY86
CCDC88A
SNX3
GLIPR1
HMOX1
NUMB
WDR26

RTN4
MFSD1
ARPC1B
METTL7A
ZNF706
SNX18
BACH1
SDCBP
SRGAP2B
CNPY3
ATP1B3
DICER1
CAPNS1
ACTB
CTSB
CHP1
LIMS1
HN1
FKBP5
SELPLG
MPHOSPH6
PAG1
C7orf73
ACTR2
PABPC4
ZBTB16
HACD4
RHOA
SLC25A6
XRN2
DUSP1
C4orf48
C14orf2
RGS19
KIAA1033
IFITM2
SH3BGRL
PCBP1
FGD4
ARPC2
PLAC8
GSTO1
ZCCHC6
SH3BGRL3
GMFG
FNIP2
LGALS1
GNG5
RAP1A
OAS1
WAC-AS1
AHR
SOCS3
LAMTOR4
LRRFIP1
HCLS1
YBX1
AP2S1

NR4A1
ANXA2
PFDN5
EIF1
MYO1G
BASP1
NAP1L1
MCL1
GIMAP2
ABRACL
ABI3
BLOC1S1
SLC8A1
MT-ND4
IL32
RGS1
HLA-A
MT-ATP6
CHMP1B
SLC25A5
CD37
NACA
VAMP8
EVI2A
CD3D
ITGB2
CTSC
RPL31
SLC35E3
CD52
LAPTM5
LCP1
CCNI
CD2
FYB
CYTIP
MT-ND1
RPL23A
CD3E
GZMA
CCL5
TRAC
CST7
TRBC2
RPS29
RPS3
SYNE2
RPL3
CD69
CD3G
ETS1
IGHA1
LCK
ITM2A
TRBC1
MALAT1
CLEC2D
HSPA1A

CD7
RPS26
ACAP1
STK17A
GZMK
OCIAD2
PIK3IP1
RPSA
CD96
CD27
RPLP0
DUSP2
PRDM1
CCL4
IL7R
HMGB1
IGKC
PRKCH
C12orf57
PIK3R1
EVL
GYPC
SYTL3
AAK1
CD99
C1QA
ODF2L
KIAA1551
RARRES3
IL2RG
JUN
SRSF7
HBB
LDHB
APOBEC3G
NKG7
RNF19A
CMC1
RGCC
EMB
HILPDA
EGLN3
BNIP3
LINC01320
CCND1
RARRES2
VEGFA
ERRFI1
ADM
CA9
NUPR1
NOL3
MGST1
CP
PLOD2
LOX
CA12
CMBL

KRT8
EDN1
TFPI
IRX3
NPTX2
TCEA3
AK4
ADSSL1
P3H2
MAOB
BHMT
FSTL3
EMX2
UGT2B7
ANG
CDK18
ENPP3
FBXO17
RP11-14N7.2
BICC1
RP1-60O19.1
KISS1R
ZNF395
RP11-11N9.4
PCOLCE2
FABP6
P4HA2
CDCA2
EDIL3
MAPT
SPINK13
GALNT14
CDH2
STC2
BACE2
ARSE
BHMT2
SLC22A2
FMO2
SPAG4
RASSF6
RP11-798K3.2
CLEC18A
SLC2A1
SOX4
ANGPTL4
CXCL14
CD24
SLC3A1
FAM13A
ASS1
PROS1
CKB
NR1H4
RBPMS
HSF4
MT3
C14orf105

TMEM91
CRNDE
KRT18
ENPEP
SLC39A14
BHLHE41
SEZ6L2
SEPP1
ALDOC
PFKP
DPCD
GATM
TMEM176A
SSPN
SLC37A4
FOLR1
AEBP1
MGLL
PHLDA3
P4HA1
SDC2
FHL1
WFDC2
ALDH1A1
NMB
EGFR
C1S
ATP1B1
EFEMP2
TMEM45A
DIXDC1
CAV1
C1orf186
C19orf33
NDRG1
SERINC2
INSIG2
PDLIM1
CAV2
WDR34
C1R
PMP22
HSD3B7
TMEM176B
CALD1
GPRC5C
S100A16
MYL6B
RRAD
MIR4458HG
SNHG19
PDZK1IP1
AKR1C3
PRDX4
ENO2
CLU
NDUFA4L2
CCL28

KCTD3
TMEM37
NT5C3B
PGRMC1
CFAP36
S100A13
FAM213A
NNMT
CRYAB
YBX3
EIF4EBP1
PDK4
C10orf10
FARP1
CYB5A
NEK6
TRIP6
PDZK1
EPB41L4A-AS1
SMPDL3A
PRKCDBP
CNN3
SPINT2
FAIM
SLC16A3
TMEM27
HSBP1L1
CRYZ
HNMT
PLEKHA1
IMPA2
DOC2A
HPCAL1
PMM1
CD151
RP11-798M19.6
ADIRF
MAP3K13
GPX3
PTGR1
TGM2
DARS
FAM134B
RPF2
GADD45A
BLVRB
RAB13
MT1F
SHMT1
MXI1
UGDH
FAHD1
MT1X
TSPAN12
GATM-AS1
SCCPDH
RASSF4
BNIP3L

KIF1BP
CCNB1IP1
DEFB1
SMIM3
TMSB4X
SLC25A37
CES4A
VKORC1
SPP1
POLD2
ANXA4
PLIN2
TPM1
C5orf46
B2M
SPATS2L
PPP1R16A
EGR1
SERPING1
KHK
ENO1
STC1
LDHA
BOD1
RCN1
PIGP
BEX2
ESD
AK2
PLA2G16
IGFBP4
C16orf13
GCSH
RBP5
MIF
FXYD2
ABCA1
POLR2H
ALDH3A2
SLC39A1
LGALS3
RHOB
TSC22D1
TMEM205
IGFBP3
BCKDK
TMCC1
PGRMC2
ERO1A
UBR4
CYR61
MRPS18B
ARPC1A
RAB34
PTTG1IP
DAB2
RNASET2
FAM162A

LINC00998
SOCS3
MORF4L2
CISD1
LRRC41
HCFC1R1
CTHRC1
TNIP1
WRB
PGK1
SOD2
ZFAS1
CDKN1A
ACTN1
PCBD1
ERGIC1
VIM
ALDOA
GAPDH
CERS2
CRYL1
POLR2I
C5orf15
RNF187
TPI1
WDR60
NFIA
HLA-B
RPLP0
NDUFC1
VDAC1
PKM
SERPINE2
LAPTM4A
SNHG8
RBM47
ARL1
NFIC
KDSR
CYB5R3
RPS5
AIG1
TIPIN
FLOT1
MT2A
PEBP1
ODF3B
SNX10
MT1E
IFITM3
NDUFS4
SYPL1
APEX1
NDUFB4
MRPS33
EBPL
MGST3
SELM

CNDP2
HES1
SEC61G
RPL36
DDIT4
MRPL23
PDCD5
NDUFA5
RPS8
PCNP
ST13
HSPB1
FUNDC2
SNRPE
SRGN
TCEAL4
STOML2
OCIAD2
MYC
CEBPD
GPI
YWHAE
MMP24-AS1
HLA-C
ZBTB20
VDAC2
TNFSF10
RPL7
TOMM20
S100A10
PHPT1
TMEM123
EIF4B
RPS27L
PERP
PLSCR1
PSMC1
CFDP1
NENF
ACTB
RPL24
ACAA2
KCNQ1OT1
DUSP23
CAMLG
UBC
FTH1
PRDX6
RPL9
TUBB
SERPINE1
AQP3
RAD23A
ZFAND5
ATF3
TMEM14C
SLC17A3
NPM1

TBCA
GSTP1
FOXO3
NELFE
RSL1D1
CHCHD10
PGF
ATF4
RSL24D1
RPL18
RPS19BP1
RPL18A
RPL5
RPL3
IGHA1
BTF3
FBL
HSP90AB1
ZNF90
MZT2A
PTPRC
RPS4X
MRFAP1
SERPINA1
MZT2B
GLRX
EIF3E
HLA-A
RPL8
NACA
RPL41
LSM5
RPL11
PGAM1
RPL10
ARHGDIB
RPL22
RPS6
P4HB
DUSP1
TIPARP
EEF1A1
NDUFS5
BTG1
CCT4
MALAT1
EEF2
ANXA2
RTN4
TXN
RPS2
CEBPB
RPL36A
TAF1D
S100A4
RPS13
ATP5E
CXCR4

HLA-E
TPT1
RPS28
CD63
ZFP36L2
RPS18
TMSB10
RGS1
GLTSCR2
RPL35A
RPL13
RPL38
RPL34
CORO1A
RPL37
HCST
HSD17B7
IER2
LAPTM5
UQCRH
EZR
CFL1
CITED2
GMFG
RPS21
FYB
CD74
COX7C
SLC25A6
RPL31
SH3BGRL3
RPS25
ARPC2
RPL6
DDX5
RPL35
CELF2
PFN1
CCNI
CD37
CD53
IGKC
HLA-DPB1
PNRC1
CD2
EVL
GADD45B
HNRNPA1
RP11-347P5.1
CD44
CYBA
TMA7
MYL12A
SLA
STK4
IGFBP7
ZFP36L1
CYTIP

TGFBI
TRAC
CD3E
CD69
CD48
CCL5
CD3D
LSP1
GPSM3
IL2RG
CD52
HLA-DPA1
LCP1
RAC2
ITGB2
FXYD5
ARPC1B
STK17B
GZMA
FOS
WIPF1
ETS1
SH3KBP1
EVI2B
CD3G
EVI2A
PTMA
HBA2
GIMAP7
GIMAP4
LIMD2
PIK3IP1
HLA-F
DUSP2
CST7
KIAA1551
SAMSN1
MYO1F
SMCHD1
LCK
FNBP1
HCLS1
EPAS1
RCSD1
UCP2
6-Sep
AAK1
BIN2
ITM2A
HLA-DRA
AKAP13
RPS29
RGS10
SPARC
STK17A
TNFAIP3
PRDM1
TRBC2

APOBEC3G
REL
ISG20
APOE
SERF2
EMB
SYTL3
IL10RA
PPP2R5C
HBB
ELF1
ACAP1
GLIPR1
ACTA2
CD27
ABRACL
FYN
TRAF3IP3
SMAP2
CTSS
LTB
DOCK8
HLA-DRB1
CD96
ANKRD44
ALOX5AP
PRKCH
NR4A2
S100A9
CRIP1
GZMK
TRBC1
PIK3R1
RGS2
AKNA
RGCC
PCSK7
IL7R
CD7
S100A8
MYL6
NKG7
CCNH
GRN
HLA-DQA1
CARD16
CREM
PRPF38B
TRIM22
C9orf16
NKTR
FAM49B
COTL1
OAZ1
CCL4
CLEC2D
ARHGEF1
RHOA

ICAM3
FOXP1
RSRP1
RNF213
GNAS
PSAP
DEFB1
S100A1
KRT19
MT1G
C19orf33
GSTA2
MACROD2
RP11-284H18.1
PPP1R14D
FABP6
RP11-1C1.6
SAA1
EGFR-AS1
PDZK1IP1
LYG1
WFDC2
C5orf46
FXYD2
GSTA1
REG1A
TSPAN1
SAA2
SLPI
CRYAB
MT1E
UGT2A3
ADIRF
CD24
MAOA
KRT18
RBP5
S100A13
MGST1
CYB5A
PRAP1
NNMT
TPM1
TMEM176A
NDUFC1
BNIP3
CLU
IFI27
MT1F
MALAT1
NDUFA4L2
MIF
SNHG25
CXCL14
GAPDH
TMSB4X
TCEA3
HCFC1R1

KRT8
LDHA
CCDC146
SERPINA1
B2M
FTH1
TMEM27
UGT2B7
TMSB10
MT-CO1
HP
MIR4458HG
SRGN
SLC17A3
SMIM24
C11orf54
TGFBI
MT-CO2
S100A10
BTG1
HLA-B
CTHRC1
LGALS2
NUPR1
CD70
ANXA4
HLA-C
MT-ND2
DDX5
CXCR4
MT-ND3
RPS27L
SOD2
JUNB
HLA-E
DSTN
ZFP36L2
MT-ATP6
SERPING1
UQCRQ
RARRES2
FTL
C1orf186
NAT8
GNB2L1
MT-CO3
RPL21
ALDOA
S100A11
RPS24
ARHGDIB
TMEM176B
NEAT1
RPL37
RPS29
PTPRC
DUSP23
KLF6

FOS
HRSP12
GSTP1
TXNIP
MT-ND1
ZFP36
MT-ND4
HSPB1
ATP5I
DYNLRB1
S100A6
HINT1
OCIAD2
MT-CYB
JUN
HLA-A
PLIN2
S100A4
IFITM3
RPL10
TXN
CORO1A
RGS1
COX7C
CD52
SON
HCST
GMFG
NDUFB4
GAMT
CISD1
CCL5
ACTB
HMGB1
PPIA
WBP5
CD69
USMG5
SRSF7
PTMA
N4BP2L2
PPDPF
RPL31
TPI1
RNASET2
SRSF5
AC090498.1
RPL41
RPSA
PCBD1
MYL6B
H3F3B
CD3D
PNISR
ZFAS1
PKIG
GPX3
ROMO1

CYTIP
STK4
RPS20
XIST
ATP5H
H2AFJ
TNFAIP3
SARAF
FYB
CD44
SPP1
PRDX1
MCL1
HSP90AA1
NIT2
EVL
ATP5J
NFKBIA
CYSTM1
LAPTM5
ELF1
ARPC2
NACA
DUSP1
PRDX2
CD2
ENO1
COX5B
DDX24
CD53
GZMA
TRAC
DUSP2
RPS3
TSC22D3
RPLP0
ARPC1B
TMEM256
MPC2
UQCR10
SOD1
ANXA2
NKG7
TIMP1
RPL36
NPM1
PPP2R5C
PABPC1
NDUFS5
CD3E
SLA
POLR2I
RPL38
LSP1
RP11-347P5.1
DDX3X
CD37
STK17B

LRRFIP1
RBM39
AKAP13
PHPT1
RPL17
VAMP2
ITGB1
RPS12
HLA-DRB5
CCL4
CD48
RPL22
CLIC4
CCNL1
POLR2J3
CELF2
FUS
COX6A1
IFI16
ITGB2
CFLAR
RAC2
PRPF38B
FOSB
IL2RG
DYNLL1
FNBP1
ARGLU1
JAK1
AAK1
NAA38
ARL6IP5
ID2
RNF213
IER2
SEC61G
HIST1H1C
ANKRD12
GPSM3
COX6C
LCP1
IDS
RPS3A
TRBC2
COTL1
PRRC2C
PIK3IP1
CST7
NDUFA2
WIPF1
KMT2E
RPL15
TXNDC17
PDCD5
UBR4
MT-ND5
ATP5C1
MYL6

RPS8
RSRP1
COX7B
GZMK
NKTR
EVI2B
ATRX
HSBP1
ARF6
LINC00493
STK17A
NGFRAP1
ETS1
MBNL1
PRKCDBP
DDX17
C12orf75
ROCK1
NDUFA7
HLA-F
WSB1
PNRC1
KIAA1551
TCF25
UQCRB
ARID4B
HNRNPA2B1
RNF181
HERPUD1
AKAP9
UQCRH
PPP1R15A
SCAF11
MINOS1
RBM25
STAT3
IRF1
HEBP2
HNRNPA0
ITM2A
CLK1
HMGB2
SMCHD1
PCSK7
SAMSN1
ADI1
CETN2
MYH9
CD3G
CCND3
CTSS
HNRNPA3
TRBC1
FAM162A
SRSF2
JUND
PPP1R2
LTB

WASF2
GIMAP4
ACAP1
MZT2A
TRA2B
BTG2
SYNE2
TNRC6B
LCK
RSRC2
ADGRE5
CYBA
7-Sep
SP100
SRRM2
NCOR1
ACTR2
RGCC
RPS27
PSMA3-AS1
NDUFA1
DNPH1
GCC2
NCL
IL7R
LAMTOR5
TUBA1A
REL
BCLAF1
NDUFB10
CLEC2B
KIF5B
RPL36A
RPL8
LIMD2
CLEC2D
MTDH
CD7
MSN
XRCC5
SLC25A6
CD99
ATP5G3
CFAP36
6-Sep
POLR2L
EVI2A
SH3KBP1
FOXP1
TERF2IP
YWHAZ
LUC7L3
PRDM1
VDAC1
MYO1F
PDCD4
CFL1
FKBP5

ATPIF1
RBPJ
SYTL3
FAM133B
RPL7
PEBP1
FKBP2
SF1
FYN
CRIP1
MPHOSPH8
TRAF3IP3
SERF2
USP15
ZFP36L1
GLIPR1
PNN
SYTL2
CDC42SE2
MRPL51
EMB
KTN1
IQGAP1
NR4A2
IL10RA
GLTSCR2
BNIP3L
TBCA
HCLS1
SLC2A3
ANKRD44
SRSF11
THRAP3
ARHGEF1
NDUFA4
RPL24
FXYD5
PPP1R12A
RPL9
BIN2
TAOK3
GNLY
DOCK8
HNRNPU
MT-ND4L
RCSD1
ODF2L
FAM49B
EPC1
BOLA3
PRKCH
SH3BGRL3
ATP5O
CD96
PDLIM1
RPA3
GIMAP7
AKNA

PIK3R1
CNN3
RPS21
MYCBP2
EMP3
ATM
BTN3A2
EIF4A2
UCP2
NHP2
CREM
SRRM1
ANGPTL4
LEPROTL1
ISG20
CHMP4A
GLRX
PSMB9
PPIG
EEF2
9-Sep
BRD2
COX6B1
YPEL5
CAP1
TLN1
RPL23
DBI
ARPC4
MACF1
RPS11
DDX6
JMJD1C
PFKP
SF3B1
DDX46
CARHSP1
NDUFB6
MGST3
CCNH
RNF19A
CD27
APOBEC3G
ATOX1
TAPBP
RGS10
SMAP2
MMP24-AS1
HNRNPM
TGOLN2
RNPS1
EIF5
MGEA5
DHX36
WNK1
TPR
BLVRB
UBXN1

UBB
DNAJB6
BUB3
PAPOLA
VIM
DNAJA1
RGS2
CFDP1
RHOG
SLTM
TAF7
MYL12A
WDR1
GNAI2
ACTR3
HLA-DPB1
MRPL40
ZFAND5
CKLF
CTSC
HNRNPH1
ICAM3
RTF1
CCDC85B
ATP5G1
ALOX5AP
NDUFB9
PRDX6
DPY30
PRKAR1A
RAB13
GABPB1
RABAC1
OST4
UQCR11
ANXA6
NFE2L2
LAMP1
HSPA8
PCM1
FKBP8
SELM
RNF149
FUNDC2
HNRNPR
YWHAB
DNAJB1
RAP1A
MGST2
SF3B2
SFPQ
CD164
SELT
ATP6AP2
S100A9
TRIM22
VMP1
RSF1

GNG5
ATP1B3
ARPC5
RPL36AL
NDUFB7
TCEB2
CALM1
CASP4
POLR3GL
NDUFV2
MT2A
TMEM14C
LITAF
PSME1
ATP1B1
FCER1G
LIMS1
PPP1CA
TUBA4A
ATP6V1G1
GABARAP
PRDX5
CMC1
DDT
2-Sep
ARID5B
PLA2G16
HILPDA
IFNGR1
PPA1
FAM96B
C10orf54
SAT1
LAPTM4A
EIF4A1
SDCBP
SSU72
RHOA
DRAP1
YBX3
MRPL22
SNRNP70
HLA-DPA1
BZW1
HSPE1
C16orf13
NDUFS4
HLA-DQA1
GGNBP2
ST13
LINC00152
GTF2H5
TYROBP
ZNHIT1
MRPS21
MRPS6
ATP5G2
CAPZB

CD63
COX8A
AIF1
PKM
H2AFV
EIF1AX
CSTB
VKORC1
COA4
C9orf78
GYPC
GPX4
ANXA1
CHMP2A
CDC37
HMGN3
ZCRB1
C19orf70
TAGLN2
PRR13
LYZ
IGKC
TIMM13
CD151
VAMP5
LINC00998
RAP1B
SNRPB2
NDUFA11
NDUFB2
MEA1
BIRC3
SPINT2
CEBPB
BAX
HBB
ATP5E
COX5A
RNF5
YWHAE
C3
MRPS36
TCEAL4
PPIB
SHFM1
TRAPPC2L
CNDP2
GLUL
TIMM8B
NDUFC2
COA3
EDF1
CEBPD
CWC15
NDUFB5
TRMT112
ECH1
MORF4L2

POLR2J
ATP6V1F
TMEM258
EIF4EBP1
MZT2B
ATP5L
CAMTA1
CD68
PGAM1
GUK1
SRP14
EIF5A
TMEM230
JCHAIN
IGHGP
CD79A
IGHG4
IGHG3
IGHG1
MZB1
IGHM
IGLC3
IGLC2
IGKC
IGHA1
B2M
HLA-A
GAPDH
HLA-B
IL32
TMSB4X
VIM
HCST
ID2
FYB
TMSB10
CD2
MYL6
ARPC2
ACTG1
CD3E
CD3D
ITM2B
PTPRC
UQCR11
TNFAIP3
ACTB
ITGB1
CCL5
S100A10
GIMAP4
HLA-C
ATP5E
COX6C
AAK1
SRP14
GZMA
SSR4

ITGB2
CST7
MYL12A
SKP1
SUMO2
MYL12B
RARRES3
ENO1
PSMA7
GUK1
HSP90AA1
NDUFS5
CD99
ARL6IP5
CCL4
HSPA8
HMGB1
YWHAB
HLA-E
SON
HMGN2
COX5B
TMA7
RPS24
GZMK
TPM3
PIK3R1
GIMAP7
ITM2A
CTSD
TRBC2
CD3G
PRKCH
RGS1
DUSP2
PIM2
TRAC
USMG5
PRMT2
COMMD6
LINC00152
RNF213
S100A11
CD7
CALM1
S100A4
HNRNPA1
SLA
ATP5I
LCK
TPI1
VAMP8
CFL1
ANXA1
FYN
RAC1
ARPC1B
RHOA

APOE
NUCKS1
IFITM3
LDHA
SHFM1
JAK1
PFN1
SH3BGRL3
ATP5J2
SYNE2
SERF2
CLEC2B
PSME1
MSN
GSTK1
CD96
MYO1F
HMGB2
UBL5
LCP1
IFI16
SOD1
PSMB8
BTG1
TRBC1
SARAF
FKBP5
SEPW1
DNAJB1
ATP5G2
HNRNPC
DYNLT1
CAPZB
CTSC
TPT1
TSPO
EVL
CLIC1
UBB
RHOC
LDHB
SUB1
NDUFA3
IGFBP7
MBNL1
DYNLRB1
UQCRB
GLRX
GNAS
NEDD8
COX7C
IL7R
ALDOA
NKG7
GBP2
COX8A
COX6A1
PSMB9

RBPJ
PA2G4
AKAP13
HSPB1
NDUFB8
HLA-F
SYTL3
RTN4
CD44
PPIA
DDIT4
BSG
WIPF1
JUN
LEPROTL1
7-Sep
MCL1
NDUFC2
MIF
PGK1
ARHGDIB
HSPE1
PSMB1
UBE2L3
CAP1
XRCC5
GSTO1
HNRNPK
SRSF7
TMEM50A
PTMA
RBX1
CBX3
COX7A2
PRPF38B
HNRNPA2B1
AP2M1
FUS
PAIP2
SERBP1
YWHAQ
SCP2
FXYD5
COX7B
EID1
RGCC
SNRPD2
HNRNPA3
PPP1CA
S100A8
C12orf57
ERH
PKM
WNK1
HSP90AB1
TXN
KIF5B
H2AFZ

VAMP5
OST4
PIK3IP1
PTP4A2
ODF2L
CARD16
HP1BP3
ARF1
SH3KBP1
CCNH
RAB7A
BLOC1S1
COX6B1
GPX4
CKLF
MORF4L1
VMP1
PLIN2
MPC2
CSNK1A1
BAX
S100A9
UGP2
MACF1
CEBPD
HNRNPF
UQCR10
PPP1R12A
TLN1
CALM2
GPX1
HNRNPU
RBM25
ETS1
ALOX5AP
RAN
PSME2
RGS10
CELF2
PEBP1
EMB
MYH9
APOBEC3G
NDUFA4L2
SNRPB2
NFKBIA
YWHAH
GABARAPL2
LIMS1
MINOS1
SDCBP
SNRPG
ROCK1
RANBP1
GCC2
IDS
PCBP1
NDUFC1

CSTB
BIN2
NDUFA13
DHRS7
SEC11A
DDX24
MGST3
FKBP1A
HCLS1
CALM3
LGALS1
C9orf78
DNAJC8
KTN1
RPS27L
ACTR2
PPP2R5C
CIB1
HMGN1
DYNLL1
DNAJA1
SAP18
SSBP1
SH3GLB1
EIF3H
CST3
VPS28
AHNAK
SRI
CHURC1
GNAI2
NDUFB9
BUB3
RNF149
C14orf166
LSP1
DDX3X
PSMA4
ROMO1
IQGAP1
NCL
RWDD1
TBCA
ATP5J
TCF25
SRSF11
ANXA5
NKTR
TUBA1B
TMEM219
RBM8A
STAT3
SRP9
LY6E
DDX46
APOC1
PRRC2C
ANP32B

IFI27L2
POMP
ATP5F1
NDUFB4
CD63
FIS1
PRDX1
ATP6V0E1
PGAM1
COX14
TGOLN2
DNAJB6
SFPQ
MT1X
LAPTM4A
CMC1
RNF7
WSB1
HMGN3
ARID4B
HNRNPM
SF3B2
DDX17
PRELID1
CDC37
DSTN
RNF181
APLP2
YWHAE
ACAP1
GYPC
CCT8
NDUFB5
PCSK7
UBC
ACTR3
ENY2
SSB
ZFP36
ZFP36L2
C1QA
ATP1B3
AC090498.1
PHPT1
EIF3A
MT-ND4L
ARHGDIA
PPP1R2
TAOK3
KHDRBS1
ATP5B
RHOB
NFE2L2
SRRM2
STK4
RNF19A
RHOG
POLR1D

RNH1
SF3B6
NCOR1
TAGLN2
MZT2B
HERPUD1
LAMTOR5
PDCD4
C9orf142
PPIG
TERF2IP
HSPD1
NNMT
ST13
TMEM256
CRIP1
UBXN1
CRYAB
XIST
NSA2
PNN
EIF1B
SQSTM1
SERPINA1
RGS5
ACTA2
TAGLN
BGN
CALD1
MYL9
TPM2
CPE
GJA4
FRZB
PLAC9
PDGFRB
MAP1B
CD36
THY1
RASD1
PLN
COL6A2
TINAGL1
LHFP
MYLK
SOD3
COL1A2
COL18A1
MFGE8
MYH11
PGF
TPPP3
ID1
MCAM
FABP4
CYGB
COX4I2
COL4A2

PPP1R14A
EPS8
KCNE4
PALLD
NOTCH3
CSRP2
KCNJ8
TGFB1I1
HIGD1B
GJC1
COL6A1
FILIP1
CRISPLD2
KANK2
DKK3
NR2F2
CLMN
EBF1
S1PR3
KLHL23
PLS3
MYO1B
COL3A1
VCL
C11orf96
FXYD6
COX7A1
NES
4-Sep
PLXDC1
CCDC102B
NEXN
CARMN
JAG1
TMEM47
TBX2
ID4
AC013461.1
GUCY1A3
KCNAB1
CDH6
ADAMTS1
SSTR2
VASN
AKAP12
SLC38A11
PARM1
CNN1
EFHD1
OLFML2A
PTK2
DLC1
SLIT3
PTN
CHST2
FRMD3
RCAN2
GPRC5C

ADGRF5
CSPG4
GUCY1B3
AGTR1
HOXC9
FHL5
FOXS1
RERG
DAAM2
HEYL
MSRB3
FOXC2
LGI4
NFASC
COL5A2
NGFR
ARHGEF17
MOCS1
ACAN
PRRX1
LMOD1
RGL3
RBP1
C1QTNF1
MAP2
SELENBP1
PTGIR
ABCC9
NGF
SYDE1
NDN
LRRC17
SMOC2
SLC7A2
NR2F2-AS1
RASL12
PDGFA
GUCY1A2
LURAP1L
PRKG1
TCF21
MRVI1
C9orf47
PDE5A
TBX2-AS1
LZTS1
ITGA7
FBLN1
SGIP1
CCDC3
GPR176
EDNRA
DDR2
TBX3
ECM1
ANO1
CD248
HTR1F

SEMA5A
HEY2
TUSC3
ANGPT1
ADGRA2
EBF2
ECM2
PRR16
NEURL1B
PDE1A
FAM162B
RNF180
ISYNA1
EPB41L1
ID3
CDC42EP4
COL4A1
SNAI2
CAV2
CPM
MGP
SCGN
CNN3
ACTN1
LGALS3BP
FILIP1L
PCOLCE
CSRP1
FERMT2
ENAH
PARVA
HES4
EPAS1
UACA
PTPRG
PHLDA1
RBMS3
MAP3K7CL
ANTXR1
PLEKHA4
ARHGAP42
TIMP3
APBB2
TNS1
SLC12A2
PDLIM3
EHD2
DCBLD2
TMEM98
SPARCL1
ADAMTS4
IGFBP5
LAMB2
FAT1
ENPEP
MEIS2
CDC42BPA
PMEPA1

EDIL3
TPM1
MT1M
PTPRK
PPP1R12B
SPARC
SMTN
OLFML2B
ITM2C
STEAP4
DMD
PTRF
PLEKHH3
PPIC
COBLL1
NR2F1
P2RY14
CAV1
SERPING1
LIMA1
PLAT
IGFBP4
TMEM38B
TNS2
ADIRF
SEPP1
SDC2
COL1A1
NRP1
TMEM204
FHL1
CLIC4
PAWR
NDRG2
ESAM
CTGF
CLEC11A
BCAM
ITGA1
GSN
INPP4B
IGFBP7
PDLIM7
DSTN
TPM4
FAM213A
CRIP2
NGFRAP1
NID1
PDLIM1
APOLD1
FHL2
LAMC1
RND3
FSTL1
RBFOX2
MDK
FKBP10

ROCK2
SPECC1
SMIM3
TLE1
ST5
CTTN
LPP
LPL
EDNRB
WBP5
C16orf45
HSPB2
SOCS2
ZNF503
PRKCDBP
PDLIM5
PACSIN2
CFL2
NTN4
NBL1
NDUFA4L2
FBLIM1
CD151
RASAL2
11-Sep
MAP1LC3A
TSC22D1
EMCN
FBXO32
SASH1
PTMS
GNAI1
RBPMS
CYSTM1
TWSG1
CHN1
FN1
TRIP6
CAMK2N1
PRSS23
EFEMP2
GAS6
KLHDC8B
CHST7
GNG12
NUDT4
JADE1
AK1
B3GNT2
MEF2C
MAGED2
UBA2
H1F0
PKIG
ILK
RAPGEF5
TIMP1
GSTM3

TRIB2
TCF4
LGALS1
SERPINH1
SLCO3A1
IFITM3
FARP1
OAZ2
C4orf32
ARHGAP10
GOLIM4
C1orf54
TJP1
ARHGAP29
COL6A3
PPFIBP1
A2M
CYB5R3
HSPB1
UBE2E2
ZFHX3
SPTBN1
SGCB
CD59
SPRY2
RGS16
EPHX1
TSPAN3
PTEN
FAM127A
AXL
SORBS2
LMNA
APP
ENG
GPX3
IFI27
PPARG
ARMCX2
DST
GRK5
ZBTB16
FAM46A
MYO1C
FLNA
SERPINI1
ITGB1
ARHGAP17
SELM
MYL6
TCF7L2
MARCKSL1
ARHGEF7
WBP4
CDKN1A
HIP1
TUBB6
LEPROT

CALU
MGST3
PTPN9
ARHGAP1
SORT1
SOX4
EPB41L2
FSTL3
H2AFJ
CSNK1E
CST3
TRAK1
TOB1
KLF9
ASAP1
PHACTR2
CTBP2
TCEAL4
HES1
TACC1
CPQ
MTHFD2
INAFM1
IFIT3
UTRN
NFIA
PELO
MTUS1
RPS29
ADAP2
7-Sep
MAGEF1
CD63
KIAA0040
C5orf24
PRKAR1A
EGR1
AAMDC
GRAMD3
TFPI
RHOB
RSU1
LAPTM4A
CRIM1
FHL3
TNS3
EID1
SPATS2L
ITGAV
SCPEP1
STOM
DYNLT3
CBX6
OSBPL1A
NENF
RPL28
HTRA1
ACTN4

SH3BGRL
MIR4435-2HG
SSPN
BCL6
RCN2
IFI6
CEBPD
ZMAT3
PPP1R12A
CYBRD1
FABP5
HCFC1R1
CD9
ARMCX3
ZBTB20
ZHX1
SEPW1
ADI1
DYNC1I2
HIGD1A
SLC39A6
RPS27
TAX1BP3
ADD3
CDC5L
RPS15A
SBDS
TIMP2
AIG1
ANXA6
DYNLL1
BCAP29
RPS28
PEBP1
MT2A
RPS2
RPL39
CALM2
BSG
RPLP2
F2R
RAC1
CXCR4
EI24
OLFML3
RAB2A
RRAGA
RPL41
RABGAP1
HSD17B12
CRTC3
SMARCB1
MAP4
ARHGEF12
ADD1
ARFGAP3
EEA1
ABI2

RNASEH2C
CBR1
TLN1
MT-CYB
NUCKS1
PTPRC
DCTN2
ZNF428
RPS12
AP2M1
SKP1
CHD9
FNIP2
ACTR10
AHCYL1
SYPL1
DDAH2
VIMP
FDX1
KCNMB1
GBP2
RTN4
RPL10
CHCHD10
RPS25
TMEM14C
RPS3
ATP5B
RPL26
SYNE2
OSBPL9
BST2
ACADVL
KTN1
RHOC
VASP
PTPN12
ZFP36L2
TMSB10
NDUFS2
TMEM59
SLC25A3
RGS1
ITFG1
ARF4
POLR2F
CLTA
ZFAND5
RPLP1
MTRNR2L8
TBC1D1
2-Sep
RPS14
CORO1A
ROCK1
VMP1
RPS21
RPL23A

COX17
MRFAP1
NSRP1
ATP6AP2
TIPARP
HSP90AA1
RBMS1
TMEM165
SUMO2
CCND1
HCST
RPL27A
RPS24
ISG15
RPS6
EIF4A1
TMEM30A
HSP90B1
RPL37
SRGN
ERH
DENND3
RPS18
PAK1
RPL11
RPL30
S100A6
LAPTM5
RPL13
MT-ATP6
VIM
MYH9
RPL36
RPS19
RPL34
FKBP5
RPL18A
GADD45B
LUC7L3
CD2
CD164
CD37
IL32
RPL19
UBA52
LINC00152
ELOVL5
RPL38
RPS20
FYB
RPL31
ACTB
BTG1
RPL13A
S100A10
RPS26
CD69
CYTIP

TXNIP
MAPRE1
SLA
CD3E
RPS17
CD53
RPS27A
STK4
RPSA
CELF2
CD74
DUSP2
TRAC
JUN
RPL21
CCL5
CD52
CD3D
SH3BGRL3
FOSB
GZMA
RPS15
RAC2
EVI2B
FAU
S100A4
LSP1
CD48
LCP1
IL2RG
RPL36A
SAMSN1
CST7
RPL18
SYTL3
ACAP1
VAMP8
ITGB2
RPS16
CD3G
EVI2A
RP11-347P5.1
KLF6
TNFAIP3
UCP2
HLA-DRB1
GIMAP4
CLEC2B
RPS10
TRBC2
ID2
FOS
BIRC3
FAM49B
ICAM3
FXYD5
STK17A
ALOX5AP

PPP2R5C
STK17B
CCL4
RGS10
ITM2A
HMGB2
CYBA
GZMK
GIMAP7
LTB
EMB
HLA-DPA1
CLEC2D
BIN2
GPSM3
TRAF3IP3
TRBC1
CD7
ATP5E
PIK3IP1
NDUFA4
WIPF1
TUBA4A
PRDM1
LCK
EVL
HLA-DPB1
COTL1
IL10RA
CD27
CD96
NKG7
IL7R
DUSP1
LITAF
PRKCH
OCIAD2
CDC42SE2
CTSS
RPS5
TPM3
APOBEC3G
DOCK8
FTL
SARAF
RPL24
RGS2
REL
OAZ1
RPLP0
MYO1F
DDIT4
RNASET2
SAT1
HLA-DRB5
HLA-E
HLA-DMA
ISG20

HLA-DQB1
MKI67
TOP2A
KIAA0101
NUSAP1
CENPF
UBE2C
ASPM
TYMS
BIRC5
RRM2
TPX2
CDK1
CCNA2
ZWINT
CENPM
CDKN3
MAD2L1
CENPU
PRC1
AURKB
CLSPN
ASF1B
TK1
NUF2
CDCA8
UBE2T
CDC20
GTSE1
FAM111B
TCF19
KIF15
CASC5
SPC25
CCNB2
HIST1H1B
CDT1
BRCA1
KIF23
CENPW
FEN1
RAD51AP1
KIFC1
DLGAP5
KIF11
CENPA
CKAP2L
SPC24
KIF2C
HJURP
CENPN
CDCA3
CEP55
SGOL1
ESCO2
HMMR
CDCA5
NCAPG

FANCI
UHRF1
KIF14
RAD51
TROAP
PBK
MELK
DEPDC1B
MCM10
MND1
CDC45
SPAG5
SGOL2
KIAA1524
CENPK
NCAPG2
BUB1
ATAD5
SHCBP1
MXD3
GGH
CENPE
STMN1
ARHGAP11A
FBXO5
NCAPH
ECT2
SMC2
RACGAP1
HELLS
MCM4
CKS1B
SAC3D1
EZH2
BRCA2
CHEK1
MCM2
ARHGAP11B
GINS2
RMI2
CENPH
ATAD2
CDCA7
AURKA
CCDC34
HMGB3
PCNA
C21orf58
MCM7
PTTG1
LMNB1
CDCA4
BARD1
NDC80
DNAJC9
DHFR
DTYMK
FANCD2

NCAPD2
CKS2
WDR76
WHSC1
PHF19
TACC3
H2AFX
MCM6
RRM1
CHAF1A
SNRNP25
MCM3
SMC4
KIF20B
RFC3
GMNN
RFC4
TUBB
HIRIP3
RP11-545E17.3
BCL2L12
TMEM106C
YEATS4
TUBA1B
RNASEH2A
HMGB2
NCAPD3
LIG1
SKA2
USP1
KIF22
TTF2
VRK1
TMPO
CEP152
H2AFZ
H2AFV
UBE2S
POLD3
FANCA
CDC25B
PXMP2
HIST1H1D
HIST1H4C
ANP32E
DNMT1
PRIM1
TEX30
HMGN2
NUDT1
KPNA2
SAE1
RPA3
HIST2H2AC
CDKN2C
TUBB4B
RPL39L
SMC1A

CKAP2
ACYP1
DDX39A
MCM5
CARHSP1
CBX5
HIST1H1E
MAD2L2
C12orf75
RAD51C
WDR34
CALM3
RBBP7
ORC6
PTMA
CRIP1
HMGB1
MZT1
RANBP1
TOPBP1
DEK
DUT
CKAP5
SLBP
SUZ12
FABP5
RAD21
SMC3
MIR155HG
PSIP1
HN1
FAM111A
NUCKS1
CCDC167
EBP
NUP37
ANP32B
CBX1
RAN
HLTF
IDH2
LSM2
SIVA1
CDKN2D
SUPT16H
APOBEC3H
HNRNPAB
LBR
ZDHHC12
HMGXB4
TFDP1
HDAC1
LAG3
HPRT1
DCTN3
HMGN1
C19orf48
SH2D1A

SNRPD1
TNFRSF9
MPHOSPH9
HSPB11
CEP78
ACTL6A
SNRPF
LSM4
BUB3
HDGF
NASP
CDK5RAP2
UQCC2
TOX
CTCF
SNRPB
ACTB
STRA13
NUDT21
HNRNPD
DUSP4
COX8A
CDK2AP2
MIS18BP1
CFL1
LSM5
PMVK
SRSF10
BAZ1B
HAT1
HIST1H1C
SSRP1
CEP57
ARPC5L
ANXA6
ANKRD36C
TUBA1C
SIT1
H2AFY
SRRT
PPIA
PFN1
ACTG1
CDKN2A
HNRNPR
PPM1G
SLC25A5
KIF2A
FAF1
ARL6IP1
ARL6IP6
BTG3
CD8B
PPP1CA
CD8A
NONO
RAB27A
PMF1

NFATC2IP
PSMB2
XPO1
HNRNPF
SRP9
BANF1
HNRNPA2B1
GZMA
ASH2L
HNRNPA3
VCAM1
DDB2
VBP1
CMC2
CBX3
SUB1
PSMC3
CNTRL
TAP1
COX5A
MZT2B
RALY
UBE2A
PDIA6
RUNX3
DCXR
PIN1
CD82
GAPDH
PARP1
MAPRE1
SFPQ
MAGOH
SNRPD3
APOBEC3C
ILF2
FTL
CORO1A
PSMB9
RPL34
SRSF2
HSD17B10
RAC2
EIF4A3
BLVRA
RPL13
KPNB1
HNRNPM
SNRPG
RPS14
NUCB2
RHEB
PMAIP1
LSM3
APOBEC3G
SPN
NDUFC2
SRSF3

STAG1
BLOC1S1
CD27
HP1BP3
NOP56
PSMB8
PGAM1
CXCR3
WNK1
SDF2L1
TPR
YWHAQ
HNRNPA0
PTPN11
TPM3
LAT
NDUFB3
PLP2
PSMB6
C9orf142
TIMM10
CRTAM
WDR1
PSMA4
TUBA4A
GTF3C6
LINC00152
IFI16
SUMO3
PSMA5
CD3G
UBALD2
LYST
ATP5G3
PSME2
FAM195B
CYB5B
PRPF4B
NDUFS6
NDUFA2
TALDO1
CHCHD2
TRAC
PARK7
ITM2A
HNRNPA1L2
RBX1
XRCC5
PKM
NDUFB11
PSMG2
NKG7
RPL41
COX17
IFI27L2
LCP1
RP11-160E2.6
GALM

PSMA3
ATPIF1
RPL10
CALM2
SET
ERH
NABP2
RHNO1
CD3D
SUMO2
XRCC6
PTPN7
HMGN3
HNRNPA1
RPS27
ANXA5
UBB
PSMD8
NAA38
RNPS1
TSTD1
ATP5L
RP11-386I14.4
RPL3
ABRACL
TMSB4X
ARPC2
DBI
ANAPC11
PSMB10
THRAP3
PTMS
ZNF706
EEF1A1
RPL26
SVIP
MPDU1
SNRPC
COX6C
NCL
DNAJC8
PPP4C
ATP5J2
RBM8A
GNG5
ARPC5
TPT1
PNRC2
IL2RG
LSM7
VDAC1
EIF2S2
POLR2G
RBMX
PA2G4
NAP1L1
RPL13A
RPL39

HLA-A
C11orf31
BZW1
RPL35A
TXNIP
SPCS2
RHOA
YBX1
GZMK
CTSW
HNRNPC
CKLF
CD2
ATP5E
HNRNPU
RPS18
HINT1
SF3B2
RPL9
LIMD2
DUSP1
COTL1
CAPZB
HLA-F
RPL30
CD99
HSPA8
SRSF7
FAM3C
TRBC2
LY6E
CALM1
RPS25
MALAT1

	7-Sep

CLTA
PCBP2
FTH1
LSP1
ARPC1B
ITM2B
TRBC1
NR4A2
RPS13
TSC22D3
PNRC1
PFDN5
JUNB
DDX5
FOS
TOMM7
RBPJ
NEAT1
BTG1
LINC01320
C1orf186
ACSM2B
ACSM2A

PNCK
EMX2
DOC2A
KCNJ16
PRUNE2
MACROD2
UGT2A3
PPP2R3A
PAX8
ELF3
PAX2
CDHR5
ERBB3
SLC16A12
KIF12
DNAH11
RP11-807H17.1
CFB
ITGB8
AOC1
PKHD1
AHNAK2
SLC39A5
PRSS8
PROM1
HOGA1
DCDC2
KCNJ15
LINC01559
MUC1
FAM150B
NEFL
SLC34A2
SLC44A4
RAB42
FAM65C
PTPRM
HHLA2
HSF4
CLDN3
POU5F1
MST1
SLC28A1
ARHGEF28
UGT2B7
ADAMTS9-AS1
ABCC3
TACSTD2
ZNF608
CLDN4
SLC39A14
RHOBTB1
TNFRSF11B
CDK18
PLCB4
SLC6A8
ABHD11-AS1
SLC17A3

EGFR
HPN
GATM-AS1
CCDC64B
ERRFI1
PRKAA2
LINC01358
AR
VEGFA
CLRN3
BICC1
SEMA5B
GAL3ST1
AMN
CAPN12
SLC44A3
FBXO17
PPP1R14D
TMEM98
ASPA
ALPK2
PDZK1
C1S
PARD6B
FAT1
HSD3B7
SLC3A1
MET
CDH2
RP11-1C1.6
MAOA
DMKN
EMX2OS
EGFR-AS1
EPCAM
TSPAN1
ACY3
STARD4-AS1
SLC16A4
RERG
MVK
SLPI
EVA1A
ADAMTS9-AS2
ANPEP
TMEM139
SLC4A4
KRT19
CLEC18C
COBLL1
DIXDC1
SLC22A2
RP11-1C1.4
MTMR11
DDR1
CDH6
CP
HIF1A-AS2

CA9
SLC47A1
CFI
ACMSD
CUBN
ZNF320
EPS8L2
DLG5
CLDN7
NR1H4
RP11-14N7.2
S100A1
FN3K
CA12
ACSM5
SERPINF2
ENPEP
KBTBD11
MYEOV
SERINC2
HILPDA
HSPB8
HOOK2
REG1A
CNKSR3
GNG12
SLC6A13
CTHRC1
LINC00887
MIOX
MIR210HG
LRRC41
CFH
TMEM45A
PPP1R3C
WFDC2
PAWR
HRCT1
PLOD2
AEBP1
C1R
RAB3IP
ZNF395
ANGPTL4
LMO7
PLEKHA5
SLC37A4
ITGA3
KISS1R
GSTA2
APOL1
CDCA2
C19orf33
C11orf54
FAM134B
ZNF248
SAA2
INADL

RDH5
MT1H
CES4A
TFPI
FAM13A
CES2
FABP3
PPP1R13L
BHMT2
PDK1
GSTA1
ENPP3
SAA1
AK4
PTER
TNFAIP6
GALE
PPP1R16A
GGT1
KRT8
HAUS7
SDC4
CCL28
TMEM91
CYP3A5
NBL1
TCEA3
MMP7
KCTD3
ENOSF1
FARP1
C5orf46
TMEM27
TGM2
RASSF4
DYNC2LI1
RP11-83A24.2
MT1G
RBPMS
ENO2
TLN2
SMIM24
SPINT1
TMSB4X
TNFRSF12A
MACC1
MGST1
RRAD
PPFIBP1
P3H2
GATM
B2M
RGS12
PTPRF
CALD1
TPM1
CXCL14
ZMYND8

FNBP1L
MXRA7
AQP3
WDR60
RBP5
RND3
FHL1
TMEM37
TSPAN12
RDH13
ASS1
DPCD
DEFB1
BHLHE41
EGLN3
OXR1
SORBS2
FABP6
CD70
NFIB
PROS1
EFNA1
KHK
PPP1R3B
CCDC146
WDR34
PRRG4
ANK3
NEAT1
RPS12
RPL21
NAT8
ZBTB20
CMBL
RP1-60O19.1
TRIP10
FGGY
LINC00969
CLU
CRYZ
PDZK1IP1
NOL3
PTGR1
TJP2
PTMA
RPS29
TSPAN4
MYO6
LACTB2
CKB
FAIM
EPHX2
LOX
YBX3
SMPDL3A
MUM1
TCN2
PILRB

MGRN1
ECHDC2
SYTL2
GAMT
MGLL
RPS27
PLEKHA1
NDRG1
NNMT
RPL32
ZNF83
RPS4X
PLXNB2
FSTL3
SERPING1
CRLS1
CAV2
AKAP12
FXYD2
SOD2
KRT18
C11orf1
CNDP2
ATP1B1
MXI1
PLA2G12A
FAM195A
CREB5
SHMT1
C11orf49
HSBP1L1
FAHD1
RPL27A
TMEM176A
CARS2
NIT2
SSPN
SH3BP2
ZNF611
WDR13
UACA
RPS3
MT1E
INTS3
RARRES2
SHMT2
RPL15
PLAGL1
LTBR
EVA1C
RPL13A
SNHG25
ARHGAP29
CRYAB
RP11-553L6.5
ERGIC1
IGFBP4
PFKP

RPS15A
RPL34
SRGN
DST
BDH2
HLA-C
RPLP2
AIG1
RPL28
DHTKD1
ADM
CD24
ACTB
ALDH1A1
RPS14
TRIP6
KIAA1191
SH3YL1
NET1
BNIP3
RP11-798M19.6
ANXA4
FAM127B
RPL39
SLC2A1
HLA-B
EPS8
ATP6V0A1
CLIC4
HMGCL
NFE2L1
FRMD4A
MIR4458HG
CCND1
BTG1
FNDC3B
RPS27A
RPL12
JADE1
S100A13
FAU
RASSF7
TMEM176B
LARP1B
POMGNT1
RPL14
WBP5
RPL23A
MYO9A
PHYKPL
RP11-1000B6.3
RAB34
ABHD11
LGALS3BP
VPS13A
MTR
CXCR4
BCKDK

ANKRD37
SMAGP
C5orf45
RBM41
FGD4
RPS25
RPL37A
NPEPPS
SLC16A3
EIF3J-AS1
UBA52
RNASET2
NQO2
RPL18A
SPINT2
SLC25A37
SNHG12
KCNQ1OT1
GADD45A
TPT1
ZFP36L2
RBM47
RPL26
RPSA
STC1
GPX3
ARHGDIB
BIRC3
CYB5R3
HCFC1R1
CD2AP
ISOC2
LIMA1
VCAM1
RPS26
RPS16
PTPRC
IRF7
RPL30
HPS4
NUPR1
TMA7
QTRT1
RPS15
ASPH
MAP3K13
P4HA1
C3
BSDC1
PIAS2
DAB2
UBR4
RPLP1
TM7SF3
NFIA
RUFY3
RPL31
RPL19

ARHGEF12
GSS
IFI27
POLR2J3
LRRK2
CMB9-22P13.1
S100A4
GMFG
CD52
AKAP8
KDSR
RPS19
MT1F
ATP11A
SEC31A
HCST
RPS6
SPP1
RPL29
CFL1
RPS18
TGFBI
ACADM
ATP5E
WDR73
ASL
RPS23
VMP1
MMP24-AS1
POLR2J2
CLK4
RPL35A
RPS28
RPL10A
IFI44L
ZNF37A
PTTG1IP
TMEM63A
ZSCAN18
RPS3A
SLC50A1
CD151
CD69
RPL13
CPEB4
CERS2
RNPC3
RPL11
ALDH3A2
MVP
AKR1A1
UFSP2
ALOX5
ESF1
CBWD5
METTL15
RPL27
ZNF33B

RPL36AL
AHCYL1
MYO1C
RPL18
CORO1A
YPEL2
LGALS2
MBNL2
BNIP3L
GOLGA8A
LPP
TFEC
DUSP23
ADIRF
RGS1
MDM2
CYB5A
EEF1A1
SH3BGRL3
NFIC
SMYD4
CYTIP
ACADVL
BACH1
CD3D
RPS2
MAST4
NDUFA4L2
CD2
RPL4
PLIN2
ARPC2
H3F3B
EEF1D
CDKN1A
PFN1
TOR1AIP2
MALAT1
MAVS
TMEM205
PDK4
RERE
HMGB1
ZNF397
RPS10
PDLIM5
IGFBP3
RPS13
UQCRH
RPS7
RPS24
DUSP2
RPS9
LINC00657
UGCG
CD53
RPL3
HPCAL1

C6orf89
AC159540.1
CHD9
TNFSF10
RNF114
RPL7
LEPROT
AFF1
RRBP1
FOXJ3
ANKLE2
TBL1XR1
CD37
HIPK2
CD3E
SERPINA1
ACBD3
CCL5
HLA-A
CYBA
POLR2H
RAB11FIP1
ABLIM1
RPL35
PDE4D
PRKAG2
HSPB1
RPL6
TRAC
FYB
FAM63B
ZBTB16
GOLGA2
RPL41
CD44
HNRNPA1
GOLGB1
TTPAL
DDAH2
SERF2
MYL12A
ANKRD36
CD48
CST7
SFXN1
HLA-E
TNFAIP3
11-Sep
GDI1
RPS17
PKM
MLEC
HMGN3
POLR2C
CCNL2
PDXDC1
RP11-347P5.1
PPP2R5C

RPL23
CCL4
KIAA0907
SREK1
SLC25A36
SELM
CFDP1
GZMA
SAT2
LMF2
ELF1
GLS
HLA-DRB5
CELF2
CFAP97
SOX4
RPS11
CBR4
KLHDC2
GPSM3
PFDN5
RORA
IL2RG
STK17B
LCP1
JUNB
MKLN1
COX4I1
PSME1
METTL7A
RPL38
SLA
EVL
GTF2I
WDR11
GATAD1
TRIM56
YBX1
RPL5
GZMK
RNMT
CBWD1
RAC2
BTF3
TIPARP
RPL9
USP8
SUMO2
STK4
CALM1
LTB
LMAN1
LAPTM5
CARHSP1
H3F3A
GIMAP4
OAZ1
EVI2B

UQCRB
TRBC2
DDB2
TUBGCP2
PABPC1
CD3G
EEF1B2
PARP14
CDK5RAP3
LSP1
GOLGA8B
RPL24
IARS
GIMAP7
GOSR1
PSMD5-AS1
TRBC1
PIK3IP1
SAMSN1
SPAG9
ITM2A
RPL37
LCK
STK17A
RPS5
NKG7
NBEAL1
RGCC
PRDM1
CTSA
MT-CO2
6-Mar
CD7
MGST3
RPS8
LDHA
OST4
EVI2A
CHD2
CTSD
LONP2
DDX17
ARPC1B
CTSS
ANKRD36B
LIMD2
ZNF451
ARPC3
TTC3
NDUFC1
TOMM7
SNHG9
HSP90AA1
RCSD1
SUB1
AHNAK
VEZT
TPI1

WSB1
EMB
CLEC2B
BIN2
SH3KBP1
CD27
SRP14
RPS20
SARAF
DARS
GNG11
SLC35E3
BORCS7
FNBP1
COMMD6
IER3
FYN
PNRC1
MYO1F
PHPT1
MYL12B
PRKCH
ZKSCAN1
PRDX2
NME3
WIPF1
RPL36A
FXYD5
IL7R
NR4A2
FNIP1
SRGAP1
RPL36
EXOC7
LUC7L3
ISG20
ATP5L
RPS27L
COTL1
ITGB2
CANX
EZR
GLIPR1
LAPTM4A
SQSTM1
LGALS1
CCDC14
TRAF3IP3
PEBP1
SLC38A2
NACA
AKNA
KIAA1551
IFI16
HERC4
FUNDC2
AFF4
TXNIP

OCIAD2
HM13
CD96
ID2
PHF14
EIF3E
ETS1
SLC25A6
SPG7
UBB
RPL8
IL10RA
ALDOA
6-Sep
RPL22
ANKRD44
ARF6
FOS
ACTG1
ATF6B
SMAP2
ACAP1
GABPB1-AS1
CSNK1A1
ANKRD36C
TNRC6A
SYNE1
DOCK8
DDX24
SRSF1
C14orf2
COX7A2
7-Sep
CAPZB
FOXP1
FAM49B
INSR
EIF3K
GNLY
UBL5
VIMP
MT-ND3
RTN4
BRK1
CARD16
SYTL3
ATP1A1
CRIP1
CKLF
HLA-DPB1
MYL6
P4HB
GTF3A
UQCR11
AKAP9
TTC19
JUND
EMP3

DDIT4
RGS2
RHOA
DSTN
HLA-F
CD99
SRSF10
ARHGEF1
CLEC2D
SMCHD1
ADGRE5
SNRNP70
LINC00152
HSPA8
FKBP5
S100A9
PRPF38B
EIF3F
UPF2
ARPC5
COX6B1
TES
ITGB1
MYEOV2
GNAI2
RGS10
KLF6
HMGB2
SMARCC2
NEDD9
HCLS1
MSN
DNAJB6
CDC42
CLIC1
ATP1B3
UBXN1
HLA-DQA1
ARL6IP5
SH3BGRL
NAP1L1
N4BP2L2
IFITM2
PSME2
LEPROTL1
CCNI
PSMB9
TAGLN
TPM2
RGS5
ACTA2
THY1
MGP
CALD1
PLAC9
MYL9
FRZB
IGFBP7

BGN
SPARC
LGALS1
CPE
ADIRF
CD36
RGS1
PTPRC
NDUFA4L2
CXCR4
TIMP1
SARAF
BTG1
MT2A
CD74
LAPTM5
NEAT1
TXNIP
ZFP36L2
CORO1A
HLA-E
RPS29
HCST
FYB
SRGN
PNISR
SH3BGRL3
KLF6
CCL5
SAT1
EVL
SRSF7
ID3
STK4
ID2
LSP1
HMGN1
MT-ND1
ZFP36
CD44
NDUFA4
CD53
TMA7
S100A10
CD37
DSTN
CELF2
CD52
TPM3
S100A6
HLA-DPA1
TRAC
HLA-DPB1
CD2
ITGB2
MT-ND2
AC090498.1
S100A4

HLA-DRB1
VAMP8
GMFG
HLA-F
GZMA
FXYD5
MALAT1
RPL17
RPS15A
CYTIP
MT-ND5
PABPC1
HMGB2
TSPO
TNFAIP3
DAZAP2
SNHG8
CD69
PRRC2C
MCL1
CCNL1
CD3E
ARGLU1
LCP1
HLA-DRA
HLA-DQB1
FAM49B
SPCS1
RSRP1
DDX3X
COTL1
TPM1
FOSB
CD3D
KMT2E
MYL6
EIF3F
IL2RG
PRPF38B
IFI16
DUSP2
CD48
RAC2
TRBC2
MT-ND3
CLEC2B
ELF1
RNF213
IFITM3
HMGN2
CCL4
IER2
POLR2J3
AAK1
KIAA1551
DNAJB1
FUS
MT-ND4L

LDHA
PRR13
RPSA
HERPUD1
ARL6IP5
LITAF
UCP2
ATRX
SRRM2
ANKRD12
AKAP13
SERP1
ACAP1
ARF6
WIPF1
LEPROTL1
REL
DDX17
DOCK8
IDS
ISG20
GPSM3
ALOX5AP
PIK3IP1
TRBC1
SLA
MBNL1
CTSS
GCC2
RNASET2
XIST
BTG2
LIMD2
RP11-347P5.1
RSRC2
TCF25
CST7
YPEL5
STK17A
FNBP1
TRA2B
SPARCL1
AKAP9
TSC22D3
CCND3
EVI2B
ZNF90
RGS2
GPBP1
ARL6IP1
EVI2A
SMAP2
RPS21
CLEC2D
ITM2A
CDC42SE2
NR4A2
EIF4B

IRF1
LCK
SMCHD1
RPL28
SERPINA1
TAF1D
GZMK
PRKCH
HSPB1
MAP1LC3B
NKG7
ANXA1
HSD17B11
SERPINB1
SYTL3
DUSP1
PPP1CA
CNN3
LTB
ETS1
PLP2
CCNH
DHRS7
ADGRE5
UFC1
HLA-DMA
GLRX
PPP1R15A
TUBA4A
SAMSN1
GNAI2
GIMAP4
MYO1F
6-Sep
PTP4A2
CD3G
PNN
PCSK7
RGS10
SMDT1
APOBEC3G
EMB
PIK3R1
PSMA3-AS1
IQGAP1
BIRC3
TYROBP
ATM
CD27
AKNA
ENSA
CREM
OCIAD2
IL32
RNF19A
PSAP
HLA-DRB5
GIMAP7

MGEA5
TMCO1
MT-ND4
ZNF207
DHX36
ORMDL1
HLA-DQA1
TYMP
GPX1
BTN3A2
HLA-A
FYN
PRDM1
DPP7
TRAF3IP3
IDH2
PLIN2
EPC1
CD96
IL7R
GLUL
CTSB
WNK1
IL10RA
RAB2A
C9orf142
PHLDA1
FCER1G
CD7
CMC1
C10orf54
BIN2
ODF2L
RPL30
RGCC
NPC2
ACTB
CUTA
COX8A
SOD2
TRMT112
LYZ
VPS28
APOC1
SEC61B
RHOA
SEC11A
REEP5
ATP5J2
APOE
NUCKS1
AIF1
COX5A
CAPZA1
NEDD8
NOP10
FKBP8
CST3

NDUFA13
KTN1
C14orf166
COX6C
ATP5G3
ACKR1
COL8A1
RAMP3
CPE
MPZL2
SLCO2A1
FBLN2
POSTN
PTPRB
PALMD
NTS
COL3A1
TFF3
CCL14
ECSCR
IL33
HSPA2
NR2F2
MMRN1
MMRN2
PPIC
MALL
MEIS2
ADAMTS18
TPD52L1
RSPO3
ELN
SELP
NRN1
SRPX
CPXM2
TLL1
TEK
VEGFC
RASSF9
GPM6A
PTGIS
DOC2B
RAB3C
MMP28
LHX6
THSD7A
ITGB4
CYYR1
PCAT19
TM4SF1
EPHB4
TSPAN7
CNTNAP3B
BMP4
PCDH17
ERG
ZNF521

CYP1B1
FAM167B
ADGRG6
TMTC1
CXorf36
FAM171A1
CLEC14A
HSPG2
PGM5
SNCG
CALCRL
NUAK1
RAMP2
FAM107A
MTUS1
SYT15
HEG1
PLSCR4
WWTR1
PIR
PROCR
FGD5
VWF
LIMS2
PLA1A
CDC42EP5
JAM2
SEMA6A
ADGRL4
LDB2
PALM
PLAT
NPDC1
RNASE1
SDPR
HYAL1
EGFL7
HYAL2
LRP5
NOSTRIN
PLCB4
EMCN
HSPA12B
EMP1
TIE1
RHOJ
CTGF
FBN1
PTRF
MEDAG
LRRC1
LIFR
ZNF385D
DNASE1L3
EFEMP1
SEPN1
ROBO4
SH3BGRL2

MID1
PODXL
LMO2
LMCD1
LIMCH1
FRY
PLPP3
FLNB
PCGF2
CADPS2
DOCK9
FGFR1
CDH5
PBX1
EMP2
MANSC1
ADCY4
IL1R1
MIR99AHG
TSHZ2
PTPRG
RP11-382A20.3
CD109
AQP1
TGM2
CD34
RAI14
FAM198B
ACVRL1
LAMA4
ADAM15
MARCKSL1
FAM84B
EPB41L4A
ENG
TFPI
NFIB
TCN2
CRIP2
MAP2
MYCT1
RUNX1T1
CTNNAL1
CFH
MYO5C
TGFBR3
ITGA6
SASH1
FN1
ACKR3
MECOM
SLC9A3R2
HRCT1
SHE
ADAMTS1
HAPLN3
FZD4
IGFBP4

GAS6
SEMA3F
CRIM1
C8orf4
TMEM255B
PLAC9
RFK
DIXDC1
AHNAK2
SOCS2
EPAS1
IFI27
S100A16
ESAM
TINAGL1
S1PR1
PDLIM4
SESN3
CFI
NPR1
PTPN14
SPTBN1
TCF4
RAPGEF5
NID1
ST6GALNAC3
TJP1
DLC1
ATP8B1
FXYD6
LDLRAD3
ASS1
ITGA5
EDN1
BMPR2
NFIA
SPARCL1
APP
MPDZ
PREX2
PRSS23
AKAP12
HOXB7
CNN3
TEAD1
CDC42BPA
ECE1
CLDN5
TANC1
TIMP3
CEP112
GOLM1
APBB2
SULF2
FZD6
PDLIM1
RPS6KA2
PTPRM

CLEC1A
LHFP
GNG11
PECAM1
EPB41L3
TUBB6
TSPAN9
ACTN1
CLU
NCKAP1
FSTL1
HTRA1
SPRY1
ABLIM1
LRRC32
BCAM
AFAP1
CD59
WBP5
RPGR
CDA
TNS2
EHD2
CAV1
COX7A1
ITGA10
PXDC1
CALD1
CD93
ABCG2
SH3BP5
CRNDE
ADIRF
PLEKHG1
MYOF
IPO11
MARCKS
FAM213A
RASA4
ARHGAP29
ELK3
PLXNA2
YBX3
FERMT2
PRCP
SULF1
CHSY1
MSRB3
COL4A1
BACE2
WWP1
LAMB2
RBMS2
FKBP9
CNRIP1
SNTB2
CAV2
ST6GAL1

HS3ST1
NREP
LIMA1
TMEM150C
CTTN
KIAA0355
ARL4A
S100A13
NGFRAP1
PHACTR2
RBP1
PMP22
MRAS
MET
PKP4
C16orf45
LAMC1
MDK
INPP1
RAPH1
MKL2
PDLIM3
CYR61
PLPP1
PLS3
PVRL2
THBD
SPARC
TACC1
PDLIM5
KLF9
CCDC50
CDC42BPB
AGRN
FKBP1A
C10orf128
PIK3C2A
HOXD8
ALDH1A1
IL6ST
ID1
ZFYVE21
STAB1
PLXND1
ID3
SHC1
IFITM3
AK1
HHEX
C4orf32
NFE2L1
GFOD2
SYNPO
TRIOBP
PLVAP
GJA1
TNFAIP1
PLK2

PTMS
HES1
COL4A2
IGFBP7
GALNT15
NEDD9
ASAP1
TNS1
TGFBR2
DUSP23
CLIC2
SSBP3
ZBTB8A
BGN
MYO1C
RFX3
LMNA
RALGAPA2
PTK2
RAB13
MGP
PTTG1IP
ITPR2
TM4SF18
BTBD3
DAAM1
TCEAL4
EPHX1
EXOC6
ITPRIP
PIEZO1
SERPINB6
GSE1
CTNND1
STOM
ASRGL1
RP11-553L6.5
NRP2
TSPAN3
TPM4
TSPAN4
MCAM
ACACB
ANXA2
MEF2C
S100A6
DKK3
PPM1F
SWAP70
DSTN
MLLT4
ARHGAP31
C1orf21
EVA1C
JAG1
CD151
TMEM47
IRF2BP2

GNAQ
SPTAN1
MGST2
SUN1
SSFA2
DOCK4
NDRG1
LRRC16A
LEPROT
PRKCDBP
SLC40A1
HSD17B12
CCDC85B
TMCC3
PINK1
TMEM30A
SLC44A2
ARHGEF12
MAPK3
GNG12
ASPH
AHR
SEC14L1
ATP11C
GALNT1
PARVB
SLC38A2
RCN1
HDAC7
DUSP6
TIMP1
ARL2
PSMB5
ARPC1A
CSGALNACT1
NOC3L
EBPL
MTRNR2L1
EHD4
WARS
PLEC
ABL2
FNIP2
ZMAT3
CLIC4
PXN
COL18A1
NCOA7
VGLL4
LTC4S
IFITM2
CD320
FCHO2
PERP
RAB11A
CHMP3
ZEB1
UGCG

C1orf54
NAA10
NFIC
FAM43A
GIMAP6
TRIB2
MKNK2
GSN
ATL3
SLK
GNAI2
AKT3
DPYSL2
ATP11A
FOXO1
RASAL2
UBE2H
NFAT5
TPST2
TNFSF10
KLF2
SERPINE1
VAT1
ADD1
HEBP1
GUK1
S100A10
SEPW1
TCEAL3
TIMP2
DNAJC10
HSPB1
PON2
IFI44L
METTL7A
CTTNBP2NL
DYNC1LI2
RDX
MAN1A1
SELM
DST
VIM
FILIP1
AFF1
MYL12B
ICAM2
BST2
GIMAP7
AHNAK
IKBIP
KLF7
FEZ2
YWHAE
ENTPD1
MT-CYB
PEA15
NAA38
SRP14

CTNNA1
C4orf48
GINM1
TNFRSF1A
FRMD4B
CTNNB1
CDC37
RRBP1
VAMP3
KLF4
FNDC3B
SNRK
TMEM205
CALU
QKI
NTAN1
RHOC
BTBD7
NKTR
CSRP1
FOXP1
MBNL2
KTN1
RPS27L
NUCB2
LINC00657
RRM2B
GLS
ZKSCAN1
FAM3C
RPS27
MACF1
CHD9
ETS2
MED13L
CXCR4
MYH9
VCAM1
NDUFA8
CAPNS1
FLI1
HERC2
VAMP5
CARHSP1
ZBTB20
MGST3
SERINC3
CYBA
XAF1
CD63
CD55
APLP2
TMOD3
TANC2
PLSCR1
EID1
DYNC1I2
MTRNR2L8

CLTA
ITGB1
SERINC1
PRDX2
HIF1A
PIM3
UTRN
VPS13A
CRTAP
PTPRC
FILIP1L
GIMAP4
LAPTM4A
RAC1
GBP4
ATOX1
IFITM1
POLR2L
CDC42EP3
HSP90B1
CCPG1
C5orf24
APOL1
LPP
ENY2
TNRC6A
LGALS9
SH3GLB1
NDUFC2
MT-ND6
RTN4
MCFD2
LIMS1
NUCB1
SNHG7
ITM2B
BRI3
PCMTD1
ATP1A1
TAGLN2
MT-ND1
CD81
RNF115
IL13RA1
RGS1
LAP3
SOCS3
TRIM56
CNIH1
SCARB2
PRDX1
SEC62
BNIP2
SPAG9
DYNLL1
ACLY
HCST
MYL12A

TM9SF3
JMJD1C
FIS1
ZFP36L2
CORO1A
XIST
TPRKB
GNG5
ARL6IP1
MT-ND4
WSB1
RBMS1
ENPP2
BAZ2B
S100A4
LAPTM5
POLE4
ARRDC3
STC1
RPL28
BTG1
ACTB
CCL5
CD69
NEAT1
SERBP1
PARP14
NUCKS1
LRRFIP1
RPS29
MT-CO3
CD37
TRAC
CD44
MTRNR2L12
FYB
STK17B
HLA-E
SLA
MT-ND4L
GOLGB1
CYTIP
CD52
LSP1
CD2
RPL41
HP1BP3
SYNGR2
CST7
ITGB2
MT-ND5
GMFG
DUSP2
CD3E
GZMA
CD53
SAMSN1
RP11-347P5.1

LCP1
RAC2
RPLP2
GLIPR1
H3F3B
CD3D
RPL27A
RPL18A
TXNIP
CD48
EVI2B
RGS10
STK4
UCP2
TRBC2
CD3G
ARHGDIB
IL2RG
IL32
EVL
CELF2
SYTL3
PRDM1
EVI2A
LTB
ADGRE5
RCSD1
PIK3R1
ACAP1
CLEC2D
IL10RA
TRAF3IP3
LCK
RNASET2
TRBC1
BIN2
TPSB2
TPSAB1
CPA3
CTSG
MS4A2
HPGDS
RGS13
VWA5A
SLC18A2
GATA2
IL1RL1
KIT
HDC
RP11-354E11.2
MLPH
TPSD1
GCSAML
STXBP6
AC004791.2
KRT1
NTM
CALB2

P2RX1
C1orf186
FLJ21408
MAOB
LTC4S
HPGD
RAB27B
SMYD3
HS3ST1
FCER1A
SDPR
CAPG
BTK
AREG
ACOT7
CD9
CSF1
LAPTM4A
LMO4
CLU
EGR1
ANKRD28
LMNA
STX3
RENBP
FER
CD63
ALOX5AP
MAML1
CD69
ANXA1
JUN
ACSL4
ALOX5
FOXP1
PPP1R15A
PTGS1
FOS
MITF
CTNNBL1
SLC26A2
PLAUR
RNF130
GLUL
LEO1
H3F3B
SAMSN1
FTH1
BEX4
CD82
CXCR4
FOSB
IER2
TESPA1
ARHGAP18
RGS2
SRGN
RAB32

NR4A1
NFKBIA
FCER1G
TMSB10
ASAH1
GALC
RHOBTB3
MAST4
MSRA
FAM46A
CD33
MT-ND4L
RPL28
TXNIP
RPL37A
RGS1
ADRB2
PTMA
2-Sep
RPL34
SLC44A1
CRBN
ZSWIM4
STMN1
C4orf48
CPM
HLA-B
GRAP2
MT-ND3
CKLF
MT-ATP6
TMEM154
RPL7
PLIN2
TSC22D1
CTD-3252C9.4
ITM2C
SEMA4A
NFKBIZ
IL32
CCL5
NDUFA4
HLA-DPB1
FYB
PEBP1
HLA-A
CORO1A
KCNQ1OT1
SOCS1
MT-ND4
PFN1
MT-CO2
HLA-C
TMSB4X
JUNB
CD74
GPR65
LYL1

FXYD5
HLA-DPA1
ATP6V1F
CD3E
PRNP
SELK
HINT1
S100A6
LAT2
CYTIP
LPCAT2
CD2
TRAC
DUSP10
BHLHE40
GPX4
FOSL2
AHI1
ACTB
PLGRKT
SPINT2
SNX29
SDCBP
S100A10
EVL
RPS6KA5
EMP3
TSPAN4
ANXA2
STK17A
BST2
CFLAR
TRBC2
BTG2
GZMA
TSEN54
DUSP1
GADD45B
ACTG1
CTSD
RPL36AL
HCST
MAPRE1
ACER3
PRDX6
ETS1
GIMAP4
SYAP1
IL18
CD3D
SYNE2
TRBC1
ISG20
