# Supplementary material for: Single-Cell and Transcriptome-Based Immune Cell-Related Prognostic Model in Clear Cell Renal Cell Carcinoma
Source: J Oncol. 2023 Mar 7;2023:5355269. doi: 10.1155/2023/5355269 (PMC10014191; doi:10.1155/2023/5355269)
Supplement: Supplementary Materials — Supplementary Table 1: Notes on cell clustering. Supplementary Table 2: Differential genes in each cell cluster. Supplementary Table 3: Ligand-receptor relationship pair. Supplementary Table 4: Immune cell multifactor network relationship pair. Supplementary Table 5: Intersection genes in immune cell multifactor network relationship pair and TCGA. Supplementary Table 6: Genes in black and magenta models of WGCNA. [file 5355269.f1.zip › Supplementary Table 3. Ligand-receptor relationship pair.pdf]

	Ligand.Syr	Pair.Name	Receptor.Symbol
1	A2M	A2M_LRP1	LRP1
2	ADAM10	ADAM10_/	AXL
3	ADAM12	ADAM12_I	ITGB1
4	ADAM12	ADAM12_S	SDC4
5	ADAM15	ADAM15_I	ITGB1
6	ADAM17	ADAM17_I	ITGB1
7	ADAM2	ADAM2_C	CD9
8	ADAM2	ADAM2_IT	ITGB1
9	ADAM28	ADAM28_I	ITGA4
10	ADAM9	ADAM9_IT	ITGA3
11	ADAM9	ADAM9_IT	ITGB1
12	ADM	ADM_CAL	CALCR
13	ADM	ADM_CAL	CALCRL
14	ADM	ADM_GPR	GPR182
15	ADM	ADM_MR(	MRGPRX2
16	ADM	ADM_RAM	RAMP2
17	AHSG	AHSG_INS	INSR
18	ALOX5AP	ALOX5AP_	ALOX5
19	AMELX	AMELX_LA	LAMP1
20	AMELY	AMELY_LA	LAMP1
21	AMH	AMH_EGFI	EGFR
22	ANGPT1	ANGPT1_I	ITGB1
23	ANGPTL4	ANGPTL4_	TIE1
24	ANXA1	ANXA1_D	DYSF
25	ANXA1	ANXA1_EC	EGFR
26	ANXA1	ANXA1_FF	FPR1
27	ANXA1	ANXA1_FF	FPR2
28	ANXA1	ANXA1_FF	FPR3
29	APOB	APOB_ITG.	ITGAM
30	APOB	APOB_ITG	ITGB2
31	APOB	APOB_OLI	OLR1
32	APOC3	APOC3_TL	TLR2
33	APOE	APOE_CHI	CHRNA4
34	APOE	APOE_LDL	LDLR
35	APOE	APOE_LRP	LRP1
36	APOE	APOE_LRP	LRP2
37	APOE	APOE_LRP	LRP5
38	APOE	APOE_LRP	LRP8
39	APOE	APOE_SCA	SCARB1
40	APOE	APOE_SOI	SORL1
41	APOE	APOE_VLD	VLDLR
42	APP	APP_CAV1	CAV1
43	APP	APP_CD74	CD74
44	APP	APP_FPR2	FPR2
45	APP	APP_GPC1	GPC1
46	APP	APP_LRP1	LRP1
47	APP	APP_NCST	NCSTN
48	APP	APP_NGFF	NGFR
49	APP	APP_SLC4!	SLC45A3
50	APP	APP_TNFR	TNFRSF21
51	AREG	AREG_EGF	EGFR
52	AREG	AREG_ERB	ERBB3
53	AREGB	AREGB_EG	EGFR
54	AREGB	AREGB_ER	ERBB3
55	ARF1	ARF1_CHR	CHRM3
56	ARF1	ARF1_INSI	INSR
57	ARF1	ARF1_PLD:	PLD2

58	ASIP	ASIP_MGR	MGRN1
59	B2M	B2M_CD1	CD1A
60	B2M	B2M_CD1	CD1B
61	B2M	B2M_CD2	CD247
62	B2M	B2M_CD3	CD3D
63	B2M	B2M_CD3	CD3G
64	B2M	B2M_HFE	HFE
65	B2M	B2M_HLA-	HLA-F
66	B2M	B2M_KIR2	KIR2DL1
67	B2M	B2M_KIR2	KIR2DL3
68	B2M	B2M_KIR3	KIR3DL1
69	B2M	B2M_KLRC	KLRC1
70	B2M	B2M_KLRD	KLRD1
71	B2M	B2M_LILRE	LILRB1
72	B2M	B2M_LILRE	LILRB2
73	B2M	B2M_TFRC	TFRC
74	BCAN	BCAN_EGF	EGFR
75	BDNF	BDNF_DDF	DDR1
76	BDNF	BDNF_NGI	NGFRAP1
77	BGN	BGN_LY96	LY96
78	BGN	BGN_TLR2	TLR2
79	BGN	BGN_TLR4	TLR4
80	BST1	BST1_CAV	CAV1
81	BTC	BTC_EGFR	EGFR
82	BTC	BTC_ERBB	ERBB3
83	BTLA	BTLA_CD2	CD247
84	BTLA	BTLA_CD7	CD79A
85	BTLA	BTLA_TNF	TNFRSF14
86	BTLA	BTLA_VTC	VTCN1
87	C1orf200	C1orf200_	SLC16A4
88	C1QA	C1QA_CD	CD93
89	C1QA	C1QA_CR1	CR1
90	C1QA	C1QA_CSF	CSPG4
91	C1QB	C1QB_LRP	LRP1
92	C3	C3_C3AR1	C3AR1
93	C3	C3_C5AR2	C5AR2
94	C3	C3_CD19	CD19
95	C3	C3_CD46	CD46
96	C3	C3_CD81	CD81
97	C3	C3_CR1	CR1
98	C3	C3_IFITM1	IFITM1
99	C3	C3_ITGAM	ITGAM
100	C3	C3_ITGAX	ITGAX
101	C3	C3_ITGB2	ITGB2
102	C3	C3_LRP1	LRP1
103	C4A	C4A_C3AR	C3AR1
104	C4BPA	C4BPA_CD	CD40
105	C4BPA	C4BPA_LR	LRP1
106	C5	C5_C5AR1	C5AR1
107	C6orf15	C6orf15_D	DDR1
108	CALM1	CALM1_AE	ABCA1
109	CALM1	CALM1_AI	ADCY8
110	CALM1	CALM1_AI	ADCYAP1R1
111	CALM1	CALM1_A	AQP6
112	CALM1	CALM1_C	CACNA1C
113	CALM1	CALM1_C	CALCR
114	CALM1	CALM1_CI	CNGA2
115	CALM1	CALM1_CI	CRHR1

116	CALM1	CALM1_EG	EGFR
117	CALM1	CALM1_FA	FAS
118	CALM1	CALM1_GL	GLP1R
119	CALM1	CALM1_GL	GLP2R
120	CALM1	CALM1_GF	GP6
121	CALM1	CALM1_GF	GRM3
122	CALM1	CALM1_GF	GRM4
123	CALM1	CALM1_GF	GRM5
124	CALM1	CALM1_GF	GRM7
125	CALM1	CALM1_HI	HMMR
126	CALM1	CALM1_H	HTR2C
127	CALM1	CALM1_IN	INSR
128	CALM1	CALM1_KC	KCNN4
129	CALM1	CALM1_KC	KCNQ1
130	CALM1	CALM1_KC	KCNQ3
131	CALM1	CALM1_KC	KCNQ5
132	CALM1	CALM1_M	MIP
133	CALM1	CALM1_M	MYLK
134	CALM1	CALM1_M	MYLK2
135	CALM1	CALM1_OI	OPRM1
136	CALM1	CALM1_PD	PDE1A
137	CALM1	CALM1_PD	PDE1B
138	CALM1	CALM1_PD	PDE1C
139	CALM1	CALM1_PF	PPAPDC2
140	CALM1	CALM1_PT	PTH2R
141	CALM1	CALM1_PT	PTPRA
142	CALM1	CALM1_SC	SCN10A
143	CALM1	CALM1_SC	SCN4A
144	CALM1	CALM1_SC	SCTR
145	CALM1	CALM1_SE	SELL
146	CALM1	CALM1_TF	TRPC3
147	CALM1	CALM1_TF	TRPC5
148	CALM1	CALM1_VI	VIPR1
149	CALM2	CALM2_AE	ABCA1
150	CALM2	CALM2_AD	ADCY8
151	CALM2	CALM2_AC	AQP6
152	CALM2	CALM2_CA	CACNA1C
153	CALM2	CALM2_EG	EGFR
154	CALM2	CALM2_GF	GP6
155	CALM2	CALM2_GF	GRM5
156	CALM2	CALM2_GF	GRM7
157	CALM2	CALM2_IN	INSR
158	CALM2	CALM2_KC	KCNQ1
159	CALM2	CALM2_KC	KCNQ3
160	CALM2	CALM2_KC	KCNQ5
161	CALM2	CALM2_M	MYLK
162	CALM2	CALM2_M	MYLK2
163	CALM2	CALM2_PD	PDE1A
164	CALM2	CALM2_PD	PDE1B
165	CALM2	CALM2_PD	PDE1C
166	CALM2	CALM2_PF	PPAPDC2
167	CALM2	CALM2_SC	SCN10A
168	CALM2	CALM2_SC	SCN4A
169	CALM2	CALM2_SE	SELL
170	CALM2	CALM2_TF	TRPC5
171	CALM3	CALM3_AE	ABCA1
172	CALM3	CALM3_AD	ADCY8
173	CALM3	CALM3_AC	AQP6

174	CALM3	CALM3_EG	EGFR
175	CALM3	CALM3_GF	GP6
176	CALM3	CALM3_GF	GRM5
177	CALM3	CALM3_GF	GRM7
178	CALM3	CALM3_IN	INSR
179	CALM3	CALM3_KC	KCNQ1
180	CALM3	CALM3_KC	KCNQ3
181	CALM3	CALM3_KC	KCNQ5
182	CALM3	CALM3_M	MYLK
183	CALM3	CALM3_M	MYLK2
184	CALM3	CALM3_PD	PDE1A
185	CALM3	CALM3_PD	PDE1B
186	CALM3	CALM3_PD	PDE1C
187	CALM3	CALM3_PF	PPAPDC2
188	CALM3	CALM3_SC	SCN10A
189	CALM3	CALM3_SC	SCN4A
190	CALM3	CALM3_SE	SELL
191	CALM3	CALM3_TF	TRPC5
192	CALR	CALR_HLA	HLA-F
193	CALR	CALR_ITGA	ITGA2B
194	CALR	CALR_ITGA	ITGA3
195	CALR	CALR_ITGA	ITGAV
196	CALR	CALR_LRP1	LRP1
197	CALR	CALR_MTN	MTNR1A
198	CALR	CALR_SCA	SCARF1
199	CALR	CALR_TSH	TSHR
200	CAMP	CAMP_EGI	EGFR
201	CAMP	CAMP_FPF	FPR2
202	CCL11	CCL11_CX	CXCR3
203	CCL13	CCL13_CX	CXCR3
204	CCL19	CCL19_CC	CCR7
205	CCL19	CCL19_CX	CXCR3
206	CCL20	CCL20_CC	CCR6
207	CCL20	CCL20_CX	CXCR3
208	CCL21	CCL21_CC	CCR7
209	CCL21	CCL21_CX	CXCR3
210	CCL28	CCL28_AC	ACKR2
211	CCL28	CCL28_CC	CCR10
212	CCL28	CCL28_CC	CCR3
213	CCL3	CCL3_ACK	ACKR2
214	CCL3	CCL3_CCR	CCR1
215	CCL3	CCL3_CCR	CCR3
216	CCL3	CCL3_CCR	CCR4
217	CCL3	CCL3_CCR	CCR5
218	CCL3L3	CCL3L3_A	ACKR2
219	CCL3L3	CCL3L3_C	CCR5
220	CCL4	CCL4_ACK	ACKR2
221	CCL4	CCL4_CCR	CCR1
222	CCL4	CCL4_CCR	CCR3
223	CCL4	CCL4_CCR	CCR5
224	CCL4	CCL4_CCR	CCR8
225	CCL5	CCL5_ACK	ACKR2
226	CCL5	CCL5_ACK	ACKR4
227	CCL5	CCL5_CCR	CCR1
228	CCL5	CCL5_CCR	CCR3
229	CCL5	CCL5_CCR	CCR4
230	CCL5	CCL5_CCR	CCR5
231	CCL5	CCL5_CXC	CXCR3

232	CCL5	CCL5_DAR	DARC
233	CCL5	CCL5_GPR	GPR75
234	CCL5	CCL5_SDC	SDC1
235	CCL5	CCL5_SDC	SDC4
236	CCL7	CCL7_CXC	CXCR3
237	CD14	CD14_ITG	ITGA4
238	CD14	CD14_ITG	ITGB1
239	CD24	CD24_SEL	SELP
240	CD34	CD34_SEL	SELL
241	CD40LG	CD40LG_C	CD40
242	CD40LG	CD40LG_I	ITGAM
243	CD40LG	CD40LG_I	ITGB2
244	CD40LG	CD40LG_T	TRAF3
245	CD55	CD55_CD	CD97
246	CD55	CD55_CR1	CR1
247	CD55	CD55_EM	EMR2
248	CD70	CD70_CD	CD27
249	CDH1	CDH1_CD	CDH2
250	CDH1	CDH1_EG	EGFR
251	CDH1	CDH1_ER	ERBB3
252	CDH1	CDH1_KLR	KLRG1
253	CDH1	CDH1_PT	PTPRF
254	CDH1	CDH1_PT	PTPRM
255	CEL	CEL_CXCR	CXCR4
256	CFH	CFH_ITGA	ITGAM
257	CFH	CFH_SELL	SELL
258	CGN	CGN_TGF	TGFBR1
259	CHAD	CHAD_ITG	ITGB1
260	CLEC11A	CLEC11A_	KIT
261	CLEC3A	CLEC3A_C	CLEC10A
262	CLEC3A	CLEC3A_C	CLEC2B
263	COL11A1	COL11A1_	DDR1
264	COL11A1	COL11A1_	ITGB1
265	COL14A1	COL14A1_	CD44
266	COL18A1	COL18A1_	ITGB1
267	COL1A1	COL1A1_C	CD36
268	COL1A1	COL1A1_C	CD44
269	COL1A1	COL1A1_D	DDR1
270	COL1A1	COL1A1_I	ITGB1
271	COL1A2	COL1A2_C	CD36
272	COL1A2	COL1A2_C	CD44
273	COL1A2	COL1A2_I	ITGB1
274	COL2A1	COL2A1_D	DDR1
275	COL2A1	COL2A1_I	ITGB1
276	COL3A1	COL3A1_D	DDR1
277	COL3A1	COL3A1_I	ITGB1
278	COL4A1	COL4A1_C	CD47
279	COL4A1	COL4A1_I	ITGB1
280	COL4A1	COL4A1_I	ITGB8
281	COL4A3	COL4A3_C	CD47
282	COL4A3	COL4A3_I	ITGB1
283	COL4A4	COL4A4_C	CD47
284	COL4A4	COL4A4_C	CD93
285	COL4A4	COL4A4_I	ITGA1
286	COL4A4	COL4A4_I	ITGA2
287	COL4A4	COL4A4_I	ITGAV
288	COL4A4	COL4A4_I	ITGB1
289	COL4A5	COL4A5_C	CD47

290	COL4A5	COL4A5_IT	ITGB1
291	COL4A6	COL4A6_C	CD47
292	COL4A6	COL4A6_IT	ITGB1
293	COL5A1	COL5A1_IT	ITGB1
294	COL5A2	COL5A2_D	DDR1
295	COL5A2	COL5A2_IT	ITGB1
296	COL6A1	COL6A1_IT	ITGB1
297	COL6A2	COL6A2_IT	ITGB1
298	COL6A3	COL6A3_IT	ITGB1
299	COL7A1	COL7A1_IT	ITGB1
300	COL9A3	COL9A3_M	MAG
301	CP	CP_SLC40A	SLC40A1
302	CRP	CRP_OLR1	OLR1
303	CSF1	CSF1_CSF1	CSF1R
304	CSF2	CSF2_CSF1	CSF1R
305	CSF2	CSF2_CSF2	CSF2RA
306	CSF2	CSF2_CSF3	CSF3R
307	CSF2	CSF2_ITGB	ITGB1
308	CSF3	CSF3_CSF1	CSF1R
309	CSF3	CSF3_CSF3	CSF3R
310	CTGF	CTGF_ITGA	ITGAM
311	CTGF	CTGF_ITGB	ITGB2
312	CTGF	CTGF_LRP1	LRP1
313	CTHRC1	CTHRC1_F	FZD3
314	CTHRC1	CTHRC1_F	FZD5
315	CTHRC1	CTHRC1_F	FZD6
316	CUBN	CUBN_LRP	LRP2
317	CXCL10	CXCL10_C	CXCR3
318	CXCL10	CXCL10_SI	SDC4
319	CXCL11	CXCL11_C	CXCR3
320	CXCL12	CXCL12_C	CD4
321	CXCL12	CXCL12_C	CXCR3
322	CXCL12	CXCL12_C	CXCR4
323	CXCL12	CXCL12_IT	ITGB1
324	CXCL12	CXCL12_SI	SDC4
325	CXCL13	CXCL13_C	CXCR3
326	CXCL13	CXCL13_C	CXCR5
327	CXCL16	CXCL16_C	CXCR6
328	CXCL9	CXCL9_CX	CXCR3
329	CYR61	CYR61_CA	CAV1
330	CYR61	CYR61_ITG	ITGAM
331	CYR61	CYR61_ITG	ITGB2
332	DCN	DCN_EGFR	EGFR
333	DCN	DCN_MET	MET
334	DEFB1	DEFB1_CC	CCR6
335	DEFB103A	DEFB103A	CCR6
336	DEFB103B	DEFB103B_	CCR6
337	DEFB4A	DEFB4A_C	CCR6
338	DEFB4A	DEFB4A_TI	TLR4
339	DEFB4B	DEFB4B_C	CCR6
340	DHH	DHH_PTCH	PTCH2
341	DUSP18	DUSP18_C	CD151
342	DUSP18	DUSP18_IT	ITGA3
343	DUSP18	DUSP18_IT	ITGB1
344	DUSP18	DUSP18_R	RPSA
345	EFEMP1	EFEMP1_E	EGFR
346	EFNA1	EFNA1_EP	EPHA1
347	EFNA1	EFNA1_EP	EPHA2

348	EFNA1	EFNA1_EPI	EPHA3
349	EFNA1	EFNA1_EPI	EPHA4
350	EFNA1	EFNA1_EPI	EPHA5
351	EFNA1	EFNA1_EPI	EPHA6
352	EFNA1	EFNA1_EPI	EPHA7
353	EFNA1	EFNA1_EPI	EPHA8
354	EFNA1	EFNA1_EPI	EPHB1
355	EFNA1	EFNA1_EPI	EPHB6
356	EGF	EGF_CAV1	CAV1
357	EGF	EGF_EGFR	EGFR
358	EGF	EGF_ERBB:	ERBB3
359	EPGN	EPGN_EGF	EGFR
360	EREG	EREG_EGFI	EGFR
361	EREG	EREG_ERBI	ERBB2
362	EREG	EREG_ERBI	ERBB3
363	EREG	EREG_ERBI	ERBB4
364	F10	F10_ITGAN	ITGAM
365	F10	F10_ITGB2	ITGB2
366	F13A1	F13A1_ITG	ITGA4
367	F13A1	F13A1_ITG	ITGB1
368	F2	F2_THBD	THBD
369	F8	F8_LRP1	LRP1
370	F9	F9_LRP1	LRP1
371	FASLG	FASLG_FA:	FAS
372	FASLG	FASLG_TN	TNFRSF1A
373	FASLG	FASLG_TN	TNFRSF6B
374	FBLN1	FBLN1_ITG	ITGB1
375	FBN1	FBN1_ITGI	ITGB1
376	FCN2	FCN2_LRP	LRP1
377	FGA	FGA_ITGAI	ITGAM
378	FGA	FGA_ITGA:	ITGAX
379	FGA	FGA_ITGB:	ITGB1
380	FGA	FGA_ITGB:	ITGB2
381	FGA	FGA_PLAU	PLAUR
382	FGB	FGB_ITGAI	ITGAM
383	FGB	FGB_ITGB1	ITGB1
384	FGB	FGB_ITGB2	ITGB2
385	FGF1	FGF1_CD4	CD44
386	FGF1	FGF1_EGFI	EGFR
387	FGF13	FGF13_EGI	EGFR
388	FGF2	FGF2_CD4	CD44
389	FGF2	FGF2_SDC	SDC1
390	FGF2	FGF2_SDC	SDC4
391	FGF6	FGF6_SDC	SDC4
392	FGG	FGG_ITGB:	ITGB1
393	FGG	FGG_ITGB:	ITGB2
394	FGL1	FGL1_EGFI	EGFR
395	FIGF	FIGF_ITGA	ITGA4
396	FIGF	FIGF_ITGB:	ITGB1
397	FN1	FN1_C5AR	C5AR1
398	FN1	FN1_CD44	CD44
399	FN1	FN1_CD79	CD79A
400	FN1	FN1_COL1	COL13A1
401	FN1	FN1_FLT4	FLT4
402	FN1	FN1_IL17R	IL17RC
403	FN1	FN1_ITGA:	ITGA2
404	FN1	FN1_ITGA:	ITGA2B
405	FN1	FN1_ITGA:	ITGA3

406	FN1	FN1_ITGA4	ITGA4
407	FN1	FN1_ITGA5	ITGA5
408	FN1	FN1_ITGA6	ITGA6
409	FN1	FN1_ITGA8	ITGA8
410	FN1	FN1_ITGA9	ITGA9
411	FN1	FN1_ITGAV	ITGAV
412	FN1	FN1_ITGB1	ITGB1
413	FN1	FN1_ITGB3	ITGB3
414	FN1	FN1_ITGB6	ITGB6
415	FN1	FN1_ITGB7	ITGB7
416	FN1	FN1_ITGB8	ITGB8
417	FN1	FN1_MAG	MAG
418	FN1	FN1_NT5E	NT5E
419	FN1	FN1_PLAU	PLAUR
420	FN1	FN1_ROBC	ROBO4
421	FN1	FN1_SDC2	SDC2
422	FN1	FN1_TMPF	TMPRSS6
423	FN1	FN1_TNFR	TNFRSF11B
424	FN1	FN1_TSHR	TSHR
425	GAS6	GAS6_AXL	AXL
426	GAS6	GAS6_MEF	MERTK
427	GDF9	GDF9_OR/	ORAI2
428	GDF9	GDF9_TGF	TGFBR1
429	GIP	GIP_INSR	INSR
430	GNAI2	GNAI2_AD	ADCY1
431	GNAI2	GNAI2_AD	ADCY7
432	GNAI2	GNAI2_AD	ADCY8
433	GNAI2	GNAI2_AD	ADCY9
434	GNAI2	GNAI2_AD	ADORA1
435	GNAI2	GNAI2_AD	ADRA2A
436	GNAI2	GNAI2_AD	ADRA2B
437	GNAI2	GNAI2_AG	AGTR2
438	GNAI2	GNAI2_C5.	C5AR1
439	GNAI2	GNAI2_CA	CAV1
440	GNAI2	GNAI2_CC	CCR5
441	GNAI2	GNAI2_CH	CHRM1
442	GNAI2	GNAI2_CN	CNR1
443	GNAI2	GNAI2_CX	CXCR1
444	GNAI2	GNAI2_CX	CXCR2
445	GNAI2	GNAI2_CX	CXCR3
446	GNAI2	GNAI2_DR	DRD2
447	GNAI2	GNAI2_ED	EDNRA
448	GNAI2	GNAI2_ED	EDNRB
449	GNAI2	GNAI2_EG	EGFR
450	GNAI2	GNAI2_F2I	F2R
451	GNAI2	GNAI2_FPI	FPR1
452	GNAI2	GNAI2_IGI	IGF1R
453	GNAI2	GNAI2_LH	LHCGR
454	GNAI2	GNAI2_LP/	LPAR3
455	GNAI2	GNAI2_MT	MTNR1A
456	GNAI2	GNAI2_MT	MTNR1B
457	GNAI2	GNAI2_OP	OPRD1
458	GNAI2	GNAI2_OP	OPRM1
459	GNAI2	GNAI2_P2I	P2RY12
460	GNAI2	GNAI2_PTI	PTPRU
461	GNAI2	GNAI2_S1I	S1PR1
462	GNAI2	GNAI2_S1I	S1PR3
463	GNAI2	GNAI2_S1I	S1PR4

464	GNAI2	GNAI2_S1I	S1PR5
465	GNAI2	GNAI2_TB	TBXA2R
466	GNAI2	GNAI2_TSI	TSHR
467	GNAI2	GNAI2_UN	UNC5B
468	GNAS	GNAS_AD	ADCY1
469	GNAS	GNAS_AD	ADCY7
470	GNAS	GNAS_AD	ADCY8
471	GNAS	GNAS_AD	ADCY9
472	GNAS	GNAS_AD	ADORA1
473	GNAS	GNAS_ADI	ADRB3
474	GNAS	GNAS_AVI	AVPR2
475	GNAS	GNAS_CRI	CRHR1
476	GNAS	GNAS_GC	GCGR
477	GNAS	GNAS_GLI	GLP1R
478	GNAS	GNAS_HTI	HTR6
479	GNAS	GNAS_LH	LHCGR
480	GNAS	GNAS_PT	PTGDR
481	GNAS	GNAS_PT	PTGIR
482	GNAS	GNAS_TSI	TSHR
483	GNAS	GNAS_VIP	VIPR1
484	GNB3	GNB3_TGI	TGFBR1
485	GPC3	GPC3_CD	CD81
486	GSTP1	GSTP1_TR	TRAF2
487	GZMB	GZMB_CH	CHRM3
488	GZMB	GZMB_IGF	IGF2R
489	GZMB	GZMB_PGI	PGRMC1
490	HAS2	HAS2_CD	CD44
491	HAS2	HAS2_HM	HMMR
492	HBEGF	HBEGF_CD	CD44
493	HBEGF	HBEGF_CD	CD82
494	HBEGF	HBEGF_CD	CD9
495	HBEGF	HBEGF_EG	EGFR
496	HBEGF	HBEGF_ER	ERBB2
497	HBEGF	HBEGF_ER	ERBB4
498	HBEGF	HBEGF_PR	PRLR
499	HDC	HDC_HRH	HRH1
500	HDC	HDC_HRH	HRH2
501	HDC	HDC_HRH	HRH3
502	HDC	HDC_HRH	HRH4
503	HEBP1	HEBP1_FPI	FPR3
504	HGF	HGF_CD44	CD44
505	HGF	HGF_MET	MET
506	HGF	HGF_SDC1	SDC1
507	HGF	HGF_ST14	ST14
508	HLA-A	HLA-A_AP	APLP2
509	HLA-A	HLA-A_CD	CD3D
510	HLA-A	HLA-A_CD	CD3G
511	HLA-A	HLA-A_ER	ERBB2
512	HLA-A	HLA-A_KII	KIR2DL1
513	HLA-A	HLA-A_KII	KIR2DL3
514	HLA-A	HLA-A_KII	KIR3DL1
515	HLA-A	HLA-A_KII	KIR3DL2
516	HLA-A	HLA-A_LIL	LILRB1
517	HLA-A	HLA-A_LIL	LILRB2
518	HLA-B	HLA-B_CA	CANX
519	HLA-B	HLA-B_CD	CD3D
520	HLA-B	HLA-B_CD	CD3G
521	HLA-B	HLA-B_KII	KIR2DL3

522	HLA-B	HLA-B_KIF	KIR3DL1
523	HLA-B	HLA-B_KLI	KLRD1
524	HLA-B	HLA-B_LIL	LILRB1
525	HLA-B	HLA-B_LIL	LILRB2
526	HLA-C	HLA-C_CD	CD3D
527	HLA-C	HLA-C_CD	CD3G
528	HLA-C	HLA-C_DD	DDR1
529	HLA-C	HLA-C_KIF	KIR2DL1
530	HLA-C	HLA-C_KIF	KIR2DL3
531	HLA-C	HLA-C_KIF	KIR2DS4
532	HLA-C	HLA-C_KIF	KIR3DL1
533	HLA-C	HLA-C_LIL	LILRA3
534	HLA-C	HLA-C_LIL	LILRB1
535	HLA-C	HLA-C_LIL	LILRB2
536	HLA-C	HLA-C_NC	NOTCH4
537	HLA-C	HLA-C_SL	SLC9C2
538	HLA-E	HLA-E_KIF	KIR3DL1
539	HLA-E	HLA-E_KLI	KLRC1
540	HLA-E	HLA-E_KLI	KLRC2
541	HLA-E	HLA-E_KLI	KLRD1
542	HLA-E	HLA-E_SL	SLC16A4
543	HLA-G	HLA-G_CD	CD4
544	HLA-G	HLA-G_KL	KLRD1
545	HLA-G	HLA-G_LIL	LILRB1
546	HLA-G	HLA-G_LIL	LILRB2
547	HMGB1	HMGB1_SI	SDC1
548	HMGB1	HMGB1_TI	THBD
549	HP	HP_ASGR1	ASGR1
550	HP	HP_ASGR2	ASGR2
551	HP	HP_ITGAM	ITGAM
552	HP	HP_ITGB2	ITGB2
553	HRAS	HRAS_CA\	CAV1
554	HRAS	HRAS_INSI	INSR
555	HRAS	HRAS_TLR	TLR2
556	HRG	HRG_FCGF	FCGR1A
557	HSP90AA1	HSP90AA1	CFTR
558	HSP90AA1	HSP90AA1	EGFR
559	HSP90AA1	HSP90AA1	FGFR3
560	HSP90B1	HSP90B1_	ASGR1
561	HSP90B1	HSP90B1_	ERBB2
562	HSP90B1	HSP90B1_	LRP1
563	HSP90B1	HSP90B1_	TLR1
564	HSP90B1	HSP90B1_	TLR2
565	HSP90B1	HSP90B1_	TLR4
566	HSP90B1	HSP90B1_	TLR7
567	HSP90B1	HSP90B1_	TLR9
568	HSPA1A	HSPA1A_G	GRIN2D
569	HSPA1A	HSPA1A_T	TLR4
570	HSPG2	HSPG2_IT	ITGB1
571	HSPG2	HSPG2_SD	SDC1
572	ICAM1	ICAM1_CA	CAV1
573	ICAM1	ICAM1_EG	EGFR
574	ICAM1	ICAM1_IL2	IL2RA
575	ICAM1	ICAM1_IL2	IL2RG
576	ICAM1	ICAM1_IT	ITGAL
577	ICAM1	ICAM1_IT	ITGAM
578	ICAM1	ICAM1_IT	ITGAX
579	ICAM1	ICAM1_IT	ITGB2

580	ICAM2	ICAM2_ITC	ITGAL
581	ICAM2	ICAM2_ITC	ITGAM
582	ICAM2	ICAM2_ITC	ITGB2
583	ICAM3	ICAM3_CL	CLEC4M
584	ICAM3	ICAM3_ITC	ITGAD
585	ICAM3	ICAM3_ITC	ITGAL
586	ICAM3	ICAM3_ITC	ITGB2
587	ICAM4	ICAM4_ITC	ITGA4
588	ICAM4	ICAM4_ITC	ITGAL
589	ICAM4	ICAM4_ITC	ITGAM
590	ICAM4	ICAM4_ITC	ITGB1
591	ICAM4	ICAM4_ITC	ITGB2
592	ICAM5	ICAM5_ITC	ITGAL
593	ICAM5	ICAM5_ITC	ITGB2
594	IFNG	IFNG_IFNG	IFNGR1
595	IFNG	IFNG_IFNG	IFNGR2
596	IGF1	IGF1_INSR	INSR
597	IGF2	IGF2_INSR	INSR
598	IGFBP4	IGFBP4_FZ	FZD8
599	IGFBP4	IGFBP4_LR	LRP6
600	IHH	IHH_PTCH	PTCH2
601	IL10	IL10_IL10R	IL10RA
602	IL10	IL10_SIRPC	SIRPG
603	IL12A	IL12A_CD2	CD28
604	IL12A	IL12A_IL12	IL12RB1
605	IL12A	IL12A_IL12	IL12RB2
606	IL13	IL13_IL13R	IL13RA1
607	IL13	IL13_IL2RC	IL2RG
608	IL15	IL15_IL2RA	IL2RA
609	IL15	IL15_IL2RB	IL2RB
610	IL15	IL15_IL2RC	IL2RG
611	IL16	IL16_CCR5	CCR5
612	IL16	IL16_CD4	CD4
613	IL16	IL16_GRIN	GRIN2C
614	IL16	IL16_GRIN	GRIN2D
615	IL16	IL16_KCNA	KCNA3
616	IL16	IL16_KCND	KCND1
617	IL16	IL16_KCND	KCND2
618	IL16	IL16_KCNJ	KCNJ10
619	IL16	IL16_KCNJ	KCNJ15
620	IL16	IL16_KCNJ	KCNJ4
621	IL18	IL18_CD48	CD48
622	IL18	IL18_IL18R	IL18R1
623	IL18	IL18_IL18R	IL18RAP
624	IL18	IL18_IL1RA	IL1RAPL1
625	IL18	IL18_IL1RL	IL1RL2
626	IL1A	IL1A_IL1R2	IL1R2
627	IL1B	IL1B_ADRE	ADRB2
628	IL1B	IL1B_IL1R1	IL1R1
629	IL1B	IL1B_IL1R2	IL1R2
630	IL1B	IL1B_IL1RA	IL1RAP
631	IL1RN	IL1RN_IL1F	IL1R2
632	IL2	IL2_CD53	CD53
633	IL2	IL2_IL2RA	IL2RA
634	IL2	IL2_IL2RB	IL2RB
635	IL2	IL2_IL2RG	IL2RG
636	IL21	IL21_IL2RC	IL2RG
637	IL22	IL22_IL10R	IL10RA

638	IL34	IL34_CSF1I	CSF1R
639	IL4	IL4_CD53	CD53
640	IL4	IL4_IL13RA	IL13RA1
641	IL4	IL4_IL2RG	IL2RG
642	IL5	IL5_IL5RA	IL5RA
643	IL6	IL6_F3	F3
644	IL6	IL6_IL6R	IL6R
645	IL6	IL6_IL6ST	IL6ST
646	IL7	IL7_IL2RG	IL2RG
647	IL7	IL7_IL7R	IL7R
648	IL8	IL8_CD79A	CD79A
649	IL8	IL8_SDC1	SDC1
650	IL9	IL9_IL2RG	IL2RG
651	INS	INS_INSR	INSR
652	ITIH2	ITIH2_FCEI	FCER1A
653	KISS1	KISS1_KISS	KISS1R
654	KITLG	KITLG_KIT	KIT
655	KNG1	KNG1_ITG	ITGAM
656	KNG1	KNG1_ITGI	ITGB2
657	KNG1	KNG1_PLA	PLAUR
658	L1CAM	L1CAM_EG	EGFR
659	L1CAM	L1CAM_EF	ERBB3
660	LACRT	LACRT_SD	SDC1
661	LAMA1	LAMA1_IT	ITGA3
662	LAMA1	LAMA1_IT	ITGB1
663	LAMA1	LAMA1_IT	ITGB8
664	LAMA1	LAMA1_N	NT5E
665	LAMA1	LAMA1_RI	RPSA
666	LAMA1	LAMA1_SI	SDC4
667	LAMA2	LAMA2_IT	ITGA3
668	LAMA2	LAMA2_IT	ITGB1
669	LAMA2	LAMA2_RI	RPSA
670	LAMA4	LAMA4_IT	ITGA3
671	LAMA4	LAMA4_IT	ITGB1
672	LAMA5	LAMA5_IT	ITGA3
673	LAMA5	LAMA5_IT	ITGB1
674	LAMA5	LAMA5_SI	SDC1
675	LAMB1	LAMB1_IT	ITGA3
676	LAMB1	LAMB1_IT	ITGB1
677	LAMB2	LAMB2_RI	RPSA
678	LAMB3	LAMB3_CI	CD151
679	LAMB3	LAMB3_IT	ITGA3
680	LAMB3	LAMB3_IT	ITGB1
681	LAMC1	LAMC1_IT	ITGA3
682	LAMC1	LAMC1_IT	ITGB1
683	LAMC2	LAMC2_CI	CD151
684	LAMC2	LAMC2_IT	ITGA3
685	LAMC2	LAMC2_IT	ITGB1
686	LAMC3	LAMC3_IT	ITGA3
687	LAMC3	LAMC3_IT	ITGB1
688	LGALS3BP	LGALS3BP	ITGB1
689	LGALS3BP	LGALS3BP	VANGL1
690	LIPC	LIPC_LRP1	LRP1
691	LPA	LPA_ITGAI	ITGAM
692	LPA	LPA_ITGB2	ITGB2
693	LPL	LPL_CD44	CD44
694	LPL	LPL_GPIHE	GPIHBP1
695	LPL	LPL_LRP1	LRP1

696	LPL	LPL_LRP2	LRP2
697	LPL	LPL_SDC1	SDC1
698	LPL	LPL_VLDLF	VLDLR
699	LRP1B	LRP1B_PLA	PLAUR
700	LRPAP1	LRPAP1_LF	LRP1
701	LRPAP1	LRPAP1_S(	SORL1
702	LTA	LTA_LTBR	LTBR
703	LTA	LTA_TNFRS	TNFRSF1B
704	LTB	LTB_CD40	CD40
705	LTB	LTB_LTBR	LTBR
706	LTB	LTB_TNFRS	TNFRSF1A
707	LTF	LTF_LRP1	LRP1
708	LY86	LY86_CD18	CD180
709	LYZ	LYZ_ITGAL	ITGAL
710	MADCAM1	MADCAM1	CD44
711	MADCAM1	MADCAM1	ITGA4
712	MATN1	MATN1_IT	ITGB1
713	MDK	MDK_ITGA	ITGA4
714	MDK	MDK_ITGB	ITGB1
715	MDK	MDK_LRP1	LRP1
716	MDK	MDK_SDC1	SDC1
717	MDK	MDK_SDC4	SDC4
718	MDK	MDK_TSPA	TSPAN1
719	MMP1	MMP1_CD	CD44
720	MMP12	MMP12_PI	PLAUR
721	MMP13	MMP13_LF	LRP1
722	MMP2	MMP2_SD	SDC2
723	MMP7	MMP7_CD	CD151
724	MMP7	MMP7_CD	CD44
725	MMP7	MMP7_CD	CDH6
726	MMP7	MMP7_ERI	ERBB4
727	MMP9	MMP9_CD	CD44
728	MMP9	MMP9_ITG	ITGAM
729	MMP9	MMP9_ITG	ITGB2
730	MMP9	MMP9_LRI	LRP1
731	MST1	MST1_MST	MST1R
732	MUC7	MUC7_SEL	SELL
733	NAMPT	NAMPT_IN	INSR
734	NCAM1	NCAM1_PT	PTPRA
735	NCAN	NCAN_CD	CDH2
736	NGF	NGF_NGFI	NGFRAP1
737	NID1	NID1_ITGA	ITGA3
738	NID1	NID1_ITGE	ITGB1
739	NID1	NID1_PTPI	PTPRF
740	NPNT	NPNT_ITG	ITGB1
741	NRG1	NRG1_ERB	ERBB3
742	NRG2	NRG2_ERB	ERBB3
743	NRG4	NRG4_EGF	EGFR
744	NTF4	NTF4_NGF	NGFRAP1
745	NUCB2	NUCB2_ER	ERAP1
746	OMG	OMG_TNF	TNFRSF1B
747	P4HB	P4HB_GPR	GPR162
748	PDGFB	PDGFB_LR	LRP1
749	PF4	PF4_CXCR	CXCR3
750	PF4	PF4_THBD	THBD
751	PIP	PIP_CD4	CD4
752	PKM	PKM_CD44	CD44
753	PLAT	PLAT_ITGA	ITGAM

754	PLAT	PLAT_ITGE	ITGB2
755	PLAT	PLAT_LRP:	LRP1
756	PLAU	PLAU_ITGA	ITGA3
757	PLAU	PLAU_ITGA	ITGAM
758	PLAU	PLAU_ITGE	ITGB1
759	PLAU	PLAU_ITGE	ITGB2
760	PLAU	PLAU_LRP.	LRP1
761	PLAU	PLAU_PLA	PLAUR
762	PLAU	PLAU_ST1	ST14
763	PLG	PLG_ITGAI	ITGAM
764	PLG	PLG_ITGB1	ITGB1
765	PLG	PLG_ITGB2	ITGB2
766	PLG	PLG_PLAU	PLAUR
767	PLG	PLG_PLGR	PLGRKT
768	PLTP	PLTP_ABC.	ABCA1
769	PNOC	PNOC_OP	OPRL1
770	PODXL	PODXL_SE	SELL
771	PODXL2	PODXL2_S	SELL
772	PRND	PRND_RPS	RPSA
773	PROC	PROC_ITG.	ITGAM
774	PROC	PROC_ITG	ITGB2
775	PROC	PROC_THE	THBD
776	PROS1	PROS1_AX	AXL
777	PROS1	PROS1_TY	TYRO3
778	PSAP	PSAP_CD1	CD1B
779	PSAP	PSAP_CEL	CELSR1
780	PSAP	PSAP_GPR	GPR37
781	PSAP	PSAP_GPR	GPR37L1
782	PSAP	PSAP_LRP:	LRP1
783	PSAP	PSAP_SOR	SORT1
784	PSEN1	PSEN1_CD	CD44
785	PTGS2	PTGS2_AL	ALOX5
786	PTGS2	PTGS2_CA	CAV1
787	PTMA	PTMA_VIP	VIPR1
788	PTN	PTN_PLXN	PLXNB2
789	PTN	PTN_SDC1	SDC1
790	RARRES2	RARRES2_	CCRL2
791	RARRES2	RARRES2_	CMKLR1
792	RARRES2	RARRES2_	GPR1
793	RELN	RELN_ITGA	ITGA3
794	RELN	RELN_ITGE	ITGB1
795	REN	REN_ATP6	ATP6AP2
796	RIMS1	RIMS1_SL	SLC18A2
797	RNASE2	RNASE2_T	TLR2
798	RPS19	RPS19_C5	C5AR1
799	RSPO3	RSPO3_SD	SDC4
800	RTN4	RTN4_CN	CNTNAP1
801	RTN4	RTN4_GJB	GJB2
802	RTN4	RTN4_LIN	LINGO1
803	RTN4	RTN4_NGI	NGFR
804	RTN4	RTN4_RTN	RTN4R
805	RTN4	RTN4_RTN	RTN4RL1
806	RTN4	RTN4_TNF	TNFRSF19
807	S100A8	S100A8_TL	TLR4
808	S100A9	S100A9_TL	TLR4
809	SAA1	SAA1_FPR	FPR1
810	SAA1	SAA1_FPR	FPR2
811	SAA1	SAA1_SCA	SCARB1

812	SELPLG	SELPLG_ES	ESAM
813	SELPLG	SELPLG_IT(	ITGAM
814	SELPLG	SELPLG_IT(	ITGB2
815	SELPLG	SELPLG_SE	SELE
816	SELPLG	SELPLG_SE	SELL
817	SELPLG	SELPLG_SE	SELP
818	SEMA4A	SEMA4A_F	PLXND1
819	SEMA4B	SEMA4B_D	DCBLD2
820	SEMA4C	SEMA4C_F	PLXNB2
821	SEMA4D	SEMA4D_N	MET
822	SEMA4D	SEMA4D_F	PLXNB2
823	SEMA4G	SEMA4G_F	PLXNB2
824	SEMA5A	SEMA5A_N	MET
825	SEMA6D	SEMA6D_T	TREM2
826	SEMA6D	SEMA6D_T	TYROBP
827	SEMA7A	SEMA7A_I	ITGB1
828	SEMA7A	SEMA7A_F	PLXNC1
829	SERPINA1	SERPINA1_	LRP1
830	SERPINC1	SERPINC1_	LRP1
831	SERPINE1	SERPINE1_	LRP1
832	SERPINE1	SERPINE1_	PLAUR
833	SERPINE2	SERPINE2_	LRP1
834	SERPING1	SERPING1_	LRP1
835	SERPING1	SERPING1_	SELE
836	SERPING1	SERPING1_	SELP
837	SFTPA1	SFTPA1_TL	TLR2
838	SFTPD	SFTPD_LYS	LY96
839	SFTPD	SFTPD_TLF	TLR4
840	SHBG	SHBG_CLD	CLDN4
841	SHH	SHH_PTCH	PTCH2
842	SLIT1	SLIT1_SDC	SDC1
843	SLIT2	SLIT2_SDC	SDC1
844	SLPI	SLPI_CD4	CD4
845	SORBS1	SORBS1_IN	INSR
846	SPINK1	SPINK1_EG	EGFR
847	SPINT1	SPINT1_ST	ST14
848	SPON2	SPON2_IT(	ITGAM
849	SPON2	SPON2_IT(	ITGB2
850	SPP1	SPP1_CD4	CD44
851	SPP1	SPP1_ITGA	ITGA4
852	SPP1	SPP1_ITGA	ITGA5
853	SPP1	SPP1_ITGA	ITGA9
854	SPP1	SPP1_ITGA	ITGAV
855	SPP1	SPP1_ITGB	ITGB1
856	SPP1	SPP1_S1PF	S1PR1
857	SYTL3	SYTL3_NR)	NRXN1
858	TCN2	TCN2_CNF	CNR1
859	TCN2	TCN2_LRP	LRP2
860	TFPI	TFPI_F3	F3
861	TFPI	TFPI_LRP1	LRP1
862	TFPI	TFPI_SDC4	SDC4
863	TFPI	TFPI_VLDL	VLDLR
864	TG	TG_ASGR1	ASGR1
865	TGFA	TGFA_EGF	EGFR
866	TGFA	TGFA_ERB	ERBB3
867	TGFB1	TGFB1_CA	CAV1
868	TGFB1	TGFB1_CX(	CXCR4
869	TGFB1	TGFB1_ITG	ITGB8

870	TGFB1	TGFB1_TG	TGFBR1
871	TGFB2	TGFB2_TG	TGFBR1
872	TGFB3	TGFB3_TG	TGFBR1
873	TGM2	TGM2_GPI	GPR56
874	TGM2	TGM2_ITG	ITGA4
875	TGM2	TGM2_ITG	ITGA9
876	TGM2	TGM2_ITG	ITGB1
877	TGM2	TGM2_ITG	ITGB3
878	TGM2	TGM2_SD(	SDC4
879	TGM2	TGM2_TB)	TBXA2R
880	THBS1	THBS1_CD	CD36
881	THBS1	THBS1_CD	CD47
882	THBS1	THBS1_ITC	ITGA2B
883	THBS1	THBS1_ITC	ITGA3
884	THBS1	THBS1_ITC	ITGA4
885	THBS1	THBS1_ITC	ITGA6
886	THBS1	THBS1_ITC	ITGB1
887	THBS1	THBS1_ITC	ITGB3
888	THBS1	THBS1_LRI	LRP1
889	THBS1	THBS1_LRI	LRP5
890	THBS1	THBS1_SC.	SCARB1
891	THBS1	THBS1_SD	SDC1
892	THBS1	THBS1_SD	SDC4
893	THBS1	THBS1_TN	TNFRSF11B
894	THBS2	THBS2_CD	CD47
895	THBS2	THBS2_ITC	ITGA4
896	THBS2	THBS2_ITC	ITGB1
897	TIMP1	TIMP1_CD	CD63
898	TIMP2	TIMP2_ITC	ITGA3
899	TIMP2	TIMP2_ITC	ITGB1
900	TNC	TNC_EGFR	EGFR
901	TNC	TNC_ITGB:	ITGB1
902	TNC	TNC_NT5E	NT5E
903	TNC	TNC_SDC1	SDC1
904	TNC	TNC_SDC4	SDC4
905	TNF	TNF_LTBR	LTBR
906	TNF	TNF_TNFR	TNFRSF1B
907	TNFSF10	TNFSF10_T	TNFRSF10A
908	TNFSF10	TNFSF10_T	TNFRSF10B
909	TNFSF10	TNFSF10_T	TNFRSF10C
910	TNFSF10	TNFSF10_T	TNFRSF10D
911	TNFSF10	TNFSF10_T	TNFRSF11B
912	TNFSF11	TNFSF11_T	TNFRSF11B
913	TNFSF12	TNFSF12_T	TNFRSF12A
914	TNFSF12	TNFSF12_T	TNFRSF25
915	TNFSF13	TNFSF13_T	TNFRSF11B
916	TNFSF13	TNFSF13_T	TNFRSF13B
917	TNFSF13	TNFSF13_T	TNFRSF17
918	TNFSF13B	TNFSF13B_	TNFRSF13B
919	TNFSF13B	TNFSF13B_	TNFRSF13C
920	TNFSF13B	TNFSF13B_	TNFRSF17
921	TNFSF14	TNFSF14_L	LTBR
922	TNFSF18	TNFSF18_T	TNFRSF18
923	TNFSF4	TNFSF4_TN	TNFRSF4
924	TNFSF9	TNFSF9_TN	TNFRSF9
925	TSLP	TSLP_IL7R	IL7R
926	UBA52	UBA52_AC	ACVR1
927	UBA52	UBA52_AG	AGTR1

928	UBA52	UBA52_BM	BMPR1B
929	UBA52	UBA52_EG	EGFR
930	UBA52	UBA52_ER	ERBB2
931	UBA52	UBA52_FSI	FSHR
932	UBA52	UBA52_NC	NOTCH1
933	UBA52	UBA52_TG	TGFBR1
934	UBA52	UBA52_TG	TGFBR2
935	VASP	VASP_CXC	CXCR2
936	VCAM1	VCAM1_IT	ITGA4
937	VCAM1	VCAM1_IT	ITGA9
938	VCAM1	VCAM1_IT	ITGAD
939	VCAM1	VCAM1_IT	ITGB1
940	VCAM1	VCAM1_IT	ITGB2
941	VCAM1	VCAM1_IT	ITGB7
942	VCAN	VCAN_CD4	CD44
943	VCAN	VCAN_EGI	EGFR
944	VCAN	VCAN_ITG	ITGA4
945	VCAN	VCAN_ITG	ITGB1
946	VCAN	VCAN_SEL	SELL
947	VCAN	VCAN_SEL	SELP
948	VCAN	VCAN_TLR	TLR1
949	VCAN	VCAN_TLR	TLR2
950	VEGFA	VEGFA_EG	EGFR
951	VEGFA	VEGFA_EP	EPHB2
952	VEGFA	VEGFA_FLT	FLT1
953	VEGFA	VEGFA_GP	GPC1
954	VEGFA	VEGFA_IT(	ITGA9
955	VEGFA	VEGFA_IT(	ITGAV
956	VEGFA	VEGFA_IT(	ITGB1
957	VEGFA	VEGFA_KD	KDR
958	VEGFA	VEGFA_NF	NRP1
959	VEGFA	VEGFA_NF	NRP2
960	VEGFA	VEGFA_RE	RET
961	VEGFA	VEGFA_SIF	SIRPA
962	VEGFA	VEGFA_TY	TYRO3
963	VEGFC	VEGFC_IT(	ITGB1
964	VIM	VIM_CD44	CD44
965	VTN	VTN_CD47	CD47
966	VTN	VTN_ITGA:	ITGA3
967	VTN	VTN_ITGB:	ITGB1
968	VTN	VTN_ITGB8	ITGB8
969	VTN	VTN_PLAU	PLAUR
970	VTN	VTN_TNFR	TNFRSF11B
971	VWF	VWF_TNFF	TNFRSF11B
972	WNT3A	WNT3A_A	ATP6AP2
973	WNT3A	WNT3A_LF	LRP1
974	XCL1	XCL1_XCR	XCR1
975	XCL2	XCL2_XCR	XCR1
976	ZG16B	ZG16B_CX	CXCR4
977	ZG16B	ZG16B_TLI	TLR2
978	ZG16B	ZG16B_TLI	TLR4
979	ZG16B	ZG16B_TLI	TLR5
980	ZP3	ZP3_EGFR	EGFR
981	ZP3	ZP3_MERT	MERTK
