# Supplementary material for: Single-Cell and Transcriptome-Based Immune Cell-Related Prognostic Model in Clear Cell Renal Cell Carcinoma
Source: J Oncol. 2023 Mar 7;2023:5355269. doi: 10.1155/2023/5355269 (PMC10014191; doi:10.1155/2023/5355269)
Supplement: Supplementary Materials — Supplementary Table 1: Notes on cell clustering. Supplementary Table 2: Differential genes in each cell cluster. Supplementary Table 3: Ligand-receptor relationship pair. Supplementary Table 4: Immune cell multifactor network relationship pair. Supplementary Table 5: Intersection genes in immune cell multifactor network relationship pair and TCGA. Supplementary Table 6: Genes in black and magenta models of WGCNA. [file 5355269.f1.zip › Supplementary Table 4. Immune cell multifactor network relationship pair.pdf]

	TF	Ligand.Symbol	Receptor.Symbol	Pair.Name
1	STAT3	A2M	LRP1	A2M_LRP1
2	TFCP2	A2M	LRP1	A2M_LRP1
3	NFKB1	A2M	LRP1	A2M_LRP1
4	SP1	ADAM10	AXL	ADAM10_AXL
5	PAX2	ADAM10	AXL	ADAM10_AXL
6	SKIL	ADAM12	SDC4	ADAM12_SDC4
7	SKIL	ADAM12	ITGB1	ADAM12_ITGB1
8	SP1	ADAM17	ITGB1	ADAM17_ITGB1
9	CEBPB	ADM	RAMP2	ADM_RAMP2
10	CEBPB	ADM	CALCR	ADM_CALCR
11	CEBPB	ADM	GPR182	ADM_GPR182
12	CEBPB	ADM	MRGPRX2	ADM_MRGPRX2
13	CEBPB	ADM	CALCRL	ADM_CALCRL
14	TFAP2A	ADM	RAMP2	ADM_RAMP2
15	TFAP2A	ADM	CALCR	ADM_CALCR
16	TFAP2A	ADM	GPR182	ADM_GPR182
17	TFAP2A	ADM	MRGPRX2	ADM_MRGPRX2
18	TFAP2A	ADM	CALCRL	ADM_CALCRL
19	GTF3A	ADM	RAMP2	ADM_RAMP2
20	GTF3A	ADM	CALCR	ADM_CALCR
21	GTF3A	ADM	GPR182	ADM_GPR182
22	GTF3A	ADM	MRGPRX2	ADM_MRGPRX2
23	GTF3A	ADM	CALCRL	ADM_CALCRL
24	NFKB1	ALOX5AP	ALOX5	ALOX5AP_ALOX5
25	HIF1A	ALOX5AP	ALOX5	ALOX5AP_ALOX5
26	RELA	ALOX5AP	ALOX5	ALOX5AP_ALOX5
27	SF1	AMH	EGFR	AMH_EGFR
28	NFKB1	AMH	EGFR	AMH_EGFR
29	GATA4	AMH	EGFR	AMH_EGFR
30	NR5A1	AMH	EGFR	AMH_EGFR
31	RELA	AMH	EGFR	AMH_EGFR
32	WT1	AMH	EGFR	AMH_EGFR
33	ESR1	AMH	EGFR	AMH_EGFR
34	SOX9	AMH	EGFR	AMH_EGFR
35	VDR	AMH	EGFR	AMH_EGFR
36	NR0B1	AMH	EGFR	AMH_EGFR
37	ING4	ANGPT1	ITGB1	ANGPT1_ITGB1
38	SMAD3	ANGPTL4	TIE1	ANGPTL4_TIE1
39	PPARD	ANGPTL4	TIE1	ANGPTL4_TIE1
40	PPARG	ANGPTL4	TIE1	ANGPTL4_TIE1
41	PPARA	ANGPTL4	TIE1	ANGPTL4_TIE1
42	NR2F1	APOB	ITGB2	APOB_ITGB2
43	NR2F1	APOB	OLR1	APOB_OLR1
44	NR2F1	APOB	ITGAM	APOB_ITGAM
45	NR2F6	APOB	ITGB2	APOB_ITGB2
46	NR2F6	APOB	OLR1	APOB_OLR1
47	NR2F6	APOB	ITGAM	APOB_ITGAM
48	FOXA1	APOB	ITGB2	APOB_ITGB2
49	FOXA1	APOB	OLR1	APOB_OLR1
50	FOXA1	APOB	ITGAM	APOB_ITGAM
51	PPARG	APOB	ITGB2	APOB_ITGB2
52	PPARG	APOB	OLR1	APOB_OLR1
53	PPARG	APOB	ITGAM	APOB_ITGAM
54	PPARA	APOB	ITGB2	APOB_ITGB2
55	PPARA	APOB	OLR1	APOB_OLR1
56	PPARA	APOB	ITGAM	APOB_ITGAM
57	HNF4A	APOB	ITGB2	APOB_ITGB2

58	HNF4A	APOB	OLR1	APOB_OLR1
59	HNF4A	APOB	ITGAM	APOB_ITGAM
60	ATF2	APOC3	TLR2	APOC3_TLR2
61	NR2F6	APOC3	TLR2	APOC3_TLR2
62	NR2F1	APOC3	TLR2	APOC3_TLR2
63	HDAC1	APOC3	TLR2	APOC3_TLR2
64	SP1	APOC3	TLR2	APOC3_TLR2
65	JUN	APOC3	TLR2	APOC3_TLR2
66	HNF4A	APOC3	TLR2	APOC3_TLR2
67	HDAC3	APOC3	TLR2	APOC3_TLR2
68	SMAD3	APOC3	TLR2	APOC3_TLR2
69	PPARGC1A	APOC3	TLR2	APOC3_TLR2
70	PPARA	APOC3	TLR2	APOC3_TLR2
71	SMAD4	APOC3	TLR2	APOC3_TLR2
72	NR0B2	APOC3	TLR2	APOC3_TLR2
73	SP1	APOE	LRP5	APOE_LRP5
74	SP1	APOE	LRP2	APOE_LRP2
75	SP1	APOE	CHRNA4	APOE_CHRNA4
76	SP1	APOE	LDLR	APOE_LDLR
77	SP1	APOE	LRP1	APOE_LRP1
78	SP1	APOE	SCARB1	APOE_SCARB1
79	SP1	APOE	LRP8	APOE_LRP8
80	SP1	APOE	VLDLR	APOE_VLDLR
81	SP1	APOE	SORL1	APOE_SORL1
82	FOXM1	APOE	LRP5	APOE_LRP5
83	FOXM1	APOE	LRP2	APOE_LRP2
84	FOXM1	APOE	CHRNA4	APOE_CHRNA4
85	FOXM1	APOE	LDLR	APOE_LDLR
86	FOXM1	APOE	LRP1	APOE_LRP1
87	FOXM1	APOE	SCARB1	APOE_SCARB1
88	FOXM1	APOE	LRP8	APOE_LRP8
89	FOXM1	APOE	VLDLR	APOE_VLDLR
90	FOXM1	APOE	SORL1	APOE_SORL1
91	ATF4	APOE	LRP5	APOE_LRP5
92	ATF4	APOE	LRP2	APOE_LRP2
93	ATF4	APOE	CHRNA4	APOE_CHRNA4
94	ATF4	APOE	LDLR	APOE_LDLR
95	ATF4	APOE	LRP1	APOE_LRP1
96	ATF4	APOE	SCARB1	APOE_SCARB1
97	ATF4	APOE	LRP8	APOE_LRP8
98	ATF4	APOE	VLDLR	APOE_VLDLR
99	ATF4	APOE	SORL1	APOE_SORL1
100	ETS2	APP	LRP1	APP_LRP1
101	ETS2	APP	NGFR	APP_NGFR
102	ETS2	APP	CAV1	APP_CAV1
103	ETS2	APP	FPR2	APP_FPR2
104	ETS2	APP	NCSTN	APP_NCSTN
105	ETS2	APP	TNFRSF21	APP_TNFRSF21
106	ETS2	APP	GPC1	APP_GPC1
107	ETS2	APP	CD74	APP_CD74
108	ETS2	APP	SLC45A3	APP_SLC45A3
109	TBP	APP	LRP1	APP_LRP1
110	TBP	APP	NGFR	APP_NGFR
111	TBP	APP	CAV1	APP_CAV1
112	TBP	APP	FPR2	APP_FPR2
113	TBP	APP	NCSTN	APP_NCSTN
114	TBP	APP	TNFRSF21	APP_TNFRSF21
115	TBP	APP	GPC1	APP_GPC1

116	TBP	APP	CD74	APP_CD74
117	TBP	APP	SLC45A3	APP_SLC45A3
118	TFAP2A	APP	LRP1	APP_LRP1
119	TFAP2A	APP	NGFR	APP_NGFR
120	TFAP2A	APP	CAV1	APP_CAV1
121	TFAP2A	APP	FPR2	APP_FPR2
122	TFAP2A	APP	NCSTN	APP_NCSTN
123	TFAP2A	APP	TNFRSF21	APP_TNFRSF21
124	TFAP2A	APP	GPC1	APP_GPC1
125	TFAP2A	APP	CD74	APP_CD74
126	TFAP2A	APP	SLC45A3	APP_SLC45A3
127	HDAC9	APP	LRP1	APP_LRP1
128	HDAC9	APP	NGFR	APP_NGFR
129	HDAC9	APP	CAV1	APP_CAV1
130	HDAC9	APP	FPR2	APP_FPR2
131	HDAC9	APP	NCSTN	APP_NCSTN
132	HDAC9	APP	TNFRSF21	APP_TNFRSF21
133	HDAC9	APP	GPC1	APP_GPC1
134	HDAC9	APP	CD74	APP_CD74
135	HDAC9	APP	SLC45A3	APP_SLC45A3
136	CTCF	APP	LRP1	APP_LRP1
137	CTCF	APP	NGFR	APP_NGFR
138	CTCF	APP	CAV1	APP_CAV1
139	CTCF	APP	FPR2	APP_FPR2
140	CTCF	APP	NCSTN	APP_NCSTN
141	CTCF	APP	TNFRSF21	APP_TNFRSF21
142	CTCF	APP	GPC1	APP_GPC1
143	CTCF	APP	CD74	APP_CD74
144	CTCF	APP	SLC45A3	APP_SLC45A3
145	NFIL3	APP	LRP1	APP_LRP1
146	NFIL3	APP	NGFR	APP_NGFR
147	NFIL3	APP	CAV1	APP_CAV1
148	NFIL3	APP	FPR2	APP_FPR2
149	NFIL3	APP	NCSTN	APP_NCSTN
150	NFIL3	APP	TNFRSF21	APP_TNFRSF21
151	NFIL3	APP	GPC1	APP_GPC1
152	NFIL3	APP	CD74	APP_CD74
153	NFIL3	APP	SLC45A3	APP_SLC45A3
154	SP1	APP	LRP1	APP_LRP1
155	SP1	APP	NGFR	APP_NGFR
156	SP1	APP	CAV1	APP_CAV1
157	SP1	APP	FPR2	APP_FPR2
158	SP1	APP	NCSTN	APP_NCSTN
159	SP1	APP	TNFRSF21	APP_TNFRSF21
160	SP1	APP	GPC1	APP_GPC1
161	SP1	APP	CD74	APP_CD74
162	SP1	APP	SLC45A3	APP_SLC45A3
163	JUN	APP	LRP1	APP_LRP1
164	JUN	APP	NGFR	APP_NGFR
165	JUN	APP	CAV1	APP_CAV1
166	JUN	APP	FPR2	APP_FPR2
167	JUN	APP	NCSTN	APP_NCSTN
168	JUN	APP	TNFRSF21	APP_TNFRSF21
169	JUN	APP	GPC1	APP_GPC1
170	JUN	APP	CD74	APP_CD74
171	JUN	APP	SLC45A3	APP_SLC45A3
172	STAT1	APP	LRP1	APP_LRP1
173	STAT1	APP	NGFR	APP_NGFR

174	STAT1	APP	CAV1	APP_CAV1
175	STAT1	APP	FPR2	APP_FPR2
176	STAT1	APP	NCSTN	APP_NCSTN
177	STAT1	APP	TNFRSF21	APP_TNFRSF21
178	STAT1	APP	GPC1	APP_GPC1
179	STAT1	APP	CD74	APP_CD74
180	STAT1	APP	SLC45A3	APP_SLC45A3
181	BRCA1	AREG	EGFR	AREG_EGFR
182	BRCA1	AREG	ERBB3	AREG_ERBB3
183	SP1	AREG	EGFR	AREG_EGFR
184	SP1	AREG	ERBB3	AREG_ERBB3
185	NFIL3	AREG	EGFR	AREG_EGFR
186	NFIL3	AREG	ERBB3	AREG_ERBB3
187	ZBTB2	ARF1	PLD2	ARF1_PLD2
188	ZBTB2	ARF1	INSR	ARF1_INSR
189	ZBTB2	ARF1	CHRM3	ARF1_CHRM3
190	USF2	B2M	CD3D	B2M_CD3D
191	USF2	B2M	HLA-F	B2M_HLA-F
192	USF2	B2M	KLRC1	B2M_KLRC1
193	USF2	B2M	KIR2DL3	B2M_KIR2DL3
194	USF2	B2M	TFRC	B2M_TFRC
195	USF2	B2M	KIR2DL1	B2M_KIR2DL1
196	USF2	B2M	LILRB2	B2M_LILRB2
197	USF2	B2M	CD3G	B2M_CD3G
198	USF2	B2M	KLRD1	B2M_KLRD1
199	USF2	B2M	KIR3DL1	B2M_KIR3DL1
200	USF2	B2M	HFE	B2M_HFE
201	USF2	B2M	CD1A	B2M_CD1A
202	USF2	B2M	CD247	B2M_CD247
203	USF2	B2M	CD1B	B2M_CD1B
204	USF2	B2M	LILRB1	B2M_LILRB1
205	NFKB1	B2M	CD3D	B2M_CD3D
206	NFKB1	B2M	HLA-F	B2M_HLA-F
207	NFKB1	B2M	KLRC1	B2M_KLRC1
208	NFKB1	B2M	KIR2DL3	B2M_KIR2DL3
209	NFKB1	B2M	TFRC	B2M_TFRC
210	NFKB1	B2M	KIR2DL1	B2M_KIR2DL1
211	NFKB1	B2M	LILRB2	B2M_LILRB2
212	NFKB1	B2M	CD3G	B2M_CD3G
213	NFKB1	B2M	KLRD1	B2M_KLRD1
214	NFKB1	B2M	KIR3DL1	B2M_KIR3DL1
215	NFKB1	B2M	HFE	B2M_HFE
216	NFKB1	B2M	CD1A	B2M_CD1A
217	NFKB1	B2M	CD247	B2M_CD247
218	NFKB1	B2M	CD1B	B2M_CD1B
219	NFKB1	B2M	LILRB1	B2M_LILRB1
220	USF1	B2M	CD3D	B2M_CD3D
221	USF1	B2M	HLA-F	B2M_HLA-F
222	USF1	B2M	KLRC1	B2M_KLRC1
223	USF1	B2M	KIR2DL3	B2M_KIR2DL3
224	USF1	B2M	TFRC	B2M_TFRC
225	USF1	B2M	KIR2DL1	B2M_KIR2DL1
226	USF1	B2M	LILRB2	B2M_LILRB2
227	USF1	B2M	CD3G	B2M_CD3G
228	USF1	B2M	KLRD1	B2M_KLRD1
229	USF1	B2M	KIR3DL1	B2M_KIR3DL1
230	USF1	B2M	HFE	B2M_HFE
231	USF1	B2M	CD1A	B2M_CD1A

232	USF1	B2M	CD247	B2M_CD247
233	USF1	B2M	CD1B	B2M_CD1B
234	USF1	B2M	LILRB1	B2M_LILRB1
235	CREB1	BDNF	NGFRAP1	BDNF_NGFRAP1
236	CREB1	BDNF	DDR1	BDNF_DDR1
237	REST	BDNF	NGFRAP1	BDNF_NGFRAP1
238	REST	BDNF	DDR1	BDNF_DDR1
239	NFKB1	BGN	TLR2	BGN_TLR2
240	NFKB1	BGN	LY96	BGN_LY96
241	NFKB1	BGN	TLR4	BGN_TLR4
242	RELA	BGN	TLR2	BGN_TLR2
243	RELA	BGN	LY96	BGN_LY96
244	RELA	BGN	TLR4	BGN_TLR4
245	NFKB1	BST1	CAV1	BST1_CAV1
246	CEBPD	C3	IFITM1	C3_IFITM1
247	CEBPD	C3	ITGAX	C3_ITGAX
248	CEBPD	C3	CD81	C3_CD81
249	CEBPD	C3	ITGB2	C3_ITGB2
250	CEBPD	C3	C5AR2	C3_C5AR2
251	CEBPD	C3	LRP1	C3_LRP1
252	CEBPD	C3	CR1	C3_CR1
253	CEBPD	C3	C3AR1	C3_C3AR1
254	CEBPD	C3	ITGAM	C3_ITGAM
255	CEBPD	C3	CD19	C3_CD19
256	CEBPD	C3	CD46	C3_CD46
257	SP1	C4A	C3AR1	C4A_C3AR1
258	WT1	CAMP	FPR2	CAMP_FPR2
259	WT1	CAMP	EGFR	CAMP_EGFR
260	VDR	CAMP	FPR2	CAMP_FPR2
261	VDR	CAMP	EGFR	CAMP_EGFR
262	NR0B1	CAMP	FPR2	CAMP_FPR2
263	NR0B1	CAMP	EGFR	CAMP_EGFR
264	STAT3	CCL11	CXCR3	CCL11_CXCR3
265	RELA	CCL11	CXCR3	CCL11_CXCR3
266	NFKB1	CCL11	CXCR3	CCL11_CXCR3
267	STAT6	CCL11	CXCR3	CCL11_CXCR3
268	YY1	CCL13	CXCR3	CCL13_CXCR3
269	NFKB1	CCL13	CXCR3	CCL13_CXCR3
270	RELA	CCL13	CXCR3	CCL13_CXCR3
271	RELB	CCL19	CCR7	CCL19_CCR7
272	RELB	CCL19	CXCR3	CCL19_CXCR3
273	RELA	CCL19	CCR7	CCL19_CCR7
274	RELA	CCL19	CXCR3	CCL19_CXCR3
275	NFKB1	CCL19	CCR7	CCL19_CCR7
276	NFKB1	CCL19	CXCR3	CCL19_CXCR3
277	RELA	CCL20	CCR6	CCL20_CCR6
278	RELA	CCL20	CXCR3	CCL20_CXCR3
279	CEBPD	CCL20	CCR6	CCL20_CCR6
280	CEBPD	CCL20	CXCR3	CCL20_CXCR3
281	NFKB1	CCL20	CCR6	CCL20_CCR6
282	NFKB1	CCL20	CXCR3	CCL20_CXCR3
283	NFKB2	CCL21	CXCR3	CCL21_CXCR3
284	NFKB2	CCL21	CCR7	CCL21_CCR7
285	RELB	CCL21	CXCR3	CCL21_CXCR3
286	RELB	CCL21	CCR7	CCL21_CCR7
287	RELA	CCL3	ACKR2	CCL3_ACKR2
288	RELA	CCL3	CCR4	CCL3_CCR4
289	RELA	CCL3	CCR1	CCL3_CCR1

290	RELA	CCL3	CCR3	CCL3_CCR3
291	RELA	CCL3	CCR5	CCL3_CCR5
292	E2F1	CCL3	ACKR2	CCL3_ACKR2
293	E2F1	CCL3	CCR4	CCL3_CCR4
294	E2F1	CCL3	CCR1	CCL3_CCR1
295	E2F1	CCL3	CCR3	CCL3_CCR3
296	E2F1	CCL3	CCR5	CCL3_CCR5
297	STAT1	CCL3	ACKR2	CCL3_ACKR2
298	STAT1	CCL3	CCR4	CCL3_CCR4
299	STAT1	CCL3	CCR1	CCL3_CCR1
300	STAT1	CCL3	CCR3	CCL3_CCR3
301	STAT1	CCL3	CCR5	CCL3_CCR5
302	NFKB1	CCL3	ACKR2	CCL3_ACKR2
303	NFKB1	CCL3	CCR4	CCL3_CCR4
304	NFKB1	CCL3	CCR1	CCL3_CCR1
305	NFKB1	CCL3	CCR3	CCL3_CCR3
306	NFKB1	CCL3	CCR5	CCL3_CCR5
307	RUNX1	CCL3	ACKR2	CCL3_ACKR2
308	RUNX1	CCL3	CCR4	CCL3_CCR4
309	RUNX1	CCL3	CCR1	CCL3_CCR1
310	RUNX1	CCL3	CCR3	CCL3_CCR3
311	RUNX1	CCL3	CCR5	CCL3_CCR5
312	CREM	CCL4	CCR1	CCL4_CCR1
313	CREM	CCL4	ACKR2	CCL4_ACKR2
314	CREM	CCL4	CCR8	CCL4_CCR8
315	CREM	CCL4	CCR5	CCL4_CCR5
316	CREM	CCL4	CCR3	CCL4_CCR3
317	RELA	CCL4	CCR1	CCL4_CCR1
318	RELA	CCL4	ACKR2	CCL4_ACKR2
319	RELA	CCL4	CCR8	CCL4_CCR8
320	RELA	CCL4	CCR5	CCL4_CCR5
321	RELA	CCL4	CCR3	CCL4_CCR3
322	NFKB1	CCL4	CCR1	CCL4_CCR1
323	NFKB1	CCL4	ACKR2	CCL4_ACKR2
324	NFKB1	CCL4	CCR8	CCL4_CCR8
325	NFKB1	CCL4	CCR5	CCL4_CCR5
326	NFKB1	CCL4	CCR3	CCL4_CCR3
327	JUND	CCL5	SDC1	CCL5_SDC1
328	JUND	CCL5	CCR5	CCL5_CCR5
329	JUND	CCL5	CCR4	CCL5_CCR4
330	JUND	CCL5	CCR1	CCL5_CCR1
331	JUND	CCL5	GPR75	CCL5_GPR75
332	JUND	CCL5	SDC4	CCL5_SDC4
333	JUND	CCL5	ACKR4	CCL5_ACKR4
334	JUND	CCL5	ACKR2	CCL5_ACKR2
335	JUND	CCL5	CXCR3	CCL5_CXCR3
336	JUND	CCL5	DARC	CCL5_DARC
337	JUND	CCL5	CCR3	CCL5_CCR3
338	SPI1	CCL5	SDC1	CCL5_SDC1
339	SPI1	CCL5	CCR5	CCL5_CCR5
340	SPI1	CCL5	CCR4	CCL5_CCR4
341	SPI1	CCL5	CCR1	CCL5_CCR1
342	SPI1	CCL5	GPR75	CCL5_GPR75
343	SPI1	CCL5	SDC4	CCL5_SDC4
344	SPI1	CCL5	ACKR4	CCL5_ACKR4
345	SPI1	CCL5	ACKR2	CCL5_ACKR2
346	SPI1	CCL5	CXCR3	CCL5_CXCR3
347	SPI1	CCL5	DARC	CCL5_DARC

348 SPI1	CCL5	CCR3	CCL5_CCR3
349 NFKBIA	CCL5	SDC1	CCL5_SDC1
350 NFKBIA	CCL5	CCR5	CCL5_CCR5
351 NFKBIA	CCL5	CCR4	CCL5_CCR4
352 NFKBIA	CCL5	CCR1	CCL5_CCR1
353 NFKBIA	CCL5	GPR75	CCL5_GPR75
354 NFKBIA	CCL5	SDC4	CCL5_SDC4
355 NFKBIA	CCL5	ACKR4	CCL5_ACKR4
356 NFKBIA	CCL5	ACKR2	CCL5_ACKR2
357 NFKBIA	CCL5	CXCR3	CCL5_CXCR3
358 NFKBIA	CCL5	DARC	CCL5_DARC
359 NFKBIA	CCL5	CCR3	CCL5_CCR3
360 KLF13	CCL5	SDC1	CCL5_SDC1
361 KLF13	CCL5	CCR5	CCL5_CCR5
362 KLF13	CCL5	CCR4	CCL5_CCR4
363 KLF13	CCL5	CCR1	CCL5_CCR1
364 KLF13	CCL5	GPR75	CCL5_GPR75
365 KLF13	CCL5	SDC4	CCL5_SDC4
366 KLF13	CCL5	ACKR4	CCL5_ACKR4
367 KLF13	CCL5	ACKR2	CCL5_ACKR2
368 KLF13	CCL5	CXCR3	CCL5_CXCR3
369 KLF13	CCL5	DARC	CCL5_DARC
370 KLF13	CCL5	CCR3	CCL5_CCR3
371 NFKB1	CCL5	SDC1	CCL5_SDC1
372 NFKB1	CCL5	CCR5	CCL5_CCR5
373 NFKB1	CCL5	CCR4	CCL5_CCR4
374 NFKB1	CCL5	CCR1	CCL5_CCR1
375 NFKB1	CCL5	GPR75	CCL5_GPR75
376 NFKB1	CCL5	SDC4	CCL5_SDC4
377 NFKB1	CCL5	ACKR4	CCL5_ACKR4
378 NFKB1	CCL5	ACKR2	CCL5_ACKR2
379 NFKB1	CCL5	CXCR3	CCL5_CXCR3
380 NFKB1	CCL5	DARC	CCL5_DARC
381 NFKB1	CCL5	CCR3	CCL5_CCR3
382 IRF7	CCL5	SDC1	CCL5_SDC1
383 IRF7	CCL5	CCR5	CCL5_CCR5
384 IRF7	CCL5	CCR4	CCL5_CCR4
385 IRF7	CCL5	CCR1	CCL5_CCR1
386 IRF7	CCL5	GPR75	CCL5_GPR75
387 IRF7	CCL5	SDC4	CCL5_SDC4
388 IRF7	CCL5	ACKR4	CCL5_ACKR4
389 IRF7	CCL5	ACKR2	CCL5_ACKR2
390 IRF7	CCL5	CXCR3	CCL5_CXCR3
391 IRF7	CCL5	DARC	CCL5_DARC
392 IRF7	CCL5	CCR3	CCL5_CCR3
393 ATF3	CCL5	SDC1	CCL5_SDC1
394 ATF3	CCL5	CCR5	CCL5_CCR5
395 ATF3	CCL5	CCR4	CCL5_CCR4
396 ATF3	CCL5	CCR1	CCL5_CCR1
397 ATF3	CCL5	GPR75	CCL5_GPR75
398 ATF3	CCL5	SDC4	CCL5_SDC4
399 ATF3	CCL5	ACKR4	CCL5_ACKR4
400 ATF3	CCL5	ACKR2	CCL5_ACKR2
401 ATF3	CCL5	CXCR3	CCL5_CXCR3
402 ATF3	CCL5	DARC	CCL5_DARC
403 ATF3	CCL5	CCR3	CCL5_CCR3
404 SP1	CCL5	SDC1	CCL5_SDC1
405 SP1	CCL5	CCR5	CCL5_CCR5

406	SP1	CCL5	CCR4	CCL5_CCR4
407	SP1	CCL5	CCR1	CCL5_CCR1
408	SP1	CCL5	GPR75	CCL5_GPR75
409	SP1	CCL5	SDC4	CCL5_SDC4
410	SP1	CCL5	ACKR4	CCL5_ACKR4
411	SP1	CCL5	ACKR2	CCL5_ACKR2
412	SP1	CCL5	CXCR3	CCL5_CXCR3
413	SP1	CCL5	DARC	CCL5_DARC
414	SP1	CCL5	CCR3	CCL5_CCR3
415	CREB1	CCL5	SDC1	CCL5_SDC1
416	CREB1	CCL5	CCR5	CCL5_CCR5
417	CREB1	CCL5	CCR4	CCL5_CCR4
418	CREB1	CCL5	CCR1	CCL5_CCR1
419	CREB1	CCL5	GPR75	CCL5_GPR75
420	CREB1	CCL5	SDC4	CCL5_SDC4
421	CREB1	CCL5	ACKR4	CCL5_ACKR4
422	CREB1	CCL5	ACKR2	CCL5_ACKR2
423	CREB1	CCL5	CXCR3	CCL5_CXCR3
424	CREB1	CCL5	DARC	CCL5_DARC
425	CREB1	CCL5	CCR3	CCL5_CCR3
426	IRF1	CCL5	SDC1	CCL5_SDC1
427	IRF1	CCL5	CCR5	CCL5_CCR5
428	IRF1	CCL5	CCR4	CCL5_CCR4
429	IRF1	CCL5	CCR1	CCL5_CCR1
430	IRF1	CCL5	GPR75	CCL5_GPR75
431	IRF1	CCL5	SDC4	CCL5_SDC4
432	IRF1	CCL5	ACKR4	CCL5_ACKR4
433	IRF1	CCL5	ACKR2	CCL5_ACKR2
434	IRF1	CCL5	CXCR3	CCL5_CXCR3
435	IRF1	CCL5	DARC	CCL5_DARC
436	IRF1	CCL5	CCR3	CCL5_CCR3
437	RELA	CCL5	SDC1	CCL5_SDC1
438	RELA	CCL5	CCR5	CCL5_CCR5
439	RELA	CCL5	CCR4	CCL5_CCR4
440	RELA	CCL5	CCR1	CCL5_CCR1
441	RELA	CCL5	GPR75	CCL5_GPR75
442	RELA	CCL5	SDC4	CCL5_SDC4
443	RELA	CCL5	ACKR4	CCL5_ACKR4
444	RELA	CCL5	ACKR2	CCL5_ACKR2
445	RELA	CCL5	CXCR3	CCL5_CXCR3
446	RELA	CCL5	DARC	CCL5_DARC
447	RELA	CCL5	CCR3	CCL5_CCR3
448	IRF3	CCL5	SDC1	CCL5_SDC1
449	IRF3	CCL5	CCR5	CCL5_CCR5
450	IRF3	CCL5	CCR4	CCL5_CCR4
451	IRF3	CCL5	CCR1	CCL5_CCR1
452	IRF3	CCL5	GPR75	CCL5_GPR75
453	IRF3	CCL5	SDC4	CCL5_SDC4
454	IRF3	CCL5	ACKR4	CCL5_ACKR4
455	IRF3	CCL5	ACKR2	CCL5_ACKR2
456	IRF3	CCL5	CXCR3	CCL5_CXCR3
457	IRF3	CCL5	DARC	CCL5_DARC
458	IRF3	CCL5	CCR3	CCL5_CCR3
459	REL	CCL5	SDC1	CCL5_SDC1
460	REL	CCL5	CCR5	CCL5_CCR5
461	REL	CCL5	CCR4	CCL5_CCR4
462	REL	CCL5	CCR1	CCL5_CCR1
463	REL	CCL5	GPR75	CCL5_GPR75

464	REL	CCL5	SDC4	CCL5_SDC4
465	REL	CCL5	ACKR4	CCL5_ACKR4
466	REL	CCL5	ACKR2	CCL5_ACKR2
467	REL	CCL5	CXCR3	CCL5_CXCR3
468	REL	CCL5	DARC	CCL5_DARC
469	REL	CCL5	CCR3	CCL5_CCR3
470	JUN	CCL5	SDC1	CCL5_SDC1
471	JUN	CCL5	CCR5	CCL5_CCR5
472	JUN	CCL5	CCR4	CCL5_CCR4
473	JUN	CCL5	CCR1	CCL5_CCR1
474	JUN	CCL5	GPR75	CCL5_GPR75
475	JUN	CCL5	SDC4	CCL5_SDC4
476	JUN	CCL5	ACKR4	CCL5_ACKR4
477	JUN	CCL5	ACKR2	CCL5_ACKR2
478	JUN	CCL5	CXCR3	CCL5_CXCR3
479	JUN	CCL5	DARC	CCL5_DARC
480	JUN	CCL5	CCR3	CCL5_CCR3
481	LEF1	CCL7	CXCR3	CCL7_CXCR3
482	SP3	CD14	ITGB1	CD14_ITGB1
483	SP3	CD14	ITGA4	CD14_ITGA4
484	SP1	CD14	ITGB1	CD14_ITGB1
485	SP1	CD14	ITGA4	CD14_ITGA4
486	WT1	CD14	ITGB1	CD14_ITGB1
487	WT1	CD14	ITGA4	CD14_ITGA4
488	MEF2D	CD14	ITGB1	CD14_ITGB1
489	MEF2D	CD14	ITGA4	CD14_ITGA4
490	SP2	CD14	ITGB1	CD14_ITGB1
491	SP2	CD14	ITGA4	CD14_ITGA4
492	KLF4	CD14	ITGB1	CD14_ITGB1
493	KLF4	CD14	ITGA4	CD14_ITGA4
494	VDR	CD14	ITGB1	CD14_ITGB1
495	VDR	CD14	ITGA4	CD14_ITGA4
496	ESR1	CD24	SELP	CD24_SELP
497	MYB	CD34	SELL	CD34_SELL
498	TAL1	CD34	SELL	CD34_SELL
499	RUNX1	CD34	SELL	CD34_SELL
500	MZF1	CD34	SELL	CD34_SELL
501	NFKB1	CD40LG	CD40	CD40LG_CD40
502	NFKB1	CD40LG	TRAF3	CD40LG_TRAF3
503	NFKB1	CD40LG	ITGB2	CD40LG_ITGB2
504	NFKB1	CD40LG	ITGAM	CD40LG_ITGAM
505	RELA	CD40LG	CD40	CD40LG_CD40
506	RELA	CD40LG	TRAF3	CD40LG_TRAF3
507	RELA	CD40LG	ITGB2	CD40LG_ITGB2
508	RELA	CD40LG	ITGAM	CD40LG_ITGAM
509	FOS	CD40LG	CD40	CD40LG_CD40
510	FOS	CD40LG	TRAF3	CD40LG_TRAF3
511	FOS	CD40LG	ITGB2	CD40LG_ITGB2
512	FOS	CD40LG	ITGAM	CD40LG_ITGAM
513	STAT1	CD40LG	CD40	CD40LG_CD40
514	STAT1	CD40LG	TRAF3	CD40LG_TRAF3
515	STAT1	CD40LG	ITGB2	CD40LG_ITGB2
516	STAT1	CD40LG	ITGAM	CD40LG_ITGAM
517	GATA3	CD40LG	CD40	CD40LG_CD40
518	GATA3	CD40LG	TRAF3	CD40LG_TRAF3
519	GATA3	CD40LG	ITGB2	CD40LG_ITGB2
520	GATA3	CD40LG	ITGAM	CD40LG_ITGAM
521	JUND	CD40LG	CD40	CD40LG_CD40

522	JUND	CD40LG	TRAF3	CD40LG_TRAF3
523	JUND	CD40LG	ITGB2	CD40LG_ITGB2
524	JUND	CD40LG	ITGAM	CD40LG_ITGAM
525	REL	CD40LG	CD40	CD40LG_CD40
526	REL	CD40LG	TRAF3	CD40LG_TRAF3
527	REL	CD40LG	ITGB2	CD40LG_ITGB2
528	REL	CD40LG	ITGAM	CD40LG_ITGAM
529	RFX1	CD70	CD27	CD70_CD27
530	HDAC1	CDH1	CDH2	CDH1_CDH2
531	HDAC1	CDH1	PTPRM	CDH1_PTPRM
532	HDAC1	CDH1	ERBB3	CDH1_ERBB3
533	HDAC1	CDH1	KLRG1	CDH1_KLRG1
534	HDAC1	CDH1	PTPRF	CDH1_PTPRF
535	HDAC1	CDH1	EGFR	CDH1_EGFR
536	PARP1	CDH1	CDH2	CDH1_CDH2
537	PARP1	CDH1	PTPRM	CDH1_PTPRM
538	PARP1	CDH1	ERBB3	CDH1_ERBB3
539	PARP1	CDH1	KLRG1	CDH1_KLRG1
540	PARP1	CDH1	PTPRF	CDH1_PTPRF
541	PARP1	CDH1	EGFR	CDH1_EGFR
542	DNMT1	CDH1	CDH2	CDH1_CDH2
543	DNMT1	CDH1	PTPRM	CDH1_PTPRM
544	DNMT1	CDH1	ERBB3	CDH1_ERBB3
545	DNMT1	CDH1	KLRG1	CDH1_KLRG1
546	DNMT1	CDH1	PTPRF	CDH1_PTPRF
547	DNMT1	CDH1	EGFR	CDH1_EGFR
548	YBX1	CDH1	CDH2	CDH1_CDH2
549	YBX1	CDH1	PTPRM	CDH1_PTPRM
550	YBX1	CDH1	ERBB3	CDH1_ERBB3
551	YBX1	CDH1	KLRG1	CDH1_KLRG1
552	YBX1	CDH1	PTPRF	CDH1_PTPRF
553	YBX1	CDH1	EGFR	CDH1_EGFR
554	RELA	CDH1	CDH2	CDH1_CDH2
555	RELA	CDH1	PTPRM	CDH1_PTPRM
556	RELA	CDH1	ERBB3	CDH1_ERBB3
557	RELA	CDH1	KLRG1	CDH1_KLRG1
558	RELA	CDH1	PTPRF	CDH1_PTPRF
559	RELA	CDH1	EGFR	CDH1_EGFR
560	TCF12	CDH1	CDH2	CDH1_CDH2
561	TCF12	CDH1	PTPRM	CDH1_PTPRM
562	TCF12	CDH1	ERBB3	CDH1_ERBB3
563	TCF12	CDH1	KLRG1	CDH1_KLRG1
564	TCF12	CDH1	PTPRF	CDH1_PTPRF
565	TCF12	CDH1	EGFR	CDH1_EGFR
566	TWIST1	CDH1	CDH2	CDH1_CDH2
567	TWIST1	CDH1	PTPRM	CDH1_PTPRM
568	TWIST1	CDH1	ERBB3	CDH1_ERBB3
569	TWIST1	CDH1	KLRG1	CDH1_KLRG1
570	TWIST1	CDH1	PTPRF	CDH1_PTPRF
571	TWIST1	CDH1	EGFR	CDH1_EGFR
572	SIX1	CDH1	CDH2	CDH1_CDH2
573	SIX1	CDH1	PTPRM	CDH1_PTPRM
574	SIX1	CDH1	ERBB3	CDH1_ERBB3
575	SIX1	CDH1	KLRG1	CDH1_KLRG1
576	SIX1	CDH1	PTPRF	CDH1_PTPRF
577	SIX1	CDH1	EGFR	CDH1_EGFR
578	FOXF1	CDH1	CDH2	CDH1_CDH2
579	FOXF1	CDH1	PTPRM	CDH1_PTPRM

580	FOXF1	CDH1	ERBB3	CDH1_ERBB3
581	FOXF1	CDH1	KLRG1	CDH1_KLRG1
582	FOXF1	CDH1	PTPRF	CDH1_PTPRF
583	FOXF1	CDH1	EGFR	CDH1_EGFR
584	GLI2	CDH1	CDH2	CDH1_CDH2
585	GLI2	CDH1	PTPRM	CDH1_PTPRM
586	GLI2	CDH1	ERBB3	CDH1_ERBB3
587	GLI2	CDH1	KLRG1	CDH1_KLRG1
588	GLI2	CDH1	PTPRF	CDH1_PTPRF
589	GLI2	CDH1	EGFR	CDH1_EGFR
590	MTA2	CDH1	CDH2	CDH1_CDH2
591	MTA2	CDH1	PTPRM	CDH1_PTPRM
592	MTA2	CDH1	ERBB3	CDH1_ERBB3
593	MTA2	CDH1	KLRG1	CDH1_KLRG1
594	MTA2	CDH1	PTPRF	CDH1_PTPRF
595	MTA2	CDH1	EGFR	CDH1_EGFR
596	HDAC3	CDH1	CDH2	CDH1_CDH2
597	HDAC3	CDH1	PTPRM	CDH1_PTPRM
598	HDAC3	CDH1	ERBB3	CDH1_ERBB3
599	HDAC3	CDH1	KLRG1	CDH1_KLRG1
600	HDAC3	CDH1	PTPRF	CDH1_PTPRF
601	HDAC3	CDH1	EGFR	CDH1_EGFR
602	FOXQ1	CDH1	CDH2	CDH1_CDH2
603	FOXQ1	CDH1	PTPRM	CDH1_PTPRM
604	FOXQ1	CDH1	ERBB3	CDH1_ERBB3
605	FOXQ1	CDH1	KLRG1	CDH1_KLRG1
606	FOXQ1	CDH1	PTPRF	CDH1_PTPRF
607	FOXQ1	CDH1	EGFR	CDH1_EGFR
608	SPDEF	CDH1	CDH2	CDH1_CDH2
609	SPDEF	CDH1	PTPRM	CDH1_PTPRM
610	SPDEF	CDH1	ERBB3	CDH1_ERBB3
611	SPDEF	CDH1	KLRG1	CDH1_KLRG1
612	SPDEF	CDH1	PTPRF	CDH1_PTPRF
613	SPDEF	CDH1	EGFR	CDH1_EGFR
614	TFAP2A	CDH1	CDH2	CDH1_CDH2
615	TFAP2A	CDH1	PTPRM	CDH1_PTPRM
616	TFAP2A	CDH1	ERBB3	CDH1_ERBB3
617	TFAP2A	CDH1	KLRG1	CDH1_KLRG1
618	TFAP2A	CDH1	PTPRF	CDH1_PTPRF
619	TFAP2A	CDH1	EGFR	CDH1_EGFR
620	KLF8	CDH1	CDH2	CDH1_CDH2
621	KLF8	CDH1	PTPRM	CDH1_PTPRM
622	KLF8	CDH1	ERBB3	CDH1_ERBB3
623	KLF8	CDH1	KLRG1	CDH1_KLRG1
624	KLF8	CDH1	PTPRF	CDH1_PTPRF
625	KLF8	CDH1	EGFR	CDH1_EGFR
626	SMARCA4	CDH1	CDH2	CDH1_CDH2
627	SMARCA4	CDH1	PTPRM	CDH1_PTPRM
628	SMARCA4	CDH1	ERBB3	CDH1_ERBB3
629	SMARCA4	CDH1	KLRG1	CDH1_KLRG1
630	SMARCA4	CDH1	PTPRF	CDH1_PTPRF
631	SMARCA4	CDH1	EGFR	CDH1_EGFR
632	FOXA2	CDH1	CDH2	CDH1_CDH2
633	FOXA2	CDH1	PTPRM	CDH1_PTPRM
634	FOXA2	CDH1	ERBB3	CDH1_ERBB3
635	FOXA2	CDH1	KLRG1	CDH1_KLRG1
636	FOXA2	CDH1	PTPRF	CDH1_PTPRF
637	FOXA2	CDH1	EGFR	CDH1_EGFR

638	TWIST2	CDH1	CDH2	CDH1_CDH2
639	TWIST2	CDH1	PTPRM	CDH1_PTPRM
640	TWIST2	CDH1	ERBB3	CDH1_ERBB3
641	TWIST2	CDH1	KLRG1	CDH1_KLRG1
642	TWIST2	CDH1	PTPRF	CDH1_PTPRF
643	TWIST2	CDH1	EGFR	CDH1_EGFR
644	WT1	CDH1	CDH2	CDH1_CDH2
645	WT1	CDH1	PTPRM	CDH1_PTPRM
646	WT1	CDH1	ERBB3	CDH1_ERBB3
647	WT1	CDH1	KLRG1	CDH1_KLRG1
648	WT1	CDH1	PTPRF	CDH1_PTPRF
649	WT1	CDH1	EGFR	CDH1_EGFR
650	HMGA1	CDH1	CDH2	CDH1_CDH2
651	HMGA1	CDH1	PTPRM	CDH1_PTPRM
652	HMGA1	CDH1	ERBB3	CDH1_ERBB3
653	HMGA1	CDH1	KLRG1	CDH1_KLRG1
654	HMGA1	CDH1	PTPRF	CDH1_PTPRF
655	HMGA1	CDH1	EGFR	CDH1_EGFR
656	ZEB1	CDH1	CDH2	CDH1_CDH2
657	ZEB1	CDH1	PTPRM	CDH1_PTPRM
658	ZEB1	CDH1	ERBB3	CDH1_ERBB3
659	ZEB1	CDH1	KLRG1	CDH1_KLRG1
660	ZEB1	CDH1	PTPRF	CDH1_PTPRF
661	ZEB1	CDH1	EGFR	CDH1_EGFR
662	EZH2	CDH1	CDH2	CDH1_CDH2
663	EZH2	CDH1	PTPRM	CDH1_PTPRM
664	EZH2	CDH1	ERBB3	CDH1_ERBB3
665	EZH2	CDH1	KLRG1	CDH1_KLRG1
666	EZH2	CDH1	PTPRF	CDH1_PTPRF
667	EZH2	CDH1	EGFR	CDH1_EGFR
668	HOXA7	CDH1	CDH2	CDH1_CDH2
669	HOXA7	CDH1	PTPRM	CDH1_PTPRM
670	HOXA7	CDH1	ERBB3	CDH1_ERBB3
671	HOXA7	CDH1	KLRG1	CDH1_KLRG1
672	HOXA7	CDH1	PTPRF	CDH1_PTPRF
673	HOXA7	CDH1	EGFR	CDH1_EGFR
674	ESR1	CDH1	CDH2	CDH1_CDH2
675	ESR1	CDH1	PTPRM	CDH1_PTPRM
676	ESR1	CDH1	ERBB3	CDH1_ERBB3
677	ESR1	CDH1	KLRG1	CDH1_KLRG1
678	ESR1	CDH1	PTPRF	CDH1_PTPRF
679	ESR1	CDH1	EGFR	CDH1_EGFR
680	SNAI2	CDH1	CDH2	CDH1_CDH2
681	SNAI2	CDH1	PTPRM	CDH1_PTPRM
682	SNAI2	CDH1	ERBB3	CDH1_ERBB3
683	SNAI2	CDH1	KLRG1	CDH1_KLRG1
684	SNAI2	CDH1	PTPRF	CDH1_PTPRF
685	SNAI2	CDH1	EGFR	CDH1_EGFR
686	HIF1A	CDH1	CDH2	CDH1_CDH2
687	HIF1A	CDH1	PTPRM	CDH1_PTPRM
688	HIF1A	CDH1	ERBB3	CDH1_ERBB3
689	HIF1A	CDH1	KLRG1	CDH1_KLRG1
690	HIF1A	CDH1	PTPRF	CDH1_PTPRF
691	HIF1A	CDH1	EGFR	CDH1_EGFR
692	SIRT1	CDH1	CDH2	CDH1_CDH2
693	SIRT1	CDH1	PTPRM	CDH1_PTPRM
694	SIRT1	CDH1	ERBB3	CDH1_ERBB3
695	SIRT1	CDH1	KLRG1	CDH1_KLRG1

696	SIRT1	CDH1	PTPRF	CDH1_PTPRF
697	SIRT1	CDH1	EGFR	CDH1_EGFR
698	SALL4	CDH1	CDH2	CDH1_CDH2
699	SALL4	CDH1	PTPRM	CDH1_PTPRM
700	SALL4	CDH1	ERBB3	CDH1_ERBB3
701	SALL4	CDH1	KLRG1	CDH1_KLRG1
702	SALL4	CDH1	PTPRF	CDH1_PTPRF
703	SALL4	CDH1	EGFR	CDH1_EGFR
704	ZEB2	CDH1	CDH2	CDH1_CDH2
705	ZEB2	CDH1	PTPRM	CDH1_PTPRM
706	ZEB2	CDH1	ERBB3	CDH1_ERBB3
707	ZEB2	CDH1	KLRG1	CDH1_KLRG1
708	ZEB2	CDH1	PTPRF	CDH1_PTPRF
709	ZEB2	CDH1	EGFR	CDH1_EGFR
710	VDR	CDH1	CDH2	CDH1_CDH2
711	VDR	CDH1	PTPRM	CDH1_PTPRM
712	VDR	CDH1	ERBB3	CDH1_ERBB3
713	VDR	CDH1	KLRG1	CDH1_KLRG1
714	VDR	CDH1	PTPRF	CDH1_PTPRF
715	VDR	CDH1	EGFR	CDH1_EGFR
716	KLF6	CDH1	CDH2	CDH1_CDH2
717	KLF6	CDH1	PTPRM	CDH1_PTPRM
718	KLF6	CDH1	ERBB3	CDH1_ERBB3
719	KLF6	CDH1	KLRG1	CDH1_KLRG1
720	KLF6	CDH1	PTPRF	CDH1_PTPRF
721	KLF6	CDH1	EGFR	CDH1_EGFR
722	RBBP7	CDH1	CDH2	CDH1_CDH2
723	RBBP7	CDH1	PTPRM	CDH1_PTPRM
724	RBBP7	CDH1	ERBB3	CDH1_ERBB3
725	RBBP7	CDH1	KLRG1	CDH1_KLRG1
726	RBBP7	CDH1	PTPRF	CDH1_PTPRF
727	RBBP7	CDH1	EGFR	CDH1_EGFR
728	GATA3	CDH1	CDH2	CDH1_CDH2
729	GATA3	CDH1	PTPRM	CDH1_PTPRM
730	GATA3	CDH1	ERBB3	CDH1_ERBB3
731	GATA3	CDH1	KLRG1	CDH1_KLRG1
732	GATA3	CDH1	PTPRF	CDH1_PTPRF
733	GATA3	CDH1	EGFR	CDH1_EGFR
734	NFKB1	CDH1	CDH2	CDH1_CDH2
735	NFKB1	CDH1	PTPRM	CDH1_PTPRM
736	NFKB1	CDH1	ERBB3	CDH1_ERBB3
737	NFKB1	CDH1	KLRG1	CDH1_KLRG1
738	NFKB1	CDH1	PTPRF	CDH1_PTPRF
739	NFKB1	CDH1	EGFR	CDH1_EGFR
740	LCOR	CDH1	CDH2	CDH1_CDH2
741	LCOR	CDH1	PTPRM	CDH1_PTPRM
742	LCOR	CDH1	ERBB3	CDH1_ERBB3
743	LCOR	CDH1	KLRG1	CDH1_KLRG1
744	LCOR	CDH1	PTPRF	CDH1_PTPRF
745	LCOR	CDH1	EGFR	CDH1_EGFR
746	TCF3	CDH1	CDH2	CDH1_CDH2
747	TCF3	CDH1	PTPRM	CDH1_PTPRM
748	TCF3	CDH1	ERBB3	CDH1_ERBB3
749	TCF3	CDH1	KLRG1	CDH1_KLRG1
750	TCF3	CDH1	PTPRF	CDH1_PTPRF
751	TCF3	CDH1	EGFR	CDH1_EGFR
752	MTA1	CDH1	CDH2	CDH1_CDH2
753	MTA1	CDH1	PTPRM	CDH1_PTPRM

754	MTA1	CDH1	ERBB3	CDH1_ERBB3
755	MTA1	CDH1	KLRG1	CDH1_KLRG1
756	MTA1	CDH1	PTPRF	CDH1_PTPRF
757	MTA1	CDH1	EGFR	CDH1_EGFR
758	HDGF	CDH1	CDH2	CDH1_CDH2
759	HDGF	CDH1	PTPRM	CDH1_PTPRM
760	HDGF	CDH1	ERBB3	CDH1_ERBB3
761	HDGF	CDH1	KLRG1	CDH1_KLRG1
762	HDGF	CDH1	PTPRF	CDH1_PTPRF
763	HDGF	CDH1	EGFR	CDH1_EGFR
764	AR	CDH1	CDH2	CDH1_CDH2
765	AR	CDH1	PTPRM	CDH1_PTPRM
766	AR	CDH1	ERBB3	CDH1_ERBB3
767	AR	CDH1	KLRG1	CDH1_KLRG1
768	AR	CDH1	PTPRF	CDH1_PTPRF
769	AR	CDH1	EGFR	CDH1_EGFR
770	ZNF217	CDH1	CDH2	CDH1_CDH2
771	ZNF217	CDH1	PTPRM	CDH1_PTPRM
772	ZNF217	CDH1	ERBB3	CDH1_ERBB3
773	ZNF217	CDH1	KLRG1	CDH1_KLRG1
774	ZNF217	CDH1	PTPRF	CDH1_PTPRF
775	ZNF217	CDH1	EGFR	CDH1_EGFR
776	STAT3	CDH1	CDH2	CDH1_CDH2
777	STAT3	CDH1	PTPRM	CDH1_PTPRM
778	STAT3	CDH1	ERBB3	CDH1_ERBB3
779	STAT3	CDH1	KLRG1	CDH1_KLRG1
780	STAT3	CDH1	PTPRF	CDH1_PTPRF
781	STAT3	CDH1	EGFR	CDH1_EGFR
782	SNAI1	CDH1	CDH2	CDH1_CDH2
783	SNAI1	CDH1	PTPRM	CDH1_PTPRM
784	SNAI1	CDH1	ERBB3	CDH1_ERBB3
785	SNAI1	CDH1	KLRG1	CDH1_KLRG1
786	SNAI1	CDH1	PTPRF	CDH1_PTPRF
787	SNAI1	CDH1	EGFR	CDH1_EGFR
788	RB1	CDH1	CDH2	CDH1_CDH2
789	RB1	CDH1	PTPRM	CDH1_PTPRM
790	RB1	CDH1	ERBB3	CDH1_ERBB3
791	RB1	CDH1	KLRG1	CDH1_KLRG1
792	RB1	CDH1	PTPRF	CDH1_PTPRF
793	RB1	CDH1	EGFR	CDH1_EGFR
794	LMO2	CDH1	CDH2	CDH1_CDH2
795	LMO2	CDH1	PTPRM	CDH1_PTPRM
796	LMO2	CDH1	ERBB3	CDH1_ERBB3
797	LMO2	CDH1	KLRG1	CDH1_KLRG1
798	LMO2	CDH1	PTPRF	CDH1_PTPRF
799	LMO2	CDH1	EGFR	CDH1_EGFR
800	HMGA2	CDH1	CDH2	CDH1_CDH2
801	HMGA2	CDH1	PTPRM	CDH1_PTPRM
802	HMGA2	CDH1	ERBB3	CDH1_ERBB3
803	HMGA2	CDH1	KLRG1	CDH1_KLRG1
804	HMGA2	CDH1	PTPRF	CDH1_PTPRF
805	HMGA2	CDH1	EGFR	CDH1_EGFR
806	FOXM1	CDH1	CDH2	CDH1_CDH2
807	FOXM1	CDH1	PTPRM	CDH1_PTPRM
808	FOXM1	CDH1	ERBB3	CDH1_ERBB3
809	FOXM1	CDH1	KLRG1	CDH1_KLRG1
810	FOXM1	CDH1	PTPRF	CDH1_PTPRF
811	FOXM1	CDH1	EGFR	CDH1_EGFR

812 KLF4	CDH1	CDH2	CDH1_CDH2
813 KLF4	CDH1	PTPRM	CDH1_PTPRM
814 KLF4	CDH1	ERBB3	CDH1_ERBB3
815 KLF4	CDH1	KLRG1	CDH1_KLRG1
816 KLF4	CDH1	PTPRF	CDH1_PTPRF
817 KLF4	CDH1	EGFR	CDH1_EGFR
818 HDAC2	CDH1	CDH2	CDH1_CDH2
819 HDAC2	CDH1	PTPRM	CDH1_PTPRM
820 HDAC2	CDH1	ERBB3	CDH1_ERBB3
821 HDAC2	CDH1	KLRG1	CDH1_KLRG1
822 HDAC2	CDH1	PTPRF	CDH1_PTPRF
823 HDAC2	CDH1	EGFR	CDH1_EGFR
824 CBX7	CDH1	CDH2	CDH1_CDH2
825 CBX7	CDH1	PTPRM	CDH1_PTPRM
826 CBX7	CDH1	ERBB3	CDH1_ERBB3
827 CBX7	CDH1	KLRG1	CDH1_KLRG1
828 CBX7	CDH1	PTPRF	CDH1_PTPRF
829 CBX7	CDH1	EGFR	CDH1_EGFR
830 STAT5A	CEL	CXCR4	CEL_CXCR4
831 STAT5B	CEL	CXCR4	CEL_CXCR4
832 PTF1A	CEL	CXCR4	CEL_CXCR4
833 SIRT1	CFH	ITGAM	CFH_ITGAM
834 SIRT1	CFH	SELL	CFH_SELL
835 CEBPZ	COL11A1	ITGB1	COL11A1_ITGB1
836 CEBPZ	COL11A1	DDR1	COL11A1_DDR1
837 SP1	COL18A1	ITGB1	COL18A1_ITGB1
838 FOXA2	COL18A1	ITGB1	COL18A1_ITGB1
839 NFIC	COL18A1	ITGB1	COL18A1_ITGB1
840 CIITA	COL1A1	CD36	COL1A1_CD36
841 CIITA	COL1A1	ITGB1	COL1A1_ITGB1
842 CIITA	COL1A1	CD44	COL1A1_CD44
843 CIITA	COL1A1	DDR1	COL1A1_DDR1
844 MYBL2	COL1A1	CD36	COL1A1_CD36
845 MYBL2	COL1A1	ITGB1	COL1A1_ITGB1
846 MYBL2	COL1A1	CD44	COL1A1_CD44
847 MYBL2	COL1A1	DDR1	COL1A1_DDR1
848 NFIC	COL1A1	CD36	COL1A1_CD36
849 NFIC	COL1A1	ITGB1	COL1A1_ITGB1
850 NFIC	COL1A1	CD44	COL1A1_CD44
851 NFIC	COL1A1	DDR1	COL1A1_DDR1
852 SP1	COL1A1	CD36	COL1A1_CD36
853 SP1	COL1A1	ITGB1	COL1A1_ITGB1
854 SP1	COL1A1	CD44	COL1A1_CD44
855 SP1	COL1A1	DDR1	COL1A1_DDR1
856 ETS1	COL1A1	CD36	COL1A1_CD36
857 ETS1	COL1A1	ITGB1	COL1A1_ITGB1
858 ETS1	COL1A1	CD44	COL1A1_CD44
859 ETS1	COL1A1	DDR1	COL1A1_DDR1
860 SP3	COL1A1	CD36	COL1A1_CD36
861 SP3	COL1A1	ITGB1	COL1A1_ITGB1
862 SP3	COL1A1	CD44	COL1A1_CD44
863 SP3	COL1A1	DDR1	COL1A1_DDR1
864 MYB	COL1A1	CD36	COL1A1_CD36
865 MYB	COL1A1	ITGB1	COL1A1_ITGB1
866 MYB	COL1A1	CD44	COL1A1_CD44
867 MYB	COL1A1	DDR1	COL1A1_DDR1
868 STAT6	COL1A1	CD36	COL1A1_CD36
869 STAT6	COL1A1	ITGB1	COL1A1_ITGB1

870	STAT6	COL1A1	CD44	COL1A1_CD44
871	STAT6	COL1A1	DDR1	COL1A1_DDR1
872	TFAP2A	COL1A1	CD36	COL1A1_CD36
873	TFAP2A	COL1A1	ITGB1	COL1A1_ITGB1
874	TFAP2A	COL1A1	CD44	COL1A1_CD44
875	TFAP2A	COL1A1	DDR1	COL1A1_DDR1
876	MKL1	COL1A1	CD36	COL1A1_CD36
877	MKL1	COL1A1	ITGB1	COL1A1_ITGB1
878	MKL1	COL1A1	CD44	COL1A1_CD44
879	MKL1	COL1A1	DDR1	COL1A1_DDR1
880	NFKB1	COL1A1	CD36	COL1A1_CD36
881	NFKB1	COL1A1	ITGB1	COL1A1_ITGB1
882	NFKB1	COL1A1	CD44	COL1A1_CD44
883	NFKB1	COL1A1	DDR1	COL1A1_DDR1
884	RELA	COL1A1	CD36	COL1A1_CD36
885	RELA	COL1A1	ITGB1	COL1A1_ITGB1
886	RELA	COL1A1	CD44	COL1A1_CD44
887	RELA	COL1A1	DDR1	COL1A1_DDR1
888	NFKB1	COL1A2	CD36	COL1A2_CD36
889	NFKB1	COL1A2	CD44	COL1A2_CD44
890	NFKB1	COL1A2	ITGB1	COL1A2_ITGB1
891	CEBPZ	COL1A2	CD36	COL1A2_CD36
892	CEBPZ	COL1A2	CD44	COL1A2_CD44
893	CEBPZ	COL1A2	ITGB1	COL1A2_ITGB1
894	RFX5	COL1A2	CD36	COL1A2_CD36
895	RFX5	COL1A2	CD44	COL1A2_CD44
896	RFX5	COL1A2	ITGB1	COL1A2_ITGB1
897	HDAC2	COL1A2	CD36	COL1A2_CD36
898	HDAC2	COL1A2	CD44	COL1A2_CD44
899	HDAC2	COL1A2	ITGB1	COL1A2_ITGB1
900	HDAC1	COL1A2	CD36	COL1A2_CD36
901	HDAC1	COL1A2	CD44	COL1A2_CD44
902	HDAC1	COL1A2	ITGB1	COL1A2_ITGB1
903	CIITA	COL1A2	CD36	COL1A2_CD36
904	CIITA	COL1A2	CD44	COL1A2_CD44
905	CIITA	COL1A2	ITGB1	COL1A2_ITGB1
906	FLI1	COL1A2	CD36	COL1A2_CD36
907	FLI1	COL1A2	CD44	COL1A2_CD44
908	FLI1	COL1A2	ITGB1	COL1A2_ITGB1
909	RELA	COL1A2	CD36	COL1A2_CD36
910	RELA	COL1A2	CD44	COL1A2_CD44
911	RELA	COL1A2	ITGB1	COL1A2_ITGB1
912	RFX1	COL1A2	CD36	COL1A2_CD36
913	RFX1	COL1A2	CD44	COL1A2_CD44
914	RFX1	COL1A2	ITGB1	COL1A2_ITGB1
915	YY1	COL1A2	CD36	COL1A2_CD36
916	YY1	COL1A2	CD44	COL1A2_CD44
917	YY1	COL1A2	ITGB1	COL1A2_ITGB1
918	SIRT1	COL1A2	CD36	COL1A2_CD36
919	SIRT1	COL1A2	CD44	COL1A2_CD44
920	SIRT1	COL1A2	ITGB1	COL1A2_ITGB1
921	EP300	COL1A2	CD36	COL1A2_CD36
922	EP300	COL1A2	CD44	COL1A2_CD44
923	EP300	COL1A2	ITGB1	COL1A2_ITGB1
924	KLF11	COL1A2	CD36	COL1A2_CD36
925	KLF11	COL1A2	CD44	COL1A2_CD44
926	KLF11	COL1A2	ITGB1	COL1A2_ITGB1
927	STAT6	COL1A2	CD36	COL1A2_CD36

928	STAT6	COL1A2	CD44	COL1A2_CD44
929	STAT6	COL1A2	ITGB1	COL1A2_ITGB1
930	MYB	COL1A2	CD36	COL1A2_CD36
931	MYB	COL1A2	CD44	COL1A2_CD44
932	MYB	COL1A2	ITGB1	COL1A2_ITGB1
933	SOX9	COL2A1	ITGB1	COL2A1_ITGB1
934	SOX9	COL2A1	DDR1	COL2A1_DDR1
935	SP1	COL2A1	ITGB1	COL2A1_ITGB1
936	SP1	COL2A1	DDR1	COL2A1_DDR1
937	NFKB1	COL2A1	ITGB1	COL2A1_ITGB1
938	NFKB1	COL2A1	DDR1	COL2A1_DDR1
939	SP3	COL2A1	ITGB1	COL2A1_ITGB1
940	SP3	COL2A1	DDR1	COL2A1_DDR1
941	RELA	COL2A1	ITGB1	COL2A1_ITGB1
942	RELA	COL2A1	DDR1	COL2A1_DDR1
943	LMX1B	COL4A1	ITGB1	COL4A1_ITGB1
944	LMX1B	COL4A1	ITGB8	COL4A1_ITGB8
945	LMX1B	COL4A1	CD47	COL4A1_CD47
946	ZEB1	COL4A3	ITGB1	COL4A3_ITGB1
947	ZEB1	COL4A3	CD47	COL4A3_CD47
948	LMX1B	COL4A4	CD93	COL4A4_CD93
949	LMX1B	COL4A4	CD47	COL4A4_CD47
950	LMX1B	COL4A4	ITGAV	COL4A4_ITGAV
951	LMX1B	COL4A4	ITGA2	COL4A4_ITGA2
952	LMX1B	COL4A4	ITGA1	COL4A4_ITGA1
953	LMX1B	COL4A4	ITGB1	COL4A4_ITGB1
954	SMAD3	COL7A1	ITGB1	COL7A1_ITGB1
955	NFKB1	COL7A1	ITGB1	COL7A1_ITGB1
956	SP1	COL7A1	ITGB1	COL7A1_ITGB1
957	SMAD4	COL7A1	ITGB1	COL7A1_ITGB1
958	RELA	COL7A1	ITGB1	COL7A1_ITGB1
959	STAT3	CRP	OLR1	CRP_OLR1
960	NFKB1	CRP	OLR1	CRP_OLR1
961	TBP	CRP	OLR1	CRP_OLR1
962	FOS	CRP	OLR1	CRP_OLR1
963	RELA	CRP	OLR1	CRP_OLR1
964	HNF1A	CRP	OLR1	CRP_OLR1
965	REL	CRP	OLR1	CRP_OLR1
966	CEBPB	CRP	OLR1	CRP_OLR1
967	CEBPA	CSF1	CSF1R	CSF1_CSF1R
968	ABL1	CSF1	CSF1R	CSF1_CSF1R
969	NFKB1	CSF1	CSF1R	CSF1_CSF1R
970	RUNX1	CSF1	CSF1R	CSF1_CSF1R
971	RELA	CSF1	CSF1R	CSF1_CSF1R
972	PPARG	CSF1	CSF1R	CSF1_CSF1R
973	SPI1	CSF1	CSF1R	CSF1_CSF1R
974	JUN	CSF1	CSF1R	CSF1_CSF1R
975	JUN	CSF2	CSF1R	CSF2_CSF1R
976	JUN	CSF2	CSF2RA	CSF2_CSF2RA
977	JUN	CSF2	ITGB1	CSF2_ITGB1
978	JUN	CSF2	CSF3R	CSF2_CSF3R
979	CREBBP	CSF2	CSF1R	CSF2_CSF1R
980	CREBBP	CSF2	CSF2RA	CSF2_CSF2RA
981	CREBBP	CSF2	ITGB1	CSF2_ITGB1
982	CREBBP	CSF2	CSF3R	CSF2_CSF3R
983	CTCF	CSF2	CSF1R	CSF2_CSF1R
984	CTCF	CSF2	CSF2RA	CSF2_CSF2RA
985	CTCF	CSF2	ITGB1	CSF2_ITGB1

986	CTCF	CSF2	CSF3R	CSF2_CSF3R
987	NFKB1	CSF2	CSF1R	CSF2_CSF1R
988	NFKB1	CSF2	CSF2RA	CSF2_CSF2RA
989	NFKB1	CSF2	ITGB1	CSF2_ITGB1
990	NFKB1	CSF2	CSF3R	CSF2_CSF3R
991	ETS2	CSF2	CSF1R	CSF2_CSF1R
992	ETS2	CSF2	CSF2RA	CSF2_CSF2RA
993	ETS2	CSF2	ITGB1	CSF2_ITGB1
994	ETS2	CSF2	CSF3R	CSF2_CSF3R
995	ETS1	CSF2	CSF1R	CSF2_CSF1R
996	ETS1	CSF2	CSF2RA	CSF2_CSF2RA
997	ETS1	CSF2	ITGB1	CSF2_ITGB1
998	ETS1	CSF2	CSF3R	CSF2_CSF3R
999	RUNX1	CSF2	CSF1R	CSF2_CSF1R
1000	RUNX1	CSF2	CSF2RA	CSF2_CSF2RA
1001	RUNX1	CSF2	ITGB1	CSF2_ITGB1
1002	RUNX1	CSF2	CSF3R	CSF2_CSF3R
1003	RELA	CSF2	CSF1R	CSF2_CSF1R
1004	RELA	CSF2	CSF2RA	CSF2_CSF2RA
1005	RELA	CSF2	ITGB1	CSF2_ITGB1
1006	RELA	CSF2	CSF3R	CSF2_CSF3R
1007	HIF1A	CTGF	ITGB2	CTGF_ITGB2
1008	HIF1A	CTGF	ITGAM	CTGF_ITGAM
1009	HIF1A	CTGF	LRP1	CTGF_LRP1
1010	ETS1	CTGF	ITGB2	CTGF_ITGB2
1011	ETS1	CTGF	ITGAM	CTGF_ITGAM
1012	ETS1	CTGF	LRP1	CTGF_LRP1
1013	FLI1	CTGF	ITGB2	CTGF_ITGB2
1014	FLI1	CTGF	ITGAM	CTGF_ITGAM
1015	FLI1	CTGF	LRP1	CTGF_LRP1
1016	WT1	CTGF	ITGB2	CTGF_ITGB2
1017	WT1	CTGF	ITGAM	CTGF_ITGAM
1018	WT1	CTGF	LRP1	CTGF_LRP1
1019	SP1	CTGF	ITGB2	CTGF_ITGB2
1020	SP1	CTGF	ITGAM	CTGF_ITGAM
1021	SP1	CTGF	LRP1	CTGF_LRP1
1022	STAT3	CTGF	ITGB2	CTGF_ITGB2
1023	STAT3	CTGF	ITGAM	CTGF_ITGAM
1024	STAT3	CTGF	LRP1	CTGF_LRP1
1025	JUN	CTGF	ITGB2	CTGF_ITGB2
1026	JUN	CTGF	ITGAM	CTGF_ITGAM
1027	JUN	CTGF	LRP1	CTGF_LRP1
1028	STAT1	CXCL10	CXCR3	CXCL10_CXCR3
1029	STAT1	CXCL10	SDC4	CXCL10_SDC4
1030	RELA	CXCL10	CXCR3	CXCL10_CXCR3
1031	RELA	CXCL10	SDC4	CXCL10_SDC4
1032	NFKB1	CXCL10	CXCR3	CXCL10_CXCR3
1033	NFKB1	CXCL10	SDC4	CXCL10_SDC4
1034	IRF3	CXCL10	CXCR3	CXCL10_CXCR3
1035	IRF3	CXCL10	SDC4	CXCL10_SDC4
1036	IRF1	CXCL10	CXCR3	CXCL10_CXCR3
1037	IRF1	CXCL10	SDC4	CXCL10_SDC4
1038	IRF7	CXCL10	CXCR3	CXCL10_CXCR3
1039	IRF7	CXCL10	SDC4	CXCL10_SDC4
1040	RELA	CXCL12	CD4	CXCL12_CD4
1041	RELA	CXCL12	ITGB1	CXCL12_ITGB1
1042	RELA	CXCL12	CXCR4	CXCL12_CXCR4
1043	RELA	CXCL12	SDC4	CXCL12_SDC4

1044	RELA	CXCL12	CXCR3	CXCL12_CXCR3
1045	NFKB1	CXCL12	CD4	CXCL12_CD4
1046	NFKB1	CXCL12	ITGB1	CXCL12_ITGB1
1047	NFKB1	CXCL12	CXCR4	CXCL12_CXCR4
1048	NFKB1	CXCL12	SDC4	CXCL12_SDC4
1049	NFKB1	CXCL12	CXCR3	CXCL12_CXCR3
1050	HIF1A	CXCL12	CD4	CXCL12_CD4
1051	HIF1A	CXCL12	ITGB1	CXCL12_ITGB1
1052	HIF1A	CXCL12	CXCR4	CXCL12_CXCR4
1053	HIF1A	CXCL12	SDC4	CXCL12_SDC4
1054	HIF1A	CXCL12	CXCR3	CXCL12_CXCR3
1055	SNAI2	CXCL12	CD4	CXCL12_CD4
1056	SNAI2	CXCL12	ITGB1	CXCL12_ITGB1
1057	SNAI2	CXCL12	CXCR4	CXCL12_CXCR4
1058	SNAI2	CXCL12	SDC4	CXCL12_SDC4
1059	SNAI2	CXCL12	CXCR3	CXCL12_CXCR3
1060	ESR1	CXCL12	CD4	CXCL12_CD4
1061	ESR1	CXCL12	ITGB1	CXCL12_ITGB1
1062	ESR1	CXCL12	CXCR4	CXCL12_CXCR4
1063	ESR1	CXCL12	SDC4	CXCL12_SDC4
1064	ESR1	CXCL12	CXCR3	CXCL12_CXCR3
1065	FOXF2	CXCL12	CD4	CXCL12_CD4
1066	FOXF2	CXCL12	ITGB1	CXCL12_ITGB1
1067	FOXF2	CXCL12	CXCR4	CXCL12_CXCR4
1068	FOXF2	CXCL12	SDC4	CXCL12_SDC4
1069	FOXF2	CXCL12	CXCR3	CXCL12_CXCR3
1070	GLI1	CYR61	ITGB2	CYR61_ITGB2
1071	GLI1	CYR61	CAV1	CYR61_CAV1
1072	GLI1	CYR61	ITGAM	CYR61_ITGAM
1073	STAT3	CYR61	ITGB2	CYR61_ITGB2
1074	STAT3	CYR61	CAV1	CYR61_CAV1
1075	STAT3	CYR61	ITGAM	CYR61_ITGAM
1076	NR1H4	DCN	MET	DCN_MET
1077	NR1H4	DCN	EGFR	DCN_EGFR
1078	JUN	DCN	MET	DCN_MET
1079	JUN	DCN	EGFR	DCN_EGFR
1080	HDAC1	DEFB1	CCR6	DEFB1_CCR6
1081	PPARG	DEFB103A	CCR6	DEFB103A_CCR6
1082	NFKB1	DEFB4A	CCR6	DEFB4A_CCR6
1083	NFKB1	DEFB4A	TLR4	DEFB4A_TLR4
1084	NFKBIZ	DEFB4A	CCR6	DEFB4A_CCR6
1085	NFKBIZ	DEFB4A	TLR4	DEFB4A_TLR4
1086	RELA	DEFB4A	CCR6	DEFB4A_CCR6
1087	RELA	DEFB4A	TLR4	DEFB4A_TLR4
1088	SP1	EGF	CAV1	EGF_CAV1
1089	SP1	EGF	ERBB3	EGF_ERBB3
1090	SP1	EGF	EGFR	EGF_EGFR
1091	KDM2A	EREG	EGFR	EREG_EGFR
1092	KDM2A	EREG	ERBB2	EREG_ERBB2
1093	KDM2A	EREG	ERBB3	EREG_ERBB3
1094	KDM2A	EREG	ERBB4	EREG_ERBB4
1095	WT1	EREG	EGFR	EREG_EGFR
1096	WT1	EREG	ERBB2	EREG_ERBB2
1097	WT1	EREG	ERBB3	EREG_ERBB3
1098	WT1	EREG	ERBB4	EREG_ERBB4
1099	NFIL3	EREG	EGFR	EREG_EGFR
1100	NFIL3	EREG	ERBB2	EREG_ERBB2
1101	NFIL3	EREG	ERBB3	EREG_ERBB3

1102	NFIL3	EREG	ERBB4	EREG_ERBB4
1103	SP1	F10	ITGB2	F10_ITGB2
1104	SP1	F10	ITGAM	F10_ITGAM
1105	GATA1	F13A1	ITGB1	F13A1_ITGB1
1106	GATA1	F13A1	ITGA4	F13A1_ITGA4
1107	ETS1	F13A1	ITGB1	F13A1_ITGB1
1108	ETS1	F13A1	ITGA4	F13A1_ITGA4
1109	RELA	F8	LRP1	F8_LRP1
1110	CEBPB	F8	LRP1	F8_LRP1
1111	APC	F8	LRP1	F8_LRP1
1112	NFKB1	F8	LRP1	F8_LRP1
1113	CEBPA	F9	LRP1	F9_LRP1
1114	NR4A1	FASLG	TNFRSF1A	FASLG_TNFRSF1A
1115	NR4A1	FASLG	FAS	FASLG_FAS
1116	NR4A1	FASLG	TNFRSF6B	FASLG_TNFRSF6B
1117	EGR2	FASLG	TNFRSF1A	FASLG_TNFRSF1A
1118	EGR2	FASLG	FAS	FASLG_FAS
1119	EGR2	FASLG	TNFRSF6B	FASLG_TNFRSF6B
1120	CEBPB	FASLG	TNFRSF1A	FASLG_TNFRSF1A
1121	CEBPB	FASLG	FAS	FASLG_FAS
1122	CEBPB	FASLG	TNFRSF6B	FASLG_TNFRSF6B
1123	JUN	FASLG	TNFRSF1A	FASLG_TNFRSF1A
1124	JUN	FASLG	FAS	FASLG_FAS
1125	JUN	FASLG	TNFRSF6B	FASLG_TNFRSF6B
1126	PDCD11	FASLG	TNFRSF1A	FASLG_TNFRSF1A
1127	PDCD11	FASLG	FAS	FASLG_FAS
1128	PDCD11	FASLG	TNFRSF6B	FASLG_TNFRSF6B
1129	CREM	FASLG	TNFRSF1A	FASLG_TNFRSF1A
1130	CREM	FASLG	FAS	FASLG_FAS
1131	CREM	FASLG	TNFRSF6B	FASLG_TNFRSF6B
1132	SP1	FASLG	TNFRSF1A	FASLG_TNFRSF1A
1133	SP1	FASLG	FAS	FASLG_FAS
1134	SP1	FASLG	TNFRSF6B	FASLG_TNFRSF6B
1135	CIITA	FASLG	TNFRSF1A	FASLG_TNFRSF1A
1136	CIITA	FASLG	FAS	FASLG_FAS
1137	CIITA	FASLG	TNFRSF6B	FASLG_TNFRSF6B
1138	EGR1	FASLG	TNFRSF1A	FASLG_TNFRSF1A
1139	EGR1	FASLG	FAS	FASLG_FAS
1140	EGR1	FASLG	TNFRSF6B	FASLG_TNFRSF6B
1141	RELA	FASLG	TNFRSF1A	FASLG_TNFRSF1A
1142	RELA	FASLG	FAS	FASLG_FAS
1143	RELA	FASLG	TNFRSF6B	FASLG_TNFRSF6B
1144	NFKB1	FASLG	TNFRSF1A	FASLG_TNFRSF1A
1145	NFKB1	FASLG	FAS	FASLG_FAS
1146	NFKB1	FASLG	TNFRSF6B	FASLG_TNFRSF6B
1147	IRF1	FASLG	TNFRSF1A	FASLG_TNFRSF1A
1148	IRF1	FASLG	FAS	FASLG_FAS
1149	IRF1	FASLG	TNFRSF6B	FASLG_TNFRSF6B
1150	PPARG	FASLG	TNFRSF1A	FASLG_TNFRSF1A
1151	PPARG	FASLG	FAS	FASLG_FAS
1152	PPARG	FASLG	TNFRSF6B	FASLG_TNFRSF6B
1153	MAX	FASLG	TNFRSF1A	FASLG_TNFRSF1A
1154	MAX	FASLG	FAS	FASLG_FAS
1155	MAX	FASLG	TNFRSF6B	FASLG_TNFRSF6B
1156	ATF2	FASLG	TNFRSF1A	FASLG_TNFRSF1A
1157	ATF2	FASLG	FAS	FASLG_FAS
1158	ATF2	FASLG	TNFRSF6B	FASLG_TNFRSF6B
1159	MYC	FASLG	TNFRSF1A	FASLG_TNFRSF1A

1160	MYC	FASLG	FAS	FASLG_FAS
1161	MYC	FASLG	TNFRSF6B	FASLG_TNFRSF6B
1162	FOXO3	FASLG	TNFRSF1A	FASLG_TNFRSF1A
1163	FOXO3	FASLG	FAS	FASLG_FAS
1164	FOXO3	FASLG	TNFRSF6B	FASLG_TNFRSF6B
1165	SP3	FBLN1	ITGB1	FBLN1_ITGB1
1166	SP1	FBLN1	ITGB1	FBLN1_ITGB1
1167	SP1	FBN1	ITGB1	FBN1_ITGB1
1168	PARP1	FBN1	ITGB1	FBN1_ITGB1
1169	TFCP2	FGA	ITGAM	FGA_ITGAM
1170	TFCP2	FGA	PLAUR	FGA_PLAUR
1171	TFCP2	FGA	ITGB2	FGA_ITGB2
1172	TFCP2	FGA	ITGB1	FGA_ITGB1
1173	TFCP2	FGA	ITGAX	FGA_ITGAX
1174	FOXM1	FGB	ITGB2	FGB_ITGB2
1175	FOXM1	FGB	ITGB1	FGB_ITGB1
1176	FOXM1	FGB	ITGAM	FGB_ITGAM
1177	RFX1	FGF1	EGFR	FGF1_EGFR
1178	RFX1	FGF1	CD44	FGF1_CD44
1179	RFX2	FGF1	EGFR	FGF1_EGFR
1180	RFX2	FGF1	CD44	FGF1_CD44
1181	STAT3	FGF1	EGFR	FGF1_EGFR
1182	STAT3	FGF1	CD44	FGF1_CD44
1183	PPARG	FGF1	EGFR	FGF1_EGFR
1184	PPARG	FGF1	CD44	FGF1_CD44
1185	RFX3	FGF1	EGFR	FGF1_EGFR
1186	RFX3	FGF1	CD44	FGF1_CD44
1187	STAT3	FGF2	CD44	FGF2_CD44
1188	STAT3	FGF2	SDC4	FGF2_SDC4
1189	STAT3	FGF2	SDC1	FGF2_SDC1
1190	ERG	FGF2	CD44	FGF2_CD44
1191	ERG	FGF2	SDC4	FGF2_SDC4
1192	ERG	FGF2	SDC1	FGF2_SDC1
1193	HOXB7	FGF2	CD44	FGF2_CD44
1194	HOXB7	FGF2	SDC4	FGF2_SDC4
1195	HOXB7	FGF2	SDC1	FGF2_SDC1
1196	HIF1A	FGF2	CD44	FGF2_CD44
1197	HIF1A	FGF2	SDC4	FGF2_SDC4
1198	HIF1A	FGF2	SDC1	FGF2_SDC1
1199	EGR1	FGF2	CD44	FGF2_CD44
1200	EGR1	FGF2	SDC4	FGF2_SDC4
1201	EGR1	FGF2	SDC1	FGF2_SDC1
1202	PTTG1	FGF2	CD44	FGF2_CD44
1203	PTTG1	FGF2	SDC4	FGF2_SDC4
1204	PTTG1	FGF2	SDC1	FGF2_SDC1
1205	STAT1	FGF2	CD44	FGF2_CD44
1206	STAT1	FGF2	SDC4	FGF2_SDC4
1207	STAT1	FGF2	SDC1	FGF2_SDC1
1208	TP53	FGF2	CD44	FGF2_CD44
1209	TP53	FGF2	SDC4	FGF2_SDC4
1210	TP53	FGF2	SDC1	FGF2_SDC1
1211	HDAC5	FGF2	CD44	FGF2_CD44
1212	HDAC5	FGF2	SDC4	FGF2_SDC4
1213	HDAC5	FGF2	SDC1	FGF2_SDC1
1214	LMO2	FGF2	CD44	FGF2_CD44
1215	LMO2	FGF2	SDC4	FGF2_SDC4
1216	LMO2	FGF2	SDC1	FGF2_SDC1
1217	STAT3	FGG	ITGB1	FGG_ITGB1

1218	STAT3	FGG	ITGB2	FGG_ITGB2
1219	STAT3	FGL1	EGFR	FGL1_EGFR
1220	RELA	FN1	CD79A	FN1_CD79A
1221	RELA	FN1	ITGAV	FN1_ITGAV
1222	RELA	FN1	TSHR	FN1_TSHR
1223	RELA	FN1	ITGA6	FN1_ITGA6
1224	RELA	FN1	COL13A1	FN1_COL13A1
1225	RELA	FN1	ITGA8	FN1_ITGA8
1226	RELA	FN1	ITGA5	FN1_ITGA5
1227	RELA	FN1	ITGA9	FN1_ITGA9
1228	RELA	FN1	CD44	FN1_CD44
1229	RELA	FN1	IL17RC	FN1_IL17RC
1230	RELA	FN1	ITGB6	FN1_ITGB6
1231	RELA	FN1	NT5E	FN1_NT5E
1232	RELA	FN1	C5AR1	FN1_C5AR1
1233	RELA	FN1	FLT4	FN1_FLT4
1234	RELA	FN1	ITGB3	FN1_ITGB3
1235	RELA	FN1	ROBO4	FN1_ROBO4
1236	RELA	FN1	PLAUR	FN1_PLAUR
1237	RELA	FN1	ITGA2B	FN1_ITGA2B
1238	RELA	FN1	ITGA3	FN1_ITGA3
1239	RELA	FN1	SDC2	FN1_SDC2
1240	RELA	FN1	ITGA4	FN1_ITGA4
1241	RELA	FN1	TNFRSF11B	FN1_TNFRSF11B
1242	RELA	FN1	ITGB8	FN1_ITGB8
1243	RELA	FN1	ITGB1	FN1_ITGB1
1244	RELA	FN1	ITGA2	FN1_ITGA2
1245	RELA	FN1	TMPRSS6	FN1_TMPRSS6
1246	RELA	FN1	MAG	FN1_MAG
1247	RELA	FN1	ITGB7	FN1_ITGB7
1248	EGR1	FN1	CD79A	FN1_CD79A
1249	EGR1	FN1	ITGAV	FN1_ITGAV
1250	EGR1	FN1	TSHR	FN1_TSHR
1251	EGR1	FN1	ITGA6	FN1_ITGA6
1252	EGR1	FN1	COL13A1	FN1_COL13A1
1253	EGR1	FN1	ITGA8	FN1_ITGA8
1254	EGR1	FN1	ITGA5	FN1_ITGA5
1255	EGR1	FN1	ITGA9	FN1_ITGA9
1256	EGR1	FN1	CD44	FN1_CD44
1257	EGR1	FN1	IL17RC	FN1_IL17RC
1258	EGR1	FN1	ITGB6	FN1_ITGB6
1259	EGR1	FN1	NT5E	FN1_NT5E
1260	EGR1	FN1	C5AR1	FN1_C5AR1
1261	EGR1	FN1	FLT4	FN1_FLT4
1262	EGR1	FN1	ITGB3	FN1_ITGB3
1263	EGR1	FN1	ROBO4	FN1_ROBO4
1264	EGR1	FN1	PLAUR	FN1_PLAUR
1265	EGR1	FN1	ITGA2B	FN1_ITGA2B
1266	EGR1	FN1	ITGA3	FN1_ITGA3
1267	EGR1	FN1	SDC2	FN1_SDC2
1268	EGR1	FN1	ITGA4	FN1_ITGA4
1269	EGR1	FN1	TNFRSF11B	FN1_TNFRSF11B
1270	EGR1	FN1	ITGB8	FN1_ITGB8
1271	EGR1	FN1	ITGB1	FN1_ITGB1
1272	EGR1	FN1	ITGA2	FN1_ITGA2
1273	EGR1	FN1	TMPRSS6	FN1_TMPRSS6
1274	EGR1	FN1	MAG	FN1_MAG
1275	EGR1	FN1	ITGB7	FN1_ITGB7

1276	NFKB1	FN1	CD79A	FN1_CD79A
1277	NFKB1	FN1	ITGAV	FN1_ITGAV
1278	NFKB1	FN1	TSHR	FN1_TSHR
1279	NFKB1	FN1	ITGA6	FN1_ITGA6
1280	NFKB1	FN1	COL13A1	FN1_COL13A1
1281	NFKB1	FN1	ITGA8	FN1_ITGA8
1282	NFKB1	FN1	ITGA5	FN1_ITGA5
1283	NFKB1	FN1	ITGA9	FN1_ITGA9
1284	NFKB1	FN1	CD44	FN1_CD44
1285	NFKB1	FN1	IL17RC	FN1_IL17RC
1286	NFKB1	FN1	ITGB6	FN1_ITGB6
1287	NFKB1	FN1	NT5E	FN1_NT5E
1288	NFKB1	FN1	C5AR1	FN1_C5AR1
1289	NFKB1	FN1	FLT4	FN1_FLT4
1290	NFKB1	FN1	ITGB3	FN1_ITGB3
1291	NFKB1	FN1	ROBO4	FN1_ROBO4
1292	NFKB1	FN1	PLAUR	FN1_PLAUR
1293	NFKB1	FN1	ITGA2B	FN1_ITGA2B
1294	NFKB1	FN1	ITGA3	FN1_ITGA3
1295	NFKB1	FN1	SDC2	FN1_SDC2
1296	NFKB1	FN1	ITGA4	FN1_ITGA4
1297	NFKB1	FN1	TNFRSF11B	FN1_TNFRSF11B
1298	NFKB1	FN1	ITGB8	FN1_ITGB8
1299	NFKB1	FN1	ITGB1	FN1_ITGB1
1300	NFKB1	FN1	ITGA2	FN1_ITGA2
1301	NFKB1	FN1	TMPRSS6	FN1_TMPRSS6
1302	NFKB1	FN1	MAG	FN1_MAG
1303	NFKB1	FN1	ITGB7	FN1_ITGB7
1304	PARP1	FN1	CD79A	FN1_CD79A
1305	PARP1	FN1	ITGAV	FN1_ITGAV
1306	PARP1	FN1	TSHR	FN1_TSHR
1307	PARP1	FN1	ITGA6	FN1_ITGA6
1308	PARP1	FN1	COL13A1	FN1_COL13A1
1309	PARP1	FN1	ITGA8	FN1_ITGA8
1310	PARP1	FN1	ITGA5	FN1_ITGA5
1311	PARP1	FN1	ITGA9	FN1_ITGA9
1312	PARP1	FN1	CD44	FN1_CD44
1313	PARP1	FN1	IL17RC	FN1_IL17RC
1314	PARP1	FN1	ITGB6	FN1_ITGB6
1315	PARP1	FN1	NT5E	FN1_NT5E
1316	PARP1	FN1	C5AR1	FN1_C5AR1
1317	PARP1	FN1	FLT4	FN1_FLT4
1318	PARP1	FN1	ITGB3	FN1_ITGB3
1319	PARP1	FN1	ROBO4	FN1_ROBO4
1320	PARP1	FN1	PLAUR	FN1_PLAUR
1321	PARP1	FN1	ITGA2B	FN1_ITGA2B
1322	PARP1	FN1	ITGA3	FN1_ITGA3
1323	PARP1	FN1	SDC2	FN1_SDC2
1324	PARP1	FN1	ITGA4	FN1_ITGA4
1325	PARP1	FN1	TNFRSF11B	FN1_TNFRSF11B
1326	PARP1	FN1	ITGB8	FN1_ITGB8
1327	PARP1	FN1	ITGB1	FN1_ITGB1
1328	PARP1	FN1	ITGA2	FN1_ITGA2
1329	PARP1	FN1	TMPRSS6	FN1_TMPRSS6
1330	PARP1	FN1	MAG	FN1_MAG
1331	PARP1	FN1	ITGB7	FN1_ITGB7
1332	CEBPA	FN1	CD79A	FN1_CD79A
1333	CEBPA	FN1	ITGAV	FN1_ITGAV

1334 CEBPA	FN1	TSHR	FN1_TSHR
1335 CEBPA	FN1	ITGA6	FN1_ITGA6
1336 CEBPA	FN1	COL13A1	FN1_COL13A1
1337 CEBPA	FN1	ITGA8	FN1_ITGA8
1338 CEBPA	FN1	ITGA5	FN1_ITGA5
1339 CEBPA	FN1	ITGA9	FN1_ITGA9
1340 CEBPA	FN1	CD44	FN1_CD44
1341 CEBPA	FN1	IL17RC	FN1_IL17RC
1342 CEBPA	FN1	ITGB6	FN1_ITGB6
1343 CEBPA	FN1	NT5E	FN1_NT5E
1344 CEBPA	FN1	C5AR1	FN1_C5AR1
1345 CEBPA	FN1	FLT4	FN1_FLT4
1346 CEBPA	FN1	ITGB3	FN1_ITGB3
1347 CEBPA	FN1	ROBO4	FN1_ROBO4
1348 CEBPA	FN1	PLAUR	FN1_PLAUR
1349 CEBPA	FN1	ITGA2B	FN1_ITGA2B
1350 CEBPA	FN1	ITGA3	FN1_ITGA3
1351 CEBPA	FN1	SDC2	FN1_SDC2
1352 CEBPA	FN1	ITGA4	FN1_ITGA4
1353 CEBPA	FN1	TNFRSF11B	FN1_TNFRSF11B
1354 CEBPA	FN1	ITGB8	FN1_ITGB8
1355 CEBPA	FN1	ITGB1	FN1_ITGB1
1356 CEBPA	FN1	ITGA2	FN1_ITGA2
1357 CEBPA	FN1	TMPRSS6	FN1_TMPRSS6
1358 CEBPA	FN1	MAG	FN1_MAG
1359 CEBPA	FN1	ITGB7	FN1_ITGB7
1360 SOX17	FN1	CD79A	FN1_CD79A
1361 SOX17	FN1	ITGAV	FN1_ITGAV
1362 SOX17	FN1	TSHR	FN1_TSHR
1363 SOX17	FN1	ITGA6	FN1_ITGA6
1364 SOX17	FN1	COL13A1	FN1_COL13A1
1365 SOX17	FN1	ITGA8	FN1_ITGA8
1366 SOX17	FN1	ITGA5	FN1_ITGA5
1367 SOX17	FN1	ITGA9	FN1_ITGA9
1368 SOX17	FN1	CD44	FN1_CD44
1369 SOX17	FN1	IL17RC	FN1_IL17RC
1370 SOX17	FN1	ITGB6	FN1_ITGB6
1371 SOX17	FN1	NT5E	FN1_NT5E
1372 SOX17	FN1	C5AR1	FN1_C5AR1
1373 SOX17	FN1	FLT4	FN1_FLT4
1374 SOX17	FN1	ITGB3	FN1_ITGB3
1375 SOX17	FN1	ROBO4	FN1_ROBO4
1376 SOX17	FN1	PLAUR	FN1_PLAUR
1377 SOX17	FN1	ITGA2B	FN1_ITGA2B
1378 SOX17	FN1	ITGA3	FN1_ITGA3
1379 SOX17	FN1	SDC2	FN1_SDC2
1380 SOX17	FN1	ITGA4	FN1_ITGA4
1381 SOX17	FN1	TNFRSF11B	FN1_TNFRSF11B
1382 SOX17	FN1	ITGB8	FN1_ITGB8
1383 SOX17	FN1	ITGB1	FN1_ITGB1
1384 SOX17	FN1	ITGA2	FN1_ITGA2
1385 SOX17	FN1	TMPRSS6	FN1_TMPRSS6
1386 SOX17	FN1	MAG	FN1_MAG
1387 SOX17	FN1	ITGB7	FN1_ITGB7
1388 ATF2	FN1	CD79A	FN1_CD79A
1389 ATF2	FN1	ITGAV	FN1_ITGAV
1390 ATF2	FN1	TSHR	FN1_TSHR
1391 ATF2	FN1	ITGA6	FN1_ITGA6

1392	ATF2	FN1	COL13A1	FN1_COL13A1
1393	ATF2	FN1	ITGA8	FN1_ITGA8
1394	ATF2	FN1	ITGA5	FN1_ITGA5
1395	ATF2	FN1	ITGA9	FN1_ITGA9
1396	ATF2	FN1	CD44	FN1_CD44
1397	ATF2	FN1	IL17RC	FN1_IL17RC
1398	ATF2	FN1	ITGB6	FN1_ITGB6
1399	ATF2	FN1	NT5E	FN1_NT5E
1400	ATF2	FN1	C5AR1	FN1_C5AR1
1401	ATF2	FN1	FLT4	FN1_FLT4
1402	ATF2	FN1	ITGB3	FN1_ITGB3
1403	ATF2	FN1	ROBO4	FN1_ROBO4
1404	ATF2	FN1	PLAUR	FN1_PLAUR
1405	ATF2	FN1	ITGA2B	FN1_ITGA2B
1406	ATF2	FN1	ITGA3	FN1_ITGA3
1407	ATF2	FN1	SDC2	FN1_SDC2
1408	ATF2	FN1	ITGA4	FN1_ITGA4
1409	ATF2	FN1	TNFRSF11B	FN1_TNFRSF11B
1410	ATF2	FN1	ITGB8	FN1_ITGB8
1411	ATF2	FN1	ITGB1	FN1_ITGB1
1412	ATF2	FN1	ITGA2	FN1_ITGA2
1413	ATF2	FN1	TMPRSS6	FN1_TMPRSS6
1414	ATF2	FN1	MAG	FN1_MAG
1415	ATF2	FN1	ITGB7	FN1_ITGB7
1416	TWIST1	FN1	CD79A	FN1_CD79A
1417	TWIST1	FN1	ITGAV	FN1_ITGAV
1418	TWIST1	FN1	TSHR	FN1_TSHR
1419	TWIST1	FN1	ITGA6	FN1_ITGA6
1420	TWIST1	FN1	COL13A1	FN1_COL13A1
1421	TWIST1	FN1	ITGA8	FN1_ITGA8
1422	TWIST1	FN1	ITGA5	FN1_ITGA5
1423	TWIST1	FN1	ITGA9	FN1_ITGA9
1424	TWIST1	FN1	CD44	FN1_CD44
1425	TWIST1	FN1	IL17RC	FN1_IL17RC
1426	TWIST1	FN1	ITGB6	FN1_ITGB6
1427	TWIST1	FN1	NT5E	FN1_NT5E
1428	TWIST1	FN1	C5AR1	FN1_C5AR1
1429	TWIST1	FN1	FLT4	FN1_FLT4
1430	TWIST1	FN1	ITGB3	FN1_ITGB3
1431	TWIST1	FN1	ROBO4	FN1_ROBO4
1432	TWIST1	FN1	PLAUR	FN1_PLAUR
1433	TWIST1	FN1	ITGA2B	FN1_ITGA2B
1434	TWIST1	FN1	ITGA3	FN1_ITGA3
1435	TWIST1	FN1	SDC2	FN1_SDC2
1436	TWIST1	FN1	ITGA4	FN1_ITGA4
1437	TWIST1	FN1	TNFRSF11B	FN1_TNFRSF11B
1438	TWIST1	FN1	ITGB8	FN1_ITGB8
1439	TWIST1	FN1	ITGB1	FN1_ITGB1
1440	TWIST1	FN1	ITGA2	FN1_ITGA2
1441	TWIST1	FN1	TMPRSS6	FN1_TMPRSS6
1442	TWIST1	FN1	MAG	FN1_MAG
1443	TWIST1	FN1	ITGB7	FN1_ITGB7
1444	TWIST2	FN1	CD79A	FN1_CD79A
1445	TWIST2	FN1	ITGAV	FN1_ITGAV
1446	TWIST2	FN1	TSHR	FN1_TSHR
1447	TWIST2	FN1	ITGA6	FN1_ITGA6
1448	TWIST2	FN1	COL13A1	FN1_COL13A1
1449	TWIST2	FN1	ITGA8	FN1_ITGA8

1450	TWIST2	FN1	ITGA5	FN1_ITGA5
1451	TWIST2	FN1	ITGA9	FN1_ITGA9
1452	TWIST2	FN1	CD44	FN1_CD44
1453	TWIST2	FN1	IL17RC	FN1_IL17RC
1454	TWIST2	FN1	ITGB6	FN1_ITGB6
1455	TWIST2	FN1	NT5E	FN1_NT5E
1456	TWIST2	FN1	C5AR1	FN1_C5AR1
1457	TWIST2	FN1	FLT4	FN1_FLT4
1458	TWIST2	FN1	ITGB3	FN1_ITGB3
1459	TWIST2	FN1	ROBO4	FN1_ROBO4
1460	TWIST2	FN1	PLAUR	FN1_PLAUR
1461	TWIST2	FN1	ITGA2B	FN1_ITGA2B
1462	TWIST2	FN1	ITGA3	FN1_ITGA3
1463	TWIST2	FN1	SDC2	FN1_SDC2
1464	TWIST2	FN1	ITGA4	FN1_ITGA4
1465	TWIST2	FN1	TNFRSF11B	FN1_TNFRSF11B
1466	TWIST2	FN1	ITGB8	FN1_ITGB8
1467	TWIST2	FN1	ITGB1	FN1_ITGB1
1468	TWIST2	FN1	ITGA2	FN1_ITGA2
1469	TWIST2	FN1	TMPRSS6	FN1_TMPRSS6
1470	TWIST2	FN1	MAG	FN1_MAG
1471	TWIST2	FN1	ITGB7	FN1_ITGB7
1472	KLF8	FN1	CD79A	FN1_CD79A
1473	KLF8	FN1	ITGAV	FN1_ITGAV
1474	KLF8	FN1	TSHR	FN1_TSHR
1475	KLF8	FN1	ITGA6	FN1_ITGA6
1476	KLF8	FN1	COL13A1	FN1_COL13A1
1477	KLF8	FN1	ITGA8	FN1_ITGA8
1478	KLF8	FN1	ITGA5	FN1_ITGA5
1479	KLF8	FN1	ITGA9	FN1_ITGA9
1480	KLF8	FN1	CD44	FN1_CD44
1481	KLF8	FN1	IL17RC	FN1_IL17RC
1482	KLF8	FN1	ITGB6	FN1_ITGB6
1483	KLF8	FN1	NT5E	FN1_NT5E
1484	KLF8	FN1	C5AR1	FN1_C5AR1
1485	KLF8	FN1	FLT4	FN1_FLT4
1486	KLF8	FN1	ITGB3	FN1_ITGB3
1487	KLF8	FN1	ROBO4	FN1_ROBO4
1488	KLF8	FN1	PLAUR	FN1_PLAUR
1489	KLF8	FN1	ITGA2B	FN1_ITGA2B
1490	KLF8	FN1	ITGA3	FN1_ITGA3
1491	KLF8	FN1	SDC2	FN1_SDC2
1492	KLF8	FN1	ITGA4	FN1_ITGA4
1493	KLF8	FN1	TNFRSF11B	FN1_TNFRSF11B
1494	KLF8	FN1	ITGB8	FN1_ITGB8
1495	KLF8	FN1	ITGB1	FN1_ITGB1
1496	KLF8	FN1	ITGA2	FN1_ITGA2
1497	KLF8	FN1	TMPRSS6	FN1_TMPRSS6
1498	KLF8	FN1	MAG	FN1_MAG
1499	KLF8	FN1	ITGB7	FN1_ITGB7
1500	AR	FN1	CD79A	FN1_CD79A
1501	AR	FN1	ITGAV	FN1_ITGAV
1502	AR	FN1	TSHR	FN1_TSHR
1503	AR	FN1	ITGA6	FN1_ITGA6
1504	AR	FN1	COL13A1	FN1_COL13A1
1505	AR	FN1	ITGA8	FN1_ITGA8
1506	AR	FN1	ITGA5	FN1_ITGA5
1507	AR	FN1	ITGA9	FN1_ITGA9

1508	AR	FN1	CD44	FN1_CD44
1509	AR	FN1	IL17RC	FN1_IL17RC
1510	AR	FN1	ITGB6	FN1_ITGB6
1511	AR	FN1	NT5E	FN1_NT5E
1512	AR	FN1	C5AR1	FN1_C5AR1
1513	AR	FN1	FLT4	FN1_FLT4
1514	AR	FN1	ITGB3	FN1_ITGB3
1515	AR	FN1	ROBO4	FN1_ROBO4
1516	AR	FN1	PLAUR	FN1_PLAUR
1517	AR	FN1	ITGA2B	FN1_ITGA2B
1518	AR	FN1	ITGA3	FN1_ITGA3
1519	AR	FN1	SDC2	FN1_SDC2
1520	AR	FN1	ITGA4	FN1_ITGA4
1521	AR	FN1	TNFRSF11B	FN1_TNFRSF11B
1522	AR	FN1	ITGB8	FN1_ITGB8
1523	AR	FN1	ITGB1	FN1_ITGB1
1524	AR	FN1	ITGA2	FN1_ITGA2
1525	AR	FN1	TMPRSS6	FN1_TMPRSS6
1526	AR	FN1	MAG	FN1_MAG
1527	AR	FN1	ITGB7	FN1_ITGB7
1528	SNAI1	FN1	CD79A	FN1_CD79A
1529	SNAI1	FN1	ITGAV	FN1_ITGAV
1530	SNAI1	FN1	TSHR	FN1_TSHR
1531	SNAI1	FN1	ITGA6	FN1_ITGA6
1532	SNAI1	FN1	COL13A1	FN1_COL13A1
1533	SNAI1	FN1	ITGA8	FN1_ITGA8
1534	SNAI1	FN1	ITGA5	FN1_ITGA5
1535	SNAI1	FN1	ITGA9	FN1_ITGA9
1536	SNAI1	FN1	CD44	FN1_CD44
1537	SNAI1	FN1	IL17RC	FN1_IL17RC
1538	SNAI1	FN1	ITGB6	FN1_ITGB6
1539	SNAI1	FN1	NT5E	FN1_NT5E
1540	SNAI1	FN1	C5AR1	FN1_C5AR1
1541	SNAI1	FN1	FLT4	FN1_FLT4
1542	SNAI1	FN1	ITGB3	FN1_ITGB3
1543	SNAI1	FN1	ROBO4	FN1_ROBO4
1544	SNAI1	FN1	PLAUR	FN1_PLAUR
1545	SNAI1	FN1	ITGA2B	FN1_ITGA2B
1546	SNAI1	FN1	ITGA3	FN1_ITGA3
1547	SNAI1	FN1	SDC2	FN1_SDC2
1548	SNAI1	FN1	ITGA4	FN1_ITGA4
1549	SNAI1	FN1	TNFRSF11B	FN1_TNFRSF11B
1550	SNAI1	FN1	ITGB8	FN1_ITGB8
1551	SNAI1	FN1	ITGB1	FN1_ITGB1
1552	SNAI1	FN1	ITGA2	FN1_ITGA2
1553	SNAI1	FN1	TMPRSS6	FN1_TMPRSS6
1554	SNAI1	FN1	MAG	FN1_MAG
1555	SNAI1	FN1	ITGB7	FN1_ITGB7
1556	GATA1	GNAS	LHCGR	GNAS_LHCGR
1557	GATA1	GNAS	GCGR	GNAS_GCGR
1558	GATA1	GNAS	ADORA1	GNAS_ADORA1
1559	GATA1	GNAS	ADCY9	GNAS_ADCY9
1560	GATA1	GNAS	PTGDR	GNAS_PTGDR
1561	GATA1	GNAS	GLP1R	GNAS_GLP1R
1562	GATA1	GNAS	ADRB3	GNAS_ADRB3
1563	GATA1	GNAS	ADCY8	GNAS_ADCY8
1564	GATA1	GNAS	HTR6	GNAS_HTR6
1565	GATA1	GNAS	CRHR1	GNAS_CRHR1

1566	GATA1	GNAS	AVPR2	GNAS_AVPR2
1567	GATA1	GNAS	ADCY1	GNAS_ADCY1
1568	GATA1	GNAS	ADCY7	GNAS_ADCY7
1569	GATA1	GNAS	VIPR1	GNAS_VIPR1
1570	GATA1	GNAS	PTGIR	GNAS_PTGIR
1571	GATA1	GNAS	TSHR	GNAS_TSHR
1572	YY1	GNAS	LHCGR	GNAS_LHCGR
1573	YY1	GNAS	GCGR	GNAS_GCGR
1574	YY1	GNAS	ADORA1	GNAS_ADORA1
1575	YY1	GNAS	ADCY9	GNAS_ADCY9
1576	YY1	GNAS	PTGDR	GNAS_PTGDR
1577	YY1	GNAS	GLP1R	GNAS_GLP1R
1578	YY1	GNAS	ADRB3	GNAS_ADRB3
1579	YY1	GNAS	ADCY8	GNAS_ADCY8
1580	YY1	GNAS	HTR6	GNAS_HTR6
1581	YY1	GNAS	CRHR1	GNAS_CRHR1
1582	YY1	GNAS	AVPR2	GNAS_AVPR2
1583	YY1	GNAS	ADCY1	GNAS_ADCY1
1584	YY1	GNAS	ADCY7	GNAS_ADCY7
1585	YY1	GNAS	VIPR1	GNAS_VIPR1
1586	YY1	GNAS	PTGIR	GNAS_PTGIR
1587	YY1	GNAS	TSHR	GNAS_TSHR
1588	NFKB1	GSTP1	TRAF2	GSTP1_TRAF2
1589	JUN	GSTP1	TRAF2	GSTP1_TRAF2
1590	SP1	GSTP1	TRAF2	GSTP1_TRAF2
1591	TP53	GSTP1	TRAF2	GSTP1_TRAF2
1592	CDX2	GSTP1	TRAF2	GSTP1_TRAF2
1593	MBD2	GSTP1	TRAF2	GSTP1_TRAF2
1594	FOS	GSTP1	TRAF2	GSTP1_TRAF2
1595	RELA	GSTP1	TRAF2	GSTP1_TRAF2
1596	CEBPZ	GZMB	IGF2R	GZMB_IGF2R
1597	CEBPZ	GZMB	PGRMC1	GZMB_PGRMC1
1598	CEBPZ	GZMB	CHRM3	GZMB_CHRM3
1599	SP1	HAS2	CD44	HAS2_CD44
1600	SP1	HAS2	HMMR	HAS2_HMMR
1601	YY1	HAS2	CD44	HAS2_CD44
1602	YY1	HAS2	HMMR	HAS2_HMMR
1603	SP1	HBEGF	PRLR	HBEGF_PRLR
1604	SP1	HBEGF	ERBB2	HBEGF_ERBB2
1605	SP1	HBEGF	EGFR	HBEGF_EGFR
1606	SP1	HBEGF	CD82	HBEGF_CD82
1607	SP1	HBEGF	CD44	HBEGF_CD44
1608	SP1	HBEGF	CD9	HBEGF_CD9
1609	SP1	HBEGF	ERBB4	HBEGF_ERBB4
1610	SP3	HBEGF	PRLR	HBEGF_PRLR
1611	SP3	HBEGF	ERBB2	HBEGF_ERBB2
1612	SP3	HBEGF	EGFR	HBEGF_EGFR
1613	SP3	HBEGF	CD82	HBEGF_CD82
1614	SP3	HBEGF	CD44	HBEGF_CD44
1615	SP3	HBEGF	CD9	HBEGF_CD9
1616	SP3	HBEGF	ERBB4	HBEGF_ERBB4
1617	WT1	HBEGF	PRLR	HBEGF_PRLR
1618	WT1	HBEGF	ERBB2	HBEGF_ERBB2
1619	WT1	HBEGF	EGFR	HBEGF_EGFR
1620	WT1	HBEGF	CD82	HBEGF_CD82
1621	WT1	HBEGF	CD44	HBEGF_CD44
1622	WT1	HBEGF	CD9	HBEGF_CD9
1623	WT1	HBEGF	ERBB4	HBEGF_ERBB4

1624 HIF1A	HBEGF	PRLR	HBEGF_PRLR
1625 HIF1A	HBEGF	ERBB2	HBEGF_ERBB2
1626 HIF1A	HBEGF	EGFR	HBEGF_EGFR
1627 HIF1A	HBEGF	CD82	HBEGF_CD82
1628 HIF1A	HBEGF	CD44	HBEGF_CD44
1629 HIF1A	HBEGF	CD9	HBEGF_CD9
1630 HIF1A	HBEGF	ERBB4	HBEGF_ERBB4
1631 NFKB1	HBEGF	PRLR	HBEGF_PRLR
1632 NFKB1	HBEGF	ERBB2	HBEGF_ERBB2
1633 NFKB1	HBEGF	EGFR	HBEGF_EGFR
1634 NFKB1	HBEGF	CD82	HBEGF_CD82
1635 NFKB1	HBEGF	CD44	HBEGF_CD44
1636 NFKB1	HBEGF	CD9	HBEGF_CD9
1637 NFKB1	HBEGF	ERBB4	HBEGF_ERBB4
1638 SNAI1	HBEGF	PRLR	HBEGF_PRLR
1639 SNAI1	HBEGF	ERBB2	HBEGF_ERBB2
1640 SNAI1	HBEGF	EGFR	HBEGF_EGFR
1641 SNAI1	HBEGF	CD82	HBEGF_CD82
1642 SNAI1	HBEGF	CD44	HBEGF_CD44
1643 SNAI1	HBEGF	CD9	HBEGF_CD9
1644 SNAI1	HBEGF	ERBB4	HBEGF_ERBB4
1645 MYOD1	HBEGF	PRLR	HBEGF_PRLR
1646 MYOD1	HBEGF	ERBB2	HBEGF_ERBB2
1647 MYOD1	HBEGF	EGFR	HBEGF_EGFR
1648 MYOD1	HBEGF	CD82	HBEGF_CD82
1649 MYOD1	HBEGF	CD44	HBEGF_CD44
1650 MYOD1	HBEGF	CD9	HBEGF_CD9
1651 MYOD1	HBEGF	ERBB4	HBEGF_ERBB4
1652 RELA	HBEGF	PRLR	HBEGF_PRLR
1653 RELA	HBEGF	ERBB2	HBEGF_ERBB2
1654 RELA	HBEGF	EGFR	HBEGF_EGFR
1655 RELA	HBEGF	CD82	HBEGF_CD82
1656 RELA	HBEGF	CD44	HBEGF_CD44
1657 RELA	HBEGF	CD9	HBEGF_CD9
1658 RELA	HBEGF	ERBB4	HBEGF_ERBB4
1659 KAT5	HDC	HRH1	HDC_HRH1
1660 KAT5	HDC	HRH4	HDC_HRH4
1661 KAT5	HDC	HRH2	HDC_HRH2
1662 KAT5	HDC	HRH3	HDC_HRH3
1663 KLF4	HDC	HRH1	HDC_HRH1
1664 KLF4	HDC	HRH4	HDC_HRH4
1665 KLF4	HDC	HRH2	HDC_HRH2
1666 KLF4	HDC	HRH3	HDC_HRH3
1667 HDAC7	HDC	HRH1	HDC_HRH1
1668 HDAC7	HDC	HRH4	HDC_HRH4
1669 HDAC7	HDC	HRH2	HDC_HRH2
1670 HDAC7	HDC	HRH3	HDC_HRH3
1671 TFAP2A	HGF	ST14	HGF_ST14
1672 TFAP2A	HGF	SDC1	HGF_SDC1
1673 TFAP2A	HGF	MET	HGF_MET
1674 TFAP2A	HGF	CD44	HGF_CD44
1675 SP1	HGF	ST14	HGF_ST14
1676 SP1	HGF	SDC1	HGF_SDC1
1677 SP1	HGF	MET	HGF_MET
1678 SP1	HGF	CD44	HGF_CD44
1679 NFIC	HGF	ST14	HGF_ST14
1680 NFIC	HGF	SDC1	HGF_SDC1
1681 NFIC	HGF	MET	HGF_MET

1682	NFIC	HGF	CD44	HGF_CD44
1683	SP3	HGF	ST14	HGF_ST14
1684	SP3	HGF	SDC1	HGF_SDC1
1685	SP3	HGF	MET	HGF_MET
1686	SP3	HGF	CD44	HGF_CD44
1687	STAT3	HGF	ST14	HGF_ST14
1688	STAT3	HGF	SDC1	HGF_SDC1
1689	STAT3	HGF	MET	HGF_MET
1690	STAT3	HGF	CD44	HGF_CD44
1691	HIVEP2	HLA-A	LILRB2	HLA-A_LILRB2
1692	HIVEP2	HLA-A	APLP2	HLA-A_APLP2
1693	HIVEP2	HLA-A	KIR2DL3	HLA-A_KIR2DL3
1694	HIVEP2	HLA-A	CD3D	HLA-A_CD3D
1695	HIVEP2	HLA-A	KIR3DL1	HLA-A_KIR3DL1
1696	HIVEP2	HLA-A	CD3G	HLA-A_CD3G
1697	HIVEP2	HLA-A	KIR3DL2	HLA-A_KIR3DL2
1698	HIVEP2	HLA-A	LILRB1	HLA-A_LILRB1
1699	HIVEP2	HLA-A	KIR2DL1	HLA-A_KIR2DL1
1700	HIVEP2	HLA-A	ERBB2	HLA-A_ERBB2
1701	CIITA	HLA-A	LILRB2	HLA-A_LILRB2
1702	CIITA	HLA-A	APLP2	HLA-A_APLP2
1703	CIITA	HLA-A	KIR2DL3	HLA-A_KIR2DL3
1704	CIITA	HLA-A	CD3D	HLA-A_CD3D
1705	CIITA	HLA-A	KIR3DL1	HLA-A_KIR3DL1
1706	CIITA	HLA-A	CD3G	HLA-A_CD3G
1707	CIITA	HLA-A	KIR3DL2	HLA-A_KIR3DL2
1708	CIITA	HLA-A	LILRB1	HLA-A_LILRB1
1709	CIITA	HLA-A	KIR2DL1	HLA-A_KIR2DL1
1710	CIITA	HLA-A	ERBB2	HLA-A_ERBB2
1711	RELA	HLA-A	LILRB2	HLA-A_LILRB2
1712	RELA	HLA-A	APLP2	HLA-A_APLP2
1713	RELA	HLA-A	KIR2DL3	HLA-A_KIR2DL3
1714	RELA	HLA-A	CD3D	HLA-A_CD3D
1715	RELA	HLA-A	KIR3DL1	HLA-A_KIR3DL1
1716	RELA	HLA-A	CD3G	HLA-A_CD3G
1717	RELA	HLA-A	KIR3DL2	HLA-A_KIR3DL2
1718	RELA	HLA-A	LILRB1	HLA-A_LILRB1
1719	RELA	HLA-A	KIR2DL1	HLA-A_KIR2DL1
1720	RELA	HLA-A	ERBB2	HLA-A_ERBB2
1721	MYCN	HLA-A	LILRB2	HLA-A_LILRB2
1722	MYCN	HLA-A	APLP2	HLA-A_APLP2
1723	MYCN	HLA-A	KIR2DL3	HLA-A_KIR2DL3
1724	MYCN	HLA-A	CD3D	HLA-A_CD3D
1725	MYCN	HLA-A	KIR3DL1	HLA-A_KIR3DL1
1726	MYCN	HLA-A	CD3G	HLA-A_CD3G
1727	MYCN	HLA-A	KIR3DL2	HLA-A_KIR3DL2
1728	MYCN	HLA-A	LILRB1	HLA-A_LILRB1
1729	MYCN	HLA-A	KIR2DL1	HLA-A_KIR2DL1
1730	MYCN	HLA-A	ERBB2	HLA-A_ERBB2
1731	MYC	HLA-A	LILRB2	HLA-A_LILRB2
1732	MYC	HLA-A	APLP2	HLA-A_APLP2
1733	MYC	HLA-A	KIR2DL3	HLA-A_KIR2DL3
1734	MYC	HLA-A	CD3D	HLA-A_CD3D
1735	MYC	HLA-A	KIR3DL1	HLA-A_KIR3DL1
1736	MYC	HLA-A	CD3G	HLA-A_CD3G
1737	MYC	HLA-A	KIR3DL2	HLA-A_KIR3DL2
1738	MYC	HLA-A	LILRB1	HLA-A_LILRB1
1739	MYC	HLA-A	KIR2DL1	HLA-A_KIR2DL1

1740	MYC	HLA-A	ERBB2	HLA-A_ERBB2
1741	IRF1	HLA-A	LILRB2	HLA-A_LILRB2
1742	IRF1	HLA-A	APLP2	HLA-A_APLP2
1743	IRF1	HLA-A	KIR2DL3	HLA-A_KIR2DL3
1744	IRF1	HLA-A	CD3D	HLA-A_CD3D
1745	IRF1	HLA-A	KIR3DL1	HLA-A_KIR3DL1
1746	IRF1	HLA-A	CD3G	HLA-A_CD3G
1747	IRF1	HLA-A	KIR3DL2	HLA-A_KIR3DL2
1748	IRF1	HLA-A	LILRB1	HLA-A_LILRB1
1749	IRF1	HLA-A	KIR2DL1	HLA-A_KIR2DL1
1750	IRF1	HLA-A	ERBB2	HLA-A_ERBB2
1751	HIVEP2	HLA-B	CANX	HLA-B_CANX
1752	HIVEP2	HLA-B	KIR2DL3	HLA-B_KIR2DL3
1753	HIVEP2	HLA-B	CD3D	HLA-B_CD3D
1754	HIVEP2	HLA-B	CD3G	HLA-B_CD3G
1755	HIVEP2	HLA-B	KIR3DL1	HLA-B_KIR3DL1
1756	HIVEP2	HLA-B	KLRD1	HLA-B_KLRD1
1757	HIVEP2	HLA-B	LILRB2	HLA-B_LILRB2
1758	HIVEP2	HLA-B	LILRB1	HLA-B_LILRB1
1759	MYC	HLA-B	CANX	HLA-B_CANX
1760	MYC	HLA-B	KIR2DL3	HLA-B_KIR2DL3
1761	MYC	HLA-B	CD3D	HLA-B_CD3D
1762	MYC	HLA-B	CD3G	HLA-B_CD3G
1763	MYC	HLA-B	KIR3DL1	HLA-B_KIR3DL1
1764	MYC	HLA-B	KLRD1	HLA-B_KLRD1
1765	MYC	HLA-B	LILRB2	HLA-B_LILRB2
1766	MYC	HLA-B	LILRB1	HLA-B_LILRB1
1767	MYCN	HLA-B	CANX	HLA-B_CANX
1768	MYCN	HLA-B	KIR2DL3	HLA-B_KIR2DL3
1769	MYCN	HLA-B	CD3D	HLA-B_CD3D
1770	MYCN	HLA-B	CD3G	HLA-B_CD3G
1771	MYCN	HLA-B	KIR3DL1	HLA-B_KIR3DL1
1772	MYCN	HLA-B	KLRD1	HLA-B_KLRD1
1773	MYCN	HLA-B	LILRB2	HLA-B_LILRB2
1774	MYCN	HLA-B	LILRB1	HLA-B_LILRB1
1775	CIITA	HLA-B	CANX	HLA-B_CANX
1776	CIITA	HLA-B	KIR2DL3	HLA-B_KIR2DL3
1777	CIITA	HLA-B	CD3D	HLA-B_CD3D
1778	CIITA	HLA-B	CD3G	HLA-B_CD3G
1779	CIITA	HLA-B	KIR3DL1	HLA-B_KIR3DL1
1780	CIITA	HLA-B	KLRD1	HLA-B_KLRD1
1781	CIITA	HLA-B	LILRB2	HLA-B_LILRB2
1782	CIITA	HLA-B	LILRB1	HLA-B_LILRB1
1783	CIITA	HLA-C	KIR2DL1	HLA-C_KIR2DL1
1784	CIITA	HLA-C	KIR2DS4	HLA-C_KIR2DS4
1785	CIITA	HLA-C	DDR1	HLA-C_DDR1
1786	CIITA	HLA-C	KIR2DL3	HLA-C_KIR2DL3
1787	CIITA	HLA-C	LILRB2	HLA-C_LILRB2
1788	CIITA	HLA-C	KIR3DL1	HLA-C_KIR3DL1
1789	CIITA	HLA-C	CD3G	HLA-C_CD3G
1790	CIITA	HLA-C	SLC9C2	HLA-C_SLC9C2
1791	CIITA	HLA-C	LILRB1	HLA-C_LILRB1
1792	CIITA	HLA-C	LILRA3	HLA-C_LILRA3
1793	CIITA	HLA-C	CD3D	HLA-C_CD3D
1794	CIITA	HLA-C	NOTCH4	HLA-C_NOTCH4
1795	HIVEP2	HLA-C	KIR2DL1	HLA-C_KIR2DL1
1796	HIVEP2	HLA-C	KIR2DS4	HLA-C_KIR2DS4
1797	HIVEP2	HLA-C	DDR1	HLA-C_DDR1

1798	HIVEP2	HLA-C	KIR2DL3	HLA-C_KIR2DL3
1799	HIVEP2	HLA-C	LILRB2	HLA-C_LILRB2
1800	HIVEP2	HLA-C	KIR3DL1	HLA-C_KIR3DL1
1801	HIVEP2	HLA-C	CD3G	HLA-C_CD3G
1802	HIVEP2	HLA-C	SLC9C2	HLA-C_SLC9C2
1803	HIVEP2	HLA-C	LILRB1	HLA-C_LILRB1
1804	HIVEP2	HLA-C	LILRA3	HLA-C_LILRA3
1805	HIVEP2	HLA-C	CD3D	HLA-C_CD3D
1806	HIVEP2	HLA-C	NOTCH4	HLA-C_NOTCH4
1807	MYC	HLA-C	KIR2DL1	HLA-C_KIR2DL1
1808	MYC	HLA-C	KIR2DS4	HLA-C_KIR2DS4
1809	MYC	HLA-C	DDR1	HLA-C_DDR1
1810	MYC	HLA-C	KIR2DL3	HLA-C_KIR2DL3
1811	MYC	HLA-C	LILRB2	HLA-C_LILRB2
1812	MYC	HLA-C	KIR3DL1	HLA-C_KIR3DL1
1813	MYC	HLA-C	CD3G	HLA-C_CD3G
1814	MYC	HLA-C	SLC9C2	HLA-C_SLC9C2
1815	MYC	HLA-C	LILRB1	HLA-C_LILRB1
1816	MYC	HLA-C	LILRA3	HLA-C_LILRA3
1817	MYC	HLA-C	CD3D	HLA-C_CD3D
1818	MYC	HLA-C	NOTCH4	HLA-C_NOTCH4
1819	MYCN	HLA-C	KIR2DL1	HLA-C_KIR2DL1
1820	MYCN	HLA-C	KIR2DS4	HLA-C_KIR2DS4
1821	MYCN	HLA-C	DDR1	HLA-C_DDR1
1822	MYCN	HLA-C	KIR2DL3	HLA-C_KIR2DL3
1823	MYCN	HLA-C	LILRB2	HLA-C_LILRB2
1824	MYCN	HLA-C	KIR3DL1	HLA-C_KIR3DL1
1825	MYCN	HLA-C	CD3G	HLA-C_CD3G
1826	MYCN	HLA-C	SLC9C2	HLA-C_SLC9C2
1827	MYCN	HLA-C	LILRB1	HLA-C_LILRB1
1828	MYCN	HLA-C	LILRA3	HLA-C_LILRA3
1829	MYCN	HLA-C	CD3D	HLA-C_CD3D
1830	MYCN	HLA-C	NOTCH4	HLA-C_NOTCH4
1831	HIVEP2	HLA-E	KLRC1	HLA-E_KLRC1
1832	HIVEP2	HLA-E	KIR3DL1	HLA-E_KIR3DL1
1833	HIVEP2	HLA-E	KLRC2	HLA-E_KLRC2
1834	HIVEP2	HLA-E	KLRD1	HLA-E_KLRD1
1835	HIVEP2	HLA-E	SLC16A4	HLA-E_SLC16A4
1836	MYCN	HLA-E	KLRC1	HLA-E_KLRC1
1837	MYCN	HLA-E	KIR3DL1	HLA-E_KIR3DL1
1838	MYCN	HLA-E	KLRC2	HLA-E_KLRC2
1839	MYCN	HLA-E	KLRD1	HLA-E_KLRD1
1840	MYCN	HLA-E	SLC16A4	HLA-E_SLC16A4
1841	MYC	HLA-E	KLRC1	HLA-E_KLRC1
1842	MYC	HLA-E	KIR3DL1	HLA-E_KIR3DL1
1843	MYC	HLA-E	KLRC2	HLA-E_KLRC2
1844	MYC	HLA-E	KLRD1	HLA-E_KLRD1
1845	MYC	HLA-E	SLC16A4	HLA-E_SLC16A4
1846	STAT1	HLA-E	KLRC1	HLA-E_KLRC1
1847	STAT1	HLA-E	KIR3DL1	HLA-E_KIR3DL1
1848	STAT1	HLA-E	KLRC2	HLA-E_KLRC2
1849	STAT1	HLA-E	KLRD1	HLA-E_KLRD1
1850	STAT1	HLA-E	SLC16A4	HLA-E_SLC16A4
1851	MYCN	HLA-G	LILRB1	HLA-G_LILRB1
1852	MYCN	HLA-G	KLRD1	HLA-G_KLRD1
1853	MYCN	HLA-G	LILRB2	HLA-G_LILRB2
1854	MYCN	HLA-G	CD4	HLA-G_CD4
1855	HSF1	HLA-G	LILRB1	HLA-G_LILRB1

1856	HSF1	HLA-G	KLRD1	HLA-G_KLRD1
1857	HSF1	HLA-G	LILRB2	HLA-G_LILRB2
1858	HSF1	HLA-G	CD4	HLA-G_CD4
1859	IRF1	HLA-G	LILRB1	HLA-G_LILRB1
1860	IRF1	HLA-G	KLRD1	HLA-G_KLRD1
1861	IRF1	HLA-G	LILRB2	HLA-G_LILRB2
1862	IRF1	HLA-G	CD4	HLA-G_CD4
1863	CIITA	HLA-G	LILRB1	HLA-G_LILRB1
1864	CIITA	HLA-G	KLRD1	HLA-G_KLRD1
1865	CIITA	HLA-G	LILRB2	HLA-G_LILRB2
1866	CIITA	HLA-G	CD4	HLA-G_CD4
1867	MYC	HLA-G	LILRB1	HLA-G_LILRB1
1868	MYC	HLA-G	KLRD1	HLA-G_KLRD1
1869	MYC	HLA-G	LILRB2	HLA-G_LILRB2
1870	MYC	HLA-G	CD4	HLA-G_CD4
1871	GLI3	HLA-G	LILRB1	HLA-G_LILRB1
1872	GLI3	HLA-G	KLRD1	HLA-G_KLRD1
1873	GLI3	HLA-G	LILRB2	HLA-G_LILRB2
1874	GLI3	HLA-G	CD4	HLA-G_CD4
1875	SP1	HLA-G	LILRB1	HLA-G_LILRB1
1876	SP1	HLA-G	KLRD1	HLA-G_KLRD1
1877	SP1	HLA-G	LILRB2	HLA-G_LILRB2
1878	SP1	HLA-G	CD4	HLA-G_CD4
1879	HIVEP2	HLA-G	LILRB1	HLA-G_LILRB1
1880	HIVEP2	HLA-G	KLRD1	HLA-G_KLRD1
1881	HIVEP2	HLA-G	LILRB2	HLA-G_LILRB2
1882	HIVEP2	HLA-G	CD4	HLA-G_CD4
1883	NFIC	HMGB1	THBD	HMGB1_THBD
1884	NFIC	HMGB1	SDC1	HMGB1_SDC1
1885	NR1I2	HP	ASGR1	HP_ASGR1
1886	NR1I2	HP	ITGB2	HP_ITGB2
1887	NR1I2	HP	ITGAM	HP_ITGAM
1888	NR1I2	HP	ASGR2	HP_ASGR2
1889	CEBPB	HP	ASGR1	HP_ASGR1
1890	CEBPB	HP	ITGB2	HP_ITGB2
1891	CEBPB	HP	ITGAM	HP_ITGAM
1892	CEBPB	HP	ASGR2	HP_ASGR2
1893	STAT3	HP	ASGR1	HP_ASGR1
1894	STAT3	HP	ITGB2	HP_ITGB2
1895	STAT3	HP	ITGAM	HP_ITGAM
1896	STAT3	HP	ASGR2	HP_ASGR2
1897	SMAD4	HP	ASGR1	HP_ASGR1
1898	SMAD4	HP	ITGB2	HP_ITGB2
1899	SMAD4	HP	ITGAM	HP_ITGAM
1900	SMAD4	HP	ASGR2	HP_ASGR2
1901	BTG2	HRAS	CAV1	HRAS_CAV1
1902	BTG2	HRAS	TLR2	HRAS_TLR2
1903	BTG2	HRAS	INSR	HRAS_INSR
1904	TP53	HRAS	CAV1	HRAS_CAV1
1905	TP53	HRAS	TLR2	HRAS_TLR2
1906	TP53	HRAS	INSR	HRAS_INSR
1907	MAZ	HRAS	CAV1	HRAS_CAV1
1908	MAZ	HRAS	TLR2	HRAS_TLR2
1909	MAZ	HRAS	INSR	HRAS_INSR
1910	SP1	HRAS	CAV1	HRAS_CAV1
1911	SP1	HRAS	TLR2	HRAS_TLR2
1912	SP1	HRAS	INSR	HRAS_INSR
1913	POU4F1	HSP90AA1	FGFR3	HSP90AA1_FGFR3

1914	POU4F1	HSP90AA1	CFTR	HSP90AA1_CFTR
1915	POU4F1	HSP90AA1	EGFR	HSP90AA1_EGFR
1916	POU4F2	HSP90AA1	FGFR3	HSP90AA1_FGFR3
1917	POU4F2	HSP90AA1	CFTR	HSP90AA1_CFTR
1918	POU4F2	HSP90AA1	EGFR	HSP90AA1_EGFR
1919	STAT1	HSP90AA1	FGFR3	HSP90AA1_FGFR3
1920	STAT1	HSP90AA1	CFTR	HSP90AA1_CFTR
1921	STAT1	HSP90AA1	EGFR	HSP90AA1_EGFR
1922	FOXA1	HSPA1A	TLR4	HSPA1A_TLR4
1923	FOXA1	HSPA1A	GRIN2D	HSPA1A_GRIN2D
1924	XBP1	HSPA1A	TLR4	HSPA1A_TLR4
1925	XBP1	HSPA1A	GRIN2D	HSPA1A_GRIN2D
1926	HSF1	HSPA1A	TLR4	HSPA1A_TLR4
1927	HSF1	HSPA1A	GRIN2D	HSPA1A_GRIN2D
1928	RELA	ICAM1	CAV1	ICAM1_CAV1
1929	RELA	ICAM1	IL2RA	ICAM1_IL2RA
1930	RELA	ICAM1	ITGB2	ICAM1_ITGB2
1931	RELA	ICAM1	ITGAL	ICAM1_ITGAL
1932	RELA	ICAM1	EGFR	ICAM1_EGFR
1933	RELA	ICAM1	ITGAX	ICAM1_ITGAX
1934	RELA	ICAM1	ITGAM	ICAM1_ITGAM
1935	RELA	ICAM1	IL2RG	ICAM1_IL2RG
1936	ETV5	ICAM1	CAV1	ICAM1_CAV1
1937	ETV5	ICAM1	IL2RA	ICAM1_IL2RA
1938	ETV5	ICAM1	ITGB2	ICAM1_ITGB2
1939	ETV5	ICAM1	ITGAL	ICAM1_ITGAL
1940	ETV5	ICAM1	EGFR	ICAM1_EGFR
1941	ETV5	ICAM1	ITGAX	ICAM1_ITGAX
1942	ETV5	ICAM1	ITGAM	ICAM1_ITGAM
1943	ETV5	ICAM1	IL2RG	ICAM1_IL2RG
1944	RARA	ICAM1	CAV1	ICAM1_CAV1
1945	RARA	ICAM1	IL2RA	ICAM1_IL2RA
1946	RARA	ICAM1	ITGB2	ICAM1_ITGB2
1947	RARA	ICAM1	ITGAL	ICAM1_ITGAL
1948	RARA	ICAM1	EGFR	ICAM1_EGFR
1949	RARA	ICAM1	ITGAX	ICAM1_ITGAX
1950	RARA	ICAM1	ITGAM	ICAM1_ITGAM
1951	RARA	ICAM1	IL2RG	ICAM1_IL2RG
1952	SP1	ICAM1	CAV1	ICAM1_CAV1
1953	SP1	ICAM1	IL2RA	ICAM1_IL2RA
1954	SP1	ICAM1	ITGB2	ICAM1_ITGB2
1955	SP1	ICAM1	ITGAL	ICAM1_ITGAL
1956	SP1	ICAM1	EGFR	ICAM1_EGFR
1957	SP1	ICAM1	ITGAX	ICAM1_ITGAX
1958	SP1	ICAM1	ITGAM	ICAM1_ITGAM
1959	SP1	ICAM1	IL2RG	ICAM1_IL2RG
1960	TWIST1	ICAM1	CAV1	ICAM1_CAV1
1961	TWIST1	ICAM1	IL2RA	ICAM1_IL2RA
1962	TWIST1	ICAM1	ITGB2	ICAM1_ITGB2
1963	TWIST1	ICAM1	ITGAL	ICAM1_ITGAL
1964	TWIST1	ICAM1	EGFR	ICAM1_EGFR
1965	TWIST1	ICAM1	ITGAX	ICAM1_ITGAX
1966	TWIST1	ICAM1	ITGAM	ICAM1_ITGAM
1967	TWIST1	ICAM1	IL2RG	ICAM1_IL2RG
1968	HDAC1	ICAM1	CAV1	ICAM1_CAV1
1969	HDAC1	ICAM1	IL2RA	ICAM1_IL2RA
1970	HDAC1	ICAM1	ITGB2	ICAM1_ITGB2
1971	HDAC1	ICAM1	ITGAL	ICAM1_ITGAL

1972	HDAC1	ICAM1	EGFR	ICAM1_EGFR
1973	HDAC1	ICAM1	ITGAX	ICAM1_ITGAX
1974	HDAC1	ICAM1	ITGAM	ICAM1_ITGAM
1975	HDAC1	ICAM1	IL2RG	ICAM1_IL2RG
1976	STAT3	ICAM1	CAV1	ICAM1_CAV1
1977	STAT3	ICAM1	IL2RA	ICAM1_IL2RA
1978	STAT3	ICAM1	ITGB2	ICAM1_ITGB2
1979	STAT3	ICAM1	ITGAL	ICAM1_ITGAL
1980	STAT3	ICAM1	EGFR	ICAM1_EGFR
1981	STAT3	ICAM1	ITGAX	ICAM1_ITGAX
1982	STAT3	ICAM1	ITGAM	ICAM1_ITGAM
1983	STAT3	ICAM1	IL2RG	ICAM1_IL2RG
1984	ERG	ICAM1	CAV1	ICAM1_CAV1
1985	ERG	ICAM1	IL2RA	ICAM1_IL2RA
1986	ERG	ICAM1	ITGB2	ICAM1_ITGB2
1987	ERG	ICAM1	ITGAL	ICAM1_ITGAL
1988	ERG	ICAM1	EGFR	ICAM1_EGFR
1989	ERG	ICAM1	ITGAX	ICAM1_ITGAX
1990	ERG	ICAM1	ITGAM	ICAM1_ITGAM
1991	ERG	ICAM1	IL2RG	ICAM1_IL2RG
1992	MYB	ICAM1	CAV1	ICAM1_CAV1
1993	MYB	ICAM1	IL2RA	ICAM1_IL2RA
1994	MYB	ICAM1	ITGB2	ICAM1_ITGB2
1995	MYB	ICAM1	ITGAL	ICAM1_ITGAL
1996	MYB	ICAM1	EGFR	ICAM1_EGFR
1997	MYB	ICAM1	ITGAX	ICAM1_ITGAX
1998	MYB	ICAM1	ITGAM	ICAM1_ITGAM
1999	MYB	ICAM1	IL2RG	ICAM1_IL2RG
2000	ETS2	ICAM1	CAV1	ICAM1_CAV1
2001	ETS2	ICAM1	IL2RA	ICAM1_IL2RA
2002	ETS2	ICAM1	ITGB2	ICAM1_ITGB2
2003	ETS2	ICAM1	ITGAL	ICAM1_ITGAL
2004	ETS2	ICAM1	EGFR	ICAM1_EGFR
2005	ETS2	ICAM1	ITGAX	ICAM1_ITGAX
2006	ETS2	ICAM1	ITGAM	ICAM1_ITGAM
2007	ETS2	ICAM1	IL2RG	ICAM1_IL2RG
2008	PPARG	ICAM1	CAV1	ICAM1_CAV1
2009	PPARG	ICAM1	IL2RA	ICAM1_IL2RA
2010	PPARG	ICAM1	ITGB2	ICAM1_ITGB2
2011	PPARG	ICAM1	ITGAL	ICAM1_ITGAL
2012	PPARG	ICAM1	EGFR	ICAM1_EGFR
2013	PPARG	ICAM1	ITGAX	ICAM1_ITGAX
2014	PPARG	ICAM1	ITGAM	ICAM1_ITGAM
2015	PPARG	ICAM1	IL2RG	ICAM1_IL2RG
2016	NFKB1	ICAM1	CAV1	ICAM1_CAV1
2017	NFKB1	ICAM1	IL2RA	ICAM1_IL2RA
2018	NFKB1	ICAM1	ITGB2	ICAM1_ITGB2
2019	NFKB1	ICAM1	ITGAL	ICAM1_ITGAL
2020	NFKB1	ICAM1	EGFR	ICAM1_EGFR
2021	NFKB1	ICAM1	ITGAX	ICAM1_ITGAX
2022	NFKB1	ICAM1	ITGAM	ICAM1_ITGAM
2023	NFKB1	ICAM1	IL2RG	ICAM1_IL2RG
2024	CEBPA	ICAM1	CAV1	ICAM1_CAV1
2025	CEBPA	ICAM1	IL2RA	ICAM1_IL2RA
2026	CEBPA	ICAM1	ITGB2	ICAM1_ITGB2
2027	CEBPA	ICAM1	ITGAL	ICAM1_ITGAL
2028	CEBPA	ICAM1	EGFR	ICAM1_EGFR
2029	CEBPA	ICAM1	ITGAX	ICAM1_ITGAX

2030	CEBPA	ICAM1	ITGAM	ICAM1_ITGAM
2031	CEBPA	ICAM1	IL2RG	ICAM1_IL2RG
2032	NFKBIA	ICAM1	CAV1	ICAM1_CAV1
2033	NFKBIA	ICAM1	IL2RA	ICAM1_IL2RA
2034	NFKBIA	ICAM1	ITGB2	ICAM1_ITGB2
2035	NFKBIA	ICAM1	ITGAL	ICAM1_ITGAL
2036	NFKBIA	ICAM1	EGFR	ICAM1_EGFR
2037	NFKBIA	ICAM1	ITGAX	ICAM1_ITGAX
2038	NFKBIA	ICAM1	ITGAM	ICAM1_ITGAM
2039	NFKBIA	ICAM1	IL2RG	ICAM1_IL2RG
2040	IFI16	ICAM1	CAV1	ICAM1_CAV1
2041	IFI16	ICAM1	IL2RA	ICAM1_IL2RA
2042	IFI16	ICAM1	ITGB2	ICAM1_ITGB2
2043	IFI16	ICAM1	ITGAL	ICAM1_ITGAL
2044	IFI16	ICAM1	EGFR	ICAM1_EGFR
2045	IFI16	ICAM1	ITGAX	ICAM1_ITGAX
2046	IFI16	ICAM1	ITGAM	ICAM1_ITGAM
2047	IFI16	ICAM1	IL2RG	ICAM1_IL2RG
2048	REL	ICAM1	CAV1	ICAM1_CAV1
2049	REL	ICAM1	IL2RA	ICAM1_IL2RA
2050	REL	ICAM1	ITGB2	ICAM1_ITGB2
2051	REL	ICAM1	ITGAL	ICAM1_ITGAL
2052	REL	ICAM1	EGFR	ICAM1_EGFR
2053	REL	ICAM1	ITGAX	ICAM1_ITGAX
2054	REL	ICAM1	ITGAM	ICAM1_ITGAM
2055	REL	ICAM1	IL2RG	ICAM1_IL2RG
2056	STAT1	ICAM1	CAV1	ICAM1_CAV1
2057	STAT1	ICAM1	IL2RA	ICAM1_IL2RA
2058	STAT1	ICAM1	ITGB2	ICAM1_ITGB2
2059	STAT1	ICAM1	ITGAL	ICAM1_ITGAL
2060	STAT1	ICAM1	EGFR	ICAM1_EGFR
2061	STAT1	ICAM1	ITGAX	ICAM1_ITGAX
2062	STAT1	ICAM1	ITGAM	ICAM1_ITGAM
2063	STAT1	ICAM1	IL2RG	ICAM1_IL2RG
2064	TWIST2	ICAM1	CAV1	ICAM1_CAV1
2065	TWIST2	ICAM1	IL2RA	ICAM1_IL2RA
2066	TWIST2	ICAM1	ITGB2	ICAM1_ITGB2
2067	TWIST2	ICAM1	ITGAL	ICAM1_ITGAL
2068	TWIST2	ICAM1	EGFR	ICAM1_EGFR
2069	TWIST2	ICAM1	ITGAX	ICAM1_ITGAX
2070	TWIST2	ICAM1	ITGAM	ICAM1_ITGAM
2071	TWIST2	ICAM1	IL2RG	ICAM1_IL2RG
2072	RARG	ICAM1	CAV1	ICAM1_CAV1
2073	RARG	ICAM1	IL2RA	ICAM1_IL2RA
2074	RARG	ICAM1	ITGB2	ICAM1_ITGB2
2075	RARG	ICAM1	ITGAL	ICAM1_ITGAL
2076	RARG	ICAM1	EGFR	ICAM1_EGFR
2077	RARG	ICAM1	ITGAX	ICAM1_ITGAX
2078	RARG	ICAM1	ITGAM	ICAM1_ITGAM
2079	RARG	ICAM1	IL2RG	ICAM1_IL2RG
2080	SIRT1	ICAM1	CAV1	ICAM1_CAV1
2081	SIRT1	ICAM1	IL2RA	ICAM1_IL2RA
2082	SIRT1	ICAM1	ITGB2	ICAM1_ITGB2
2083	SIRT1	ICAM1	ITGAL	ICAM1_ITGAL
2084	SIRT1	ICAM1	EGFR	ICAM1_EGFR
2085	SIRT1	ICAM1	ITGAX	ICAM1_ITGAX
2086	SIRT1	ICAM1	ITGAM	ICAM1_ITGAM
2087	SIRT1	ICAM1	IL2RG	ICAM1_IL2RG

2088	NFATC1	ICAM1	CAV1	ICAM1_CAV1
2089	NFATC1	ICAM1	IL2RA	ICAM1_IL2RA
2090	NFATC1	ICAM1	ITGB2	ICAM1_ITGB2
2091	NFATC1	ICAM1	ITGAL	ICAM1_ITGAL
2092	NFATC1	ICAM1	EGFR	ICAM1_EGFR
2093	NFATC1	ICAM1	ITGAX	ICAM1_ITGAX
2094	NFATC1	ICAM1	ITGAM	ICAM1_ITGAM
2095	NFATC1	ICAM1	IL2RG	ICAM1_IL2RG
2096	ERG	ICAM2	ITGAL	ICAM2_ITGAL
2097	ERG	ICAM2	ITGB2	ICAM2_ITGB2
2098	ERG	ICAM2	ITGAM	ICAM2_ITGAM
2099	RUNX3	ICAM3	ITGAL	ICAM3_ITGAL
2100	RUNX3	ICAM3	ITGB2	ICAM3_ITGB2
2101	RUNX3	ICAM3	ITGAD	ICAM3_ITGAD
2102	RUNX3	ICAM3	CLEC4M	ICAM3_CLEC4M
2103	STAT1	IFNG	IFNGR2	IFNG_IFNGR2
2104	STAT1	IFNG	IFNGR1	IFNG_IFNGR1
2105	NFKB1	IFNG	IFNGR2	IFNG_IFNGR2
2106	NFKB1	IFNG	IFNGR1	IFNG_IFNGR1
2107	CREB1	IFNG	IFNGR2	IFNG_IFNGR2
2108	CREB1	IFNG	IFNGR1	IFNG_IFNGR1
2109	PARP1	IFNG	IFNGR2	IFNG_IFNGR2
2110	PARP1	IFNG	IFNGR1	IFNG_IFNGR1
2111	MYCN	IFNG	IFNGR2	IFNG_IFNGR2
2112	MYCN	IFNG	IFNGR1	IFNG_IFNGR1
2113	USF1	IFNG	IFNGR2	IFNG_IFNGR2
2114	USF1	IFNG	IFNGR1	IFNG_IFNGR1
2115	STAT3	IFNG	IFNGR2	IFNG_IFNGR2
2116	STAT3	IFNG	IFNGR1	IFNG_IFNGR1
2117	JUN	IFNG	IFNGR2	IFNG_IFNGR2
2118	JUN	IFNG	IFNGR1	IFNG_IFNGR1
2119	TBX21	IFNG	IFNGR2	IFNG_IFNGR2
2120	TBX21	IFNG	IFNGR1	IFNG_IFNGR1
2121	ATF3	IFNG	IFNGR2	IFNG_IFNGR2
2122	ATF3	IFNG	IFNGR1	IFNG_IFNGR1
2123	NFATC1	IFNG	IFNGR2	IFNG_IFNGR2
2124	NFATC1	IFNG	IFNGR1	IFNG_IFNGR1
2125	STAT4	IFNG	IFNGR2	IFNG_IFNGR2
2126	STAT4	IFNG	IFNGR1	IFNG_IFNGR1
2127	RELA	IFNG	IFNGR2	IFNG_IFNGR2
2128	RELA	IFNG	IFNGR1	IFNG_IFNGR1
2129	YY1	IFNG	IFNGR2	IFNG_IFNGR2
2130	YY1	IFNG	IFNGR1	IFNG_IFNGR1
2131	NFIL3	IFNG	IFNGR2	IFNG_IFNGR2
2132	NFIL3	IFNG	IFNGR1	IFNG_IFNGR1
2133	STAT5B	IFNG	IFNGR2	IFNG_IFNGR2
2134	STAT5B	IFNG	IFNGR1	IFNG_IFNGR1
2135	MSC	IFNG	IFNGR2	IFNG_IFNGR2
2136	MSC	IFNG	IFNGR1	IFNG_IFNGR1
2137	PROX1	IFNG	IFNGR2	IFNG_IFNGR2
2138	PROX1	IFNG	IFNGR1	IFNG_IFNGR1
2139	EGR1	IFNG	IFNGR2	IFNG_IFNGR2
2140	EGR1	IFNG	IFNGR1	IFNG_IFNGR1
2141	GATA1	IFNG	IFNGR2	IFNG_IFNGR2
2142	GATA1	IFNG	IFNGR1	IFNG_IFNGR1
2143	GATA3	IFNG	IFNGR2	IFNG_IFNGR2
2144	GATA3	IFNG	IFNGR1	IFNG_IFNGR1
2145	ATF2	IFNG	IFNGR2	IFNG_IFNGR2

2146	ATF2	IFNG	IFNGR1	IFNG_IFNGR1
2147	TFAP4	IFNG	IFNGR2	IFNG_IFNGR2
2148	TFAP4	IFNG	IFNGR1	IFNG_IFNGR1
2149	EOMES	IFNG	IFNGR2	IFNG_IFNGR2
2150	EOMES	IFNG	IFNGR1	IFNG_IFNGR1
2151	STAT5A	IFNG	IFNGR2	IFNG_IFNGR2
2152	STAT5A	IFNG	IFNGR1	IFNG_IFNGR1
2153	EP300	IFNG	IFNGR2	IFNG_IFNGR2
2154	EP300	IFNG	IFNGR1	IFNG_IFNGR1
2155	NFATC2	IFNG	IFNGR2	IFNG_IFNGR2
2156	NFATC2	IFNG	IFNGR1	IFNG_IFNGR1
2157	IRF1	IFNG	IFNGR2	IFNG_IFNGR2
2158	IRF1	IFNG	IFNGR1	IFNG_IFNGR1
2159	RFX5	IFNG	IFNGR2	IFNG_IFNGR2
2160	RFX5	IFNG	IFNGR1	IFNG_IFNGR1
2161	NCOR1	IGF1	INSR	IGF1_INSR
2162	RB1	IGF1	INSR	IGF1_INSR
2163	EP300	IGF1	INSR	IGF1_INSR
2164	HDAC2	IGF1	INSR	IGF1_INSR
2165	NCOR2	IGF1	INSR	IGF1_INSR
2166	BRCA1	IGF1	INSR	IGF1_INSR
2167	CEBPA	IGF1	INSR	IGF1_INSR
2168	WT1	IGF1	INSR	IGF1_INSR
2169	STAT5B	IGF1	INSR	IGF1_INSR
2170	OTX2	IGF1	INSR	IGF1_INSR
2171	MBD1	IGF2	INSR	IGF2_INSR
2172	SP1	IGF2	INSR	IGF2_INSR
2173	EGR1	IGF2	INSR	IGF2_INSR
2174	ASCL1	IGF2	INSR	IGF2_INSR
2175	PLAGL2	IGF2	INSR	IGF2_INSR
2176	KHDRBS1	IGF2	INSR	IGF2_INSR
2177	PLAG1	IGF2	INSR	IGF2_INSR
2178	WT1	IGF2	INSR	IGF2_INSR
2179	CTCF	IGF2	INSR	IGF2_INSR
2180	EGR2	IGF2	INSR	IGF2_INSR
2181	DDX5	IGF2	INSR	IGF2_INSR
2182	CEBPA	IGF2	INSR	IGF2_INSR
2183	SUZ12	IGF2	INSR	IGF2_INSR
2184	HDAC5	IGF2	INSR	IGF2_INSR
2185	SP1	IGFBP4	FZD8	IGFBP4_FZD8
2186	SP1	IGFBP4	LRP6	IGFBP4_LRP6
2187	TBP	IGFBP4	FZD8	IGFBP4_FZD8
2188	TBP	IGFBP4	LRP6	IGFBP4_LRP6
2189	SP3	IGFBP4	FZD8	IGFBP4_FZD8
2190	SP3	IGFBP4	LRP6	IGFBP4_LRP6
2191	RUNX2	IHH	PTCH2	IHH_PTCH2
2192	STAT1	IL10	IL10RA	IL10_IL10RA
2193	STAT1	IL10	SIRPG	IL10_SIRPG
2194	SP1	IL10	IL10RA	IL10_IL10RA
2195	SP1	IL10	SIRPG	IL10_SIRPG
2196	HDAC11	IL10	IL10RA	IL10_IL10RA
2197	HDAC11	IL10	SIRPG	IL10_SIRPG
2198	GATA3	IL10	IL10RA	IL10_IL10RA
2199	GATA3	IL10	SIRPG	IL10_SIRPG
2200	CREB1	IL10	IL10RA	IL10_IL10RA
2201	CREB1	IL10	SIRPG	IL10_SIRPG
2202	FLI1	IL10	IL10RA	IL10_IL10RA
2203	FLI1	IL10	SIRPG	IL10_SIRPG

2204	STAT3	IL10	IL10RA	IL10_IL10RA
2205	STAT3	IL10	SIRPG	IL10_SIRPG
2206	REL	IL10	IL10RA	IL10_IL10RA
2207	REL	IL10	SIRPG	IL10_SIRPG
2208	MSC	IL10	IL10RA	IL10_IL10RA
2209	MSC	IL10	SIRPG	IL10_SIRPG
2210	NR1I2	IL10	IL10RA	IL10_IL10RA
2211	NR1I2	IL10	SIRPG	IL10_SIRPG
2212	IRF8	IL10	IL10RA	IL10_IL10RA
2213	IRF8	IL10	SIRPG	IL10_SIRPG
2214	VDR	IL10	IL10RA	IL10_IL10RA
2215	VDR	IL10	SIRPG	IL10_SIRPG
2216	CEBPA	IL10	IL10RA	IL10_IL10RA
2217	CEBPA	IL10	SIRPG	IL10_SIRPG
2218	NFKB1	IL10	IL10RA	IL10_IL10RA
2219	NFKB1	IL10	SIRPG	IL10_SIRPG
2220	HDAC4	IL10	IL10RA	IL10_IL10RA
2221	HDAC4	IL10	SIRPG	IL10_SIRPG
2222	RELA	IL10	IL10RA	IL10_IL10RA
2223	RELA	IL10	SIRPG	IL10_SIRPG
2224	PGR	IL10	IL10RA	IL10_IL10RA
2225	PGR	IL10	SIRPG	IL10_SIRPG
2226	CEBPB	IL10	IL10RA	IL10_IL10RA
2227	CEBPB	IL10	SIRPG	IL10_SIRPG
2228	TBX21	IL10	IL10RA	IL10_IL10RA
2229	TBX21	IL10	SIRPG	IL10_SIRPG
2230	ATF1	IL10	IL10RA	IL10_IL10RA
2231	ATF1	IL10	SIRPG	IL10_SIRPG
2232	FOXP3	IL10	IL10RA	IL10_IL10RA
2233	FOXP3	IL10	SIRPG	IL10_SIRPG
2234	PARP1	IL10	IL10RA	IL10_IL10RA
2235	PARP1	IL10	SIRPG	IL10_SIRPG
2236	IRF1	IL10	IL10RA	IL10_IL10RA
2237	IRF1	IL10	SIRPG	IL10_SIRPG
2238	RELA	IL12A	CD28	IL12A_CD28
2239	RELA	IL12A	IL12RB2	IL12A_IL12RB2
2240	RELA	IL12A	IL12RB1	IL12A_IL12RB1
2241	IRF1	IL12A	CD28	IL12A_CD28
2242	IRF1	IL12A	IL12RB2	IL12A_IL12RB2
2243	IRF1	IL12A	IL12RB1	IL12A_IL12RB1
2244	IRF8	IL12A	CD28	IL12A_CD28
2245	IRF8	IL12A	IL12RB2	IL12A_IL12RB2
2246	IRF8	IL12A	IL12RB1	IL12A_IL12RB1
2247	NFKB1	IL12A	CD28	IL12A_CD28
2248	NFKB1	IL12A	IL12RB2	IL12A_IL12RB2
2249	NFKB1	IL12A	IL12RB1	IL12A_IL12RB1
2250	JUN	IL12A	CD28	IL12A_CD28
2251	JUN	IL12A	IL12RB2	IL12A_IL12RB2
2252	JUN	IL12A	IL12RB1	IL12A_IL12RB1
2253	E2F6	IL13	IL13RA1	IL13_IL13RA1
2254	E2F6	IL13	IL2RG	IL13_IL2RG
2255	CEBPB	IL13	IL13RA1	IL13_IL13RA1
2256	CEBPB	IL13	IL2RG	IL13_IL2RG
2257	TBX21	IL13	IL13RA1	IL13_IL13RA1
2258	TBX21	IL13	IL2RG	IL13_IL2RG
2259	STAT6	IL13	IL13RA1	IL13_IL13RA1
2260	STAT6	IL13	IL2RG	IL13_IL2RG
2261	AHR	IL13	IL13RA1	IL13_IL13RA1

2262	AHR	IL13	IL2RG	IL13_IL2RG
2263	HOXA10	IL15	IL2RA	IL15_IL2RA
2264	HOXA10	IL15	IL2RG	IL15_IL2RG
2265	HOXA10	IL15	IL2RB	IL15_IL2RB
2266	MYB	IL15	IL2RA	IL15_IL2RA
2267	MYB	IL15	IL2RG	IL15_IL2RG
2268	MYB	IL15	IL2RB	IL15_IL2RB
2269	NFKB1	IL18	CD48	IL18_CD48
2270	NFKB1	IL18	IL1RL2	IL18_IL1RL2
2271	NFKB1	IL18	IL1RAPL1	IL18_IL1RAPL1
2272	NFKB1	IL18	IL18R1	IL18_IL18R1
2273	NFKB1	IL18	IL18RAP	IL18_IL18RAP
2274	RELA	IL18	CD48	IL18_CD48
2275	RELA	IL18	IL1RL2	IL18_IL1RL2
2276	RELA	IL18	IL1RAPL1	IL18_IL1RAPL1
2277	RELA	IL18	IL18R1	IL18_IL18R1
2278	RELA	IL18	IL18RAP	IL18_IL18RAP
2279	HDAC9	IL18	CD48	IL18_CD48
2280	HDAC9	IL18	IL1RL2	IL18_IL1RL2
2281	HDAC9	IL18	IL1RAPL1	IL18_IL1RAPL1
2282	HDAC9	IL18	IL18R1	IL18_IL18R1
2283	HDAC9	IL18	IL18RAP	IL18_IL18RAP
2284	SPI1	IL18	CD48	IL18_CD48
2285	SPI1	IL18	IL1RL2	IL18_IL1RL2
2286	SPI1	IL18	IL1RAPL1	IL18_IL1RAPL1
2287	SPI1	IL18	IL18R1	IL18_IL18R1
2288	SPI1	IL18	IL18RAP	IL18_IL18RAP
2289	FOS	IL1A	IL1R2	IL1A_IL1R2
2290	RELA	IL1A	IL1R2	IL1A_IL1R2
2291	JUN	IL1A	IL1R2	IL1A_IL1R2
2292	NFKB1	IL1A	IL1R2	IL1A_IL1R2
2293	SPI1	IL1B	IL1R1	IL1B_IL1R1
2294	SPI1	IL1B	ADRB2	IL1B_ADRB2
2295	SPI1	IL1B	IL1RAP	IL1B_IL1RAP
2296	SPI1	IL1B	IL1R2	IL1B_IL1R2
2297	CEBPB	IL1B	IL1R1	IL1B_IL1R1
2298	CEBPB	IL1B	ADRB2	IL1B_ADRB2
2299	CEBPB	IL1B	IL1RAP	IL1B_IL1RAP
2300	CEBPB	IL1B	IL1R2	IL1B_IL1R2
2301	HMGA1	IL1B	IL1R1	IL1B_IL1R1
2302	HMGA1	IL1B	ADRB2	IL1B_ADRB2
2303	HMGA1	IL1B	IL1RAP	IL1B_IL1RAP
2304	HMGA1	IL1B	IL1R2	IL1B_IL1R2
2305	REL	IL1B	IL1R1	IL1B_IL1R1
2306	REL	IL1B	ADRB2	IL1B_ADRB2
2307	REL	IL1B	IL1RAP	IL1B_IL1RAP
2308	REL	IL1B	IL1R2	IL1B_IL1R2
2309	SUGP1	IL1B	IL1R1	IL1B_IL1R1
2310	SUGP1	IL1B	ADRB2	IL1B_ADRB2
2311	SUGP1	IL1B	IL1RAP	IL1B_IL1RAP
2312	SUGP1	IL1B	IL1R2	IL1B_IL1R2
2313	NFKB1	IL1B	IL1R1	IL1B_IL1R1
2314	NFKB1	IL1B	ADRB2	IL1B_ADRB2
2315	NFKB1	IL1B	IL1RAP	IL1B_IL1RAP
2316	NFKB1	IL1B	IL1R2	IL1B_IL1R2
2317	NFKBIA	IL1B	IL1R1	IL1B_IL1R1
2318	NFKBIA	IL1B	ADRB2	IL1B_ADRB2
2319	NFKBIA	IL1B	IL1RAP	IL1B_IL1RAP

2320	NFKBIA	IL1B	IL1R2	IL1B_IL1R2
2321	SIRT1	IL1B	IL1R1	IL1B_IL1R1
2322	SIRT1	IL1B	ADRB2	IL1B_ADRB2
2323	SIRT1	IL1B	IL1RAP	IL1B_IL1RAP
2324	SIRT1	IL1B	IL1R2	IL1B_IL1R2
2325	RELA	IL1B	IL1R1	IL1B_IL1R1
2326	RELA	IL1B	ADRB2	IL1B_ADRB2
2327	RELA	IL1B	IL1RAP	IL1B_IL1RAP
2328	RELA	IL1B	IL1R2	IL1B_IL1R2
2329	KLF4	IL1B	IL1R1	IL1B_IL1R1
2330	KLF4	IL1B	ADRB2	IL1B_ADRB2
2331	KLF4	IL1B	IL1RAP	IL1B_IL1RAP
2332	KLF4	IL1B	IL1R2	IL1B_IL1R2
2333	YY1	IL1B	IL1R1	IL1B_IL1R1
2334	YY1	IL1B	ADRB2	IL1B_ADRB2
2335	YY1	IL1B	IL1RAP	IL1B_IL1RAP
2336	YY1	IL1B	IL1R2	IL1B_IL1R2
2337	JUN	IL1B	IL1R1	IL1B_IL1R1
2338	JUN	IL1B	ADRB2	IL1B_ADRB2
2339	JUN	IL1B	IL1RAP	IL1B_IL1RAP
2340	JUN	IL1B	IL1R2	IL1B_IL1R2
2341	JUNB	IL1B	IL1R1	IL1B_IL1R1
2342	JUNB	IL1B	ADRB2	IL1B_ADRB2
2343	JUNB	IL1B	IL1RAP	IL1B_IL1RAP
2344	JUNB	IL1B	IL1R2	IL1B_IL1R2
2345	E2F1	IL1B	IL1R1	IL1B_IL1R1
2346	E2F1	IL1B	ADRB2	IL1B_ADRB2
2347	E2F1	IL1B	IL1RAP	IL1B_IL1RAP
2348	E2F1	IL1B	IL1R2	IL1B_IL1R2
2349	NFIL3	IL1B	IL1R1	IL1B_IL1R1
2350	NFIL3	IL1B	ADRB2	IL1B_ADRB2
2351	NFIL3	IL1B	IL1RAP	IL1B_IL1RAP
2352	NFIL3	IL1B	IL1R2	IL1B_IL1R2
2353	HSF1	IL1B	IL1R1	IL1B_IL1R1
2354	HSF1	IL1B	ADRB2	IL1B_ADRB2
2355	HSF1	IL1B	IL1RAP	IL1B_IL1RAP
2356	HSF1	IL1B	IL1R2	IL1B_IL1R2
2357	STAT1	IL1B	IL1R1	IL1B_IL1R1
2358	STAT1	IL1B	ADRB2	IL1B_ADRB2
2359	STAT1	IL1B	IL1RAP	IL1B_IL1RAP
2360	STAT1	IL1B	IL1R2	IL1B_IL1R2
2361	IRF8	IL1B	IL1R1	IL1B_IL1R1
2362	IRF8	IL1B	ADRB2	IL1B_ADRB2
2363	IRF8	IL1B	IL1RAP	IL1B_IL1RAP
2364	IRF8	IL1B	IL1R2	IL1B_IL1R2
2365	AHR	IL1B	IL1R1	IL1B_IL1R1
2366	AHR	IL1B	ADRB2	IL1B_ADRB2
2367	AHR	IL1B	IL1RAP	IL1B_IL1RAP
2368	AHR	IL1B	IL1R2	IL1B_IL1R2
2369	RELA	IL1RN	IL1R2	IL1RN_IL1R2
2370	STAT3	IL1RN	IL1R2	IL1RN_IL1R2
2371	NFKB1	IL1RN	IL1R2	IL1RN_IL1R2
2372	NFKB1	IL2	IL2RG	IL2_IL2RG
2373	NFKB1	IL2	IL2RB	IL2_IL2RB
2374	NFKB1	IL2	IL2RA	IL2_IL2RA
2375	NFKB1	IL2	CD53	IL2_CD53
2376	HDAC1	IL2	IL2RG	IL2_IL2RG
2377	HDAC1	IL2	IL2RB	IL2_IL2RB

2378	HDAC1	IL2	IL2RA	IL2_IL2RA
2379	HDAC1	IL2	CD53	IL2_CD53
2380	CREM	IL2	IL2RG	IL2_IL2RG
2381	CREM	IL2	IL2RB	IL2_IL2RB
2382	CREM	IL2	IL2RA	IL2_IL2RA
2383	CREM	IL2	CD53	IL2_CD53
2384	POU2F1	IL2	IL2RG	IL2_IL2RG
2385	POU2F1	IL2	IL2RB	IL2_IL2RB
2386	POU2F1	IL2	IL2RA	IL2_IL2RA
2387	POU2F1	IL2	CD53	IL2_CD53
2388	ILF3	IL2	IL2RG	IL2_IL2RG
2389	ILF3	IL2	IL2RB	IL2_IL2RB
2390	ILF3	IL2	IL2RA	IL2_IL2RA
2391	ILF3	IL2	CD53	IL2_CD53
2392	VDR	IL2	IL2RG	IL2_IL2RG
2393	VDR	IL2	IL2RB	IL2_IL2RB
2394	VDR	IL2	IL2RA	IL2_IL2RA
2395	VDR	IL2	CD53	IL2_CD53
2396	RELA	IL2	IL2RG	IL2_IL2RG
2397	RELA	IL2	IL2RB	IL2_IL2RB
2398	RELA	IL2	IL2RA	IL2_IL2RA
2399	RELA	IL2	CD53	IL2_CD53
2400	JUN	IL2	IL2RG	IL2_IL2RG
2401	JUN	IL2	IL2RB	IL2_IL2RB
2402	JUN	IL2	IL2RA	IL2_IL2RA
2403	JUN	IL2	CD53	IL2_CD53
2404	ETS1	IL2	IL2RG	IL2_IL2RG
2405	ETS1	IL2	IL2RB	IL2_IL2RB
2406	ETS1	IL2	IL2RA	IL2_IL2RA
2407	ETS1	IL2	CD53	IL2_CD53
2408	STAT3	IL2	IL2RG	IL2_IL2RG
2409	STAT3	IL2	IL2RB	IL2_IL2RB
2410	STAT3	IL2	IL2RA	IL2_IL2RA
2411	STAT3	IL2	CD53	IL2_CD53
2412	FOXK2	IL2	IL2RG	IL2_IL2RG
2413	FOXK2	IL2	IL2RB	IL2_IL2RB
2414	FOXK2	IL2	IL2RA	IL2_IL2RA
2415	FOXK2	IL2	CD53	IL2_CD53
2416	CREB1	IL2	IL2RG	IL2_IL2RG
2417	CREB1	IL2	IL2RB	IL2_IL2RB
2418	CREB1	IL2	IL2RA	IL2_IL2RA
2419	CREB1	IL2	CD53	IL2_CD53
2420	TOB1	IL2	IL2RG	IL2_IL2RG
2421	TOB1	IL2	IL2RB	IL2_IL2RB
2422	TOB1	IL2	IL2RA	IL2_IL2RA
2423	TOB1	IL2	CD53	IL2_CD53
2424	SATB1	IL2	IL2RG	IL2_IL2RG
2425	SATB1	IL2	IL2RB	IL2_IL2RB
2426	SATB1	IL2	IL2RA	IL2_IL2RA
2427	SATB1	IL2	CD53	IL2_CD53
2428	ILF2	IL2	IL2RG	IL2_IL2RG
2429	ILF2	IL2	IL2RB	IL2_IL2RB
2430	ILF2	IL2	IL2RA	IL2_IL2RA
2431	ILF2	IL2	CD53	IL2_CD53
2432	REL	IL2	IL2RG	IL2_IL2RG
2433	REL	IL2	IL2RB	IL2_IL2RB
2434	REL	IL2	IL2RA	IL2_IL2RA
2435	REL	IL2	CD53	IL2_CD53

2436	SP1	IL2	IL2RG	IL2_IL2RG
2437	SP1	IL2	IL2RB	IL2_IL2RB
2438	SP1	IL2	IL2RA	IL2_IL2RA
2439	SP1	IL2	CD53	IL2_CD53
2440	BACH2	IL2	IL2RG	IL2_IL2RG
2441	BACH2	IL2	IL2RB	IL2_IL2RB
2442	BACH2	IL2	IL2RA	IL2_IL2RA
2443	BACH2	IL2	CD53	IL2_CD53
2444	KLF2	IL2	IL2RG	IL2_IL2RG
2445	KLF2	IL2	IL2RB	IL2_IL2RB
2446	KLF2	IL2	IL2RA	IL2_IL2RA
2447	KLF2	IL2	CD53	IL2_CD53
2448	EGR1	IL2	IL2RG	IL2_IL2RG
2449	EGR1	IL2	IL2RB	IL2_IL2RB
2450	EGR1	IL2	IL2RA	IL2_IL2RA
2451	EGR1	IL2	CD53	IL2_CD53
2452	NFATC1	IL2	IL2RG	IL2_IL2RG
2453	NFATC1	IL2	IL2RB	IL2_IL2RB
2454	NFATC1	IL2	IL2RA	IL2_IL2RA
2455	NFATC1	IL2	CD53	IL2_CD53
2456	FOXP3	IL2	IL2RG	IL2_IL2RG
2457	FOXP3	IL2	IL2RB	IL2_IL2RB
2458	FOXP3	IL2	IL2RA	IL2_IL2RA
2459	FOXP3	IL2	CD53	IL2_CD53
2460	KAT5	IL2	IL2RG	IL2_IL2RG
2461	KAT5	IL2	IL2RB	IL2_IL2RB
2462	KAT5	IL2	IL2RA	IL2_IL2RA
2463	KAT5	IL2	CD53	IL2_CD53
2464	RUNX1	IL2	IL2RG	IL2_IL2RG
2465	RUNX1	IL2	IL2RB	IL2_IL2RB
2466	RUNX1	IL2	IL2RA	IL2_IL2RA
2467	RUNX1	IL2	CD53	IL2_CD53
2468	ZFP36	IL2	IL2RG	IL2_IL2RG
2469	ZFP36	IL2	IL2RB	IL2_IL2RB
2470	ZFP36	IL2	IL2RA	IL2_IL2RA
2471	ZFP36	IL2	CD53	IL2_CD53
2472	NFKB1	IL21	IL2RG	IL21_IL2RG
2473	STAT3	IL21	IL2RG	IL21_IL2RG
2474	RORC	IL22	IL10RA	IL22_IL10RA
2475	CEBPB	IL4	CD53	IL4_CD53
2476	CEBPB	IL4	IL13RA1	IL4_IL13RA1
2477	CEBPB	IL4	IL2RG	IL4_IL2RG
2478	POU2F1	IL4	CD53	IL4_CD53
2479	POU2F1	IL4	IL13RA1	IL4_IL13RA1
2480	POU2F1	IL4	IL2RG	IL4_IL2RG
2481	JUNB	IL4	CD53	IL4_CD53
2482	JUNB	IL4	IL13RA1	IL4_IL13RA1
2483	JUNB	IL4	IL2RG	IL4_IL2RG
2484	CREBBP	IL4	CD53	IL4_CD53
2485	CREBBP	IL4	IL13RA1	IL4_IL13RA1
2486	CREBBP	IL4	IL2RG	IL4_IL2RG
2487	STAT6	IL4	CD53	IL4_CD53
2488	STAT6	IL4	IL13RA1	IL4_IL13RA1
2489	STAT6	IL4	IL2RG	IL4_IL2RG
2490	YY1	IL4	CD53	IL4_CD53
2491	YY1	IL4	IL13RA1	IL4_IL13RA1
2492	YY1	IL4	IL2RG	IL4_IL2RG
2493	IRF2	IL4	CD53	IL4_CD53

2494	IRF2	IL4	IL13RA1	IL4_IL13RA1
2495	IRF2	IL4	IL2RG	IL4_IL2RG
2496	TBX21	IL4	CD53	IL4_CD53
2497	TBX21	IL4	IL13RA1	IL4_IL13RA1
2498	TBX21	IL4	IL2RG	IL4_IL2RG
2499	CIITA	IL4	CD53	IL4_CD53
2500	CIITA	IL4	IL13RA1	IL4_IL13RA1
2501	CIITA	IL4	IL2RG	IL4_IL2RG
2502	NFKB1	IL4	CD53	IL4_CD53
2503	NFKB1	IL4	IL13RA1	IL4_IL13RA1
2504	NFKB1	IL4	IL2RG	IL4_IL2RG
2505	SATB1	IL4	CD53	IL4_CD53
2506	SATB1	IL4	IL13RA1	IL4_IL13RA1
2507	SATB1	IL4	IL2RG	IL4_IL2RG
2508	SPIC	IL4	CD53	IL4_CD53
2509	SPIC	IL4	IL13RA1	IL4_IL13RA1
2510	SPIC	IL4	IL2RG	IL4_IL2RG
2511	MAF	IL4	CD53	IL4_CD53
2512	MAF	IL4	IL13RA1	IL4_IL13RA1
2513	MAF	IL4	IL2RG	IL4_IL2RG
2514	RELA	IL4	CD53	IL4_CD53
2515	RELA	IL4	IL13RA1	IL4_IL13RA1
2516	RELA	IL4	IL2RG	IL4_IL2RG
2517	GATA3	IL4	CD53	IL4_CD53
2518	GATA3	IL4	IL13RA1	IL4_IL13RA1
2519	GATA3	IL4	IL2RG	IL4_IL2RG
2520	EP300	IL5	IL5RA	IL5_IL5RA
2521	MAF	IL5	IL5RA	IL5_IL5RA
2522	JUND	IL5	IL5RA	IL5_IL5RA
2523	YY1	IL5	IL5RA	IL5_IL5RA
2524	GATA4	IL5	IL5RA	IL5_IL5RA
2525	FOSL2	IL5	IL5RA	IL5_IL5RA
2526	POU2F2	IL5	IL5RA	IL5_IL5RA
2527	SPI1	IL5	IL5RA	IL5_IL5RA
2528	ETS2	IL5	IL5RA	IL5_IL5RA
2529	GATA3	IL5	IL5RA	IL5_IL5RA
2530	ETS1	IL5	IL5RA	IL5_IL5RA
2531	CEBPA	IL5	IL5RA	IL5_IL5RA
2532	HDAC4	IL5	IL5RA	IL5_IL5RA
2533	POU2F1	IL5	IL5RA	IL5_IL5RA
2534	SATB1	IL5	IL5RA	IL5_IL5RA
2535	CEBPB	IL5	IL5RA	IL5_IL5RA
2536	XBP1	IL6	F3	IL6_F3
2537	XBP1	IL6	IL6R	IL6_IL6R
2538	XBP1	IL6	IL6ST	IL6_IL6ST
2539	GBX2	IL6	F3	IL6_F3
2540	GBX2	IL6	IL6R	IL6_IL6R
2541	GBX2	IL6	IL6ST	IL6_IL6ST
2542	CEBPB	IL6	F3	IL6_F3
2543	CEBPB	IL6	IL6R	IL6_IL6R
2544	CEBPB	IL6	IL6ST	IL6_IL6ST
2545	NFKB1	IL6	F3	IL6_F3
2546	NFKB1	IL6	IL6R	IL6_IL6R
2547	NFKB1	IL6	IL6ST	IL6_IL6ST
2548	MYC	IL6	F3	IL6_F3
2549	MYC	IL6	IL6R	IL6_IL6R
2550	MYC	IL6	IL6ST	IL6_IL6ST
2551	STAT1	IL6	F3	IL6_F3

2552	STAT1	IL6	IL6R	IL6_IL6R
2553	STAT1	IL6	IL6ST	IL6_IL6ST
2554	STAT3	IL6	F3	IL6_F3
2555	STAT3	IL6	IL6R	IL6_IL6R
2556	STAT3	IL6	IL6ST	IL6_IL6ST
2557	DDIT3	IL6	F3	IL6_F3
2558	DDIT3	IL6	IL6R	IL6_IL6R
2559	DDIT3	IL6	IL6ST	IL6_IL6ST
2560	ATF4	IL6	F3	IL6_F3
2561	ATF4	IL6	IL6R	IL6_IL6R
2562	ATF4	IL6	IL6ST	IL6_IL6ST
2563	AHR	IL6	F3	IL6_F3
2564	AHR	IL6	IL6R	IL6_IL6R
2565	AHR	IL6	IL6ST	IL6_IL6ST
2566	ZFP36	IL6	F3	IL6_F3
2567	ZFP36	IL6	IL6R	IL6_IL6R
2568	ZFP36	IL6	IL6ST	IL6_IL6ST
2569	FOXO1	IL6	F3	IL6_F3
2570	FOXO1	IL6	IL6R	IL6_IL6R
2571	FOXO1	IL6	IL6ST	IL6_IL6ST
2572	TBP	IL6	F3	IL6_F3
2573	TBP	IL6	IL6R	IL6_IL6R
2574	TBP	IL6	IL6ST	IL6_IL6ST
2575	REL	IL6	F3	IL6_F3
2576	REL	IL6	IL6R	IL6_IL6R
2577	REL	IL6	IL6ST	IL6_IL6ST
2578	RELA	IL6	F3	IL6_F3
2579	RELA	IL6	IL6R	IL6_IL6R
2580	RELA	IL6	IL6ST	IL6_IL6ST
2581	JUND	IL6	F3	IL6_F3
2582	JUND	IL6	IL6R	IL6_IL6R
2583	JUND	IL6	IL6ST	IL6_IL6ST
2584	EGR1	IL6	F3	IL6_F3
2585	EGR1	IL6	IL6R	IL6_IL6R
2586	EGR1	IL6	IL6ST	IL6_IL6ST
2587	JUN	IL6	F3	IL6_F3
2588	JUN	IL6	IL6R	IL6_IL6R
2589	JUN	IL6	IL6ST	IL6_IL6ST
2590	RBPJ	IL6	F3	IL6_F3
2591	RBPJ	IL6	IL6R	IL6_IL6R
2592	RBPJ	IL6	IL6ST	IL6_IL6ST
2593	EGR2	IL6	F3	IL6_F3
2594	EGR2	IL6	IL6R	IL6_IL6R
2595	EGR2	IL6	IL6ST	IL6_IL6ST
2596	CEBPA	IL6	F3	IL6_F3
2597	CEBPA	IL6	IL6R	IL6_IL6R
2598	CEBPA	IL6	IL6ST	IL6_IL6ST
2599	KLF4	IL6	F3	IL6_F3
2600	KLF4	IL6	IL6R	IL6_IL6R
2601	KLF4	IL6	IL6ST	IL6_IL6ST
2602	SP1	IL6	F3	IL6_F3
2603	SP1	IL6	IL6R	IL6_IL6R
2604	SP1	IL6	IL6ST	IL6_IL6ST
2605	VDR	IL6	F3	IL6_F3
2606	VDR	IL6	IL6R	IL6_IL6R
2607	VDR	IL6	IL6ST	IL6_IL6ST
2608	OTX2	IL6	F3	IL6_F3
2609	OTX2	IL6	IL6R	IL6_IL6R

2610	OTX2	IL6	IL6ST	IL6_IL6ST
2611	CREB1	IL6	F3	IL6_F3
2612	CREB1	IL6	IL6R	IL6_IL6R
2613	CREB1	IL6	IL6ST	IL6_IL6ST
2614	EP300	IL6	F3	IL6_F3
2615	EP300	IL6	IL6R	IL6_IL6R
2616	EP300	IL6	IL6ST	IL6_IL6ST
2617	PPARA	IL6	F3	IL6_F3
2618	PPARA	IL6	IL6R	IL6_IL6R
2619	PPARA	IL6	IL6ST	IL6_IL6ST
2620	ZNF300	IL6	F3	IL6_F3
2621	ZNF300	IL6	IL6R	IL6_IL6R
2622	ZNF300	IL6	IL6ST	IL6_IL6ST
2623	ZMYND11	IL6	F3	IL6_F3
2624	ZMYND11	IL6	IL6R	IL6_IL6R
2625	ZMYND11	IL6	IL6ST	IL6_IL6ST
2626	MYB	IL7	IL7R	IL7_IL7R
2627	MYB	IL7	IL2RG	IL7_IL2RG
2628	NEUROD1	INS	INSR	INS_INSR
2629	PDX1	INS	INSR	INS_INSR
2630	PAX4	INS	INSR	INS_INSR
2631	ATF2	INS	INSR	INS_INSR
2632	GLIS3	INS	INSR	INS_INSR
2633	KLF11	INS	INSR	INS_INSR
2634	NR1H4	INS	INSR	INS_INSR
2635	TCF3	INS	INSR	INS_INSR
2636	MAFA	INS	INSR	INS_INSR
2637	SP1	KISS1	KISS1R	KISS1_KISS1R
2638	TFAP2A	KISS1	KISS1R	KISS1_KISS1R
2639	AR	KISS1	KISS1R	KISS1_KISS1R
2640	MED23	KISS1	KISS1R	KISS1_KISS1R
2641	PAX2	L1CAM	EGFR	L1CAM_EGFR
2642	PAX2	L1CAM	ERBB3	L1CAM_ERBB3
2643	TFAP2A	L1CAM	EGFR	L1CAM_EGFR
2644	TFAP2A	L1CAM	ERBB3	L1CAM_ERBB3
2645	KLF6	LAMA1	SDC4	LAMA1_SDC4
2646	KLF6	LAMA1	RPSA	LAMA1_RPSA
2647	KLF6	LAMA1	ITGB1	LAMA1_ITGB1
2648	KLF6	LAMA1	NT5E	LAMA1_NT5E
2649	KLF6	LAMA1	ITGB8	LAMA1_ITGB8
2650	KLF6	LAMA1	ITGA3	LAMA1_ITGA3
2651	SP3	LAMA1	SDC4	LAMA1_SDC4
2652	SP3	LAMA1	RPSA	LAMA1_RPSA
2653	SP3	LAMA1	ITGB1	LAMA1_ITGB1
2654	SP3	LAMA1	NT5E	LAMA1_NT5E
2655	SP3	LAMA1	ITGB8	LAMA1_ITGB8
2656	SP3	LAMA1	ITGA3	LAMA1_ITGA3
2657	SP1	LAMA1	SDC4	LAMA1_SDC4
2658	SP1	LAMA1	RPSA	LAMA1_RPSA
2659	SP1	LAMA1	ITGB1	LAMA1_ITGB1
2660	SP1	LAMA1	NT5E	LAMA1_NT5E
2661	SP1	LAMA1	ITGB8	LAMA1_ITGB8
2662	SP1	LAMA1	ITGA3	LAMA1_ITGA3
2663	KLF4	LAMA1	SDC4	LAMA1_SDC4
2664	KLF4	LAMA1	RPSA	LAMA1_RPSA
2665	KLF4	LAMA1	ITGB1	LAMA1_ITGB1
2666	KLF4	LAMA1	NT5E	LAMA1_NT5E
2667	KLF4	LAMA1	ITGB8	LAMA1_ITGB8

2668	KLF4	LAMA1	ITGA3	LAMA1_ITGA3
2669	SSB	LAMB1	ITGA3	LAMB1_ITGA3
2670	SSB	LAMB1	ITGB1	LAMB1_ITGB1
2671	NANOG	LAMB1	ITGA3	LAMB1_ITGA3
2672	NANOG	LAMB1	ITGB1	LAMB1_ITGB1
2673	ZEB1	LAMC2	ITGB1	LAMC2_ITGB1
2674	ZEB1	LAMC2	CD151	LAMC2_CD151
2675	ZEB1	LAMC2	ITGA3	LAMC2_ITGA3
2676	CREB5	LGALS3BP	ITGB1	LGALS3BP_ITGB1
2677	CREB5	LGALS3BP	VANGL1	LGALS3BP_VANGL1
2678	STAT3	LGALS3BP	ITGB1	LGALS3BP_ITGB1
2679	STAT3	LGALS3BP	VANGL1	LGALS3BP_VANGL1
2680	HNF4A	LIPC	LRP1	LIPC_LRP1
2681	SREBF2	LIPC	LRP1	LIPC_LRP1
2682	USF2	LIPC	LRP1	LIPC_LRP1
2683	USF1	LIPC	LRP1	LIPC_LRP1
2684	PPARA	LPL	LRP1	LPL_LRP1
2685	PPARA	LPL	CD44	LPL_CD44
2686	PPARA	LPL	GPIHBP1	LPL_GPIHBP1
2687	PPARA	LPL	VLDLR	LPL_VLDLR
2688	PPARA	LPL	LRP2	LPL_LRP2
2689	PPARA	LPL	SDC1	LPL_SDC1
2690	NFKB1	LPL	LRP1	LPL_LRP1
2691	NFKB1	LPL	CD44	LPL_CD44
2692	NFKB1	LPL	GPIHBP1	LPL_GPIHBP1
2693	NFKB1	LPL	VLDLR	LPL_VLDLR
2694	NFKB1	LPL	LRP2	LPL_LRP2
2695	NFKB1	LPL	SDC1	LPL_SDC1
2696	SP3	LPL	LRP1	LPL_LRP1
2697	SP3	LPL	CD44	LPL_CD44
2698	SP3	LPL	GPIHBP1	LPL_GPIHBP1
2699	SP3	LPL	VLDLR	LPL_VLDLR
2700	SP3	LPL	LRP2	LPL_LRP2
2701	SP3	LPL	SDC1	LPL_SDC1
2702	RELA	LPL	LRP1	LPL_LRP1
2703	RELA	LPL	CD44	LPL_CD44
2704	RELA	LPL	GPIHBP1	LPL_GPIHBP1
2705	RELA	LPL	VLDLR	LPL_VLDLR
2706	RELA	LPL	LRP2	LPL_LRP2
2707	RELA	LPL	SDC1	LPL_SDC1
2708	PPARD	LPL	LRP1	LPL_LRP1
2709	PPARD	LPL	CD44	LPL_CD44
2710	PPARD	LPL	GPIHBP1	LPL_GPIHBP1
2711	PPARD	LPL	VLDLR	LPL_VLDLR
2712	PPARD	LPL	LRP2	LPL_LRP2
2713	PPARD	LPL	SDC1	LPL_SDC1
2714	SP1	LPL	LRP1	LPL_LRP1
2715	SP1	LPL	CD44	LPL_CD44
2716	SP1	LPL	GPIHBP1	LPL_GPIHBP1
2717	SP1	LPL	VLDLR	LPL_VLDLR
2718	SP1	LPL	LRP2	LPL_LRP2
2719	SP1	LPL	SDC1	LPL_SDC1
2720	MSC	LTA	LTBR	LTA_LTBR
2721	MSC	LTA	TNFRSF1B	LTA_TNFRSF1B
2722	NFAT5	LTB	LTBR	LTB_LTBR
2723	NFAT5	LTB	CD40	LTB_CD40
2724	NFAT5	LTB	TNFRSF1A	LTB_TNFRSF1A
2725	EGR1	LTB	LTBR	LTB_LTBR

2726	EGR1	LTB	CD40	LTB_CD40
2727	EGR1	LTB	TNFRSF1A	LTB_TNFRSF1A
2728	CEBPE	LTF	LRP1	LTF_LRP1
2729	SP1	LTF	LRP1	LTF_LRP1
2730	SP1	LYZ	ITGAL	LYZ_ITGAL
2731	RELA	MADCAM1	CD44	MADCAM1_CD44
2732	RELA	MADCAM1	ITGA4	MADCAM1_ITGA4
2733	BTF3	MADCAM1	CD44	MADCAM1_CD44
2734	BTF3	MADCAM1	ITGA4	MADCAM1_ITGA4
2735	NFKB1	MADCAM1	CD44	MADCAM1_CD44
2736	NFKB1	MADCAM1	ITGA4	MADCAM1_ITGA4
2737	NFIC	MATN1	ITGB1	MATN1_ITGB1
2738	NFKBIA	MMP1	CD44	MMP1_CD44
2739	PAX5	MMP1	CD44	MMP1_CD44
2740	GATA3	MMP1	CD44	MMP1_CD44
2741	TWIST1	MMP1	CD44	MMP1_CD44
2742	TP53	MMP1	CD44	MMP1_CD44
2743	PPARG	MMP1	CD44	MMP1_CD44
2744	FOS	MMP1	CD44	MMP1_CD44
2745	JUN	MMP1	CD44	MMP1_CD44
2746	FLI1	MMP1	CD44	MMP1_CD44
2747	RELA	MMP1	CD44	MMP1_CD44
2748	ETS1	MMP1	CD44	MMP1_CD44
2749	NR4A2	MMP1	CD44	MMP1_CD44
2750	ETS2	MMP1	CD44	MMP1_CD44
2751	CEBPB	MMP1	CD44	MMP1_CD44
2752	ETV3	MMP1	CD44	MMP1_CD44
2753	FOSL1	MMP1	CD44	MMP1_CD44
2754	STAT3	MMP1	CD44	MMP1_CD44
2755	ETV4	MMP1	CD44	MMP1_CD44
2756	NFKB1	MMP1	CD44	MMP1_CD44
2757	BACH1	MMP1	CD44	MMP1_CD44
2758	CITED2	MMP1	CD44	MMP1_CD44
2759	RFWD2	MMP1	CD44	MMP1_CD44
2760	ZNF410	MMP1	CD44	MMP1_CD44
2761	MAZ	MMP1	CD44	MMP1_CD44
2762	JUN	MMP12	PLAUR	MMP12_PLAUR
2763	RELA	MMP12	PLAUR	MMP12_PLAUR
2764	YBX1	MMP13	LRP1	MMP13_LRP1
2765	RUNX2	MMP13	LRP1	MMP13_LRP1
2766	MAF	MMP13	LRP1	MMP13_LRP1
2767	ESR1	MMP13	LRP1	MMP13_LRP1
2768	ETS1	MMP13	LRP1	MMP13_LRP1
2769	HDAC4	MMP13	LRP1	MMP13_LRP1
2770	HDAC9	MMP13	LRP1	MMP13_LRP1
2771	JUN	MMP13	LRP1	MMP13_LRP1
2772	CITED2	MMP13	LRP1	MMP13_LRP1
2773	RELA	MMP13	LRP1	MMP13_LRP1
2774	NFKB1	MMP13	LRP1	MMP13_LRP1
2775	ING2	MMP13	LRP1	MMP13_LRP1
2776	STAT1	MMP13	LRP1	MMP13_LRP1
2777	HTATIP2	MMP2	SDC2	MMP2_SDC2
2778	SRF	MMP2	SDC2	MMP2_SDC2
2779	HDAC3	MMP2	SDC2	MMP2_SDC2
2780	ATF2	MMP2	SDC2	MMP2_SDC2
2781	STAT3	MMP2	SDC2	MMP2_SDC2
2782	NR4A1	MMP2	SDC2	MMP2_SDC2
2783	HOXB7	MMP2	SDC2	MMP2_SDC2

2784	TP53	MMP2	SDC2	MMP2_SDC2
2785	KLF4	MMP2	SDC2	MMP2_SDC2
2786	EZH2	MMP2	SDC2	MMP2_SDC2
2787	TWIST1	MMP2	SDC2	MMP2_SDC2
2788	YBX1	MMP2	SDC2	MMP2_SDC2
2789	SP3	MMP2	SDC2	MMP2_SDC2
2790	ATF3	MMP2	SDC2	MMP2_SDC2
2791	SSX2	MMP2	SDC2	MMP2_SDC2
2792	JUN	MMP2	SDC2	MMP2_SDC2
2793	CREB1	MMP2	SDC2	MMP2_SDC2
2794	HIF1A	MMP2	SDC2	MMP2_SDC2
2795	RUNX2	MMP2	SDC2	MMP2_SDC2
2796	CEBPE	MMP2	SDC2	MMP2_SDC2
2797	TFAP2C	MMP2	SDC2	MMP2_SDC2
2798	SP1	MMP2	SDC2	MMP2_SDC2
2799	ETV4	MMP2	SDC2	MMP2_SDC2
2800	ETS2	MMP2	SDC2	MMP2_SDC2
2801	MZF1	MMP2	SDC2	MMP2_SDC2
2802	PAX6	MMP2	SDC2	MMP2_SDC2
2803	MSC	MMP2	SDC2	MMP2_SDC2
2804	ETV5	MMP2	SDC2	MMP2_SDC2
2805	RELA	MMP2	SDC2	MMP2_SDC2
2806	TWIST2	MMP2	SDC2	MMP2_SDC2
2807	NFKB1	MMP2	SDC2	MMP2_SDC2
2808	TFAP2A	MMP2	SDC2	MMP2_SDC2
2809	CTNNB1	MMP7	CDH6	MMP7_CDH6
2810	CTNNB1	MMP7	ERBB4	MMP7_ERBB4
2811	CTNNB1	MMP7	CD44	MMP7_CD44
2812	CTNNB1	MMP7	CD151	MMP7_CD151
2813	STAT3	MMP7	CDH6	MMP7_CDH6
2814	STAT3	MMP7	ERBB4	MMP7_ERBB4
2815	STAT3	MMP7	CD44	MMP7_CD44
2816	STAT3	MMP7	CD151	MMP7_CD151
2817	JUN	MMP7	CDH6	MMP7_CDH6
2818	JUN	MMP7	ERBB4	MMP7_ERBB4
2819	JUN	MMP7	CD44	MMP7_CD44
2820	JUN	MMP7	CD151	MMP7_CD151
2821	FOXA2	MMP7	CDH6	MMP7_CDH6
2822	FOXA2	MMP7	ERBB4	MMP7_ERBB4
2823	FOXA2	MMP7	CD44	MMP7_CD44
2824	FOXA2	MMP7	CD151	MMP7_CD151
2825	HNF4A	MMP7	CDH6	MMP7_CDH6
2826	HNF4A	MMP7	ERBB4	MMP7_ERBB4
2827	HNF4A	MMP7	CD44	MMP7_CD44
2828	HNF4A	MMP7	CD151	MMP7_CD151
2829	ETV4	MMP7	CDH6	MMP7_CDH6
2830	ETV4	MMP7	ERBB4	MMP7_ERBB4
2831	ETV4	MMP7	CD44	MMP7_CD44
2832	ETV4	MMP7	CD151	MMP7_CD151
2833	NCOA3	MMP7	CDH6	MMP7_CDH6
2834	NCOA3	MMP7	ERBB4	MMP7_ERBB4
2835	NCOA3	MMP7	CD44	MMP7_CD44
2836	NCOA3	MMP7	CD151	MMP7_CD151
2837	FOS	MMP7	CDH6	MMP7_CDH6
2838	FOS	MMP7	ERBB4	MMP7_ERBB4
2839	FOS	MMP7	CD44	MMP7_CD44
2840	FOS	MMP7	CD151	MMP7_CD151
2841	LEF1	MMP7	CDH6	MMP7_CDH6

2842	LEF1	MMP7	ERBB4	MMP7_ERBB4
2843	LEF1	MMP7	CD44	MMP7_CD44
2844	LEF1	MMP7	CD151	MMP7_CD151
2845	SP1	MMP9	LRP1	MMP9_LRP1
2846	SP1	MMP9	CD44	MMP9_CD44
2847	SP1	MMP9	ITGB2	MMP9_ITGB2
2848	SP1	MMP9	ITGAM	MMP9_ITGAM
2849	SMAD3	MMP9	LRP1	MMP9_LRP1
2850	SMAD3	MMP9	CD44	MMP9_CD44
2851	SMAD3	MMP9	ITGB2	MMP9_ITGB2
2852	SMAD3	MMP9	ITGAM	MMP9_ITGAM
2853	SPDEF	MMP9	LRP1	MMP9_LRP1
2854	SPDEF	MMP9	CD44	MMP9_CD44
2855	SPDEF	MMP9	ITGB2	MMP9_ITGB2
2856	SPDEF	MMP9	ITGAM	MMP9_ITGAM
2857	IRF1	MMP9	LRP1	MMP9_LRP1
2858	IRF1	MMP9	CD44	MMP9_CD44
2859	IRF1	MMP9	ITGB2	MMP9_ITGB2
2860	IRF1	MMP9	ITGAM	MMP9_ITGAM
2861	ETS2	MMP9	LRP1	MMP9_LRP1
2862	ETS2	MMP9	CD44	MMP9_CD44
2863	ETS2	MMP9	ITGB2	MMP9_ITGB2
2864	ETS2	MMP9	ITGAM	MMP9_ITGAM
2865	CIITA	MMP9	LRP1	MMP9_LRP1
2866	CIITA	MMP9	CD44	MMP9_CD44
2867	CIITA	MMP9	ITGB2	MMP9_ITGB2
2868	CIITA	MMP9	ITGAM	MMP9_ITGAM
2869	KLF6	MMP9	LRP1	MMP9_LRP1
2870	KLF6	MMP9	CD44	MMP9_CD44
2871	KLF6	MMP9	ITGB2	MMP9_ITGB2
2872	KLF6	MMP9	ITGAM	MMP9_ITGAM
2873	SIRT1	MMP9	LRP1	MMP9_LRP1
2874	SIRT1	MMP9	CD44	MMP9_CD44
2875	SIRT1	MMP9	ITGB2	MMP9_ITGB2
2876	SIRT1	MMP9	ITGAM	MMP9_ITGAM
2877	RELA	MMP9	LRP1	MMP9_LRP1
2878	RELA	MMP9	CD44	MMP9_CD44
2879	RELA	MMP9	ITGB2	MMP9_ITGB2
2880	RELA	MMP9	ITGAM	MMP9_ITGAM
2881	KLF5	MMP9	LRP1	MMP9_LRP1
2882	KLF5	MMP9	CD44	MMP9_CD44
2883	KLF5	MMP9	ITGB2	MMP9_ITGB2
2884	KLF5	MMP9	ITGAM	MMP9_ITGAM
2885	NFKB1	MMP9	LRP1	MMP9_LRP1
2886	NFKB1	MMP9	CD44	MMP9_CD44
2887	NFKB1	MMP9	ITGB2	MMP9_ITGB2
2888	NFKB1	MMP9	ITGAM	MMP9_ITGAM
2889	ELF4	MMP9	LRP1	MMP9_LRP1
2890	ELF4	MMP9	CD44	MMP9_CD44
2891	ELF4	MMP9	ITGB2	MMP9_ITGB2
2892	ELF4	MMP9	ITGAM	MMP9_ITGAM
2893	STAT3	MMP9	LRP1	MMP9_LRP1
2894	STAT3	MMP9	CD44	MMP9_CD44
2895	STAT3	MMP9	ITGB2	MMP9_ITGB2
2896	STAT3	MMP9	ITGAM	MMP9_ITGAM
2897	HDAC1	MMP9	LRP1	MMP9_LRP1
2898	HDAC1	MMP9	CD44	MMP9_CD44
2899	HDAC1	MMP9	ITGB2	MMP9_ITGB2

2900	HDAC1	MMP9	ITGAM	MMP9_ITGAM
2901	ELF3	MMP9	LRP1	MMP9_LRP1
2902	ELF3	MMP9	CD44	MMP9_CD44
2903	ELF3	MMP9	ITGB2	MMP9_ITGB2
2904	ELF3	MMP9	ITGAM	MMP9_ITGAM
2905	FOS	MMP9	LRP1	MMP9_LRP1
2906	FOS	MMP9	CD44	MMP9_CD44
2907	FOS	MMP9	ITGB2	MMP9_ITGB2
2908	FOS	MMP9	ITGAM	MMP9_ITGAM
2909	PPARA	MMP9	LRP1	MMP9_LRP1
2910	PPARA	MMP9	CD44	MMP9_CD44
2911	PPARA	MMP9	ITGB2	MMP9_ITGB2
2912	PPARA	MMP9	ITGAM	MMP9_ITGAM
2913	KLF8	MMP9	LRP1	MMP9_LRP1
2914	KLF8	MMP9	CD44	MMP9_CD44
2915	KLF8	MMP9	ITGB2	MMP9_ITGB2
2916	KLF8	MMP9	ITGAM	MMP9_ITGAM
2917	TFAP2A	MMP9	LRP1	MMP9_LRP1
2918	TFAP2A	MMP9	CD44	MMP9_CD44
2919	TFAP2A	MMP9	ITGB2	MMP9_ITGB2
2920	TFAP2A	MMP9	ITGAM	MMP9_ITGAM
2921	RUNX3	MMP9	LRP1	MMP9_LRP1
2922	RUNX3	MMP9	CD44	MMP9_CD44
2923	RUNX3	MMP9	ITGB2	MMP9_ITGB2
2924	RUNX3	MMP9	ITGAM	MMP9_ITGAM
2925	JUN	MMP9	LRP1	MMP9_LRP1
2926	JUN	MMP9	CD44	MMP9_CD44
2927	JUN	MMP9	ITGB2	MMP9_ITGB2
2928	JUN	MMP9	ITGAM	MMP9_ITGAM
2929	MAL	MMP9	LRP1	MMP9_LRP1
2930	MAL	MMP9	CD44	MMP9_CD44
2931	MAL	MMP9	ITGB2	MMP9_ITGB2
2932	MAL	MMP9	ITGAM	MMP9_ITGAM
2933	STAT1	MMP9	LRP1	MMP9_LRP1
2934	STAT1	MMP9	CD44	MMP9_CD44
2935	STAT1	MMP9	ITGB2	MMP9_ITGB2
2936	STAT1	MMP9	ITGAM	MMP9_ITGAM
2937	PPARG	MMP9	LRP1	MMP9_LRP1
2938	PPARG	MMP9	CD44	MMP9_CD44
2939	PPARG	MMP9	ITGB2	MMP9_ITGB2
2940	PPARG	MMP9	ITGAM	MMP9_ITGAM
2941	MAZ	MMP9	LRP1	MMP9_LRP1
2942	MAZ	MMP9	CD44	MMP9_CD44
2943	MAZ	MMP9	ITGB2	MMP9_ITGB2
2944	MAZ	MMP9	ITGAM	MMP9_ITGAM
2945	SP2	MMP9	LRP1	MMP9_LRP1
2946	SP2	MMP9	CD44	MMP9_CD44
2947	SP2	MMP9	ITGB2	MMP9_ITGB2
2948	SP2	MMP9	ITGAM	MMP9_ITGAM
2949	SNAI2	MMP9	LRP1	MMP9_LRP1
2950	SNAI2	MMP9	CD44	MMP9_CD44
2951	SNAI2	MMP9	ITGB2	MMP9_ITGB2
2952	SNAI2	MMP9	ITGAM	MMP9_ITGAM
2953	EP300	MMP9	LRP1	MMP9_LRP1
2954	EP300	MMP9	CD44	MMP9_CD44
2955	EP300	MMP9	ITGB2	MMP9_ITGB2
2956	EP300	MMP9	ITGAM	MMP9_ITGAM
2957	IKBKB	MMP9	LRP1	MMP9_LRP1

2958	IKBKB	MMP9	CD44	MMP9_CD44
2959	IKBKB	MMP9	ITGB2	MMP9_ITGB2
2960	IKBKB	MMP9	ITGAM	MMP9_ITGAM
2961	ETS1	MMP9	LRP1	MMP9_LRP1
2962	ETS1	MMP9	CD44	MMP9_CD44
2963	ETS1	MMP9	ITGB2	MMP9_ITGB2
2964	ETS1	MMP9	ITGAM	MMP9_ITGAM
2965	SRF	MMP9	LRP1	MMP9_LRP1
2966	SRF	MMP9	CD44	MMP9_CD44
2967	SRF	MMP9	ITGB2	MMP9_ITGB2
2968	SRF	MMP9	ITGAM	MMP9_ITGAM
2969	MTA1	MMP9	LRP1	MMP9_LRP1
2970	MTA1	MMP9	CD44	MMP9_CD44
2971	MTA1	MMP9	ITGB2	MMP9_ITGB2
2972	MTA1	MMP9	ITGAM	MMP9_ITGAM
2973	NFKBIA	MMP9	LRP1	MMP9_LRP1
2974	NFKBIA	MMP9	CD44	MMP9_CD44
2975	NFKBIA	MMP9	ITGB2	MMP9_ITGB2
2976	NFKBIA	MMP9	ITGAM	MMP9_ITGAM
2977	MYC	MST1	MST1R	MST1_MST1R
2978	EZH2	MST1	MST1R	MST1_MST1R
2979	JUN	NAMPT	INSR	NAMPT_INSR
2980	CLOCK	NAMPT	INSR	NAMPT_INSR
2981	NFKB1	NAMPT	INSR	NAMPT_INSR
2982	RELA	NAMPT	INSR	NAMPT_INSR
2983	RELA	NCAM1	PTPRA	NCAM1_PTPRA
2984	RUNX1	NCAM1	PTPRA	NCAM1_PTPRA
2985	PAX8	NCAM1	PTPRA	NCAM1_PTPRA
2986	PAX2	NCAM1	PTPRA	NCAM1_PTPRA
2987	NFKB1	NCAM1	PTPRA	NCAM1_PTPRA
2988	SOX4	NCAM1	PTPRA	NCAM1_PTPRA
2989	IRF9	NCAM1	PTPRA	NCAM1_PTPRA
2990	ING4	NGF	NGFRAP1	NGF_NGFRAP1
2991	JUN	NGF	NGFRAP1	NGF_NGFRAP1
2992	FOS	NGF	NGFRAP1	NGF_NGFRAP1
2993	ELK1	PDGFB	LRP1	PDGFB_LRP1
2994	FOXO1	PDGFB	LRP1	PDGFB_LRP1
2995	EGR1	PDGFB	LRP1	PDGFB_LRP1
2996	SP1	PDGFB	LRP1	PDGFB_LRP1
2997	USF1	PF4	THBD	PF4_THBD
2998	USF1	PF4	CXCR3	PF4_CXCR3
2999	PBX2	PF4	THBD	PF4_THBD
3000	PBX2	PF4	CXCR3	PF4_CXCR3
3001	ETS1	PF4	THBD	PF4_THBD
3002	ETS1	PF4	CXCR3	PF4_CXCR3
3003	RUNX1	PF4	THBD	PF4_THBD
3004	RUNX1	PF4	CXCR3	PF4_CXCR3
3005	MEIS1	PF4	THBD	PF4_THBD
3006	MEIS1	PF4	CXCR3	PF4_CXCR3
3007	FLI1	PF4	THBD	PF4_THBD
3008	FLI1	PF4	CXCR3	PF4_CXCR3
3009	PBX1	PF4	THBD	PF4_THBD
3010	PBX1	PF4	CXCR3	PF4_CXCR3
3011	USF2	PF4	THBD	PF4_THBD
3012	USF2	PF4	CXCR3	PF4_CXCR3
3013	PKNOX1	PF4	THBD	PF4_THBD
3014	PKNOX1	PF4	CXCR3	PF4_CXCR3
3015	TIAL1	PF4	THBD	PF4_THBD

3016	TIAL1	PF4	CXCR3	PF4_CXCR3
3017	GATA1	PF4	THBD	PF4_THBD
3018	GATA1	PF4	CXCR3	PF4_CXCR3
3019	AR	PIP	CD4	PIP_CD4
3020	RUNX2	PIP	CD4	PIP_CD4
3021	ZBTB7A	PKM	CD44	PKM_CD44
3022	CREM	PLAT	LRP1	PLAT_LRP1
3023	CREM	PLAT	ITGB2	PLAT_ITGB2
3024	CREM	PLAT	ITGAM	PLAT_ITGAM
3025	SP1	PLAT	LRP1	PLAT_LRP1
3026	SP1	PLAT	ITGB2	PLAT_ITGB2
3027	SP1	PLAT	ITGAM	PLAT_ITGAM
3028	NF1	PLAT	LRP1	PLAT_LRP1
3029	NF1	PLAT	ITGB2	PLAT_ITGB2
3030	NF1	PLAT	ITGAM	PLAT_ITGAM
3031	SP3	PLAT	LRP1	PLAT_LRP1
3032	SP3	PLAT	ITGB2	PLAT_ITGB2
3033	SP3	PLAT	ITGAM	PLAT_ITGAM
3034	ATF2	PLAT	LRP1	PLAT_LRP1
3035	ATF2	PLAT	ITGB2	PLAT_ITGB2
3036	ATF2	PLAT	ITGAM	PLAT_ITGAM
3037	CREB1	PLAT	LRP1	PLAT_LRP1
3038	CREB1	PLAT	ITGB2	PLAT_ITGB2
3039	CREB1	PLAT	ITGAM	PLAT_ITGAM
3040	JUN	PLAT	LRP1	PLAT_LRP1
3041	JUN	PLAT	ITGB2	PLAT_ITGB2
3042	JUN	PLAT	ITGAM	PLAT_ITGAM
3043	JUND	PLAT	LRP1	PLAT_LRP1
3044	JUND	PLAT	ITGB2	PLAT_ITGB2
3045	JUND	PLAT	ITGAM	PLAT_ITGAM
3046	POU2F1	PLAU	ITGB1	PLAU_ITGB1
3047	POU2F1	PLAU	LRP1	PLAU_LRP1
3048	POU2F1	PLAU	ST14	PLAU_ST14
3049	POU2F1	PLAU	PLAUR	PLAU_PLAUR
3050	POU2F1	PLAU	ITGAM	PLAU_ITGAM
3051	POU2F1	PLAU	ITGA3	PLAU_ITGA3
3052	POU2F1	PLAU	ITGB2	PLAU_ITGB2
3053	NFKB1	PLAU	ITGB1	PLAU_ITGB1
3054	NFKB1	PLAU	LRP1	PLAU_LRP1
3055	NFKB1	PLAU	ST14	PLAU_ST14
3056	NFKB1	PLAU	PLAUR	PLAU_PLAUR
3057	NFKB1	PLAU	ITGAM	PLAU_ITGAM
3058	NFKB1	PLAU	ITGA3	PLAU_ITGA3
3059	NFKB1	PLAU	ITGB2	PLAU_ITGB2
3060	SRF	PLAU	ITGB1	PLAU_ITGB1
3061	SRF	PLAU	LRP1	PLAU_LRP1
3062	SRF	PLAU	ST14	PLAU_ST14
3063	SRF	PLAU	PLAUR	PLAU_PLAUR
3064	SRF	PLAU	ITGAM	PLAU_ITGAM
3065	SRF	PLAU	ITGA3	PLAU_ITGA3
3066	SRF	PLAU	ITGB2	PLAU_ITGB2
3067	ATF4	PLAU	ITGB1	PLAU_ITGB1
3068	ATF4	PLAU	LRP1	PLAU_LRP1
3069	ATF4	PLAU	ST14	PLAU_ST14
3070	ATF4	PLAU	PLAUR	PLAU_PLAUR
3071	ATF4	PLAU	ITGAM	PLAU_ITGAM
3072	ATF4	PLAU	ITGA3	PLAU_ITGA3
3073	ATF4	PLAU	ITGB2	PLAU_ITGB2

3074	JUN	PLAU	ITGB1	PLAU_ITGB1
3075	JUN	PLAU	LRP1	PLAU_LRP1
3076	JUN	PLAU	ST14	PLAU_ST14
3077	JUN	PLAU	PLAUR	PLAU_PLAUR
3078	JUN	PLAU	ITGAM	PLAU_ITGAM
3079	JUN	PLAU	ITGA3	PLAU_ITGA3
3080	JUN	PLAU	ITGB2	PLAU_ITGB2
3081	HIF1A	PLAU	ITGB1	PLAU_ITGB1
3082	HIF1A	PLAU	LRP1	PLAU_LRP1
3083	HIF1A	PLAU	ST14	PLAU_ST14
3084	HIF1A	PLAU	PLAUR	PLAU_PLAUR
3085	HIF1A	PLAU	ITGAM	PLAU_ITGAM
3086	HIF1A	PLAU	ITGA3	PLAU_ITGA3
3087	HIF1A	PLAU	ITGB2	PLAU_ITGB2
3088	SP1	PLAU	ITGB1	PLAU_ITGB1
3089	SP1	PLAU	LRP1	PLAU_LRP1
3090	SP1	PLAU	ST14	PLAU_ST14
3091	SP1	PLAU	PLAUR	PLAU_PLAUR
3092	SP1	PLAU	ITGAM	PLAU_ITGAM
3093	SP1	PLAU	ITGA3	PLAU_ITGA3
3094	SP1	PLAU	ITGB2	PLAU_ITGB2
3095	RFWD2	PLAU	ITGB1	PLAU_ITGB1
3096	RFWD2	PLAU	LRP1	PLAU_LRP1
3097	RFWD2	PLAU	ST14	PLAU_ST14
3098	RFWD2	PLAU	PLAUR	PLAU_PLAUR
3099	RFWD2	PLAU	ITGAM	PLAU_ITGAM
3100	RFWD2	PLAU	ITGA3	PLAU_ITGA3
3101	RFWD2	PLAU	ITGB2	PLAU_ITGB2
3102	GATA6	PLAU	ITGB1	PLAU_ITGB1
3103	GATA6	PLAU	LRP1	PLAU_LRP1
3104	GATA6	PLAU	ST14	PLAU_ST14
3105	GATA6	PLAU	PLAUR	PLAU_PLAUR
3106	GATA6	PLAU	ITGAM	PLAU_ITGAM
3107	GATA6	PLAU	ITGA3	PLAU_ITGA3
3108	GATA6	PLAU	ITGB2	PLAU_ITGB2
3109	EGR1	PLAU	ITGB1	PLAU_ITGB1
3110	EGR1	PLAU	LRP1	PLAU_LRP1
3111	EGR1	PLAU	ST14	PLAU_ST14
3112	EGR1	PLAU	PLAUR	PLAU_PLAUR
3113	EGR1	PLAU	ITGAM	PLAU_ITGAM
3114	EGR1	PLAU	ITGA3	PLAU_ITGA3
3115	EGR1	PLAU	ITGB2	PLAU_ITGB2
3116	ILF3	PLAU	ITGB1	PLAU_ITGB1
3117	ILF3	PLAU	LRP1	PLAU_LRP1
3118	ILF3	PLAU	ST14	PLAU_ST14
3119	ILF3	PLAU	PLAUR	PLAU_PLAUR
3120	ILF3	PLAU	ITGAM	PLAU_ITGAM
3121	ILF3	PLAU	ITGA3	PLAU_ITGA3
3122	ILF3	PLAU	ITGB2	PLAU_ITGB2
3123	RUNX2	PLAU	ITGB1	PLAU_ITGB1
3124	RUNX2	PLAU	LRP1	PLAU_LRP1
3125	RUNX2	PLAU	ST14	PLAU_ST14
3126	RUNX2	PLAU	PLAUR	PLAU_PLAUR
3127	RUNX2	PLAU	ITGAM	PLAU_ITGAM
3128	RUNX2	PLAU	ITGA3	PLAU_ITGA3
3129	RUNX2	PLAU	ITGB2	PLAU_ITGB2
3130	CREB1	PLAU	ITGB1	PLAU_ITGB1
3131	CREB1	PLAU	LRP1	PLAU_LRP1

3132	CREB1	PLAU	ST14	PLAU_ST14
3133	CREB1	PLAU	PLAUR	PLAU_PLAUR
3134	CREB1	PLAU	ITGAM	PLAU_ITGAM
3135	CREB1	PLAU	ITGA3	PLAU_ITGA3
3136	CREB1	PLAU	ITGB2	PLAU_ITGB2
3137	ETS1	PLAU	ITGB1	PLAU_ITGB1
3138	ETS1	PLAU	LRP1	PLAU_LRP1
3139	ETS1	PLAU	ST14	PLAU_ST14
3140	ETS1	PLAU	PLAUR	PLAU_PLAUR
3141	ETS1	PLAU	ITGAM	PLAU_ITGAM
3142	ETS1	PLAU	ITGA3	PLAU_ITGA3
3143	ETS1	PLAU	ITGB2	PLAU_ITGB2
3144	ATF2	PLAU	ITGB1	PLAU_ITGB1
3145	ATF2	PLAU	LRP1	PLAU_LRP1
3146	ATF2	PLAU	ST14	PLAU_ST14
3147	ATF2	PLAU	PLAUR	PLAU_PLAUR
3148	ATF2	PLAU	ITGAM	PLAU_ITGAM
3149	ATF2	PLAU	ITGA3	PLAU_ITGA3
3150	ATF2	PLAU	ITGB2	PLAU_ITGB2
3151	RELA	PLAU	ITGB1	PLAU_ITGB1
3152	RELA	PLAU	LRP1	PLAU_LRP1
3153	RELA	PLAU	ST14	PLAU_ST14
3154	RELA	PLAU	PLAUR	PLAU_PLAUR
3155	RELA	PLAU	ITGAM	PLAU_ITGAM
3156	RELA	PLAU	ITGA3	PLAU_ITGA3
3157	RELA	PLAU	ITGB2	PLAU_ITGB2
3158	FOS	PLAU	ITGB1	PLAU_ITGB1
3159	FOS	PLAU	LRP1	PLAU_LRP1
3160	FOS	PLAU	ST14	PLAU_ST14
3161	FOS	PLAU	PLAUR	PLAU_PLAUR
3162	FOS	PLAU	ITGAM	PLAU_ITGAM
3163	FOS	PLAU	ITGA3	PLAU_ITGA3
3164	FOS	PLAU	ITGB2	PLAU_ITGB2
3165	RARA	PLAU	ITGB1	PLAU_ITGB1
3166	RARA	PLAU	LRP1	PLAU_LRP1
3167	RARA	PLAU	ST14	PLAU_ST14
3168	RARA	PLAU	PLAUR	PLAU_PLAUR
3169	RARA	PLAU	ITGAM	PLAU_ITGAM
3170	RARA	PLAU	ITGA3	PLAU_ITGA3
3171	RARA	PLAU	ITGB2	PLAU_ITGB2
3172	HOXD3	PLAU	ITGB1	PLAU_ITGB1
3173	HOXD3	PLAU	LRP1	PLAU_LRP1
3174	HOXD3	PLAU	ST14	PLAU_ST14
3175	HOXD3	PLAU	PLAUR	PLAU_PLAUR
3176	HOXD3	PLAU	ITGAM	PLAU_ITGAM
3177	HOXD3	PLAU	ITGA3	PLAU_ITGA3
3178	HOXD3	PLAU	ITGB2	PLAU_ITGB2
3179	SRF	PLG	ITGB2	PLG_ITGB2
3180	SRF	PLG	ITGAM	PLG_ITGAM
3181	SRF	PLG	PLAUR	PLG_PLAUR
3182	SRF	PLG	ITGB1	PLG_ITGB1
3183	SRF	PLG	PLGRKT	PLG_PLGRKT
3184	NR1H4	PLTP	ABCA1	PLTP_ABCA1
3185	SP1	PNOC	OPRL1	PNOC_OPRL1
3186	VDR	PODXL	SELL	PODXL_SELL
3187	WT1	PODXL	SELL	PODXL_SELL
3188	ONECUT1	PROC	THBD	PROC_THBD
3189	ONECUT1	PROC	ITGB2	PROC_ITGB2

3190	ONECUT1	PROC	ITGAM	PROC_ITGAM
3191	STAT3	PROS1	TYRO3	PROS1_TYRO3
3192	STAT3	PROS1	AXL	PROS1_AXL
3193	ELK1	PSEN1	CD44	PSEN1_CD44
3194	SP1	PSEN1	CD44	PSEN1_CD44
3195	CREB1	PSEN1	CD44	PSEN1_CD44
3196	CEBPB	PTGS2	CAV1	PTGS2_CAV1
3197	CEBPB	PTGS2	ALOX5	PTGS2_ALOX5
3198	PPARA	PTGS2	CAV1	PTGS2_CAV1
3199	PPARA	PTGS2	ALOX5	PTGS2_ALOX5
3200	HDAC4	PTGS2	CAV1	PTGS2_CAV1
3201	HDAC4	PTGS2	ALOX5	PTGS2_ALOX5
3202	SETBP1	PTGS2	CAV1	PTGS2_CAV1
3203	SETBP1	PTGS2	ALOX5	PTGS2_ALOX5
3204	STAT2	PTGS2	CAV1	PTGS2_CAV1
3205	STAT2	PTGS2	ALOX5	PTGS2_ALOX5
3206	STAT1	PTGS2	CAV1	PTGS2_CAV1
3207	STAT1	PTGS2	ALOX5	PTGS2_ALOX5
3208	USF1	PTGS2	CAV1	PTGS2_CAV1
3209	USF1	PTGS2	ALOX5	PTGS2_ALOX5
3210	ETV4	PTGS2	CAV1	PTGS2_CAV1
3211	ETV4	PTGS2	ALOX5	PTGS2_ALOX5
3212	PPARG	PTGS2	CAV1	PTGS2_CAV1
3213	PPARG	PTGS2	ALOX5	PTGS2_ALOX5
3214	ENO1	PTGS2	CAV1	PTGS2_CAV1
3215	ENO1	PTGS2	ALOX5	PTGS2_ALOX5
3216	EGR2	PTGS2	CAV1	PTGS2_CAV1
3217	EGR2	PTGS2	ALOX5	PTGS2_ALOX5
3218	ATF2	PTGS2	CAV1	PTGS2_CAV1
3219	ATF2	PTGS2	ALOX5	PTGS2_ALOX5
3220	ATF4	PTGS2	CAV1	PTGS2_CAV1
3221	ATF4	PTGS2	ALOX5	PTGS2_ALOX5
3222	APC	PTGS2	CAV1	PTGS2_CAV1
3223	APC	PTGS2	ALOX5	PTGS2_ALOX5
3224	JUNB	PTGS2	CAV1	PTGS2_CAV1
3225	JUNB	PTGS2	ALOX5	PTGS2_ALOX5
3226	DR1	PTGS2	CAV1	PTGS2_CAV1
3227	DR1	PTGS2	ALOX5	PTGS2_ALOX5
3228	HDAC1	PTGS2	CAV1	PTGS2_CAV1
3229	HDAC1	PTGS2	ALOX5	PTGS2_ALOX5
3230	CEBPD	PTGS2	CAV1	PTGS2_CAV1
3231	CEBPD	PTGS2	ALOX5	PTGS2_ALOX5
3232	ELF3	PTGS2	CAV1	PTGS2_CAV1
3233	ELF3	PTGS2	ALOX5	PTGS2_ALOX5
3234	ING4	PTGS2	CAV1	PTGS2_CAV1
3235	ING4	PTGS2	ALOX5	PTGS2_ALOX5
3236	STAT3	PTGS2	CAV1	PTGS2_CAV1
3237	STAT3	PTGS2	ALOX5	PTGS2_ALOX5
3238	SP1	PTGS2	CAV1	PTGS2_CAV1
3239	SP1	PTGS2	ALOX5	PTGS2_ALOX5
3240	FOS	PTGS2	CAV1	PTGS2_CAV1
3241	FOS	PTGS2	ALOX5	PTGS2_ALOX5
3242	JUND	PTGS2	CAV1	PTGS2_CAV1
3243	JUND	PTGS2	ALOX5	PTGS2_ALOX5
3244	CTNNB1	PTGS2	CAV1	PTGS2_CAV1
3245	CTNNB1	PTGS2	ALOX5	PTGS2_ALOX5
3246	AR	PTGS2	CAV1	PTGS2_CAV1
3247	AR	PTGS2	ALOX5	PTGS2_ALOX5

3248	NFIL3	PTGS2	CAV1	PTGS2_CAV1
3249	NFIL3	PTGS2	ALOX5	PTGS2_ALOX5
3250	JUN	PTGS2	CAV1	PTGS2_CAV1
3251	JUN	PTGS2	ALOX5	PTGS2_ALOX5
3252	RELA	PTGS2	CAV1	PTGS2_CAV1
3253	RELA	PTGS2	ALOX5	PTGS2_ALOX5
3254	HMGA1	PTGS2	CAV1	PTGS2_CAV1
3255	HMGA1	PTGS2	ALOX5	PTGS2_ALOX5
3256	NR0B2	PTGS2	CAV1	PTGS2_CAV1
3257	NR0B2	PTGS2	ALOX5	PTGS2_ALOX5
3258	PGR	PTGS2	CAV1	PTGS2_CAV1
3259	PGR	PTGS2	ALOX5	PTGS2_ALOX5
3260	USF2	PTGS2	CAV1	PTGS2_CAV1
3261	USF2	PTGS2	ALOX5	PTGS2_ALOX5
3262	CDX2	PTGS2	CAV1	PTGS2_CAV1
3263	CDX2	PTGS2	ALOX5	PTGS2_ALOX5
3264	CREBBP	PTGS2	CAV1	PTGS2_CAV1
3265	CREBBP	PTGS2	ALOX5	PTGS2_ALOX5
3266	STAT6	PTGS2	CAV1	PTGS2_CAV1
3267	STAT6	PTGS2	ALOX5	PTGS2_ALOX5
3268	EP300	PTGS2	CAV1	PTGS2_CAV1
3269	EP300	PTGS2	ALOX5	PTGS2_ALOX5
3270	NFKB1	PTGS2	CAV1	PTGS2_CAV1
3271	NFKB1	PTGS2	ALOX5	PTGS2_ALOX5
3272	EGR1	PTGS2	CAV1	PTGS2_CAV1
3273	EGR1	PTGS2	ALOX5	PTGS2_ALOX5
3274	CDX1	PTGS2	CAV1	PTGS2_CAV1
3275	CDX1	PTGS2	ALOX5	PTGS2_ALOX5
3276	CREB1	PTGS2	CAV1	PTGS2_CAV1
3277	CREB1	PTGS2	ALOX5	PTGS2_ALOX5
3278	TFAP2A	PTGS2	CAV1	PTGS2_CAV1
3279	TFAP2A	PTGS2	ALOX5	PTGS2_ALOX5
3280	TCF7L2	PTGS2	CAV1	PTGS2_CAV1
3281	TCF7L2	PTGS2	ALOX5	PTGS2_ALOX5
3282	ESR1	PTMA	VIPR1	PTMA_VIPR1
3283	JUN	PTN	SDC1	PTN_SDC1
3284	JUN	PTN	PLXNB2	PTN_PLXNB2
3285	SP1	PTN	SDC1	PTN_SDC1
3286	SP1	PTN	PLXNB2	PTN_PLXNB2
3287	HOXA5	PTN	SDC1	PTN_SDC1
3288	HOXA5	PTN	PLXNB2	PTN_PLXNB2
3289	DNMT1	RELN	ITGB1	RELN_ITGB1
3290	DNMT1	RELN	ITGA3	RELN_ITGA3
3291	MECP2	RELN	ITGB1	RELN_ITGB1
3292	MECP2	RELN	ITGA3	RELN_ITGA3
3293	HDAC1	RELN	ITGB1	RELN_ITGB1
3294	HDAC1	RELN	ITGA3	RELN_ITGA3
3295	TBR1	RELN	ITGB1	RELN_ITGB1
3296	TBR1	RELN	ITGA3	RELN_ITGA3
3297	PPARG	REN	ATP6AP2	REN_ATP6AP2
3298	POU1F1	REN	ATP6AP2	REN_ATP6AP2
3299	JUN	REN	ATP6AP2	REN_ATP6AP2
3300	SPI1	RNASE2	TLR2	RNASE2_TLR2
3301	HNF4A	RNASE2	TLR2	RNASE2_TLR2
3302	CEBPA	S100A9	TLR4	S100A9_TLR4
3303	GLI1	S100A9	TLR4	S100A9_TLR4
3304	CEBPB	S100A9	TLR4	S100A9_TLR4
3305	CEBPB	SAA1	FPR2	SAA1_FPR2

3306	CEBPB	SAA1	FPR1	SAA1_FPR1
3307	CEBPB	SAA1	SCARB1	SAA1_SCARB1
3308	NFKB1	SAA1	FPR2	SAA1_FPR2
3309	NFKB1	SAA1	FPR1	SAA1_FPR1
3310	NFKB1	SAA1	SCARB1	SAA1_SCARB1
3311	STAT3	SAA1	FPR2	SAA1_FPR2
3312	STAT3	SAA1	FPR1	SAA1_FPR1
3313	STAT3	SAA1	SCARB1	SAA1_SCARB1
3314	CEBPA	SAA1	FPR2	SAA1_FPR2
3315	CEBPA	SAA1	FPR1	SAA1_FPR1
3316	CEBPA	SAA1	SCARB1	SAA1_SCARB1
3317	RELA	SAA1	FPR2	SAA1_FPR2
3318	RELA	SAA1	FPR1	SAA1_FPR1
3319	RELA	SAA1	SCARB1	SAA1_SCARB1
3320	CEBPA	SERPINC1	LRP1	SERPINC1_LRP1
3321	FOXA1	SERPINC1	LRP1	SERPINC1_LRP1
3322	NR2F1	SERPINC1	LRP1	SERPINC1_LRP1
3323	RXRA	SERPINC1	LRP1	SERPINC1_LRP1
3324	FOXA2	SERPINC1	LRP1	SERPINC1_LRP1
3325	HNF4A	SERPINC1	LRP1	SERPINC1_LRP1
3326	THRA	SERPINC1	LRP1	SERPINC1_LRP1
3327	NFKB1	SERPINE1	LRP1	SERPINE1_LRP1
3328	NFKB1	SERPINE1	PLAUR	SERPINE1_PLAUR
3329	RELA	SERPINE1	LRP1	SERPINE1_LRP1
3330	RELA	SERPINE1	PLAUR	SERPINE1_PLAUR
3331	ESR2	SERPINE1	LRP1	SERPINE1_LRP1
3332	ESR2	SERPINE1	PLAUR	SERPINE1_PLAUR
3333	NR4A1	SERPINE1	LRP1	SERPINE1_LRP1
3334	NR4A1	SERPINE1	PLAUR	SERPINE1_PLAUR
3335	SRF	SERPINE1	LRP1	SERPINE1_LRP1
3336	SRF	SERPINE1	PLAUR	SERPINE1_PLAUR
3337	SP1	SERPINE1	LRP1	SERPINE1_LRP1
3338	SP1	SERPINE1	PLAUR	SERPINE1_PLAUR
3339	SMAD3	SERPINE1	LRP1	SERPINE1_LRP1
3340	SMAD3	SERPINE1	PLAUR	SERPINE1_PLAUR
3341	PPARA	SERPINE1	LRP1	SERPINE1_LRP1
3342	PPARA	SERPINE1	PLAUR	SERPINE1_PLAUR
3343	SMAD4	SERPINE1	LRP1	SERPINE1_LRP1
3344	SMAD4	SERPINE1	PLAUR	SERPINE1_PLAUR
3345	ARNTL2	SERPINE1	LRP1	SERPINE1_LRP1
3346	ARNTL2	SERPINE1	PLAUR	SERPINE1_PLAUR
3347	EPAS1	SERPINE1	LRP1	SERPINE1_LRP1
3348	EPAS1	SERPINE1	PLAUR	SERPINE1_PLAUR
3349	PARP1	SERPINE1	LRP1	SERPINE1_LRP1
3350	PARP1	SERPINE1	PLAUR	SERPINE1_PLAUR
3351	PPARG	SERPINE1	LRP1	SERPINE1_LRP1
3352	PPARG	SERPINE1	PLAUR	SERPINE1_PLAUR
3353	KLF10	SERPINE1	LRP1	SERPINE1_LRP1
3354	KLF10	SERPINE1	PLAUR	SERPINE1_PLAUR
3355	USF1	SERPINE1	LRP1	SERPINE1_LRP1
3356	USF1	SERPINE1	PLAUR	SERPINE1_PLAUR
3357	TGIF1	SERPINE1	LRP1	SERPINE1_LRP1
3358	TGIF1	SERPINE1	PLAUR	SERPINE1_PLAUR
3359	ESR1	SERPINE1	LRP1	SERPINE1_LRP1
3360	ESR1	SERPINE1	PLAUR	SERPINE1_PLAUR
3361	E2F1	SERPINE1	LRP1	SERPINE1_LRP1
3362	E2F1	SERPINE1	PLAUR	SERPINE1_PLAUR
3363	HIF1A	SERPINE1	LRP1	SERPINE1_LRP1

3364	HIF1A	SERPINE1	PLAUR	SERPINE1_PLAUR
3365	HSF1	SERPINE1	LRP1	SERPINE1_LRP1
3366	HSF1	SERPINE1	PLAUR	SERPINE1_PLAUR
3367	RELA	SFTPA1	TLR2	SFTPA1_TLR2
3368	NKX2-1	SFTPA1	TLR2	SFTPA1_TLR2
3369	NFKB1	SFTPA1	TLR2	SFTPA1_TLR2
3370	JUNB	SFTPD	LY96	SFTPD_LY96
3371	JUNB	SFTPD	TLR4	SFTPD_TLR4
3372	CEBPG	SFTPD	LY96	SFTPD_LY96
3373	CEBPG	SFTPD	TLR4	SFTPD_TLR4
3374	JUND	SFTPD	LY96	SFTPD_LY96
3375	JUND	SFTPD	TLR4	SFTPD_TLR4
3376	FOSL1	SFTPD	LY96	SFTPD_LY96
3377	FOSL1	SFTPD	TLR4	SFTPD_TLR4
3378	RB1	SFTPD	LY96	SFTPD_LY96
3379	RB1	SFTPD	TLR4	SFTPD_TLR4
3380	CEBPA	SFTPD	LY96	SFTPD_LY96
3381	CEBPA	SFTPD	TLR4	SFTPD_TLR4
3382	CEBPB	SFTPD	LY96	SFTPD_LY96
3383	CEBPB	SFTPD	TLR4	SFTPD_TLR4
3384	HNF4A	SHBG	CLDN4	SHBG_CLDN4
3385	PAX3	SHH	PTCH2	SHH_PTCH2
3386	STAT3	SHH	PTCH2	SHH_PTCH2
3387	SOX9	SHH	PTCH2	SHH_PTCH2
3388	HDAC5	SLIT2	SDC1	SLIT2_SDC1
3389	IRF1	SLPI	CD4	SLPI_CD4
3390	TFCP2	SPP1	S1PR1	SPP1_S1PR1
3391	TFCP2	SPP1	CD44	SPP1_CD44
3392	TFCP2	SPP1	ITGA5	SPP1_ITGA5
3393	TFCP2	SPP1	ITGB1	SPP1_ITGB1
3394	TFCP2	SPP1	ITGAV	SPP1_ITGAV
3395	TFCP2	SPP1	ITGA4	SPP1_ITGA4
3396	TFCP2	SPP1	ITGA9	SPP1_ITGA9
3397	NR3C1	SPP1	S1PR1	SPP1_S1PR1
3398	NR3C1	SPP1	CD44	SPP1_CD44
3399	NR3C1	SPP1	ITGA5	SPP1_ITGA5
3400	NR3C1	SPP1	ITGB1	SPP1_ITGB1
3401	NR3C1	SPP1	ITGAV	SPP1_ITGAV
3402	NR3C1	SPP1	ITGA4	SPP1_ITGA4
3403	NR3C1	SPP1	ITGA9	SPP1_ITGA9
3404	POU5F1	SPP1	S1PR1	SPP1_S1PR1
3405	POU5F1	SPP1	CD44	SPP1_CD44
3406	POU5F1	SPP1	ITGA5	SPP1_ITGA5
3407	POU5F1	SPP1	ITGB1	SPP1_ITGB1
3408	POU5F1	SPP1	ITGAV	SPP1_ITGAV
3409	POU5F1	SPP1	ITGA4	SPP1_ITGA4
3410	POU5F1	SPP1	ITGA9	SPP1_ITGA9
3411	FOXD3	SPP1	S1PR1	SPP1_S1PR1
3412	FOXD3	SPP1	CD44	SPP1_CD44
3413	FOXD3	SPP1	ITGA5	SPP1_ITGA5
3414	FOXD3	SPP1	ITGB1	SPP1_ITGB1
3415	FOXD3	SPP1	ITGAV	SPP1_ITGAV
3416	FOXD3	SPP1	ITGA4	SPP1_ITGA4
3417	FOXD3	SPP1	ITGA9	SPP1_ITGA9
3418	HTATIP2	SPP1	S1PR1	SPP1_S1PR1
3419	HTATIP2	SPP1	CD44	SPP1_CD44
3420	HTATIP2	SPP1	ITGA5	SPP1_ITGA5
3421	HTATIP2	SPP1	ITGB1	SPP1_ITGB1

3422	HTATIP2	SPP1	ITGAV	SPP1_ITGAV
3423	HTATIP2	SPP1	ITGA4	SPP1_ITGA4
3424	HTATIP2	SPP1	ITGA9	SPP1_ITGA9
3425	CEBPA	SPP1	S1PR1	SPP1_S1PR1
3426	CEBPA	SPP1	CD44	SPP1_CD44
3427	CEBPA	SPP1	ITGA5	SPP1_ITGA5
3428	CEBPA	SPP1	ITGB1	SPP1_ITGB1
3429	CEBPA	SPP1	ITGAV	SPP1_ITGAV
3430	CEBPA	SPP1	ITGA4	SPP1_ITGA4
3431	CEBPA	SPP1	ITGA9	SPP1_ITGA9
3432	POU2F1	SPP1	S1PR1	SPP1_S1PR1
3433	POU2F1	SPP1	CD44	SPP1_CD44
3434	POU2F1	SPP1	ITGA5	SPP1_ITGA5
3435	POU2F1	SPP1	ITGB1	SPP1_ITGB1
3436	POU2F1	SPP1	ITGAV	SPP1_ITGAV
3437	POU2F1	SPP1	ITGA4	SPP1_ITGA4
3438	POU2F1	SPP1	ITGA9	SPP1_ITGA9
3439	HDAC1	SPP1	S1PR1	SPP1_S1PR1
3440	HDAC1	SPP1	CD44	SPP1_CD44
3441	HDAC1	SPP1	ITGA5	SPP1_ITGA5
3442	HDAC1	SPP1	ITGB1	SPP1_ITGB1
3443	HDAC1	SPP1	ITGAV	SPP1_ITGAV
3444	HDAC1	SPP1	ITGA4	SPP1_ITGA4
3445	HDAC1	SPP1	ITGA9	SPP1_ITGA9
3446	ING4	SPP1	S1PR1	SPP1_S1PR1
3447	ING4	SPP1	CD44	SPP1_CD44
3448	ING4	SPP1	ITGA5	SPP1_ITGA5
3449	ING4	SPP1	ITGB1	SPP1_ITGB1
3450	ING4	SPP1	ITGAV	SPP1_ITGAV
3451	ING4	SPP1	ITGA4	SPP1_ITGA4
3452	ING4	SPP1	ITGA9	SPP1_ITGA9
3453	SP1	SPP1	S1PR1	SPP1_S1PR1
3454	SP1	SPP1	CD44	SPP1_CD44
3455	SP1	SPP1	ITGA5	SPP1_ITGA5
3456	SP1	SPP1	ITGB1	SPP1_ITGB1
3457	SP1	SPP1	ITGAV	SPP1_ITGAV
3458	SP1	SPP1	ITGA4	SPP1_ITGA4
3459	SP1	SPP1	ITGA9	SPP1_ITGA9
3460	POU2F2	SPP1	S1PR1	SPP1_S1PR1
3461	POU2F2	SPP1	CD44	SPP1_CD44
3462	POU2F2	SPP1	ITGA5	SPP1_ITGA5
3463	POU2F2	SPP1	ITGB1	SPP1_ITGB1
3464	POU2F2	SPP1	ITGAV	SPP1_ITGAV
3465	POU2F2	SPP1	ITGA4	SPP1_ITGA4
3466	POU2F2	SPP1	ITGA9	SPP1_ITGA9
3467	ERG	SPP1	S1PR1	SPP1_S1PR1
3468	ERG	SPP1	CD44	SPP1_CD44
3469	ERG	SPP1	ITGA5	SPP1_ITGA5
3470	ERG	SPP1	ITGB1	SPP1_ITGB1
3471	ERG	SPP1	ITGAV	SPP1_ITGAV
3472	ERG	SPP1	ITGA4	SPP1_ITGA4
3473	ERG	SPP1	ITGA9	SPP1_ITGA9
3474	USF2	TCN2	LRP2	TCN2_LRP2
3475	USF2	TCN2	CNR1	TCN2_CNR1
3476	USF1	TCN2	LRP2	TCN2_LRP2
3477	USF1	TCN2	CNR1	TCN2_CNR1
3478	SP1	TCN2	LRP2	TCN2_LRP2
3479	SP1	TCN2	CNR1	TCN2_CNR1

3480	SP3	TCN2	LRP2	TCN2_LRP2
3481	SP3	TCN2	CNR1	TCN2_CNR1
3482	PAX8	TG	ASGR1	TG_ASGR1
3483	KCNIP3	TG	ASGR1	TG_ASGR1
3484	PPARG	TG	ASGR1	TG_ASGR1
3485	ESR1	TGFA	EGFR	TGFA_EGFR
3486	ESR1	TGFA	ERBB3	TGFA_ERBB3
3487	ESR2	TGFA	EGFR	TGFA_EGFR
3488	ESR2	TGFA	ERBB3	TGFA_ERBB3
3489	SP1	TGFA	EGFR	TGFA_EGFR
3490	SP1	TGFA	ERBB3	TGFA_ERBB3
3491	AR	TGFA	EGFR	TGFA_EGFR
3492	AR	TGFA	ERBB3	TGFA_ERBB3
3493	FOSL2	TGFB1	CXCR4	TGFB1_CXCR4
3494	FOSL2	TGFB1	CAV1	TGFB1_CAV1
3495	FOSL2	TGFB1	TGFBR1	TGFB1_TGFBR1
3496	FOSL2	TGFB1	ITGB8	TGFB1_ITGB8
3497	NFKB1	TGFB1	CXCR4	TGFB1_CXCR4
3498	NFKB1	TGFB1	CAV1	TGFB1_CAV1
3499	NFKB1	TGFB1	TGFBR1	TGFB1_TGFBR1
3500	NFKB1	TGFB1	ITGB8	TGFB1_ITGB8
3501	USF2	TGFB1	CXCR4	TGFB1_CXCR4
3502	USF2	TGFB1	CAV1	TGFB1_CAV1
3503	USF2	TGFB1	TGFBR1	TGFB1_TGFBR1
3504	USF2	TGFB1	ITGB8	TGFB1_ITGB8
3505	SP1	TGFB1	CXCR4	TGFB1_CXCR4
3506	SP1	TGFB1	CAV1	TGFB1_CAV1
3507	SP1	TGFB1	TGFBR1	TGFB1_TGFBR1
3508	SP1	TGFB1	ITGB8	TGFB1_ITGB8
3509	STAT3	TGFB1	CXCR4	TGFB1_CXCR4
3510	STAT3	TGFB1	CAV1	TGFB1_CAV1
3511	STAT3	TGFB1	TGFBR1	TGFB1_TGFBR1
3512	STAT3	TGFB1	ITGB8	TGFB1_ITGB8
3513	FOSB	TGFB1	CXCR4	TGFB1_CXCR4
3514	FOSB	TGFB1	CAV1	TGFB1_CAV1
3515	FOSB	TGFB1	TGFBR1	TGFB1_TGFBR1
3516	FOSB	TGFB1	ITGB8	TGFB1_ITGB8
3517	SMAD4	TGFB1	CXCR4	TGFB1_CXCR4
3518	SMAD4	TGFB1	CAV1	TGFB1_CAV1
3519	SMAD4	TGFB1	TGFBR1	TGFB1_TGFBR1
3520	SMAD4	TGFB1	ITGB8	TGFB1_ITGB8
3521	SMAD3	TGFB1	CXCR4	TGFB1_CXCR4
3522	SMAD3	TGFB1	CAV1	TGFB1_CAV1
3523	SMAD3	TGFB1	TGFBR1	TGFB1_TGFBR1
3524	SMAD3	TGFB1	ITGB8	TGFB1_ITGB8
3525	SMAD7	TGFB1	CXCR4	TGFB1_CXCR4
3526	SMAD7	TGFB1	CAV1	TGFB1_CAV1
3527	SMAD7	TGFB1	TGFBR1	TGFB1_TGFBR1
3528	SMAD7	TGFB1	ITGB8	TGFB1_ITGB8
3529	ASH1L	TGFB1	CXCR4	TGFB1_CXCR4
3530	ASH1L	TGFB1	CAV1	TGFB1_CAV1
3531	ASH1L	TGFB1	TGFBR1	TGFB1_TGFBR1
3532	ASH1L	TGFB1	ITGB8	TGFB1_ITGB8
3533	USF1	TGFB1	CXCR4	TGFB1_CXCR4
3534	USF1	TGFB1	CAV1	TGFB1_CAV1
3535	USF1	TGFB1	TGFBR1	TGFB1_TGFBR1
3536	USF1	TGFB1	ITGB8	TGFB1_ITGB8
3537	JUND	TGFB1	CXCR4	TGFB1_CXCR4

3538	JUND	TGFB1	CAV1	TGFB1_CAV1
3539	JUND	TGFB1	TGFBR1	TGFB1_TGFBR1
3540	JUND	TGFB1	ITGB8	TGFB1_ITGB8
3541	ATF1	TGFB2	TGFBR1	TGFB2_TGFBR1
3542	ATF2	TGFB2	TGFBR1	TGFB2_TGFBR1
3543	USF2	TGFB2	TGFBR1	TGFB2_TGFBR1
3544	USF1	TGFB2	TGFBR1	TGFB2_TGFBR1
3545	HIF1A	TGFB3	TGFBR1	TGFB3_TGFBR1
3546	SNAI1	THBS1	CD36	THBS1_CD36
3547	SNAI1	THBS1	CD47	THBS1_CD47
3548	SNAI1	THBS1	SDC1	THBS1_SDC1
3549	SNAI1	THBS1	SDC4	THBS1_SDC4
3550	SNAI1	THBS1	TNFRSF11B	THBS1_TNFRSF11B
3551	SNAI1	THBS1	ITGA6	THBS1_ITGA6
3552	SNAI1	THBS1	ITGA3	THBS1_ITGA3
3553	SNAI1	THBS1	SCARB1	THBS1_SCARB1
3554	SNAI1	THBS1	ITGA4	THBS1_ITGA4
3555	SNAI1	THBS1	ITGB3	THBS1_ITGB3
3556	SNAI1	THBS1	LRP1	THBS1_LRP1
3557	SNAI1	THBS1	ITGA2B	THBS1_ITGA2B
3558	SNAI1	THBS1	ITGB1	THBS1_ITGB1
3559	SNAI1	THBS1	LRP5	THBS1_LRP5
3560	E2F1	THBS1	CD36	THBS1_CD36
3561	E2F1	THBS1	CD47	THBS1_CD47
3562	E2F1	THBS1	SDC1	THBS1_SDC1
3563	E2F1	THBS1	SDC4	THBS1_SDC4
3564	E2F1	THBS1	TNFRSF11B	THBS1_TNFRSF11B
3565	E2F1	THBS1	ITGA6	THBS1_ITGA6
3566	E2F1	THBS1	ITGA3	THBS1_ITGA3
3567	E2F1	THBS1	SCARB1	THBS1_SCARB1
3568	E2F1	THBS1	ITGA4	THBS1_ITGA4
3569	E2F1	THBS1	ITGB3	THBS1_ITGB3
3570	E2F1	THBS1	LRP1	THBS1_LRP1
3571	E2F1	THBS1	ITGA2B	THBS1_ITGA2B
3572	E2F1	THBS1	ITGB1	THBS1_ITGB1
3573	E2F1	THBS1	LRP5	THBS1_LRP5
3574	TP53	THBS1	CD36	THBS1_CD36
3575	TP53	THBS1	CD47	THBS1_CD47
3576	TP53	THBS1	SDC1	THBS1_SDC1
3577	TP53	THBS1	SDC4	THBS1_SDC4
3578	TP53	THBS1	TNFRSF11B	THBS1_TNFRSF11B
3579	TP53	THBS1	ITGA6	THBS1_ITGA6
3580	TP53	THBS1	ITGA3	THBS1_ITGA3
3581	TP53	THBS1	SCARB1	THBS1_SCARB1
3582	TP53	THBS1	ITGA4	THBS1_ITGA4
3583	TP53	THBS1	ITGB3	THBS1_ITGB3
3584	TP53	THBS1	LRP1	THBS1_LRP1
3585	TP53	THBS1	ITGA2B	THBS1_ITGA2B
3586	TP53	THBS1	ITGB1	THBS1_ITGB1
3587	TP53	THBS1	LRP5	THBS1_LRP5
3588	WT1	THBS1	CD36	THBS1_CD36
3589	WT1	THBS1	CD47	THBS1_CD47
3590	WT1	THBS1	SDC1	THBS1_SDC1
3591	WT1	THBS1	SDC4	THBS1_SDC4
3592	WT1	THBS1	TNFRSF11B	THBS1_TNFRSF11B
3593	WT1	THBS1	ITGA6	THBS1_ITGA6
3594	WT1	THBS1	ITGA3	THBS1_ITGA3
3595	WT1	THBS1	SCARB1	THBS1_SCARB1

3596	WT1	THBS1	ITGA4	THBS1_ITGA4
3597	WT1	THBS1	ITGB3	THBS1_ITGB3
3598	WT1	THBS1	LRP1	THBS1_LRP1
3599	WT1	THBS1	ITGA2B	THBS1_ITGA2B
3600	WT1	THBS1	ITGB1	THBS1_ITGB1
3601	WT1	THBS1	LRP5	THBS1_LRP5
3602	USF2	THBS1	CD36	THBS1_CD36
3603	USF2	THBS1	CD47	THBS1_CD47
3604	USF2	THBS1	SDC1	THBS1_SDC1
3605	USF2	THBS1	SDC4	THBS1_SDC4
3606	USF2	THBS1	TNFRSF11B	THBS1_TNFRSF11B
3607	USF2	THBS1	ITGA6	THBS1_ITGA6
3608	USF2	THBS1	ITGA3	THBS1_ITGA3
3609	USF2	THBS1	SCARB1	THBS1_SCARB1
3610	USF2	THBS1	ITGA4	THBS1_ITGA4
3611	USF2	THBS1	ITGB3	THBS1_ITGB3
3612	USF2	THBS1	LRP1	THBS1_LRP1
3613	USF2	THBS1	ITGA2B	THBS1_ITGA2B
3614	USF2	THBS1	ITGB1	THBS1_ITGB1
3615	USF2	THBS1	LRP5	THBS1_LRP5
3616	NR1I2	THBS1	CD36	THBS1_CD36
3617	NR1I2	THBS1	CD47	THBS1_CD47
3618	NR1I2	THBS1	SDC1	THBS1_SDC1
3619	NR1I2	THBS1	SDC4	THBS1_SDC4
3620	NR1I2	THBS1	TNFRSF11B	THBS1_TNFRSF11B
3621	NR1I2	THBS1	ITGA6	THBS1_ITGA6
3622	NR1I2	THBS1	ITGA3	THBS1_ITGA3
3623	NR1I2	THBS1	SCARB1	THBS1_SCARB1
3624	NR1I2	THBS1	ITGA4	THBS1_ITGA4
3625	NR1I2	THBS1	ITGB3	THBS1_ITGB3
3626	NR1I2	THBS1	LRP1	THBS1_LRP1
3627	NR1I2	THBS1	ITGA2B	THBS1_ITGA2B
3628	NR1I2	THBS1	ITGB1	THBS1_ITGB1
3629	NR1I2	THBS1	LRP5	THBS1_LRP5
3630	HOXB7	THBS2	ITGA4	THBS2_ITGA4
3631	HOXB7	THBS2	CD47	THBS2_CD47
3632	HOXB7	THBS2	ITGB1	THBS2_ITGB1
3633	FOSB	TIMP1	CD63	TIMP1_CD63
3634	STAT3	TIMP1	CD63	TIMP1_CD63
3635	TWIST1	TIMP1	CD63	TIMP1_CD63
3636	SP3	TIMP1	CD63	TIMP1_CD63
3637	SP1	TIMP1	CD63	TIMP1_CD63
3638	FOSL2	TIMP1	CD63	TIMP1_CD63
3639	RELA	TIMP1	CD63	TIMP1_CD63
3640	PPARD	TIMP1	CD63	TIMP1_CD63
3641	JUND	TIMP1	CD63	TIMP1_CD63
3642	PAX5	TIMP1	CD63	TIMP1_CD63
3643	NFKB1	TIMP1	CD63	TIMP1_CD63
3644	RUNX3	TIMP1	CD63	TIMP1_CD63
3645	STAT1	TIMP1	CD63	TIMP1_CD63
3646	RUNX1	TIMP1	CD63	TIMP1_CD63
3647	ASH1L	TIMP1	CD63	TIMP1_CD63
3648	NR4A1	TIMP2	ITGA3	TIMP2_ITGA3
3649	NR4A1	TIMP2	ITGB1	TIMP2_ITGB1
3650	PTTG1	TIMP2	ITGA3	TIMP2_ITGA3
3651	PTTG1	TIMP2	ITGB1	TIMP2_ITGB1
3652	JUN	TIMP2	ITGA3	TIMP2_ITGA3
3653	JUN	TIMP2	ITGB1	TIMP2_ITGB1

3654	SP1	TIMP2	ITGA3	TIMP2_ITGA3
3655	SP1	TIMP2	ITGB1	TIMP2_ITGB1
3656	ETV4	TIMP2	ITGA3	TIMP2_ITGA3
3657	ETV4	TIMP2	ITGB1	TIMP2_ITGB1
3658	SP3	TIMP2	ITGA3	TIMP2_ITGA3
3659	SP3	TIMP2	ITGB1	TIMP2_ITGB1
3660	HIF1A	TIMP2	ITGA3	TIMP2_ITGA3
3661	HIF1A	TIMP2	ITGB1	TIMP2_ITGB1
3662	TFAP2A	TIMP2	ITGA3	TIMP2_ITGA3
3663	TFAP2A	TIMP2	ITGB1	TIMP2_ITGB1
3664	SMAD3	TNC	NT5E	TNC_NT5E
3665	SMAD3	TNC	EGFR	TNC_EGFR
3666	SMAD3	TNC	SDC1	TNC_SDC1
3667	SMAD3	TNC	SDC4	TNC_SDC4
3668	SMAD3	TNC	ITGB1	TNC_ITGB1
3669	NFKB1	TNC	NT5E	TNC_NT5E
3670	NFKB1	TNC	EGFR	TNC_EGFR
3671	NFKB1	TNC	SDC1	TNC_SDC1
3672	NFKB1	TNC	SDC4	TNC_SDC4
3673	NFKB1	TNC	ITGB1	TNC_ITGB1
3674	SP1	TNC	NT5E	TNC_NT5E
3675	SP1	TNC	EGFR	TNC_EGFR
3676	SP1	TNC	SDC1	TNC_SDC1
3677	SP1	TNC	SDC4	TNC_SDC4
3678	SP1	TNC	ITGB1	TNC_ITGB1
3679	ETS2	TNC	NT5E	TNC_NT5E
3680	ETS2	TNC	EGFR	TNC_EGFR
3681	ETS2	TNC	SDC1	TNC_SDC1
3682	ETS2	TNC	SDC4	TNC_SDC4
3683	ETS2	TNC	ITGB1	TNC_ITGB1
3684	RELA	TNC	NT5E	TNC_NT5E
3685	RELA	TNC	EGFR	TNC_EGFR
3686	RELA	TNC	SDC1	TNC_SDC1
3687	RELA	TNC	SDC4	TNC_SDC4
3688	RELA	TNC	ITGB1	TNC_ITGB1
3689	ETS1	TNC	NT5E	TNC_NT5E
3690	ETS1	TNC	EGFR	TNC_EGFR
3691	ETS1	TNC	SDC1	TNC_SDC1
3692	ETS1	TNC	SDC4	TNC_SDC4
3693	ETS1	TNC	ITGB1	TNC_ITGB1
3694	JUN	TNC	NT5E	TNC_NT5E
3695	JUN	TNC	EGFR	TNC_EGFR
3696	JUN	TNC	SDC1	TNC_SDC1
3697	JUN	TNC	SDC4	TNC_SDC4
3698	JUN	TNC	ITGB1	TNC_ITGB1
3699	SMAD4	TNC	NT5E	TNC_NT5E
3700	SMAD4	TNC	EGFR	TNC_EGFR
3701	SMAD4	TNC	SDC1	TNC_SDC1
3702	SMAD4	TNC	SDC4	TNC_SDC4
3703	SMAD4	TNC	ITGB1	TNC_ITGB1
3704	ETV4	TNF	TNFRSF1B	TNF_TNFRSF1B
3705	ETV4	TNF	LTBR	TNF_LTBR
3706	SP1	TNF	TNFRSF1B	TNF_TNFRSF1B
3707	SP1	TNF	LTBR	TNF_LTBR
3708	SIRT1	TNF	TNFRSF1B	TNF_TNFRSF1B
3709	SIRT1	TNF	LTBR	TNF_LTBR
3710	LRRFIP1	TNF	TNFRSF1B	TNF_TNFRSF1B
3711	LRRFIP1	TNF	LTBR	TNF_LTBR

3712	CEBPD	TNF	TNFRSF1B	TNF_TNFRSF1B
3713	CEBPD	TNF	LTBR	TNF_LTBR
3714	NFAT5	TNF	TNFRSF1B	TNF_TNFRSF1B
3715	NFAT5	TNF	LTBR	TNF_LTBR
3716	ATF2	TNF	TNFRSF1B	TNF_TNFRSF1B
3717	ATF2	TNF	LTBR	TNF_LTBR
3718	RELA	TNF	TNFRSF1B	TNF_TNFRSF1B
3719	RELA	TNF	LTBR	TNF_LTBR
3720	SPI1	TNF	TNFRSF1B	TNF_TNFRSF1B
3721	SPI1	TNF	LTBR	TNF_LTBR
3722	NR4A1	TNF	TNFRSF1B	TNF_TNFRSF1B
3723	NR4A1	TNF	LTBR	TNF_LTBR
3724	CEBPB	TNF	TNFRSF1B	TNF_TNFRSF1B
3725	CEBPB	TNF	LTBR	TNF_LTBR
3726	JUN	TNF	TNFRSF1B	TNF_TNFRSF1B
3727	JUN	TNF	LTBR	TNF_LTBR
3728	EGR1	TNF	TNFRSF1B	TNF_TNFRSF1B
3729	EGR1	TNF	LTBR	TNF_LTBR
3730	HMGB2	TNF	TNFRSF1B	TNF_TNFRSF1B
3731	HMGB2	TNF	LTBR	TNF_LTBR
3732	E2F1	TNF	TNFRSF1B	TNF_TNFRSF1B
3733	E2F1	TNF	LTBR	TNF_LTBR
3734	HDAC3	TNF	TNFRSF1B	TNF_TNFRSF1B
3735	HDAC3	TNF	LTBR	TNF_LTBR
3736	HDAC11	TNF	TNFRSF1B	TNF_TNFRSF1B
3737	HDAC11	TNF	LTBR	TNF_LTBR
3738	NFKB1	TNF	TNFRSF1B	TNF_TNFRSF1B
3739	NFKB1	TNF	LTBR	TNF_LTBR
3740	IRF5	TNF	TNFRSF1B	TNF_TNFRSF1B
3741	IRF5	TNF	LTBR	TNF_LTBR
3742	HSF1	TNF	TNFRSF1B	TNF_TNFRSF1B
3743	HSF1	TNF	LTBR	TNF_LTBR
3744	RELA	TNFSF10	TNFRSF10A	TNFSF10_TNFRSF10A
3745	RELA	TNFSF10	TNFRSF10D	TNFSF10_TNFRSF10D
3746	RELA	TNFSF10	TNFRSF10B	TNFSF10_TNFRSF10B
3747	RELA	TNFSF10	TNFRSF10C	TNFSF10_TNFRSF10C
3748	RELA	TNFSF10	TNFRSF11B	TNFSF10_TNFRSF11B
3749	NFKB1	TNFSF10	TNFRSF10A	TNFSF10_TNFRSF10A
3750	NFKB1	TNFSF10	TNFRSF10D	TNFSF10_TNFRSF10D
3751	NFKB1	TNFSF10	TNFRSF10B	TNFSF10_TNFRSF10B
3752	NFKB1	TNFSF10	TNFRSF10C	TNFSF10_TNFRSF10C
3753	NFKB1	TNFSF10	TNFRSF11B	TNFSF10_TNFRSF11B
3754	FOXO4	TNFSF10	TNFRSF10A	TNFSF10_TNFRSF10A
3755	FOXO4	TNFSF10	TNFRSF10D	TNFSF10_TNFRSF10D
3756	FOXO4	TNFSF10	TNFRSF10B	TNFSF10_TNFRSF10B
3757	FOXO4	TNFSF10	TNFRSF10C	TNFSF10_TNFRSF10C
3758	FOXO4	TNFSF10	TNFRSF11B	TNFSF10_TNFRSF11B
3759	SP1	TNFSF10	TNFRSF10A	TNFSF10_TNFRSF10A
3760	SP1	TNFSF10	TNFRSF10D	TNFSF10_TNFRSF10D
3761	SP1	TNFSF10	TNFRSF10B	TNFSF10_TNFRSF10B
3762	SP1	TNFSF10	TNFRSF10C	TNFSF10_TNFRSF10C
3763	SP1	TNFSF10	TNFRSF11B	TNFSF10_TNFRSF11B
3764	NFIL3	TNFSF10	TNFRSF10A	TNFSF10_TNFRSF10A
3765	NFIL3	TNFSF10	TNFRSF10D	TNFSF10_TNFRSF10D
3766	NFIL3	TNFSF10	TNFRSF10B	TNFSF10_TNFRSF10B
3767	NFIL3	TNFSF10	TNFRSF10C	TNFSF10_TNFRSF10C
3768	NFIL3	TNFSF10	TNFRSF11B	TNFSF10_TNFRSF11B
3769	EGR1	TNFSF10	TNFRSF10A	TNFSF10_TNFRSF10A

3770	EGR1	TNFSF10	TNFRSF10D	TNFSF10_TNFRSF10D
3771	EGR1	TNFSF10	TNFRSF10B	TNFSF10_TNFRSF10B
3772	EGR1	TNFSF10	TNFRSF10C	TNFSF10_TNFRSF10C
3773	EGR1	TNFSF10	TNFRSF11B	TNFSF10_TNFRSF11B
3774	FOXO3	TNFSF10	TNFRSF10A	TNFSF10_TNFRSF10A
3775	FOXO3	TNFSF10	TNFRSF10D	TNFSF10_TNFRSF10D
3776	FOXO3	TNFSF10	TNFRSF10B	TNFSF10_TNFRSF10B
3777	FOXO3	TNFSF10	TNFRSF10C	TNFSF10_TNFRSF10C
3778	FOXO3	TNFSF10	TNFRSF11B	TNFSF10_TNFRSF11B
3779	HDAC2	TNFSF10	TNFRSF10A	TNFSF10_TNFRSF10A
3780	HDAC2	TNFSF10	TNFRSF10D	TNFSF10_TNFRSF10D
3781	HDAC2	TNFSF10	TNFRSF10B	TNFSF10_TNFRSF10B
3782	HDAC2	TNFSF10	TNFRSF10C	TNFSF10_TNFRSF10C
3783	HDAC2	TNFSF10	TNFRSF11B	TNFSF10_TNFRSF11B
3784	FOXO1	TNFSF10	TNFRSF10A	TNFSF10_TNFRSF10A
3785	FOXO1	TNFSF10	TNFRSF10D	TNFSF10_TNFRSF10D
3786	FOXO1	TNFSF10	TNFRSF10B	TNFSF10_TNFRSF10B
3787	FOXO1	TNFSF10	TNFRSF10C	TNFSF10_TNFRSF10C
3788	FOXO1	TNFSF10	TNFRSF11B	TNFSF10_TNFRSF11B
3789	PML	TNFSF10	TNFRSF10A	TNFSF10_TNFRSF10A
3790	PML	TNFSF10	TNFRSF10D	TNFSF10_TNFRSF10D
3791	PML	TNFSF10	TNFRSF10B	TNFSF10_TNFRSF10B
3792	PML	TNFSF10	TNFRSF10C	TNFSF10_TNFRSF10C
3793	PML	TNFSF10	TNFRSF11B	TNFSF10_TNFRSF11B
3794	NFKBIA	TNFSF10	TNFRSF10A	TNFSF10_TNFRSF10A
3795	NFKBIA	TNFSF10	TNFRSF10D	TNFSF10_TNFRSF10D
3796	NFKBIA	TNFSF10	TNFRSF10B	TNFSF10_TNFRSF10B
3797	NFKBIA	TNFSF10	TNFRSF10C	TNFSF10_TNFRSF10C
3798	NFKBIA	TNFSF10	TNFRSF11B	TNFSF10_TNFRSF11B
3799	IRF1	TNFSF10	TNFRSF10A	TNFSF10_TNFRSF10A
3800	IRF1	TNFSF10	TNFRSF10D	TNFSF10_TNFRSF10D
3801	IRF1	TNFSF10	TNFRSF10B	TNFSF10_TNFRSF10B
3802	IRF1	TNFSF10	TNFRSF10C	TNFSF10_TNFRSF10C
3803	IRF1	TNFSF10	TNFRSF11B	TNFSF10_TNFRSF11B
3804	DACH1	TNFSF11	TNFRSF11B	TNFSF11_TNFRSF11B
3805	HSF2	TNFSF11	TNFRSF11B	TNFSF11_TNFRSF11B
3806	E2F1	TNFSF11	TNFRSF11B	TNFSF11_TNFRSF11B
3807	STAT1	TNFSF13B	TNFRSF13C	TNFSF13B_TNFRSF13C
3808	STAT1	TNFSF13B	TNFRSF17	TNFSF13B_TNFRSF17
3809	STAT1	TNFSF13B	TNFRSF13B	TNFSF13B_TNFRSF13B
3810	NFKB1	TNFSF13B	TNFRSF13C	TNFSF13B_TNFRSF13C
3811	NFKB1	TNFSF13B	TNFRSF17	TNFSF13B_TNFRSF17
3812	NFKB1	TNFSF13B	TNFRSF13B	TNFSF13B_TNFRSF13B
3813	NFKB1	TNFSF14	LTBR	TNFSF14_LTBR
3814	SP1	TNFSF14	LTBR	TNFSF14_LTBR
3815	RELA	TNFSF14	LTBR	TNFSF14_LTBR
3816	ETS1	TNFSF14	LTBR	TNFSF14_LTBR
3817	HDAC11	TNFSF4	TNFRSF4	TNFSF4_TNFRSF4
3818	NFKB1	TSLP	IL7R	TSLP_IL7R
3819	RELA	TSLP	IL7R	TSLP_IL7R
3820	JUN	VCAM1	ITGA9	VCAM1_ITGA9
3821	JUN	VCAM1	ITGA4	VCAM1_ITGA4
3822	JUN	VCAM1	ITGB7	VCAM1_ITGB7
3823	JUN	VCAM1	ITGAD	VCAM1_ITGAD
3824	JUN	VCAM1	ITGB1	VCAM1_ITGB1
3825	JUN	VCAM1	ITGB2	VCAM1_ITGB2
3826	HDAC4	VCAM1	ITGA9	VCAM1_ITGA9
3827	HDAC4	VCAM1	ITGA4	VCAM1_ITGA4

3828	HDAC4	VCAM1	ITGB7	VCAM1_ITGB7
3829	HDAC4	VCAM1	ITGAD	VCAM1_ITGAD
3830	HDAC4	VCAM1	ITGB1	VCAM1_ITGB1
3831	HDAC4	VCAM1	ITGB2	VCAM1_ITGB2
3832	IRF1	VCAM1	ITGA9	VCAM1_ITGA9
3833	IRF1	VCAM1	ITGA4	VCAM1_ITGA4
3834	IRF1	VCAM1	ITGB7	VCAM1_ITGB7
3835	IRF1	VCAM1	ITGAD	VCAM1_ITGAD
3836	IRF1	VCAM1	ITGB1	VCAM1_ITGB1
3837	IRF1	VCAM1	ITGB2	VCAM1_ITGB2
3838	RELA	VCAM1	ITGA9	VCAM1_ITGA9
3839	RELA	VCAM1	ITGA4	VCAM1_ITGA4
3840	RELA	VCAM1	ITGB7	VCAM1_ITGB7
3841	RELA	VCAM1	ITGAD	VCAM1_ITGAD
3842	RELA	VCAM1	ITGB1	VCAM1_ITGB1
3843	RELA	VCAM1	ITGB2	VCAM1_ITGB2
3844	NFKB1	VCAM1	ITGA9	VCAM1_ITGA9
3845	NFKB1	VCAM1	ITGA4	VCAM1_ITGA4
3846	NFKB1	VCAM1	ITGB7	VCAM1_ITGB7
3847	NFKB1	VCAM1	ITGAD	VCAM1_ITGAD
3848	NFKB1	VCAM1	ITGB1	VCAM1_ITGB1
3849	NFKB1	VCAM1	ITGB2	VCAM1_ITGB2
3850	POU2F1	VCAM1	ITGA9	VCAM1_ITGA9
3851	POU2F1	VCAM1	ITGA4	VCAM1_ITGA4
3852	POU2F1	VCAM1	ITGB7	VCAM1_ITGB7
3853	POU2F1	VCAM1	ITGAD	VCAM1_ITGAD
3854	POU2F1	VCAM1	ITGB1	VCAM1_ITGB1
3855	POU2F1	VCAM1	ITGB2	VCAM1_ITGB2
3856	IKBKB	VCAM1	ITGA9	VCAM1_ITGA9
3857	IKBKB	VCAM1	ITGA4	VCAM1_ITGA4
3858	IKBKB	VCAM1	ITGB7	VCAM1_ITGB7
3859	IKBKB	VCAM1	ITGAD	VCAM1_ITGAD
3860	IKBKB	VCAM1	ITGB1	VCAM1_ITGB1
3861	IKBKB	VCAM1	ITGB2	VCAM1_ITGB2
3862	HOXA9	VCAM1	ITGA9	VCAM1_ITGA9
3863	HOXA9	VCAM1	ITGA4	VCAM1_ITGA4
3864	HOXA9	VCAM1	ITGB7	VCAM1_ITGB7
3865	HOXA9	VCAM1	ITGAD	VCAM1_ITGAD
3866	HOXA9	VCAM1	ITGB1	VCAM1_ITGB1
3867	HOXA9	VCAM1	ITGB2	VCAM1_ITGB2
3868	EP300	VCAM1	ITGA9	VCAM1_ITGA9
3869	EP300	VCAM1	ITGA4	VCAM1_ITGA4
3870	EP300	VCAM1	ITGB7	VCAM1_ITGB7
3871	EP300	VCAM1	ITGAD	VCAM1_ITGAD
3872	EP300	VCAM1	ITGB1	VCAM1_ITGB1
3873	EP300	VCAM1	ITGB2	VCAM1_ITGB2
3874	TP53	VCAN	EGFR	VCAN_EGFR
3875	TP53	VCAN	ITGB1	VCAN_ITGB1
3876	TP53	VCAN	SELL	VCAN_SELL
3877	TP53	VCAN	SELP	VCAN_SELP
3878	TP53	VCAN	CD44	VCAN_CD44
3879	TP53	VCAN	ITGA4	VCAN_ITGA4
3880	TP53	VCAN	TLR2	VCAN_TLR2
3881	TP53	VCAN	TLR1	VCAN_TLR1
3882	TCF4	VCAN	EGFR	VCAN_EGFR
3883	TCF4	VCAN	ITGB1	VCAN_ITGB1
3884	TCF4	VCAN	SELL	VCAN_SELL
3885	TCF4	VCAN	SELP	VCAN_SELP

3886	TCF4	VCAN	CD44	VCAN_CD44
3887	TCF4	VCAN	ITGA4	VCAN_ITGA4
3888	TCF4	VCAN	TLR2	VCAN_TLR2
3889	TCF4	VCAN	TLR1	VCAN_TLR1
3890	HDAC4	VEGFA	NRP2	VEGFA_NRP2
3891	HDAC4	VEGFA	RET	VEGFA_RET
3892	HDAC4	VEGFA	NRP1	VEGFA_NRP1
3893	HDAC4	VEGFA	EGFR	VEGFA_EGFR
3894	HDAC4	VEGFA	EPHB2	VEGFA_EPHB2
3895	HDAC4	VEGFA	ITGB1	VEGFA_ITGB1
3896	HDAC4	VEGFA	KDR	VEGFA_KDR
3897	HDAC4	VEGFA	TYRO3	VEGFA_TYRO3
3898	HDAC4	VEGFA	ITGA9	VEGFA_ITGA9
3899	HDAC4	VEGFA	ITGAV	VEGFA_ITGAV
3900	HDAC4	VEGFA	SIRPA	VEGFA_SIRPA
3901	HDAC4	VEGFA	GPC1	VEGFA_GPC1
3902	HDAC4	VEGFA	FLT1	VEGFA_FLT1
3903	RB1	VEGFA	NRP2	VEGFA_NRP2
3904	RB1	VEGFA	RET	VEGFA_RET
3905	RB1	VEGFA	NRP1	VEGFA_NRP1
3906	RB1	VEGFA	EGFR	VEGFA_EGFR
3907	RB1	VEGFA	EPHB2	VEGFA_EPHB2
3908	RB1	VEGFA	ITGB1	VEGFA_ITGB1
3909	RB1	VEGFA	KDR	VEGFA_KDR
3910	RB1	VEGFA	TYRO3	VEGFA_TYRO3
3911	RB1	VEGFA	ITGA9	VEGFA_ITGA9
3912	RB1	VEGFA	ITGAV	VEGFA_ITGAV
3913	RB1	VEGFA	SIRPA	VEGFA_SIRPA
3914	RB1	VEGFA	GPC1	VEGFA_GPC1
3915	RB1	VEGFA	FLT1	VEGFA_FLT1
3916	RELA	VEGFA	NRP2	VEGFA_NRP2
3917	RELA	VEGFA	RET	VEGFA_RET
3918	RELA	VEGFA	NRP1	VEGFA_NRP1
3919	RELA	VEGFA	EGFR	VEGFA_EGFR
3920	RELA	VEGFA	EPHB2	VEGFA_EPHB2
3921	RELA	VEGFA	ITGB1	VEGFA_ITGB1
3922	RELA	VEGFA	KDR	VEGFA_KDR
3923	RELA	VEGFA	TYRO3	VEGFA_TYRO3
3924	RELA	VEGFA	ITGA9	VEGFA_ITGA9
3925	RELA	VEGFA	ITGAV	VEGFA_ITGAV
3926	RELA	VEGFA	SIRPA	VEGFA_SIRPA
3927	RELA	VEGFA	GPC1	VEGFA_GPC1
3928	RELA	VEGFA	FLT1	VEGFA_FLT1
3929	TEAD4	VEGFA	NRP2	VEGFA_NRP2
3930	TEAD4	VEGFA	RET	VEGFA_RET
3931	TEAD4	VEGFA	NRP1	VEGFA_NRP1
3932	TEAD4	VEGFA	EGFR	VEGFA_EGFR
3933	TEAD4	VEGFA	EPHB2	VEGFA_EPHB2
3934	TEAD4	VEGFA	ITGB1	VEGFA_ITGB1
3935	TEAD4	VEGFA	KDR	VEGFA_KDR
3936	TEAD4	VEGFA	TYRO3	VEGFA_TYRO3
3937	TEAD4	VEGFA	ITGA9	VEGFA_ITGA9
3938	TEAD4	VEGFA	ITGAV	VEGFA_ITGAV
3939	TEAD4	VEGFA	SIRPA	VEGFA_SIRPA
3940	TEAD4	VEGFA	GPC1	VEGFA_GPC1
3941	TEAD4	VEGFA	FLT1	VEGFA_FLT1
3942	HDAC3	VEGFA	NRP2	VEGFA_NRP2
3943	HDAC3	VEGFA	RET	VEGFA_RET

3944	HDAC3	VEGFA	NRP1	VEGFA_NRP1
3945	HDAC3	VEGFA	EGFR	VEGFA_EGFR
3946	HDAC3	VEGFA	EPHB2	VEGFA_EPHB2
3947	HDAC3	VEGFA	ITGB1	VEGFA_ITGB1
3948	HDAC3	VEGFA	KDR	VEGFA_KDR
3949	HDAC3	VEGFA	TYRO3	VEGFA_TYRO3
3950	HDAC3	VEGFA	ITGA9	VEGFA_ITGA9
3951	HDAC3	VEGFA	ITGAV	VEGFA_ITGAV
3952	HDAC3	VEGFA	SIRPA	VEGFA_SIRPA
3953	HDAC3	VEGFA	GPC1	VEGFA_GPC1
3954	HDAC3	VEGFA	FLT1	VEGFA_FLT1
3955	ATM	VEGFA	NRP2	VEGFA_NRP2
3956	ATM	VEGFA	RET	VEGFA_RET
3957	ATM	VEGFA	NRP1	VEGFA_NRP1
3958	ATM	VEGFA	EGFR	VEGFA_EGFR
3959	ATM	VEGFA	EPHB2	VEGFA_EPHB2
3960	ATM	VEGFA	ITGB1	VEGFA_ITGB1
3961	ATM	VEGFA	KDR	VEGFA_KDR
3962	ATM	VEGFA	TYRO3	VEGFA_TYRO3
3963	ATM	VEGFA	ITGA9	VEGFA_ITGA9
3964	ATM	VEGFA	ITGAV	VEGFA_ITGAV
3965	ATM	VEGFA	SIRPA	VEGFA_SIRPA
3966	ATM	VEGFA	GPC1	VEGFA_GPC1
3967	ATM	VEGFA	FLT1	VEGFA_FLT1
3968	VHL	VEGFA	NRP2	VEGFA_NRP2
3969	VHL	VEGFA	RET	VEGFA_RET
3970	VHL	VEGFA	NRP1	VEGFA_NRP1
3971	VHL	VEGFA	EGFR	VEGFA_EGFR
3972	VHL	VEGFA	EPHB2	VEGFA_EPHB2
3973	VHL	VEGFA	ITGB1	VEGFA_ITGB1
3974	VHL	VEGFA	KDR	VEGFA_KDR
3975	VHL	VEGFA	TYRO3	VEGFA_TYRO3
3976	VHL	VEGFA	ITGA9	VEGFA_ITGA9
3977	VHL	VEGFA	ITGAV	VEGFA_ITGAV
3978	VHL	VEGFA	SIRPA	VEGFA_SIRPA
3979	VHL	VEGFA	GPC1	VEGFA_GPC1
3980	VHL	VEGFA	FLT1	VEGFA_FLT1
3981	E2F3	VEGFA	NRP2	VEGFA_NRP2
3982	E2F3	VEGFA	RET	VEGFA_RET
3983	E2F3	VEGFA	NRP1	VEGFA_NRP1
3984	E2F3	VEGFA	EGFR	VEGFA_EGFR
3985	E2F3	VEGFA	EPHB2	VEGFA_EPHB2
3986	E2F3	VEGFA	ITGB1	VEGFA_ITGB1
3987	E2F3	VEGFA	KDR	VEGFA_KDR
3988	E2F3	VEGFA	TYRO3	VEGFA_TYRO3
3989	E2F3	VEGFA	ITGA9	VEGFA_ITGA9
3990	E2F3	VEGFA	ITGAV	VEGFA_ITGAV
3991	E2F3	VEGFA	SIRPA	VEGFA_SIRPA
3992	E2F3	VEGFA	GPC1	VEGFA_GPC1
3993	E2F3	VEGFA	FLT1	VEGFA_FLT1
3994	ATF4	VEGFA	NRP2	VEGFA_NRP2
3995	ATF4	VEGFA	RET	VEGFA_RET
3996	ATF4	VEGFA	NRP1	VEGFA_NRP1
3997	ATF4	VEGFA	EGFR	VEGFA_EGFR
3998	ATF4	VEGFA	EPHB2	VEGFA_EPHB2
3999	ATF4	VEGFA	ITGB1	VEGFA_ITGB1
4000	ATF4	VEGFA	KDR	VEGFA_KDR
4001	ATF4	VEGFA	TYRO3	VEGFA_TYRO3

4002	ATF4	VEGFA	ITGA9	VEGFA_ITGA9
4003	ATF4	VEGFA	ITGAV	VEGFA_ITGAV
4004	ATF4	VEGFA	SIRPA	VEGFA_SIRPA
4005	ATF4	VEGFA	GPC1	VEGFA_GPC1
4006	ATF4	VEGFA	FLT1	VEGFA_FLT1
4007	RUNX2	VEGFA	NRP2	VEGFA_NRP2
4008	RUNX2	VEGFA	RET	VEGFA_RET
4009	RUNX2	VEGFA	NRP1	VEGFA_NRP1
4010	RUNX2	VEGFA	EGFR	VEGFA_EGFR
4011	RUNX2	VEGFA	EPHB2	VEGFA_EPHB2
4012	RUNX2	VEGFA	ITGB1	VEGFA_ITGB1
4013	RUNX2	VEGFA	KDR	VEGFA_KDR
4014	RUNX2	VEGFA	TYRO3	VEGFA_TYRO3
4015	RUNX2	VEGFA	ITGA9	VEGFA_ITGA9
4016	RUNX2	VEGFA	ITGAV	VEGFA_ITGAV
4017	RUNX2	VEGFA	SIRPA	VEGFA_SIRPA
4018	RUNX2	VEGFA	GPC1	VEGFA_GPC1
4019	RUNX2	VEGFA	FLT1	VEGFA_FLT1
4020	TFAP2A	VEGFA	NRP2	VEGFA_NRP2
4021	TFAP2A	VEGFA	RET	VEGFA_RET
4022	TFAP2A	VEGFA	NRP1	VEGFA_NRP1
4023	TFAP2A	VEGFA	EGFR	VEGFA_EGFR
4024	TFAP2A	VEGFA	EPHB2	VEGFA_EPHB2
4025	TFAP2A	VEGFA	ITGB1	VEGFA_ITGB1
4026	TFAP2A	VEGFA	KDR	VEGFA_KDR
4027	TFAP2A	VEGFA	TYRO3	VEGFA_TYRO3
4028	TFAP2A	VEGFA	ITGA9	VEGFA_ITGA9
4029	TFAP2A	VEGFA	ITGAV	VEGFA_ITGAV
4030	TFAP2A	VEGFA	SIRPA	VEGFA_SIRPA
4031	TFAP2A	VEGFA	GPC1	VEGFA_GPC1
4032	TFAP2A	VEGFA	FLT1	VEGFA_FLT1
4033	FOXQ1	VEGFA	NRP2	VEGFA_NRP2
4034	FOXQ1	VEGFA	RET	VEGFA_RET
4035	FOXQ1	VEGFA	NRP1	VEGFA_NRP1
4036	FOXQ1	VEGFA	EGFR	VEGFA_EGFR
4037	FOXQ1	VEGFA	EPHB2	VEGFA_EPHB2
4038	FOXQ1	VEGFA	ITGB1	VEGFA_ITGB1
4039	FOXQ1	VEGFA	KDR	VEGFA_KDR
4040	FOXQ1	VEGFA	TYRO3	VEGFA_TYRO3
4041	FOXQ1	VEGFA	ITGA9	VEGFA_ITGA9
4042	FOXQ1	VEGFA	ITGAV	VEGFA_ITGAV
4043	FOXQ1	VEGFA	SIRPA	VEGFA_SIRPA
4044	FOXQ1	VEGFA	GPC1	VEGFA_GPC1
4045	FOXQ1	VEGFA	FLT1	VEGFA_FLT1
4046	HDGF	VEGFA	NRP2	VEGFA_NRP2
4047	HDGF	VEGFA	RET	VEGFA_RET
4048	HDGF	VEGFA	NRP1	VEGFA_NRP1
4049	HDGF	VEGFA	EGFR	VEGFA_EGFR
4050	HDGF	VEGFA	EPHB2	VEGFA_EPHB2
4051	HDGF	VEGFA	ITGB1	VEGFA_ITGB1
4052	HDGF	VEGFA	KDR	VEGFA_KDR
4053	HDGF	VEGFA	TYRO3	VEGFA_TYRO3
4054	HDGF	VEGFA	ITGA9	VEGFA_ITGA9
4055	HDGF	VEGFA	ITGAV	VEGFA_ITGAV
4056	HDGF	VEGFA	SIRPA	VEGFA_SIRPA
4057	HDGF	VEGFA	GPC1	VEGFA_GPC1
4058	HDGF	VEGFA	FLT1	VEGFA_FLT1
4059	WT1	VEGFA	NRP2	VEGFA_NRP2

4060	WT1	VEGFA	RET	VEGFA_RET
4061	WT1	VEGFA	NRP1	VEGFA_NRP1
4062	WT1	VEGFA	EGFR	VEGFA_EGFR
4063	WT1	VEGFA	EPHB2	VEGFA_EPHB2
4064	WT1	VEGFA	ITGB1	VEGFA_ITGB1
4065	WT1	VEGFA	KDR	VEGFA_KDR
4066	WT1	VEGFA	TYRO3	VEGFA_TYRO3
4067	WT1	VEGFA	ITGA9	VEGFA_ITGA9
4068	WT1	VEGFA	ITGAV	VEGFA_ITGAV
4069	WT1	VEGFA	SIRPA	VEGFA_SIRPA
4070	WT1	VEGFA	GPC1	VEGFA_GPC1
4071	WT1	VEGFA	FLT1	VEGFA_FLT1
4072	HOXB7	VEGFA	NRP2	VEGFA_NRP2
4073	HOXB7	VEGFA	RET	VEGFA_RET
4074	HOXB7	VEGFA	NRP1	VEGFA_NRP1
4075	HOXB7	VEGFA	EGFR	VEGFA_EGFR
4076	HOXB7	VEGFA	EPHB2	VEGFA_EPHB2
4077	HOXB7	VEGFA	ITGB1	VEGFA_ITGB1
4078	HOXB7	VEGFA	KDR	VEGFA_KDR
4079	HOXB7	VEGFA	TYRO3	VEGFA_TYRO3
4080	HOXB7	VEGFA	ITGA9	VEGFA_ITGA9
4081	HOXB7	VEGFA	ITGAV	VEGFA_ITGAV
4082	HOXB7	VEGFA	SIRPA	VEGFA_SIRPA
4083	HOXB7	VEGFA	GPC1	VEGFA_GPC1
4084	HOXB7	VEGFA	FLT1	VEGFA_FLT1
4085	ZFP36	VEGFA	NRP2	VEGFA_NRP2
4086	ZFP36	VEGFA	RET	VEGFA_RET
4087	ZFP36	VEGFA	NRP1	VEGFA_NRP1
4088	ZFP36	VEGFA	EGFR	VEGFA_EGFR
4089	ZFP36	VEGFA	EPHB2	VEGFA_EPHB2
4090	ZFP36	VEGFA	ITGB1	VEGFA_ITGB1
4091	ZFP36	VEGFA	KDR	VEGFA_KDR
4092	ZFP36	VEGFA	TYRO3	VEGFA_TYRO3
4093	ZFP36	VEGFA	ITGA9	VEGFA_ITGA9
4094	ZFP36	VEGFA	ITGAV	VEGFA_ITGAV
4095	ZFP36	VEGFA	SIRPA	VEGFA_SIRPA
4096	ZFP36	VEGFA	GPC1	VEGFA_GPC1
4097	ZFP36	VEGFA	FLT1	VEGFA_FLT1
4098	EPAS1	VEGFA	NRP2	VEGFA_NRP2
4099	EPAS1	VEGFA	RET	VEGFA_RET
4100	EPAS1	VEGFA	NRP1	VEGFA_NRP1
4101	EPAS1	VEGFA	EGFR	VEGFA_EGFR
4102	EPAS1	VEGFA	EPHB2	VEGFA_EPHB2
4103	EPAS1	VEGFA	ITGB1	VEGFA_ITGB1
4104	EPAS1	VEGFA	KDR	VEGFA_KDR
4105	EPAS1	VEGFA	TYRO3	VEGFA_TYRO3
4106	EPAS1	VEGFA	ITGA9	VEGFA_ITGA9
4107	EPAS1	VEGFA	ITGAV	VEGFA_ITGAV
4108	EPAS1	VEGFA	SIRPA	VEGFA_SIRPA
4109	EPAS1	VEGFA	GPC1	VEGFA_GPC1
4110	EPAS1	VEGFA	FLT1	VEGFA_FLT1
4111	NKX3-1	VEGFA	NRP2	VEGFA_NRP2
4112	NKX3-1	VEGFA	RET	VEGFA_RET
4113	NKX3-1	VEGFA	NRP1	VEGFA_NRP1
4114	NKX3-1	VEGFA	EGFR	VEGFA_EGFR
4115	NKX3-1	VEGFA	EPHB2	VEGFA_EPHB2
4116	NKX3-1	VEGFA	ITGB1	VEGFA_ITGB1
4117	NKX3-1	VEGFA	KDR	VEGFA_KDR

4118	NKX3-1	VEGFA	TYRO3	VEGFA_TYRO3
4119	NKX3-1	VEGFA	ITGA9	VEGFA_ITGA9
4120	NKX3-1	VEGFA	ITGAV	VEGFA_ITGAV
4121	NKX3-1	VEGFA	SIRPA	VEGFA_SIRPA
4122	NKX3-1	VEGFA	GPC1	VEGFA_GPC1
4123	NKX3-1	VEGFA	FLT1	VEGFA_FLT1
4124	PTTG1	VEGFA	NRP2	VEGFA_NRP2
4125	PTTG1	VEGFA	RET	VEGFA_RET
4126	PTTG1	VEGFA	NRP1	VEGFA_NRP1
4127	PTTG1	VEGFA	EGFR	VEGFA_EGFR
4128	PTTG1	VEGFA	EPHB2	VEGFA_EPHB2
4129	PTTG1	VEGFA	ITGB1	VEGFA_ITGB1
4130	PTTG1	VEGFA	KDR	VEGFA_KDR
4131	PTTG1	VEGFA	TYRO3	VEGFA_TYRO3
4132	PTTG1	VEGFA	ITGA9	VEGFA_ITGA9
4133	PTTG1	VEGFA	ITGAV	VEGFA_ITGAV
4134	PTTG1	VEGFA	SIRPA	VEGFA_SIRPA
4135	PTTG1	VEGFA	GPC1	VEGFA_GPC1
4136	PTTG1	VEGFA	FLT1	VEGFA_FLT1
4137	ZNF24	VEGFA	NRP2	VEGFA_NRP2
4138	ZNF24	VEGFA	RET	VEGFA_RET
4139	ZNF24	VEGFA	NRP1	VEGFA_NRP1
4140	ZNF24	VEGFA	EGFR	VEGFA_EGFR
4141	ZNF24	VEGFA	EPHB2	VEGFA_EPHB2
4142	ZNF24	VEGFA	ITGB1	VEGFA_ITGB1
4143	ZNF24	VEGFA	KDR	VEGFA_KDR
4144	ZNF24	VEGFA	TYRO3	VEGFA_TYRO3
4145	ZNF24	VEGFA	ITGA9	VEGFA_ITGA9
4146	ZNF24	VEGFA	ITGAV	VEGFA_ITGAV
4147	ZNF24	VEGFA	SIRPA	VEGFA_SIRPA
4148	ZNF24	VEGFA	GPC1	VEGFA_GPC1
4149	ZNF24	VEGFA	FLT1	VEGFA_FLT1
4150	HIPK2	VEGFA	NRP2	VEGFA_NRP2
4151	HIPK2	VEGFA	RET	VEGFA_RET
4152	HIPK2	VEGFA	NRP1	VEGFA_NRP1
4153	HIPK2	VEGFA	EGFR	VEGFA_EGFR
4154	HIPK2	VEGFA	EPHB2	VEGFA_EPHB2
4155	HIPK2	VEGFA	ITGB1	VEGFA_ITGB1
4156	HIPK2	VEGFA	KDR	VEGFA_KDR
4157	HIPK2	VEGFA	TYRO3	VEGFA_TYRO3
4158	HIPK2	VEGFA	ITGA9	VEGFA_ITGA9
4159	HIPK2	VEGFA	ITGAV	VEGFA_ITGAV
4160	HIPK2	VEGFA	SIRPA	VEGFA_SIRPA
4161	HIPK2	VEGFA	GPC1	VEGFA_GPC1
4162	HIPK2	VEGFA	FLT1	VEGFA_FLT1
4163	HEXIM1	VEGFA	NRP2	VEGFA_NRP2
4164	HEXIM1	VEGFA	RET	VEGFA_RET
4165	HEXIM1	VEGFA	NRP1	VEGFA_NRP1
4166	HEXIM1	VEGFA	EGFR	VEGFA_EGFR
4167	HEXIM1	VEGFA	EPHB2	VEGFA_EPHB2
4168	HEXIM1	VEGFA	ITGB1	VEGFA_ITGB1
4169	HEXIM1	VEGFA	KDR	VEGFA_KDR
4170	HEXIM1	VEGFA	TYRO3	VEGFA_TYRO3
4171	HEXIM1	VEGFA	ITGA9	VEGFA_ITGA9
4172	HEXIM1	VEGFA	ITGAV	VEGFA_ITGAV
4173	HEXIM1	VEGFA	SIRPA	VEGFA_SIRPA
4174	HEXIM1	VEGFA	GPC1	VEGFA_GPC1
4175	HEXIM1	VEGFA	FLT1	VEGFA_FLT1

4176	NFKB1	VEGFA	NRP2	VEGFA_NRP2
4177	NFKB1	VEGFA	RET	VEGFA_RET
4178	NFKB1	VEGFA	NRP1	VEGFA_NRP1
4179	NFKB1	VEGFA	EGFR	VEGFA_EGFR
4180	NFKB1	VEGFA	EPHB2	VEGFA_EPHB2
4181	NFKB1	VEGFA	ITGB1	VEGFA_ITGB1
4182	NFKB1	VEGFA	KDR	VEGFA_KDR
4183	NFKB1	VEGFA	TYRO3	VEGFA_TYRO3
4184	NFKB1	VEGFA	ITGA9	VEGFA_ITGA9
4185	NFKB1	VEGFA	ITGAV	VEGFA_ITGAV
4186	NFKB1	VEGFA	SIRPA	VEGFA_SIRPA
4187	NFKB1	VEGFA	GPC1	VEGFA_GPC1
4188	NFKB1	VEGFA	FLT1	VEGFA_FLT1
4189	MYC	VEGFA	NRP2	VEGFA_NRP2
4190	MYC	VEGFA	RET	VEGFA_RET
4191	MYC	VEGFA	NRP1	VEGFA_NRP1
4192	MYC	VEGFA	EGFR	VEGFA_EGFR
4193	MYC	VEGFA	EPHB2	VEGFA_EPHB2
4194	MYC	VEGFA	ITGB1	VEGFA_ITGB1
4195	MYC	VEGFA	KDR	VEGFA_KDR
4196	MYC	VEGFA	TYRO3	VEGFA_TYRO3
4197	MYC	VEGFA	ITGA9	VEGFA_ITGA9
4198	MYC	VEGFA	ITGAV	VEGFA_ITGAV
4199	MYC	VEGFA	SIRPA	VEGFA_SIRPA
4200	MYC	VEGFA	GPC1	VEGFA_GPC1
4201	MYC	VEGFA	FLT1	VEGFA_FLT1
4202	PGR	VEGFA	NRP2	VEGFA_NRP2
4203	PGR	VEGFA	RET	VEGFA_RET
4204	PGR	VEGFA	NRP1	VEGFA_NRP1
4205	PGR	VEGFA	EGFR	VEGFA_EGFR
4206	PGR	VEGFA	EPHB2	VEGFA_EPHB2
4207	PGR	VEGFA	ITGB1	VEGFA_ITGB1
4208	PGR	VEGFA	KDR	VEGFA_KDR
4209	PGR	VEGFA	TYRO3	VEGFA_TYRO3
4210	PGR	VEGFA	ITGA9	VEGFA_ITGA9
4211	PGR	VEGFA	ITGAV	VEGFA_ITGAV
4212	PGR	VEGFA	SIRPA	VEGFA_SIRPA
4213	PGR	VEGFA	GPC1	VEGFA_GPC1
4214	PGR	VEGFA	FLT1	VEGFA_FLT1
4215	ESR1	VEGFA	NRP2	VEGFA_NRP2
4216	ESR1	VEGFA	RET	VEGFA_RET
4217	ESR1	VEGFA	NRP1	VEGFA_NRP1
4218	ESR1	VEGFA	EGFR	VEGFA_EGFR
4219	ESR1	VEGFA	EPHB2	VEGFA_EPHB2
4220	ESR1	VEGFA	ITGB1	VEGFA_ITGB1
4221	ESR1	VEGFA	KDR	VEGFA_KDR
4222	ESR1	VEGFA	TYRO3	VEGFA_TYRO3
4223	ESR1	VEGFA	ITGA9	VEGFA_ITGA9
4224	ESR1	VEGFA	ITGAV	VEGFA_ITGAV
4225	ESR1	VEGFA	SIRPA	VEGFA_SIRPA
4226	ESR1	VEGFA	GPC1	VEGFA_GPC1
4227	ESR1	VEGFA	FLT1	VEGFA_FLT1
4228	SP1	VEGFA	NRP2	VEGFA_NRP2
4229	SP1	VEGFA	RET	VEGFA_RET
4230	SP1	VEGFA	NRP1	VEGFA_NRP1
4231	SP1	VEGFA	EGFR	VEGFA_EGFR
4232	SP1	VEGFA	EPHB2	VEGFA_EPHB2
4233	SP1	VEGFA	ITGB1	VEGFA_ITGB1

4234	SP1	VEGFA	KDR	VEGFA_KDR
4235	SP1	VEGFA	TYRO3	VEGFA_TYRO3
4236	SP1	VEGFA	ITGA9	VEGFA_ITGA9
4237	SP1	VEGFA	ITGAV	VEGFA_ITGAV
4238	SP1	VEGFA	SIRPA	VEGFA_SIRPA
4239	SP1	VEGFA	GPC1	VEGFA_GPC1
4240	SP1	VEGFA	FLT1	VEGFA_FLT1
4241	FOXO3	VEGFA	NRP2	VEGFA_NRP2
4242	FOXO3	VEGFA	RET	VEGFA_RET
4243	FOXO3	VEGFA	NRP1	VEGFA_NRP1
4244	FOXO3	VEGFA	EGFR	VEGFA_EGFR
4245	FOXO3	VEGFA	EPHB2	VEGFA_EPHB2
4246	FOXO3	VEGFA	ITGB1	VEGFA_ITGB1
4247	FOXO3	VEGFA	KDR	VEGFA_KDR
4248	FOXO3	VEGFA	TYRO3	VEGFA_TYRO3
4249	FOXO3	VEGFA	ITGA9	VEGFA_ITGA9
4250	FOXO3	VEGFA	ITGAV	VEGFA_ITGAV
4251	FOXO3	VEGFA	SIRPA	VEGFA_SIRPA
4252	FOXO3	VEGFA	GPC1	VEGFA_GPC1
4253	FOXO3	VEGFA	FLT1	VEGFA_FLT1
4254	ESR2	VEGFA	NRP2	VEGFA_NRP2
4255	ESR2	VEGFA	RET	VEGFA_RET
4256	ESR2	VEGFA	NRP1	VEGFA_NRP1
4257	ESR2	VEGFA	EGFR	VEGFA_EGFR
4258	ESR2	VEGFA	EPHB2	VEGFA_EPHB2
4259	ESR2	VEGFA	ITGB1	VEGFA_ITGB1
4260	ESR2	VEGFA	KDR	VEGFA_KDR
4261	ESR2	VEGFA	TYRO3	VEGFA_TYRO3
4262	ESR2	VEGFA	ITGA9	VEGFA_ITGA9
4263	ESR2	VEGFA	ITGAV	VEGFA_ITGAV
4264	ESR2	VEGFA	SIRPA	VEGFA_SIRPA
4265	ESR2	VEGFA	GPC1	VEGFA_GPC1
4266	ESR2	VEGFA	FLT1	VEGFA_FLT1
4267	BRCA1	VEGFA	NRP2	VEGFA_NRP2
4268	BRCA1	VEGFA	RET	VEGFA_RET
4269	BRCA1	VEGFA	NRP1	VEGFA_NRP1
4270	BRCA1	VEGFA	EGFR	VEGFA_EGFR
4271	BRCA1	VEGFA	EPHB2	VEGFA_EPHB2
4272	BRCA1	VEGFA	ITGB1	VEGFA_ITGB1
4273	BRCA1	VEGFA	KDR	VEGFA_KDR
4274	BRCA1	VEGFA	TYRO3	VEGFA_TYRO3
4275	BRCA1	VEGFA	ITGA9	VEGFA_ITGA9
4276	BRCA1	VEGFA	ITGAV	VEGFA_ITGAV
4277	BRCA1	VEGFA	SIRPA	VEGFA_SIRPA
4278	BRCA1	VEGFA	GPC1	VEGFA_GPC1
4279	BRCA1	VEGFA	FLT1	VEGFA_FLT1
4280	HNF1A	VEGFA	NRP2	VEGFA_NRP2
4281	HNF1A	VEGFA	RET	VEGFA_RET
4282	HNF1A	VEGFA	NRP1	VEGFA_NRP1
4283	HNF1A	VEGFA	EGFR	VEGFA_EGFR
4284	HNF1A	VEGFA	EPHB2	VEGFA_EPHB2
4285	HNF1A	VEGFA	ITGB1	VEGFA_ITGB1
4286	HNF1A	VEGFA	KDR	VEGFA_KDR
4287	HNF1A	VEGFA	TYRO3	VEGFA_TYRO3
4288	HNF1A	VEGFA	ITGA9	VEGFA_ITGA9
4289	HNF1A	VEGFA	ITGAV	VEGFA_ITGAV
4290	HNF1A	VEGFA	SIRPA	VEGFA_SIRPA
4291	HNF1A	VEGFA	GPC1	VEGFA_GPC1

4292	HNF1A	VEGFA	FLT1	VEGFA_FLT1
4293	EGR1	VEGFA	NRP2	VEGFA_NRP2
4294	EGR1	VEGFA	RET	VEGFA_RET
4295	EGR1	VEGFA	NRP1	VEGFA_NRP1
4296	EGR1	VEGFA	EGFR	VEGFA_EGFR
4297	EGR1	VEGFA	EPHB2	VEGFA_EPHB2
4298	EGR1	VEGFA	ITGB1	VEGFA_ITGB1
4299	EGR1	VEGFA	KDR	VEGFA_KDR
4300	EGR1	VEGFA	TYRO3	VEGFA_TYRO3
4301	EGR1	VEGFA	ITGA9	VEGFA_ITGA9
4302	EGR1	VEGFA	ITGAV	VEGFA_ITGAV
4303	EGR1	VEGFA	SIRPA	VEGFA_SIRPA
4304	EGR1	VEGFA	GPC1	VEGFA_GPC1
4305	EGR1	VEGFA	FLT1	VEGFA_FLT1
4306	HIF1A	VEGFA	NRP2	VEGFA_NRP2
4307	HIF1A	VEGFA	RET	VEGFA_RET
4308	HIF1A	VEGFA	NRP1	VEGFA_NRP1
4309	HIF1A	VEGFA	EGFR	VEGFA_EGFR
4310	HIF1A	VEGFA	EPHB2	VEGFA_EPHB2
4311	HIF1A	VEGFA	ITGB1	VEGFA_ITGB1
4312	HIF1A	VEGFA	KDR	VEGFA_KDR
4313	HIF1A	VEGFA	TYRO3	VEGFA_TYRO3
4314	HIF1A	VEGFA	ITGA9	VEGFA_ITGA9
4315	HIF1A	VEGFA	ITGAV	VEGFA_ITGAV
4316	HIF1A	VEGFA	SIRPA	VEGFA_SIRPA
4317	HIF1A	VEGFA	GPC1	VEGFA_GPC1
4318	HIF1A	VEGFA	FLT1	VEGFA_FLT1
4319	E2F1	VEGFA	NRP2	VEGFA_NRP2
4320	E2F1	VEGFA	RET	VEGFA_RET
4321	E2F1	VEGFA	NRP1	VEGFA_NRP1
4322	E2F1	VEGFA	EGFR	VEGFA_EGFR
4323	E2F1	VEGFA	EPHB2	VEGFA_EPHB2
4324	E2F1	VEGFA	ITGB1	VEGFA_ITGB1
4325	E2F1	VEGFA	KDR	VEGFA_KDR
4326	E2F1	VEGFA	TYRO3	VEGFA_TYRO3
4327	E2F1	VEGFA	ITGA9	VEGFA_ITGA9
4328	E2F1	VEGFA	ITGAV	VEGFA_ITGAV
4329	E2F1	VEGFA	SIRPA	VEGFA_SIRPA
4330	E2F1	VEGFA	GPC1	VEGFA_GPC1
4331	E2F1	VEGFA	FLT1	VEGFA_FLT1
4332	SMAD3	VEGFA	NRP2	VEGFA_NRP2
4333	SMAD3	VEGFA	RET	VEGFA_RET
4334	SMAD3	VEGFA	NRP1	VEGFA_NRP1
4335	SMAD3	VEGFA	EGFR	VEGFA_EGFR
4336	SMAD3	VEGFA	EPHB2	VEGFA_EPHB2
4337	SMAD3	VEGFA	ITGB1	VEGFA_ITGB1
4338	SMAD3	VEGFA	KDR	VEGFA_KDR
4339	SMAD3	VEGFA	TYRO3	VEGFA_TYRO3
4340	SMAD3	VEGFA	ITGA9	VEGFA_ITGA9
4341	SMAD3	VEGFA	ITGAV	VEGFA_ITGAV
4342	SMAD3	VEGFA	SIRPA	VEGFA_SIRPA
4343	SMAD3	VEGFA	GPC1	VEGFA_GPC1
4344	SMAD3	VEGFA	FLT1	VEGFA_FLT1
4345	UHRF1	VEGFA	NRP2	VEGFA_NRP2
4346	UHRF1	VEGFA	RET	VEGFA_RET
4347	UHRF1	VEGFA	NRP1	VEGFA_NRP1
4348	UHRF1	VEGFA	EGFR	VEGFA_EGFR
4349	UHRF1	VEGFA	EPHB2	VEGFA_EPHB2

4350	UHRF1	VEGFA	ITGB1	VEGFA_ITGB1
4351	UHRF1	VEGFA	KDR	VEGFA_KDR
4352	UHRF1	VEGFA	TYRO3	VEGFA_TYRO3
4353	UHRF1	VEGFA	ITGA9	VEGFA_ITGA9
4354	UHRF1	VEGFA	ITGAV	VEGFA_ITGAV
4355	UHRF1	VEGFA	SIRPA	VEGFA_SIRPA
4356	UHRF1	VEGFA	GPC1	VEGFA_GPC1
4357	UHRF1	VEGFA	FLT1	VEGFA_FLT1
4358	NR1H4	VEGFA	NRP2	VEGFA_NRP2
4359	NR1H4	VEGFA	RET	VEGFA_RET
4360	NR1H4	VEGFA	NRP1	VEGFA_NRP1
4361	NR1H4	VEGFA	EGFR	VEGFA_EGFR
4362	NR1H4	VEGFA	EPHB2	VEGFA_EPHB2
4363	NR1H4	VEGFA	ITGB1	VEGFA_ITGB1
4364	NR1H4	VEGFA	KDR	VEGFA_KDR
4365	NR1H4	VEGFA	TYRO3	VEGFA_TYRO3
4366	NR1H4	VEGFA	ITGA9	VEGFA_ITGA9
4367	NR1H4	VEGFA	ITGAV	VEGFA_ITGAV
4368	NR1H4	VEGFA	SIRPA	VEGFA_SIRPA
4369	NR1H4	VEGFA	GPC1	VEGFA_GPC1
4370	NR1H4	VEGFA	FLT1	VEGFA_FLT1
4371	FOXM1	VEGFA	NRP2	VEGFA_NRP2
4372	FOXM1	VEGFA	RET	VEGFA_RET
4373	FOXM1	VEGFA	NRP1	VEGFA_NRP1
4374	FOXM1	VEGFA	EGFR	VEGFA_EGFR
4375	FOXM1	VEGFA	EPHB2	VEGFA_EPHB2
4376	FOXM1	VEGFA	ITGB1	VEGFA_ITGB1
4377	FOXM1	VEGFA	KDR	VEGFA_KDR
4378	FOXM1	VEGFA	TYRO3	VEGFA_TYRO3
4379	FOXM1	VEGFA	ITGA9	VEGFA_ITGA9
4380	FOXM1	VEGFA	ITGAV	VEGFA_ITGAV
4381	FOXM1	VEGFA	SIRPA	VEGFA_SIRPA
4382	FOXM1	VEGFA	GPC1	VEGFA_GPC1
4383	FOXM1	VEGFA	FLT1	VEGFA_FLT1
4384	XBP1	VEGFA	NRP2	VEGFA_NRP2
4385	XBP1	VEGFA	RET	VEGFA_RET
4386	XBP1	VEGFA	NRP1	VEGFA_NRP1
4387	XBP1	VEGFA	EGFR	VEGFA_EGFR
4388	XBP1	VEGFA	EPHB2	VEGFA_EPHB2
4389	XBP1	VEGFA	ITGB1	VEGFA_ITGB1
4390	XBP1	VEGFA	KDR	VEGFA_KDR
4391	XBP1	VEGFA	TYRO3	VEGFA_TYRO3
4392	XBP1	VEGFA	ITGA9	VEGFA_ITGA9
4393	XBP1	VEGFA	ITGAV	VEGFA_ITGAV
4394	XBP1	VEGFA	SIRPA	VEGFA_SIRPA
4395	XBP1	VEGFA	GPC1	VEGFA_GPC1
4396	XBP1	VEGFA	FLT1	VEGFA_FLT1
4397	COPS5	VEGFA	NRP2	VEGFA_NRP2
4398	COPS5	VEGFA	RET	VEGFA_RET
4399	COPS5	VEGFA	NRP1	VEGFA_NRP1
4400	COPS5	VEGFA	EGFR	VEGFA_EGFR
4401	COPS5	VEGFA	EPHB2	VEGFA_EPHB2
4402	COPS5	VEGFA	ITGB1	VEGFA_ITGB1
4403	COPS5	VEGFA	KDR	VEGFA_KDR
4404	COPS5	VEGFA	TYRO3	VEGFA_TYRO3
4405	COPS5	VEGFA	ITGA9	VEGFA_ITGA9
4406	COPS5	VEGFA	ITGAV	VEGFA_ITGAV
4407	COPS5	VEGFA	SIRPA	VEGFA_SIRPA

4408	COPS5	VEGFA	GPC1	VEGFA_GPC1
4409	COPS5	VEGFA	FLT1	VEGFA_FLT1
4410	AR	VEGFA	NRP2	VEGFA_NRP2
4411	AR	VEGFA	RET	VEGFA_RET
4412	AR	VEGFA	NRP1	VEGFA_NRP1
4413	AR	VEGFA	EGFR	VEGFA_EGFR
4414	AR	VEGFA	EPHB2	VEGFA_EPHB2
4415	AR	VEGFA	ITGB1	VEGFA_ITGB1
4416	AR	VEGFA	KDR	VEGFA_KDR
4417	AR	VEGFA	TYRO3	VEGFA_TYRO3
4418	AR	VEGFA	ITGA9	VEGFA_ITGA9
4419	AR	VEGFA	ITGAV	VEGFA_ITGAV
4420	AR	VEGFA	SIRPA	VEGFA_SIRPA
4421	AR	VEGFA	GPC1	VEGFA_GPC1
4422	AR	VEGFA	FLT1	VEGFA_FLT1
4423	STAT3	VEGFA	NRP2	VEGFA_NRP2
4424	STAT3	VEGFA	RET	VEGFA_RET
4425	STAT3	VEGFA	NRP1	VEGFA_NRP1
4426	STAT3	VEGFA	EGFR	VEGFA_EGFR
4427	STAT3	VEGFA	EPHB2	VEGFA_EPHB2
4428	STAT3	VEGFA	ITGB1	VEGFA_ITGB1
4429	STAT3	VEGFA	KDR	VEGFA_KDR
4430	STAT3	VEGFA	TYRO3	VEGFA_TYRO3
4431	STAT3	VEGFA	ITGA9	VEGFA_ITGA9
4432	STAT3	VEGFA	ITGAV	VEGFA_ITGAV
4433	STAT3	VEGFA	SIRPA	VEGFA_SIRPA
4434	STAT3	VEGFA	GPC1	VEGFA_GPC1
4435	STAT3	VEGFA	FLT1	VEGFA_FLT1
4436	MEF2C	VEGFA	NRP2	VEGFA_NRP2
4437	MEF2C	VEGFA	RET	VEGFA_RET
4438	MEF2C	VEGFA	NRP1	VEGFA_NRP1
4439	MEF2C	VEGFA	EGFR	VEGFA_EGFR
4440	MEF2C	VEGFA	EPHB2	VEGFA_EPHB2
4441	MEF2C	VEGFA	ITGB1	VEGFA_ITGB1
4442	MEF2C	VEGFA	KDR	VEGFA_KDR
4443	MEF2C	VEGFA	TYRO3	VEGFA_TYRO3
4444	MEF2C	VEGFA	ITGA9	VEGFA_ITGA9
4445	MEF2C	VEGFA	ITGAV	VEGFA_ITGAV
4446	MEF2C	VEGFA	SIRPA	VEGFA_SIRPA
4447	MEF2C	VEGFA	GPC1	VEGFA_GPC1
4448	MEF2C	VEGFA	FLT1	VEGFA_FLT1
4449	SP3	VEGFA	NRP2	VEGFA_NRP2
4450	SP3	VEGFA	RET	VEGFA_RET
4451	SP3	VEGFA	NRP1	VEGFA_NRP1
4452	SP3	VEGFA	EGFR	VEGFA_EGFR
4453	SP3	VEGFA	EPHB2	VEGFA_EPHB2
4454	SP3	VEGFA	ITGB1	VEGFA_ITGB1
4455	SP3	VEGFA	KDR	VEGFA_KDR
4456	SP3	VEGFA	TYRO3	VEGFA_TYRO3
4457	SP3	VEGFA	ITGA9	VEGFA_ITGA9
4458	SP3	VEGFA	ITGAV	VEGFA_ITGAV
4459	SP3	VEGFA	SIRPA	VEGFA_SIRPA
4460	SP3	VEGFA	GPC1	VEGFA_GPC1
4461	SP3	VEGFA	FLT1	VEGFA_FLT1
4462	ARNT	VEGFA	NRP2	VEGFA_NRP2
4463	ARNT	VEGFA	RET	VEGFA_RET
4464	ARNT	VEGFA	NRP1	VEGFA_NRP1
4465	ARNT	VEGFA	EGFR	VEGFA_EGFR

4466	ARNT	VEGFA	EPHB2	VEGFA_EPHB2
4467	ARNT	VEGFA	ITGB1	VEGFA_ITGB1
4468	ARNT	VEGFA	KDR	VEGFA_KDR
4469	ARNT	VEGFA	TYRO3	VEGFA_TYRO3
4470	ARNT	VEGFA	ITGA9	VEGFA_ITGA9
4471	ARNT	VEGFA	ITGAV	VEGFA_ITGAV
4472	ARNT	VEGFA	SIRPA	VEGFA_SIRPA
4473	ARNT	VEGFA	GPC1	VEGFA_GPC1
4474	ARNT	VEGFA	FLT1	VEGFA_FLT1
4475	DNMT1	VEGFA	NRP2	VEGFA_NRP2
4476	DNMT1	VEGFA	RET	VEGFA_RET
4477	DNMT1	VEGFA	NRP1	VEGFA_NRP1
4478	DNMT1	VEGFA	EGFR	VEGFA_EGFR
4479	DNMT1	VEGFA	EPHB2	VEGFA_EPHB2
4480	DNMT1	VEGFA	ITGB1	VEGFA_ITGB1
4481	DNMT1	VEGFA	KDR	VEGFA_KDR
4482	DNMT1	VEGFA	TYRO3	VEGFA_TYRO3
4483	DNMT1	VEGFA	ITGA9	VEGFA_ITGA9
4484	DNMT1	VEGFA	ITGAV	VEGFA_ITGAV
4485	DNMT1	VEGFA	SIRPA	VEGFA_SIRPA
4486	DNMT1	VEGFA	GPC1	VEGFA_GPC1
4487	DNMT1	VEGFA	FLT1	VEGFA_FLT1
4488	RUNX1	VEGFA	NRP2	VEGFA_NRP2
4489	RUNX1	VEGFA	RET	VEGFA_RET
4490	RUNX1	VEGFA	NRP1	VEGFA_NRP1
4491	RUNX1	VEGFA	EGFR	VEGFA_EGFR
4492	RUNX1	VEGFA	EPHB2	VEGFA_EPHB2
4493	RUNX1	VEGFA	ITGB1	VEGFA_ITGB1
4494	RUNX1	VEGFA	KDR	VEGFA_KDR
4495	RUNX1	VEGFA	TYRO3	VEGFA_TYRO3
4496	RUNX1	VEGFA	ITGA9	VEGFA_ITGA9
4497	RUNX1	VEGFA	ITGAV	VEGFA_ITGAV
4498	RUNX1	VEGFA	SIRPA	VEGFA_SIRPA
4499	RUNX1	VEGFA	GPC1	VEGFA_GPC1
4500	RUNX1	VEGFA	FLT1	VEGFA_FLT1
4501	ZFP36L1	VEGFA	NRP2	VEGFA_NRP2
4502	ZFP36L1	VEGFA	RET	VEGFA_RET
4503	ZFP36L1	VEGFA	NRP1	VEGFA_NRP1
4504	ZFP36L1	VEGFA	EGFR	VEGFA_EGFR
4505	ZFP36L1	VEGFA	EPHB2	VEGFA_EPHB2
4506	ZFP36L1	VEGFA	ITGB1	VEGFA_ITGB1
4507	ZFP36L1	VEGFA	KDR	VEGFA_KDR
4508	ZFP36L1	VEGFA	TYRO3	VEGFA_TYRO3
4509	ZFP36L1	VEGFA	ITGA9	VEGFA_ITGA9
4510	ZFP36L1	VEGFA	ITGAV	VEGFA_ITGAV
4511	ZFP36L1	VEGFA	SIRPA	VEGFA_SIRPA
4512	ZFP36L1	VEGFA	GPC1	VEGFA_GPC1
4513	ZFP36L1	VEGFA	FLT1	VEGFA_FLT1
4514	HDAC2	VEGFA	NRP2	VEGFA_NRP2
4515	HDAC2	VEGFA	RET	VEGFA_RET
4516	HDAC2	VEGFA	NRP1	VEGFA_NRP1
4517	HDAC2	VEGFA	EGFR	VEGFA_EGFR
4518	HDAC2	VEGFA	EPHB2	VEGFA_EPHB2
4519	HDAC2	VEGFA	ITGB1	VEGFA_ITGB1
4520	HDAC2	VEGFA	KDR	VEGFA_KDR
4521	HDAC2	VEGFA	TYRO3	VEGFA_TYRO3
4522	HDAC2	VEGFA	ITGA9	VEGFA_ITGA9
4523	HDAC2	VEGFA	ITGAV	VEGFA_ITGAV

4524	HDAC2	VEGFA	SIRPA	VEGFA_SIRPA
4525	HDAC2	VEGFA	GPC1	VEGFA_GPC1
4526	HDAC2	VEGFA	FLT1	VEGFA_FLT1
4527	TRAF6	VEGFA	NRP2	VEGFA_NRP2
4528	TRAF6	VEGFA	RET	VEGFA_RET
4529	TRAF6	VEGFA	NRP1	VEGFA_NRP1
4530	TRAF6	VEGFA	EGFR	VEGFA_EGFR
4531	TRAF6	VEGFA	EPHB2	VEGFA_EPHB2
4532	TRAF6	VEGFA	ITGB1	VEGFA_ITGB1
4533	TRAF6	VEGFA	KDR	VEGFA_KDR
4534	TRAF6	VEGFA	TYRO3	VEGFA_TYRO3
4535	TRAF6	VEGFA	ITGA9	VEGFA_ITGA9
4536	TRAF6	VEGFA	ITGAV	VEGFA_ITGAV
4537	TRAF6	VEGFA	SIRPA	VEGFA_SIRPA
4538	TRAF6	VEGFA	GPC1	VEGFA_GPC1
4539	TRAF6	VEGFA	FLT1	VEGFA_FLT1
4540	TCF4	VEGFA	NRP2	VEGFA_NRP2
4541	TCF4	VEGFA	RET	VEGFA_RET
4542	TCF4	VEGFA	NRP1	VEGFA_NRP1
4543	TCF4	VEGFA	EGFR	VEGFA_EGFR
4544	TCF4	VEGFA	EPHB2	VEGFA_EPHB2
4545	TCF4	VEGFA	ITGB1	VEGFA_ITGB1
4546	TCF4	VEGFA	KDR	VEGFA_KDR
4547	TCF4	VEGFA	TYRO3	VEGFA_TYRO3
4548	TCF4	VEGFA	ITGA9	VEGFA_ITGA9
4549	TCF4	VEGFA	ITGAV	VEGFA_ITGAV
4550	TCF4	VEGFA	SIRPA	VEGFA_SIRPA
4551	TCF4	VEGFA	GPC1	VEGFA_GPC1
4552	TCF4	VEGFA	FLT1	VEGFA_FLT1
4553	JUN	VEGFA	NRP2	VEGFA_NRP2
4554	JUN	VEGFA	RET	VEGFA_RET
4555	JUN	VEGFA	NRP1	VEGFA_NRP1
4556	JUN	VEGFA	EGFR	VEGFA_EGFR
4557	JUN	VEGFA	EPHB2	VEGFA_EPHB2
4558	JUN	VEGFA	ITGB1	VEGFA_ITGB1
4559	JUN	VEGFA	KDR	VEGFA_KDR
4560	JUN	VEGFA	TYRO3	VEGFA_TYRO3
4561	JUN	VEGFA	ITGA9	VEGFA_ITGA9
4562	JUN	VEGFA	ITGAV	VEGFA_ITGAV
4563	JUN	VEGFA	SIRPA	VEGFA_SIRPA
4564	JUN	VEGFA	GPC1	VEGFA_GPC1
4565	JUN	VEGFA	FLT1	VEGFA_FLT1
4566	KLF4	VEGFA	NRP2	VEGFA_NRP2
4567	KLF4	VEGFA	RET	VEGFA_RET
4568	KLF4	VEGFA	NRP1	VEGFA_NRP1
4569	KLF4	VEGFA	EGFR	VEGFA_EGFR
4570	KLF4	VEGFA	EPHB2	VEGFA_EPHB2
4571	KLF4	VEGFA	ITGB1	VEGFA_ITGB1
4572	KLF4	VEGFA	KDR	VEGFA_KDR
4573	KLF4	VEGFA	TYRO3	VEGFA_TYRO3
4574	KLF4	VEGFA	ITGA9	VEGFA_ITGA9
4575	KLF4	VEGFA	ITGAV	VEGFA_ITGAV
4576	KLF4	VEGFA	SIRPA	VEGFA_SIRPA
4577	KLF4	VEGFA	GPC1	VEGFA_GPC1
4578	KLF4	VEGFA	FLT1	VEGFA_FLT1
4579	TP53	VEGFA	NRP2	VEGFA_NRP2
4580	TP53	VEGFA	RET	VEGFA_RET
4581	TP53	VEGFA	NRP1	VEGFA_NRP1

4582	TP53	VEGFA	EGFR	VEGFA_EGFR
4583	TP53	VEGFA	EPHB2	VEGFA_EPHB2
4584	TP53	VEGFA	ITGB1	VEGFA_ITGB1
4585	TP53	VEGFA	KDR	VEGFA_KDR
4586	TP53	VEGFA	TYRO3	VEGFA_TYRO3
4587	TP53	VEGFA	ITGA9	VEGFA_ITGA9
4588	TP53	VEGFA	ITGAV	VEGFA_ITGAV
4589	TP53	VEGFA	SIRPA	VEGFA_SIRPA
4590	TP53	VEGFA	GPC1	VEGFA_GPC1
4591	TP53	VEGFA	FLT1	VEGFA_FLT1
4592	ID3	VEGFA	NRP2	VEGFA_NRP2
4593	ID3	VEGFA	RET	VEGFA_RET
4594	ID3	VEGFA	NRP1	VEGFA_NRP1
4595	ID3	VEGFA	EGFR	VEGFA_EGFR
4596	ID3	VEGFA	EPHB2	VEGFA_EPHB2
4597	ID3	VEGFA	ITGB1	VEGFA_ITGB1
4598	ID3	VEGFA	KDR	VEGFA_KDR
4599	ID3	VEGFA	TYRO3	VEGFA_TYRO3
4600	ID3	VEGFA	ITGA9	VEGFA_ITGA9
4601	ID3	VEGFA	ITGAV	VEGFA_ITGAV
4602	ID3	VEGFA	SIRPA	VEGFA_SIRPA
4603	ID3	VEGFA	GPC1	VEGFA_GPC1
4604	ID3	VEGFA	FLT1	VEGFA_FLT1
4605	HIC1	VEGFA	NRP2	VEGFA_NRP2
4606	HIC1	VEGFA	RET	VEGFA_RET
4607	HIC1	VEGFA	NRP1	VEGFA_NRP1
4608	HIC1	VEGFA	EGFR	VEGFA_EGFR
4609	HIC1	VEGFA	EPHB2	VEGFA_EPHB2
4610	HIC1	VEGFA	ITGB1	VEGFA_ITGB1
4611	HIC1	VEGFA	KDR	VEGFA_KDR
4612	HIC1	VEGFA	TYRO3	VEGFA_TYRO3
4613	HIC1	VEGFA	ITGA9	VEGFA_ITGA9
4614	HIC1	VEGFA	ITGAV	VEGFA_ITGAV
4615	HIC1	VEGFA	SIRPA	VEGFA_SIRPA
4616	HIC1	VEGFA	GPC1	VEGFA_GPC1
4617	HIC1	VEGFA	FLT1	VEGFA_FLT1
4618	HTATIP2	VEGFA	NRP2	VEGFA_NRP2
4619	HTATIP2	VEGFA	RET	VEGFA_RET
4620	HTATIP2	VEGFA	NRP1	VEGFA_NRP1
4621	HTATIP2	VEGFA	EGFR	VEGFA_EGFR
4622	HTATIP2	VEGFA	EPHB2	VEGFA_EPHB2
4623	HTATIP2	VEGFA	ITGB1	VEGFA_ITGB1
4624	HTATIP2	VEGFA	KDR	VEGFA_KDR
4625	HTATIP2	VEGFA	TYRO3	VEGFA_TYRO3
4626	HTATIP2	VEGFA	ITGA9	VEGFA_ITGA9
4627	HTATIP2	VEGFA	ITGAV	VEGFA_ITGAV
4628	HTATIP2	VEGFA	SIRPA	VEGFA_SIRPA
4629	HTATIP2	VEGFA	GPC1	VEGFA_GPC1
4630	HTATIP2	VEGFA	FLT1	VEGFA_FLT1
4631	EP300	VEGFA	NRP2	VEGFA_NRP2
4632	EP300	VEGFA	RET	VEGFA_RET
4633	EP300	VEGFA	NRP1	VEGFA_NRP1
4634	EP300	VEGFA	EGFR	VEGFA_EGFR
4635	EP300	VEGFA	EPHB2	VEGFA_EPHB2
4636	EP300	VEGFA	ITGB1	VEGFA_ITGB1
4637	EP300	VEGFA	KDR	VEGFA_KDR
4638	EP300	VEGFA	TYRO3	VEGFA_TYRO3
4639	EP300	VEGFA	ITGA9	VEGFA_ITGA9

4640	EP300	VEGFA	ITGAV	VEGFA_ITGAV
4641	EP300	VEGFA	SIRPA	VEGFA_SIRPA
4642	EP300	VEGFA	GPC1	VEGFA_GPC1
4643	EP300	VEGFA	FLT1	VEGFA_FLT1
4644	RUNX2	VEGFC	ITGB1	VEGFC_ITGB1
4645	SIX1	VEGFC	ITGB1	VEGFC_ITGB1
4646	ZNF148	VIM	CD44	VIM_CD44
4647	SP1	VIM	CD44	VIM_CD44
4648	ERG	VIM	CD44	VIM_CD44
4649	PARP1	VIM	CD44	VIM_CD44
4650	CTNNB1	VIM	CD44	VIM_CD44
4651	HOXA7	VIM	CD44	VIM_CD44
4652	TRIM16	VIM	CD44	VIM_CD44
4653	ETV4	VIM	CD44	VIM_CD44
4654	SOX2	VIM	CD44	VIM_CD44
4655	HIF1A	VIM	CD44	VIM_CD44
4656	KLF8	VIM	CD44	VIM_CD44
4657	AR	VIM	CD44	VIM_CD44
4658	HDGF	VIM	CD44	VIM_CD44
4659	ZEB2	VIM	CD44	VIM_CD44
4660	HIPK2	VIM	CD44	VIM_CD44
4661	TCF4	VIM	CD44	VIM_CD44
4662	ETS2	VWF	TNFRSF11B	VWF_TNFRSF11B
4663	ERG	VWF	TNFRSF11B	VWF_TNFRSF11B
4664	NFIC	VWF	TNFRSF11B	VWF_TNFRSF11B
4665	POU2F1	VWF	TNFRSF11B	VWF_TNFRSF11B
4666	GATA6	VWF	TNFRSF11B	VWF_TNFRSF11B
4667	NFIL3	VWF	TNFRSF11B	VWF_TNFRSF11B
4668	NFKB1	VWF	TNFRSF11B	VWF_TNFRSF11B
4669	RELA	VWF	TNFRSF11B	VWF_TNFRSF11B
4670	ETS1	VWF	TNFRSF11B	VWF_TNFRSF11B
4671	YY1	VWF	TNFRSF11B	VWF_TNFRSF11B
4672	FOXQ1	WNT3A	ATP6AP2	WNT3A_ATP6AP2
4673	FOXQ1	WNT3A	LRP1	WNT3A_LRP1
4674	SP3	CALM2	ABCA1	CALM2_ABCA1
4675	SP3	CALM1	ABCA1	CALM1_ABCA1
4676	SP3	PLTP	ABCA1	PLTP_ABCA1
4677	SP3	CALM3	ABCA1	CALM3_ABCA1
4678	NR1H3	CALM2	ABCA1	CALM2_ABCA1
4679	NR1H3	CALM1	ABCA1	CALM1_ABCA1
4680	NR1H3	PLTP	ABCA1	PLTP_ABCA1
4681	NR1H3	CALM3	ABCA1	CALM3_ABCA1
4682	SREBF2	CALM2	ABCA1	CALM2_ABCA1
4683	SREBF2	CALM1	ABCA1	CALM1_ABCA1
4684	SREBF2	PLTP	ABCA1	PLTP_ABCA1
4685	SREBF2	CALM3	ABCA1	CALM3_ABCA1
4686	NFKB1	CALM2	ABCA1	CALM2_ABCA1
4687	NFKB1	CALM1	ABCA1	CALM1_ABCA1
4688	NFKB1	PLTP	ABCA1	PLTP_ABCA1
4689	NFKB1	CALM3	ABCA1	CALM3_ABCA1
4690	FOXA1	CALM2	ABCA1	CALM2_ABCA1
4691	FOXA1	CALM1	ABCA1	CALM1_ABCA1
4692	FOXA1	PLTP	ABCA1	PLTP_ABCA1
4693	FOXA1	CALM3	ABCA1	CALM3_ABCA1
4694	ZNF202	CALM2	ABCA1	CALM2_ABCA1
4695	ZNF202	CALM1	ABCA1	CALM1_ABCA1
4696	ZNF202	PLTP	ABCA1	PLTP_ABCA1
4697	ZNF202	CALM3	ABCA1	CALM3_ABCA1

4698	STAT3	CALM2	ABCA1	CALM2_ABCA1
4699	STAT3	CALM1	ABCA1	CALM1_ABCA1
4700	STAT3	PLTP	ABCA1	PLTP_ABCA1
4701	STAT3	CALM3	ABCA1	CALM3_ABCA1
4702	NR1H2	CALM2	ABCA1	CALM2_ABCA1
4703	NR1H2	CALM1	ABCA1	CALM1_ABCA1
4704	NR1H2	PLTP	ABCA1	PLTP_ABCA1
4705	NR1H2	CALM3	ABCA1	CALM3_ABCA1
4706	USF1	CALM2	ABCA1	CALM2_ABCA1
4707	USF1	CALM1	ABCA1	CALM1_ABCA1
4708	USF1	PLTP	ABCA1	PLTP_ABCA1
4709	USF1	CALM3	ABCA1	CALM3_ABCA1
4710	FOXA2	CALM2	ABCA1	CALM2_ABCA1
4711	FOXA2	CALM1	ABCA1	CALM1_ABCA1
4712	FOXA2	PLTP	ABCA1	PLTP_ABCA1
4713	FOXA2	CALM3	ABCA1	CALM3_ABCA1
4714	RELA	CALM2	ABCA1	CALM2_ABCA1
4715	RELA	CALM1	ABCA1	CALM1_ABCA1
4716	RELA	PLTP	ABCA1	PLTP_ABCA1
4717	RELA	CALM3	ABCA1	CALM3_ABCA1
4718	SP1	CALM2	ABCA1	CALM2_ABCA1
4719	SP1	CALM1	ABCA1	CALM1_ABCA1
4720	SP1	PLTP	ABCA1	PLTP_ABCA1
4721	SP1	CALM3	ABCA1	CALM3_ABCA1
4722	USF2	CALM2	ABCA1	CALM2_ABCA1
4723	USF2	CALM1	ABCA1	CALM1_ABCA1
4724	USF2	PLTP	ABCA1	PLTP_ABCA1
4725	USF2	CALM3	ABCA1	CALM3_ABCA1
4726	RXRA	CALM2	ABCA1	CALM2_ABCA1
4727	RXRA	CALM1	ABCA1	CALM1_ABCA1
4728	RXRA	PLTP	ABCA1	PLTP_ABCA1
4729	RXRA	CALM3	ABCA1	CALM3_ABCA1
4730	NFKB1	GNAS	ADORA1	GNAS_ADORA1
4731	NFKB1	GNAI2	ADORA1	GNAI2_ADORA1
4732	RELA	GNAS	ADORA1	GNAS_ADORA1
4733	RELA	GNAI2	ADORA1	GNAI2_ADORA1
4734	STAT6	GNAI2	ADRA2B	GNAI2_ADRA2B
4735	TFAP2A	GNAI2	ADRA2B	GNAI2_ADRA2B
4736	SP1	GNAI2	ADRA2B	GNAI2_ADRA2B
4737	EZH2	IL1B	ADRB2	IL1B_ADRB2
4738	SP1	GNAS	ADRB3	GNAS_ADRB3
4739	ETV4	UBA52	AGTR1	UBA52_AGTR1
4740	SND1	UBA52	AGTR1	UBA52_AGTR1
4741	SP1	UBA52	AGTR1	UBA52_AGTR1
4742	HIF1A	UBA52	AGTR1	UBA52_AGTR1
4743	SP3	UBA52	AGTR1	UBA52_AGTR1
4744	HDAC3	ALOX5AP	ALOX5	ALOX5AP_ALOX5
4745	HDAC3	PTGS2	ALOX5	PTGS2_ALOX5
4746	EGR1	ALOX5AP	ALOX5	ALOX5AP_ALOX5
4747	SP1	ALOX5AP	ALOX5	ALOX5AP_ALOX5
4748	MBD1	ALOX5AP	ALOX5	ALOX5AP_ALOX5
4749	MBD1	PTGS2	ALOX5	PTGS2_ALOX5
4750	TP53	ALOX5AP	ALOX5	ALOX5AP_ALOX5
4751	TP53	PTGS2	ALOX5	PTGS2_ALOX5
4752	MBD2	ALOX5AP	ALOX5	ALOX5AP_ALOX5
4753	MBD2	PTGS2	ALOX5	PTGS2_ALOX5
4754	HDAC2	ALOX5AP	ALOX5	ALOX5AP_ALOX5
4755	HDAC2	PTGS2	ALOX5	PTGS2_ALOX5

4756	MECP2	ALOX5AP	ALOX5	ALOX5AP_ALOX5
4757	MECP2	PTGS2	ALOX5	PTGS2_ALOX5
4758	EZH2	ADAM10	AXL	ADAM10_AXL
4759	EZH2	GAS6	AXL	GAS6_AXL
4760	EZH2	PROS1	AXL	PROS1_AXL
4761	PPARG	CYR61	CAV1	CYR61_CAV1
4762	PPARG	GNAI2	CAV1	GNAI2_CAV1
4763	PPARG	BST1	CAV1	BST1_CAV1
4764	PPARG	APP	CAV1	APP_CAV1
4765	PPARG	EGF	CAV1	EGF_CAV1
4766	PPARG	TGFB1	CAV1	TGFB1_CAV1
4767	PPARG	HRAS	CAV1	HRAS_CAV1
4768	TFDP1	CYR61	CAV1	CYR61_CAV1
4769	TFDP1	GNAI2	CAV1	GNAI2_CAV1
4770	TFDP1	BST1	CAV1	BST1_CAV1
4771	TFDP1	PTGS2	CAV1	PTGS2_CAV1
4772	TFDP1	APP	CAV1	APP_CAV1
4773	TFDP1	ICAM1	CAV1	ICAM1_CAV1
4774	TFDP1	EGF	CAV1	EGF_CAV1
4775	TFDP1	TGFB1	CAV1	TGFB1_CAV1
4776	TFDP1	HRAS	CAV1	HRAS_CAV1
4777	SP1	CYR61	CAV1	CYR61_CAV1
4778	SP1	GNAI2	CAV1	GNAI2_CAV1
4779	SP1	BST1	CAV1	BST1_CAV1
4780	GATA6	CYR61	CAV1	CYR61_CAV1
4781	GATA6	GNAI2	CAV1	GNAI2_CAV1
4782	GATA6	BST1	CAV1	BST1_CAV1
4783	GATA6	PTGS2	CAV1	PTGS2_CAV1
4784	GATA6	APP	CAV1	APP_CAV1
4785	GATA6	ICAM1	CAV1	ICAM1_CAV1
4786	GATA6	EGF	CAV1	EGF_CAV1
4787	GATA6	TGFB1	CAV1	TGFB1_CAV1
4788	GATA6	HRAS	CAV1	HRAS_CAV1
4789	SREBF1	CYR61	CAV1	CYR61_CAV1
4790	SREBF1	GNAI2	CAV1	GNAI2_CAV1
4791	SREBF1	BST1	CAV1	BST1_CAV1
4792	SREBF1	PTGS2	CAV1	PTGS2_CAV1
4793	SREBF1	APP	CAV1	APP_CAV1
4794	SREBF1	ICAM1	CAV1	ICAM1_CAV1
4795	SREBF1	EGF	CAV1	EGF_CAV1
4796	SREBF1	TGFB1	CAV1	TGFB1_CAV1
4797	SREBF1	HRAS	CAV1	HRAS_CAV1
4798	TP53	CYR61	CAV1	CYR61_CAV1
4799	TP53	GNAI2	CAV1	GNAI2_CAV1
4800	TP53	BST1	CAV1	BST1_CAV1
4801	TP53	PTGS2	CAV1	PTGS2_CAV1
4802	TP53	APP	CAV1	APP_CAV1
4803	TP53	ICAM1	CAV1	ICAM1_CAV1
4804	TP53	EGF	CAV1	EGF_CAV1
4805	TP53	TGFB1	CAV1	TGFB1_CAV1
4806	STAT1	CCL5	CCR1	CCL5_CCR1
4807	STAT1	CCL4	CCR1	CCL4_CCR1
4808	NFKB1	CCL28	CCR3	CCL28_CCR3
4809	RELA	CCL28	CCR3	CCL28_CCR3
4810	GATA1	CCL28	CCR3	CCL28_CCR3
4811	GATA1	CCL3	CCR3	CCL3_CCR3
4812	GATA1	CCL5	CCR3	CCL5_CCR3
4813	GATA1	CCL4	CCR3	CCL4_CCR3

4814	FOSL2	CCL5	CCR4	CCL5_CCR4
4815	FOSL2	CCL3	CCR4	CCL3_CCR4
4816	JUND	CCL3	CCR4	CCL3_CCR4
4817	FOXP3	CCL5	CCR4	CCL5_CCR4
4818	FOXP3	CCL3	CCR4	CCL3_CCR4
4819	TBX21	CCL5	CCR4	CCL5_CCR4
4820	TBX21	CCL3	CCR4	CCL3_CCR4
4821	YY1	CCL3	CCR5	CCL3_CCR5
4822	YY1	CCL3L3	CCR5	CCL3L3_CCR5
4823	YY1	GNAI2	CCR5	GNAI2_CCR5
4824	YY1	IL16	CCR5	IL16_CCR5
4825	YY1	CCL4	CCR5	CCL4_CCR5
4826	YY1	CCL5	CCR5	CCL5_CCR5
4827	KLF2	CCL3	CCR5	CCL3_CCR5
4828	KLF2	CCL3L3	CCR5	CCL3L3_CCR5
4829	KLF2	GNAI2	CCR5	GNAI2_CCR5
4830	KLF2	IL16	CCR5	IL16_CCR5
4831	KLF2	CCL4	CCR5	CCL4_CCR5
4832	KLF2	CCL5	CCR5	CCL5_CCR5
4833	NFKB1	CCL3L3	CCR5	CCL3L3_CCR5
4834	NFKB1	GNAI2	CCR5	GNAI2_CCR5
4835	NFKB1	IL16	CCR5	IL16_CCR5
4836	RELA	CCL3L3	CCR5	CCL3L3_CCR5
4837	RELA	GNAI2	CCR5	GNAI2_CCR5
4838	RELA	IL16	CCR5	IL16_CCR5
4839	NR3C2	CCL3	CCR5	CCL3_CCR5
4840	NR3C2	CCL3L3	CCR5	CCL3L3_CCR5
4841	NR3C2	GNAI2	CCR5	GNAI2_CCR5
4842	NR3C2	IL16	CCR5	IL16_CCR5
4843	NR3C2	CCL4	CCR5	CCL4_CCR5
4844	NR3C2	CCL5	CCR5	CCL5_CCR5
4845	NR3C2	DEFB1	CCR6	DEFB1_CCR6
4846	NR3C2	DEFB103B	CCR6	DEFB103B_CCR6
4847	NR3C2	DEFB103A	CCR6	DEFB103A_CCR6
4848	NR3C2	DEFB4B	CCR6	DEFB4B_CCR6
4849	NR3C2	DEFB4A	CCR6	DEFB4A_CCR6
4850	NR3C2	CCL20	CCR6	CCL20_CCR6
4851	RELA	CCL21	CCR7	CCL21_CCR7
4852	NFKB1	CCL21	CCR7	CCL21_CCR7
4853	KLF2	CCL19	CCR7	CCL19_CCR7
4854	KLF2	CCL21	CCR7	CCL21_CCR7
4855	TRERF1	CCL19	CCR7	CCL19_CCR7
4856	TRERF1	CCL21	CCR7	CCL21_CCR7
4857	HIF1A	CCL19	CCR7	CCL19_CCR7
4858	HIF1A	CCL21	CCR7	CCL21_CCR7
4859	EPAS1	CCL19	CCR7	CCL19_CCR7
4860	EPAS1	CCL21	CCR7	CCL21_CCR7
4861	SP1	LAMC2	CD151	LAMC2_CD151
4862	SP1	DUSP18	CD151	DUSP18_CD151
4863	SP1	MMP7	CD151	MMP7_CD151
4864	SP1	LAMB3	CD151	LAMB3_CD151
4865	PAX5	C3	CD19	C3_CD19
4866	APEX1	C3	CD19	C3_CD19
4867	CREM	B2M	CD247	B2M_CD247
4868	CREM	BTLA	CD247	BTLA_CD247
4869	SP1	IL12A	CD28	IL12A_CD28
4870	EGR1	IL12A	CD28	IL12A_CD28
4871	PPARG	COL1A1	CD36	COL1A1_CD36

4872	PPARG	THBS1	CD36	THBS1_CD36
4873	PPARG	COL1A2	CD36	COL1A2_CD36
4874	PPARA	COL1A1	CD36	COL1A1_CD36
4875	PPARA	THBS1	CD36	THBS1_CD36
4876	PPARA	COL1A2	CD36	COL1A2_CD36
4877	RUNX3	COL1A1	CD36	COL1A1_CD36
4878	RUNX3	THBS1	CD36	THBS1_CD36
4879	RUNX3	COL1A2	CD36	COL1A2_CD36
4880	NFATC2	HLA-B	CD3G	HLA-B_CD3G
4881	NFATC2	HLA-A	CD3G	HLA-A_CD3G
4882	NFATC2	HLA-C	CD3G	HLA-C_CD3G
4883	NFATC2	B2M	CD3G	B2M_CD3G
4884	NFATC1	HLA-B	CD3G	HLA-B_CD3G
4885	NFATC1	HLA-A	CD3G	HLA-A_CD3G
4886	NFATC1	HLA-C	CD3G	HLA-C_CD3G
4887	NFATC1	B2M	CD3G	B2M_CD3G
4888	NFKB1	HLA-B	CD3G	HLA-B_CD3G
4889	NFKB1	HLA-A	CD3G	HLA-A_CD3G
4890	NFKB1	HLA-C	CD3G	HLA-C_CD3G
4891	MAZ	CXCL12	CD4	CXCL12_CD4
4892	MAZ	IL16	CD4	IL16_CD4
4893	MAZ	PIP	CD4	PIP_CD4
4894	MAZ	HLA-G	CD4	HLA-G_CD4
4895	MAZ	SLPI	CD4	SLPI_CD4
4896	TCF3	CXCL12	CD4	CXCL12_CD4
4897	TCF3	IL16	CD4	IL16_CD4
4898	TCF3	PIP	CD4	PIP_CD4
4899	TCF3	HLA-G	CD4	HLA-G_CD4
4900	TCF3	SLPI	CD4	SLPI_CD4
4901	EIF2AK2	CXCL12	CD4	CXCL12_CD4
4902	EIF2AK2	IL16	CD4	IL16_CD4
4903	EIF2AK2	PIP	CD4	PIP_CD4
4904	EIF2AK2	HLA-G	CD4	HLA-G_CD4
4905	EIF2AK2	SLPI	CD4	SLPI_CD4
4906	MYB	CXCL12	CD4	CXCL12_CD4
4907	MYB	IL16	CD4	IL16_CD4
4908	MYB	PIP	CD4	PIP_CD4
4909	MYB	HLA-G	CD4	HLA-G_CD4
4910	MYB	SLPI	CD4	SLPI_CD4
4911	CREB1	CXCL12	CD4	CXCL12_CD4
4912	CREB1	IL16	CD4	IL16_CD4
4913	CREB1	PIP	CD4	PIP_CD4
4914	CREB1	HLA-G	CD4	HLA-G_CD4
4915	CREB1	SLPI	CD4	SLPI_CD4
4916	HES1	CXCL12	CD4	CXCL12_CD4
4917	HES1	IL16	CD4	IL16_CD4
4918	HES1	PIP	CD4	PIP_CD4
4919	HES1	HLA-G	CD4	HLA-G_CD4
4920	HES1	SLPI	CD4	SLPI_CD4
4921	RELA	C4BPA	CD40	C4BPA_CD40
4922	RELA	LTB	CD40	LTB_CD40
4923	STAT1	C4BPA	CD40	C4BPA_CD40
4924	STAT1	LTB	CD40	LTB_CD40
4925	SPI1	CD40LG	CD40	CD40LG_CD40
4926	SPI1	C4BPA	CD40	C4BPA_CD40
4927	SPI1	LTB	CD40	LTB_CD40
4928	NFKB1	C4BPA	CD40	C4BPA_CD40
4929	NFKB1	LTB	CD40	LTB_CD40

4930	XRCC6	CD40LG	CD40	CD40LG_CD40
4931	XRCC6	C4BPA	CD40	C4BPA_CD40
4932	XRCC6	LTB	CD40	LTB_CD40
4933	NFKBIA	CD40LG	CD40	CD40LG_CD40
4934	NFKBIA	C4BPA	CD40	C4BPA_CD40
4935	NFKBIA	LTB	CD40	LTB_CD40
4936	IRF1	CD40LG	CD40	CD40LG_CD40
4937	IRF1	C4BPA	CD40	C4BPA_CD40
4938	IRF1	LTB	CD40	LTB_CD40
4939	TRERF1	CD40LG	CD40	CD40LG_CD40
4940	TRERF1	C4BPA	CD40	C4BPA_CD40
4941	TRERF1	LTB	CD40	LTB_CD40
4942	STAT6	CD40LG	CD40	CD40LG_CD40
4943	STAT6	C4BPA	CD40	C4BPA_CD40
4944	STAT6	LTB	CD40	LTB_CD40
4945	XRCC5	CD40LG	CD40	CD40LG_CD40
4946	XRCC5	C4BPA	CD40	C4BPA_CD40
4947	XRCC5	LTB	CD40	LTB_CD40
4948	SPIB	CD40LG	CD40	CD40LG_CD40
4949	SPIB	C4BPA	CD40	C4BPA_CD40
4950	SPIB	LTB	CD40	LTB_CD40
4951	RELB	CD40LG	CD40	CD40LG_CD40
4952	RELB	C4BPA	CD40	C4BPA_CD40
4953	RELB	LTB	CD40	LTB_CD40
4954	TRAF6	CD40LG	CD40	CD40LG_CD40
4955	TRAF6	C4BPA	CD40	C4BPA_CD40
4956	TRAF6	LTB	CD40	LTB_CD40
4957	NR3C2	CD40LG	CD40	CD40LG_CD40
4958	NR3C2	C4BPA	CD40	C4BPA_CD40
4959	NR3C2	LTB	CD40	LTB_CD40
4960	TWIST2	HAS2	CD44	HAS2_CD44
4961	TWIST2	COL1A2	CD44	COL1A2_CD44
4962	TWIST2	VIM	CD44	VIM_CD44
4963	TWIST2	COL14A1	CD44	COL14A1_CD44
4964	TWIST2	SPP1	CD44	SPP1_CD44
4965	TWIST2	MMP9	CD44	MMP9_CD44
4966	TWIST2	FGF2	CD44	FGF2_CD44
4967	TWIST2	FGF1	CD44	FGF1_CD44
4968	TWIST2	PKM	CD44	PKM_CD44
4969	TWIST2	HBEGF	CD44	HBEGF_CD44
4970	TWIST2	MMP1	CD44	MMP1_CD44
4971	TWIST2	VCAN	CD44	VCAN_CD44
4972	TWIST2	MADCAM1	CD44	MADCAM1_CD44
4973	TWIST2	MMP7	CD44	MMP7_CD44
4974	TWIST2	HGF	CD44	HGF_CD44
4975	TWIST2	COL1A1	CD44	COL1A1_CD44
4976	TWIST2	PSEN1	CD44	PSEN1_CD44
4977	TWIST2	LPL	CD44	LPL_CD44
4978	TWIST1	HAS2	CD44	HAS2_CD44
4979	TWIST1	COL1A2	CD44	COL1A2_CD44
4980	TWIST1	VIM	CD44	VIM_CD44
4981	TWIST1	COL14A1	CD44	COL14A1_CD44
4982	TWIST1	SPP1	CD44	SPP1_CD44
4983	TWIST1	MMP9	CD44	MMP9_CD44
4984	TWIST1	FGF2	CD44	FGF2_CD44
4985	TWIST1	FGF1	CD44	FGF1_CD44
4986	TWIST1	PKM	CD44	PKM_CD44
4987	TWIST1	HBEGF	CD44	HBEGF_CD44

4988	TWIST1	VCAN	CD44	VCAN_CD44
4989	TWIST1	MADCAM1	CD44	MADCAM1_CD44
4990	TWIST1	MMP7	CD44	MMP7_CD44
4991	TWIST1	HGF	CD44	HGF_CD44
4992	TWIST1	COL1A1	CD44	COL1A1_CD44
4993	TWIST1	PSEN1	CD44	PSEN1_CD44
4994	TWIST1	LPL	CD44	LPL_CD44
4995	SMARCA4	HAS2	CD44	HAS2_CD44
4996	SMARCA4	COL1A2	CD44	COL1A2_CD44
4997	SMARCA4	VIM	CD44	VIM_CD44
4998	SMARCA4	COL14A1	CD44	COL14A1_CD44
4999	SMARCA4	SPP1	CD44	SPP1_CD44
5000	SMARCA4	MMP9	CD44	MMP9_CD44
5001	SMARCA4	FGF2	CD44	FGF2_CD44
5002	SMARCA4	FGF1	CD44	FGF1_CD44
5003	SMARCA4	PKM	CD44	PKM_CD44
5004	SMARCA4	HBEGF	CD44	HBEGF_CD44
5005	SMARCA4	MMP1	CD44	MMP1_CD44
5006	SMARCA4	VCAN	CD44	VCAN_CD44
5007	SMARCA4	FN1	CD44	FN1_CD44
5008	SMARCA4	MADCAM1	CD44	MADCAM1_CD44
5009	SMARCA4	MMP7	CD44	MMP7_CD44
5010	SMARCA4	HGF	CD44	HGF_CD44
5011	SMARCA4	COL1A1	CD44	COL1A1_CD44
5012	SMARCA4	PSEN1	CD44	PSEN1_CD44
5013	SMARCA4	LPL	CD44	LPL_CD44
5014	SMARCA1	HAS2	CD44	HAS2_CD44
5015	SMARCA1	COL1A2	CD44	COL1A2_CD44
5016	SMARCA1	VIM	CD44	VIM_CD44
5017	SMARCA1	COL14A1	CD44	COL14A1_CD44
5018	SMARCA1	SPP1	CD44	SPP1_CD44
5019	SMARCA1	MMP9	CD44	MMP9_CD44
5020	SMARCA1	FGF2	CD44	FGF2_CD44
5021	SMARCA1	FGF1	CD44	FGF1_CD44
5022	SMARCA1	PKM	CD44	PKM_CD44
5023	SMARCA1	HBEGF	CD44	HBEGF_CD44
5024	SMARCA1	MMP1	CD44	MMP1_CD44
5025	SMARCA1	VCAN	CD44	VCAN_CD44
5026	SMARCA1	FN1	CD44	FN1_CD44
5027	SMARCA1	MADCAM1	CD44	MADCAM1_CD44
5028	SMARCA1	MMP7	CD44	MMP7_CD44
5029	SMARCA1	HGF	CD44	HGF_CD44
5030	SMARCA1	COL1A1	CD44	COL1A1_CD44
5031	SMARCA1	PSEN1	CD44	PSEN1_CD44
5032	SMARCA1	LPL	CD44	LPL_CD44
5033	SP1	COL1A2	CD44	COL1A2_CD44
5034	SP1	COL14A1	CD44	COL14A1_CD44
5035	SP1	FGF2	CD44	FGF2_CD44
5036	SP1	FGF1	CD44	FGF1_CD44
5037	SP1	PKM	CD44	PKM_CD44
5038	SP1	MMP1	CD44	MMP1_CD44
5039	SP1	VCAN	CD44	VCAN_CD44
5040	SP1	FN1	CD44	FN1_CD44
5041	SP1	MADCAM1	CD44	MADCAM1_CD44
5042	SP1	MMP7	CD44	MMP7_CD44
5043	TCF4	HAS2	CD44	HAS2_CD44
5044	TCF4	COL1A2	CD44	COL1A2_CD44
5045	TCF4	COL14A1	CD44	COL14A1_CD44

5046 TCF4	SPP1	CD44	SPP1_CD44
5047 TCF4	MMP9	CD44	MMP9_CD44
5048 TCF4	FGF2	CD44	FGF2_CD44
5049 TCF4	FGF1	CD44	FGF1_CD44
5050 TCF4	PKM	CD44	PKM_CD44
5051 TCF4	HBEGF	CD44	HBEGF_CD44
5052 TCF4	MMP1	CD44	MMP1_CD44
5053 TCF4	FN1	CD44	FN1_CD44
5054 TCF4	MADCAM1	CD44	MADCAM1_CD44
5055 TCF4	MMP7	CD44	MMP7_CD44
5056 TCF4	HGF	CD44	HGF_CD44
5057 TCF4	COL1A1	CD44	COL1A1_CD44
5058 TCF4	PSEN1	CD44	PSEN1_CD44
5059 TCF4	LPL	CD44	LPL_CD44
5060 CTNNB1	HAS2	CD44	HAS2_CD44
5061 CTNNB1	COL1A2	CD44	COL1A2_CD44
5062 CTNNB1	COL14A1	CD44	COL14A1_CD44
5063 CTNNB1	SPP1	CD44	SPP1_CD44
5064 CTNNB1	MMP9	CD44	MMP9_CD44
5065 CTNNB1	FGF2	CD44	FGF2_CD44
5066 CTNNB1	FGF1	CD44	FGF1_CD44
5067 CTNNB1	PKM	CD44	PKM_CD44
5068 CTNNB1	HBEGF	CD44	HBEGF_CD44
5069 CTNNB1	MMP1	CD44	MMP1_CD44
5070 CTNNB1	VCAN	CD44	VCAN_CD44
5071 CTNNB1	FN1	CD44	FN1_CD44
5072 CTNNB1	MADCAM1	CD44	MADCAM1_CD44
5073 CTNNB1	HGF	CD44	HGF_CD44
5074 CTNNB1	COL1A1	CD44	COL1A1_CD44
5075 CTNNB1	PSEN1	CD44	PSEN1_CD44
5076 CTNNB1	LPL	CD44	LPL_CD44
5077 SNAI2	HAS2	CD44	HAS2_CD44
5078 SNAI2	COL1A2	CD44	COL1A2_CD44
5079 SNAI2	VIM	CD44	VIM_CD44
5080 SNAI2	COL14A1	CD44	COL14A1_CD44
5081 SNAI2	SPP1	CD44	SPP1_CD44
5082 SNAI2	FGF2	CD44	FGF2_CD44
5083 SNAI2	FGF1	CD44	FGF1_CD44
5084 SNAI2	PKM	CD44	PKM_CD44
5085 SNAI2	HBEGF	CD44	HBEGF_CD44
5086 SNAI2	MMP1	CD44	MMP1_CD44
5087 SNAI2	VCAN	CD44	VCAN_CD44
5088 SNAI2	FN1	CD44	FN1_CD44
5089 SNAI2	MADCAM1	CD44	MADCAM1_CD44
5090 SNAI2	MMP7	CD44	MMP7_CD44
5091 SNAI2	HGF	CD44	HGF_CD44
5092 SNAI2	COL1A1	CD44	COL1A1_CD44
5093 SNAI2	PSEN1	CD44	PSEN1_CD44
5094 SNAI2	LPL	CD44	LPL_CD44
5095 HMGA1	HAS2	CD44	HAS2_CD44
5096 HMGA1	COL1A2	CD44	COL1A2_CD44
5097 HMGA1	VIM	CD44	VIM_CD44
5098 HMGA1	COL14A1	CD44	COL14A1_CD44
5099 HMGA1	SPP1	CD44	SPP1_CD44
5100 HMGA1	MMP9	CD44	MMP9_CD44
5101 HMGA1	FGF2	CD44	FGF2_CD44
5102 HMGA1	FGF1	CD44	FGF1_CD44
5103 HMGA1	PKM	CD44	PKM_CD44

5104	HMGA1	HBEGF	CD44	HBEGF_CD44
5105	HMGA1	MMP1	CD44	MMP1_CD44
5106	HMGA1	VCAN	CD44	VCAN_CD44
5107	HMGA1	FN1	CD44	FN1_CD44
5108	HMGA1	MADCAM1	CD44	MADCAM1_CD44
5109	HMGA1	MMP7	CD44	MMP7_CD44
5110	HMGA1	HGF	CD44	HGF_CD44
5111	HMGA1	COL1A1	CD44	COL1A1_CD44
5112	HMGA1	PSEN1	CD44	PSEN1_CD44
5113	HMGA1	LPL	CD44	LPL_CD44
5114	HDAC1	HAS2	CD44	HAS2_CD44
5115	HDAC1	VIM	CD44	VIM_CD44
5116	HDAC1	COL14A1	CD44	COL14A1_CD44
5117	HDAC1	FGF2	CD44	FGF2_CD44
5118	HDAC1	FGF1	CD44	FGF1_CD44
5119	HDAC1	PKM	CD44	PKM_CD44
5120	HDAC1	HBEGF	CD44	HBEGF_CD44
5121	HDAC1	MMP1	CD44	MMP1_CD44
5122	HDAC1	VCAN	CD44	VCAN_CD44
5123	HDAC1	FN1	CD44	FN1_CD44
5124	HDAC1	MADCAM1	CD44	MADCAM1_CD44
5125	HDAC1	MMP7	CD44	MMP7_CD44
5126	HDAC1	HGF	CD44	HGF_CD44
5127	HDAC1	COL1A1	CD44	COL1A1_CD44
5128	HDAC1	PSEN1	CD44	PSEN1_CD44
5129	HDAC1	LPL	CD44	LPL_CD44
5130	IKBKB	HAS2	CD44	HAS2_CD44
5131	IKBKB	COL1A2	CD44	COL1A2_CD44
5132	IKBKB	VIM	CD44	VIM_CD44
5133	IKBKB	COL14A1	CD44	COL14A1_CD44
5134	IKBKB	SPP1	CD44	SPP1_CD44
5135	IKBKB	FGF2	CD44	FGF2_CD44
5136	IKBKB	FGF1	CD44	FGF1_CD44
5137	IKBKB	PKM	CD44	PKM_CD44
5138	IKBKB	HBEGF	CD44	HBEGF_CD44
5139	IKBKB	MMP1	CD44	MMP1_CD44
5140	IKBKB	VCAN	CD44	VCAN_CD44
5141	IKBKB	FN1	CD44	FN1_CD44
5142	IKBKB	MADCAM1	CD44	MADCAM1_CD44
5143	IKBKB	MMP7	CD44	MMP7_CD44
5144	IKBKB	HGF	CD44	HGF_CD44
5145	IKBKB	COL1A1	CD44	COL1A1_CD44
5146	IKBKB	PSEN1	CD44	PSEN1_CD44
5147	IKBKB	LPL	CD44	LPL_CD44
5148	SMARCB1	HAS2	CD44	HAS2_CD44
5149	SMARCB1	COL1A2	CD44	COL1A2_CD44
5150	SMARCB1	VIM	CD44	VIM_CD44
5151	SMARCB1	COL14A1	CD44	COL14A1_CD44
5152	SMARCB1	SPP1	CD44	SPP1_CD44
5153	SMARCB1	MMP9	CD44	MMP9_CD44
5154	SMARCB1	FGF2	CD44	FGF2_CD44
5155	SMARCB1	FGF1	CD44	FGF1_CD44
5156	SMARCB1	PKM	CD44	PKM_CD44
5157	SMARCB1	HBEGF	CD44	HBEGF_CD44
5158	SMARCB1	MMP1	CD44	MMP1_CD44
5159	SMARCB1	VCAN	CD44	VCAN_CD44
5160	SMARCB1	FN1	CD44	FN1_CD44
5161	SMARCB1	MADCAM1	CD44	MADCAM1_CD44

5162	SMARCB1	MMP7	CD44	MMP7_CD44
5163	SMARCB1	HGF	CD44	HGF_CD44
5164	SMARCB1	COL1A1	CD44	COL1A1_CD44
5165	SMARCB1	PSEN1	CD44	PSEN1_CD44
5166	SMARCB1	LPL	CD44	LPL_CD44
5167	MYCN	HAS2	CD44	HAS2_CD44
5168	MYCN	COL1A2	CD44	COL1A2_CD44
5169	MYCN	VIM	CD44	VIM_CD44
5170	MYCN	COL14A1	CD44	COL14A1_CD44
5171	MYCN	SPP1	CD44	SPP1_CD44
5172	MYCN	MMP9	CD44	MMP9_CD44
5173	MYCN	FGF2	CD44	FGF2_CD44
5174	MYCN	FGF1	CD44	FGF1_CD44
5175	MYCN	PKM	CD44	PKM_CD44
5176	MYCN	HBEGF	CD44	HBEGF_CD44
5177	MYCN	MMP1	CD44	MMP1_CD44
5178	MYCN	VCAN	CD44	VCAN_CD44
5179	MYCN	FN1	CD44	FN1_CD44
5180	MYCN	MADCAM1	CD44	MADCAM1_CD44
5181	MYCN	MMP7	CD44	MMP7_CD44
5182	MYCN	HGF	CD44	HGF_CD44
5183	MYCN	COL1A1	CD44	COL1A1_CD44
5184	MYCN	PSEN1	CD44	PSEN1_CD44
5185	MYCN	LPL	CD44	LPL_CD44
5186	STAT3	C3	CD46	C3_CD46
5187	NFKB1	APP	CD74	APP_CD74
5188	RELA	APP	CD74	APP_CD74
5189	CHD4	BTLA	CD79A	BTLA_CD79A
5190	CHD4	FN1	CD79A	FN1_CD79A
5191	CHD4	IL8	CD79A	IL8_CD79A
5192	PAX5	BTLA	CD79A	BTLA_CD79A
5193	PAX5	FN1	CD79A	FN1_CD79A
5194	PAX5	IL8	CD79A	IL8_CD79A
5195	TP53	HBEGF	CD82	HBEGF_CD82
5196	HDAC1	HBEGF	CD82	HBEGF_CD82
5197	TFAP2A	HBEGF	CD82	HBEGF_CD82
5198	JUNB	HBEGF	CD82	HBEGF_CD82
5199	ATF3	HBEGF	CD82	HBEGF_CD82
5200	JUN	HBEGF	CD82	HBEGF_CD82
5201	SP1	NCAN	CDH2	NCAN_CDH2
5202	SP1	CDH1	CDH2	CDH1_CDH2
5203	TWIST2	NCAN	CDH2	NCAN_CDH2
5204	TWIST1	NCAN	CDH2	NCAN_CDH2
5205	SP3	NCAN	CDH2	NCAN_CDH2
5206	SP3	CDH1	CDH2	CDH1_CDH2
5207	KLF8	NCAN	CDH2	NCAN_CDH2
5208	MZF1	NCAN	CDH2	NCAN_CDH2
5209	MZF1	CDH1	CDH2	CDH1_CDH2
5210	SNAI1	NCAN	CDH2	NCAN_CDH2
5211	MSX2	NCAN	CDH2	NCAN_CDH2
5212	MSX2	CDH1	CDH2	CDH1_CDH2
5213	SNAI2	NCAN	CDH2	NCAN_CDH2
5214	AR	NCAN	CDH2	NCAN_CDH2
5215	DNMT3A	MMP7	CDH6	MMP7_CDH6
5216	SP3	HSP90AA1	CFTR	HSP90AA1_CFTR
5217	CDX2	HSP90AA1	CFTR	HSP90AA1_CFTR
5218	FOXA2	HSP90AA1	CFTR	HSP90AA1_CFTR
5219	SP1	HSP90AA1	CFTR	HSP90AA1_CFTR

5220	FOXI1	HSP90AA1	CFTR	HSP90AA1_CFTR
5221	ATF1	HSP90AA1	CFTR	HSP90AA1_CFTR
5222	CUX1	HSP90AA1	CFTR	HSP90AA1_CFTR
5223	RELA	HSP90AA1	CFTR	HSP90AA1_CFTR
5224	NFKB1	HSP90AA1	CFTR	HSP90AA1_CFTR
5225	TFAP2A	HSP90AA1	CFTR	HSP90AA1_CFTR
5226	YY1	HSP90AA1	CFTR	HSP90AA1_CFTR
5227	NFE2L2	HSP90AA1	CFTR	HSP90AA1_CFTR
5228	SRF	HSP90AA1	CFTR	HSP90AA1_CFTR
5229	FOXA1	HSP90AA1	CFTR	HSP90AA1_CFTR
5230	USF2	HSP90AA1	CFTR	HSP90AA1_CFTR
5231	STAT6	GNAI2	CNR1	GNAI2_CNR1
5232	STAT6	TCN2	CNR1	TCN2_CNR1
5233	ETS1	CD55	CR1	CD55_CR1
5234	ETS1	C3	CR1	C3_CR1
5235	ETS1	C1QA	CR1	C1QA_CR1
5236	RELA	GNAS	CRHR1	GNAS_CRHR1
5237	RELA	CALM1	CRHR1	CALM1_CRHR1
5238	NFKB1	GNAS	CRHR1	GNAS_CRHR1
5239	NFKB1	CALM1	CRHR1	CALM1_CRHR1
5240	RUNX1	IL34	CSF1R	IL34_CSF1R
5241	RUNX1	CSF3	CSF1R	CSF3_CSF1R
5242	NR3C1	IL34	CSF1R	IL34_CSF1R
5243	NR3C1	CSF2	CSF1R	CSF2_CSF1R
5244	NR3C1	CSF3	CSF1R	CSF3_CSF1R
5245	NR3C1	CSF1	CSF1R	CSF1_CSF1R
5246	CEBPA	CSF2	CSF2RA	CSF2_CSF2RA
5247	SPI1	CSF2	CSF2RA	CSF2_CSF2RA
5248	CEBPA	CSF2	CSF3R	CSF2_CSF3R
5249	CEBPA	CSF3	CSF3R	CSF3_CSF3R
5250	MYB	CSF2	CSF3R	CSF2_CSF3R
5251	MYB	CSF3	CSF3R	CSF3_CSF3R
5252	CEBPB	CSF2	CSF3R	CSF2_CSF3R
5253	CEBPB	CSF3	CSF3R	CSF3_CSF3R
5254	SPI1	CSF2	CSF3R	CSF2_CSF3R
5255	SPI1	CSF3	CSF3R	CSF3_CSF3R
5256	ETS2	CSF3	CSF3R	CSF3_CSF3R
5257	ETS1	CSF3	CSF3R	CSF3_CSF3R
5258	SPI1	GNAI2	CXCR1	GNAI2_CXCR1
5259	NFKB1	VASP	CXCR2	VASP_CXCR2
5260	NFKB1	GNAI2	CXCR2	GNAI2_CXCR2
5261	CEBPA	VASP	CXCR2	VASP_CXCR2
5262	CEBPA	GNAI2	CXCR2	GNAI2_CXCR2
5263	USF2	VASP	CXCR2	VASP_CXCR2
5264	USF2	GNAI2	CXCR2	GNAI2_CXCR2
5265	RELA	VASP	CXCR2	VASP_CXCR2
5266	RELA	GNAI2	CXCR2	GNAI2_CXCR2
5267	TBX21	CCL5	CXCR3	CCL5_CXCR3
5268	TBX21	CCL20	CXCR3	CCL20_CXCR3
5269	TBX21	CCL21	CXCR3	CCL21_CXCR3
5270	TBX21	CCL13	CXCR3	CCL13_CXCR3
5271	TBX21	CXCL12	CXCR3	CXCL12_CXCR3
5272	TBX21	GNAI2	CXCR3	GNAI2_CXCR3
5273	TBX21	PF4	CXCR3	PF4_CXCR3
5274	TBX21	CCL19	CXCR3	CCL19_CXCR3
5275	TBX21	CXCL9	CXCR3	CXCL9_CXCR3
5276	TBX21	CXCL11	CXCR3	CXCL11_CXCR3
5277	TBX21	CXCL10	CXCR3	CXCL10_CXCR3

5278	TBX21	CCL11	CXCR3	CCL11_CXCR3
5279	TBX21	CXCL13	CXCR3	CXCL13_CXCR3
5280	TBX21	CCL7	CXCR3	CCL7_CXCR3
5281	NRF1	CXCL12	CXCR4	CXCL12_CXCR4
5282	NRF1	CEL	CXCR4	CEL_CXCR4
5283	NRF1	ZG16B	CXCR4	ZG16B_CXCR4
5284	NRF1	TGFB1	CXCR4	TGFB1_CXCR4
5285	USF2	CXCL12	CXCR4	CXCL12_CXCR4
5286	USF2	CEL	CXCR4	CEL_CXCR4
5287	USF2	ZG16B	CXCR4	ZG16B_CXCR4
5288	HIF1A	CEL	CXCR4	CEL_CXCR4
5289	HIF1A	ZG16B	CXCR4	ZG16B_CXCR4
5290	HIF1A	TGFB1	CXCR4	TGFB1_CXCR4
5291	ZBTB16	CXCL12	CXCR4	CXCL12_CXCR4
5292	ZBTB16	CEL	CXCR4	CEL_CXCR4
5293	ZBTB16	ZG16B	CXCR4	ZG16B_CXCR4
5294	ZBTB16	TGFB1	CXCR4	TGFB1_CXCR4
5295	CREB1	CXCL12	CXCR4	CXCL12_CXCR4
5296	CREB1	CEL	CXCR4	CEL_CXCR4
5297	CREB1	ZG16B	CXCR4	ZG16B_CXCR4
5298	CREB1	TGFB1	CXCR4	TGFB1_CXCR4
5299	YY1	CXCL12	CXCR4	CXCL12_CXCR4
5300	YY1	CEL	CXCR4	CEL_CXCR4
5301	YY1	ZG16B	CXCR4	ZG16B_CXCR4
5302	YY1	TGFB1	CXCR4	TGFB1_CXCR4
5303	NFKB1	CEL	CXCR4	CEL_CXCR4
5304	NFKB1	ZG16B	CXCR4	ZG16B_CXCR4
5305	KLF2	CXCL12	CXCR4	CXCL12_CXCR4
5306	KLF2	CEL	CXCR4	CEL_CXCR4
5307	KLF2	ZG16B	CXCR4	ZG16B_CXCR4
5308	KLF2	TGFB1	CXCR4	TGFB1_CXCR4
5309	YBX1	CXCL12	CXCR4	CXCL12_CXCR4
5310	YBX1	CEL	CXCR4	CEL_CXCR4
5311	YBX1	ZG16B	CXCR4	ZG16B_CXCR4
5312	YBX1	TGFB1	CXCR4	TGFB1_CXCR4
5313	DNMT1	CXCL12	CXCR4	CXCL12_CXCR4
5314	DNMT1	CEL	CXCR4	CEL_CXCR4
5315	DNMT1	ZG16B	CXCR4	ZG16B_CXCR4
5316	DNMT1	TGFB1	CXCR4	TGFB1_CXCR4
5317	CREB3	CXCL12	CXCR4	CXCL12_CXCR4
5318	CREB3	CEL	CXCR4	CEL_CXCR4
5319	CREB3	ZG16B	CXCR4	ZG16B_CXCR4
5320	CREB3	TGFB1	CXCR4	TGFB1_CXCR4
5321	SNAI2	CEL	CXCR4	CEL_CXCR4
5322	SNAI2	ZG16B	CXCR4	ZG16B_CXCR4
5323	SNAI2	TGFB1	CXCR4	TGFB1_CXCR4
5324	IRF2	CXCL12	CXCR4	CXCL12_CXCR4
5325	IRF2	CEL	CXCR4	CEL_CXCR4
5326	IRF2	ZG16B	CXCR4	ZG16B_CXCR4
5327	IRF2	TGFB1	CXCR4	TGFB1_CXCR4
5328	RELA	CEL	CXCR4	CEL_CXCR4
5329	RELA	ZG16B	CXCR4	ZG16B_CXCR4
5330	RELA	TGFB1	CXCR4	TGFB1_CXCR4
5331	KLF5	CXCL12	CXCR4	CXCL12_CXCR4
5332	KLF5	CEL	CXCR4	CEL_CXCR4
5333	KLF5	ZG16B	CXCR4	ZG16B_CXCR4
5334	KLF5	TGFB1	CXCR4	TGFB1_CXCR4
5335	VHL	CXCL12	CXCR4	CXCL12_CXCR4

5336	VHL	CEL	CXCR4	CEL_CXCR4
5337	VHL	ZG16B	CXCR4	ZG16B_CXCR4
5338	VHL	TGFB1	CXCR4	TGFB1_CXCR4
5339	FOXO1	CXCL12	CXCR4	CXCL12_CXCR4
5340	FOXO1	CEL	CXCR4	CEL_CXCR4
5341	FOXO1	ZG16B	CXCR4	ZG16B_CXCR4
5342	FOXO1	TGFB1	CXCR4	TGFB1_CXCR4
5343	HDAC3	CXCL12	CXCR4	CXCL12_CXCR4
5344	HDAC3	CEL	CXCR4	CEL_CXCR4
5345	HDAC3	ZG16B	CXCR4	ZG16B_CXCR4
5346	HDAC3	TGFB1	CXCR4	TGFB1_CXCR4
5347	USF1	CXCL12	CXCR4	CXCL12_CXCR4
5348	USF1	CEL	CXCR4	CEL_CXCR4
5349	USF1	ZG16B	CXCR4	ZG16B_CXCR4
5350	ETS1	CXCL12	CXCR4	CXCL12_CXCR4
5351	ETS1	CEL	CXCR4	CEL_CXCR4
5352	ETS1	ZG16B	CXCR4	ZG16B_CXCR4
5353	ETS1	TGFB1	CXCR4	TGFB1_CXCR4
5354	MYC	CXCL12	CXCR4	CXCL12_CXCR4
5355	MYC	CEL	CXCR4	CEL_CXCR4
5356	MYC	ZG16B	CXCR4	ZG16B_CXCR4
5357	MYC	TGFB1	CXCR4	TGFB1_CXCR4
5358	PAX3	CXCL12	CXCR4	CXCL12_CXCR4
5359	PAX3	CEL	CXCR4	CEL_CXCR4
5360	PAX3	ZG16B	CXCR4	ZG16B_CXCR4
5361	PAX3	TGFB1	CXCR4	TGFB1_CXCR4
5362	ERG	CXCL12	CXCR4	CXCL12_CXCR4
5363	ERG	CEL	CXCR4	CEL_CXCR4
5364	ERG	ZG16B	CXCR4	ZG16B_CXCR4
5365	ERG	TGFB1	CXCR4	TGFB1_CXCR4
5366	RARA	CXCL13	CXCR5	CXCL13_CXCR5
5367	RXRA	CXCL13	CXCR5	CXCL13_CXCR5
5368	TFAP2A	SEMA4B	DCBLD2	SEMA4B_DCBLD2
5369	NFKB1	GNAI2	DRD2	GNAI2_DRD2
5370	RELA	GNAI2	DRD2	GNAI2_DRD2
5371	SP1	GNAI2	EDNRB	GNAI2_EDNRB
5372	SOX10	GNAI2	EDNRB	GNAI2_EDNRB
5373	HIF1A	GNAI2	EDNRB	GNAI2_EDNRB
5374	BRCA1	FGL1	EGFR	FGL1_EGFR
5375	BRCA1	GNAI2	EGFR	GNAI2_EGFR
5376	BRCA1	CALM2	EGFR	CALM2_EGFR
5377	BRCA1	CDH1	EGFR	CDH1_EGFR
5378	BRCA1	BTC	EGFR	BTC_EGFR
5379	BRCA1	UBA52	EGFR	UBA52_EGFR
5380	BRCA1	CAMP	EGFR	CAMP_EGFR
5381	BRCA1	ANXA1	EGFR	ANXA1_EGFR
5382	BRCA1	AREGB	EGFR	AREGB_EGFR
5383	BRCA1	NRG4	EGFR	NRG4_EGFR
5384	BRCA1	VCAN	EGFR	VCAN_EGFR
5385	BRCA1	ICAM1	EGFR	ICAM1_EGFR
5386	BRCA1	TNC	EGFR	TNC_EGFR
5387	BRCA1	EREG	EGFR	EREG_EGFR
5388	BRCA1	L1CAM	EGFR	L1CAM_EGFR
5389	BRCA1	CALM3	EGFR	CALM3_EGFR
5390	BRCA1	HSP90AA1	EGFR	HSP90AA1_EGFR
5391	BRCA1	AMH	EGFR	AMH_EGFR
5392	BRCA1	CALM1	EGFR	CALM1_EGFR
5393	BRCA1	SPINK1	EGFR	SPINK1_EGFR

5394	BRCA1	FGF1	EGFR	FGF1_EGFR
5395	BRCA1	FGF13	EGFR	FGF13_EGFR
5396	BRCA1	TGFA	EGFR	TGFA_EGFR
5397	BRCA1	HBEGF	EGFR	HBEGF_EGFR
5398	BRCA1	EGF	EGFR	EGF_EGFR
5399	BRCA1	ZP3	EGFR	ZP3_EGFR
5400	BRCA1	BCAN	EGFR	BCAN_EGFR
5401	BRCA1	EPGN	EGFR	EPGN_EGFR
5402	BRCA1	DCN	EGFR	DCN_EGFR
5403	BRCA1	EFEMP1	EGFR	EFEMP1_EGFR
5404	TSG101	FGL1	EGFR	FGL1_EGFR
5405	TSG101	GNAI2	EGFR	GNAI2_EGFR
5406	TSG101	CALM2	EGFR	CALM2_EGFR
5407	TSG101	CDH1	EGFR	CDH1_EGFR
5408	TSG101	BTC	EGFR	BTC_EGFR
5409	TSG101	UBA52	EGFR	UBA52_EGFR
5410	TSG101	CAMP	EGFR	CAMP_EGFR
5411	TSG101	ANXA1	EGFR	ANXA1_EGFR
5412	TSG101	AREGB	EGFR	AREGB_EGFR
5413	TSG101	NRG4	EGFR	NRG4_EGFR
5414	TSG101	VCAN	EGFR	VCAN_EGFR
5415	TSG101	ICAM1	EGFR	ICAM1_EGFR
5416	TSG101	TNC	EGFR	TNC_EGFR
5417	TSG101	EREG	EGFR	EREG_EGFR
5418	TSG101	L1CAM	EGFR	L1CAM_EGFR
5419	TSG101	CALM3	EGFR	CALM3_EGFR
5420	TSG101	HSP90AA1	EGFR	HSP90AA1_EGFR
5421	TSG101	AMH	EGFR	AMH_EGFR
5422	TSG101	CALM1	EGFR	CALM1_EGFR
5423	TSG101	VEGFA	EGFR	VEGFA_EGFR
5424	TSG101	SPINK1	EGFR	SPINK1_EGFR
5425	TSG101	FGF1	EGFR	FGF1_EGFR
5426	TSG101	AREG	EGFR	AREG_EGFR
5427	TSG101	FGF13	EGFR	FGF13_EGFR
5428	TSG101	TGFA	EGFR	TGFA_EGFR
5429	TSG101	HBEGF	EGFR	HBEGF_EGFR
5430	TSG101	EGF	EGFR	EGF_EGFR
5431	TSG101	ZP3	EGFR	ZP3_EGFR
5432	TSG101	BCAN	EGFR	BCAN_EGFR
5433	TSG101	EPGN	EGFR	EPGN_EGFR
5434	TSG101	DCN	EGFR	DCN_EGFR
5435	TSG101	EFEMP1	EGFR	EFEMP1_EGFR
5436	JUNB	FGL1	EGFR	FGL1_EGFR
5437	JUNB	GNAI2	EGFR	GNAI2_EGFR
5438	JUNB	CALM2	EGFR	CALM2_EGFR
5439	JUNB	CDH1	EGFR	CDH1_EGFR
5440	JUNB	BTC	EGFR	BTC_EGFR
5441	JUNB	UBA52	EGFR	UBA52_EGFR
5442	JUNB	CAMP	EGFR	CAMP_EGFR
5443	JUNB	ANXA1	EGFR	ANXA1_EGFR
5444	JUNB	AREGB	EGFR	AREGB_EGFR
5445	JUNB	NRG4	EGFR	NRG4_EGFR
5446	JUNB	VCAN	EGFR	VCAN_EGFR
5447	JUNB	ICAM1	EGFR	ICAM1_EGFR
5448	JUNB	TNC	EGFR	TNC_EGFR
5449	JUNB	EREG	EGFR	EREG_EGFR
5450	JUNB	L1CAM	EGFR	L1CAM_EGFR
5451	JUNB	CALM3	EGFR	CALM3_EGFR

5452 JUNB	HSP90AA1	EGFR	HSP90AA1_EGFR
5453 JUNB	AMH	EGFR	AMH_EGFR
5454 JUNB	CALM1	EGFR	CALM1_EGFR
5455 JUNB	VEGFA	EGFR	VEGFA_EGFR
5456 JUNB	SPINK1	EGFR	SPINK1_EGFR
5457 JUNB	FGF1	EGFR	FGF1_EGFR
5458 JUNB	AREG	EGFR	AREG_EGFR
5459 JUNB	FGF13	EGFR	FGF13_EGFR
5460 JUNB	TGFA	EGFR	TGFA_EGFR
5461 JUNB	HBEGF	EGFR	HBEGF_EGFR
5462 JUNB	EGF	EGFR	EGF_EGFR
5463 JUNB	ZP3	EGFR	ZP3_EGFR
5464 JUNB	BCAN	EGFR	BCAN_EGFR
5465 JUNB	EPGN	EGFR	EPGN_EGFR
5466 JUNB	DCN	EGFR	DCN_EGFR
5467 JUNB	EFEMP1	EGFR	EFEMP1_EGFR
5468 ESR1	FGL1	EGFR	FGL1_EGFR
5469 ESR1	GNAI2	EGFR	GNAI2_EGFR
5470 ESR1	CALM2	EGFR	CALM2_EGFR
5471 ESR1	BTC	EGFR	BTC_EGFR
5472 ESR1	UBA52	EGFR	UBA52_EGFR
5473 ESR1	CAMP	EGFR	CAMP_EGFR
5474 ESR1	ANXA1	EGFR	ANXA1_EGFR
5475 ESR1	AREGB	EGFR	AREGB_EGFR
5476 ESR1	NRG4	EGFR	NRG4_EGFR
5477 ESR1	VCAN	EGFR	VCAN_EGFR
5478 ESR1	ICAM1	EGFR	ICAM1_EGFR
5479 ESR1	TNC	EGFR	TNC_EGFR
5480 ESR1	EREG	EGFR	EREG_EGFR
5481 ESR1	L1CAM	EGFR	L1CAM_EGFR
5482 ESR1	CALM3	EGFR	CALM3_EGFR
5483 ESR1	HSP90AA1	EGFR	HSP90AA1_EGFR
5484 ESR1	CALM1	EGFR	CALM1_EGFR
5485 ESR1	SPINK1	EGFR	SPINK1_EGFR
5486 ESR1	FGF1	EGFR	FGF1_EGFR
5487 ESR1	AREG	EGFR	AREG_EGFR
5488 ESR1	FGF13	EGFR	FGF13_EGFR
5489 ESR1	HBEGF	EGFR	HBEGF_EGFR
5490 ESR1	EGF	EGFR	EGF_EGFR
5491 ESR1	ZP3	EGFR	ZP3_EGFR
5492 ESR1	BCAN	EGFR	BCAN_EGFR
5493 ESR1	EPGN	EGFR	EPGN_EGFR
5494 ESR1	DCN	EGFR	DCN_EGFR
5495 ESR1	EFEMP1	EGFR	EFEMP1_EGFR
5496 YY1	FGL1	EGFR	FGL1_EGFR
5497 YY1	GNAI2	EGFR	GNAI2_EGFR
5498 YY1	CALM2	EGFR	CALM2_EGFR
5499 YY1	CDH1	EGFR	CDH1_EGFR
5500 YY1	BTC	EGFR	BTC_EGFR
5501 YY1	UBA52	EGFR	UBA52_EGFR
5502 YY1	CAMP	EGFR	CAMP_EGFR
5503 YY1	ANXA1	EGFR	ANXA1_EGFR
5504 YY1	AREGB	EGFR	AREGB_EGFR
5505 YY1	NRG4	EGFR	NRG4_EGFR
5506 YY1	VCAN	EGFR	VCAN_EGFR
5507 YY1	ICAM1	EGFR	ICAM1_EGFR
5508 YY1	TNC	EGFR	TNC_EGFR
5509 YY1	EREG	EGFR	EREG_EGFR

5510	YY1	L1CAM	EGFR	L1CAM_EGFR
5511	YY1	CALM3	EGFR	CALM3_EGFR
5512	YY1	HSP90AA1	EGFR	HSP90AA1_EGFR
5513	YY1	AMH	EGFR	AMH_EGFR
5514	YY1	CALM1	EGFR	CALM1_EGFR
5515	YY1	VEGFA	EGFR	VEGFA_EGFR
5516	YY1	SPINK1	EGFR	SPINK1_EGFR
5517	YY1	FGF1	EGFR	FGF1_EGFR
5518	YY1	AREG	EGFR	AREG_EGFR
5519	YY1	FGF13	EGFR	FGF13_EGFR
5520	YY1	TGFA	EGFR	TGFA_EGFR
5521	YY1	HBEGF	EGFR	HBEGF_EGFR
5522	YY1	EGF	EGFR	EGF_EGFR
5523	YY1	ZP3	EGFR	ZP3_EGFR
5524	YY1	BCAN	EGFR	BCAN_EGFR
5525	YY1	EPGN	EGFR	EPGN_EGFR
5526	YY1	DCN	EGFR	DCN_EGFR
5527	YY1	EFEMP1	EGFR	EFEMP1_EGFR
5528	HOXB7	FGL1	EGFR	FGL1_EGFR
5529	HOXB7	GNAI2	EGFR	GNAI2_EGFR
5530	HOXB7	CALM2	EGFR	CALM2_EGFR
5531	HOXB7	CDH1	EGFR	CDH1_EGFR
5532	HOXB7	BTC	EGFR	BTC_EGFR
5533	HOXB7	UBA52	EGFR	UBA52_EGFR
5534	HOXB7	CAMP	EGFR	CAMP_EGFR
5535	HOXB7	ANXA1	EGFR	ANXA1_EGFR
5536	HOXB7	AREGB	EGFR	AREGB_EGFR
5537	HOXB7	NRG4	EGFR	NRG4_EGFR
5538	HOXB7	VCAN	EGFR	VCAN_EGFR
5539	HOXB7	ICAM1	EGFR	ICAM1_EGFR
5540	HOXB7	TNC	EGFR	TNC_EGFR
5541	HOXB7	EREG	EGFR	EREG_EGFR
5542	HOXB7	L1CAM	EGFR	L1CAM_EGFR
5543	HOXB7	CALM3	EGFR	CALM3_EGFR
5544	HOXB7	HSP90AA1	EGFR	HSP90AA1_EGFR
5545	HOXB7	AMH	EGFR	AMH_EGFR
5546	HOXB7	CALM1	EGFR	CALM1_EGFR
5547	HOXB7	SPINK1	EGFR	SPINK1_EGFR
5548	HOXB7	FGF1	EGFR	FGF1_EGFR
5549	HOXB7	AREG	EGFR	AREG_EGFR
5550	HOXB7	FGF13	EGFR	FGF13_EGFR
5551	HOXB7	TGFA	EGFR	TGFA_EGFR
5552	HOXB7	HBEGF	EGFR	HBEGF_EGFR
5553	HOXB7	EGF	EGFR	EGF_EGFR
5554	HOXB7	ZP3	EGFR	ZP3_EGFR
5555	HOXB7	BCAN	EGFR	BCAN_EGFR
5556	HOXB7	EPGN	EGFR	EPGN_EGFR
5557	HOXB7	DCN	EGFR	DCN_EGFR
5558	HOXB7	EFEMP1	EGFR	EFEMP1_EGFR
5559	BCL3	FGL1	EGFR	FGL1_EGFR
5560	BCL3	GNAI2	EGFR	GNAI2_EGFR
5561	BCL3	CALM2	EGFR	CALM2_EGFR
5562	BCL3	CDH1	EGFR	CDH1_EGFR
5563	BCL3	BTC	EGFR	BTC_EGFR
5564	BCL3	UBA52	EGFR	UBA52_EGFR
5565	BCL3	CAMP	EGFR	CAMP_EGFR
5566	BCL3	ANXA1	EGFR	ANXA1_EGFR
5567	BCL3	AREGB	EGFR	AREGB_EGFR

5568	BCL3	NRG4	EGFR	NRG4_EGFR
5569	BCL3	VCAN	EGFR	VCAN_EGFR
5570	BCL3	ICAM1	EGFR	ICAM1_EGFR
5571	BCL3	TNC	EGFR	TNC_EGFR
5572	BCL3	EREG	EGFR	EREG_EGFR
5573	BCL3	L1CAM	EGFR	L1CAM_EGFR
5574	BCL3	CALM3	EGFR	CALM3_EGFR
5575	BCL3	HSP90AA1	EGFR	HSP90AA1_EGFR
5576	BCL3	AMH	EGFR	AMH_EGFR
5577	BCL3	CALM1	EGFR	CALM1_EGFR
5578	BCL3	VEGFA	EGFR	VEGFA_EGFR
5579	BCL3	SPINK1	EGFR	SPINK1_EGFR
5580	BCL3	FGF1	EGFR	FGF1_EGFR
5581	BCL3	AREG	EGFR	AREG_EGFR
5582	BCL3	FGF13	EGFR	FGF13_EGFR
5583	BCL3	TGFA	EGFR	TGFA_EGFR
5584	BCL3	HBEGF	EGFR	HBEGF_EGFR
5585	BCL3	EGF	EGFR	EGF_EGFR
5586	BCL3	ZP3	EGFR	ZP3_EGFR
5587	BCL3	BCAN	EGFR	BCAN_EGFR
5588	BCL3	EPGN	EGFR	EPGN_EGFR
5589	BCL3	DCN	EGFR	DCN_EGFR
5590	BCL3	EFEMP1	EGFR	EFEMP1_EGFR
5591	YBX1	FGL1	EGFR	FGL1_EGFR
5592	YBX1	GNAI2	EGFR	GNAI2_EGFR
5593	YBX1	CALM2	EGFR	CALM2_EGFR
5594	YBX1	BTC	EGFR	BTC_EGFR
5595	YBX1	UBA52	EGFR	UBA52_EGFR
5596	YBX1	CAMP	EGFR	CAMP_EGFR
5597	YBX1	ANXA1	EGFR	ANXA1_EGFR
5598	YBX1	AREGB	EGFR	AREGB_EGFR
5599	YBX1	NRG4	EGFR	NRG4_EGFR
5600	YBX1	VCAN	EGFR	VCAN_EGFR
5601	YBX1	ICAM1	EGFR	ICAM1_EGFR
5602	YBX1	TNC	EGFR	TNC_EGFR
5603	YBX1	EREG	EGFR	EREG_EGFR
5604	YBX1	L1CAM	EGFR	L1CAM_EGFR
5605	YBX1	CALM3	EGFR	CALM3_EGFR
5606	YBX1	HSP90AA1	EGFR	HSP90AA1_EGFR
5607	YBX1	AMH	EGFR	AMH_EGFR
5608	YBX1	CALM1	EGFR	CALM1_EGFR
5609	YBX1	VEGFA	EGFR	VEGFA_EGFR
5610	YBX1	SPINK1	EGFR	SPINK1_EGFR
5611	YBX1	FGF1	EGFR	FGF1_EGFR
5612	YBX1	AREG	EGFR	AREG_EGFR
5613	YBX1	FGF13	EGFR	FGF13_EGFR
5614	YBX1	TGFA	EGFR	TGFA_EGFR
5615	YBX1	HBEGF	EGFR	HBEGF_EGFR
5616	YBX1	EGF	EGFR	EGF_EGFR
5617	YBX1	ZP3	EGFR	ZP3_EGFR
5618	YBX1	BCAN	EGFR	BCAN_EGFR
5619	YBX1	EPGN	EGFR	EPGN_EGFR
5620	YBX1	DCN	EGFR	DCN_EGFR
5621	YBX1	EFEMP1	EGFR	EFEMP1_EGFR
5622	EGR1	FGL1	EGFR	FGL1_EGFR
5623	EGR1	GNAI2	EGFR	GNAI2_EGFR
5624	EGR1	CALM2	EGFR	CALM2_EGFR
5625	EGR1	CDH1	EGFR	CDH1_EGFR

5626	EGR1	BTC	EGFR	BTC_EGFR
5627	EGR1	UBA52	EGFR	UBA52_EGFR
5628	EGR1	CAMP	EGFR	CAMP_EGFR
5629	EGR1	ANXA1	EGFR	ANXA1_EGFR
5630	EGR1	AREGB	EGFR	AREGB_EGFR
5631	EGR1	NRG4	EGFR	NRG4_EGFR
5632	EGR1	VCAN	EGFR	VCAN_EGFR
5633	EGR1	ICAM1	EGFR	ICAM1_EGFR
5634	EGR1	TNC	EGFR	TNC_EGFR
5635	EGR1	EREG	EGFR	EREG_EGFR
5636	EGR1	L1CAM	EGFR	L1CAM_EGFR
5637	EGR1	CALM3	EGFR	CALM3_EGFR
5638	EGR1	HSP90AA1	EGFR	HSP90AA1_EGFR
5639	EGR1	AMH	EGFR	AMH_EGFR
5640	EGR1	CALM1	EGFR	CALM1_EGFR
5641	EGR1	SPINK1	EGFR	SPINK1_EGFR
5642	EGR1	FGF1	EGFR	FGF1_EGFR
5643	EGR1	AREG	EGFR	AREG_EGFR
5644	EGR1	FGF13	EGFR	FGF13_EGFR
5645	EGR1	TGFA	EGFR	TGFA_EGFR
5646	EGR1	HBEGF	EGFR	HBEGF_EGFR
5647	EGR1	EGF	EGFR	EGF_EGFR
5648	EGR1	ZP3	EGFR	ZP3_EGFR
5649	EGR1	BCAN	EGFR	BCAN_EGFR
5650	EGR1	EPGN	EGFR	EPGN_EGFR
5651	EGR1	DCN	EGFR	DCN_EGFR
5652	EGR1	EFEMP1	EGFR	EFEMP1_EGFR
5653	STAT3	GNAI2	EGFR	GNAI2_EGFR
5654	STAT3	CALM2	EGFR	CALM2_EGFR
5655	STAT3	BTC	EGFR	BTC_EGFR
5656	STAT3	UBA52	EGFR	UBA52_EGFR
5657	STAT3	CAMP	EGFR	CAMP_EGFR
5658	STAT3	ANXA1	EGFR	ANXA1_EGFR
5659	STAT3	AREGB	EGFR	AREGB_EGFR
5660	STAT3	NRG4	EGFR	NRG4_EGFR
5661	STAT3	VCAN	EGFR	VCAN_EGFR
5662	STAT3	TNC	EGFR	TNC_EGFR
5663	STAT3	EREG	EGFR	EREG_EGFR
5664	STAT3	L1CAM	EGFR	L1CAM_EGFR
5665	STAT3	CALM3	EGFR	CALM3_EGFR
5666	STAT3	HSP90AA1	EGFR	HSP90AA1_EGFR
5667	STAT3	AMH	EGFR	AMH_EGFR
5668	STAT3	CALM1	EGFR	CALM1_EGFR
5669	STAT3	SPINK1	EGFR	SPINK1_EGFR
5670	STAT3	AREG	EGFR	AREG_EGFR
5671	STAT3	FGF13	EGFR	FGF13_EGFR
5672	STAT3	TGFA	EGFR	TGFA_EGFR
5673	STAT3	HBEGF	EGFR	HBEGF_EGFR
5674	STAT3	EGF	EGFR	EGF_EGFR
5675	STAT3	ZP3	EGFR	ZP3_EGFR
5676	STAT3	BCAN	EGFR	BCAN_EGFR
5677	STAT3	EPGN	EGFR	EPGN_EGFR
5678	STAT3	DCN	EGFR	DCN_EGFR
5679	STAT3	EFEMP1	EGFR	EFEMP1_EGFR
5680	MTA1	FGL1	EGFR	FGL1_EGFR
5681	MTA1	GNAI2	EGFR	GNAI2_EGFR
5682	MTA1	CALM2	EGFR	CALM2_EGFR
5683	MTA1	BTC	EGFR	BTC_EGFR

5684	MTA1	UBA52	EGFR	UBA52_EGFR
5685	MTA1	CAMP	EGFR	CAMP_EGFR
5686	MTA1	ANXA1	EGFR	ANXA1_EGFR
5687	MTA1	AREGB	EGFR	AREGB_EGFR
5688	MTA1	NRG4	EGFR	NRG4_EGFR
5689	MTA1	VCAN	EGFR	VCAN_EGFR
5690	MTA1	ICAM1	EGFR	ICAM1_EGFR
5691	MTA1	TNC	EGFR	TNC_EGFR
5692	MTA1	EREG	EGFR	EREG_EGFR
5693	MTA1	L1CAM	EGFR	L1CAM_EGFR
5694	MTA1	CALM3	EGFR	CALM3_EGFR
5695	MTA1	HSP90AA1	EGFR	HSP90AA1_EGFR
5696	MTA1	AMH	EGFR	AMH_EGFR
5697	MTA1	CALM1	EGFR	CALM1_EGFR
5698	MTA1	VEGFA	EGFR	VEGFA_EGFR
5699	MTA1	SPINK1	EGFR	SPINK1_EGFR
5700	MTA1	FGF1	EGFR	FGF1_EGFR
5701	MTA1	AREG	EGFR	AREG_EGFR
5702	MTA1	FGF13	EGFR	FGF13_EGFR
5703	MTA1	TGFA	EGFR	TGFA_EGFR
5704	MTA1	HBEGF	EGFR	HBEGF_EGFR
5705	MTA1	EGF	EGFR	EGF_EGFR
5706	MTA1	ZP3	EGFR	ZP3_EGFR
5707	MTA1	BCAN	EGFR	BCAN_EGFR
5708	MTA1	EPGN	EGFR	EPGN_EGFR
5709	MTA1	DCN	EGFR	DCN_EGFR
5710	MTA1	EFEMP1	EGFR	EFEMP1_EGFR
5711	SP1	FGL1	EGFR	FGL1_EGFR
5712	SP1	GNAI2	EGFR	GNAI2_EGFR
5713	SP1	CALM2	EGFR	CALM2_EGFR
5714	SP1	CDH1	EGFR	CDH1_EGFR
5715	SP1	BTC	EGFR	BTC_EGFR
5716	SP1	UBA52	EGFR	UBA52_EGFR
5717	SP1	CAMP	EGFR	CAMP_EGFR
5718	SP1	ANXA1	EGFR	ANXA1_EGFR
5719	SP1	AREGB	EGFR	AREGB_EGFR
5720	SP1	NRG4	EGFR	NRG4_EGFR
5721	SP1	VCAN	EGFR	VCAN_EGFR
5722	SP1	EREG	EGFR	EREG_EGFR
5723	SP1	L1CAM	EGFR	L1CAM_EGFR
5724	SP1	CALM3	EGFR	CALM3_EGFR
5725	SP1	HSP90AA1	EGFR	HSP90AA1_EGFR
5726	SP1	AMH	EGFR	AMH_EGFR
5727	SP1	CALM1	EGFR	CALM1_EGFR
5728	SP1	SPINK1	EGFR	SPINK1_EGFR
5729	SP1	FGF1	EGFR	FGF1_EGFR
5730	SP1	FGF13	EGFR	FGF13_EGFR
5731	SP1	ZP3	EGFR	ZP3_EGFR
5732	SP1	BCAN	EGFR	BCAN_EGFR
5733	SP1	EPGN	EGFR	EPGN_EGFR
5734	SP1	DCN	EGFR	DCN_EGFR
5735	SP1	EFEMP1	EGFR	EFEMP1_EGFR
5736	RELA	FGL1	EGFR	FGL1_EGFR
5737	RELA	GNAI2	EGFR	GNAI2_EGFR
5738	RELA	CALM2	EGFR	CALM2_EGFR
5739	RELA	BTC	EGFR	BTC_EGFR
5740	RELA	UBA52	EGFR	UBA52_EGFR
5741	RELA	CAMP	EGFR	CAMP_EGFR

5742	RELA	ANXA1	EGFR	ANXA1_EGFR
5743	RELA	AREGB	EGFR	AREGB_EGFR
5744	RELA	NRG4	EGFR	NRG4_EGFR
5745	RELA	VCAN	EGFR	VCAN_EGFR
5746	RELA	EREG	EGFR	EREG_EGFR
5747	RELA	L1CAM	EGFR	L1CAM_EGFR
5748	RELA	CALM3	EGFR	CALM3_EGFR
5749	RELA	HSP90AA1	EGFR	HSP90AA1_EGFR
5750	RELA	CALM1	EGFR	CALM1_EGFR
5751	RELA	SPINK1	EGFR	SPINK1_EGFR
5752	RELA	FGF1	EGFR	FGF1_EGFR
5753	RELA	AREG	EGFR	AREG_EGFR
5754	RELA	FGF13	EGFR	FGF13_EGFR
5755	RELA	TGFA	EGFR	TGFA_EGFR
5756	RELA	EGF	EGFR	EGF_EGFR
5757	RELA	ZP3	EGFR	ZP3_EGFR
5758	RELA	BCAN	EGFR	BCAN_EGFR
5759	RELA	EPGN	EGFR	EPGN_EGFR
5760	RELA	DCN	EGFR	DCN_EGFR
5761	RELA	EFEMP1	EGFR	EFEMP1_EGFR
5762	PGR	FGL1	EGFR	FGL1_EGFR
5763	PGR	GNAI2	EGFR	GNAI2_EGFR
5764	PGR	CALM2	EGFR	CALM2_EGFR
5765	PGR	CDH1	EGFR	CDH1_EGFR
5766	PGR	BTC	EGFR	BTC_EGFR
5767	PGR	UBA52	EGFR	UBA52_EGFR
5768	PGR	CAMP	EGFR	CAMP_EGFR
5769	PGR	ANXA1	EGFR	ANXA1_EGFR
5770	PGR	AREGB	EGFR	AREGB_EGFR
5771	PGR	NRG4	EGFR	NRG4_EGFR
5772	PGR	VCAN	EGFR	VCAN_EGFR
5773	PGR	ICAM1	EGFR	ICAM1_EGFR
5774	PGR	TNC	EGFR	TNC_EGFR
5775	PGR	EREG	EGFR	EREG_EGFR
5776	PGR	L1CAM	EGFR	L1CAM_EGFR
5777	PGR	CALM3	EGFR	CALM3_EGFR
5778	PGR	HSP90AA1	EGFR	HSP90AA1_EGFR
5779	PGR	AMH	EGFR	AMH_EGFR
5780	PGR	CALM1	EGFR	CALM1_EGFR
5781	PGR	SPINK1	EGFR	SPINK1_EGFR
5782	PGR	FGF1	EGFR	FGF1_EGFR
5783	PGR	AREG	EGFR	AREG_EGFR
5784	PGR	FGF13	EGFR	FGF13_EGFR
5785	PGR	TGFA	EGFR	TGFA_EGFR
5786	PGR	HBEGF	EGFR	HBEGF_EGFR
5787	PGR	EGF	EGFR	EGF_EGFR
5788	PGR	ZP3	EGFR	ZP3_EGFR
5789	PGR	BCAN	EGFR	BCAN_EGFR
5790	PGR	EPGN	EGFR	EPGN_EGFR
5791	PGR	DCN	EGFR	DCN_EGFR
5792	PGR	EFEMP1	EGFR	EFEMP1_EGFR
5793	KLF10	FGL1	EGFR	FGL1_EGFR
5794	KLF10	GNAI2	EGFR	GNAI2_EGFR
5795	KLF10	CALM2	EGFR	CALM2_EGFR
5796	KLF10	CDH1	EGFR	CDH1_EGFR
5797	KLF10	BTC	EGFR	BTC_EGFR
5798	KLF10	UBA52	EGFR	UBA52_EGFR
5799	KLF10	CAMP	EGFR	CAMP_EGFR

5800	KLF10	ANXA1	EGFR	ANXA1_EGFR
5801	KLF10	AREGB	EGFR	AREGB_EGFR
5802	KLF10	NRG4	EGFR	NRG4_EGFR
5803	KLF10	VCAN	EGFR	VCAN_EGFR
5804	KLF10	ICAM1	EGFR	ICAM1_EGFR
5805	KLF10	TNC	EGFR	TNC_EGFR
5806	KLF10	EREG	EGFR	EREG_EGFR
5807	KLF10	L1CAM	EGFR	L1CAM_EGFR
5808	KLF10	CALM3	EGFR	CALM3_EGFR
5809	KLF10	HSP90AA1	EGFR	HSP90AA1_EGFR
5810	KLF10	AMH	EGFR	AMH_EGFR
5811	KLF10	CALM1	EGFR	CALM1_EGFR
5812	KLF10	VEGFA	EGFR	VEGFA_EGFR
5813	KLF10	SPINK1	EGFR	SPINK1_EGFR
5814	KLF10	FGF1	EGFR	FGF1_EGFR
5815	KLF10	AREG	EGFR	AREG_EGFR
5816	KLF10	FGF13	EGFR	FGF13_EGFR
5817	KLF10	TGFA	EGFR	TGFA_EGFR
5818	KLF10	HBEGF	EGFR	HBEGF_EGFR
5819	KLF10	EGF	EGFR	EGF_EGFR
5820	KLF10	ZP3	EGFR	ZP3_EGFR
5821	KLF10	BCAN	EGFR	BCAN_EGFR
5822	KLF10	EPGN	EGFR	EPGN_EGFR
5823	KLF10	DCN	EGFR	DCN_EGFR
5824	KLF10	EFEMP1	EGFR	EFEMP1_EGFR
5825	CREBBP	FGL1	EGFR	FGL1_EGFR
5826	CREBBP	GNAI2	EGFR	GNAI2_EGFR
5827	CREBBP	CALM2	EGFR	CALM2_EGFR
5828	CREBBP	CDH1	EGFR	CDH1_EGFR
5829	CREBBP	BTC	EGFR	BTC_EGFR
5830	CREBBP	UBA52	EGFR	UBA52_EGFR
5831	CREBBP	CAMP	EGFR	CAMP_EGFR
5832	CREBBP	ANXA1	EGFR	ANXA1_EGFR
5833	CREBBP	AREGB	EGFR	AREGB_EGFR
5834	CREBBP	NRG4	EGFR	NRG4_EGFR
5835	CREBBP	VCAN	EGFR	VCAN_EGFR
5836	CREBBP	ICAM1	EGFR	ICAM1_EGFR
5837	CREBBP	TNC	EGFR	TNC_EGFR
5838	CREBBP	EREG	EGFR	EREG_EGFR
5839	CREBBP	L1CAM	EGFR	L1CAM_EGFR
5840	CREBBP	CALM3	EGFR	CALM3_EGFR
5841	CREBBP	HSP90AA1	EGFR	HSP90AA1_EGFR
5842	CREBBP	AMH	EGFR	AMH_EGFR
5843	CREBBP	CALM1	EGFR	CALM1_EGFR
5844	CREBBP	VEGFA	EGFR	VEGFA_EGFR
5845	CREBBP	SPINK1	EGFR	SPINK1_EGFR
5846	CREBBP	FGF1	EGFR	FGF1_EGFR
5847	CREBBP	AREG	EGFR	AREG_EGFR
5848	CREBBP	FGF13	EGFR	FGF13_EGFR
5849	CREBBP	TGFA	EGFR	TGFA_EGFR
5850	CREBBP	HBEGF	EGFR	HBEGF_EGFR
5851	CREBBP	EGF	EGFR	EGF_EGFR
5852	CREBBP	ZP3	EGFR	ZP3_EGFR
5853	CREBBP	BCAN	EGFR	BCAN_EGFR
5854	CREBBP	EPGN	EGFR	EPGN_EGFR
5855	CREBBP	DCN	EGFR	DCN_EGFR
5856	CREBBP	EFEMP1	EGFR	EFEMP1_EGFR
5857	TP53	FGL1	EGFR	FGL1_EGFR

5858 TP53	GNAI2	EGFR	GNAI2_EGFR
5859 TP53	CALM2	EGFR	CALM2_EGFR
5860 TP53	CDH1	EGFR	CDH1_EGFR
5861 TP53	BTC	EGFR	BTC_EGFR
5862 TP53	UBA52	EGFR	UBA52_EGFR
5863 TP53	CAMP	EGFR	CAMP_EGFR
5864 TP53	ANXA1	EGFR	ANXA1_EGFR
5865 TP53	AREGB	EGFR	AREGB_EGFR
5866 TP53	NRG4	EGFR	NRG4_EGFR
5867 TP53	ICAM1	EGFR	ICAM1_EGFR
5868 TP53	TNC	EGFR	TNC_EGFR
5869 TP53	EREG	EGFR	EREG_EGFR
5870 TP53	L1CAM	EGFR	L1CAM_EGFR
5871 TP53	CALM3	EGFR	CALM3_EGFR
5872 TP53	HSP90AA1	EGFR	HSP90AA1_EGFR
5873 TP53	AMH	EGFR	AMH_EGFR
5874 TP53	CALM1	EGFR	CALM1_EGFR
5875 TP53	SPINK1	EGFR	SPINK1_EGFR
5876 TP53	FGF1	EGFR	FGF1_EGFR
5877 TP53	AREG	EGFR	AREG_EGFR
5878 TP53	FGF13	EGFR	FGF13_EGFR
5879 TP53	TGFA	EGFR	TGFA_EGFR
5880 TP53	HBEGF	EGFR	HBEGF_EGFR
5881 TP53	EGF	EGFR	EGF_EGFR
5882 TP53	ZP3	EGFR	ZP3_EGFR
5883 TP53	BCAN	EGFR	BCAN_EGFR
5884 TP53	EPGN	EGFR	EPGN_EGFR
5885 TP53	DCN	EGFR	DCN_EGFR
5886 TP53	EFEMP1	EGFR	EFEMP1_EGFR
5887 NFKB1	FGL1	EGFR	FGL1_EGFR
5888 NFKB1	GNAI2	EGFR	GNAI2_EGFR
5889 NFKB1	CALM2	EGFR	CALM2_EGFR
5890 NFKB1	BTC	EGFR	BTC_EGFR
5891 NFKB1	UBA52	EGFR	UBA52_EGFR
5892 NFKB1	CAMP	EGFR	CAMP_EGFR
5893 NFKB1	ANXA1	EGFR	ANXA1_EGFR
5894 NFKB1	AREGB	EGFR	AREGB_EGFR
5895 NFKB1	NRG4	EGFR	NRG4_EGFR
5896 NFKB1	VCAN	EGFR	VCAN_EGFR
5897 NFKB1	EREG	EGFR	EREG_EGFR
5898 NFKB1	L1CAM	EGFR	L1CAM_EGFR
5899 NFKB1	CALM3	EGFR	CALM3_EGFR
5900 NFKB1	HSP90AA1	EGFR	HSP90AA1_EGFR
5901 NFKB1	CALM1	EGFR	CALM1_EGFR
5902 NFKB1	SPINK1	EGFR	SPINK1_EGFR
5903 NFKB1	FGF1	EGFR	FGF1_EGFR
5904 NFKB1	AREG	EGFR	AREG_EGFR
5905 NFKB1	FGF13	EGFR	FGF13_EGFR
5906 NFKB1	TGFA	EGFR	TGFA_EGFR
5907 NFKB1	EGF	EGFR	EGF_EGFR
5908 NFKB1	ZP3	EGFR	ZP3_EGFR
5909 NFKB1	BCAN	EGFR	BCAN_EGFR
5910 NFKB1	EPGN	EGFR	EPGN_EGFR
5911 NFKB1	DCN	EGFR	DCN_EGFR
5912 NFKB1	EFEMP1	EGFR	EFEMP1_EGFR
5913 VDR	FGL1	EGFR	FGL1_EGFR
5914 VDR	GNAI2	EGFR	GNAI2_EGFR
5915 VDR	CALM2	EGFR	CALM2_EGFR

5916	VDR	BTC	EGFR	BTC_EGFR
5917	VDR	UBA52	EGFR	UBA52_EGFR
5918	VDR	ANXA1	EGFR	ANXA1_EGFR
5919	VDR	AREGB	EGFR	AREGB_EGFR
5920	VDR	NRG4	EGFR	NRG4_EGFR
5921	VDR	VCAN	EGFR	VCAN_EGFR
5922	VDR	ICAM1	EGFR	ICAM1_EGFR
5923	VDR	TNC	EGFR	TNC_EGFR
5924	VDR	EREG	EGFR	EREG_EGFR
5925	VDR	L1CAM	EGFR	L1CAM_EGFR
5926	VDR	CALM3	EGFR	CALM3_EGFR
5927	VDR	HSP90AA1	EGFR	HSP90AA1_EGFR
5928	VDR	CALM1	EGFR	CALM1_EGFR
5929	VDR	VEGFA	EGFR	VEGFA_EGFR
5930	VDR	SPINK1	EGFR	SPINK1_EGFR
5931	VDR	FGF1	EGFR	FGF1_EGFR
5932	VDR	AREG	EGFR	AREG_EGFR
5933	VDR	FGF13	EGFR	FGF13_EGFR
5934	VDR	TGFA	EGFR	TGFA_EGFR
5935	VDR	HBEGF	EGFR	HBEGF_EGFR
5936	VDR	EGF	EGFR	EGF_EGFR
5937	VDR	ZP3	EGFR	ZP3_EGFR
5938	VDR	BCAN	EGFR	BCAN_EGFR
5939	VDR	EPGN	EGFR	EPGN_EGFR
5940	VDR	DCN	EGFR	DCN_EGFR
5941	VDR	EFEMP1	EGFR	EFEMP1_EGFR
5942	HDAC1	FGL1	EGFR	FGL1_EGFR
5943	HDAC1	GNAI2	EGFR	GNAI2_EGFR
5944	HDAC1	CALM2	EGFR	CALM2_EGFR
5945	HDAC1	BTC	EGFR	BTC_EGFR
5946	HDAC1	UBA52	EGFR	UBA52_EGFR
5947	HDAC1	CAMP	EGFR	CAMP_EGFR
5948	HDAC1	ANXA1	EGFR	ANXA1_EGFR
5949	HDAC1	AREGB	EGFR	AREGB_EGFR
5950	HDAC1	NRG4	EGFR	NRG4_EGFR
5951	HDAC1	VCAN	EGFR	VCAN_EGFR
5952	HDAC1	TNC	EGFR	TNC_EGFR
5953	HDAC1	EREG	EGFR	EREG_EGFR
5954	HDAC1	L1CAM	EGFR	L1CAM_EGFR
5955	HDAC1	CALM3	EGFR	CALM3_EGFR
5956	HDAC1	HSP90AA1	EGFR	HSP90AA1_EGFR
5957	HDAC1	AMH	EGFR	AMH_EGFR
5958	HDAC1	CALM1	EGFR	CALM1_EGFR
5959	HDAC1	VEGFA	EGFR	VEGFA_EGFR
5960	HDAC1	SPINK1	EGFR	SPINK1_EGFR
5961	HDAC1	FGF1	EGFR	FGF1_EGFR
5962	HDAC1	AREG	EGFR	AREG_EGFR
5963	HDAC1	FGF13	EGFR	FGF13_EGFR
5964	HDAC1	TGFA	EGFR	TGFA_EGFR
5965	HDAC1	HBEGF	EGFR	HBEGF_EGFR
5966	HDAC1	EGF	EGFR	EGF_EGFR
5967	HDAC1	ZP3	EGFR	ZP3_EGFR
5968	HDAC1	BCAN	EGFR	BCAN_EGFR
5969	HDAC1	EPGN	EGFR	EPGN_EGFR
5970	HDAC1	DCN	EGFR	DCN_EGFR
5971	HDAC1	EFEMP1	EGFR	EFEMP1_EGFR
5972	WT1	FGL1	EGFR	FGL1_EGFR
5973	WT1	GNAI2	EGFR	GNAI2_EGFR

5974	WT1	CALM2	EGFR	CALM2_EGFR
5975	WT1	BTC	EGFR	BTC_EGFR
5976	WT1	UBA52	EGFR	UBA52_EGFR
5977	WT1	ANXA1	EGFR	ANXA1_EGFR
5978	WT1	AREGB	EGFR	AREGB_EGFR
5979	WT1	NRG4	EGFR	NRG4_EGFR
5980	WT1	VCAN	EGFR	VCAN_EGFR
5981	WT1	ICAM1	EGFR	ICAM1_EGFR
5982	WT1	TNC	EGFR	TNC_EGFR
5983	WT1	L1CAM	EGFR	L1CAM_EGFR
5984	WT1	CALM3	EGFR	CALM3_EGFR
5985	WT1	HSP90AA1	EGFR	HSP90AA1_EGFR
5986	WT1	CALM1	EGFR	CALM1_EGFR
5987	WT1	SPINK1	EGFR	SPINK1_EGFR
5988	WT1	FGF1	EGFR	FGF1_EGFR
5989	WT1	AREG	EGFR	AREG_EGFR
5990	WT1	FGF13	EGFR	FGF13_EGFR
5991	WT1	TGFA	EGFR	TGFA_EGFR
5992	WT1	EGF	EGFR	EGF_EGFR
5993	WT1	ZP3	EGFR	ZP3_EGFR
5994	WT1	BCAN	EGFR	BCAN_EGFR
5995	WT1	EPGN	EGFR	EPGN_EGFR
5996	WT1	DCN	EGFR	DCN_EGFR
5997	WT1	EFEMP1	EGFR	EFEMP1_EGFR
5998	TFAP2A	FGL1	EGFR	FGL1_EGFR
5999	TFAP2A	GNAI2	EGFR	GNAI2_EGFR
6000	TFAP2A	CALM2	EGFR	CALM2_EGFR
6001	TFAP2A	BTC	EGFR	BTC_EGFR
6002	TFAP2A	UBA52	EGFR	UBA52_EGFR
6003	TFAP2A	CAMP	EGFR	CAMP_EGFR
6004	TFAP2A	ANXA1	EGFR	ANXA1_EGFR
6005	TFAP2A	AREGB	EGFR	AREGB_EGFR
6006	TFAP2A	NRG4	EGFR	NRG4_EGFR
6007	TFAP2A	VCAN	EGFR	VCAN_EGFR
6008	TFAP2A	ICAM1	EGFR	ICAM1_EGFR
6009	TFAP2A	TNC	EGFR	TNC_EGFR
6010	TFAP2A	EREG	EGFR	EREG_EGFR
6011	TFAP2A	CALM3	EGFR	CALM3_EGFR
6012	TFAP2A	HSP90AA1	EGFR	HSP90AA1_EGFR
6013	TFAP2A	AMH	EGFR	AMH_EGFR
6014	TFAP2A	CALM1	EGFR	CALM1_EGFR
6015	TFAP2A	SPINK1	EGFR	SPINK1_EGFR
6016	TFAP2A	FGF1	EGFR	FGF1_EGFR
6017	TFAP2A	AREG	EGFR	AREG_EGFR
6018	TFAP2A	FGF13	EGFR	FGF13_EGFR
6019	TFAP2A	TGFA	EGFR	TGFA_EGFR
6020	TFAP2A	HBEGF	EGFR	HBEGF_EGFR
6021	TFAP2A	EGF	EGFR	EGF_EGFR
6022	TFAP2A	ZP3	EGFR	ZP3_EGFR
6023	TFAP2A	BCAN	EGFR	BCAN_EGFR
6024	TFAP2A	EPGN	EGFR	EPGN_EGFR
6025	TFAP2A	DCN	EGFR	DCN_EGFR
6026	TFAP2A	EFEMP1	EGFR	EFEMP1_EGFR
6027	LRRFIP1	FGL1	EGFR	FGL1_EGFR
6028	LRRFIP1	GNAI2	EGFR	GNAI2_EGFR
6029	LRRFIP1	CALM2	EGFR	CALM2_EGFR
6030	LRRFIP1	CDH1	EGFR	CDH1_EGFR
6031	LRRFIP1	BTC	EGFR	BTC_EGFR

6032	LRRFIP1	UBA52	EGFR	UBA52_EGFR
6033	LRRFIP1	CAMP	EGFR	CAMP_EGFR
6034	LRRFIP1	ANXA1	EGFR	ANXA1_EGFR
6035	LRRFIP1	AREGB	EGFR	AREGB_EGFR
6036	LRRFIP1	NRG4	EGFR	NRG4_EGFR
6037	LRRFIP1	VCAN	EGFR	VCAN_EGFR
6038	LRRFIP1	ICAM1	EGFR	ICAM1_EGFR
6039	LRRFIP1	TNC	EGFR	TNC_EGFR
6040	LRRFIP1	EREG	EGFR	EREG_EGFR
6041	LRRFIP1	L1CAM	EGFR	L1CAM_EGFR
6042	LRRFIP1	CALM3	EGFR	CALM3_EGFR
6043	LRRFIP1	HSP90AA1	EGFR	HSP90AA1_EGFR
6044	LRRFIP1	AMH	EGFR	AMH_EGFR
6045	LRRFIP1	CALM1	EGFR	CALM1_EGFR
6046	LRRFIP1	VEGFA	EGFR	VEGFA_EGFR
6047	LRRFIP1	SPINK1	EGFR	SPINK1_EGFR
6048	LRRFIP1	FGF1	EGFR	FGF1_EGFR
6049	LRRFIP1	AREG	EGFR	AREG_EGFR
6050	LRRFIP1	FGF13	EGFR	FGF13_EGFR
6051	LRRFIP1	TGFA	EGFR	TGFA_EGFR
6052	LRRFIP1	HBEGF	EGFR	HBEGF_EGFR
6053	LRRFIP1	EGF	EGFR	EGF_EGFR
6054	LRRFIP1	ZP3	EGFR	ZP3_EGFR
6055	LRRFIP1	BCAN	EGFR	BCAN_EGFR
6056	LRRFIP1	EPGN	EGFR	EPGN_EGFR
6057	LRRFIP1	DCN	EGFR	DCN_EGFR
6058	LRRFIP1	EFEMP1	EGFR	EFEMP1_EGFR
6059	NR3C2	FGL1	EGFR	FGL1_EGFR
6060	NR3C2	GNAI2	EGFR	GNAI2_EGFR
6061	NR3C2	CALM2	EGFR	CALM2_EGFR
6062	NR3C2	CDH1	EGFR	CDH1_EGFR
6063	NR3C2	BTC	EGFR	BTC_EGFR
6064	NR3C2	UBA52	EGFR	UBA52_EGFR
6065	NR3C2	CAMP	EGFR	CAMP_EGFR
6066	NR3C2	ANXA1	EGFR	ANXA1_EGFR
6067	NR3C2	AREGB	EGFR	AREGB_EGFR
6068	NR3C2	NRG4	EGFR	NRG4_EGFR
6069	NR3C2	VCAN	EGFR	VCAN_EGFR
6070	NR3C2	ICAM1	EGFR	ICAM1_EGFR
6071	NR3C2	TNC	EGFR	TNC_EGFR
6072	NR3C2	EREG	EGFR	EREG_EGFR
6073	NR3C2	L1CAM	EGFR	L1CAM_EGFR
6074	NR3C2	CALM3	EGFR	CALM3_EGFR
6075	NR3C2	HSP90AA1	EGFR	HSP90AA1_EGFR
6076	NR3C2	AMH	EGFR	AMH_EGFR
6077	NR3C2	CALM1	EGFR	CALM1_EGFR
6078	NR3C2	VEGFA	EGFR	VEGFA_EGFR
6079	NR3C2	SPINK1	EGFR	SPINK1_EGFR
6080	NR3C2	FGF1	EGFR	FGF1_EGFR
6081	NR3C2	AREG	EGFR	AREG_EGFR
6082	NR3C2	FGF13	EGFR	FGF13_EGFR
6083	NR3C2	TGFA	EGFR	TGFA_EGFR
6084	NR3C2	HBEGF	EGFR	HBEGF_EGFR
6085	NR3C2	EGF	EGFR	EGF_EGFR
6086	NR3C2	ZP3	EGFR	ZP3_EGFR
6087	NR3C2	BCAN	EGFR	BCAN_EGFR
6088	NR3C2	EPGN	EGFR	EPGN_EGFR
6089	NR3C2	DCN	EGFR	DCN_EGFR

6090	NR3C2	EFEMP1	EGFR	EFEMP1_EGFR
6091	STAT1	FGL1	EGFR	FGL1_EGFR
6092	STAT1	GNAI2	EGFR	GNAI2_EGFR
6093	STAT1	CALM2	EGFR	CALM2_EGFR
6094	STAT1	CDH1	EGFR	CDH1_EGFR
6095	STAT1	BTC	EGFR	BTC_EGFR
6096	STAT1	UBA52	EGFR	UBA52_EGFR
6097	STAT1	CAMP	EGFR	CAMP_EGFR
6098	STAT1	ANXA1	EGFR	ANXA1_EGFR
6099	STAT1	AREGB	EGFR	AREGB_EGFR
6100	STAT1	NRG4	EGFR	NRG4_EGFR
6101	STAT1	VCAN	EGFR	VCAN_EGFR
6102	STAT1	TNC	EGFR	TNC_EGFR
6103	STAT1	EREG	EGFR	EREG_EGFR
6104	STAT1	L1CAM	EGFR	L1CAM_EGFR
6105	STAT1	CALM3	EGFR	CALM3_EGFR
6106	STAT1	AMH	EGFR	AMH_EGFR
6107	STAT1	CALM1	EGFR	CALM1_EGFR
6108	STAT1	VEGFA	EGFR	VEGFA_EGFR
6109	STAT1	SPINK1	EGFR	SPINK1_EGFR
6110	STAT1	FGF1	EGFR	FGF1_EGFR
6111	STAT1	AREG	EGFR	AREG_EGFR
6112	STAT1	FGF13	EGFR	FGF13_EGFR
6113	STAT1	TGFA	EGFR	TGFA_EGFR
6114	STAT1	HBEGF	EGFR	HBEGF_EGFR
6115	STAT1	EGF	EGFR	EGF_EGFR
6116	STAT1	ZP3	EGFR	ZP3_EGFR
6117	STAT1	BCAN	EGFR	BCAN_EGFR
6118	STAT1	EPGN	EGFR	EPGN_EGFR
6119	STAT1	DCN	EGFR	DCN_EGFR
6120	STAT1	EFEMP1	EGFR	EFEMP1_EGFR
6121	AR	FGL1	EGFR	FGL1_EGFR
6122	AR	GNAI2	EGFR	GNAI2_EGFR
6123	AR	CALM2	EGFR	CALM2_EGFR
6124	AR	BTC	EGFR	BTC_EGFR
6125	AR	UBA52	EGFR	UBA52_EGFR
6126	AR	CAMP	EGFR	CAMP_EGFR
6127	AR	ANXA1	EGFR	ANXA1_EGFR
6128	AR	AREGB	EGFR	AREGB_EGFR
6129	AR	NRG4	EGFR	NRG4_EGFR
6130	AR	VCAN	EGFR	VCAN_EGFR
6131	AR	ICAM1	EGFR	ICAM1_EGFR
6132	AR	TNC	EGFR	TNC_EGFR
6133	AR	EREG	EGFR	EREG_EGFR
6134	AR	L1CAM	EGFR	L1CAM_EGFR
6135	AR	CALM3	EGFR	CALM3_EGFR
6136	AR	HSP90AA1	EGFR	HSP90AA1_EGFR
6137	AR	AMH	EGFR	AMH_EGFR
6138	AR	CALM1	EGFR	CALM1_EGFR
6139	AR	SPINK1	EGFR	SPINK1_EGFR
6140	AR	FGF1	EGFR	FGF1_EGFR
6141	AR	AREG	EGFR	AREG_EGFR
6142	AR	FGF13	EGFR	FGF13_EGFR
6143	AR	HBEGF	EGFR	HBEGF_EGFR
6144	AR	EGF	EGFR	EGF_EGFR
6145	AR	ZP3	EGFR	ZP3_EGFR
6146	AR	BCAN	EGFR	BCAN_EGFR
6147	AR	EPGN	EGFR	EPGN_EGFR

6148	AR	DCN	EGFR	DCN_EGFR
6149	AR	EFEMP1	EGFR	EFEMP1_EGFR
6150	PML	FGL1	EGFR	FGL1_EGFR
6151	PML	GNAI2	EGFR	GNAI2_EGFR
6152	PML	CALM2	EGFR	CALM2_EGFR
6153	PML	CDH1	EGFR	CDH1_EGFR
6154	PML	BTC	EGFR	BTC_EGFR
6155	PML	UBA52	EGFR	UBA52_EGFR
6156	PML	CAMP	EGFR	CAMP_EGFR
6157	PML	ANXA1	EGFR	ANXA1_EGFR
6158	PML	AREGB	EGFR	AREGB_EGFR
6159	PML	NRG4	EGFR	NRG4_EGFR
6160	PML	VCAN	EGFR	VCAN_EGFR
6161	PML	ICAM1	EGFR	ICAM1_EGFR
6162	PML	TNC	EGFR	TNC_EGFR
6163	PML	EREG	EGFR	EREG_EGFR
6164	PML	L1CAM	EGFR	L1CAM_EGFR
6165	PML	CALM3	EGFR	CALM3_EGFR
6166	PML	HSP90AA1	EGFR	HSP90AA1_EGFR
6167	PML	AMH	EGFR	AMH_EGFR
6168	PML	CALM1	EGFR	CALM1_EGFR
6169	PML	VEGFA	EGFR	VEGFA_EGFR
6170	PML	SPINK1	EGFR	SPINK1_EGFR
6171	PML	FGF1	EGFR	FGF1_EGFR
6172	PML	AREG	EGFR	AREG_EGFR
6173	PML	FGF13	EGFR	FGF13_EGFR
6174	PML	TGFA	EGFR	TGFA_EGFR
6175	PML	HBEGF	EGFR	HBEGF_EGFR
6176	PML	EGF	EGFR	EGF_EGFR
6177	PML	ZP3	EGFR	ZP3_EGFR
6178	PML	BCAN	EGFR	BCAN_EGFR
6179	PML	EPGN	EGFR	EPGN_EGFR
6180	PML	DCN	EGFR	DCN_EGFR
6181	PML	EFEMP1	EGFR	EFEMP1_EGFR
6182	HDAC3	FGL1	EGFR	FGL1_EGFR
6183	HDAC3	GNAI2	EGFR	GNAI2_EGFR
6184	HDAC3	CALM2	EGFR	CALM2_EGFR
6185	HDAC3	BTC	EGFR	BTC_EGFR
6186	HDAC3	UBA52	EGFR	UBA52_EGFR
6187	HDAC3	CAMP	EGFR	CAMP_EGFR
6188	HDAC3	ANXA1	EGFR	ANXA1_EGFR
6189	HDAC3	AREGB	EGFR	AREGB_EGFR
6190	HDAC3	NRG4	EGFR	NRG4_EGFR
6191	HDAC3	VCAN	EGFR	VCAN_EGFR
6192	HDAC3	ICAM1	EGFR	ICAM1_EGFR
6193	HDAC3	TNC	EGFR	TNC_EGFR
6194	HDAC3	EREG	EGFR	EREG_EGFR
6195	HDAC3	L1CAM	EGFR	L1CAM_EGFR
6196	HDAC3	CALM3	EGFR	CALM3_EGFR
6197	HDAC3	HSP90AA1	EGFR	HSP90AA1_EGFR
6198	HDAC3	AMH	EGFR	AMH_EGFR
6199	HDAC3	CALM1	EGFR	CALM1_EGFR
6200	HDAC3	SPINK1	EGFR	SPINK1_EGFR
6201	HDAC3	FGF1	EGFR	FGF1_EGFR
6202	HDAC3	AREG	EGFR	AREG_EGFR
6203	HDAC3	FGF13	EGFR	FGF13_EGFR
6204	HDAC3	TGFA	EGFR	TGFA_EGFR
6205	HDAC3	HBEGF	EGFR	HBEGF_EGFR

6206 HDAC3	EGF	EGFR	EGF_EGFR
6207 HDAC3	ZP3	EGFR	ZP3_EGFR
6208 HDAC3	BCAN	EGFR	BCAN_EGFR
6209 HDAC3	EPGN	EGFR	EPGN_EGFR
6210 HDAC3	DCN	EGFR	DCN_EGFR
6211 HDAC3	EFEMP1	EGFR	EFEMP1_EGFR
6212 PPARG	FGL1	EGFR	FGL1_EGFR
6213 PPARG	GNAI2	EGFR	GNAI2_EGFR
6214 PPARG	CALM2	EGFR	CALM2_EGFR
6215 PPARG	CDH1	EGFR	CDH1_EGFR
6216 PPARG	BTC	EGFR	BTC_EGFR
6217 PPARG	UBA52	EGFR	UBA52_EGFR
6218 PPARG	CAMP	EGFR	CAMP_EGFR
6219 PPARG	ANXA1	EGFR	ANXA1_EGFR
6220 PPARG	AREGB	EGFR	AREGB_EGFR
6221 PPARG	NRG4	EGFR	NRG4_EGFR
6222 PPARG	VCAN	EGFR	VCAN_EGFR
6223 PPARG	TNC	EGFR	TNC_EGFR
6224 PPARG	EREG	EGFR	EREG_EGFR
6225 PPARG	L1CAM	EGFR	L1CAM_EGFR
6226 PPARG	CALM3	EGFR	CALM3_EGFR
6227 PPARG	HSP90AA1	EGFR	HSP90AA1_EGFR
6228 PPARG	AMH	EGFR	AMH_EGFR
6229 PPARG	CALM1	EGFR	CALM1_EGFR
6230 PPARG	VEGFA	EGFR	VEGFA_EGFR
6231 PPARG	SPINK1	EGFR	SPINK1_EGFR
6232 PPARG	AREG	EGFR	AREG_EGFR
6233 PPARG	FGF13	EGFR	FGF13_EGFR
6234 PPARG	TGFA	EGFR	TGFA_EGFR
6235 PPARG	HBEGF	EGFR	HBEGF_EGFR
6236 PPARG	EGF	EGFR	EGF_EGFR
6237 PPARG	ZP3	EGFR	ZP3_EGFR
6238 PPARG	BCAN	EGFR	BCAN_EGFR
6239 PPARG	EPGN	EGFR	EPGN_EGFR
6240 PPARG	DCN	EGFR	DCN_EGFR
6241 PPARG	EFEMP1	EGFR	EFEMP1_EGFR
6242 JUN	FGL1	EGFR	FGL1_EGFR
6243 JUN	GNAI2	EGFR	GNAI2_EGFR
6244 JUN	CALM2	EGFR	CALM2_EGFR
6245 JUN	CDH1	EGFR	CDH1_EGFR
6246 JUN	BTC	EGFR	BTC_EGFR
6247 JUN	UBA52	EGFR	UBA52_EGFR
6248 JUN	CAMP	EGFR	CAMP_EGFR
6249 JUN	ANXA1	EGFR	ANXA1_EGFR
6250 JUN	AREGB	EGFR	AREGB_EGFR
6251 JUN	NRG4	EGFR	NRG4_EGFR
6252 JUN	VCAN	EGFR	VCAN_EGFR
6253 JUN	ICAM1	EGFR	ICAM1_EGFR
6254 JUN	EREG	EGFR	EREG_EGFR
6255 JUN	L1CAM	EGFR	L1CAM_EGFR
6256 JUN	CALM3	EGFR	CALM3_EGFR
6257 JUN	HSP90AA1	EGFR	HSP90AA1_EGFR
6258 JUN	AMH	EGFR	AMH_EGFR
6259 JUN	CALM1	EGFR	CALM1_EGFR
6260 JUN	SPINK1	EGFR	SPINK1_EGFR
6261 JUN	FGF1	EGFR	FGF1_EGFR
6262 JUN	AREG	EGFR	AREG_EGFR
6263 JUN	FGF13	EGFR	FGF13_EGFR

6264	JUN	TGFA	EGFR	TGFA_EGFR
6265	JUN	HBEGF	EGFR	HBEGF_EGFR
6266	JUN	EGF	EGFR	EGF_EGFR
6267	JUN	ZP3	EGFR	ZP3_EGFR
6268	JUN	BCAN	EGFR	BCAN_EGFR
6269	JUN	EPGN	EGFR	EPGN_EGFR
6270	JUN	EFEMP1	EGFR	EFEMP1_EGFR
6271	MTA1	EFNA1	EPHA2	EFNA1_EPHA2
6272	TP53	EFNA1	EPHA2	EFNA1_EPHA2
6273	HIC1	EFNA1	EPHA2	EFNA1_EPHA2
6274	AES	EFNA1	EPHA3	EFNA1_EPHA3
6275	REL	VEGFA	EPHB2	VEGFA_EPHB2
6276	BTF3	VEGFA	EPHB2	VEGFA_EPHB2
6277	NFKB1	NUCB2	ERAP1	NUCB2_ERAP1
6278	RELA	NUCB2	ERAP1	NUCB2_ERAP1
6279	PURA	HLA-A	ERBB2	HLA-A_ERBB2
6280	PURA	UBA52	ERBB2	UBA52_ERBB2
6281	PURA	HBEGF	ERBB2	HBEGF_ERBB2
6282	PURA	HSP90B1	ERBB2	HSP90B1_ERBB2
6283	PURA	EREG	ERBB2	EREG_ERBB2
6284	EP300	HLA-A	ERBB2	HLA-A_ERBB2
6285	EP300	UBA52	ERBB2	UBA52_ERBB2
6286	EP300	HBEGF	ERBB2	HBEGF_ERBB2
6287	EP300	HSP90B1	ERBB2	HSP90B1_ERBB2
6288	EP300	EREG	ERBB2	EREG_ERBB2
6289	GATA4	HLA-A	ERBB2	HLA-A_ERBB2
6290	GATA4	UBA52	ERBB2	UBA52_ERBB2
6291	GATA4	HBEGF	ERBB2	HBEGF_ERBB2
6292	GATA4	HSP90B1	ERBB2	HSP90B1_ERBB2
6293	GATA4	EREG	ERBB2	EREG_ERBB2
6294	VDR	HLA-A	ERBB2	HLA-A_ERBB2
6295	VDR	UBA52	ERBB2	UBA52_ERBB2
6296	VDR	HBEGF	ERBB2	HBEGF_ERBB2
6297	VDR	HSP90B1	ERBB2	HSP90B1_ERBB2
6298	VDR	EREG	ERBB2	EREG_ERBB2
6299	TFAP2C	HLA-A	ERBB2	HLA-A_ERBB2
6300	TFAP2C	UBA52	ERBB2	UBA52_ERBB2
6301	TFAP2C	HBEGF	ERBB2	HBEGF_ERBB2
6302	TFAP2C	HSP90B1	ERBB2	HSP90B1_ERBB2
6303	TFAP2C	EREG	ERBB2	EREG_ERBB2
6304	GABPA	HLA-A	ERBB2	HLA-A_ERBB2
6305	GABPA	UBA52	ERBB2	UBA52_ERBB2
6306	GABPA	HBEGF	ERBB2	HBEGF_ERBB2
6307	GABPA	HSP90B1	ERBB2	HSP90B1_ERBB2
6308	GABPA	EREG	ERBB2	EREG_ERBB2
6309	YBX1	HLA-A	ERBB2	HLA-A_ERBB2
6310	YBX1	UBA52	ERBB2	UBA52_ERBB2
6311	YBX1	HBEGF	ERBB2	HBEGF_ERBB2
6312	YBX1	HSP90B1	ERBB2	HSP90B1_ERBB2
6313	YBX1	EREG	ERBB2	EREG_ERBB2
6314	ETV4	HLA-A	ERBB2	HLA-A_ERBB2
6315	ETV4	UBA52	ERBB2	UBA52_ERBB2
6316	ETV4	HBEGF	ERBB2	HBEGF_ERBB2
6317	ETV4	HSP90B1	ERBB2	HSP90B1_ERBB2
6318	ETV4	EREG	ERBB2	EREG_ERBB2
6319	SP4	HLA-A	ERBB2	HLA-A_ERBB2
6320	SP4	UBA52	ERBB2	UBA52_ERBB2
6321	SP4	HBEGF	ERBB2	HBEGF_ERBB2

6322 SP4	HSP90B1	ERBB2	HSP90B1_ERBB2
6323 SP4	EREG	ERBB2	EREG_ERBB2
6324 CREB1	HLA-A	ERBB2	HLA-A_ERBB2
6325 CREB1	UBA52	ERBB2	UBA52_ERBB2
6326 CREB1	HBEGF	ERBB2	HBEGF_ERBB2
6327 CREB1	HSP90B1	ERBB2	HSP90B1_ERBB2
6328 CREB1	EREG	ERBB2	EREG_ERBB2
6329 AR	HLA-A	ERBB2	HLA-A_ERBB2
6330 AR	UBA52	ERBB2	UBA52_ERBB2
6331 AR	HBEGF	ERBB2	HBEGF_ERBB2
6332 AR	HSP90B1	ERBB2	HSP90B1_ERBB2
6333 AR	EREG	ERBB2	EREG_ERBB2
6334 PAX2	HLA-A	ERBB2	HLA-A_ERBB2
6335 PAX2	UBA52	ERBB2	UBA52_ERBB2
6336 PAX2	HBEGF	ERBB2	HBEGF_ERBB2
6337 PAX2	HSP90B1	ERBB2	HSP90B1_ERBB2
6338 PAX2	EREG	ERBB2	EREG_ERBB2
6339 FOXP3	HLA-A	ERBB2	HLA-A_ERBB2
6340 FOXP3	UBA52	ERBB2	UBA52_ERBB2
6341 FOXP3	HBEGF	ERBB2	HBEGF_ERBB2
6342 FOXP3	HSP90B1	ERBB2	HSP90B1_ERBB2
6343 FOXP3	EREG	ERBB2	EREG_ERBB2
6344 ATF7	HLA-A	ERBB2	HLA-A_ERBB2
6345 ATF7	UBA52	ERBB2	UBA52_ERBB2
6346 ATF7	HBEGF	ERBB2	HBEGF_ERBB2
6347 ATF7	HSP90B1	ERBB2	HSP90B1_ERBB2
6348 ATF7	EREG	ERBB2	EREG_ERBB2
6349 SP3	HLA-A	ERBB2	HLA-A_ERBB2
6350 SP3	UBA52	ERBB2	UBA52_ERBB2
6351 SP3	HSP90B1	ERBB2	HSP90B1_ERBB2
6352 SP3	EREG	ERBB2	EREG_ERBB2
6353 YY1	HLA-A	ERBB2	HLA-A_ERBB2
6354 YY1	UBA52	ERBB2	UBA52_ERBB2
6355 YY1	HBEGF	ERBB2	HBEGF_ERBB2
6356 YY1	HSP90B1	ERBB2	HSP90B1_ERBB2
6357 YY1	EREG	ERBB2	EREG_ERBB2
6358 XRCC5	HLA-A	ERBB2	HLA-A_ERBB2
6359 XRCC5	UBA52	ERBB2	UBA52_ERBB2
6360 XRCC5	HBEGF	ERBB2	HBEGF_ERBB2
6361 XRCC5	HSP90B1	ERBB2	HSP90B1_ERBB2
6362 XRCC5	EREG	ERBB2	EREG_ERBB2
6363 SP1	HLA-A	ERBB2	HLA-A_ERBB2
6364 SP1	UBA52	ERBB2	UBA52_ERBB2
6365 SP1	HSP90B1	ERBB2	HSP90B1_ERBB2
6366 SP1	EREG	ERBB2	EREG_ERBB2
6367 ATF1	HLA-A	ERBB2	HLA-A_ERBB2
6368 ATF1	UBA52	ERBB2	UBA52_ERBB2
6369 ATF1	HBEGF	ERBB2	HBEGF_ERBB2
6370 ATF1	HSP90B1	ERBB2	HSP90B1_ERBB2
6371 ATF1	EREG	ERBB2	EREG_ERBB2
6372 ELK1	HLA-A	ERBB2	HLA-A_ERBB2
6373 ELK1	UBA52	ERBB2	UBA52_ERBB2
6374 ELK1	HBEGF	ERBB2	HBEGF_ERBB2
6375 ELK1	HSP90B1	ERBB2	HSP90B1_ERBB2
6376 ELK1	EREG	ERBB2	EREG_ERBB2
6377 PGR	HLA-A	ERBB2	HLA-A_ERBB2
6378 PGR	UBA52	ERBB2	UBA52_ERBB2
6379 PGR	HBEGF	ERBB2	HBEGF_ERBB2

6380 PGR	HSP90B1	ERBB2	HSP90B1_ERBB2
6381 PGR	EREG	ERBB2	EREG_ERBB2
6382 MYB	HLA-A	ERBB2	HLA-A_ERBB2
6383 MYB	UBA52	ERBB2	UBA52_ERBB2
6384 MYB	HBEGF	ERBB2	HBEGF_ERBB2
6385 MYB	HSP90B1	ERBB2	HSP90B1_ERBB2
6386 MYB	EREG	ERBB2	EREG_ERBB2
6387 ELF1	HLA-A	ERBB2	HLA-A_ERBB2
6388 ELF1	UBA52	ERBB2	UBA52_ERBB2
6389 ELF1	HBEGF	ERBB2	HBEGF_ERBB2
6390 ELF1	HSP90B1	ERBB2	HSP90B1_ERBB2
6391 ELF1	EREG	ERBB2	EREG_ERBB2
6392 DENND4A	HLA-A	ERBB2	HLA-A_ERBB2
6393 DENND4A	UBA52	ERBB2	UBA52_ERBB2
6394 DENND4A	HBEGF	ERBB2	HBEGF_ERBB2
6395 DENND4A	HSP90B1	ERBB2	HSP90B1_ERBB2
6396 DENND4A	EREG	ERBB2	EREG_ERBB2
6397 JUND	HLA-A	ERBB2	HLA-A_ERBB2
6398 JUND	UBA52	ERBB2	UBA52_ERBB2
6399 JUND	HBEGF	ERBB2	HBEGF_ERBB2
6400 JUND	HSP90B1	ERBB2	HSP90B1_ERBB2
6401 JUND	EREG	ERBB2	EREG_ERBB2
6402 TFAP2A	HLA-A	ERBB2	HLA-A_ERBB2
6403 TFAP2A	UBA52	ERBB2	UBA52_ERBB2
6404 TFAP2A	HBEGF	ERBB2	HBEGF_ERBB2
6405 TFAP2A	HSP90B1	ERBB2	HSP90B1_ERBB2
6406 TFAP2A	EREG	ERBB2	EREG_ERBB2
6407 TFAP2B	HLA-A	ERBB2	HLA-A_ERBB2
6408 TFAP2B	UBA52	ERBB2	UBA52_ERBB2
6409 TFAP2B	HBEGF	ERBB2	HBEGF_ERBB2
6410 TFAP2B	HSP90B1	ERBB2	HSP90B1_ERBB2
6411 TFAP2B	EREG	ERBB2	EREG_ERBB2
6412 XRCC6	HLA-A	ERBB2	HLA-A_ERBB2
6413 XRCC6	UBA52	ERBB2	UBA52_ERBB2
6414 XRCC6	HBEGF	ERBB2	HBEGF_ERBB2
6415 XRCC6	HSP90B1	ERBB2	HSP90B1_ERBB2
6416 XRCC6	EREG	ERBB2	EREG_ERBB2
6417 NCOA3	HLA-A	ERBB2	HLA-A_ERBB2
6418 NCOA3	UBA52	ERBB2	UBA52_ERBB2
6419 NCOA3	HBEGF	ERBB2	HBEGF_ERBB2
6420 NCOA3	HSP90B1	ERBB2	HSP90B1_ERBB2
6421 NCOA3	EREG	ERBB2	EREG_ERBB2
6422 AR	NRG1	ERBB3	NRG1_ERBB3
6423 AR	AREGB	ERBB3	AREGB_ERBB3
6424 AR	L1CAM	ERBB3	L1CAM_ERBB3
6425 AR	NRG2	ERBB3	NRG2_ERBB3
6426 AR	EREG	ERBB3	EREG_ERBB3
6427 AR	BTC	ERBB3	BTC_ERBB3
6428 AR	AREG	ERBB3	AREG_ERBB3
6429 AR	EGF	ERBB3	EGF_ERBB3
6430 TWIST1	NRG1	ERBB3	NRG1_ERBB3
6431 TWIST1	TGFA	ERBB3	TGFA_ERBB3
6432 TWIST1	AREGB	ERBB3	AREGB_ERBB3
6433 TWIST1	L1CAM	ERBB3	L1CAM_ERBB3
6434 TWIST1	NRG2	ERBB3	NRG2_ERBB3
6435 TWIST1	EREG	ERBB3	EREG_ERBB3
6436 TWIST1	BTC	ERBB3	BTC_ERBB3
6437 TWIST1	AREG	ERBB3	AREG_ERBB3

6438	TWIST1	EGF	ERBB3	EGF_ERBB3
6439	YBX1	NRG1	ERBB3	NRG1_ERBB3
6440	YBX1	TGFA	ERBB3	TGFA_ERBB3
6441	YBX1	AREGB	ERBB3	AREGB_ERBB3
6442	YBX1	L1CAM	ERBB3	L1CAM_ERBB3
6443	YBX1	NRG2	ERBB3	NRG2_ERBB3
6444	YBX1	EREG	ERBB3	EREG_ERBB3
6445	YBX1	BTC	ERBB3	BTC_ERBB3
6446	YBX1	AREG	ERBB3	AREG_ERBB3
6447	YBX1	EGF	ERBB3	EGF_ERBB3
6448	TWIST2	NRG1	ERBB3	NRG1_ERBB3
6449	TWIST2	TGFA	ERBB3	TGFA_ERBB3
6450	TWIST2	AREGB	ERBB3	AREGB_ERBB3
6451	TWIST2	L1CAM	ERBB3	L1CAM_ERBB3
6452	TWIST2	NRG2	ERBB3	NRG2_ERBB3
6453	TWIST2	EREG	ERBB3	EREG_ERBB3
6454	TWIST2	BTC	ERBB3	BTC_ERBB3
6455	TWIST2	AREG	ERBB3	AREG_ERBB3
6456	TWIST2	EGF	ERBB3	EGF_ERBB3
6457	WWP1	HBEGF	ERBB4	HBEGF_ERBB4
6458	WWP1	MMP7	ERBB4	MMP7_ERBB4
6459	WWP1	EREG	ERBB4	EREG_ERBB4
6460	STAT3	GNAI2	F2R	GNAI2_F2R
6461	SP1	GNAI2	F2R	GNAI2_F2R
6462	SP3	GNAI2	F2R	GNAI2_F2R
6463	TFAP2A	GNAI2	F2R	GNAI2_F2R
6464	TWIST2	GNAI2	F2R	GNAI2_F2R
6465	TWIST1	GNAI2	F2R	GNAI2_F2R
6466	JUN	TFPI	F3	TFPI_F3
6467	JUND	TFPI	F3	TFPI_F3
6468	SP1	TFPI	F3	TFPI_F3
6469	EGR1	TFPI	F3	TFPI_F3
6470	REL	TFPI	F3	TFPI_F3
6471	NFKB1	TFPI	F3	TFPI_F3
6472	RELA	TFPI	F3	TFPI_F3
6473	GLI2	CALM1	FAS	CALM1_FAS
6474	GLI2	FASLG	FAS	FASLG_FAS
6475	NFKB1	CALM1	FAS	CALM1_FAS
6476	RELA	CALM1	FAS	CALM1_FAS
6477	SP1	CALM1	FAS	CALM1_FAS
6478	PURA	CALM1	FAS	CALM1_FAS
6479	PURA	FASLG	FAS	FASLG_FAS
6480	TP53	CALM1	FAS	CALM1_FAS
6481	TP53	FASLG	FAS	FASLG_FAS
6482	JUN	CALM1	FAS	CALM1_FAS
6483	STAT1	CALM1	FAS	CALM1_FAS
6484	STAT1	FASLG	FAS	FASLG_FAS
6485	SREBF1	CALM1	FAS	CALM1_FAS
6486	SREBF1	FASLG	FAS	FASLG_FAS
6487	NFYC	CALM1	FAS	CALM1_FAS
6488	NFYC	FASLG	FAS	FASLG_FAS
6489	GLI1	CALM1	FAS	CALM1_FAS
6490	GLI1	FASLG	FAS	FASLG_FAS
6491	YY1	CALM1	FAS	CALM1_FAS
6492	YY1	FASLG	FAS	FASLG_FAS
6493	HDGF	CALM1	FAS	CALM1_FAS
6494	HDGF	FASLG	FAS	FASLG_FAS
6495	NFKBIA	CALM1	FAS	CALM1_FAS

6496	NFKBIA	FASLG	FAS	FASLG_FAS
6497	SREBF2	CALM1	FAS	CALM1_FAS
6498	SREBF2	FASLG	FAS	FASLG_FAS
6499	NFYB	CALM1	FAS	CALM1_FAS
6500	NFYB	FASLG	FAS	FASLG_FAS
6501	YBX1	CALM1	FAS	CALM1_FAS
6502	YBX1	FASLG	FAS	FASLG_FAS
6503	NFYA	CALM1	FAS	CALM1_FAS
6504	NFYA	FASLG	FAS	FASLG_FAS
6505	ATM	CALM1	FAS	CALM1_FAS
6506	ATM	FASLG	FAS	FASLG_FAS
6507	STAT3	CALM1	FAS	CALM1_FAS
6508	STAT3	FASLG	FAS	FASLG_FAS
6509	TFAP2A	CALM1	FAS	CALM1_FAS
6510	TFAP2A	FASLG	FAS	FASLG_FAS
6511	FOS	CALM1	FAS	CALM1_FAS
6512	FOS	FASLG	FAS	FASLG_FAS
6513	ATF2	CALM1	FAS	CALM1_FAS
6514	EGR1	CALM1	FAS	CALM1_FAS
6515	USF1	ITIH2	FCER1A	ITIH2_FCER1A
6516	GATA2	ITIH2	FCER1A	ITIH2_FCER1A
6517	SPI1	ITIH2	FCER1A	ITIH2_FCER1A
6518	YY1	ITIH2	FCER1A	ITIH2_FCER1A
6519	GATA1	ITIH2	FCER1A	ITIH2_FCER1A
6520	USF2	ITIH2	FCER1A	ITIH2_FCER1A
6521	ELF1	ITIH2	FCER1A	ITIH2_FCER1A
6522	SPI1	HRG	FCGR1A	HRG_FCGR1A
6523	STAT1	HRG	FCGR1A	HRG_FCGR1A
6524	SP3	HSP90AA1	FGFR3	HSP90AA1_FGFR3
6525	SHOX	HSP90AA1	FGFR3	HSP90AA1_FGFR3
6526	SP4	HSP90AA1	FGFR3	HSP90AA1_FGFR3
6527	SP1	HSP90AA1	FGFR3	HSP90AA1_FGFR3
6528	ETS1	VEGFA	FLT1	VEGFA_FLT1
6529	CREB1	VEGFA	FLT1	VEGFA_FLT1
6530	ATF1	VEGFA	FLT1	VEGFA_FLT1
6531	NR5A1	UBA52	FSHR	UBA52_FSHR
6532	E2F5	UBA52	FSHR	UBA52_FSHR
6533	E2F1	UBA52	FSHR	UBA52_FSHR
6534	MTA2	UBA52	FSHR	UBA52_FSHR
6535	ECD	GNAS	GCGR	GNAS_GCGR
6536	ETS1	CALM2	GP6	CALM2_GP6
6537	ETS1	CALM1	GP6	CALM1_GP6
6538	ETS1	CALM3	GP6	CALM3_GP6
6539	FLI1	CALM2	GP6	CALM2_GP6
6540	FLI1	CALM1	GP6	CALM1_GP6
6541	FLI1	CALM3	GP6	CALM3_GP6
6542	GATA1	CALM2	GP6	CALM2_GP6
6543	GATA1	CALM1	GP6	CALM1_GP6
6544	GATA1	CALM3	GP6	CALM3_GP6
6545	PARP1	B2M	HFE	B2M_HFE
6546	MYC	CALR	HLA-F	CALR_HLA-F
6547	MYC	B2M	HLA-F	B2M_HLA-F
6548	MYCN	CALR	HLA-F	CALR_HLA-F
6549	MYCN	B2M	HLA-F	B2M_HLA-F
6550	RELA	CALR	HLA-F	CALR_HLA-F
6551	RELA	B2M	HLA-F	B2M_HLA-F
6552	NFKB1	CALR	HLA-F	CALR_HLA-F
6553	HIVEP2	CALR	HLA-F	CALR_HLA-F

6554	HIVEP2	B2M	HLA-F	B2M_HLA-F
6555	CIITA	CALR	HLA-F	CALR_HLA-F
6556	CIITA	B2M	HLA-F	B2M_HLA-F
6557	AR	HAS2	HMMR	HAS2_HMMR
6558	AR	CALM1	HMMR	CALM1_HMMR
6559	STAT5A	HDC	HRH1	HDC_HRH1
6560	NR3C1	HDC	HRH1	HDC_HRH1
6561	MYB	HDC	HRH1	HDC_HRH1
6562	SP1	IFNG	IFNGR1	IFNG_IFNGR1
6563	IRF2	IFNG	IFNGR1	IFNG_IFNGR1
6564	VHL	GNAI2	IGF1R	GNAI2_IGF1R
6565	KLF6	GNAI2	IGF1R	GNAI2_IGF1R
6566	WT1	GNAI2	IGF1R	GNAI2_IGF1R
6567	AR	GNAI2	IGF1R	GNAI2_IGF1R
6568	BRCA1	GNAI2	IGF1R	GNAI2_IGF1R
6569	SP1	GNAI2	IGF1R	GNAI2_IGF1R
6570	TP53	GNAI2	IGF1R	GNAI2_IGF1R
6571	ATM	GNAI2	IGF1R	GNAI2_IGF1R
6572	NKX3-1	GNAI2	IGF1R	GNAI2_IGF1R
6573	USF2	GZMB	IGF2R	GZMB_IGF2R
6574	GATA3	IL12A	IL12RB2	IL12A_IL12RB2
6575	STAT6	IL1B	IL1R1	IL1B_IL1R1
6576	GLI1	IL1RN	IL1R2	IL1RN_IL1R2
6577	GLI1	IL1A	IL1R2	IL1A_IL1R2
6578	GLI1	IL1B	IL1R2	IL1B_IL1R2
6579	RELA	IL15	IL2RA	IL15_IL2RA
6580	REL	IL15	IL2RA	IL15_IL2RA
6581	STAT4	IL2	IL2RA	IL2_IL2RA
6582	STAT4	IL15	IL2RA	IL15_IL2RA
6583	STAT4	ICAM1	IL2RA	ICAM1_IL2RA
6584	STAT1	IL2	IL2RA	IL2_IL2RA
6585	STAT1	IL15	IL2RA	IL15_IL2RA
6586	STAT3	IL15	IL2RA	IL15_IL2RA
6587	RXRA	IL2	IL2RA	IL2_IL2RA
6588	RXRA	IL15	IL2RA	IL15_IL2RA
6589	RXRA	ICAM1	IL2RA	ICAM1_IL2RA
6590	POU2F1	IL15	IL2RA	IL15_IL2RA
6591	POU2F1	ICAM1	IL2RA	ICAM1_IL2RA
6592	NFKB1	IL15	IL2RA	IL15_IL2RA
6593	FOXP3	IL15	IL2RA	IL15_IL2RA
6594	FOXP3	ICAM1	IL2RA	ICAM1_IL2RA
6595	SATB1	IL15	IL2RA	IL15_IL2RA
6596	SATB1	ICAM1	IL2RA	ICAM1_IL2RA
6597	MSC	IL2	IL2RA	IL2_IL2RA
6598	MSC	IL15	IL2RA	IL15_IL2RA
6599	MSC	ICAM1	IL2RA	ICAM1_IL2RA
6600	WT1	IL15	IL2RB	IL15_IL2RB
6601	WT1	IL2	IL2RB	IL2_IL2RB
6602	RXRA	IL15	IL2RB	IL15_IL2RB
6603	RXRA	IL2	IL2RB	IL2_IL2RB
6604	ETS1	IL15	IL2RB	IL15_IL2RB
6605	EGR1	IL15	IL2RB	IL15_IL2RB
6606	SP1	IL15	IL2RB	IL15_IL2RB
6607	RXRA	IL9	IL2RG	IL9_IL2RG
6608	RXRA	IL21	IL2RG	IL21_IL2RG
6609	RXRA	IL15	IL2RG	IL15_IL2RG
6610	RXRA	IL4	IL2RG	IL4_IL2RG
6611	RXRA	IL2	IL2RG	IL2_IL2RG

6612	RXRA	IL7	IL2RG	IL7_IL2RG
6613	RXRA	ICAM1	IL2RG	ICAM1_IL2RG
6614	RXRA	IL13	IL2RG	IL13_IL2RG
6615	TCF3	IL5	IL5RA	IL5_IL5RA
6616	RFX1	IL5	IL5RA	IL5_IL5RA
6617	RFX3	IL5	IL5RA	IL5_IL5RA
6618	CREM	IL5	IL5RA	IL5_IL5RA
6619	JUN	IL5	IL5RA	IL5_IL5RA
6620	CREB1	IL5	IL5RA	IL5_IL5RA
6621	RFX2	IL5	IL5RA	IL5_IL5RA
6622	SP1	CALM3	INSR	CALM3_INSR
6623	SP1	IGF1	INSR	IGF1_INSR
6624	SP1	CALM1	INSR	CALM1_INSR
6625	SP1	AHSG	INSR	AHSG_INSR
6626	SP1	SORBS1	INSR	SORBS1_INSR
6627	SP1	CALM2	INSR	CALM2_INSR
6628	SP1	INS	INSR	INS_INSR
6629	SP1	ARF1	INSR	ARF1_INSR
6630	SP1	NAMPT	INSR	NAMPT_INSR
6631	SP1	GIP	INSR	GIP_INSR
6632	HMGA1	CALM3	INSR	CALM3_INSR
6633	HMGA1	HRAS	INSR	HRAS_INSR
6634	HMGA1	IGF1	INSR	IGF1_INSR
6635	HMGA1	CALM1	INSR	CALM1_INSR
6636	HMGA1	IGF2	INSR	IGF2_INSR
6637	HMGA1	AHSG	INSR	AHSG_INSR
6638	HMGA1	SORBS1	INSR	SORBS1_INSR
6639	HMGA1	CALM2	INSR	CALM2_INSR
6640	HMGA1	INS	INSR	INS_INSR
6641	HMGA1	ARF1	INSR	ARF1_INSR
6642	HMGA1	NAMPT	INSR	NAMPT_INSR
6643	HMGA1	GIP	INSR	GIP_INSR
6644	CEBPB	CALM3	INSR	CALM3_INSR
6645	CEBPB	HRAS	INSR	HRAS_INSR
6646	CEBPB	IGF1	INSR	IGF1_INSR
6647	CEBPB	CALM1	INSR	CALM1_INSR
6648	CEBPB	IGF2	INSR	IGF2_INSR
6649	CEBPB	AHSG	INSR	AHSG_INSR
6650	CEBPB	SORBS1	INSR	SORBS1_INSR
6651	CEBPB	CALM2	INSR	CALM2_INSR
6652	CEBPB	INS	INSR	INS_INSR
6653	CEBPB	ARF1	INSR	ARF1_INSR
6654	CEBPB	NAMPT	INSR	NAMPT_INSR
6655	CEBPB	GIP	INSR	GIP_INSR
6656	ESR2	CALM3	INSR	CALM3_INSR
6657	ESR2	HRAS	INSR	HRAS_INSR
6658	ESR2	IGF1	INSR	IGF1_INSR
6659	ESR2	CALM1	INSR	CALM1_INSR
6660	ESR2	IGF2	INSR	IGF2_INSR
6661	ESR2	AHSG	INSR	AHSG_INSR
6662	ESR2	SORBS1	INSR	SORBS1_INSR
6663	ESR2	CALM2	INSR	CALM2_INSR
6664	ESR2	INS	INSR	INS_INSR
6665	ESR2	ARF1	INSR	ARF1_INSR
6666	ESR2	NAMPT	INSR	NAMPT_INSR
6667	ESR2	GIP	INSR	GIP_INSR
6668	CEBPA	CALM3	INSR	CALM3_INSR
6669	CEBPA	HRAS	INSR	HRAS_INSR

6670	CEBPA	CALM1	INSR	CALM1_INSR
6671	CEBPA	AHSG	INSR	AHSG_INSR
6672	CEBPA	SORBS1	INSR	SORBS1_INSR
6673	CEBPA	CALM2	INSR	CALM2_INSR
6674	CEBPA	INS	INSR	INS_INSR
6675	CEBPA	ARF1	INSR	ARF1_INSR
6676	CEBPA	NAMPT	INSR	NAMPT_INSR
6677	CEBPA	GIP	INSR	GIP_INSR
6678	SP3	COL4A4	ITGA2	COL4A4_ITGA2
6679	SP3	FN1	ITGA2	FN1_ITGA2
6680	SP1	COL4A4	ITGA2	COL4A4_ITGA2
6681	SP1	FN1	ITGA2	FN1_ITGA2
6682	STAT6	CALR	ITGA2B	CALR_ITGA2B
6683	STAT6	THBS1	ITGA2B	THBS1_ITGA2B
6684	STAT6	FN1	ITGA2B	FN1_ITGA2B
6685	SPI1	CALR	ITGA2B	CALR_ITGA2B
6686	SPI1	THBS1	ITGA2B	THBS1_ITGA2B
6687	SPI1	FN1	ITGA2B	FN1_ITGA2B
6688	RUNX1	CALR	ITGA2B	CALR_ITGA2B
6689	RUNX1	THBS1	ITGA2B	THBS1_ITGA2B
6690	RUNX1	FN1	ITGA2B	FN1_ITGA2B
6691	FLI1	CALR	ITGA2B	CALR_ITGA2B
6692	FLI1	THBS1	ITGA2B	THBS1_ITGA2B
6693	FLI1	FN1	ITGA2B	FN1_ITGA2B
6694	GATA1	CALR	ITGA2B	CALR_ITGA2B
6695	GATA1	THBS1	ITGA2B	THBS1_ITGA2B
6696	GATA1	FN1	ITGA2B	FN1_ITGA2B
6697	SP1	FN1	ITGA5	FN1_ITGA5
6698	NFIC	SPP1	ITGA5	SPP1_ITGA5
6699	NFIC	FN1	ITGA5	FN1_ITGA5
6700	PAX6	SPP1	ITGA5	SPP1_ITGA5
6701	PAX6	FN1	ITGA5	FN1_ITGA5
6702	HOXD3	SPP1	ITGA5	SPP1_ITGA5
6703	HOXD3	FN1	ITGA5	FN1_ITGA5
6704	ZEB2	SPP1	ITGA5	SPP1_ITGA5
6705	ZEB2	FN1	ITGA5	FN1_ITGA5
6706	POU5F1	FN1	ITGA6	FN1_ITGA6
6707	POU5F1	THBS1	ITGA6	THBS1_ITGA6
6708	SOX2	FN1	ITGA6	FN1_ITGA6
6709	SOX2	THBS1	ITGA6	THBS1_ITGA6
6710	TBX5	VEGFA	ITGA9	VEGFA_ITGA9
6711	TBX5	TGM2	ITGA9	TGM2_ITGA9
6712	TBX5	SPP1	ITGA9	SPP1_ITGA9
6713	TBX5	VCAM1	ITGA9	VCAM1_ITGA9
6714	TBX5	FN1	ITGA9	FN1_ITGA9
6715	MEIS1	VEGFA	ITGA9	VEGFA_ITGA9
6716	MEIS1	TGM2	ITGA9	TGM2_ITGA9
6717	MEIS1	SPP1	ITGA9	SPP1_ITGA9
6718	MEIS1	VCAM1	ITGA9	VCAM1_ITGA9
6719	MEIS1	FN1	ITGA9	FN1_ITGA9
6720	KLF10	ICAM3	ITGAD	ICAM3_ITGAD
6721	KLF10	VCAM1	ITGAD	VCAM1_ITGAD
6722	SP1	ICAM3	ITGAD	ICAM3_ITGAD
6723	SP1	VCAM1	ITGAD	VCAM1_ITGAD
6724	SP3	ICAM3	ITGAD	ICAM3_ITGAD
6725	SP3	VCAM1	ITGAD	VCAM1_ITGAD
6726	RFX1	ICAM3	ITGAL	ICAM3_ITGAL
6727	RFX1	ICAM5	ITGAL	ICAM5_ITGAL

6728	RFX1	ICAM2	ITGAL	ICAM2_ITGAL
6729	RFX1	ICAM4	ITGAL	ICAM4_ITGAL
6730	RFX1	ICAM1	ITGAL	ICAM1_ITGAL
6731	RFX1	LYZ	ITGAL	LYZ_ITGAL
6732	SPI1	F10	ITGAM	F10_ITGAM
6733	SPI1	ICAM1	ITGAM	ICAM1_ITGAM
6734	SPI1	ICAM2	ITGAM	ICAM2_ITGAM
6735	SPI1	PLAU	ITGAM	PLAU_ITGAM
6736	SPI1	FGB	ITGAM	FGB_ITGAM
6737	SPI1	LPA	ITGAM	LPA_ITGAM
6738	SPI1	APOB	ITGAM	APOB_ITGAM
6739	SPI1	MMP9	ITGAM	MMP9_ITGAM
6740	SPI1	HP	ITGAM	HP_ITGAM
6741	SPI1	CD40LG	ITGAM	CD40LG_ITGAM
6742	SPI1	SELPLG	ITGAM	SELPLG_ITGAM
6743	SPI1	CYR61	ITGAM	CYR61_ITGAM
6744	SPI1	PLAT	ITGAM	PLAT_ITGAM
6745	SPI1	C3	ITGAM	C3_ITGAM
6746	SPI1	PLG	ITGAM	PLG_ITGAM
6747	SPI1	FGA	ITGAM	FGA_ITGAM
6748	SPI1	CFH	ITGAM	CFH_ITGAM
6749	SPI1	SPON2	ITGAM	SPON2_ITGAM
6750	SPI1	ICAM4	ITGAM	ICAM4_ITGAM
6751	SPI1	KNG1	ITGAM	KNG1_ITGAM
6752	SPI1	CTGF	ITGAM	CTGF_ITGAM
6753	SPI1	PROC	ITGAM	PROC_ITGAM
6754	SP3	CALR	ITGAV	CALR_ITGAV
6755	SP3	COL4A4	ITGAV	COL4A4_ITGAV
6756	SP3	SPP1	ITGAV	SPP1_ITGAV
6757	SP3	FN1	ITGAV	FN1_ITGAV
6758	HOXD3	CALR	ITGAV	CALR_ITGAV
6759	HOXD3	COL4A4	ITGAV	COL4A4_ITGAV
6760	HOXD3	VEGFA	ITGAV	VEGFA_ITGAV
6761	HOXD3	SPP1	ITGAV	SPP1_ITGAV
6762	HOXD3	FN1	ITGAV	FN1_ITGAV
6763	SP1	CALR	ITGAV	CALR_ITGAV
6764	SP1	COL4A4	ITGAV	COL4A4_ITGAV
6765	SP1	FN1	ITGAV	FN1_ITGAV
6766	JUND	CALR	ITGAV	CALR_ITGAV
6767	JUND	COL4A4	ITGAV	COL4A4_ITGAV
6768	JUND	VEGFA	ITGAV	VEGFA_ITGAV
6769	JUND	SPP1	ITGAV	SPP1_ITGAV
6770	JUND	FN1	ITGAV	FN1_ITGAV
6771	FOSL1	CALR	ITGAV	CALR_ITGAV
6772	FOSL1	COL4A4	ITGAV	COL4A4_ITGAV
6773	FOSL1	VEGFA	ITGAV	VEGFA_ITGAV
6774	FOSL1	SPP1	ITGAV	SPP1_ITGAV
6775	FOSL1	FN1	ITGAV	FN1_ITGAV
6776	SPI1	ICAM1	ITGAX	ICAM1_ITGAX
6777	SPI1	C3	ITGAX	C3_ITGAX
6778	SPI1	FGA	ITGAX	FGA_ITGAX
6779	WT1	ICAM1	ITGAX	ICAM1_ITGAX
6780	WT1	C3	ITGAX	C3_ITGAX
6781	WT1	FGA	ITGAX	FGA_ITGAX
6782	NFKB1	C3	ITGAX	C3_ITGAX
6783	NFKB1	FGA	ITGAX	FGA_ITGAX
6784	RELA	C3	ITGAX	C3_ITGAX
6785	RELA	FGA	ITGAX	FGA_ITGAX

6786	CEBPA	C3	ITGAX	C3_ITGAX
6787	CEBPA	FGA	ITGAX	FGA_ITGAX
6788	SP1	C3	ITGAX	C3_ITGAX
6789	SP1	FGA	ITGAX	FGA_ITGAX
6790	JUN	ICAM1	ITGAX	ICAM1_ITGAX
6791	JUN	C3	ITGAX	C3_ITGAX
6792	JUN	FGA	ITGAX	FGA_ITGAX
6793	HOXD1	PLAU	ITGB1	PLAU_ITGB1
6794	HOXD1	LAMB1	ITGB1	LAMB1_ITGB1
6795	HOXD1	COL11A1	ITGB1	COL11A1_ITGB1
6796	HOXD1	ADAM17	ITGB1	ADAM17_ITGB1
6797	HOXD1	FGA	ITGB1	FGA_ITGB1
6798	HOXD1	VCAN	ITGB1	VCAN_ITGB1
6799	HOXD1	TGM2	ITGB1	TGM2_ITGB1
6800	HOXD1	ANGPT1	ITGB1	ANGPT1_ITGB1
6801	HOXD1	COL1A2	ITGB1	COL1A2_ITGB1
6802	HOXD1	COL4A5	ITGB1	COL4A5_ITGB1
6803	HOXD1	VTN	ITGB1	VTN_ITGB1
6804	HOXD1	VEGFA	ITGB1	VEGFA_ITGB1
6805	HOXD1	DUSP18	ITGB1	DUSP18_ITGB1
6806	HOXD1	COL6A2	ITGB1	COL6A2_ITGB1
6807	HOXD1	FIGF	ITGB1	FIGF_ITGB1
6808	HOXD1	ADAM15	ITGB1	ADAM15_ITGB1
6809	HOXD1	TNC	ITGB1	TNC_ITGB1
6810	HOXD1	NID1	ITGB1	NID1_ITGB1
6811	HOXD1	ADAM2	ITGB1	ADAM2_ITGB1
6812	HOXD1	COL6A3	ITGB1	COL6A3_ITGB1
6813	HOXD1	F13A1	ITGB1	F13A1_ITGB1
6814	HOXD1	HSPG2	ITGB1	HSPG2_ITGB1
6815	HOXD1	ADAM12	ITGB1	ADAM12_ITGB1
6816	HOXD1	COL2A1	ITGB1	COL2A1_ITGB1
6817	HOXD1	TIMP2	ITGB1	TIMP2_ITGB1
6818	HOXD1	COL5A1	ITGB1	COL5A1_ITGB1
6819	HOXD1	COL4A3	ITGB1	COL4A3_ITGB1
6820	HOXD1	PLG	ITGB1	PLG_ITGB1
6821	HOXD1	FGG	ITGB1	FGG_ITGB1
6822	HOXD1	MDK	ITGB1	MDK_ITGB1
6823	HOXD1	COL1A1	ITGB1	COL1A1_ITGB1
6824	HOXD1	COL7A1	ITGB1	COL7A1_ITGB1
6825	HOXD1	COL4A4	ITGB1	COL4A4_ITGB1
6826	HOXD1	CHAD	ITGB1	CHAD_ITGB1
6827	HOXD1	LAMA1	ITGB1	LAMA1_ITGB1
6828	HOXD1	SEMA7A	ITGB1	SEMA7A_ITGB1
6829	HOXD1	LAMC1	ITGB1	LAMC1_ITGB1
6830	HOXD1	COL18A1	ITGB1	COL18A1_ITGB1
6831	HOXD1	COL4A1	ITGB1	COL4A1_ITGB1
6832	HOXD1	ADAM9	ITGB1	ADAM9_ITGB1
6833	HOXD1	NPNT	ITGB1	NPNT_ITGB1
6834	HOXD1	LAMC3	ITGB1	LAMC3_ITGB1
6835	HOXD1	CXCL12	ITGB1	CXCL12_ITGB1
6836	HOXD1	LAMC2	ITGB1	LAMC2_ITGB1
6837	HOXD1	ICAM4	ITGB1	ICAM4_ITGB1
6838	HOXD1	LGALS3BP	ITGB1	LGALS3BP_ITGB1
6839	HOXD1	LAMB3	ITGB1	LAMB3_ITGB1
6840	HOXD1	LAMA2	ITGB1	LAMA2_ITGB1
6841	HOXD1	FGB	ITGB1	FGB_ITGB1
6842	HOXD1	FN1	ITGB1	FN1_ITGB1
6843	HOXD1	COL6A1	ITGB1	COL6A1_ITGB1

6844	HOXD1	COL5A2	ITGB1	COL5A2_ITGB1
6845	HOXD1	CD14	ITGB1	CD14_ITGB1
6846	HOXD1	THBS1	ITGB1	THBS1_ITGB1
6847	HOXD1	CSF2	ITGB1	CSF2_ITGB1
6848	HOXD1	VEGFC	ITGB1	VEGFC_ITGB1
6849	HOXD1	FBN1	ITGB1	FBN1_ITGB1
6850	HOXD1	SPP1	ITGB1	SPP1_ITGB1
6851	HOXD1	THBS2	ITGB1	THBS2_ITGB1
6852	HOXD1	RELN	ITGB1	RELN_ITGB1
6853	HOXD1	COL4A6	ITGB1	COL4A6_ITGB1
6854	HOXD1	LAMA4	ITGB1	LAMA4_ITGB1
6855	HOXD1	FBLN1	ITGB1	FBLN1_ITGB1
6856	HOXD1	MATN1	ITGB1	MATN1_ITGB1
6857	HOXD1	COL3A1	ITGB1	COL3A1_ITGB1
6858	HOXD1	LAMA5	ITGB1	LAMA5_ITGB1
6859	HOXD1	VCAM1	ITGB1	VCAM1_ITGB1
6860	PAX6	PLAU	ITGB1	PLAU_ITGB1
6861	PAX6	LAMB1	ITGB1	LAMB1_ITGB1
6862	PAX6	COL11A1	ITGB1	COL11A1_ITGB1
6863	PAX6	ADAM17	ITGB1	ADAM17_ITGB1
6864	PAX6	FGA	ITGB1	FGA_ITGB1
6865	PAX6	VCAN	ITGB1	VCAN_ITGB1
6866	PAX6	TGM2	ITGB1	TGM2_ITGB1
6867	PAX6	ANGPT1	ITGB1	ANGPT1_ITGB1
6868	PAX6	COL1A2	ITGB1	COL1A2_ITGB1
6869	PAX6	COL4A5	ITGB1	COL4A5_ITGB1
6870	PAX6	VTN	ITGB1	VTN_ITGB1
6871	PAX6	VEGFA	ITGB1	VEGFA_ITGB1
6872	PAX6	DUSP18	ITGB1	DUSP18_ITGB1
6873	PAX6	COL6A2	ITGB1	COL6A2_ITGB1
6874	PAX6	FIGF	ITGB1	FIGF_ITGB1
6875	PAX6	ADAM15	ITGB1	ADAM15_ITGB1
6876	PAX6	TNC	ITGB1	TNC_ITGB1
6877	PAX6	NID1	ITGB1	NID1_ITGB1
6878	PAX6	ADAM2	ITGB1	ADAM2_ITGB1
6879	PAX6	COL6A3	ITGB1	COL6A3_ITGB1
6880	PAX6	F13A1	ITGB1	F13A1_ITGB1
6881	PAX6	HSPG2	ITGB1	HSPG2_ITGB1
6882	PAX6	ADAM12	ITGB1	ADAM12_ITGB1
6883	PAX6	COL2A1	ITGB1	COL2A1_ITGB1
6884	PAX6	TIMP2	ITGB1	TIMP2_ITGB1
6885	PAX6	COL5A1	ITGB1	COL5A1_ITGB1
6886	PAX6	COL4A3	ITGB1	COL4A3_ITGB1
6887	PAX6	PLG	ITGB1	PLG_ITGB1
6888	PAX6	FGG	ITGB1	FGG_ITGB1
6889	PAX6	MDK	ITGB1	MDK_ITGB1
6890	PAX6	COL1A1	ITGB1	COL1A1_ITGB1
6891	PAX6	COL7A1	ITGB1	COL7A1_ITGB1
6892	PAX6	COL4A4	ITGB1	COL4A4_ITGB1
6893	PAX6	CHAD	ITGB1	CHAD_ITGB1
6894	PAX6	LAMA1	ITGB1	LAMA1_ITGB1
6895	PAX6	SEMA7A	ITGB1	SEMA7A_ITGB1
6896	PAX6	LAMC1	ITGB1	LAMC1_ITGB1
6897	PAX6	COL18A1	ITGB1	COL18A1_ITGB1
6898	PAX6	COL4A1	ITGB1	COL4A1_ITGB1
6899	PAX6	ADAM9	ITGB1	ADAM9_ITGB1
6900	PAX6	NPNT	ITGB1	NPNT_ITGB1
6901	PAX6	LAMC3	ITGB1	LAMC3_ITGB1

6902	PAX6	CXCL12	ITGB1	CXCL12_ITGB1
6903	PAX6	LAMC2	ITGB1	LAMC2_ITGB1
6904	PAX6	ICAM4	ITGB1	ICAM4_ITGB1
6905	PAX6	LGALS3BP	ITGB1	LGALS3BP_ITGB1
6906	PAX6	LAMB3	ITGB1	LAMB3_ITGB1
6907	PAX6	LAMA2	ITGB1	LAMA2_ITGB1
6908	PAX6	FGB	ITGB1	FGB_ITGB1
6909	PAX6	FN1	ITGB1	FN1_ITGB1
6910	PAX6	COL6A1	ITGB1	COL6A1_ITGB1
6911	PAX6	COL5A2	ITGB1	COL5A2_ITGB1
6912	PAX6	CD14	ITGB1	CD14_ITGB1
6913	PAX6	THBS1	ITGB1	THBS1_ITGB1
6914	PAX6	CSF2	ITGB1	CSF2_ITGB1
6915	PAX6	VEGFC	ITGB1	VEGFC_ITGB1
6916	PAX6	FBN1	ITGB1	FBN1_ITGB1
6917	PAX6	SPP1	ITGB1	SPP1_ITGB1
6918	PAX6	THBS2	ITGB1	THBS2_ITGB1
6919	PAX6	RELN	ITGB1	RELN_ITGB1
6920	PAX6	COL4A6	ITGB1	COL4A6_ITGB1
6921	PAX6	LAMA4	ITGB1	LAMA4_ITGB1
6922	PAX6	FBLN1	ITGB1	FBLN1_ITGB1
6923	PAX6	MATN1	ITGB1	MATN1_ITGB1
6924	PAX6	COL3A1	ITGB1	COL3A1_ITGB1
6925	PAX6	LAMA5	ITGB1	LAMA5_ITGB1
6926	PAX6	VCAM1	ITGB1	VCAM1_ITGB1
6927	TWIST1	PLAU	ITGB1	PLAU_ITGB1
6928	TWIST1	LAMB1	ITGB1	LAMB1_ITGB1
6929	TWIST1	COL11A1	ITGB1	COL11A1_ITGB1
6930	TWIST1	ADAM17	ITGB1	ADAM17_ITGB1
6931	TWIST1	FGA	ITGB1	FGA_ITGB1
6932	TWIST1	VCAN	ITGB1	VCAN_ITGB1
6933	TWIST1	TGM2	ITGB1	TGM2_ITGB1
6934	TWIST1	ANGPT1	ITGB1	ANGPT1_ITGB1
6935	TWIST1	COL1A2	ITGB1	COL1A2_ITGB1
6936	TWIST1	COL4A5	ITGB1	COL4A5_ITGB1
6937	TWIST1	VTN	ITGB1	VTN_ITGB1
6938	TWIST1	VEGFA	ITGB1	VEGFA_ITGB1
6939	TWIST1	DUSP18	ITGB1	DUSP18_ITGB1
6940	TWIST1	COL6A2	ITGB1	COL6A2_ITGB1
6941	TWIST1	FIGF	ITGB1	FIGF_ITGB1
6942	TWIST1	ADAM15	ITGB1	ADAM15_ITGB1
6943	TWIST1	TNC	ITGB1	TNC_ITGB1
6944	TWIST1	NID1	ITGB1	NID1_ITGB1
6945	TWIST1	ADAM2	ITGB1	ADAM2_ITGB1
6946	TWIST1	COL6A3	ITGB1	COL6A3_ITGB1
6947	TWIST1	F13A1	ITGB1	F13A1_ITGB1
6948	TWIST1	HSPG2	ITGB1	HSPG2_ITGB1
6949	TWIST1	ADAM12	ITGB1	ADAM12_ITGB1
6950	TWIST1	COL2A1	ITGB1	COL2A1_ITGB1
6951	TWIST1	TIMP2	ITGB1	TIMP2_ITGB1
6952	TWIST1	COL5A1	ITGB1	COL5A1_ITGB1
6953	TWIST1	COL4A3	ITGB1	COL4A3_ITGB1
6954	TWIST1	PLG	ITGB1	PLG_ITGB1
6955	TWIST1	FGG	ITGB1	FGG_ITGB1
6956	TWIST1	MDK	ITGB1	MDK_ITGB1
6957	TWIST1	COL1A1	ITGB1	COL1A1_ITGB1
6958	TWIST1	COL7A1	ITGB1	COL7A1_ITGB1
6959	TWIST1	COL4A4	ITGB1	COL4A4_ITGB1

6960	TWIST1	CHAD	ITGB1	CHAD_ITGB1
6961	TWIST1	LAMA1	ITGB1	LAMA1_ITGB1
6962	TWIST1	SEMA7A	ITGB1	SEMA7A_ITGB1
6963	TWIST1	LAMC1	ITGB1	LAMC1_ITGB1
6964	TWIST1	COL18A1	ITGB1	COL18A1_ITGB1
6965	TWIST1	COL4A1	ITGB1	COL4A1_ITGB1
6966	TWIST1	ADAM9	ITGB1	ADAM9_ITGB1
6967	TWIST1	NPNT	ITGB1	NPNT_ITGB1
6968	TWIST1	LAMC3	ITGB1	LAMC3_ITGB1
6969	TWIST1	CXCL12	ITGB1	CXCL12_ITGB1
6970	TWIST1	LAMC2	ITGB1	LAMC2_ITGB1
6971	TWIST1	ICAM4	ITGB1	ICAM4_ITGB1
6972	TWIST1	LGALS3BP	ITGB1	LGALS3BP_ITGB1
6973	TWIST1	LAMB3	ITGB1	LAMB3_ITGB1
6974	TWIST1	LAMA2	ITGB1	LAMA2_ITGB1
6975	TWIST1	FGB	ITGB1	FGB_ITGB1
6976	TWIST1	COL6A1	ITGB1	COL6A1_ITGB1
6977	TWIST1	COL5A2	ITGB1	COL5A2_ITGB1
6978	TWIST1	CD14	ITGB1	CD14_ITGB1
6979	TWIST1	THBS1	ITGB1	THBS1_ITGB1
6980	TWIST1	CSF2	ITGB1	CSF2_ITGB1
6981	TWIST1	VEGFC	ITGB1	VEGFC_ITGB1
6982	TWIST1	FBN1	ITGB1	FBN1_ITGB1
6983	TWIST1	SPP1	ITGB1	SPP1_ITGB1
6984	TWIST1	THBS2	ITGB1	THBS2_ITGB1
6985	TWIST1	RELN	ITGB1	RELN_ITGB1
6986	TWIST1	COL4A6	ITGB1	COL4A6_ITGB1
6987	TWIST1	LAMA4	ITGB1	LAMA4_ITGB1
6988	TWIST1	FBLN1	ITGB1	FBLN1_ITGB1
6989	TWIST1	MATN1	ITGB1	MATN1_ITGB1
6990	TWIST1	COL3A1	ITGB1	COL3A1_ITGB1
6991	TWIST1	LAMA5	ITGB1	LAMA5_ITGB1
6992	TWIST1	VCAM1	ITGB1	VCAM1_ITGB1
6993	HOXD3	LAMB1	ITGB1	LAMB1_ITGB1
6994	HOXD3	COL11A1	ITGB1	COL11A1_ITGB1
6995	HOXD3	ADAM17	ITGB1	ADAM17_ITGB1
6996	HOXD3	FGA	ITGB1	FGA_ITGB1
6997	HOXD3	VCAN	ITGB1	VCAN_ITGB1
6998	HOXD3	TGM2	ITGB1	TGM2_ITGB1
6999	HOXD3	ANGPT1	ITGB1	ANGPT1_ITGB1
7000	HOXD3	COL1A2	ITGB1	COL1A2_ITGB1
7001	HOXD3	COL4A5	ITGB1	COL4A5_ITGB1
7002	HOXD3	VTN	ITGB1	VTN_ITGB1
7003	HOXD3	VEGFA	ITGB1	VEGFA_ITGB1
7004	HOXD3	DUSP18	ITGB1	DUSP18_ITGB1
7005	HOXD3	COL6A2	ITGB1	COL6A2_ITGB1
7006	HOXD3	FIGF	ITGB1	FIGF_ITGB1
7007	HOXD3	ADAM15	ITGB1	ADAM15_ITGB1
7008	HOXD3	TNC	ITGB1	TNC_ITGB1
7009	HOXD3	NID1	ITGB1	NID1_ITGB1
7010	HOXD3	ADAM2	ITGB1	ADAM2_ITGB1
7011	HOXD3	COL6A3	ITGB1	COL6A3_ITGB1
7012	HOXD3	F13A1	ITGB1	F13A1_ITGB1
7013	HOXD3	HSPG2	ITGB1	HSPG2_ITGB1
7014	HOXD3	ADAM12	ITGB1	ADAM12_ITGB1
7015	HOXD3	COL2A1	ITGB1	COL2A1_ITGB1
7016	HOXD3	TIMP2	ITGB1	TIMP2_ITGB1
7017	HOXD3	COL5A1	ITGB1	COL5A1_ITGB1

7018	HOXD3	COL4A3	ITGB1	COL4A3_ITGB1
7019	HOXD3	PLG	ITGB1	PLG_ITGB1
7020	HOXD3	FGG	ITGB1	FGG_ITGB1
7021	HOXD3	MDK	ITGB1	MDK_ITGB1
7022	HOXD3	COL1A1	ITGB1	COL1A1_ITGB1
7023	HOXD3	COL7A1	ITGB1	COL7A1_ITGB1
7024	HOXD3	COL4A4	ITGB1	COL4A4_ITGB1
7025	HOXD3	CHAD	ITGB1	CHAD_ITGB1
7026	HOXD3	LAMA1	ITGB1	LAMA1_ITGB1
7027	HOXD3	SEMA7A	ITGB1	SEMA7A_ITGB1
7028	HOXD3	LAMC1	ITGB1	LAMC1_ITGB1
7029	HOXD3	COL18A1	ITGB1	COL18A1_ITGB1
7030	HOXD3	COL4A1	ITGB1	COL4A1_ITGB1
7031	HOXD3	ADAM9	ITGB1	ADAM9_ITGB1
7032	HOXD3	NPNT	ITGB1	NPNT_ITGB1
7033	HOXD3	LAMC3	ITGB1	LAMC3_ITGB1
7034	HOXD3	CXCL12	ITGB1	CXCL12_ITGB1
7035	HOXD3	LAMC2	ITGB1	LAMC2_ITGB1
7036	HOXD3	ICAM4	ITGB1	ICAM4_ITGB1
7037	HOXD3	LGALS3BP	ITGB1	LGALS3BP_ITGB1
7038	HOXD3	LAMB3	ITGB1	LAMB3_ITGB1
7039	HOXD3	LAMA2	ITGB1	LAMA2_ITGB1
7040	HOXD3	FGB	ITGB1	FGB_ITGB1
7041	HOXD3	FN1	ITGB1	FN1_ITGB1
7042	HOXD3	COL6A1	ITGB1	COL6A1_ITGB1
7043	HOXD3	COL5A2	ITGB1	COL5A2_ITGB1
7044	HOXD3	CD14	ITGB1	CD14_ITGB1
7045	HOXD3	THBS1	ITGB1	THBS1_ITGB1
7046	HOXD3	CSF2	ITGB1	CSF2_ITGB1
7047	HOXD3	VEGFC	ITGB1	VEGFC_ITGB1
7048	HOXD3	FBN1	ITGB1	FBN1_ITGB1
7049	HOXD3	SPP1	ITGB1	SPP1_ITGB1
7050	HOXD3	THBS2	ITGB1	THBS2_ITGB1
7051	HOXD3	RELN	ITGB1	RELN_ITGB1
7052	HOXD3	COL4A6	ITGB1	COL4A6_ITGB1
7053	HOXD3	LAMA4	ITGB1	LAMA4_ITGB1
7054	HOXD3	FBLN1	ITGB1	FBLN1_ITGB1
7055	HOXD3	MATN1	ITGB1	MATN1_ITGB1
7056	HOXD3	COL3A1	ITGB1	COL3A1_ITGB1
7057	HOXD3	LAMA5	ITGB1	LAMA5_ITGB1
7058	HOXD3	VCAM1	ITGB1	VCAM1_ITGB1
7059	TWIST2	PLAU	ITGB1	PLAU_ITGB1
7060	TWIST2	LAMB1	ITGB1	LAMB1_ITGB1
7061	TWIST2	COL11A1	ITGB1	COL11A1_ITGB1
7062	TWIST2	ADAM17	ITGB1	ADAM17_ITGB1
7063	TWIST2	FGA	ITGB1	FGA_ITGB1
7064	TWIST2	VCAN	ITGB1	VCAN_ITGB1
7065	TWIST2	TGM2	ITGB1	TGM2_ITGB1
7066	TWIST2	ANGPT1	ITGB1	ANGPT1_ITGB1
7067	TWIST2	COL1A2	ITGB1	COL1A2_ITGB1
7068	TWIST2	COL4A5	ITGB1	COL4A5_ITGB1
7069	TWIST2	VTN	ITGB1	VTN_ITGB1
7070	TWIST2	VEGFA	ITGB1	VEGFA_ITGB1
7071	TWIST2	DUSP18	ITGB1	DUSP18_ITGB1
7072	TWIST2	COL6A2	ITGB1	COL6A2_ITGB1
7073	TWIST2	FIGF	ITGB1	FIGF_ITGB1
7074	TWIST2	ADAM15	ITGB1	ADAM15_ITGB1
7075	TWIST2	TNC	ITGB1	TNC_ITGB1

7076	TWIST2	NID1	ITGB1	NID1_ITGB1
7077	TWIST2	ADAM2	ITGB1	ADAM2_ITGB1
7078	TWIST2	COL6A3	ITGB1	COL6A3_ITGB1
7079	TWIST2	F13A1	ITGB1	F13A1_ITGB1
7080	TWIST2	HSPG2	ITGB1	HSPG2_ITGB1
7081	TWIST2	ADAM12	ITGB1	ADAM12_ITGB1
7082	TWIST2	COL2A1	ITGB1	COL2A1_ITGB1
7083	TWIST2	TIMP2	ITGB1	TIMP2_ITGB1
7084	TWIST2	COL5A1	ITGB1	COL5A1_ITGB1
7085	TWIST2	COL4A3	ITGB1	COL4A3_ITGB1
7086	TWIST2	PLG	ITGB1	PLG_ITGB1
7087	TWIST2	FGG	ITGB1	FGG_ITGB1
7088	TWIST2	MDK	ITGB1	MDK_ITGB1
7089	TWIST2	COL1A1	ITGB1	COL1A1_ITGB1
7090	TWIST2	COL7A1	ITGB1	COL7A1_ITGB1
7091	TWIST2	COL4A4	ITGB1	COL4A4_ITGB1
7092	TWIST2	CHAD	ITGB1	CHAD_ITGB1
7093	TWIST2	LAMA1	ITGB1	LAMA1_ITGB1
7094	TWIST2	SEMA7A	ITGB1	SEMA7A_ITGB1
7095	TWIST2	LAMC1	ITGB1	LAMC1_ITGB1
7096	TWIST2	COL18A1	ITGB1	COL18A1_ITGB1
7097	TWIST2	COL4A1	ITGB1	COL4A1_ITGB1
7098	TWIST2	ADAM9	ITGB1	ADAM9_ITGB1
7099	TWIST2	NPNT	ITGB1	NPNT_ITGB1
7100	TWIST2	LAMC3	ITGB1	LAMC3_ITGB1
7101	TWIST2	CXCL12	ITGB1	CXCL12_ITGB1
7102	TWIST2	LAMC2	ITGB1	LAMC2_ITGB1
7103	TWIST2	ICAM4	ITGB1	ICAM4_ITGB1
7104	TWIST2	LGALS3BP	ITGB1	LGALS3BP_ITGB1
7105	TWIST2	LAMB3	ITGB1	LAMB3_ITGB1
7106	TWIST2	LAMA2	ITGB1	LAMA2_ITGB1
7107	TWIST2	FGB	ITGB1	FGB_ITGB1
7108	TWIST2	COL6A1	ITGB1	COL6A1_ITGB1
7109	TWIST2	COL5A2	ITGB1	COL5A2_ITGB1
7110	TWIST2	CD14	ITGB1	CD14_ITGB1
7111	TWIST2	THBS1	ITGB1	THBS1_ITGB1
7112	TWIST2	CSF2	ITGB1	CSF2_ITGB1
7113	TWIST2	VEGFC	ITGB1	VEGFC_ITGB1
7114	TWIST2	FBN1	ITGB1	FBN1_ITGB1
7115	TWIST2	SPP1	ITGB1	SPP1_ITGB1
7116	TWIST2	THBS2	ITGB1	THBS2_ITGB1
7117	TWIST2	RELN	ITGB1	RELN_ITGB1
7118	TWIST2	COL4A6	ITGB1	COL4A6_ITGB1
7119	TWIST2	LAMA4	ITGB1	LAMA4_ITGB1
7120	TWIST2	FBLN1	ITGB1	FBLN1_ITGB1
7121	TWIST2	MATN1	ITGB1	MATN1_ITGB1
7122	TWIST2	COL3A1	ITGB1	COL3A1_ITGB1
7123	TWIST2	LAMA5	ITGB1	LAMA5_ITGB1
7124	TWIST2	VCAM1	ITGB1	VCAM1_ITGB1
7125	FOXF2	PLAU	ITGB1	PLAU_ITGB1
7126	FOXF2	LAMB1	ITGB1	LAMB1_ITGB1
7127	FOXF2	COL11A1	ITGB1	COL11A1_ITGB1
7128	FOXF2	ADAM17	ITGB1	ADAM17_ITGB1
7129	FOXF2	FGA	ITGB1	FGA_ITGB1
7130	FOXF2	VCAN	ITGB1	VCAN_ITGB1
7131	FOXF2	TGM2	ITGB1	TGM2_ITGB1
7132	FOXF2	ANGPT1	ITGB1	ANGPT1_ITGB1
7133	FOXF2	COL1A2	ITGB1	COL1A2_ITGB1

7134	FOXF2	COL4A5	ITGB1	COL4A5_ITGB1
7135	FOXF2	VTN	ITGB1	VTN_ITGB1
7136	FOXF2	VEGFA	ITGB1	VEGFA_ITGB1
7137	FOXF2	DUSP18	ITGB1	DUSP18_ITGB1
7138	FOXF2	COL6A2	ITGB1	COL6A2_ITGB1
7139	FOXF2	FIGF	ITGB1	FIGF_ITGB1
7140	FOXF2	ADAM15	ITGB1	ADAM15_ITGB1
7141	FOXF2	TNC	ITGB1	TNC_ITGB1
7142	FOXF2	NID1	ITGB1	NID1_ITGB1
7143	FOXF2	ADAM2	ITGB1	ADAM2_ITGB1
7144	FOXF2	COL6A3	ITGB1	COL6A3_ITGB1
7145	FOXF2	F13A1	ITGB1	F13A1_ITGB1
7146	FOXF2	HSPG2	ITGB1	HSPG2_ITGB1
7147	FOXF2	ADAM12	ITGB1	ADAM12_ITGB1
7148	FOXF2	COL2A1	ITGB1	COL2A1_ITGB1
7149	FOXF2	TIMP2	ITGB1	TIMP2_ITGB1
7150	FOXF2	COL5A1	ITGB1	COL5A1_ITGB1
7151	FOXF2	COL4A3	ITGB1	COL4A3_ITGB1
7152	FOXF2	PLG	ITGB1	PLG_ITGB1
7153	FOXF2	FGG	ITGB1	FGG_ITGB1
7154	FOXF2	MDK	ITGB1	MDK_ITGB1
7155	FOXF2	COL1A1	ITGB1	COL1A1_ITGB1
7156	FOXF2	COL7A1	ITGB1	COL7A1_ITGB1
7157	FOXF2	COL4A4	ITGB1	COL4A4_ITGB1
7158	FOXF2	CHAD	ITGB1	CHAD_ITGB1
7159	FOXF2	LAMA1	ITGB1	LAMA1_ITGB1
7160	FOXF2	SEMA7A	ITGB1	SEMA7A_ITGB1
7161	FOXF2	LAMC1	ITGB1	LAMC1_ITGB1
7162	FOXF2	COL18A1	ITGB1	COL18A1_ITGB1
7163	FOXF2	COL4A1	ITGB1	COL4A1_ITGB1
7164	FOXF2	ADAM9	ITGB1	ADAM9_ITGB1
7165	FOXF2	NPNT	ITGB1	NPNT_ITGB1
7166	FOXF2	LAMC3	ITGB1	LAMC3_ITGB1
7167	FOXF2	LAMC2	ITGB1	LAMC2_ITGB1
7168	FOXF2	ICAM4	ITGB1	ICAM4_ITGB1
7169	FOXF2	LGALS3BP	ITGB1	LGALS3BP_ITGB1
7170	FOXF2	LAMB3	ITGB1	LAMB3_ITGB1
7171	FOXF2	LAMA2	ITGB1	LAMA2_ITGB1
7172	FOXF2	FGB	ITGB1	FGB_ITGB1
7173	FOXF2	FN1	ITGB1	FN1_ITGB1
7174	FOXF2	COL6A1	ITGB1	COL6A1_ITGB1
7175	FOXF2	COL5A2	ITGB1	COL5A2_ITGB1
7176	FOXF2	CD14	ITGB1	CD14_ITGB1
7177	FOXF2	THBS1	ITGB1	THBS1_ITGB1
7178	FOXF2	CSF2	ITGB1	CSF2_ITGB1
7179	FOXF2	VEGFC	ITGB1	VEGFC_ITGB1
7180	FOXF2	FBN1	ITGB1	FBN1_ITGB1
7181	FOXF2	SPP1	ITGB1	SPP1_ITGB1
7182	FOXF2	THBS2	ITGB1	THBS2_ITGB1
7183	FOXF2	RELN	ITGB1	RELN_ITGB1
7184	FOXF2	COL4A6	ITGB1	COL4A6_ITGB1
7185	FOXF2	LAMA4	ITGB1	LAMA4_ITGB1
7186	FOXF2	FBLN1	ITGB1	FBLN1_ITGB1
7187	FOXF2	MATN1	ITGB1	MATN1_ITGB1
7188	FOXF2	COL3A1	ITGB1	COL3A1_ITGB1
7189	FOXF2	LAMA5	ITGB1	LAMA5_ITGB1
7190	FOXF2	VCAM1	ITGB1	VCAM1_ITGB1
7191	SPI1	ICAM3	ITGB2	ICAM3_ITGB2

7192	SPI1	FGA	ITGB2	FGA_ITGB2
7193	SPI1	SPON2	ITGB2	SPON2_ITGB2
7194	SPI1	ICAM2	ITGB2	ICAM2_ITGB2
7195	SPI1	PLAU	ITGB2	PLAU_ITGB2
7196	SPI1	PROC	ITGB2	PROC_ITGB2
7197	SPI1	ICAM5	ITGB2	ICAM5_ITGB2
7198	SPI1	FGB	ITGB2	FGB_ITGB2
7199	SPI1	KNG1	ITGB2	KNG1_ITGB2
7200	SPI1	CYR61	ITGB2	CYR61_ITGB2
7201	SPI1	LPA	ITGB2	LPA_ITGB2
7202	SPI1	CTGF	ITGB2	CTGF_ITGB2
7203	SPI1	HP	ITGB2	HP_ITGB2
7204	SPI1	PLAT	ITGB2	PLAT_ITGB2
7205	SPI1	MMP9	ITGB2	MMP9_ITGB2
7206	SPI1	SELPLG	ITGB2	SELPLG_ITGB2
7207	SPI1	C3	ITGB2	C3_ITGB2
7208	SPI1	ICAM1	ITGB2	ICAM1_ITGB2
7209	SPI1	ICAM4	ITGB2	ICAM4_ITGB2
7210	SPI1	F10	ITGB2	F10_ITGB2
7211	SPI1	FGG	ITGB2	FGG_ITGB2
7212	SPI1	APOB	ITGB2	APOB_ITGB2
7213	SPI1	CD40LG	ITGB2	CD40LG_ITGB2
7214	SPI1	VCAM1	ITGB2	VCAM1_ITGB2
7215	SPI1	PLG	ITGB2	PLG_ITGB2
7216	KLF5	ICAM3	ITGB2	ICAM3_ITGB2
7217	KLF5	FGA	ITGB2	FGA_ITGB2
7218	KLF5	SPON2	ITGB2	SPON2_ITGB2
7219	KLF5	ICAM2	ITGB2	ICAM2_ITGB2
7220	KLF5	PLAU	ITGB2	PLAU_ITGB2
7221	KLF5	PROC	ITGB2	PROC_ITGB2
7222	KLF5	ICAM5	ITGB2	ICAM5_ITGB2
7223	KLF5	FGB	ITGB2	FGB_ITGB2
7224	KLF5	KNG1	ITGB2	KNG1_ITGB2
7225	KLF5	CYR61	ITGB2	CYR61_ITGB2
7226	KLF5	LPA	ITGB2	LPA_ITGB2
7227	KLF5	CTGF	ITGB2	CTGF_ITGB2
7228	KLF5	HP	ITGB2	HP_ITGB2
7229	KLF5	PLAT	ITGB2	PLAT_ITGB2
7230	KLF5	SELPLG	ITGB2	SELPLG_ITGB2
7231	KLF5	C3	ITGB2	C3_ITGB2
7232	KLF5	ICAM1	ITGB2	ICAM1_ITGB2
7233	KLF5	ICAM4	ITGB2	ICAM4_ITGB2
7234	KLF5	F10	ITGB2	F10_ITGB2
7235	KLF5	FGG	ITGB2	FGG_ITGB2
7236	KLF5	APOB	ITGB2	APOB_ITGB2
7237	KLF5	CD40LG	ITGB2	CD40LG_ITGB2
7238	KLF5	VCAM1	ITGB2	VCAM1_ITGB2
7239	KLF5	PLG	ITGB2	PLG_ITGB2
7240	HIF1A	ICAM3	ITGB2	ICAM3_ITGB2
7241	HIF1A	FGA	ITGB2	FGA_ITGB2
7242	HIF1A	SPON2	ITGB2	SPON2_ITGB2
7243	HIF1A	ICAM2	ITGB2	ICAM2_ITGB2
7244	HIF1A	PROC	ITGB2	PROC_ITGB2
7245	HIF1A	ICAM5	ITGB2	ICAM5_ITGB2
7246	HIF1A	FGB	ITGB2	FGB_ITGB2
7247	HIF1A	KNG1	ITGB2	KNG1_ITGB2
7248	HIF1A	CYR61	ITGB2	CYR61_ITGB2
7249	HIF1A	LPA	ITGB2	LPA_ITGB2

7250	HIF1A	HP	ITGB2	HP_ITGB2
7251	HIF1A	PLAT	ITGB2	PLAT_ITGB2
7252	HIF1A	MMP9	ITGB2	MMP9_ITGB2
7253	HIF1A	SELPLG	ITGB2	SELPLG_ITGB2
7254	HIF1A	C3	ITGB2	C3_ITGB2
7255	HIF1A	ICAM1	ITGB2	ICAM1_ITGB2
7256	HIF1A	ICAM4	ITGB2	ICAM4_ITGB2
7257	HIF1A	F10	ITGB2	F10_ITGB2
7258	HIF1A	FGG	ITGB2	FGG_ITGB2
7259	HIF1A	APOB	ITGB2	APOB_ITGB2
7260	HIF1A	CD40LG	ITGB2	CD40LG_ITGB2
7261	HIF1A	VCAM1	ITGB2	VCAM1_ITGB2
7262	HIF1A	PLG	ITGB2	PLG_ITGB2
7263	RUNX1	ICAM3	ITGB2	ICAM3_ITGB2
7264	RUNX1	FGA	ITGB2	FGA_ITGB2
7265	RUNX1	SPON2	ITGB2	SPON2_ITGB2
7266	RUNX1	ICAM2	ITGB2	ICAM2_ITGB2
7267	RUNX1	PLAU	ITGB2	PLAU_ITGB2
7268	RUNX1	PROC	ITGB2	PROC_ITGB2
7269	RUNX1	ICAM5	ITGB2	ICAM5_ITGB2
7270	RUNX1	FGB	ITGB2	FGB_ITGB2
7271	RUNX1	KNG1	ITGB2	KNG1_ITGB2
7272	RUNX1	CYR61	ITGB2	CYR61_ITGB2
7273	RUNX1	LPA	ITGB2	LPA_ITGB2
7274	RUNX1	CTGF	ITGB2	CTGF_ITGB2
7275	RUNX1	HP	ITGB2	HP_ITGB2
7276	RUNX1	PLAT	ITGB2	PLAT_ITGB2
7277	RUNX1	MMP9	ITGB2	MMP9_ITGB2
7278	RUNX1	SELPLG	ITGB2	SELPLG_ITGB2
7279	RUNX1	C3	ITGB2	C3_ITGB2
7280	RUNX1	ICAM1	ITGB2	ICAM1_ITGB2
7281	RUNX1	ICAM4	ITGB2	ICAM4_ITGB2
7282	RUNX1	F10	ITGB2	F10_ITGB2
7283	RUNX1	FGG	ITGB2	FGG_ITGB2
7284	RUNX1	APOB	ITGB2	APOB_ITGB2
7285	RUNX1	CD40LG	ITGB2	CD40LG_ITGB2
7286	RUNX1	VCAM1	ITGB2	VCAM1_ITGB2
7287	RUNX1	PLG	ITGB2	PLG_ITGB2
7288	SP1	ICAM3	ITGB2	ICAM3_ITGB2
7289	SP1	FGA	ITGB2	FGA_ITGB2
7290	SP1	SPON2	ITGB2	SPON2_ITGB2
7291	SP1	ICAM2	ITGB2	ICAM2_ITGB2
7292	SP1	PROC	ITGB2	PROC_ITGB2
7293	SP1	ICAM5	ITGB2	ICAM5_ITGB2
7294	SP1	FGB	ITGB2	FGB_ITGB2
7295	SP1	KNG1	ITGB2	KNG1_ITGB2
7296	SP1	CYR61	ITGB2	CYR61_ITGB2
7297	SP1	LPA	ITGB2	LPA_ITGB2
7298	SP1	HP	ITGB2	HP_ITGB2
7299	SP1	SELPLG	ITGB2	SELPLG_ITGB2
7300	SP1	C3	ITGB2	C3_ITGB2
7301	SP1	ICAM4	ITGB2	ICAM4_ITGB2
7302	SP1	FGG	ITGB2	FGG_ITGB2
7303	SP1	APOB	ITGB2	APOB_ITGB2
7304	SP1	CD40LG	ITGB2	CD40LG_ITGB2
7305	SP1	VCAM1	ITGB2	VCAM1_ITGB2
7306	SP1	PLG	ITGB2	PLG_ITGB2
7307	JUND	FN1	ITGB3	FN1_ITGB3

7308	JUND	TGM2	ITGB3	TGM2_ITGB3
7309	JUND	THBS1	ITGB3	THBS1_ITGB3
7310	SP1	FN1	ITGB3	FN1_ITGB3
7311	SP1	TGM2	ITGB3	TGM2_ITGB3
7312	SP1	THBS1	ITGB3	THBS1_ITGB3
7313	FOSL1	FN1	ITGB3	FN1_ITGB3
7314	FOSL1	TGM2	ITGB3	TGM2_ITGB3
7315	FOSL1	THBS1	ITGB3	THBS1_ITGB3
7316	HOXA10	FN1	ITGB3	FN1_ITGB3
7317	HOXA10	TGM2	ITGB3	TGM2_ITGB3
7318	HOXA10	THBS1	ITGB3	THBS1_ITGB3
7319	ETS1	FN1	ITGB3	FN1_ITGB3
7320	ETS1	TGM2	ITGB3	TGM2_ITGB3
7321	ETS1	THBS1	ITGB3	THBS1_ITGB3
7322	HOXD3	FN1	ITGB3	FN1_ITGB3
7323	HOXD3	TGM2	ITGB3	TGM2_ITGB3
7324	HOXD3	THBS1	ITGB3	THBS1_ITGB3
7325	MAF	FN1	ITGB7	FN1_ITGB7
7326	MAF	VCAM1	ITGB7	VCAM1_ITGB7
7327	JUN	FN1	ITGB8	FN1_ITGB8
7328	JUN	VTN	ITGB8	VTN_ITGB8
7329	JUN	TGFB1	ITGB8	TGFB1_ITGB8
7330	JUN	LAMA1	ITGB8	LAMA1_ITGB8
7331	JUN	COL4A1	ITGB8	COL4A1_ITGB8
7332	ATF2	VTN	ITGB8	VTN_ITGB8
7333	ATF2	TGFB1	ITGB8	TGFB1_ITGB8
7334	ATF2	LAMA1	ITGB8	LAMA1_ITGB8
7335	ATF2	COL4A1	ITGB8	COL4A1_ITGB8
7336	REST	CALM1	KCNN4	CALM1_KCNN4
7337	REST	CALM3	KCNQ3	CALM3_KCNQ3
7338	REST	CALM2	KCNQ3	CALM2_KCNQ3
7339	REST	CALM1	KCNQ3	CALM1_KCNQ3
7340	SP1	CALM3	KCNQ3	CALM3_KCNQ3
7341	SP1	CALM2	KCNQ3	CALM2_KCNQ3
7342	SP1	CALM1	KCNQ3	CALM1_KCNQ3
7343	SP4	VEGFA	KDR	VEGFA_KDR
7344	NANOG	VEGFA	KDR	VEGFA_KDR
7345	HEY1	VEGFA	KDR	VEGFA_KDR
7346	DNMT1	HLA-A	KIR2DL3	HLA-A_KIR2DL3
7347	DNMT1	B2M	KIR2DL3	B2M_KIR2DL3
7348	DNMT1	HLA-C	KIR2DL3	HLA-C_KIR2DL3
7349	DNMT1	HLA-B	KIR2DL3	HLA-B_KIR2DL3
7350	E2F1	HLA-C	KIR3DL1	HLA-C_KIR3DL1
7351	E2F1	B2M	KIR3DL1	B2M_KIR3DL1
7352	E2F1	HLA-A	KIR3DL1	HLA-A_KIR3DL1
7353	E2F1	HLA-B	KIR3DL1	HLA-B_KIR3DL1
7354	E2F1	HLA-E	KIR3DL1	HLA-E_KIR3DL1
7355	YY1	HLA-C	KIR3DL1	HLA-C_KIR3DL1
7356	YY1	B2M	KIR3DL1	B2M_KIR3DL1
7357	YY1	HLA-A	KIR3DL1	HLA-A_KIR3DL1
7358	YY1	HLA-B	KIR3DL1	HLA-B_KIR3DL1
7359	YY1	HLA-E	KIR3DL1	HLA-E_KIR3DL1
7360	TFAP2A	KITLG	KIT	KITLG_KIT
7361	TFAP2A	CLEC11A	KIT	CLEC11A_KIT
7362	MITF	KITLG	KIT	KITLG_KIT
7363	MITF	CLEC11A	KIT	CLEC11A_KIT
7364	SP1	KITLG	KIT	KITLG_KIT
7365	SP1	CLEC11A	KIT	CLEC11A_KIT

7366	RBMX	KITLG	KIT	KITLG_KIT
7367	RBMX	CLEC11A	KIT	CLEC11A_KIT
7368	EGR1	APOE	LDLR	APOE_LDLR
7369	YY1	APOE	LDLR	APOE_LDLR
7370	SREBF2	APOE	LDLR	APOE_LDLR
7371	SREBF1	APOE	LDLR	APOE_LDLR
7372	ATF3	APOE	LDLR	APOE_LDLR
7373	HNF4A	APOE	LDLR	APOE_LDLR
7374	KLF13	APOE	LDLR	APOE_LDLR
7375	NR2C2	GNAI2	LHCGR	GNAI2_LHCGR
7376	NR2C2	GNAS	LHCGR	GNAS_LHCGR
7377	NR2F6	GNAI2	LHCGR	GNAI2_LHCGR
7378	NR2F6	GNAS	LHCGR	GNAS_LHCGR
7379	NR2F1	GNAI2	LHCGR	GNAI2_LHCGR
7380	NR2F1	GNAS	LHCGR	GNAS_LHCGR
7381	TRERF1	HLA-G	LILRB1	HLA-G_LILRB1
7382	TRERF1	B2M	LILRB1	B2M_LILRB1
7383	TRERF1	HLA-A	LILRB1	HLA-A_LILRB1
7384	TRERF1	HLA-C	LILRB1	HLA-C_LILRB1
7385	TRERF1	HLA-B	LILRB1	HLA-B_LILRB1
7386	TRERF1	HLA-C	LILRB2	HLA-C_LILRB2
7387	TRERF1	B2M	LILRB2	B2M_LILRB2
7388	TRERF1	HLA-B	LILRB2	HLA-B_LILRB2
7389	TRERF1	HLA-G	LILRB2	HLA-G_LILRB2
7390	TRERF1	HLA-A	LILRB2	HLA-A_LILRB2
7391	SREBF2	PLAT	LRP1	PLAT_LRP1
7392	SREBF2	CTGF	LRP1	CTGF_LRP1
7393	SREBF2	MDK	LRP1	MDK_LRP1
7394	SREBF2	C3	LRP1	C3_LRP1
7395	SREBF2	APP	LRP1	APP_LRP1
7396	SREBF2	LTF	LRP1	LTF_LRP1
7397	SREBF2	SERPINE2	LRP1	SERPINE2_LRP1
7398	SREBF2	MMP9	LRP1	MMP9_LRP1
7399	SREBF2	C4BPA	LRP1	C4BPA_LRP1
7400	SREBF2	SERPING1	LRP1	SERPING1_LRP1
7401	SREBF2	SERPINA1	LRP1	SERPINA1_LRP1
7402	SREBF2	APOE	LRP1	APOE_LRP1
7403	SREBF2	A2M	LRP1	A2M_LRP1
7404	SREBF2	F8	LRP1	F8_LRP1
7405	SREBF2	TFPI	LRP1	TFPI_LRP1
7406	SREBF2	SERPINC1	LRP1	SERPINC1_LRP1
7407	SREBF2	C1QB	LRP1	C1QB_LRP1
7408	SREBF2	HSP90B1	LRP1	HSP90B1_LRP1
7409	SREBF2	WNT3A	LRP1	WNT3A_LRP1
7410	SREBF2	THBS1	LRP1	THBS1_LRP1
7411	SREBF2	MMP13	LRP1	MMP13_LRP1
7412	SREBF2	LRPAP1	LRP1	LRPAP1_LRP1
7413	SREBF2	LPL	LRP1	LPL_LRP1
7414	SREBF2	F9	LRP1	F9_LRP1
7415	SREBF2	CALR	LRP1	CALR_LRP1
7416	SREBF2	FCN2	LRP1	FCN2_LRP1
7417	SREBF2	SERPINE1	LRP1	SERPINE1_LRP1
7418	SREBF2	PDGFB	LRP1	PDGFB_LRP1
7419	SREBF2	PLAU	LRP1	PLAU_LRP1
7420	SREBF2	PSAP	LRP1	PSAP_LRP1
7421	SREBF1	PLAT	LRP1	PLAT_LRP1
7422	SREBF1	CTGF	LRP1	CTGF_LRP1
7423	SREBF1	MDK	LRP1	MDK_LRP1

7424	SREBF1	C3	LRP1	C3_LRP1
7425	SREBF1	APP	LRP1	APP_LRP1
7426	SREBF1	LTF	LRP1	LTF_LRP1
7427	SREBF1	SERPINE2	LRP1	SERPINE2_LRP1
7428	SREBF1	MMP9	LRP1	MMP9_LRP1
7429	SREBF1	LIPC	LRP1	LIPC_LRP1
7430	SREBF1	C4BPA	LRP1	C4BPA_LRP1
7431	SREBF1	SERPING1	LRP1	SERPING1_LRP1
7432	SREBF1	SERPINA1	LRP1	SERPINA1_LRP1
7433	SREBF1	APOE	LRP1	APOE_LRP1
7434	SREBF1	A2M	LRP1	A2M_LRP1
7435	SREBF1	F8	LRP1	F8_LRP1
7436	SREBF1	TFPI	LRP1	TFPI_LRP1
7437	SREBF1	SERPINC1	LRP1	SERPINC1_LRP1
7438	SREBF1	C1QB	LRP1	C1QB_LRP1
7439	SREBF1	HSP90B1	LRP1	HSP90B1_LRP1
7440	SREBF1	WNT3A	LRP1	WNT3A_LRP1
7441	SREBF1	THBS1	LRP1	THBS1_LRP1
7442	SREBF1	MMP13	LRP1	MMP13_LRP1
7443	SREBF1	LRPAP1	LRP1	LRPAP1_LRP1
7444	SREBF1	LPL	LRP1	LPL_LRP1
7445	SREBF1	F9	LRP1	F9_LRP1
7446	SREBF1	CALR	LRP1	CALR_LRP1
7447	SREBF1	FCN2	LRP1	FCN2_LRP1
7448	SREBF1	SERPINE1	LRP1	SERPINE1_LRP1
7449	SREBF1	PDGFB	LRP1	PDGFB_LRP1
7450	SREBF1	PLAU	LRP1	PLAU_LRP1
7451	SREBF1	PSAP	LRP1	PSAP_LRP1
7452	KLF15	THBS1	LRP5	THBS1_LRP5
7453	KLF15	APOE	LRP5	APOE_LRP5
7454	SP1	THBS1	LRP5	THBS1_LRP5
7455	RUNX2	THBS1	LRP5	THBS1_LRP5
7456	RUNX2	APOE	LRP5	APOE_LRP5
7457	HIC1	APOE	LRP8	APOE_LRP8
7458	WWTR1	LTB	LTBR	LTB_LTBR
7459	WWTR1	LTA	LTBR	LTA_LTBR
7460	WWTR1	TNF	LTBR	TNF_LTBR
7461	WWTR1	TNFSF14	LTBR	TNFSF14_LTBR
7462	CREB5	SFTPD	LY96	SFTPD_LY96
7463	CREB5	BGN	LY96	BGN_LY96
7464	STAT1	SFTPD	LY96	SFTPD_LY96
7465	STAT1	BGN	LY96	BGN_LY96
7466	PAX5	DCN	MET	DCN_MET
7467	PAX5	HGF	MET	HGF_MET
7468	PAX5	SEMA5A	MET	SEMA5A_MET
7469	PAX5	SEMA4D	MET	SEMA4D_MET
7470	SP1	DCN	MET	DCN_MET
7471	SP1	SEMA5A	MET	SEMA5A_MET
7472	SP1	SEMA4D	MET	SEMA4D_MET
7473	PAX3	DCN	MET	DCN_MET
7474	PAX3	HGF	MET	HGF_MET
7475	PAX3	SEMA5A	MET	SEMA5A_MET
7476	PAX3	SEMA4D	MET	SEMA4D_MET
7477	YBX1	DCN	MET	DCN_MET
7478	YBX1	HGF	MET	HGF_MET
7479	YBX1	SEMA5A	MET	SEMA5A_MET
7480	YBX1	SEMA4D	MET	SEMA4D_MET
7481	MITF	DCN	MET	DCN_MET

7482	MITF	HGF	MET	HGF_MET
7483	MITF	SEMA5A	MET	SEMA5A_MET
7484	MITF	SEMA4D	MET	SEMA4D_MET
7485	TP53	DCN	MET	DCN_MET
7486	TP53	HGF	MET	HGF_MET
7487	TP53	SEMA5A	MET	SEMA5A_MET
7488	TP53	SEMA4D	MET	SEMA4D_MET
7489	SMAD7	DCN	MET	DCN_MET
7490	SMAD7	HGF	MET	HGF_MET
7491	SMAD7	SEMA5A	MET	SEMA5A_MET
7492	SMAD7	SEMA4D	MET	SEMA4D_MET
7493	PAX6	DCN	MET	DCN_MET
7494	PAX6	HGF	MET	HGF_MET
7495	PAX6	SEMA5A	MET	SEMA5A_MET
7496	PAX6	SEMA4D	MET	SEMA4D_MET
7497	FOXP2	DCN	MET	DCN_MET
7498	FOXP2	HGF	MET	HGF_MET
7499	FOXP2	SEMA5A	MET	SEMA5A_MET
7500	FOXP2	SEMA4D	MET	SEMA4D_MET
7501	HIF1A	DCN	MET	DCN_MET
7502	HIF1A	HGF	MET	HGF_MET
7503	HIF1A	SEMA5A	MET	SEMA5A_MET
7504	HIF1A	SEMA4D	MET	SEMA4D_MET
7505	PITX3	CALM1	MIP	CALM1_MIP
7506	MYCN	APP	NGFR	APP_NGFR
7507	MYCN	RTN4	NGFR	RTN4_NGFR
7508	KLF5	UBA52	NOTCH1	UBA52_NOTCH1
7509	SIRT1	UBA52	NOTCH1	UBA52_NOTCH1
7510	PPARG	HLA-C	NOTCH4	HLA-C_NOTCH4
7511	REST	VEGFA	NRP1	VEGFA_NRP1
7512	TCF4	SYTL3	NRXN1	SYTL3_NRXN1
7513	HNF1A	LAMA1	NT5E	LAMA1_NT5E
7514	HNF1A	FN1	NT5E	FN1_NT5E
7515	HNF1A	TNC	NT5E	TNC_NT5E
7516	LEF1	LAMA1	NT5E	LAMA1_NT5E
7517	LEF1	FN1	NT5E	FN1_NT5E
7518	LEF1	TNC	NT5E	TNC_NT5E
7519	HIF1A	LAMA1	NT5E	LAMA1_NT5E
7520	HIF1A	FN1	NT5E	FN1_NT5E
7521	HIF1A	TNC	NT5E	TNC_NT5E
7522	SP1	FN1	NT5E	FN1_NT5E
7523	HR	CRP	OLR1	CRP_OLR1
7524	HR	APOB	OLR1	APOB_OLR1
7525	NFKB1	APOB	OLR1	APOB_OLR1
7526	RELA	APOB	OLR1	APOB_OLR1
7527	JUN	CALM1	OPRM1	CALM1_OPRM1
7528	JUN	GNAI2	OPRM1	GNAI2_OPRM1
7529	SP3	CALM1	OPRM1	CALM1_OPRM1
7530	SP3	GNAI2	OPRM1	GNAI2_OPRM1
7531	STAT6	CALM1	OPRM1	CALM1_OPRM1
7532	STAT6	GNAI2	OPRM1	GNAI2_OPRM1
7533	NFKB1	CALM1	OPRM1	CALM1_OPRM1
7534	NFKB1	GNAI2	OPRM1	GNAI2_OPRM1
7535	REST	CALM1	OPRM1	CALM1_OPRM1
7536	REST	GNAI2	OPRM1	GNAI2_OPRM1
7537	STAT3	CALM1	OPRM1	CALM1_OPRM1
7538	STAT3	GNAI2	OPRM1	GNAI2_OPRM1
7539	YY1	CALM1	OPRM1	CALM1_OPRM1

7540	YY1	GNAI2	OPRM1	GNAI2_OPRM1
7541	STAT1	CALM1	OPRM1	CALM1_OPRM1
7542	STAT1	GNAI2	OPRM1	GNAI2_OPRM1
7543	SP1	CALM1	OPRM1	CALM1_OPRM1
7544	SP1	GNAI2	OPRM1	GNAI2_OPRM1
7545	RELA	VTN	PLAUR	VTN_PLAUR
7546	RELA	FGA	PLAUR	FGA_PLAUR
7547	RELA	LRP1B	PLAUR	LRP1B_PLAUR
7548	RELA	KNG1	PLAUR	KNG1_PLAUR
7549	RELA	PLG	PLAUR	PLG_PLAUR
7550	JUN	VTN	PLAUR	VTN_PLAUR
7551	JUN	FGA	PLAUR	FGA_PLAUR
7552	JUN	LRP1B	PLAUR	LRP1B_PLAUR
7553	JUN	FN1	PLAUR	FN1_PLAUR
7554	JUN	KNG1	PLAUR	KNG1_PLAUR
7555	JUN	PLG	PLAUR	PLG_PLAUR
7556	JUN	SERPINE1	PLAUR	SERPINE1_PLAUR
7557	SP3	MMP12	PLAUR	MMP12_PLAUR
7558	SP3	PLAU	PLAUR	PLAU_PLAUR
7559	SP3	VTN	PLAUR	VTN_PLAUR
7560	SP3	FGA	PLAUR	FGA_PLAUR
7561	SP3	LRP1B	PLAUR	LRP1B_PLAUR
7562	SP3	FN1	PLAUR	FN1_PLAUR
7563	SP3	KNG1	PLAUR	KNG1_PLAUR
7564	SP3	PLG	PLAUR	PLG_PLAUR
7565	SP3	SERPINE1	PLAUR	SERPINE1_PLAUR
7566	NFKB1	MMP12	PLAUR	MMP12_PLAUR
7567	NFKB1	VTN	PLAUR	VTN_PLAUR
7568	NFKB1	FGA	PLAUR	FGA_PLAUR
7569	NFKB1	LRP1B	PLAUR	LRP1B_PLAUR
7570	NFKB1	KNG1	PLAUR	KNG1_PLAUR
7571	NFKB1	PLG	PLAUR	PLG_PLAUR
7572	SPDEF	MMP12	PLAUR	MMP12_PLAUR
7573	SPDEF	PLAU	PLAUR	PLAU_PLAUR
7574	SPDEF	VTN	PLAUR	VTN_PLAUR
7575	SPDEF	FGA	PLAUR	FGA_PLAUR
7576	SPDEF	LRP1B	PLAUR	LRP1B_PLAUR
7577	SPDEF	FN1	PLAUR	FN1_PLAUR
7578	SPDEF	KNG1	PLAUR	KNG1_PLAUR
7579	SPDEF	PLG	PLAUR	PLG_PLAUR
7580	SPDEF	SERPINE1	PLAUR	SERPINE1_PLAUR
7581	EGR1	MMP12	PLAUR	MMP12_PLAUR
7582	EGR1	VTN	PLAUR	VTN_PLAUR
7583	EGR1	FGA	PLAUR	FGA_PLAUR
7584	EGR1	LRP1B	PLAUR	LRP1B_PLAUR
7585	EGR1	KNG1	PLAUR	KNG1_PLAUR
7586	EGR1	PLG	PLAUR	PLG_PLAUR
7587	EGR1	SERPINE1	PLAUR	SERPINE1_PLAUR
7588	SP1	MMP12	PLAUR	MMP12_PLAUR
7589	SP1	VTN	PLAUR	VTN_PLAUR
7590	SP1	FGA	PLAUR	FGA_PLAUR
7591	SP1	LRP1B	PLAUR	LRP1B_PLAUR
7592	SP1	FN1	PLAUR	FN1_PLAUR
7593	SP1	KNG1	PLAUR	KNG1_PLAUR
7594	SP1	PLG	PLAUR	PLG_PLAUR
7595	FOXP2	MMP12	PLAUR	MMP12_PLAUR
7596	FOXP2	PLAU	PLAUR	PLAU_PLAUR
7597	FOXP2	VTN	PLAUR	VTN_PLAUR

7598	FOXP2	FGA	PLAUR	FGA_PLAUR
7599	FOXP2	LRP1B	PLAUR	LRP1B_PLAUR
7600	FOXP2	FN1	PLAUR	FN1_PLAUR
7601	FOXP2	KNG1	PLAUR	KNG1_PLAUR
7602	FOXP2	PLG	PLAUR	PLG_PLAUR
7603	FOXP2	SERPINE1	PLAUR	SERPINE1_PLAUR
7604	ATF1	MMP12	PLAUR	MMP12_PLAUR
7605	ATF1	PLAU	PLAUR	PLAU_PLAUR
7606	ATF1	VTN	PLAUR	VTN_PLAUR
7607	ATF1	FGA	PLAUR	FGA_PLAUR
7608	ATF1	LRP1B	PLAUR	LRP1B_PLAUR
7609	ATF1	FN1	PLAUR	FN1_PLAUR
7610	ATF1	KNG1	PLAUR	KNG1_PLAUR
7611	ATF1	PLG	PLAUR	PLG_PLAUR
7612	ATF1	SERPINE1	PLAUR	SERPINE1_PLAUR
7613	FOS	MMP12	PLAUR	MMP12_PLAUR
7614	FOS	VTN	PLAUR	VTN_PLAUR
7615	FOS	FGA	PLAUR	FGA_PLAUR
7616	FOS	LRP1B	PLAUR	LRP1B_PLAUR
7617	FOS	FN1	PLAUR	FN1_PLAUR
7618	FOS	KNG1	PLAUR	KNG1_PLAUR
7619	FOS	PLG	PLAUR	PLG_PLAUR
7620	FOS	SERPINE1	PLAUR	SERPINE1_PLAUR
7621	FOSL2	MMP12	PLAUR	MMP12_PLAUR
7622	FOSL2	PLAU	PLAUR	PLAU_PLAUR
7623	FOSL2	VTN	PLAUR	VTN_PLAUR
7624	FOSL2	FGA	PLAUR	FGA_PLAUR
7625	FOSL2	LRP1B	PLAUR	LRP1B_PLAUR
7626	FOSL2	FN1	PLAUR	FN1_PLAUR
7627	FOSL2	KNG1	PLAUR	KNG1_PLAUR
7628	FOSL2	PLG	PLAUR	PLG_PLAUR
7629	FOSL2	SERPINE1	PLAUR	SERPINE1_PLAUR
7630	TFAP2A	MMP12	PLAUR	MMP12_PLAUR
7631	TFAP2A	PLAU	PLAUR	PLAU_PLAUR
7632	TFAP2A	VTN	PLAUR	VTN_PLAUR
7633	TFAP2A	FGA	PLAUR	FGA_PLAUR
7634	TFAP2A	LRP1B	PLAUR	LRP1B_PLAUR
7635	TFAP2A	FN1	PLAUR	FN1_PLAUR
7636	TFAP2A	KNG1	PLAUR	KNG1_PLAUR
7637	TFAP2A	PLG	PLAUR	PLG_PLAUR
7638	TFAP2A	SERPINE1	PLAUR	SERPINE1_PLAUR
7639	ETV4	MMP12	PLAUR	MMP12_PLAUR
7640	ETV4	PLAU	PLAUR	PLAU_PLAUR
7641	ETV4	VTN	PLAUR	VTN_PLAUR
7642	ETV4	FGA	PLAUR	FGA_PLAUR
7643	ETV4	LRP1B	PLAUR	LRP1B_PLAUR
7644	ETV4	FN1	PLAUR	FN1_PLAUR
7645	ETV4	KNG1	PLAUR	KNG1_PLAUR
7646	ETV4	PLG	PLAUR	PLG_PLAUR
7647	ETV4	SERPINE1	PLAUR	SERPINE1_PLAUR
7648	FOSL1	MMP12	PLAUR	MMP12_PLAUR
7649	FOSL1	PLAU	PLAUR	PLAU_PLAUR
7650	FOSL1	VTN	PLAUR	VTN_PLAUR
7651	FOSL1	FGA	PLAUR	FGA_PLAUR
7652	FOSL1	LRP1B	PLAUR	LRP1B_PLAUR
7653	FOSL1	FN1	PLAUR	FN1_PLAUR
7654	FOSL1	KNG1	PLAUR	KNG1_PLAUR
7655	FOSL1	PLG	PLAUR	PLG_PLAUR

7656	FOSL1	SERPINE1	PLAUR	SERPINE1_PLAUR
7657	JUND	MMP12	PLAUR	MMP12_PLAUR
7658	JUND	PLAU	PLAUR	PLAU_PLAUR
7659	JUND	VTN	PLAUR	VTN_PLAUR
7660	JUND	FGA	PLAUR	FGA_PLAUR
7661	JUND	LRP1B	PLAUR	LRP1B_PLAUR
7662	JUND	FN1	PLAUR	FN1_PLAUR
7663	JUND	KNG1	PLAUR	KNG1_PLAUR
7664	JUND	PLG	PLAUR	PLG_PLAUR
7665	JUND	SERPINE1	PLAUR	SERPINE1_PLAUR
7666	SPI1	GNAS	PTGIR	GNAS_PTGIR
7667	POU2F1	GNAS	PTGIR	GNAS_PTGIR
7668	SP1	GNAS	PTGIR	GNAS_PTGIR
7669	TFAP2C	VEGFA	RET	VEGFA_RET
7670	FOXA1	VEGFA	RET	VEGFA_RET
7671	SOX10	VEGFA	RET	VEGFA_RET
7672	NKX2-1	VEGFA	RET	VEGFA_RET
7673	STAT3	GNAI2	S1PR1	GNAI2_S1PR1
7674	STAT3	SPP1	S1PR1	SPP1_S1PR1
7675	STAT2	SAA1	SCARB1	SAA1_SCARB1
7676	STAT2	APOE	SCARB1	APOE_SCARB1
7677	STAT2	THBS1	SCARB1	THBS1_SCARB1
7678	STAT1	SAA1	SCARB1	SAA1_SCARB1
7679	STAT1	APOE	SCARB1	APOE_SCARB1
7680	STAT1	THBS1	SCARB1	THBS1_SCARB1
7681	NR1H4	SAA1	SCARB1	SAA1_SCARB1
7682	NR1H4	APOE	SCARB1	APOE_SCARB1
7683	NR1H4	THBS1	SCARB1	THBS1_SCARB1
7684	SPI1	SAA1	SCARB1	SAA1_SCARB1
7685	SPI1	APOE	SCARB1	APOE_SCARB1
7686	SPI1	THBS1	SCARB1	THBS1_SCARB1
7687	ZNF444	CALR	SCARF1	CALR_SCARF1
7688	SP3	CALM1	SCTR	CALM1_SCTR
7689	SP1	CALM1	SCTR	CALM1_SCTR
7690	RELA	HSPG2	SDC1	HSPG2_SDC1
7691	RELA	THBS1	SDC1	THBS1_SDC1
7692	RELA	PTN	SDC1	PTN_SDC1
7693	RELA	HMGB1	SDC1	HMGB1_SDC1
7694	RELA	IL8	SDC1	IL8_SDC1
7695	RELA	LAMA5	SDC1	LAMA5_SDC1
7696	RELA	MDK	SDC1	MDK_SDC1
7697	RELA	SLIT2	SDC1	SLIT2_SDC1
7698	RELA	SLIT1	SDC1	SLIT1_SDC1
7699	RELA	HGF	SDC1	HGF_SDC1
7700	RELA	FGF2	SDC1	FGF2_SDC1
7701	RELA	LACRT	SDC1	LACRT_SDC1
7702	NFKB1	HSPG2	SDC1	HSPG2_SDC1
7703	NFKB1	THBS1	SDC1	THBS1_SDC1
7704	NFKB1	PTN	SDC1	PTN_SDC1
7705	NFKB1	HMGB1	SDC1	HMGB1_SDC1
7706	NFKB1	IL8	SDC1	IL8_SDC1
7707	NFKB1	LAMA5	SDC1	LAMA5_SDC1
7708	NFKB1	MDK	SDC1	MDK_SDC1
7709	NFKB1	SLIT2	SDC1	SLIT2_SDC1
7710	NFKB1	SLIT1	SDC1	SLIT1_SDC1
7711	NFKB1	HGF	SDC1	HGF_SDC1
7712	NFKB1	FGF2	SDC1	FGF2_SDC1
7713	NFKB1	LACRT	SDC1	LACRT_SDC1

7714	POU2F1	ADAM12	SDC4	ADAM12_SDC4
7715	POU2F1	MDK	SDC4	MDK_SDC4
7716	POU2F1	FGF2	SDC4	FGF2_SDC4
7717	POU2F1	LAMA1	SDC4	LAMA1_SDC4
7718	POU2F1	TFPI	SDC4	TFPI_SDC4
7719	POU2F1	TNC	SDC4	TNC_SDC4
7720	POU2F1	TGM2	SDC4	TGM2_SDC4
7721	POU2F1	CXCL10	SDC4	CXCL10_SDC4
7722	POU2F1	THBS1	SDC4	THBS1_SDC4
7723	POU2F1	CXCL12	SDC4	CXCL12_SDC4
7724	POU2F1	RSPO3	SDC4	RSPO3_SDC4
7725	POU2F1	FGF6	SDC4	FGF6_SDC4
7726	POU2F1	CCL5	SDC4	CCL5_SDC4
7727	NFKB1	SERPING1	SELE	SERPING1_SELE
7728	NFKB1	SELPLG	SELE	SELPLG_SELE
7729	REL	SERPING1	SELE	SERPING1_SELE
7730	REL	SELPLG	SELE	SELPLG_SELE
7731	RELA	SERPING1	SELE	SERPING1_SELE
7732	RELA	SELPLG	SELE	SELPLG_SELE
7733	HOXA9	SERPING1	SELE	SERPING1_SELE
7734	HOXA9	SELPLG	SELE	SELPLG_SELE
7735	SIRT1	SERPING1	SELE	SERPING1_SELE
7736	SIRT1	SELPLG	SELE	SELPLG_SELE
7737	ATF3	SERPING1	SELE	SERPING1_SELE
7738	ATF3	SELPLG	SELE	SELPLG_SELE
7739	STAT6	SERPING1	SELE	SERPING1_SELE
7740	STAT6	SELPLG	SELE	SELPLG_SELE
7741	KLF2	PODXL2	SELL	PODXL2_SELL
7742	KLF2	VCAN	SELL	VCAN_SELL
7743	KLF2	CALM3	SELL	CALM3_SELL
7744	KLF2	PODXL	SELL	PODXL_SELL
7745	KLF2	MUC7	SELL	MUC7_SELL
7746	KLF2	CFH	SELL	CFH_SELL
7747	KLF2	CD34	SELL	CD34_SELL
7748	KLF2	CALM2	SELL	CALM2_SELL
7749	KLF2	SELPLG	SELL	SELPLG_SELL
7750	KLF2	CALM1	SELL	CALM1_SELL
7751	STAT6	SELPLG	SELP	SELPLG_SELP
7752	STAT6	VCAN	SELP	VCAN_SELP
7753	STAT6	CD24	SELP	CD24_SELP
7754	STAT6	SERPING1	SELP	SERPING1_SELP
7755	NFKB2	SELPLG	SELP	SELPLG_SELP
7756	NFKB2	VCAN	SELP	VCAN_SELP
7757	NFKB2	CD24	SELP	CD24_SELP
7758	NFKB2	SERPING1	SELP	SERPING1_SELP
7759	ETS1	TGM2	TBXA2R	TGM2_TBXA2R
7760	ETS1	GNAI2	TBXA2R	GNAI2_TBXA2R
7761	EGR1	TGM2	TBXA2R	TGM2_TBXA2R
7762	EGR1	GNAI2	TBXA2R	GNAI2_TBXA2R
7763	SP1	TGM2	TBXA2R	TGM2_TBXA2R
7764	SP1	GNAI2	TBXA2R	GNAI2_TBXA2R
7765	GATA1	TGM2	TBXA2R	TGM2_TBXA2R
7766	GATA1	GNAI2	TBXA2R	GNAI2_TBXA2R
7767	NFE2	TGM2	TBXA2R	TGM2_TBXA2R
7768	NFE2	GNAI2	TBXA2R	GNAI2_TBXA2R
7769	WT1	TGM2	TBXA2R	TGM2_TBXA2R
7770	WT1	GNAI2	TBXA2R	GNAI2_TBXA2R
7771	VHL	B2M	TFRC	B2M_TFRC

7772	MYC	B2M	TFRC	B2M_TFRC
7773	YY1	B2M	TFRC	B2M_TFRC
7774	HIF1A	B2M	TFRC	B2M_TFRC
7775	WWP1	GNB3	TGFBR1	GNB3_TGFBR1
7776	WWP1	TGFB2	TGFBR1	TGFB2_TGFBR1
7777	WWP1	TGFB1	TGFBR1	TGFB1_TGFBR1
7778	WWP1	UBA52	TGFBR1	UBA52_TGFBR1
7779	WWP1	TGFB3	TGFBR1	TGFB3_TGFBR1
7780	WWP1	CGN	TGFBR1	CGN_TGFBR1
7781	WWP1	GDF9	TGFBR1	GDF9_TGFBR1
7782	SMAD7	GNB3	TGFBR1	GNB3_TGFBR1
7783	SMAD7	TGFB2	TGFBR1	TGFB2_TGFBR1
7784	SMAD7	UBA52	TGFBR1	UBA52_TGFBR1
7785	SMAD7	TGFB3	TGFBR1	TGFB3_TGFBR1
7786	SMAD7	CGN	TGFBR1	CGN_TGFBR1
7787	SMAD7	GDF9	TGFBR1	GDF9_TGFBR1
7788	EWSR1	UBA52	TGFBR2	UBA52_TGFBR2
7789	FLI1	UBA52	TGFBR2	UBA52_TGFBR2
7790	HDAC2	UBA52	TGFBR2	UBA52_TGFBR2
7791	KLF14	UBA52	TGFBR2	UBA52_TGFBR2
7792	ETS1	UBA52	TGFBR2	UBA52_TGFBR2
7793	SP1	UBA52	TGFBR2	UBA52_TGFBR2
7794	EGR1	UBA52	TGFBR2	UBA52_TGFBR2
7795	ETV4	UBA52	TGFBR2	UBA52_TGFBR2
7796	RELA	F2	THBD	F2_THBD
7797	RELA	PF4	THBD	PF4_THBD
7798	RELA	HMGB1	THBD	HMGB1_THBD
7799	RELA	PROC	THBD	PROC_THBD
7800	NFKB1	F2	THBD	F2_THBD
7801	NFKB1	PF4	THBD	PF4_THBD
7802	NFKB1	HMGB1	THBD	HMGB1_THBD
7803	NFKB1	PROC	THBD	PROC_THBD
7804	PARP1	F2	THBD	F2_THBD
7805	PARP1	PF4	THBD	PF4_THBD
7806	PARP1	HMGB1	THBD	HMGB1_THBD
7807	PARP1	PROC	THBD	PROC_THBD
7808	RARB	F2	THBD	F2_THBD
7809	RARB	PF4	THBD	PF4_THBD
7810	RARB	HMGB1	THBD	HMGB1_THBD
7811	RARB	PROC	THBD	PROC_THBD
7812	RARA	F2	THBD	F2_THBD
7813	RARA	PF4	THBD	PF4_THBD
7814	RARA	HMGB1	THBD	HMGB1_THBD
7815	RARA	PROC	THBD	PROC_THBD
7816	RXRA	F2	THBD	F2_THBD
7817	RXRA	PF4	THBD	PF4_THBD
7818	RXRA	HMGB1	THBD	HMGB1_THBD
7819	RXRA	PROC	THBD	PROC_THBD
7820	RARG	F2	THBD	F2_THBD
7821	RARG	PF4	THBD	PF4_THBD
7822	RARG	HMGB1	THBD	HMGB1_THBD
7823	RARG	PROC	THBD	PROC_THBD
7824	EP300	F2	THBD	F2_THBD
7825	EP300	PF4	THBD	PF4_THBD
7826	EP300	HMGB1	THBD	HMGB1_THBD
7827	EP300	PROC	THBD	PROC_THBD
7828	SP1	F2	THBD	F2_THBD
7829	SP1	PF4	THBD	PF4_THBD

7830	SP1	HMGB1	THBD	HMGB1_THBD
7831	SP1	PROC	THBD	PROC_THBD
7832	KLF2	F2	THBD	F2_THBD
7833	KLF2	PF4	THBD	PF4_THBD
7834	KLF2	HMGB1	THBD	HMGB1_THBD
7835	KLF2	PROC	THBD	PROC_THBD
7836	KLF4	F2	THBD	F2_THBD
7837	KLF4	PF4	THBD	PF4_THBD
7838	KLF4	HMGB1	THBD	HMGB1_THBD
7839	KLF4	PROC	THBD	PROC_THBD
7840	SP1	SFTPA1	TLR2	SFTPA1_TLR2
7841	SP1	HSP90B1	TLR2	HSP90B1_TLR2
7842	SP1	ZG16B	TLR2	ZG16B_TLR2
7843	SP1	RNASE2	TLR2	RNASE2_TLR2
7844	SP1	VCAN	TLR2	VCAN_TLR2
7845	SP1	BGN	TLR2	BGN_TLR2
7846	NFKB1	HSP90B1	TLR2	HSP90B1_TLR2
7847	NFKB1	APOC3	TLR2	APOC3_TLR2
7848	NFKB1	ZG16B	TLR2	ZG16B_TLR2
7849	NFKB1	RNASE2	TLR2	RNASE2_TLR2
7850	NFKB1	HRAS	TLR2	HRAS_TLR2
7851	NFKB1	VCAN	TLR2	VCAN_TLR2
7852	HIF1A	SFTPA1	TLR2	SFTPA1_TLR2
7853	HIF1A	HSP90B1	TLR2	HSP90B1_TLR2
7854	HIF1A	APOC3	TLR2	APOC3_TLR2
7855	HIF1A	ZG16B	TLR2	ZG16B_TLR2
7856	HIF1A	RNASE2	TLR2	RNASE2_TLR2
7857	HIF1A	HRAS	TLR2	HRAS_TLR2
7858	HIF1A	VCAN	TLR2	VCAN_TLR2
7859	HIF1A	BGN	TLR2	BGN_TLR2
7860	RELA	HSP90B1	TLR2	HSP90B1_TLR2
7861	RELA	APOC3	TLR2	APOC3_TLR2
7862	RELA	ZG16B	TLR2	ZG16B_TLR2
7863	RELA	RNASE2	TLR2	RNASE2_TLR2
7864	RELA	HRAS	TLR2	HRAS_TLR2
7865	RELA	VCAN	TLR2	VCAN_TLR2
7866	SPI1	SFTPD	TLR4	SFTPD_TLR4
7867	SPI1	BGN	TLR4	BGN_TLR4
7868	SPI1	ZG16B	TLR4	ZG16B_TLR4
7869	SPI1	HSPA1A	TLR4	HSPA1A_TLR4
7870	SPI1	DEFB4A	TLR4	DEFB4A_TLR4
7871	SPI1	HSP90B1	TLR4	HSP90B1_TLR4
7872	SPI1	S100A9	TLR4	S100A9_TLR4
7873	SPI1	S100A8	TLR4	S100A8_TLR4
7874	STAT6	SFTPD	TLR4	SFTPD_TLR4
7875	STAT6	BGN	TLR4	BGN_TLR4
7876	STAT6	ZG16B	TLR4	ZG16B_TLR4
7877	STAT6	HSPA1A	TLR4	HSPA1A_TLR4
7878	STAT6	DEFB4A	TLR4	DEFB4A_TLR4
7879	STAT6	HSP90B1	TLR4	HSP90B1_TLR4
7880	STAT6	S100A9	TLR4	S100A9_TLR4
7881	STAT6	S100A8	TLR4	S100A8_TLR4
7882	IRF3	SFTPD	TLR4	SFTPD_TLR4
7883	IRF3	BGN	TLR4	BGN_TLR4
7884	IRF3	ZG16B	TLR4	ZG16B_TLR4
7885	IRF3	HSPA1A	TLR4	HSPA1A_TLR4
7886	IRF3	DEFB4A	TLR4	DEFB4A_TLR4
7887	IRF3	HSP90B1	TLR4	HSP90B1_TLR4

7888	IRF3	S100A9	TLR4	S100A9_TLR4
7889	IRF3	S100A8	TLR4	S100A8_TLR4
7890	IRF8	SFTPD	TLR4	SFTPD_TLR4
7891	IRF8	BGN	TLR4	BGN_TLR4
7892	IRF8	ZG16B	TLR4	ZG16B_TLR4
7893	IRF8	HSPA1A	TLR4	HSPA1A_TLR4
7894	IRF8	DEFB4A	TLR4	DEFB4A_TLR4
7895	IRF8	HSP90B1	TLR4	HSP90B1_TLR4
7896	IRF8	S100A9	TLR4	S100A9_TLR4
7897	IRF8	S100A8	TLR4	S100A8_TLR4
7898	ZNF160	SFTPD	TLR4	SFTPD_TLR4
7899	ZNF160	BGN	TLR4	BGN_TLR4
7900	ZNF160	ZG16B	TLR4	ZG16B_TLR4
7901	ZNF160	HSPA1A	TLR4	HSPA1A_TLR4
7902	ZNF160	DEFB4A	TLR4	DEFB4A_TLR4
7903	ZNF160	HSP90B1	TLR4	HSP90B1_TLR4
7904	ZNF160	S100A9	TLR4	S100A9_TLR4
7905	ZNF160	S100A8	TLR4	S100A8_TLR4
7906	RELA	HSP90B1	TLR7	HSP90B1_TLR7
7907	NFKB1	HSP90B1	TLR7	HSP90B1_TLR7
7908	HDAC3	HSP90B1	TLR9	HSP90B1_TLR9
7909	NFKB1	HSP90B1	TLR9	HSP90B1_TLR9
7910	CREB1	HSP90B1	TLR9	HSP90B1_TLR9
7911	ELK1	HSP90B1	TLR9	HSP90B1_TLR9
7912	ELF1	HSP90B1	TLR9	HSP90B1_TLR9
7913	CEBPA	HSP90B1	TLR9	HSP90B1_TLR9
7914	RELA	HSP90B1	TLR9	HSP90B1_TLR9
7915	PTMA	HSP90B1	TLR9	HSP90B1_TLR9
7916	ETS2	HSP90B1	TLR9	HSP90B1_TLR9
7917	HDAC1	TNFSF10	TNFRSF10A	TNFSF10_TNFRSF10A
7918	TP53	TNFSF10	TNFRSF10A	TNFSF10_TNFRSF10A
7919	JUN	TNFSF10	TNFRSF10A	TNFSF10_TNFRSF10A
7920	DDIT3	TNFSF10	TNFRSF10B	TNFSF10_TNFRSF10B
7921	MYC	TNFSF10	TNFRSF10B	TNFSF10_TNFRSF10B
7922	ATF4	TNFSF10	TNFRSF10B	TNFSF10_TNFRSF10B
7923	ELK1	TNFSF10	TNFRSF10B	TNFSF10_TNFRSF10B
7924	HDAC9	TNFSF10	TNFRSF10B	TNFSF10_TNFRSF10B
7925	GLI1	TNFSF10	TNFRSF10B	TNFSF10_TNFRSF10B
7926	TP53	TNFSF10	TNFRSF10B	TNFSF10_TNFRSF10B
7927	STAT3	TNFSF10	TNFRSF10B	TNFSF10_TNFRSF10B
7928	DNMT1	TNFSF10	TNFRSF10B	TNFSF10_TNFRSF10B
7929	GLI2	TNFSF10	TNFRSF10B	TNFSF10_TNFRSF10B
7930	YY1	TNFSF10	TNFRSF10B	TNFSF10_TNFRSF10B
7931	ATM	TNFSF10	TNFRSF10B	TNFSF10_TNFRSF10B
7932	SIRT1	TNFSF10	TNFRSF10B	TNFSF10_TNFRSF10B
7933	SMAD1	VTN	TNFRSF11B	VTN_TNFRSF11B
7934	SMAD1	FN1	TNFRSF11B	FN1_TNFRSF11B
7935	SMAD1	TNFSF13	TNFRSF11B	TNFSF13_TNFRSF11B
7936	SMAD1	TNFSF11	TNFRSF11B	TNFSF11_TNFRSF11B
7937	SMAD1	VWF	TNFRSF11B	VWF_TNFRSF11B
7938	SMAD1	THBS1	TNFRSF11B	THBS1_TNFRSF11B
7939	SMAD1	TNFSF10	TNFRSF11B	TNFSF10_TNFRSF11B
7940	PPARG	VTN	TNFRSF11B	VTN_TNFRSF11B
7941	PPARG	FN1	TNFRSF11B	FN1_TNFRSF11B
7942	PPARG	TNFSF13	TNFRSF11B	TNFSF13_TNFRSF11B
7943	PPARG	TNFSF11	TNFRSF11B	TNFSF11_TNFRSF11B
7944	PPARG	VWF	TNFRSF11B	VWF_TNFRSF11B
7945	PPARG	THBS1	TNFRSF11B	THBS1_TNFRSF11B

7946	PPARG	TNFSF10	TNFRSF11B	TNFSF10_TNFRSF11B
7947	CREB5	VTN	TNFRSF11B	VTN_TNFRSF11B
7948	CREB5	FN1	TNFRSF11B	FN1_TNFRSF11B
7949	CREB5	TNFSF13	TNFRSF11B	TNFSF13_TNFRSF11B
7950	CREB5	TNFSF11	TNFRSF11B	TNFSF11_TNFRSF11B
7951	CREB5	VWF	TNFRSF11B	VWF_TNFRSF11B
7952	CREB5	THBS1	TNFRSF11B	THBS1_TNFRSF11B
7953	CREB5	TNFSF10	TNFRSF11B	TNFSF10_TNFRSF11B
7954	HOXC8	VTN	TNFRSF11B	VTN_TNFRSF11B
7955	HOXC8	FN1	TNFRSF11B	FN1_TNFRSF11B
7956	HOXC8	TNFSF13	TNFRSF11B	TNFSF13_TNFRSF11B
7957	HOXC8	TNFSF11	TNFRSF11B	TNFSF11_TNFRSF11B
7958	HOXC8	VWF	TNFRSF11B	VWF_TNFRSF11B
7959	HOXC8	THBS1	TNFRSF11B	THBS1_TNFRSF11B
7960	HOXC8	TNFSF10	TNFRSF11B	TNFSF10_TNFRSF11B
7961	MAML1	TNFSF12	TNFRSF12A	TNFSF12_TNFRSF12A
7962	NR1I2	TNFSF12	TNFRSF12A	TNFSF12_TNFRSF12A
7963	RELA	TNFSF13B	TNFRSF13C	TNFSF13B_TNFRSF13C
7964	POU2AF1	TNFSF13B	TNFRSF17	TNFSF13B_TNFRSF17
7965	POU2AF1	TNFSF13	TNFRSF17	TNFSF13_TNFRSF17
7966	JUN	OMG	TNFRSF1B	OMG_TNFRSF1B
7967	JUN	LTA	TNFRSF1B	LTA_TNFRSF1B
7968	VDR	APP	TNFRSF21	APP_TNFRSF21
7969	NFKB1	TNFSF9	TNFRSF9	TNFSF9_TNFRSF9
7970	RELA	TNFSF9	TNFRSF9	TNFSF9_TNFRSF9
7971	PAX8	FN1	TSHR	FN1_TSHR
7972	PAX8	GNAI2	TSHR	GNAI2_TSHR
7973	PAX8	CALR	TSHR	CALR_TSHR
7974	PAX8	GNAS	TSHR	GNAS_TSHR
7975	PPARG	FN1	TSHR	FN1_TSHR
7976	PPARG	GNAI2	TSHR	GNAI2_TSHR
7977	PPARG	CALR	TSHR	CALR_TSHR
7978	PPARG	GNAS	TSHR	GNAS_TSHR
7979	CEBPA	TFPI	VLDLR	TFPI_VLDLR
7980	CEBPA	LPL	VLDLR	LPL_VLDLR
7981	CEBPA	APOE	VLDLR	APOE_VLDLR
7982	HIC1	TFPI	VLDLR	TFPI_VLDLR
7983	HIC1	LPL	VLDLR	LPL_VLDLR
7984	HIC1	APOE	VLDLR	APOE_VLDLR
7985	PPARG	TFPI	VLDLR	TFPI_VLDLR
7986	PPARG	LPL	VLDLR	LPL_VLDLR
7987	PPARG	APOE	VLDLR	APOE_VLDLR
