# Supplementary material for: Single-Cell and Transcriptome-Based Immune Cell-Related Prognostic Model in Clear Cell Renal Cell Carcinoma
Source: J Oncol. 2023 Mar 7;2023:5355269. doi: 10.1155/2023/5355269 (PMC10014191; doi:10.1155/2023/5355269)
Supplement: Supplementary Materials — Supplementary Table 1: Notes on cell clustering. Supplementary Table 2: Differential genes in each cell cluster. Supplementary Table 3: Ligand-receptor relationship pair. Supplementary Table 4: Immune cell multifactor network relationship pair. Supplementary Table 5: Intersection genes in immune cell multifactor network relationship pair and TCGA. Supplementary Table 6: Genes in black and magenta models of WGCNA. [file 5355269.f1.zip › Supplementary Table 5.pdf]

Table 5. Intersection genes in immune cell multifactor network relationship pair and TCG[illegible]

1	STAT3
2	TFCP2
3	NFKB1
4	SP1
5	PAX2
6	SKIL
7	CEBPB
8	TFAP2A
9	GTF3A
10	HIF1A
11	RELA
12	SF1
13	GATA4
14	NR5A1
15	WT1
16	ESR1
17	SOX9
18	VDR
19	NR0B1
20	ING4
21	SMAD3
22	PPARD
23	PPARG
24	PPARA
25	NR2F1
26	NR2F6
27	FOXA1
28	HNF4A
29	ATF2
30	HDAC1
31	JUN
32	HDAC3
33	PPARGC1A
34	SMAD4
35	NR0B2
36	FOXM1
37	ATF4
38	ETS2
39	TBP
40	HDAC9
41	CTCF
42	NFIL3
43	STAT1
44	BRCA1
45	ZBTB2
46	USF2
47	USF1
48	CREB1
49	REST
50	CEBPD
51	STAT6
52	YY1
53	RELB
54	NFKB2
55	E2F1
56	RUNX1
57	CREM

58 JUND
59 SPI1
60 NFKBIA
61 KLF13
62 IRF7
63 ATF3
64 IRF1
65 IRF3
66 REL
67 LEF1
68 SP3
69 MEF2D
70 SP2
71 KLF4
72 MYB
73 TAL1
74 MZF1
75 FOS
76 GATA3
77 RFX1
78 PARP1
79 DNMT1
80 YBX1
81 TCF12
82 TWIST1
83 SIX1
84 FOXF1
85 GLI2
86 MTA2
87 FOXQ1
88 SPDEF
89 KLF8
90 SMARCA4
91 FOXA2
92 TWIST2
93 HMGA1
94 ZEB1
95 EZH2
96 HOXA7
97 SNAI2
98 SIRT1
99 SALL4
100 ZEB2
101 KLF6
102 RBBP7
103 LCOR
104 TCF3
105 MTA1
106 HDGF
107 AR
108 ZNF217
109 SNAI1
110 RB1
111 LMO2
112 HMGA2
113 HDAC2
114 CBX7
115 STAT5A

116 STAT5B
117 PTF1A
118 CEBPZ
119 NFIC
120 CIITA
121 MYBL2
122 ETS1
123 MKL1
124 RFX5
125 FLI1
126 EP300
127 KLF11
128 LMX1B
129 HNF1A
130 CEBPA
131 ABL1
132 CREBBP
133 FOXF2
134 GLI1
135 NR1H4
136 NFKBIZ
137 KDM2A
138 GATA1
139 APC
140 NR4A1
141 EGR2
142 PDCD11
143 EGR1
144 MAX
145 MYC
146 FOXO3
147 RFX2
148 RFX3
149 ERG
150 HOXB7
151 PTTG1
152 TP53
153 HDAC5
154 SOX17
155 CDX2
156 MBD2
157 MYOD1
158 KAT5
159 HDAC7
160 HIVEP2
161 MYCN
162 HSF1
163 GLI3
164 NR1I2
165 BTG2
166 MAZ
167 POU4F1
168 POU4F2
169 XBP1P1
170 ETV5
171 RARA
172 IFI16
173 RARG

174 NFATC1
175 RUNX3
176 TBX21
177 STAT4
178 MSC
179 PROX1
180 TFAP4
181 EOMES
182 NFATC2
183 NCOR1
184 NCOR2
185 OTX2
186 MBD1
187 ASCL1
188 PLAGL2
189 KHDRBS1
190 PLAG1
191 DDX5
192 SUZ12
193 RUNX2
194 HDAC11
195 IRF8
196 HDAC4
197 PGR
198 ATF1
199 FOXP3
200 E2F6
201 AHR
202 HOXA10
203 SUGP1
204 JUNB
205 POU2F1
206 ILF3
207 FOXK2
208 TOB1
209 SATB1
210 ILF2
211 BACH2
212 KLF2
213 ZFP36
214 RORC
215 IRF2
216 SPIC
217 MAF
218 FOSL2
219 POU2F2
220 GBX2
221 DDIT3
222 FOXO1
223 RBPJ
224 ZNF300
225 ZMYND11
226 NEUROD1
227 PDX1
228 PAX4
229 GLIS3
230 MAFA
231 MED23

232 SSB
233 NANOG
234 CREB5
235 SREBF2
236 NFAT5
237 CEBPE
238 BTF3
239 PAX5
240 NR4A2
241 ETV3
242 FOSL1
243 ETV4
244 BACH1
245 CITED2
246 RFWD2
247 ZNF410
248 ING2
249 HTATIP2
250 SRF
251 SSX2
252 TFAP2C
253 PAX6
254 CTNNB1
255 NCOA3
256 KLF5
257 ELF4
258 ELF3
259 MAL
260 IKBKB
261 CLOCK
262 PAX8
263 SOX4
264 IRF9
265 ELK1
266 PBX2
267 MEIS1
268 PBX1
269 PKNOX1
270 TIAL1
271 ZBTB7A
272 NF1
273 GATA6
274 HOXD3
275 ONECUT1
276 SETBP1
277 STAT2
278 ENO1
279 DR1
280 CDX1
281 TCF7L2
282 HOXA5
283 MECP2
284 TBR1
285 POU1F1
286 RXRA
287 THRA
288 ESR2
289 ARNTL2

290 EPAS1
291 KLF10
292 TGIF1
293 NKX2.1
294 CEBPG
295 PAX3
296 NR3C1
297 POU5F1
298 FOXD3
299 KCNIP3
300 FOSB
301 SMAD7
302 ASH1L
303 LRRFIP1
304 HMGB2
305 IRF5
306 FOXO4
307 PML
308 DACH1
309 HSF2
310 HOXA9
311 TCF4
312 TEAD4
313 ATM
314 VHL
315 E2F3
316 NKX3.1
317 ZNF24
318 HIPK2
319 HEXIM1
320 UHRF1
321 COPS5
322 MEF2C
323 ARNT
324 ZFP36L1
325 TRAF6
326 ID3
327 HIC1
328 ZNF148
329 TRIM16
330 SOX2
331 NR1H3
332 ZNF202
333 NR1H2
334 SND1
335 TFDP1
336 SREBF1
337 NR3C2
338 TRERF1
339 APEX1
340 EIF2AK2
341 HES1
342 XRCC6
343 XRCC5
344 SPIB
345 SMARCA1
346 SMARCB1
347 CHD4

348 MSX2
349 DNMT3A
350 FOXI1
351 CUX1
352 NFE2L2
353 NRF1
354 ZBTB16
355 CREB3
356 TSG101
357 BCL3
358 AES
359 PURA
360 GABPA
361 SP4
362 ATF7
363 ELF1
364 DENND4A
365 TFAP2B
366 WWP1
367 NFYC
368 NFYB
369 NFYA
370 GATA2
371 SHOX
372 E2F5
373 ECD
374 TBX5
375 HOXD1
376 HEY1
377 MITF
378 RBMX
379 NR2C2
380 KLF15
381 WWTR1
382 FOXP2
383 PITX3
384 HR
385 ZNF444
386 NFE2
387 EWSR1
388 KLF14
389 RARB
390 ZNF160
391 PTMA
392 SMAD1
393 HOXC8
394 MAML1
395 POU2AF1
396 A2M
397 ADAM10
398 ADAM12
399 ADAM17
400 ADM
401 ALOX5AP
402 AMH
403 ANGPT1
404 ANGPTL4
405 APOB

406 APOC3
407 APOE
408 APP
409 AREG
410 ARF1
411 B2M
412 BDNF
413 BGN
414 BST1
415 C3
416 C4A
417 CAMP
418 CCL11
419 CCL13
420 CCL19
421 CCL20
422 CCL21
423 CCL3
424 CCL4
425 CCL5
426 CCL7
427 CD14
428 CD24
429 CD34
430 CD40LG
431 CD70
432 CDH1
433 CEL
434 CFH
435 COL11A1
436 COL18A1
437 COL1A1
438 COL1A2
439 COL2A1
440 COL4A1
441 COL4A3
442 COL4A4
443 COL7A1
444 CRP
445 CSF1
446 CSF2
447 CTGF
448 CXCL10
449 CXCL12
450 CYR61
451 DCN
452 DEFB1
453 DEFB103A
454 DEFB4A
455 EGF
456 EREG
457 F10
458 F13A1
459 F8
460 F9
461 FASLG
462 FBLN1
463 FBN1

464 FGA
465 FGB
466 FGF1
467 FGF2
468 FGG
469 FGL1
470 FN1
471 GNAS
472 GSTP1
473 GZMB
474 HAS2
475 HBEGF
476 HDC
477 HGF
478 HLA.A
479 HLA.B
480 HLA.C
481 HLA.E
482 HLA.G
483 HMGB1
484 HP
485 HRAS
486 HSP90AA1
487 HSPA1A
488 ICAM1
489 ICAM2
490 ICAM3
491 IFNG
492 IGF1
493 IGF2
494 IGFBP4
495 IHH
496 IL10
497 IL12A
498 IL13
499 IL15
500 IL18
501 IL1A
502 IL1B
503 IL1RN
504 IL2
505 IL21
506 IL22
507 IL4
508 IL5
509 IL6
510 IL7
511 INS
512 KISS1
513 L1CAM
514 LAMA1
515 LAMB1
516 LAMC2
517 LGALS3BP
518 LIPC
519 LPL
520 LTA
521 LTB

522 LTF
523 LYZ
524 MADCAM1
525 MATN1
526 MMP1
527 MMP12
528 MMP13
529 MMP2
530 MMP7
531 MMP9
532 MST1
533 NAMPT
534 NCAM1
535 NGF
536 PDGFB
537 PF4
538 PIP
539 PKM
540 PLAT
541 PLAU
542 PLG
543 PLTP
544 PNOC
545 PODXL
546 PROC
547 PROS1
548 PSEN1
549 PTGS2
550 PTN
551 RELN
552 REN
553 RNASE2
554 S100A9
555 SAA1
556 SERPINC1
557 SERPINE1
558 SFTPA1
559 SFTPD
560 SHBG
561 SHH
562 SLIT2
563 SLPI
564 SPP1
565 TCN2
566 TG
567 TGFA
568 TGFB1
569 TGFB2
570 TGFB3
571 THBS1
572 THBS2
573 TIMP1
574 TIMP2
575 TNC
576 TNF
577 TNFSF10
578 TNFSF11
579 TNFSF13B

580 TNFSF14
581 TNFSF4
582 TSLP
583 VCAM1
584 VCAN
585 VEGFA
586 VEGFC
587 VIM
588 VWF
589 WNT3A
590 CALM2
591 CALM1
592 CALM3
593 GNAI2
594 UBA52
595 GAS6
596 CCL28
597 CCL3L3
598 IL16
599 DEFB103B
600 DEFB4B
601 DUSP18
602 LAMB3
603 BTLA
604 C4BPA
605 COL14A1
606 NCAN
607 CD55
608 C1QA
609 IL34
610 CSF3
611 VASP
612 CXCL9
613 CXCL11
614 CXCL13
615 ZG16B
616 SEMA4B
617 BTC
618 ANXA1
619 NRG4
620 SPINK1
621 FGF13
622 ZP3
623 BCAN
624 EPGN
625 EFEMP1
626 EFNA1
627 NUCB2
628 HSP90B1
629 NRG1
630 NRG2
631 TFPI
632 ITIH2
633 HRG
634 CALR
635 IL9
636 AHSG
637 SORBS1

638 GIP
639 TGM2
640 ICAM5
641 LPA
642 SELPLG
643 SPON2
644 KNG1
645 COL4A5
646 VTN
647 COL6A2
648 FIGF
649 ADAM15
650 NID1
651 ADAM2
652 COL6A3
653 HSPG2
654 COL5A1
655 MDK
656 CHAD
657 SEMA7A
658 LAMC1
659 ADAM9
660 NPNT
661 LAMC3
662 LAMA2
663 COL6A1
664 COL5A2
665 COL4A6
666 LAMA4
667 COL3A1
668 LAMA5
669 KITLG
670 CLEC11A
671 SERPINE2
672 SERPING1
673 SERPINA1
674 C1QB
675 LRPAP1
676 FCN2
677 PSAP
678 SEMA5A
679 SEMA4D
680 RTN4
681 SYTL3
682 LRP1B
683 SLIT1
684 LACRT
685 RSPO3
686 FGF6
687 PODXL2
688 MUC7
689 GNB3
690 CGN
691 GDF9
692 F2
693 S100A8
694 TNFSF13
695 TNFSF12

696 OMG
697 TNFSF9
698 LRP1
699 AXL
700 SDC4
701 ITGB1
702 RAMP2
703 CALCR
704 GPR182
705 MRGPRX2
706 CALCRL
707 ALOX5
708 EGFR
709 TIE1
710 ITGB2
711 OLR1
712 ITGAM
713 TLR2
714 LRP5
715 LRP2
716 CHRNA4
717 LDLR
718 SCARB1
719 LRP8
720 VLDLR
721 SORL1
722 NGFR
723 CAV1
724 FPR2
725 NCSTN
726 TNFRSF21
727 GPC1
728 CD74
729 SLC45A3
730 ERBB3
731 PLD2
732 INSR
733 CHRM3
734 CD3D
735 HLA.F
736 KLRC1
737 KIR2DL3
738 TFRC
739 KIR2DL1
740 LILRB2
741 CD3G
742 KLRD1
743 KIR3DL1
744 HFE
745 CD1A
746 CD247
747 CD1B
748 LILRB1
749 NGFRAP1
750 DDR1
751 LY96
752 TLR4
753 IFITM1

754 ITGAX
755 CD81
756 C5AR2
757 CR1
758 C3AR1
759 CD19
760 CD46
761 CXCR3
762 CCR7
763 CCR6
764 ACKR2
765 CCR4
766 CCR1
767 CCR3
768 CCR5
769 CCR8
770 SDC1
771 GPR75
772 ACKR4
773 ITGA4
774 SELP
775 SELL
776 CD40
777 TRAF3
778 CD27
779 CDH2
780 PTPRM
781 KLRG1
782 PTPRF
783 CXCR4
784 CD36
785 CD44
786 ITGB8
787 CD47
788 CD93
789 ITGAV
790 ITGA2
791 ITGA1
792 CSF1R
793 CSF2RA
794 CSF3R
795 CD4
796 MET
797 ERBB2
798 ERBB4
799 TNFRSF1A
800 FAS
801 PLAUR
802 CD79A
803 TSHR
804 ITGA6
805 COL13A1
806 ITGA8
807 ITGA5
808 ITGA9
809 IL17RC
810 ITGB6
811 NT5E

812 C5AR1
813 FLT4
814 ITGB3
815 ROBO4
816 ITGA2B
817 ITGA3
818 SDC2
819 TNFRSF11B
820 TMPRSS6
821 MAG
822 ITGB7
823 LHCGR
824 GCGR
825 ADORA1
826 ADCY9
827 PTGDR
828 GLP1R
829 ADRB3
830 ADCY8
831 HTR6
832 CRHR1
833 AVPR2
834 ADCY1
835 ADCY7
836 VIPR1
837 PTGIR
838 TRAF2
839 IGF2R
840 PGRMC1
841 HMMR
842 PRLR
843 CD82
844 CD9
845 HRH1
846 HRH4
847 HRH2
848 HRH3
849 ST14
850 APLP2
851 KIR3DL2
852 CANX
853 KIR2DS4
854 SLC9C2
855 NOTCH4
856 KLRC2
857 SLC16A4
858 THBD
859 ASGR1
860 ASGR2
861 FGFR3
862 CFTR
863 GRIN2D
864 IL2RA
865 ITGAL
866 IL2RG
867 ITGAD
868 CLEC4M
869 IFNGR2

870 IFNGR1
871 FZD8
872 LRP6
873 PTCH2
874 IL10RA
875 SIRPG
876 CD28
877 IL12RB2
878 IL12RB1
879 IL13RA1
880 IL2RB
881 CD48
882 IL1RL2
883 IL1RAPL1
884 IL18R1
885 IL18RAP
886 IL1R2
887 IL1R1
888 ADRB2
889 IL1RAP
890 CD53
891 IL5RA
892 F3
893 IL6R
894 IL6ST
895 IL7R
896 KISS1R
897 RPSA
898 CD151
899 VANGL1
900 GPIHBP1
901 LTBR
902 TNFRSF1B
903 CDH6
904 MST1R
905 PTPRA
906 PLGRKT
907 ABCA1
908 OPRL1
909 TYRO3
910 PLXNB2
911 ATP6AP2
912 FPR1
913 CLDN4
914 S1PR1
915 CNR1
916 TGFBR1
917 CD63
918 TNFRSF10A
919 TNFRSF10D
920 TNFRSF10B
921 TNFRSF10C
922 TNFRSF13C
923 TNFRSF17
924 TNFRSF13B
925 TNFRSF4
926 TLR1
927 NRP2

928 RET
929 NRP1
930 EPHB2
931 KDR
932 SIRPA
933 FLT1
934 ADRA2B
935 AGTR1
936 CXCR1
937 CXCR2
938 CXCR5
939 DCBLD2
940 DRD2
941 EDNRB
942 EPHA2
943 EPHA3
944 ERAP1
945 F2R
946 FCER1A
947 FCGR1A
948 FSHR
949 GP6
950 IGF1R
951 KCNN4
952 KCNQ3
953 KIT
954 MIP
955 NOTCH1
956 NRXN1
957 OPRM1
958 SCARF1
959 SCTR
960 SELE
961 TBXA2R
962 TGFBR2
963 TLR7
964 TLR9
965 TNFRSF12A
966 TNFRSF9

A
