# Supplementary material for: Single-Cell and Transcriptome-Based Immune Cell-Related Prognostic Model in Clear Cell Renal Cell Carcinoma
Source: J Oncol. 2023 Mar 7;2023:5355269. doi: 10.1155/2023/5355269 (PMC10014191; doi:10.1155/2023/5355269)
Supplement: Supplementary Materials — Supplementary Table 1: Notes on cell clustering. Supplementary Table 2: Differential genes in each cell cluster. Supplementary Table 3: Ligand-receptor relationship pair. Supplementary Table 4: Immune cell multifactor network relationship pair. Supplementary Table 5: Intersection genes in immune cell multifactor network relationship pair and TCGA. Supplementary Table 6: Genes in black and magenta models of WGCNA. [file 5355269.f1.zip › Supplementary Table 6. Genes in black and magenta models of WGCNA.pdf]

	x
1	PAX2
2	NFIL3
3	NFKBIZ
4	IRF2
5	NKX2.1
6	LRRFIP1
7	DACH1
8	TEAD4
9	HIPK2
10	HEXIM1
11	DNMT3A
12	FOXI1
13	CD14
14	CD40LG
15	CDH1
16	COL1A1
17	COL4A3
18	CXCL12
19	IL12A
20	INS
21	MMP2
22	SHH
23	DEFB103B
24	HSP90B1
25	NRG1
26	COL5A2
27	RSPO3
28	GPR182
29	CCR3
30	ITGA6
31	CD151
32	HDAC1
33	EOMES
34	CDX1
35	HOXA9
36	ZNF24
37	E2F5
38	TBX5
39	KLF15
40	APP
41	AREG
42	FGF1
43	L1CAM
44	MST1
45	SERPINC1
46	VCAM1
47	ZP3
48	KNG1
49	COL3A1
50	VLDLR
51	DDR1
52	CSF1R
53	EPHA3
